# Shared and Unique Patterns of DNA Methylation in Systemic Lupus Erythematosus and Primary Sjögren's Syndrome

**DOI:** 10.3389/fimmu.2019.01686

**Published:** 2019-07-30

**Authors:** Juliana Imgenberg-Kreuz, Jonas Carlsson Almlöf, Dag Leonard, Christopher Sjöwall, Ann-Christine Syvänen, Lars Rönnblom, Johanna K. Sandling, Gunnel Nordmark

**Affiliations:** ^1^Section of Rheumatology and Science for Life Laboratory, Department of Medical Sciences, Uppsala University, Uppsala, Sweden; ^2^Molecular Medicine and Science for Life Laboratory, Department of Medical Sciences, Uppsala University, Uppsala, Sweden; ^3^Rheumatology, Division of Neuro and Inflammation Sciences, Department of Clinical and Experimental Medicine, Linköping University, Linköping, Sweden

**Keywords:** systemic lupus erythematosus, primary Sjögren's syndrome, DNA methylation, EWAS, epigenetics, autoimmunity, type I interferon, random forest

## Abstract

**Objectives:** To perform a cross-comparative analysis of DNA methylation in patients with systemic lupus erythematosus (SLE), patients with primary Sjögren's syndrome (pSS), and healthy controls addressing the question of epigenetic sharing and aiming to detect disease-specific alterations.

**Methods:** DNA extracted from peripheral blood from 347 cases with SLE, 100 cases with pSS, and 400 healthy controls were analyzed on the Human Methylation 450k array, targeting 485,000 CpG sites across the genome. A linear regression model including age, sex, and blood cell type distribution as covariates was fitted, and association *p*-values were Bonferroni corrected. A random forest machine learning classifier was designed for prediction of disease status based on DNA methylation data.

**Results:** We established a combined set of 4,945 shared differentially methylated CpG sites (DMCs) in SLE and pSS compared to controls. In pSS, hypomethylation at type I interferon induced genes was mainly driven by patients who were positive for Ro/SSA and/or La/SSB autoantibodies. Analysis of differential methylation between SLE and pSS identified 2,244 DMCs with a majority of sites showing decreased methylation in SLE compared to pSS. The random forest classifier demonstrated good performance in discerning between disease status with an area under the curve (AUC) between 0.83 and 0.96.

**Conclusions:** The majority of differential DNA methylation is shared between SLE and pSS, however, important quantitative differences exist. Our data highlight neutrophil dysregulation as a shared mechanism, emphasizing the role of neutrophils in the pathogenesis of systemic autoimmune diseases. The current study provides evidence for genes and molecular pathways driving common and disease-specific pathogenic mechanisms.

## Introduction

Systemic lupus erythematosus (SLE) and primary Sjögren's syndrome (pSS) are two clinically and immunologically related chronic inflammatory autoimmune diseases with a multifactorial etiology. Both diseases have a clear female predominance and share certain clinical features, such as arthralgia, myalgia, non-erosive arthritis and leukopenia, while other clinical manifestations are more disease-specific, e.g., serositis or glomerulonephritis in patients with SLE, and major salivary gland swelling or purpura in pSS. B cell hyperactivity resulting in hypergammaglobulinemia and autoantibody production is a characteristic feature of SLE and pSS (1, 2). Furthermore, both diseases are associated with an increased risk for development of B cell lymphoma, although more prevalent in pSS (3–5). Another hallmark of both diseases is the activation of the type I interferon (IFN) system with elevated plasma levels of IFN-α and transcriptional upregulation of IFN regulated genes, referred to as IFN signature (6–8). While alternating flares and remissions are common in SLE, pSS most often has a stable disease course (9).

Although the precise etiology of SLE and pSS remains elusive, they are considered to be complex diseases where genetic predisposition, environmental triggers, and epigenetic mechanisms contribute to disease development. A substantial number of major genetic susceptibility loci are shared between both diseases, such as variants at *HLA class II, BLK, IRF5*, and *STAT4* as well as at many other loci with smaller effect sizes (10–13). Genetic variants associated with risk for SLE and pSS are predominately found in non-coding regions in the genome and their functional impact has in most cases not yet been deciphered. It is thought that genetic risk variants at least partly may exert their impact on disease susceptibility via their effects on epigenetic mechanisms resulting in altered gene expression in target cells and tissues (14). In recent studies, increasing evidence has been assigned to the contributing role of epigenetic mechanisms in initiation and progression of systemic autoimmune diseases, and widespread changes in DNA methylation have been identified in SLE and pSS by epigenome-wide association studies (EWAS) comparing affected cases and control individuals (15–18). Albeit results from these EWASs point to the existence of shared epigenetic mechanisms across different inflammatory autoimmune diseases, systematic cross-comparative analyses on the genome-wide scale have not been performed.

In the current study, we systematically investigated DNA methylation changes in SLE and pSS addressing the question of epigenetic sharing and aiming to detect disease-specific alterations. We performed comparative analyses of genome-wide DNA methylation profiles in peripheral blood samples from well-characterized cohorts of patients with SLE, patients with pSS and healthy control individuals.

## Materials and Methods

### Subjects and Samples

Patients with SLE (*n* = 347; 86.5% women; mean age 47.0 ± 17.2 years) and patients with pSS (*n* = 100; 89% women; 56.1 ± 13.6 years) attending the Rheumatology Units at the Uppsala and Linköping University Hospitals, Sweden, and control individuals from the Uppsala Bioresource of healthy blood donors (*n* = 400; 87.7% women; 47.1 ± 13.2 years) visiting the Department of Transfusion Medicine, Uppsala University Hospital, Sweden, were included in the study as previously described (19, 20). All patients with SLE fulfilled the American College for Rheumatology (ACR) 1982 SLE classification criteria (21). All patients with pSS fulfilled the American European Consensus Group (AECG) criteria (22), and 75% of the pSS patients were positive for autoantibodies against Ro/SSA and/or La/SSB. All subjects included in the study provided informed consent to participate. The study protocol was approved by the Regional Ethics boards and the study was conducted in accordance with the principles of the Helsinki Declaration.

### Analysis of DNA Methylation

Venous blood samples from patients and controls were collected in EDTA tubes and kept at −20°C until DNA extraction. Isolation of genomic DNA was performed using the QIAamp Blood Mini/Midi Kit (Qiagen). The Illumina Human Methylation 450k array (HM450k), which targets > 485,000 CpG sites across the genome, was used to interrogate DNA methylation in peripheral blood samples from patients with SLE, patients with pSS, and healthy controls (23). Samples were randomized on the BeadChip to avoid batch effects. Data acquisition, preprocessing, quality control (QC), and normalization of methylation data have been described previously (19, 20). The post-QC dataset comprised 385,962 autosomal CpG sites.

Publicly available reference DNA methylation signatures of flow sorted blood cell types were used to estimate blood cell type distribution for each of the study samples applying the method by Houseman et al. (24) implemented in the R package *minfi* (25) (Supplementary Figure S1).

### Statistical Analyses

For the EWASs, a linear regression model was fitted including sex, age at sampling, and blood cell type distribution as covariates. DMCs were defined as *p* < 1.3 × 10^−7^ based on Bonferroni correction for the number of tested sites and an absolute average difference in the methylation β-value of |Δβ| > 0.05 between groups.

To classify as shared DMCs, case-control DMCs additionally had to fulfill the following criterion: *p* < 6.6 × 10^−6^ (Bonferroni adjusted significance threshold based on the 7,625 DMCs identified in the SLE case-control EWAS; the same *p*-value threshold was applied to both diseases) and same direction of effect in the EWAS analysis of the other disease compared to control individuals.

A combined set of shared DMCs between SLE and pSS was obtained by merging the shared DMCs identified with both aforementioned approaches and removing duplicate DMCs.

To call specific DMCs for one of the two autoimmune diseases, a DMC had to have a *p*-value of >0.05 in the EWAS comparing the other disease to control individuals.

### Pathway Analyses and Functional Genomic Distribution of DMCs

Probe mapping and annotation in relation to gene regions were performed as previously described (26). For classification of IFN regulated genes the Interferome v2.01 database was used (27). Publicly available data on chromatin modification states from primary CD3^+^ T cells and CD19^+^ B cells were obtained from the NIH Roadmaps Epigenomics Project for the histone modification marks H3K4me1, H3K4me3, H3K27ac, H3K36me3, H3K9me3, H3K27me3, and DNase I hypersensitive sites (DHS) as previously described (19, 28). Chromatin mark peaks from these reference blood cells were investigated for overlap with the genomic coordinates of DMCs from our case-case analysis of differential DNA methylation. The regional distribution of all probes (post QC-probe set) was compared with the distribution of associated CpG sites using the X_2_-test, where significance was defined at *p* < 0.0035 after Bonferroni correction.

Functional gene-set enrichment analyses were conducted using the ToppGene Suite database (29). For differential DNA methylation uniquely associated with SLE in the SLE case-control EWAS, all unique genes (*n* = 401) were included in the analysis. Pathway analyses of shared differential methylation between SLE and pSS compared to controls and of differential methylation identified between SLE and pSS in the case-case EWAS, the 1000 most significantly associated DMCs with a unique gene name annotation each were included.

### Random Forest Predictions of Disease Status

Predictions of disease status were calculated based on the DNA methylation data interrogated on the HM450k array using a random forest machine learning method (30) similarly as previously performed for genotype data (31). The computations were run using the R package *Emil* (32) which in turn uses the R package *Random Forest* (33). Disease status was predicted based on methylation β-values in three iterations with five cross-validation folds per iteration, where each of the 15 cross-validation runs used 80% of the data for training of the classifier and 20% for testing. To improve prediction performance and reduce computational time, CpG sites were selected based on a linear regression test where approximately the top 1000 associated sites were included. The CpG site selection was performed once per fold and was calculated only on training data. The number of variables selected per tree (mtry) and number of trees (tree) for the random forest algorithm were set to 300 and 1,000, respectively. In total, four random forest based predictions of disease status were performed: (a) SLE compared to controls, (b) pSS (all patients) compared to controls, (c) pSS (SSA/SSB positive patients) compared to controls, and (d) pSS (all patients) compared to SLE.

## Results

In order to advance our understanding of how DNA methylation contributes to common and specific features of SLE and pSS we applied several strategies: first, we identified the differentially methylated CpG sites (DMCs) that are shared and unique between SLE and pSS when comparing methylation patterns in the patient groups to control individuals. Second, we performed a case-case analysis of differential methylation directly between patients with SLE and with pSS. Finally, we evaluated the performance of a random forest machine learning method to predict disease status based on DNA methylation data.

### Shared Differential DNA Methylation in SLE and pSS Compared to Controls

**Figure 1:**
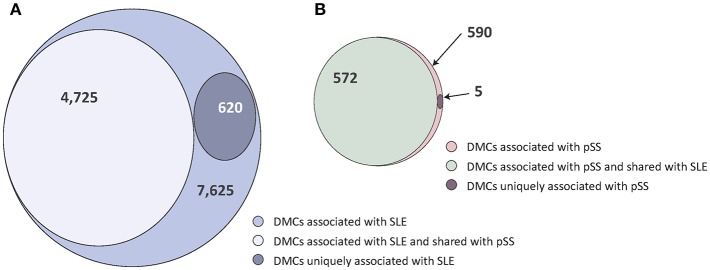
Venn diagrams of the degree of shared and disease-specific differential methylation between the SLE case-control EWAS and the pSS case-control EWAS. **(A)** The SLE case-control EWAS identified a total number of *n* = 7,625 DMCs (indicated in light purple), of which *n* = 4,725 were shared with pSS (in gray) and *n* = 620 were uniquely associated with SLE (in dark purple). **(B)** The pSS case-control EWAS identified a total number of *n* = 590 DMCs (indicated in light red), of which *n* = 572 were shared with SLE (in green) and *n* = 5 were uniquely associated with pSS (in dark red).

In two previous studies, we identified patterns of DNA methylation associated with SLE and pSS, respectively, comparing patients and healthy blood donor controls (19, 20). In the SLE case-control EWAS, we had identified 7,625 DMCs. In the current study we found that 4,725 (62%) of the SLE DMCs were also associated with pSS (*p* < 6.6 × 10^−6^ and same direction of effect, Figure 1A, Supplementary Table S1). Applying the same significance criteria as in the SLE case-control EWAS, our previously published pSS case-control EWAS identified a total number of 590 DMCs associated to pSS (20). The vast majority of these DMCs (*n* = 572; 97%) were also found in the SLE case-control association analysis (Figure 1B, Supplementary Table S2).

**Figure 2:**
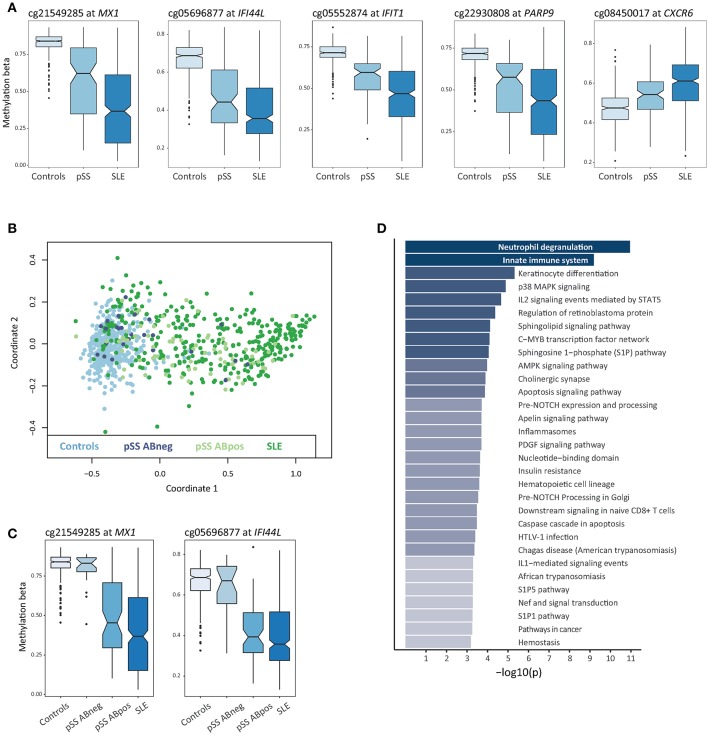
Differential DNA methylation shared between SLE and pSS. **(A)** Box plots of DNA methylation levels for controls (*n* = 400), pSS patients (*n* = 100), and SLE patients (*n* = 347) with a notch indicating the group median methylation β, at shared DMCs in SLE and pSS compared to controls at *MX1, IFI44L, IFIT1, PARP9*, and *CXCR6*. **(B)** Multidimensional scaling (MDS) plot based on DNA methylation levels at IFN regulated genes for all individuals included in the study. For each sample in the analysis DNA methylation levels at CpG sites located at 10 type I IFN regulated genes (*IFI44L, IFIT1, IFITM1, IFITM3, IRF7, MX1, OAS1, PARP9, PLSCR1*, and *RSAD2*) were used to plot coordinate one and two. Data from controls are indicated in light blue, anti-SSA/SSB negative pSS in dark blue, anti-SSA/SSB positive pSS in light green and SLE in dark green. **(C)** Box plots of DNA methylation levels at *MX1* and *IFI44L* with pSS patients stratified for anti-SSA/SSB negative pSS (*n* = 25) and anti-SSA/SSB positive pSS (*n* = 75). **(D)** Functional pathway analysis. The bar plot depicts the results of the functional pathway analysis of the 1,000 most significantly associated DMC based on their association *p*-value in the SLE case-control EWAS exhibiting a gene name annotation in the combined set of shared DMCs between SLE and pSS. Significantly enriched pathways are presented on the y-axis with their corresponding –log_10_(p) on the x-axis.

In total, a combined set of 4,945 shared DMCs was identified using both approaches, with a majority of DMCs showing hypomethylation in SLE and pSS patients compared to controls (*n* = 3,572; 72%) (Supplementary Table S3). The shared DMCs with the largest effect sizes were located at type I IFN regulated genes (Figure 2A, Table 1). It has been suggested that SSA/SSB antibody positive pSS has a more pronounced activation of the IFN system (34). Multidimensional scaling (MDS) analysis based on methylation levels of highly significant DMCs at 10 IFN induced genes revealed that the majority of SSA/SSB negative patients with pSS clustered together with the control individuals (Figure 2B). For example, at promoter regions of the IFN regulated genes *MX1* and *IFI44L*, we observed that SSA/SSB positive pSS patients had similar methylation levels as SLE patients, whereas levels in SSA/SSB negative pSS were more similar to the control group (Figure 2C). Gene-set enrichment analysis was performed on the top 1000 unique genes annotated to the shared DMCs in SLE and pSS, and showed the importance of functional pathways related to neutrophil degranulation (*p* = 1.1 × 10^−11^), innate immune system (*p* = 6.5 × 10^−10^), keratinocyte differentiation (*p* = 4.9 × 10^−6^), and p38 MAPK signaling (*p* = 1.3 × 10^−5^) (Figure 2D, Supplementary Table S4).

**Table 1:** Top shared differentially methylated sites (DMCs) between the SLE case-control EWAS and the pSS case-control EWAS.

**CpG site**	**Position (chr:bp)**	**Gene**	**Gene name**	**Mean meth-β in SLE**	**Mean meth-β in pSS**	**Mean meth-β in ctrl**	**Δβ SLE- ctrl^**§**^**	**Δβ pSS- ctrl^**§**^**	***p*-value EWAS SLE-ctrl^‡^**	***p*-value EWAS pSS- ctrl^‡^**
cg03607951	1:79085586	*IFI44L*	Interferon induced protein 44 like	0.34	0.41	0.59	−0.25	−0.18	3.0 × 10^−141^	9.9 × 10^−67^
cg05696877	1:79088769	*IFI44L*	Interferon induced protein 44 like	0.41	0.47	0.67	−0.26	−0.20	1.9 × 10^−120^	2.9 × 10^−50^
cg01028142	2:7004578	*CMPK2*	Cytidine/uridine monophosphate kinase 2	0.73	0.81	0.88	−0.15	−0.07	1.2 × 10^−64^	2.8 × 10^−32^
cg10959651	2:7018020	*RSAD2*	Radical S-adenosyl methionine domain containing 2	0.16	0.19	0.27	−0.11	−0.07	2.9 × 10^−110^	1.3 × 10^−34^
cg22930808	3:122281881	*PARP9*	Poly(ADP-ribose) polymerase family member	0.43	0.52	0.71	−0.27	−0.19	1.4 × 10^−105^	2.4 × 10^−55^
cg00959259	3:122281975	*PARP9*	Poly(ADP-ribose) polymerase family member	0.35	0.41	0.58	−0.23	−0.17	9.3 × 10^−105^	2.6 × 10^−48^
cg06981309	3:146260954	*PLSCR1*	Phospholipid scramblase 1	0.30	0.39	0.54	−0.24	−0.15	4.9 × 10^−157^	4.1 × 10^−51^
cg17608381	6:29911550	*HLA-A*	Major histocompatibility complex, class I, A	0.49	0.48	0.60	−0.11	−0.12	4.8 × 10^−25^	3.4 × 10^−15^
cg07180897	6:32729130	*HLA-DQB2*	Major histocompatibility complex, class II, DQ beta 2	0.74	0.73	0.81	−0.07	−0.08	2.7 × 10^−10^	8.9 × 10^−07^
cg10152449	7:2444534	*CHST12*	Carbohydrate sulfotransferase 12	0.27	0.31	0.39	−0.11	−0.08	3.6 × 10^−102^	9.5 × 10^−28^
cg14864167	8:66751182	*PDE7A*	Phosphodiesterase 7A	0.51	0.57	0.65	−0.14	−0.08	3.6 × 10^−41^	1.3 × 10^−18^
cg11317199	9:100850391	*TRIM14*	Tripartite motif containing 14	0.68	0.66	0.59	0.09	0.07	1.5 × 10^−33^	1.1 × 10^−15^
cg05552874	10:91153143	*IFIT1*	Interferon induced protein with tetratricopeptide repeats 1	0.46	0.57	0.71	−0.25	−0.14	2.5 × 10^−128^	2.7 × 10^−56^
cg01971407	11:313624	*IFITM1*	Interferon induced transmembrane protein 1	0.40	0.41	0.48	−0.08	−0.07	3.8 × 10^−53^	3.3 × 10^−30^
cg23570810	11:315102	*IFITM1*	Interferon induced transmembrane protein 1	0.49	0.55	0.69	−0.20	−0.14	1.6 × 10^−75^	6.1 × 10^−38^
cg03038262	11:315262	*IFITM1*	Interferon induced transmembrane protein 1	0.45	0.48	0.57	−0.12	−0.09	1.6 × 10^−50^	2.7 × 10^−32^
cg20045320	11:319555	NA		0.43	0.46	0.55	−0.13	−0.10	1.0 × 10^−63^	5.3 × 10^−26^
cg09122035	11:319667	NA		0.36	0.40	0.49	−0.13	−0.09	3.1 × 10^−72^	1.5 × 10^−20^
cg17990365	11:319718	*IFITM3*	Interferon induced transmembrane protein 3	0.50	0.53	0.61	−0.11	−0.08	9.5 × 10^−56^	5.9 × 10^−23^
cg08926253	11:614761	*IRF7*	Interferon regulatory factor 7	0.47	0.52	0.60	−0.13	−0.08	6.8 × 10^−81^	3.8 × 10^−34^
cg27209729	11:64428925	*NRXN2*	Neurexin 2	0.54	0.53	0.61	−0.08	−0.08	9.3 × 10^−50^	4.6 × 10^−27^
cg03172657	16:89163625	*ACSF3*	Acyl-CoA synthetase family member 3	0.53	0.53	0.45	0.08	0.07	4.6 × 10^−34^	3.8 × 10^−13^
cg10604476	19:10403908	*ICAM5*	Intercellular adhesion molecule 5	0.55	0.57	0.48	0.07	0.09	1.1 × 10^−13^	5.8 × 10^−11^
cg05825244	20:2730488	*EBF4*	EBF family member 4	0.54	0.57	0.47	0.07	0.11	2.3 × 10^−11^	2.4 × 10^−11^
cg22862003	21:42797588	*MX1*	MX dynamin like GTPase 1	0.43	0.51	0.70	−0.27	−0.19	2.5 × 10^−126^	7.9 × 10^−63^
cg26312951	21:42797847	*MX1*	MX dynamin like GTPase 1	0.26	0.29	0.44	−0.18	−0.14	1.3 × 10^−82^	1.3 × 10^−41^
cg21549285	21:42799141	*MX1*	MX dynamin like GTPase 1	0.41	0.57	0.83	−0.42	−0.26	3.5 × 10^−139^	6.9 × 10^−59^
cg20098015	22:50971140	*ODF3B*	Outer dense fiber of sperm tails 3B	0.33	0.39	0.49	−0.15	−0.10	7.1 × 10^−96^	2.1 × 10^−33^
cg05523603	22:50973101	NA		0.59	0.64	0.72	−0.13	−0.08	4.7 × 10^−71^	3.8 × 10^−26^

DMCs with methylation |Δβ| > 0.07 in both case-control EWASs are listed ordered by their chromosomal position.

§ Methylation Δβ refers to the difference in mean methylation β between patients with SLE, respectively, pSS and control individuals, with a negative value representing decreased methylation in the patients.

‡ P-value for the case-control EWAS.

EWAS, epigenome-wide association study; meth-β, methylation β; NA, not annotated.

### Unique Differential DNA Methylation in SLE

**Figure 3:**
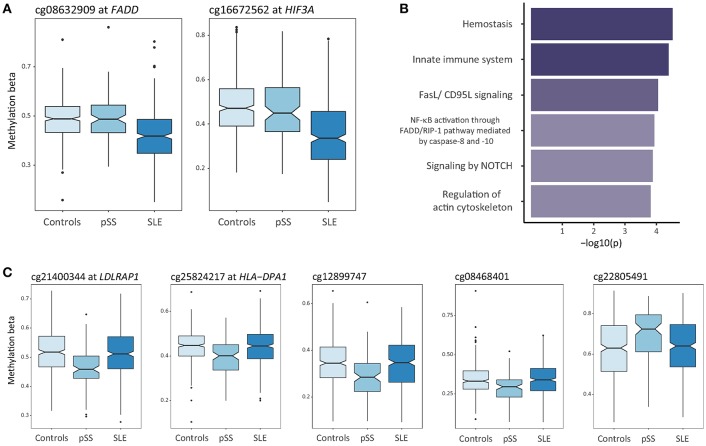
Differential DNA methylation uniquely associated to SLE or to pSS. **(A)** Box plots showing DNA methylation levels at unique DMCs in SLE compared to controls at *FADD* and *HIF3A*. **(B)** Functional pathway analysis of SLE-specific differential methylation. The bar plot depicts the results of the functional pathway analysis of the unique genes (*n* = 401) that were uniquely associated with SLE in the SLE case-ctrl EWAS. Significantly enriched pathways are presented on the y-axis with their corresponding –log_10_(p) on the x-axis. **(C)** Box plots showing DNA methylation levels at unique DMCs in pSS compared to controls at *LDLRAP1, HLA-DPA1*, cg12899747 (intergenic, chr 3), cg08468401 (intergenic, chr 3), and cg22805491 (intergenic, chr 14).

Next, we sought to define disease-specific DNA methylation changes to isolate the unique components of SLE and pSS. DMCs in the SLE case-ctrl EWAS (*n* = 7,625 DMCs) were considered as SLE-specific if the same DMCs displayed a *p*-value >0.05 in the pSS case-control EWAS. Here, we identified 620 SLE-specific DMCs (453 hypo- and 167 hypermethylated sites) annotated to 401 unique genes (Figure 1A, Supplementary Table S1). Figure 3A provides two examples of SLE-specific DMCs identified at Fas associated via death domain (*FADD*, cg08632909) and at hypoxia inducible factor 3 subunit alpha (*HIF3A*, cg16672562). To further characterize the unique DMCs in SLE, we performed a gene ontology enrichment analysis of the genes harboring SLE-specific differential methylation, and identified hemostasis (*p* = 3.0 × 10^−5^), innate immune system (*p* = 4.0 × 10^−5^), and FasL/CD95L signaling (*p* = 8.8 × 10^−5^) as the most significantly enriched functional pathways specific for SLE (Figure 3B, Supplementary Table S5).

### Unique Differential DNA Methylation in pSS

We found methylation changes at five DMCs that were uniquely associated with pSS (Figure 1B, Table 2). These DMCs were annotated to the low density lipoprotein receptor adaptor protein 1 gene (*LDLRAP1*, cg21400344), to major histocompatibility complex (MHC), class II, DP alpha 1 (*HLA-DPA1*, cg25824217), and to intergenic regions on chromosome 3 and 14 (cg1289974, cg08468401, cg22805491) (Figure 3C). With the exception of the hypermethylated DMC on chromosome 14, all DMCs specific for pSS presented with decreased methylation in pSS compared to control individuals, while no significant difference in methylation at these sites was observed between patients with SLE compared to controls. The DMCs at *LDLRAP1* and *HLA-DPA1* were overlapping with the genomic position of histone marks for active promoter (H3K4me3) and enhancer (H3K27ac) regions in reference B cells and T cells, and with DNase hypersensitivity sites (DHS), indicating that expression of these genes may be up-regulated in patients with pSS (Table 2).

**Table 2:** Differentially methylated CpG sites (DMCs) uniquely associated with primary Sjögren's syndrome (pSS)^*^.

**CpG site**	**Position (chr:bp)**	**Gene**	***p*-value EWAS pSS-ctrl^**§**^**	**Δβ pSS-ctrl^**#**^**	***p*-value EWAS SLE-ctrl^†^**	**Δβ SLE-ctrl^**#**^**	**Enhancer^‡^**	**Promoter^**¶**^**	DHS^**^
cg21400344	1:25870172	*LDLRAP1*	2.7 × 10^−12^	−0.06	0.13	−0.0079	No	Yes^a, b^	Yes^a, b^
cg25824217	6:33040535	*HLA-DPA1*	1.0 × 10^−11^	−0.05	0.48	−0.0036	Yes^a, b^	Yes^b^	Yes^a, b^
cg12899747	3:25391527	NA	1.6 × 10^−11^	−0.06	0.21	−0.0076	No	No	No
cg08468401	3:14303131	NA	1.2 × 10^−9^	−0.05	0.85	−0.0011	No	No	Yes^b^
cg22805491	14:51172404	NA	1.0 × 10^−7^	0.08	0.10	0.0158	No	No	No

* DMCs with p < 1.3 × 10^−7^ and average methylation difference |Δβ| > 0.05 in the pSS case-control EWAS, while p > 0.05 in the SLE case-control EWAS.

§ pSS case-control EWAS p-value.

# Methylation Δβ refers to the difference in mean methylation β between patients with pSS, respectively, SLE and control individuals with a negative value representing decreased methylation in the patients.

† SLE case-control EWAS p-value.

‡ Genomic location of DMC overlapping H3K27ac (active enhancer mark) peak in a) reference CD3^+^ T cells, and/or b) reference CD19^+^ B cells.

¶ Genomic location of DMC overlapping H3K4me3 (active promoter mark) peak in a) reference CD3^+^ T cells, and/or b) reference CD19^+^ B cells.

** Genomic location of DMC overlapping DHS (indicating euchromatin) in a) reference CD3 ^+^ T cells, and/or ^b)^ reference CD19^+^ B cells.

*DHS, DNase hypersensitivity site; EWAS, epigenome-wide association study; HLA-DPA1, major histocompatibility complex, class II, DP alpha 1; LDLRAP1, low density lipoprotein receptor adaptor protein 1; NA, not annotated*.

## Differential DNA Methylation Between SLE and pSS

Next, we performed an association analysis directly interrogating DNA methylation changes across the genome between patients with SLE and patients with pSS to identify the CpG sites with the largest methylation differences between the two diseases.

**Figure 4:**
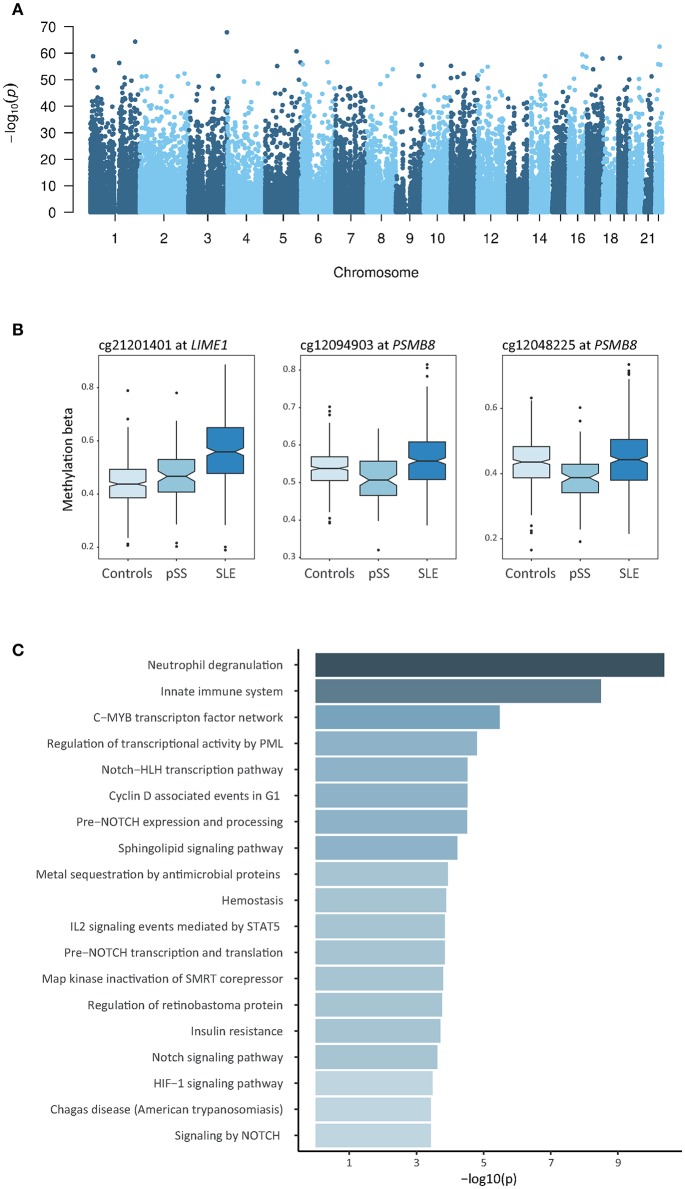
Results of the cross-comparative analysis of differential DNA methylation between SLE and pSS. **(A)** Manhattan plot showing the results of the analysis comparing DNA methylation in patients with SLE to patients with pSS. Presented are the –log_10_ transformed *p*-values of the association between the tested CpG sites and the disease status against the chromosomal position of the investigated sites. **(B)** Box plots of DNA methylation levels at DMCs identified in the analysis between patients with SLE compared to patients with pSS at *LIME1* and at two neighboring CpG sites within the *PSMB8-TAP2* locus. **(C)** Functional pathway analysis. The bar plot depicts the results of the functional pathway analysis of the 1,000 most significantly associated DMC in the SLE-pSS case-case analysis that have a unique gene name annotation. Significantly enriched pathways are presented on the y-axis with their corresponding –log_10_(p) on the x-axis.

Using a linear regression model with age, sex, and blood cell type distribution as covariates, we identified 2,244 DMCs between SLE and pSS which were annotated to 1,309 unique genes (Bonferroni adjusted *p*-value < 1.3 × 10^−7^ and an average methylation difference |Δβ| > 0.05, Figure 4A, Supplementary Table S6). In Table 3, the DMCs with the most prominent differences according to their methylation |Δβ| in the case-case analysis between SLE and pSS are presented. In contrast to our strategy above to identify unique DMCs for each disease, this direct analysis also has the potential to identify shared DMCs with differential methylation between SLE and pSS. We noted that a substantial fraction of the SLE-pSS DMCs were also differentially methylated between patients and controls in both diseases (*n* = 1,162; 52%). This suggests that although a large number of DMCs are shared between SLE and pSS, important quantitative differences exist.

**Table 3:** Top differentially methylated CpG sites (DMCs) in the case-case analysis between patients with systemic lupus erythematosus (SLE) and patients with primary Sjögren's syndrome (pSS).

**CpG site**	**Position (chr:bp)**	**Gene**	**Gene name**	**Mean meth-β in SLE**	**Mean meth-β in pSS**	**Δβ SLE-pSS^**§**^**	***p*-value SLE-pSS^‡^**
cg21549285	21:42799141	*MX1*	MX dynamin like GTPase 1	0.41	0.57	−0.16	1.1 × 10^−08^
cg05552874	10:91153143	*IFIT1*	Interferon induced protein with tetratricopeptide repeats 1	0.46	0.57	−0.11	8.1 × 10^−11^
cg03546163	6:35654363	*FKBP5*	FKBP prolyl isomerase 5	0.47	0.58	−0.10	1.8 × 10^−16^
cg04858164	15:57324333	*TCF12*	Transcription factor 12	0.46	0.56	−0.10	2.9 × 10^−31^
cg09166556	1:156724277	NA		0.55	0.65	−0.10	1.3 × 10^−40^
cg09010699	3:195171693	NA		0.43	0.53	−0.10	1.3 × 10^−68^
cg13984928	17:3704574	*ITGAE*	Integrin subunit alpha E	0.38	0.48	−0.09	2.0 × 10^−33^
cg21201401	20:62367884	*LIME1*	Lck interacting transmembrane adaptor 1	0.56	0.47	0.09	6.5 × 10^−23^
cg16672562	19:46801672	*HIF3A*	Hypoxia inducible factor 3 subunit alpha	0.36	0.45	−0.09	2.3 × 10^−11^
cg19055828	12:51139321	*DIP2B*	Disco interacting protein 2 homolog B	0.40	0.49	−0.09	1.3 × 10^−55^
cg15086439	1:236563070	*EDARADD*	EDAR associated death domain	0.38	0.47	−0.09	1.9 × 10^−34^
cg20934416	5:17444401	NA		0.43	0.52	−0.09	4.3 × 10^−34^
cg01079652	1:79118191	*IFI44*	Interferon induced protein 44	0.71	0.80	−0.09	2.0 × 10^−09^
cg20700740	1:9339683	NA		0.36	0.45	−0.09	1.6 × 10^−42^
cg19460836	17:79047872	*BAIAP2*	BAI1 associated protein 2	0.44	0.53	−0.09	2.4 × 10^−40^
cg00446123	20:62367888	*LIME1*	Lck interacting transmembrane adaptor 1	0.66	0.56	0.09	8.1 × 10^−38^
cg10408731	7:65214843	*LOC441242*		0.42	0.51	−0.09	5.3 × 10^−36^
cg07110356	17:56355431	*MPO*	Myeloperoxidase	0.42	0.51	−0.09	4.1 × 10^−40^
cg00980622	14:75884845	NA		0.41	0.50	−0.09	2.9 × 10^−43^
cg26298914	14:68798365	*RAD51B*	RAD51 paralog B	0.38	0.47	−0.09	5.9 × 10^−49^
cg13381110	18:60646614	*PHLPP1*	PH domain and leucine rich repeat protein phosphatase 1	0.53	0.62	−0.09	2.8 × 10^−20^
cg03637218	5:115209107	*AP3S1*	Adaptor related protein complex 3 subunit sigma 1	0.44	0.53	−0.09	3.9 × 10^−37^
cg16125725	15:70101302	NA		0.41	0.50	−0.09	9.7 × 10^−39^
cg25757820	2:224819307	NA		0.46	0.55	−0.09	4.7 × 10^−53^
cg25600606	11:33308345	*HIPK3*	Homeodomain interacting protein kinase 3	0.48	0.57	−0.09	1.0 × 10^−51^
cg10665891	12:117042917	NA		0.38	0.48	−0.09	2.8 × 10^−32^
cg25344401	7:4755415	*FOXK1*	Forkhead box K1	0.44	0.53	−0.09	7.0 × 10^−34^
cg03340036	4:89446409	*PIGY*	Phosphatidylinositol glycan anchor biosynthesis class Y	0.45	0.54	−0.09	1.5 × 10^−37^
cg13618969	9:129184186	*MVB12B*	Multivesicular body subunit 12B (*FAM125B*)	0.49	0.58	−0.09	8.1 × 10^−39^
cg23338668	8:74240259	NA		0.47	0.56	−0.09	1.3 × 10^−37^

The 30 DMCs with the largest absolute difference in mean methylation (|Δβ|) comparing SLE and pSS are listed.

§ Methylation Δβ refers to the difference in mean methylation β between patients with SLE and pSS, with a negative value representing decreased methylation levels in SLE.

‡ P-value of the case-case association analysis of differential DNA methylation between SLE and pSS.

*EWAS, epigenome-wide association study; meth-β, methylation β; NA, not annotated*.

The vast majority of SLE-pSS DMCs had decreased methylation in patients with SLE compared to patients with pSS (*n* = 2,002; 89%). Multiple sites among the relatively few top DMCs with increased methylation in SLE compared to pSS were located at the transcription start site region of the Lck interacting transmembrane adaptor 1 gene, *LIME1* (Figure 4B). LIME1 plays a role in the regulation of the adaptive immune system by linking B cell and T cell receptor stimulation to downstream signaling pathways (35, 36). Two DMCs located between the proteasome subunit beta 8 gene (*PSMB8*, also known as *LMP7*) and the transporter 2, ATP binding cassette subfamily B member gene (*TAP2*) on chromosome 6 showed decreased methylation levels in pSS (cg12094903, *p* = 4.1 × 10^−15^, and cg12048225, *p* = 1.6 × 10^−9^) (Figure 4B). Of note, hypomethylation at these sites was only observed in SSA/SSB positive pSS (Supplementary Figure S2). *PSMB8* encodes a subunit of the immunoproteasome, which is induced by IFN-γ (37).

In order to further characterize the DMCs identified in the analysis of patients with SLE compared to patients with pSS, we investigated a possible enrichment of DMCs for functional genomic annotation in relation to CpG islands and gene property, and for regulatory regions in reference B cells and T cells. We observed depletion of DMCs between SLE and pSS for location in CpG islands and transcription start sites. Instead, the DMCs were enriched in gene bodies and at histone marks for actively transcribed genes (Supplementary Figure S3). Conversely, DMCs between SLE and pSS where underrepresented at regions indicative of repressed transcription.

We further conducted a functional pathway analysis including the genes which were annotated to the most significant DMCs identified in the association analysis between patients with SLE and patients with pSS, and found neutrophil degranulation (*p* = 4.2 × 10^−11^), innate immune system (*p* = 3.2 × 10^−9^), and C-MYB transcription factor network (*p* = 3.4 × 10^−6^) as the most significantly enriched pathways (Figure 4C, Supplementary Table S7).

### Random Forest Prediction of Disease Status

**Figure 5:**
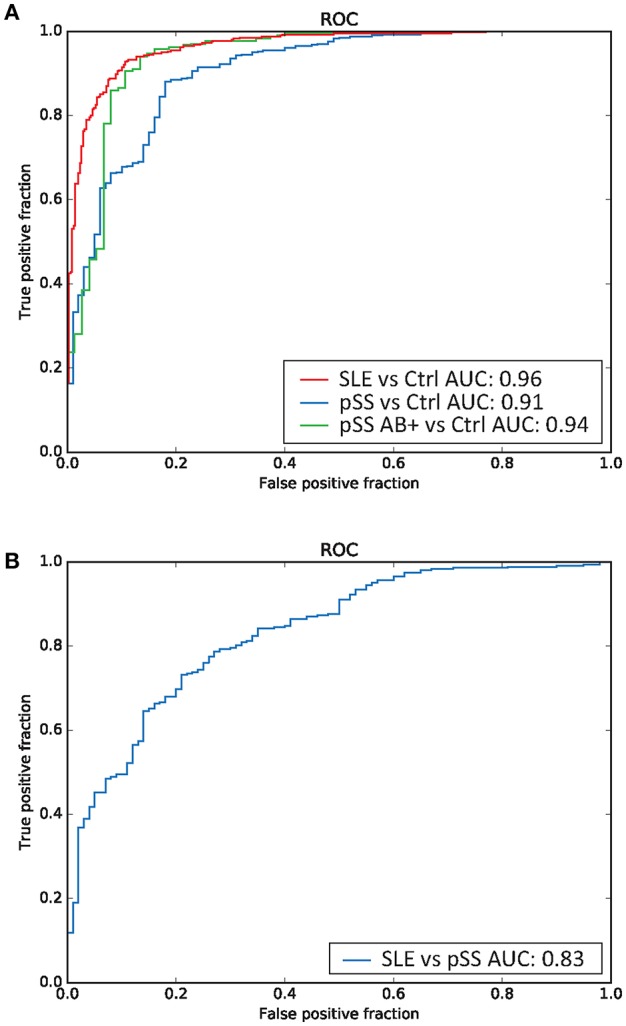
Random forest based prediction of disease status. Receiver operator characteristic (ROC) curves of the prediction accuracy for the DNA methylation data based random forest disease status classifications measured by the area under the curve (AUC), for **(A)** classification of SLE vs. controls (indicated in red), pSS (all patients) vs. controls (in blue), anti-SSA/SSB positive pSS vs. controls (in green), and **(B)** classification of SLE vs. pSS (in blue).

Finally, we sought to investigate whether DNA methylation profiles can be utilized for classification of disease status in a machine learning based approach. We applied a random forest model to build a classifier for discerning between disease status in healthy controls and in patients with SLE or pSS, respectively. In addition, we also performed a stratified classification in the subgroup of pSS patients that were positive for SSA/SSB autoantibodies. We found that the classifier performed well in distinguishing patients with SLE (AUC = 0.96) and patients with pSS from controls (AUC = 0.91) as displayed in Figure 5A. Only including SSA/SSB positive pSS patients in the analysis, resulted in an improved AUC value of 0.94 for discrimination between pSS cases and controls (Figure 5A). Importantly, reasonable good performance was also obtained in predicting disease status between SLE and pSS with an AUC of 0.83 (Figure 5B).

## Discussion

The cross-comparative analysis of DNA methylation performed in the current study facilitates identification of shared and disease-specific molecular signatures of SLE and pSS. We observed a widespread shared epigenetic architecture in SLE and pSS compared to healthy controls, underlining the concept of similar pathogenic mechanisms contributing to SLE and pSS (8, 38, 39), although we cannot exclude that the observed differences are reactive. The effect sizes of DNA methylation changes differed between the two diseases, while the direction of effect was usually the same; typically, with intermediate methylation levels in pSS compared to SLE and controls. We further noted that DNA hypomethylation at type I IFN regulated genes in pSS was mainly driven by SSA and/or SSB positive patients, which is in line with the notion that the IFN signature on the gene expression level is more pronounced in SSA/SSB positive pSS patients (34, 40). Neutrophil degranulation was the most significantly enriched functional pathway for shared DMCs, emphasizing the role of neutrophils in the pathogenesis of systemic autoimmune diseases (41).

Analysis of differential methylation that is not shared between SLE and pSS revealed that the extent of disease-specific differential methylation is limited, providing evidence for the hypothesis of largely similar epigenetic landscapes in SLE and pSS. The rare exceptional disease-specific alterations in methylation may, however, contribute to disease-specific pathogenesis and the variation in phenotype between SLE and pSS. Functional gene ontology analysis of the genes where SLE-specific differential DNA methylation was found, revealed beside an enrichment for broader functions of hemostasis and innate immunity, an overrepresentation of genes involved in induction and regulation of apoptotic processes and NFκB activation with a key role for *FADD* and *CASP-8* and *-9*. The importance of Fas/FasL-mediated apoptotic signaling in autoimmunity has been described, and increased apoptosis is a feature of SLE rather than pSS (42). Epigenetic changes at central genes within this pathway may contribute to the pathogenic mechanisms unique to SLE.

While the overwhelming majority of DMCs in the pSS case-control EWAS were also found when comparing patients with SLE to control individuals, we identified five sites with a differential methylation profile specific for pSS. The most significant of these pSS-specific DMCs was located in the *LDLRAP1* gene (cg21400344) and showed hypomethylation in a CpG island co-localizing with H3K4me3, a histone mark for active gene promoters, and DHS indicating open chromatin conformation. Indeed, revisiting our previously published transcriptome study analyzing peripheral B cells from patients with pSS and controls, we found upregulated gene expression of *LDLRAP1* in pSS B cells (43). The protein encoded by *LDLRAP1* interacts with the intracellular part of the LDL receptor in mediating endocytosis of cholesterol-rich LDL. The impact of the upregulated *LDLRAP1* gene expression in pSS pathogenesis is yet to be determined. Decreased methylation uniquely associated with pSS was also observed at the MHC class II locus *HLA-DPA1* (cg25824217) which plays a central role in the immune system by presentation of exogenous peptide antigens. Genetic variants at *HLA-DPA1* have been associated with a number of traits, including allergic disease and systemic sclerosis (44, 45), and upregulated protein expression of HLA-DP molecules has been reported by a small study in pSS salivary gland tissue (46).

Another region where disease-specific methylation patterns in pSS were found is the *TAP2-PSMB8-TAP1-PSMB9* locus in the MHC region. Cole et al. have described an extended region of hypomethylation around the *PSMB8* locus in their study investigating DNA methylation in salivary gland tissue from patients with pSS (47). While they observed the most prominent signal in the promoter of the non-coding RNA *PSMB8-AS* upstream of the *PSMB8* gene, we identified in our study of whole blood samples, two neighboring DMCs located between the 3′UTR of *PSMB8* and the promoter of *TAP2* which were hypomethylated in pSS compared to SLE. Both *PSMB8* and *TAP2* have pivotal functions in antigen presentation. *PSMB8* encodes the β5i subunit of the immunoproteasome, which plays a critical role in degradation of intracellular proteins for presentation by MHC class I molecules (48). Dysregulated expression of the proteasomal subunits β5i and β1i (PSMB9, also known as LMP2) in peripheral leukocytes and in inflammatory infiltrates of salivary gland tissue in patients with pSS has been reported in several studies (49–52), suggesting the immunoproteasome as a potential drug target (53, 54).

Environmental exposures and genetic information can be integrated at the level of epigenetic variation, where DNA methylation has the potential to propagate activity states in immune cells. Mechanically this is accomplished by altered methylation at gene regulatory regions which in turn affects transcriptional events. DNA methylation is established *de novo* and maintained during the cell cycle by DNA methyl transferases (DNMTs). Active demethylation is carried out by ten eleven translocation enzymes (TETs), while indirect loss of methylation can take place when DNMTs are inhibited during the process of DNA replication. It has also been suggested that (constitutively) altered activity of DNMT and TET enzymes in autoimmune diseases is causing the observed epigenetic dysregulation, as reviewed in (55). However, as shown here and by others, differential methylation is found at specific genes, with decreased methylation at genes in the IFN system as the most prominent feature in systemic autoimmune diseases (18). This suggests that also more targeted mechanisms are at play in the epigenetic dysregulation.

One of the strengths of the study is the analysis of a large cohort of clinically well-characterized patients and control individuals. Interrogation of DNA methylation on the HM450k array was performed simultaneously for all 847 samples included in the study, thus potential confounding of associations by batch effects is unlikely in our study. We further applied rigorous normalization and QC procedures and strict multiple testing correction by Bonferroni adjustment of the statistical analyses to ascertain robust results.

A limitation of these data is that we could only adjust for major blood cell types in the analyses and cannot exclude persisting effects from differential distribution of cell type subpopulations. Some of the patients with SLE in our study may have secondary Sjögren's syndrome (sSS) and studies on epigenetic features of sSS are currently lacking. It would therefore be valuable to map the epigenetic landscape in patients with SLE with sSS. Also, during the disease course, some patients with pSS will develop clinical or laboratory features of SLE and fulfill classification criteria for both diseases, often designated pSS/SLE overlap (55). A longitudinal study in patients with pSS to pinpoint DNA methylation markers predictive for development of SLE would be of great interest.

The current study serves as a proof-of-principle of the ability of machine learning to extract relevant traits from genome-wide DNA methylation patterns in systemic autoimmune diseases. The classification facilitates discrimination between SLE and pSS status with high accuracy. While these results are encouraging, they need to be validated and replicated in future studies in additional cohorts. Future approaches within the field of autoimmunity may be directed toward identification of methylation signatures that correlate with disease course, development of severe manifestations and complications, such as lupus nephritis or lymphomagenesis, and with response to certain treatments. Similar efforts have already been undertaken in the field of cancer research, were DNA methylation data are used for cancer subtype classification and outcome prediction (56–59).

In conclusion, our current study comparing DNA methylation across the genome between patients with SLE and pSS reveals more similarities than differences. Given the genetic background with similar HLA-associations, pathogenic mechanisms of type I IFN and B cell activation as well as overlapping clinical features, this may not be surprising (8, 10, 39, 55). However, disease-specific DNA methylation changes occur, indicating specific pathways possibly contributing to the different phenotypes of SLE and pSS. Future studies will elucidate whether epigenetic signatures could serve as a complement to conventional clinical practice in identification of predictive parameters, a prerequisite for efficient precision medicine.

## Data Availability

Normalized or raw intensity data (IDAT) of the HM450k array are available upon request from the authors on a collaborative basis.

## Ethics Statement

The study protocol was approved by the Regional Ethics boards in Uppsala and Linköping with decision nrs 227/2000, 217/2006, M75-08/2008, 013/2009, and 155/2016.

## Author Contributions

JI-K, A-CS, JS, and GN designed the study. DL, CS, LR, and GN collected patient and control material and clinical data. JI-K and JCA analyzed the data. JI-K, JS, and GN wrote the manuscript. All authors read, provided critical review, and accepted the final version of the manuscript.

### Conflict of Interest Statement

The authors declare that the research was conducted in the absence of any commercial or financial relationships that could be construed as a potential conflict of interest.

## References

[1] Lipsky PE. Systemic lupus erythematosus: an autoimmune disease of B cell hyperactivity. Nat Immunol. (2001) 2:764–6. 10.1038/ni0901-764 11526379

[2] Mariette X Criswell LA. Primary Sjogren's syndrome. N Engl J Med. (2018) 378:931–9. 10.1056/NEJMcp1702514 29514034

[3] Bernatsky S Ramsey-Goldman R Labrecque J Joseph L Boivin JF Petri M. Cancer risk in systemic lupus: an updated international multi-centre cohort study. J Autoimmun. (2013) 42:130–5. 10.1016/j.jaut.2012.12.009 23410586 PMC3646904

[4] Theander E Henriksson G Ljungberg O Mandl T Manthorpe R Jacobsson LT. Lymphoma and other malignancies in primary Sjogren's syndrome: a cohort study on cancer incidence and lymphoma predictors. Ann Rheum Dis. (2006) 65:796–803. 10.1136/ard.2005.041186 16284097 PMC1798187

[5] Johnsen SJ Brun JG Gøransson LG Småstuen MC Johannesen TB Haldorsen K. Risk of non-Hodgkin's lymphoma in primary Sjogren's syndrome: a population-based study. Arthritis Care Res. (2013) 65:816–21. 10.1002/acr.21887 23139233

[6] Baechler EC Batliwalla FM Karypis G Gaffney PM Ortmann WA Espe KJ. Interferon-inducible gene expression signature in peripheral blood cells of patients with severe lupus. Proc Natl Acad Sci USA. (2003) 100:2610–5. 10.1073/pnas.0337679100 12604793 PMC151388

[7] Wildenberg ME van Helden-Meeuwsen CG van de Merwe JP Drexhage HA Versnel MA. Systemic increase in type I interferon activity in Sjogren's syndrome: a putative role for plasmacytoid dendritic cells. Eur J Immunol. (2008) 38:2024–33. 10.1002/eji.200738008 18581327

[8] Thorlacius GE Wahren-Herlenius M Rönnblom L. An update on the role of type I interferons in systemic lupus erythematosus and Sjogren's syndrome. Curr Opin Rheumatol. (2018) 30:471–81. 10.1097/BOR.0000000000000524 29889694

[9] Györi N Giannakou I Chatzidionysiou K Magder L van Vollenhoven RF Petri M. Disease activity patterns over time in patients with SLE: analysis of the Hopkins Lupus cohort. Lupus Sci Med. (2017) 4:e000192. 10.1136/lupus-2016-000192 28243457 PMC5307372

[10] Teruel M Alarcón-Riquelme ME. Genetics of systemic lupus erythematosus and Sjogren's syndrome: an update. Curr Opin Rheumatol. (2016) 28:506–14. 10.1097/BOR.0000000000000310 27227345

[11] Richard-Miceli C Criswell LA. Emerging patterns of genetic overlap across autoimmune disorders. Genome Med. (2012) 4:6. 10.1186/gm305 22284131 PMC3334554

[12] Deng Y Tsao BP. Updates in lupus genetics. Curr Rheumatol Rep. (2017) 19:68. 10.1007/s11926-017-0695-z 28983873

[13] Imgenberg-Kreuz J Rasmussen A Sivils K Nordmark G. Genetics and epigenetics in primary Sjogren's syndrome. Rheumatology. (2019). [Epub ahead of print]. 10.1093/rheumatology/key330 30770922 PMC8121440

[14] Liu Y Aryee MJ Padyukov L Fallin MD Hesselberg E Runarsson A. Epigenome-wide association data implicate DNA methylation as an intermediary of genetic risk in rheumatoid arthritis. Nat Biotechnol. (2013) 31:142–7. 10.1038/nbt.2487 23334450 PMC3598632

[15] Mazzone R Zwergel C Artico M Taurone S Ralli M Greco A. The emerging role of epigenetics in human autoimmune disorders. Clin Epigenetics. (2019) 11:34. 10.1186/s13148-019-0632-2 30808407 PMC6390373

[16] Imgenberg-Kreuz J Sandling JK Nordmark G. Epigenetic alterations in primary Sjogren's syndrome - an overview. Clin Immunol. (2018) 196:12–20. 10.1016/j.clim.2018.04.004 29649576

[17] Lanata CM Chung SA Criswell LA. DNA methylation 101: what is important to know about DNA methylation and its role in SLE risk and disease heterogeneity. Lupus Sci Med. (2018) 5:e000285. 10.1136/lupus-2018-000285 30094041 PMC6069928

[18] Carnero-Montoro E Alarcón-Riquelme ME. Epigenome-wide association studies for systemic autoimmune diseases: the road behind and the road ahead. Clin Immunol. (2018) 196:21–33. 10.1016/j.clim.2018.03.014 29605707

[19] Imgenberg-Kreuz J Carlsson Almlöf J Leonard D Alexsson A Nordmark G Eloranta ML. DNA methylation mapping identifies gene regulatory effects in patients with systemic lupus erythematosus. Ann Rheum Dis. (2018) 77:736–43. 10.1136/annrheumdis-2017-212379 29437559 PMC5909746

[20] Imgenberg-Kreuz J Sandling JK Almlöf JC Nordlund J Signér L Norheim KB. Genome-wide DNA methylation analysis in multiple tissues in primary Sjogren's syndrome reveals regulatory effects at interferon-induced genes. Ann Rheum Dis. (2016) 75:2029–36. 10.1136/annrheumdis-2015-208659 26857698 PMC5099203

[21] Tan EM Cohen AS Fries JF Masi AT McShane DJ Rothfield NF. The 1982 revised criteria for the classification of systemic lupus erythematosus. Arthritis Rheum. (1982) 25:1271–7. 10.1002/art.1780251101 7138600

[22] Vitali C Bombardieri S Jonsson R Moutsopoulos HM Alexander EL Carsons SE. Classification criteria for Sjogren's syndrome: a revised version of the European criteria proposed by the American-European Consensus Group. Ann Rheum Dis. (2002) 61:554–8. 10.1136/ard.61.6.554 12006334 PMC1754137

[23] Bibikova M Barnes B Tsan C Ho V Klotzle B Le JM. High density DNA methylation array with single CpG site resolution. Genomics. (2011) 98:288–95. 10.1016/j.ygeno.2011.07.007 21839163

[24] Houseman EA Accomando WP Koestler DC Christensen BC Marsit CJ Nelson HH. DNA methylation arrays as surrogate measures of cell mixture distribution. BMC Bioinformatics. (2012) 13:86. 10.1186/1471-2105-13-86 22568884 PMC3532182

[25] Aryee MJ Jaffe AE Corrada-Bravo H Ladd-Acosta C Feinberg AP Hansen KD. Minfi: a flexible and comprehensive Bioconductor package for the analysis of Infinium DNA methylation microarrays. Bioinformatics. (2014) 30:1363–9. 10.1093/bioinformatics/btu049 24478339 PMC4016708

[26] Grundberg E Meduri E Sandling JK Hedman AK Keildson S Buil A. Global analysis of DNA methylation variation in adipose tissue from twins reveals links to disease-associated variants in distal regulatory elements. Am J Hum Genet. (2013) 93:876–90. 10.1016/j.ajhg.2013.11.003 24183450 PMC3824131

[27] Rusinova I Forster S Yu S Kannan A Masse M Cumming H. Interferome v2.0: an updated database of annotated interferon-regulated genes. Nucleic Acids Res. (2013) 41(Database issue):D1040–6. 10.1093/nar/gks1215 23203888 PMC3531205

[28] Bernstein BE Stamatoyannopoulos JA Costello JF Ren B Milosavljevic A Meissner A. The NIH roadmap epigenomics mapping consortium. Nat Biotechnol. (2010) 28:1045–8. 10.1038/nbt1010-1045 20944595 PMC3607281

[29] Chen J Bardes EE Aronow BJ Jegga AG. ToppGene Suite for gene list enrichment analysis and candidate gene prioritization. Nucleic Acids Res. (2009) 37(Web Server issue):W305–11. 10.1093/nar/gkp427 19465376 PMC2703978

[30] Breiman L Random forest. Mach Learn. (2001) 45:5–32. 10.1023/A:1010933404324

[31] Almlöf JC Alexsson A Imgenberg-Kreuz J Sylwan L Bäcklin C Leonard D. Novel risk genes for systemic lupus erythematosus predicted by random forest classification. Sci Rep. (2017) 7:6236. 10.1038/s41598-017-06516-1 28740209 PMC5524838

[32] Bäcklin CL Gustafsson MG Developer-friendly and computationally efficient predictive modeling without information leakage: the emil package for R. J Stat Softw. (2018) 85:1–30. 10.18637/jss.v085.i1330505247

[33] Liaw A Classification and regression by randomForest. R News. (2003) 3:18–22.

[34] Brkic Z Maria NI van Helden-Meeuwsen CG van de Merwe JP van Daele PL Dalm VA. Prevalence of interferon type I signature in CD14 monocytes of patients with Sjogren's syndrome and association with disease activity and BAFF gene expression. Ann Rheum Dis. (2013) 72:728–35. 10.1136/annrheumdis-2012-201381 22736090 PMC3618683

[35] Brdicková N Brdicka T Angelisová P Horváth O Spicka J Hilgert I. LIME: a new membrane Raft-associated adaptor protein involved in CD4 and CD8 coreceptor signaling. J Exp Med. (2003) 198:1453–62. 10.1084/jem.20031484 14610046 PMC2194115

[36] Ahn E Lee H Yun Y. LIME acts as a transmembrane adapter mediating BCR-dependent B-cell activation. Blood. (2006) 107:1521–7. 10.1182/blood-2005-05-1859 16249387

[37] Heink S Ludwig D Kloetzel PM Krüger E. IFN-gamma-induced immune adaptation of the proteasome system is an accelerated and transient response. Proc Natl Acad Sci USA. (2005) 102:9241–6. 10.1073/pnas.0501711102 15944226 PMC1166598

[38] Bird AK Meednu N Anolik JH. New insights into B cell biology in systemic lupus erythematosus and Sjogren's syndrome. Curr Opin Rheumatol. (2015) 27:461–7. 10.1097/BOR.0000000000000201 26164595 PMC4554755

[39] Pasoto SG Adriano de Oliveira Martins V Bonfa E. Sjogren's syndrome and systemic lupus erythematosus: links and risks. Open Access Rheumatol. (2019) 11:33–45. 10.2147/OARRR.S167783 30774485 PMC6357904

[40] Bodewes ILA Al-Ali S van Helden-Meeuwsen CG Maria NI Tarn J Lendrem DW. Systemic interferon type I and type II signatures in primary Sjogren's syndrome reveal differences in biological disease activity. Rheumatology. (2018) 57:921–30. 10.1093/rheumatology/kex490 29474655

[41] Gupta S Kaplan MJ. The role of neutrophils and NETosis in autoimmune and renal diseases. Nat Rev Nephrol. (2016) 12:402–13. 10.1038/nrneph.2016.71 27241241 PMC5510606

[42] Cuda CM Pope RM Perlman H. The inflammatory role of phagocyte apoptotic pathways in rheumatic diseases. Nat Rev Rheumatol. (2016) 12:543–58. 10.1038/nrrheum.2016.132 27549026 PMC5297631

[43] Imgenberg-Kreuz J Sandling JK Björk A Nordlund J Kvarnström M Eloranta ML. Transcription profiling of peripheral B cells in antibody-positive primary Sjogren's syndrome reveals upregulated expression of CX3CR1 and a type I and type II interferon signature. Scand J Immunol. (2018) 87:e12662. 10.1111/sji.12662 29655283

[44] Ferreira MA Vonk JM Baurecht H Marenholz I Tian C Hoffman JD. Shared genetic origin of asthma, hay fever and eczema elucidates allergic disease biology. Nat Genet. (2017) 49:1752–7. 10.1038/ng.3985 29083406 PMC5989923

[45] Gorlova O Martin JE Rueda B Koeleman BP Ying J Teruel M. Identification of novel genetic markers associated with clinical phenotypes of systemic sclerosis through a genome-wide association strategy. PLoS Genet. (2011) 7:e1002178. 10.1371/annotation/7a52649c-0942-4bd8-a5d3-3cdacca03cd8 21779181 PMC3136437

[46] Thrane PS Halstensen TS Haanaes HR Brandtzaeg P. Increased epithelial expression of HLA-DQ and HLA-DP molecules in salivary glands from patients with Sjogren's syndrome compared with obstructive sialadenitis. Clin Exp Immunol. (1993) 92:256–62. 10.1111/j.1365-2249.1993.tb03389.x 8485911 PMC1554793

[47] Cole MB Quach H Quach D Baker A Taylor KE Barcellos LF. Epigenetic signatures of salivary gland inflammation in Sjogren's syndrome. Arthritis Rheumatol. (2016) 68:2936–44. 10.1002/art.39792 27332624 PMC5132022

[48] Basler M Kirk CJ Groettrup M. The immunoproteasome in antigen processing and other immunological functions. Curr Opin Immunol. (2013) 25:74–80. 10.1016/j.coi.2012.11.004 23219269

[49] Egerer T Martinez-Gamboa L Dankof A Stuhlmüller B Dörner T Krenn V. Tissue-specific up-regulation of the proteasome subunit beta5i (LMP7) in Sjogren's syndrome. Arthritis Rheum. (2006) 54:1501–8. 10.1002/art.21782 16646031

[50] Morawietz L Martinez-Gamboa L Scheffler S Hausdorf G Dankof A Kuckelkorn U. Expression of proteasomal immunosubunit beta1i is dysregulated in inflammatory infiltrates of minor salivary glands in Sjogren's syndrome. J Rheumatol. (2009) 36:2694–703. 10.3899/jrheum.081098 19833746

[51] Krause S Kuckelkorn U Dörner T Burmester GR Feist E Kloetzel PM Immunoproteasome subunit LMP2 expression is deregulated in Sjogren's syndrome but not in other autoimmune disorders. Ann Rheum Dis. (2006) 65:1021–7. 10.1136/ard.2005.045930 16414974 PMC1798250

[52] Arellano-Garcia ME Misuno K Tran SD Hu S. Interferon-gamma induces immunoproteasomes and the presentation of MHC I-associated peptides on human salivary gland cells. PLoS ONE. (2014) 9:e102878. 10.1371/journal.pone.0102878 25102056 PMC4125149

[53] Basler M Mundt S Bitzer A Schmidt C Groettrup M. The immunoproteasome: a novel drug target for autoimmune diseases. Clin Exp Rheumatol. (2015) 33(4 Suppl 92):S74–9. 26458097

[54] Verbrugge SE Scheper RJ Lems WF de Gruijl TD Jansen G. Proteasome inhibitors as experimental therapeutics of autoimmune diseases. Arthritis Res Ther. (2015) 17:17. 10.1186/s13075-015-0529-1 25889583 PMC4308859

[55] Manoussakis MN Georgopoulou C Zintzaras E Spyropoulou M Stavropoulou A Skopouli FN. Sjogren's syndrome associated with systemic lupus erythematosus: clinical and laboratory profiles and comparison with primary Sjogren's syndrome. Arthritis Rheum. (2004) 50:882–91. 10.1002/art.20093 15022331

[56] Figueroa ME Lugthart S Li Y Erpelinck-Verschueren C Deng X Christos PJ. DNA methylation signatures identify biologically distinct subtypes in acute myeloid leukemia. Cancer Cell. (2010) 17:13–27. 10.1016/j.ccr.2009.11.020 20060365 PMC3008568

[57] Nordlund J Bäcklin CL Zachariadis V Cavelier L Dahlberg J Öfverholm I. DNA methylation-based subtype prediction for pediatric acute lymphoblastic leukemia. Clin Epigenetics. (2015) 7:11. 10.1186/s13148-014-0039-z 25729447 PMC4343276

[58] Olar A Wani KM Wilson CD Zadeh G DeMonte F Jones DT. Global epigenetic profiling identifies methylation subgroups associated with recurrence-free survival in meningioma. Acta Neuropathol. (2017) 133:431–44. 10.1007/s00401-017-1678-x 28130639 PMC5600514

[59] Capper D Jones DTW Sill M Hovestadt V Schrimpf D Sturm D. DNA methylation-based classification of central nervous system tumours. Nature. (2018) 555:469–74. 10.1038/nature26000 29539639 PMC6093218

