# Supplementary material for: Shared and Unique Patterns of DNA Methylation in Systemic Lupus Erythematosus and Primary Sjögren's Syndrome
Source: Front Immunol. 2019 Jul 30;10:1686. doi: 10.3389/fimmu.2019.01686 (PMC6688520; doi:10.3389/fimmu.2019.01686)
Supplement: Supplementary file 1 [file Table_1.pdf]

Supplementary Table S1 *Differentially methylated CpG sites (DMCs; n=7,626) in the SLE case-ctrl EWAS (p<1.3E-7, $|\Delta\beta|$>0.05) and their results in the pSS case-ctrl EWAS. DMCs with shared association in the pSS case-ctrl EWAS (p<6.6E-6 and same direction of effect; n=4,725) are indicated in italics. DMCs uniquely associated with SLE (p>0.05 in the pSS case-ctrl EWAS; n=620) are indicated in bold.*

CpG site	Chromosome	Position	p-value SLE-ctrl	Mean β SLE	Mean β ctrl	Methylation Δβ SLE-ctrl	Gene	p-value pSS-ctrl	Methylation Δβ pSS-ctrl
cg17087974	11	65171397	2.35E-214	0.292	0.380	-0.088	*FRMD8*	1.67E-29	-0.033
cg25505880	11	62574338	1.87E-210	0.306	0.410	-0.103	*STX5*	1.33E-37	-0.049
cg10313337	16	68823690	3.29E-209	0.369	0.483	-0.114	*CDH1*	5.09E-23	-0.041
cg08628635	6	20483859	8.69E-208	0.354	0.482	-0.129	*E2F3*	1.77E-28	-0.055
cg10718056	6	28884599	1.34E-203	0.321	0.434	-0.113	*TRIM27*	3.17E-34	-0.054
cg11193767	4	83930905	3.67E-203	0.315	0.417	-0.102	*LIN54*	3.98E-23	-0.040
cg25444339	7	75194698	2.30E-201	0.294	0.394	-0.101	*HIP1*	8.76E-32	-0.045
cg04430911	1	36914349	4.57E-201	0.330	0.421	-0.092	*OSCP1*	4.03E-28	-0.034
cg17833106	1	161060000	1.21E-200	0.369	0.465	-0.097	*PVRL4*	8.86E-31	-0.045
cg04730794	5	169144438	2.27E-200	0.414	0.550	-0.136	*DOCK2*	7.06E-25	-0.052
cg20686403	22	38438206	2.40E-200	0.329	0.420	-0.090	NA	6.92E-16	-0.028
cg07714276	6	7169632	1.52E-199	0.279	0.370	-0.091	*RREB1*	1.29E-32	-0.041
cg08287940	2	160491979	3.13E-198	0.442	0.558	-0.116	NA	2.64E-25	-0.044
cg03082779	20	45947025	5.14E-195	0.263	0.341	-0.078	*ZMYND8*	5.80E-24	-0.031
cg16640599	4	119732131	7.57E-195	0.325	0.437	-0.112	*SEC24D*	3.84E-28	-0.050
cg11313468	19	41782183	6.68E-194	0.374	0.492	-0.118	*HNRNPUL1*	2.65E-25	-0.054
cg09010699	3	195171693	8.57E-194	0.431	0.558	-0.126	NA	5.13E-10	-0.030
cg10667895	2	102577876	5.02E-193	0.320	0.408	-0.088	NA	2.23E-25	-0.036
cg16452651	21	35016873	1.78E-191	0.365	0.480	-0.116	*ITSN1*	5.66E-26	-0.052
cg18038894	17	48506385	2.42E-191	0.420	0.536	-0.116	*ACSF2*	3.70E-23	-0.046
cg22381196	16	72041376	3.09E-191	0.384	0.502	-0.118	*DHODH*	1.27E-19	-0.042
cg03889044	2	242802099	2.94E-190	0.685	0.594	0.091	*PDCD1*	5.34E-18	0.032
cg25305879	2	106814630	3.84E-190	0.389	0.505	-0.116	NA	1.80E-20	-0.043
cg24427660	11	818892	2.60E-189	0.329	0.430	-0.101	*PNPLA2*	6.95E-20	-0.037
cg10511985	1	24053853	2.13E-187	0.326	0.416	-0.089	NA	1.92E-16	-0.028
cg03857198	3	152927597	4.65E-187	0.358	0.470	-0.112	NA	7.78E-22	-0.042
cg12182708	10	99223744	3.38E-186	0.333	0.447	-0.114	*MMS19*	5.15E-25	-0.048
cg24967811	12	123503709	1.24E-185	0.301	0.420	-0.119	*PITPNM2*	3.29E-25	-0.051
cg14742445	22	30195299	8.95E-185	0.312	0.403	-0.091	*ASCC2*	2.21E-25	-0.037
cg19739596	11	59824161	1.53E-184	0.359	0.469	-0.110	*MS4A3*	1.24E-30	-0.053
cg11254053	1	33497125	1.68E-184	0.360	0.475	-0.115	*AK2*	3.05E-23	-0.047
cg01406317	16	4397291	3.69E-184	0.330	0.441	-0.110	*PAM16*	1.31E-23	-0.046
cg21991396	1	247581417	5.82E-184	0.396	0.521	-0.125	*NLRP3*	4.22E-27	-0.057
cg27230882	1	110976749	9.12E-184	0.335	0.447	-0.112	NA	3.61E-28	-0.053
cg04831327	12	114299076	1.06E-183	0.373	0.477	-0.103	*RBM19*	3.03E-23	-0.044
cg25600606	11	33308345	1.68E-183	0.476	0.611	-0.136	*HIPK3*	3.70E-17	-0.045
cg21854332	1	144989624	4.07E-183	0.270	0.365	-0.096	*PDE4DIP*	2.22E-27	-0.043
cg25757820	2	224819307	6.52E-183	0.460	0.587	-0.127	NA	6.87E-13	-0.037
cg06070324	15	75322058	8.12E-183	0.271	0.364	-0.093	*PPCDC*	2.47E-25	-0.040
cg06851151	5	133436368	9.94E-183	0.623	0.545	0.077	NA	1.70E-23	0.029
cg21882356	14	21153451	1.59E-182	0.400	0.497	-0.097	*ANG*	6.27E-28	-0.044
cg05642546	7	37298927	9.78E-182	0.323	0.448	-0.124	*ELMO1*	4.99E-26	-0.054
cg00501765	1	172410217	1.12E-181	0.410	0.506	-0.096	*C1orf105*	1.71E-18	-0.035
cg08622198	1	239979505	1.58E-181	0.349	0.444	-0.095	*CHRM3*	1.31E-18	-0.034
cg21640432	19	35939561	5.55E-181	0.256	0.344	-0.088	*FFAR2*	1.63E-23	-0.039
cg09470754	19	55838021	8.46E-181	0.301	0.391	-0.090	*TMEM150B*	4.35E-19	-0.033
cg25739938	2	9610621	9.59E-181	0.451	0.572	-0.121	*CPSF3*	1.21E-18	-0.042
cg04071967	17	53511321	1.77E-180	0.314	0.411	-0.097	NA	3.83E-21	-0.040
cg08688659	11	35249965	1.90E-180	0.351	0.470	-0.119	*CD44*	7.39E-19	-0.047
cg07356342	1	161183820	3.42E-180	0.332	0.428	-0.096	*NDUFS2*	8.71E-20	-0.037
cg23772226	22	29225779	3.84E-180	0.321	0.435	-0.114	NA	1.06E-30	-0.058
cg13633625	9	114706971	4.22E-180	0.401	0.530	-0.129	NA	2.17E-16	-0.046
cg23302989	7	25174492	6.17E-180	0.422	0.542	-0.120	*C7orf31*	6.45E-17	-0.043
cg13178361	1	162532502	7.32E-180	0.402	0.513	-0.112	*UAP1*	2.56E-21	-0.047
cg04484126	7	65839403	8.69E-180	0.344	0.453	-0.109	NA	7.16E-26	-0.045
cg08154963	17	33426885	2.06E-179	0.370	0.478	-0.107	*RAD51L3-RFFL*	9.00E-14	-0.035
cg08539965	1	21396338	2.19E-179	0.368	0.492	-0.125	*EIF4G3*	3.06E-14	-0.041
cg20241876	6	32180045	2.49E-179	0.316	0.393	-0.077	*NOTCH4*	7.93E-16	-0.025
cg22713444	1	145512660	2.61E-179	0.332	0.451	-0.118	*RBM8A*	2.00E-16	-0.041
cg17980508	1	79085713	3.77E-179	0.216	0.356	-0.140	*IFI44L*	2.50E-27	-0.061
cg07733719	7	75683547	5.86E-179	0.347	0.438	-0.091	*MDH2*	1.44E-23	-0.038
cg07986469	10	129795003	1.40E-178	0.349	0.441	-0.093	*PTPRE*	9.97E-19	-0.033
cg05455036	1	202828149	1.60E-178	0.308	0.402	-0.094	NA	1.48E-34	-0.051
cg05453411	16	89300123	1.74E-178	0.254	0.324	-0.070	NA	4.40E-11	-0.019
cg17756730	1	172608644	1.08E-177	0.733	0.644	0.088	NA	5.26E-19	0.035
cg01268901	12	1744957	1.73E-177	0.321	0.423	-0.102	*WNT5B*	5.39E-25	-0.045

cg14919455	6	7167468	1.84E-177	0.357	0.458	-0.101	*RREB1*	2.31E-14	-0.031
cg20609803	1	161184305	2.54E-177	0.320	0.427	-0.106	*FCER1G*	4.77E-25	-0.049
cg24420366	10	3938541	6.17E-177	0.408	0.530	-0.121	NA	1.89E-20	-0.050
cg08764162	10	31147088	6.46E-177	0.365	0.489	-0.124	*ZNF438*	4.62E-25	-0.058
cg06270401	12	4699085	6.61E-177	0.372	0.484	-0.113	*DYRK4*	2.46E-19	-0.044
cg00483030	17	77810509	1.22E-176	0.305	0.401	-0.096	*CBX4*	1.45E-26	-0.043
cg03568673	13	20796216	1.78E-176	0.379	0.503	-0.125	*GJB6*	3.55E-23	-0.052
cg12433559	10	72362730	1.87E-176	0.712	0.634	0.078	*PRF1*	8.44E-23	0.033
cg02341503	20	45947123	2.57E-176	0.313	0.386	-0.073	*ZMYND8*	1.37E-15	-0.025
cg05904013	7	128579933	3.59E-176	0.331	0.418	-0.087	*IRF5*	1.06E-16	-0.031
cg13152690	14	91695017	4.38E-176	0.732	0.642	0.090	NA	1.59E-17	0.033
cg18920397	1	160765805	5.37E-176	0.678	0.587	0.090	*LY9*	3.70E-22	0.034
cg18313182	11	818903	5.71E-176	0.364	0.443	-0.079	*PNPLA2*	1.64E-14	-0.024
cg22805603	2	32490766	7.55E-176	0.389	0.481	-0.092	*NLRC4*	5.64E-14	-0.029
cg00340958	20	48328658	1.54E-175	0.373	0.485	-0.112	*B4GALT5*	6.41E-20	-0.046
cg21602257	20	44538675	2.36E-175	0.351	0.451	-0.100	*PLTP*	4.94E-26	-0.042
cg23990942	19	40946878	3.51E-175	0.365	0.444	-0.079	*SERTAD3*	2.13E-23	-0.033
cg26434370	16	10909361	4.74E-175	0.362	0.466	-0.104	*FAM18A*	1.29E-25	-0.046
cg07033722	1	40539032	1.00E-174	0.375	0.499	-0.125	*PPT1*	1.94E-23	-0.052
cg04686354	17	73261880	1.12E-174	0.413	0.507	-0.093	*MRPS7*	1.31E-22	-0.035
cg15015109	17	65136480	1.14E-174	0.363	0.467	-0.103	*HELZ*	1.92E-18	-0.039
cg06298740	1	227125826	1.85E-174	0.312	0.421	-0.109	NA	1.44E-28	-0.055
cg19584038	19	3553032	7.13E-174	0.269	0.344	-0.075	*MFSD12*	1.05E-32	-0.039
cg14649507	2	231735037	8.47E-174	0.278	0.361	-0.083	*ITM2C*	1.08E-25	-0.037
cg17852482	17	80833054	1.19E-173	0.373	0.475	-0.102	*TBCD*	1.29E-12	-0.033
cg15545247	12	123201372	3.75E-173	0.377	0.496	-0.119	*HCAR3*	9.26E-29	-0.056
cg03022510	2	192476634	4.47E-173	0.312	0.401	-0.090	NA	1.14E-15	-0.031
cg22501942	1	11798278	7.35E-173	0.253	0.332	-0.080	*AGTRAP*	8.35E-30	-0.041
cg26233331	4	6695614	9.13E-173	0.276	0.366	-0.090	*S100P*	4.92E-22	-0.037
cg06257058	7	99683264	1.88E-172	0.350	0.471	-0.121	NA	6.89E-26	-0.056
cg02331303	11	67777823	2.43E-172	0.311	0.401	-0.090	*ALDH3B1*	6.44E-30	-0.043
cg02052762	17	4090525	2.75E-172	0.399	0.534	-0.136	*ANKFY1*	5.78E-23	-0.055
cg24414363	22	42336273	2.84E-172	0.668	0.584	0.083	*CENPM*	9.54E-10	0.020
cg01699630	6	131894023	3.54E-172	0.439	0.558	-0.120	*ARG1*	1.74E-10	-0.032
cg13086983	1	21664810	5.90E-172	0.310	0.412	-0.102	*ECE1*	9.62E-27	-0.048
cg20611272	8	103548145	1.67E-171	0.742	0.651	0.091	NA	1.86E-17	0.036
cg10456459	12	22843015	2.82E-171	0.384	0.507	-0.123	*ETNK1*	9.63E-13	-0.036
cg03739609	6	31555016	8.24E-171	0.352	0.461	-0.108	*LST1*	5.88E-29	-0.055
cg18915856	14	21483641	1.66E-170	0.333	0.435	-0.102	NA	5.86E-24	-0.048
cg05627557	2	37418009	2.62E-170	0.295	0.377	-0.082	NA	3.68E-16	-0.031
cg03945538	1	173447427	4.21E-170	0.320	0.415	-0.095	*PRDX6*	9.31E-34	-0.050
cg17837191	17	62318673	7.47E-170	0.379	0.497	-0.118	*TEX2*	3.20E-20	-0.049
cg15994604	11	34676683	1.10E-169	0.287	0.391	-0.104	*EHF*	5.15E-29	-0.055
cg10264529	14	24562064	1.13E-169	0.435	0.527	-0.092	*PCK2*	1.89E-21	-0.040
cg21249659	12	10324843	2.46E-169	0.335	0.446	-0.111	*OLR1*	5.04E-27	-0.050
cg00026033	12	56414490	3.82E-169	0.298	0.380	-0.083	*IKZF4*	3.03E-17	-0.028
cg11357013	15	73588054	1.34E-168	0.335	0.449	-0.115	*NEO1*	4.67E-21	-0.050
cg19351604	8	1870722	1.74E-168	0.411	0.534	-0.122	*ARHGEF10*	1.51E-26	-0.057
cg21341487	20	60760919	2.11E-168	0.331	0.427	-0.097	*GTPBP5*	5.87E-19	-0.037
cg11014468	3	53362965	4.96E-168	0.431	0.528	-0.097	*DCP1A*	1.57E-17	-0.036
cg03998264	12	53932355	5.74E-168	0.290	0.401	-0.111	*ATF7*	3.09E-18	-0.046
cg07917901	14	35870184	5.86E-168	0.330	0.446	-0.117	NA	1.36E-18	-0.046
cg02057747	14	69408849	7.97E-168	0.290	0.357	-0.067	*ACTN1*	8.19E-18	-0.025
cg07472373	10	50595181	1.00E-167	0.376	0.507	-0.130	*DRGX*	5.58E-17	-0.050
cg05928849	11	122993208	1.57E-167	0.379	0.483	-0.104	*CLMP*	7.86E-15	-0.037
cg23445003	7	101535821	2.36E-167	0.344	0.460	-0.116	*CUX1*	6.52E-18	-0.047
cg16449084	11	67069814	3.32E-167	0.314	0.407	-0.093	*SSH3*	5.40E-19	-0.036
cg26831416	19	39142011	4.97E-167	0.298	0.393	-0.094	*ACTN4*	1.34E-25	-0.040
cg23114435	1	167442612	5.43E-167	0.692	0.597	0.095	*CD247*	1.11E-22	0.040
cg24909660	11	47276469	7.11E-167	0.289	0.377	-0.088	*NR1H3*	1.28E-25	-0.040
cg11690666	17	80415469	7.51E-167	0.475	0.582	-0.107	*NARF*	1.74E-20	-0.041
cg16651347	1	175042297	1.06E-166	0.314	0.404	-0.090	*TNN*	1.49E-18	-0.032
cg02319986	19	8568712	2.71E-166	0.348	0.445	-0.097	*PRAM1*	1.09E-09	-0.027
cg03801286	21	35884508	3.17E-166	0.307	0.402	-0.095	*KCNE1*	6.89E-22	-0.040
cg16163847	13	52165356	3.60E-166	0.362	0.474	-0.112	*WDFY2*	1.58E-25	-0.053
cg21702188	11	62574403	4.15E-166	0.257	0.344	-0.087	*STX5*	5.05E-28	-0.045
cg08850243	6	30169768	6.34E-166	0.262	0.365	-0.102	*TRIM26*	8.52E-20	-0.040
cg26443127	10	31987230	8.06E-166	0.362	0.453	-0.091	NA	3.53E-19	-0.035
cg13595556	9	129648322	2.18E-165	0.424	0.546	-0.122	NA	6.61E-12	-0.037
cg05991009	2	102080674	2.78E-165	0.347	0.459	-0.112	*RFX8*	2.13E-22	-0.048
cg18084554	19	929046	4.45E-165	0.313	0.394	-0.081	*ARID3A*	3.55E-19	-0.031
cg08363067	16	16170085	4.92E-165	0.315	0.401	-0.086	*ABCC1*	2.34E-20	-0.034
cg02226192	16	89461734	8.49E-165	0.393	0.484	-0.091	*ANKRD11*	1.06E-06	-0.018

cg12058064	15	86315201	8.68E-165	0.369	0.454	-0.085	*KLHL25*	6.13E-15	-0.028
cg05467716	17	62774079	9.76E-165	0.312	0.402	-0.090	*LOC146880*	2.34E-29	-0.046
cg20636248	10	121439845	1.47E-164	0.344	0.443	-0.099	NA	2.16E-19	-0.038
cg26341831	1	226036279	1.76E-164	0.679	0.579	0.100	*TMEM63A*	8.57E-06	0.018
cg04353769	11	59951557	5.49E-164	0.303	0.387	-0.084	*MS4A6A*	1.19E-26	-0.039
cg26298914	14	68798365	5.57E-164	0.375	0.502	-0.127	*RAD51B*	6.69E-10	-0.036
cg15412772	17	38175389	1.02E-163	0.468	0.555	-0.087	*MED24*	2.59E-13	-0.027
cg05954120	1	156254757	2.20E-163	0.334	0.426	-0.092	*TMEM79*	1.46E-20	-0.038
cg02806156	12	89728752	2.48E-163	0.358	0.480	-0.122	NA	9.68E-14	-0.042
cg05753799	20	3746315	3.70E-163	0.287	0.369	-0.082	*C20orf27*	6.71E-20	-0.035
cg14112356	21	46348443	3.99E-163	0.654	0.567	0.087	*ITGB2-AS1*	2.68E-12	0.027
cg24448340	1	179921042	4.13E-163	0.301	0.414	-0.113	NA	2.43E-28	-0.060
cg25634666	11	71846788	6.12E-163	0.293	0.384	-0.091	*FOLR3*	7.21E-16	-0.033
cg24475517	2	118737667	7.00E-163	0.379	0.491	-0.112	*CCDC93*	1.89E-22	-0.050
cg02012974	3	66492992	7.87E-163	0.331	0.443	-0.112	*LRIG1*	8.38E-28	-0.056
cg20720686	7	75582881	1.05E-162	0.456	0.560	-0.104	*POR*	2.75E-16	-0.039
cg23054181	15	99048945	1.33E-162	0.405	0.523	-0.117	*FAM169B*	5.96E-16	-0.039
cg19123356	8	130898833	1.92E-162	0.706	0.615	0.091	*FAM49B*	5.00E-15	0.032
cg23670353	1	59833489	5.18E-162	0.306	0.421	-0.115	*FGGY*	4.40E-16	-0.046
cg01374870	6	32905127	6.58E-162	0.328	0.437	-0.110	*HLA-DMB*	3.15E-20	-0.048
cg18150584	1	23887816	9.90E-162	0.254	0.349	-0.095	NA	2.70E-29	-0.051
cg00066854	10	113987376	1.51E-161	0.306	0.402	-0.096	NA	3.58E-17	-0.038
cg02856190	11	94282491	2.91E-161	0.317	0.424	-0.107	*FUT4*	3.38E-23	-0.051
cg04747180	4	39116218	3.77E-161	0.351	0.470	-0.119	*KLHL5*	2.91E-20	-0.050
cg05380759	3	45579354	5.44E-161	0.316	0.409	-0.094	*LARS2*	4.34E-15	-0.035
cg02607972	2	25964061	7.33E-161	0.396	0.511	-0.115	*ASXL2*	6.09E-19	-0.046
cg00278392	6	16802505	7.99E-161	0.324	0.439	-0.115	NA	3.51E-13	-0.039
cg01479396	17	7529693	8.40E-161	0.253	0.348	-0.096	*SAT2*	1.08E-17	-0.035
cg02138358	17	56358318	2.76E-160	0.324	0.400	-0.075	*MPO*	4.57E-17	-0.029
cg04993279	1	8940460	2.96E-160	0.333	0.438	-0.104	*ENO1*	1.04E-20	-0.046
cg08180028	15	41796044	3.19E-160	0.382	0.456	-0.074	*LTK*	5.26E-13	-0.024
cg23327851	14	21423684	3.29E-160	0.310	0.385	-0.075	*RNASE2*	5.70E-15	-0.028
cg26701826	4	108814604	3.94E-160	0.314	0.413	-0.099	*SGMS2*	6.02E-16	-0.039
cg26065247	7	37734052	4.02E-160	0.288	0.363	-0.075	NA	6.23E-28	-0.038
cg17322655	2	242802127	5.79E-160	0.691	0.604	0.086	*PDCD1*	2.04E-14	0.030
cg17932662	1	202123442	9.02E-160	0.393	0.501	-0.107	*PTPN7*	2.56E-12	-0.033
cg00409104	17	8762014	1.64E-159	0.275	0.358	-0.083	*PIK3R6*	2.90E-22	-0.036
cg19573490	17	79870317	4.48E-159	0.333	0.443	-0.111	*PCYT2*	2.68E-17	-0.046
cg24941342	11	95093809	9.22E-159	0.305	0.419	-0.114	NA	2.40E-19	-0.049
cg14654385	11	63973006	1.16E-158	0.350	0.437	-0.087	*FERMT3*	8.15E-19	-0.033
cg04855678	3	195946921	1.52E-158	0.272	0.368	-0.095	*SLC51A*	1.08E-16	-0.036
cg07102397	14	89864727	2.37E-158	0.478	0.594	-0.115	*FOXN3*	1.85E-10	-0.030
cg16786178	14	75764329	2.39E-158	0.291	0.386	-0.095	NA	1.14E-22	-0.046
cg10533990	11	71846733	3.54E-158	0.301	0.388	-0.087	*FOLR3*	1.94E-23	-0.040
cg08557970	6	166856094	6.39E-158	0.383	0.494	-0.110	*RPS6KA2*	2.40E-25	-0.053
cg21170682	11	122205382	8.17E-158	0.356	0.462	-0.106	NA	3.66E-26	-0.049
cg09145126	13	50570018	9.69E-158	0.375	0.497	-0.121	*DLEU2*	7.58E-21	-0.050
cg21221263	2	134933179	1.30E-157	0.324	0.425	-0.101	NA	9.05E-16	-0.037
cg05919238	17	79244158	1.83E-157	0.546	0.669	-0.123	*SLC38A10*	6.39E-18	-0.046
cg24637417	12	51636921	2.43E-157	0.286	0.387	-0.101	*DAZAP2*	3.92E-17	-0.040
cg19055828	12	51139321	3.64E-157	0.396	0.516	-0.120	*DIP2B*	6.10E-07	-0.027
cg06981309	3	146260954	4.93E-157	0.304	0.540	-0.237	*PLSCR1*	4.10E-51	-0.149
cg13870520	4	77131705	6.39E-157	0.370	0.479	-0.109	*SCARB2*	3.81E-21	-0.052
cg10718614	1	234914480	1.29E-156	0.255	0.328	-0.074	NA	2.21E-15	-0.026
cg02863947	3	119499190	1.50E-156	0.336	0.434	-0.098	*NR1I2*	5.03E-19	-0.041
cg10271819	7	139426030	1.85E-156	0.302	0.387	-0.086	*HIPK2*	2.63E-22	-0.039
cg10487428	5	59559218	1.97E-156	0.402	0.507	-0.104	*PDE4D*	7.79E-09	-0.030
cg15033269	3	42631489	2.12E-156	0.359	0.474	-0.115	*SEC22C*	2.49E-18	-0.047
cg21578596	6	32906460	2.42E-156	0.271	0.364	-0.093	*HLA-DMB*	1.02E-21	-0.043
cg12485428	17	1549098	2.84E-156	0.326	0.410	-0.084	*SCARF1*	5.48E-24	-0.038
cg00951869	14	24805349	2.88E-156	0.263	0.336	-0.074	*RIPK3*	2.69E-30	-0.041
cg00774102	6	31590870	3.00E-156	0.333	0.419	-0.086	*PRRC2A*	1.30E-21	-0.038
cg04569651	5	177956199	3.10E-156	0.337	0.416	-0.079	*COL23A1*	1.14E-22	-0.034
cg05000446	9	35042395	3.19E-156	0.423	0.553	-0.129	*C9orf131*	9.61E-13	-0.043
cg04609694	6	44204009	4.27E-156	0.409	0.530	-0.120	NA	1.92E-11	-0.036
cg16125725	15	70101302	4.57E-156	0.411	0.544	-0.133	NA	1.45E-13	-0.043
cg14414154	21	27538021	5.53E-156	0.356	0.475	-0.119	*APP*	1.08E-16	-0.049
cg05296619	13	114834683	7.91E-156	0.377	0.471	-0.094	*RASA3*	5.92E-15	-0.033
cg11608958	2	207836769	9.04E-156	0.400	0.489	-0.089	NA	3.69E-20	-0.039
cg09109411	5	149319112	1.23E-155	0.372	0.473	-0.101	*PDE6A*	1.33E-22	-0.046
cg21519701	17	62252524	1.42E-155	0.312	0.435	-0.123	*TEX2*	9.75E-23	-0.059
cg26313511	3	125053815	1.75E-155	0.330	0.416	-0.086	*ZNF148*	3.01E-17	-0.033
cg03731797	19	42900540	2.02E-155	0.223	0.299	-0.076	NA	2.08E-26	-0.037

cg16256243	2	85648512	2.18E-155	0.381	0.483	-0.102	NA	1.19E-16	-0.041
cg24595152	3	43733831	2.29E-155	0.295	0.378	-0.082	*ABHD5*	1.35E-18	-0.031
cg03463948	11	122612858	2.83E-155	0.399	0.523	-0.124	*UBASH3B*	5.74E-12	-0.039
cg18065599	11	47968995	3.72E-155	0.278	0.364	-0.086	NA	4.30E-22	-0.040
cg15889847	14	65183532	3.97E-155	0.356	0.439	-0.083	*PLEKHG3*	6.02E-15	-0.029
cg14214797	14	91860613	4.92E-155	0.763	0.696	0.067	*CCDC88C*	8.10E-18	0.027
cg07599389	6	4890278	5.82E-155	0.289	0.377	-0.088	*CDYL*	2.22E-23	-0.039
cg14516183	3	122928186	6.09E-155	0.407	0.530	-0.123	*SEC22A*	1.85E-09	-0.034
cg05936004	3	130693828	8.01E-155	0.415	0.530	-0.115	*ATP2C1*	1.96E-16	-0.044
cg13753351	9	127134207	1.42E-154	0.347	0.464	-0.116	*PSMB7*	1.19E-15	-0.045
cg18659081	22	44588350	2.49E-154	0.350	0.466	-0.116	*PARVG*	3.92E-19	-0.050
cg20222562	6	113993995	2.80E-154	0.391	0.520	-0.129	NA	2.08E-26	-0.065
cg07708453	1	14032034	2.86E-154	0.342	0.454	-0.112	*PRDM2*	3.40E-07	-0.028
cg04958055	14	69404437	2.95E-154	0.251	0.325	-0.073	*ACTN1*	9.51E-14	-0.026
cg15135286	2	33359281	3.56E-154	0.376	0.480	-0.105	*LTBP1*	9.57E-18	-0.044
cg05454562	6	33254447	5.16E-154	0.409	0.511	-0.103	*WDR46*	2.42E-12	-0.033
cg25099490	14	23583133	5.41E-154	0.292	0.379	-0.087	NA	7.43E-16	-0.034
cg05322500	16	30824294	9.23E-154	0.198	0.264	-0.066	NA	1.21E-21	-0.031
cg03778909	17	80833393	1.53E-153	0.348	0.447	-0.099	*TBCD*	6.92E-18	-0.040
cg03689552	16	3292971	1.77E-153	0.333	0.443	-0.110	*MEFV*	1.16E-19	-0.046
cg03278564	13	49200201	1.83E-153	0.372	0.485	-0.113	NA	5.76E-16	-0.041
cg00812557	4	38073835	1.88E-153	0.255	0.330	-0.075	*TBC1D1*	1.96E-27	-0.040
cg26226650	3	50276265	1.95E-153	0.280	0.366	-0.086	*GNAI2*	4.23E-19	-0.037
cg01924292	12	1815334	2.02E-153	0.386	0.499	-0.112	*ADIPOR2*	1.62E-11	-0.037
cg00118808	9	132097931	2.52E-153	0.260	0.341	-0.081	NA	2.87E-21	-0.035
cg02115302	16	27237719	2.54E-153	0.798	0.732	0.066	*NSMCE1*	1.31E-08	0.018
cg15058645	2	175528343	2.58E-153	0.302	0.408	-0.106	*WIPF1*	2.94E-24	-0.054
cg07702424	11	12306515	3.24E-153	0.484	0.591	-0.107	NA	1.35E-13	-0.038
cg15275312	3	50363059	3.25E-153	0.255	0.347	-0.091	*TUSC2*	5.12E-23	-0.043
cg05569131	6	36665620	3.40E-153	0.304	0.384	-0.080	*RAB44*	1.52E-20	-0.034
cg16416987	1	155177561	5.12E-153	0.284	0.361	-0.078	*MTX1*	3.32E-22	-0.037
cg02435083	16	8943436	5.90E-153	0.293	0.385	-0.092	NA	7.92E-19	-0.038
cg25025545	6	14136628	7.02E-153	0.321	0.424	-0.103	*CD83*	5.44E-15	-0.040
cg06885823	5	82675005	7.30E-153	0.376	0.486	-0.110	NA	1.11E-15	-0.043
cg26164488	2	64440295	7.48E-153	0.277	0.382	-0.105	NA	2.80E-29	-0.058
cg11860005	22	32149125	1.11E-152	0.265	0.336	-0.071	*DEPDC5*	2.26E-26	-0.035
cg09635667	17	2300514	1.12E-152	0.327	0.409	-0.082	*MNT*	1.70E-14	-0.027
cg17385088	4	153611560	1.41E-152	0.398	0.489	-0.091	NA	1.55E-12	-0.032
cg07227049	2	58335008	1.45E-152	0.451	0.576	-0.124	*VRK2*	1.07E-14	-0.041
cg12517576	7	128048308	1.67E-152	0.276	0.367	-0.090	*IMPDH1*	5.21E-18	-0.037
cg13977235	19	33172072	1.75E-152	0.351	0.467	-0.115	NA	8.71E-13	-0.039
cg26514623	6	13295561	2.06E-152	0.448	0.565	-0.117	*LOC10013035*	5.06E-12	-0.036
cg01697902	14	25046117	2.39E-152	0.341	0.450	-0.109	*CTSG*	1.04E-14	-0.041
cg18059369	20	44543173	3.02E-152	0.202	0.279	-0.077	NA	3.64E-17	-0.033
cg20090290	11	46543695	3.41E-152	0.352	0.466	-0.114	*AMBRA1*	1.36E-14	-0.041
cg17056676	10	116301354	3.89E-152	0.660	0.581	0.079	*ABLIM1*	7.84E-21	0.034
cg22491058	1	207277466	6.29E-152	0.306	0.416	-0.110	*C4BPA*	2.43E-19	-0.048
cg17250262	2	30457110	6.44E-152	0.605	0.525	0.080	*LBH*	3.86E-18	0.029
cg24534743	1	27884345	6.50E-152	0.338	0.398	-0.060	*AHDC1*	1.90E-13	-0.020
cg11638399	8	29441416	6.60E-152	0.330	0.443	-0.114	NA	1.74E-21	-0.051
cg14028598	1	171714300	8.83E-152	0.465	0.590	-0.124	NA	1.18E-14	-0.042
cg17496887	1	153387707	9.51E-152	0.436	0.525	-0.090	*S100A7A*	1.77E-16	-0.035
cg11098259	15	58430391	1.14E-151	0.286	0.395	-0.109	*AQP9*	2.52E-20	-0.050
cg27307183	6	4890095	1.31E-151	0.314	0.418	-0.104	*CDYL*	1.80E-19	-0.047
cg16565031	14	70186289	1.35E-151	0.320	0.419	-0.099	NA	1.56E-17	-0.041
cg08900384	11	72546168	1.41E-151	0.290	0.364	-0.074	NA	2.35E-17	-0.029
cg18325315	7	73897230	2.13E-151	0.427	0.547	-0.121	*GTF2IRD1*	4.64E-14	-0.040
cg05501357	11	33308269	2.21E-151	0.365	0.472	-0.107	*HIPK3*	3.24E-09	-0.029
cg13053608	17	40345673	2.73E-151	0.291	0.379	-0.088	*GHDC*	3.21E-23	-0.041
cg12645852	18	74198774	2.95E-151	0.336	0.441	-0.105	*ZNF516*	9.51E-22	-0.048
cg13709496	9	101011718	4.77E-151	0.317	0.407	-0.090	*TBC1D2*	7.68E-17	-0.034
cg25930786	19	54584636	5.10E-151	0.325	0.427	-0.102	*TARM1*	1.81E-14	-0.041
cg08703231	16	8738304	5.15E-151	0.343	0.433	-0.090	*METTL22*	1.01E-17	-0.036
cg00405190	2	175545838	5.35E-151	0.332	0.443	-0.111	*WIPF1*	4.44E-18	-0.045
cg26746309	10	101943528	6.29E-151	0.259	0.356	-0.097	*ERLIN1*	2.80E-22	-0.049
cg21923525	18	9474143	6.41E-151	0.279	0.353	-0.074	*RALBP1*	1.02E-15	-0.029
cg03961551	1	25251730	8.68E-151	0.645	0.547	0.098	*RUNX3*	3.61E-08	0.023
cg16684117	5	148808456	1.11E-150	0.248	0.326	-0.078	*MIR143HG*	2.64E-12	-0.026
cg04487659	17	43510575	1.15E-150	0.278	0.352	-0.074	*ARHGAP27*	8.00E-36	-0.041
cg00980622	14	75884845	1.57E-150	0.413	0.538	-0.126	NA	1.87E-09	-0.034
cg10325497	1	32739049	1.57E-150	0.776	0.706	0.070	*LCK*	2.94E-32	0.039
cg23403980	17	2060623	1.79E-150	0.274	0.346	-0.072	*SMG6*	1.79E-22	-0.030
cg17291166	6	37051189	1.86E-150	0.451	0.540	-0.089	NA	1.17E-17	-0.037

cg03301498	19	39889222	2.01E-150	0.289	0.373	-0.084	*MED29*	1.67E-23	-0.041
cg26476925	19	45245446	2.80E-150	0.290	0.365	-0.075	NA	9.02E-13	-0.026
cg22228439	1	46766431	2.91E-150	0.329	0.411	-0.082	*LRRC41*	3.17E-15	-0.032
cg27123392	15	50570473	3.21E-150	0.419	0.531	-0.112	*GABPB1*	3.54E-19	-0.045
cg01558916	19	11804727	3.30E-150	0.331	0.434	-0.104	NA	1.73E-13	-0.038
cg23363263	2	224751989	3.53E-150	0.461	0.589	-0.128	*WDFY1*	9.13E-13	-0.042
cg10411221	6	32904317	4.28E-150	0.350	0.429	-0.078	*HLA-DMB*	7.34E-11	-0.024
cg06030535	5	158637684	6.25E-150	0.301	0.401	-0.100	*RNF145*	2.71E-13	-0.036
cg01087254	15	57052190	6.82E-150	0.295	0.412	-0.118	NA	8.56E-21	-0.055
cg19620994	1	12774904	1.66E-149	0.357	0.468	-0.111	*AADACL3*	2.40E-12	-0.037
cg02862467	1	19407897	2.03E-149	0.348	0.466	-0.117	*UBR4*	3.02E-15	-0.046
cg02505177	10	103574626	2.07E-149	0.685	0.600	0.085	*MGEA5*	1.39E-11	0.023
cg07285167	1	36948981	2.08E-149	0.282	0.388	-0.107	*CSF3R*	7.25E-18	-0.048
cg05436845	11	65378622	2.44E-149	0.330	0.416	-0.086	*MAP3K11*	8.07E-17	-0.030
cg08300570	7	92353454	3.29E-149	0.370	0.480	-0.110	*CDK6*	8.22E-17	-0.044
cg09303642	12	54690818	3.47E-149	0.339	0.442	-0.103	*NFE2*	4.27E-16	-0.042
cg04407063	11	67051977	5.05E-149	0.350	0.453	-0.103	*ADRBK1*	6.31E-24	-0.047
cg25647583	15	91427184	5.84E-149	0.353	0.436	-0.084	*FES*	1.03E-17	-0.033
cg19037107	11	134126323	7.63E-149	0.496	0.603	-0.107	*ACAD8*	2.26E-06	-0.024
cg25437886	3	45677346	8.85E-149	0.401	0.524	-0.123	*LIMD1*	2.86E-15	-0.048
cg06987246	7	6659785	1.23E-148	0.645	0.543	0.102	*ZNF853*	4.53E-15	0.037
cg08276042	16	2740348	1.23E-148	0.311	0.399	-0.088	*KCTD5*	9.74E-20	-0.035
cg23595304	11	46879516	1.59E-148	0.297	0.396	-0.099	*LRP4*	2.08E-24	-0.049
cg07512258	10	75619420	2.51E-148	0.776	0.702	0.074	*CAMK2G*	1.92E-13	0.028
cg04051365	3	193586394	2.53E-148	0.340	0.444	-0.104	NA	3.88E-17	-0.044
cg26776551	13	51944507	2.66E-148	0.293	0.386	-0.093	*INTS6*	1.23E-17	-0.039
cg07298177	16	85577847	3.23E-148	0.373	0.471	-0.098	NA	2.36E-18	-0.039
cg01044849	6	30002723	4.21E-148	0.473	0.572	-0.098	*ZNRD1-AS1*	3.26E-08	-0.025
cg05057534	2	28497669	6.31E-148	0.336	0.450	-0.114	*BRE*	2.31E-20	-0.058
cg18095675	3	197272311	6.48E-148	0.529	0.637	-0.108	*BDH1*	4.35E-11	-0.032
cg13008174	8	11640960	7.55E-148	0.267	0.339	-0.072	*NEIL2*	3.28E-23	-0.033
cg25140783	1	24861872	8.02E-148	0.674	0.560	0.114	*RCAN3*	5.55E-22	0.058
cg01323964	7	65219171	9.85E-148	0.397	0.516	-0.120	*CCT6P1*	1.40E-10	-0.036
cg14417099	6	150073879	9.87E-148	0.331	0.439	-0.108	*PCMT1*	1.74E-13	-0.038
cg24305156	11	45871936	1.27E-147	0.310	0.394	-0.084	*CRY2*	1.86E-16	-0.034
cg13345558	5	40796738	1.29E-147	0.427	0.517	-0.090	*PRKAA1*	1.65E-23	-0.043
cg01618151	6	35707957	2.42E-147	0.355	0.445	-0.090	*ARMC12*	2.41E-21	-0.040
cg15553418	1	16696547	2.63E-147	0.324	0.428	-0.104	*SZRD1*	5.77E-20	-0.046
cg26861460	22	44575455	2.68E-147	0.338	0.449	-0.111	*PARVG*	1.23E-20	-0.052
cg23672659	6	30648020	4.01E-147	0.703	0.629	0.074	*PPP1R18*	6.21E-14	0.026
cg21310090	20	44538669	6.13E-147	0.350	0.446	-0.097	*PLTP*	1.31E-16	-0.038
cg26837399	1	236209798	7.29E-147	0.345	0.458	-0.113	*NID1*	1.23E-17	-0.046
cg06690535	3	196352142	7.93E-147	0.321	0.416	-0.095	NA	8.82E-20	-0.044
cg05059480	15	40633202	8.33E-147	0.369	0.438	-0.069	*C15orf52*	1.21E-14	-0.023
cg21190228	2	240132342	8.99E-147	0.307	0.415	-0.108	*HDAC4*	2.16E-19	-0.050
cg24079727	15	57317980	1.01E-146	0.348	0.461	-0.113	*TCF12*	1.03E-18	-0.051
cg04674060	17	7792063	1.19E-146	0.618	0.535	0.082	*CHD3*	2.98E-17	0.033
cg09524686	11	829182	1.42E-146	0.276	0.350	-0.074	*EFCAB4A*	1.13E-16	-0.028
cg19619956	5	176967557	1.47E-146	0.442	0.539	-0.096	*FAM193B*	7.04E-12	-0.031
cg13995774	5	179189810	1.54E-146	0.327	0.438	-0.111	*MAML1*	3.78E-19	-0.044
cg24504349	4	120974344	1.89E-146	0.335	0.448	-0.113	NA	5.87E-15	-0.044
cg17734802	1	54358862	2.16E-146	0.342	0.449	-0.107	*DIO1*	1.37E-17	-0.046
cg06650246	1	206897270	2.24E-146	0.271	0.354	-0.083	*MAPKAPK2*	5.37E-08	-0.023
cg24553417	13	50244794	2.27E-146	0.378	0.491	-0.113	*EBPL*	3.03E-16	-0.042
cg10334489	17	25798878	2.43E-146	0.716	0.647	0.069	*KSR1*	8.28E-09	0.020
cg04972459	1	9460204	4.43E-146	0.311	0.391	-0.081	NA	5.22E-16	-0.035
cg10338518	4	87895137	5.06E-146	0.345	0.461	-0.117	*AFF1*	1.29E-17	-0.046
cg26001125	22	24823050	7.35E-146	0.745	0.678	0.066	*ADORA2A*	1.73E-09	0.020
cg02240622	15	40601467	8.13E-146	0.337	0.427	-0.089	*PLCB2*	1.67E-16	-0.036
cg08458487	10	81709191	9.11E-146	0.290	0.389	-0.099	*SFTPD*	3.67E-22	-0.047
cg16137928	6	30720491	9.71E-146	0.357	0.449	-0.092	NA	5.66E-22	-0.045
cg27461196	19	35630106	1.01E-145	0.302	0.376	-0.074	*FXYD1*	3.10E-17	-0.027
cg22164009	6	14729030	1.28E-145	0.303	0.397	-0.094	NA	4.56E-24	-0.045
cg12306086	4	106117747	1.49E-145	0.301	0.409	-0.108	*TET2*	1.78E-22	-0.056
cg05132828	12	123560479	3.09E-145	0.653	0.576	0.078	*PITPNM2*	1.76E-13	0.025
cg27260684	16	85063742	4.20E-145	0.359	0.468	-0.109	*KIAA0513*	4.31E-13	-0.041
cg18655369	19	1177575	4.21E-145	0.299	0.382	-0.083	NA	1.34E-18	-0.036
cg12299361	1	53796769	4.46E-145	0.328	0.420	-0.092	NA	2.10E-11	-0.026
cg14397918	9	72078829	5.33E-145	0.312	0.411	-0.099	*APBA1*	6.54E-16	-0.042
cg07495389	2	27233872	5.77E-145	0.309	0.401	-0.092	*MAPRE3*	7.38E-18	-0.040
cg27051231	2	232213012	5.96E-145	0.338	0.444	-0.105	NA	4.86E-16	-0.044
cg23280720	6	139483193	7.44E-145	0.708	0.614	0.094	*HECA*	6.52E-09	0.026
cg25103337	1	9293583	9.64E-145	0.347	0.459	-0.112	*H6PD*	9.78E-18	-0.048

cg13652008	2	33359356	1.17E-144	0.444	0.554	-0.110	*LTBP1*	3.56E-14	-0.041
cg01796438	3	11312864	1.40E-144	0.363	0.451	-0.088	*ATG7*	1.61E-15	-0.035
cg15125868	21	35746798	1.45E-144	0.299	0.394	-0.096	*FAM165B*	1.58E-15	-0.040
cg19676553	14	105751629	1.56E-144	0.386	0.472	-0.085	*BRF1*	5.72E-15	-0.032
cg02334333	2	60687392	1.56E-144	0.311	0.389	-0.078	*BCL11A*	8.79E-17	-0.034
cg14543285	14	103129037	1.74E-144	0.427	0.519	-0.092	*RCOR1*	1.05E-11	-0.029
cg13844341	7	2116368	1.82E-144	0.479	0.568	-0.089	*MAD1L1*	2.52E-12	-0.028
cg13618516	17	79129078	1.86E-144	0.411	0.528	-0.118	*AATK*	8.22E-18	-0.052
cg12926693	6	36665611	1.89E-144	0.321	0.401	-0.080	*RAB44*	1.20E-20	-0.033
cg17934470	5	49959703	2.07E-144	0.324	0.434	-0.110	NA	9.51E-18	-0.048
cg18446110	5	138714654	2.33E-144	0.340	0.446	-0.106	*SLC23A1*	1.34E-13	-0.040
cg16268734	12	56690194	3.19E-144	0.395	0.497	-0.102	*CS*	3.67E-12	-0.036
cg11377213	3	69370218	3.49E-144	0.264	0.350	-0.086	*FRMD4B*	2.13E-29	-0.048
cg15929395	11	117695828	4.89E-144	0.343	0.424	-0.082	*FXYD2*	1.93E-15	-0.030
cg24663338	1	8005108	5.22E-144	0.379	0.501	-0.122	NA	1.23E-13	-0.046
cg23056923	10	73497629	5.57E-144	0.373	0.442	-0.069	*C10orf105*	1.38E-20	-0.032
cg11061343	17	73993843	6.58E-144	0.234	0.303	-0.069	*TEN1-CDK3*	2.70E-15	-0.028
cg21685770	12	10324918	7.48E-144	0.403	0.517	-0.114	*OLR1*	9.81E-12	-0.036
cg23762465	22	50360628	8.23E-144	0.319	0.396	-0.077	NA	1.28E-14	-0.030
cg08173915	21	34774164	8.32E-144	0.377	0.490	-0.113	*IFNGR2*	1.39E-15	-0.043
cg06172871	16	72088244	8.53E-144	0.304	0.398	-0.094	*HP*	3.42E-22	-0.046
cg14523734	7	128020864	9.12E-144	0.779	0.715	0.064	NA	6.01E-21	0.031
cg19869035	7	2653955	1.18E-143	0.350	0.447	-0.096	*IQCE*	1.10E-12	-0.033
cg23893332	14	94576048	1.27E-143	0.373	0.451	-0.078	*IFI27*	1.13E-14	-0.030
cg09358725	11	33914088	1.67E-143	0.344	0.445	-0.101	*LMO2*	8.46E-35	-0.065
cg25605731	19	13054434	1.69E-143	0.314	0.432	-0.118	*CALR*	1.00E-09	-0.038
cg00030296	17	75318732	2.02E-143	0.335	0.416	-0.081	*SEPT9*	1.22E-18	-0.038
cg03574571	19	35820181	2.03E-143	0.365	0.461	-0.096	*CD22*	2.32E-25	-0.052
cg12110801	19	5992284	2.21E-143	0.391	0.497	-0.106	*LOC10012856*	9.99E-21	-0.051
cg05979400	2	27295591	2.33E-143	0.253	0.317	-0.064	*OST4*	7.29E-22	-0.029
cg17442155	7	99955675	3.50E-143	0.407	0.522	-0.115	*PILRB*	4.52E-14	-0.049
cg01823925	5	156967901	5.65E-143	0.753	0.669	0.084	*ADAM19*	0.004	0.011
cg05599723	1	12241073	5.76E-143	0.376	0.492	-0.116	*TNFRSF1B*	3.37E-12	-0.038
cg15075988	10	76974078	8.17E-143	0.340	0.453	-0.113	*VDAC2*	4.15E-13	-0.040
cg02309841	3	11758425	9.57E-143	0.375	0.473	-0.098	*VGLL4*	2.98E-22	-0.045
cg01048372	8	144655484	1.35E-142	0.360	0.438	-0.078	*C8orf73*	1.24E-13	-0.026
cg02147126	19	827715	1.39E-142	0.363	0.452	-0.089	*AZU1*	5.62E-15	-0.030
cg23526087	14	68973466	1.57E-142	0.695	0.622	0.073	*RAD51B*	8.83E-14	0.027
cg27196880	17	40570233	1.59E-142	0.305	0.401	-0.096	*PTRF*	7.16E-21	-0.047
cg04411201	3	99614305	1.74E-142	0.312	0.407	-0.094	*FILIP1L*	5.10E-20	-0.043
cg20312012	2	97331035	2.07E-142	0.366	0.481	-0.115	*FER1L5*	1.48E-14	-0.042
cg00168694	21	40193056	2.09E-142	0.338	0.452	-0.115	*ETS2*	8.47E-14	-0.042
cg05552543	16	75322715	2.09E-142	0.651	0.594	0.057	NA	2.15E-19	0.025
cg02796279	2	208101334	2.56E-142	0.351	0.466	-0.115	NA	1.58E-14	-0.043
cg21285555	8	52771466	3.10E-142	0.380	0.483	-0.102	*PCMTD1*	2.31E-14	-0.040
cg13549277	12	6659520	3.30E-142	0.325	0.397	-0.072	*IFFO1*	8.14E-14	-0.027
cg22032626	12	50203811	3.66E-142	0.311	0.381	-0.070	*NCKAP5L*	1.09E-30	-0.037
cg03116466	7	2116512	3.74E-142	0.363	0.432	-0.068	*MAD1L1*	2.42E-13	-0.023
cg14520947	1	225942842	4.72E-142	0.389	0.513	-0.123	NA	4.86E-10	-0.039
cg09835408	20	31352399	5.23E-142	0.304	0.386	-0.082	*DNMT3B*	1.91E-21	-0.039
cg26460483	7	97914016	7.14E-142	0.263	0.328	-0.065	*BRI3*	2.22E-17	-0.027
cg00291213	21	36398056	7.76E-142	0.278	0.363	-0.084	*RUNX1*	2.75E-25	-0.046
cg02891579	10	102823812	9.53E-142	0.255	0.316	-0.061	*KAZALD1*	1.46E-17	-0.025
cg26478599	7	41747322	1.19E-141	0.393	0.506	-0.113	*INHBA-AS1*	1.06E-19	-0.054
cg00071250	1	172628263	1.34E-141	0.770	0.693	0.078	*FASLG*	7.27E-11	0.026
cg07481273	14	93653027	1.59E-141	0.399	0.515	-0.116	*TMEM251*	7.60E-17	-0.050
cg06984156	1	20929640	1.67E-141	0.285	0.365	-0.080	*CDA*	1.01E-15	-0.031
cg18263572	1	6649337	1.67E-141	0.327	0.411	-0.083	*ZBTB48*	1.57E-31	-0.049
cg21328082	6	41254471	1.82E-141	0.324	0.435	-0.110	*TREM1*	7.11E-14	-0.042
cg27627524	8	131325625	2.13E-141	0.314	0.418	-0.104	*ASAP1*	1.86E-16	-0.046
cg20612299	8	131308633	2.56E-141	0.352	0.447	-0.095	*ASAP1*	6.16E-20	-0.045
cg12729838	8	27469186	2.91E-141	0.265	0.334	-0.068	*CLU*	1.02E-16	-0.027
cg03607951	1	79085586	2.96E-141	0.339	0.590	-0.251	*IFI44L*	9.92E-67	-0.175
cg05343404	19	3608327	4.15E-141	0.228	0.278	-0.050	*TBXA2R*	1.13E-15	-0.019
cg00593607	12	93530780	4.37E-141	0.354	0.464	-0.110	*LOC643339*	2.13E-15	-0.045
cg06001524	17	9264709	4.38E-141	0.383	0.492	-0.109	*STX8*	1.76E-16	-0.046
cg10287970	6	7115345	4.40E-141	0.329	0.428	-0.099	*RREB1*	2.43E-21	-0.048
cg17313269	6	25965330	7.88E-141	0.374	0.488	-0.114	*TRIM38*	4.21E-09	-0.033
cg02041484	7	129266958	1.03E-140	0.308	0.409	-0.101	*NRF1*	2.83E-10	-0.032
cg13536447	17	36188913	1.08E-140	0.322	0.442	-0.121	NA	1.21E-11	-0.042
cg04932082	6	91113506	1.13E-140	0.282	0.370	-0.088	NA	2.70E-16	-0.036
cg13229972	1	110335018	1.33E-140	0.303	0.373	-0.070	NA	2.31E-20	-0.034
cg07188863	11	110055425	1.51E-140	0.323	0.398	-0.075	*RDX*	4.10E-13	-0.026

cg13221924	17	6495080	1.51E-140	0.378	0.479	-0.101	*KIAA0753*	1.55E-18	-0.045
cg04858148	4	81117016	2.08E-140	0.243	0.333	-0.090	*PRDM8*	7.90E-30	-0.053
cg03389538	3	128779498	2.27E-140	0.306	0.377	-0.071	*GP9*	1.26E-16	-0.030
cg08044454	7	37024552	2.28E-140	0.312	0.414	-0.102	*ELMO1*	4.52E-16	-0.044
cg00974864	1	161601053	2.57E-140	0.309	0.390	-0.081	*FCGR3B*	4.52E-18	-0.034
cg09501509	6	89745356	3.98E-140	0.390	0.498	-0.108	NA	8.44E-15	-0.045
cg20977312	5	172748917	4.11E-140	0.459	0.569	-0.109	*STC2*	3.66E-08	-0.030
cg10826999	1	225862288	4.21E-140	0.349	0.458	-0.109	NA	2.08E-14	-0.042
cg11948905	5	53920730	4.44E-140	0.284	0.393	-0.109	NA	1.41E-17	-0.046
cg05581469	12	49413435	5.08E-140	0.321	0.422	-0.101	*PRKAG1*	1.42E-14	-0.040
cg09130674	6	39195019	5.57E-140	0.217	0.292	-0.075	*KCNK5*	5.15E-24	-0.039
cg03363565	16	474528	7.10E-140	0.330	0.436	-0.106	*RAB11FIP3*	5.23E-14	-0.039
cg02262553	4	87849250	7.19E-140	0.315	0.426	-0.111	*LOC10050674*	9.52E-16	-0.046
cg19244300	1	110113304	7.46E-140	0.326	0.420	-0.094	*GNAI3*	1.15E-12	-0.035
cg02905900	1	55013517	1.00E-139	0.266	0.328	-0.062	*ACOT11*	2.68E-19	-0.029
cg15530560	19	851290	1.01E-139	0.409	0.487	-0.078	*ELANE*	5.78E-09	-0.022
cg06958535	1	203734478	1.06E-139	0.733	0.638	0.095	*LAX1*	2.45E-14	0.039
cg26244575	12	76354015	1.20E-139	0.682	0.592	0.090	NA	1.09E-14	0.035
cg01313363	3	48471695	1.69E-139	0.277	0.342	-0.066	*PLXNB1*	7.60E-13	-0.021
cg08106973	1	40399833	1.94E-139	0.287	0.390	-0.103	NA	4.33E-24	-0.054
cg21946374	5	1108401	2.71E-139	0.342	0.416	-0.074	*SLC12A7*	3.38E-12	-0.025
cg12157761	15	71005778	2.91E-139	0.388	0.502	-0.115	*UACA*	2.47E-17	-0.044
cg17258387	2	174924430	3.18E-139	0.305	0.367	-0.062	NA	8.30E-18	-0.024
cg24548817	21	45774294	3.30E-139	0.368	0.475	-0.106	*TRPM2*	7.51E-10	-0.034
cg21549285	21	42799141	3.46E-139	0.405	0.827	-0.422	*MX1*	6.86E-59	-0.260
cg25491402	21	44101491	3.51E-139	0.734	0.665	0.069	*PDE9A*	5.60E-15	0.027
cg19696103	5	132354130	3.60E-139	0.404	0.502	-0.098	*ZCCHC10*	6.55E-06	-0.024
cg22318806	6	31540411	3.81E-139	0.632	0.549	0.083	*LTA*	1.58E-12	0.030
cg09376835	8	131347294	4.61E-139	0.297	0.393	-0.096	*ASAP1*	2.18E-24	-0.054
cg18542546	7	1982333	5.47E-139	0.420	0.539	-0.119	*MAD1L1*	6.31E-07	-0.030
cg04903759	3	167238645	5.60E-139	0.345	0.465	-0.121	*WDR49*	1.37E-16	-0.053
cg23254569	6	35451487	5.95E-139	0.360	0.465	-0.105	*TEAD3*	5.07E-12	-0.035
cg17184477	15	67360705	6.10E-139	0.739	0.668	0.070	*SMAD3*	8.42E-16	0.030
cg04519775	2	231849693	6.28E-139	0.762	0.688	0.074	*LOC348761*	3.50E-11	0.025
cg10471743	5	49739158	7.22E-139	0.322	0.435	-0.113	NA	3.20E-07	-0.030
cg13358186	17	7461775	7.46E-139	0.224	0.288	-0.065	*TNFSF13*	2.36E-16	-0.027
cg27644327	6	90845852	8.01E-139	0.243	0.329	-0.086	*BACH2*	2.97E-07	-0.022
cg15878616	20	52492209	8.92E-139	0.403	0.517	-0.114	*SUMO1P1*	2.75E-06	-0.028
cg03626208	12	2443169	9.85E-139	0.321	0.425	-0.104	*CACNA1C*	2.07E-21	-0.054
cg10687644	11	72527565	1.09E-138	0.314	0.394	-0.080	*ATG16L2*	7.71E-16	-0.030
cg19717347	12	129304956	1.11E-138	0.379	0.489	-0.110	*SLC15A4*	3.36E-13	-0.043
cg23671196	15	50405520	1.37E-138	0.259	0.339	-0.079	*ATP8B4*	2.88E-21	-0.038
cg12044599	11	67206308	1.54E-138	0.746	0.659	0.087	*PTPRCAP*	3.44E-14	0.032
cg22840076	19	41782156	1.65E-138	0.435	0.523	-0.089	*HNRNPUL1*	3.86E-16	-0.037
cg06373940	2	128052778	1.85E-138	0.438	0.566	-0.128	*ERCC3*	4.90E-18	-0.050
cg08078028	10	120802096	1.98E-138	0.576	0.677	-0.101	*EIF3A*	5.16E-19	-0.042
cg15580458	10	121155124	2.03E-138	0.324	0.416	-0.092	*GRK5*	1.52E-14	-0.037
cg24777399	2	109855574	2.47E-138	0.368	0.456	-0.088	*SH3RF3*	1.99E-30	-0.053
cg17846016	17	80181015	2.89E-138	0.375	0.495	-0.120	NA	5.24E-12	-0.042
cg13534791	9	35042344	3.05E-138	0.341	0.409	-0.068	*C9orf131*	1.29E-15	-0.026
cg11389756	6	28875354	3.15E-138	0.317	0.423	-0.107	*TRIM27*	5.14E-13	-0.037
cg20847110	4	39482781	4.58E-138	0.322	0.423	-0.101	*LOC401127*	2.33E-16	-0.044
cg01402255	1	153800699	4.63E-138	0.332	0.431	-0.099	*GATAD2B*	4.04E-12	-0.037
cg16405432	14	95973710	4.78E-138	0.327	0.405	-0.077	NA	4.78E-18	-0.034
cg03308839	16	15797297	4.97E-138	0.384	0.503	-0.119	*NDE1*	2.09E-12	-0.045
cg01479187	2	43158610	5.33E-138	0.271	0.367	-0.095	NA	9.72E-15	-0.044
cg13277939	14	39735211	5.82E-138	0.334	0.441	-0.107	*CTAGE5*	7.83E-17	-0.044
cg07730301	11	67777952	6.14E-138	0.345	0.415	-0.070	*ALDH3B1*	3.92E-21	-0.030
cg12711832	8	68258899	6.40E-138	0.323	0.424	-0.101	NA	7.39E-04	-0.018
cg04425551	6	30297338	6.95E-138	0.435	0.532	-0.097	*TRIM39*	3.44E-09	-0.028
cg22396850	17	41121153	7.17E-138	0.270	0.349	-0.079	*PTGES3L-AARS*	1.03E-14	-0.032
cg16481332	7	2654053	7.33E-138	0.346	0.451	-0.104	*IQCE*	8.83E-15	-0.043
cg03837680	17	77967529	7.82E-138	0.251	0.335	-0.084	*TBC1D16*	3.93E-12	-0.029
cg10725937	6	41130978	9.19E-138	0.365	0.468	-0.102	*TREM2*	3.62E-19	-0.046
cg25684151	8	126588554	9.39E-138	0.346	0.441	-0.095	NA	2.33E-13	-0.037
cg18463686	7	141646690	1.19E-137	0.343	0.454	-0.111	*CLEC5A*	1.85E-10	-0.036
cg03280622	8	145023013	1.33E-137	0.271	0.346	-0.075	*PLEC*	6.81E-25	-0.042
cg12323063	17	67497879	1.53E-137	0.442	0.548	-0.106	*MAP2K6*	1.75E-10	-0.035
cg16844053	7	148404978	1.78E-137	0.387	0.479	-0.093	*CUL1*	4.01E-09	-0.028
cg21932814	3	122044172	1.89E-137	0.334	0.432	-0.098	*CSTA*	9.82E-19	-0.046
cg06422467	6	30720484	2.19E-137	0.321	0.412	-0.091	NA	1.50E-25	-0.052
cg04213390	2	85743934	2.64E-137	0.388	0.499	-0.112	NA	1.86E-11	-0.037
cg23807570	12	110787107	2.76E-137	0.371	0.476	-0.105	*ATP2A2*	1.50E-12	-0.039

cg17517296	2	43107458	2.77E-137	0.350	0.434	-0.085	NA	1.70E-13	-0.032
cg08169020	14	69256888	3.00E-137	0.681	0.572	0.109	*ZFP36L1*	5.39E-18	0.049
cg24401262	1	31956405	3.10E-137	0.276	0.378	-0.102	NA	3.21E-17	-0.049
cg23312431	21	45773997	3.60E-137	0.342	0.435	-0.093	*TRPM2*	7.14E-13	-0.031
cg20489909	2	242711046	3.76E-137	0.310	0.377	-0.067	NA	7.83E-10	-0.020
cg04384031	17	19631485	5.21E-137	0.225	0.296	-0.071	NA	2.75E-14	-0.029
cg07283015	18	22039857	7.08E-137	0.414	0.534	-0.120	*HRH4*	3.98E-15	-0.053
cg23648239	14	52701606	7.78E-137	0.361	0.467	-0.107	NA	1.32E-10	-0.037
cg11358199	2	8453529	8.54E-137	0.740	0.667	0.073	*LINC00299*	7.94E-06	0.016
cg01119452	7	37287850	8.64E-137	0.307	0.418	-0.110	*ELMO1*	2.95E-23	-0.059
cg23261343	6	45413792	9.02E-137	0.279	0.373	-0.094	*RUNX2*	1.47E-19	-0.046
cg10718809	11	64087106	9.31E-137	0.319	0.401	-0.081	*PRDX5*	5.83E-09	-0.023
cg17959183	2	26297671	9.39E-137	0.409	0.527	-0.118	*RAB10*	8.49E-13	-0.041
cg14522803	11	818834	9.45E-137	0.355	0.428	-0.073	*PNPLA2*	1.69E-08	-0.020
cg01580228	11	93467095	9.82E-137	0.316	0.420	-0.104	NA	5.33E-13	-0.040
cg18503912	19	35630279	1.26E-136	0.361	0.477	-0.116	*FXYD1*	1.89E-15	-0.048
cg07496207	5	172280684	1.57E-136	0.381	0.484	-0.102	*ERGIC1*	1.67E-11	-0.038
cg18571045	13	113238006	1.61E-136	0.282	0.383	-0.100	*TUBGCP3*	3.47E-15	-0.041
cg12774429	14	21853470	1.67E-136	0.298	0.387	-0.088	*SUPT16H*	9.82E-22	-0.045
cg19499884	10	102760724	1.91E-136	0.335	0.431	-0.096	*LZTS2*	3.88E-17	-0.042
cg05314350	6	28874702	2.15E-136	0.381	0.485	-0.104	*TRIM27*	1.37E-14	-0.041
cg13413384	9	137302231	2.16E-136	0.374	0.445	-0.071	*RXRA*	1.01E-10	-0.022
cg02308232	17	7742762	2.46E-136	0.361	0.466	-0.105	*KDM6B*	2.66E-13	-0.038
cg22957691	2	36790185	2.58E-136	0.376	0.495	-0.119	*FEZ2*	1.63E-11	-0.041
cg12141659	11	2847462	2.59E-136	0.199	0.264	-0.065	*KCNQ1*	9.65E-18	-0.029
cg26336059	1	153958977	2.65E-136	0.232	0.318	-0.086	*RAB13*	2.78E-11	-0.029
cg09255910	1	221055790	2.72E-136	0.394	0.477	-0.082	*HLX*	5.97E-10	-0.025
cg13999433	9	117156883	2.80E-136	0.380	0.488	-0.107	*AKNA*	2.04E-10	-0.036
cg01357222	6	137619143	3.36E-136	0.369	0.467	-0.098	NA	9.51E-19	-0.045
cg11263420	19	50019310	3.71E-136	0.213	0.284	-0.070	*FCGRT*	1.95E-24	-0.039
cg07488141	7	47560215	3.91E-136	0.338	0.442	-0.104	*TNS3*	4.76E-22	-0.055
cg02175033	14	70098523	4.49E-136	0.317	0.418	-0.101	*KIAA0247*	3.72E-07	-0.027
cg26930596	1	2082315	4.66E-136	0.424	0.550	-0.126	*PRKCZ*	1.42E-09	-0.038
cg24501381	1	43001072	5.03E-136	0.421	0.537	-0.116	*CCDC30*	9.47E-10	-0.035
cg24211388	6	31582837	5.34E-136	0.331	0.435	-0.104	*AIF1*	6.55E-16	-0.042
cg09553839	10	74358778	5.52E-136	0.415	0.517	-0.103	*MICU1*	3.95E-21	-0.048
cg05299836	16	31119067	5.83E-136	0.753	0.681	0.071	*BCKDK*	3.49E-07	0.018
cg26217402	14	74238381	5.92E-136	0.639	0.550	0.088	*C14orf43*	1.01E-13	0.033
cg24608504	2	54760330	6.09E-136	0.395	0.503	-0.108	*SPTBN1*	8.26E-12	-0.037
cg01620379	14	21052361	7.03E-136	0.364	0.467	-0.104	*RNASE11*	1.55E-14	-0.044
cg08796342	14	92334029	7.47E-136	0.708	0.628	0.080	*TC2N*	4.11E-09	0.026
cg03330678	17	75316233	8.26E-136	0.322	0.407	-0.086	*SEPT9*	6.03E-11	-0.027
cg11151395	17	56355299	8.78E-136	0.442	0.552	-0.110	*MPO*	5.01E-10	-0.034
cg12792363	11	63274030	8.78E-136	0.245	0.320	-0.075	*LGALS12*	4.08E-11	-0.027
cg02019444	21	35016787	9.84E-136	0.240	0.315	-0.075	*ITSN1*	1.71E-14	-0.031
cg00855901	1	79085765	1.00E-135	0.167	0.275	-0.108	*IFI44L*	7.45E-24	-0.057
cg00892324	7	2565575	1.03E-135	0.299	0.376	-0.076	*LFNG*	4.43E-13	-0.029
cg17355865	11	76887139	1.06E-135	0.308	0.390	-0.082	*MYO7A*	7.65E-20	-0.038
cg07165260	16	85062881	1.09E-135	0.734	0.649	0.085	*KIAA0513*	1.67E-11	0.029
cg20806175	1	161186839	1.13E-135	0.274	0.348	-0.074	*FCER1G*	3.87E-15	-0.029
cg00534022	2	8686087	1.14E-135	0.376	0.474	-0.098	NA	4.05E-17	-0.046
cg24405567	15	70787565	1.17E-135	0.373	0.487	-0.114	NA	1.87E-27	-0.066
cg17935536	2	60755743	1.20E-135	0.298	0.371	-0.073	*BCL11A*	1.06E-11	-0.025
cg08772789	17	75318655	1.37E-135	0.350	0.448	-0.098	*SEPT9*	3.28E-17	-0.045
cg03634729	18	21452829	1.48E-135	0.752	0.676	0.075	*LAMA3*	2.77E-22	0.040
cg23815853	12	48147034	1.53E-135	0.424	0.540	-0.117	*RAPGEF3*	4.28E-19	-0.052
cg26027052	6	36821364	1.78E-135	0.271	0.364	-0.093	NA	5.89E-20	-0.045
cg01591037	12	15134481	1.89E-135	0.371	0.485	-0.114	*PDE6H*	2.42E-09	-0.034
cg18447740	19	54604187	1.97E-135	0.262	0.342	-0.079	*OSCAR*	9.05E-17	-0.033
cg04252203	3	194696866	2.06E-135	0.337	0.425	-0.088	NA	5.12E-13	-0.032
cg16762030	15	50148234	2.24E-135	0.330	0.438	-0.108	NA	9.99E-16	-0.045
cg09799980	12	4398618	2.30E-135	0.251	0.334	-0.083	*CCND2*	2.60E-16	-0.036
cg17013990	1	161091682	2.40E-135	0.306	0.404	-0.099	*DEDD*	2.41E-15	-0.041
cg08472008	4	3531603	2.92E-135	0.287	0.384	-0.097	*LRPAP1*	3.20E-11	-0.033
cg13255542	11	134120785	3.26E-135	0.359	0.458	-0.099	*THYN1*	6.48E-11	-0.034
cg06465076	5	96027378	3.51E-135	0.466	0.583	-0.117	*CAST*	8.78E-07	-0.029
cg10061770	16	68366844	3.51E-135	0.280	0.377	-0.098	*PRMT7*	5.62E-13	-0.039
cg15511120	11	6598119	3.60E-135	0.293	0.388	-0.095	NA	1.93E-17	-0.043
cg00965578	22	36560801	3.77E-135	0.248	0.324	-0.077	*APOL3*	6.54E-23	-0.038
cg11327408	6	30297329	5.00E-135	0.433	0.535	-0.102	*TRIM39*	2.47E-08	-0.029
cg11703212	13	114271629	5.34E-135	0.430	0.553	-0.123	*TFDP1*	1.28E-12	-0.043
cg14183540	11	3175007	5.87E-135	0.333	0.416	-0.083	*OSBPL5*	1.78E-23	-0.042
cg01561719	10	90611855	5.97E-135	0.353	0.454	-0.101	*ANKRD22*	1.62E-19	-0.049

cg24236839	19	5038822	7.04E-135	0.337	0.410	-0.073	*KDM4B*	8.90E-20	-0.032
cg10981439	6	41254433	8.07E-135	0.363	0.474	-0.111	*TREM1*	2.42E-12	-0.039
cg17159187	8	144420118	8.56E-135	0.371	0.466	-0.095	*TOP1MT*	1.98E-13	-0.035
cg08229360	3	37904475	8.95E-135	0.210	0.262	-0.052	*CTDSPL*	7.09E-24	-0.027
cg07573020	5	14707001	9.64E-135	0.795	0.728	0.067	*ANKH*	1.49E-15	0.028
cg04118119	13	47371987	9.95E-135	0.388	0.498	-0.110	*ESD*	2.13E-12	-0.042
cg04226002	11	113953462	1.02E-134	0.331	0.433	-0.102	*ZBTB16*	1.52E-19	-0.045
cg23595413	11	76574308	1.13E-134	0.279	0.376	-0.097	*ACER3*	2.40E-12	-0.038
cg20700740	1	9339683	1.28E-134	0.359	0.471	-0.112	NA	6.96E-04	-0.019
cg11606261	12	53775336	1.33E-134	0.237	0.323	-0.086	*SP1*	7.75E-08	-0.025
cg05171937	12	27396765	1.56E-134	0.418	0.538	-0.120	*STK38L*	3.55E-15	-0.051
cg01925950	13	115022228	1.62E-134	0.300	0.390	-0.090	*CDC16*	5.66E-17	-0.041
cg14290576	9	94181612	1.70E-134	0.305	0.395	-0.090	*NFIL3*	2.19E-21	-0.047
cg15572396	3	58321349	1.92E-134	0.315	0.399	-0.084	*PXK*	3.11E-16	-0.036
cg03821121	11	12148288	2.10E-134	0.285	0.354	-0.069	*MICAL2*	4.22E-17	-0.029
cg01565774	12	56414533	2.20E-134	0.216	0.275	-0.059	*IKZF4*	2.41E-14	-0.025
cg01431057	1	153362927	2.24E-134	0.373	0.461	-0.088	*S100A8*	1.15E-16	-0.035
cg13733708	20	44597685	2.27E-134	0.402	0.485	-0.082	*ZNF335*	6.49E-18	-0.038
cg13171679	8	41258634	2.28E-134	0.297	0.390	-0.093	NA	2.41E-15	-0.041
cg10679182	7	133225096	2.53E-134	0.376	0.482	-0.106	*EXOC4*	2.10E-07	-0.027
cg08059719	12	122444450	3.45E-134	0.775	0.701	0.074	NA	3.13E-16	0.033
cg07136111	7	30829005	3.49E-134	0.325	0.423	-0.098	*FAM188B*	4.69E-16	-0.044
cg21821982	3	31547255	3.99E-134	0.340	0.457	-0.116	NA	2.02E-16	-0.050
cg12763828	1	9129646	4.52E-134	0.307	0.397	-0.090	*SLC2A5*	4.28E-17	-0.042
cg01627252	2	31397283	5.26E-134	0.373	0.474	-0.101	*CAPN14*	3.12E-16	-0.043
cg22074114	3	134326341	5.26E-134	0.358	0.457	-0.099	*KY*	2.18E-18	-0.044
cg17344091	6	28885444	5.31E-134	0.261	0.366	-0.105	*TRIM27*	7.73E-14	-0.042
cg05008854	10	7825899	5.49E-134	0.380	0.468	-0.089	*KIN*	6.97E-13	-0.035
cg08539991	19	36203832	6.77E-134	0.788	0.715	0.072	*ZBTB32*	9.50E-10	0.023
cg07564563	19	3548977	8.57E-134	0.403	0.497	-0.094	*MFSD12*	1.40E-10	-0.030
cg06294475	11	2698623	1.05E-133	0.280	0.370	-0.090	*KCNQ1OT1*	3.13E-13	-0.035
cg04903089	6	32905190	1.10E-133	0.268	0.351	-0.083	*HLA-DMB*	1.11E-17	-0.038
cg00112238	1	54719938	1.15E-133	0.261	0.331	-0.070	*SSBP3*	3.59E-17	-0.030
cg04089901	7	7557590	1.16E-133	0.323	0.424	-0.101	*COL28A1*	3.72E-18	-0.048
cg24403649	4	39172243	1.24E-133	0.391	0.454	-0.063	NA	1.01E-14	-0.026
cg11557901	9	128022358	1.27E-133	0.377	0.501	-0.123	NA	5.29E-10	-0.039
cg10738119	7	75957040	1.35E-133	0.342	0.434	-0.093	*YWHAG*	1.50E-12	-0.036
cg26942829	6	13408158	1.62E-133	0.387	0.503	-0.116	*GFOD1*	9.15E-12	-0.039
cg07213830	1	1093940	1.74E-133	0.328	0.403	-0.075	NA	8.24E-17	-0.025
cg04046364	12	58210661	1.85E-133	0.296	0.392	-0.095	*AVIL*	6.71E-12	-0.033
cg04266202	17	56352895	2.35E-133	0.418	0.524	-0.106	*MPO*	2.20E-12	-0.039
cg07241090	12	124864594	2.66E-133	0.298	0.380	-0.082	*NCOR2*	2.51E-14	-0.032
cg08944026	5	10626811	2.75E-133	0.295	0.389	-0.094	*ANKRD33B*	5.55E-15	-0.037
cg05830425	13	21654356	3.20E-133	0.411	0.515	-0.104	NA	3.01E-07	-0.028
cg18229071	19	2695245	4.01E-133	0.254	0.314	-0.060	*GNG7*	8.83E-20	-0.029
cg13304638	17	80834089	4.76E-133	0.432	0.565	-0.133	*TBCD*	5.37E-16	-0.054
cg18242288	4	54793620	5.40E-133	0.503	0.615	-0.111	NA	5.38E-14	-0.043
cg01574390	16	21623651	6.18E-133	0.300	0.382	-0.082	*METTL9*	2.03E-15	-0.033
cg06749872	16	30198509	6.39E-133	0.649	0.570	0.079	*CORO1A*	4.17E-09	0.023
cg08368934	16	57701455	6.41E-133	0.359	0.436	-0.077	*GPR97*	3.64E-11	-0.026
cg13410614	9	136341915	6.80E-133	0.308	0.411	-0.102	*SLC2A6*	9.08E-18	-0.048
cg24504014	11	110297121	8.67E-133	0.461	0.555	-0.093	NA	5.19E-09	-0.028
cg26783127	17	79128918	1.01E-132	0.353	0.480	-0.128	*AATK*	3.41E-12	-0.043
cg00030432	7	100028974	1.11E-132	0.382	0.464	-0.082	*MEPCE*	9.58E-07	-0.020
cg05580141	12	49071788	1.19E-132	0.394	0.506	-0.112	*KANSL2*	4.35E-19	-0.052
cg26508200	12	109235071	1.28E-132	0.458	0.572	-0.115	*SSH1*	3.77E-13	-0.041
cg04099543	11	72983405	1.57E-132	0.299	0.368	-0.069	*P2RY6*	1.56E-15	-0.027
cg14480046	12	122444580	1.58E-132	0.781	0.707	0.074	NA	7.30E-12	0.027
cg23468927	11	67206263	1.66E-132	0.713	0.641	0.072	*PTPRCAP*	4.53E-15	0.029
cg07375836	17	35717813	1.92E-132	0.254	0.358	-0.104	*ACACA*	2.53E-19	-0.052
cg16945415	3	125980095	2.02E-132	0.258	0.328	-0.070	NA	1.81E-16	-0.027
cg10011091	8	104424477	2.04E-132	0.301	0.406	-0.105	*SLC25A32*	3.26E-20	-0.050
cg13468144	17	4081428	2.12E-132	0.490	0.609	-0.119	*ANKFY1*	1.38E-12	-0.043
cg00211174	3	127332098	2.61E-132	0.387	0.513	-0.126	*MCM2*	1.36E-12	-0.051
cg19975346	10	126366131	2.96E-132	0.426	0.508	-0.082	*FAM53B*	2.88E-10	-0.028
cg00382999	3	136649333	3.70E-132	0.343	0.446	-0.102	*NCK1*	2.12E-13	-0.039
cg22145559	20	57583009	3.89E-132	0.270	0.348	-0.078	*CTSZ*	1.04E-22	-0.041
cg06470558	5	176816828	4.53E-132	0.646	0.586	0.061	*SLC34A1*	0.023	0.007
cg06706156	1	230183251	4.83E-132	0.367	0.474	-0.107	NA	2.53E-15	-0.047
cg18554789	7	139432178	5.14E-132	0.264	0.361	-0.097	*HIPK2*	9.15E-14	-0.039
cg17326313	2	37383568	5.37E-132	0.087	0.156	-0.068	*EIF2AK2*	2.51E-28	-0.044
cg24414325	12	56414442	5.42E-132	0.365	0.465	-0.100	*IKZF4*	1.04E-10	-0.032
cg24361586	17	62744534	5.46E-132	0.465	0.556	-0.091	NA	9.56E-13	-0.033

cg07305933	8	129079496	5.55E-132	0.328	0.423	-0.095	*PVT1*	1.20E-12	-0.037
cg04552418	1	31958245	6.08E-132	0.353	0.474	-0.121	NA	2.03E-12	-0.043
cg19913563	6	30720261	6.36E-132	0.252	0.347	-0.094	NA	9.18E-18	-0.045
cg19011001	14	93539613	6.89E-132	0.305	0.381	-0.075	*ITPK1*	3.03E-11	-0.024
cg18888137	6	32945759	7.95E-132	0.329	0.434	-0.105	*BRD2*	6.64E-15	-0.043
cg16850690	3	129318459	8.21E-132	0.275	0.338	-0.063	*PLXND1*	6.43E-17	-0.025
cg04451770	10	97515372	8.66E-132	0.311	0.404	-0.093	*ENTPD1*	1.85E-14	-0.041
cg15157945	3	47023603	8.76E-132	0.251	0.317	-0.066	*NBEAL2*	1.27E-23	-0.037
cg07000116	16	30616649	9.62E-132	0.434	0.552	-0.118	*ZNF689*	1.10E-15	-0.050
cg02331198	6	106988121	9.88E-132	0.300	0.380	-0.080	*AIM1*	2.35E-16	-0.035
cg18036081	22	29605147	1.06E-131	0.330	0.399	-0.069	*EMID1*	3.67E-20	-0.033
cg13599613	9	130524902	1.29E-131	0.439	0.557	-0.118	*SH2D3C*	1.38E-07	-0.030
cg00407944	9	139424152	1.34E-131	0.256	0.322	-0.066	*NOTCH1*	6.72E-07	-0.017
cg11119767	2	174024669	1.34E-131	0.334	0.441	-0.106	*ZAK*	2.14E-13	-0.043
cg01565508	17	8869961	1.57E-131	0.367	0.472	-0.105	*PIK3R5*	3.34E-11	-0.037
cg00261690	1	28856281	1.83E-131	0.394	0.499	-0.105	*RCC1*	6.53E-14	-0.039
cg04304036	16	68334619	1.88E-131	0.332	0.430	-0.097	*SLC7A6OS*	7.47E-11	-0.032
cg07377519	19	50269083	2.00E-131	0.269	0.366	-0.097	*AP2A1*	1.44E-16	-0.043
cg02679745	9	139927646	2.12E-131	0.245	0.309	-0.064	*FUT7*	3.64E-22	-0.033
cg12045443	12	89777963	2.69E-131	0.273	0.357	-0.084	NA	1.80E-12	-0.031
cg15527515	14	23630709	2.98E-131	0.303	0.406	-0.103	*SLC7A8*	9.42E-12	-0.039
cg24406240	11	88153520	3.37E-131	0.373	0.476	-0.103	NA	1.27E-09	-0.031
cg27583010	16	30198505	3.65E-131	0.622	0.508	0.114	*CORO1A*	3.30E-08	0.030
cg11283860	1	8273352	3.80E-131	0.326	0.409	-0.083	NA	2.66E-06	-0.020
cg24786875	5	94027853	3.82E-131	0.412	0.504	-0.093	*ANKRD32*	3.92E-11	-0.031
cg09727050	2	152214177	4.82E-131	0.349	0.450	-0.101	*TNFAIP6*	8.79E-11	-0.033
cg08961793	16	28628118	5.92E-131	0.328	0.423	-0.095	*SULT1A1*	2.06E-20	-0.048
cg13618969	9	129184186	6.21E-131	0.494	0.611	-0.117	*FAM125B*	1.58E-06	-0.027
cg22991506	14	50468241	7.01E-131	0.665	0.589	0.075	*C14orf182*	3.04E-14	0.028
cg13916080	9	129624994	8.17E-131	0.247	0.336	-0.089	*ZBTB34*	1.08E-11	-0.032
cg00905101	16	1988798	8.63E-131	0.274	0.350	-0.076	*MSRB1*	5.12E-19	-0.036
cg21127597	11	118109650	9.58E-131	0.306	0.398	-0.092	*MPZL3*	1.50E-09	-0.029
cg05949397	11	44976423	1.29E-130	0.426	0.540	-0.115	NA	1.46E-17	-0.050
cg10531637	22	51000048	1.35E-130	0.670	0.611	0.060	*SYCE3*	8.51E-08	0.016
cg08450017	3	45984838	1.44E-130	0.602	0.473	0.130	*CXCR6*	1.30E-18	0.062
cg16586406	1	154164994	1.52E-130	0.259	0.341	-0.082	*TPM3*	9.77E-25	-0.045
cg19502359	11	72912441	1.62E-130	0.306	0.398	-0.092	NA	5.13E-07	-0.026
cg14186336	9	124045139	1.70E-130	0.351	0.438	-0.087	*GSN*	3.97E-19	-0.045
cg14685356	6	152639548	1.85E-130	0.345	0.438	-0.092	*SYNE1*	2.67E-15	-0.038
cg14333394	9	139812357	1.88E-130	0.381	0.450	-0.068	*TRAF2*	1.49E-09	-0.019
cg14827807	1	150943828	2.11E-130	0.319	0.381	-0.062	*CERS2*	8.67E-15	-0.023
cg14057303	9	109683834	2.37E-130	0.717	0.642	0.075	*ZNF462*	2.58E-09	0.025
cg10611016	11	6225759	4.61E-130	0.410	0.522	-0.112	*C11orf42*	2.64E-09	-0.035
cg04112866	2	105925965	4.64E-130	0.324	0.429	-0.104	*TGFBRAP1*	2.15E-23	-0.058
cg16954385	7	23246896	4.70E-130	0.397	0.507	-0.110	NA	4.74E-11	-0.036
cg01366670	16	89004378	4.74E-130	0.319	0.379	-0.060	*CBFA2T3*	2.27E-14	-0.024
cg04902811	10	99478719	4.77E-130	0.242	0.301	-0.059	NA	1.33E-24	-0.033
cg07053114	10	129794994	4.82E-130	0.272	0.360	-0.088	*PTPRE*	6.83E-11	-0.029
cg04225088	11	33278186	4.91E-130	0.410	0.526	-0.115	*HIPK3*	2.76E-11	-0.039
cg21326139	4	1294783	6.05E-130	0.297	0.390	-0.092	*MAEA*	8.18E-14	-0.036
cg22489510	2	8675599	6.22E-130	0.361	0.447	-0.086	NA	7.31E-17	-0.039
cg27064845	19	48252623	6.64E-130	0.708	0.643	0.065	*GLTSCR2*	7.03E-08	0.019
cg21126943	19	42259395	6.70E-130	0.264	0.342	-0.078	*CEACAM6*	1.86E-22	-0.041
cg04919592	7	2607232	7.19E-130	0.382	0.467	-0.084	*IQCE*	1.65E-07	-0.023
cg03514239	1	153329781	7.27E-130	0.219	0.277	-0.059	*S100A9*	5.77E-20	-0.030
cg06806891	3	128323079	7.57E-130	0.411	0.473	-0.063	NA	7.13E-08	-0.016
cg26488183	15	75195105	9.18E-130	0.269	0.366	-0.097	*FAM219B*	1.13E-19	-0.050
cg09571972	6	2104322	1.00E-129	0.280	0.372	-0.092	*GMDS*	2.73E-14	-0.039
cg27172287	22	46974976	1.04E-129	0.416	0.483	-0.067	NA	5.83E-12	-0.025
cg12929983	15	40399004	1.07E-129	0.294	0.391	-0.096	*BMF*	7.73E-11	-0.034
cg13471188	2	20638399	1.12E-129	0.323	0.423	-0.100	NA	4.84E-14	-0.042
cg02192746	7	99971016	1.12E-129	0.283	0.356	-0.073	*PILRA*	3.94E-15	-0.029
cg13683534	9	139221076	1.58E-129	0.268	0.339	-0.071	*DKFZP434A06.*	4.79E-21	-0.037
cg13071069	3	13005914	1.62E-129	0.317	0.393	-0.076	*IQSEC1*	1.64E-14	-0.028
cg23202722	1	33793808	1.73E-129	0.312	0.416	-0.104	*PHC2*	1.42E-14	-0.044
cg02073304	1	33457500	1.74E-129	0.235	0.305	-0.071	NA	2.01E-21	-0.037
cg10317164	3	10274142	1.93E-129	0.287	0.362	-0.076	*IRAK2*	1.43E-10	-0.026
cg14138235	14	56281968	2.05E-129	0.356	0.449	-0.093	NA	3.55E-19	-0.049
cg06287548	21	34773372	2.11E-129	0.351	0.441	-0.090	NA	1.92E-16	-0.039
cg10972897	1	165859714	2.12E-129	0.322	0.404	-0.082	*UCK2*	2.96E-12	-0.033
cg07302959	7	92198639	2.19E-129	0.286	0.387	-0.101	*FAM133DP*	6.23E-12	-0.038
cg02847588	1	8271997	2.53E-129	0.331	0.410	-0.079	NA	6.16E-11	-0.025
cg02600394	4	48136234	2.61E-129	0.718	0.647	0.071	*TXK*	4.07E-21	0.036

cg05744184	17	40463806	2.86E-129	0.274	0.345	-0.071	*STAT5A*	5.89E-11	-0.022
cg00259404	6	28885568	3.16E-129	0.274	0.374	-0.100	*TRIM27*	5.31E-09	-0.033
cg13321166	11	59824038	3.48E-129	0.296	0.387	-0.091	*MS4A3*	2.00E-15	-0.035
cg12196294	3	129575053	3.66E-129	0.363	0.469	-0.105	*TMCC1*	2.29E-06	-0.026
cg27200630	3	138494226	4.60E-129	0.245	0.333	-0.088	NA	1.02E-17	-0.041
cg23581252	6	32897912	4.68E-129	0.384	0.475	-0.091	NA	8.92E-12	-0.032
cg04460364	17	8370017	4.89E-129	0.417	0.514	-0.097	*NDEL1*	1.07E-08	-0.029
cg15382933	11	33745399	5.17E-129	0.353	0.464	-0.110	*CD59*	8.95E-18	-0.054
cg14315912	14	102677294	5.66E-129	0.484	0.557	-0.073	*WDR20*	1.18E-07	-0.019
cg00221718	3	128914668	5.67E-129	0.250	0.334	-0.084	NA	1.30E-13	-0.035
cg23547429	11	57195025	7.02E-129	0.272	0.330	-0.058	*SLC43A3*	1.17E-26	-0.033
cg16563370	17	33775952	8.45E-129	0.327	0.408	-0.080	*SLFN13*	9.51E-18	-0.036
cg21263566	1	95561502	1.08E-128	0.355	0.455	-0.099	*TMEM56*	2.29E-13	-0.038
cg17540545	19	35630355	1.28E-128	0.241	0.315	-0.074	*FXYD1*	6.23E-14	-0.032
cg21426759	6	30303126	1.32E-128	0.469	0.581	-0.112	*TRIM39*	4.71E-13	-0.043
cg06739107	3	188399479	1.42E-128	0.382	0.495	-0.114	*LPP*	3.22E-08	-0.032
cg02999224	14	23284559	1.43E-128	0.344	0.439	-0.096	*SLC7A7*	5.52E-11	-0.034
cg15134583	1	224015075	1.72E-128	0.292	0.382	-0.089	*TP53BP2*	2.54E-15	-0.040
cg05886087	2	102758186	1.92E-128	0.327	0.433	-0.106	NA	3.46E-12	-0.040
cg24737761	6	106245659	2.03E-128	0.469	0.577	-0.108	NA	1.43E-05	-0.025
cg00828556	3	196351986	2.16E-128	0.295	0.400	-0.105	NA	1.06E-07	-0.031
cg04886849	10	118997146	2.36E-128	0.308	0.385	-0.077	NA	1.00E-09	-0.026
cg20147326	11	33913538	2.51E-128	0.204	0.275	-0.071	*LMO2*	1.79E-11	-0.028
cg05552874	10	91153143	2.54E-128	0.461	0.710	-0.249	*IFIT1*	2.71E-56	-0.142
cg25853622	3	188425256	2.99E-128	0.312	0.416	-0.103	*LPP*	8.90E-11	-0.039
cg09874482	15	86098694	3.21E-128	0.280	0.365	-0.085	*AKAP13*	7.88E-16	-0.038
cg07324245	17	75445905	3.24E-128	0.788	0.726	0.062	*SEPT9*	3.73E-23	0.036
cg24898863	1	153363580	3.44E-128	0.247	0.322	-0.075	*S100A8*	7.52E-21	-0.037
cg17023856	1	2036508	3.93E-128	0.388	0.480	-0.092	*PRKCZ*	7.76E-20	-0.045
cg05162166	22	41684460	4.06E-128	0.387	0.485	-0.098	NA	2.66E-17	-0.048
cg11814087	5	32391135	4.71E-128	0.420	0.534	-0.115	*ZFR*	4.03E-12	-0.044
cg20794855	8	67365340	4.86E-128	0.391	0.482	-0.092	*ADHFE1*	2.47E-08	-0.029
cg16374343	17	1014352	5.15E-128	0.329	0.391	-0.062	*ABR*	1.46E-16	-0.027
cg19081101	1	203156625	5.67E-128	0.354	0.466	-0.112	*CHI3L1*	6.22E-07	-0.030
cg10426076	11	57158282	7.05E-128	0.370	0.474	-0.103	*PRG2*	1.62E-12	-0.039
cg06738887	15	60688010	7.27E-128	0.273	0.362	-0.090	*ANXA2*	1.91E-15	-0.036
cg05533539	17	44104521	7.41E-128	0.317	0.406	-0.089	*MAPT*	9.97E-08	-0.027
cg02855045	3	72777245	8.53E-128	0.325	0.410	-0.085	NA	1.93E-17	-0.038
cg00799984	8	134307105	9.52E-128	0.409	0.479	-0.070	*NDRG1*	1.42E-08	-0.020
cg26391219	11	3189207	9.56E-128	0.337	0.419	-0.082	NA	1.91E-14	-0.032
cg14614581	10	73844130	1.25E-127	0.804	0.733	0.071	*SPOCK2*	4.20E-16	0.032
cg12614630	12	57387318	1.56E-127	0.392	0.497	-0.105	*GPR182*	5.48E-12	-0.042
cg08428292	16	85981373	1.62E-127	0.597	0.502	0.095	NA	6.98E-07	0.023
cg07970040	1	9789165	1.64E-127	0.797	0.733	0.064	*CLSTN1*	2.00E-16	0.030
cg14600987	11	1952678	1.80E-127	0.299	0.384	-0.085	*TNNT3*	7.31E-29	-0.051
cg01827633	2	219610103	2.58E-127	0.294	0.403	-0.109	*TTLL4*	5.41E-16	-0.051
cg01870865	3	48507087	2.95E-127	0.286	0.365	-0.079	*TREX1*	2.49E-20	-0.038
cg08243626	10	6442501	3.19E-127	0.309	0.416	-0.107	NA	7.61E-18	-0.053
cg01012242	7	140043078	3.51E-127	0.325	0.411	-0.086	*SLC37A3*	1.61E-14	-0.037
cg13603332	9	108053809	3.98E-127	0.321	0.423	-0.102	*SLC44A1*	4.46E-09	-0.032
cg19982684	3	128403035	4.16E-127	0.318	0.408	-0.090	NA	6.38E-17	-0.037
cg03348161	11	59824089	5.59E-127	0.339	0.450	-0.111	*MS4A3*	3.25E-16	-0.044
cg02788021	13	30882905	5.77E-127	0.310	0.405	-0.095	*KATNAL1*	1.44E-16	-0.045
cg16785938	4	10022984	6.24E-127	0.347	0.423	-0.076	*SLC2A9*	1.82E-15	-0.031
cg25771026	3	196352027	6.71E-127	0.361	0.442	-0.082	NA	2.71E-06	-0.019
cg24371033	2	241807924	7.16E-127	0.293	0.365	-0.073	*AGXT*	7.30E-20	-0.036
cg12866960	12	66635398	7.20E-127	0.389	0.501	-0.112	*IRAK3*	6.22E-10	-0.035
cg18907610	12	11992925	7.21E-127	0.322	0.417	-0.095	*RNU6-19*	3.63E-20	-0.053
cg00907842	5	39219245	7.87E-127	0.256	0.353	-0.097	*FYB*	5.14E-10	-0.033
cg27585822	20	49253552	7.98E-127	0.332	0.405	-0.073	*FAM65C*	5.67E-16	-0.035
cg26266876	3	23957486	8.66E-127	0.217	0.294	-0.077	*RPL15*	4.93E-17	-0.036
cg09832245	16	85494611	8.71E-127	0.291	0.380	-0.090	NA	5.70E-16	-0.041
cg25705508	1	203199023	9.00E-127	0.360	0.441	-0.081	*CHIT1*	4.83E-07	-0.023
cg00578614	6	30070403	9.03E-127	0.333	0.434	-0.101	NA	1.74E-13	-0.040
cg14677983	13	111318022	9.68E-127	0.375	0.447	-0.073	*CARS2*	5.83E-20	-0.037
cg10075819	2	109229337	1.01E-126	0.774	0.697	0.077	*LIMS1*	6.82E-10	0.025
cg03762242	17	9940004	1.02E-126	0.260	0.332	-0.073	*GAS7*	1.32E-15	-0.031
cg19654743	17	75446592	1.07E-126	0.762	0.689	0.073	*SEPT9*	2.75E-16	0.030
cg00530564	12	125036429	1.09E-126	0.254	0.323	-0.069	*NCOR2*	8.78E-08	-0.021
cg22742001	12	110435418	1.10E-126	0.305	0.410	-0.105	*GIT2*	4.11E-15	-0.045
cg25124300	2	31607761	1.30E-126	0.429	0.534	-0.105	*XDH*	2.94E-18	-0.050
cg01787084	16	87371097	1.43E-126	0.294	0.420	-0.126	*FBXO31*	1.05E-19	-0.066
cg19240569	11	63331821	1.65E-126	0.748	0.674	0.074	*HRASLS2*	7.46E-07	0.021

cg15248035	9	36169949	1.70E-126	0.351	0.460	-0.109	CCIN	1.35E-15	-0.045
cg02067584	17	8094552	1.77E-126	0.345	0.461	-0.116	C17orf59	1.11E-10	-0.041
cg16303353	12	2393684	1.78E-126	0.310	0.408	-0.099	CACNA1C	1.72E-15	-0.045
cg02656594	16	27412496	2.12E-126	0.365	0.465	-0.100	IL21R	9.04E-28	-0.064
cg01833923	17	47286719	2.15E-126	0.603	0.542	0.061	GNGT2	3.15E-20	0.030
cg22666015	2	233981885	2.33E-126	0.331	0.450	-0.119	INPP5D	8.42E-10	-0.039
cg02423817	11	67203661	2.47E-126	0.681	0.598	0.083	PTPRCAP	8.07E-04	0.013
cg22862003	21	42797588	2.54E-126	0.427	0.698	-0.270	MX1	7.87E-63	-0.187
cg01057573	5	118683890	2.74E-126	0.369	0.477	-0.108	TNFAIP8	5.69E-11	-0.038
cg02551745	3	156809115	3.67E-126	0.322	0.424	-0.103	LOC10049885	1.16E-14	-0.043
cg26925688	19	4303957	4.11E-126	0.595	0.527	0.068	FSD1	6.14E-16	0.026
cg04346283	6	82459454	4.29E-126	0.274	0.365	-0.091	FAM46A	1.93E-20	-0.050
cg19405329	15	49440632	5.00E-126	0.391	0.497	-0.106	COPS2	8.93E-12	-0.040
cg03655701	1	24885664	5.16E-126	0.294	0.389	-0.096	NCMAP	1.40E-21	-0.049
cg02952913	1	247336686	5.28E-126	0.317	0.430	-0.113	ZNF124	1.64E-19	-0.059
cg12030667	3	48471659	5.88E-126	0.211	0.277	-0.066	PLXNB1	9.64E-09	-0.020
cg21492378	9	123850279	6.82E-126	0.388	0.500	-0.113	CNTRL	9.19E-09	-0.036
cg06491415	6	166825479	7.43E-126	0.767	0.703	0.065	RPS6KA2	1.05E-13	0.025
cg22111527	11	69260136	7.86E-126	0.297	0.392	-0.094	NA	5.93E-08	-0.028
cg21002528	11	45903754	8.04E-126	0.380	0.474	-0.094	CRY2	8.64E-15	-0.039
cg22526531	1	35913782	8.51E-126	0.469	0.575	-0.106	KIAA0319L	1.79E-07	-0.030
cg04144521	2	191276183	8.99E-126	0.348	0.442	-0.094	MFSD6	1.84E-13	-0.039
cg06857116	17	15885326	1.03E-125	0.419	0.518	-0.099	ZSWIM7	9.88E-23	-0.054
cg02374486	10	72362809	1.21E-125	0.725	0.639	0.086	PRF1	3.72E-13	0.035
cg01182697	1	54520432	1.39E-125	0.296	0.384	-0.088	TCEANC2	3.06E-16	-0.040
cg21696012	1	24131000	1.42E-125	0.392	0.498	-0.106	HMGCL	3.97E-11	-0.037
cg17586302	6	144013969	2.06E-125	0.420	0.523	-0.103	PHACTR2	2.95E-10	-0.036
cg07051833	12	93808395	2.16E-125	0.314	0.388	-0.074	UBE2N	3.40E-12	-0.028
cg25957124	16	21171084	2.64E-125	0.278	0.356	-0.079	TMEM159	1.61E-09	-0.025
cg14165142	20	3778655	2.81E-125	0.738	0.653	0.085	CDC25B	2.60E-07	0.025
cg27057329	15	40803244	3.27E-125	0.269	0.353	-0.085	NA	5.22E-13	-0.037
cg16391678	16	30485597	3.61E-125	0.787	0.700	0.087	ITGAL	1.59E-06	0.022
cg18473733	19	16437362	3.96E-125	0.654	0.588	0.065	KLF2	1.67E-15	0.026
cg09121543	17	61774794	4.58E-125	0.516	0.405	0.111	LIMD2	7.44E-10	0.035
cg25017777	1	9298817	4.87E-125	0.354	0.422	-0.068	H6PD	5.87E-15	-0.028
cg25823926	12	94153360	4.87E-125	0.239	0.321	-0.082	CRADD	4.03E-26	-0.051
cg02654449	16	27254785	4.90E-125	0.198	0.252	-0.054	NSMCE1	8.23E-18	-0.027
cg02573357	1	203738799	5.01E-125	0.280	0.370	-0.089	LAX1	4.19E-20	-0.044
cg14369981	9	129294452	5.24E-125	0.356	0.438	-0.083	NA	2.07E-07	-0.021
cg04340595	1	9789174	5.32E-125	0.624	0.529	0.095	CLSTN1	7.68E-18	0.045
cg08700306	19	33686390	5.63E-125	0.332	0.399	-0.068	LRP3	5.80E-19	-0.032
cg14094409	12	122712093	6.16E-125	0.765	0.691	0.074	DIABLO	5.18E-12	0.028
cg17451941	1	236254344	6.17E-125	0.302	0.397	-0.096	NA	4.13E-18	-0.050
cg17007896	11	69261044	6.83E-125	0.309	0.385	-0.076	NA	1.96E-14	-0.031
cg08132858	7	101361395	8.02E-125	0.319	0.398	-0.079	NA	5.43E-16	-0.031
cg06242730	7	1064100	9.29E-125	0.253	0.328	-0.075	C7orf50	2.30E-17	-0.037
cg06719445	14	89925468	9.57E-125	0.328	0.432	-0.104	FOXN3	1.50E-15	-0.046
cg09020199	6	30297320	1.09E-124	0.422	0.516	-0.093	TRIM39	1.53E-11	-0.032
cg12165551	11	8385712	1.09E-124	0.259	0.340	-0.080	NA	3.25E-16	-0.036
cg08181251	10	99443455	1.10E-124	0.297	0.383	-0.086	AVPI1	9.10E-15	-0.036
cg12129117	1	223940656	1.21E-124	0.322	0.393	-0.071	CAPN2	1.19E-11	-0.022
cg27051683	2	242802069	1.24E-124	0.594	0.526	0.067	PDCD1	3.59E-07	0.015
cg23756264	11	68605511	1.24E-124	0.248	0.319	-0.071	CPT1A	6.11E-15	-0.029
cg22062741	8	1897075	1.30E-124	0.412	0.512	-0.100	ARHGEF10	2.84E-12	-0.037
cg02249390	6	31550612	1.56E-124	0.757	0.687	0.070	LTB	4.83E-13	0.026
cg22358291	4	10101553	1.66E-124	0.799	0.735	0.064	WDR1	1.15E-12	0.027
cg14554468	17	28696496	1.71E-124	0.279	0.380	-0.101	NA	1.80E-14	-0.041
cg21242144	5	148808487	1.77E-124	0.252	0.318	-0.065	MIR143HG	3.54E-15	-0.028
cg26655340	5	154230141	1.97E-124	0.341	0.421	-0.080	C5orf4	2.45E-18	-0.037
cg07280097	3	128779601	2.01E-124	0.296	0.383	-0.087	GP9	2.54E-11	-0.034
cg01623438	20	57582894	2.13E-124	0.262	0.345	-0.083	CTSZ	4.95E-17	-0.040
cg12940181	2	145353012	2.25E-124	0.281	0.380	-0.099	NA	2.90E-09	-0.034
cg07030287	2	242174797	2.38E-124	0.306	0.372	-0.066	HDLBP	1.01E-10	-0.023
cg16024530	11	128673534	2.57E-124	0.257	0.309	-0.051	FLI1	1.39E-17	-0.024
cg12707346	12	64960957	2.63E-124	0.316	0.407	-0.092	NA	1.44E-16	-0.044
cg09825327	1	211503219	2.71E-124	0.770	0.696	0.074	TRAF5	1.09E-14	0.031
cg25941751	2	46613544	2.89E-124	0.305	0.399	-0.093	EPAS1	1.13E-11	-0.036
cg06655349	19	10332165	2.95E-124	0.377	0.490	-0.114	S1PR2	7.62E-11	-0.041
cg19595244	1	110526158	3.51E-124	0.386	0.479	-0.092	AHCYL1	3.74E-13	-0.039
cg21727359	6	82459006	3.55E-124	0.328	0.424	-0.096	FAM46A	6.69E-16	-0.042
cg18535410	13	113793268	3.62E-124	0.323	0.417	-0.094	F10	1.27E-11	-0.036
**cg01582066**	22	32057065	3.83E-124	0.593	0.510	0.083	NA	0.519	0.003
cg03610527	2	242174889	4.47E-124	0.454	0.553	-0.099	HDLBP	9.62E-08	-0.025

cg18838431	11	74178800	4.91E-124	0.271	0.353	-0.081	*KCNE3*	2.82E-12	-0.031
cg16501560	21	46078007	5.72E-124	0.668	0.584	0.084	*TSPEAR*	1.39E-10	0.025
cg04846203	1	167690438	5.93E-124	0.234	0.332	-0.098	*MPZL1*	2.37E-20	-0.052
cg06758539	5	75975285	6.09E-124	0.459	0.553	-0.094	*IQGAP2*	3.80E-04	-0.018
cg15453278	11	67134607	6.11E-124	0.320	0.385	-0.065	*CLCF1*	6.74E-12	-0.023
cg20682563	5	133460874	6.11E-124	0.694	0.624	0.070	*TCF7*	1.21E-13	0.030
cg12136387	12	116820108	6.16E-124	0.293	0.374	-0.081	NA	2.14E-14	-0.035
cg07804470	13	99201496	6.70E-124	0.309	0.414	-0.105	*STK24*	4.99E-13	-0.044
cg12971694	9	35618412	8.98E-124	0.266	0.343	-0.077	*CD72*	4.00E-19	-0.036
cg09046309	20	45947226	9.41E-124	0.349	0.457	-0.108	*ZMYND8*	4.75E-11	-0.039
cg24597363	4	1294996	9.95E-124	0.393	0.506	-0.113	*MAEA*	1.35E-09	-0.037
cg03211593	11	44731693	1.11E-123	0.250	0.313	-0.063	NA	4.90E-17	-0.028
cg21073459	19	54604098	1.25E-123	0.329	0.400	-0.072	*OSCAR*	2.05E-08	-0.021
cg20157577	17	61780203	1.36E-123	0.744	0.679	0.065	*STRADA*	7.37E-18	0.032
cg03926751	4	88140261	1.48E-123	0.315	0.424	-0.109	*KLHL8*	3.19E-10	-0.041
cg21658670	19	41930894	1.51E-123	0.352	0.429	-0.076	*BCKDHA*	7.67E-07	-0.021
cg13298466	12	6658164	1.72E-123	0.463	0.579	-0.116	*IFFO1*	1.34E-11	-0.044
cg14118850	2	10447890	1.75E-123	0.384	0.485	-0.101	*HPCAL1*	1.96E-15	-0.044
cg26162326	7	75957061	1.90E-123	0.364	0.459	-0.095	*YWHAG*	5.51E-07	-0.026
cg12118504	6	33265393	1.90E-123	0.314	0.395	-0.081	*RGL2*	1.33E-14	-0.035
cg03920999	3	10334760	2.03E-123	0.270	0.342	-0.072	*GHRLOS*	4.91E-17	-0.033
cg16509045	9	77502963	2.54E-123	0.389	0.489	-0.100	*TRPM6*	6.95E-11	-0.037
cg16502747	14	23531625	2.62E-123	0.356	0.432	-0.077	*ACIN1*	1.57E-10	-0.027
cg19023589	13	114182159	2.75E-123	0.470	0.593	-0.123	*TMCO3*	6.47E-11	-0.042
cg13831136	6	30070342	2.93E-123	0.336	0.428	-0.093	NA	7.16E-10	-0.033
cg24422316	11	60930346	2.94E-123	0.369	0.461	-0.092	*VPS37C*	2.02E-25	-0.052
cg25356504	17	26707476	2.99E-123	0.268	0.348	-0.079	*SARM1*	2.72E-11	-0.029
cg09789874	12	125262045	3.11E-123	0.349	0.467	-0.118	NA	4.62E-11	-0.044
cg06352352	16	67181466	3.13E-123	0.392	0.477	-0.086	*C16orf70*	8.86E-13	-0.034
cg02241759	3	119528300	3.17E-123	0.301	0.399	-0.098	*NR1I2*	1.17E-09	-0.032
cg20981615	4	48136280	3.19E-123	0.756	0.682	0.074	*TXK*	5.05E-21	0.040
cg25888881	10	31288287	3.23E-123	0.334	0.420	-0.086	*ZNF438*	1.49E-20	-0.044
cg03040292	1	200847096	3.61E-123	0.768	0.692	0.076	NA	6.84E-18	0.032
cg02570354	3	31728749	4.18E-123	0.286	0.385	-0.099	*OSBPL10*	5.57E-20	-0.049
cg02770745	11	3926837	4.19E-123	0.368	0.481	-0.112	*STIM1*	2.19E-08	-0.034
cg01014262	16	48533930	4.89E-123	0.235	0.311	-0.076	NA	1.48E-18	-0.039
cg11525409	2	74236068	5.17E-123	0.427	0.519	-0.092	NA	2.00E-12	-0.033
cg01692842	11	134126364	5.19E-123	0.414	0.522	-0.108	*ACAD8*	0.002	-0.019
cg08773226	6	135061398	5.72E-123	0.404	0.507	-0.103	NA	2.04E-08	-0.031
cg16408081	11	67205869	5.80E-123	0.625	0.547	0.078	*PTPRCAP*	1.10E-13	0.027
cg00499700	5	76116088	7.34E-123	0.367	0.443	-0.076	*F2RL1*	9.81E-15	-0.032
cg24760467	10	102760784	8.00E-123	0.474	0.580	-0.105	*LZTS2*	9.33E-24	-0.058
cg10283505	11	94278912	8.06E-123	0.428	0.523	-0.095	*FUT4*	2.83E-15	-0.041
cg12192749	7	17029795	8.42E-123	0.354	0.449	-0.095	NA	5.18E-19	-0.048
cg09378756	14	24569543	9.19E-123	0.258	0.340	-0.082	*PCK2*	1.32E-13	-0.036
cg14102437	2	33359688	9.20E-123	0.300	0.402	-0.102	*LTBP1*	5.71E-22	-0.057
cg07434244	1	209847729	9.52E-123	0.278	0.337	-0.060	*G0S2*	2.10E-11	-0.022
cg22626169	6	6890951	1.04E-122	0.407	0.507	-0.101	NA	1.17E-19	-0.050
cg06801028	10	51575592	1.21E-122	0.307	0.398	-0.091	*NCOA4*	1.61E-11	-0.035
cg18160691	11	68081686	1.28E-122	0.315	0.383	-0.068	*LRP5*	1.56E-10	-0.023
cg05249836	22	45609402	1.54E-122	0.430	0.545	-0.116	*KIAA0930*	1.66E-11	-0.041
cg23361127	5	73939283	1.55E-122	0.382	0.477	-0.095	NA	2.10E-16	-0.040
cg11922563	5	75016955	1.59E-122	0.367	0.475	-0.108	NA	6.77E-09	-0.035
cg11083848	14	76371681	1.59E-122	0.352	0.457	-0.105	*TTLL5*	8.21E-14	-0.042
cg07866632	16	28584802	1.76E-122	0.301	0.372	-0.070	*CCDC101*	5.68E-09	-0.022
cg17839611	17	47286802	1.86E-122	0.686	0.611	0.075	*GNGT2*	4.25E-19	0.036
cg00446123	20	62367888	1.88E-122	0.656	0.533	0.123	*LIME1*	2.90E-06	0.030
cg00760729	2	161236088	1.91E-122	0.312	0.407	-0.095	*RBMS1*	6.55E-12	-0.036
cg09519218	10	126390317	1.93E-122	0.317	0.377	-0.061	*FAM53B*	4.41E-17	-0.027
cg02481000	1	2082349	2.03E-122	0.321	0.385	-0.065	*PRKCZ*	4.03E-08	-0.021
cg19348484	15	91413236	2.08E-122	0.233	0.306	-0.073	*FURIN*	9.39E-12	-0.027
cg10959672	1	35972127	2.44E-122	0.786	0.716	0.071	*KIAA0319L*	3.00E-14	0.032
cg13703437	5	39219698	2.52E-122	0.331	0.435	-0.104	*FYB*	4.42E-12	-0.041
cg22535089	13	114184462	2.57E-122	0.351	0.444	-0.094	*TMCO3*	5.18E-07	-0.026
cg26076724	6	15090163	2.63E-122	0.334	0.423	-0.089	NA	2.04E-10	-0.031
cg07814932	1	120437090	2.69E-122	0.499	0.609	-0.110	*ADAM30*	5.48E-14	-0.044
cg00554993	14	23588616	3.23E-122	0.251	0.338	-0.087	*CEBPE*	1.26E-12	-0.035
cg04334723	19	13054427	3.31E-122	0.342	0.441	-0.099	*CALR*	2.40E-15	-0.044
cg14272075	17	55122538	3.86E-122	0.390	0.501	-0.111	NA	1.05E-11	-0.040
cg11293275	5	131543977	3.88E-122	0.273	0.348	-0.075	*P4HA2*	9.75E-16	-0.037
cg18351781	19	4950888	4.04E-122	0.355	0.468	-0.113	*UHRF1*	3.62E-08	-0.033
cg08408668	19	1173241	4.15E-122	0.336	0.407	-0.071	*SBNO2*	3.65E-22	-0.034
cg23079808	12	6493003	4.52E-122	0.250	0.345	-0.095	*LTBR*	1.22E-09	-0.034

cg12809098	8	142165310	4.94E-122	0.500	0.612	-0.111	*DENND3*	3.24E-06	-0.028
cg22516975	17	1617465	5.54E-122	0.307	0.376	-0.068	*MIR22HG*	9.88E-11	-0.025
cg01923724	1	58864209	5.56E-122	0.398	0.479	-0.081	NA	2.63E-09	-0.026
cg08817867	17	19656554	5.64E-122	0.235	0.304	-0.070	NA	4.69E-20	-0.031
cg14377681	17	37875709	5.90E-122	0.338	0.420	-0.082	*ERBB2*	2.35E-18	-0.041
cg17841267	10	112117449	6.01E-122	0.800	0.727	0.074	NA	1.69E-08	0.024
cg16657152	8	125784769	6.92E-122	0.309	0.406	-0.097	NA	1.57E-16	-0.047
cg24686551	3	134047274	7.10E-122	0.326	0.436	-0.109	NA	8.55E-09	-0.035
cg06311422	6	56406336	7.24E-122	0.288	0.384	-0.096	*RNU6-71*	2.90E-25	-0.060
cg11311352	19	56058710	7.90E-122	0.222	0.286	-0.064	NA	5.01E-18	-0.031
cg08947915	4	1742463	8.00E-122	0.310	0.406	-0.096	*TACC3*	7.01E-06	-0.024
cg08038629	1	17751673	8.21E-122	0.222	0.290	-0.068	*RCC2*	9.25E-18	-0.035
cg07863022	17	75315108	8.82E-122	0.229	0.313	-0.084	*SEPT9*	4.19E-04	-0.018
cg09354050	21	43824262	9.24E-122	0.793	0.721	0.072	*UBASH3A*	2.66E-13	0.030
cg26215727	12	6485537	9.33E-122	0.388	0.470	-0.082	*SCNN1A*	1.15E-07	-0.025
cg12313149	2	136763629	9.79E-122	0.389	0.499	-0.110	NA	1.77E-11	-0.040
cg06380691	13	114828264	9.94E-122	0.257	0.331	-0.074	*RASA3*	1.11E-10	-0.027
cg01178099	3	177391387	1.03E-121	0.385	0.491	-0.106	*LINC00578*	1.60E-10	-0.037
cg26534477	17	42147600	1.07E-121	0.238	0.313	-0.075	*G6PC3*	2.46E-13	-0.028
cg15029631	12	52078762	1.09E-121	0.383	0.484	-0.101	*SCN8A*	5.78E-09	-0.033
cg09467433	3	171283606	1.09E-121	0.375	0.490	-0.115	NA	1.10E-09	-0.039
cg07118376	8	62624872	1.13E-121	0.368	0.466	-0.098	*ASPH*	2.45E-17	-0.049
cg03910874	6	209712	1.28E-121	0.677	0.601	0.076	NA	7.28E-06	0.018
cg16102040	4	146685656	1.29E-121	0.518	0.614	-0.096	*ZNF827*	1.42E-10	-0.030
cg20070090	1	153363489	1.29E-121	0.345	0.444	-0.098	*S100A8*	4.04E-13	-0.038
cg20893838	6	7145478	1.40E-121	0.306	0.384	-0.078	*RREB1*	1.21E-09	-0.027
cg06812844	21	45773782	1.42E-121	0.312	0.406	-0.094	*TRPM2*	4.30E-14	-0.041
cg22848646	1	203171499	1.60E-121	0.362	0.467	-0.105	NA	1.10E-17	-0.052
cg02673417	6	137105249	1.64E-121	0.250	0.330	-0.081	*MAP3K5*	2.35E-18	-0.040
cg08044694	19	15391927	1.77E-121	0.316	0.408	-0.093	*BRD4*	2.01E-14	-0.039
cg14578677	4	38859770	1.78E-121	0.398	0.516	-0.119	*TLR6*	6.38E-10	-0.043
cg18813599	8	29937472	2.04E-121	0.747	0.679	0.068	*MIR548O2*	4.08E-23	0.036
cg22824738	12	54765988	2.15E-121	0.241	0.303	-0.062	*ZNF385A*	2.12E-12	-0.025
cg01821022	12	42775908	2.17E-121	0.306	0.395	-0.089	*PPHLN1*	4.39E-09	-0.030
cg22242148	15	74215283	2.45E-121	0.306	0.391	-0.086	*LOXL1-AS1*	9.09E-13	-0.035
cg08730245	12	53902893	2.66E-121	0.509	0.592	-0.083	*NPFF*	1.27E-10	-0.031
cg21697512	1	208081541	2.69E-121	0.435	0.530	-0.096	*CD34*	3.03E-19	-0.050
cg08473858	11	118476352	2.74E-121	0.326	0.391	-0.065	*PHLDB1*	9.21E-20	-0.031
cg11125805	5	150678162	2.88E-121	0.309	0.416	-0.107	*SLC36A3*	1.92E-12	-0.041
cg08908247	11	66055170	2.99E-121	0.324	0.391	-0.067	*YIF1A*	1.20E-12	-0.026
cg11893955	8	28918821	3.04E-121	0.759	0.685	0.074	NA	6.36E-13	0.029
cg07055315	2	32490835	3.15E-121	0.340	0.446	-0.106	*NLRC4*	4.23E-12	-0.041
cg13477111	1	150951737	3.23E-121	0.227	0.295	-0.068	NA	3.83E-16	-0.032
cg18997983	5	1109031	3.28E-121	0.366	0.463	-0.097	*SLC12A7*	1.56E-12	-0.042
cg13736811	19	41873930	3.41E-121	0.295	0.394	-0.100	*TMEM91*	8.51E-15	-0.046
cg11324504	22	38474147	4.17E-121	0.316	0.402	-0.086	*SLC16A8*	4.88E-09	-0.025
cg00841141	2	37416819	4.38E-121	0.426	0.522	-0.096	*SULT6B1*	1.87E-09	-0.032
cg08633074	16	11837719	4.40E-121	0.300	0.404	-0.104	*TXNDC11*	6.27E-12	-0.045
cg07546654	11	1320546	4.45E-121	0.354	0.464	-0.109	*TOLLIP*	1.94E-13	-0.046
cg26873329	5	32522933	6.63E-121	0.411	0.520	-0.109	NA	1.84E-08	-0.030
cg15377871	14	23588325	7.07E-121	0.502	0.594	-0.092	*CEBPE*	8.66E-13	-0.036
cg14210726	17	76136952	7.16E-121	0.356	0.429	-0.073	*TMC8*	1.10E-07	-0.020
cg03005293	1	151805241	7.70E-121	0.272	0.346	-0.074	*RORC*	3.36E-14	-0.029
cg09740468	6	3025325	9.32E-121	0.370	0.460	-0.090	NA	3.13E-11	-0.033
cg04165073	11	128388475	9.51E-121	0.720	0.645	0.074	*ETS1*	1.10E-14	0.032
cg00913954	1	36852956	9.63E-121	0.435	0.535	-0.100	*STK40*	6.43E-23	-0.056
cg12497543	10	97515377	1.01E-120	0.307	0.396	-0.089	*ENTPD1*	3.52E-13	-0.040
cg01870907	17	77775687	1.14E-120	0.182	0.242	-0.060	NA	1.02E-11	-0.023
cg07110356	17	56355431	1.20E-120	0.423	0.543	-0.119	*MPO*	6.03E-05	-0.028
cg21547649	2	9142741	1.22E-120	0.325	0.408	-0.083	*MBOAT2*	1.27E-11	-0.034
cg01999566	6	30624449	1.22E-120	0.256	0.322	-0.065	*DHX16*	1.18E-16	-0.031
cg10453850	6	32905320	1.34E-120	0.283	0.376	-0.093	*HLA-DMB*	2.07E-15	-0.042
cg16998950	5	95252658	1.42E-120	0.332	0.447	-0.115	*ELL2*	1.17E-09	-0.037
cg11628739	17	60731254	1.72E-120	0.231	0.284	-0.052	*MRC2*	1.57E-19	-0.028
cg16824282	3	128779590	1.81E-120	0.377	0.479	-0.102	*GP9*	7.48E-10	-0.035
cg05844788	3	10336053	1.88E-120	0.227	0.292	-0.065	*GHRL*	1.35E-22	-0.034
cg26843872	16	30368792	1.90E-120	0.271	0.358	-0.088	*TBC1D10B*	2.53E-14	-0.038
cg05696877	1	79088769	1.93E-120	0.408	0.671	-0.263	*IFI44L*	2.85E-50	-0.196
cg25070639	17	41170693	2.06E-120	0.263	0.346	-0.082	*VAT1*	7.48E-10	-0.028
cg02787852	16	27414536	2.15E-120	0.737	0.669	0.067	*IL21R*	2.19E-13	0.027
cg04079215	1	95095221	2.21E-120	0.366	0.449	-0.083	NA	8.10E-11	-0.031
cg12709880	18	21163172	2.80E-120	0.448	0.554	-0.106	*NPC1*	9.24E-10	-0.036
cg10257049	5	154230308	3.23E-120	0.310	0.390	-0.081	*C5orf4*	1.52E-12	-0.032

cg01440934	1	47051752	3.38E-120	0.376	0.483	-0.107	*MKNK1*	1.21E-09	-0.038
cg14611767	9	134127575	3.52E-120	0.433	0.539	-0.106	NA	1.88E-12	-0.040
cg17740645	17	37894413	3.71E-120	0.275	0.349	-0.073	*GRB7*	2.70E-12	-0.025
cg14885762	17	75446450	3.76E-120	0.768	0.692	0.076	*SEPT9*	1.48E-08	0.025
cg26002008	5	1477719	4.95E-120	0.304	0.378	-0.074	*LPCAT1*	2.16E-09	-0.023
cg25968394	7	104604511	5.63E-120	0.424	0.525	-0.101	NA	6.78E-08	-0.028
cg24914483	18	2653997	5.96E-120	0.338	0.443	-0.105	*CBX3P2*	5.36E-07	-0.030
cg26732720	11	64392533	6.73E-120	0.218	0.278	-0.061	*NRXN2*	1.45E-25	-0.037
cg26538140	12	6996791	6.77E-120	0.300	0.391	-0.091	NA	8.07E-08	-0.029
cg23924737	17	55928753	6.86E-120	0.431	0.527	-0.096	*MRPS23*	1.89E-13	-0.039
cg08840010	1	8000314	7.14E-120	0.350	0.461	-0.111	*TNFRSF9*	1.40E-06	-0.030
cg15457217	2	224780409	7.36E-120	0.505	0.602	-0.097	*WDFY1*	1.78E-07	-0.026
cg26518580	1	111742515	7.64E-120	0.770	0.700	0.070	*DENND2D*	3.21E-10	0.027
cg02097152	13	29291701	8.56E-120	0.390	0.482	-0.092	*SLC46A3*	5.68E-19	-0.046
cg20127859	8	144656271	8.76E-120	0.216	0.280	-0.064	*C8orf73*	3.78E-09	-0.022
cg09017001	1	31870508	9.38E-120	0.353	0.440	-0.087	NA	1.21E-11	-0.033
cg00691123	3	11632974	9.40E-120	0.408	0.498	-0.090	*VGLL4*	2.24E-15	-0.041
cg11464160	2	131870398	9.71E-120	0.307	0.398	-0.091	*PLEKHB2*	4.71E-07	-0.026
cg12903530	1	181097897	9.82E-120	0.398	0.456	-0.059	NA	1.26E-20	-0.030
cg17102495	17	79421644	1.03E-119	0.353	0.424	-0.071	*BAHCC1*	1.13E-07	-0.021
cg19513582	7	129481640	1.09E-119	0.465	0.561	-0.096	*UBE2H*	2.73E-06	-0.025
cg24906819	8	8231531	1.13E-119	0.372	0.475	-0.102	*SGK223*	2.21E-11	-0.037
cg12249234	17	25867613	1.16E-119	0.320	0.428	-0.108	*KSR1*	1.45E-11	-0.043
cg00759807	16	89390789	1.36E-119	0.729	0.635	0.094	*LOC10028703*	1.52E-09	0.032
cg01636582	11	844593	1.39E-119	0.228	0.284	-0.056	*TSPAN4*	2.12E-13	-0.023
cg21163444	12	54765670	1.68E-119	0.381	0.453	-0.073	*ZNF385A*	6.89E-12	-0.026
cg08195176	11	116781303	1.69E-119	0.436	0.511	-0.075	*SIK3*	1.51E-13	-0.028
cg22941573	20	48761279	1.81E-119	0.460	0.559	-0.099	*TMEM189*	2.57E-18	-0.045
cg25936902	13	30982971	1.86E-119	0.328	0.407	-0.079	NA	3.31E-11	-0.029
cg13047308	11	47471339	1.90E-119	0.516	0.611	-0.095	*RAPSN*	1.27E-08	-0.031
cg25025181	1	246378510	1.92E-119	0.303	0.402	-0.099	*SMYD3*	8.03E-12	-0.040
cg24777950	14	25046121	2.06E-119	0.313	0.412	-0.099	*CTSG*	3.51E-08	-0.030
cg03187073	6	4890079	2.28E-119	0.304	0.404	-0.100	*CDYL*	8.72E-15	-0.045
cg18569623	14	89631502	2.30E-119	0.277	0.356	-0.079	*FOXN3*	4.77E-18	-0.038
cg14628914	6	24659344	2.32E-119	0.448	0.539	-0.090	*TDP2*	1.30E-10	-0.031
cg05403316	2	55339939	2.71E-119	0.238	0.312	-0.074	NA	1.12E-15	-0.032
cg01710147	1	207233419	2.93E-119	0.299	0.380	-0.081	*PFKFB2*	8.94E-15	-0.036
cg09859659	8	142180109	2.97E-119	0.520	0.626	-0.106	*DENND3*	3.59E-10	-0.036
cg01154505	2	112940409	3.09E-119	0.837	0.767	0.069	*FBLN7*	4.84E-15	0.032
cg23954655	13	99223562	3.16E-119	0.541	0.472	0.069	*STK24*	0.015	0.008
cg14648237	17	64422393	3.45E-119	0.285	0.361	-0.077	*PRKCA*	3.86E-14	-0.033
cg09569347	6	30460548	3.69E-119	0.649	0.583	0.066	*HLA-E*	1.16E-14	0.026
cg08859278	2	98329691	3.72E-119	0.736	0.661	0.075	*ZAP70*	5.88E-15	0.032
cg07241925	4	1294566	3.88E-119	0.330	0.416	-0.086	*MAEA*	7.36E-11	-0.030
cg16728539	12	2451169	3.91E-119	0.329	0.426	-0.097	*CACNA1C*	1.65E-14	-0.042
cg12339328	20	18491484	4.28E-119	0.414	0.509	-0.096	*SEC23B*	5.74E-15	-0.043
cg09782560	15	92058845	4.66E-119	0.290	0.381	-0.091	NA	1.77E-17	-0.046
cg02927682	1	54844424	5.61E-119	0.319	0.428	-0.109	*SSBP3*	1.68E-05	-0.028
cg20805133	2	242802192	6.06E-119	0.706	0.610	0.096	*PDCD1*	9.10E-08	0.029
cg02078710	14	59066094	6.37E-119	0.730	0.661	0.068	NA	2.47E-15	0.028
cg26535158	17	40175841	6.42E-119	0.315	0.389	-0.074	*NKIRAS2*	1.01E-06	-0.018
cg18603538	7	127894591	6.89E-119	0.734	0.660	0.074	*LEP*	0.014	0.010
cg23528247	3	99832772	6.96E-119	0.298	0.398	-0.100	*C3orf26*	4.41E-14	-0.042
cg15982099	11	47399813	7.03E-119	0.293	0.390	-0.097	*SPI1*	2.37E-14	-0.041
cg22528270	7	151505116	7.93E-119	0.279	0.377	-0.098	*PRKAG2*	2.29E-04	-0.021
cg18638581	2	75059602	8.01E-119	0.279	0.350	-0.071	*HK2*	3.09E-13	-0.031
cg13807386	9	139098502	8.88E-119	0.325	0.403	-0.079	*QSOX2*	1.33E-12	-0.029
cg09379497	6	30624467	8.98E-119	0.435	0.524	-0.089	*DHX16*	6.24E-11	-0.035
cg21638533	12	6658625	9.10E-119	0.269	0.364	-0.095	*IFFO1*	4.94E-07	-0.028
cg19238415	11	32916120	9.92E-119	0.279	0.374	-0.096	*QSER1*	7.18E-17	-0.047
cg26588076	17	17741631	1.06E-118	0.242	0.322	-0.080	*SREBF1*	3.75E-10	-0.030
cg27485921	2	46747379	1.08E-118	0.295	0.377	-0.082	*ATP6V1E2*	1.08E-09	-0.030
cg18245281	20	57583076	1.09E-118	0.256	0.329	-0.073	*CTSZ*	5.95E-15	-0.029
cg13135241	12	104765292	1.12E-118	0.321	0.404	-0.083	NA	1.68E-15	-0.037
cg21893651	6	30001368	1.16E-118	0.363	0.460	-0.097	*ZNRD1-AS1*	3.68E-05	-0.023
cg24505713	11	10770035	1.18E-118	0.229	0.301	-0.072	NA	9.74E-14	-0.032
cg15519096	1	24833311	1.22E-118	0.810	0.743	0.068	*RCAN3*	1.24E-10	0.026
cg07005444	17	3820796	1.37E-118	0.317	0.386	-0.069	*P2RX1*	7.48E-10	-0.022
cg24339704	19	2529022	1.41E-118	0.432	0.534	-0.102	*GNG7*	4.92E-08	-0.031
cg24428600	1	37952273	1.43E-118	0.188	0.242	-0.054	NA	1.83E-14	-0.024
cg21574244	15	42568411	1.55E-118	0.347	0.448	-0.101	*GANC*	3.84E-10	-0.035
cg26253663	22	25799504	1.55E-118	0.374	0.486	-0.112	NA	6.20E-15	-0.045
cg00201133	10	35740216	1.55E-118	0.286	0.380	-0.094	*CCNY*	2.13E-23	-0.052

cg09032544	1	167487295	1.86E-118	0.763	0.681	0.082	*CD247*	5.18E-11	0.030
cg25170017	11	64644487	1.94E-118	0.635	0.536	0.099	*EHD1*	0.002	0.017
cg00483217	2	25321917	1.99E-118	0.365	0.448	-0.083	*EFR3B*	8.68E-09	-0.029
cg06312985	7	32961866	2.25E-118	0.278	0.364	-0.086	*RP9P*	4.32E-13	-0.038
cg03321813	4	84257153	2.39E-118	0.262	0.335	-0.074	*HPSE*	2.64E-11	-0.028
cg00733115	6	37105406	2.41E-118	0.706	0.642	0.064	NA	5.93E-05	0.014
cg08830502	16	87588559	2.41E-118	0.494	0.576	-0.082	NA	1.52E-11	-0.033
cg20184271	12	14413090	2.52E-118	0.387	0.484	-0.097	NA	3.33E-13	-0.038
cg00044665	16	66400411	2.65E-118	0.304	0.373	-0.069	*CDH5*	7.76E-17	-0.031
cg14135809	9	140502926	3.30E-118	0.300	0.374	-0.073	*ARRDC1*	6.90E-12	-0.028
cg04261496	7	4753002	3.32E-118	0.299	0.390	-0.091	*FOXK1*	1.39E-07	-0.025
cg18512352	11	47633146	3.82E-118	0.262	0.336	-0.074	NA	7.71E-15	-0.035
cg15958424	3	132036067	3.84E-118	0.320	0.406	-0.085	*ACPP*	1.75E-12	-0.035
cg02420027	13	41632640	4.80E-118	0.361	0.432	-0.071	NA	8.63E-10	-0.026
cg20909017	12	54805345	5.03E-118	0.228	0.298	-0.069	*ITGA5*	7.87E-15	-0.028
cg13399261	2	102681071	5.19E-118	0.249	0.326	-0.077	NA	1.77E-16	-0.038
cg01462353	2	169939873	5.53E-118	0.365	0.467	-0.102	*DHRS9*	2.91E-08	-0.030
cg01800253	22	22290866	5.55E-118	0.267	0.359	-0.092	*PPM1F*	3.02E-11	-0.036
cg15296856	17	80199608	5.73E-118	0.263	0.318	-0.056	NA	1.39E-17	-0.027
cg13084345	1	36948733	7.41E-118	0.164	0.218	-0.054	*CSF3R*	3.55E-17	-0.027
cg14421700	17	77755965	7.59E-118	0.284	0.353	-0.069	*CBX2*	1.10E-13	-0.026
cg00528616	16	75036318	7.65E-118	0.241	0.311	-0.070	*ZNRF1*	2.59E-15	-0.032
cg13226290	20	1448595	7.89E-118	0.373	0.472	-0.099	*NSFL1C*	7.02E-09	-0.032
cg21211213	5	158127763	7.89E-118	0.223	0.289	-0.066	*EBF1*	3.26E-24	-0.040
cg02212836	6	6589075	8.14E-118	0.647	0.575	0.073	*LY86-AS1*	4.27E-12	0.026
cg02677635	9	129099507	8.58E-118	0.343	0.448	-0.105	*FAM125B*	3.24E-10	-0.038
cg19284751	2	119699789	8.89E-118	0.305	0.390	-0.085	*MARCO*	1.05E-11	-0.035
cg10471548	6	7403054	1.10E-117	0.358	0.456	-0.098	*RIOK1*	5.83E-08	-0.030
cg20078972	19	15391832	1.16E-117	0.306	0.412	-0.106	*BRD4*	4.88E-14	-0.046
cg25949447	2	128169860	1.19E-117	0.412	0.526	-0.114	NA	1.37E-10	-0.037
cg07892374	3	130568122	1.35E-117	0.238	0.299	-0.061	*ATP2C1*	1.35E-11	-0.024
cg06578434	19	1155225	1.36E-117	0.423	0.504	-0.081	*SBNO2*	5.52E-07	-0.021
cg23379806	10	14650660	1.42E-117	0.250	0.321	-0.071	*FAM107B*	1.46E-19	-0.036
cg24155427	1	31242051	1.99E-117	0.248	0.310	-0.062	NA	1.20E-15	-0.029
cg08534016	5	35771584	2.00E-117	0.494	0.591	-0.097	*SPEF2*	4.78E-11	-0.035
cg10085474	4	1221935	2.03E-117	0.322	0.395	-0.073	*CTBP1*	2.29E-08	-0.024
cg17571559	3	11267525	2.24E-117	0.332	0.436	-0.104	*HRH1*	9.22E-12	-0.044
cg16265859	1	19401069	2.25E-117	0.740	0.661	0.078	*UBR4*	8.93E-16	0.037
cg26118358	14	23321005	2.39E-117	0.335	0.417	-0.081	NA	1.01E-09	-0.028
cg00992048	1	1695585	2.71E-117	0.336	0.406	-0.070	*NADK*	1.86E-06	-0.017
cg26348243	6	31540461	2.77E-117	0.642	0.525	0.116	*LTA*	6.27E-14	0.047
cg22284398	2	46119607	2.84E-117	0.219	0.288	-0.070	*PRKCE*	2.15E-16	-0.033
cg21346966	2	238341793	2.91E-117	0.648	0.595	0.053	NA	1.71E-11	0.022
cg26381210	10	88632654	2.92E-117	0.290	0.387	-0.097	*BMPR1A*	1.05E-05	-0.026
cg07799277	5	98368443	3.15E-117	0.315	0.402	-0.087	NA	7.12E-14	-0.038
cg05988358	10	74082489	3.72E-117	0.709	0.652	0.057	NA	5.37E-23	0.032
cg01381934	3	52529064	3.82E-117	0.272	0.361	-0.089	*STAB1*	3.60E-10	-0.032
cg10557578	8	37758453	4.87E-117	0.361	0.433	-0.072	*RAB11FIP1*	4.56E-08	-0.023
cg00795812	2	242802009	5.09E-117	0.658	0.584	0.074	*PDCD1*	3.47E-08	0.023
cg09489567	2	43364053	5.81E-117	0.658	0.597	0.062	NA	3.77E-14	0.027
cg14531665	9	91058614	6.03E-117	0.363	0.430	-0.066	*SPIN1*	1.29E-07	-0.017
cg01081737	8	142238752	6.85E-117	0.448	0.546	-0.098	*SLC45A4*	1.50E-14	-0.042
cg06898168	7	5447353	7.13E-117	0.369	0.429	-0.060	*TNRC18*	1.46E-09	-0.020
cg17344321	7	22617382	8.03E-117	0.247	0.342	-0.094	NA	5.47E-12	-0.038
cg20772590	17	75446431	8.56E-117	0.770	0.699	0.070	*SEPT9*	1.60E-14	0.031
cg23745290	4	106392021	9.51E-117	0.398	0.505	-0.107	*PPA2*	1.37E-05	-0.025
cg04341343	2	8784813	9.82E-117	0.216	0.282	-0.066	NA	2.72E-11	-0.025
cg00859441	12	113799660	1.06E-116	0.446	0.552	-0.106	*PLBD2*	2.55E-10	-0.039
cg09416908	11	86384670	1.19E-116	0.323	0.430	-0.107	*ME3*	2.15E-14	-0.048
cg18112953	20	47448545	1.33E-116	0.395	0.490	-0.095	NA	2.00E-10	-0.036
cg09086087	16	68000763	1.49E-116	0.347	0.404	-0.057	*SLC12A4*	1.40E-09	-0.019
cg26184474	5	61623163	1.60E-116	0.381	0.480	-0.099	*KIF2A*	1.86E-08	-0.029
cg18646851	15	92934809	1.76E-116	0.319	0.411	-0.093	NA	1.90E-10	-0.033
cg10877430	12	113527336	2.11E-116	0.558	0.489	0.070	*DTX1*	1.50E-08	0.018
cg11358405	15	91382972	2.19E-116	0.176	0.250	-0.074	NA	6.16E-09	-0.025
cg02381279	19	16394366	2.46E-116	0.308	0.414	-0.106	NA	4.67E-10	-0.039
cg04789529	8	25072319	2.75E-116	0.374	0.468	-0.094	*DOCK5*	1.07E-19	-0.045
cg23085846	20	62522518	2.82E-116	0.415	0.493	-0.078	*TPD52L2*	5.84E-08	-0.023
cg14268557	6	28874547	2.83E-116	0.372	0.485	-0.112	*TRIM27*	1.47E-06	-0.033
cg03143046	6	43758007	3.21E-116	0.340	0.454	-0.114	NA	1.83E-13	-0.050
cg15210526	1	167733310	3.25E-116	0.405	0.519	-0.114	*MPZL1*	8.64E-09	-0.035
cg25576997	14	56257750	4.11E-116	0.341	0.456	-0.114	*LINC00520*	1.57E-14	-0.051
cg25932290	15	89939252	5.50E-116	0.325	0.420	-0.096	*LOC254559*	5.31E-20	-0.051

cg03594078	8	22131675	5.61E-116	0.361	0.460	-0.099	*PIWIL2*	7.72E-12	-0.038
cg14085060	9	107827215	6.08E-116	0.238	0.303	-0.066	NA	5.32E-17	-0.030
cg06233202	2	64501134	6.14E-116	0.235	0.302	-0.067	NA	2.20E-13	-0.029
cg20830994	17	62401400	6.59E-116	0.698	0.633	0.065	*PECAM1*	3.19E-09	0.021
cg05148465	17	7034129	6.77E-116	0.261	0.346	-0.085	NA	4.63E-09	-0.030
cg19410609	2	31541350	7.05E-116	0.345	0.455	-0.110	NA	5.24E-08	-0.034
cg01763719	2	239463774	7.11E-116	0.366	0.481	-0.115	*LOC151171*	1.43E-05	-0.030
cg10422334	11	67183616	7.15E-116	0.701	0.640	0.060	*CARNS1*	4.13E-14	0.025
cg08545593	20	57582856	8.09E-116	0.327	0.429	-0.102	*CTSZ*	4.00E-12	-0.041
cg26847100	19	10748778	8.61E-116	0.290	0.380	-0.090	*SLC44A2*	4.32E-10	-0.030
cg02111865	2	30371990	8.70E-116	0.384	0.487	-0.103	*YPEL5*	9.15E-05	-0.022
cg06856840	15	80446451	9.74E-116	0.268	0.344	-0.075	*FAH*	3.02E-16	-0.037
cg20281375	19	13946770	9.81E-116	0.267	0.334	-0.067	*LOC284454*	5.15E-22	-0.038
cg27044455	5	149878183	1.12E-115	0.377	0.462	-0.086	NA	1.90E-11	-0.033
cg07906625	1	145021703	1.12E-115	0.223	0.279	-0.056	*PDE4DIP*	1.40E-12	-0.023
cg21773646	17	80085082	1.12E-115	0.665	0.592	0.072	*CCDC57*	2.86E-13	0.030
cg17090611	8	17017866	1.19E-115	0.432	0.545	-0.113	*ZDHHC2*	4.10E-05	-0.024
cg04166500	14	100571607	1.23E-115	0.735	0.670	0.065	*EVL*	2.04E-14	0.028
cg07785552	1	26869620	1.23E-115	0.305	0.368	-0.062	*RPS6KA1*	3.50E-15	-0.026
cg07569288	19	50002551	1.44E-115	0.706	0.624	0.081	*RPS11*	1.41E-12	0.035
cg25248278	17	38696507	1.47E-115	0.729	0.658	0.071	NA	7.93E-14	0.029
cg21204860	17	75446565	1.48E-115	0.627	0.560	0.067	*SEPT9*	1.03E-07	0.020
cg04264075	19	51876470	1.49E-115	0.213	0.266	-0.053	*NKG7*	2.21E-24	-0.033
cg18731680	22	19953712	1.50E-115	0.338	0.423	-0.085	*COMT*	2.52E-15	-0.039
cg06634140	14	95956325	1.74E-115	0.428	0.524	-0.096	NA	1.28E-11	-0.038
cg25960038	17	46667789	1.92E-115	0.293	0.386	-0.093	NA	3.64E-17	-0.043
cg24681307	1	110526191	2.02E-115	0.323	0.419	-0.096	*AHCYL1*	4.17E-17	-0.051
cg20180364	10	94448532	2.05E-115	0.299	0.389	-0.090	*HHEX*	1.05E-16	-0.043
cg23447233	4	1295047	2.33E-115	0.247	0.327	-0.080	*MAEA*	1.85E-06	-0.022
cg24863152	6	151134876	2.48E-115	0.654	0.599	0.056	*PLEKHG1*	5.50E-06	0.014
cg17980404	17	38601676	2.55E-115	0.282	0.363	-0.081	*IGFBP4*	1.22E-24	-0.050
cg13505393	11	76377572	3.14E-115	0.284	0.345	-0.061	*LRRC32*	5.72E-13	-0.027
cg22125902	1	26002535	3.73E-115	0.682	0.604	0.078	*MAN1C1*	4.21E-10	0.029
cg26406563	1	55117536	3.97E-115	0.296	0.367	-0.072	*HEATR8-TTC4*	2.62E-14	-0.033
cg25197500	17	80581805	4.32E-115	0.495	0.624	-0.129	*WDR45L*	9.42E-10	-0.040
cg14746387	5	172879299	4.78E-115	0.323	0.418	-0.095	NA	1.30E-10	-0.035
cg10206397	20	2085344	4.94E-115	0.454	0.556	-0.102	*STK35*	9.97E-09	-0.034
cg03621504	12	116571240	5.71E-115	0.257	0.339	-0.082	*MED13L*	3.27E-05	-0.021
cg13270625	18	21452819	5.83E-115	0.817	0.759	0.057	*LAMA3*	3.54E-21	0.033
cg04070601	12	50067887	6.40E-115	0.757	0.694	0.063	*FMNL3*	8.73E-20	0.035
cg25084760	13	100886412	6.66E-115	0.383	0.486	-0.103	*PCCA*	5.69E-08	-0.030
cg17547928	4	737930	1.00E-114	0.360	0.428	-0.068	*PCGF3*	4.60E-05	-0.016
cg20875821	18	61557735	1.10E-114	0.359	0.453	-0.094	*SERPINB2*	1.90E-19	-0.047
cg23587176	7	4746837	1.17E-114	0.559	0.658	-0.099	*FOXK1*	1.73E-16	-0.043
cg19725377	14	69535979	1.23E-114	0.289	0.377	-0.088	*DCAF5*	3.59E-12	-0.036
cg03322353	4	141069770	1.26E-114	0.394	0.498	-0.104	*MAML3*	6.16E-12	-0.043
cg16967583	2	241807859	1.35E-114	0.267	0.353	-0.086	*AGXT*	2.32E-24	-0.051
cg09417547	3	43289436	1.36E-114	0.402	0.507	-0.104	NA	7.27E-11	-0.039
cg01404893	16	89043547	1.42E-114	0.285	0.361	-0.076	*CBFA2T3*	1.31E-20	-0.039
cg24159247	3	4575483	1.52E-114	0.210	0.277	-0.068	*ITPR1*	2.36E-09	-0.026
cg13084677	4	39482740	1.65E-114	0.336	0.444	-0.108	*LOC401127*	4.56E-10	-0.041
cg02035751	1	206729099	1.71E-114	0.752	0.679	0.072	*RASSF5*	3.99E-19	0.034
cg14187687	14	55249770	1.76E-114	0.403	0.517	-0.114	*SAMD4A*	2.59E-12	-0.045
cg14427668	7	105319437	1.81E-114	0.227	0.282	-0.056	*ATXN7L1*	1.96E-08	-0.019
cg23206115	6	158066900	2.12E-114	0.428	0.533	-0.105	*ZDHHC14*	1.10E-10	-0.039
cg15700582	2	68960656	2.34E-114	0.280	0.362	-0.082	*ARHGAP25*	4.69E-16	-0.042
cg01290568	2	113593859	2.48E-114	0.333	0.419	-0.086	*IL1B*	1.10E-16	-0.044
cg02579377	16	85047901	2.74E-114	0.300	0.394	-0.094	NA	2.91E-09	-0.031
cg22894805	15	41983773	3.01E-114	0.349	0.437	-0.087	*MGA*	1.90E-10	-0.033
cg22022716	8	26276541	3.36E-114	0.510	0.599	-0.089	NA	9.68E-13	-0.036
cg05209306	19	39899376	3.60E-114	0.762	0.681	0.082	*ZFP36*	1.18E-13	0.033
cg09455513	10	3792283	3.88E-114	0.279	0.363	-0.085	NA	1.47E-13	-0.036
cg04370174	4	8207250	3.91E-114	0.303	0.376	-0.073	*SH3TC1*	7.22E-18	-0.037
cg16378063	17	47329552	3.96E-114	0.379	0.482	-0.103	*FLJ40194*	7.86E-10	-0.036
cg13710553	7	129598625	4.04E-114	0.267	0.343	-0.076	NA	1.29E-19	-0.043
cg08165960	15	101777800	4.42E-114	0.246	0.320	-0.074	*CHSY1*	9.34E-12	-0.029
cg08183317	13	114261934	4.44E-114	0.254	0.331	-0.077	*TFDP1*	6.01E-08	-0.024
cg12353788	17	7792061	4.74E-114	0.736	0.659	0.077	*CHD3*	3.33E-09	0.027
cg17904988	1	161168451	4.81E-114	0.252	0.326	-0.073	*NDUFS2*	5.41E-16	-0.037
cg26780125	8	125388806	4.93E-114	0.285	0.377	-0.092	NA	1.91E-11	-0.039
cg16283183	3	45718805	5.01E-114	0.331	0.427	-0.096	*LIMD1*	4.56E-08	-0.030
cg15244101	12	49627624	5.14E-114	0.269	0.350	-0.081	NA	7.18E-11	-0.031
cg16463452	13	29195249	5.66E-114	0.385	0.485	-0.101	NA	6.11E-18	-0.052

cg17852326	1	221055964	5.68E-114	0.333	0.414	-0.081	*HLX*	8.03E-14	-0.035
cg25724895	20	58630390	6.20E-114	0.289	0.355	-0.066	*C20orf197*	2.88E-14	-0.028
cg27369423	16	56228901	6.65E-114	0.223	0.306	-0.082	*GNAO1*	1.17E-07	-0.027
cg15046489	17	48857353	7.35E-114	0.827	0.769	0.058	NA	4.09E-13	0.025
cg14378231	1	98404032	8.26E-114	0.486	0.577	-0.091	NA	1.12E-10	-0.034
cg01612883	12	125023115	9.55E-114	0.314	0.369	-0.055	*NCOR2*	8.48E-10	-0.019
cg18213545	11	57529400	9.85E-114	0.265	0.336	-0.071	*TMX2-CTNND1*	4.52E-11	-0.026
cg19446990	11	61739400	1.09E-113	0.341	0.418	-0.077	NA	2.01E-24	-0.045
cg04756491	14	23385384	1.26E-113	0.350	0.463	-0.112	*RBM23*	1.88E-06	-0.030
cg05162858	10	80697460	1.27E-113	0.267	0.366	-0.100	NA	3.82E-14	-0.048
cg01688936	16	30770794	1.52E-113	0.390	0.457	-0.067	*C16orf93*	4.21E-10	-0.023
cg09868035	20	62492074	1.52E-113	0.277	0.335	-0.058	*ABHD16B*	2.75E-14	-0.024
cg09010707	12	26392567	1.61E-113	0.322	0.387	-0.065	NA	3.56E-18	-0.033
cg26664457	17	47394423	1.64E-113	0.329	0.418	-0.089	*ZNF652*	3.60E-15	-0.043
cg14399183	9	124048308	1.72E-113	0.218	0.291	-0.073	*GSN*	6.63E-10	-0.027
cg19280540	19	16553711	1.87E-113	0.332	0.400	-0.068	*EPS15L1*	8.60E-07	-0.018
cg05180856	15	91428056	2.24E-113	0.364	0.428	-0.064	*FES*	3.47E-11	-0.023
cg09101941	5	133775258	2.30E-113	0.285	0.381	-0.096	NA	6.22E-18	-0.052
cg05463966	11	67803951	2.44E-113	0.259	0.335	-0.076	*NDUFS8*	1.48E-12	-0.032
cg02227879	10	75576192	2.46E-113	0.375	0.486	-0.111	*CAMK2G*	4.80E-09	-0.037
cg26999345	22	37584441	2.74E-113	0.310	0.377	-0.068	*C1QTNF6*	9.27E-12	-0.026
cg06640822	1	25291472	3.00E-113	0.723	0.653	0.070	*RUNX3*	3.43E-19	0.037
cg11316887	10	29924694	3.38E-113	0.813	0.746	0.067	*SVIL*	5.04E-11	0.026
cg16959758	20	36796513	3.39E-113	0.283	0.346	-0.063	NA	3.54E-14	-0.026
cg17296078	10	99259721	3.43E-113	0.256	0.330	-0.075	*MMS19*	2.72E-13	-0.033
cg01858712	16	12156525	3.63E-113	0.257	0.335	-0.078	*SNX29*	1.43E-13	-0.035
cg25711558	7	101499638	3.94E-113	0.298	0.398	-0.100	*CUX1*	2.75E-12	-0.040
cg00869668	17	1549012	4.09E-113	0.308	0.390	-0.082	*SCARF1*	1.72E-15	-0.036
cg05971678	10	125770089	4.23E-113	0.398	0.508	-0.111	*CHST15*	1.04E-05	-0.030
cg03382501	16	11794641	4.38E-113	0.225	0.306	-0.081	*TXNDC11*	5.56E-11	-0.032
cg21442998	3	11597936	4.63E-113	0.363	0.441	-0.078	*VGLL4*	1.62E-09	-0.027
cg06834507	1	200876957	4.71E-113	0.246	0.308	-0.062	*C1orf106*	1.82E-23	-0.036
cg20090162	2	47261900	4.86E-113	0.356	0.442	-0.086	*TTC7A*	2.75E-06	-0.022
cg04042333	17	1104665	5.84E-113	0.768	0.675	0.094	NA	5.47E-11	0.040
cg05768044	16	53243559	6.57E-113	0.345	0.438	-0.093	*CHD9*	9.71E-17	-0.048
cg07561747	1	200983313	6.72E-113	0.386	0.469	-0.083	*KIF21B*	9.26E-06	-0.020
cg18665334	2	231735434	6.75E-113	0.290	0.362	-0.072	*ITM2C*	6.85E-23	-0.041
cg24496021	11	72662461	7.65E-113	0.398	0.504	-0.107	*FCHSD2*	2.36E-07	-0.030
cg11683242	1	32716557	7.85E-113	0.838	0.779	0.059	*LCK*	1.54E-15	0.029
cg11792874	14	24014501	8.12E-113	0.326	0.386	-0.061	*THTPA*	3.85E-08	-0.018
cg07530172	11	95431373	8.53E-113	0.258	0.341	-0.083	NA	1.46E-16	-0.043
cg24481882	6	136450701	9.08E-113	0.386	0.472	-0.086	*PDE7B*	1.03E-13	-0.037
cg26792900	1	167074894	9.15E-113	0.257	0.335	-0.078	*DUSP27*	4.06E-21	-0.044
cg14049990	5	1514140	9.95E-113	0.317	0.389	-0.072	*LPCAT1*	4.41E-11	-0.028
cg21536783	1	59041407	1.17E-112	0.368	0.444	-0.076	*TACSTD2*	1.48E-07	-0.025
cg06468347	17	3705875	1.17E-112	0.281	0.371	-0.090	*ITGAE*	9.41E-09	-0.033
cg11123644	2	219597614	1.19E-112	0.260	0.346	-0.086	*TTLL4*	8.21E-12	-0.035
cg20618651	4	56718365	1.19E-112	0.766	0.692	0.074	*EXOC1*	2.04E-22	0.046
cg19383974	1	33463643	1.20E-112	0.286	0.350	-0.063	NA	1.78E-08	-0.020
cg01877450	7	97915802	1.26E-112	0.362	0.428	-0.065	*BRI3*	1.27E-08	-0.019
cg17568962	6	35456312	1.28E-112	0.215	0.269	-0.054	*TEAD3*	4.10E-15	-0.026
cg01700462	20	45179230	1.30E-112	0.291	0.388	-0.096	*OCSTAMP*	3.12E-12	-0.041
cg06654079	16	21169179	1.33E-112	0.362	0.463	-0.101	*TMEM159*	9.50E-09	-0.036
cg17825194	12	124864681	1.41E-112	0.305	0.375	-0.069	*NCOR2*	6.73E-11	-0.024
cg15167202	5	149997153	1.48E-112	0.434	0.523	-0.089	*SYNPO*	4.42E-12	-0.035
cg06005892	11	3177622	1.62E-112	0.339	0.420	-0.080	*OSBPL5*	2.00E-10	-0.029
cg11180667	10	101415865	1.67E-112	0.308	0.406	-0.098	NA	1.63E-11	-0.039
cg22084410	3	51987688	1.82E-112	0.317	0.403	-0.087	NA	3.31E-12	-0.037
cg08418670	19	4374567	1.89E-112	0.460	0.555	-0.095	*SH3GL1*	1.48E-05	-0.021
cg01448863	14	70264043	1.94E-112	0.302	0.405	-0.102	*SLC10A1*	4.47E-11	-0.042
cg18991321	7	100144475	1.96E-112	0.835	0.776	0.059	*AGFG2*	5.47E-05	0.015
cg03919657	17	48502312	2.05E-112	0.361	0.425	-0.064	*ACSF2*	8.03E-16	-0.026
cg21964466	5	39526937	2.36E-112	0.298	0.383	-0.085	NA	5.26E-24	-0.054
cg14081270	11	118286105	2.46E-112	0.226	0.300	-0.074	*LOC10013162*	9.75E-13	-0.033
cg22684041	19	1904554	2.57E-112	0.222	0.285	-0.063	*SCAMP4*	8.36E-13	-0.026
cg08837215	17	74443080	2.65E-112	0.462	0.556	-0.094	*UBE2O*	5.19E-09	-0.035
cg25006077	3	152176018	2.69E-112	0.488	0.580	-0.092	*MBNL1*	1.32E-04	-0.020
cg06823060	16	81616874	2.91E-112	0.503	0.597	-0.095	*CMIP*	1.32E-07	-0.028
cg18325192	8	19540396	3.45E-112	0.292	0.353	-0.061	*CSGALNACT1*	2.43E-09	-0.020
cg07188523	6	44528793	3.62E-112	0.422	0.511	-0.089	NA	6.96E-14	-0.042
cg27531366	1	27627058	3.79E-112	0.282	0.362	-0.080	*WDTC1*	2.45E-21	-0.044
cg13345299	22	26876075	3.87E-112	0.226	0.301	-0.075	*HPS4*	4.80E-19	-0.040
cg07834069	17	72524918	4.25E-112	0.316	0.418	-0.102	*CD300LB*	5.50E-15	-0.045

cg16139316	1	153330758	4.56E-112	0.302	0.394	-0.092	*S100A9*	3.39E-10	-0.036
cg06659338	1	54562040	4.59E-112	0.367	0.441	-0.074	*TCEANC2*	3.11E-14	-0.032
cg21511321	7	1004997	4.73E-112	0.300	0.371	-0.071	*COX19*	1.10E-12	-0.030
cg00122406	8	26431736	4.97E-112	0.193	0.250	-0.057	*DPYSL2*	3.18E-14	-0.025
cg15942979	8	22497061	5.22E-112	0.705	0.636	0.069	*BIN3*	4.91E-09	0.025
cg18437792	14	31699236	5.77E-112	0.310	0.401	-0.091	NA	1.90E-08	-0.032
cg19867914	2	144234430	5.79E-112	0.331	0.415	-0.084	*ARHGAP15*	1.52E-10	-0.033
cg23334433	17	79129051	6.40E-112	0.377	0.501	-0.124	*AATK*	5.33E-08	-0.038
cg02655351	1	3498101	6.40E-112	0.273	0.354	-0.081	*MEGF6*	7.30E-17	-0.042
cg14138540	15	93571988	7.08E-112	0.221	0.288	-0.067	NA	7.00E-09	-0.023
cg17742559	17	27068495	7.62E-112	0.298	0.365	-0.067	*NEK8*	2.53E-11	-0.026
cg12656896	6	8438818	7.74E-112	0.285	0.374	-0.089	*LOC10050620*	2.58E-09	-0.033
cg15078958	1	10997656	7.88E-112	0.722	0.656	0.066	NA	6.45E-11	0.026
cg01360627	6	31544931	9.85E-112	0.762	0.675	0.087	*TNF*	1.91E-06	0.024
cg06684088	7	119131	1.11E-111	0.312	0.422	-0.110	NA	2.85E-07	-0.034
cg07006935	7	55620641	1.16E-111	0.288	0.383	-0.095	*VOPP1*	6.60E-06	-0.026
cg13079571	6	30297257	1.16E-111	0.447	0.548	-0.101	*TRIM39*	5.63E-06	-0.026
cg01190666	20	62204908	1.20E-111	0.443	0.545	-0.102	*PRIC285*	8.81E-41	-0.068
cg02348449	19	58630429	1.39E-111	0.360	0.426	-0.066	*ZSCAN18*	1.89E-12	-0.025
cg04468671	12	132337407	1.39E-111	0.416	0.481	-0.065	NA	1.35E-12	-0.027
cg07409471	17	80833638	1.56E-111	0.483	0.589	-0.106	*TBCD*	2.06E-11	-0.039
cg11377047	1	26881009	1.59E-111	0.286	0.384	-0.099	*RPS6KA1*	1.81E-19	-0.055
cg01850135	16	4516078	1.64E-111	0.758	0.679	0.080	*NMRAL1*	3.49E-05	0.017
cg27479162	10	98450737	1.78E-111	0.322	0.403	-0.082	*PIK3AP1*	7.29E-22	-0.043
cg10408731	7	65214843	1.79E-111	0.419	0.530	-0.111	*LOC441242*	0.003	-0.019
cg24973755	4	1304972	2.07E-111	0.807	0.724	0.084	*MAEA*	1.07E-13	0.036
cg23240927	11	1320497	2.12E-111	0.399	0.514	-0.115	*TOLLIP*	1.17E-09	-0.041
cg02098075	8	49643972	2.26E-111	0.368	0.463	-0.095	*EFCAB1*	1.45E-06	-0.026
cg19342782	1	70821806	3.37E-111	0.378	0.502	-0.124	*HHLA3*	1.13E-13	-0.054
cg14023350	14	89631066	3.42E-111	0.326	0.396	-0.071	*FOXN3*	5.03E-16	-0.034
cg21359303	10	106068573	3.71E-111	0.830	0.770	0.060	NA	5.43E-08	0.020
cg00501919	6	31540750	3.78E-111	0.649	0.578	0.071	*LTA*	2.67E-07	0.021
cg21244086	15	45479462	3.78E-111	0.338	0.405	-0.067	*SHF*	9.68E-13	-0.026
cg04554122	3	137938348	4.05E-111	0.333	0.418	-0.086	*ARMC8*	4.39E-12	-0.037
cg01023672	12	47477223	4.18E-111	0.349	0.454	-0.105	NA	3.06E-07	-0.033
cg13057663	8	37805275	4.48E-111	0.434	0.547	-0.113	NA	1.51E-05	-0.028
cg00675600	5	1255458	4.55E-111	0.803	0.746	0.057	*TERT*	2.37E-23	0.033
cg01351315	17	46667737	5.71E-111	0.377	0.485	-0.108	NA	9.12E-12	-0.040
cg19268453	6	32905114	6.52E-111	0.409	0.495	-0.086	*HLA-DMB*	2.04E-11	-0.032
cg19982668	17	61753497	6.83E-111	0.304	0.378	-0.074	*MAP3K3*	1.26E-16	-0.038
cg17446956	12	109235032	6.96E-111	0.455	0.543	-0.088	*SSH1*	5.91E-06	-0.024
cg10346364	11	67142268	7.03E-111	0.285	0.354	-0.069	*CLCF1*	9.91E-10	-0.024
cg03370752	11	61136373	7.07E-111	0.503	0.594	-0.091	*TMEM138*	1.36E-05	-0.022
cg11775521	11	74178795	8.54E-111	0.235	0.312	-0.077	*KCNE3*	1.65E-12	-0.032
cg21320567	8	67975880	8.60E-111	0.397	0.503	-0.107	*CSPP1*	9.75E-11	-0.040
cg06617636	14	69256690	8.98E-111	0.830	0.770	0.061	*ZFP36L1*	2.76E-11	0.026
cg16604566	18	74845829	9.18E-111	0.296	0.390	-0.094	*MBP*	5.39E-15	-0.046
cg13383814	10	98745012	9.34E-111	0.174	0.231	-0.058	*C10orf12*	1.15E-08	-0.021
cg03169059	2	240145022	9.35E-111	0.319	0.411	-0.091	*HDAC4*	3.31E-10	-0.035
**cg13429095**	1	206913187	9.41E-111	0.639	0.556	0.083	NA	0.122	0.007
cg03720100	6	30720263	1.20E-110	0.263	0.349	-0.085	NA	4.44E-16	-0.043
cg11967765	7	2774195	1.48E-110	0.280	0.352	-0.072	*GNA12*	1.18E-07	-0.023
cg14021478	6	33384537	1.59E-110	0.652	0.580	0.072	*CUTA*	5.61E-05	0.016
cg07905808	6	30297389	1.60E-110	0.505	0.585	-0.080	*TRIM39*	1.68E-06	-0.022
cg26562921	16	84760429	1.80E-110	0.273	0.350	-0.077	*USP10*	3.92E-08	-0.024
cg04093633	15	82469848	1.80E-110	0.372	0.454	-0.082	*EFTUD1*	2.89E-16	-0.042
cg12678686	3	127327369	2.01E-110	0.338	0.431	-0.092	*MCM2*	1.98E-07	-0.030
cg00444740	8	129162178	2.71E-110	0.319	0.411	-0.092	NA	2.30E-10	-0.035
cg25166437	17	1549284	2.76E-110	0.219	0.281	-0.063	*SCARF1*	1.74E-16	-0.031
cg02399831	3	112113161	2.77E-110	0.372	0.473	-0.101	NA	1.03E-05	-0.025
cg07231045	8	142219444	2.81E-110	0.414	0.523	-0.109	NA	1.32E-11	-0.045
cg09418321	12	4699618	2.91E-110	0.333	0.413	-0.080	*DYRK4*	4.80E-13	-0.037
cg10959651	2	7018020	2.92E-110	0.157	0.268	-0.110	*RSAD2*	1.34E-34	-0.075
cg12380764	1	206971195	2.94E-110	0.311	0.408	-0.097	*IL19*	1.55E-12	-0.041
cg15739581	2	166626783	2.99E-110	0.394	0.479	-0.085	*GALNT3*	5.12E-08	-0.028
cg06740950	3	171878318	3.00E-110	0.335	0.435	-0.100	*FNDC3B*	2.34E-13	-0.047
cg09993145	1	25291905	3.05E-110	0.552	0.445	0.107	*RUNX3*	6.28E-10	0.036
cg12002745	8	124179875	3.12E-110	0.240	0.307	-0.067	NA	3.21E-22	-0.037
cg17419815	12	27234924	3.26E-110	0.480	0.593	-0.113	*C12orf71*	1.17E-09	-0.038
cg13819552	9	95799870	3.28E-110	0.332	0.398	-0.065	NA	5.59E-15	-0.028
cg06143615	3	13462903	3.41E-110	0.647	0.587	0.060	*NUP210*	7.78E-21	0.031
cg19190900	2	37553621	3.43E-110	0.262	0.339	-0.077	NA	1.64E-17	-0.042
cg14663914	19	827739	3.44E-110	0.319	0.397	-0.078	*AZU1*	6.05E-15	-0.035

cg15610437	19	827821	3.53E-110	0.416	0.487	-0.070	AZU1	1.08E-10	-0.023
cg00333528	6	89927485	4.02E-110	0.269	0.340	-0.072	GABRR1	6.55E-21	-0.040
cg06053959	17	1617291	4.27E-110	0.330	0.407	-0.077	MIR22HG	2.33E-28	-0.046
cg26207239	11	102231936	4.40E-110	0.720	0.661	0.058	BIRC2	1.51E-19	0.032
cg26414257	4	6888038	4.54E-110	0.245	0.307	-0.062	NA	1.01E-15	-0.029
cg16149628	11	1792574	4.63E-110	0.510	0.612	-0.102	NA	4.54E-04	-0.018
cg21988813	11	61730880	4.66E-110	0.231	0.309	-0.078	BEST1	2.11E-15	-0.038
cg00967073	1	244443687	4.66E-110	0.230	0.311	-0.080	NA	4.30E-19	-0.048
cg14752089	8	128773042	4.81E-110	0.237	0.311	-0.074	NA	3.83E-16	-0.035
cg16498391	18	12421072	4.89E-110	0.295	0.368	-0.072	SLMO1	1.17E-09	-0.025
cg00705730	2	106438120	5.12E-110	0.477	0.588	-0.110	NCK2	2.57E-04	-0.024
cg04525852	4	125632538	5.29E-110	0.234	0.322	-0.088	ANKRD50	6.72E-15	-0.044
cg17745697	2	38138126	5.37E-110	0.282	0.367	-0.085	NA	6.75E-06	-0.024
cg03418002	4	140655478	5.68E-110	0.373	0.479	-0.105	MGST2	4.70E-08	-0.032
cg21163717	8	21769903	5.72E-110	0.694	0.603	0.090	DOK2	6.73E-04	0.016
cg22331200	17	56355362	5.73E-110	0.474	0.569	-0.095	MPO	4.88E-13	-0.036
cg20186396	17	80275498	5.79E-110	0.688	0.629	0.059	CD7	1.06E-13	0.025
cg03340036	4	89446409	6.87E-110	0.448	0.560	-0.112	PIGY	1.94E-04	-0.022
cg11642298	3	133193741	6.90E-110	0.372	0.460	-0.089	BFSP2	1.18E-17	-0.050
cg08305575	5	158018481	7.34E-110	0.348	0.438	-0.090	NA	1.08E-15	-0.046
cg12684668	5	150403466	7.36E-110	0.444	0.530	-0.086	GPX3	9.26E-11	-0.034
cg04466886	16	66521191	8.24E-110	0.336	0.392	-0.057	BEAN1	1.69E-18	-0.030
cg23597162	7	28102341	8.78E-110	0.425	0.514	-0.090	JAZF1	3.30E-10	-0.033
cg19636224	1	44176844	9.06E-110	0.428	0.520	-0.092	ST3GAL3	3.39E-08	-0.029
cg19460836	17	79047872	9.35E-110	0.437	0.552	-0.115	BAIAP2	7.76E-04	-0.021
cg20740903	17	76170799	9.52E-110	0.398	0.493	-0.094	TK1	1.93E-10	-0.032
cg00660167	17	80200974	9.80E-110	0.387	0.502	-0.115	CSNK1D	1.20E-06	-0.034
cg17617491	19	2607726	1.03E-109	0.781	0.719	0.061	GNG7	1.90E-17	0.033
cg15945999	11	65355408	1.10E-109	0.695	0.634	0.062	EHBP1L1	4.87E-05	0.013
cg12554857	4	95264019	1.23E-109	0.256	0.332	-0.077	HPGDS	8.78E-06	-0.021
cg10785340	5	134715577	1.24E-109	0.246	0.329	-0.082	H2AFY	8.45E-09	-0.030
cg01723892	1	233004866	1.28E-109	0.278	0.370	-0.092	NA	3.39E-13	-0.042
cg23590660	12	8205602	1.35E-109	0.241	0.323	-0.082	FOXJ2	7.26E-13	-0.038
cg16041412	19	2460037	1.41E-109	0.396	0.512	-0.116	NA	7.07E-05	-0.029
cg08843850	1	199092823	1.59E-109	0.322	0.412	-0.090	NA	4.87E-04	-0.019
cg06407843	6	43279607	1.60E-109	0.352	0.455	-0.103	NA	2.70E-08	-0.034
cg01861537	11	121507724	1.65E-109	0.380	0.485	-0.105	NA	1.53E-05	-0.028
cg05007997	2	219246985	1.65E-109	0.291	0.364	-0.073	SLC11A1	1.45E-10	-0.028
cg02889774	8	21824555	1.72E-109	0.443	0.527	-0.084	XPO7	1.02E-14	-0.038
cg18278694	1	179945973	1.90E-109	0.346	0.428	-0.082	CEP350	1.71E-11	-0.035
cg16541275	17	78821754	1.98E-109	0.642	0.568	0.074	RPTOR	1.79E-17	0.036
cg09704136	7	143082002	2.10E-109	0.293	0.384	-0.091	ZYX	7.43E-23	-0.052
cg15297628	11	69240844	2.26E-109	0.705	0.627	0.078	NA	1.67E-18	0.040
cg00009088	11	60930188	2.38E-109	0.407	0.520	-0.113	VPS37C	1.24E-07	-0.035
cg09146459	6	32921200	2.56E-109	0.250	0.335	-0.085	HLA-DMA	3.73E-12	-0.034
cg18011672	2	190522601	3.01E-109	0.727	0.644	0.082	NA	6.69E-12	0.037
cg04084354	1	56721795	3.09E-109	0.258	0.343	-0.086	NA	5.36E-23	-0.055
cg13679804	3	59734950	3.11E-109	0.325	0.427	-0.103	NA	7.57E-15	-0.051
cg16261114	15	99551997	3.25E-109	0.333	0.425	-0.092	PGPEP1L	1.47E-16	-0.049
cg21826784	1	11795937	3.48E-109	0.239	0.313	-0.074	AGTRAP	3.31E-11	-0.029
cg06208288	1	58858074	3.61E-109	0.739	0.644	0.095	NA	1.21E-11	0.036
cg18637761	12	123468780	3.64E-109	0.185	0.240	-0.055	PITPNM2	1.03E-18	-0.029
cg06465011	16	84860871	4.03E-109	0.366	0.469	-0.103	CRISPLD2	5.96E-12	-0.039
cg10126923	19	51875451	4.11E-109	0.290	0.365	-0.076	NKG7	6.55E-11	-0.030
cg11236515	2	74213762	4.39E-109	0.815	0.753	0.062	NA	7.57E-09	0.021
cg09205945	7	137662133	4.86E-109	0.356	0.460	-0.104	CREB3L2	5.62E-11	-0.039
cg14693090	12	11899281	4.90E-109	0.276	0.363	-0.087	ETV6	7.12E-07	-0.027
cg13424393	17	80569923	5.03E-109	0.403	0.506	-0.103	NA	1.77E-08	-0.033
cg25948982	20	45179157	5.34E-109	0.176	0.232	-0.056	OCSTAMP	2.80E-09	-0.021
cg13852284	10	77548353	5.35E-109	0.431	0.522	-0.092	C10orf11	1.00E-09	-0.034
cg18568335	14	105792505	5.38E-109	0.376	0.484	-0.108	PACS2	6.49E-14	-0.052
cg24484138	20	31070190	5.63E-109	0.328	0.427	-0.100	C20orf112	5.33E-06	-0.029
cg20118717	6	33400505	5.74E-109	0.366	0.457	-0.091	SYNGAP1	2.94E-10	-0.033
cg06068369	6	105837504	6.33E-109	0.416	0.495	-0.079	PREP	7.72E-06	-0.022
cg15527643	1	11795946	6.59E-109	0.300	0.366	-0.067	AGTRAP	1.18E-17	-0.032
cg05323324	3	194120023	8.47E-109	0.257	0.332	-0.075	GP5	2.47E-17	-0.041
cg20227511	13	28670602	8.66E-109	0.426	0.526	-0.100	FLT3	1.18E-06	-0.028
cg01544270	9	140300227	1.00E-108	0.282	0.351	-0.069	EXD3	2.86E-11	-0.027
cg17206393	20	33681223	1.04E-108	0.275	0.375	-0.100	TRPC4AP	1.08E-11	-0.043
cg23575688	11	119486443	1.14E-108	0.258	0.336	-0.078	NA	8.76E-09	-0.027
cg23189692	3	184050393	1.16E-108	0.297	0.381	-0.084	EIF4G1	5.29E-08	-0.029
cg25811633	3	71626654	1.32E-108	0.288	0.376	-0.088	FOXP1	3.62E-15	-0.041
cg21805118	12	122712153	1.43E-108	0.765	0.698	0.067	DIABLO	9.38E-11	0.027

cg19157819	10	75405260	1.52E-108	0.278	0.367	-0.089	*SYNPO2L*	2.37E-09	-0.034
cg20477259	6	31544960	1.60E-108	0.752	0.666	0.086	*TNF*	4.55E-04	0.018
cg20133901	3	51602121	1.73E-108	0.239	0.305	-0.066	*RAD54L2*	1.26E-12	-0.029
cg09166556	1	156724277	1.82E-108	0.552	0.665	-0.113	NA	0.047	-0.012
cg25420477	2	70319121	1.95E-108	0.765	0.690	0.074	NA	2.72E-11	0.030
cg05714729	18	33234096	2.08E-108	0.334	0.423	-0.089	*GALNT1*	1.61E-16	-0.046
cg19510565	1	31217240	2.52E-108	0.713	0.645	0.068	*LAPTM5*	9.74E-04	0.014
cg16350446	2	172338747	2.53E-108	0.314	0.403	-0.090	*DCAF17*	2.85E-06	-0.026
cg14115756	9	125795935	2.95E-108	0.283	0.371	-0.088	*GPR21*	7.78E-10	-0.035
cg12617080	1	156509844	2.95E-108	0.306	0.362	-0.055	*IQGAP3*	1.40E-28	-0.036
cg26842080	5	42922968	3.02E-108	0.402	0.498	-0.096	NA	2.07E-09	-0.034
cg14184693	15	44085947	3.15E-108	0.373	0.432	-0.060	*SERF2*	2.09E-15	-0.030
cg09706586	1	9775838	3.26E-108	0.741	0.675	0.065	*PIK3CD*	2.47E-20	0.037
cg20253872	1	110166571	3.32E-108	0.326	0.385	-0.059	*AMPD2*	5.35E-13	-0.025
cg17161520	11	67174843	3.91E-108	0.758	0.679	0.079	*TBC1D10C*	7.03E-06	0.024
cg20121012	12	93516072	4.58E-108	0.490	0.570	-0.080	*LOC643339*	9.56E-08	-0.024
cg16768966	17	9940227	4.82E-108	0.370	0.463	-0.093	*GAS7*	1.24E-12	-0.040
cg15587792	11	75584802	4.97E-108	0.234	0.302	-0.069	*UVRAG*	2.22E-13	-0.034
cg12586150	6	2840792	5.19E-108	0.248	0.330	-0.082	*SERPINB1*	8.91E-10	-0.033
cg20107506	4	100874826	5.44E-108	0.396	0.508	-0.111	NA	1.15E-07	-0.032
cg08130912	14	64687388	5.45E-108	0.235	0.310	-0.076	*SYNE2*	1.44E-04	-0.017
cg08642285	15	68498589	5.48E-108	0.180	0.236	-0.056	*CALML4*	8.21E-08	-0.018
cg23510807	7	2567893	5.51E-108	0.702	0.649	0.052	*LFNG*	2.59E-08	0.018
cg15373592	6	56405167	5.83E-108	0.296	0.381	-0.085	*RNU6-71*	1.53E-26	-0.057
cg14413165	9	130524573	6.04E-108	0.297	0.348	-0.051	*SH2D3C*	1.62E-09	-0.018
cg25429672	11	73692155	6.04E-108	0.786	0.714	0.071	*UCP2*	3.42E-10	0.029
cg15964132	11	3175636	6.50E-108	0.403	0.495	-0.092	*OSBPL5*	3.31E-07	-0.027
cg00423035	7	105319486	7.12E-108	0.319	0.405	-0.086	*ATXN7L1*	7.61E-11	-0.033
cg26108120	3	72440793	7.60E-108	0.295	0.382	-0.087	*RYBP*	7.66E-20	-0.048
cg08032476	17	27045176	7.78E-108	0.288	0.354	-0.066	*RAB34*	9.84E-09	-0.023
cg14405137	12	50219283	8.37E-108	0.252	0.329	-0.077	*NCKAP5L*	3.45E-14	-0.037
cg04605681	4	8229159	8.40E-108	0.266	0.337	-0.071	*SH3TC1*	3.71E-08	-0.023
cg05880248	10	48432664	8.78E-108	0.257	0.315	-0.058	*GDF10*	1.38E-13	-0.027
cg21611170	5	114938389	9.86E-108	0.277	0.340	-0.062	*TMED7-TICAM*	1.34E-15	-0.031
cg06072036	11	822402	1.13E-107	0.334	0.426	-0.092	*PNPLA2*	2.06E-10	-0.033
cg04100124	10	69611755	1.37E-107	0.365	0.460	-0.095	NA	2.59E-07	-0.032
cg06208158	16	29168730	1.66E-107	0.305	0.372	-0.067	NA	1.97E-11	-0.027
cg09535475	2	42444827	1.97E-107	0.405	0.510	-0.105	*EML4*	8.12E-07	-0.031
cg14245199	14	100540291	2.01E-107	0.786	0.725	0.062	*EVL*	4.03E-12	0.028
cg15293582	10	72362866	2.07E-107	0.756	0.690	0.066	*PRF1*	1.52E-06	0.021
cg25539505	5	95634103	2.08E-107	0.446	0.543	-0.097	NA	4.15E-06	-0.027
cg16603916	1	21615863	2.11E-107	0.348	0.422	-0.074	*ECE1*	3.49E-07	-0.022
cg00901982	2	70257298	2.16E-107	0.303	0.404	-0.101	*PCBP1-AS1*	2.29E-16	-0.047
cg16846069	12	6462681	2.20E-107	0.351	0.432	-0.081	*SCNN1A*	3.01E-11	-0.031
cg10800346	13	114829496	2.24E-107	0.327	0.434	-0.107	*RASA3*	2.19E-09	-0.040
cg12890903	14	24867954	2.43E-107	0.256	0.321	-0.065	*NYNRIN*	5.41E-14	-0.031
cg26633373	19	54377836	2.75E-107	0.344	0.425	-0.081	*MYADM*	1.69E-06	-0.022
cg26794830	19	3193804	2.76E-107	0.256	0.323	-0.067	*NCLN*	2.87E-14	-0.032
cg10094994	2	240162323	2.83E-107	0.280	0.370	-0.090	*HDAC4*	2.36E-08	-0.034
cg02726291	3	128779596	3.37E-107	0.302	0.383	-0.082	*GP9*	3.34E-09	-0.030
cg17369406	21	34918578	3.50E-107	0.299	0.380	-0.081	*SON*	5.69E-04	-0.017
cg21473786	2	44311918	4.16E-107	0.435	0.518	-0.083	NA	7.26E-12	-0.036
cg09800500	12	24992256	4.63E-107	0.828	0.762	0.066	*BCAT1*	7.62E-04	0.014
cg13355542	2	97199248	4.83E-107	0.252	0.323	-0.071	NA	3.04E-13	-0.032
cg02769705	1	202975059	5.07E-107	0.385	0.487	-0.102	*LOC401980*	1.16E-10	-0.038
cg04960169	17	2839005	5.34E-107	0.320	0.403	-0.083	*RAP1GAP2*	2.45E-09	-0.032
cg12481212	6	44020245	5.61E-107	0.311	0.390	-0.078	NA	9.57E-14	-0.034
cg24926276	19	4539943	5.75E-107	0.443	0.525	-0.082	*LRG1*	6.83E-10	-0.028
cg03055520	11	121460793	5.77E-107	0.230	0.288	-0.058	*SORL1*	1.88E-18	-0.033
cg02555923	3	14319984	5.81E-107	0.240	0.297	-0.057	NA	3.05E-13	-0.026
cg26696162	6	151709522	6.17E-107	0.261	0.343	-0.082	*ZBTB2*	8.55E-11	-0.033
cg17591816	1	172239092	6.40E-107	0.428	0.526	-0.098	*DNM3*	1.26E-10	-0.036
cg18854765	1	74665362	6.72E-107	0.301	0.389	-0.088	*FPGT*	3.74E-06	-0.025
cg05464506	2	420052	6.75E-107	0.750	0.689	0.061	NA	3.55E-13	0.027
cg23682913	1	2080710	7.02E-107	0.226	0.291	-0.064	*PRKCZ*	4.82E-09	-0.023
cg05697976	12	29376483	7.17E-107	0.305	0.404	-0.099	*FAR2*	8.35E-10	-0.037
cg25068347	11	128343784	8.78E-107	0.828	0.770	0.058	*ETS1*	4.44E-06	0.017
cg12413156	20	62368256	9.42E-107	0.659	0.567	0.092	*LIME1*	6.61E-10	0.032
cg23819411	3	182929273	9.43E-107	0.312	0.404	-0.093	*MCF2L2*	0.035	-0.012
cg24593372	1	54562121	1.01E-106	0.323	0.386	-0.063	*TCEANC2*	1.55E-16	-0.031
cg18101140	11	67142001	1.07E-106	0.206	0.278	-0.073	*CLCF1*	1.96E-17	-0.039
cg26605164	10	102821565	1.08E-106	0.404	0.501	-0.098	*KAZALD1*	2.65E-09	-0.033
cg04087207	20	31669392	1.11E-106	0.321	0.415	-0.094	*BPIFB4*	9.46E-13	-0.041

cg23939096	5	1555791	1.16E-106	0.324	0.429	-0.105	NA	2.81E-08	-0.037
cg16899991	3	127868625	1.28E-106	0.341	0.441	-0.100	NA	5.34E-13	-0.040
cg08784966	6	37248768	1.32E-106	0.474	0.535	-0.060	*TBC1D22B*	3.08E-11	-0.023
cg18897025	5	110413671	1.41E-106	0.414	0.516	-0.102	*TSLP*	4.92E-09	-0.034
cg24635112	13	114908901	1.46E-106	0.822	0.765	0.057	NA	9.82E-06	0.016
cg23825057	12	124014409	1.55E-106	0.405	0.491	-0.086	*RILPL1*	1.32E-06	-0.026
cg00945209	17	76801579	1.58E-106	0.682	0.609	0.073	*USP36*	6.27E-06	0.018
cg26290716	14	91862813	1.67E-106	0.337	0.411	-0.074	*CCDC88C*	2.00E-09	-0.026
cg25031824	17	3819363	1.68E-106	0.413	0.511	-0.098	*P2RX1*	4.31E-17	-0.044
cg16393899	6	5136301	1.79E-106	0.393	0.495	-0.102	*LYRM4*	1.16E-08	-0.037
cg17100176	1	207096358	1.84E-106	0.676	0.597	0.079	*FAIM3*	6.23E-10	0.028
cg03446062	6	14477353	2.00E-106	0.389	0.479	-0.091	NA	1.01E-14	-0.043
cg12573289	7	45075791	2.02E-106	0.528	0.469	0.058	*CCM2*	1.52E-08	0.018
cg15418499	11	112028288	2.05E-106	0.360	0.453	-0.093	*IL18*	1.87E-17	-0.051
cg12209946	3	14987434	2.06E-106	0.422	0.523	-0.101	*FGD5-AS1*	2.18E-08	-0.035
cg14144728	1	1101462	2.15E-106	0.739	0.684	0.055	NA	4.29E-06	0.015
cg18548864	3	112995278	2.16E-106	0.279	0.353	-0.074	*BOC*	4.37E-12	-0.029
cg25007705	1	21588799	2.17E-106	0.264	0.342	-0.078	*ECE1*	1.88E-08	-0.027
cg27112972	17	74378252	2.21E-106	0.244	0.306	-0.063	NA	3.56E-04	-0.014
cg20748065	7	75583421	2.37E-106	0.246	0.315	-0.069	*POR*	7.75E-10	-0.027
cg11554650	6	30653191	2.68E-106	0.678	0.619	0.059	*PPP1R18*	8.62E-08	0.018
cg20357538	15	101777761	3.11E-106	0.219	0.287	-0.068	*CHSY1*	4.31E-08	-0.023
cg04330326	19	2770448	3.17E-106	0.294	0.355	-0.061	*SGTA*	2.11E-10	-0.022
cg14209730	2	64632636	3.27E-106	0.532	0.637	-0.105	NA	2.67E-17	-0.048
cg13186559	10	80854035	3.90E-106	0.313	0.378	-0.066	*ZMIZ1*	1.77E-11	-0.027
cg19635401	6	118873071	4.13E-106	0.405	0.509	-0.104	*CEP85L*	0.002	-0.019
cg17928895	20	57583091	4.20E-106	0.261	0.342	-0.080	*CTSZ*	1.13E-13	-0.039
cg02527527	22	38303310	4.35E-106	0.295	0.372	-0.077	*MICALL1*	2.66E-13	-0.033
cg13474450	8	142222685	4.51E-106	0.453	0.557	-0.104	*SLC45A4*	2.11E-05	-0.027
cg16280132	6	31540459	4.79E-106	0.613	0.525	0.089	*LTA*	2.65E-11	0.034
cg11239575	17	3705765	5.35E-106	0.478	0.577	-0.100	*ITGAE*	2.56E-07	-0.029
cg08700083	4	48382815	5.51E-106	0.319	0.408	-0.089	*SLAIN2*	6.05E-08	-0.030
cg02021919	5	86707074	5.94E-106	0.439	0.541	-0.102	*CCNH*	2.33E-04	-0.023
cg27641961	16	75097037	6.08E-106	0.282	0.361	-0.079	*ZNRF1*	8.07E-12	-0.035
cg20430631	11	76340765	6.31E-106	0.305	0.405	-0.099	NA	6.28E-14	-0.046
cg06173720	7	127670993	6.70E-106	0.248	0.317	-0.069	*LRRC4*	3.36E-09	-0.027
cg27050612	17	46133198	7.49E-106	0.255	0.312	-0.058	*NFE2L1*	3.27E-16	-0.027
cg20704555	7	28127748	7.55E-106	0.407	0.506	-0.099	*JAZF1*	1.13E-08	-0.034
cg09342997	7	129339065	8.57E-106	0.419	0.525	-0.107	*NRF1*	0.004	-0.018
cg17434008	1	38974111	8.66E-106	0.295	0.381	-0.087	NA	4.96E-12	-0.038
cg10659811	12	54833402	1.07E-105	0.418	0.494	-0.076	NA	4.53E-22	-0.048
cg09122593	1	203020141	1.08E-105	0.262	0.319	-0.057	*PPFIA4*	3.63E-26	-0.037
cg14112601	6	10520874	1.11E-105	0.217	0.280	-0.063	*GCNT2*	1.91E-15	-0.034
cg16732654	17	79129022	1.34E-105	0.351	0.454	-0.104	*AATK*	5.52E-14	-0.049
cg22930808	3	122281881	1.39E-105	0.435	0.706	-0.271	*PARP9*	2.40E-55	-0.190
cg23338668	8	74240259	1.43E-105	0.466	0.573	-0.107	NA	0.006	-0.017
cg20429104	18	74114570	1.71E-105	0.275	0.365	-0.090	*ZNF516*	0.001	-0.018
cg11218434	13	45911764	1.80E-105	0.825	0.775	0.051	*TPT1*	8.36E-08	0.017
cg09001777	19	5851504	2.31E-105	0.230	0.286	-0.056	*FUT3*	1.10E-13	-0.027
cg20242427	11	61115736	2.35E-105	0.366	0.480	-0.114	*DAK*	1.46E-06	-0.033
cg00601368	7	149318081	2.50E-105	0.737	0.669	0.068	*ZNF767*	9.72E-07	0.020
cg00235484	6	136825415	2.52E-105	0.404	0.511	-0.107	*MAP7*	5.85E-08	-0.037
cg04205769	1	101488098	2.53E-105	0.399	0.503	-0.104	*DPH5*	7.70E-08	-0.032
cg20937934	18	21452788	2.56E-105	0.856	0.794	0.062	*LAMA3*	9.98E-15	0.031
cg11184109	3	53164962	2.63E-105	0.211	0.291	-0.080	*RFT1*	2.95E-11	-0.035
cg15132282	2	64488961	2.72E-105	0.284	0.385	-0.101	NA	1.55E-14	-0.051
cg06850285	22	21921269	2.76E-105	0.174	0.234	-0.060	*UBE2L3*	4.34E-18	-0.031
cg03664994	2	55246602	2.87E-105	0.331	0.425	-0.093	*RTN4*	4.92E-10	-0.034
cg20673721	17	25859382	3.18E-105	0.418	0.524	-0.107	*KSR1*	7.48E-14	-0.048
cg17356733	21	34774627	3.77E-105	0.449	0.557	-0.107	*IFNGR2*	2.67E-08	-0.035
cg24091474	19	36399185	4.15E-105	0.316	0.387	-0.071	*TYROBP*	4.96E-08	-0.023
cg21088438	14	70264869	4.38E-105	0.327	0.427	-0.100	*SLC10A1*	1.85E-10	-0.040
cg25079915	2	120192027	4.74E-105	0.313	0.392	-0.079	*TMEM37*	2.10E-07	-0.026
cg05798664	1	36825645	4.88E-105	0.299	0.375	-0.076	*STK40*	1.19E-06	-0.023
cg01231381	2	180130081	4.92E-105	0.428	0.517	-0.090	*SESTD1*	2.09E-08	-0.032
cg14018648	17	7083015	5.23E-105	0.344	0.427	-0.082	*ASGR1*	1.07E-10	-0.032
cg07253384	6	166877002	5.41E-105	0.255	0.345	-0.090	*RPS6KA2*	1.56E-14	-0.044
cg01492538	7	2774543	5.55E-105	0.311	0.394	-0.083	*GNA12*	4.97E-05	-0.019
cg01655008	14	93652954	5.63E-105	0.502	0.597	-0.096	*TMEM251*	2.15E-11	-0.038
cg01261013	21	37691747	5.70E-105	0.454	0.551	-0.097	*MORC3*	1.38E-09	-0.033
cg05875463	18	60385273	5.83E-105	0.351	0.442	-0.091	*PHLPP1*	1.59E-11	-0.039
cg04350202	10	111653363	5.83E-105	0.417	0.494	-0.077	*XPNPEP1*	1.90E-12	-0.033
cg07695566	17	61525112	5.87E-105	0.419	0.484	-0.065	*CYB561*	1.90E-13	-0.027

cg27586417	16	85577800	5.89E-105	0.461	0.562	-0.101	NA	2.69E-05	-0.026
cg00736299	16	4730465	5.89E-105	0.431	0.513	-0.082	MGRN1	2.41E-06	-0.020
cg24595580	15	100890996	6.16E-105	0.346	0.440	-0.094	NA	5.21E-14	-0.041
cg16647844	1	232903627	7.51E-105	0.327	0.420	-0.093	NA	3.11E-15	-0.046
cg07076915	16	2174754	7.65E-105	0.331	0.408	-0.077	PKD1	2.02E-04	-0.017
cg00876141	4	47837775	7.84E-105	0.311	0.381	-0.070	CORIN	9.11E-20	-0.038
cg20208009	22	19974048	8.42E-105	0.290	0.371	-0.081	ARVCF	3.09E-10	-0.033
cg02593884	16	2984574	8.63E-105	0.448	0.527	-0.079	FLYWCH1	6.68E-07	-0.021
cg24880665	1	202772164	9.03E-105	0.426	0.531	-0.105	KDM5B	1.59E-09	-0.042
cg00959259	3	122281975	9.28E-105	0.349	0.578	-0.229	PARP9	2.60E-48	-0.170
cg01142676	11	117695591	9.96E-105	0.230	0.311	-0.082	FXYD2	2.04E-12	-0.037
cg01950511	2	239289051	1.04E-104	0.345	0.445	-0.100	TRAF3IP1	5.22E-07	-0.030
cg16713889	14	74409552	1.17E-104	0.401	0.511	-0.109	FAM161B	3.14E-11	-0.042
cg13413719	6	3592887	1.17E-104	0.254	0.342	-0.088	NA	8.32E-25	-0.060
cg23384708	6	31544934	1.20E-104	0.718	0.647	0.071	TNF	3.62E-05	0.018
cg07880727	7	47846424	1.24E-104	0.266	0.345	-0.080	C7orf69	7.25E-15	-0.039
cg10702418	7	157090031	1.31E-104	0.371	0.469	-0.098	NA	3.47E-09	-0.036
cg21331324	20	57583000	1.34E-104	0.299	0.372	-0.074	CTSZ	8.94E-31	-0.055
cg19257402	12	132356988	1.35E-104	0.297	0.362	-0.065	NA	7.02E-09	-0.024
cg17262810	10	35676874	1.62E-104	0.374	0.468	-0.094	CCNY	4.86E-08	-0.032
cg13533061	17	74712429	1.74E-104	0.254	0.329	-0.075	JMJD6	2.47E-18	-0.042
cg10435245	11	47399998	1.82E-104	0.346	0.442	-0.096	SPI1	7.60E-10	-0.035
cg22045288	10	134258421	1.85E-104	0.284	0.347	-0.063	C10orf91	2.69E-16	-0.030
cg04234631	16	8738441	2.31E-104	0.421	0.493	-0.072	METTL22	1.85E-08	-0.026
cg22751954	6	143023932	2.48E-104	0.222	0.280	-0.057	NA	4.19E-13	-0.028
cg23511285	19	48205761	2.88E-104	0.459	0.542	-0.083	GLTSCR1	3.13E-07	-0.024
cg17639959	2	73297338	2.99E-104	0.316	0.387	-0.071	SFXN5	1.34E-14	-0.035
cg07970799	6	6614719	3.03E-104	0.802	0.740	0.062	LY86-AS1	3.20E-10	0.025
cg02889001	16	1519785	3.12E-104	0.339	0.435	-0.097	CLCN7	3.01E-08	-0.034
cg06330618	15	91428456	3.17E-104	0.314	0.375	-0.061	FES	3.61E-14	-0.028
cg12415479	3	129311698	3.57E-104	0.192	0.272	-0.080	PLXND1	4.11E-04	-0.019
cg20094462	19	848071	3.85E-104	0.334	0.410	-0.076	PRTN3	8.04E-06	-0.019
cg01288724	5	142575122	4.68E-104	0.335	0.432	-0.097	ARHGAP26	7.48E-09	-0.037
cg03126694	13	49001104	4.79E-104	0.323	0.405	-0.082	RB1	8.60E-07	-0.026
cg23193059	11	116706090	5.16E-104	0.354	0.456	-0.102	NA	3.50E-06	-0.032
cg02275530	17	59328313	5.25E-104	0.380	0.469	-0.089	BCAS3	1.96E-12	-0.041
cg25913761	15	90727560	5.53E-104	0.699	0.623	0.076	SEMA4B	3.06E-05	0.022
cg14192130	6	167535764	5.54E-104	0.770	0.700	0.070	CCR6	0.027	0.010
cg25734726	19	40948296	5.67E-104	0.436	0.520	-0.084	SERTAD3	5.72E-13	-0.037
cg11168614	14	87179368	5.81E-104	0.418	0.511	-0.093	NA	3.08E-05	-0.026
cg09163720	10	134400506	6.29E-104	0.490	0.600	-0.110	INPP5A	1.23E-07	-0.032
cg23696618	18	61583699	6.36E-104	0.362	0.447	-0.085	SERPINB10	1.36E-13	-0.039
cg11884933	7	2774414	6.40E-104	0.297	0.387	-0.090	GNA12	0.001	-0.018
cg08008352	16	27322356	6.89E-104	0.280	0.356	-0.076	NA	9.39E-10	-0.028
cg16558770	15	90548037	7.79E-104	0.322	0.413	-0.091	ZNF710	3.64E-05	-0.023
cg01332683	16	16190393	7.88E-104	0.368	0.443	-0.075	ABCC1	3.13E-16	-0.040
cg09315134	20	57583195	7.99E-104	0.252	0.326	-0.074	CTSZ	9.49E-12	-0.033
cg12555844	22	44568337	8.24E-104	0.262	0.331	-0.069	PARVG	9.80E-14	-0.032
cg20969424	5	73690902	8.43E-104	0.224	0.302	-0.078	NA	5.70E-19	-0.048
cg19090861	11	66085958	8.97E-104	0.190	0.251	-0.061	CD248	2.26E-05	-0.015
cg17585031	17	25798942	9.28E-104	0.624	0.569	0.055	KSR1	1.86E-05	0.013
cg19476647	12	6492948	1.22E-103	0.259	0.336	-0.077	LTBR	2.18E-10	-0.030
cg17164954	6	157345266	1.24E-103	0.281	0.364	-0.083	ARID1B	0.011	-0.014
cg03305017	7	151036715	1.25E-103	0.329	0.417	-0.088	NA	3.67E-08	-0.030
cg07186962	6	11393478	1.28E-103	0.281	0.367	-0.086	NA	1.89E-10	-0.034
cg22152446	4	48754953	1.29E-103	0.375	0.477	-0.101	FRYL	1.95E-08	-0.033
cg23956760	19	19178708	1.35E-103	0.410	0.492	-0.082	SLC25A42	3.59E-14	-0.038
cg04930596	12	124864528	1.39E-103	0.370	0.447	-0.077	NCOR2	2.21E-09	-0.028
cg26514961	12	94566784	1.44E-103	0.347	0.444	-0.097	PLXNC1	5.61E-08	-0.034
cg00435173	17	40284046	1.49E-103	0.405	0.494	-0.090	RAB5C	1.07E-17	-0.045
cg10236239	2	108994514	1.60E-103	0.252	0.324	-0.073	SULT1C4	4.90E-17	-0.036
cg13994241	17	46667725	1.64E-103	0.407	0.501	-0.094	NA	2.05E-18	-0.046
cg04904784	12	27170664	1.85E-103	0.452	0.540	-0.088	NA	2.54E-07	-0.028
cg03249630	10	90611782	1.88E-103	0.345	0.452	-0.108	ANKRD22	2.62E-09	-0.040
cg06829969	1	10460205	1.93E-103	0.488	0.582	-0.095	PGD	3.99E-16	-0.048
cg01526217	3	12524194	1.95E-103	0.327	0.430	-0.103	NA	4.59E-09	-0.037
cg26112639	1	247580106	1.98E-103	0.245	0.303	-0.058	NLRP3	6.84E-16	-0.030
cg12125117	16	57701461	2.12E-103	0.364	0.428	-0.064	GPR97	1.20E-08	-0.022
cg08359464	3	128370361	2.12E-103	0.412	0.518	-0.106	RPN1	4.27E-09	-0.036
cg04891053	1	161053558	2.23E-103	0.365	0.439	-0.074	PVRL4	5.16E-17	-0.035
cg26288715	6	135506834	2.27E-103	0.280	0.370	-0.090	MYB	4.57E-10	-0.035
cg27430637	1	26646801	2.31E-103	0.570	0.514	0.056	CD52	9.49E-10	0.019
cg07197230	22	17956641	2.34E-103	0.232	0.316	-0.085	CECR2	1.20E-08	-0.033

cg01987202	15	40633194	2.48E-103	0.258	0.316	-0.058	*C15orf52*	9.70E-15	-0.028
cg12647574	2	12824729	2.55E-103	0.315	0.389	-0.074	NA	3.58E-11	-0.030
cg08244301	19	17610751	2.63E-103	0.242	0.326	-0.084	*SLC27A1*	2.75E-04	-0.020
cg10099732	14	69256977	2.70E-103	0.680	0.594	0.085	*ZFP36L1*	5.21E-09	0.030
cg14180511	1	206946187	2.72E-103	0.327	0.419	-0.092	*IL10*	8.40E-23	-0.056
cg19477361	19	2607903	2.96E-103	0.680	0.628	0.052	*GNG7*	1.90E-15	0.026
cg04682905	7	5523636	3.06E-103	0.405	0.474	-0.069	*FBXL18*	9.85E-08	-0.022
cg23575275	6	36653973	3.22E-103	0.271	0.356	-0.085	*CDKN1A*	1.10E-09	-0.034
cg10575089	17	56606542	3.41E-103	0.764	0.702	0.062	*SEPT4*	6.39E-07	0.018
cg14619064	17	56355331	3.44E-103	0.555	0.633	-0.077	*MPO*	2.45E-05	-0.020
cg14438453	6	39786566	3.50E-103	0.403	0.476	-0.073	*DAAM2*	5.44E-04	-0.014
cg15736127	2	157292127	3.50E-103	0.363	0.469	-0.105	*GPD2*	1.59E-06	-0.031
cg03517226	16	89408322	3.57E-103	0.805	0.752	0.053	*ANKRD11*	2.81E-07	0.017
cg20055861	15	68055293	3.64E-103	0.772	0.705	0.068	*MAP2K5*	5.13E-05	0.017
cg22242539	17	1665220	3.91E-103	0.302	0.365	-0.063	*SERPINF1*	2.36E-14	-0.030
cg21210041	17	27443831	4.07E-103	0.433	0.506	-0.073	*MYO18A*	2.19E-07	-0.023
cg08945450	1	11795905	4.10E-103	0.261	0.329	-0.068	*AGTRAP*	1.04E-11	-0.029
cg16788865	12	46778801	4.17E-103	0.332	0.432	-0.100	NA	1.16E-13	-0.045
cg19399532	1	178512495	4.20E-103	0.248	0.305	-0.057	*C1orf220*	8.51E-10	-0.022
cg24596116	12	68665317	4.23E-103	0.328	0.400	-0.072	NA	2.55E-09	-0.027
cg05103064	10	7311500	4.26E-103	0.842	0.782	0.059	*SFMBT2*	9.35E-14	0.027
cg06122230	20	47887219	4.32E-103	0.489	0.596	-0.107	*ZNFX1*	5.56E-11	-0.043
cg17187521	12	125003379	4.74E-103	0.373	0.464	-0.091	*NCOR2*	1.48E-10	-0.038
cg08241318	2	64885581	4.96E-103	0.329	0.413	-0.084	NA	5.32E-11	-0.035
cg01944288	9	135036217	5.85E-103	0.227	0.306	-0.079	*NTNG2*	2.58E-05	-0.022
cg08472633	16	88907370	6.41E-103	0.324	0.396	-0.072	*GALNS*	4.77E-06	-0.021
cg06867755	2	225397387	7.16E-103	0.425	0.502	-0.077	*CUL3*	6.04E-13	-0.032
cg10357682	20	3745817	7.45E-103	0.429	0.536	-0.107	*C20orf27*	8.21E-04	-0.022
cg27664407	16	5116674	7.76E-103	0.309	0.398	-0.089	*C16orf89*	8.66E-06	-0.025
cg26928972	3	122043799	8.03E-103	0.385	0.490	-0.105	*CSTA*	1.49E-05	-0.027
cg13469851	9	26936447	8.57E-103	0.474	0.570	-0.095	*PLAA*	1.42E-04	-0.021
cg03559915	11	67201998	8.81E-103	0.732	0.661	0.071	*RPS6KB2*	1.08E-15	0.034
cg25198847	4	113192515	9.16E-103	0.440	0.547	-0.107	NA	2.20E-08	-0.037
cg03823431	17	79229385	9.23E-103	0.328	0.387	-0.059	*SLC38A10*	1.31E-06	-0.016
cg25907743	10	90519680	1.02E-102	0.221	0.298	-0.077	*LIPN*	1.94E-10	-0.032
cg01718139	19	54566838	1.12E-102	0.446	0.555	-0.109	*VSTM1*	1.02E-06	-0.033
cg13584531	2	99280070	1.13E-102	0.799	0.740	0.059	*MGAT4A*	2.21E-10	0.023
cg22820233	19	55385581	1.15E-102	0.356	0.460	-0.104	*FCAR*	2.35E-09	-0.041
cg08315613	19	11074303	1.16E-102	0.625	0.554	0.071	*SMARCA4*	7.28E-13	0.029
cg02678305	6	30460322	1.18E-102	0.753	0.699	0.055	*HLA-E*	3.62E-10	0.020
cg11529819	1	27695677	1.26E-102	0.263	0.324	-0.060	*FCN3*	1.23E-13	-0.028
cg05968188	6	16483997	1.27E-102	0.807	0.748	0.059	*ATXN1*	5.27E-08	0.020
cg12787553	20	57583188	1.28E-102	0.258	0.330	-0.072	*CTSZ*	2.37E-14	-0.035
cg02311193	17	46660002	1.29E-102	0.381	0.470	-0.089	NA	7.11E-11	-0.038
cg07201717	1	25031947	1.29E-102	0.243	0.302	-0.059	NA	2.04E-12	-0.026
cg23035449	15	90578555	1.32E-102	0.297	0.361	-0.065	*ZNF710*	2.71E-11	-0.027
cg23731826	15	90371692	1.37E-102	0.435	0.511	-0.076	NA	1.72E-05	-0.019
cg16692439	22	26831446	1.37E-102	0.287	0.369	-0.082	*ASPHD2*	9.55E-06	-0.022
cg03221483	17	75315081	1.46E-102	0.342	0.442	-0.100	*SEPT9*	5.08E-04	-0.023
cg14480116	2	65594890	1.51E-102	0.386	0.491	-0.104	*SPRED2*	6.08E-04	-0.022
cg11596902	11	123324834	1.51E-102	0.357	0.453	-0.097	NA	6.97E-12	-0.042
cg08053935	6	41691270	1.55E-102	0.301	0.351	-0.050	*TFEB*	8.38E-13	-0.021
cg18444544	8	22852741	1.68E-102	0.309	0.396	-0.087	*RHOBTB2*	2.06E-10	-0.036
cg11554295	7	5523691	1.70E-102	0.412	0.476	-0.063	*FBXL18*	1.33E-06	-0.019
cg23865980	10	80904376	1.78E-102	0.257	0.335	-0.078	*ZMIZ1*	1.47E-10	-0.033
cg02969426	13	45775005	1.95E-102	0.565	0.653	-0.088	*GTF2F2*	3.44E-06	-0.025
cg20566766	5	138723816	2.21E-102	0.338	0.391	-0.053	*MZB1*	1.92E-12	-0.023
cg02575483	6	117826706	2.22E-102	0.288	0.378	-0.090	*DCBLD1*	3.49E-11	-0.039
cg13914531	7	128579876	2.29E-102	0.201	0.260	-0.059	*IRF5*	4.97E-16	-0.031
cg14510299	1	27928494	2.97E-102	0.284	0.363	-0.079	*AHDC1*	5.66E-09	-0.028
cg20954870	5	173070254	3.14E-102	0.426	0.529	-0.102	NA	5.94E-12	-0.044
cg09452568	5	54275198	3.25E-102	0.282	0.373	-0.091	*ESM1*	7.00E-04	-0.019
cg06513247	17	75446661	3.38E-102	0.612	0.544	0.069	*SEPT9*	2.88E-08	0.021
cg10152449	7	2444534	3.58E-102	0.275	0.388	-0.113	*CHST12*	9.45E-28	-0.081
cg08175413	5	96294187	3.69E-102	0.226	0.316	-0.090	*LNPEP*	4.86E-17	-0.050
cg27513684	1	27729053	3.75E-102	0.225	0.283	-0.058	NA	3.60E-15	-0.028
cg01543184	17	79881543	3.85E-102	0.356	0.456	-0.100	*MAFG*	3.21E-04	-0.021
cg15253304	6	209809	3.89E-102	0.765	0.693	0.073	NA	4.50E-08	0.024
cg05331340	17	7083064	3.91E-102	0.415	0.481	-0.067	*ASGR1*	6.05E-10	-0.026
cg04764812	10	76582315	4.41E-102	0.274	0.356	-0.082	NA	9.14E-13	-0.038
cg08494738	16	4369512	4.65E-102	0.777	0.705	0.072	NA	9.79E-08	0.024
cg22409100	2	40658918	4.82E-102	0.273	0.347	-0.073	*SLC8A1*	1.76E-19	-0.042
cg16356622	1	36351841	4.94E-102	0.401	0.480	-0.079	*EIF2C1*	5.27E-16	-0.038

cg14288424	9	132429922	4.97E-102	0.333	0.391	-0.058	*PRRX2*	1.66E-14	-0.027
cg19210276	11	57529465	5.25E-102	0.285	0.371	-0.086	*TMX2-CTNND.*	7.63E-08	-0.029
cg14647287	4	6927183	5.30E-102	0.334	0.407	-0.073	*TBC1D14*	1.04E-15	-0.032
cg24738611	2	29149895	5.59E-102	0.310	0.386	-0.076	*WDR43*	1.41E-09	-0.028
cg13554018	9	91091556	5.76E-102	0.720	0.647	0.073	*SPIN1*	4.95E-12	0.030
cg00582663	17	7815834	5.83E-102	0.309	0.393	-0.084	*CHD3*	1.29E-11	-0.038
cg06635351	1	8882925	5.93E-102	0.304	0.396	-0.092	NA	8.20E-13	-0.043
cg21649013	12	132381683	6.14E-102	0.277	0.333	-0.056	*ULK1*	9.66E-10	-0.021
cg02251850	17	78851503	6.33E-102	0.793	0.719	0.074	*RPTOR*	1.55E-06	0.024
cg18276112	7	4755032	6.64E-102	0.388	0.495	-0.107	*FOXK1*	8.95E-06	-0.031
cg13916298	3	108896099	7.32E-102	0.233	0.295	-0.061	NA	1.08E-16	-0.033
cg18507018	8	92035517	7.52E-102	0.394	0.498	-0.104	*TMEM55A*	2.07E-06	-0.029
cg22459924	19	2607850	7.56E-102	0.717	0.640	0.077	*GNG7*	2.19E-09	0.030
cg09128944	2	169967580	7.94E-102	0.309	0.402	-0.092	NA	7.33E-09	-0.034
cg09092280	8	62646214	8.14E-102	0.276	0.359	-0.083	NA	2.33E-19	-0.048
cg14505439	9	134605210	8.34E-102	0.728	0.674	0.055	*RAPGEF1*	7.09E-06	0.015
cg19828970	2	106371647	8.78E-102	0.320	0.415	-0.094	*NCK2*	4.47E-15	-0.048
cg10699171	6	24936965	9.20E-102	0.703	0.620	0.082	NA	7.16E-16	0.045
cg07150145	1	47656137	9.42E-102	0.299	0.350	-0.051	*PDZK1IP1*	8.03E-13	-0.022
cg08669096	11	121229765	1.08E-101	0.245	0.331	-0.086	NA	2.12E-19	-0.052
cg23568192	10	44289380	1.10E-101	0.387	0.490	-0.103	NA	5.56E-06	-0.029
cg17479280	1	156466088	1.13E-101	0.745	0.678	0.067	*MEF2D*	0.003	0.011
cg25875049	15	43531947	1.23E-101	0.307	0.385	-0.078	*TGM5*	1.36E-21	-0.047
cg27510066	8	19536244	1.27E-101	0.446	0.554	-0.108	*CSGALNACT1*	5.81E-04	-0.023
cg06966839	17	55685941	1.33E-101	0.371	0.438	-0.067	*MSI2*	2.15E-06	-0.020
cg09890699	1	54821853	1.35E-101	0.189	0.250	-0.061	*SSBP3*	3.24E-05	-0.016
cg16814786	10	51575763	1.68E-101	0.309	0.401	-0.092	*NCOA4*	1.97E-06	-0.027
cg24065504	10	90613015	1.73E-101	0.289	0.376	-0.087	*ANKRD22*	1.37E-08	-0.032
cg23008718	2	43188851	1.83E-101	0.252	0.320	-0.068	NA	5.57E-08	-0.023
cg21937128	1	150971889	1.84E-101	0.251	0.335	-0.085	*FAM63A*	7.14E-06	-0.025
cg01923089	12	49627746	1.97E-101	0.271	0.343	-0.072	NA	5.81E-11	-0.029
cg10588135	17	59329903	1.98E-101	0.244	0.311	-0.068	*BCAS3*	2.57E-14	-0.034
cg21406144	2	59919051	2.08E-101	0.488	0.568	-0.080	NA	7.07E-07	-0.026
cg02635407	4	8200549	2.39E-101	0.263	0.315	-0.051	*SH3TC1*	1.09E-05	-0.014
cg15538607	19	10416150	2.67E-101	0.303	0.372	-0.069	*ZGLP1*	1.43E-12	-0.030
cg26437697	14	55143755	2.77E-101	0.479	0.566	-0.088	*SAMD4A*	1.06E-06	-0.028
cg08253808	14	102676957	3.15E-101	0.400	0.509	-0.109	*WDR20*	5.22E-06	-0.031
cg14160422	19	10735548	3.48E-101	0.795	0.732	0.063	*SLC44A2*	2.69E-10	0.025
cg01519464	1	24861818	3.94E-101	0.824	0.760	0.064	*RCAN3*	1.69E-14	0.033
cg03254928	11	118214810	3.99E-101	0.708	0.642	0.066	*CD3G*	8.47E-12	0.026
cg15142192	1	36521401	4.13E-101	0.345	0.448	-0.103	*EIF2C3*	2.49E-07	-0.033
cg10111352	16	16082095	5.75E-101	0.304	0.386	-0.082	*ABCC1*	1.47E-09	-0.031
cg07814567	4	100736658	6.03E-101	0.403	0.489	-0.086	*DAPP1*	2.40E-04	-0.019
cg22079077	15	41061384	6.89E-101	0.316	0.376	-0.060	*DNAJC17*	9.31E-09	-0.021
cg17952939	1	9154250	6.95E-101	0.329	0.409	-0.080	NA	4.77E-07	-0.025
cg26049998	17	65602730	7.06E-101	0.304	0.377	-0.073	*PITPNC1*	3.47E-13	-0.034
cg16110541	1	68679600	7.19E-101	0.327	0.395	-0.069	*WLS*	1.11E-09	-0.024
cg25344401	7	4755415	7.22E-101	0.436	0.552	-0.116	*FOXK1*	1.03E-04	-0.025
cg26333564	14	105147781	7.36E-101	0.314	0.374	-0.059	NA	1.06E-18	-0.032
cg04556210	19	47840110	8.33E-101	0.238	0.302	-0.064	*GPR77*	1.87E-08	-0.023
cg18537222	3	12435731	8.35E-101	0.415	0.513	-0.098	*PPARG*	1.49E-07	-0.031
cg01229658	15	70924395	8.40E-101	0.333	0.424	-0.092	NA	1.22E-07	-0.031
cg01564818	3	9464436	9.00E-101	0.220	0.283	-0.062	*SETD5*	9.64E-15	-0.030
cg17940587	10	89292231	9.12E-101	0.233	0.304	-0.071	*MINPP1*	2.32E-10	-0.028
cg17934775	15	93450129	9.29E-101	0.630	0.553	0.077	*CHD2*	7.08E-15	0.038
cg17973115	1	25333445	9.36E-101	0.292	0.387	-0.095	NA	4.54E-07	-0.031
cg17080697	6	30297382	9.59E-101	0.404	0.490	-0.085	*TRIM39*	0.001	-0.017
cg00517080	11	134098583	1.01E-100	0.790	0.731	0.058	*VPS26B*	3.80E-05	0.015
cg15945333	11	47399213	1.02E-100	0.294	0.380	-0.086	*SPI1*	6.25E-10	-0.035
cg01880463	20	45179413	1.05E-100	0.199	0.268	-0.069	*OCSTAMP*	4.06E-16	-0.038
cg01211396	6	30624478	1.11E-100	0.475	0.548	-0.073	*DHX16*	9.29E-08	-0.025
cg22534097	10	92681082	1.17E-100	0.382	0.477	-0.095	*ANKRD1*	3.83E-10	-0.039
cg17525495	17	56401734	1.23E-100	0.400	0.494	-0.094	*BZRAP1*	2.36E-05	-0.023
cg21208539	11	93864142	1.65E-100	0.278	0.358	-0.081	*PANX1*	2.80E-15	-0.040
cg03412153	10	13139217	1.75E-100	0.745	0.682	0.063	NA	1.98E-08	0.023
cg21272996	11	57529255	1.83E-100	0.304	0.394	-0.090	*TMX2-CTNND.*	9.62E-16	-0.048
cg07234097	1	184761873	1.88E-100	0.252	0.316	-0.063	*FAM129A*	8.64E-12	-0.026
cg06706813	3	194826411	1.95E-100	0.545	0.637	-0.092	*XXYLT1*	1.83E-04	-0.020
cg13424029	10	101297508	1.98E-100	0.265	0.356	-0.091	NA	5.45E-19	-0.050
cg14389547	19	3398778	2.13E-100	0.411	0.474	-0.062	*NFIC*	1.39E-08	-0.021
cg05025071	19	6887530	2.25E-100	0.349	0.447	-0.098	*EMR1*	0.009	-0.016
cg27227507	2	232546142	2.85E-100	0.344	0.413	-0.069	NA	1.30E-12	-0.032
cg03591753	6	35659141	2.93E-100	0.458	0.515	-0.058	*FKBP5*	1.27E-07	-0.019

cg01626885	15	45937757	3.00E-100	0.269	0.332	-0.063	*SQRDL*	6.45E-15	-0.031
cg27317813	12	4398508	3.02E-100	0.246	0.324	-0.078	*CCND2*	3.18E-07	-0.026
cg04344000	6	117869857	3.05E-100	0.793	0.730	0.063	*DCBLD1*	5.62E-09	0.024
cg09566995	11	1320634	3.09E-100	0.250	0.341	-0.090	*TOLLIP*	1.52E-12	-0.043
cg22018086	22	45124555	3.32E-100	0.258	0.327	-0.069	*PRR5-ARHGAI*	9.34E-14	-0.034
cg16226866	6	110418140	3.50E-100	0.371	0.477	-0.105	NA	6.42E-07	-0.032
cg01522592	11	75235311	3.55E-100	0.788	0.733	0.055	*GDPD5*	8.34E-09	0.020
cg21232161	2	135011626	3.72E-100	0.295	0.361	-0.066	*MGAT5*	0.004	-0.012
cg14324675	6	31554848	3.77E-100	0.267	0.343	-0.076	*LST1*	9.29E-14	-0.036
cg18780288	10	111659903	3.87E-100	0.230	0.305	-0.075	*XPNPEP1*	1.76E-09	-0.030
cg17627898	12	118782453	4.02E-100	0.323	0.409	-0.086	*TAOK3*	6.05E-06	-0.025
cg01442843	1	206729040	4.09E-100	0.782	0.718	0.064	*RASSF5*	4.41E-09	0.025
cg27097542	16	11706435	4.49E-100	0.290	0.361	-0.071	NA	1.93E-07	-0.022
cg21242356	17	46667683	4.53E-100	0.310	0.390	-0.080	NA	2.23E-13	-0.034
cg09088496	13	24825973	4.74E-100	0.810	0.745	0.065	*SPATA13*	3.51E-11	0.029
cg08402433	7	3020155	4.80E-100	0.278	0.351	-0.073	*CARD11*	3.74E-05	-0.019
cg12244275	2	70355397	5.04E-100	0.167	0.226	-0.059	NA	1.41E-19	-0.036
cg00648883	13	32524761	5.43E-100	0.248	0.326	-0.078	*EEF1DP3*	1.31E-13	-0.039
cg04722215	2	97205147	5.59E-100	0.664	0.589	0.075	*ARID5A*	9.29E-16	0.038
cg22324981	18	77283493	5.90E-100	0.702	0.618	0.084	*NFATC1*	6.05E-16	0.046
cg13541713	4	119947251	5.91E-100	0.355	0.449	-0.094	*SYNPO2*	5.58E-08	-0.031
cg05377120	12	10456885	5.93E-100	0.826	0.767	0.059	*KLRD1*	2.79E-09	0.023
cg23527902	17	61515708	6.06E-100	0.287	0.356	-0.068	*CYB561*	1.23E-10	-0.027
cg26528311	1	38462546	6.71E-100	0.280	0.354	-0.074	*FHL3*	4.02E-09	-0.028
cg06109482	2	232531087	6.74E-100	0.328	0.391	-0.063	NA	1.29E-11	-0.022
cg04382396	19	852311	6.81E-100	0.415	0.501	-0.087	*ELANE*	6.65E-06	-0.021
cg03050965	1	101705237	6.90E-100	0.782	0.718	0.063	*S1PR1*	5.29E-12	0.028
cg10505658	17	80084571	7.18E-100	0.707	0.631	0.076	*CCDC57*	2.99E-11	0.031
cg23258615	10	81961468	7.50E-100	0.251	0.308	-0.057	*ANXA11*	6.44E-06	-0.017
cg18591228	11	3175552	7.83E-100	0.413	0.501	-0.088	*OSBPL5*	1.59E-07	-0.028
cg01938023	5	138855699	7.87E-100	0.395	0.465	-0.070	*TMEM173*	2.50E-16	-0.038
cg02211741	11	46383924	8.71E-100	0.229	0.293	-0.064	*DGKZ*	1.12E-09	-0.024
cg12375586	7	43698429	8.76E-100	0.820	0.759	0.061	*COA1*	7.88E-07	0.019
cg25349990	1	229294380	8.82E-100	0.320	0.412	-0.092	NA	5.16E-10	-0.039
cg03471150	1	201797198	8.90E-100	0.430	0.501	-0.071	*IPO9*	2.87E-20	-0.044
cg03624195	19	14090310	9.43E-100	0.311	0.386	-0.075	*RFX1*	4.15E-06	-0.020
cg16140253	1	110166563	1.02E-99	0.290	0.351	-0.061	*AMPD2*	7.09E-11	-0.024
cg14768164	2	145025049	1.05E-99	0.794	0.739	0.054	*GTDC1*	7.29E-10	0.022
cg26306329	4	124400196	1.10E-99	0.251	0.327	-0.075	NA	1.11E-06	-0.024
cg08223235	18	60903834	1.12E-99	0.272	0.374	-0.101	*BCL2*	3.44E-08	-0.038
cg07543138	21	16434067	1.15E-99	0.297	0.378	-0.081	*NRIP1*	8.14E-18	-0.046
cg13015616	6	26086585	1.16E-99	0.800	0.747	0.053	*HFE*	6.58E-13	0.025
cg13984746	17	80398412	1.40E-99	0.324	0.412	-0.088	*HEXDC*	7.90E-13	-0.040
cg06526020	6	34308880	1.47E-99	0.447	0.549	-0.101	*RPS10-NUDT3*	0.007	-0.016
cg14834893	12	4398032	1.54E-99	0.276	0.355	-0.079	*CCND2*	2.17E-10	-0.033
cg00514723	20	40244770	1.55E-99	0.311	0.395	-0.085	*CHD6*	7.18E-10	-0.034
cg22794304	5	169734762	1.63E-99	0.276	0.344	-0.068	NA	8.51E-13	-0.030
cg09878888	11	57529614	1.72E-99	0.238	0.313	-0.075	*TMX2-CTNND.*	3.71E-06	-0.022
cg11690884	2	60533586	1.73E-99	0.238	0.308	-0.070	NA	1.40E-16	-0.039
cg11235297	5	1108315	1.76E-99	0.287	0.374	-0.088	*SLC12A7*	1.01E-05	-0.026
cg11564239	2	241644188	1.77E-99	0.637	0.579	0.058	NA	8.49E-11	0.022
cg14378925	11	64635728	1.81E-99	0.809	0.741	0.068	*EHD1*	7.15E-12	0.029
cg10161121	1	172628020	2.30E-99	0.818	0.757	0.061	*FASLG*	1.12E-11	0.028
cg24165638	19	831456	2.33E-99	0.467	0.548	-0.081	*AZU1*	2.28E-05	-0.021
cg05758804	12	57917389	2.42E-99	0.243	0.295	-0.052	*MBD6*	7.82E-12	-0.024
cg07499142	1	9788715	2.46E-99	0.591	0.508	0.082	*PIK3CD*	8.91E-11	0.035
cg04697624	12	69002178	2.47E-99	0.259	0.333	-0.074	NA	1.51E-11	-0.034
cg00277591	17	4079652	2.47E-99	0.784	0.707	0.077	*ANKFY1*	2.93E-17	0.036
cg01418188	11	3145609	2.53E-99	0.273	0.332	-0.058	*OSBPL5*	3.15E-18	-0.033
cg15586392	8	142238691	2.97E-99	0.445	0.539	-0.094	*SLC45A4*	2.74E-10	-0.039
cg21501175	3	194893148	3.06E-99	0.360	0.459	-0.099	*XXYLT1*	3.22E-08	-0.037
cg00201760	16	28518385	3.39E-99	0.324	0.400	-0.076	*IL27*	1.75E-10	-0.032
cg04084348	10	75677011	3.67E-99	0.218	0.283	-0.065	*PLAU*	2.66E-13	-0.032
cg24252148	11	68081257	3.68E-99	0.266	0.332	-0.067	*LRP5*	0.009	-0.011
cg12793803	17	62084217	3.80E-99	0.269	0.327	-0.058	*ICAM2*	1.10E-16	-0.031
cg10210690	1	55138059	3.90E-99	0.723	0.658	0.065	*HEATR8-TTC4*	0.028	0.010
cg16348358	1	32731477	3.98E-99	0.398	0.472	-0.073	*LCK*	1.76E-15	-0.037
cg13736939	7	157647241	4.15E-99	0.397	0.484	-0.086	*PTPRN2*	1.23E-11	-0.035
cg00754357	1	53019727	4.22E-99	0.377	0.465	-0.088	*ZCCHC11*	6.81E-14	-0.046
cg22365313	4	108636320	4.28E-99	0.308	0.391	-0.082	*PAPSS1*	7.19E-13	-0.040
cg09674546	16	30721220	4.33E-99	0.273	0.336	-0.063	*SRCAP*	1.12E-10	-0.025
cg05061769	15	59700360	4.64E-99	0.380	0.470	-0.091	NA	3.99E-07	-0.029
cg02520804	3	71629054	5.00E-99	0.421	0.523	-0.102	*FOXP1*	1.17E-07	-0.032

cg02740606	11	67206418	5.03E-99	0.829	0.764	0.065	*PTPRCAP*	0.002	0.013
cg01502428	17	76850266	5.19E-99	0.464	0.576	-0.112	*TIMP2*	1.74E-04	-0.026
cg23353000	17	42120896	5.21E-99	0.319	0.405	-0.086	*LSM12*	2.85E-05	-0.023
cg06964027	11	1073496	5.23E-99	0.211	0.269	-0.058	*MUC2*	2.83E-14	-0.029
cg22153407	1	230290089	5.60E-99	0.439	0.527	-0.088	*GALNT2*	5.71E-10	-0.033
cg15702277	1	8477935	5.75E-99	0.253	0.316	-0.063	*RERE*	3.24E-06	-0.019
cg13854219	1	101757037	5.93E-99	0.287	0.369	-0.082	NA	4.37E-19	-0.050
cg02538772	7	127699264	6.57E-99	0.749	0.679	0.070	*SND1*	2.84E-13	0.034
cg18478531	7	74204775	6.75E-99	0.354	0.442	-0.088	NA	8.59E-04	-0.019
cg03682581	2	85832389	7.01E-99	0.332	0.430	-0.098	*C2orf68*	8.36E-12	-0.045
cg27609217	11	46258039	7.04E-99	0.317	0.403	-0.087	NA	3.89E-10	-0.034
cg20487608	6	15288126	7.35E-99	0.262	0.349	-0.087	*JARID2*	1.22E-10	-0.038
cg25213720	5	176734343	7.78E-99	0.266	0.351	-0.085	*MXD3*	2.04E-16	-0.043
cg03547581	1	205684841	7.82E-99	0.247	0.328	-0.081	*NUCKS1*	0.004	-0.016
cg19060895	17	5419436	8.11E-99	0.343	0.402	-0.060	*NLRP1*	5.44E-08	-0.021
cg23251057	14	69480702	8.32E-99	0.260	0.326	-0.066	NA	1.32E-20	-0.040
cg09455881	3	47388736	8.52E-99	0.284	0.365	-0.081	NA	0.001	-0.019
cg16162970	14	105779952	8.83E-99	0.273	0.351	-0.078	*PACS2*	9.00E-07	-0.026
cg01352090	16	4103533	9.07E-99	0.189	0.251	-0.062	*ADCY9*	2.65E-09	-0.024
cg16115689	17	78764256	9.09E-99	0.531	0.466	0.065	*RPTOR*	9.82E-07	0.020
cg05065948	4	26275089	9.47E-99	0.422	0.512	-0.089	NA	2.42E-11	-0.036
cg21422623	1	226912213	9.99E-99	0.744	0.668	0.076	*ITPKB*	2.75E-10	0.031
cg07948875	1	169187004	1.02E-98	0.281	0.359	-0.078	*NME7*	1.91E-05	-0.021
cg02215171	4	89379156	1.12E-98	0.316	0.414	-0.098	*HERC5*	4.17E-20	-0.053
cg04943741	8	145651369	1.17E-98	0.481	0.552	-0.072	*VPS28*	2.45E-07	-0.022
cg25278941	6	139795527	1.19E-98	0.248	0.320	-0.072	*LOC645434*	5.33E-26	-0.050
cg24804768	12	754911	1.20E-98	0.355	0.434	-0.079	*NINJ2*	1.70E-18	-0.043
cg11054816	11	117183853	1.23E-98	0.361	0.455	-0.094	*BACE1*	2.40E-08	-0.034
cg13573745	9	97405553	1.25E-98	0.275	0.360	-0.085	NA	2.16E-13	-0.040
cg14059339	6	28875356	1.28E-98	0.376	0.483	-0.107	*TRIM27*	3.07E-05	-0.027
cg03427191	16	4697112	1.43E-98	0.785	0.725	0.060	*MGRN1*	1.21E-10	0.027
cg15518883	9	35650561	1.46E-98	0.776	0.712	0.064	*SIT1*	5.30E-13	0.029
**cg14134128**	17	76774832	1.49E-98	0.733	0.674	0.059	*CYTH1*	0.201	0.005
cg24453664	11	33758413	1.57E-98	0.269	0.350	-0.081	*CD59*	1.20E-07	-0.028
cg19320820	4	87966326	1.65E-98	0.229	0.310	-0.081	*AFF1*	1.27E-07	-0.029
cg27343325	20	3649247	1.68E-98	0.692	0.638	0.053	*ADAM33*	0.006	0.009
cg24024833	11	1892608	1.68E-98	0.588	0.523	0.066	*LSP1*	2.98E-13	0.029
cg00439981	6	170684299	1.75E-98	0.449	0.562	-0.113	*FAM120B*	8.93E-05	-0.026
cg02889973	1	234977572	1.77E-98	0.244	0.331	-0.087	NA	3.64E-12	-0.041
cg04528038	16	21171097	1.79E-98	0.213	0.279	-0.067	*TMEM159*	6.27E-06	-0.018
cg02266731	12	69357333	1.86E-98	0.262	0.338	-0.077	*CPM*	1.26E-07	-0.026
cg17209188	7	23387396	2.01E-98	0.382	0.467	-0.085	*IGF2BP3*	1.19E-11	-0.037
cg04730825	16	16116191	2.03E-98	0.299	0.357	-0.058	*ABCC1*	1.98E-12	-0.027
cg24025721	7	1220962	2.12E-98	0.482	0.569	-0.087	NA	3.37E-12	-0.035
cg05389236	6	36635087	2.19E-98	0.389	0.474	-0.085	NA	5.07E-13	-0.035
cg09978533	22	46465160	2.34E-98	0.253	0.353	-0.100	NA	5.72E-10	-0.043
cg10377921	19	15391946	2.42E-98	0.447	0.511	-0.064	*BRD4*	1.10E-15	-0.032
cg02938045	16	48222834	2.59E-98	0.635	0.707	-0.072	*ABCC11*	4.27E-08	-0.023
cg06784563	18	77284509	2.65E-98	0.576	0.502	0.074	*NFATC1*	7.58E-10	0.027
cg17889831	1	205181581	2.97E-98	0.297	0.384	-0.087	*DSTYK*	9.95E-15	-0.043
cg01377358	20	30948211	3.07E-98	0.683	0.615	0.068	*ASXL1*	4.69E-04	0.015
cg20405809	13	49117085	3.09E-98	0.289	0.350	-0.061	NA	1.14E-10	-0.026
cg17310882	6	158066609	3.25E-98	0.351	0.432	-0.081	*ZDHHC14*	4.74E-10	-0.032
cg11849798	8	102209920	3.43E-98	0.314	0.395	-0.081	*ZNF706*	1.37E-16	-0.044
cg24809269	17	74260692	3.60E-98	0.778	0.711	0.068	*FAM100B*	5.23E-08	0.024
cg20732703	7	50744032	3.75E-98	0.329	0.421	-0.091	*GRB10*	4.61E-11	-0.038
cg05399244	17	179611	3.77E-98	0.336	0.412	-0.075	*LOC10050638*	1.87E-18	-0.042
cg24310395	3	39309435	3.88E-98	0.792	0.728	0.064	*CX3CR1*	1.14E-07	0.023
cg19132462	11	10476608	4.02E-98	0.327	0.410	-0.083	*AMPD3*	1.01E-08	-0.031
cg04851702	16	81528974	4.24E-98	0.235	0.304	-0.069	*CMIP*	3.28E-06	-0.022
cg03977385	16	48468609	4.25E-98	0.206	0.268	-0.062	NA	1.36E-13	-0.031
cg13085627	15	101597169	4.48E-98	0.547	0.625	-0.078	*LRRK1*	1.44E-07	-0.024
cg19772161	6	3746570	4.49E-98	0.266	0.318	-0.052	*PXDC1*	4.31E-09	-0.020
cg16624482	21	43548126	4.62E-98	0.348	0.442	-0.094	*UMODL1*	2.26E-05	-0.027
cg20334115	1	226107899	4.73E-98	0.322	0.393	-0.070	*PYCR2*	1.17E-07	-0.023
cg03462322	16	126397	5.03E-98	0.211	0.279	-0.069	*MPG*	1.66E-06	-0.022
cg14039779	17	41857714	5.10E-98	0.421	0.507	-0.087	*DUSP3*	1.79E-10	-0.034
cg21211882	20	34770405	5.26E-98	0.267	0.332	-0.065	*EPB41L1*	5.93E-08	-0.021
cg09595185	17	46667812	5.29E-98	0.357	0.456	-0.098	NA	1.37E-12	-0.044
cg07507418	6	42372284	5.53E-98	0.611	0.552	0.059	*TRERF1*	0.001	0.011
cg05557991	16	89003641	6.42E-98	0.207	0.281	-0.074	*CBFA2T3*	5.68E-06	-0.024
cg16922167	1	27961746	6.91E-98	0.414	0.501	-0.087	*FGR*	5.03E-10	-0.033
cg18146927	1	200835888	8.17E-98	0.743	0.675	0.068	NA	4.34E-05	0.018

cg23374992	2	136872067	8.27E-98	0.227	0.306	-0.078	*CXCR4*	7.89E-09	-0.029
cg12073436	1	206958014	9.58E-98	0.707	0.619	0.088	NA	7.88E-09	0.035
cg19389293	18	13641872	1.03E-97	0.324	0.408	-0.085	*C18orf1*	8.77E-11	-0.036
cg19340455	7	1095720	1.07E-97	0.230	0.290	-0.059	*C7orf50*	2.75E-07	-0.020
cg01980222	6	41130917	1.09E-97	0.299	0.380	-0.081	*TREM2*	4.77E-12	-0.034
cg27627381	17	75452100	1.17E-97	0.771	0.715	0.056	*SEPT9*	7.06E-09	0.021
cg03056494	11	124595710	1.17E-97	0.274	0.347	-0.073	NA	1.46E-16	-0.040
cg00699569	1	157536296	1.20E-97	0.297	0.377	-0.080	NA	4.02E-11	-0.035
cg23012600	1	244088110	1.21E-97	0.333	0.427	-0.094	*LOC339529*	1.38E-08	-0.035
cg21005412	2	29149902	1.31E-97	0.474	0.546	-0.072	*WDR43*	8.97E-10	-0.028
cg15798221	17	62773704	1.40E-97	0.208	0.263	-0.055	*LOC146880*	1.01E-07	-0.019
cg15375424	5	131823451	1.47E-97	0.758	0.687	0.072	*IRF1*	2.59E-08	0.023
cg22652378	17	78533842	1.51E-97	0.416	0.490	-0.074	*RPTOR*	9.34E-05	-0.019
cg25408950	1	198290080	1.62E-97	0.307	0.390	-0.083	*NEK7*	5.12E-17	-0.045
cg19607845	6	52934457	1.78E-97	0.327	0.417	-0.089	*FBXO9*	4.70E-05	-0.023
cg15835339	10	10828105	1.88E-97	0.462	0.559	-0.097	*SFTA1P*	7.98E-06	-0.026
cg00858400	16	87904580	1.95E-97	0.452	0.525	-0.073	*SLC7A5*	7.64E-18	-0.039
cg17951713	8	130698161	2.13E-97	0.316	0.408	-0.091	NA	1.79E-09	-0.038
cg17801864	20	45179345	2.23E-97	0.250	0.323	-0.074	*OCSTAMP*	4.64E-11	-0.033
cg27058497	1	25291546	2.26E-97	0.623	0.530	0.092	*RUNX3*	1.35E-10	0.038
cg00465970	3	129024601	2.33E-97	0.346	0.427	-0.081	NA	1.64E-05	-0.023
cg13569051	9	124051703	2.39E-97	0.443	0.526	-0.083	*GSN*	1.75E-07	-0.028
cg23098195	5	141770115	2.40E-97	0.234	0.289	-0.055	NA	8.65E-20	-0.033
cg10220544	1	60171660	2.56E-97	0.323	0.415	-0.091	*FGGY*	1.26E-08	-0.036
cg08792703	11	60779666	2.68E-97	0.208	0.270	-0.062	*CD6*	2.82E-06	-0.020
cg07052231	12	7363540	2.70E-97	0.428	0.526	-0.098	*PEX5*	0.009	-0.016
cg13876315	9	35650526	2.76E-97	0.749	0.691	0.058	*SIT1*	6.02E-11	0.024
cg23206873	20	1303672	2.81E-97	0.316	0.390	-0.074	*SDCBP2*	2.72E-11	-0.031
cg13550410	1	36004549	2.82E-97	0.782	0.725	0.058	*KIAA0319L*	3.54E-06	0.017
cg23933241	17	33823690	2.90E-97	0.386	0.473	-0.087	NA	8.05E-05	-0.020
cg00463732	17	3704621	3.32E-97	0.369	0.448	-0.079	*ITGAE*	4.23E-07	-0.024
cg07043361	12	114404971	3.46E-97	0.300	0.393	-0.093	*RBM19*	1.23E-11	-0.042
cg11597277	20	52492248	3.50E-97	0.584	0.693	-0.109	*SUMO1P1*	2.19E-05	-0.024
cg16162590	5	159360802	3.55E-97	0.705	0.636	0.069	*ADRA1B*	1.24E-05	0.020
cg21281463	19	38423760	3.58E-97	0.248	0.324	-0.076	*SIPA1L3*	6.04E-14	-0.037
cg13023677	5	137675418	4.29E-97	0.200	0.270	-0.070	*FAM53C*	1.49E-07	-0.027
cg15296767	11	64122743	4.34E-97	0.311	0.387	-0.075	*CCDC88B*	0.020	-0.010
cg02508830	7	102576358	4.92E-97	0.235	0.309	-0.074	*FBXL13*	2.56E-11	-0.033
cg18517055	17	80581701	5.01E-97	0.499	0.608	-0.109	*WDR45L*	1.67E-04	-0.024
cg06884401	4	89978251	5.02E-97	0.239	0.309	-0.070	*FAM13A*	2.17E-09	-0.026
cg02990302	16	58155189	5.44E-97	0.347	0.428	-0.082	*C16orf80*	7.31E-11	-0.037
cg08827454	6	30922981	5.59E-97	0.269	0.342	-0.073	NA	1.69E-06	-0.024
cg08794544	11	45825518	6.00E-97	0.288	0.353	-0.065	*SLC35C1*	3.07E-13	-0.031
cg18814344	5	139040546	6.05E-97	0.330	0.396	-0.066	*CXXC5*	9.87E-13	-0.029
cg26130864	20	3208100	6.31E-97	0.427	0.502	-0.075	*SLC4A11*	1.94E-12	-0.036
cg20039443	20	50108912	6.41E-97	0.389	0.456	-0.067	*NFATC2*	1.95E-08	-0.021
cg21161403	8	1894604	6.52E-97	0.389	0.456	-0.067	*ARHGEF10*	6.22E-05	-0.017
cg23054840	10	45920485	6.56E-97	0.303	0.384	-0.081	*ALOX5*	1.10E-12	-0.037
cg22948808	11	46360763	7.10E-97	0.337	0.405	-0.068	*DGKZ*	2.31E-14	-0.035
cg10752406	19	827776	7.17E-97	0.258	0.322	-0.064	*AZU1*	2.57E-08	-0.020
cg03209720	6	157727126	7.42E-97	0.284	0.341	-0.057	*TMEM242*	2.38E-07	-0.019
cg18399451	6	16421273	7.89E-97	0.820	0.763	0.057	*ATXN1*	0.001	0.012
cg22437987	17	3820910	9.21E-97	0.195	0.251	-0.056	*P2RX1*	1.55E-11	-0.024
cg19674851	2	224720888	9.46E-97	0.429	0.530	-0.101	NA	6.58E-09	-0.035
cg07578772	3	150420821	9.68E-97	0.788	0.715	0.073	*FAM194A*	1.40E-15	0.038
cg24068972	11	60833459	9.79E-97	0.703	0.640	0.063	NA	6.59E-09	0.022
cg10145533	8	20350779	1.01E-96	0.817	0.750	0.067	NA	4.94E-09	0.028
cg04642165	7	150063431	1.17E-96	0.290	0.367	-0.077	NA	9.30E-11	-0.033
cg27616007	6	31554829	1.19E-96	0.187	0.252	-0.065	*LST1*	5.64E-17	-0.037
cg08594651	11	47415397	1.28E-96	0.307	0.387	-0.080	NA	1.84E-08	-0.031
cg18517540	18	32446543	1.30E-96	0.779	0.716	0.063	*DTNA*	7.39E-06	0.019
cg23506143	5	55761274	1.32E-96	0.366	0.459	-0.094	NA	8.54E-12	-0.040
cg01400750	4	145956168	1.35E-96	0.393	0.488	-0.095	*ANAPC10*	0.003	-0.019
cg13067714	14	69091453	1.36E-96	0.490	0.558	-0.068	NA	6.14E-13	-0.034
cg04742719	7	100463759	1.45E-96	0.335	0.413	-0.078	*TRIP6*	4.55E-10	-0.029
cg18461635	3	46990143	1.53E-96	0.858	0.805	0.053	*CCDC12*	5.50E-06	0.017
cg08562099	17	55389809	1.53E-96	0.365	0.442	-0.077	*MSI2*	3.58E-16	-0.042
cg13448978	1	27961796	1.55E-96	0.291	0.383	-0.091	*FGR*	5.85E-04	-0.023
cg00668519	3	11597941	1.69E-96	0.273	0.336	-0.063	*VGLL4*	1.29E-07	-0.022
cg19963522	6	154677796	1.70E-96	0.282	0.359	-0.077	*IPCEF1*	3.91E-19	-0.042
cg21243631	18	21154260	1.71E-96	0.570	0.500	0.070	*NPC1*	1.39E-08	0.025
cg12070987	11	67804055	1.71E-96	0.437	0.490	-0.054	*NDUFS8*	4.08E-10	-0.017
cg21513254	6	30624502	1.72E-96	0.343	0.418	-0.076	*DHX16*	5.49E-06	-0.022

cg01719995	4	144104893	1.79E-96	0.258	0.334	-0.076	*USP38*	7.53E-09	-0.030
cg19993258	15	78914264	1.79E-96	0.719	0.658	0.061	*CHRNA3*	6.58E-13	0.026
cg17984638	4	48136452	1.88E-96	0.838	0.778	0.061	*TXK*	4.96E-15	0.034
cg04862556	17	33775933	1.90E-96	0.298	0.365	-0.067	*SLFN13*	3.37E-09	-0.025
cg13359689	2	55636555	1.92E-96	0.324	0.380	-0.056	*CCDC88A*	2.62E-16	-0.029
cg10675058	11	63858880	1.99E-96	0.398	0.470	-0.073	*MACROD1*	1.15E-08	-0.025
cg20063095	2	134977141	2.07E-96	0.374	0.467	-0.093	NA	7.95E-09	-0.037
cg24171453	14	78079686	2.14E-96	0.211	0.277	-0.066	*SPTLC2*	7.99E-18	-0.037
cg12770539	7	36304062	2.28E-96	0.181	0.240	-0.058	*EEPD1*	0.002	-0.014
cg25006194	12	94288553	2.34E-96	0.251	0.323	-0.073	NA	5.38E-07	-0.023
cg03580292	4	174411723	2.40E-96	0.263	0.349	-0.087	NA	9.04E-18	-0.049
cg15089806	1	43418199	2.44E-96	0.730	0.666	0.064	*SLC2A1*	0.003	0.012
cg07346187	6	149775621	2.45E-96	0.227	0.296	-0.068	*ZC3H12D*	3.06E-09	-0.025
cg20613972	4	84037158	2.53E-96	0.466	0.568	-0.101	*PLAC8*	5.97E-05	-0.025
cg02054108	2	61607478	2.75E-96	0.551	0.637	-0.085	*USP34*	6.03E-10	-0.032
cg22675447	1	24745395	2.85E-96	0.349	0.446	-0.097	*NIPAL3*	2.61E-10	-0.040
cg09504873	16	66774626	2.96E-96	0.352	0.438	-0.085	*DYNC1LI2*	6.26E-07	-0.028
cg05656688	1	25254088	2.98E-96	0.723	0.644	0.078	*RUNX3*	3.47E-04	0.017
cg17972213	1	101704898	3.06E-96	0.788	0.710	0.078	*S1PR1*	4.69E-10	0.030
cg26736341	6	31545342	3.12E-96	0.803	0.735	0.068	*TNF*	8.48E-05	0.018
cg07377178	6	3025064	3.13E-96	0.328	0.396	-0.068	NA	6.64E-06	-0.019
cg13761998	17	37894403	3.13E-96	0.290	0.362	-0.072	*GRB7*	1.90E-07	-0.022
cg06100973	19	852114	3.45E-96	0.320	0.419	-0.099	*ELANE*	3.11E-11	-0.044
cg04407248	16	31148196	3.46E-96	0.491	0.582	-0.092	*PRSS8*	6.90E-09	-0.032
cg09404516	2	54832147	3.51E-96	0.248	0.307	-0.059	*SPTBN1*	1.06E-04	-0.014
cg21500909	19	41870640	3.80E-96	0.358	0.449	-0.091	*TMEM91*	1.64E-14	-0.044
cg20513976	20	62367893	3.88E-96	0.565	0.454	0.111	*LIME1*	5.25E-06	0.029
cg06317209	12	58210878	4.04E-96	0.334	0.438	-0.104	*AVIL*	2.82E-05	-0.029
cg13443575	17	33775961	4.07E-96	0.276	0.345	-0.070	*SLFN13*	2.57E-09	-0.025
cg12405599	3	128370463	4.11E-96	0.416	0.523	-0.107	*RPN1*	1.67E-05	-0.029
cg01978937	20	2671574	4.19E-96	0.203	0.277	-0.074	NA	5.93E-08	-0.027
cg00830621	16	18799106	4.42E-96	0.847	0.792	0.055	*RPS15A*	5.79E-04	0.012
cg09714919	16	85981947	4.45E-96	0.325	0.394	-0.069	NA	7.27E-13	-0.030
cg07896832	8	67975874	4.46E-96	0.378	0.484	-0.107	*CSPP1*	1.99E-07	-0.035
cg05908241	7	143109367	4.63E-96	0.237	0.303	-0.066	*EPHA1-AS1*	1.53E-17	-0.038
cg15159104	15	43809865	4.66E-96	0.267	0.326	-0.058	*RNU6-28*	1.43E-12	-0.027
cg10133462	4	1305113	4.66E-96	0.803	0.741	0.062	*MAEA*	1.57E-17	0.034
cg02685484	11	67203360	4.89E-96	0.711	0.655	0.056	*PTPRCAP*	7.03E-12	0.025
cg22761077	19	850975	5.32E-96	0.193	0.253	-0.060	*ELANE*	1.50E-07	-0.021
cg25190513	12	123201362	5.42E-96	0.451	0.563	-0.112	*HCAR3*	4.72E-08	-0.037
cg26923863	4	1221838	5.45E-96	0.204	0.281	-0.077	*CTBP1*	2.16E-08	-0.028
cg07538204	8	62612575	6.21E-96	0.216	0.295	-0.079	*ASPH*	8.81E-09	-0.031
cg06632549	6	31529993	6.78E-96	0.716	0.638	0.077	NA	3.66E-04	0.016
cg15146752	1	16482767	6.96E-96	0.456	0.513	-0.057	*EPHA2*	5.09E-13	-0.026
cg20098015	22	50971140	7.09E-96	0.335	0.490	-0.155	*ODF3B*	2.15E-33	-0.100
cg25298189	19	935259	8.05E-96	0.419	0.493	-0.074	*ARID3A*	2.99E-08	-0.022
cg11977605	4	1076247	8.07E-96	0.247	0.308	-0.061	*RNF212*	1.80E-16	-0.033
cg01128574	7	2058865	8.71E-96	0.344	0.410	-0.066	*MAD1L1*	1.25E-11	-0.028
cg26106166	10	16821670	9.06E-96	0.703	0.606	0.097	*RSU1*	1.32E-21	0.060
cg15361215	10	120873630	9.24E-96	0.263	0.345	-0.082	*FAM45B*	1.79E-10	-0.036
cg05729480	17	75276428	9.46E-96	0.382	0.456	-0.074	*SEPT9*	3.88E-08	-0.027
cg21149266	15	59146882	9.84E-96	0.212	0.283	-0.071	*FAM63B*	4.47E-11	-0.030
cg19529732	12	122712101	1.07E-95	0.626	0.539	0.086	*DIABLO*	1.54E-07	0.030
cg21959598	7	55637719	1.07E-95	0.782	0.712	0.070	*VOPP1*	8.52E-11	0.031
cg18382353	10	45495981	1.22E-95	0.204	0.272	-0.068	*ZNF22*	0.015	-0.011
cg26719831	16	67279967	1.23E-95	0.237	0.292	-0.054	*FHOD1*	1.69E-14	-0.028
cg17547295	5	157282503	1.31E-95	0.429	0.520	-0.091	*CLINT1*	8.79E-12	-0.042
cg03906115	2	33359529	1.41E-95	0.267	0.369	-0.102	*LTBP1*	4.33E-13	-0.051
cg17346246	12	123214864	1.48E-95	0.851	0.789	0.061	*HCAR1*	2.08E-20	0.039
cg18471664	3	5028143	1.55E-95	0.228	0.286	-0.058	NA	6.08E-20	-0.034
cg23210971	4	88049275	1.61E-95	0.490	0.582	-0.092	*AFF1*	1.04E-04	-0.022
cg19317258	1	201662881	1.74E-95	0.808	0.754	0.055	*NAV1*	1.53E-04	0.013
cg04603130	19	2550027	1.82E-95	0.359	0.469	-0.110	*GNG7*	2.80E-09	-0.043
cg14849855	1	67807960	1.92E-95	0.768	0.707	0.062	*IL12RB2*	2.79E-08	0.022
cg06496803	4	6940915	1.98E-95	0.370	0.443	-0.073	*TBC1D14*	1.05E-08	-0.028
cg03048029	21	34101413	1.99E-95	0.370	0.471	-0.101	*GCFC1-AS1*	1.59E-04	-0.024
**cg18505752**	6	32808752	2.28E-95	0.715	0.653	0.062	*PSMB8*	0.164	0.005
cg01565130	1	167690726	2.73E-95	0.268	0.357	-0.089	*MPZL1*	2.03E-10	-0.040
cg03543954	16	85116335	2.92E-95	0.217	0.290	-0.072	*KIAA0513*	1.44E-06	-0.025
cg11173131	20	45179226	3.04E-95	0.254	0.340	-0.086	*OCSTAMP*	5.52E-09	-0.035
cg04739200	6	135517046	3.37E-95	0.404	0.505	-0.101	*MYB*	1.14E-04	-0.024
cg10168494	3	187635183	3.43E-95	0.228	0.303	-0.075	NA	1.57E-21	-0.049
cg18493214	6	17764205	3.73E-95	0.298	0.385	-0.087	*KIF13A*	1.57E-12	-0.044

cg21179618	9	139424642	3.95E-95	0.346	0.420	-0.073	*NOTCH1*	0.003	-0.013
cg24103651	10	113987401	4.04E-95	0.232	0.302	-0.070	NA	1.06E-18	-0.041
cg13522882	2	102316496	4.17E-95	0.835	0.783	0.052	*MAP4K4*	4.34E-09	0.021
cg25662857	17	80275542	4.36E-95	0.686	0.619	0.066	*CD7*	2.68E-09	0.025
cg23414595	22	30129607	4.54E-95	0.310	0.361	-0.051	*ZMAT5*	5.89E-13	-0.024
cg18004847	19	1155056	4.64E-95	0.422	0.496	-0.074	*SBNO2*	2.50E-05	-0.021
cg00404861	12	131590393	4.74E-95	0.651	0.580	0.071	*GPR133*	1.13E-09	0.026
cg13594542	9	138850547	5.11E-95	0.264	0.331	-0.067	*UBAC1*	1.14E-06	-0.021
cg03639929	6	32765402	5.42E-95	0.200	0.281	-0.081	NA	7.70E-08	-0.031
cg16555909	1	23721285	5.63E-95	0.493	0.573	-0.080	*TCEA3*	1.75E-10	-0.034
cg23514374	11	128372328	5.68E-95	0.850	0.795	0.056	*ETS1*	1.92E-12	0.028
cg01581222	19	10958952	6.54E-95	0.178	0.231	-0.053	*C19orf38*	5.76E-05	-0.015
cg03121834	7	99970448	6.59E-95	0.273	0.333	-0.061	*PILRA*	9.34E-15	-0.030
cg12115800	3	73144435	7.27E-95	0.446	0.516	-0.070	NA	2.65E-06	-0.022
cg08140114	6	139482238	7.49E-95	0.785	0.720	0.065	*HECA*	5.69E-05	0.017
cg20168849	3	188995030	8.59E-95	0.391	0.476	-0.085	*TPRG1*	6.88E-13	-0.040
cg10752508	3	98313516	8.91E-95	0.353	0.446	-0.093	*CPOX*	3.47E-08	-0.034
cg00181432	3	194393818	8.91E-95	0.353	0.431	-0.077	*LSG1*	2.00E-15	-0.044
cg01686975	7	138816336	9.37E-95	0.248	0.320	-0.072	NA	3.62E-13	-0.036
cg13573582	1	44887933	9.76E-95	0.401	0.495	-0.094	*RNF220*	9.62E-09	-0.035
cg08574915	17	79924772	1.11E-94	0.255	0.331	-0.076	NA	1.85E-08	-0.027
cg22193912	17	79881523	1.16E-94	0.316	0.419	-0.103	*MAFG*	0.003	-0.019
cg03431524	7	100142441	1.20E-94	0.716	0.647	0.069	*AGFG2*	0.011	0.011
cg26841425	10	45958771	1.20E-94	0.381	0.463	-0.082	*MARCH8*	1.26E-04	-0.019
cg02291556	1	47656140	1.23E-94	0.208	0.262	-0.054	*PDZK1IP1*	3.14E-06	-0.018
cg00374672	7	100463416	1.35E-94	0.249	0.326	-0.077	*SLC12A9*	8.96E-13	-0.036
cg04075726	2	69499425	1.44E-94	0.390	0.483	-0.093	NA	1.08E-06	-0.029
cg09131339	1	109914235	1.46E-94	0.241	0.306	-0.065	*SORT1*	1.17E-11	-0.029
cg09481483	16	11734383	1.50E-94	0.222	0.291	-0.069	NA	1.54E-08	-0.027
cg11939496	1	160833560	1.56E-94	0.484	0.572	-0.088	*CD244*	2.90E-10	-0.033
cg08670658	11	67052992	1.61E-94	0.380	0.468	-0.088	*ADRBK1*	4.58E-04	-0.019
cg22767754	4	48038871	1.84E-94	0.239	0.297	-0.058	*NIPAL1*	4.44E-08	-0.023
cg05859308	14	71712645	1.85E-94	0.763	0.693	0.070	NA	7.23E-08	0.025
cg07638500	3	123371420	1.90E-94	0.253	0.307	-0.055	*MYLK*	3.49E-09	-0.021
cg24056365	1	110834615	2.02E-94	0.394	0.487	-0.092	*LOC440600*	1.25E-04	-0.023
cg13423759	1	37937403	2.03E-94	0.179	0.252	-0.073	*LOC728431*	1.15E-10	-0.030
cg21171320	9	73178360	2.03E-94	0.287	0.365	-0.078	*TRPM3*	2.85E-24	-0.054
cg16506970	4	6809109	2.06E-94	0.350	0.448	-0.098	*KIAA0232*	1.75E-07	-0.033
cg00159439	22	38597193	2.16E-94	0.743	0.692	0.051	*MAFF*	5.47E-06	0.015
cg17346145	17	77755547	2.19E-94	0.297	0.362	-0.065	*CBX2*	0.013	-0.010
cg05888181	8	1952319	2.23E-94	0.363	0.462	-0.099	*KBTBD11*	4.43E-08	-0.035
cg14584535	9	125797187	2.28E-94	0.359	0.449	-0.090	*GPR21*	4.64E-12	-0.042
cg04695882	16	20756332	2.34E-94	0.294	0.373	-0.079	NA	1.36E-05	-0.024
cg23828876	3	196001706	2.48E-94	0.801	0.736	0.065	*PCYT1A*	0.008	0.011
cg19673549	5	54916621	2.53E-94	0.423	0.530	-0.106	NA	3.85E-04	-0.024
cg05667818	7	116786870	2.58E-94	0.302	0.384	-0.082	*ST7*	1.35E-16	-0.046
cg15658793	19	3398810	2.71E-94	0.400	0.462	-0.062	*NFIC*	8.05E-08	-0.019
cg12465678	1	27953336	2.71E-94	0.275	0.348	-0.072	*FGR*	3.14E-12	-0.033
cg14018735	9	136270734	2.74E-94	0.388	0.459	-0.071	*C9orf96*	4.56E-14	-0.036
cg00610577	12	12008666	3.19E-94	0.178	0.244	-0.066	*RNU6-19*	0.006	-0.013
cg13334949	20	44471068	3.38E-94	0.247	0.306	-0.059	*SNX21*	3.54E-18	-0.036
cg24621362	12	6492890	3.50E-94	0.245	0.312	-0.067	*LTBR*	1.12E-14	-0.037
cg00357958	12	123215010	3.59E-94	0.662	0.583	0.079	*HCAR1*	1.64E-08	0.029
cg03244997	17	43503144	3.65E-94	0.358	0.415	-0.057	*ARHGAP27*	1.55E-11	-0.026
cg12559197	5	76654783	3.67E-94	0.323	0.403	-0.080	*PDE8B*	2.55E-09	-0.031
cg09803764	7	107572753	3.78E-94	0.363	0.450	-0.087	*LAMB1*	2.02E-09	-0.035
cg02836135	6	108052093	3.80E-94	0.816	0.759	0.057	*SCML4*	1.76E-08	0.023
cg27027055	16	79306292	3.91E-94	0.798	0.738	0.059	NA	6.20E-13	0.029
cg21400896	17	47288569	3.93E-94	0.783	0.724	0.059	*GNGT2*	6.34E-05	0.016
cg11908131	2	106685045	4.25E-94	0.373	0.467	-0.093	*C2orf40*	7.66E-05	-0.022
cg11316905	17	7035367	4.89E-94	0.273	0.328	-0.054	NA	6.00E-10	-0.021
cg20900050	15	31689509	5.19E-94	0.383	0.461	-0.077	NA	3.61E-08	-0.030
cg15086439	1	236563070	5.33E-94	0.377	0.484	-0.108	*EDARADD*	0.040	-0.014
cg07880943	13	46744500	5.59E-94	0.670	0.612	0.058	*LCP1*	1.11E-08	0.022
cg15382568	22	25800078	5.85E-94	0.254	0.337	-0.082	NA	8.85E-09	-0.033
cg14876761	4	683206	6.79E-94	0.214	0.268	-0.054	*MFSD7*	1.65E-05	-0.014
cg10454258	16	27443933	6.92E-94	0.354	0.436	-0.082	*IL21R*	2.13E-14	-0.042
cg04500986	1	6526531	6.92E-94	0.682	0.623	0.059	*PLEKHG5*	2.49E-22	0.037
cg03311274	15	58624344	6.99E-94	0.228	0.287	-0.059	NA	9.68E-18	-0.036
cg23661721	14	95991371	7.14E-94	0.354	0.445	-0.090	NA	3.29E-07	-0.032
cg10540573	2	218869684	7.49E-94	0.775	0.721	0.054	NA	0.005	0.010
cg16245431	7	1005089	7.69E-94	0.284	0.361	-0.077	*COX19*	5.86E-09	-0.031
cg13815684	6	31540440	7.79E-94	0.771	0.719	0.052	*LTA*	5.32E-08	0.018

cg26814703	13	31019673	7.93E-94	0.248	0.316	-0.067	NA	1.55E-15	-0.033
cg20181887	12	123753272	8.20E-94	0.325	0.435	-0.111	*CDK2AP1*	0.002	-0.022
cg01279933	19	10663970	8.47E-94	0.613	0.555	0.058	*ATG4D*	3.95E-11	0.023
cg00864954	16	70587308	8.67E-94	0.796	0.745	0.051	*SF3B3*	4.03E-08	0.020
cg23866916	19	1155738	8.71E-94	0.486	0.553	-0.067	*SBNO2*	1.14E-07	-0.023
cg27193519	16	4714443	8.73E-94	0.275	0.354	-0.079	*MGRN1*	0.038	-0.011
cg10009801	6	30168645	9.04E-94	0.300	0.382	-0.081	*TRIM26*	4.34E-14	-0.042
cg11327657	21	46388162	9.44E-94	0.250	0.334	-0.085	*FAM207A*	1.02E-07	-0.032
cg05989054	19	1402626	9.45E-94	0.189	0.243	-0.054	*GAMT*	1.61E-08	-0.020
cg27534520	15	99343918	9.45E-94	0.266	0.340	-0.074	*IGF1R*	1.83E-09	-0.031
cg08842616	16	70733772	1.19E-93	0.413	0.473	-0.060	*VAC14*	7.72E-06	-0.016
cg07608052	5	176937527	1.29E-93	0.372	0.437	-0.064	*DOK3*	1.55E-08	-0.023
cg06289802	17	72748189	1.29E-93	0.309	0.381	-0.072	*SLC9A3R1*	4.27E-10	-0.029
cg10614223	19	18111389	1.35E-93	0.653	0.594	0.059	*ARRDC2*	1.27E-07	0.020
cg19977179	3	128779500	1.42E-93	0.240	0.292	-0.052	*GP9*	2.81E-05	-0.016
cg02776283	2	220074531	1.42E-93	0.181	0.246	-0.065	*ABCB6*	1.04E-08	-0.024
cg25578781	11	128446070	1.42E-93	0.270	0.345	-0.075	*ETS1*	3.49E-13	-0.039
cg27176729	1	181056650	1.47E-93	0.330	0.429	-0.099	*IER5*	0.004	-0.020
cg09499849	2	158695158	1.47E-93	0.239	0.308	-0.069	*ACVR1*	6.48E-11	-0.033
cg04381888	12	48195549	1.47E-93	0.253	0.314	-0.060	*HDAC7*	4.56E-13	-0.029
cg08425796	22	50981121	1.54E-93	0.289	0.384	-0.095	NA	5.87E-07	-0.031
cg12836863	13	32889023	1.58E-93	0.418	0.529	-0.111	*BRCA2*	9.13E-17	-0.061
cg21567649	2	43245642	1.67E-93	0.342	0.407	-0.065	NA	7.52E-05	-0.016
cg14039246	1	53365446	1.69E-93	0.341	0.430	-0.089	*ECHDC2*	1.52E-07	-0.032
cg25363080	5	81452987	1.74E-93	0.269	0.344	-0.076	*ATG10*	3.02E-21	-0.052
**cg22595920**	1	9716050	1.97E-93	0.257	0.325	-0.068	*PIK3CD*	0.052	-0.009
cg07363330	5	55148493	2.00E-93	0.359	0.451	-0.093	*IL31RA*	1.36E-11	-0.040
cg10070185	14	94857151	2.29E-93	0.397	0.449	-0.053	*SERPINA1*	2.25E-11	-0.023
cg03240301	7	23387383	2.50E-93	0.276	0.360	-0.085	*IGF2BP3*	1.89E-17	-0.051
cg06872313	12	14413185	2.59E-93	0.348	0.426	-0.077	NA	1.26E-08	-0.027
cg18107006	2	38831166	2.66E-93	0.256	0.334	-0.078	*HNRPLL*	6.57E-18	-0.047
cg13423554	1	42317463	2.70E-93	0.200	0.267	-0.067	*HIVEP3*	3.00E-07	-0.023
cg26052586	18	20717411	2.71E-93	0.256	0.320	-0.064	*CABLES1*	3.56E-07	-0.022
cg07168232	1	209822928	2.73E-93	0.376	0.439	-0.063	*LAMB3*	1.68E-16	-0.036
cg01468420	6	33393112	3.08E-93	0.219	0.272	-0.053	*SYNGAP1*	0.002	-0.010
cg17220933	1	206729613	3.12E-93	0.746	0.687	0.059	*RASSF5*	1.35E-06	0.019
cg14053764	14	55761432	3.21E-93	0.796	0.740	0.056	*FBXO34*	1.35E-14	0.030
cg19601328	17	43368729	3.33E-93	0.259	0.334	-0.075	*MAP3K14*	2.28E-09	-0.031
cg15997393	7	1961869	3.37E-93	0.751	0.666	0.085	*MAD1L1*	3.91E-16	0.044
cg20001791	6	16239799	3.43E-93	0.305	0.405	-0.100	*GMPR*	8.39E-06	-0.030
cg19369955	12	2030178	3.55E-93	0.193	0.253	-0.060	NA	3.92E-13	-0.030
cg05384139	12	123548027	3.67E-93	0.283	0.341	-0.058	*PITPNM2*	1.72E-19	-0.031
cg20305595	1	9293833	3.95E-93	0.410	0.468	-0.058	*H6PD*	4.34E-15	-0.030
cg25137372	12	96390059	4.62E-93	0.287	0.365	-0.077	*HAL*	1.32E-11	-0.034
cg19869443	1	84766821	5.01E-93	0.331	0.417	-0.087	*SAMD13*	1.14E-10	-0.040
cg18390025	10	103986736	5.02E-93	0.317	0.418	-0.100	*ELOVL3*	8.85E-04	-0.023
cg14985891	1	116270626	5.09E-93	0.355	0.452	-0.097	*CASQ2*	5.52E-05	-0.024
cg14830166	12	11821908	5.10E-93	0.258	0.330	-0.071	*ETV6*	1.40E-13	-0.036
cg15723222	17	59222476	5.51E-93	0.292	0.360	-0.067	*BCAS3*	4.92E-08	-0.022
cg24886563	17	3820150	5.96E-93	0.231	0.282	-0.052	*P2RX1*	1.83E-09	-0.022
cg25365958	13	42615991	6.03E-93	0.206	0.266	-0.060	*DGKH*	3.03E-10	-0.026
cg04716530	16	30485684	7.53E-93	0.835	0.781	0.054	*ITGAL*	0.012	0.009
cg23314866	19	48016020	7.96E-93	0.225	0.285	-0.060	*NAPA*	5.09E-10	-0.024
cg04176246	17	55434640	8.07E-93	0.290	0.360	-0.070	*MSI2*	1.80E-08	-0.027
cg16177481	11	61740158	8.33E-93	0.160	0.217	-0.056	NA	1.29E-10	-0.027
cg15068318	6	2958750	9.14E-93	0.297	0.359	-0.062	*SERPINB6*	5.85E-06	-0.019
cg27087781	16	30108404	9.29E-93	0.292	0.371	-0.079	*YPEL3*	1.93E-05	-0.022
cg26306994	12	6493521	9.48E-93	0.349	0.415	-0.066	*LTBR*	8.54E-14	-0.033
cg17707487	13	114261869	9.51E-93	0.377	0.449	-0.072	*TFDP1*	7.67E-06	-0.021
cg00203037	3	16360321	9.67E-93	0.416	0.523	-0.107	*RFTN1*	1.26E-07	-0.039
cg05872923	17	7461260	9.73E-93	0.244	0.322	-0.078	*TNFSF13*	1.61E-07	-0.029
cg27360727	2	62408302	9.74E-93	0.253	0.309	-0.056	NA	4.51E-12	-0.026
cg19303748	11	844085	1.21E-92	0.181	0.235	-0.054	*TSPAN4*	8.22E-08	-0.020
cg08351131	17	43242591	1.26E-92	0.337	0.401	-0.064	*HEXIM2*	2.76E-21	-0.036
cg23646614	11	48083100	1.34E-92	0.191	0.243	-0.053	*PTPRJ*	1.68E-14	-0.028
cg00135888	11	57407543	1.37E-92	0.237	0.290	-0.052	NA	4.83E-12	-0.026
cg19867991	19	13051573	1.39E-92	0.331	0.403	-0.072	*CALR*	7.35E-13	-0.035
cg24743156	14	39734964	1.49E-92	0.347	0.433	-0.086	*CTAGE5*	4.46E-07	-0.029
cg12963656	3	101659687	1.50E-92	0.242	0.328	-0.086	NA	6.93E-05	-0.023
cg00708789	4	16083440	1.51E-92	0.267	0.342	-0.075	*PROM1*	1.99E-14	-0.038
cg15814508	3	150996078	1.52E-92	0.191	0.252	-0.061	*P2RY14*	1.61E-14	-0.033
cg22175006	2	223757281	1.55E-92	0.434	0.528	-0.094	*ACSL3*	7.59E-10	-0.037
cg17744604	1	206946166	1.68E-92	0.308	0.399	-0.092	*IL10*	3.19E-11	-0.041

cg23128949	12	124986124	1.73E-92	0.294	0.368	-0.074	*NCOR2*	6.68E-06	-0.023
cg02380585	17	33776683	1.80E-92	0.262	0.345	-0.082	*SLFN13*	2.30E-11	-0.039
cg24474182	3	151047307	1.86E-92	0.362	0.456	-0.095	*P2RY13*	9.41E-05	-0.026
cg14613594	20	1878646	1.97E-92	0.323	0.384	-0.061	*SIRPA*	5.60E-12	-0.027
cg00005619	11	47608722	2.14E-92	0.499	0.570	-0.071	*FAM180B*	3.35E-10	-0.031
cg04940329	17	39093054	2.14E-92	0.309	0.406	-0.096	*KRT23*	3.26E-16	-0.054
cg09734761	16	85569506	2.17E-92	0.436	0.515	-0.079	NA	3.75E-09	-0.031
cg24323958	1	108741884	2.24E-92	0.280	0.364	-0.084	*SLC25A24*	7.86E-14	-0.042
cg10593922	10	30711382	2.27E-92	0.339	0.430	-0.091	NA	1.99E-04	-0.023
cg01135648	4	41983611	2.76E-92	0.456	0.536	-0.081	*DCAF4L1*	1.08E-05	-0.022
cg00411595	9	136339349	2.76E-92	0.240	0.296	-0.055	*SLC2A6*	1.36E-09	-0.024
cg07525077	14	21359943	2.87E-92	0.297	0.387	-0.089	*RNASE3*	3.35E-10	-0.037
cg14016236	3	171792545	2.91E-92	0.347	0.430	-0.083	*FNDC3B*	1.62E-11	-0.038
cg05758467	2	240035894	2.96E-92	0.402	0.489	-0.086	*HDAC4*	1.67E-13	-0.045
cg04399899	4	160189254	3.06E-92	0.321	0.407	-0.086	*RAPGEF2*	5.33E-08	-0.033
cg03509949	12	56236869	3.21E-92	0.469	0.541	-0.073	*MMP19*	3.26E-17	-0.042
cg16312968	6	6900945	3.28E-92	0.252	0.327	-0.075	NA	1.85E-10	-0.033
cg24058365	1	110923328	3.59E-92	0.340	0.427	-0.087	*SLC16A4*	6.16E-06	-0.026
cg01449715	13	51491406	3.62E-92	0.217	0.289	-0.072	*RNASEH2B*	2.47E-09	-0.031
**cg15690542**	1	151172905	3.66E-92	0.739	0.679	0.060	*PIP5K1A*	0.096	0.007
cg25367558	6	41840102	4.06E-92	0.533	0.626	-0.093	*USP49*	2.98E-07	-0.030
cg14933494	7	2661902	4.34E-92	0.296	0.354	-0.058	NA	9.86E-20	-0.032
cg10058766	1	12186079	4.59E-92	0.294	0.366	-0.072	*TNFRSF8*	1.21E-06	-0.024
cg23213327	2	7016509	4.69E-92	0.395	0.497	-0.102	*RSAD2*	4.04E-17	-0.052
cg09115713	16	88832476	4.74E-92	0.836	0.762	0.074	*PIEZO1*	1.12E-04	0.019
cg14642045	12	109538736	5.16E-92	0.260	0.337	-0.077	*UNG*	0.004	-0.015
cg07493197	17	53573969	5.38E-92	0.263	0.352	-0.088	NA	1.89E-19	-0.052
cg01556552	1	10695686	5.39E-92	0.464	0.557	-0.093	NA	9.01E-06	-0.027
cg21228270	9	133871033	5.40E-92	0.261	0.325	-0.064	NA	5.63E-13	-0.030
cg05071334	8	86195487	5.41E-92	0.391	0.468	-0.077	*CA13*	4.33E-08	-0.029
cg11147278	10	34062234	5.93E-92	0.226	0.303	-0.077	NA	4.29E-08	-0.030
cg16764778	15	41061410	5.99E-92	0.278	0.339	-0.061	*DNAJC17*	2.41E-07	-0.021
cg20443278	17	77962098	6.09E-92	0.673	0.623	0.050	*TBC1D16*	1.35E-10	0.021
cg02307823	10	89675901	6.14E-92	0.384	0.480	-0.095	*PTEN*	1.10E-05	-0.027
cg27050111	16	8738240	6.20E-92	0.288	0.360	-0.072	*METTL22*	1.20E-06	-0.025
cg11071202	14	88462554	6.36E-92	0.268	0.340	-0.073	NA	7.99E-07	-0.025
cg07879785	13	21095712	6.48E-92	0.511	0.585	-0.074	*CRYL1*	1.47E-08	-0.027
cg22115126	22	30283578	6.55E-92	0.250	0.323	-0.073	*MTMR3*	1.54E-06	-0.025
cg20062681	11	94988642	8.13E-92	0.624	0.693	-0.070	NA	6.07E-16	-0.035
cg07675031	11	47399893	8.86E-92	0.269	0.347	-0.078	*SPI1*	1.41E-06	-0.024
cg13471990	10	97515222	9.06E-92	0.236	0.309	-0.073	*ENTPD1*	1.18E-06	-0.026
cg15742777	2	55339218	9.25E-92	0.182	0.238	-0.057	NA	6.13E-19	-0.033
cg00067720	5	67521141	9.52E-92	0.262	0.346	-0.084	*PIK3R1*	1.00E-11	-0.039
cg22250531	12	2030200	1.08E-91	0.190	0.248	-0.058	NA	9.23E-11	-0.026
cg25750408	19	44159698	1.12E-91	0.598	0.661	-0.063	*PLAUR*	4.76E-11	-0.027
cg27592794	10	74454766	1.14E-91	0.216	0.284	-0.068	*MCU*	3.82E-09	-0.026
cg03381237	7	155446645	1.30E-91	0.398	0.475	-0.077	*RBM33*	1.33E-06	-0.026
cg00036976	16	85343281	1.30E-91	0.219	0.274	-0.055	NA	4.10E-14	-0.028
cg00450651	5	139485902	1.31E-91	0.228	0.296	-0.068	NA	1.12E-25	-0.051
cg16389209	10	63809121	1.40E-91	0.745	0.674	0.070	*ARID5B*	4.67E-10	0.027
cg14564351	6	135644405	1.41E-91	0.247	0.329	-0.082	*AHI1*	4.93E-08	-0.031
cg00701064	4	6280414	1.48E-91	0.295	0.363	-0.068	*WFS1*	1.99E-05	-0.019
cg14609407	20	43883172	1.54E-91	0.323	0.410	-0.087	*SLPI*	6.08E-06	-0.026
cg04304802	10	73499965	1.54E-91	0.207	0.275	-0.068	*CDH23*	2.94E-12	-0.035
cg14586500	9	130697656	1.61E-91	0.262	0.342	-0.081	*DPM2*	1.22E-14	-0.045
cg02979487	4	88014081	1.68E-91	0.241	0.300	-0.059	*AFF1*	5.16E-06	-0.019
cg12690978	12	8801526	1.68E-91	0.820	0.761	0.059	*MFAP5*	3.61E-07	0.020
cg02049955	11	118045910	1.75E-91	0.390	0.457	-0.068	*SCN2B*	2.01E-11	-0.032
cg01731783	14	74211788	1.87E-91	0.716	0.663	0.053	*C14orf43*	2.41E-04	0.013
cg03182688	17	33825300	1.93E-91	0.284	0.358	-0.075	NA	3.85E-11	-0.032
cg16815882	1	35908609	2.06E-91	0.305	0.365	-0.060	*KIAA0319L*	0.021	-0.010
cg05492904	15	51604503	2.08E-91	0.440	0.526	-0.086	*CYP19A1*	8.47E-07	-0.029
cg13937155	17	1548881	2.17E-91	0.296	0.377	-0.081	*SCARF1*	9.68E-13	-0.041
cg00956142	1	28765031	2.28E-91	0.259	0.343	-0.084	*PHACTR4*	4.61E-15	-0.044
cg07790752	1	147101904	2.32E-91	0.394	0.495	-0.101	NA	2.19E-07	-0.031
cg20367388	7	5258485	2.38E-91	0.316	0.419	-0.103	*WIPI2*	1.51E-09	-0.045
cg08659421	16	3117862	2.39E-91	0.742	0.682	0.060	*IL32*	2.87E-14	0.030
cg16374333	1	157103641	2.40E-91	0.816	0.754	0.063	*ETV3*	8.52E-10	0.026
cg23314364	8	29230998	2.44E-91	0.308	0.395	-0.087	NA	0.002	-0.019
cg19222525	20	62492220	2.66E-91	0.273	0.325	-0.052	*ABHD16B*	5.26E-09	-0.019
cg23847017	1	28764854	2.68E-91	0.231	0.309	-0.078	*PHACTR4*	7.46E-09	-0.032
cg19528654	11	67251154	2.79E-91	0.825	0.772	0.053	*AIP*	9.80E-09	0.021
cg03035167	2	201336269	2.90E-91	0.757	0.684	0.073	*SPATS2L*	1.07E-04	0.016

cg01445100	16	88103339	2.92E-91	0.757	0.700	0.057	*BANP*	2.85E-10	0.026
cg14057383	2	100636602	3.03E-91	0.570	0.640	-0.071	*AFF3*	7.01E-07	-0.022
cg02276314	1	9301104	3.16E-91	0.592	0.658	-0.066	*H6PD*	5.93E-08	-0.025
cg16151538	20	33676388	3.35E-91	0.549	0.615	-0.066	*TRPC4AP*	8.42E-15	-0.033
cg16001689	6	135042205	3.39E-91	0.279	0.359	-0.080	NA	2.90E-19	-0.049
cg12332674	6	34252820	4.22E-91	0.182	0.234	-0.052	NA	5.49E-04	-0.012
cg25801976	8	48648112	4.27E-91	0.260	0.335	-0.076	*KIAA0146*	1.23E-04	-0.019
cg14918744	8	23018464	4.92E-91	0.790	0.733	0.057	*TNFRSF10D*	6.59E-08	0.023
cg06905453	19	14089284	4.93E-91	0.221	0.285	-0.064	*RFX1*	6.37E-11	-0.028
cg10228555	16	3088480	5.41E-91	0.330	0.399	-0.070	*LOC10012877(*	2.57E-06	-0.020
cg11485152	3	3171664	5.74E-91	0.342	0.421	-0.078	*TRNT1*	1.36E-13	-0.042
cg02193956	4	103680201	6.12E-91	0.319	0.413	-0.093	*MANBA*	5.64E-08	-0.035
cg04204452	17	1479213	6.25E-91	0.380	0.449	-0.069	*SLC43A2*	1.57E-06	-0.021
cg27449572	10	100024278	6.27E-91	0.254	0.335	-0.081	*LOXL4*	1.02E-04	-0.022
cg05398700	14	102677141	6.27E-91	0.439	0.545	-0.106	*WDR20*	2.85E-10	-0.041
cg15698299	3	52233019	6.31E-91	0.249	0.305	-0.056	*ALAS1*	3.78E-10	-0.023
cg24591861	15	66697173	6.82E-91	0.246	0.323	-0.077	*MAP2K1*	2.92E-05	-0.021
cg00796963	6	84936558	6.94E-91	0.369	0.449	-0.079	*KIAA1009*	8.15E-05	-0.022
cg05160234	11	118214762	7.09E-91	0.734	0.668	0.066	*CD3G*	4.62E-13	0.032
cg14325025	2	102608155	7.35E-91	0.219	0.289	-0.070	*IL1R2*	1.06E-11	-0.035
cg07019638	11	64635774	7.69E-91	0.855	0.798	0.057	*EHD1*	1.15E-13	0.031
cg25760227	8	130691556	8.18E-91	0.335	0.414	-0.079	NA	1.91E-17	-0.047
cg03677056	17	33817217	8.89E-91	0.336	0.410	-0.073	NA	3.22E-10	-0.030
cg10958452	1	44114346	8.99E-91	0.345	0.441	-0.095	*KDM4A*	2.93E-04	-0.023
cg10145196	6	30647649	9.24E-91	0.782	0.720	0.062	*PPP1R18*	9.31E-06	0.020
cg18315380	7	1095666	9.40E-91	0.290	0.356	-0.066	*C7orf50*	2.19E-04	-0.015
cg20934416	5	17444401	9.49E-91	0.432	0.538	-0.107	NA	0.044	-0.013
cg07248017	4	8230689	9.70E-91	0.727	0.670	0.057	*SH3TC1*	1.67E-15	0.030
cg01766396	2	198436483	9.82E-91	0.364	0.452	-0.087	*RFTN2*	2.00E-04	-0.021
cg05111779	14	72990359	1.02E-90	0.329	0.421	-0.092	*RGS6*	8.96E-09	-0.035
cg20162696	8	102167694	1.05E-90	0.245	0.322	-0.077	NA	1.62E-09	-0.032
cg26661640	11	64458846	1.13E-90	0.802	0.744	0.058	*NRXN2*	0.027	0.008
cg24525364	1	35730504	1.15E-90	0.182	0.237	-0.055	NA	1.02E-11	-0.028
cg01393945	5	10457034	1.16E-90	0.352	0.435	-0.083	*ROPN1L*	8.53E-04	-0.020
cg15621731	19	5074616	1.19E-90	0.333	0.415	-0.082	*KDM4B*	1.05E-08	-0.031
cg12626076	15	92399195	1.75E-90	0.836	0.782	0.054	*SLCO3A1*	0.034	0.008
cg10627428	3	122514814	1.80E-90	0.223	0.292	-0.069	*DIRC2*	1.29E-09	-0.028
cg18044543	10	91144795	1.92E-90	0.298	0.373	-0.076	*IFIT1B*	3.28E-08	-0.029
cg05641903	19	7767584	1.97E-90	0.370	0.443	-0.073	*FCER2*	4.65E-13	-0.034
cg08874645	10	73093425	2.11E-90	0.234	0.287	-0.053	*SLC29A3*	5.96E-06	-0.015
cg09080173	8	22605334	2.40E-90	0.216	0.275	-0.059	*PEBP4*	2.10E-14	-0.032
cg06101180	2	157185558	2.41E-90	0.357	0.417	-0.060	*NR4A2*	1.37E-10	-0.026
cg17208953	12	108054842	2.44E-90	0.502	0.582	-0.081	NA	1.09E-08	-0.032
cg05265081	15	64723065	2.61E-90	0.276	0.344	-0.069	*TRIP4*	2.56E-09	-0.028
cg00446046	3	187686918	2.67E-90	0.387	0.482	-0.095	NA	0.004	-0.017
cg00591868	19	19729048	2.72E-90	0.700	0.627	0.074	*PBX4*	1.40E-04	0.016
cg14575356	6	130013903	2.82E-90	0.455	0.552	-0.097	*ARHGAP18*	0.002	-0.019
cg15986644	10	516683	2.85E-90	0.871	0.819	0.052	*DIP2C*	5.28E-07	0.019
cg14897188	17	6917185	2.87E-90	0.749	0.692	0.056	*RNASEK*	7.84E-04	0.013
cg25044876	22	43041146	2.91E-90	0.348	0.434	-0.086	*CYB5R3*	3.31E-11	-0.040
cg01735277	15	75077691	2.96E-90	0.686	0.614	0.072	*CSK*	1.54E-05	0.020
cg15407162	6	28192457	3.17E-90	0.296	0.382	-0.086	*ZNF193*	2.57E-18	-0.052
cg08367804	16	3639160	3.39E-90	0.420	0.492	-0.072	*SLX4*	1.14E-06	-0.022
cg06078469	17	55335955	3.79E-90	0.241	0.295	-0.054	*MSI2*	4.28E-14	-0.027
cg15880738	11	118215112	3.94E-90	0.760	0.690	0.070	*CD3G*	1.27E-08	0.026
cg21284779	1	155910526	3.95E-90	0.337	0.392	-0.056	*RXFP4*	1.18E-13	-0.024
cg07477602	1	56961319	4.08E-90	0.362	0.458	-0.096	*PPAP2B*	2.54E-13	-0.049
cg17775490	20	45179354	4.13E-90	0.229	0.316	-0.086	*OCSTAMP*	7.32E-06	-0.028
cg26096752	5	66530611	4.26E-90	0.256	0.321	-0.065	NA	7.20E-12	-0.031
cg26297462	1	45292204	4.36E-90	0.257	0.311	-0.054	*PTCH2*	2.29E-11	-0.024
cg22406102	6	139947852	4.44E-90	0.137	0.190	-0.052	NA	5.20E-13	-0.028
cg02566391	1	67805528	4.51E-90	0.788	0.730	0.059	*IL12RB2*	2.82E-05	0.017
cg18081863	19	50015527	4.61E-90	0.317	0.392	-0.075	*FCGRT*	2.18E-09	-0.028
cg07613391	3	30708746	4.79E-90	0.735	0.673	0.061	*TGFBR2*	1.03E-05	0.019
cg03836521	16	30721270	5.10E-90	0.385	0.437	-0.052	*SRCAP*	2.46E-09	-0.019
cg25104397	10	104535920	5.40E-90	0.251	0.333	-0.083	*WBP1L*	6.92E-06	-0.025
cg03776194	16	88770966	5.42E-90	0.527	0.457	0.070	*RNF166*	2.67E-11	0.030
cg00645579	11	617140	6.07E-90	0.332	0.393	-0.061	*CDHR5*	8.36E-04	-0.014
cg06502279	16	474467	6.21E-90	0.456	0.559	-0.103	*RAB11FIP3*	6.09E-04	-0.025
cg15891546	8	142179908	6.36E-90	0.501	0.612	-0.111	*DENND3*	2.24E-08	-0.042
cg02927448	12	100532162	6.55E-90	0.281	0.352	-0.071	*UHRF1BP1L*	4.00E-11	-0.034
cg15042302	7	47981215	6.76E-90	0.340	0.431	-0.091	*PKD1L1*	3.55E-05	-0.026
cg18404652	8	42009622	6.79E-90	0.687	0.622	0.065	*AP3M2*	5.32E-09	0.025

cg26724841	7	5816628	7.20E-90	0.786	0.727	0.059	*RNF216*	0.002	0.012
cg07744430	12	132345502	7.21E-90	0.416	0.473	-0.057	NA	1.50E-18	-0.035
cg25338454	12	26900022	7.54E-90	0.538	0.614	-0.077	*ITPR2*	4.90E-10	-0.032
cg08399444	12	13248548	7.68E-90	0.440	0.539	-0.098	*GSG1*	5.52E-05	-0.026
cg24012880	11	44880910	7.68E-90	0.364	0.465	-0.101	*TSPAN18*	7.63E-11	-0.045
cg01226748	12	133277966	7.92E-90	0.270	0.334	-0.064	*PXMP2*	8.62E-06	-0.021
cg07584494	19	5074758	8.00E-90	0.428	0.494	-0.065	*KDM4B*	3.63E-06	-0.019
cg02185182	7	2185550	8.25E-90	0.420	0.509	-0.089	*MAD1L1*	1.44E-06	-0.024
cg07586235	1	200385219	8.45E-90	0.337	0.423	-0.086	NA	3.51E-06	-0.026
cg25194194	12	69143431	8.61E-90	0.432	0.495	-0.064	*SLC35E3*	1.18E-08	-0.024
cg16402415	15	57021851	8.88E-90	0.374	0.463	-0.089	*ZNF280D*	3.46E-09	-0.034
cg01500402	16	2256716	9.50E-90	0.197	0.247	-0.050	*MLST8*	1.97E-13	-0.026
cg01367992	1	160766535	1.01E-89	0.838	0.783	0.056	*LY9*	9.54E-10	0.025
cg22044342	17	3819339	1.06E-89	0.367	0.453	-0.086	*P2RX1*	5.63E-06	-0.026
cg25179876	10	33483109	1.10E-89	0.296	0.375	-0.079	*NRP1*	3.57E-13	-0.040
cg23051349	14	104094779	1.30E-89	0.192	0.265	-0.072	*KLC1*	2.78E-06	-0.024
cg21870229	21	45145841	1.32E-89	0.240	0.315	-0.075	*PDXK*	2.32E-04	-0.019
cg16826777	17	43376882	1.33E-89	0.395	0.451	-0.056	*MAP3K14*	3.83E-16	-0.033
cg22544881	7	130712346	1.39E-89	0.238	0.302	-0.065	*FLJ43663*	8.27E-14	-0.033
cg15816464	17	2026533	1.41E-89	0.265	0.328	-0.063	*SMG6*	6.00E-08	-0.023
cg26962618	15	70767183	1.42E-89	0.256	0.341	-0.084	NA	1.71E-08	-0.033
cg08161480	1	226114565	1.48E-89	0.314	0.385	-0.072	NA	0.005	-0.014
cg26828017	16	22409023	1.52E-89	0.369	0.459	-0.090	NA	2.06E-07	-0.031
cg14419046	9	90043268	1.67E-89	0.358	0.438	-0.080	NA	1.81E-06	-0.025
cg01408486	5	139027024	1.73E-89	0.232	0.288	-0.056	*CXXC5*	6.83E-15	-0.027
cg09351315	17	77775823	1.79E-89	0.276	0.337	-0.062	NA	1.13E-12	-0.029
cg08279189	6	28875370	1.89E-89	0.389	0.495	-0.107	*TRIM27*	1.47E-06	-0.033
cg14171527	21	44473840	1.90E-89	0.354	0.445	-0.091	*CBS*	8.26E-11	-0.039
cg15078838	2	28114349	1.99E-89	0.198	0.277	-0.079	*RBKS*	4.74E-09	-0.035
cg18546006	13	50569769	2.05E-89	0.202	0.272	-0.070	*DLEU2*	2.23E-14	-0.037
cg17158941	7	1073255	2.14E-89	0.816	0.765	0.052	*C7orf50*	2.49E-07	0.019
cg01288337	14	92991035	2.18E-89	0.277	0.344	-0.067	*RIN3*	1.02E-06	-0.022
cg19356022	1	154943932	2.20E-89	0.371	0.472	-0.101	*SHC1*	3.45E-05	-0.028
cg13740985	9	80930413	2.31E-89	0.290	0.372	-0.082	*PSAT1*	3.78E-08	-0.033
cg23679819	18	13730665	2.33E-89	0.665	0.584	0.081	*RNMT*	8.57E-13	0.039
cg09293816	19	50015532	2.38E-89	0.347	0.416	-0.069	*FCGRT*	1.88E-10	-0.027
cg23599820	17	73456199	2.47E-89	0.333	0.387	-0.054	*KIAA0195*	2.39E-12	-0.026
cg27614309	16	85478580	2.48E-89	0.785	0.733	0.052	NA	2.23E-14	0.028
cg25467634	10	49684658	2.64E-89	0.231	0.286	-0.055	*ARHGAP22*	9.88E-13	-0.026
cg17679427	10	35480749	2.70E-89	0.329	0.408	-0.079	*CREM*	4.01E-13	-0.039
cg26786407	1	3415673	2.76E-89	0.813	0.757	0.056	*MEGF6*	9.65E-05	0.013
cg03956820	17	80190154	2.82E-89	0.362	0.434	-0.072	*SLC16A3*	1.69E-16	-0.033
cg05216056	6	28887836	2.85E-89	0.546	0.635	-0.088	*TRIM27*	7.74E-06	-0.026
cg07744502	11	93271083	3.00E-89	0.243	0.297	-0.054	*C11orf75*	9.03E-17	-0.030
cg16962442	13	114828455	3.03E-89	0.370	0.445	-0.075	*RASA3*	6.80E-12	-0.034
cg24157392	3	112217973	3.35E-89	0.698	0.626	0.071	*BTLA*	5.64E-13	0.035
cg21872009	17	26573693	3.53E-89	0.708	0.656	0.053	NA	0.006	0.010
cg14565465	9	74298933	3.58E-89	0.295	0.368	-0.073	*TMEM2*	1.33E-07	-0.028
cg23530553	17	46622536	3.63E-89	0.575	0.648	-0.074	*HOXB2*	3.47E-19	-0.047
cg16588163	3	190251464	4.17E-89	0.287	0.358	-0.070	*IL1RAP*	5.06E-12	-0.033
cg04020309	1	247496094	4.39E-89	0.348	0.429	-0.080	*ZNF496*	2.10E-16	-0.045
cg09447811	3	121972621	4.71E-89	0.352	0.439	-0.087	*CASR*	9.03E-11	-0.039
cg01088404	12	48214523	4.71E-89	0.292	0.387	-0.095	*HDAC7*	1.01E-13	-0.049
cg01521397	20	60590872	4.72E-89	0.390	0.490	-0.100	*TAF4*	4.87E-06	-0.031
cg14714629	7	99495452	5.48E-89	0.285	0.351	-0.066	*TRIM4*	8.00E-07	-0.024
cg15209885	17	77753199	5.49E-89	0.265	0.319	-0.054	*CBX2*	6.30E-04	-0.011
cg01860774	14	64969374	6.44E-89	0.810	0.753	0.056	*ZBTB25*	2.17E-10	0.025
cg12655416	17	38077870	6.54E-89	0.175	0.225	-0.051	*ORMDL3*	4.55E-07	-0.018
cg13185046	6	32165321	6.69E-89	0.308	0.371	-0.063	*NOTCH4*	1.28E-15	-0.037
cg12682870	6	43766804	6.70E-89	0.226	0.290	-0.063	NA	5.94E-16	-0.036
cg18875357	14	105391263	6.93E-89	0.220	0.271	-0.051	*PLD4*	6.07E-06	-0.015
cg23090046	14	104094619	7.08E-89	0.471	0.525	-0.054	*KLC1*	8.18E-07	-0.017
cg09183124	1	173092323	7.14E-89	0.363	0.460	-0.097	NA	1.45E-07	-0.034
cg10800620	2	196398826	7.16E-89	0.741	0.673	0.068	NA	3.50E-09	0.028
cg00255699	1	40307257	7.25E-89	0.774	0.708	0.065	*TRIT1*	2.07E-06	0.022
cg19504184	17	1170806	7.30E-89	0.267	0.324	-0.057	NA	2.91E-20	-0.034
cg00154119	15	68174054	7.34E-89	0.390	0.451	-0.061	NA	1.87E-08	-0.025
cg04505750	1	12774815	7.39E-89	0.266	0.341	-0.075	*AADACL3*	3.74E-06	-0.026
cg11554335	11	57336941	7.79E-89	0.460	0.536	-0.077	*UBE2L6*	4.14E-08	-0.030
cg23415995	4	128704651	7.83E-89	0.386	0.473	-0.087	*HSPA4L*	1.94E-05	-0.025
cg16329896	7	47515060	8.18E-89	0.296	0.370	-0.074	*TNS3*	6.49E-09	-0.028
cg10351914	17	19290690	8.57E-89	0.224	0.278	-0.054	*MFAP4*	8.79E-06	-0.018
cg13765621	1	158149228	8.65E-89	0.249	0.326	-0.078	*CD1D*	1.56E-11	-0.041

cg15501219	3	44375962	9.13E-89	0.394	0.462	-0.068	NA	8.33E-19	-0.042
cg05824218	17	38499096	9.30E-89	0.526	0.594	-0.068	*RARA*	3.95E-11	-0.029
cg08937107	16	3136857	9.64E-89	0.271	0.335	-0.065	NA	2.23E-08	-0.024
cg21852792	2	71678463	9.66E-89	0.354	0.445	-0.091	NA	2.06E-06	-0.028
cg01797169	12	107711489	9.76E-89	0.275	0.355	-0.080	*BTBD11*	8.17E-14	-0.042
cg25784280	18	32289350	1.02E-88	0.318	0.404	-0.086	*DTNA*	2.35E-13	-0.044
cg25139649	1	2165579	1.04E-88	0.308	0.405	-0.097	*SKI*	6.27E-08	-0.036
cg20628942	13	114171501	1.06E-88	0.411	0.488	-0.077	*TMCO3*	2.91E-07	-0.028
cg01300096	6	33384490	1.07E-88	0.704	0.650	0.054	*CUTA*	9.14E-07	0.017
cg13371705	12	2452955	1.11E-88	0.354	0.440	-0.086	*CACNA1C*	9.78E-13	-0.043
cg01905773	17	79297618	1.12E-88	0.412	0.489	-0.077	*TMEM105*	1.86E-09	-0.030
cg11384744	7	16794623	1.23E-88	0.292	0.370	-0.078	*TSPAN13*	2.83E-11	-0.038
cg08806632	19	13214412	1.23E-88	0.194	0.264	-0.070	*LYL1*	5.39E-13	-0.035
cg17222829	11	70433293	1.36E-88	0.336	0.429	-0.094	*SHANK2*	2.49E-10	-0.042
cg17463083	17	62153284	1.52E-88	0.288	0.375	-0.086	*ERN1*	2.56E-04	-0.023
cg03919488	12	63179691	1.57E-88	0.370	0.444	-0.074	*PPM1H*	3.67E-07	-0.026
cg20317748	15	40633124	1.86E-88	0.348	0.439	-0.092	*C15orf52*	1.36E-09	-0.038
cg07920381	12	132303685	1.87E-88	0.468	0.553	-0.085	NA	3.40E-11	-0.038
cg20672711	3	110965888	1.98E-88	0.423	0.514	-0.091	NA	6.58E-09	-0.037
cg10927461	2	122530159	2.00E-88	0.254	0.330	-0.077	NA	1.91E-12	-0.040
cg13565499	9	139592589	2.11E-88	0.243	0.322	-0.079	NA	2.02E-14	-0.046
cg05573378	15	42876795	2.15E-88	0.457	0.516	-0.058	*STARD9*	4.46E-12	-0.028
cg09914304	10	72362292	2.18E-88	0.606	0.552	0.054	*PRF1*	5.52E-10	0.022
cg10512278	16	68027839	2.38E-88	0.310	0.365	-0.055	*DPEP2*	7.73E-10	-0.021
cg07630255	15	75185989	2.49E-88	0.820	0.767	0.053	*MPI*	5.23E-11	0.025
cg20488123	6	20689061	2.50E-88	0.713	0.643	0.070	*CDKAL1*	4.94E-09	0.028
cg14117392	5	139017942	2.53E-88	0.306	0.371	-0.065	NA	1.28E-04	-0.018
cg07895132	17	33825172	2.55E-88	0.346	0.430	-0.084	NA	6.75E-04	-0.018
cg21661768	2	106512578	2.62E-88	0.737	0.676	0.061	NA	4.62E-08	0.024
cg21278103	20	2928233	2.63E-88	0.325	0.402	-0.077	*PTPRA*	5.06E-13	-0.038
cg13912964	7	100867281	2.64E-88	0.535	0.614	-0.079	*ZNHIT1*	9.50E-04	-0.019
cg14715778	17	73840989	3.02E-88	0.180	0.233	-0.053	*UNC13D*	2.83E-16	-0.029
cg11411220	10	35630062	3.05E-88	0.313	0.386	-0.073	*CCNY*	7.65E-10	-0.029
cg17879101	10	126329354	3.06E-88	0.264	0.334	-0.070	*FAM53B*	5.56E-04	-0.018
cg22900266	2	65089000	3.07E-88	0.403	0.471	-0.068	NA	1.27E-08	-0.024
cg13051977	1	45148001	3.20E-88	0.288	0.365	-0.077	*C1orf228*	1.35E-08	-0.032
cg13719443	6	35700382	3.22E-88	0.162	0.217	-0.055	*LOC285847*	2.66E-06	-0.019
cg19069360	12	1922058	3.29E-88	0.387	0.486	-0.099	*CACNA2D4*	7.17E-04	-0.020
cg07646791	6	166667648	3.37E-88	0.194	0.267	-0.073	NA	2.25E-06	-0.025
cg16141752	5	133802474	3.51E-88	0.246	0.315	-0.069	NA	4.52E-07	-0.025
cg12213811	12	122251152	3.77E-88	0.558	0.631	-0.073	*SETD1B*	7.38E-06	-0.022
**cg11153071**	17	78748077	3.84E-88	0.453	0.553	-0.100	*RPTOR*	0.054	-0.012
cg13461622	1	25291385	3.93E-88	0.795	0.735	0.061	*RUNX3*	1.45E-07	0.023
cg08542715	17	34217960	4.16E-88	0.783	0.718	0.065	NA	7.60E-05	0.017
cg03094134	6	30297174	4.34E-88	0.387	0.464	-0.078	*TRIM39*	7.23E-05	-0.021
cg22135566	12	124416315	4.42E-88	0.754	0.694	0.061	*DNAH10*	3.54E-10	0.026
cg08356637	11	8385767	4.71E-88	0.315	0.395	-0.079	NA	1.01E-24	-0.056
cg26750893	2	38043481	4.97E-88	0.528	0.616	-0.088	NA	4.54E-19	-0.052
cg04790874	19	42381950	5.21E-88	0.326	0.399	-0.073	*CD79A*	3.61E-07	-0.026
cg00401972	16	66400404	5.38E-88	0.406	0.470	-0.064	*CDH5*	3.23E-13	-0.031
cg15285436	17	59226714	5.39E-88	0.370	0.471	-0.102	*BCAS3*	5.24E-09	-0.037
**cg09572125**	6	33400477	5.43E-88	0.211	0.300	-0.089	*SYNGAP1*	0.143	-0.010
cg03962678	11	118398094	5.66E-88	0.286	0.344	-0.059	*TTC36*	5.03E-07	-0.021
cg16239536	19	1079617	6.06E-88	0.747	0.678	0.069	*HMHA1*	3.27E-09	0.028
**cg17554464**	12	56864388	6.25E-88	0.382	0.464	-0.081	*SPRYD4*	0.090	-0.009
cg00861646	4	160026608	6.57E-88	0.290	0.369	-0.079	NA	6.08E-08	-0.030
cg08269188	12	54655894	6.70E-88	0.187	0.248	-0.061	*CBX5*	5.17E-18	-0.038
cg00533990	18	3255878	6.89E-88	0.357	0.432	-0.075	*MYL12A*	5.05E-07	-0.027
cg27295342	3	195849368	6.97E-88	0.202	0.269	-0.066	NA	0.006	-0.012
cg20040747	15	74715105	7.39E-88	0.291	0.355	-0.064	*SEMA7A*	1.60E-12	-0.029
cg07063912	2	238598832	7.62E-88	0.294	0.378	-0.085	*LRRFIP1*	3.37E-11	-0.040
cg15593510	1	158369112	7.90E-88	0.826	0.773	0.053	*OR10T2*	4.39E-09	0.022
cg14414903	2	240171712	7.93E-88	0.292	0.376	-0.084	*HDAC4*	0.020	-0.014
cg26561681	1	21376633	8.31E-88	0.330	0.428	-0.099	*EIF4G3*	1.31E-05	-0.032
cg18758433	17	78623601	8.51E-88	0.753	0.682	0.071	*RPTOR*	5.95E-18	0.042
cg09868299	11	69259957	8.59E-88	0.397	0.458	-0.060	NA	2.33E-05	-0.016
cg05252264	19	55385587	8.77E-88	0.280	0.376	-0.096	*FCAR*	2.14E-08	-0.040
cg21814550	14	53170147	8.80E-88	0.425	0.516	-0.091	NA	2.90E-05	-0.024
cg06708720	12	1099075	8.84E-88	0.457	0.521	-0.065	*ERC1*	8.07E-29	-0.055
cg24476449	17	25799212	8.91E-88	0.740	0.680	0.060	*KSR1*	2.80E-07	0.022
cg02217815	22	36890079	9.42E-88	0.179	0.234	-0.054	*FOXRED2*	0.003	-0.011
cg15227982	10	104535854	9.77E-88	0.253	0.320	-0.067	*WBP1L*	7.96E-05	-0.017
cg22274234	12	109888858	9.86E-88	0.282	0.343	-0.060	*KCTD10*	1.17E-12	-0.028

cg21887193	7	150786082	9.95E-88	0.205	0.291	-0.086	*AGAP3*	1.49E-06	-0.029
cg24723883	19	2608495	1.02E-87	0.816	0.753	0.063	*GNG7*	8.33E-07	0.023
cg20849109	8	1954777	1.04E-87	0.284	0.365	-0.081	*KBTBD11*	5.48E-11	-0.035
cg18964319	6	36665554	1.11E-87	0.256	0.337	-0.082	*RAB44*	1.31E-08	-0.030
cg15744128	2	27850964	1.16E-87	0.314	0.395	-0.081	*GPN1*	1.28E-05	-0.026
cg10499832	17	41450195	1.21E-87	0.357	0.432	-0.074	*LOC10013058.*	4.23E-06	-0.024
cg01526089	17	3819390	1.22E-87	0.317	0.373	-0.056	*P2RX1*	2.46E-05	-0.015
cg02394698	4	86594376	1.25E-87	0.260	0.329	-0.068	*ARHGAP24*	3.34E-10	-0.032
cg23036852	21	44809983	1.40E-87	0.217	0.281	-0.064	NA	1.25E-11	-0.032
cg03161309	17	6563585	1.41E-87	0.256	0.332	-0.076	*ALOX15P1*	1.95E-07	-0.029
cg25588844	2	10037561	1.47E-87	0.293	0.372	-0.079	*TAF1B*	1.03E-14	-0.042
cg00711072	17	46669489	1.49E-87	0.254	0.345	-0.091	*HOXB-AS3*	5.59E-06	-0.030
cg10914115	6	31704828	1.51E-87	0.247	0.300	-0.053	*CLIC1*	4.43E-13	-0.027
cg06379435	19	3344273	1.62E-87	0.351	0.449	-0.099	NA	0.005	-0.018
cg01357892	3	126191181	1.73E-87	0.182	0.232	-0.050	*ZXDC*	7.13E-15	-0.027
cg12959048	11	73096162	1.79E-87	0.346	0.416	-0.070	*RELT*	1.59E-16	-0.040
cg00637104	20	1785303	1.82E-87	0.399	0.483	-0.084	NA	2.36E-06	-0.025
cg25389087	18	74824413	1.87E-87	0.432	0.534	-0.102	*MBP*	4.71E-04	-0.023
cg07684519	12	29303184	1.88E-87	0.199	0.281	-0.083	NA	8.11E-09	-0.036
cg14082886	11	35164485	1.93E-87	0.571	0.487	0.085	*CD44*	8.46E-13	0.042
cg26008365	1	153958797	2.03E-87	0.184	0.261	-0.077	*RAB13*	0.006	-0.016
cg14596967	11	113956944	2.10E-87	0.421	0.496	-0.076	*ZBTB16*	3.44E-12	-0.037
cg05021029	10	76947956	2.38E-87	0.811	0.755	0.056	NA	4.80E-05	0.015
cg27255275	11	129766154	2.41E-87	0.380	0.468	-0.089	*NFRKB*	3.89E-11	-0.037
**cg26105956**	1	154471433	2.44E-87	0.423	0.523	-0.101	*SHE*	0.091	-0.011
cg07769015	8	142238770	2.59E-87	0.482	0.565	-0.083	*SLC45A4*	1.65E-08	-0.031
cg06519434	3	38664257	2.60E-87	0.748	0.689	0.060	*SCN5A*	0.026	0.008
cg09370867	1	27961680	2.88E-87	0.315	0.387	-0.072	*FGR*	1.30E-04	-0.018
cg07929642	16	89390685	2.94E-87	0.826	0.768	0.058	*LOC10028703(*	4.94E-06	0.018
cg04416898	5	139040055	2.94E-87	0.271	0.336	-0.066	*CXXC5*	5.97E-23	-0.047
cg17387577	12	124864657	3.03E-87	0.297	0.371	-0.075	*NCOR2*	1.11E-07	-0.027
cg00625110	16	53741731	3.19E-87	0.335	0.417	-0.082	*FTO*	2.63E-07	-0.029
cg17113968	15	41061527	3.28E-87	0.237	0.301	-0.064	*DNAJC17*	0.002	-0.014
cg23463608	19	2607757	3.39E-87	0.559	0.475	0.084	*GNG7*	7.85E-04	0.018
cg00760203	17	37254921	3.45E-87	0.223	0.296	-0.072	*PLXDC1*	3.27E-06	-0.022
cg03658236	12	104063640	3.69E-87	0.435	0.533	-0.098	*STAB2*	3.60E-05	-0.026
cg25306006	11	71932630	3.82E-87	0.305	0.371	-0.066	*FOLR2*	5.11E-16	-0.038
cg06303635	10	45958759	3.90E-87	0.394	0.470	-0.076	*MARCH8*	8.05E-04	-0.015
cg24437859	12	7066614	4.32E-87	0.799	0.727	0.072	*PTPN6*	3.62E-06	0.025
cg17036458	17	28037399	4.40E-87	0.390	0.488	-0.098	*SSH2*	9.02E-06	-0.031
cg04346672	14	69229910	4.57E-87	0.374	0.436	-0.062	NA	2.50E-10	-0.028
cg10498502	3	185928164	4.57E-87	0.356	0.427	-0.070	*DGKG*	1.50E-14	-0.038
cg16465939	11	2554410	4.71E-87	0.198	0.249	-0.051	*KCNQ1*	0.004	-0.010
cg01309569	1	5950209	4.88E-87	0.611	0.681	-0.070	*NPHP4*	3.54E-04	-0.016
cg23606023	16	89042948	5.19E-87	0.189	0.239	-0.051	*CBFA2T3*	8.46E-11	-0.023
cg25234117	3	155423168	5.59E-87	0.275	0.342	-0.067	*PLCH1*	6.91E-09	-0.028
cg27355141	17	80190136	6.13E-87	0.261	0.316	-0.055	*SLC16A3*	4.38E-14	-0.027
cg00765705	12	124865130	6.18E-87	0.292	0.383	-0.091	*NCOR2*	0.004	-0.017
cg02997962	20	20036779	6.38E-87	0.337	0.439	-0.102	*CRNKL1*	6.81E-06	-0.034
cg19374752	12	52404151	6.53E-87	0.453	0.513	-0.060	*GRASP*	6.43E-22	-0.039
cg13650740	10	80516517	6.59E-87	0.294	0.352	-0.058	NA	5.02E-08	-0.020
cg16310095	1	155952959	7.09E-87	0.354	0.406	-0.052	NA	1.40E-08	-0.021
cg07212327	8	130947389	7.12E-87	0.457	0.562	-0.105	*FAM49B*	2.77E-04	-0.025
cg03234557	4	99404186	7.45E-87	0.796	0.745	0.051	*TSPAN5*	4.19E-07	0.017
cg20049422	15	26044289	7.49E-87	0.327	0.410	-0.083	*ATP10A*	5.41E-07	-0.030
cg07141504	3	194030706	7.50E-87	0.192	0.247	-0.054	NA	2.93E-12	-0.027
cg17429236	7	150694028	7.87E-87	0.190	0.259	-0.070	*NOS3*	0.032	-0.011
cg26404422	11	128367010	8.49E-87	0.699	0.627	0.073	*ETS1*	1.49E-22	0.052
cg23128584	10	323649	8.86E-87	0.375	0.465	-0.090	*DIP2C*	2.10E-09	-0.036
cg18522931	10	63776828	8.97E-87	0.281	0.362	-0.081	*ARID5B*	6.09E-14	-0.045
cg08091050	4	26208051	1.02E-86	0.481	0.575	-0.093	NA	0.001	-0.019
cg05873568	14	105155145	1.02E-86	0.214	0.271	-0.056	*INF2*	2.38E-08	-0.022
cg13918640	9	94351568	1.02E-86	0.355	0.450	-0.095	NA	2.71E-11	-0.044
cg07133930	10	70821371	1.03E-86	0.271	0.350	-0.079	NA	1.45E-05	-0.025
cg06755438	2	219885614	1.05E-86	0.361	0.451	-0.090	*CCDC108*	2.73E-09	-0.038
cg26305174	7	100463583	1.13E-86	0.341	0.423	-0.083	*TRIP6*	4.76E-09	-0.030
cg14662750	6	15280024	1.15E-86	0.361	0.435	-0.073	*JARID2*	6.94E-20	-0.046
cg05876069	5	150020288	1.20E-86	0.375	0.426	-0.050	*SYNPO*	1.64E-09	-0.019
cg07781197	21	46046362	1.23E-86	0.818	0.766	0.052	*KRTAP10-9*	1.44E-10	0.024
cg02832512	6	22113516	1.26E-86	0.446	0.521	-0.074	*LINC00340*	3.52E-13	-0.036
cg16436762	11	94307971	1.27E-86	0.339	0.430	-0.091	*PIWIL4*	2.69E-08	-0.035
cg11579421	10	134211857	1.29E-86	0.283	0.352	-0.069	*PWWP2B*	5.53E-06	-0.021
cg19092837	1	10271724	1.32E-86	0.447	0.541	-0.094	*KIF1B*	1.82E-04	-0.024

cg08256939	1	112058236	1.32E-86	0.277	0.344	-0.066	*ADORA3*	1.80E-05	-0.019
cg15361231	1	193075191	1.35E-86	0.417	0.507	-0.090	*GLRX2*	7.79E-04	-0.021
cg01869896	1	40420299	1.35E-86	0.523	0.596	-0.073	*MFSD2A*	1.21E-10	-0.031
cg19275653	2	175532338	1.44E-86	0.212	0.284	-0.072	*WIPF1*	7.31E-07	-0.025
cg00324097	17	75446549	1.51E-86	0.556	0.495	0.061	*SEPT9*	0.013	0.010
cg27287167	19	18267904	1.57E-86	0.365	0.416	-0.050	*PIK3R2*	2.71E-13	-0.024
cg10020520	16	30976186	1.66E-86	0.424	0.492	-0.068	*SETD1A*	0.008	-0.012
cg12230709	19	840873	1.67E-86	0.186	0.246	-0.060	*PRTN3*	9.37E-07	-0.022
cg03917473	17	38764244	1.72E-86	0.747	0.680	0.068	NA	5.36E-09	0.027
cg08338281	12	56322667	1.77E-86	0.724	0.653	0.070	*WIBG*	5.82E-12	0.035
cg15939920	17	35457385	1.80E-86	0.352	0.416	-0.064	*ACACA*	1.41E-10	-0.027
cg25199552	1	23350054	1.87E-86	0.392	0.484	-0.092	*KDM1A*	0.003	-0.019
cg02301079	16	11591651	1.87E-86	0.222	0.275	-0.052	NA	2.74E-09	-0.020
cg09855140	13	100004097	1.92E-86	0.809	0.753	0.056	*UBAC2*	3.81E-10	0.025
cg06512263	6	20709867	2.01E-86	0.218	0.283	-0.065	*CDKAL1*	3.27E-08	-0.026
cg07037635	15	55615438	2.04E-86	0.284	0.358	-0.073	*PIGB*	4.64E-12	-0.039
cg04340258	19	3398706	2.10E-86	0.436	0.490	-0.054	*NFIC*	4.62E-04	-0.012
cg19312427	1	198174503	2.12E-86	0.331	0.407	-0.076	*NEK7*	2.12E-06	-0.027
cg05756492	1	206729685	2.20E-86	0.752	0.689	0.063	*RASSF5*	4.37E-06	0.021
cg02276944	2	157291826	2.27E-86	0.437	0.497	-0.060	*GPD2*	1.06E-11	-0.028
cg14242936	12	52404134	2.30E-86	0.373	0.441	-0.069	*GRASP*	1.26E-17	-0.041
cg05146536	4	77140678	2.34E-86	0.234	0.304	-0.070	*FAM47E*	4.73E-18	-0.041
cg01553231	5	141072866	2.52E-86	0.721	0.631	0.090	NA	3.65E-04	0.023
cg22622057	1	182053203	2.54E-86	0.194	0.248	-0.054	NA	6.37E-09	-0.022
cg01329756	17	80346912	2.56E-86	0.375	0.427	-0.052	NA	0.001	-0.012
cg18812353	15	56385430	2.57E-86	0.503	0.575	-0.072	*RFX7*	1.24E-04	-0.019
cg20956594	13	29202889	2.72E-86	0.409	0.502	-0.093	NA	2.52E-06	-0.029
cg26495711	2	240169280	2.75E-86	0.766	0.712	0.054	*HDAC4*	3.37E-07	0.021
cg10133100	11	68276883	2.79E-86	0.385	0.457	-0.073	*PPP6R3*	1.32E-12	-0.036
cg08400494	13	111318490	2.93E-86	0.296	0.377	-0.082	*CARS2*	1.19E-08	-0.030
**cg10168457**	17	79882876	2.95E-86	0.246	0.297	-0.052	*MAFG*	0.793	-0.001
cg14641757	21	36410464	3.17E-86	0.805	0.747	0.059	*RUNX1-IT1*	3.63E-11	0.027
cg06902099	6	35002597	3.31E-86	0.234	0.304	-0.070	*ANKS1A*	2.32E-12	-0.034
cg00865849	5	54518208	3.56E-86	0.179	0.233	-0.054	*MCIN*	0.005	-0.011
cg17490196	2	232507842	3.80E-86	0.343	0.414	-0.071	NA	5.61E-18	-0.044
cg12024104	12	6662581	3.95E-86	0.430	0.508	-0.078	*IFFO1*	2.48E-09	-0.030
cg26763394	3	45957664	3.96E-86	0.738	0.681	0.057	NA	2.76E-16	0.030
cg26697605	7	1850801	4.00E-86	0.293	0.385	-0.093	NA	4.41E-08	-0.037
cg14594044	4	6914621	4.02E-86	0.479	0.540	-0.061	*TBC1D14*	7.37E-09	-0.025
cg22378252	6	10521452	4.13E-86	0.203	0.266	-0.063	*GCNT2*	5.80E-13	-0.032
cg16943126	10	134311114	4.21E-86	0.281	0.349	-0.068	NA	5.91E-09	-0.025
cg22539431	7	127423253	4.34E-86	0.340	0.422	-0.082	*SND1*	1.26E-12	-0.041
cg13584244	9	124030048	4.51E-86	0.339	0.406	-0.067	*GSN*	6.85E-08	-0.026
cg09825309	12	124990897	4.63E-86	0.231	0.297	-0.066	*NCOR2*	9.71E-05	-0.019
**cg12832726**	11	68096165	4.65E-86	0.190	0.254	-0.064	*LRP5*	0.606	-0.002
cg01632240	8	42009223	5.17E-86	0.406	0.482	-0.076	*AP3M2*	1.97E-06	-0.025
cg02347002	5	138719090	5.91E-86	0.350	0.425	-0.075	*SLC23A1*	0.002	-0.016
cg05628049	3	42113624	5.98E-86	0.267	0.334	-0.067	NA	1.57E-04	-0.017
cg20569108	10	21822303	6.48E-86	0.243	0.322	-0.079	*MLLT10*	5.46E-07	-0.029
cg10004897	4	7054700	6.82E-86	0.211	0.268	-0.057	*TADA2B*	6.10E-04	-0.013
cg22726155	21	45575559	7.05E-86	0.770	0.701	0.069	NA	9.36E-13	0.033
cg09465142	20	50109375	7.21E-86	0.363	0.426	-0.063	*NFATC2*	1.00E-05	-0.017
cg20203469	11	134126284	7.57E-86	0.532	0.628	-0.096	*ACAD8*	1.64E-04	-0.023
cg09393619	3	195897833	7.72E-86	0.367	0.453	-0.086	NA	0.006	-0.016
cg20922701	18	11978319	8.04E-86	0.508	0.589	-0.081	NA	1.97E-04	-0.021
cg11006453	8	141599185	8.05E-86	0.783	0.724	0.059	*EIF2C2*	9.51E-10	0.026
cg10233454	12	57529389	8.13E-86	0.358	0.437	-0.080	*LRP1*	8.09E-16	-0.046
cg15661753	12	49627010	8.29E-86	0.231	0.307	-0.076	NA	6.82E-13	-0.041
cg02832357	17	40429406	8.97E-86	0.192	0.256	-0.065	*STAT5B*	3.57E-21	-0.047
cg02042712	16	57702239	9.10E-86	0.258	0.314	-0.056	*GPR97*	3.12E-19	-0.035
cg05624376	2	169939876	9.30E-86	0.341	0.431	-0.090	*DHRS9*	7.27E-04	-0.021
cg15719903	12	6570167	9.43E-86	0.799	0.743	0.056	*TAPBPL*	5.94E-05	0.016
cg04864179	7	128579964	9.67E-86	0.482	0.559	-0.077	*IRF5*	4.76E-13	-0.039
cg12877335	12	94539319	9.83E-86	0.393	0.450	-0.057	NA	8.22E-20	-0.036
cg15334250	3	184297495	9.92E-86	0.287	0.387	-0.099	*EPHB3*	1.02E-07	-0.039
cg17996401	7	47611235	1.04E-85	0.268	0.323	-0.055	*TNS3*	5.49E-08	-0.022
**cg03637218**	5	115209107	1.06E-85	0.442	0.540	-0.098	*AP3S1*	0.238	-0.008
cg23825480	22	31336785	1.08E-85	0.622	0.533	0.089	*MORC2*	1.17E-12	0.043
cg03044513	11	116706153	1.09E-85	0.301	0.364	-0.063	NA	3.05E-08	-0.025
cg22006208	17	27309169	1.13E-85	0.390	0.460	-0.070	*SEZ6*	1.26E-09	-0.031
cg25554496	17	63036095	1.16E-85	0.830	0.776	0.054	*GNA13*	4.55E-09	0.023
cg01797899	14	22978195	1.19E-85	0.811	0.754	0.056	NA	3.35E-12	0.029
cg07679948	12	56329641	1.30E-85	0.854	0.795	0.059	*DGKA*	3.77E-15	0.035

cg04794690	1	17768059	1.42E-85	0.203	0.264	-0.061	NA	1.55E-05	-0.019
cg03324138	1	2250410	1.47E-85	0.713	0.658	0.055	NA	9.94E-06	0.016
cg10710218	1	55012989	1.47E-85	0.324	0.411	-0.087	*ACOT11*	2.16E-09	-0.037
cg02830749	4	154451068	1.50E-85	0.293	0.374	-0.081	*KIAA0922*	7.20E-06	-0.027
cg08231648	1	235011814	1.57E-85	0.275	0.343	-0.068	NA	7.91E-13	-0.034
cg20335425	1	153363264	1.67E-85	0.323	0.384	-0.061	*S100A8*	3.71E-07	-0.020
cg05382012	16	67517841	1.68E-85	0.406	0.485	-0.079	*AGRP*	3.61E-10	-0.035
cg01822050	20	304156	1.69E-85	0.421	0.518	-0.097	NA	8.50E-04	-0.022
cg12510708	7	26193805	1.77E-85	0.252	0.337	-0.085	*NFE2L3*	2.00E-23	-0.063
cg14779825	19	43099845	2.05E-85	0.267	0.335	-0.068	*CEACAM8*	1.02E-11	-0.034
cg17737835	12	2027805	2.05E-85	0.369	0.435	-0.066	*CACNA2D4*	1.56E-15	-0.038
cg06392753	2	102783282	2.28E-85	0.320	0.399	-0.079	*IL1R1*	1.14E-04	-0.023
cg14464245	9	139580482	2.42E-85	0.463	0.530	-0.068	*AGPAT2*	6.71E-17	-0.041
cg17105014	2	127413363	2.49E-85	0.284	0.368	-0.084	*GYPC*	9.52E-07	-0.032
cg03714676	8	48174649	2.65E-85	0.441	0.527	-0.086	*KIAA0146*	4.07E-07	-0.030
cg27432242	2	85777747	2.66E-85	0.299	0.374	-0.075	*GGCX*	2.08E-07	-0.027
cg02078525	16	66400395	2.68E-85	0.304	0.368	-0.064	*CDH5*	4.70E-14	-0.032
cg07805500	6	151380818	2.69E-85	0.303	0.386	-0.083	*MTHFD1L*	3.26E-06	-0.028
cg26983198	11	1952756	2.77E-85	0.242	0.315	-0.073	*TNNT3*	3.21E-10	-0.033
cg22825487	6	133055183	2.81E-85	0.385	0.483	-0.098	*VNN3*	8.06E-07	-0.035
cg00010078	2	109967172	2.97E-85	0.457	0.534	-0.077	*SH3RF3*	6.23E-15	-0.044
cg20618695	12	13251495	3.02E-85	0.222	0.290	-0.068	*GSG1*	4.59E-07	-0.024
cg13134916	12	25707564	3.06E-85	0.312	0.399	-0.088	*IFLTD1*	7.75E-07	-0.030
cg20386303	2	238350541	3.07E-85	0.772	0.718	0.054	NA	1.44E-06	0.019
cg14296561	11	102203542	3.14E-85	0.417	0.493	-0.076	*BIRC3*	3.16E-10	-0.035
cg11361201	5	156690796	3.38E-85	0.252	0.320	-0.068	NA	0.001	-0.016
cg00581764	6	158183853	3.53E-85	0.234	0.294	-0.061	NA	4.60E-08	-0.024
cg26413942	5	124081751	3.56E-85	0.352	0.421	-0.070	*ZNF608*	9.45E-18	-0.042
cg04388548	13	114905640	3.80E-85	0.655	0.590	0.065	NA	7.51E-09	0.024
cg01366985	6	25167695	3.82E-85	0.225	0.299	-0.073	NA	1.23E-04	-0.021
cg09637172	6	31545252	3.89E-85	0.840	0.776	0.064	*TNF*	3.85E-07	0.023
cg19228647	6	6901002	4.15E-85	0.220	0.276	-0.056	NA	8.28E-13	-0.027
cg08165796	8	117484298	4.84E-85	0.370	0.474	-0.105	NA	1.67E-04	-0.027
cg18449389	14	23776482	4.89E-85	0.217	0.276	-0.060	*BCL2L2*	5.44E-10	-0.026
cg20884605	12	112205368	4.98E-85	0.233	0.292	-0.059	*ALDH2*	1.22E-06	-0.020
cg01248878	17	58155376	5.66E-85	0.180	0.238	-0.058	*HEATR6*	1.05E-09	-0.027
cg07850987	17	46645972	5.74E-85	0.239	0.306	-0.067	*HOXB3*	1.79E-04	-0.018
cg27574991	7	47537004	5.77E-85	0.288	0.361	-0.073	*TNS3*	6.25E-12	-0.037
cg11833768	14	63940209	5.78E-85	0.366	0.455	-0.089	*PPP2R5E*	2.12E-08	-0.036
cg09247175	1	12203571	5.90E-85	0.251	0.302	-0.051	*TNFRSF8*	4.20E-09	-0.022
cg27368333	3	182677779	5.97E-85	0.249	0.320	-0.070	*DCUN1D1*	4.71E-06	-0.024
cg10207553	15	65277977	5.99E-85	0.205	0.264	-0.059	*SPG21*	3.50E-07	-0.023
cg05372753	6	11804635	6.46E-85	0.442	0.527	-0.085	NA	3.24E-05	-0.023
cg06033531	5	176980153	6.76E-85	0.382	0.481	-0.099	*FAM193B*	5.06E-05	-0.030
cg04552852	11	844390	7.04E-85	0.396	0.456	-0.060	*TSPAN4*	9.22E-15	-0.033
cg18854872	2	198292151	7.30E-85	0.446	0.513	-0.068	*SF3B1*	8.50E-08	-0.025
cg14533068	6	158507953	7.49E-85	0.460	0.557	-0.096	*SYNJ2*	0.022	-0.015
cg08525429	7	1553444	7.81E-85	0.312	0.387	-0.075	NA	4.19E-08	-0.030
cg01212284	3	182814959	9.16E-85	0.530	0.602	-0.072	*MCCC1*	2.99E-04	-0.017
cg24489015	17	56316162	9.19E-85	0.280	0.362	-0.083	*LPO*	1.20E-07	-0.033
cg26877678	17	25856540	9.63E-85	0.375	0.450	-0.075	*KSR1*	0.002	-0.016
cg02596779	5	179496893	9.66E-85	0.159	0.211	-0.052	*RNF130*	4.46E-08	-0.019
cg16519923	16	30485810	9.71E-85	0.838	0.777	0.061	*ITGAL*	7.73E-05	0.017
cg09050670	16	1521617	9.82E-85	0.154	0.216	-0.062	*CLCN7*	7.46E-04	-0.017
cg15356966	11	33913187	9.89E-85	0.295	0.374	-0.080	*LMO2*	1.90E-06	-0.028
cg17494034	1	12513497	9.98E-85	0.723	0.666	0.058	*VPS13D*	7.04E-16	0.034
cg22243039	17	42147465	1.04E-84	0.229	0.301	-0.072	*G6PC3*	1.44E-04	-0.019
cg24157349	22	47081751	1.16E-84	0.252	0.321	-0.069	*CERK*	4.62E-11	-0.033
cg18743793	7	50464661	1.20E-84	0.737	0.674	0.063	*IKZF1*	1.05E-11	0.032
cg10138630	17	74024966	1.22E-84	0.304	0.357	-0.053	*EVPL*	1.08E-11	-0.025
cg11804414	12	122712137	1.27E-84	0.783	0.729	0.054	*DIABLO*	6.26E-05	0.016
cg21769117	6	31705273	1.28E-84	0.155	0.208	-0.053	*CLIC1*	2.21E-08	-0.022
cg00995854	1	157802305	1.28E-84	0.343	0.434	-0.091	*CD5L*	2.77E-11	-0.043
cg02668248	19	16437789	1.31E-84	0.623	0.545	0.078	*KLF2*	5.15E-14	0.039
cg05637296	17	76129475	1.48E-84	0.765	0.710	0.056	*TMC8*	6.72E-11	0.027
cg27579771	5	142431272	1.53E-84	0.348	0.433	-0.085	*ARHGAP26*	1.70E-14	-0.046
cg15526535	1	12238546	1.67E-84	0.809	0.757	0.052	*TNFRSF1B*	1.14E-04	0.015
cg05255811	6	39192009	1.68E-84	0.356	0.445	-0.089	*KCNK5*	3.06E-05	-0.026
cg08441850	17	37956902	1.68E-84	0.655	0.588	0.066	*IKZF3*	2.67E-15	0.038
cg27094376	16	3639688	1.69E-84	0.419	0.483	-0.064	*SLX4*	1.29E-04	-0.016
cg06710328	11	33217195	1.69E-84	0.249	0.323	-0.074	NA	2.38E-10	-0.034
cg15549637	7	105489998	1.76E-84	0.738	0.671	0.067	*ATXN7L1*	7.92E-05	0.018
cg17680611	19	16191217	1.79E-84	0.843	0.790	0.053	*TPM4*	0.007	0.010

cg05762671	16	67345737	1.86E-84	0.818	0.767	0.051	*KCTD19*	2.48E-05	0.016
cg22438810	9	130911792	1.88E-84	0.464	0.536	-0.072	*LCN2*	4.47E-05	-0.021
cg07852557	17	79422586	1.88E-84	0.386	0.444	-0.059	*BAHCC1*	2.32E-05	-0.016
cg04132186	3	184322709	1.94E-84	0.228	0.292	-0.064	NA	6.07E-08	-0.025
cg16024891	17	2839082	1.95E-84	0.242	0.313	-0.071	*RAP1GAP2*	0.002	-0.016
cg17534540	3	129024712	1.96E-84	0.217	0.274	-0.057	NA	1.12E-05	-0.018
cg01804679	1	32707303	2.06E-84	0.224	0.280	-0.056	*MTMR9LP*	3.70E-08	-0.023
cg00994616	12	6855223	2.11E-84	0.214	0.291	-0.077	NA	6.62E-05	-0.024
cg03834031	22	46465717	2.13E-84	0.179	0.240	-0.061	NA	1.89E-13	-0.034
cg19384905	6	153299788	2.16E-84	0.279	0.349	-0.070	*FBXO5*	1.01E-06	-0.026
cg11826104	15	25650611	2.35E-84	0.526	0.613	-0.087	*UBE3A*	4.39E-06	-0.027
cg14511156	19	54604124	2.56E-84	0.239	0.296	-0.056	*OSCAR*	3.27E-06	-0.019
cg12759387	1	27849177	2.62E-84	0.555	0.498	0.058	NA	3.94E-09	0.022
cg00310940	1	9129648	2.63E-84	0.207	0.262	-0.055	*SLC2A5*	4.71E-09	-0.025
cg03553758	5	121456948	2.67E-84	0.287	0.355	-0.067	NA	4.22E-18	-0.041
cg22429121	1	27901805	3.06E-84	0.439	0.501	-0.062	*AHDC1*	2.23E-04	-0.016
cg02263932	20	50109099	3.10E-84	0.318	0.375	-0.057	*NFATC2*	2.13E-04	-0.015
cg15117516	10	72576586	3.16E-84	0.260	0.316	-0.055	*SGPL1*	6.84E-06	-0.018
cg11183072	17	37894397	3.21E-84	0.184	0.259	-0.075	*GRB7*	1.89E-04	-0.018
cg01595717	9	140586201	3.31E-84	0.170	0.224	-0.055	*EHMT1*	7.64E-08	-0.021
cg20533957	11	94278538	3.43E-84	0.397	0.457	-0.060	*FUT4*	8.61E-05	-0.015
cg00524900	5	118609965	3.63E-84	0.275	0.347	-0.072	*TNFAIP8*	7.53E-08	-0.029
cg24710655	8	145086714	3.82E-84	0.219	0.281	-0.062	*SPATC1*	2.15E-08	-0.024
cg18463607	4	56718320	3.84E-84	0.827	0.770	0.057	*EXOC1*	4.57E-11	0.030
cg08622666	6	13695743	3.96E-84	0.785	0.712	0.073	*RANBP9*	1.36E-11	0.037
cg05418105	22	50981406	4.07E-84	0.507	0.627	-0.120	NA	1.91E-05	-0.034
cg01554529	1	11722935	4.12E-84	0.304	0.387	-0.083	*FBXO6*	0.015	-0.014
cg26494044	10	129760954	4.17E-84	0.756	0.696	0.060	*PTPRE*	1.49E-04	0.016
cg12967723	7	100464145	4.28E-84	0.356	0.426	-0.069	*TRIP6*	2.55E-07	-0.021
cg20670923	18	9915771	4.50E-84	0.190	0.247	-0.057	*VAPA*	3.29E-07	-0.020
cg16802439	16	88907184	4.82E-84	0.402	0.473	-0.071	*GALNS*	0.003	-0.014
cg25989526	13	49147573	4.82E-84	0.302	0.385	-0.082	NA	2.15E-11	-0.039
cg17974145	7	101671556	4.83E-84	0.474	0.546	-0.072	*CUX1*	2.56E-04	-0.019
cg12212198	13	114302021	4.90E-84	0.234	0.309	-0.075	NA	1.96E-04	-0.019
cg12633102	1	35676489	5.23E-84	0.751	0.687	0.064	NA	2.20E-11	0.029
cg07718444	1	1094080	5.27E-84	0.289	0.341	-0.052	NA	8.91E-08	-0.019
cg25708364	17	18905779	5.41E-84	0.384	0.451	-0.067	*SLC5A10*	1.70E-10	-0.029
**cg16625218**	8	30272502	5.83E-84	0.737	0.671	0.066	*RBPMS*	0.352	0.004
cg06257110	16	21658497	6.02E-84	0.820	0.768	0.052	*METTL9*	4.72E-04	0.014
cg21201401	20	62367884	6.34E-84	0.563	0.440	0.123	*LIME1*	4.10E-04	0.028
cg02430430	15	74532450	6.65E-84	0.272	0.326	-0.054	*CCDC33*	5.12E-15	-0.029
cg16701266	14	21024898	6.81E-84	0.308	0.395	-0.087	*RNASE9*	1.13E-09	-0.038
cg11760500	11	128385880	6.90E-84	0.825	0.756	0.069	*ETS1*	2.93E-11	0.036
cg11721610	1	200580225	7.06E-84	0.273	0.347	-0.074	*KIF14*	1.53E-08	-0.032
cg25874953	15	69082449	7.19E-84	0.271	0.327	-0.056	*ANP32A*	1.61E-11	-0.026
cg16478536	6	408730	7.65E-84	0.277	0.350	-0.073	*IRF4*	1.31E-05	-0.022
cg12445422	11	113920436	7.67E-84	0.464	0.534	-0.070	NA	8.55E-16	-0.041
cg14066298	6	30297565	8.07E-84	0.405	0.474	-0.069	*TRIM39*	9.92E-04	-0.015
cg09098522	10	31435673	8.74E-84	0.302	0.379	-0.077	NA	1.03E-05	-0.024
cg17709873	6	31540456	8.97E-84	0.750	0.668	0.082	*LTA*	2.44E-11	0.040
cg23090653	6	32765352	8.99E-84	0.189	0.247	-0.058	NA	1.03E-08	-0.026
cg11839020	1	90309998	9.01E-84	0.636	0.563	0.073	*LRRC8D*	1.99E-07	0.025
cg25218152	11	128554586	9.31E-84	0.402	0.490	-0.088	NA	4.42E-05	-0.025
cg26801613	1	87793510	9.44E-84	0.599	0.675	-0.076	*LMO4*	8.59E-32	-0.062
cg24736734	5	102114455	9.64E-84	0.762	0.696	0.066	NA	1.90E-04	0.018
cg14288403	1	28503368	9.81E-84	0.225	0.276	-0.050	*PTAFR*	6.06E-07	-0.018
cg13740185	10	73486801	1.01E-83	0.491	0.441	0.050	*CDH23*	4.77E-04	0.011
cg06760238	10	134400036	1.13E-83	0.341	0.431	-0.091	*INPP5A*	1.09E-08	-0.038
cg14230719	9	123492289	1.18E-83	0.219	0.279	-0.061	NA	2.39E-12	-0.029
cg10838410	12	6659524	1.25E-83	0.199	0.262	-0.062	*IFFO1*	8.80E-11	-0.029
cg07236781	1	25291041	1.29E-83	0.846	0.791	0.054	*RUNX3*	2.02E-08	0.022
cg23723486	7	101361745	1.30E-83	0.352	0.414	-0.061	NA	2.82E-11	-0.027
cg19725489	5	72252795	1.34E-83	0.165	0.226	-0.061	*FCHO2*	1.82E-10	-0.030
cg13788819	20	31352228	1.36E-83	0.239	0.304	-0.064	*DNMT3B*	5.10E-13	-0.036
cg25032321	13	99227533	1.37E-83	0.736	0.674	0.062	*STK24*	7.71E-12	0.031
cg04350675	6	111873161	1.45E-83	0.309	0.394	-0.085	*TRAF3IP2-AS1*	1.08E-06	-0.032
cg15512851	6	36973518	1.50E-83	0.253	0.322	-0.070	*FGD2*	2.09E-11	-0.036
cg07212384	7	99761053	1.53E-83	0.769	0.703	0.066	*GAL3ST4*	2.95E-07	0.025
cg26389330	5	151057860	1.67E-83	0.278	0.351	-0.073	*SPARC*	4.67E-06	-0.023
cg04709771	16	646395	1.75E-83	0.364	0.418	-0.054	*RAB40C*	6.24E-04	-0.013
cg18338021	19	544349	1.81E-83	0.773	0.719	0.054	*GZMM*	3.05E-10	0.023
cg23013137	14	24676542	1.84E-83	0.721	0.662	0.059	*TSSK4*	0.037	0.008
cg08534147	3	48471771	1.92E-83	0.263	0.330	-0.066	*PLXNB1*	4.29E-05	-0.019

cg23018755	17	79881529	2.05E-83	0.356	0.440	-0.084	*MAFG*	9.14E-05	-0.022
cg09485853	15	72495878	2.08E-83	0.411	0.470	-0.059	*PKM*	5.24E-04	-0.014
cg21057323	14	103412980	2.10E-83	0.653	0.566	0.086	*CDC42BPB*	6.48E-06	0.028
cg00563107	4	141173528	2.18E-83	0.491	0.574	-0.084	NA	1.58E-16	-0.048
cg14453145	2	242801896	2.20E-83	0.799	0.737	0.061	*PDCD1*	1.06E-06	0.022
cg14080050	9	33159025	2.23E-83	0.192	0.249	-0.058	*B4GALT1*	1.67E-12	-0.028
cg03035162	14	22993190	2.27E-83	0.797	0.743	0.055	NA	8.95E-08	0.021
cg02956542	1	153321421	2.29E-83	0.259	0.340	-0.080	*PGLYRP4*	3.50E-11	-0.038
cg19259111	11	46478214	2.38E-83	0.596	0.672	-0.075	*AMBRA1*	3.18E-04	-0.018
cg26639076	2	152331806	2.62E-83	0.293	0.369	-0.076	*RIF1*	1.05E-07	-0.030
cg10859442	11	73716367	2.89E-83	0.784	0.726	0.059	*UCP3*	6.83E-05	0.017
cg22729539	12	122519119	2.99E-83	0.161	0.221	-0.061	*MLXIP*	6.35E-07	-0.023
cg11947782	1	9775985	3.05E-83	0.820	0.749	0.071	*PIK3CD*	8.48E-09	0.030
cg03155200	11	2833259	3.11E-83	0.349	0.442	-0.093	*KCNQ1*	5.18E-11	-0.046
**cg05344747**	11	33754357	3.23E-83	0.465	0.556	-0.091	*CD59*	0.152	-0.009
cg11748260	6	30624395	3.28E-83	0.320	0.392	-0.072	*DHX16*	8.57E-08	-0.030
cg04605532	1	234843592	3.35E-83	0.432	0.517	-0.085	NA	1.64E-04	-0.022
cg09530650	5	137024163	3.36E-83	0.559	0.627	-0.068	*KLHL3*	5.87E-08	-0.025
cg24296397	3	49692537	3.43E-83	0.627	0.552	0.075	*BSN*	1.65E-14	0.038
cg13480197	17	79799358	3.47E-83	0.489	0.549	-0.060	NA	0.006	-0.012
cg02102075	16	474430	3.48E-83	0.465	0.556	-0.091	*RAB11FIP3*	2.94E-05	-0.027
cg15581429	19	39369353	3.66E-83	0.716	0.648	0.068	*SIRT2*	5.28E-11	0.031
cg16199747	6	147235033	3.83E-83	0.232	0.307	-0.075	*STXBP5-AS1*	3.43E-17	-0.048
cg15612947	5	14464064	3.95E-83	0.391	0.477	-0.086	*TRIO*	2.47E-20	-0.051
cg16336494	6	151188259	4.04E-83	0.278	0.357	-0.079	*MTHFD1L*	2.49E-14	-0.045
cg16511445	1	145116797	4.15E-83	0.219	0.287	-0.068	*SEC22B*	1.98E-08	-0.028
cg06043201	8	28974428	4.38E-83	0.366	0.442	-0.075	*KIF13B*	0.002	-0.016
cg23731089	8	141599208	4.50E-83	0.822	0.764	0.058	*EIF2C2*	4.64E-06	0.021
cg04904468	10	81202867	4.52E-83	0.465	0.549	-0.084	*ZCCHC24*	1.10E-05	-0.025
cg10552473	2	206525681	4.67E-83	0.803	0.748	0.055	NA	3.70E-05	0.017
cg11938455	6	15622032	5.18E-83	0.177	0.231	-0.054	*DTNBP1*	2.16E-11	-0.027
cg26247093	7	2773812	5.51E-83	0.181	0.239	-0.058	*GNA12*	0.023	-0.012
cg12744859	17	46669492	5.51E-83	0.192	0.269	-0.077	*HOXB-AS3*	3.45E-05	-0.023
cg16602097	6	32182013	5.90E-83	0.312	0.378	-0.066	*NOTCH4*	3.69E-17	-0.039
cg02327522	19	14633030	5.91E-83	0.848	0.791	0.057	NA	3.51E-14	0.031
cg16942632	12	121726240	5.92E-83	0.354	0.449	-0.095	*CAMKK2*	8.36E-09	-0.039
cg04857672	19	11495077	6.40E-83	0.177	0.228	-0.051	*EPOR*	7.65E-09	-0.021
cg03438773	2	219974186	6.45E-83	0.213	0.268	-0.056	*NHEJ1*	3.44E-05	-0.017
cg04496824	1	38276835	6.57E-83	0.235	0.298	-0.063	*MTF1*	3.01E-07	-0.024
cg11915901	10	31074432	6.66E-83	0.220	0.276	-0.057	NA	4.47E-06	-0.019
cg14027204	1	117529478	6.72E-83	0.765	0.709	0.056	*PTGFRN*	4.47E-16	0.033
cg15724534	2	211018363	6.81E-83	0.513	0.595	-0.082	*KANSL1L*	8.78E-07	-0.026
cg05211189	16	2055403	7.47E-83	0.338	0.411	-0.073	*ZNF598*	1.10E-09	-0.035
cg12649238	6	36669758	7.57E-83	0.520	0.614	-0.094	*RAB44*	0.008	-0.018
cg18385440	15	29212889	7.60E-83	0.302	0.353	-0.051	*APBA2*	1.20E-12	-0.024
cg00777445	17	48970357	7.88E-83	0.450	0.543	-0.093	NA	2.48E-05	-0.027
cg17083575	7	4746131	8.07E-83	0.215	0.275	-0.060	*FOXK1*	1.39E-05	-0.019
cg24522654	12	19535154	8.18E-83	0.762	0.700	0.062	NA	8.37E-10	0.027
cg06172950	13	46075084	8.50E-83	0.239	0.305	-0.067	*COG3*	9.93E-13	-0.035
cg14085840	19	40939429	8.63E-83	0.253	0.308	-0.055	NA	5.87E-13	-0.028
cg10819238	19	1155184	8.75E-83	0.237	0.299	-0.062	*SBNO2*	2.67E-08	-0.024
cg11229273	3	57015101	8.86E-83	0.242	0.308	-0.067	*ARHGEF3*	8.44E-12	-0.033
cg17741993	6	31544694	9.08E-83	0.632	0.548	0.084	*TNF*	0.001	0.019
cg21495704	19	36399346	9.53E-83	0.286	0.361	-0.075	*TYROBP*	4.23E-07	-0.027
cg06453916	16	29690524	1.00E-82	0.276	0.341	-0.065	*QPRT*	1.72E-17	-0.042
cg08136416	21	44060833	1.00E-82	0.262	0.328	-0.066	NA	1.82E-21	-0.045
cg23534216	10	102821552	1.18E-82	0.332	0.396	-0.064	*KAZALD1*	2.69E-07	-0.022
cg21108554	7	150061328	1.18E-82	0.238	0.311	-0.072	NA	2.05E-08	-0.030
cg07123796	1	950971	1.19E-82	0.395	0.455	-0.060	NA	3.80E-14	-0.028
cg10925991	4	40324096	1.31E-82	0.296	0.366	-0.070	NA	2.77E-10	-0.032
cg26312951	21	42797847	1.34E-82	0.259	0.435	-0.176	*MX1*	1.32E-41	-0.141
cg14801864	20	17540975	1.35E-82	0.417	0.485	-0.068	*BFSP1*	1.05E-09	-0.027
cg10549986	2	7018153	1.41E-82	0.083	0.165	-0.081	*RSAD2*	2.40E-23	-0.060
cg10287137	11	72929054	1.42E-82	0.378	0.439	-0.061	*P2RY2*	0.005	-0.011
cg10421029	16	30936028	1.50E-82	0.246	0.301	-0.055	*FBXL19*	5.86E-20	-0.038
cg07409200	13	43354674	1.54E-82	0.295	0.390	-0.095	*FAM216B*	1.56E-12	-0.047
cg06706159	19	18260350	1.61E-82	0.899	0.823	0.077	*MAST3*	5.67E-07	0.032
cg09586924	11	130032815	1.65E-82	0.193	0.248	-0.055	*ST14*	5.46E-10	-0.027
cg19453686	14	92334271	1.68E-82	0.820	0.758	0.063	*TC2N*	2.23E-08	0.024
cg01015899	12	120663812	1.75E-82	0.268	0.327	-0.059	*PXN*	4.44E-07	-0.021
cg08106108	17	695095	1.76E-82	0.626	0.701	-0.075	*RNMTL1*	1.99E-07	-0.026
cg19397991	2	242048218	1.78E-82	0.688	0.625	0.064	*PASK*	3.06E-11	0.030
cg00454592	2	12013993	1.85E-82	0.497	0.585	-0.088	NA	4.59E-04	-0.022

cg08123074	1	28764523	1.89E-82	0.148	0.212	-0.063	*PHACTR4*	2.06E-06	-0.024
cg08220149	16	15766327	2.05E-82	0.252	0.339	-0.087	*NDE1*	4.44E-06	-0.030
cg08645907	7	105319679	2.08E-82	0.220	0.278	-0.058	*ATXN7L1*	4.10E-08	-0.021
cg07571745	1	32715428	2.09E-82	0.553	0.492	0.061	*LCK*	3.01E-07	0.020
cg04280772	2	235372807	2.12E-82	0.823	0.770	0.053	NA	4.11E-11	0.024
cg15388107	7	36700916	2.54E-82	0.365	0.433	-0.068	*AOAH*	2.32E-10	-0.031
cg09599062	3	141995701	2.58E-82	0.266	0.348	-0.082	NA	7.37E-14	-0.044
cg13579752	5	153577937	2.88E-82	0.719	0.663	0.056	*GALNT10*	1.84E-10	0.025
cg16292768	8	27467783	2.89E-82	0.528	0.619	-0.091	*CLU*	1.32E-10	-0.040
cg18188739	1	2058941	3.02E-82	0.201	0.265	-0.064	*PRKCZ*	4.00E-08	-0.024
cg14725580	6	34663155	3.06E-82	0.219	0.283	-0.064	*C6orf106*	5.86E-07	-0.024
cg19770281	3	132261993	3.12E-82	0.237	0.306	-0.070	NA	2.04E-08	-0.030
cg13464573	2	7172097	3.17E-82	0.339	0.405	-0.066	*RNF144A*	7.40E-05	-0.019
cg10540110	1	202775609	3.36E-82	0.143	0.196	-0.053	*KDM5B*	1.06E-05	-0.018
cg10821261	12	54822725	3.36E-82	0.289	0.352	-0.062	NA	3.04E-10	-0.030
cg27543538	1	27902687	3.62E-82	0.310	0.377	-0.067	*AHDC1*	2.11E-07	-0.024
cg19266387	3	183596123	3.63E-82	0.234	0.316	-0.082	*PARL*	2.17E-04	-0.023
cg02375208	5	77804381	3.76E-82	0.257	0.329	-0.072	*LHFPL2*	3.09E-14	-0.040
cg06611426	12	52404161	3.96E-82	0.397	0.472	-0.075	*GRASP*	1.89E-18	-0.045
cg14181576	1	27961563	4.09E-82	0.203	0.272	-0.069	*FGR*	1.73E-07	-0.028
cg10142436	15	42209178	4.11E-82	0.233	0.297	-0.064	*EHD4*	1.21E-06	-0.024
cg01627405	19	2235127	4.25E-82	0.269	0.350	-0.081	*PLEKHJ1*	1.20E-04	-0.025
**cg12897164**	8	124529273	4.31E-82	0.743	0.677	0.066	*FBXO32*	0.274	0.005
cg11671363	5	148810177	4.32E-82	0.301	0.381	-0.080	*MIR143HG*	0.005	-0.017
cg00917471	1	43228316	4.60E-82	0.302	0.375	-0.074	*LEPRE1*	5.53E-10	-0.033
**cg05949667**	11	134126504	4.84E-82	0.487	0.551	-0.064	*ACAD8*	0.086	-0.008
cg16684817	1	28202173	4.94E-82	0.376	0.478	-0.102	*THEMIS2*	0.004	-0.022
cg18307978	7	30953124	6.03E-82	0.709	0.658	0.051	*AQP1*	0.006	0.010
cg08241785	5	75919281	6.47E-82	0.259	0.328	-0.068	*IQGAP2*	1.75E-07	-0.027
cg19299755	11	116706051	6.83E-82	0.398	0.465	-0.067	NA	1.11E-09	-0.026
cg09853238	6	149532290	6.86E-82	0.184	0.254	-0.070	NA	1.45E-20	-0.052
cg05128520	16	84676746	7.12E-82	0.353	0.431	-0.078	NA	4.91E-10	-0.038
cg23073974	2	8039990	7.36E-82	0.353	0.430	-0.077	NA	1.84E-10	-0.036
cg21224380	12	109240533	7.59E-82	0.436	0.518	-0.082	*SSH1*	1.16E-04	-0.022
cg23275644	5	10457079	7.69E-82	0.460	0.546	-0.085	*ROPN1L*	3.78E-08	-0.034
cg07636142	7	69292759	8.52E-82	0.155	0.211	-0.056	*AUTS2*	2.02E-05	-0.020
cg04349839	4	25789390	8.61E-82	0.480	0.559	-0.080	*SEL1L3*	2.31E-11	-0.040
cg24450653	1	205060293	9.01E-82	0.535	0.594	-0.059	*RBBP5*	8.69E-08	-0.024
cg08550517	16	85414644	9.43E-82	0.398	0.477	-0.079	NA	8.06E-13	-0.037
cg27058077	1	200337470	9.52E-82	0.249	0.307	-0.059	*C1orf98*	3.53E-06	-0.019
cg03746834	8	28196926	1.00E-81	0.287	0.357	-0.070	*PNOC*	1.11E-06	-0.025
cg20072118	12	52653637	1.11E-81	0.209	0.272	-0.063	NA	6.94E-05	-0.019
cg18734433	7	150086169	1.11E-81	0.394	0.448	-0.054	*ZNF775*	5.99E-10	-0.022
cg17223520	1	32739114	1.14E-81	0.816	0.766	0.051	*LCK*	6.57E-17	0.030
cg21545535	5	176809747	1.15E-81	0.279	0.335	-0.056	NA	6.95E-06	-0.018
cg25790531	6	113755716	1.19E-81	0.509	0.601	-0.092	NA	0.025	-0.014
cg04452195	13	20762982	1.20E-81	0.377	0.472	-0.096	*GJB2*	0.002	-0.021
cg04834204	2	69423553	1.33E-81	0.392	0.475	-0.083	*ANTXR1*	2.76E-06	-0.028
cg05327068	15	34634045	1.33E-81	0.425	0.512	-0.087	*NOP10*	1.54E-09	-0.037
cg04572930	7	4754834	1.41E-81	0.375	0.471	-0.097	*FOXK1*	3.88E-05	-0.029
cg08120831	12	122669186	1.49E-81	0.236	0.295	-0.059	*LRRC43*	6.28E-07	-0.021
cg13048008	11	71725340	1.52E-81	0.391	0.444	-0.053	*NUMA1*	1.28E-06	-0.018
cg01504447	13	31041221	1.52E-81	0.187	0.249	-0.062	*HMGB1*	1.12E-17	-0.042
cg21407196	1	46751975	1.54E-81	0.778	0.722	0.056	*LRRC41*	8.05E-16	0.034
cg10665891	12	117042917	1.76E-81	0.385	0.491	-0.106	NA	0.034	-0.016
cg00488091	6	144536326	1.77E-81	0.359	0.429	-0.070	NA	3.41E-09	-0.028
cg26768584	3	18480242	1.78E-81	0.807	0.743	0.064	*SATB1*	5.26E-05	0.021
cg14523804	17	75995968	1.81E-81	0.539	0.621	-0.081	NA	0.002	-0.018
cg21483922	17	48205411	1.82E-81	0.176	0.227	-0.052	*SAMD14*	0.004	-0.011
cg12509499	17	2095179	1.84E-81	0.461	0.546	-0.085	*SMG6*	3.98E-06	-0.027
cg08441138	11	128555233	1.97E-81	0.230	0.294	-0.064	*FLI1*	9.02E-04	-0.016
cg23230830	1	248903100	2.16E-81	0.264	0.349	-0.085	*LYPD8*	9.55E-09	-0.036
cg10378348	1	6244619	2.18E-81	0.193	0.246	-0.053	NA	9.92E-13	-0.030
cg04347477	12	125002007	2.20E-81	0.280	0.369	-0.089	*NCOR2*	0.007	-0.018
cg01943221	1	9776133	2.27E-81	0.753	0.687	0.066	*PIK3CD*	1.23E-07	0.024
cg17471425	11	10614014	2.33E-81	0.185	0.241	-0.056	*MRVI1-AS1*	1.55E-16	-0.035
cg23247968	10	104535961	2.35E-81	0.232	0.284	-0.052	*WBP1L*	0.018	-0.008
cg02976588	1	150135546	2.37E-81	0.261	0.341	-0.080	NA	2.00E-05	-0.026
cg02802072	17	79229124	2.41E-81	0.297	0.374	-0.077	*SLC38A10*	0.001	-0.017
cg17766305	10	90147030	2.47E-81	0.292	0.383	-0.091	*RNLS*	1.51E-06	-0.032
cg19323289	11	19463903	2.53E-81	0.744	0.683	0.061	*NAV2*	0.027	0.010
cg24457403	17	39770337	2.61E-81	0.791	0.737	0.054	*KRT16*	3.40E-04	0.014
cg05699739	13	47373579	2.64E-81	0.245	0.309	-0.065	NA	6.20E-08	-0.025

cg22224704	11	67352041	2.74E-81	0.290	0.345	-0.056	*GSTP1*	9.21E-14	-0.031
cg03072035	10	73076664	3.01E-81	0.447	0.527	-0.080	NA	8.38E-06	-0.027
cg21440776	12	6658378	3.05E-81	0.340	0.445	-0.105	*IFFO1*	1.95E-04	-0.031
cg07095530	7	100859728	3.17E-81	0.451	0.509	-0.058	*ZNHIT1*	9.97E-06	-0.017
cg16656520	6	31800885	3.24E-81	0.293	0.371	-0.077	NA	6.13E-07	-0.029
cg10705306	8	37337009	3.54E-81	0.187	0.247	-0.060	NA	1.83E-10	-0.031
cg04774043	10	104498430	3.92E-81	0.266	0.336	-0.069	*SFXN2*	1.02E-07	-0.027
cg24585377	1	26857774	4.18E-81	0.320	0.394	-0.074	*RPS6KA1*	1.01E-13	-0.043
cg25936358	13	114301996	4.44E-81	0.255	0.328	-0.073	NA	3.28E-04	-0.018
cg22898082	19	11074428	4.44E-81	0.602	0.542	0.060	*SMARCA4*	5.09E-13	0.029
cg27055685	6	33258248	4.82E-81	0.221	0.282	-0.061	*WDR46*	6.96E-07	-0.023
cg26711333	8	8821019	5.31E-81	0.257	0.321	-0.064	NA	1.15E-04	-0.018
cg06436185	7	151442351	5.43E-81	0.297	0.387	-0.091	*PRKAG2*	0.011	-0.018
cg01610123	5	134770100	5.63E-81	0.300	0.371	-0.072	NA	1.07E-09	-0.033
cg05349024	14	101471543	5.96E-81	0.245	0.298	-0.053	NA	1.08E-05	-0.017
cg07071036	19	50014987	6.53E-81	0.235	0.295	-0.060	*FCGRT*	1.64E-10	-0.028
cg05206633	5	177913434	6.69E-81	0.491	0.605	-0.114	*COL23A1*	4.36E-07	-0.034
cg11009736	2	119699682	6.70E-81	0.261	0.341	-0.079	*MARCO*	7.66E-16	-0.049
cg08926253	11	614761	6.80E-81	0.468	0.597	-0.128	*IRF7*	3.84E-34	-0.077
cg07044422	22	42828516	6.97E-81	0.195	0.254	-0.059	*NFAM1*	3.55E-10	-0.029
cg25273707	11	76037066	7.03E-81	0.624	0.547	0.078	NA	4.31E-04	0.020
cg21471199	1	19218212	7.43E-81	0.466	0.553	-0.087	*ALDH4A1*	1.42E-04	-0.024
cg23846712	19	3607608	7.68E-81	0.299	0.363	-0.064	*TBXA2R*	1.08E-16	-0.040
cg03984055	16	31117318	8.47E-81	0.286	0.337	-0.051	NA	5.69E-07	-0.016
cg02933639	4	8207325	8.59E-81	0.374	0.434	-0.060	*SH3TC1*	2.26E-07	-0.021
cg04658021	17	8056967	8.68E-81	0.319	0.400	-0.082	*PER1*	0.010	-0.015
cg02287007	6	166877038	8.82E-81	0.284	0.372	-0.088	*RPS6KA2*	4.07E-08	-0.036
cg01963224	14	71791671	8.89E-81	0.134	0.186	-0.052	NA	0.018	-0.009
cg11095743	10	49815243	8.92E-81	0.230	0.293	-0.063	*ARHGAP22*	2.63E-23	-0.046
cg17500228	5	448790	9.21E-81	0.457	0.554	-0.098	*EXOC3*	0.012	-0.017
cg17922998	8	61863697	9.66E-81	0.222	0.290	-0.068	NA	4.08E-08	-0.028
cg07268332	11	10476494	1.03E-80	0.405	0.480	-0.075	*AMPD3*	0.002	-0.019
cg13932501	9	94060853	1.05E-80	0.459	0.537	-0.078	*AUH*	2.51E-09	-0.032
cg10802680	12	122712075	1.10E-80	0.691	0.633	0.059	*DIABLO*	1.93E-09	0.025
cg03393802	1	33738772	1.25E-80	0.746	0.680	0.066	*ZNF362*	5.70E-05	0.019
cg20146241	1	24861604	1.25E-80	0.500	0.412	0.088	*RCAN3*	1.71E-07	0.032
cg26899598	15	91429088	1.36E-80	0.193	0.247	-0.054	*FES*	2.54E-06	-0.019
cg26562691	16	23850404	1.39E-80	0.828	0.777	0.051	*PRKCB*	0.022	0.008
cg03091668	22	24903402	1.41E-80	0.308	0.382	-0.073	*UPB1*	8.21E-08	-0.032
cg13300301	11	94279068	1.43E-80	0.385	0.469	-0.083	*FUT4*	1.74E-09	-0.038
cg26985681	16	15135397	1.61E-80	0.348	0.427	-0.079	*NTAN1*	6.19E-08	-0.032
cg14479344	3	108126795	1.72E-80	0.337	0.412	-0.076	*MYH15*	9.30E-07	-0.028
cg17173423	11	59823993	1.82E-80	0.287	0.362	-0.074	*MS4A3*	1.47E-12	-0.038
cg11674933	1	36948313	1.87E-80	0.220	0.276	-0.057	*CSF3R*	4.25E-11	-0.028
cg09684429	11	124768015	1.92E-80	0.275	0.330	-0.055	*ROBO4*	1.86E-13	-0.031
cg19649900	19	1155030	1.98E-80	0.366	0.432	-0.067	*SBNO2*	2.26E-06	-0.021
cg14918548	2	101350221	2.05E-80	0.308	0.394	-0.086	NA	6.82E-11	-0.044
cg07586956	2	70336043	2.05E-80	0.810	0.746	0.064	NA	4.54E-15	0.037
cg12459932	1	25292018	2.21E-80	0.830	0.776	0.054	*RUNX3*	6.71E-05	0.016
cg08894588	16	58027941	2.24E-80	0.538	0.607	-0.069	NA	6.61E-07	-0.024
cg09042678	20	18117663	2.66E-80	0.329	0.414	-0.085	*PET117*	2.80E-05	-0.027
cg02138331	20	32893975	2.75E-80	0.599	0.679	-0.080	*AHCY*	1.27E-07	-0.027
cg25303150	10	134211874	2.78E-80	0.235	0.301	-0.066	*PWWP2B*	8.80E-04	-0.016
cg25252561	10	75610927	2.82E-80	0.237	0.288	-0.051	*CAMK2G*	1.61E-16	-0.029
cg20986887	6	28887284	2.85E-80	0.273	0.353	-0.080	*TRIM27*	4.16E-07	-0.030
cg21491107	16	85649026	2.87E-80	0.299	0.368	-0.068	*KIAA0182*	8.57E-05	-0.018
cg16964946	11	27442973	2.93E-80	0.429	0.514	-0.085	*LGR4*	1.80E-07	-0.031
cg10620680	1	178025100	2.97E-80	0.238	0.309	-0.071	NA	2.42E-09	-0.032
cg27316369	17	79799376	3.01E-80	0.518	0.576	-0.058	NA	0.003	-0.012
cg04529938	17	73084494	3.16E-80	0.238	0.294	-0.056	*SLC16A5*	4.41E-04	-0.014
cg24495643	8	29230953	3.23E-80	0.260	0.317	-0.057	NA	1.39E-05	-0.018
cg13751113	11	118085214	3.23E-80	0.205	0.266	-0.061	*AMICA1*	3.89E-08	-0.025
cg06107293	15	22915986	3.83E-80	0.356	0.434	-0.078	*CYFIP1*	1.07E-04	-0.022
cg10057295	13	99230004	3.98E-80	0.250	0.313	-0.063	*STK24*	1.77E-16	-0.040
cg20014974	1	8271918	4.04E-80	0.256	0.312	-0.057	NA	1.15E-07	-0.022
cg17646250	6	6759768	4.05E-80	0.283	0.338	-0.056	NA	1.68E-10	-0.026
cg08510456	3	49591008	4.26E-80	0.275	0.355	-0.080	*BSN*	5.71E-08	-0.035
cg05486924	3	194979565	4.30E-80	0.258	0.335	-0.077	*XXYLT1*	0.002	-0.017
cg16145324	6	36020012	4.30E-80	0.463	0.539	-0.075	*MAPK14*	5.39E-05	-0.023
cg02233614	1	207227965	4.37E-80	0.196	0.255	-0.059	*PFKFB2*	1.08E-09	-0.027
cg13984330	17	58637589	4.57E-80	0.505	0.583	-0.077	NA	1.38E-07	-0.029
cg07038243	5	149868855	4.65E-80	0.285	0.343	-0.058	NA	5.83E-06	-0.019
cg03924164	16	88515063	4.87E-80	0.210	0.267	-0.057	NA	1.03E-05	-0.019

cg06102602	12	96895446	5.00E-80	0.358	0.442	-0.084	NA	2.30E-05	-0.024
**cg23944572**	5	154101659	5.02E-80	0.656	0.596	0.060	*LARP1*	0.586	0.002
cg00219816	8	96280555	5.61E-80	0.428	0.504	-0.077	*C8orf37*	1.36E-11	-0.037
cg13765961	11	60223238	5.83E-80	0.797	0.738	0.060	*MS4A1*	3.57E-05	0.019
**cg25332499**	11	64756382	6.76E-80	0.799	0.744	0.055	*BATF2*	0.461	0.003
cg23357789	19	848026	6.89E-80	0.250	0.314	-0.064	*PRTN3*	8.64E-09	-0.028
cg02929855	12	1922067	7.28E-80	0.406	0.504	-0.099	*CACNA2D4*	5.00E-04	-0.024
cg12927730	2	97260774	7.41E-80	0.635	0.557	0.078	*KANSL3*	8.11E-05	0.022
cg13601595	8	10468038	7.63E-80	0.413	0.488	-0.074	*RP1L1*	1.83E-09	-0.029
cg09472600	1	183537770	8.09E-80	0.258	0.333	-0.075	*NCF2*	2.36E-07	-0.029
cg02592133	2	99526035	8.39E-80	0.210	0.268	-0.058	*KIAA1211L*	4.53E-06	-0.020
cg06538141	20	30229335	8.53E-80	0.749	0.694	0.055	*COX4I2*	0.002	0.012
cg13109911	1	160178210	8.68E-80	0.410	0.463	-0.053	*PEA15*	1.66E-05	-0.017
**cg11767757**	21	40145404	8.70E-80	0.722	0.651	0.071	NA	0.150	0.007
cg02061820	3	152046751	9.13E-80	0.244	0.314	-0.069	*MBNL1*	0.004	-0.015
cg23428738	12	4312337	9.91E-80	0.457	0.544	-0.087	NA	0.011	-0.015
cg17552471	7	4678661	1.11E-79	0.701	0.651	0.050	NA	3.27E-05	0.016
cg11781421	19	19367084	1.16E-79	0.392	0.470	-0.078	*HAPLN4*	2.31E-04	-0.021
cg09855706	1	244965648	1.18E-79	0.271	0.346	-0.075	NA	1.08E-07	-0.031
cg08132815	19	4950822	1.21E-79	0.530	0.624	-0.094	*UHRF1*	0.027	-0.013
cg09088576	1	36947785	1.27E-79	0.196	0.251	-0.054	*CSF3R*	6.61E-04	-0.013
cg06449094	11	822397	1.37E-79	0.470	0.550	-0.081	*PNPLA2*	1.31E-06	-0.026
cg10909080	17	79881468	1.43E-79	0.334	0.405	-0.071	*MAFG*	0.002	-0.016
cg03030267	20	57394000	1.47E-79	0.822	0.769	0.053	*GNAS-AS1*	1.61E-11	0.026
cg16349093	5	90485840	1.47E-79	0.323	0.422	-0.099	NA	4.65E-14	-0.059
cg02088996	7	41817771	1.49E-79	0.224	0.285	-0.061	*INHBA-AS1*	4.70E-20	-0.042
cg04992150	3	13457267	1.50E-79	0.232	0.285	-0.053	*NUP210*	7.04E-05	-0.016
**cg06092869**	1	225642746	1.61E-79	0.617	0.564	0.053	NA	0.207	0.005
cg26262049	11	10628012	1.61E-79	0.326	0.404	-0.077	*MRVI1*	4.01E-16	-0.047
cg01372811	16	85970529	1.64E-79	0.825	0.774	0.052	NA	1.35E-04	0.015
cg20516032	8	128994030	1.66E-79	0.802	0.747	0.055	*PVT1*	2.96E-10	0.025
cg25114611	6	35696870	1.69E-79	0.290	0.361	-0.071	*LOC285847*	3.59E-16	-0.042
cg06196379	6	41254885	1.75E-79	0.269	0.334	-0.065	*TREM1*	1.50E-16	-0.037
cg05088356	5	66124167	1.77E-79	0.817	0.761	0.056	*MAST4*	4.41E-10	0.027
cg08178956	2	42345201	1.81E-79	0.293	0.368	-0.076	NA	2.31E-07	-0.031
cg19118951	21	35575070	1.84E-79	0.271	0.341	-0.069	NA	5.05E-04	-0.016
cg05651511	17	78754090	1.87E-79	0.304	0.394	-0.090	*RPTOR*	0.013	-0.018
cg06098215	10	51575702	1.87E-79	0.270	0.345	-0.075	*NCOA4*	3.31E-04	-0.021
cg18083248	15	26108946	1.99E-79	0.301	0.359	-0.057	*ATP10A*	1.75E-09	-0.026
cg04208434	3	129513427	2.00E-79	0.212	0.280	-0.068	*TMCC1*	5.96E-05	-0.020
cg05671644	7	102632303	2.00E-79	0.406	0.483	-0.077	*FBXL13*	1.73E-11	-0.036
cg23856138	10	35658321	2.23E-79	0.192	0.256	-0.064	*CCNY*	1.04E-06	-0.025
cg16281322	17	43510478	2.25E-79	0.384	0.458	-0.074	*ARHGAP27*	1.59E-06	-0.026
cg23975840	12	117042895	2.45E-79	0.527	0.625	-0.098	NA	3.73E-06	-0.030
cg25541209	20	45947892	2.48E-79	0.350	0.427	-0.077	*ZMYND8*	1.23E-06	-0.027
cg20719001	17	79297435	2.58E-79	0.308	0.381	-0.073	*TMEM105*	1.19E-05	-0.024
cg19782686	4	124180616	2.60E-79	0.363	0.440	-0.077	*SPATA5*	9.53E-13	-0.041
cg15980656	8	97583236	2.72E-79	0.355	0.439	-0.084	*SDC2*	4.17E-08	-0.033
cg18141622	2	238525524	2.79E-79	0.361	0.442	-0.081	NA	2.37E-13	-0.043
cg00782708	2	44933278	2.96E-79	0.513	0.587	-0.074	*CAMKMT*	2.18E-05	-0.023
cg13488078	8	27469338	3.00E-79	0.263	0.325	-0.062	*CLU*	1.75E-09	-0.028
cg14184400	3	49460057	3.06E-79	0.381	0.444	-0.064	*AMT*	1.94E-08	-0.025
cg27262850	18	74826769	3.13E-79	0.268	0.329	-0.062	*MBP*	4.11E-05	-0.019
cg01557792	14	70162755	3.44E-79	0.156	0.211	-0.055	*KIAA0247*	0.021	-0.010
cg22989958	2	74783039	3.68E-79	0.361	0.433	-0.072	*DOK1*	3.14E-09	-0.032
cg26704043	6	5282702	3.94E-79	0.343	0.407	-0.064	*FARS2*	1.25E-06	-0.021
cg02659854	13	24824078	4.02E-79	0.337	0.403	-0.066	*SPATA13*	4.15E-09	-0.030
cg15902038	15	42693448	4.66E-79	0.245	0.306	-0.061	*CAPN3*	9.50E-09	-0.027
cg25181170	10	25162298	4.69E-79	0.347	0.434	-0.087	*PRTFDC1*	2.04E-04	-0.025
cg05438727	11	2800047	4.81E-79	0.293	0.348	-0.055	*KCNQ1*	1.18E-07	-0.022
cg26729913	17	79135071	4.89E-79	0.355	0.423	-0.068	*AATK*	2.74E-08	-0.026
cg08024038	17	17616235	4.97E-79	0.424	0.489	-0.065	*RAI1*	1.17E-07	-0.026
cg01586609	11	113846937	4.98E-79	0.407	0.520	-0.113	*HTR3A*	7.35E-04	-0.024
cg05915981	6	131571669	5.05E-79	0.188	0.239	-0.051	*AKAP7*	1.54E-04	-0.015
cg17150898	12	1702116	5.26E-79	0.666	0.592	0.074	*FBXL14*	1.27E-06	0.024
cg05338397	1	15466556	5.27E-79	0.649	0.589	0.060	*C1orf126*	2.15E-05	0.019
cg26412374	11	63437874	5.48E-79	0.282	0.361	-0.079	*ATL3*	7.44E-06	-0.028
cg13746740	11	128554939	5.82E-79	0.225	0.283	-0.057	*FLI1*	1.77E-07	-0.023
cg00479463	19	33726786	6.58E-79	0.673	0.600	0.074	NA	8.40E-04	0.018
cg26118943	5	53816563	6.79E-79	0.265	0.339	-0.074	*SNX18*	4.71E-08	-0.029
cg25606842	1	86052061	7.19E-79	0.825	0.774	0.051	NA	0.003	0.012
cg16678522	20	32149963	7.38E-79	0.516	0.592	-0.076	*CBFA2T2*	2.57E-10	-0.037
cg01572694	17	46657555	7.40E-79	0.465	0.561	-0.096	NA	3.81E-16	-0.056

cg23098018	1	9775755	7.76E-79	0.757	0.684	0.073	*PIK3CD*	8.08E-09	0.028
cg25573640	14	64228599	7.83E-79	0.525	0.611	-0.086	NA	1.82E-04	-0.023
cg11384661	1	238025414	7.88E-79	0.254	0.323	-0.070	NA	1.16E-08	-0.032
cg13160251	15	56286575	7.95E-79	0.290	0.363	-0.073	*NEDD4*	1.51E-08	-0.032
cg25050026	3	158519410	7.98E-79	0.178	0.237	-0.059	*MFSD1*	1.12E-09	-0.028
cg10522770	7	158646253	8.03E-79	0.258	0.318	-0.061	NA	4.39E-09	-0.026
cg25952192	18	21656374	8.29E-79	0.669	0.604	0.065	*TTC39C*	2.71E-09	0.029
cg00987918	6	30115829	8.39E-79	0.760	0.698	0.062	*TRIM40*	0.010	0.012
cg01059100	16	31214607	9.10E-79	0.268	0.328	-0.060	*PYCARD*	2.92E-13	-0.033
cg09646392	13	108921052	9.21E-79	0.364	0.448	-0.084	*TNFSF13B*	2.08E-06	-0.029
cg04231677	1	184808004	9.22E-79	0.326	0.394	-0.068	*FAM129A*	2.58E-09	-0.029
cg14196186	17	72732698	9.28E-79	0.207	0.268	-0.062	*RAB37*	2.19E-09	-0.028
cg07805604	19	56158885	9.29E-79	0.252	0.314	-0.061	*CCDC106*	1.45E-05	-0.020
cg14547461	8	48176671	9.53E-79	0.484	0.565	-0.082	*KIAA0146*	5.06E-06	-0.026
cg01774027	19	947712	9.56E-79	0.321	0.389	-0.067	*ARID3A*	9.18E-09	-0.029
cg01105337	20	2308707	1.06E-78	0.778	0.725	0.054	*TGM3*	8.25E-05	0.015
cg19344545	21	45575573	1.08E-78	0.754	0.685	0.069	NA	1.00E-11	0.034
cg07996532	1	21620812	1.13E-78	0.643	0.579	0.063	*LOC10050680.*	0.002	0.015
cg11854981	6	42219847	1.17E-78	0.340	0.415	-0.075	*TRERF1*	9.60E-08	-0.029
cg13509702	8	144896307	1.25E-78	0.423	0.495	-0.071	*SCRIB*	1.19E-10	-0.032
cg14454680	3	99794010	1.27E-78	0.342	0.429	-0.087	*FILIP1L*	3.38E-07	-0.032
cg22076474	21	30677172	1.33E-78	0.356	0.437	-0.081	*BACH1*	4.80E-08	-0.033
cg01015663	1	23729692	1.37E-78	0.406	0.487	-0.080	*TCEA3*	1.98E-13	-0.043
cg24575128	3	52502445	1.43E-78	0.406	0.476	-0.070	*NISCH*	5.79E-12	-0.038
cg13374432	15	86192745	1.49E-78	0.273	0.350	-0.078	*AKAP13*	5.51E-09	-0.033
cg10959668	3	195897912	1.59E-78	0.352	0.416	-0.065	NA	0.002	-0.015
cg20008101	14	55595227	1.60E-78	0.368	0.457	-0.089	*LGALS3*	4.28E-04	-0.021
cg13366774	12	10336861	1.69E-78	0.517	0.611	-0.094	*TMEM52B*	0.002	-0.021
cg01058360	7	151442371	1.81E-78	0.430	0.539	-0.108	*PRKAG2*	4.18E-04	-0.028
cg19638572	1	206733139	1.81E-78	0.538	0.464	0.074	*RASSF5*	3.10E-04	0.019
cg03106245	11	47399980	1.83E-78	0.388	0.483	-0.096	*SPI1*	5.21E-04	-0.024
cg23137881	4	159857583	1.90E-78	0.431	0.523	-0.092	*C4orf45*	8.53E-04	-0.020
cg09643312	2	160655081	1.94E-78	0.389	0.475	-0.086	*CD302*	9.36E-13	-0.046
cg18173184	12	6718378	1.99E-78	0.274	0.345	-0.071	NA	1.01E-05	-0.024
cg13925011	1	111216387	2.03E-78	0.731	0.667	0.063	*KCNA3*	3.49E-07	0.021
cg26056277	2	166982925	2.05E-78	0.333	0.409	-0.077	*SCN1A*	1.89E-05	-0.025
cg18715793	19	4518890	2.13E-78	0.454	0.542	-0.088	*PLIN4*	5.82E-04	-0.019
cg04189326	14	103481271	2.15E-78	0.347	0.403	-0.056	*CDC42BPB*	4.85E-08	-0.023
cg03379681	3	151058527	2.16E-78	0.207	0.263	-0.056	*P2RY12*	1.24E-19	-0.038
**cg04813880**	16	21171067	2.37E-78	0.159	0.229	-0.070	*TMEM159*	0.215	-0.007
cg08905487	19	55013821	2.38E-78	0.384	0.483	-0.099	*LAIR2*	0.005	-0.019
cg17390918	5	10502512	2.53E-78	0.359	0.432	-0.073	NA	1.23E-07	-0.030
cg04238983	11	65210600	2.56E-78	0.367	0.447	-0.081	NA	4.86E-04	-0.022
cg11906021	17	47467221	2.63E-78	0.788	0.736	0.052	NA	0.027	0.009
cg01170124	12	108962900	3.02E-78	0.818	0.755	0.063	*ISCU*	1.24E-05	0.021
cg03727968	1	167451830	3.37E-78	0.819	0.761	0.058	*CD247*	8.97E-13	0.032
cg24032269	5	149756966	3.63E-78	0.835	0.784	0.051	*TCOF1*	0.009	0.010
cg12081645	4	157920476	3.68E-78	0.263	0.340	-0.077	NA	1.11E-09	-0.037
cg02827175	7	1986245	3.75E-78	0.577	0.666	-0.089	*MAD1L1*	2.15E-06	-0.029
cg18739675	2	43041125	3.80E-78	0.231	0.303	-0.071	NA	1.52E-15	-0.043
cg02853355	16	85132373	3.86E-78	0.453	0.537	-0.084	*FAM92B*	3.49E-07	-0.031
cg27076223	4	40632858	3.87E-78	0.310	0.380	-0.069	*RBM47*	1.83E-04	-0.019
cg21691367	6	151325642	3.92E-78	0.322	0.402	-0.080	*MTHFD1L*	1.25E-14	-0.046
cg25941354	2	218989983	3.97E-78	0.301	0.355	-0.054	*CXCR2*	4.33E-23	-0.037
cg14278300	1	167486978	3.98E-78	0.822	0.767	0.055	*CD247*	6.66E-05	0.019
cg02343275	1	212589072	4.06E-78	0.237	0.294	-0.056	*TMEM206*	2.14E-11	-0.030
cg00980058	1	233248709	4.34E-78	0.182	0.233	-0.052	*PCNXL2*	1.03E-09	-0.025
cg24949488	10	98064362	4.44E-78	0.833	0.780	0.053	*DNTT*	0.007	0.010
cg10552964	6	35991802	4.52E-78	0.410	0.484	-0.074	*SLC26A8*	4.02E-04	-0.019
cg00084577	16	30721139	4.54E-78	0.517	0.599	-0.082	*SRCAP*	4.70E-06	-0.026
cg24392132	12	6753626	4.64E-78	0.182	0.234	-0.052	*ACRBP*	1.01E-08	-0.023
cg24304425	19	840985	4.83E-78	0.303	0.367	-0.064	*PRTN3*	1.95E-06	-0.023
cg14619949	5	76116169	5.29E-78	0.393	0.476	-0.082	*F2RL1*	7.10E-09	-0.037
cg01441777	22	38714416	5.46E-78	0.407	0.463	-0.056	*CSNK1E*	2.47E-16	-0.030
cg13878360	3	64069147	5.61E-78	0.290	0.354	-0.064	*LOC10028787!*	6.60E-08	-0.028
cg21598343	13	114261986	5.74E-78	0.357	0.415	-0.057	*TFDP1*	2.67E-05	-0.017
cg03296935	3	195946851	5.76E-78	0.237	0.293	-0.055	*SLC51A*	4.41E-11	-0.027
cg08468732	2	95722029	5.97E-78	0.716	0.658	0.058	NA	7.13E-10	0.026
cg16422316	16	71844250	6.11E-78	0.412	0.502	-0.090	*AP1G1*	0.004	-0.018
cg11723077	6	158508188	6.48E-78	0.327	0.400	-0.073	*SYNJ2*	9.25E-04	-0.018
cg10028625	3	172494364	6.52E-78	0.420	0.516	-0.096	*ECT2*	4.32E-05	-0.029
cg12487088	3	9958686	6.72E-78	0.238	0.299	-0.061	*IL17RC*	2.38E-05	-0.018
cg14030719	20	32266794	6.89E-78	0.769	0.704	0.065	*E2F1*	0.001	0.016

cg04389994	10	74590936	6.94E-78	0.551	0.629	-0.077	*MCU*	3.95E-09	-0.036
cg05141432	3	81812214	7.26E-78	0.267	0.332	-0.064	*GBE1*	1.65E-05	-0.022
cg19905757	15	68924127	7.33E-78	0.816	0.760	0.056	*CORO2B*	5.24E-05	0.017
cg17705615	19	47991237	7.48E-78	0.356	0.406	-0.051	*NAPA*	2.35E-09	-0.022
cg19295314	6	1635640	7.71E-78	0.409	0.469	-0.060	*GMDS*	2.53E-07	-0.023
cg14665690	1	155188904	8.03E-78	0.177	0.234	-0.056	*GBAP1*	1.31E-05	-0.020
cg11733958	17	17023057	8.07E-78	0.776	0.724	0.052	*MPRIP*	2.19E-09	0.025
cg04231085	16	85561302	8.47E-78	0.383	0.459	-0.076	NA	3.42E-05	-0.021
cg04536922	4	89978566	8.54E-78	0.224	0.303	-0.079	*FAM13A*	5.18E-08	-0.035
**cg13984928**	17	3704574	8.59E-78	0.383	0.481	-0.098	*ITGAE*	0.742	-0.003
cg16567676	4	95264074	9.32E-78	0.318	0.394	-0.076	*HPGDS*	6.27E-05	-0.025
cg04776231	7	77168113	9.51E-78	0.377	0.478	-0.101	*PTPN12*	8.55E-05	-0.028
cg04364261	2	219233650	9.61E-78	0.252	0.327	-0.075	NA	9.83E-05	-0.021
cg06238667	8	29086832	9.63E-78	0.763	0.693	0.070	*KIF13B*	4.66E-08	0.030
cg14838970	11	7533817	1.00E-77	0.301	0.369	-0.068	*PPFIBP2*	4.38E-07	-0.024
cg12077963	17	4079306	1.03E-77	0.766	0.683	0.084	*ANKFY1*	2.31E-05	0.025
cg13422921	9	37788648	1.07E-77	0.395	0.482	-0.086	NA	1.80E-11	-0.045
cg04958236	6	74276973	1.11E-77	0.565	0.632	-0.067	NA	3.51E-09	-0.028
cg05083852	16	81480610	1.12E-77	0.267	0.333	-0.066	*CMIP*	3.23E-04	-0.019
cg13289202	11	34322226	1.14E-77	0.269	0.336	-0.067	*ABTB2*	1.56E-06	-0.025
cg14132103	11	60680165	1.17E-77	0.742	0.687	0.055	*TMEM109*	4.59E-07	0.020
cg14665366	4	90227740	1.18E-77	0.817	0.757	0.060	*GPRIN3*	7.64E-10	0.029
cg19716090	22	17560707	1.21E-77	0.538	0.617	-0.079	NA	4.09E-11	-0.040
cg13466002	17	4621252	1.25E-77	0.430	0.519	-0.089	*ARRB2*	6.14E-05	-0.025
cg11804928	17	38220298	1.25E-77	0.256	0.324	-0.069	*THRA*	2.41E-08	-0.029
cg15339435	11	128169380	1.28E-77	0.781	0.731	0.050	NA	1.09E-08	0.022
cg20438472	3	184293365	1.35E-77	0.473	0.543	-0.070	*EPHB3*	2.52E-10	-0.033
cg06788267	1	95258162	1.56E-77	0.236	0.298	-0.062	NA	3.49E-13	-0.034
cg06154903	11	64642558	1.64E-77	0.886	0.826	0.061	*EHD1*	0.014	0.011
cg00686823	3	127311038	1.74E-77	0.293	0.385	-0.092	*TPRA1*	1.24E-04	-0.027
cg05240166	16	21171239	1.74E-77	0.356	0.443	-0.087	*TMEM159*	1.08E-07	-0.037
cg22628286	19	35643121	1.86E-77	0.200	0.260	-0.060	*FXYD7*	2.64E-11	-0.031
cg19439123	17	17687621	1.89E-77	0.199	0.261	-0.062	*RAI1*	2.20E-05	-0.020
cg14939082	10	104535990	1.90E-77	0.246	0.306	-0.060	*WBP1L*	5.39E-06	-0.020
cg19462635	11	121237843	1.90E-77	0.365	0.442	-0.077	NA	6.25E-13	-0.043
**cg15401418**	17	75316383	1.92E-77	0.399	0.480	-0.080	*SEPT9*	0.136	-0.009
cg20945221	1	28423646	2.01E-77	0.431	0.493	-0.062	NA	5.84E-12	-0.032
cg26472802	21	45713719	2.07E-77	0.782	0.702	0.080	*AIRE*	2.33E-10	0.038
cg13581015	12	1769824	2.13E-77	0.353	0.450	-0.097	NA	5.08E-05	-0.030
cg03916225	1	111768837	2.50E-77	0.762	0.699	0.062	*CHI3L2*	1.04E-04	0.019
cg17952262	4	683240	2.65E-77	0.271	0.324	-0.053	*MFSD7*	2.19E-10	-0.024
cg11363527	4	72125918	2.67E-77	0.220	0.277	-0.057	*SLC4A4*	1.20E-11	-0.031
cg08622923	12	122712116	2.78E-77	0.762	0.711	0.051	*DIABLO*	2.78E-05	0.016
cg16246489	5	134735675	2.88E-77	0.312	0.412	-0.100	*H2AFY*	4.02E-12	-0.052
cg22854223	11	44586505	2.99E-77	0.180	0.233	-0.053	*CD82*	6.99E-10	-0.025
cg21253130	13	32609104	3.06E-77	0.427	0.493	-0.065	*FRY*	8.62E-07	-0.023
cg04936009	2	223846845	3.07E-77	0.466	0.549	-0.082	NA	4.48E-06	-0.028
cg03341377	3	39309355	3.13E-77	0.733	0.676	0.057	*CX3CR1*	3.05E-05	0.018
cg24044501	10	72056549	3.17E-77	0.199	0.251	-0.052	NA	8.36E-06	-0.017
cg05604874	17	80200785	3.18E-77	0.353	0.448	-0.094	*CSNK1D*	3.91E-06	-0.033
cg05894970	3	119041204	3.28E-77	0.246	0.318	-0.073	*ARHGAP31*	7.70E-20	-0.053
cg20086579	19	11665102	3.33E-77	0.715	0.648	0.066	*ELOF1*	4.06E-08	0.029
cg27099293	10	71895572	3.44E-77	0.551	0.618	-0.066	NA	0.001	-0.015
cg14088811	11	47399994	3.57E-77	0.312	0.402	-0.090	*SPI1*	2.89E-04	-0.024
cg24404823	1	247496053	3.84E-77	0.333	0.398	-0.065	*ZNF496*	5.24E-14	-0.038
cg08091707	19	859554	3.85E-77	0.190	0.250	-0.060	*CFD*	1.32E-07	-0.025
cg00990740	21	39871287	3.92E-77	0.240	0.301	-0.062	*ERG*	3.83E-06	-0.022
cg20048655	2	7975491	3.94E-77	0.277	0.342	-0.066	NA	2.28E-05	-0.023
cg21648069	12	1157718	3.99E-77	0.352	0.437	-0.085	*ERC1*	4.09E-06	-0.029
cg26406891	12	125002940	4.01E-77	0.262	0.327	-0.065	*NCOR2*	1.34E-11	-0.033
cg24126180	7	128580582	4.08E-77	0.208	0.277	-0.069	*IRF5*	9.40E-07	-0.028
cg09848074	7	139172610	4.17E-77	0.237	0.298	-0.061	NA	4.49E-06	-0.022
cg08215318	16	86016387	4.64E-77	0.422	0.497	-0.074	NA	1.16E-04	-0.018
cg25117600	4	10023201	4.75E-77	0.254	0.338	-0.085	*SLC2A9*	2.27E-12	-0.048
cg04340435	13	114145973	5.07E-77	0.277	0.339	-0.062	*DCUN1D2*	0.021	-0.011
cg14022913	4	178229651	5.51E-77	0.333	0.416	-0.084	*NEIL3*	6.16E-08	-0.033
cg24493367	4	106635507	5.55E-77	0.310	0.383	-0.074	*GSTCD*	2.46E-04	-0.021
cg02849956	19	4634827	5.63E-77	0.219	0.279	-0.061	NA	4.52E-19	-0.042
cg03366884	17	8844506	5.64E-77	0.693	0.638	0.055	*PIK3R5*	0.015	0.010
cg13728797	8	126465410	5.89E-77	0.537	0.618	-0.081	NA	3.57E-05	-0.025
cg24232444	13	99545448	6.07E-77	0.228	0.299	-0.071	*DOCK9*	9.17E-06	-0.024
cg21088259	17	81039990	6.19E-77	0.444	0.520	-0.076	*METRNL*	0.006	-0.016
cg09942999	1	32707389	6.21E-77	0.217	0.280	-0.063	NA	3.08E-09	-0.028

cg23304647	7	2778058	6.35E-77	0.270	0.347	-0.077	*GNA12*	0.040	-0.012
cg05494008	8	145579285	7.31E-77	0.229	0.280	-0.051	*TMEM249*	1.60E-11	-0.025
cg17679987	8	106535882	7.45E-77	0.764	0.707	0.057	*ZFPM2*	2.03E-06	0.021
cg17477578	8	129552914	7.74E-77	0.796	0.731	0.065	NA	6.43E-09	0.031
cg10266490	1	55013709	7.81E-77	0.241	0.314	-0.073	*ACOT11*	2.50E-13	-0.043
cg01937819	9	130616110	8.02E-77	0.194	0.250	-0.056	*ENG*	4.38E-13	-0.033
cg14236758	9	137252129	8.03E-77	0.430	0.494	-0.064	*RXRA*	5.28E-10	-0.027
cg08130265	15	77519170	8.04E-77	0.408	0.486	-0.079	*PEAK1*	1.19E-08	-0.034
cg01008256	7	100463985	8.48E-77	0.280	0.354	-0.074	*TRIP6*	3.22E-09	-0.032
cg04924511	3	10334731	8.49E-77	0.211	0.275	-0.064	*GHRLOS*	9.90E-04	-0.017
**cg24800754**	19	6234327	9.24E-77	0.273	0.334	-0.061	*MLLT1*	0.050	-0.009
cg04528931	2	240162187	9.80E-77	0.207	0.281	-0.074	*HDAC4*	3.17E-05	-0.025
cg26003388	17	76129533	1.03E-76	0.866	0.816	0.050	*TMC8*	2.81E-08	0.022
cg20449670	1	153498959	1.05E-76	0.411	0.509	-0.098	NA	0.001	-0.022
cg03154580	2	121009176	1.10E-76	0.369	0.437	-0.068	*RALB*	2.44E-07	-0.028
cg26898932	17	28442480	1.12E-76	0.404	0.509	-0.105	*NSRP1*	0.003	-0.022
cg25948980	11	76777325	1.12E-76	0.184	0.241	-0.058	*CAPN5*	1.71E-07	-0.023
cg05306109	1	226924257	1.16E-76	0.773	0.718	0.055	*ITPKB*	3.11E-09	0.025
cg25843426	7	1497307	1.17E-76	0.304	0.367	-0.063	*MICALL2*	2.87E-09	-0.026
cg16646054	5	150157726	1.26E-76	0.449	0.535	-0.086	*C5orf62*	2.76E-19	-0.058
cg11162888	13	40762188	1.27E-76	0.787	0.731	0.056	*LINC00332*	2.50E-13	0.031
cg27380499	2	219233446	1.43E-76	0.252	0.314	-0.062	NA	3.12E-06	-0.021
cg16045731	14	24777708	1.43E-76	0.507	0.566	-0.059	*CIDEB*	1.63E-06	-0.020
cg05251269	16	2083128	1.43E-76	0.285	0.342	-0.057	*SLC9A3R2*	2.64E-09	-0.023
cg05315365	16	82096597	1.49E-76	0.610	0.680	-0.070	*HSD17B2*	1.46E-07	-0.025
cg26692749	1	24861919	1.49E-76	0.758	0.686	0.072	*RCAN3*	2.86E-10	0.035
cg25467652	1	11795976	1.49E-76	0.203	0.259	-0.056	*AGTRAP*	3.06E-15	-0.033
cg02670686	16	57125674	1.55E-76	0.201	0.258	-0.057	*CPNE2*	3.42E-13	-0.030
cg02430584	7	102065866	1.60E-76	0.393	0.444	-0.051	*LOC10063092.*	2.32E-05	-0.015
cg13451886	17	33568791	1.61E-76	0.810	0.751	0.059	*SLFN5*	1.15E-10	0.031
cg24725263	12	56101328	1.62E-76	0.170	0.232	-0.062	*ITGA7*	7.43E-06	-0.022
cg05656486	1	161171383	1.69E-76	0.252	0.331	-0.079	*NDUFS2*	5.80E-05	-0.025
cg21775570	1	2171834	1.76E-76	0.255	0.320	-0.066	*SKI*	0.001	-0.016
cg17824540	2	161260031	1.77E-76	0.770	0.717	0.053	*RBMS1*	0.015	0.010
cg23690893	19	14671371	1.80E-76	0.374	0.442	-0.068	*TECR*	2.39E-09	-0.030
cg03726817	12	62650767	1.89E-76	0.204	0.269	-0.065	NA	5.62E-06	-0.022
cg04911669	6	73697178	2.05E-76	0.464	0.558	-0.094	*KCNQ5*	1.73E-09	-0.042
cg23007665	7	43690247	2.16E-76	0.673	0.591	0.082	*COA1*	1.22E-12	0.045
cg26819753	6	33265313	2.32E-76	0.286	0.337	-0.050	*RGL2*	9.99E-11	-0.024
cg16469046	2	99062999	2.38E-76	0.771	0.703	0.068	*INPP4A*	5.10E-15	0.042
cg24386894	8	8863572	2.40E-76	0.566	0.641	-0.075	*ERI1*	8.61E-06	-0.025
cg26405020	15	91427363	2.51E-76	0.265	0.328	-0.063	*FES*	0.007	-0.012
cg14665413	4	38859728	2.62E-76	0.203	0.266	-0.063	*TLR6*	1.08E-12	-0.036
cg02297838	13	92002454	2.66E-76	0.230	0.297	-0.067	*MIR17HG*	4.86E-23	-0.052
cg12691572	10	114574959	2.86E-76	0.247	0.323	-0.077	*VTI1A*	0.002	-0.020
cg17928286	1	205414220	2.92E-76	0.277	0.344	-0.068	NA	0.005	-0.013
cg00727912	20	61583736	3.01E-76	0.228	0.281	-0.053	*SLC17A9*	1.64E-13	-0.030
cg25779483	4	89978300	3.07E-76	0.231	0.302	-0.072	*FAM13A*	0.036	-0.012
cg10082165	14	25043758	3.07E-76	0.474	0.525	-0.052	*CTSG*	8.59E-06	-0.016
cg09934892	16	3559551	3.17E-76	0.734	0.676	0.058	*CLUAP1*	1.66E-06	0.020
cg06654628	5	150018914	3.19E-76	0.264	0.323	-0.059	*SYNPO*	4.01E-11	-0.027
cg25427871	11	2920735	3.20E-76	0.281	0.337	-0.056	*SLC22A18AS*	8.12E-09	-0.025
**cg08776660**	16	1670473	3.21E-76	0.789	0.730	0.059	*CRAMP1L*	0.109	0.007
cg00423969	2	97359879	3.56E-76	0.253	0.305	-0.052	*FER1L5*	5.49E-10	-0.024
**cg03892838**	2	238599734	3.75E-76	0.186	0.256	-0.070	*LRRFIP1*	0.221	-0.007
cg14328641	11	59822727	3.80E-76	0.290	0.372	-0.081	*MS4A3*	1.14E-07	-0.032
cg07786220	17	78683082	3.97E-76	0.287	0.363	-0.077	*RPTOR*	3.63E-05	-0.024
cg27535502	15	91395896	4.39E-76	0.800	0.745	0.055	NA	8.75E-08	0.022
cg21593001	12	113531060	4.40E-76	0.345	0.405	-0.060	*DTX1*	1.18E-12	-0.028
cg07658508	4	973177	4.44E-76	0.378	0.473	-0.094	*SLC26A1*	0.013	-0.019
cg22027204	6	36216248	4.49E-76	0.225	0.292	-0.067	*PNPLA1*	5.51E-14	-0.040
cg25016112	8	142192718	4.51E-76	0.202	0.270	-0.068	*DENND3*	3.44E-05	-0.024
cg25627098	17	7328734	4.81E-76	0.190	0.242	-0.052	*C17orf74*	1.82E-07	-0.021
**cg07474797**	16	89185916	4.87E-76	0.314	0.377	-0.064	*ACSF3*	0.073	-0.008
cg16568681	11	33758505	4.92E-76	0.276	0.353	-0.077	*CD59*	4.32E-09	-0.035
cg14780449	11	44578801	4.98E-76	0.557	0.613	-0.056	NA	8.62E-08	-0.023
cg19447962	17	17628656	5.70E-76	0.315	0.412	-0.097	*RAI1*	0.010	-0.020
cg05036173	2	108994528	5.71E-76	0.212	0.269	-0.057	*SULT1C4*	5.13E-07	-0.021
cg08606497	8	28244769	5.87E-76	0.459	0.529	-0.069	*ZNF395*	1.98E-16	-0.047
cg07205154	1	176147105	6.09E-76	0.814	0.763	0.051	*RFWD2*	2.85E-05	0.017
cg11905488	12	10123604	6.09E-76	0.471	0.547	-0.076	*CLEC12A*	2.45E-07	-0.030
cg14854517	12	48152428	6.19E-76	0.287	0.367	-0.080	*RAPGEF3*	9.09E-06	-0.028
cg02455383	22	27014116	6.60E-76	0.339	0.402	-0.062	*CRYBB1*	1.83E-19	-0.043

cg17142183	2	102608192	6.61E-76	0.338	0.424	-0.086	*IL1R2*	2.27E-07	-0.034
cg05902522	3	149348590	6.70E-76	0.542	0.608	-0.066	*WWTR1*	5.69E-06	-0.022
cg25375916	3	155570275	6.75E-76	0.245	0.310	-0.065	*SLC33A1*	5.66E-08	-0.028
cg24219974	6	14729722	7.09E-76	0.800	0.746	0.054	NA	3.83E-13	0.026
cg02501827	11	11862879	7.51E-76	0.198	0.263	-0.064	*USP47*	1.88E-07	-0.026
cg14788242	3	127773954	7.66E-76	0.351	0.431	-0.080	*SEC61A1*	5.57E-06	-0.025
cg21762695	7	36022695	7.79E-76	0.382	0.471	-0.089	NA	2.37E-09	-0.040
cg21834463	6	134538515	7.94E-76	0.402	0.490	-0.088	*SGK1*	2.80E-09	-0.039
cg21470711	3	195995846	8.00E-76	0.233	0.303	-0.070	*PCYT1A*	8.32E-08	-0.031
cg06012428	6	157477204	8.14E-76	0.640	0.698	-0.058	*ARID1B*	7.98E-10	-0.027
cg03432176	2	160655066	8.23E-76	0.327	0.416	-0.089	*CD302*	1.08E-05	-0.031
cg14844236	12	123753212	8.25E-76	0.312	0.380	-0.068	*CDK2AP1*	5.60E-04	-0.017
cg04859918	10	71893017	9.29E-76	0.436	0.497	-0.060	*AIFM2*	1.03E-09	-0.028
cg09971811	20	24930099	9.72E-76	0.293	0.370	-0.077	*CST7*	3.14E-15	-0.047
cg15743985	19	35819809	9.82E-76	0.283	0.364	-0.080	*CD22*	3.94E-06	-0.031
cg24010658	8	144544041	1.01E-75	0.829	0.777	0.051	*ZC3H3*	6.98E-06	0.018
cg06218079	17	80834228	1.02E-75	0.433	0.543	-0.110	*TBCD*	1.52E-06	-0.036
cg22052056	20	31351813	1.03E-75	0.624	0.690	-0.066	*DNMT3B*	9.00E-23	-0.049
cg27571769	4	185704063	1.13E-75	0.178	0.235	-0.057	*ACSL1*	1.66E-04	-0.017
cg12063847	6	158184542	1.16E-75	0.292	0.343	-0.051	NA	7.96E-09	-0.022
cg04654716	5	74162924	1.16E-75	0.466	0.521	-0.056	*FAM169A*	2.35E-17	-0.037
cg24347562	19	839678	1.17E-75	0.342	0.407	-0.065	*PRTN3*	1.85E-10	-0.030
cg21308575	19	6887544	1.19E-75	0.220	0.281	-0.061	*EMR1*	0.002	-0.015
cg13299325	6	447777	1.26E-75	0.705	0.636	0.069	NA	0.001	0.016
cg05644602	2	74795186	1.27E-75	0.496	0.578	-0.082	*C2orf65*	9.60E-07	-0.030
cg18347010	17	55888532	1.29E-75	0.215	0.279	-0.064	NA	3.96E-04	-0.017
cg23829102	4	124476694	1.32E-75	0.188	0.251	-0.063	NA	3.48E-16	-0.042
cg24351694	8	144655447	1.33E-75	0.237	0.292	-0.055	*C8orf73*	7.19E-09	-0.025
cg03658557	4	96012317	1.44E-75	0.228	0.290	-0.062	*BMPR1B*	1.51E-11	-0.035
cg17554875	5	179520998	1.48E-75	0.243	0.300	-0.057	NA	3.37E-06	-0.021
cg22051146	5	177895047	1.49E-75	0.285	0.359	-0.074	*COL23A1*	5.54E-07	-0.030
cg22027399	19	15217635	1.49E-75	0.274	0.336	-0.061	*SYDE1*	2.76E-07	-0.026
cg20995188	2	106713059	1.49E-75	0.798	0.741	0.057	*UXS1*	1.87E-12	0.029
cg05081167	6	156967138	1.50E-75	0.202	0.266	-0.064	NA	1.59E-17	-0.044
cg23570810	11	315102	1.57E-75	0.490	0.686	-0.196	*IFITM1*	6.14E-38	-0.141
cg24895173	17	33825374	1.58E-75	0.324	0.402	-0.078	NA	3.87E-07	-0.031
cg20608306	11	116969690	1.63E-75	0.210	0.264	-0.054	*SIK3*	1.25E-06	-0.021
cg25066857	2	85921438	1.66E-75	0.792	0.738	0.054	*GNLY*	0.007	0.011
cg14507533	13	114066074	1.71E-75	0.548	0.625	-0.077	NA	4.19E-09	-0.031
cg11276093	10	95123774	1.75E-75	0.454	0.527	-0.073	*MYOF*	5.69E-09	-0.033
cg02127980	9	137252116	1.89E-75	0.367	0.432	-0.065	*RXRA*	4.58E-08	-0.025
cg01606027	22	31607212	1.94E-75	0.270	0.345	-0.075	*LIMK2*	2.50E-06	-0.028
cg09455342	17	80346849	2.00E-75	0.465	0.549	-0.084	NA	4.32E-07	-0.031
cg05271255	17	79821342	2.10E-75	0.188	0.249	-0.061	NA	2.96E-09	-0.028
cg26706803	16	50293530	2.13E-75	0.254	0.338	-0.084	NA	0.001	-0.023
cg05783185	8	119121281	2.13E-75	0.303	0.377	-0.074	*EXT1*	6.98E-08	-0.032
**cg19030682**	17	79218991	2.14E-75	0.819	0.757	0.062	*SLC38A10*	0.369	0.005
cg19008371	11	126226766	2.18E-75	0.250	0.309	-0.059	*ST3GAL4*	2.29E-10	-0.030
cg27058988	15	67200879	2.22E-75	0.179	0.245	-0.066	NA	1.04E-13	-0.041
cg11842502	6	111862202	2.31E-75	0.415	0.503	-0.089	*TRAF3IP2-AS1*	0.002	-0.020
cg11224765	22	50971109	2.41E-75	0.236	0.324	-0.088	*ODF3B*	7.44E-08	-0.037
cg01233897	5	56060091	2.48E-75	0.258	0.323	-0.064	NA	1.01E-15	-0.041
cg20391948	16	89408367	2.55E-75	0.712	0.654	0.058	*ANKRD11*	3.73E-06	0.020
cg05981785	2	74210960	2.67E-75	0.367	0.427	-0.061	NA	3.11E-04	-0.017
cg10056728	12	56497073	2.86E-75	0.735	0.683	0.052	*ERBB3*	1.26E-05	0.017
cg19989043	6	47468193	2.88E-75	0.291	0.363	-0.072	*CD2AP*	6.60E-04	-0.019
cg05580073	22	24803248	2.89E-75	0.306	0.379	-0.073	*SPECC1L*	4.21E-14	-0.044
cg02219601	14	35835511	2.94E-75	0.244	0.298	-0.054	NA	8.51E-16	-0.035
cg16649560	16	27338391	3.00E-75	0.172	0.227	-0.056	*IL4R*	2.66E-05	-0.019
cg20391895	4	10686394	3.20E-75	0.245	0.301	-0.056	*CLNK*	2.09E-13	-0.033
cg19957411	11	63273333	3.60E-75	0.350	0.410	-0.060	*LGALS12*	5.47E-07	-0.022
cg06526620	11	94278324	3.70E-75	0.446	0.522	-0.076	*FUT4*	1.82E-06	-0.025
cg22033586	2	157292113	3.73E-75	0.326	0.424	-0.098	*GPD2*	0.005	-0.021
cg12599598	4	185923887	4.17E-75	0.407	0.492	-0.084	NA	4.51E-07	-0.032
cg00874051	21	35570816	4.43E-75	0.289	0.373	-0.084	NA	0.003	-0.021
**cg26396370**	2	10194752	4.64E-75	0.505	0.601	-0.096	*KLF11*	0.073	-0.011
cg08423562	1	38466544	4.82E-75	0.254	0.320	-0.065	*FHL3*	1.03E-05	-0.023
cg07298473	11	47279183	4.87E-75	0.265	0.338	-0.074	*NR1H3*	1.13E-05	-0.025
cg20907456	11	57405372	4.95E-75	0.228	0.281	-0.052	NA	4.74E-09	-0.023
cg23955417	1	26880928	4.98E-75	0.214	0.280	-0.067	*RPS6KA1*	1.88E-14	-0.039
cg00207280	8	126441393	5.22E-75	0.225	0.283	-0.058	*TRIB1*	4.02E-09	-0.027
cg00546897	21	45232232	6.03E-75	0.470	0.527	-0.057	*LOC284837*	6.62E-06	-0.019
cg16000989	4	41983716	6.47E-75	0.449	0.532	-0.083	*DCAF4L1*	2.35E-06	-0.031

cg24231804	15	67316861	6.74E-75	0.207	0.269	-0.062	NA	3.04E-20	-0.047
cg21727223	17	66755770	6.86E-75	0.347	0.419	-0.072	NA	1.38E-11	-0.038
cg06665622	6	46293571	7.15E-75	0.807	0.754	0.053	*RCAN2*	6.82E-04	0.014
cg15699693	5	150054944	7.24E-75	0.513	0.604	-0.091	*MYOZ3*	3.39E-04	-0.023
cg07643696	12	25844221	7.43E-75	0.346	0.419	-0.073	NA	1.36E-09	-0.035
cg19265103	12	51784644	7.61E-75	0.201	0.252	-0.052	*GALNT6*	7.83E-10	-0.025
cg22396959	1	57013771	7.80E-75	0.457	0.534	-0.077	*PPAP2B*	1.02E-08	-0.032
cg25050392	6	24933103	8.30E-75	0.769	0.690	0.079	NA	3.29E-16	0.051
cg20267828	6	128222390	8.70E-75	0.778	0.721	0.058	*THEMIS*	3.25E-09	0.026
cg15797314	17	65437724	9.11E-75	0.806	0.751	0.055	*PITPNC1*	4.35E-07	0.023
cg22353097	1	116521539	9.41E-75	0.405	0.487	-0.081	*SLC22A15*	1.14E-08	-0.034
cg13390284	1	65531864	9.55E-75	0.418	0.485	-0.067	NA	4.99E-09	-0.032
cg04172533	16	50743027	9.67E-75	0.253	0.315	-0.062	*NOD2*	6.49E-14	-0.037
cg15316716	8	21769797	1.00E-74	0.746	0.687	0.059	*DOK2*	0.003	0.012
cg24902461	10	121031392	1.01E-74	0.327	0.406	-0.078	*GRK5*	3.00E-08	-0.034
cg11481582	10	74080805	1.02E-74	0.181	0.240	-0.059	NA	1.39E-05	-0.021
cg17228105	21	39871301	1.03E-74	0.432	0.511	-0.078	*ERG*	6.55E-09	-0.034
cg11227278	2	23749277	1.09E-74	0.727	0.667	0.060	*KLHL29*	2.04E-09	0.027
cg14342532	2	233916534	1.12E-74	0.281	0.361	-0.080	NA	2.48E-06	-0.031
cg10573932	20	17549697	1.13E-74	0.258	0.314	-0.056	*DSTN*	7.81E-06	-0.019
cg05768005	17	8094486	1.14E-74	0.252	0.316	-0.064	*C17orf59*	1.25E-05	-0.022
cg06639585	6	36236936	1.14E-74	0.420	0.475	-0.055	*PNPLA1*	4.62E-08	-0.022
cg02137956	7	157092982	1.16E-74	0.461	0.521	-0.060	NA	0.006	-0.012
cg00791854	1	154392070	1.16E-74	0.233	0.291	-0.058	*IL6R*	1.85E-10	-0.029
cg07881210	17	398090	1.22E-74	0.256	0.319	-0.063	NA	1.34E-08	-0.028
cg15100426	2	219187432	1.28E-74	0.520	0.598	-0.077	*PNKD*	1.80E-04	-0.022
cg01042641	16	1575979	1.34E-74	0.620	0.559	0.061	*IFT140*	1.15E-09	0.029
cg25451120	17	47287444	1.36E-74	0.704	0.650	0.054	*GNGT2*	3.16E-06	0.017
cg03989987	2	113885277	1.45E-74	0.222	0.288	-0.066	*IL1RN*	1.13E-10	-0.033
cg27214730	3	184297522	1.49E-74	0.190	0.253	-0.063	*EPHB3*	3.35E-06	-0.025
cg19339902	4	8292261	1.51E-74	0.313	0.383	-0.070	*HTRA3*	1.55E-09	-0.033
cg19378631	10	22606072	1.55E-74	0.188	0.253	-0.065	*COMMD3*	7.61E-07	-0.024
cg15010903	17	76850256	1.56E-74	0.458	0.541	-0.083	*TIMP2*	0.002	-0.019
cg10296205	6	159084032	1.61E-74	0.272	0.356	-0.084	*SYTL3*	0.001	-0.022
cg02795981	10	81045119	1.67E-74	0.796	0.743	0.054	*ZMIZ1*	4.01E-05	0.018
cg22798362	17	30850485	1.67E-74	0.843	0.782	0.061	*MYO1D*	7.92E-13	0.034
cg13429423	22	44568775	1.71E-74	0.290	0.357	-0.066	*PARVG*	4.60E-05	-0.020
**cg05422796**	18	5297381	1.72E-74	0.366	0.451	-0.086	*ZFP161*	0.148	-0.009
cg10491452	16	88866627	1.74E-74	0.214	0.288	-0.074	NA	3.90E-07	-0.030
cg01141721	20	36010619	1.85E-74	0.421	0.508	-0.087	*SRC*	9.39E-07	-0.033
cg12847502	2	43437911	1.88E-74	0.357	0.410	-0.053	NA	2.00E-05	-0.014
cg20923498	2	99096920	1.95E-74	0.770	0.714	0.057	*INPP4A*	2.19E-11	0.030
cg25769469	5	71643841	2.01E-74	0.299	0.375	-0.076	*PTCD2*	1.52E-05	-0.025
cg26620147	12	58210716	2.05E-74	0.274	0.348	-0.074	*AVIL*	1.85E-08	-0.032
cg05345285	19	2215718	2.22E-74	0.866	0.815	0.051	*DOT1L*	5.99E-10	0.025
cg06305891	3	195897904	2.24E-74	0.299	0.367	-0.067	NA	0.003	-0.015
cg12488187	12	65671664	2.28E-74	0.437	0.520	-0.083	*MSRB3*	4.58E-04	-0.022
cg21340621	12	122020166	2.28E-74	0.286	0.346	-0.060	*KDM2B*	5.35E-06	-0.021
cg13241645	3	52235959	2.38E-74	0.491	0.566	-0.075	*ALAS1*	9.41E-12	-0.039
cg26954951	6	167507568	2.40E-74	0.607	0.553	0.054	NA	5.35E-12	0.026
cg25155064	11	118100782	2.42E-74	0.724	0.653	0.071	*MPZL3*	1.28E-11	0.039
cg12004276	11	33913586	2.52E-74	0.167	0.228	-0.061	*LMO2*	6.63E-04	-0.017
cg14356919	21	46890997	2.62E-74	0.793	0.726	0.067	*COL18A1*	1.07E-05	0.024
cg10274029	16	85981336	2.64E-74	0.636	0.561	0.075	NA	1.38E-05	0.025
cg12016746	11	68084453	2.71E-74	0.396	0.495	-0.099	*LRP5*	1.09E-05	-0.034
cg05383910	7	37431792	2.71E-74	0.219	0.285	-0.066	*ELMO1*	1.86E-08	-0.032
cg08463297	17	19976435	2.80E-74	0.219	0.292	-0.073	*SPECC1*	0.006	-0.018
**cg17566541**	11	1912287	2.82E-74	0.285	0.352	-0.068	*LSP1*	0.126	-0.007
cg20069407	11	121460973	2.95E-74	0.226	0.297	-0.071	*SORL1*	9.36E-16	-0.047
cg14789828	11	62201168	2.99E-74	0.180	0.232	-0.051	*AHNAK*	1.56E-09	-0.025
cg02749463	17	46646359	3.24E-74	0.345	0.424	-0.080	*HOXB3*	1.28E-04	-0.023
cg07671644	2	37545668	3.26E-74	0.246	0.312	-0.066	*PRKD3*	6.05E-08	-0.030
cg00336376	20	57798096	3.45E-74	0.191	0.247	-0.056	*ZNF831*	2.53E-04	-0.016
cg25256924	11	67205739	3.55E-74	0.682	0.598	0.084	*PTPRCAP*	1.58E-06	0.028
cg01867764	14	22986466	3.66E-74	0.729	0.674	0.056	NA	1.74E-06	0.020
cg09548403	1	232840764	3.69E-74	0.194	0.251	-0.058	NA	3.09E-11	-0.032
cg17661220	20	3693179	3.71E-74	0.301	0.359	-0.058	NA	5.85E-07	-0.021
cg07904073	10	74017122	3.73E-74	0.208	0.280	-0.072	NA	1.53E-09	-0.033
cg03371275	11	95974543	3.78E-74	0.450	0.534	-0.084	*MAML2*	7.52E-04	-0.021
cg19977428	19	35819985	4.70E-74	0.201	0.255	-0.055	*CD22*	4.23E-08	-0.024
cg15009198	2	97429502	4.75E-74	0.295	0.373	-0.077	*CNNM4*	8.41E-07	-0.030
cg08866695	12	109596187	5.01E-74	0.263	0.328	-0.065	*ACACB*	2.14E-06	-0.024
cg25564535	2	98329463	5.50E-74	0.764	0.709	0.055	*ZAP70*	3.21E-06	0.018

cg26885400	13	27842278	5.56E-74	0.315	0.377	-0.061	NA	1.78E-12	-0.034
cg08126118	13	43930918	5.62E-74	0.351	0.432	-0.081	*ENOX1*	0.020	-0.013
cg09360044	2	68557960	6.11E-74	0.813	0.758	0.055	NA	7.43E-04	0.014
cg19896824	11	128555529	6.35E-74	0.303	0.360	-0.057	*FLI1*	4.88E-07	-0.022
cg10175753	19	50219606	6.36E-74	0.299	0.361	-0.061	NA	1.90E-05	-0.021
cg10913077	3	57080664	6.43E-74	0.796	0.741	0.055	*ARHGEF3*	0.002	0.013
cg24182521	20	44541013	6.44E-74	0.216	0.282	-0.065	*PLTP*	2.85E-04	-0.019
cg01399219	7	44145110	6.57E-74	0.172	0.226	-0.054	*AEBP1*	4.18E-08	-0.024
cg05616969	2	118982661	6.70E-74	0.230	0.297	-0.067	NA	0.006	-0.014
cg05554494	16	3306685	7.01E-74	0.435	0.488	-0.052	*MEFV*	9.47E-04	-0.013
**cg13770399**	6	64281639	7.04E-74	0.236	0.311	-0.075	*PTP4A1*	0.672	0.002
cg05429448	3	101659630	7.13E-74	0.212	0.281	-0.070	NA	0.009	-0.015
cg09529437	16	24136792	7.17E-74	0.289	0.347	-0.058	*PRKCB*	6.08E-09	-0.027
cg04952446	2	219197867	7.31E-74	0.241	0.301	-0.060	*PNKD*	4.03E-06	-0.021
cg14224600	12	117477926	7.39E-74	0.432	0.498	-0.066	*TESC*	1.76E-08	-0.026
cg17420028	2	240142589	7.85E-74	0.572	0.641	-0.070	*HDAC4*	4.61E-07	-0.025
cg00029521	6	39272634	7.95E-74	0.772	0.721	0.052	*KCNK17*	0.002	0.011
cg04776489	22	22597965	8.41E-74	0.738	0.684	0.054	*VPREB1*	0.016	0.010
cg13725590	2	96809174	8.54E-74	0.750	0.690	0.060	*DUSP2*	0.002	0.016
cg12882572	17	35851204	8.86E-74	0.244	0.309	-0.064	*DUSP14*	2.79E-05	-0.020
**cg25752703**	3	128710390	9.00E-74	0.283	0.359	-0.076	*KIAA1257*	0.122	-0.010
cg12120750	3	11592700	9.56E-74	0.254	0.314	-0.060	*ATG7*	2.00E-06	-0.021
cg14644204	12	123518833	9.77E-74	0.277	0.334	-0.057	*PITPNM2*	0.007	-0.010
cg06474225	10	124228770	9.84E-74	0.284	0.353	-0.069	*HTRA1*	3.25E-10	-0.035
cg15686615	4	6781565	9.90E-74	0.453	0.504	-0.051	NA	6.34E-11	-0.025
cg03548857	19	35940174	1.01E-73	0.144	0.199	-0.055	*FFAR2*	1.60E-04	-0.018
cg22637435	8	141361108	1.01E-73	0.306	0.363	-0.057	*TRAPPC9*	4.91E-05	-0.018
cg18577167	13	74805590	1.01E-73	0.751	0.698	0.053	NA	2.40E-10	0.027
cg01616876	12	113544928	1.01E-73	0.237	0.309	-0.072	*RASAL1*	5.33E-13	-0.041
cg05650238	5	55215955	1.03E-73	0.291	0.358	-0.068	*IL31RA*	1.14E-10	-0.035
cg10475153	3	190024765	1.04E-73	0.634	0.576	0.057	*CLDN1*	3.43E-09	0.025
cg18865207	1	160765919	1.08E-73	0.417	0.361	0.056	*LY9*	4.69E-04	0.014
cg22570970	6	15401067	1.10E-73	0.247	0.321	-0.074	*JARID2*	0.047	-0.011
cg13537102	9	132360781	1.10E-73	0.461	0.520	-0.059	NA	7.30E-08	-0.024
cg23366234	21	45713704	1.13E-73	0.838	0.779	0.059	*AIRE*	8.34E-11	0.030
cg06640997	12	32081573	1.13E-73	0.797	0.736	0.060	NA	3.69E-11	0.029
cg18172358	1	76734569	1.14E-73	0.791	0.739	0.051	*ST6GALNAC3*	0.002	0.013
cg12491659	5	57786356	1.16E-73	0.244	0.303	-0.059	*GAPT*	8.76E-09	-0.028
cg13475822	3	9210116	1.16E-73	0.687	0.628	0.059	*SRGAP3*	6.29E-10	0.027
cg13062455	14	81978021	1.18E-73	0.345	0.421	-0.076	*SEL1L*	8.15E-06	-0.027
cg14992144	3	10334743	1.22E-73	0.207	0.266	-0.059	*GHRLOS*	1.90E-05	-0.020
cg20605134	6	15400462	1.26E-73	0.248	0.305	-0.058	*JARID2*	2.53E-04	-0.016
**cg10852154**	6	42421757	1.37E-73	0.274	0.340	-0.065	NA	0.053	-0.010
cg26395694	3	4783306	1.37E-73	0.173	0.227	-0.054	*ITPR1*	1.14E-05	-0.019
**cg03406844**	2	152220529	1.38E-73	0.450	0.531	-0.081	*TNFAIP6*	0.160	-0.008
cg08129583	2	127413367	1.40E-73	0.303	0.382	-0.079	*GYPC*	4.96E-05	-0.026
cg17813891	14	100532036	1.63E-73	0.765	0.703	0.062	*EVL*	4.35E-08	0.026
cg21932542	7	4850345	1.64E-73	0.434	0.520	-0.086	*RADIL*	4.36E-05	-0.027
cg14488317	11	3181446	1.64E-73	0.224	0.286	-0.061	*OSBPL5*	7.68E-07	-0.025
cg19713567	17	37895429	1.74E-73	0.202	0.258	-0.056	*GRB7*	0.013	-0.011
cg13765061	9	78830510	1.81E-73	0.296	0.370	-0.075	*PCSK5*	3.90E-07	-0.031
cg17825714	6	31695903	1.87E-73	0.386	0.450	-0.065	*DDAH2*	0.003	-0.014
cg08783253	17	40996565	1.88E-73	0.500	0.581	-0.081	*AOC2*	4.28E-05	-0.026
**cg02243157**	2	64047802	1.91E-73	0.477	0.562	-0.084	NA	0.146	-0.009
cg26868156	11	1892180	1.91E-73	0.730	0.661	0.069	*LSP1*	3.14E-10	0.031
cg23735745	1	230248121	1.95E-73	0.274	0.348	-0.074	*GALNT2*	5.30E-04	-0.021
cg15644413	1	36185939	2.06E-73	0.503	0.561	-0.058	*C1orf216*	4.89E-04	-0.017
cg22147446	20	4879261	2.07E-73	0.372	0.432	-0.059	*SLC23A2*	3.60E-09	-0.025
cg07417146	6	33400528	2.11E-73	0.541	0.627	-0.087	*SYNGAP1*	0.014	-0.015
cg05587627	11	118657363	2.20E-73	0.772	0.710	0.061	*DDX6*	2.59E-09	0.028
cg22458832	17	43506497	2.21E-73	0.213	0.267	-0.054	NA	8.27E-04	-0.015
cg12104698	7	139529130	2.21E-73	0.268	0.321	-0.054	*TBXAS1*	1.61E-05	-0.018
cg06494464	22	24992604	2.23E-73	0.560	0.632	-0.072	*GGT1*	0.025	-0.012
cg23500396	11	44896984	2.41E-73	0.665	0.726	-0.062	*TSPAN18*	1.85E-06	-0.021
cg05175803	11	44597119	2.46E-73	0.628	0.577	0.051	*CD82*	7.72E-06	0.016
cg21781157	20	47874111	2.58E-73	0.433	0.506	-0.073	*ZNFX1*	4.89E-05	-0.023
cg24500630	11	110436181	2.60E-73	0.296	0.364	-0.068	NA	3.36E-07	-0.030
cg17060157	17	78724127	2.77E-73	0.187	0.250	-0.063	*RPTOR*	3.16E-05	-0.021
cg22153728	1	25291584	2.79E-73	0.382	0.320	0.062	*RUNX3*	1.56E-05	0.019
cg05868564	11	123351497	2.83E-73	0.175	0.232	-0.057	NA	6.29E-08	-0.026
cg22229039	1	7842159	2.86E-73	0.247	0.327	-0.081	NA	5.21E-10	-0.039
cg18771737	3	43448041	3.10E-73	0.434	0.501	-0.067	*ANO10*	2.01E-12	-0.037
cg18802998	3	172444423	3.19E-73	0.360	0.436	-0.076	NA	8.95E-10	-0.035

cg14180696	3	194089473	3.22E-73	0.653	0.593	0.059	*LRRC15*	2.67E-05	0.019
cg20211629	18	48352490	3.47E-73	0.453	0.529	-0.076	*MRO*	8.10E-10	-0.036
cg13088432	8	9522664	3.50E-73	0.367	0.448	-0.081	*TNKS*	0.042	-0.014
cg24137511	19	18260330	3.61E-73	0.913	0.858	0.055	*MAST3*	1.00E-06	0.024
**cg18279004**	3	111802305	3.78E-73	0.453	0.541	-0.088	NA	0.063	-0.012
cg16572540	19	13947229	4.11E-73	0.342	0.394	-0.052	NA	4.24E-18	-0.033
cg07728874	11	118213272	4.12E-73	0.797	0.744	0.053	*CD3D*	1.19E-11	0.027
cg21579539	18	43751229	4.25E-73	0.544	0.606	-0.062	NA	1.48E-06	-0.023
**cg11809958**	12	32654929	4.26E-73	0.435	0.516	-0.080	*FGD4*	0.160	-0.008
cg23123250	17	4440116	4.40E-73	0.371	0.450	-0.079	*SPNS2*	1.76E-09	-0.036
cg00661777	7	106511741	4.49E-73	0.633	0.557	0.076	*PIK3CG*	3.99E-04	0.021
cg13178170	1	92270513	4.60E-73	0.626	0.574	0.053	*TGFBR3*	2.21E-05	0.017
cg23582982	11	47416109	4.77E-73	0.280	0.333	-0.053	NA	1.92E-06	-0.017
cg09308580	2	43405947	5.07E-73	0.323	0.378	-0.056	NA	1.50E-10	-0.028
cg06853894	1	12204096	5.15E-73	0.180	0.234	-0.054	*TNFRSF8*	0.005	-0.013
cg11866943	3	52529351	5.19E-73	0.193	0.244	-0.051	*STAB1*	0.001	-0.012
cg21726551	12	131590460	5.21E-73	0.700	0.630	0.070	*GPR133*	2.59E-09	0.030
cg04650322	7	119499	5.36E-73	0.264	0.317	-0.053	NA	7.93E-07	-0.020
cg12858593	8	22131899	5.47E-73	0.284	0.358	-0.073	*PIWIL2*	1.90E-12	-0.040
cg08944236	16	53242355	5.84E-73	0.262	0.330	-0.068	*CHD9*	0.003	-0.016
cg09915396	17	2907895	5.87E-73	0.273	0.333	-0.060	*RAP1GAP2*	6.25E-06	-0.019
cg23631538	1	111743537	5.99E-73	0.800	0.743	0.056	*DENND2D*	2.04E-04	0.016
**cg21999229**	6	31540014	6.00E-73	0.776	0.720	0.056	*LTA*	0.244	0.005
cg20633321	1	87793607	6.32E-73	0.260	0.331	-0.071	*LMO4*	2.33E-15	-0.046
cg13638427	6	29617320	6.37E-73	0.210	0.274	-0.064	NA	3.78E-09	-0.030
cg06620723	1	12404945	6.56E-73	0.197	0.261	-0.064	*VPS13D*	2.04E-06	-0.025
**cg09647108**	17	41934923	6.64E-73	0.781	0.725	0.056	*CD300LG*	0.060	0.008
cg03316587	8	142245164	6.73E-73	0.291	0.360	-0.069	NA	5.50E-07	-0.028
cg04781916	2	48013473	6.97E-73	0.598	0.693	-0.095	*MSH6*	8.12E-09	-0.036
cg23632656	17	56362296	7.17E-73	0.176	0.229	-0.053	NA	6.32E-07	-0.022
cg22755445	2	102772036	7.42E-73	0.540	0.609	-0.069	*IL1R1*	1.17E-05	-0.024
cg14306709	6	31547704	8.05E-73	0.693	0.633	0.059	NA	0.003	0.012
cg05799811	1	167487396	8.11E-73	0.737	0.661	0.076	*CD247*	6.59E-04	0.022
cg09699830	16	72516874	8.52E-73	0.769	0.693	0.076	NA	1.13E-14	0.046
cg17925829	19	36399328	8.54E-73	0.275	0.333	-0.058	*TYROBP*	8.40E-10	-0.029
cg10827767	22	31794522	9.15E-73	0.586	0.655	-0.069	*DRG1*	5.15E-09	-0.029
cg14043527	11	64642516	9.64E-73	0.798	0.747	0.051	*EHD1*	1.56E-04	0.015
cg12063992	15	75865604	9.73E-73	0.313	0.384	-0.072	*PTPN9*	1.21E-08	-0.032
**cg23599224**	4	68334446	1.02E-72	0.446	0.535	-0.089	NA	0.098	-0.011
cg18109874	19	14115611	1.03E-72	0.439	0.496	-0.057	*RFX1*	4.38E-11	-0.028
cg18934187	18	51882189	1.04E-72	0.509	0.595	-0.086	*STARD6*	0.009	-0.017
cg19016694	17	80821826	1.04E-72	0.544	0.483	0.060	*TBCD*	8.46E-06	0.018
cg01550148	5	134735544	1.07E-72	0.253	0.314	-0.060	*H2AFY*	1.13E-06	-0.023
cg12345526	2	173159062	1.08E-72	0.472	0.549	-0.077	NA	3.56E-07	-0.029
cg11636504	2	99439883	1.11E-72	0.389	0.443	-0.054	*KIAA1211L*	9.13E-06	-0.018
cg25929198	2	69867834	1.15E-72	0.777	0.725	0.052	*AAK1*	3.92E-08	0.023
cg23893629	1	203733971	1.17E-72	0.780	0.710	0.070	*LAX1*	7.13E-13	0.040
cg06501716	22	19436948	1.22E-72	0.791	0.736	0.054	*C22orf39*	0.001	0.013
cg14753356	6	30720108	1.29E-72	0.397	0.484	-0.087	NA	4.69E-18	-0.060
**cg13194425**	17	7341936	1.30E-72	0.264	0.336	-0.073	*FGF11*	0.212	-0.008
cg25446061	2	169967583	1.31E-72	0.282	0.364	-0.082	NA	0.001	-0.020
cg05329317	16	30126595	1.34E-72	0.796	0.742	0.053	*MAPK3*	2.94E-13	0.033
cg00175403	1	12223543	1.42E-72	0.581	0.529	0.052	NA	8.55E-04	0.013
cg00609630	12	123258653	1.47E-72	0.221	0.289	-0.068	*CCDC62*	1.43E-04	-0.021
cg04408104	2	160106670	1.47E-72	0.173	0.243	-0.070	*WDSUB1*	4.91E-05	-0.024
cg07116972	3	32463713	1.53E-72	0.170	0.226	-0.056	*CMTM7*	9.67E-06	-0.022
cg09123760	12	122712212	1.57E-72	0.693	0.635	0.057	*DIABLO*	7.77E-04	0.016
**cg12524168**	5	76028910	1.66E-72	0.779	0.728	0.051	*F2R*	0.067	0.007
cg11958128	12	3226445	1.71E-72	0.216	0.274	-0.058	*TSPAN9*	8.42E-09	-0.026
**cg05304531**	1	26797576	1.78E-72	0.240	0.300	-0.060	*DHDDS*	0.276	-0.005
cg22028727	6	30796243	1.94E-72	0.332	0.400	-0.068	NA	5.32E-17	-0.045
cg10142237	22	38714395	2.13E-72	0.399	0.460	-0.061	*CSNK1E*	1.01E-13	-0.032
cg04088945	7	157090146	2.28E-72	0.462	0.548	-0.086	NA	4.25E-07	-0.035
cg06132620	6	138820503	2.99E-72	0.187	0.244	-0.057	*NHSL1*	1.68E-10	-0.030
cg02538681	10	90611911	3.05E-72	0.458	0.539	-0.081	*ANKRD22*	3.70E-05	-0.025
cg15681255	8	27192141	3.08E-72	0.214	0.270	-0.055	*PTK2B*	1.72E-04	-0.016
cg01373166	22	24823389	3.10E-72	0.683	0.628	0.056	*ADORA2A*	1.49E-07	0.021
cg09122035	11	319667	3.11E-72	0.363	0.492	-0.129	NA	1.46E-20	-0.094
cg01403010	22	38609534	3.28E-72	0.340	0.393	-0.053	*MAFF*	4.49E-07	-0.020
cg12473916	1	154943651	3.30E-72	0.296	0.346	-0.050	*SHC1*	2.76E-17	-0.031
cg13622546	9	132402607	3.36E-72	0.531	0.607	-0.076	*ASB6*	2.00E-05	-0.025
cg17951878	6	7698374	3.39E-72	0.232	0.298	-0.065	NA	1.00E-12	-0.040
cg03800150	7	150216489	3.59E-72	0.798	0.733	0.065	*GIMAP7*	3.07E-14	0.040

cg00664609	16	2198075	3.91E-72	0.196	0.246	-0.051	*RAB26*	2.05E-04	-0.015
cg21480743	10	89621419	4.25E-72	0.205	0.259	-0.055	*KLLN*	7.51E-04	-0.015
cg07145284	11	66084631	4.31E-72	0.259	0.338	-0.079	*CD248*	0.002	-0.020
cg00928816	10	63809098	4.50E-72	0.796	0.739	0.057	*ARID5B*	4.58E-08	0.024
cg07504763	1	198575077	4.60E-72	0.489	0.565	-0.076	NA	1.26E-05	-0.026
cg07905054	12	102272136	4.63E-72	0.205	0.268	-0.063	*DRAM1*	9.05E-05	-0.020
cg16289618	6	29705939	4.71E-72	0.268	0.337	-0.069	*HLA-F-AS1*	5.33E-05	-0.022
cg15288800	17	6555742	4.81E-72	0.239	0.298	-0.059	*C17orf100*	3.06E-05	-0.020
cg24107728	1	53760337	4.87E-72	0.581	0.500	0.081	*LRP8*	2.27E-06	0.030
cg01616956	2	232393196	5.08E-72	0.801	0.735	0.066	*NMUR1*	4.98E-07	0.027
cg25953692	6	31695415	5.49E-72	0.240	0.310	-0.070	*DDAH2*	0.045	-0.011
cg03215181	4	122873487	5.66E-72	0.221	0.292	-0.071	*TRPC3*	0.016	-0.015
cg07328519	19	4064300	6.46E-72	0.283	0.337	-0.054	*ZBTB7A*	1.21E-16	-0.033
cg10588962	17	46667587	7.22E-72	0.286	0.376	-0.090	NA	6.57E-04	-0.024
**cg01172150**	16	30817443	7.43E-72	0.210	0.267	-0.056	NA	0.319	-0.004
cg15552238	19	14785749	7.75E-72	0.176	0.233	-0.057	*EMR3*	7.93E-10	-0.030
cg04556418	4	184367379	7.93E-72	0.550	0.621	-0.071	*CDKN2AIP*	8.62E-09	-0.031
cg01829672	5	157282617	8.19E-72	0.244	0.307	-0.063	*CLINT1*	1.33E-09	-0.032
cg10318744	14	69282256	8.39E-72	0.705	0.624	0.081	NA	1.42E-08	0.039
cg16672637	17	74138356	8.73E-72	0.506	0.566	-0.061	*FOXJ1*	2.63E-14	-0.036
cg02792780	3	52529341	8.84E-72	0.248	0.299	-0.051	*STAB1*	4.78E-06	-0.018
cg01498832	17	78682934	8.94E-72	0.412	0.496	-0.084	*RPTOR*	6.33E-05	-0.025
cg01841306	17	908923	9.42E-72	0.323	0.379	-0.056	*ABR*	3.07E-08	-0.024
**cg08145373**	11	2407008	9.58E-72	0.843	0.793	0.050	*CD81*	0.313	0.004
**cg26382697**	11	2406712	9.65E-72	0.816	0.761	0.055	*CD81*	0.847	0.000
cg19640821	19	18503517	9.94E-72	0.195	0.248	-0.054	*LRRC25*	3.14E-10	-0.026
cg13589463	14	100920326	1.01E-71	0.498	0.577	-0.078	*WDR25*	0.002	-0.017
cg03469804	17	72732432	1.02E-71	0.443	0.517	-0.074	*RAB37*	1.89E-16	-0.048
cg05978306	17	1373774	1.05E-71	0.284	0.361	-0.077	*MYO1C*	0.029	-0.013
**cg13189271**	6	15365693	1.07E-71	0.216	0.282	-0.066	*JARID2*	0.207	-0.007
cg09113070	2	86114036	1.09E-71	0.377	0.443	-0.066	*ST3GAL5*	1.13E-08	-0.031
cg26620021	19	40788926	1.13E-71	0.789	0.727	0.062	*AKT2*	0.026	0.011
cg08598221	8	121824929	1.14E-71	0.483	0.555	-0.072	*SNTB1*	9.06E-15	-0.042
cg01432609	17	78638554	1.16E-71	0.224	0.287	-0.063	*RPTOR*	0.033	-0.012
cg09018107	1	67798929	1.21E-71	0.806	0.730	0.076	*IL12RB2*	7.44E-13	0.044
cg09221159	10	90031426	1.30E-71	0.712	0.651	0.061	NA	1.06E-04	0.018
cg07484739	2	177356020	1.33E-71	0.729	0.661	0.067	NA	7.23E-09	0.029
cg25383568	19	39217721	1.41E-71	0.863	0.808	0.055	*ACTN4*	5.78E-04	0.016
cg04540712	16	89009691	1.45E-71	0.773	0.721	0.052	*CBFA2T3*	7.43E-08	0.021
cg16061354	17	1528465	1.64E-71	0.392	0.490	-0.098	*SLC43A2*	0.007	-0.020
cg11239720	4	152967415	1.67E-71	0.404	0.488	-0.084	NA	2.67E-05	-0.028
cg21007852	7	27203546	1.84E-71	0.202	0.258	-0.056	*HOXA10-HOXA*	1.60E-12	-0.033
cg20042612	14	23027728	1.87E-71	0.838	0.776	0.062	NA	1.49E-09	0.030
cg24690479	6	10521142	1.92E-71	0.284	0.348	-0.064	*GCNT2*	1.98E-12	-0.035
cg19047868	17	46669485	1.99E-71	0.198	0.271	-0.074	*HOXB-AS3*	0.028	-0.013
cg16596440	4	140684596	2.00E-71	0.206	0.263	-0.057	*MAML3*	4.41E-11	-0.031
cg26329274	5	156607853	2.12E-71	0.673	0.607	0.067	*ITK*	4.46E-05	0.021
cg25806655	19	4543498	2.14E-71	0.406	0.477	-0.072	*SEMA6B*	1.08E-07	-0.026
cg19872095	1	37941263	2.19E-71	0.687	0.624	0.063	*ZC3H12A*	9.68E-06	0.019
cg15704521	7	2773877	2.27E-71	0.513	0.615	-0.102	*GNA12*	5.95E-04	-0.026
cg20870000	8	38584896	2.32E-71	0.793	0.741	0.051	*TACC1*	2.17E-11	0.027
cg26530485	1	23496304	2.48E-71	0.159	0.211	-0.052	*LUZP1*	0.014	-0.012
cg14527029	3	120401095	2.61E-71	0.704	0.772	-0.068	*HGD*	1.45E-06	-0.023
cg06401414	8	141599436	2.71E-71	0.717	0.639	0.078	*EIF2C2*	0.021	0.015
cg09294998	8	97281863	2.71E-71	0.327	0.393	-0.067	*PTDSS1*	2.70E-05	-0.023
cg08060645	6	110503093	2.84E-71	0.225	0.287	-0.062	*CDC40*	1.78E-05	-0.022
cg11584690	19	42574196	2.89E-71	0.198	0.251	-0.054	NA	0.038	-0.009
cg05257202	13	99934873	3.07E-71	0.390	0.321	0.069	*UBAC2*	1.02E-06	0.023
cg01849093	11	110876141	3.14E-71	0.776	0.722	0.054	NA	2.42E-05	0.017
cg19997662	15	101784653	3.15E-71	0.272	0.353	-0.081	*CHSY1*	9.93E-08	-0.035
cg07092111	10	32621403	3.16E-71	0.655	0.589	0.066	*EPC1*	9.99E-04	0.016
cg13523125	11	35072710	3.27E-71	0.277	0.351	-0.074	NA	6.38E-06	-0.026
cg18634760	13	46679242	3.32E-71	0.358	0.453	-0.095	*CPB2*	9.65E-04	-0.024
cg14101485	19	3369759	3.39E-71	0.342	0.439	-0.096	*NFIC*	3.36E-05	-0.028
cg24882332	17	37063649	3.41E-71	0.510	0.564	-0.054	*LASP1*	2.65E-04	-0.016
cg04882213	8	61880392	3.45E-71	0.236	0.322	-0.085	NA	4.52E-10	-0.045
cg12361262	17	36571637	3.47E-71	0.259	0.339	-0.080	NA	1.09E-09	-0.039
**cg21875980**	1	231553510	3.61E-71	0.385	0.485	-0.100	*EGLN1*	0.081	-0.013
cg11789534	22	37655614	3.74E-71	0.316	0.404	-0.088	NA	1.61E-04	-0.025
cg11412713	11	94278413	3.80E-71	0.494	0.550	-0.056	*FUT4*	0.030	-0.008
cg24300607	7	73699346	3.83E-71	0.406	0.473	-0.067	NA	1.36E-05	-0.024
cg12586707	4	74738902	3.95E-71	0.222	0.281	-0.059	NA	1.62E-15	-0.038
cg15684702	1	45297445	4.11E-71	0.348	0.428	-0.080	*PTCH2*	7.73E-08	-0.032

cg19736900	1	150132950	4.18E-71	0.223	0.280	-0.057	NA	5.70E-13	-0.034
cg16452866	14	99655676	4.30E-71	0.815	0.753	0.062	*BCL11B*	1.46E-07	0.028
cg02930866	3	15482580	4.35E-71	0.201	0.255	-0.054	*EAF1*	2.31E-09	-0.025
cg00350296	11	66084841	4.52E-71	0.301	0.358	-0.056	*CD248*	4.67E-05	-0.019
cg05523603	22	50973101	4.72E-71	0.591	0.718	-0.127	NA	3.78E-26	-0.076
cg18410685	14	34838703	4.86E-71	0.262	0.326	-0.064	NA	8.05E-12	-0.038
cg24807547	6	37504484	4.95E-71	0.329	0.403	-0.074	NA	1.81E-04	-0.022
cg21577598	17	80084751	4.95E-71	0.754	0.683	0.071	*CCDC57*	8.22E-08	0.029
cg05191839	13	50819265	4.97E-71	0.821	0.764	0.056	NA	4.01E-05	0.019
cg26363039	12	1609549	5.02E-71	0.195	0.257	-0.063	NA	8.45E-07	-0.026
cg21664636	3	70432664	5.36E-71	0.804	0.742	0.061	NA	2.61E-05	0.021
cg23378033	12	65672031	5.36E-71	0.359	0.459	-0.100	*MSRB3*	5.03E-11	-0.051
cg26668919	6	35311534	5.48E-71	0.197	0.256	-0.059	*PPARD*	9.26E-12	-0.033
cg11050793	12	124876650	6.02E-71	0.225	0.292	-0.067	*NCOR2*	0.023	-0.012
cg06892898	7	37024713	6.16E-71	0.165	0.219	-0.054	*ELMO1*	3.37E-04	-0.016
cg02640604	16	29690271	6.24E-71	0.376	0.445	-0.068	*QPRT*	5.76E-12	-0.037
cg24864241	1	204329158	6.59E-71	0.211	0.275	-0.065	*PLEKHA6*	7.74E-06	-0.024
cg24805411	19	56158851	6.79E-71	0.288	0.340	-0.052	*CCDC106*	1.11E-08	-0.024
cg07345108	10	49893463	6.95E-71	0.194	0.245	-0.051	*WDFY4*	4.88E-21	-0.038
cg07006075	6	16251782	6.99E-71	0.610	0.679	-0.069	*GMPR*	3.50E-04	-0.016
cg06881398	1	55661307	7.55E-71	0.447	0.530	-0.083	*USP24*	0.011	-0.016
cg00808648	14	105779910	7.96E-71	0.268	0.343	-0.075	*PACS2*	0.001	-0.021
cg10225149	19	15491808	8.10E-71	0.405	0.487	-0.083	*AKAP8*	0.003	-0.020
cg10871721	21	46235052	8.16E-71	0.387	0.443	-0.057	*SUMO3*	4.38E-09	-0.029
cg17852032	6	158066811	8.41E-71	0.250	0.306	-0.056	*ZDHHC14*	7.76E-10	-0.027
cg16903174	2	9468351	8.62E-71	0.442	0.510	-0.067	*ASAP2*	1.20E-09	-0.034
cg08398132	2	8557381	9.18E-71	0.479	0.549	-0.071	NA	3.47E-04	-0.020
**cg08696931**	12	123754071	9.21E-71	0.174	0.229	-0.056	*CDK2AP1*	0.251	-0.005
cg04603976	19	4052706	9.21E-71	0.319	0.372	-0.053	*ZBTB7A*	2.83E-11	-0.027
cg26895569	1	204329222	9.61E-71	0.259	0.324	-0.065	*PLEKHA6*	9.50E-08	-0.029
cg13359998	1	230241764	1.00E-70	0.194	0.258	-0.064	*GALNT2*	4.95E-15	-0.041
cg21082028	17	46659993	1.02E-70	0.204	0.280	-0.076	NA	0.012	-0.016
cg26752663	2	25142016	1.07E-70	0.769	0.713	0.056	*ADCY3*	3.35E-07	0.022
cg06837040	1	180530353	1.09E-70	0.784	0.731	0.053	NA	0.009	0.011
cg01475325	11	76498701	1.09E-70	0.779	0.726	0.053	*TSKU*	6.86E-04	0.015
cg03835987	4	111120249	1.10E-70	0.337	0.422	-0.085	*ELOVL6*	0.003	-0.020
cg23770271	17	3704471	1.13E-70	0.375	0.431	-0.057	*ITGAE*	5.07E-04	-0.016
cg01297806	2	235949554	1.16E-70	0.407	0.463	-0.056	*SH3BP4*	7.66E-09	-0.026
cg14499058	8	141129321	1.16E-70	0.344	0.417	-0.073	*TRAPPC9*	1.47E-18	-0.051
cg03408904	22	17956462	1.18E-70	0.354	0.431	-0.077	*CECR2*	0.004	-0.018
cg15448894	6	52840469	1.20E-70	0.351	0.432	-0.081	NA	4.48E-07	-0.030
cg25154482	5	179497098	1.31E-70	0.241	0.306	-0.065	*RNF130*	1.33E-07	-0.030
cg06779253	1	9375627	1.32E-70	0.597	0.546	0.050	*SPSB1*	8.40E-07	0.018
cg25664938	3	119030023	1.34E-70	0.427	0.504	-0.077	*ARHGAP31*	0.004	-0.015
cg27315109	2	100677411	1.34E-70	0.814	0.763	0.051	*AFF3*	8.49E-10	0.023
cg24441810	2	120436039	1.36E-70	0.671	0.590	0.081	*TMEM177*	9.76E-14	0.050
cg25814649	13	107143980	1.38E-70	0.297	0.368	-0.071	*EFNB2*	1.43E-10	-0.037
cg22700848	12	124990942	1.39E-70	0.350	0.407	-0.057	*NCOR2*	0.028	-0.009
**cg17900199**	10	1156548	1.41E-70	0.685	0.629	0.056	*WDR37*	0.156	0.005
cg10151085	12	54654090	1.49E-70	0.191	0.244	-0.053	*CBX5*	0.015	-0.011
cg26647135	7	99954400	1.50E-70	0.431	0.489	-0.058	*PILRB*	1.20E-04	-0.017
cg08469255	6	30851069	1.50E-70	0.748	0.698	0.051	*DDR1*	4.89E-13	0.029
cg06931418	19	1402419	1.53E-70	0.214	0.264	-0.050	*GAMT*	3.20E-05	-0.018
cg19220272	2	43312367	1.57E-70	0.234	0.284	-0.050	NA	2.87E-08	-0.023
cg11231701	20	31946710	1.67E-70	0.396	0.470	-0.074	*CDK5RAP1*	2.97E-07	-0.030
cg12565788	2	241644240	1.85E-70	0.717	0.643	0.074	NA	5.38E-08	0.032
cg18899117	2	206411031	1.86E-70	0.314	0.384	-0.071	*PARD3B*	0.001	-0.018
cg10521852	19	19739820	1.86E-70	0.222	0.279	-0.057	*LPAR2*	4.36E-05	-0.017
cg13231954	2	8724414	2.14E-70	0.231	0.296	-0.065	NA	7.76E-08	-0.029
cg07319315	2	135149599	2.20E-70	0.543	0.616	-0.072	*MGAT5*	4.63E-04	-0.020
cg25324164	11	61598330	2.27E-70	0.219	0.284	-0.065	*FADS2*	1.22E-11	-0.037
cg22088368	22	37404888	2.44E-70	0.222	0.278	-0.055	*TEX33*	8.20E-08	-0.024
cg07906046	16	4131584	2.57E-70	0.631	0.561	0.070	*ADCY9*	4.05E-06	0.025
cg10763374	10	22608858	2.68E-70	0.298	0.361	-0.063	*COMMD3*	8.44E-15	-0.039
cg20427144	7	5518918	2.73E-70	0.381	0.451	-0.071	*FBXL18*	2.71E-06	-0.028
cg02768785	19	45912979	2.85E-70	0.238	0.300	-0.062	*ERCC1*	2.46E-08	-0.027
cg27186013	4	95264127	3.07E-70	0.271	0.338	-0.068	*HPGDS*	0.004	-0.016
cg01267797	5	79542470	3.23E-70	0.258	0.312	-0.054	*SERINC5*	7.03E-09	-0.024
cg02159996	6	89927233	3.59E-70	0.457	0.516	-0.059	*GABRR1*	3.33E-15	-0.036
cg00591421	10	106468736	3.64E-70	0.595	0.678	-0.083	*SORCS3*	9.85E-05	-0.024
cg12708994	6	116691460	3.88E-70	0.332	0.417	-0.085	*DSE*	0.015	-0.018
cg12049875	20	19955868	4.10E-70	0.461	0.526	-0.065	*RIN2*	7.07E-04	-0.015
cg11152302	14	24867472	4.27E-70	0.507	0.573	-0.065	*NYNRIN*	2.87E-14	-0.039

cg06638529	1	44704073	4.45E-70	0.336	0.406	-0.071	*ERI3*	4.29E-05	-0.022
cg22777724	17	46622516	4.73E-70	0.378	0.449	-0.071	*HOXB2*	5.12E-07	-0.028
cg08297985	16	85343488	4.76E-70	0.219	0.281	-0.062	NA	0.015	-0.012
cg09763373	16	596899	4.91E-70	0.463	0.534	-0.072	*SOLH*	0.010	-0.013
cg05233670	12	109058033	5.23E-70	0.359	0.426	-0.067	*CORO1C*	3.27E-11	-0.036
cg18477949	15	69760994	5.33E-70	0.667	0.606	0.062	NA	0.010	0.012
cg19647567	5	176935305	5.49E-70	0.367	0.425	-0.057	*DOK3*	6.93E-05	-0.016
cg07069934	15	89192956	5.56E-70	0.739	0.663	0.075	*ISG20*	4.47E-06	0.025
**cg15668538**	17	15931082	5.57E-70	0.794	0.743	0.051	*TTC19*	0.055	0.008
cg20316614	1	226065637	5.65E-70	0.194	0.250	-0.056	*TMEM63A*	0.005	-0.013
cg24727216	20	30947724	5.81E-70	0.601	0.545	0.055	*ASXL1*	2.17E-06	0.020
cg07327299	17	37896063	5.88E-70	0.366	0.434	-0.068	*GRB7*	1.15E-06	-0.024
cg14587065	17	3807247	5.89E-70	0.411	0.466	-0.056	*P2RX1*	3.11E-07	-0.024
cg01428095	1	228134229	5.93E-70	0.209	0.261	-0.052	*WNT9A*	3.49E-10	-0.027
cg25072666	17	79533843	6.12E-70	0.166	0.223	-0.057	*NPLOC4*	0.001	-0.017
cg18443571	15	90547692	6.22E-70	0.453	0.511	-0.058	*ZNF710*	5.38E-10	-0.029
cg21237418	17	27045043	6.74E-70	0.236	0.301	-0.065	*RAB34*	1.72E-04	-0.019
cg16387467	18	72166016	7.06E-70	0.169	0.223	-0.054	*CNDP2*	1.30E-09	-0.027
cg15228694	11	7692131	7.20E-70	0.420	0.514	-0.094	*CYB5R2*	7.79E-11	-0.046
cg23416081	6	35693573	7.43E-70	0.161	0.213	-0.052	*FKBP5*	0.002	-0.013
cg04598292	7	158495856	7.99E-70	0.246	0.305	-0.060	*NCAPG2*	3.12E-10	-0.030
cg16166559	22	45072588	8.03E-70	0.151	0.202	-0.051	*PRR5*	1.92E-07	-0.022
cg24163242	8	48648813	8.05E-70	0.326	0.396	-0.070	NA	3.11E-06	-0.026
cg04590170	11	33893895	8.48E-70	0.391	0.461	-0.071	*LMO2*	3.74E-08	-0.033
cg19160629	11	116742145	8.56E-70	0.281	0.347	-0.066	*SIK3*	1.68E-09	-0.034
cg11848173	12	56121015	8.64E-70	0.289	0.357	-0.069	*CD63*	0.005	-0.016
cg09080522	22	44568387	8.75E-70	0.200	0.268	-0.068	*PARVG*	3.45E-06	-0.025
**cg01283625**	10	13568099	9.87E-70	0.416	0.494	-0.078	NA	0.067	-0.011
cg06147863	11	47400113	1.00E-69	0.165	0.217	-0.052	*SPI1*	5.58E-05	-0.017
cg17033891	3	159852976	1.03E-69	0.187	0.245	-0.057	NA	1.42E-05	-0.022
cg25773585	16	30721715	1.05E-69	0.368	0.447	-0.078	*SRCAP*	8.30E-07	-0.031
cg12401918	6	32164723	1.10E-69	0.230	0.295	-0.065	*NOTCH4*	6.19E-10	-0.035
cg02743878	2	169937094	1.12E-69	0.278	0.339	-0.061	*DHRS9*	1.95E-09	-0.030
cg20491695	11	45719826	1.25E-69	0.760	0.707	0.053	NA	0.004	0.013
cg09697978	2	37501815	1.27E-69	0.358	0.436	-0.079	*PRKD3*	0.002	-0.018
cg08543327	5	1108651	1.31E-69	0.174	0.231	-0.058	*SLC12A7*	0.007	-0.014
cg10541466	1	113425263	1.32E-69	0.223	0.280	-0.056	NA	1.88E-14	-0.034
cg04998379	4	129491708	1.33E-69	0.167	0.227	-0.060	NA	1.85E-09	-0.029
cg20305578	8	1908301	1.35E-69	0.265	0.331	-0.066	NA	4.90E-06	-0.025
cg05523906	1	17751761	1.35E-69	0.411	0.476	-0.065	*RCC2*	6.59E-08	-0.028
cg23837109	10	75670435	1.36E-69	0.306	0.390	-0.084	*PLAU*	6.97E-05	-0.028
cg25945642	3	143321098	1.36E-69	0.396	0.486	-0.090	*SLC9A9*	5.21E-09	-0.043
cg19445690	2	74210890	1.37E-69	0.311	0.366	-0.055	NA	9.17E-07	-0.021
cg00792968	13	22635873	1.38E-69	0.812	0.756	0.056	NA	3.81E-06	0.023
cg20760057	6	130017522	1.46E-69	0.387	0.462	-0.075	*ARHGAP18*	2.53E-04	-0.023
cg02566775	6	144382964	1.52E-69	0.386	0.454	-0.067	*PLAGL1*	2.06E-08	-0.030
cg07737292	16	56892460	1.54E-69	0.352	0.450	-0.098	*MIR138-2*	0.028	-0.017
**cg21095280**	6	36724036	1.68E-69	0.741	0.685	0.057	*CPNE5*	0.288	0.004
cg01012879	1	244613363	1.70E-69	0.547	0.616	-0.069	*ADSS*	4.02E-09	-0.032
cg13074203	8	121798364	1.88E-69	0.308	0.377	-0.069	*SNTB1*	3.93E-09	-0.035
cg02648847	1	167408735	1.90E-69	0.236	0.292	-0.056	*CD247*	2.96E-04	-0.017
cg18884741	7	66204797	1.94E-69	0.434	0.504	-0.070	*RABGEF1*	7.61E-05	-0.023
cg15011899	13	111854118	2.12E-69	0.287	0.358	-0.071	*ARHGEF7*	0.008	-0.017
cg11645674	17	56401800	2.13E-69	0.203	0.257	-0.054	*BZRAP1*	1.87E-05	-0.020
cg15188623	15	90608429	2.15E-69	0.311	0.397	-0.086	*ZNF710*	0.005	-0.021
cg03565868	11	47400146	2.17E-69	0.345	0.422	-0.077	*SPI1*	0.005	-0.018
cg17510957	11	121466629	2.22E-69	0.565	0.645	-0.080	*SORL1*	4.38E-08	-0.035
cg13524037	6	32904074	2.23E-69	0.292	0.376	-0.084	*HLA-DMB*	0.001	-0.020
cg16847428	4	26095438	2.37E-69	0.513	0.607	-0.094	NA	0.001	-0.022
cg07223106	1	233155919	2.44E-69	0.422	0.483	-0.061	*PCNXL2*	2.28E-08	-0.027
cg02737268	20	3780182	2.48E-69	0.517	0.418	0.098	*CDC25B*	4.16E-06	0.033
cg05082563	7	137775882	2.56E-69	0.270	0.341	-0.070	*AKR1D1*	8.58E-06	-0.024
cg17922695	17	75451809	2.57E-69	0.683	0.627	0.056	*SEPT9*	3.65E-08	0.024
cg13857354	2	241519705	2.84E-69	0.408	0.492	-0.084	NA	1.31E-04	-0.024
cg20336172	7	2773782	2.87E-69	0.288	0.356	-0.068	*GNA12*	0.006	-0.017
cg09791366	1	228661675	2.91E-69	0.329	0.399	-0.070	NA	0.002	-0.018
cg01799015	19	707791	3.11E-69	0.541	0.613	-0.072	*PALM*	2.87E-08	-0.028
cg02046552	8	21914287	3.12E-69	0.228	0.293	-0.065	*EPB49*	2.03E-05	-0.021
cg19418318	19	17219073	3.25E-69	0.416	0.511	-0.094	*MYO9B*	1.92E-06	-0.034
cg19584136	10	111975951	3.27E-69	0.396	0.478	-0.082	*MXI1*	0.005	-0.018
cg19676182	4	24981695	3.38E-69	0.334	0.408	-0.074	*CCDC149*	0.005	-0.017
cg27170268	14	104171695	3.40E-69	0.335	0.416	-0.081	*XRCC3*	1.79E-08	-0.037
cg15381475	7	6436101	3.48E-69	0.193	0.256	-0.063	*RAC1*	4.17E-05	-0.021

cg01442620	19	33162885	3.49E-69	0.208	0.268	-0.060	*ANKRD27*	4.46E-05	-0.021
**cg09684160**	1	17054957	3.71E-69	0.724	0.669	0.055	NA	0.299	0.004
cg01624414	4	77119206	3.71E-69	0.260	0.323	-0.063	*SCARB2*	3.34E-05	-0.022
cg01249544	10	69372655	3.72E-69	0.692	0.631	0.061	*CTNNA3*	0.025	0.010
cg23474890	12	122467179	3.75E-69	0.776	0.700	0.076	*BCL7A*	8.46E-08	0.034
**cg10937802**	4	26177098	3.78E-69	0.804	0.752	0.052	NA	0.128	0.006
cg24762231	12	54690793	3.78E-69	0.355	0.450	-0.095	*NFE2*	0.007	-0.021
cg06559756	17	65464297	3.78E-69	0.723	0.669	0.054	*PITPNC1*	1.18E-04	0.016
cg19457909	17	59206070	3.80E-69	0.588	0.649	-0.061	*BCAS3*	1.46E-06	-0.022
cg04860291	3	53286651	3.81E-69	0.283	0.358	-0.075	*TKT*	0.012	-0.015
cg14630206	11	77121864	3.87E-69	0.406	0.474	-0.068	*PAK1*	2.25E-11	-0.036
cg03922423	8	144408588	3.90E-69	0.363	0.423	-0.061	*TOP1MT*	0.014	-0.011
cg24171555	2	69852693	4.09E-69	0.383	0.444	-0.061	*AAK1*	4.19E-06	-0.024
cg06160853	3	174155855	4.14E-69	0.303	0.380	-0.077	NA	2.85E-06	-0.031
cg08693490	12	116757896	4.36E-69	0.203	0.261	-0.058	NA	0.001	-0.015
cg02059176	2	44327637	4.37E-69	0.304	0.376	-0.072	NA	7.37E-09	-0.034
cg07297964	13	48895970	4.41E-69	0.521	0.592	-0.071	*RB1*	1.96E-04	-0.023
cg03717755	6	16136539	4.46E-69	0.688	0.621	0.067	*MYLIP*	1.97E-11	0.039
cg05569328	6	32044384	4.84E-69	0.193	0.247	-0.054	*TNXB*	4.46E-09	-0.028
cg02739870	5	1113320	4.90E-69	0.278	0.349	-0.071	*SLC12A7*	7.54E-14	-0.043
cg23513930	3	10334717	4.97E-69	0.232	0.292	-0.060	*GHRLOS*	0.004	-0.014
cg23229770	2	129491004	5.20E-69	0.350	0.431	-0.081	NA	1.68E-09	-0.040
cg07567256	6	163757048	5.32E-69	0.222	0.277	-0.056	NA	4.60E-06	-0.020
cg15447231	1	101537255	5.42E-69	0.327	0.390	-0.063	NA	1.76E-08	-0.031
cg20709530	20	36040249	5.67E-69	0.280	0.339	-0.058	NA	4.05E-15	-0.040
cg11916609	2	102927488	5.68E-69	0.388	0.475	-0.087	*IL1RL1*	0.006	-0.019
cg15649236	17	46657504	6.25E-69	0.302	0.388	-0.086	NA	8.07E-07	-0.032
cg07420362	11	3647419	6.45E-69	0.215	0.285	-0.069	NA	9.79E-06	-0.027
cg13917614	17	40125660	6.50E-69	0.825	0.760	0.065	*CNP*	0.006	0.016
cg14099345	9	132631088	6.56E-69	0.784	0.732	0.052	*USP20*	9.44E-06	0.019
cg25232795	5	150860100	6.70E-69	0.556	0.617	-0.061	*SLC36A1*	4.06E-05	-0.021
cg00107970	1	156119457	6.82E-69	0.239	0.300	-0.060	*SEMA4A*	1.39E-05	-0.022
cg20561863	17	66905630	6.82E-69	0.179	0.231	-0.052	*ABCA8*	3.18E-14	-0.033
cg08129092	1	153746211	7.07E-69	0.190	0.253	-0.064	*INTS3*	9.61E-20	-0.048
cg15410418	5	59559180	7.16E-69	0.426	0.504	-0.077	*PDE4D*	8.85E-04	-0.022
cg23657099	7	1102138	7.29E-69	0.263	0.332	-0.069	*C7orf50*	1.76E-09	-0.036
cg10502324	12	76372038	7.40E-69	0.628	0.562	0.065	NA	1.77E-06	0.023
**cg00505318**	3	69130918	7.50E-69	0.703	0.651	0.052	*UBA3*	0.214	0.005
cg07480608	3	15352493	7.57E-69	0.823	0.765	0.058	*SH3BP5*	2.41E-07	0.024
cg06983746	1	172628065	8.06E-69	0.741	0.685	0.057	*FASLG*	1.91E-09	0.029
cg19851816	22	50657907	8.10E-69	0.221	0.303	-0.082	*TUBGCP6*	4.89E-04	-0.024
cg11610626	6	128222053	8.46E-69	0.667	0.604	0.063	*THEMIS*	8.14E-08	0.028
cg18747197	6	158110124	8.54E-69	0.468	0.528	-0.061	NA	1.83E-11	-0.031
**cg02417473**	22	38434179	8.76E-69	0.692	0.636	0.056	NA	0.173	0.006
cg07313319	22	38714426	8.85E-69	0.452	0.529	-0.076	*CSNK1E*	7.57E-14	-0.046
cg16104584	1	9149734	8.87E-69	0.276	0.349	-0.073	NA	3.89E-06	-0.028
cg23582644	18	61557687	1.00E-68	0.478	0.552	-0.074	*SERPINB2*	2.33E-08	-0.030
cg00121045	16	1416053	1.02E-68	0.386	0.467	-0.080	*UNKL*	2.88E-04	-0.024
cg02314339	10	91020653	1.02E-68	0.682	0.753	-0.071	NA	4.70E-13	-0.036
cg02243630	4	154386540	1.07E-68	0.244	0.309	-0.065	*KIAA0922*	0.021	-0.013
cg06766034	22	47082260	1.07E-68	0.369	0.467	-0.098	*CERK*	0.002	-0.024
cg08716584	11	60157161	1.10E-68	0.322	0.415	-0.093	*MS4A7*	1.27E-07	-0.044
cg24549289	5	148809664	1.11E-68	0.564	0.622	-0.058	*MIR143HG*	3.49E-04	-0.016
cg10755085	17	72732722	1.16E-68	0.220	0.274	-0.054	*RAB37*	6.50E-08	-0.024
cg21130221	11	2848310	1.16E-68	0.443	0.501	-0.059	*KCNQ1*	2.42E-17	-0.042
cg00834923	16	85393998	1.19E-68	0.758	0.706	0.052	NA	6.13E-09	0.023
cg09302474	17	8869948	1.23E-68	0.572	0.644	-0.072	*PIK3R5*	6.55E-05	-0.022
cg02334109	15	26044050	1.23E-68	0.263	0.328	-0.065	*ATP10A*	2.59E-05	-0.023
cg02961101	16	48533155	1.29E-68	0.245	0.309	-0.064	NA	2.27E-12	-0.037
cg26854588	17	38440015	1.36E-68	0.811	0.759	0.052	NA	7.90E-08	0.023
cg01607369	11	7598673	1.39E-68	0.313	0.367	-0.053	*PPFIBP2*	1.57E-09	-0.026
cg08065963	16	8985593	1.40E-68	0.738	0.682	0.056	NA	5.43E-04	0.016
cg17319795	14	93170808	1.43E-68	0.243	0.310	-0.067	*LGMN*	2.34E-13	-0.042
cg02579959	15	100890963	1.48E-68	0.448	0.535	-0.087	NA	0.005	-0.021
cg26193268	6	30290443	1.48E-68	0.746	0.677	0.069	*HCG18*	2.26E-06	0.027
cg04270489	11	85827505	1.53E-68	0.797	0.746	0.051	NA	0.014	0.010
cg25416319	17	37080373	1.53E-68	0.408	0.489	-0.080	NA	1.75E-05	-0.028
**cg08521995**	8	41628237	1.54E-68	0.769	0.715	0.054	*ANK1*	0.155	0.006
cg12441066	17	55456535	1.55E-68	0.328	0.382	-0.054	*MSI2*	1.29E-09	-0.026
cg02641801	2	26213508	1.62E-68	0.297	0.359	-0.063	NA	1.28E-07	-0.028
cg21437157	8	27346608	1.77E-68	0.225	0.281	-0.056	NA	3.87E-04	-0.018
cg08835847	17	72450692	1.85E-68	0.198	0.258	-0.060	NA	1.12E-05	-0.020
cg08394278	5	176937446	1.85E-68	0.221	0.275	-0.054	*DOK3*	8.33E-06	-0.019

cg11477010	17	4079262	1.89E-68	0.825	0.772	0.053	ANKFY1	7.03E-11	0.028
cg05955436	20	17660838	2.04E-68	0.256	0.337	-0.081	RRBP1	2.40E-09	-0.042
cg04739880	6	35017865	2.18E-68	0.352	0.426	-0.075	ANKS1A	1.54E-06	-0.030
cg00188748	9	130564509	2.26E-68	0.327	0.405	-0.077	FPGS	6.54E-10	-0.042
cg06702850	6	166876890	2.27E-68	0.325	0.421	-0.096	RPS6KA2	6.12E-11	-0.047
cg20765716	17	3704602	2.45E-68	0.405	0.488	-0.083	ITGAE	0.031	-0.014
cg14691671	17	73642503	2.48E-68	0.337	0.404	-0.067	RECQL5	1.55E-09	-0.034
cg20382154	2	239463716	2.51E-68	0.221	0.286	-0.065	LOC151171	9.74E-07	-0.028
cg23125506	15	41198534	2.64E-68	0.301	0.364	-0.063	NA	3.61E-07	-0.027
cg22138096	7	41772439	2.65E-68	0.387	0.459	-0.072	INHBA-AS1	1.86E-04	-0.022
cg13762691	22	17956560	2.66E-68	0.250	0.312	-0.062	CECR2	5.64E-07	-0.027
cg08677655	17	1549108	2.68E-68	0.205	0.268	-0.063	SCARF1	3.01E-10	-0.031
cg26793227	1	16483658	2.72E-68	0.499	0.563	-0.064	EPHA2	0.006	-0.014
cg19225308	19	3607139	2.78E-68	0.198	0.256	-0.057	TBXA2R	1.20E-06	-0.023
cg13237068	13	25485381	3.01E-68	0.315	0.384	-0.069	CENPJ	1.10E-12	-0.041
**cg06059360**	3	42657618	3.14E-68	0.442	0.528	-0.086	NKTR	0.637	-0.002
cg14688905	12	51403056	3.25E-68	0.202	0.256	-0.053	SLC11A2	3.00E-04	-0.015
cg01138652	22	19973978	3.28E-68	0.286	0.358	-0.072	ARVCF	3.52E-07	-0.031
cg16422365	14	106041833	3.36E-68	0.731	0.679	0.052	NA	0.020	0.009
cg27095222	11	88090861	3.38E-68	0.211	0.271	-0.060	NA	3.61E-12	-0.035
cg10577241	15	77456283	3.41E-68	0.783	0.724	0.059	PEAK1	1.52E-14	0.037
cg10576245	1	233249267	3.44E-68	0.237	0.306	-0.069	PCNXL2	0.004	-0.017
cg06830981	1	22142491	3.46E-68	0.283	0.337	-0.054	LDLRAD2	3.37E-16	-0.032
cg01137047	6	2970877	3.49E-68	0.359	0.416	-0.057	SERPINB6	3.91E-23	-0.047
cg17412005	1	45806886	3.50E-68	0.225	0.285	-0.059	TOE1	1.89E-12	-0.036
cg27342333	7	75592418	3.62E-68	0.288	0.350	-0.062	POR	4.79E-14	-0.037
cg12701674	13	114908876	3.76E-68	0.592	0.533	0.060	NA	5.06E-12	0.032
cg26360197	17	78821604	3.93E-68	0.742	0.681	0.061	RPTOR	5.69E-09	0.028
cg17156633	1	46215776	3.96E-68	0.324	0.386	-0.062	IPP	4.43E-10	-0.029
cg12785694	3	160122168	4.08E-68	0.262	0.338	-0.076	SMC4	1.23E-19	-0.057
cg09069886	8	131000415	4.23E-68	0.431	0.500	-0.069	FAM49B	0.001	-0.017
cg01116568	11	10476529	4.27E-68	0.342	0.414	-0.072	AMPD3	5.87E-06	-0.028
cg24492058	1	12203751	4.27E-68	0.239	0.289	-0.050	TNFRSF8	3.18E-06	-0.019
cg25939853	12	133343428	4.65E-68	0.359	0.452	-0.093	NA	8.28E-09	-0.045
cg12512771	7	17730027	4.68E-68	0.209	0.269	-0.060	NA	2.12E-06	-0.025
cg16389345	11	46697382	4.95E-68	0.501	0.558	-0.058	ATG13	2.51E-09	-0.029
cg18619616	7	23334877	5.04E-68	0.612	0.679	-0.068	NA	7.52E-04	-0.019
cg12595667	2	136872094	5.38E-68	0.228	0.291	-0.063	CXCR4	3.36E-07	-0.027
cg27365701	12	89744150	5.43E-68	0.282	0.358	-0.075	DUSP6	8.93E-04	-0.021
cg22585927	3	9958613	5.56E-68	0.236	0.297	-0.061	IL17RC	0.031	-0.011
cg19942256	1	111216332	5.63E-68	0.755	0.700	0.055	KCNA3	6.85E-04	0.014
cg12700074	6	131571435	5.64E-68	0.207	0.270	-0.063	AKAP7	7.79E-05	-0.022
cg12602909	3	4512309	5.95E-68	0.318	0.408	-0.090	NA	2.92E-05	-0.031
cg08632810	8	141609470	6.10E-68	0.242	0.326	-0.084	EIF2C2	0.005	-0.020
cg08675743	5	114938439	6.15E-68	0.220	0.270	-0.050	TMED7-TICAM	3.33E-09	-0.026
cg15481493	11	35133274	6.62E-68	0.478	0.544	-0.066	NA	3.61E-05	-0.024
cg14475875	3	193856076	6.72E-68	0.189	0.257	-0.068	HES1	5.43E-11	-0.037
cg18560366	3	109057564	6.87E-68	0.220	0.273	-0.053	DPPA4	1.24E-09	-0.026
cg03934069	1	109420722	7.18E-68	0.143	0.193	-0.051	GPSM2	1.03E-04	-0.017
cg24051242	11	68096138	7.54E-68	0.209	0.272	-0.063	LRP5	0.005	-0.015
cg10586870	5	75722317	7.73E-68	0.345	0.408	-0.063	IQGAP2	3.18E-08	-0.032
**cg01904296**	11	45713046	8.40E-68	0.743	0.693	0.050	NA	0.156	0.006
cg18023065	11	94278603	9.22E-68	0.430	0.487	-0.058	FUT4	0.005	-0.011
cg21551549	6	155053894	9.34E-68	0.286	0.345	-0.059	SCAF8	6.15E-06	-0.023
**cg03316570**	6	43877657	9.44E-68	0.737	0.680	0.057	LOC10013235	0.595	0.002
cg26511108	10	22608951	9.47E-68	0.219	0.282	-0.063	COMMD3	2.17E-07	-0.028
**cg06992688**	14	94491958	9.50E-68	0.415	0.500	-0.085	OTUB2	0.066	-0.013
cg04058799	7	151547969	9.53E-68	0.545	0.605	-0.061	PRKAG2	1.11E-13	-0.034
cg11839815	3	127539037	9.75E-68	0.365	0.449	-0.085	MGLL	0.003	-0.021
cg10341242	16	50347849	1.15E-67	0.326	0.387	-0.061	ADCY7	0.030	-0.011
cg26889953	15	22915992	1.15E-67	0.360	0.421	-0.061	CYFIP1	4.08E-04	-0.015
cg01813965	16	57729104	1.24E-67	0.245	0.318	-0.073	CCDC135	1.14E-05	-0.024
cg17433678	10	3135764	1.26E-67	0.317	0.390	-0.073	PFKP	2.72E-11	-0.040
cg24590708	15	52554357	1.28E-67	0.599	0.518	0.080	MYO5C	2.56E-09	0.038
cg13438337	9	78774369	1.30E-67	0.443	0.531	-0.088	PCSK5	0.036	-0.014
cg15457390	4	2844730	1.30E-67	0.174	0.228	-0.055	ADD1	1.27E-07	-0.025
cg16770832	14	56604516	1.31E-67	0.608	0.662	-0.054	PELI2	1.32E-09	-0.023
cg01426713	6	106517533	1.36E-67	0.389	0.468	-0.080	NA	3.31E-05	-0.026
cg19724567	7	112063079	1.43E-67	0.217	0.276	-0.059	IFRD1	1.00E-11	-0.035
cg09335715	7	75626860	1.44E-67	0.253	0.315	-0.062	STYXL1	3.94E-05	-0.021
cg23032421	3	3152038	1.46E-67	0.793	0.741	0.052	IL5RA	3.56E-05	0.017
cg05954830	1	159838031	1.48E-67	0.388	0.481	-0.093	NA	4.01E-05	-0.034
cg02549628	4	88896208	1.50E-67	0.189	0.243	-0.054	SPP1	1.13E-07	-0.024

cg09830866	16	771714	1.63E-67	0.585	0.505	0.080	*FAM173A*	2.66E-06	0.023
cg06917763	7	130033247	1.65E-67	0.374	0.445	-0.071	NA	3.02E-08	-0.029
**cg18905668**	11	18388147	1.67E-67	0.212	0.274	-0.062	*GTF2H1*	0.617	-0.003
cg09169413	12	48103670	1.71E-67	0.359	0.424	-0.065	*ENDOU*	2.37E-11	-0.035
cg19145858	7	129781681	1.77E-67	0.195	0.253	-0.057	NA	2.46E-05	-0.020
cg13980609	11	66883117	1.78E-67	0.293	0.358	-0.065	NA	4.58E-08	-0.028
cg05261349	16	70473841	1.82E-67	0.493	0.552	-0.059	*ST3GAL2*	3.07E-07	-0.025
cg12122241	20	1920407	1.95E-67	0.418	0.496	-0.078	*SIRPA*	0.003	-0.019
cg04703221	16	69967063	2.05E-67	0.351	0.442	-0.091	*WWP2*	1.84E-07	-0.037
cg15872458	12	54784747	2.07E-67	0.209	0.271	-0.062	*ZNF385A*	0.003	-0.016
cg07266431	7	92460113	2.21E-67	0.156	0.211	-0.055	*CDK6*	4.61E-13	-0.036
cg08038033	3	71354056	2.29E-67	0.226	0.287	-0.061	*FOXP1*	8.09E-04	-0.017
cg01157951	6	31540399	2.33E-67	0.579	0.518	0.061	*LTA*	2.05E-04	0.017
cg13758331	2	111893676	2.36E-67	0.310	0.397	-0.087	*BCL2L11*	0.002	-0.023
cg15555463	1	201851880	2.41E-67	0.266	0.333	-0.066	*IPO9*	3.62E-04	-0.020
cg22375763	19	4540003	2.45E-67	0.400	0.484	-0.084	*LRG1*	1.09E-05	-0.031
cg18369972	17	9940121	2.72E-67	0.279	0.357	-0.078	*GAS7*	1.12E-11	-0.043
cg19667460	15	60863379	2.78E-67	0.803	0.750	0.052	*RORA*	6.96E-07	0.021
cg15818008	11	112126170	2.90E-67	0.404	0.488	-0.084	*C11orf34*	3.17E-06	-0.032
cg05382956	11	10627849	2.92E-67	0.238	0.297	-0.059	*MRVI1*	2.78E-09	-0.030
cg16592596	17	79620347	3.11E-67	0.195	0.252	-0.057	*PDE6G*	0.006	-0.014
cg13149245	15	65105500	3.25E-67	0.280	0.333	-0.053	NA	4.06E-10	-0.030
cg24013213	13	51640142	3.26E-67	0.242	0.298	-0.056	*GUCY1B2*	8.16E-10	-0.030
cg12156887	17	19883716	3.33E-67	0.294	0.355	-0.061	NA	7.61E-07	-0.024
cg25717464	2	225265963	3.65E-67	0.179	0.241	-0.062	*FAM124B*	7.12E-06	-0.024
cg12930392	3	196481615	3.75E-67	0.326	0.395	-0.069	*PAK2*	0.002	-0.018
cg26767974	2	240143979	3.76E-67	0.252	0.334	-0.082	*HDAC4*	2.64E-05	-0.031
cg07062336	12	45970082	3.82E-67	0.247	0.305	-0.058	NA	1.53E-08	-0.029
cg19352830	8	141599356	3.85E-67	0.815	0.765	0.050	*EIF2C2*	2.42E-07	0.021
cg15125472	19	36248077	3.86E-67	0.212	0.262	-0.050	*HSPB6*	0.026	-0.009
cg02131853	4	39034637	3.88E-67	0.764	0.712	0.051	*TMEM156*	0.016	0.010
cg20759281	12	21796914	3.89E-67	0.222	0.284	-0.061	*LDHB*	5.02E-08	-0.029
cg20331980	6	149554503	3.90E-67	0.745	0.694	0.051	NA	4.76E-08	0.023
cg04988978	17	56359578	4.03E-67	0.182	0.236	-0.054	*MPO*	6.21E-08	-0.026
cg03834767	7	90794392	4.13E-67	0.213	0.273	-0.060	*CDK14*	2.03E-09	-0.030
cg20626983	15	58650472	4.14E-67	0.786	0.735	0.051	NA	3.15E-13	0.030
cg07194321	9	74524266	4.21E-67	0.175	0.234	-0.059	*FAM108B1*	1.90E-14	-0.039
cg07016549	16	1630488	4.23E-67	0.475	0.539	-0.064	*IFT140*	0.008	-0.013
cg11366363	13	51641069	4.51E-67	0.439	0.499	-0.059	NA	9.82E-12	-0.034
cg20793071	1	54520450	4.59E-67	0.192	0.247	-0.055	*TCEANC2*	4.39E-06	-0.022
cg04177251	4	8193123	4.98E-67	0.264	0.328	-0.065	NA	9.81E-08	-0.028
cg17342588	7	27737875	4.98E-67	0.235	0.294	-0.059	NA	3.20E-10	-0.030
cg06630241	12	93967711	4.99E-67	0.317	0.399	-0.083	*SOCS2*	1.15E-08	-0.041
cg17298326	8	142245477	5.06E-67	0.283	0.353	-0.070	NA	3.79E-04	-0.021
cg00020474	1	8214963	5.31E-67	0.259	0.322	-0.063	NA	0.008	-0.014
cg19619003	1	41846758	5.57E-67	0.358	0.421	-0.062	NA	1.20E-05	-0.023
**cg02268192**	14	92981666	5.74E-67	0.293	0.367	-0.074	*RIN3*	0.087	-0.012
cg21621482	17	79228937	5.85E-67	0.338	0.396	-0.058	*SLC38A10*	0.018	-0.010
cg20513777	2	238533299	5.99E-67	0.461	0.538	-0.077	NA	1.12E-08	-0.037
cg01620165	12	113530915	6.10E-67	0.301	0.364	-0.063	*DTX1*	4.85E-12	-0.035
cg27408171	6	2958808	6.14E-67	0.258	0.334	-0.076	*SERPINB6*	8.93E-04	-0.020
cg03546687	16	2198080	6.16E-67	0.297	0.355	-0.058	*RAB26*	0.016	-0.011
**cg24435669**	1	15546149	6.24E-67	0.749	0.699	0.050	*TMEM51*	0.946	0.000
cg01816936	12	123518888	6.27E-67	0.219	0.276	-0.058	*PITPNM2*	1.69E-04	-0.018
cg22615330	7	151502322	6.33E-67	0.401	0.466	-0.065	*PRKAG2*	2.20E-06	-0.025
cg15514380	21	38737243	6.70E-67	0.231	0.299	-0.068	NA	0.015	-0.014
cg21992696	2	9125026	6.74E-67	0.293	0.354	-0.061	*MBOAT2*	1.19E-12	-0.036
cg06141561	16	2011215	6.97E-67	0.748	0.696	0.052	*NDUFB10*	9.01E-04	0.014
cg26509250	2	134884342	7.08E-67	0.360	0.416	-0.057	NA	4.93E-04	-0.016
cg18787437	1	16499673	7.17E-67	0.250	0.304	-0.054	NA	2.79E-11	-0.033
cg16871828	1	145474701	7.47E-67	0.257	0.324	-0.066	*ANKRD34A*	0.002	-0.017
cg18578954	6	28887247	7.67E-67	0.220	0.289	-0.069	*TRIM27*	8.34E-05	-0.023
**cg16276850**	17	38498914	8.27E-67	0.355	0.421	-0.066	*RARA*	0.185	-0.007
cg24211657	17	76886742	8.50E-67	0.232	0.295	-0.063	*LOC10065351*	0.003	-0.017
**cg26934362**	5	1519265	8.54E-67	0.821	0.759	0.062	*LPCAT1*	0.200	0.007
cg07600998	1	147146108	8.75E-67	0.344	0.400	-0.056	NA	6.96E-19	-0.041
cg26876444	1	65887413	8.77E-67	0.226	0.289	-0.063	*LEPR*	6.08E-04	-0.019
cg24740647	10	72681285	9.13E-67	0.324	0.394	-0.070	NA	1.51E-04	-0.022
**cg26645401**	11	9686514	9.21E-67	0.185	0.249	-0.064	*SWAP70*	0.255	-0.007
cg16580197	8	67841925	9.63E-67	0.316	0.386	-0.069	NA	2.08E-04	-0.021
**cg06202053**	11	64109021	1.06E-66	0.507	0.558	-0.051	*CCDC88B*	0.148	-0.006
cg02499139	3	171146337	1.07E-66	0.234	0.287	-0.054	*TNIK*	1.29E-06	-0.021
**cg06798483**	3	98504973	1.14E-66	0.809	0.758	0.051	*ST3GAL6*	0.787	0.001

cg23548201	3	195623792	1.17E-66	0.472	0.562	-0.089	*TNK2*	7.51E-06	-0.032
cg20904475	8	61823027	1.19E-66	0.238	0.299	-0.060	NA	5.84E-04	-0.017
cg16364675	14	103096221	1.19E-66	0.282	0.350	-0.067	*RCOR1*	9.28E-07	-0.027
cg27321942	19	49993217	1.21E-66	0.789	0.738	0.051	*RPL13A*	7.17E-06	0.019
cg14075731	17	72527607	1.23E-66	0.201	0.253	-0.053	*CD300LB*	2.39E-13	-0.031
cg04319611	3	171784422	1.28E-66	0.334	0.403	-0.069	*FNDC3B*	5.91E-08	-0.031
cg05363534	11	65210645	1.32E-66	0.367	0.438	-0.071	NA	0.005	-0.017
cg24850296	12	25707569	1.33E-66	0.275	0.341	-0.067	*IFLTD1*	6.02E-07	-0.026
cg01465769	3	53226412	1.37E-66	0.321	0.385	-0.064	*PRKCD*	6.13E-09	-0.031
cg15225325	5	176921820	1.49E-66	0.324	0.391	-0.067	*PDLIM7*	5.72E-10	-0.034
cg03546163	6	35654363	1.51E-66	0.474	0.601	-0.127	*FKBP5*	0.002	-0.025
cg01124810	13	21144699	1.71E-66	0.516	0.571	-0.055	*IFT88*	4.10E-06	-0.019
**cg24690314**	6	144018564	1.86E-66	0.491	0.562	-0.071	*PHACTR2*	0.205	-0.007
cg24057642	19	17357641	1.87E-66	0.220	0.273	-0.053	*NR2F6*	2.02E-04	-0.016
**cg03171478**	22	37572916	2.38E-66	0.301	0.372	-0.072	NA	0.126	-0.008
cg03123320	1	229394665	2.44E-66	0.446	0.502	-0.056	NA	8.87E-14	-0.034
cg03585611	19	7675609	2.56E-66	0.741	0.688	0.053	*CAMSAP3*	6.10E-05	0.019
cg02217713	7	643155	2.64E-66	0.459	0.524	-0.065	*PRKAR1B*	2.01E-21	-0.050
cg10743102	3	177055590	2.71E-66	0.780	0.728	0.052	NA	0.013	0.011
cg08850169	1	161171469	2.72E-66	0.428	0.486	-0.058	*NDUFS2*	0.001	-0.014
cg02747254	16	29938183	2.74E-66	0.365	0.423	-0.059	*KCTD13*	1.60E-12	-0.035
cg10275770	17	62084205	2.82E-66	0.281	0.338	-0.057	*ICAM2*	1.59E-08	-0.026
cg25261331	19	51628121	2.88E-66	0.292	0.345	-0.053	*SIGLEC9*	1.64E-07	-0.021
cg05006942	16	69776039	3.07E-66	0.624	0.689	-0.065	*NOB1*	8.43E-05	-0.020
cg10204320	1	32708276	3.24E-66	0.199	0.251	-0.052	NA	1.06E-08	-0.026
cg13953458	11	73681281	3.27E-66	0.694	0.632	0.062	*DNAJB13*	8.69E-11	0.032
cg06875162	1	28184973	3.30E-66	0.421	0.486	-0.065	NA	1.49E-12	-0.040
cg02105211	3	4625188	3.36E-66	0.513	0.595	-0.082	*ITPR1*	2.69E-09	-0.040
cg02901136	1	153348305	3.37E-66	0.461	0.531	-0.070	*S100A12*	6.76E-04	-0.019
cg19089701	8	126398731	3.55E-66	0.239	0.304	-0.065	NA	3.95E-06	-0.025
cg18091385	14	99502466	3.62E-66	0.389	0.454	-0.065	NA	3.86E-07	-0.025
cg17125472	11	69259831	3.90E-66	0.459	0.531	-0.072	NA	9.61E-04	-0.018
cg13860849	11	66084469	3.94E-66	0.328	0.384	-0.055	*CD248*	8.79E-05	-0.017
**cg14437551**	6	31539986	4.01E-66	0.780	0.716	0.064	*LTA*	0.473	0.004
cg15693299	12	7916371	4.01E-66	0.820	0.759	0.061	*NANOGNB*	0.030	0.011
cg14136168	9	139129886	4.20E-66	0.224	0.288	-0.064	*QSOX2*	0.006	-0.015
cg09277376	1	47899676	4.26E-66	0.234	0.294	-0.060	*FOXD2-AS1*	0.004	-0.013
cg09971549	16	89487322	4.34E-66	0.639	0.588	0.051	*ANKRD11*	0.013	0.010
cg07842386	1	67632028	4.34E-66	0.797	0.739	0.058	*IL23R*	1.20E-10	0.032
cg00257775	6	37904681	4.38E-66	0.318	0.388	-0.070	*ZFAND3*	3.05E-04	-0.022
cg05957567	12	122467228	4.51E-66	0.667	0.611	0.056	*BCL7A*	3.52E-05	0.018
cg17806661	22	37096133	4.59E-66	0.264	0.320	-0.056	*CACNG2*	1.90E-04	-0.019
cg27107685	7	30590918	4.66E-66	0.178	0.231	-0.053	*LOC401320*	0.009	-0.012
cg04168675	2	225809655	4.71E-66	0.745	0.672	0.073	*DOCK10*	1.26E-13	0.048
cg07787977	1	962651	5.04E-66	0.702	0.632	0.070	*AGRN*	4.30E-13	0.039
cg25179758	13	36918614	5.18E-66	0.298	0.359	-0.061	*SPG20*	1.68E-08	-0.027
**cg18660064**	1	23504632	5.19E-66	0.235	0.309	-0.074	NA	0.339	-0.007
**cg06810647**	16	1665094	5.40E-66	0.186	0.244	-0.058	*CRAMP1L*	0.228	-0.006
cg07979236	1	33516461	5.96E-66	0.355	0.456	-0.100	NA	2.90E-07	-0.043
cg09076077	20	58630315	5.98E-66	0.257	0.315	-0.057	*C20orf197*	1.93E-05	-0.021
cg19048010	17	28084996	6.88E-66	0.734	0.671	0.063	*SSH2*	9.02E-06	0.021
cg06655216	12	89619329	6.93E-66	0.760	0.703	0.057	NA	3.32E-09	0.029
cg20091384	19	2700927	7.11E-66	0.747	0.683	0.064	*GNG7*	0.034	0.011
cg22757101	19	3607136	8.56E-66	0.239	0.301	-0.062	*TBXA2R*	8.35E-07	-0.026
cg02814118	16	81528945	9.20E-66	0.222	0.285	-0.063	*CMIP*	4.22E-05	-0.022
cg13674369	5	969918	9.34E-66	0.770	0.719	0.050	NA	9.42E-11	0.028
cg05290737	12	123215151	9.35E-66	0.761	0.703	0.058	*HCAR1*	1.13E-09	0.029
cg04329351	10	81955971	9.46E-66	0.808	0.750	0.058	*ANXA11*	1.11E-07	0.026
cg16203203	6	125284659	1.04E-65	0.227	0.291	-0.064	NA	1.98E-06	-0.026
cg02980566	17	21411890	1.04E-65	0.263	0.320	-0.058	NA	3.12E-10	-0.030
cg12371569	12	13278271	1.07E-65	0.193	0.253	-0.060	NA	2.13E-10	-0.034
cg11825899	9	35844849	1.12E-65	0.435	0.508	-0.073	*TMEM8B*	1.86E-13	-0.046
cg13381110	18	60646614	1.13E-65	0.529	0.639	-0.109	*PHLPP1*	0.027	-0.018
cg25761791	19	3165800	1.17E-65	0.302	0.387	-0.085	NA	0.004	-0.022
**cg03310874**	7	4850260	1.20E-65	0.380	0.453	-0.073	*RADIL*	0.102	-0.010
cg04967578	18	13641894	1.31E-65	0.485	0.575	-0.090	*C18orf1*	2.34E-07	-0.034
cg12421536	1	51426414	1.36E-65	0.208	0.268	-0.060	*FAF1*	1.49E-04	-0.020
cg09606564	17	19290353	1.52E-65	0.168	0.227	-0.059	*MFAP4*	9.62E-07	-0.023
cg24221738	4	166125552	1.65E-65	0.811	0.757	0.054	NA	6.41E-06	0.022
cg03797115	4	110625010	1.72E-65	0.301	0.391	-0.090	*CASP6*	0.024	-0.018
cg09642075	15	74303043	1.82E-65	0.601	0.660	-0.059	*PML*	1.01E-10	-0.031
cg16674484	5	149887497	1.83E-65	0.297	0.372	-0.075	*NDST1*	9.46E-08	-0.034
cg20483374	11	119211646	1.88E-65	0.339	0.393	-0.054	*C1QTNF5*	1.72E-05	-0.019

cg12754495	3	151102703	1.89E-65	0.349	0.427	-0.078	*P2RY12*	3.37E-04	-0.022
cg23889010	20	43882990	1.90E-65	0.366	0.445	-0.079	*SLPI*	0.002	-0.022
cg22045942	19	35490561	1.91E-65	0.202	0.266	-0.064	*GRAMD1A*	2.94E-05	-0.023
cg21380024	17	61042803	1.91E-65	0.551	0.483	0.068	*MIR548W*	2.53E-14	0.044
cg13914004	9	130659670	1.97E-65	0.239	0.295	-0.056	*ST6GALNAC6*	3.24E-06	-0.022
cg02259081	12	53268433	1.99E-65	0.202	0.279	-0.077	NA	4.91E-04	-0.026
cg19104015	17	46646043	2.09E-65	0.228	0.293	-0.065	*HOXB3*	8.60E-07	-0.028
cg18893596	13	91823796	2.13E-65	0.368	0.435	-0.067	*LINC00379*	1.64E-04	-0.022
**cg26189021**	1	110757770	2.18E-65	0.812	0.762	0.050	*KCNC4*	0.443	0.003
cg14260169	10	72344881	2.24E-65	0.271	0.352	-0.081	NA	1.06E-09	-0.044
cg06484100	17	41799087	2.26E-65	0.442	0.504	-0.062	NA	2.06E-06	-0.025
cg27418217	15	83518427	2.28E-65	0.222	0.273	-0.051	*HOMER2*	9.56E-04	-0.015
cg02075820	11	17300909	2.50E-65	0.230	0.293	-0.063	*NUCB2*	1.18E-05	-0.025
cg02711886	5	177762428	2.57E-65	0.225	0.281	-0.055	*COL23A1*	8.20E-04	-0.015
cg13007701	8	13078829	2.60E-65	0.554	0.617	-0.063	*DLC1*	2.43E-07	-0.027
cg22737154	2	64631614	2.72E-65	0.434	0.506	-0.071	NA	2.13E-19	-0.053
cg04514255	17	46658170	2.89E-65	0.449	0.515	-0.066	NA	5.91E-20	-0.048
cg06346307	22	19949965	2.91E-65	0.664	0.609	0.055	*COMT*	1.30E-04	0.017
cg04361126	2	190446792	2.98E-65	0.217	0.274	-0.057	*SLC40A1*	2.37E-09	-0.029
cg03763873	13	43565901	3.24E-65	0.122	0.201	-0.079	*EPSTI1*	4.93E-15	-0.057
cg00357551	5	169407472	3.26E-65	0.416	0.496	-0.080	*DOCK2*	4.12E-05	-0.026
**cg18460107**	12	7902153	3.30E-65	0.741	0.686	0.055	*CLEC4C*	0.978	0.000
cg03967798	4	145268453	3.37E-65	0.368	0.437	-0.068	NA	6.50E-11	-0.035
cg07892167	8	23144983	3.46E-65	0.177	0.230	-0.053	*R3HCC1*	1.99E-09	-0.028
cg16745604	2	202047459	3.53E-65	0.238	0.296	-0.057	*CASP10*	0.010	-0.013
cg10498921	5	139625058	3.55E-65	0.177	0.230	-0.053	*PFDN1*	0.004	-0.013
**cg24727480**	12	109901083	3.73E-65	0.193	0.244	-0.051	*KCTD10*	0.131	-0.006
**cg18535415**	1	200983238	3.94E-65	0.366	0.419	-0.053	*KIF21B*	0.055	-0.008
cg23659250	22	50174065	3.95E-65	0.449	0.525	-0.076	*BRD1*	3.89E-06	-0.027
cg01305421	12	102874286	3.99E-65	0.257	0.312	-0.055	*IGF1*	1.00E-05	-0.022
cg26841048	17	46622454	4.13E-65	0.527	0.600	-0.072	*HOXB2*	5.06E-14	-0.047
cg24542714	1	46669345	4.46E-65	0.248	0.324	-0.076	*POMGNT1*	7.08E-06	-0.028
**cg08939373**	11	33563246	4.96E-65	0.377	0.454	-0.077	*KIAA1549L*	0.288	-0.006
cg22386583	17	78753756	5.05E-65	0.506	0.581	-0.075	*RPTOR*	1.13E-07	-0.033
cg04667114	13	34185082	5.25E-65	0.802	0.749	0.053	*STARD13*	1.05E-04	0.019
cg19240637	2	7172297	5.27E-65	0.493	0.560	-0.067	*RNF144A*	4.17E-10	-0.033
cg21953058	15	41309253	5.28E-65	0.343	0.419	-0.076	*INO80*	2.77E-06	-0.032
cg21185109	17	7774912	5.40E-65	0.343	0.411	-0.069	NA	0.001	-0.018
cg01871722	12	32120103	5.64E-65	0.832	0.776	0.056	*KIAA1551*	3.56E-10	0.030
cg08572565	14	51298737	5.78E-65	0.235	0.294	-0.059	*NIN*	5.92E-10	-0.032
cg19097880	11	128160637	6.25E-65	0.707	0.644	0.063	NA	0.037	0.011
cg04157865	17	77914162	6.37E-65	0.465	0.536	-0.071	*TBC1D16*	4.38E-06	-0.025
**cg20782252**	7	2153774	6.64E-65	0.578	0.629	-0.052	*MAD1L1*	0.051	-0.008
cg24341911	21	40689521	6.64E-65	0.242	0.312	-0.070	*BRWD1-AS1*	3.56E-09	-0.035
cg11530995	15	68497992	6.82E-65	0.324	0.374	-0.050	*CALML4*	2.04E-07	-0.020
cg23975251	1	10604446	6.82E-65	0.640	0.561	0.078	*PEX14*	1.39E-18	0.054
cg08903019	17	42299928	6.86E-65	0.224	0.287	-0.063	NA	0.008	-0.015
cg26839652	2	217881615	7.02E-65	0.751	0.682	0.069	NA	2.18E-10	0.040
cg02627227	6	161664638	7.21E-65	0.276	0.350	-0.075	*AGPAT4*	7.06E-05	-0.025
cg18426551	17	42147617	7.35E-65	0.146	0.196	-0.050	*G6PC3*	3.05E-05	-0.017
cg13408795	3	143050350	7.67E-65	0.402	0.468	-0.065	*SLC9A9*	8.65E-12	-0.036
cg09357350	3	171894094	7.77E-65	0.434	0.504	-0.070	*FNDC3B*	0.018	-0.013
cg22239727	11	19264434	7.78E-65	0.412	0.466	-0.055	*E2F8*	0.031	-0.009
cg08688907	5	159890925	7.83E-65	0.581	0.641	-0.059	NA	1.03E-15	-0.039
cg20636714	10	5476521	8.03E-65	0.704	0.636	0.068	*NET1*	3.64E-16	0.047
cg13439189	9	93926553	8.12E-65	0.786	0.734	0.051	NA	0.002	0.013
cg04837616	17	75880542	8.20E-65	0.498	0.555	-0.057	NA	5.65E-11	-0.031
cg24957609	12	27125095	8.63E-65	0.552	0.481	0.071	*TM7SF3*	1.60E-14	0.044
cg24315421	1	208040253	8.66E-65	0.183	0.250	-0.067	NA	2.36E-06	-0.027
cg06418113	18	43915901	9.78E-65	0.251	0.315	-0.065	*RNF165*	2.65E-07	-0.029
cg03681335	2	108903965	9.94E-65	0.409	0.475	-0.066	*SULT1C2*	1.26E-11	-0.039
cg00298324	20	822788	1.03E-64	0.202	0.269	-0.067	*FAM110A*	0.008	-0.016
cg10898024	2	40266366	1.04E-64	0.303	0.379	-0.076	*SLC8A1-AS1*	2.93E-08	-0.033
cg16766382	10	28588024	1.05E-64	0.700	0.633	0.068	NA	5.16E-06	0.025
cg09595020	7	2654120	1.07E-64	0.348	0.409	-0.061	*IQCE*	4.44E-08	-0.029
cg02010481	7	28218524	1.09E-64	0.167	0.225	-0.058	*JAZF1*	4.52E-11	-0.032
cg24075113	7	100465289	1.12E-64	0.158	0.209	-0.052	*TRIP6*	0.002	-0.013
cg01523881	1	200985689	1.12E-64	0.239	0.297	-0.058	*KIF21B*	6.21E-09	-0.030
cg19914554	17	80275401	1.17E-64	0.663	0.611	0.052	*CD7*	1.00E-07	0.021
cg23592421	14	105147461	1.20E-64	0.360	0.437	-0.078	NA	1.10E-15	-0.053
cg01028142	2	7004578	1.24E-64	0.727	0.879	-0.152	*CMPK2*	2.83E-32	-0.073
cg09050775	12	46762708	1.25E-64	0.707	0.639	0.068	*SLC38A2*	5.32E-10	0.038
cg01477971	2	103405951	1.28E-64	0.358	0.435	-0.078	*TMEM182*	0.003	-0.020

cg03707599	7	75956931	1.30E-64	0.275	0.350	-0.075	*YWHAG*	1.45E-06	-0.032
cg12216435	3	128995479	1.32E-64	0.611	0.542	0.068	*COPG1*	2.31E-04	0.021
cg26963632	16	85558148	1.38E-64	0.233	0.287	-0.054	NA	1.89E-21	-0.042
cg20194973	22	50524676	1.41E-64	0.312	0.378	-0.066	*MLC1*	6.54E-10	-0.035
cg06876053	11	9810051	1.43E-64	0.228	0.283	-0.055	*SBF2-AS1*	5.56E-16	-0.041
cg08864944	1	164633485	1.46E-64	0.334	0.396	-0.062	*PBX1*	0.017	-0.013
cg18670846	2	68917482	1.57E-64	0.201	0.259	-0.058	NA	1.58E-08	-0.028
cg14131824	17	1490487	1.59E-64	0.318	0.383	-0.065	*SLC43A2*	1.22E-05	-0.025
cg14121282	9	137268074	1.74E-64	0.261	0.318	-0.057	*RXRA*	6.09E-04	-0.017
cg21971799	11	60932010	1.74E-64	0.226	0.289	-0.064	NA	4.63E-11	-0.036
cg23351010	3	73109785	1.75E-64	0.412	0.478	-0.066	*PPP4R2*	0.047	-0.011
cg27366162	17	66375195	1.81E-64	0.413	0.470	-0.056	*ARSG*	2.93E-07	-0.022
cg03452895	2	163199311	1.92E-64	0.519	0.587	-0.068	*GCA*	1.15E-06	-0.029
cg16547186	16	57512988	1.97E-64	0.238	0.297	-0.059	*DOK4*	6.15E-09	-0.030
cg04586126	5	176734633	2.05E-64	0.385	0.464	-0.079	*MXD3*	0.002	-0.020
cg14765206	10	101744219	2.07E-64	0.694	0.633	0.061	*DNMBP*	1.96E-05	0.022
cg08949974	4	40632860	2.10E-64	0.268	0.334	-0.066	*RBM47*	9.57E-06	-0.025
cg09009788	13	77894098	2.11E-64	0.647	0.700	-0.053	*MYCBP2*	1.25E-08	-0.023
cg14023009	8	1870798	2.12E-64	0.190	0.245	-0.055	*ARHGEF10*	7.95E-07	-0.024
cg11177980	1	41982115	2.15E-64	0.293	0.344	-0.051	*HIVEP3*	4.75E-06	-0.018
cg12106403	19	667808	2.16E-64	0.253	0.327	-0.074	NA	1.14E-04	-0.026
cg10736454	19	7739455	2.17E-64	0.273	0.330	-0.057	NA	2.28E-06	-0.022
cg08058191	17	39781130	2.26E-64	0.240	0.302	-0.062	*KRT17*	8.96E-07	-0.027
cg14398691	17	37895771	2.29E-64	0.413	0.486	-0.072	*GRB7*	3.53E-04	-0.022
cg18397975	5	33997484	2.32E-64	0.545	0.621	-0.076	*AMACR*	8.22E-04	-0.018
cg14001992	4	154073813	2.43E-64	0.262	0.337	-0.076	*TRIM2*	0.022	-0.015
cg00239353	16	3115133	2.46E-64	0.836	0.776	0.060	*IL32*	0.003	0.015
cg26644674	10	3138505	2.54E-64	0.379	0.470	-0.092	*PFKP*	2.87E-07	-0.040
cg03760308	6	76310551	2.56E-64	0.499	0.570	-0.071	*SENP6*	2.22E-08	-0.036
**cg11518509**	1	114525428	2.59E-64	0.171	0.227	-0.056	NA	0.713	0.002
cg27306802	1	3510561	2.59E-64	0.265	0.340	-0.075	*MEGF6*	1.57E-07	-0.034
cg02226672	16	68398533	2.61E-64	0.456	0.526	-0.070	*SMPD3*	0.006	-0.016
cg00268476	16	2740589	2.64E-64	0.384	0.440	-0.057	*KCTD5*	7.36E-12	-0.029
cg14919082	2	54901055	2.66E-64	0.314	0.392	-0.078	NA	8.33E-04	-0.022
cg21861151	2	239478365	2.70E-64	0.331	0.426	-0.095	NA	4.28E-04	-0.029
cg15227911	17	7792059	2.73E-64	0.334	0.264	0.069	*CHD3*	1.64E-04	0.017
**cg16638248**	11	10596059	3.03E-64	0.769	0.716	0.053	*MRVI1-AS1*	0.440	0.004
cg25788549	7	150786044	3.08E-64	0.191	0.253	-0.061	*AGAP3*	0.012	-0.013
cg13937905	12	53612551	3.14E-64	0.906	0.854	0.052	*RARG*	4.55E-20	0.043
cg15412815	11	93271088	3.32E-64	0.257	0.336	-0.079	*C11orf75*	3.44E-07	-0.037
cg14412134	14	64912417	3.40E-64	0.789	0.722	0.068	*MTHFD1*	1.67E-11	0.040
**cg22669060**	21	34774882	3.47E-64	0.170	0.228	-0.058	*IFNGR2*	0.084	-0.008
cg05294112	13	112617069	3.47E-64	0.279	0.358	-0.079	NA	6.99E-08	-0.039
cg00974761	17	33866073	3.73E-64	0.785	0.730	0.055	NA	0.005	0.014
cg10984625	2	238599858	3.81E-64	0.197	0.257	-0.060	*LRRFIP1*	5.58E-05	-0.021
cg05941027	17	61774174	3.89E-64	0.477	0.427	0.051	*LIMD2*	4.09E-05	0.016
cg08900409	19	18475669	3.90E-64	0.415	0.510	-0.096	*PGPEP1*	2.34E-05	-0.036
cg19360907	22	37977445	3.94E-64	0.191	0.247	-0.057	*LGALS2*	0.003	-0.016
cg20866075	17	77777010	4.22E-64	0.230	0.299	-0.068	NA	2.81E-04	-0.021
**cg26433561**	1	110426090	4.31E-64	0.484	0.543	-0.060	NA	0.115	-0.008
cg23202167	19	42442230	4.37E-64	0.534	0.605	-0.072	NA	9.22E-05	-0.023
cg21603144	2	102608233	4.37E-64	0.319	0.385	-0.066	*IL1R2*	7.93E-10	-0.036
cg08486269	6	154677972	4.40E-64	0.187	0.246	-0.059	*IPCEF1*	4.59E-06	-0.023
cg07427438	16	4819304	4.46E-64	0.398	0.466	-0.068	NA	9.89E-04	-0.019
cg12743031	8	27219512	4.52E-64	0.586	0.529	0.057	*PTK2B*	2.56E-05	0.019
cg12067024	1	153387689	4.59E-64	0.605	0.670	-0.065	*S100A7A*	0.011	-0.012
**cg02717454**	16	3928799	4.60E-64	0.222	0.282	-0.061	*CREBBP*	0.134	-0.008
cg24049880	1	161171211	4.65E-64	0.224	0.277	-0.053	*NDUFS2*	4.06E-10	-0.029
cg25066772	14	93136682	4.85E-64	0.228	0.286	-0.058	*RIN3*	1.34E-13	-0.038
cg16611352	17	3819429	4.92E-64	0.284	0.349	-0.065	*P2RX1*	0.001	-0.016
cg15379400	17	38655492	5.06E-64	0.352	0.404	-0.052	*TNS4*	4.12E-08	-0.024
cg00748432	22	18042722	5.08E-64	0.369	0.424	-0.055	*SLC25A18*	3.12E-04	-0.017
cg01022200	1	180881086	5.33E-64	0.218	0.277	-0.058	*KIAA1614*	2.91E-06	-0.025
cg18467978	19	47134906	5.41E-64	0.558	0.625	-0.067	NA	1.69E-09	-0.031
cg12464638	11	844400	5.41E-64	0.502	0.562	-0.061	*TSPAN4*	9.34E-13	-0.038
cg01666652	14	22987689	5.57E-64	0.781	0.727	0.054	NA	1.51E-10	0.027
cg12156512	19	47258676	6.02E-64	0.429	0.511	-0.081	*FKRP*	0.004	-0.017
cg05099952	16	30367546	6.29E-64	0.331	0.388	-0.056	*CD2BP2*	2.83E-04	-0.016
cg18281939	5	77783895	6.40E-64	0.363	0.432	-0.069	*LHFPL2*	1.24E-04	-0.023
cg15037823	3	71730474	6.46E-64	0.368	0.443	-0.075	*EIF4E3*	1.98E-07	-0.033
cg19631762	1	218698561	6.48E-64	0.202	0.260	-0.059	NA	2.46E-10	-0.034
cg20274462	8	95980625	6.78E-64	0.225	0.282	-0.057	NA	2.22E-06	-0.024
**cg05413628**	16	1521656	7.18E-64	0.344	0.439	-0.095	*CLCN7*	0.325	-0.008

cg03052078	6	147527113	7.34E-64	0.340	0.416	-0.076	*STXBP5*	2.50E-06	-0.034
cg07551364	1	27693782	7.83E-64	0.376	0.430	-0.054	*MAP3K6*	3.04E-14	-0.036
cg12058385	2	144448686	7.90E-64	0.433	0.518	-0.085	*ARHGAP15*	5.57E-04	-0.022
cg17066531	6	36922415	7.92E-64	0.207	0.269	-0.062	*PI16*	0.001	-0.017
cg01022219	18	13641735	8.08E-64	0.465	0.543	-0.078	*C18orf1*	7.84E-06	-0.027
cg09662852	16	11707685	8.52E-64	0.238	0.300	-0.062	NA	3.06E-12	-0.035
cg14781242	1	32738251	8.92E-64	0.735	0.684	0.052	*LCK*	5.07E-09	0.025
cg26551026	18	20717693	9.17E-64	0.508	0.577	-0.069	*CABLES1*	5.92E-11	-0.037
cg21932231	2	36668864	9.37E-64	0.427	0.508	-0.081	*CRIM1*	3.93E-11	-0.041
cg04256697	12	120688557	9.60E-64	0.646	0.568	0.078	*PXN*	1.39E-11	0.044
**cg02368508**	16	12060182	1.00E-63	0.778	0.727	0.051	*TNFRSF17*	0.691	-0.002
**cg04488111**	1	154897518	1.00E-63	0.430	0.517	-0.086	*PMVK*	0.135	-0.010
cg05969591	2	191044915	1.01E-63	0.497	0.551	-0.054	*C2orf88*	7.05E-08	-0.025
cg20045320	11	319555	1.01E-63	0.425	0.553	-0.128	NA	5.32E-26	-0.095
cg06939451	5	81043172	1.07E-63	0.461	0.537	-0.076	*SSBP2*	6.82E-05	-0.025
cg02656560	17	19967600	1.13E-63	0.249	0.300	-0.051	*SPECC1*	1.53E-09	-0.028
cg08826152	17	15869607	1.16E-63	0.293	0.365	-0.072	*ADORA2B*	3.69E-06	-0.029
cg04655481	9	125796809	1.17E-63	0.273	0.335	-0.062	*GPR21*	7.42E-06	-0.022
cg13661827	9	111885602	1.18E-63	0.431	0.510	-0.079	NA	1.17E-08	-0.038
cg07749597	5	42840698	1.24E-63	0.348	0.433	-0.085	NA	5.63E-13	-0.047
cg14179944	14	35867515	1.33E-63	0.381	0.443	-0.062	NA	8.61E-09	-0.031
cg03270969	2	242813189	1.36E-63	0.697	0.641	0.056	*CXXC11*	1.10E-05	0.020
cg14871225	5	139040820	1.37E-63	0.332	0.384	-0.051	*CXXC5*	0.004	-0.011
cg02640809	5	173213710	1.45E-63	0.179	0.229	-0.050	NA	4.72E-20	-0.041
cg23122901	22	19880135	1.52E-63	0.574	0.518	0.057	*TXNRD2*	1.94E-05	0.018
cg07300408	14	21058360	1.60E-63	0.341	0.436	-0.095	*RNASE12*	8.32E-07	-0.039
cg10371414	16	79451857	1.61E-63	0.379	0.461	-0.082	NA	8.77E-04	-0.024
cg13492227	17	7341436	1.61E-63	0.282	0.338	-0.056	*FGF11*	7.32E-04	-0.017
cg10858945	10	116528318	1.61E-63	0.258	0.315	-0.057	NA	1.16E-08	-0.026
cg02417427	5	79479211	1.67E-63	0.364	0.426	-0.062	*SERINC5*	9.10E-06	-0.024
cg24642483	1	159261560	1.76E-63	0.465	0.523	-0.059	*FCER1A*	3.90E-14	-0.033
cg17904575	14	102290370	1.78E-63	0.723	0.655	0.068	*PPP2R5C*	1.43E-10	0.039
cg16873414	22	44568699	1.87E-63	0.237	0.295	-0.059	*PARVG*	4.62E-06	-0.023
cg25983531	7	36765142	1.88E-63	0.304	0.371	-0.067	*AOAH*	5.96E-05	-0.023
cg18859174	2	118982679	1.91E-63	0.226	0.283	-0.058	NA	7.50E-06	-0.022
cg22566906	12	52399713	1.96E-63	0.215	0.268	-0.052	*GRASP*	6.36E-13	-0.033
cg06358171	1	54822008	2.08E-63	0.305	0.382	-0.077	*SSBP3*	0.008	-0.016
cg26645827	3	100322629	2.11E-63	0.293	0.363	-0.070	NA	8.81E-07	-0.029
cg00691729	17	58468300	2.13E-63	0.387	0.467	-0.080	*USP32*	2.41E-08	-0.038
cg07329251	11	10476662	2.30E-63	0.232	0.286	-0.054	*AMPD3*	2.12E-09	-0.027
cg17306848	14	61793064	2.33E-63	0.809	0.745	0.064	*PRKCH*	6.19E-12	0.038
cg04674832	2	72078638	2.41E-63	0.455	0.508	-0.053	NA	1.95E-05	-0.018
cg13869484	1	9140563	2.55E-63	0.658	0.592	0.065	NA	6.08E-09	0.034
cg26806779	12	9141775	2.89E-63	0.372	0.436	-0.064	*KLRG1*	1.59E-10	-0.036
cg03274669	13	74805233	3.03E-63	0.803	0.752	0.051	NA	1.94E-07	0.023
cg11581865	11	120078840	3.12E-63	0.311	0.383	-0.072	NA	2.88E-05	-0.026
cg24721964	3	150662697	3.12E-63	0.231	0.293	-0.062	*CLRN1*	2.55E-17	-0.047
cg05479618	11	351125	3.15E-63	0.287	0.363	-0.076	NA	0.002	-0.022
cg04300115	20	62200199	3.22E-63	0.645	0.572	0.073	*PRIC285*	7.83E-14	0.040
cg01471293	6	33265322	3.23E-63	0.205	0.267	-0.062	*RGL2*	1.68E-06	-0.028
cg07391141	21	44035034	3.23E-63	0.660	0.610	0.050	NA	2.53E-10	0.026
cg09447621	11	134254646	3.25E-63	0.184	0.234	-0.051	*B3GAT1*	6.49E-09	-0.025
cg17285931	2	240205408	3.37E-63	0.517	0.598	-0.081	*HDAC4*	7.06E-04	-0.022
cg23350385	3	46448134	3.51E-63	0.520	0.590	-0.070	*CCRL2*	2.17E-05	-0.025
cg24435571	10	104436140	3.55E-63	0.175	0.226	-0.051	*ARL3*	0.004	-0.013
cg25004840	2	235330993	3.60E-63	0.689	0.623	0.066	NA	3.52E-04	0.020
cg02162324	16	67196462	3.71E-63	0.268	0.324	-0.056	*HSF4*	1.19E-04	-0.019
cg11169848	11	67142030	3.75E-63	0.212	0.292	-0.080	*CLCF1*	0.003	-0.020
cg16437908	2	85640810	3.85E-63	0.253	0.329	-0.076	*CAPG*	0.012	-0.018
cg24292612	8	6735472	3.86E-63	0.351	0.422	-0.071	*DEFB1*	1.56E-04	-0.022
cg15278374	12	131576088	3.87E-63	0.806	0.750	0.056	*GPR133*	0.023	0.011
cg00735611	17	37895886	3.98E-63	0.440	0.503	-0.063	*GRB7*	7.50E-06	-0.022
cg05117208	17	40438312	4.06E-63	0.351	0.417	-0.066	*STAT5A*	2.74E-08	-0.031
cg12240358	15	83619523	4.12E-63	0.797	0.730	0.067	*HOMER2*	3.37E-05	0.025
cg03199996	20	49211573	4.34E-63	0.753	0.700	0.053	*FAM65C*	1.68E-04	0.017
**cg23372795**	6	39284679	4.56E-63	0.802	0.751	0.051	*KCNK16*	0.179	0.006
cg01011769	16	85648749	4.56E-63	0.257	0.316	-0.059	*KIAA0182*	1.02E-04	-0.020
cg07139162	16	70465351	4.66E-63	0.428	0.501	-0.073	*ST3GAL2*	0.003	-0.018
cg15598244	1	23696413	4.75E-63	0.150	0.203	-0.053	*ZNF436*	1.69E-06	-0.023
cg13304609	1	79085162	4.95E-63	0.722	0.850	-0.128	*IFI44L*	2.74E-31	-0.065
cg25992874	7	142997642	5.01E-63	0.357	0.419	-0.062	*CASP2*	2.03E-05	-0.024
cg24536349	8	143781340	5.06E-63	0.308	0.377	-0.070	*LY6K*	7.94E-05	-0.024
cg22406869	11	66276941	5.07E-63	0.380	0.435	-0.055	*DPP3*	1.07E-13	-0.035

cg22687766	1	207490952	5.17E-63	0.369	0.430	-0.061	NA	1.19E-12	-0.035
cg17651972	12	57620054	5.33E-63	0.392	0.458	-0.066	*NXPH4*	5.86E-08	-0.031
cg10172675	8	104132683	5.64E-63	0.185	0.243	-0.058	NA	1.22E-04	-0.020
cg24150662	1	2949673	5.70E-63	0.855	0.799	0.056	NA	3.57E-06	0.022
cg23646343	7	139333410	5.89E-63	0.231	0.286	-0.054	*HIPK2*	1.09E-05	-0.021
cg07079231	16	21169331	5.97E-63	0.544	0.627	-0.084	*TMEM159*	0.023	-0.015
cg07854457	5	126898200	6.39E-63	0.254	0.317	-0.063	NA	1.85E-07	-0.030
cg22523050	7	137564212	6.49E-63	0.397	0.459	-0.062	*CREB3L2*	7.03E-07	-0.027
cg03606269	5	77779546	6.83E-63	0.166	0.227	-0.061	NA	1.46E-07	-0.028
cg06852461	1	207975182	7.14E-63	0.173	0.233	-0.060	NA	4.66E-05	-0.020
cg26818573	2	46717839	7.73E-63	0.351	0.413	-0.062	NA	9.25E-06	-0.024
cg08548498	20	43883546	7.99E-63	0.224	0.284	-0.060	*SLPI*	9.32E-13	-0.036
cg22774605	6	47247047	8.10E-63	0.487	0.554	-0.067	*TNFRSF21*	3.47E-04	-0.021
cg07924575	22	26881231	8.32E-63	0.517	0.568	-0.052	*HPS4*	1.35E-07	-0.025
cg17253709	2	62442007	8.40E-63	0.341	0.403	-0.062	*B3GNT2*	5.59E-09	-0.032
cg17843665	13	24351673	8.62E-63	0.721	0.650	0.070	*MIPEP*	3.21E-05	0.025
cg15599182	6	56623606	8.63E-63	0.786	0.732	0.054	NA	2.40E-08	0.027
cg20829834	3	63946048	8.82E-63	0.408	0.473	-0.066	*ATXN7*	0.005	-0.016
cg15440661	2	43395871	9.03E-63	0.512	0.570	-0.058	NA	1.20E-05	-0.020
cg02573176	8	82607505	9.44E-63	0.342	0.402	-0.060	*SLC10A5*	1.18E-06	-0.025
cg07065759	2	198017462	9.57E-63	0.204	0.263	-0.059	*ANKRD44*	1.39E-04	-0.021
cg13583162	9	131545865	9.85E-63	0.346	0.399	-0.053	NA	9.40E-07	-0.023
cg11924260	12	125036266	9.90E-63	0.154	0.207	-0.053	*NCOR2*	9.95E-06	-0.019
cg22280671	13	22170320	1.07E-62	0.244	0.303	-0.060	*EFHA1*	1.30E-04	-0.020
cg07379703	5	78279424	1.12E-62	0.467	0.540	-0.073	*ARSB*	9.99E-06	-0.027
cg13707794	21	35889668	1.12E-62	0.259	0.325	-0.066	*RCAN1*	3.00E-06	-0.026
cg16384774	14	89626932	1.12E-62	0.605	0.668	-0.063	*FOXN3*	5.78E-07	-0.026
cg12486710	1	178512616	1.17E-62	0.354	0.414	-0.059	*C1orf220*	1.17E-08	-0.030
cg16175713	6	46617172	1.20E-62	0.296	0.375	-0.079	*CYP39A1*	1.18E-05	-0.030
cg21234955	9	97713896	1.32E-62	0.758	0.690	0.068	*C9orf3*	3.60E-06	0.028
cg17460386	1	207095668	1.36E-62	0.435	0.373	0.063	*FAIM3*	3.05E-04	0.018
cg14459032	9	78905335	1.37E-62	0.432	0.491	-0.059	*PCSK5*	1.51E-06	-0.023
cg01080713	2	190126396	1.59E-62	0.760	0.700	0.060	NA	3.34E-11	0.035
cg23615741	10	101297642	1.61E-62	0.330	0.436	-0.107	NA	3.49E-15	-0.068
cg04840813	3	196363641	1.65E-62	0.260	0.314	-0.054	NA	2.52E-07	-0.025
cg24328095	17	80205093	1.65E-62	0.229	0.281	-0.052	*CSNK1D*	1.41E-07	-0.025
cg24848787	15	58844359	1.71E-62	0.213	0.274	-0.061	*LIPC*	9.16E-08	-0.031
cg24964368	19	52264413	1.80E-62	0.176	0.229	-0.053	*FPR2*	0.035	-0.010
cg20207911	17	9802796	1.81E-62	0.445	0.515	-0.070	*RCVRN*	5.54E-06	-0.028
cg19429405	10	73497609	1.86E-62	0.422	0.499	-0.077	*C10orf105*	1.16E-04	-0.027
cg02922776	1	212420632	1.86E-62	0.315	0.384	-0.069	NA	1.91E-07	-0.032
cg21185662	12	45662797	1.92E-62	0.388	0.450	-0.062	*ANO6*	2.84E-06	-0.025
cg17753124	19	13259872	1.96E-62	0.491	0.547	-0.056	*STX10*	6.77E-08	-0.028
cg20155875	17	66452567	1.98E-62	0.513	0.578	-0.065	*WIPI1*	2.07E-08	-0.032
cg00459232	12	6309025	2.18E-62	0.197	0.250	-0.053	*CD9*	3.85E-05	-0.019
cg22728904	7	100433983	2.20E-62	0.209	0.265	-0.055	NA	8.01E-07	-0.024
cg19440734	3	48264753	2.29E-62	0.211	0.265	-0.054	*CAMP*	2.38E-07	-0.024
cg05481452	11	61717684	2.41E-62	0.763	0.709	0.054	*BEST1*	0.011	0.011
cg08939850	17	78800806	2.41E-62	0.338	0.421	-0.083	*RPTOR*	0.001	-0.023
cg26818464	10	6618723	2.42E-62	0.722	0.670	0.052	*PRKCQ*	1.03E-05	0.020
cg14921416	20	3693158	2.48E-62	0.249	0.305	-0.056	NA	0.006	-0.013
cg15352367	22	36236732	2.62E-62	0.333	0.401	-0.068	*RBFOX2*	8.00E-05	-0.024
cg18022893	1	44951225	2.64E-62	0.280	0.351	-0.072	*RNF220*	4.82E-07	-0.029
cg09377088	6	108910284	2.71E-62	0.512	0.572	-0.060	*FOXO3*	2.11E-12	-0.035
cg13286582	2	37883934	2.72E-62	0.628	0.568	0.060	*CDC42EP3*	1.34E-13	0.038
cg01636591	17	32646156	2.72E-62	0.580	0.633	-0.053	*CCL8*	8.94E-19	-0.038
cg08418872	12	6442954	2.74E-62	0.748	0.688	0.060	*TNFRSF1A*	1.67E-08	0.028
cg26833120	1	32169568	2.86E-62	0.301	0.364	-0.064	*COL16A1*	3.45E-04	-0.016
cg07187268	22	24105186	2.89E-62	0.412	0.467	-0.055	*C22orf15*	7.60E-07	-0.022
cg00094734	2	96830479	2.98E-62	0.751	0.698	0.053	NA	6.98E-08	0.025
cg17894755	10	104535794	3.11E-62	0.226	0.284	-0.058	*WBP1L*	0.038	-0.010
cg13429260	11	73356744	3.29E-62	0.409	0.487	-0.078	*PLEKHB1*	3.63E-05	-0.027
cg13717434	9	97854044	3.32E-62	0.476	0.547	-0.071	NA	9.62E-06	-0.027
cg03722295	19	10519375	3.47E-62	0.726	0.653	0.073	NA	6.86E-08	0.036
cg22831526	3	194706168	3.59E-62	0.244	0.313	-0.069	NA	4.80E-07	-0.030
cg22143698	5	10608058	3.65E-62	0.256	0.327	-0.071	*ANKRD33B*	2.49E-04	-0.024
cg25587662	18	10525447	3.71E-62	0.201	0.257	-0.057	*NAPG*	9.16E-05	-0.021
cg16692277	13	51640948	3.89E-62	0.208	0.259	-0.051	NA	9.79E-09	-0.026
**cg10599444**	14	23305941	4.01E-62	0.279	0.339	-0.060	*MMP14*	0.975	0.000
**cg12452298**	15	67134587	4.02E-62	0.157	0.214	-0.058	NA	0.063	-0.009
cg26105232	10	6105656	4.04E-62	0.376	0.453	-0.077	*IL2RA*	5.89E-06	-0.030
cg22387323	4	24975717	4.15E-62	0.483	0.549	-0.066	*CCDC149*	1.79E-05	-0.022
**cg04858164**	15	57324333	4.24E-62	0.463	0.562	-0.100	*TCF12*	0.857	0.001

cg17472111	11	75287721	4.28E-62	0.244	0.299	-0.055	NA	1.21E-10	-0.031
**cg20544852**	6	28874479	4.38E-62	0.448	0.532	-0.084	*TRIM27*	0.221	-0.008
cg04273604	1	234609003	4.38E-62	0.155	0.212	-0.057	*TARBP1*	9.90E-08	-0.028
cg20887442	11	71725424	4.52E-62	0.564	0.618	-0.054	*NUMA1*	0.003	-0.013
cg11343072	2	160473528	4.57E-62	0.408	0.478	-0.070	*BAZ2B*	0.003	-0.018
cg03720617	16	47527034	4.60E-62	0.235	0.308	-0.073	*PHKB*	9.98E-05	-0.027
cg11876048	17	55337654	4.83E-62	0.397	0.471	-0.073	*MSI2*	0.026	-0.015
cg06528601	16	84652562	4.91E-62	0.361	0.415	-0.054	*COTL1*	1.95E-08	-0.025
cg25699034	17	74721824	5.08E-62	0.268	0.339	-0.071	*METTL23*	8.33E-10	-0.038
cg14055004	2	241860995	5.19E-62	0.731	0.679	0.051	NA	0.038	0.008
cg20705781	11	67070238	5.31E-62	0.304	0.374	-0.071	*SSH3*	6.01E-06	-0.028
cg15531369	19	49993157	5.41E-62	0.677	0.624	0.053	*RPL13A*	3.72E-05	0.017
cg08672140	5	77845045	5.76E-62	0.202	0.257	-0.055	*LHFPL2*	1.16E-12	-0.035
cg02025583	17	10632861	5.89E-62	0.243	0.300	-0.058	*TMEM220*	5.01E-05	-0.019
cg01561304	5	177913485	6.43E-62	0.175	0.229	-0.054	*COL23A1*	1.35E-07	-0.026
cg21115433	1	27961868	6.47E-62	0.193	0.251	-0.058	*FGR*	1.15E-08	-0.029
cg16313807	18	77723226	6.74E-62	0.739	0.650	0.089	*HSBP1L1*	2.64E-05	0.030
cg07944862	17	38501174	6.92E-62	0.543	0.608	-0.065	*RARA*	1.05E-09	-0.035
cg26546113	1	182810711	7.25E-62	0.735	0.667	0.068	*DHX9*	5.00E-10	0.038
cg18200741	2	33780366	7.43E-62	0.246	0.298	-0.051	*RASGRP3*	1.00E-08	-0.025
cg26348226	1	21617442	7.59E-62	0.205	0.260	-0.055	*ECE1*	5.74E-07	-0.026
cg08857797	17	40927699	7.61E-62	0.534	0.599	-0.064	*VPS25*	2.12E-06	-0.026
cg15173780	3	111043756	7.62E-62	0.755	0.690	0.066	NA	7.10E-04	0.016
cg09315878	1	1152580	8.09E-62	0.796	0.715	0.081	*SDF4*	8.16E-05	0.028
cg12401842	12	8276175	8.60E-62	0.386	0.467	-0.080	*CLEC4A*	0.003	-0.022
cg01948978	1	203712327	8.74E-62	0.562	0.629	-0.067	*ATP2B4*	7.93E-04	-0.019
cg17256364	10	17495134	9.58E-62	0.221	0.278	-0.057	*ST8SIA6*	2.50E-05	-0.023
cg01734045	4	109967535	9.58E-62	0.233	0.288	-0.055	*COL25A1*	3.74E-11	-0.034
cg27073113	16	56228744	9.66E-62	0.248	0.312	-0.064	*GNAO1*	5.12E-06	-0.025
cg13630493	9	36190154	1.08E-61	0.238	0.297	-0.059	*CLTA*	1.51E-08	-0.030
cg01176028	21	43653234	1.20E-61	0.216	0.267	-0.051	*ABCG1*	9.04E-10	-0.027
cg17056618	13	50216117	1.24E-61	0.616	0.676	-0.060	NA	2.03E-08	-0.027
cg20784259	1	114518035	1.31E-61	0.160	0.210	-0.050	*HIPK1*	2.67E-06	-0.021
**cg12106976**	6	152002859	1.34E-61	0.227	0.295	-0.068	NA	0.056	-0.012
cg12688240	12	7940852	1.43E-61	0.521	0.583	-0.061	*NANOG*	1.39E-04	-0.020
cg17205313	18	21572748	1.43E-61	0.767	0.715	0.052	*TTC39C*	1.46E-08	0.026
cg07453440	18	11948154	1.45E-61	0.288	0.376	-0.089	NA	0.007	-0.022
cg02703728	7	48127997	1.47E-61	0.233	0.291	-0.058	*UPP1*	6.23E-13	-0.039
cg05094137	16	53125208	1.51E-61	0.212	0.262	-0.050	*CHD9*	3.49E-09	-0.028
cg08885142	11	69264296	1.53E-61	0.219	0.277	-0.057	NA	1.45E-04	-0.018
**cg12446199**	1	167408841	1.59E-61	0.168	0.226	-0.058	*CD247*	0.180	-0.007
cg24011341	12	24577904	1.60E-61	0.799	0.741	0.058	*SOX5*	2.65E-13	0.037
cg14953730	10	134498830	1.66E-61	0.261	0.315	-0.055	*INPP5A*	5.27E-08	-0.026
cg09473725	1	167485873	1.80E-61	0.779	0.722	0.057	*CD247*	2.08E-06	0.025
cg12949760	11	2542862	1.80E-61	0.368	0.430	-0.062	*KCNQ1*	2.33E-10	-0.035
cg17332306	17	76259456	1.85E-61	0.188	0.241	-0.053	NA	0.001	-0.014
cg10328171	16	85116332	1.86E-61	0.215	0.267	-0.052	*KIAA0513*	3.10E-05	-0.020
cg22712983	2	219187374	1.88E-61	0.586	0.684	-0.098	*PNKD*	0.006	-0.022
**cg14603031**	7	2563184	1.91E-61	0.221	0.279	-0.059	*LFNG*	0.089	-0.008
cg04502852	8	131450313	1.91E-61	0.376	0.441	-0.066	*ASAP1*	1.20E-04	-0.022
cg23610213	6	4802019	1.96E-61	0.786	0.732	0.054	*CDYL*	6.62E-10	0.030
cg09812990	16	55367838	2.01E-61	0.222	0.273	-0.051	NA	1.14E-07	-0.025
**cg23911433**	5	134611106	2.06E-61	0.429	0.507	-0.078	NA	0.390	-0.005
cg10264003	11	110198965	2.08E-61	0.556	0.494	0.061	NA	6.49E-08	0.026
**cg03479289**	11	33563377	2.10E-61	0.388	0.439	-0.051	*KIAA1549L*	0.064	-0.008
cg10827754	2	101038262	2.10E-61	0.377	0.452	-0.075	NA	0.003	-0.018
cg09363128	16	85478932	2.14E-61	0.704	0.653	0.051	NA	2.04E-06	0.021
cg08722383	7	5594654	2.21E-61	0.312	0.384	-0.072	NA	4.03E-04	-0.021
cg04005943	10	73350048	2.25E-61	0.246	0.308	-0.062	*CDH23*	1.11E-15	-0.044
cg07048516	22	24105087	2.35E-61	0.332	0.399	-0.067	*C22orf15*	1.03E-05	-0.025
**cg23274824**	1	45079966	2.41E-61	0.756	0.703	0.053	*RNF220*	0.831	0.001
cg08729927	16	31467076	2.55E-61	0.313	0.370	-0.057	NA	1.57E-09	-0.031
**cg07258507**	14	23842125	2.80E-61	0.696	0.646	0.051	*IL25*	0.083	0.007
cg08327038	16	69200251	2.92E-61	0.813	0.759	0.054	*CIRH1A*	2.27E-07	0.026
cg22469897	19	51858276	3.01E-61	0.804	0.752	0.052	*ETFB*	5.76E-10	0.029
cg06207961	1	108661230	3.10E-61	0.287	0.356	-0.069	NA	1.97E-12	-0.042
cg26022064	7	98739782	3.44E-61	0.755	0.697	0.058	*SMURF1*	4.28E-05	0.022
cg14055655	19	50117794	3.47E-61	0.852	0.800	0.051	*PRR12*	0.030	0.010
**cg06751366**	16	50280723	3.49E-61	0.209	0.270	-0.061	NA	0.059	-0.010
cg24746726	4	70726093	3.55E-61	0.403	0.476	-0.073	*SULT1E1*	0.047	-0.011
cg05127574	8	121714454	3.57E-61	0.838	0.782	0.056	*SNTB1*	1.05E-08	0.029
cg21302563	2	238936238	3.63E-61	0.376	0.442	-0.066	*UBE2F*	2.07E-09	-0.036
cg25161129	17	66452343	3.76E-61	0.338	0.415	-0.077	*WIPI1*	1.07E-06	-0.034

cg22214889	20	49410828	3.93E-61	0.188	0.241	-0.053	*BCAS4*	4.05E-08	-0.023
cg02963266	14	99681710	3.97E-61	0.738	0.679	0.059	*BCL11B*	4.67E-07	0.026
cg05190176	12	48290002	4.03E-61	0.466	0.527	-0.061	*VDR*	3.66E-06	-0.024
**cg21545720**	1	32405083	4.33E-61	0.785	0.732	0.052	*PTP4A2*	0.059	0.008
cg04454285	16	86016317	4.36E-61	0.222	0.275	-0.054	NA	0.002	-0.014
cg14622879	6	12234001	4.91E-61	0.160	0.213	-0.053	NA	5.06E-06	-0.023
**cg22313574**	8	27468981	4.99E-61	0.224	0.297	-0.073	*CLU*	0.133	-0.011
cg23842572	17	17030253	5.12E-61	0.760	0.704	0.056	*MPRIP*	3.43E-04	0.018
cg14276584	9	99318213	5.29E-61	0.227	0.290	-0.064	*CDC14B*	0.006	-0.016
cg21058391	4	160027487	5.45E-61	0.487	0.558	-0.072	NA	1.41E-14	-0.050
cg13288906	2	114656403	5.62E-61	0.582	0.505	0.077	*ACTR3*	4.65E-10	0.042
cg21108085	11	44591098	5.75E-61	0.158	0.217	-0.059	*CD82*	1.95E-13	-0.039
cg18998911	3	111718245	5.76E-61	0.316	0.401	-0.085	*TAGLN3*	2.67E-04	-0.029
cg17865265	12	56236845	5.87E-61	0.553	0.610	-0.057	*MMP19*	3.13E-09	-0.030
cg13652336	8	68864012	6.09E-61	0.211	0.263	-0.052	*PREX2*	4.91E-06	-0.023
cg21593835	3	123354036	6.22E-61	0.407	0.458	-0.051	*MYLK*	6.01E-05	-0.018
cg16394290	1	36788416	6.43E-61	0.267	0.345	-0.077	*FAM176B*	1.66E-06	-0.034
cg05824594	12	2734503	6.84E-61	0.296	0.352	-0.056	*CACNA1C*	2.95E-09	-0.028
cg21830221	1	42334284	7.24E-61	0.736	0.672	0.064	*HIVEP3*	5.19E-09	0.032
**cg17785202**	1	14870226	7.53E-61	0.410	0.493	-0.083	NA	0.264	-0.008
cg25353896	22	44568753	7.56E-61	0.245	0.304	-0.060	*PARVG*	0.012	-0.013
cg19182289	6	33265386	7.60E-61	0.177	0.227	-0.051	*RGL2*	1.10E-08	-0.027
cg08783514	15	63772233	7.67E-61	0.752	0.688	0.063	NA	2.89E-10	0.035
cg26110064	1	68154614	7.72E-61	0.264	0.324	-0.060	NA	9.16E-05	-0.022
**cg20482145**	17	8525165	7.87E-61	0.781	0.727	0.054	*MYH10*	0.128	0.007
cg18170989	13	49975383	8.08E-61	0.308	0.378	-0.071	*CAB39L*	3.91E-04	-0.022
**cg18317439**	17	643637	8.21E-61	0.343	0.428	-0.085	*FAM57A*	0.125	-0.013
cg22833612	2	9487886	8.41E-61	0.393	0.471	-0.078	*ASAP2*	3.43E-05	-0.028
cg22222281	12	6308758	8.68E-61	0.378	0.449	-0.071	*CD9*	1.19E-08	-0.034
cg14799927	6	30175074	8.83E-61	0.450	0.525	-0.075	*TRIM26*	1.01E-06	-0.030
**cg25393284**	2	942653	8.92E-61	0.709	0.653	0.056	NA	0.243	0.005
cg14032089	17	57917589	9.69E-61	0.827	0.768	0.059	*VMP1*	3.34E-08	0.030
cg18729704	10	105159484	1.04E-60	0.394	0.468	-0.074	*PDCD11*	7.44E-05	-0.025
cg01816726	10	17496989	1.06E-60	0.203	0.261	-0.058	*ST8SIA6*	1.76E-07	-0.030
cg00242341	11	72447419	1.12E-60	0.398	0.472	-0.074	*ARAP1*	0.026	-0.015
cg03217995	7	27203430	1.19E-60	0.238	0.305	-0.068	*HOXA10-HOXA*	2.95E-06	-0.029
cg13100137	1	37937945	1.37E-60	0.508	0.574	-0.066	*LOC728431*	1.16E-16	-0.048
cg17122311	16	28517278	1.41E-60	0.552	0.610	-0.059	*IL27*	1.18E-06	-0.024
cg13650156	7	99970502	1.42E-60	0.433	0.494	-0.061	*PILRA*	2.02E-05	-0.022
cg20726195	6	2892148	1.46E-60	0.662	0.588	0.074	*SERPINB9*	2.88E-07	0.034
cg12045829	17	7452926	1.47E-60	0.617	0.531	0.086	*TNFSF12*	4.04E-06	0.035
**cg23426156**	8	126557901	1.51E-60	0.769	0.711	0.058	NA	0.209	0.007
cg09251291	15	63483946	1.54E-60	0.741	0.685	0.057	*RAB8B*	3.01E-06	0.023
cg09770579	16	17407429	1.59E-60	0.429	0.490	-0.061	*XYLT1*	4.37E-14	-0.040
cg19279257	7	100463508	1.60E-60	0.379	0.448	-0.069	*TRIP6*	1.70E-07	-0.030
cg04628369	20	56247426	1.66E-60	0.157	0.211	-0.054	*PMEPA1*	0.003	-0.016
cg27215185	6	2197772	1.67E-60	0.612	0.665	-0.053	*GMDS*	1.32E-07	-0.024
cg17332198	12	58209913	1.67E-60	0.445	0.497	-0.051	*AVIL*	1.56E-08	-0.025
**cg02327530**	7	100091786	1.69E-60	0.286	0.358	-0.072	*NYAP1*	0.099	-0.011
cg12616923	8	128228107	1.74E-60	0.277	0.339	-0.062	NA	5.73E-08	-0.026
cg24990317	12	124908617	1.77E-60	0.370	0.421	-0.051	*NCOR2*	3.58E-05	-0.018
cg22924250	14	73358241	1.80E-60	0.246	0.314	-0.068	*DPF3*	1.25E-09	-0.039
cg08691235	2	179395935	1.81E-60	0.519	0.579	-0.060	*TTN-AS1*	9.30E-04	-0.016
cg11091914	8	105598224	1.92E-60	0.247	0.308	-0.060	*LRP12*	6.06E-06	-0.024
**cg00690402**	17	46669566	1.94E-60	0.235	0.285	-0.051	*HOXB-AS3*	0.164	-0.006
cg04642300	6	109288592	1.99E-60	0.232	0.292	-0.060	*ARMC2*	6.13E-11	-0.036
cg02224369	11	33758529	2.07E-60	0.254	0.316	-0.062	*CD59*	5.52E-07	-0.027
**cg18625627**	14	81426015	2.13E-60	0.329	0.257	0.072	*TSHR*	0.124	0.008
cg05274316	15	70250464	2.14E-60	0.202	0.258	-0.056	NA	4.84E-04	-0.018
cg14145074	7	5567891	2.15E-60	0.441	0.387	0.054	*ACTB*	2.09E-05	0.018
cg06616081	15	77336938	2.27E-60	0.572	0.639	-0.067	*TSPAN3*	4.68E-04	-0.020
cg04710768	5	71803282	2.30E-60	0.205	0.260	-0.055	*ZNF366*	4.83E-10	-0.033
**cg01040749**	10	134400440	2.37E-60	0.545	0.643	-0.098	*INPP5A*	0.161	-0.011
cg14181409	17	9802860	2.38E-60	0.358	0.429	-0.071	*RCVRN*	7.74E-04	-0.019
cg09788693	1	208063733	2.41E-60	0.187	0.238	-0.051	*CD34*	2.02E-11	-0.032
cg11147155	1	150595206	2.46E-60	0.357	0.431	-0.074	*ENSA*	0.001	-0.023
cg17288892	5	178956874	2.53E-60	0.245	0.303	-0.058	NA	9.49E-10	-0.031
cg18169971	5	134702492	2.61E-60	0.666	0.718	-0.052	*H2AFY*	9.82E-04	-0.015
cg06784232	8	19463150	2.63E-60	0.502	0.586	-0.083	*CSGALNACT1*	0.002	-0.021
cg12966876	2	111873850	2.71E-60	0.763	0.703	0.060	*ACOXL*	1.72E-06	0.025
cg17117981	8	144410972	2.73E-60	0.501	0.561	-0.060	*TOP1MT*	2.80E-06	-0.022
cg27054084	5	122851781	2.90E-60	0.582	0.526	0.056	*CSNK1G3*	2.74E-08	0.025
cg08069287	11	72868833	3.40E-60	0.349	0.406	-0.057	NA	4.99E-08	-0.026

cg20496896	3	46579532	3.43E-60	0.798	0.747	0.050	*LRRC2*	4.81E-07	0.021
cg25028542	7	36429120	3.52E-60	0.202	0.260	-0.058	*ANLN*	5.49E-09	-0.030
cg00944785	1	150825365	3.62E-60	0.278	0.339	-0.060	*ARNT*	1.45E-04	-0.019
cg14083146	20	20694136	3.62E-60	0.427	0.495	-0.068	*RALGAPA2*	6.46E-07	-0.029
cg25187161	1	29510025	3.67E-60	0.257	0.316	-0.059	*SRSF4*	6.08E-18	-0.042
cg19869422	17	2186014	3.71E-60	0.537	0.592	-0.055	*SMG6*	4.93E-09	-0.028
cg01801643	3	43387641	3.72E-60	0.772	0.709	0.063	*SNRK*	1.34E-12	0.041
cg10315334	17	34207332	3.92E-60	0.730	0.678	0.052	*CCL5*	0.027	0.009
cg17084653	22	26822032	4.08E-60	0.540	0.594	-0.054	NA	1.06E-04	-0.017
**cg24401737**	2	202047473	4.31E-60	0.160	0.210	-0.050	*CASP10*	0.207	-0.006
cg02178957	16	85964200	4.33E-60	0.484	0.534	-0.050	NA	9.76E-07	-0.022
cg04641860	10	43891547	4.51E-60	0.796	0.738	0.058	*HNRNPF*	0.001	0.017
cg26256901	1	23918838	4.51E-60	0.733	0.671	0.061	NA	8.49E-04	0.018
cg05980785	2	197583891	4.51E-60	0.392	0.460	-0.068	*CCDC150*	7.04E-05	-0.023
cg07870237	5	131348431	4.72E-60	0.257	0.316	-0.059	*ACSL6*	2.26E-06	-0.024
cg19787125	17	71532174	4.93E-60	0.711	0.658	0.052	*SDK2*	0.002	0.014
cg22312904	8	144639260	5.01E-60	0.427	0.490	-0.063	*GSDMD*	3.82E-06	-0.024
cg08930843	10	31182298	5.31E-60	0.354	0.427	-0.073	*ZNF438*	3.25E-04	-0.023
cg00354542	16	434356	5.60E-60	0.222	0.291	-0.069	*LOC10013436[illegible]*	6.00E-04	-0.023
cg07214920	2	31378999	5.90E-60	0.804	0.747	0.057	NA	5.14E-08	0.030
cg01178899	3	45985168	5.97E-60	0.826	0.776	0.051	*CXCR6*	1.88E-07	0.024
cg11826961	17	38221639	6.02E-60	0.525	0.578	-0.053	*THRA*	1.64E-15	-0.035
**cg00699986**	20	25057444	6.10E-60	0.343	0.418	-0.075	*VSX1*	0.224	-0.008
cg11088672	4	142678613	6.28E-60	0.307	0.370	-0.063	NA	2.01E-09	-0.032
cg26553763	20	31352874	6.46E-60	0.496	0.561	-0.065	*DNMT3B*	4.05E-17	-0.047
cg27598107	2	102759782	6.71E-60	0.341	0.425	-0.084	NA	0.004	-0.022
cg07052737	2	26224428	6.74E-60	0.438	0.520	-0.082	NA	0.016	-0.019
cg17165759	1	229577027	6.75E-60	0.741	0.681	0.060	NA	1.65E-06	0.026
cg02919090	15	64263738	6.90E-60	0.750	0.687	0.063	*DAPK2*	3.24E-05	0.021
cg15009090	11	64163340	7.02E-60	0.230	0.304	-0.074	NA	2.45E-10	-0.042
cg14054883	9	93619467	7.16E-60	0.805	0.749	0.056	*SYK*	4.90E-08	0.028
cg27633342	15	52588279	7.18E-60	0.384	0.449	-0.065	*MYO5C*	1.85E-06	-0.028
cg25727853	1	181098609	7.20E-60	0.402	0.454	-0.052	NA	5.24E-11	-0.030
cg25982657	4	8193353	8.04E-60	0.244	0.296	-0.051	NA	2.58E-08	-0.025
cg16553238	1	9154254	8.09E-60	0.254	0.325	-0.070	NA	0.036	-0.013
cg15335334	17	73642547	8.14E-60	0.161	0.212	-0.052	*RECQL5*	4.37E-05	-0.019
cg15903032	10	101297605	8.41E-60	0.433	0.486	-0.052	NA	3.46E-14	-0.033
cg26708427	18	3250274	8.50E-60	0.820	0.769	0.051	*MYL12A*	2.28E-09	0.027
cg06968912	17	40573643	8.66E-60	0.163	0.220	-0.057	*PTRF*	2.93E-09	-0.032
cg04366628	4	26290463	8.83E-60	0.301	0.374	-0.073	NA	5.51E-07	-0.033
cg24492202	4	2802612	9.11E-60	0.374	0.446	-0.071	*SH3BP2*	0.008	-0.018
cg27059140	17	47355215	9.13E-60	0.580	0.645	-0.066	NA	1.92E-09	-0.032
cg20438687	17	4761644	9.43E-60	0.684	0.741	-0.057	*MINK1*	7.13E-11	-0.030
cg17777592	13	49975418	9.87E-60	0.409	0.476	-0.068	*CAB39L*	1.04E-04	-0.022
cg23787937	12	29303160	1.02E-59	0.200	0.268	-0.067	NA	3.15E-06	-0.029
cg15613991	7	5277080	1.11E-59	0.170	0.221	-0.051	NA	2.06E-07	-0.023
cg12057576	15	78918529	1.21E-59	0.756	0.704	0.052	*CHRNB4*	1.70E-05	0.019
cg11993173	8	54857695	1.25E-59	0.288	0.353	-0.065	*RGS20*	1.87E-04	-0.022
**cg09390241**	4	8174148	1.26E-59	0.749	0.668	0.081	NA	0.677	0.002
cg13616508	7	75957123	1.28E-59	0.178	0.233	-0.055	*YWHAG*	0.003	-0.016
cg23262488	2	112468472	1.34E-59	0.205	0.255	-0.051	NA	1.90E-10	-0.028
**cg13525276**	14	81426012	1.47E-59	0.407	0.326	0.081	*TSHR*	0.468	0.005
cg16498504	11	2043711	1.51E-59	0.300	0.355	-0.055	NA	5.82E-05	-0.019
cg27665985	15	66116727	1.52E-59	0.265	0.325	-0.060	NA	3.23E-07	-0.027
cg06376949	10	91173811	1.55E-59	0.298	0.372	-0.074	*IFIT5*	2.05E-09	-0.036
cg06748146	10	71104724	1.67E-59	0.758	0.703	0.055	*HK1*	4.35E-07	0.026
cg03254137	17	17661258	1.70E-59	0.218	0.277	-0.059	*RAI1*	0.004	-0.016
cg24694018	1	145457621	1.70E-59	0.607	0.661	-0.054	*POLR3GL*	3.42E-08	-0.024
cg07237926	11	117857630	1.73E-59	0.629	0.574	0.055	*IL10RA*	4.25E-04	0.016
cg05481929	16	57505058	1.73E-59	0.518	0.577	-0.058	*POLR2C*	3.54E-08	-0.029
cg11952340	15	63814440	1.75E-59	0.448	0.516	-0.068	*USP3*	0.025	-0.013
cg09492074	15	66263624	1.95E-59	0.285	0.341	-0.056	*MEGF11*	2.01E-07	-0.026
cg06705017	18	77552402	1.97E-59	0.468	0.550	-0.083	NA	4.88E-08	-0.038
cg25467336	7	2757616	1.98E-59	0.364	0.442	-0.078	NA	0.001	-0.022
cg21594043	15	92491578	2.12E-59	0.331	0.395	-0.064	*SLCO3A1*	8.26E-11	-0.038
**cg23161218**	17	7461638	2.14E-59	0.148	0.204	-0.056	*TNFSF13*	0.130	-0.008
cg14511782	7	112724674	2.14E-59	0.332	0.405	-0.073	*GPR85*	1.28E-04	-0.025
cg22057675	7	140103545	2.25E-59	0.268	0.329	-0.061	*RAB19*	9.30E-04	-0.018
cg05670991	19	51628115	2.31E-59	0.355	0.406	-0.051	*SIGLEC9*	1.59E-04	-0.014
cg00421624	1	153746588	2.41E-59	0.262	0.328	-0.066	*SLC27A3*	1.00E-08	-0.035
cg25574175	3	119500929	2.46E-59	0.578	0.631	-0.053	*NR1I2*	2.97E-04	-0.017
cg14014799	10	22606053	2.71E-59	0.221	0.274	-0.053	*COMMD3*	2.84E-04	-0.017
cg10830496	19	35634556	2.76E-59	0.233	0.290	-0.057	*FXYD7*	7.93E-10	-0.033

cg26106778	6	33175147	2.80E-59	0.231	0.283	-0.052	*RING1*	0.031	-0.009
cg14550760	9	135936732	2.87E-59	0.372	0.426	-0.054	*CEL*	7.23E-08	-0.028
**cg09069499**	8	26216910	2.91E-59	0.532	0.609	-0.077	*PPP2R2A*	0.065	-0.012
cg12297590	6	158080750	3.01E-59	0.155	0.207	-0.052	*ZDHHC14*	1.24E-04	-0.018
cg06495586	4	54424787	3.47E-59	0.226	0.283	-0.057	*LNX1*	2.86E-13	-0.039
cg09596674	3	46607350	3.59E-59	0.242	0.298	-0.056	*LRRC2*	0.042	-0.010
cg12912872	11	330536	3.62E-59	0.727	0.673	0.054	NA	4.61E-11	0.032
cg05969360	13	80443435	3.65E-59	0.807	0.755	0.053	NA	2.67E-12	0.034
cg01545109	7	151087534	3.70E-59	0.264	0.324	-0.060	*WDR86*	4.56E-07	-0.025
cg05336188	19	18959506	3.72E-59	0.353	0.406	-0.054	*UPF1*	4.91E-14	-0.030
cg01129847	19	2281919	3.96E-59	0.301	0.362	-0.061	*C19orf35*	0.024	-0.011
cg06712013	12	49759545	4.01E-59	0.277	0.333	-0.056	*SPATS2*	9.97E-06	-0.023
cg19903071	2	160473251	4.12E-59	0.266	0.336	-0.070	*BAZ2B*	0.002	-0.020
cg06094707	17	78445867	4.25E-59	0.607	0.553	0.053	*NPTX1*	0.002	0.013
cg00391741	3	127307227	4.30E-59	0.613	0.678	-0.065	*TPRA1*	3.00E-06	-0.026
cg01879591	2	242954430	4.47E-59	0.304	0.372	-0.068	NA	1.11E-04	-0.025
cg07706695	1	111179008	4.48E-59	0.722	0.647	0.075	NA	1.03E-04	0.027
cg05224770	12	68553819	4.49E-59	0.742	0.669	0.074	*IFNG*	8.25E-11	0.044
cg12799049	1	197811487	4.55E-59	0.285	0.345	-0.061	NA	1.35E-09	-0.034
cg07806328	21	45774664	4.59E-59	0.589	0.646	-0.057	*TRPM2*	7.88E-08	-0.027
**cg26047334**	2	218785909	4.91E-59	0.683	0.624	0.060	*TNS1*	0.249	0.006
cg22941668	5	148810180	5.04E-59	0.357	0.418	-0.061	*MIR143HG*	0.003	-0.016
cg08978665	16	3115707	5.11E-59	0.819	0.764	0.055	*IL32*	4.06E-08	0.026
cg15782984	6	35993792	5.36E-59	0.671	0.728	-0.057	*SLC26A8*	1.04E-05	-0.019
cg14856606	1	207277547	5.60E-59	0.284	0.341	-0.058	*C4BPA*	4.57E-08	-0.028
cg12609063	1	116549227	5.69E-59	0.608	0.542	0.066	*SLC22A15*	2.11E-05	0.024
cg21072795	12	54920931	5.77E-59	0.293	0.357	-0.063	*NCKAP1L*	3.20E-10	-0.035
**cg08594681**	8	27468684	5.78E-59	0.244	0.316	-0.072	*CLU*	0.232	-0.008
cg21578207	1	44870088	5.97E-59	0.214	0.264	-0.050	*RNF220*	6.95E-12	-0.033
cg15834202	5	58865918	6.17E-59	0.661	0.586	0.075	*PDE4D*	2.85E-08	0.039
**cg07401324**	11	48036852	6.20E-59	0.374	0.446	-0.072	*PTPRJ*	0.070	-0.012
cg08039560	21	45575832	6.30E-59	0.779	0.724	0.055	NA	1.82E-11	0.033
cg25390440	2	127413369	6.41E-59	0.300	0.368	-0.067	*GYPC*	1.96E-04	-0.024
cg13535736	9	111863775	6.55E-59	0.663	0.582	0.080	*TMEM245*	6.04E-12	0.050
cg01482790	19	8513923	6.90E-59	0.675	0.611	0.064	*HNRNPM*	4.04E-11	0.037
cg02470874	3	160475336	7.00E-59	0.313	0.364	-0.052	*PPM1L*	1.89E-07	-0.023
cg07725579	20	1472419	7.15E-59	0.235	0.291	-0.056	*SIRPB2*	1.08E-05	-0.023
cg20610654	8	1708447	7.73E-59	0.755	0.693	0.062	NA	1.68E-07	0.029
cg17950169	1	226067900	8.14E-59	0.230	0.286	-0.056	*TMEM63A*	2.28E-05	-0.022
cg24407596	7	98718683	9.42E-59	0.379	0.441	-0.062	*SMURF1*	5.36E-06	-0.024
cg07047068	11	844686	9.43E-59	0.272	0.344	-0.072	*TSPAN4*	0.010	-0.018
cg14928932	6	33401520	9.53E-59	0.365	0.418	-0.053	*SYNGAP1*	0.007	-0.013
cg26224018	1	64942153	9.57E-59	0.326	0.387	-0.062	*CACHD1*	2.17E-06	-0.029
cg26963146	8	141572044	9.84E-59	0.467	0.519	-0.052	*EIF2C2*	0.001	-0.015
cg24054871	22	45073148	9.98E-59	0.181	0.233	-0.052	*PRR5*	0.001	-0.015
cg00883212	2	173795201	1.08E-58	0.471	0.546	-0.075	*RAPGEF4*	2.94E-06	-0.029
cg25140190	18	13641529	1.08E-58	0.385	0.465	-0.081	*C18orf1*	2.31E-06	-0.035
cg19414383	1	17528238	1.09E-58	0.563	0.505	0.058	NA	2.93E-07	0.025
cg19837174	10	6389707	1.16E-58	0.691	0.620	0.071	NA	0.003	0.017
cg18741908	3	169756818	1.24E-58	0.205	0.274	-0.069	*GPR160*	1.62E-04	-0.026
cg05451722	8	145065385	1.28E-58	0.250	0.306	-0.056	*GRINA*	2.27E-06	-0.024
**cg13801402**	1	114429965	1.33E-58	0.475	0.557	-0.082	*AP4B1-AS1*	0.314	-0.007
cg08973191	8	144894328	1.36E-58	0.565	0.635	-0.070	*SCRIB*	9.17E-05	-0.022
**cg16547579**	20	4954333	1.37E-58	0.210	0.276	-0.067	*SLC23A2*	0.058	-0.012
cg01231183	17	79924542	1.39E-58	0.299	0.364	-0.065	NA	7.15E-05	-0.023
cg17530030	6	166699419	1.41E-58	0.250	0.309	-0.059	NA	9.82E-11	-0.035
cg12068366	19	4548354	1.46E-58	0.447	0.507	-0.059	*SEMA6B*	5.55E-04	-0.019
cg17052170	8	144099482	1.50E-58	0.503	0.637	-0.134	*LOC10013366*	5.36E-11	-0.067
cg03204322	1	84767878	1.53E-58	0.199	0.260	-0.062	*SAMD13*	4.34E-07	-0.029
cg14191024	11	110070509	1.56E-58	0.496	0.565	-0.069	*RDX*	0.015	-0.016
cg04005059	2	23719838	1.59E-58	0.349	0.418	-0.070	*KLHL29*	3.24E-05	-0.026
cg14728071	10	22030034	1.59E-58	0.822	0.771	0.051	*MLLT10*	8.74E-08	0.027
cg02549595	16	85947219	1.60E-58	0.505	0.566	-0.062	*IRF8*	4.76E-06	-0.024
cg19821297	19	12890029	1.67E-58	0.479	0.545	-0.066	NA	3.05E-06	-0.026
cg01412469	7	41732237	1.81E-58	0.345	0.396	-0.050	*INHBA*	2.49E-16	-0.037
cg07658590	21	46963587	1.89E-58	0.392	0.453	-0.061	*SLC19A1*	2.46E-11	-0.037
cg14402591	19	4543487	1.90E-58	0.397	0.469	-0.072	*SEMA6B*	2.06E-05	-0.024
cg12022621	1	203734505	2.12E-58	0.531	0.470	0.062	*LAX1*	8.65E-05	0.021
cg16473141	14	24641501	2.13E-58	0.231	0.290	-0.059	*REC8*	7.92E-07	-0.026
cg06949439	1	206729034	2.20E-58	0.654	0.587	0.067	*RASSF5*	3.33E-06	0.024
cg13832670	7	137646869	2.21E-58	0.264	0.324	-0.061	*CREB3L2*	0.002	-0.017
cg13895650	9	84228185	2.35E-58	0.559	0.629	-0.070	*TLE1*	2.83E-07	-0.030
cg16569650	7	2773072	2.53E-58	0.518	0.584	-0.065	*GNA12*	1.07E-05	-0.026

cg20351875	12	65015547	2.61E-58	0.373	0.438	-0.066	*RASSF3*	0.008	-0.015
cg00159243	12	109023799	2.64E-58	0.273	0.325	-0.052	*SELPLG*	1.63E-09	-0.026
cg13358873	2	69271042	2.67E-58	0.410	0.475	-0.065	*ANTXR1*	2.37E-07	-0.031
cg26781466	6	10521612	2.71E-58	0.208	0.262	-0.054	*GCNT2*	6.31E-05	-0.021
**cg09858022**	17	38465333	2.75E-58	0.257	0.307	-0.051	*RARA*	0.067	-0.009
**cg27412857**	10	11387394	2.82E-58	0.347	0.416	-0.069	NA	0.273	-0.007
cg10933959	15	100890907	2.87E-58	0.474	0.553	-0.079	*FLJ42289*	0.003	-0.020
cg22101190	17	77896096	2.88E-58	0.269	0.324	-0.055	NA	0.001	-0.016
**cg01662869**	16	4730410	3.04E-58	0.347	0.410	-0.063	*MGRN1*	0.790	0.001
cg13973985	19	14785604	3.14E-58	0.198	0.251	-0.054	*EMR3*	1.98E-06	-0.025
cg11478024	6	35004780	3.19E-58	0.465	0.544	-0.079	*ANKS1A*	4.63E-04	-0.024
**cg02489202**	3	45505334	3.52E-58	0.310	0.362	-0.052	*LARS2*	0.286	-0.005
cg23895722	11	128776112	3.56E-58	0.310	0.380	-0.070	*KCNJ5*	1.25E-05	-0.026
cg07780377	11	77939490	3.71E-58	0.382	0.439	-0.057	*GAB2*	4.69E-14	-0.040
cg16411857	16	57023191	3.71E-58	0.190	0.265	-0.075	NA	4.01E-20	-0.063
**cg00789960**	14	61227118	4.13E-58	0.494	0.572	-0.078	*MNAT1*	0.145	-0.009
cg16355945	12	132654742	4.23E-58	0.259	0.331	-0.072	NA	8.41E-08	-0.038
cg25320328	1	92953037	4.55E-58	0.318	0.385	-0.067	*GFI1*	5.72E-07	-0.030
**cg07239716**	12	89744488	4.67E-58	0.226	0.298	-0.072	*DUSP6*	0.052	-0.013
cg13487666	9	127021157	5.18E-58	0.204	0.256	-0.052	*NEK6*	9.12E-07	-0.023
**cg05193538**	1	26097991	5.29E-58	0.238	0.293	-0.055	*MAN1C1*	0.457	-0.004
cg07669182	5	39203755	5.32E-58	0.536	0.479	0.057	*FYB*	2.56E-10	0.031
cg19699682	3	119349840	5.44E-58	0.399	0.473	-0.074	NA	5.70E-05	-0.028
cg01611017	14	95780344	5.70E-58	0.171	0.224	-0.053	*CLMN*	0.001	-0.017
cg03313271	13	113913094	5.88E-58	0.563	0.637	-0.074	*CUL4A*	5.71E-05	-0.026
**cg07125981**	10	35330496	6.37E-58	0.429	0.519	-0.090	*CUL2*	0.066	-0.016
cg14004685	11	1750302	6.96E-58	0.716	0.655	0.061	*MOB2*	2.72E-16	0.044
cg18437319	22	43542604	6.97E-58	0.203	0.260	-0.057	NA	9.43E-04	-0.018
cg02457461	8	9913680	7.07E-58	0.240	0.297	-0.057	*MSRA*	0.007	-0.015
cg10438589	4	14531493	7.54E-58	0.392	0.468	-0.076	NA	1.14E-06	-0.033
cg01234420	22	46453808	7.68E-58	0.456	0.529	-0.073	*LOC150381*	4.49E-19	-0.058
cg27615938	17	16344574	7.84E-58	0.288	0.348	-0.059	*C17orf76-AS1*	5.67E-10	-0.032
cg20911718	14	24867491	7.89E-58	0.553	0.609	-0.056	*NYNRIN*	1.25E-05	-0.023
cg20790367	1	31492493	8.13E-58	0.698	0.635	0.063	*PUM1*	0.003	0.017
cg20941855	3	111718457	8.17E-58	0.466	0.542	-0.076	*TAGLN3*	0.005	-0.019
cg00502926	14	21510056	8.23E-58	0.412	0.482	-0.069	*RNASE7*	8.67E-09	-0.036
cg25643644	11	118210017	8.33E-58	0.800	0.741	0.059	*CD3D*	3.23E-14	0.043
cg07385778	3	72320634	8.46E-58	0.241	0.309	-0.068	NA	0.006	-0.018
cg07929956	16	70437897	8.81E-58	0.745	0.695	0.050	*ST3GAL2*	0.003	0.014
cg13421412	3	52529393	9.31E-58	0.145	0.202	-0.057	*STAB1*	0.013	-0.012
cg23549972	3	58389654	9.41E-58	0.742	0.672	0.070	*PXK*	1.48E-10	0.043
cg14236443	17	943270	9.72E-58	0.416	0.469	-0.053	*ABR*	1.26E-08	-0.025
cg03100209	19	49921142	9.79E-58	0.228	0.294	-0.065	*CCDC155*	6.14E-09	-0.036
cg04159302	5	150162068	1.03E-57	0.425	0.494	-0.069	*C5orf62*	1.29E-12	-0.046
cg00682263	15	66188803	1.05E-57	0.346	0.421	-0.075	*MEGF11*	7.71E-05	-0.025
cg10524576	12	63322644	1.10E-57	0.267	0.318	-0.051	*PPM1H*	3.60E-14	-0.035
**cg26445561**	11	92925402	1.24E-57	0.523	0.594	-0.071	*SLC36A4*	0.453	-0.004
cg02806733	2	231731972	1.24E-57	0.197	0.249	-0.052	*ITM2C*	4.45E-13	-0.033
cg02082462	19	848001	1.30E-57	0.242	0.305	-0.063	*PRTN3*	1.28E-06	-0.027
cg06204938	12	125005710	1.31E-57	0.305	0.357	-0.053	*NCOR2*	0.005	-0.013
cg14833933	15	42652294	1.36E-57	0.342	0.405	-0.063	*CAPN3*	1.63E-06	-0.027
cg15352315	2	160655036	1.38E-57	0.206	0.269	-0.062	*CD302*	4.75E-05	-0.024
cg08415973	6	40346114	1.58E-57	0.332	0.391	-0.058	NA	8.80E-08	-0.027
cg09826895	1	111744309	1.61E-57	0.696	0.645	0.050	*DENND2D*	1.63E-09	0.027
**cg25026926**	17	48266268	1.75E-57	0.728	0.676	0.052	*COL1A1*	0.461	0.003
cg15175129	12	65672052	1.86E-57	0.270	0.333	-0.064	*MSRB3*	5.16E-12	-0.042
cg16093065	22	40720633	1.88E-57	0.846	0.795	0.052	*TNRC6B*	9.18E-10	0.031
cg26338428	4	148767763	2.07E-57	0.763	0.692	0.071	*ARHGAP10*	2.45E-11	0.045
cg13441156	3	10335288	2.14E-57	0.439	0.519	-0.080	*GHRL*	0.020	-0.016
cg27614723	15	92399897	2.26E-57	0.524	0.580	-0.056	*SLCO3A1*	4.70E-14	-0.037
cg10506318	5	60239580	2.30E-57	0.295	0.362	-0.067	*ERCC8*	8.23E-04	-0.021
cg15531512	8	123706630	2.34E-57	0.399	0.472	-0.073	NA	7.88E-07	-0.032
cg05538887	2	37416883	2.38E-57	0.711	0.766	-0.054	*SULT6B1*	5.77E-04	-0.015
cg24460268	13	51995368	2.43E-57	0.800	0.749	0.050	*INTS6*	1.03E-04	0.018
cg04609912	16	78528146	2.47E-57	0.672	0.730	-0.058	*WWOX*	0.002	-0.015
cg24916358	10	126315761	2.87E-57	0.372	0.300	0.072	*FAM53B*	1.12E-05	0.025
**cg04611801**	17	56402029	2.90E-57	0.304	0.367	-0.062	*BZRAP1*	0.153	-0.008
cg12756527	10	11284548	2.94E-57	0.779	0.725	0.055	*CELF2*	0.015	0.012
cg23828301	3	3123308	3.01E-57	0.419	0.485	-0.066	*IL5RA*	2.74E-05	-0.024
cg00895196	6	22147182	3.07E-57	0.845	0.789	0.056	*LINC00340*	1.61E-13	0.032
cg05933510	1	9257092	3.18E-57	0.561	0.625	-0.064	NA	1.61E-05	-0.025
cg17862558	4	106137048	3.28E-57	0.799	0.738	0.061	*TET2*	9.91E-13	0.039
cg24507266	8	145027948	3.38E-57	0.273	0.343	-0.070	*PLEC*	5.97E-04	-0.019

cg10546888	22	38890734	3.45E-57	0.544	0.483	0.060	*DDX17*	2.48E-10	0.034
cg24795903	17	36999666	3.50E-57	0.386	0.440	-0.054	NA	1.62E-05	-0.020
**cg24385652**	10	50329828	3.70E-57	0.780	0.729	0.051	NA	0.736	0.001
cg22301128	4	77011716	3.92E-57	0.261	0.326	-0.065	*ART3*	1.68E-06	-0.028
cg22163059	12	51610927	4.06E-57	0.309	0.363	-0.054	*POU6F1*	4.31E-08	-0.027
cg12013713	7	139760671	4.06E-57	0.660	0.741	-0.081	*PARP12*	1.25E-15	-0.046
cg03794617	17	78796999	4.20E-57	0.635	0.693	-0.058	*RPTOR*	3.65E-04	-0.018
cg15001615	19	35819775	4.28E-57	0.190	0.248	-0.058	*CD22*	7.98E-08	-0.030
cg19368911	1	245541456	4.61E-57	0.747	0.696	0.052	*KIF26B*	0.001	0.015
cg26206598	20	47445432	4.70E-57	0.493	0.556	-0.063	*PREX1*	1.18E-07	-0.028
cg06436854	16	89043527	4.87E-57	0.136	0.190	-0.054	*CBFA2T3*	3.36E-07	-0.026
cg17789193	19	14533491	4.98E-57	0.323	0.404	-0.081	NA	6.33E-04	-0.025
cg23098068	2	172650722	5.03E-57	0.299	0.357	-0.059	*SLC25A12*	2.42E-04	-0.021
cg12973294	4	119869807	5.11E-57	0.468	0.546	-0.078	*SYNPO2*	7.74E-07	-0.035
cg12924654	7	138348384	5.16E-57	0.574	0.632	-0.058	*SVOPL*	1.48E-14	-0.039
cg13931640	9	137277819	5.25E-57	0.475	0.541	-0.066	*RXRA*	9.75E-04	-0.019
cg10708955	17	8056950	5.46E-57	0.192	0.247	-0.055	*PER1*	0.002	-0.016
**cg11726288**	1	11902283	5.55E-57	0.217	0.279	-0.062	*CLCN6*	0.481	-0.005
cg08519905	12	6308774	5.78E-57	0.340	0.397	-0.057	*CD9*	2.57E-12	-0.037
cg26406292	7	75292248	5.83E-57	0.782	0.728	0.054	*HIP1*	9.45E-06	0.022
cg23281529	13	114823829	6.17E-57	0.349	0.435	-0.085	*RASA3*	0.001	-0.026
cg26475911	17	73056187	6.18E-57	0.388	0.454	-0.066	*KCTD2*	2.94E-04	-0.022
cg22108563	5	176936877	6.47E-57	0.184	0.239	-0.055	*DOK3*	0.036	-0.011
**cg08537367**	16	87469199	6.92E-57	0.246	0.306	-0.060	*ZCCHC14*	0.199	-0.007
cg12125614	17	76886835	6.95E-57	0.211	0.274	-0.063	*LOC10065351*	0.005	-0.019
cg08941853	1	59449033	6.96E-57	0.275	0.329	-0.054	NA	7.37E-07	-0.024
cg07376232	11	118084920	7.40E-57	0.166	0.218	-0.051	*AMICA1*	1.98E-08	-0.027
cg12690119	3	141043080	7.43E-57	0.410	0.467	-0.056	*ZBTB38*	0.004	-0.015
cg21486510	2	7171998	7.54E-57	0.277	0.328	-0.051	*RNF144A*	0.006	-0.011
cg14262955	9	132114769	7.70E-57	0.191	0.243	-0.052	NA	2.44E-05	-0.019
cg13460556	15	89709638	7.87E-57	0.490	0.550	-0.059	*ABHD2*	2.73E-08	-0.030
**cg13146674**	11	68095623	8.19E-57	0.182	0.238	-0.056	*LRP5*	0.157	-0.008
cg06815950	12	111619455	8.22E-57	0.325	0.397	-0.072	*CUX2*	0.016	-0.017
cg14140379	4	6695380	8.40E-57	0.490	0.558	-0.067	*S100P*	2.86E-04	-0.022
cg25826463	19	3369820	8.76E-57	0.412	0.482	-0.070	*NFIC*	3.63E-04	-0.019
cg08427067	11	95015466	8.78E-57	0.612	0.664	-0.053	NA	1.35E-04	-0.018
cg19334176	10	443029	8.80E-57	0.393	0.464	-0.071	*DIP2C*	5.03E-09	-0.036
cg16021018	3	46449313	8.83E-57	0.210	0.266	-0.055	*CCRL2*	6.44E-07	-0.026
cg18575809	14	21540329	8.83E-57	0.255	0.318	-0.063	*ARHGEF40*	1.75E-10	-0.039
cg08170427	8	144410815	9.10E-57	0.392	0.461	-0.068	*TOP1MT*	1.53E-08	-0.037
cg04195125	2	88327530	9.80E-57	0.292	0.359	-0.067	*KRCC1*	1.63E-04	-0.024
cg17314888	7	73442282	9.84E-57	0.248	0.305	-0.057	*ELN*	3.01E-07	-0.025
cg15474337	19	50848247	1.04E-56	0.667	0.605	0.062	NA	9.20E-04	0.018
cg24809529	7	100881286	1.05E-56	0.408	0.469	-0.061	*CLDN15*	9.55E-04	-0.019
cg01565438	17	33776554	1.10E-56	0.354	0.412	-0.058	*SLFN13*	9.05E-05	-0.020
cg18158859	13	107144026	1.10E-56	0.230	0.298	-0.068	*EFNB2*	3.81E-06	-0.030
cg09909069	13	41860793	1.11E-56	0.809	0.752	0.057	NA	3.30E-10	0.033
cg18096987	3	11623873	1.13E-56	0.377	0.437	-0.060	*VGLL4*	0.002	-0.018
cg17074014	17	3704494	1.14E-56	0.391	0.469	-0.078	*ITGAE*	0.002	-0.022
**cg15520845**	1	205688760	1.17E-56	0.501	0.579	-0.078	*NUCKS1*	0.433	-0.005
cg22217449	21	47845788	1.17E-56	0.818	0.765	0.053	*PCNT*	1.08E-07	0.027
cg22388948	6	82460558	1.18E-56	0.262	0.328	-0.066	*FAM46A*	7.06E-05	-0.026
cg09672452	15	55665236	1.19E-56	0.260	0.319	-0.058	*CCPG1*	8.90E-06	-0.024
**cg14717752**	5	14686255	1.21E-56	0.690	0.635	0.055	*FAM105B*	0.176	0.006
**cg11258381**	1	117350694	1.23E-56	0.767	0.711	0.056	NA	0.234	-0.005
cg13222915	1	184598594	1.25E-56	0.466	0.516	-0.050	NA	7.95E-10	-0.027
**cg19005210**	6	41168960	1.29E-56	0.239	0.302	-0.063	*TREML2*	0.457	-0.005
cg01810863	12	56359916	1.31E-56	0.426	0.484	-0.059	*PMEL*	5.78E-05	-0.021
cg17118262	17	32690569	1.41E-56	0.242	0.304	-0.062	*CCL1*	2.91E-04	-0.021
cg00992687	5	54275155	1.43E-56	0.639	0.711	-0.072	*ESM1*	2.77E-05	-0.027
cg19620383	15	60860767	1.44E-56	0.676	0.622	0.054	*RORA*	0.003	0.015
cg06676778	5	148209341	1.44E-56	0.663	0.597	0.066	NA	6.38E-07	0.031
cg16642299	12	7071383	1.45E-56	0.258	0.325	-0.066	NA	1.35E-06	-0.031
cg11321083	11	64638190	1.46E-56	0.290	0.345	-0.055	*EHD1*	3.63E-11	-0.031
cg25330422	17	40467382	1.47E-56	0.723	0.665	0.058	*STAT3*	1.47E-13	0.039
cg24000908	5	1113358	1.52E-56	0.284	0.335	-0.051	*SLC12A7*	8.68E-22	-0.043
cg22855020	8	42356731	1.57E-56	0.412	0.486	-0.075	*SLC20A2*	9.65E-08	-0.032
cg06752040	10	14647373	1.58E-56	0.676	0.617	0.059	*FAM107B*	5.51E-08	0.029
cg16222326	8	1708438	1.61E-56	0.751	0.689	0.062	NA	1.65E-05	0.026
cg26969179	10	127738328	1.66E-56	0.295	0.349	-0.054	*ADAM12*	3.70E-05	-0.021
cg24312537	8	38831332	1.70E-56	0.434	0.508	-0.074	*HTRA4*	0.013	-0.016
cg11284147	8	144437914	1.70E-56	0.342	0.416	-0.073	*TOP1MT*	1.03E-06	-0.033
cg18247172	15	91370233	1.71E-56	0.828	0.775	0.052	NA	6.57E-04	0.017

**cg24235882**	4	54928822	1.76E-56	0.423	0.486	-0.063	*CHIC2*	0.795	-0.001
cg04065472	2	131148175	1.84E-56	0.194	0.246	-0.052	NA	1.44E-13	-0.039
cg21380380	7	8011838	1.84E-56	0.707	0.630	0.077	*GLCCI1*	7.28E-17	0.058
cg25472296	10	129076569	1.93E-56	0.493	0.561	-0.069	*DOCK1*	0.005	-0.017
cg15484375	11	18287647	2.04E-56	0.441	0.500	-0.059	*SAA1*	1.77E-12	-0.037
cg10348234	14	31698967	2.07E-56	0.221	0.273	-0.053	NA	7.46E-07	-0.026
**cg18810664**	10	31074367	2.09E-56	0.217	0.292	-0.075	NA	0.370	-0.006
cg17635080	12	109030115	2.12E-56	0.500	0.432	0.068	NA	1.94E-05	0.027
cg22033476	2	43532275	2.23E-56	0.382	0.460	-0.078	*THADA*	5.01E-04	-0.023
**cg14419424**	10	65388604	2.27E-56	0.235	0.305	-0.070	NA	0.149	-0.010
cg13609544	17	46664647	2.27E-56	0.547	0.608	-0.062	NA	3.47E-04	-0.019
cg05687149	11	112035945	2.34E-56	0.265	0.320	-0.055	*IL18*	3.60E-07	-0.025
cg23836594	17	37894258	2.40E-56	0.317	0.373	-0.056	*GRB7*	0.002	-0.015
cg20627754	2	232582394	2.45E-56	0.454	0.525	-0.071	NA	5.08E-09	-0.036
**cg05660874**	19	2332485	2.49E-56	0.349	0.400	-0.051	*SPPL2B*	0.084	-0.007
cg25341726	16	28518331	2.68E-56	0.392	0.465	-0.073	*IL27*	3.47E-04	-0.022
cg12646786	1	178986264	2.77E-56	0.191	0.247	-0.057	NA	1.25E-05	-0.024
cg27428551	12	122155069	2.99E-56	0.561	0.632	-0.071	*TMEM120B*	0.009	-0.016
cg18229767	11	67776576	3.00E-56	0.203	0.255	-0.052	*ALDH3B1*	2.38E-06	-0.022
cg14249174	4	153814351	3.07E-56	0.610	0.671	-0.061	*ARFIP1*	2.35E-04	-0.019
cg08276755	20	30196714	3.08E-56	0.610	0.672	-0.062	NA	0.002	-0.018
cg14373410	14	81426234	3.15E-56	0.809	0.750	0.059	*TSHR*	2.05E-08	0.030
cg21203569	11	47279365	3.28E-56	0.277	0.337	-0.061	*NR1H3*	3.44E-07	-0.031
cg24334029	20	58296208	3.32E-56	0.583	0.659	-0.076	*PHACTR3*	7.01E-08	-0.035
cg12126706	1	156889128	3.36E-56	0.341	0.424	-0.083	*LRRC71*	9.33E-10	-0.041
cg17658002	19	11689997	3.38E-56	0.296	0.350	-0.054	*ACP5*	8.71E-07	-0.025
cg21824010	1	247578859	3.38E-56	0.484	0.534	-0.050	*NLRP3*	1.90E-14	-0.035
cg26311262	3	56858039	3.57E-56	0.350	0.416	-0.066	*ARHGEF3*	1.03E-05	-0.028
cg01769341	1	3526110	3.60E-56	0.425	0.486	-0.061	*MEGF6*	2.93E-16	-0.051
cg16097858	2	46765700	3.65E-56	0.227	0.279	-0.053	NA	5.01E-11	-0.034
cg16267322	14	92864074	3.95E-56	0.572	0.632	-0.060	*SLC24A4*	7.11E-05	-0.020
cg08445740	5	156594400	3.96E-56	0.760	0.699	0.061	*FAM71B*	2.85E-18	0.049
cg27093403	12	25083077	4.28E-56	0.463	0.514	-0.051	*BCAT1*	3.93E-09	-0.023
cg17614506	7	47803072	4.29E-56	0.740	0.675	0.064	*LINC00525*	0.021	0.013
cg10960375	3	42694144	4.47E-56	0.408	0.462	-0.054	*ZBTB47*	7.88E-15	-0.038
cg26814100	6	136724451	4.48E-56	0.552	0.618	-0.066	*MAP7*	8.45E-06	-0.026
cg01458054	20	62200603	4.52E-56	0.827	0.769	0.058	*PRIC285*	3.08E-04	0.016
cg19136371	6	40357140	4.77E-56	0.725	0.663	0.062	NA	1.53E-11	0.037
cg04606020	12	56139467	4.93E-56	0.326	0.392	-0.066	*GDF11*	8.45E-09	-0.035
cg01772743	21	45576085	5.19E-56	0.766	0.709	0.057	NA	2.49E-09	0.032
**cg03404662**	9	114937740	5.31E-56	0.213	0.269	-0.056	*SUSD1*	0.111	-0.009
cg21621538	7	24614206	5.37E-56	0.314	0.368	-0.054	*MPP6*	7.40E-13	-0.035
**cg24742520**	1	19506481	5.52E-56	0.383	0.454	-0.071	*UBR4*	0.130	-0.010
cg06732873	7	35731578	5.53E-56	0.706	0.634	0.071	*HERPUD2*	4.94E-12	0.043
cg15159987	19	17003890	5.74E-56	0.731	0.681	0.051	*CPAMD8*	9.30E-06	0.021
cg00739120	19	36035875	5.77E-56	0.409	0.472	-0.063	*TMEM147*	0.002	-0.018
cg08682625	8	128470793	5.80E-56	0.222	0.279	-0.057	*LOC727677*	3.41E-04	-0.021
cg08792272	8	126959444	6.23E-56	0.774	0.721	0.053	*LOC10013023.*	0.001	0.015
cg03728799	17	74978687	6.35E-56	0.788	0.734	0.054	NA	0.042	0.010
cg04256466	4	169752398	6.43E-56	0.213	0.267	-0.054	*PALLD*	9.63E-12	-0.036
cg15246085	3	49460111	6.54E-56	0.348	0.402	-0.054	*AMT*	2.61E-05	-0.019
cg04015433	8	8820844	6.55E-56	0.133	0.183	-0.050	NA	0.001	-0.015
cg19930116	14	50809588	6.65E-56	0.232	0.288	-0.056	*CDKL1*	1.21E-05	-0.024
cg09351156	6	18436845	7.22E-56	0.363	0.445	-0.082	*RNF144B*	1.66E-04	-0.027
cg13298528	11	118763863	7.24E-56	0.768	0.715	0.053	*CXCR5*	3.25E-05	0.021
cg10978799	1	206945924	7.27E-56	0.274	0.327	-0.054	*IL10*	8.78E-16	-0.040
**cg05931439**	14	23305957	7.69E-56	0.230	0.296	-0.065	*MMP14*	0.515	0.003
cg09973676	8	82006417	8.00E-56	0.459	0.338	0.121	*PAG1*	5.16E-08	0.056
cg02392124	10	28876249	8.25E-56	0.384	0.455	-0.071	*WAC*	0.001	-0.020
cg17990365	11	319718	9.48E-56	0.504	0.614	-0.110	*IFITM3*	5.92E-23	-0.083
cg18234111	1	158155757	1.01E-55	0.217	0.269	-0.052	*CD1D*	4.72E-19	-0.047
cg23665802	13	92002338	1.12E-55	0.306	0.368	-0.061	*MIR17HG*	6.88E-18	-0.048
cg13049483	18	9712461	1.15E-55	0.398	0.461	-0.063	*RAB31*	1.78E-09	-0.035
cg23029655	12	53591756	1.20E-55	0.311	0.385	-0.073	*ITGB7*	0.006	-0.018
cg11612905	2	234359654	1.22E-55	0.550	0.613	-0.062	*DGKD*	7.44E-05	-0.023
cg17611046	6	5672954	1.25E-55	0.443	0.510	-0.067	*FARS2*	5.68E-10	-0.037
cg14375890	6	13355672	1.31E-55	0.735	0.679	0.056	NA	0.002	0.017
cg00982136	2	201726139	1.41E-55	0.506	0.431	0.075	*CLK1*	0.002	0.020
cg22335223	11	117698911	1.53E-55	0.613	0.547	0.066	*FXYD2*	1.42E-04	0.022
cg18550262	7	72812030	1.54E-55	0.261	0.318	-0.057	NA	8.19E-08	-0.026
cg24537836	12	51782159	1.55E-55	0.293	0.343	-0.050	*GALNT6*	6.14E-05	-0.019
cg09306696	16	57729107	1.56E-55	0.255	0.318	-0.064	*CCDC135*	1.74E-04	-0.020
cg22127848	17	64295986	1.57E-55	0.608	0.670	-0.062	NA	1.26E-04	-0.021

cg27496339	7	43803803	1.63E-55	0.205	0.258	-0.053	*BLVRA*	4.02E-05	-0.021
cg07000713	12	62463441	1.63E-55	0.792	0.735	0.057	*FAM19A2*	1.67E-07	0.030
cg24592751	4	140088996	1.68E-55	0.411	0.477	-0.066	NA	0.022	-0.013
cg16153042	6	3282307	1.68E-55	0.518	0.571	-0.053	*SLC22A23*	1.45E-08	-0.027
cg10133935	19	1423753	1.70E-55	0.553	0.623	-0.070	*DAZAP1*	0.009	-0.016
cg04179740	10	73516760	1.72E-55	0.167	0.218	-0.051	*C10orf54*	3.11E-05	-0.022
**cg17359629**	13	114259310	1.79E-55	0.449	0.530	-0.082	*TFDP1*	0.160	-0.011
cg04173396	8	61821400	1.79E-55	0.293	0.355	-0.062	NA	0.001	-0.019
**cg27416489**	2	149823115	1.86E-55	0.459	0.527	-0.068	*KIF5C*	0.550	-0.003
cg04106092	16	47760096	1.96E-55	0.580	0.633	-0.054	NA	2.34E-12	-0.034
cg08066875	2	163197084	1.97E-55	0.504	0.559	-0.055	NA	3.47E-09	-0.029
**cg03376308**	10	3497811	1.98E-55	0.369	0.429	-0.060	NA	0.090	-0.009
cg26197915	11	48085137	1.99E-55	0.289	0.347	-0.058	*PTPRJ*	3.93E-07	-0.027
cg23006227	4	174423637	2.03E-55	0.315	0.383	-0.069	NA	0.004	-0.019
cg16396933	10	104954103	2.03E-55	0.380	0.449	-0.070	*NT5C2*	3.79E-09	-0.037
cg08347373	2	160653686	2.09E-55	0.373	0.445	-0.072	*CD302*	1.23E-05	-0.030
cg26646867	5	68785451	2.19E-55	0.352	0.419	-0.067	NA	3.99E-08	-0.033
cg08837884	19	4540782	2.29E-55	0.629	0.682	-0.054	*LRG1*	1.84E-06	-0.022
cg04217515	21	46325853	2.34E-55	0.416	0.477	-0.061	*ITGB2*	3.64E-08	-0.029
**cg01441127**	1	181012492	2.39E-55	0.478	0.551	-0.073	*MR1*	0.506	-0.004
**cg21762728**	6	170165905	2.46E-55	0.465	0.540	-0.075	*C6orf70*	0.122	-0.010
cg17398227	20	57582787	2.63E-55	0.276	0.332	-0.056	*CTSZ*	4.46E-06	-0.023
cg18038207	12	80581062	2.67E-55	0.405	0.468	-0.063	NA	2.32E-13	-0.045
cg00158770	8	19271585	2.78E-55	0.540	0.630	-0.091	*CSGALNACT1*	4.73E-09	-0.045
cg11527913	1	230410329	3.07E-55	0.273	0.333	-0.061	*GALNT2*	1.23E-06	-0.029
cg08863777	11	94278457	3.21E-55	0.423	0.473	-0.050	*FUT4*	0.006	-0.010
cg14372792	15	93188381	3.32E-55	0.264	0.321	-0.057	*FAM174B*	1.04E-07	-0.029
**cg07428959**	4	129783057	3.42E-55	0.388	0.457	-0.069	*PHF17*	0.358	-0.006
cg03717364	2	43202481	3.47E-55	0.224	0.286	-0.062	NA	3.53E-08	-0.031
cg09570614	2	112124380	3.67E-55	0.467	0.521	-0.054	NA	9.34E-13	-0.034
cg22635096	21	46550644	3.72E-55	0.347	0.399	-0.052	*ADARB1*	1.11E-14	-0.035
cg15535471	7	139332087	3.81E-55	0.790	0.733	0.057	*HIPK2*	2.98E-04	0.020
cg16526047	1	949893	3.82E-55	0.584	0.674	-0.090	*ISG15*	5.32E-17	-0.054
cg23230929	2	87883547	3.87E-55	0.458	0.509	-0.051	NA	8.71E-12	-0.031
cg03878190	2	220047358	4.05E-55	0.212	0.271	-0.060	*FAM134A*	2.16E-07	-0.030
cg08267038	3	113325595	4.11E-55	0.231	0.291	-0.061	*SIDT1*	3.98E-07	-0.029
cg06092310	1	26097918	4.25E-55	0.239	0.296	-0.057	*MAN1C1*	0.045	-0.010
cg21671607	11	36616015	4.36E-55	0.177	0.230	-0.053	*RAG2*	6.65E-08	-0.026
cg01003666	7	139929429	4.42E-55	0.634	0.574	0.060	NA	4.64E-07	0.027
cg07372034	22	48486164	4.48E-55	0.157	0.214	-0.056	NA	8.65E-05	-0.021
cg04728310	19	45947905	4.65E-55	0.232	0.290	-0.058	NA	0.012	-0.013
cg26243740	8	42012513	4.72E-55	0.597	0.525	0.072	*AP3M2*	3.19E-09	0.039
cg02310103	3	50391305	4.82E-55	0.321	0.373	-0.053	*CYB561D2*	5.10E-10	-0.030
cg22712955	1	144935409	5.16E-55	0.778	0.714	0.064	*PDE4DIP*	3.06E-07	0.030
cg18067134	3	71084634	5.42E-55	0.498	0.571	-0.073	*FOXP1*	0.006	-0.017
cg05261686	16	50743296	5.73E-55	0.289	0.348	-0.059	*NOD2*	2.43E-06	-0.026
**cg06900494**	17	48609332	6.18E-55	0.692	0.641	0.051	*EPN3*	0.369	0.004
cg21103992	22	18325505	6.30E-55	0.773	0.723	0.050	*MICAL3*	2.75E-05	0.018
cg06192883	15	52554171	6.37E-55	0.358	0.298	0.061	*MYO5C*	1.62E-15	0.040
cg14989988	7	105319819	6.52E-55	0.423	0.483	-0.060	*ATXN7L1*	1.95E-11	-0.037
cg16324409	4	3416370	6.60E-55	0.227	0.288	-0.061	*RGS12*	3.79E-04	-0.021
cg07566707	10	30980103	6.91E-55	0.591	0.658	-0.067	NA	8.27E-04	-0.020
cg26792295	3	16540091	6.92E-55	0.361	0.422	-0.061	*RFTN1*	0.012	-0.014
cg05988548	7	38695932	6.93E-55	0.329	0.379	-0.050	NA	2.36E-07	-0.023
**cg16396191**	13	42184765	6.94E-55	0.436	0.515	-0.079	*VWA8*	0.276	-0.007
cg25015371	6	108874950	7.24E-55	0.435	0.492	-0.057	NA	2.39E-10	-0.031
cg21250433	12	10283763	7.47E-55	0.572	0.628	-0.056	*CLEC7A*	5.04E-10	-0.031
cg26316423	10	6104137	7.61E-55	0.727	0.673	0.055	*IL2RA*	0.001	0.013
cg07824907	4	184320560	8.11E-55	0.228	0.287	-0.060	NA	8.75E-05	-0.023
cg11485012	3	52909345	8.57E-55	0.731	0.678	0.054	*TMEM110-MU*	1.03E-08	0.029
cg24750752	19	35819889	8.68E-55	0.174	0.238	-0.064	*CD22*	1.96E-04	-0.024
cg03068068	19	36203512	8.70E-55	0.660	0.592	0.068	*ZBTB32*	3.40E-09	0.037
cg24238409	10	93998677	8.76E-55	0.538	0.605	-0.067	*CPEB3*	4.49E-05	-0.023
cg06489037	3	107706245	8.83E-55	0.538	0.608	-0.069	NA	5.53E-05	-0.027
cg16379704	7	8302251	9.35E-55	0.230	0.282	-0.052	*ICA1*	7.00E-04	-0.018
cg10639435	8	146104221	9.61E-55	0.494	0.437	0.058	*ZNF250*	1.95E-06	0.021
cg26449680	22	38714272	9.83E-55	0.375	0.426	-0.051	*CSNK1E*	1.34E-09	-0.024
cg17711527	1	203734396	1.02E-54	0.518	0.450	0.068	*LAX1*	8.37E-05	0.025
cg14225168	9	139403827	1.03E-54	0.390	0.455	-0.065	*NOTCH1*	4.19E-06	-0.028
cg19843457	12	132288611	1.04E-54	0.214	0.267	-0.052	NA	3.22E-05	-0.021
cg27569829	18	60984485	1.10E-54	0.627	0.562	0.066	*BCL2*	5.74E-06	0.028
cg05374956	19	5838735	1.12E-54	0.232	0.289	-0.057	*FUT6*	4.76E-05	-0.022
cg01910727	1	226842455	1.15E-54	0.412	0.471	-0.059	*ITPKB*	1.57E-04	-0.020

cg16052198	19	52264411	1.22E-54	0.198	0.248	-0.050	*FPR2*	0.014	-0.013
cg21167046	4	1295078	1.28E-54	0.329	0.400	-0.070	*MAEA*	0.020	-0.015
cg17740822	12	89744609	1.28E-54	0.185	0.239	-0.054	*DUSP6*	0.002	-0.016
cg24026619	14	59027109	1.32E-54	0.223	0.283	-0.059	NA	1.46E-08	-0.031
cg05474761	16	85414689	1.35E-54	0.324	0.380	-0.056	NA	4.78E-08	-0.025
cg11588787	13	28181449	1.42E-54	0.257	0.312	-0.056	*LNX2*	6.79E-07	-0.027
cg04121771	3	149191624	1.42E-54	0.483	0.548	-0.065	*TM4SF4*	2.88E-10	-0.035
cg16460860	17	17744282	1.49E-54	0.484	0.544	-0.060	NA	4.74E-04	-0.019
cg26094805	17	80740731	1.63E-54	0.372	0.425	-0.053	*TBCD*	5.46E-04	-0.016
cg01546820	6	35453638	1.66E-54	0.772	0.722	0.050	*TEAD3*	0.015	0.010
cg27012421	12	77092891	1.66E-54	0.743	0.693	0.050	NA	0.008	0.013
cg03014241	16	11348611	1.78E-54	0.777	0.720	0.057	*SOCS1*	8.83E-15	0.039
cg16096172	6	46924482	1.82E-54	0.517	0.591	-0.074	NA	7.54E-06	-0.027
**cg03853208**	7	25989763	1.83E-54	0.240	0.311	-0.071	NA	0.515	-0.005
cg13085553	12	133248929	1.86E-54	0.397	0.467	-0.069	*POLE*	0.005	-0.020
cg07265444	7	76956562	2.08E-54	0.222	0.278	-0.056	*PION*	1.06E-04	-0.021
cg21608519	7	27199752	2.09E-54	0.189	0.253	-0.064	NA	5.71E-04	-0.022
cg12011522	2	179192421	2.10E-54	0.697	0.643	0.053	*OSBPL6*	5.80E-06	0.022
cg14942952	22	44576268	2.14E-54	0.475	0.541	-0.065	*PARVG*	2.80E-10	-0.039
cg26075966	7	44999021	2.21E-54	0.285	0.348	-0.063	NA	0.002	-0.018
cg17781669	5	149868359	2.26E-54	0.287	0.351	-0.064	NA	5.15E-09	-0.034
cg20623350	22	40289840	2.27E-54	0.439	0.500	-0.061	*ENTHD1*	8.02E-07	-0.028
cg17901584	1	55353706	2.28E-54	0.479	0.557	-0.078	*DHCR24*	4.52E-08	-0.035
cg21417204	18	48609268	2.33E-54	0.693	0.625	0.068	*SMAD4*	9.07E-14	0.045
cg16329589	4	174430141	2.46E-54	0.151	0.208	-0.056	NA	0.024	-0.013
**cg08438529**	16	1052939	2.51E-54	0.653	0.595	0.058	NA	0.352	0.004
cg26601310	11	36397123	2.53E-54	0.571	0.512	0.060	*PRR5L*	1.81E-05	0.023
cg06174407	22	29470232	2.56E-54	0.271	0.325	-0.054	*KREMEN1*	0.011	-0.013
**cg26692003**	3	13063165	2.63E-54	0.852	0.801	0.051	*IQSEC1*	0.292	-0.006
cg22705929	11	57417974	2.69E-54	0.460	0.516	-0.056	*YPEL4*	1.21E-04	-0.021
cg06159562	16	89424793	2.81E-54	0.627	0.543	0.085	*ANKRD11*	3.92E-12	0.050
cg13553498	12	9821986	2.86E-54	0.790	0.718	0.072	*CLEC2D*	1.35E-12	0.049
**cg26681770**	20	56247302	2.94E-54	0.173	0.233	-0.060	*PMEPA1*	0.068	-0.012
cg06547715	2	218990976	2.95E-54	0.296	0.360	-0.064	*CXCR2*	1.05E-05	-0.027
cg08445469	12	53970794	2.98E-54	0.451	0.502	-0.051	*ATF7*	7.82E-11	-0.029
cg18731398	3	123414733	3.00E-54	0.383	0.455	-0.072	*MYLK*	1.29E-04	-0.025
cg17714799	4	110625080	3.02E-54	0.312	0.387	-0.075	*CASP6*	0.003	-0.023
cg07151747	2	158977473	3.03E-54	0.531	0.615	-0.084	*UPP2*	0.011	-0.019
cg05557266	3	49057259	3.05E-54	0.215	0.273	-0.059	*DALRD3*	2.07E-10	-0.035
cg00754253	11	63259187	3.07E-54	0.322	0.390	-0.068	*HRASLS5*	0.001	-0.019
cg17597631	1	8443425	3.09E-54	0.215	0.276	-0.061	*RERE*	1.28E-06	-0.029
cg16103421	15	102068632	3.35E-54	0.338	0.402	-0.064	NA	1.67E-05	-0.025
cg19972822	7	138778744	3.47E-54	0.667	0.614	0.053	*ZC3HAV1*	3.61E-07	0.026
**cg13253847**	17	56402238	3.60E-54	0.312	0.365	-0.053	*BZRAP1*	0.690	-0.002
cg06825878	7	75472540	3.87E-54	0.235	0.297	-0.062	NA	8.52E-08	-0.030
cg19942731	22	45609421	3.91E-54	0.331	0.409	-0.079	*KIAA0930*	5.19E-04	-0.026
**cg02188225**	6	30459255	4.00E-54	0.696	0.640	0.056	*HLA-E*	0.250	0.006
cg24704287	19	13951481	4.00E-54	0.282	0.334	-0.051	NA	2.96E-12	-0.030
cg02043329	13	49740771	4.04E-54	0.621	0.676	-0.055	*FNDC3A*	9.09E-07	-0.024
**cg27386431**	1	200990482	4.19E-54	0.588	0.532	0.055	*KIF21B*	0.387	0.004
cg26118326	1	150547677	4.48E-54	0.727	0.664	0.063	*MCL1*	2.21E-10	0.038
cg12141056	1	184944785	4.76E-54	0.790	0.736	0.053	*FAM129A*	2.12E-10	0.033
cg09535924	2	66671659	4.82E-54	0.255	0.308	-0.053	*MEIS1*	4.85E-10	-0.030
cg10446869	1	155147431	5.21E-54	0.303	0.368	-0.065	*TRIM46*	3.88E-06	-0.028
cg25596082	10	43626561	5.26E-54	0.216	0.268	-0.052	NA	4.61E-06	-0.022
cg27576485	17	40558063	5.28E-54	0.395	0.474	-0.079	*PTRF*	0.001	-0.024
cg09326832	6	7243643	5.44E-54	0.251	0.307	-0.056	*RREB1*	2.43E-06	-0.026
**cg22731637**	11	45231545	5.92E-54	0.267	0.319	-0.052	*PRDM11*	0.071	-0.008
cg20808080	2	6962835	6.34E-54	0.793	0.727	0.066	NA	4.42E-13	0.046
cg15922057	17	25784714	6.38E-54	0.259	0.316	-0.057	NA	1.79E-07	-0.029
cg25417766	19	52191468	6.64E-54	0.326	0.388	-0.061	NA	9.07E-08	-0.033
cg17173896	4	24975754	6.75E-54	0.383	0.445	-0.061	*CCDC149*	8.88E-05	-0.021
**cg04403415**	12	124991087	7.03E-54	0.167	0.224	-0.057	*NCOR2*	0.111	-0.010
cg14207267	12	113655841	7.05E-54	0.303	0.367	-0.064	*IQCD*	3.56E-04	-0.021
**cg03736807**	12	54654045	7.09E-54	0.456	0.519	-0.063	*CBX5*	0.992	-0.001
cg23228178	1	17633681	7.12E-54	0.708	0.655	0.053	*PADI4*	0.004	0.013
cg10147974	12	1759064	7.19E-54	0.299	0.356	-0.057	NA	2.40E-04	-0.021
cg04516672	10	126411663	7.43E-54	0.661	0.601	0.060	*FAM53B*	3.38E-10	0.037
cg05576959	11	46366833	7.47E-54	0.214	0.268	-0.053	*DGKZ*	0.003	-0.014
cg26063719	10	17273187	7.62E-54	0.273	0.333	-0.059	*VIM*	1.52E-12	-0.039
cg02490034	7	130125836	7.80E-54	0.370	0.429	-0.059	*MEST*	6.07E-07	-0.027
**cg18349298**	3	158450550	8.25E-54	0.325	0.388	-0.063	*RARRES1*	0.926	0.000
cg08477158	17	58807667	8.42E-54	0.302	0.360	-0.058	*BCAS3*	4.59E-07	-0.026

**cg10101773**	14	75894136	8.57E-54	0.156	0.212	-0.056	*JDP2*	0.098	-0.009
cg00536939	11	47279352	8.57E-54	0.291	0.356	-0.065	*NR1H3*	9.27E-05	-0.025
cg25282976	17	79005047	8.91E-54	0.352	0.420	-0.068	*BAIAP2-AS1*	0.004	-0.017
cg27067618	19	15751949	9.18E-54	0.217	0.272	-0.055	*CYP4F3*	3.64E-07	-0.027
cg23009042	3	47603786	9.23E-54	0.651	0.590	0.061	*CSPG5*	1.62E-04	0.022
cg08250081	4	10125330	9.34E-54	0.399	0.470	-0.071	NA	4.55E-05	-0.028
cg25840538	13	114146138	9.64E-54	0.237	0.301	-0.064	*DCUN1D2*	0.006	-0.018
cg18356190	4	74964260	1.01E-53	0.235	0.287	-0.052	*CXCL2*	7.84E-07	-0.025
cg08362785	22	40814878	1.03E-53	0.658	0.606	0.052	*MKL1*	2.61E-10	0.030
cg03950476	1	53019814	1.05E-53	0.432	0.494	-0.062	*ZCCHC11*	8.51E-05	-0.024
cg12654199	17	78753826	1.08E-53	0.609	0.671	-0.062	*RPTOR*	6.04E-04	-0.019
cg08832851	12	92535822	1.09E-53	0.482	0.411	0.071	*LOC256021*	0.004	0.019
**cg27434890**	6	135517041	1.13E-53	0.496	0.565	-0.069	*MYB*	0.081	-0.011
cg04794141	14	92325159	1.26E-53	0.265	0.322	-0.057	*TC2N*	7.80E-08	-0.029
cg18464274	12	42537617	1.35E-53	0.330	0.388	-0.058	*GXYLT1*	6.93E-12	-0.040
cg07333021	6	30612330	1.38E-53	0.540	0.599	-0.059	*ATAT1*	0.015	-0.013
cg01056135	18	61253151	1.42E-53	0.311	0.373	-0.061	*SERPINB13*	3.69E-06	-0.028
**cg19796532**	12	50426901	1.44E-53	0.143	0.196	-0.053	NA	0.200	-0.007
cg13467628	2	365559	1.44E-53	0.446	0.526	-0.081	NA	0.022	-0.017
**cg01328500**	3	197639792	1.54E-53	0.820	0.757	0.063	*IQCG*	0.364	0.005
cg26815454	22	40296767	1.60E-53	0.497	0.437	0.060	*GRAP2*	2.97E-05	0.021
cg26160945	12	27396706	1.62E-53	0.626	0.690	-0.063	*STK38L*	0.001	-0.017
**cg01752594**	13	50696070	1.69E-53	0.458	0.533	-0.075	*DLEU2*	0.052	-0.013
cg00388871	20	62530407	1.70E-53	0.259	0.310	-0.051	*DNAJC5*	0.036	-0.009
cg08608952	1	22383198	1.76E-53	0.774	0.721	0.053	*CDC42*	7.67E-08	0.028
cg16638092	17	78800774	1.79E-53	0.468	0.541	-0.073	*RPTOR*	5.72E-05	-0.028
cg16771652	16	50730385	1.84E-53	0.214	0.268	-0.054	*NOD2*	2.12E-06	-0.023
cg16962463	12	89968675	1.84E-53	0.243	0.303	-0.060	NA	1.39E-06	-0.029
cg27150417	20	44462035	2.21E-53	0.210	0.266	-0.056	*SNX21*	0.037	-0.012
cg03419051	17	29814382	2.34E-53	0.505	0.557	-0.052	*RAB11FIP4*	1.53E-10	-0.032
cg09842118	14	21359737	2.37E-53	0.247	0.304	-0.057	*RNASE3*	1.83E-06	-0.027
cg07939743	4	57507828	2.44E-53	0.768	0.712	0.056	NA	6.35E-05	0.022
cg05412396	15	76631569	2.48E-53	0.340	0.400	-0.060	*ISL2*	0.026	-0.013
cg20749792	13	34185166	2.49E-53	0.800	0.743	0.057	*STARD13*	3.97E-04	0.019
cg18527241	7	105319558	2.67E-53	0.214	0.267	-0.053	*ATXN7L1*	0.036	-0.010
cg20634514	1	44703750	2.74E-53	0.416	0.476	-0.060	*ERI3*	5.71E-06	-0.026
cg12907644	11	18270341	2.85E-53	0.512	0.565	-0.053	*SAA2-SAA4*	2.48E-06	-0.023
**cg10807894**	12	125169804	2.92E-53	0.729	0.671	0.057	NA	0.483	0.004
**cg12475879**	17	14251824	3.00E-53	0.444	0.531	-0.087	NA	0.224	-0.009
**cg22335872**	20	896876	3.09E-53	0.727	0.675	0.053	*ANGPT4*	0.063	0.009
cg08471738	2	64373042	3.09E-53	0.656	0.589	0.067	*PELI1*	1.43E-10	0.039
cg07994696	16	11680075	3.24E-53	0.626	0.573	0.053	*LITAF*	1.34E-04	0.017
cg04185310	2	47717244	3.30E-53	0.610	0.666	-0.055	NA	7.33E-05	-0.020
cg25133951	1	178575267	3.32E-53	0.758	0.705	0.052	NA	0.021	0.012
cg01702055	6	13303065	3.40E-53	0.230	0.285	-0.055	NA	2.70E-12	-0.038
cg08079596	2	85999628	3.41E-53	0.178	0.232	-0.054	*ATOH8*	1.51E-09	-0.030
cg04537602	11	118763859	3.42E-53	0.717	0.664	0.053	*CXCR5*	7.19E-05	0.019
**cg17602126**	1	40105687	3.61E-53	0.239	0.294	-0.056	*HEYL*	0.069	-0.009
cg01971407	11	313624	3.81E-53	0.400	0.482	-0.082	*IFITM1*	3.26E-30	-0.071
cg04597393	8	142276533	4.05E-53	0.152	0.202	-0.050	NA	0.008	-0.014
cg21216258	9	140942584	4.19E-53	0.429	0.498	-0.070	*CACNA1B*	1.76E-05	-0.026
cg26472910	13	28673762	4.27E-53	0.217	0.271	-0.054	*FLT3*	7.81E-07	-0.027
cg09544144	4	185777688	4.27E-53	0.306	0.368	-0.061	NA	1.19E-10	-0.037
cg16660547	10	114574152	4.40E-53	0.519	0.592	-0.073	*VTI1A*	0.028	-0.014
cg10129391	4	54582825	4.44E-53	0.342	0.407	-0.064	NA	3.96E-11	-0.040
cg00099766	6	90120705	4.56E-53	0.187	0.242	-0.054	*RRAGD*	0.007	-0.015
**cg22707529**	6	143999715	4.69E-53	0.199	0.274	-0.075	*PHACTR2*	0.213	-0.010
**cg04947907**	12	133345285	4.70E-53	0.196	0.259	-0.063	NA	0.392	-0.005
cg12666727	1	42128487	4.79E-53	0.137	0.189	-0.053	*HIVEP3*	8.31E-07	-0.026
cg24020157	10	43697521	4.94E-53	0.313	0.378	-0.065	*RASGEF1A*	0.002	-0.020
cg04492086	1	8173451	5.21E-53	0.795	0.744	0.050	NA	0.002	0.015
cg16929850	10	18989097	5.23E-53	0.741	0.686	0.054	NA	4.90E-10	0.031
cg01802713	3	42695307	5.40E-53	0.262	0.313	-0.051	*ZBTB47*	1.13E-10	-0.031
cg08321366	14	23305835	5.58E-53	0.240	0.297	-0.057	*MMP14*	2.99E-04	-0.019
cg13584882	10	74836656	5.72E-53	0.402	0.459	-0.056	*P4HA1*	1.12E-07	-0.028
cg15069471	11	3880668	6.36E-53	0.793	0.743	0.050	*STIM1*	4.12E-09	0.030
cg02500691	17	79476186	6.63E-53	0.700	0.646	0.054	NA	9.03E-08	0.025
cg08248297	19	16311377	6.77E-53	0.561	0.637	-0.076	*AP1M1*	5.35E-04	-0.021
cg23318020	13	74805375	6.92E-53	0.732	0.662	0.070	NA	2.46E-17	0.056
cg21746120	11	68142234	7.14E-53	0.390	0.441	-0.051	*LRP5*	2.72E-07	-0.024
cg18711535	15	64736348	7.32E-53	0.775	0.712	0.063	*TRIP4*	1.19E-11	0.043
**cg14567424**	11	67349598	7.45E-53	0.520	0.584	-0.064	*GSTP1*	0.067	-0.011
cg07122905	14	92333765	7.48E-53	0.560	0.504	0.056	*TC2N*	0.026	0.011

cg18132851	6	152085641	7.59E-53	0.243	0.305	-0.063	*ESR1*	2.24E-07	-0.031
cg24312520	17	40489584	7.61E-53	0.593	0.518	0.075	*STAT3*	5.85E-08	0.038
cg05581878	3	159886981	7.87E-53	0.426	0.482	-0.056	NA	1.64E-06	-0.026
**cg04264638**	4	56411138	8.09E-53	0.245	0.296	-0.051	*CLOCK*	0.348	-0.005
cg20116800	17	74258224	8.11E-53	0.212	0.271	-0.059	NA	5.16E-05	-0.024
cg01495299	8	128003923	8.17E-53	0.243	0.295	-0.052	NA	2.70E-09	-0.029
**cg09129050**	11	64478374	8.37E-53	0.439	0.513	-0.074	*NRXN2*	0.101	-0.011
cg13303534	3	171261809	8.58E-53	0.422	0.494	-0.072	NA	4.41E-16	-0.053
cg21876925	1	173175328	8.70E-53	0.210	0.269	-0.059	*TNFSF4*	1.37E-07	-0.031
cg21878650	5	64558623	8.83E-53	0.219	0.289	-0.070	*ADAMTS6*	1.46E-14	-0.052
cg01002030	1	65211677	9.05E-53	0.220	0.278	-0.058	*RAVER2*	5.03E-04	-0.020
cg19677302	12	33048259	9.61E-53	0.511	0.577	-0.066	*PKP2*	0.020	-0.014
cg08357990	2	33359550	1.06E-52	0.151	0.205	-0.054	*LTBP1*	1.96E-11	-0.039
cg00744866	3	33701209	1.07E-52	0.271	0.339	-0.069	*CLASP2*	6.06E-04	-0.022
cg03652525	3	72149324	1.08E-52	0.408	0.460	-0.052	NA	2.22E-08	-0.029
cg17516247	1	174933574	1.09E-52	0.569	0.503	0.066	*RABGAP1L*	4.34E-05	0.026
cg21119074	6	20211654	1.09E-52	0.212	0.265	-0.053	*MBOAT1*	1.19E-15	-0.044
cg01874869	5	134735701	1.10E-52	0.283	0.338	-0.055	*H2AFY*	3.00E-04	-0.020
**cg10692693**	7	101688648	1.11E-52	0.170	0.222	-0.052	*CUX1*	0.180	-0.007
cg02722672	4	2967106	1.12E-52	0.526	0.586	-0.060	*GRK4*	0.010	-0.014
cg20363347	11	69061473	1.12E-52	0.239	0.297	-0.058	*MYEOV*	6.16E-07	-0.029
**cg15309361**	7	2152625	1.18E-52	0.272	0.346	-0.074	*MAD1L1*	0.091	-0.013
cg21695089	17	79273418	1.18E-52	0.250	0.306	-0.055	NA	6.08E-04	-0.018
cg04914946	16	21663932	1.24E-52	0.377	0.446	-0.069	*METTL9*	1.66E-05	-0.029
cg17432627	21	40124277	1.25E-52	0.410	0.488	-0.079	*LINC00114*	7.56E-07	-0.037
cg18067859	17	33776345	1.30E-52	0.545	0.622	-0.076	*SLFN13*	2.51E-04	-0.024
cg23670055	14	34481752	1.47E-52	0.717	0.661	0.056	NA	0.018	0.012
cg27370991	16	2094602	1.47E-52	0.374	0.424	-0.050	*NTHL1*	0.002	-0.014
cg17084751	11	6953558	1.65E-52	0.698	0.638	0.060	*ZNF215*	1.38E-08	0.033
cg07356722	12	131353328	1.72E-52	0.760	0.691	0.069	NA	1.28E-17	0.057
cg05681757	12	32655034	1.78E-52	0.423	0.487	-0.065	*FGD4*	0.046	-0.012
cg01894508	2	70189111	1.78E-52	0.653	0.707	-0.054	*ASPRV1*	5.59E-04	-0.016
cg00879824	6	44747344	1.78E-52	0.189	0.243	-0.054	NA	2.76E-15	-0.045
cg06682039	6	170536047	1.83E-52	0.405	0.487	-0.082	NA	1.79E-07	-0.038
cg01982833	12	27174596	1.85E-52	0.345	0.422	-0.077	*MED21*	1.52E-08	-0.041
cg25336765	8	131008537	1.88E-52	0.254	0.312	-0.058	*FAM49B*	9.20E-04	-0.018
**cg05613718**	7	38355100	1.95E-52	0.271	0.331	-0.059	NA	0.460	-0.004
cg22027471	22	32651776	1.95E-52	0.520	0.446	0.074	*SLC5A4*	4.05E-04	0.024
cg14468692	11	504784	1.95E-52	0.357	0.437	-0.080	*RNH1*	9.89E-06	-0.031
**cg09985344**	16	84648441	1.98E-52	0.809	0.754	0.055	*COTL1*	0.210	0.007
cg21710826	7	20240145	2.01E-52	0.386	0.449	-0.062	*MACC1*	6.05E-04	-0.020
cg15732149	1	207509334	2.06E-52	0.207	0.271	-0.064	*CD55*	0.002	-0.021
cg18947209	2	27960869	2.06E-52	0.374	0.433	-0.058	NA	1.38E-04	-0.022
cg27185267	3	9769333	2.12E-52	0.470	0.526	-0.056	*CPNE9*	7.27E-06	-0.025
cg00192026	4	174411279	2.32E-52	0.586	0.646	-0.059	NA	0.014	-0.013
cg20651018	11	3035856	2.36E-52	0.831	0.773	0.058	*CARS*	1.39E-04	0.023
cg17478979	6	149772150	2.60E-52	0.412	0.488	-0.076	*ZC3H12D*	0.036	-0.013
cg11771383	10	134362126	2.70E-52	0.411	0.475	-0.064	*INPP5A*	6.34E-04	-0.022
cg21602614	12	1949586	2.71E-52	0.191	0.242	-0.051	*CACNA2D4*	8.86E-07	-0.025
cg24394891	10	111646370	2.84E-52	0.395	0.456	-0.060	*XPNPEP1*	3.05E-08	-0.032
cg20813518	1	81929622	2.92E-52	0.385	0.458	-0.073	NA	0.007	-0.018
cg07651316	16	3641320	3.04E-52	0.707	0.769	-0.061	*SLX4*	4.49E-04	-0.019
**cg19047804**	6	30297500	3.05E-52	0.553	0.619	-0.065	*TRIM39*	0.647	-0.002
cg00604840	5	154230173	3.06E-52	0.226	0.290	-0.063	*C5orf4*	2.28E-05	-0.025
cg13388769	3	159665668	3.09E-52	0.823	0.771	0.052	NA	1.09E-08	0.030
cg07272654	16	86011988	3.32E-52	0.330	0.382	-0.052	NA	0.020	-0.011
cg11010552	5	131338478	3.32E-52	0.533	0.587	-0.054	*ACSL6*	5.35E-12	-0.033
cg25603108	14	68714746	3.42E-52	0.247	0.301	-0.054	*RAD51B*	7.85E-10	-0.033
cg15374924	11	126290232	3.69E-52	0.246	0.300	-0.054	NA	0.001	-0.017
cg26646659	5	55776364	3.76E-52	0.750	0.699	0.051	NA	3.67E-04	0.017
**cg02231590**	2	231737958	3.78E-52	0.772	0.711	0.062	*ITM2C*	0.600	-0.003
cg23032427	15	76635953	3.85E-52	0.392	0.450	-0.058	NA	1.75E-08	-0.032
cg20676788	13	49379791	3.95E-52	0.658	0.582	0.077	NA	7.41E-05	0.027
cg01894985	3	123589179	4.30E-52	0.728	0.667	0.060	*MYLK*	3.53E-07	0.030
cg05759347	1	243416723	4.54E-52	0.190	0.244	-0.054	*CEP170*	3.05E-04	-0.020
cg01948202	3	122400474	4.92E-52	0.097	0.156	-0.059	*PARP14*	4.93E-10	-0.041
cg14560703	9	134585592	5.27E-52	0.627	0.552	0.075	*RAPGEF1*	3.89E-10	0.047
cg18105842	17	7341440	5.63E-52	0.356	0.412	-0.055	*FGF11*	0.035	-0.011
cg26513689	1	58717632	5.78E-52	0.239	0.301	-0.061	*DAB1*	0.001	-0.020
cg16545105	5	76248749	5.82E-52	0.513	0.570	-0.057	*CRHBP*	1.44E-10	-0.035
cg26668042	5	1514077	5.86E-52	0.406	0.474	-0.068	*LPCAT1*	4.43E-05	-0.024
cg09674502	1	92953279	5.93E-52	0.302	0.374	-0.072	*GFI1*	0.001	-0.022
cg18550212	11	63435428	6.02E-52	0.243	0.302	-0.059	*ATL3*	2.29E-06	-0.028

cg04406863	20	62177351	6.09E-52	0.551	0.498	0.053	*SRMS*	0.040	0.009
cg02856688	19	2266542	6.63E-52	0.403	0.465	-0.061	NA	8.06E-09	-0.034
cg27518976	1	23886730	6.76E-52	0.372	0.446	-0.074	*ID3*	2.02E-04	-0.025
cg27342781	16	84566279	6.79E-52	0.439	0.517	-0.078	NA	0.007	-0.019
cg07218880	13	115046279	7.62E-52	0.767	0.709	0.058	*UPF3A*	1.21E-04	0.021
cg09101151	16	67581437	7.67E-52	0.263	0.324	-0.060	NA	1.77E-04	-0.021
cg07091220	4	146752115	7.68E-52	0.392	0.460	-0.067	*ZNF827*	7.25E-07	-0.031
**cg20464143**	5	16728219	7.75E-52	0.225	0.287	-0.061	*MYO10*	0.271	-0.007
cg00673191	21	37536923	8.00E-52	0.472	0.525	-0.053	*DOPEY2*	2.37E-06	-0.023
cg18906360	14	60633047	8.26E-52	0.757	0.707	0.050	*DHRS7*	0.003	0.013
**cg03661164**	7	148560646	8.32E-52	0.821	0.769	0.052	*EZH2*	0.093	0.008
cg07571344	20	1472071	8.34E-52	0.194	0.254	-0.060	*SIRPB2*	2.50E-08	-0.034
cg17615629	6	30459867	8.80E-52	0.714	0.657	0.058	*HLA-E*	5.25E-06	0.025
cg02845204	11	71259439	9.08E-52	0.293	0.350	-0.057	*KRTAP5-9*	3.00E-06	-0.025
cg06126721	17	1478065	9.10E-52	0.367	0.455	-0.088	*SLC43A2*	0.005	-0.025
cg15829728	20	31174419	9.20E-52	0.264	0.324	-0.060	NA	0.007	-0.015
cg05639533	7	5258428	1.01E-51	0.147	0.200	-0.052	*WIPI2*	0.002	-0.019
cg11803859	10	125770124	1.04E-51	0.562	0.622	-0.060	*CHST15*	0.044	-0.011
cg02789394	6	112191479	1.04E-51	0.830	0.775	0.055	*FYN*	1.10E-12	0.038
cg04640972	10	8373522	1.09E-51	0.692	0.635	0.057	NA	1.14E-04	0.019
cg10035737	17	42840005	1.16E-51	0.380	0.440	-0.060	*ADAM11*	7.10E-04	-0.019
cg25276892	22	40573076	1.18E-51	0.607	0.547	0.060	*TNRC6B*	1.06E-09	0.034
cg18976418	14	39735496	1.19E-51	0.256	0.310	-0.054	*CTAGE5*	2.50E-06	-0.025
cg14394550	8	22548400	1.24E-51	0.207	0.269	-0.062	*EGR3*	8.25E-04	-0.022
**cg18723409**	11	1911547	1.28E-51	0.203	0.267	-0.064	*LSP1*	0.290	-0.007
cg23433370	12	27396894	1.29E-51	0.205	0.264	-0.059	*STK38L*	0.001	-0.021
cg00954105	17	63739259	1.31E-51	0.811	0.757	0.054	*CEP112*	1.24E-10	0.035
cg23184226	7	97880563	1.34E-51	0.749	0.696	0.053	*TECPR1*	8.22E-06	0.022
**cg06653796**	20	62367805	1.36E-51	0.393	0.316	0.078	*LIME1*	0.085	0.011
cg06486190	7	114612269	1.36E-51	0.390	0.461	-0.071	*MDFIC*	3.22E-04	-0.022
cg08987251	16	58769104	1.36E-51	0.393	0.457	-0.064	*GOT2*	6.31E-07	-0.031
cg01840419	15	100890979	1.37E-51	0.213	0.264	-0.051	NA	1.20E-06	-0.023
cg16760587	4	17517594	1.39E-51	0.377	0.443	-0.066	*CLRN2*	0.005	-0.019
**cg04354689**	16	2660830	1.48E-51	0.609	0.684	-0.075	*LOC652276*	0.594	-0.004
cg10708271	3	112692897	1.52E-51	0.780	0.712	0.067	*CD200R1*	1.44E-10	0.042
cg22230912	3	16331335	1.54E-51	0.784	0.732	0.052	*OXNAD1*	6.31E-06	0.023
cg02474563	10	134442032	1.60E-51	0.183	0.237	-0.053	*INPP5A*	8.18E-04	-0.018
cg09554443	1	167487762	1.61E-51	0.455	0.386	0.069	*CD247*	2.42E-08	0.031
cg07861456	16	75654252	1.68E-51	0.261	0.319	-0.059	*ADAT1*	6.44E-06	-0.024
cg07732336	10	17038941	1.69E-51	0.334	0.399	-0.064	*CUBN*	3.53E-11	-0.041
**cg24131359**	12	69346994	1.71E-51	0.502	0.591	-0.089	*CPM*	0.477	-0.006
cg13384150	1	16939173	1.72E-51	0.173	0.226	-0.052	*NBPF1*	1.73E-05	-0.021
cg16061668	11	70257149	1.82E-51	0.219	0.276	-0.056	*CTTN*	8.32E-05	-0.022
cg16362595	6	15305678	1.95E-51	0.698	0.641	0.057	*JARID2*	1.24E-08	0.031
cg16689761	7	141360223	1.96E-51	0.238	0.293	-0.055	*KIAA1147*	6.64E-06	-0.025
**cg01232748**	4	54928819	1.97E-51	0.382	0.439	-0.057	*CHIC2*	0.819	-0.002
cg08372315	11	113844382	1.97E-51	0.342	0.414	-0.072	*HTR3A*	1.00E-05	-0.027
cg27263049	8	145086300	2.02E-51	0.194	0.245	-0.051	*SPATC1*	1.10E-10	-0.033
cg03427849	4	184578976	2.22E-51	0.585	0.525	0.060	*TRAPPC11*	8.45E-09	0.034
**cg02156723**	1	12100631	2.26E-51	0.816	0.761	0.055	NA	0.864	0.001
cg22491680	12	96389547	2.27E-51	0.458	0.510	-0.053	*HAL*	0.003	-0.013
cg22792910	2	70009351	2.29E-51	0.260	0.313	-0.053	*ANXA4*	1.09E-08	-0.030
cg16503724	3	17130667	2.32E-51	0.560	0.504	0.056	*PLCL2*	3.06E-06	0.023
cg16158027	2	196520930	2.36E-51	0.538	0.608	-0.070	*SLC39A10*	0.002	-0.019
cg25015038	14	89604062	2.41E-51	0.330	0.385	-0.055	NA	1.31E-06	-0.025
cg13568659	6	159438801	2.45E-51	0.700	0.639	0.061	NA	1.02E-07	0.031
cg17465423	12	54784180	2.51E-51	0.282	0.338	-0.056	*ZNF385A*	4.37E-09	-0.031
cg20852250	3	72149422	2.53E-51	0.236	0.292	-0.056	NA	7.57E-04	-0.019
cg15286847	16	84690433	2.59E-51	0.752	0.699	0.053	*KLHL36*	7.79E-06	0.023
cg04450994	6	3318592	2.67E-51	0.548	0.482	0.066	*SLC22A23*	0.003	0.019
cg25616968	17	79416203	2.74E-51	0.391	0.461	-0.071	*BAHCC1*	3.64E-08	-0.033
cg13420364	1	234857659	2.80E-51	0.396	0.451	-0.054	NA	1.83E-15	-0.042
cg02842382	7	27615496	2.88E-51	0.301	0.354	-0.053	*HIBADH*	5.77E-09	-0.030
cg03120555	7	630473	2.90E-51	0.285	0.353	-0.068	*PRKAR1B*	0.008	-0.017
cg00065048	1	27962037	3.04E-51	0.379	0.443	-0.065	*FGR*	2.03E-05	-0.027
cg17727597	5	118159086	3.16E-51	0.757	0.692	0.065	NA	1.69E-08	0.035
**cg06902025**	7	1545815	3.37E-51	0.230	0.290	-0.060	NA	0.183	-0.007
cg13682345	1	90172255	3.41E-51	0.337	0.404	-0.067	*LRRC8C*	0.008	-0.016
cg03971555	1	9788769	3.57E-51	0.372	0.292	0.080	*PIK3CD*	1.86E-09	0.044
cg26478401	10	90749920	3.60E-51	0.250	0.319	-0.069	*FAS*	2.97E-15	-0.053
cg07596065	22	50984393	3.69E-51	0.547	0.600	-0.054	NA	1.13E-15	-0.036
cg12172478	3	16357591	3.94E-51	0.767	0.717	0.050	*RFTN1*	2.30E-08	0.026
cg05264870	2	206599315	3.99E-51	0.453	0.509	-0.055	*NRP2*	5.24E-04	-0.019

cg13795831	20	57582706	4.22E-51	0.327	0.389	-0.062	*CTSZ*	0.006	-0.017
cg16696002	1	172189728	4.27E-51	0.355	0.413	-0.058	*DNM3*	4.35E-06	-0.027
cg04938381	17	66374702	4.27E-51	0.381	0.431	-0.050	*ARSG*	1.09E-06	-0.023
cg15022400	15	45028161	4.31E-51	0.163	0.214	-0.051	*TRIM69*	1.40E-15	-0.037
cg16606773	20	19955806	4.52E-51	0.496	0.556	-0.060	*RIN2*	0.002	-0.014
cg11213983	17	63652954	4.79E-51	0.772	0.721	0.051	*CEP112*	4.02E-05	0.022
cg01046511	17	7742971	4.83E-51	0.530	0.586	-0.056	*KDM6B*	0.031	-0.012
cg20157339	8	103249036	4.98E-51	0.689	0.745	-0.056	*RRM2B*	0.001	-0.015
cg07152894	8	102373664	5.32E-51	0.212	0.263	-0.052	NA	3.20E-04	-0.020
cg04494800	6	149775853	5.39E-51	0.354	0.407	-0.053	*ZC3H12D*	5.92E-06	-0.022
cg20788020	8	105677501	5.41E-51	0.731	0.676	0.055	NA	3.81E-13	0.038
cg06232205	15	40120808	5.49E-51	0.805	0.753	0.052	*GPR176*	1.48E-08	0.030
cg14917244	8	27469001	5.67E-51	0.204	0.266	-0.062	*CLU*	0.012	-0.016
cg10976376	5	96293511	5.69E-51	0.720	0.667	0.053	*LNPEP*	1.06E-04	0.019
cg10131232	20	1610358	6.11E-51	0.364	0.442	-0.078	*SIRPG*	1.77E-09	-0.043
cg08227353	10	102821670	6.59E-51	0.445	0.508	-0.063	*KAZALD1*	5.13E-04	-0.021
cg03752203	8	103874399	6.60E-51	0.717	0.660	0.058	*AZIN1*	1.17E-10	0.038
cg13955347	12	9106586	6.76E-51	0.614	0.538	0.076	NA	3.80E-08	0.040
cg19009471	14	101908998	6.88E-51	0.468	0.411	0.057	NA	5.58E-06	0.022
cg16544737	6	24925385	6.99E-51	0.710	0.650	0.060	NA	4.13E-16	0.049
cg24650940	5	123988226	7.05E-51	0.403	0.454	-0.050	*ZNF608*	4.38E-20	-0.042
cg20746552	10	63809108	7.62E-51	0.691	0.640	0.051	*ARID5B*	8.36E-05	0.017
cg10049789	4	8202531	7.87E-51	0.264	0.345	-0.080	*SH3TC1*	1.52E-09	-0.046
**cg27466532**	11	47471400	7.95E-51	0.525	0.577	-0.052	*RAPSN*	0.613	-0.003
cg26661623	17	7019262	8.04E-51	0.498	0.564	-0.066	*ASGR2*	9.11E-08	-0.035
cg04870120	15	32999748	8.15E-51	0.393	0.477	-0.084	NA	1.94E-06	-0.036
cg20813589	11	504930	8.34E-51	0.388	0.446	-0.058	*RNH1*	2.33E-09	-0.030
cg15835713	5	74147080	8.35E-51	0.225	0.275	-0.051	*FAM169A*	2.13E-09	-0.031
cg07839313	19	17514600	8.70E-51	0.444	0.507	-0.063	*BST2*	1.72E-17	-0.043
cg14604066	9	139590572	8.85E-51	0.784	0.729	0.055	NA	7.05E-05	0.021
**cg19903805**	14	92333771	9.15E-51	0.570	0.505	0.065	*TC2N*	0.187	0.008
**cg16407947**	10	5930543	9.27E-51	0.362	0.449	-0.087	*FBXO18*	0.186	-0.012
cg01185921	10	116444463	9.32E-51	0.537	0.592	-0.054	*ABLIM1*	5.35E-13	-0.039
cg06213060	3	16577726	9.42E-51	0.317	0.379	-0.062	NA	2.45E-12	-0.043
cg04730047	3	99979355	9.77E-51	0.252	0.307	-0.055	*TBC1D23*	2.13E-06	-0.027
cg15920906	12	111619414	9.80E-51	0.320	0.373	-0.053	*CUX2*	3.85E-04	-0.020
**cg22943115**	5	149522468	9.98E-51	0.384	0.454	-0.069	*PDGFRB*	0.184	-0.010
cg16346032	1	180100912	1.02E-50	0.316	0.382	-0.066	NA	0.001	-0.020
cg21253742	15	85305079	1.02E-50	0.727	0.667	0.060	*ZNF592*	6.56E-09	0.035
cg03880355	5	74162880	1.04E-50	0.295	0.365	-0.070	*FAM169A*	2.17E-06	-0.034
**cg09621572**	6	31539973	1.06E-50	0.769	0.717	0.052	*LTA*	0.175	0.007
cg11900393	12	721502	1.08E-50	0.574	0.638	-0.064	*NINJ2*	2.24E-06	-0.028
cg10250177	1	32739752	1.09E-50	0.460	0.391	0.069	*LCK*	2.64E-05	0.028
cg27198632	4	174429263	1.13E-50	0.179	0.233	-0.054	NA	7.01E-05	-0.022
cg02386311	16	12073297	1.14E-50	0.296	0.357	-0.062	*SNX29*	6.37E-16	-0.043
cg13437525	12	51318487	1.15E-50	0.124	0.178	-0.053	*METTL7A*	0.008	-0.015
cg08321942	19	34310625	1.15E-50	0.200	0.252	-0.052	NA	3.56E-04	-0.017
cg10588617	2	43027940	1.17E-50	0.614	0.564	0.050	NA	4.84E-09	0.030
cg20442379	10	60024634	1.22E-50	0.310	0.366	-0.055	*IPMK*	0.001	-0.018
cg04554131	1	25291540	1.34E-50	0.331	0.275	0.056	*RUNX3*	1.44E-04	0.020
cg03038262	11	315262	1.55E-50	0.450	0.573	-0.123	*IFITM1*	2.69E-32	-0.093
cg11093142	13	49075016	1.57E-50	0.218	0.276	-0.058	*RCBTB2*	9.26E-06	-0.028
cg03769939	1	25254129	1.60E-50	0.778	0.722	0.056	*RUNX3*	7.08E-06	0.023
**cg20697025**	7	1553203	1.61E-50	0.147	0.198	-0.051	NA	0.274	-0.005
cg17962756	5	172769199	1.61E-50	0.503	0.575	-0.072	NA	2.98E-04	-0.023
cg12277366	8	54931229	1.62E-50	0.764	0.705	0.060	*TCEA1*	2.66E-06	0.026
cg12483005	1	23474871	1.62E-50	0.778	0.719	0.059	*LUZP1*	1.28E-11	0.041
cg18642720	7	142493865	1.64E-50	0.789	0.730	0.059	NA	2.19E-08	0.034
**cg05217983**	6	45406867	1.67E-50	0.289	0.342	-0.052	*RUNX2*	0.115	-0.009
cg23668335	19	50190825	1.79E-50	0.285	0.354	-0.069	*PRMT1*	0.003	-0.020
cg19935471	8	99048265	1.81E-50	0.480	0.553	-0.073	*MATN2*	0.019	-0.016
cg02903983	14	22968031	1.86E-50	0.725	0.651	0.074	NA	2.14E-13	0.055
**cg07029002**	1	64358899	1.97E-50	0.714	0.664	0.050	*ROR1*	0.381	0.004
cg08129331	17	78560478	2.14E-50	0.229	0.285	-0.055	*RPTOR*	0.001	-0.018
**cg18271897**	17	75316784	2.15E-50	0.390	0.442	-0.052	*SEPT9*	0.112	-0.009
cg27185978	8	25095288	2.17E-50	0.432	0.498	-0.067	*DOCK5*	1.37E-05	-0.025
cg11801411	17	1628727	2.19E-50	0.352	0.407	-0.056	*WDR81*	2.26E-04	-0.020
cg10251229	7	630581	2.25E-50	0.509	0.584	-0.075	*PRKAR1B*	3.70E-06	-0.031
cg09359351	3	122509111	2.27E-50	0.415	0.480	-0.064	*HSPBAP1*	1.20E-10	-0.043
cg20824977	15	59978600	2.29E-50	0.231	0.289	-0.058	*BNIP2*	2.66E-04	-0.022
cg19735804	7	142421812	2.38E-50	0.249	0.306	-0.057	NA	7.72E-14	-0.042
cg10416784	2	58797233	2.66E-50	0.738	0.680	0.058	*FLJ30838*	6.73E-06	0.022
cg07418892	6	52150456	2.66E-50	0.835	0.781	0.054	*MCM3*	3.54E-10	0.035

cg22515654	1	10590672	2.74E-50	0.497	0.572	-0.075	*PEX14*	7.58E-08	-0.037
cg19662895	14	69074455	2.77E-50	0.362	0.419	-0.057	NA	2.20E-07	-0.028
cg01534527	3	38003247	2.84E-50	0.494	0.545	-0.050	*CTDSPL*	9.47E-11	-0.032
**cg22438763**	1	47900256	2.88E-50	0.234	0.303	-0.069	*FOXD2-AS1*	0.419	-0.005
cg09884146	2	65593908	2.92E-50	0.367	0.443	-0.076	*SPRED2*	3.19E-05	-0.033
cg20536364	5	55790102	2.97E-50	0.672	0.727	-0.056	NA	2.28E-11	-0.035
cg23398508	17	70536199	3.04E-50	0.221	0.276	-0.055	*LINC00673*	4.00E-06	-0.026
cg11688093	13	111178359	3.20E-50	0.705	0.646	0.059	*RAB20*	1.64E-04	0.021
cg09841889	2	113552033	3.22E-50	0.251	0.311	-0.060	NA	1.10E-09	-0.037
cg26856257	1	32805350	3.25E-50	0.157	0.211	-0.055	NA	4.10E-15	-0.040
cg13935558	7	138144067	3.36E-50	0.540	0.619	-0.079	*TRIM24*	0.010	-0.018
cg05961212	3	119298195	3.45E-50	0.288	0.343	-0.054	*ADPRH*	8.20E-08	-0.031
**cg19563510**	17	79881483	3.59E-50	0.225	0.287	-0.062	*MAFG*	0.384	0.005
cg10787197	6	11779941	3.64E-50	0.486	0.547	-0.061	*ADTRP*	9.79E-14	-0.044
cg07569918	1	212002970	3.71E-50	0.440	0.515	-0.075	*LPGAT1*	0.010	-0.018
cg02921623	10	105250537	3.81E-50	0.406	0.336	0.070	NA	5.33E-08	0.034
cg11187204	17	36480526	3.84E-50	0.503	0.562	-0.060	NA	0.002	-0.018
cg13420413	1	2347015	3.89E-50	0.711	0.658	0.053	NA	8.50E-07	0.023
cg26690511	3	155422103	4.10E-50	0.367	0.429	-0.062	*PLCH1*	1.19E-06	-0.028
cg03479209	3	58020999	4.11E-50	0.509	0.446	0.062	*FLNB*	0.026	0.012
**cg22096687**	11	87908817	4.13E-50	0.222	0.273	-0.051	*RAB38*	0.085	-0.008
cg00207226	5	126407100	4.38E-50	0.545	0.598	-0.053	*C5orf63*	4.68E-04	-0.019
**cg26177213**	7	4754566	4.39E-50	0.360	0.424	-0.064	*FOXK1*	0.090	-0.011
**cg11354682**	19	10978833	4.68E-50	0.762	0.704	0.058	*C19orf38*	0.586	0.003
**cg17903590**	1	28623081	5.01E-50	0.741	0.687	0.054	NA	0.998	0.000
cg05075562	2	42123725	5.03E-50	0.773	0.722	0.051	NA	2.91E-05	0.019
cg20105257	6	30460244	5.59E-50	0.813	0.761	0.052	*HLA-E*	0.007	0.014
cg19317715	17	40996578	5.85E-50	0.337	0.398	-0.060	*AOC2*	7.06E-08	-0.035
cg00345314	8	67624394	5.95E-50	0.392	0.463	-0.072	*SGK3*	6.38E-09	-0.041
cg08224563	16	20916305	6.19E-50	0.234	0.293	-0.059	*LYRM1*	0.012	-0.015
**cg03000848**	16	396164	6.35E-50	0.805	0.744	0.061	*AXIN1*	0.969	0.000
cg18892446	5	73938574	6.93E-50	0.568	0.628	-0.060	*ENC1*	0.041	-0.012
cg04645070	11	34393106	6.96E-50	0.212	0.266	-0.054	NA	2.28E-09	-0.032
cg07673230	8	98789233	7.53E-50	0.459	0.530	-0.071	*LAPTM4B*	4.23E-09	-0.043
cg01711344	11	67770404	8.05E-50	0.391	0.450	-0.059	*UNC93B1*	0.013	-0.014
cg05868813	13	26594483	8.50E-50	0.325	0.383	-0.058	*ATP8A2*	1.47E-06	-0.027
**cg08756121**	8	8820884	8.83E-50	0.152	0.207	-0.055	NA	0.277	-0.006
cg18119407	2	201980504	8.92E-50	0.693	0.643	0.050	*CFLAR*	1.06E-09	0.026
**cg08815340**	6	5026435	8.93E-50	0.182	0.234	-0.052	NA	0.154	-0.007
cg27209729	11	64428925	9.25E-50	0.535	0.614	-0.079	*NRXN2*	4.57E-27	-0.079
cg24864663	15	43532243	9.57E-50	0.425	0.496	-0.071	*TGM5*	6.22E-04	-0.024
cg01324474	12	54758545	9.73E-50	0.218	0.268	-0.050	*GPR84*	1.36E-05	-0.022
**cg02596819**	16	9045869	1.02E-49	0.536	0.480	0.056	*USP7*	0.232	0.006
cg10692363	10	80327890	1.03E-49	0.403	0.466	-0.064	NA	1.28E-08	-0.034
cg13681468	4	90227879	1.04E-49	0.796	0.737	0.060	*GPRIN3*	1.61E-08	0.035
**cg08500763**	17	37894636	1.09E-49	0.251	0.306	-0.056	*GRB7*	0.072	-0.009
cg19736179	14	50364197	1.09E-49	0.802	0.746	0.056	NA	1.51E-05	0.025
**cg11463271**	5	176936892	1.11E-49	0.190	0.244	-0.054	*DOK3*	0.136	-0.009
cg00936790	8	29027844	1.15E-49	0.751	0.693	0.058	*KIF13B*	1.30E-06	0.029
cg23181133	19	42300812	1.17E-49	0.433	0.494	-0.061	*CEACAM3*	0.001	-0.019
cg12542656	2	43269469	1.17E-49	0.324	0.376	-0.052	NA	6.88E-08	-0.028
cg21356631	4	15702461	1.24E-49	0.348	0.413	-0.065	NA	5.23E-04	-0.021
cg18434367	6	45891455	1.27E-49	0.681	0.626	0.056	*CLIC5*	8.66E-07	0.027
cg22627753	1	988623	1.32E-49	0.850	0.797	0.053	*AGRN*	0.003	0.017
cg10680210	11	64107158	1.34E-49	0.651	0.590	0.061	*CCDC88B*	0.004	0.019
cg17759224	1	235098018	1.36E-49	0.225	0.280	-0.055	NA	1.79E-09	-0.035
cg07092805	13	21005388	1.37E-49	0.496	0.554	-0.058	*CRYL1*	2.66E-04	-0.019
cg13702949	2	656917	1.44E-49	0.728	0.677	0.051	NA	1.43E-05	0.023
cg01610979	4	8193310	1.47E-49	0.279	0.341	-0.062	NA	0.001	-0.020
cg10104487	1	27329335	1.50E-49	0.511	0.570	-0.059	NA	9.57E-18	-0.047
cg12285640	6	140001095	1.51E-49	0.536	0.594	-0.057	NA	0.005	-0.015
cg16429735	5	1268949	1.54E-49	0.732	0.670	0.061	*TERT*	3.55E-10	0.039
cg02253535	21	45147292	1.56E-49	0.216	0.275	-0.059	*PDXK*	0.048	-0.011
cg06245711	17	74260776	1.58E-49	0.599	0.521	0.078	*FAM100B*	1.92E-11	0.050
cg22247041	17	73004677	1.59E-49	0.665	0.614	0.051	NA	1.91E-07	0.026
cg00028013	15	74218697	1.67E-49	0.501	0.567	-0.066	*LOXL1*	3.28E-10	-0.039
cg10713839	4	81047158	1.70E-49	0.700	0.644	0.057	NA	2.80E-11	0.036
cg27220062	15	38848671	1.71E-49	0.666	0.605	0.061	*RASGRP1*	1.55E-05	0.024
cg10798745	1	145715636	1.78E-49	0.698	0.636	0.062	*CD160*	0.031	0.012
cg27430561	7	23633208	1.93E-49	0.517	0.584	-0.067	NA	0.011	-0.017
cg01793320	8	20030779	1.99E-49	0.750	0.691	0.059	*SLC18A1*	0.002	0.018
cg25597390	10	5333615	1.99E-49	0.205	0.260	-0.055	NA	1.69E-07	-0.030
cg00719771	6	23523214	2.01E-49	0.310	0.374	-0.064	NA	1.13E-05	-0.028

cg24067911	6	16729610	2.01E-49	0.306	0.363	-0.057	*ATXN1*	1.03E-09	-0.035
**cg00199007**	20	61583910	2.05E-49	0.158	0.216	-0.058	*SLC17A9*	0.282	-0.007
**cg15322516**	14	104145810	2.11E-49	0.209	0.261	-0.051	*KLC1*	0.499	-0.004
cg26071135	11	843943	2.16E-49	0.238	0.292	-0.054	*TSPAN4*	1.46E-04	-0.022
cg20455854	5	139040849	2.27E-49	0.383	0.437	-0.054	*CXXC5*	4.29E-09	-0.027
cg23702046	12	3371110	2.28E-49	0.544	0.608	-0.064	*TSPAN9*	1.57E-07	-0.031
cg12449049	6	25088999	2.37E-49	0.603	0.663	-0.061	*CMAHP*	2.41E-15	-0.047
cg06340704	13	97877417	2.44E-49	0.767	0.705	0.061	*MBNL2*	4.56E-08	0.031
cg06873024	1	215130954	2.47E-49	0.358	0.417	-0.060	NA	5.63E-14	-0.048
cg18010131	11	10476620	2.54E-49	0.501	0.564	-0.064	*AMPD3*	2.70E-05	-0.026
cg24439686	17	76588724	2.77E-49	0.223	0.282	-0.059	NA	0.002	-0.019
cg11874426	11	118781731	3.07E-49	0.777	0.725	0.051	*BCL9L*	0.003	0.016
cg27280688	21	43823809	3.23E-49	0.793	0.736	0.058	*UBASH3A*	2.55E-08	0.032
cg07197493	11	9884649	3.24E-49	0.427	0.497	-0.070	*SBF2*	7.27E-05	-0.027
cg14522718	9	130868874	3.26E-49	0.799	0.744	0.056	*SLC25A25*	1.29E-09	0.036
cg08329754	17	78797015	3.34E-49	0.536	0.591	-0.055	*RPTOR*	0.004	-0.016
cg21842274	5	76248637	3.47E-49	0.443	0.500	-0.057	*CRHBP*	4.54E-08	-0.030
cg25101396	2	27346296	3.50E-49	0.228	0.281	-0.053	*ABHD1*	8.52E-04	-0.018
cg08461692	17	8481454	3.55E-49	0.539	0.485	0.054	*MYH10*	0.001	0.015
cg26529044	13	43354767	3.59E-49	0.208	0.260	-0.052	*FAM216B*	3.47E-13	-0.037
cg21276056	19	9965368	3.64E-49	0.781	0.731	0.051	*OLFM2*	2.96E-04	0.018
cg11937033	7	155150681	3.81E-49	0.464	0.539	-0.074	NA	0.008	-0.016
cg26742320	2	201393628	3.82E-49	0.631	0.689	-0.058	*SGOL2*	0.005	-0.015
cg20080702	10	74046947	3.87E-49	0.539	0.593	-0.054	NA	8.03E-08	-0.027
cg25902939	19	18544350	3.93E-49	0.256	0.330	-0.074	*SSBP4*	0.013	-0.017
cg03614132	12	89453916	3.99E-49	0.317	0.377	-0.060	NA	5.35E-06	-0.029
cg17416338	19	5074717	4.09E-49	0.201	0.252	-0.051	*KDM4B*	3.28E-04	-0.019
cg06336535	19	17877528	4.13E-49	0.448	0.508	-0.060	*FCHO1*	0.001	-0.018
cg10069121	1	152009711	4.24E-49	0.281	0.339	-0.059	*S100A11*	6.01E-09	-0.036
cg06972043	6	21348636	4.46E-49	0.770	0.706	0.064	NA	8.47E-09	0.038
cg05822633	20	11898531	4.46E-49	0.269	0.320	-0.051	*BTBD3*	1.46E-09	-0.031
cg11285834	4	146102486	4.67E-49	0.271	0.331	-0.061	NA	2.95E-04	-0.023
cg03590031	20	62200285	4.67E-49	0.822	0.772	0.051	*PRIC285*	1.20E-12	0.035
cg11847636	19	50015523	4.78E-49	0.490	0.547	-0.057	*FCGRT*	8.48E-06	-0.022
**cg19893585**	8	145025064	4.83E-49	0.380	0.445	-0.065	*PLEC*	0.225	-0.006
cg07563400	17	15849556	4.86E-49	0.295	0.345	-0.050	*ADORA2B*	2.89E-11	-0.031
cg24854175	5	138126560	4.92E-49	0.816	0.764	0.053	*CTNNA1*	1.33E-06	0.025
cg01281601	16	15931190	5.24E-49	0.633	0.683	-0.051	*MYH11*	5.51E-07	-0.023
cg02039987	2	157291262	5.53E-49	0.553	0.485	0.068	*GPD2*	2.87E-16	0.054
cg12033075	1	9788767	5.62E-49	0.403	0.328	0.075	*PIK3CD*	8.28E-15	0.055
cg09997082	19	46170946	5.67E-49	0.432	0.494	-0.062	*GIPR*	2.87E-11	-0.041
cg25814969	2	8726136	5.90E-49	0.249	0.304	-0.056	NA	0.004	-0.017
cg02916962	2	157180144	6.30E-49	0.223	0.287	-0.064	NA	2.07E-08	-0.034
cg08840913	2	24139933	6.32E-49	0.463	0.516	-0.053	*ATAD2B*	2.59E-07	-0.027
cg09337254	2	85640762	6.79E-49	0.189	0.249	-0.060	*CAPG*	8.81E-05	-0.025
**cg09694051**	12	27181973	6.88E-49	0.444	0.523	-0.079	*MED21*	0.216	0.009
cg26876834	16	2013573	6.91E-49	0.744	0.692	0.053	*RPS2*	0.033	0.011
cg25649895	17	48356195	7.09E-49	0.511	0.564	-0.053	*TMEM92*	6.60E-11	-0.032
cg15818109	6	33142006	7.10E-49	0.440	0.493	-0.053	*COL11A2*	1.04E-06	-0.024
cg15809217	6	31607648	7.18E-49	0.314	0.386	-0.072	*BAG6*	2.45E-08	-0.042
**cg20764656**	14	65410479	7.66E-49	0.229	0.288	-0.058	*CHURC1-FNTB*	0.120	-0.010
cg05579598	16	88989069	8.09E-49	0.365	0.422	-0.057	*CBFA2T3*	3.19E-06	-0.025
cg17685004	2	217419293	8.11E-49	0.810	0.756	0.053	NA	1.42E-04	0.021
cg09451574	4	113069076	8.56E-49	0.551	0.628	-0.076	*C4orf32*	6.85E-13	-0.049
cg07168526	7	116797907	8.98E-49	0.450	0.515	-0.065	*ST7*	8.36E-05	-0.025
cg24651941	20	21281599	9.13E-49	0.643	0.696	-0.053	NA	0.002	-0.016
cg04995291	22	37546325	9.54E-49	0.724	0.670	0.054	*IL2RB*	1.72E-07	0.029
cg22537604	19	43857074	1.02E-48	0.544	0.622	-0.078	*CD177*	0.004	-0.021
cg24084564	19	39892799	1.04E-48	0.724	0.673	0.051	NA	0.001	0.016
cg20782117	7	95865631	1.10E-48	0.539	0.594	-0.055	*SLC25A13*	2.71E-04	-0.019
cg16834726	12	57937408	1.10E-48	0.447	0.504	-0.057	*DCTN2*	2.20E-06	-0.027
cg26580413	16	54025348	1.12E-48	0.308	0.363	-0.055	*FTO*	2.37E-13	-0.038
cg07468327	16	54170217	1.13E-48	0.471	0.540	-0.069	NA	0.002	-0.019
cg18805457	5	145829759	1.14E-48	0.398	0.459	-0.062	*TCERG1*	9.11E-11	-0.036
cg23208285	5	131879579	1.21E-48	0.828	0.776	0.052	*IL5*	1.23E-08	0.031
cg01004762	9	34956991	1.24E-48	0.427	0.482	-0.056	*KIAA1045*	1.77E-08	-0.031
cg04295372	1	101184332	1.33E-48	0.399	0.461	-0.062	*VCAM1*	2.86E-09	-0.037
cg19114214	11	67349590	1.38E-48	0.309	0.363	-0.053	*GSTP1*	0.012	-0.014
cg26306976	2	9564901	1.39E-48	0.556	0.643	-0.087	*ITGB1BP1*	1.75E-06	-0.036
**cg07296256**	13	113438010	1.40E-48	0.218	0.269	-0.050	*ATP11A*	0.538	-0.003
cg08113187	16	87469329	1.42E-48	0.323	0.390	-0.067	*ZCCHC14*	0.004	-0.019
cg26955845	16	57680131	1.48E-48	0.508	0.564	-0.056	*GPR56*	1.60E-08	-0.030
cg16395183	1	210771699	1.58E-48	0.457	0.516	-0.059	*HHAT*	1.38E-06	-0.030

cg01602730	1	153671011	1.59E-48	0.230	0.284	-0.054	NA	0.002	-0.016
cg23352030	20	62198469	1.59E-48	0.788	0.702	0.086	*PRIC285*	3.35E-16	0.064
cg12646029	6	29427451	1.64E-48	0.752	0.698	0.054	*OR2H1*	5.18E-08	0.030
cg20076442	8	72745197	1.66E-48	0.357	0.412	-0.055	NA	8.26E-05	-0.022
cg16152753	10	23732088	1.72E-48	0.306	0.362	-0.056	NA	6.23E-06	-0.026
cg02556345	10	124181965	1.77E-48	0.506	0.451	0.055	*PLEKHA1*	1.89E-07	0.029
cg08516993	17	18872598	1.79E-48	0.337	0.394	-0.057	*SLC5A10*	1.26E-05	-0.025
cg08943045	1	9043919	1.97E-48	0.239	0.292	-0.053	NA	0.034	-0.011
cg01704474	11	504918	2.06E-48	0.321	0.383	-0.062	*RNH1*	2.65E-08	-0.033
cg15887927	13	29148952	2.08E-48	0.218	0.272	-0.054	NA	3.95E-04	-0.020
cg11973132	11	67052656	2.17E-48	0.467	0.521	-0.053	*ADRBK1*	0.021	-0.011
cg17352215	15	90698455	2.22E-48	0.548	0.620	-0.072	NA	0.002	-0.021
cg00232092	7	5518887	2.24E-48	0.578	0.636	-0.058	*FBXL18*	0.007	-0.015
cg04858631	10	74035570	2.33E-48	0.636	0.575	0.061	*DDIT4*	1.62E-12	0.043
cg20567280	3	101289945	2.34E-48	0.776	0.721	0.055	NA	3.26E-10	0.037
cg08984272	2	149884320	2.42E-48	0.508	0.565	-0.058	NA	7.11E-05	-0.021
cg19013753	15	75915192	2.50E-48	0.561	0.507	0.055	*SNUPN*	1.44E-07	0.027
cg21243597	18	13375540	2.51E-48	0.650	0.577	0.073	*C18orf1*	3.21E-08	0.039
cg03882382	19	4540065	2.65E-48	0.205	0.260	-0.055	*LRG1*	0.005	-0.017
cg06745740	7	81399587	2.79E-48	0.188	0.244	-0.056	*HGF*	2.07E-07	-0.031
cg23983887	1	12509707	2.80E-48	0.592	0.526	0.066	*VPS13D*	1.47E-09	0.038
**cg15628518**	8	145025059	2.93E-48	0.315	0.391	-0.076	*PLEC*	0.417	-0.005
cg11210138	17	46233945	2.93E-48	0.447	0.511	-0.064	*SKAP1*	9.73E-10	-0.035
cg14709253	8	17519419	3.00E-48	0.174	0.227	-0.053	*MTUS1*	0.002	-0.017
cg21815704	1	193075249	3.02E-48	0.388	0.455	-0.067	*GLRX2*	0.022	-0.014
cg04890607	12	66262214	3.20E-48	0.466	0.530	-0.064	*HMGA2*	0.001	-0.020
cg23616212	1	109941201	3.28E-48	0.313	0.372	-0.059	*SORT1*	1.31E-07	-0.033
cg14172094	9	100394654	3.43E-48	0.213	0.267	-0.054	*NCBP1*	2.28E-08	-0.033
cg17738213	2	60776302	3.50E-48	0.183	0.242	-0.059	*BCL11A*	1.48E-05	-0.028
cg02987482	19	6274208	3.51E-48	0.286	0.346	-0.060	*MLLT1*	0.009	-0.017
cg11654325	2	242810927	3.71E-48	0.681	0.623	0.058	*CXXC11*	4.12E-07	0.029
cg24333621	7	133994445	3.77E-48	0.563	0.629	-0.066	*SLC35B4*	0.002	-0.020
cg14426785	9	101876671	3.77E-48	0.353	0.409	-0.057	*TGFBR1*	1.69E-08	-0.033
cg17329648	2	73297389	3.86E-48	0.299	0.359	-0.060	*SFXN5*	7.11E-07	-0.031
cg01231543	16	68741748	4.16E-48	0.662	0.715	-0.053	NA	0.003	-0.016
cg12547959	5	14326153	4.16E-48	0.420	0.493	-0.073	*TRIO*	8.93E-06	-0.031
cg24983189	20	2855312	4.31E-48	0.730	0.675	0.055	*PTPRA*	1.03E-04	0.022
cg15672653	11	108371700	4.41E-48	0.636	0.579	0.057	NA	5.05E-05	0.023
**cg16015295**	17	78793479	4.42E-48	0.419	0.486	-0.067	*RPTOR*	0.199	-0.009
cg15683970	21	35747081	4.77E-48	0.254	0.310	-0.056	*FAM165B*	3.15E-09	-0.033
cg26951440	10	101291272	4.85E-48	0.254	0.311	-0.057	*NKX2-3*	0.014	-0.014
cg07210669	4	6695475	4.85E-48	0.265	0.333	-0.069	*S100P*	0.001	-0.024
cg00139681	7	106125168	4.93E-48	0.406	0.463	-0.057	NA	3.20E-07	-0.029
cg08462055	15	64944023	5.09E-48	0.494	0.424	0.070	*ZNF609*	9.82E-05	0.026
cg25743819	2	173245704	5.09E-48	0.252	0.315	-0.063	NA	7.55E-09	-0.039
**cg03064100**	2	46720801	5.32E-48	0.768	0.709	0.059	NA	0.054	0.012
**cg09273112**	2	86732413	5.60E-48	0.511	0.589	-0.078	*RNF103-CHMI*	0.714	-0.002
cg03905718	16	4289417	5.63E-48	0.434	0.499	-0.065	*SRL*	8.67E-07	-0.035
cg02116768	17	80545322	5.85E-48	0.385	0.464	-0.078	*FOXK2*	1.23E-05	-0.034
cg26192520	8	101960390	6.52E-48	0.701	0.626	0.075	*YWHAZ*	3.89E-13	0.055
cg15768138	2	219030752	7.19E-48	0.434	0.499	-0.065	*CXCR1*	9.36E-08	-0.037
cg12018969	2	43350413	7.25E-48	0.555	0.609	-0.054	NA	9.95E-06	-0.021
cg19839325	19	18761132	7.49E-48	0.395	0.452	-0.057	*KLHL26*	9.77E-07	-0.028
cg06879746	6	30883768	7.67E-48	0.598	0.664	-0.066	*VARS2*	3.35E-11	-0.042
cg19627093	8	1900892	7.79E-48	0.707	0.636	0.071	*ARHGEF10*	7.55E-05	0.028
cg11775828	2	169100915	7.88E-48	0.480	0.427	0.054	*STK39*	2.18E-13	0.040
cg03706086	8	71381216	8.07E-48	0.537	0.606	-0.069	NA	7.18E-04	-0.021
cg14285150	17	46659019	8.60E-48	0.341	0.397	-0.056	NA	5.24E-07	-0.025
cg21498062	6	21157865	8.80E-48	0.814	0.751	0.063	*CDKAL1*	3.21E-15	0.051
cg01733438	17	75276069	8.99E-48	0.451	0.512	-0.061	*SEPT9*	4.53E-04	-0.021
cg00041989	17	55828038	9.15E-48	0.338	0.400	-0.062	NA	5.01E-05	-0.024
cg17467898	7	2289054	9.68E-48	0.273	0.347	-0.074	*NUDT1*	0.018	-0.016
cg02707799	8	41814854	9.92E-48	0.559	0.491	0.068	*KAT6A*	3.11E-11	0.045
**cg01877352**	11	60775233	1.08E-47	0.295	0.348	-0.053	*CD6*	0.508	-0.004
cg11310087	19	42315447	1.09E-47	0.172	0.223	-0.051	*CEACAM3*	0.015	-0.013
cg02255484	6	41735215	1.11E-47	0.262	0.316	-0.053	NA	1.35E-06	-0.028
cg00512872	7	6268584	1.17E-47	0.263	0.320	-0.057	*CYTH3*	0.002	-0.020
cg07780074	2	145090814	1.20E-47	0.502	0.555	-0.053	*GTDC1*	2.77E-08	-0.030
cg21913301	12	20677257	1.25E-47	0.743	0.687	0.056	*PDE3A*	3.28E-09	0.034
cg00602811	2	145278564	1.30E-47	0.437	0.505	-0.067	*ZEB2*	4.13E-29	-0.073
cg12570429	17	56345839	1.32E-47	0.433	0.487	-0.054	*LPO*	0.002	-0.015
cg10397934	2	242868376	1.33E-47	0.859	0.809	0.051	NA	0.004	0.016
cg00500176	19	10396158	1.34E-47	0.494	0.550	-0.056	*ICAM1*	3.48E-09	-0.033

cg09271097	7	137620868	1.43E-47	0.292	0.371	-0.079	*CREB3L2*	1.25E-07	-0.044
cg13521018	10	134429846	1.46E-47	0.634	0.566	0.067	*INPP5A*	0.037	0.015
cg10776244	1	28916695	1.53E-47	0.301	0.355	-0.054	NA	2.21E-04	-0.020
cg24370698	1	64058614	1.54E-47	0.330	0.408	-0.078	*PGM1*	5.43E-06	-0.036
cg19103429	7	92460784	1.55E-47	0.227	0.284	-0.057	*CDK6*	2.58E-12	-0.040
cg23348081	12	14413690	1.61E-47	0.551	0.627	-0.076	NA	4.23E-05	-0.025
**cg16051954**	1	227139852	1.71E-47	0.430	0.489	-0.059	*ADCK3*	0.091	-0.010
cg12884009	2	219079038	1.82E-47	0.604	0.655	-0.052	NA	0.038	-0.011
cg10416593	22	50966123	1.82E-47	0.584	0.646	-0.062	*TYMP*	1.73E-12	-0.043
cg02210115	13	49990767	1.86E-47	0.580	0.643	-0.064	*CAB39L*	5.98E-06	-0.029
cg01765406	2	129231478	1.87E-47	0.456	0.511	-0.055	NA	1.75E-13	-0.041
cg21262032	1	154437693	1.89E-47	0.746	0.696	0.051	*IL6R*	6.35E-13	0.034
**cg16520357**	13	53625240	1.98E-47	0.494	0.554	-0.060	*OLFM4*	0.512	-0.003
**cg24257776**	3	47051546	2.06E-47	0.313	0.377	-0.064	NA	0.142	-0.009
cg23181573	17	3863976	2.07E-47	0.411	0.477	-0.066	*ATP2A3*	0.037	-0.014
cg17444090	1	232735450	2.19E-47	0.353	0.429	-0.076	NA	0.003	-0.023
cg25045893	15	83777051	2.29E-47	0.215	0.273	-0.058	*TM6SF1*	0.002	-0.018
**cg22772691**	5	1104195	2.36E-47	0.303	0.354	-0.051	*SLC12A7*	0.361	-0.004
cg26585899	16	71914820	2.45E-47	0.466	0.521	-0.055	*ZNF821*	0.002	-0.017
cg17010895	2	65731517	2.48E-47	0.562	0.625	-0.063	NA	0.007	-0.016
cg01017257	1	15059738	2.49E-47	0.574	0.653	-0.079	*KAZN*	7.71E-05	-0.029
cg06390643	3	185775470	2.65E-47	0.596	0.651	-0.055	*ETV5*	0.039	-0.012
**cg05648510**	17	943342	2.67E-47	0.422	0.486	-0.064	*ABR*	0.101	-0.010
cg15207619	13	49975643	2.69E-47	0.180	0.240	-0.060	*CAB39L*	4.07E-08	-0.034
cg08876103	1	39572323	2.76E-47	0.377	0.438	-0.062	*MACF1*	2.26E-19	-0.056
cg23786209	10	116285554	3.09E-47	0.536	0.484	0.052	*ABLIM1*	0.003	0.016
cg12487655	10	90552437	3.30E-47	0.433	0.490	-0.057	NA	0.001	-0.019
cg16294668	12	32675207	3.33E-47	0.274	0.332	-0.058	*FGD4*	8.07E-05	-0.023
**cg25397054**	7	2874568	3.34E-47	0.358	0.437	-0.079	*GNA12*	0.073	-0.013
cg17848797	1	1198269	3.34E-47	0.705	0.755	-0.051	*UBE2J2*	0.001	-0.016
cg23016776	10	99081496	3.58E-47	0.505	0.563	-0.057	*FRAT1*	7.23E-05	-0.023
cg18188717	5	154230223	3.69E-47	0.216	0.276	-0.060	*C5orf4*	1.30E-04	-0.025
**cg01422243**	10	22725309	3.79E-47	0.696	0.643	0.052	*LOC10049948*	0.187	0.007
cg00968167	1	19810215	3.96E-47	0.621	0.555	0.066	*CAPZB*	2.92E-06	0.030
cg07964754	16	84650202	3.97E-47	0.801	0.748	0.053	*COTL1*	2.24E-05	0.023
cg21137173	3	113953856	4.01E-47	0.825	0.767	0.058	*ZNF80*	3.78E-13	0.043
cg09310966	6	41254825	4.29E-47	0.605	0.659	-0.054	*TREM1*	2.68E-07	-0.029
cg06892907	8	103818062	4.33E-47	0.315	0.370	-0.056	NA	3.72E-05	-0.024
cg24279243	4	38676559	4.45E-47	0.186	0.250	-0.063	*KLF3*	2.81E-07	-0.036
cg16074643	6	116424276	4.47E-47	0.243	0.296	-0.053	*NT5DC1*	2.63E-09	-0.034
cg07047589	15	40642998	4.49E-47	0.234	0.287	-0.052	*PHGR1*	4.06E-07	-0.025
cg06942183	17	46622607	4.66E-47	0.310	0.362	-0.052	*HOXB2*	1.09E-10	-0.037
cg02404304	6	2752090	4.69E-47	0.289	0.346	-0.057	*MYLK4*	0.002	-0.020
cg20675439	6	82466362	4.71E-47	0.535	0.587	-0.053	NA	2.57E-05	-0.025
cg14479377	9	138742730	4.81E-47	0.712	0.640	0.072	*CAMSAP1*	2.84E-10	0.049
cg22706883	15	67814880	4.82E-47	0.391	0.454	-0.064	*C15orf61*	1.97E-11	-0.043
cg10536916	6	27841865	4.89E-47	0.177	0.231	-0.054	*HIST1H4L*	2.14E-04	-0.021
cg20364222	8	125259763	4.89E-47	0.257	0.309	-0.051	NA	7.84E-08	-0.029
cg07126783	17	78800767	4.92E-47	0.374	0.439	-0.065	*RPTOR*	0.006	-0.018
cg19809988	7	149569883	5.09E-47	0.337	0.391	-0.054	*ATP6V0E2*	9.83E-04	-0.017
cg13491139	9	139627019	5.20E-47	0.804	0.746	0.058	NA	0.006	0.018
cg25371036	11	94500749	5.29E-47	0.685	0.743	-0.058	*AMOTL1*	1.32E-18	-0.046
cg16384864	1	16747435	5.36E-47	0.606	0.546	0.060	*SPATA21*	0.010	0.015
cg04571130	3	128967393	5.40E-47	0.410	0.464	-0.054	*COPG1*	0.005	-0.016
cg03978682	5	53411773	5.99E-47	0.478	0.529	-0.051	*ARL15*	1.94E-10	-0.032
cg10789050	3	31713847	6.01E-47	0.307	0.371	-0.064	*OSBPL10*	2.91E-08	-0.037
**cg26955540**	15	90727570	6.36E-47	0.782	0.731	0.051	*SEMA4B*	0.051	0.012
cg00510689	5	156594470	6.76E-47	0.783	0.722	0.061	*FAM71B*	7.25E-15	0.049
**cg19254152**	7	1083473	6.88E-47	0.202	0.260	-0.058	*C7orf50*	0.218	-0.008
cg07402310	17	40424590	7.00E-47	0.586	0.533	0.053	*STAT5B*	6.19E-04	0.018
cg00476955	11	3648013	7.09E-47	0.253	0.307	-0.054	*TRPC2*	0.016	-0.014
cg18496212	3	69797108	7.11E-47	0.303	0.374	-0.071	*MITF*	0.002	-0.025
**cg04422741**	3	151102836	7.75E-47	0.352	0.414	-0.063	*P2RY12*	0.159	-0.009
cg12297231	1	31531229	7.79E-47	0.767	0.716	0.051	*PUM1*	8.17E-17	0.044
cg25230327	8	82042993	8.46E-47	0.273	0.332	-0.059	NA	2.06E-11	-0.042
cg21196487	1	153538964	8.63E-47	0.231	0.282	-0.051	*S100A2*	5.22E-07	-0.027
cg08035555	19	3558417	8.79E-47	0.198	0.250	-0.052	*MFSD12*	1.96E-05	-0.022
cg08467103	2	65593933	8.81E-47	0.426	0.503	-0.077	*SPRED2*	2.16E-04	-0.029
cg26465532	10	79446426	8.95E-47	0.793	0.740	0.053	NA	2.09E-07	0.030
cg19882784	7	134856562	9.13E-47	0.527	0.578	-0.050	*C7orf49*	1.16E-04	-0.020
cg00355804	12	117103567	9.24E-47	0.478	0.541	-0.063	NA	1.55E-08	-0.036
cg00430036	9	99145401	1.02E-46	0.348	0.405	-0.057	*SLC35D2*	0.008	-0.016
cg01544877	6	136609152	1.06E-46	0.790	0.734	0.055	*BCLAF1*	4.18E-06	0.029

cg19690214	6	154678326	1.06E-46	0.311	0.373	-0.062	*IPCEF1*	4.33E-09	-0.036
**cg07183799**	13	107214285	1.10E-46	0.742	0.691	0.051	*ARGLU1*	0.170	0.006
cg14707231	5	43001138	1.10E-46	0.311	0.380	-0.069	NA	4.98E-07	-0.035
cg05669497	13	113655468	1.14E-46	0.240	0.296	-0.056	*MCF2L*	0.037	-0.012
cg12032655	19	43856746	1.14E-46	0.470	0.546	-0.076	*CD177*	1.30E-06	-0.036
cg24733624	12	9517562	1.15E-46	0.666	0.604	0.062	NA	0.027	0.012
cg13541353	9	32426330	1.16E-46	0.455	0.508	-0.053	*ACO1*	3.33E-04	-0.019
cg00344308	3	30273887	1.17E-46	0.411	0.468	-0.057	NA	0.008	-0.016
cg04714478	7	33153274	1.18E-46	0.450	0.507	-0.056	NA	4.44E-05	-0.023
cg15825027	8	586336	1.26E-46	0.496	0.558	-0.062	NA	5.40E-04	-0.020
cg14998613	7	155554462	1.31E-46	0.237	0.288	-0.051	*RBM33*	0.007	-0.015
cg25975690	7	27199726	1.33E-46	0.334	0.397	-0.064	NA	8.27E-07	-0.034
cg02389317	12	76577488	1.38E-46	0.741	0.691	0.050	NA	4.56E-04	0.018
cg06471491	17	57917974	1.38E-46	0.612	0.546	0.067	NA	9.00E-10	0.039
**cg11707219**	8	21905756	1.43E-46	0.366	0.427	-0.061	*FGF17*	0.710	-0.002
cg23903252	7	149569998	1.43E-46	0.253	0.304	-0.051	*ATP6V0E2*	2.24E-06	-0.025
cg18154117	1	39649937	1.45E-46	0.731	0.663	0.068	*MACF1*	2.27E-14	0.053
cg22401679	1	95080047	1.46E-46	0.381	0.447	-0.066	NA	3.13E-07	-0.034
cg13441891	16	85648370	1.48E-46	0.286	0.346	-0.060	*KIAA0182*	0.001	-0.020
cg21889703	6	136607649	1.52E-46	0.703	0.641	0.062	*BCLAF1*	4.08E-08	0.034
cg16665765	3	37934293	1.53E-46	0.348	0.400	-0.051	*CTDSPL*	3.24E-14	-0.040
**cg26607031**	1	9602701	1.66E-46	0.406	0.471	-0.065	*SLC25A33*	0.075	-0.012
cg26251585	7	11456440	1.67E-46	0.740	0.682	0.057	*THSD7A*	7.57E-07	0.028
cg01508380	14	23305585	1.73E-46	0.365	0.427	-0.062	*MMP14*	5.32E-06	-0.028
cg15836722	2	113593785	1.80E-46	0.350	0.403	-0.053	*IL1B*	4.14E-07	-0.029
cg06264679	6	32121433	1.82E-46	0.374	0.439	-0.066	*PPT2*	1.39E-09	-0.042
cg26581503	22	42304580	1.94E-46	0.191	0.242	-0.052	NA	0.015	-0.013
cg12261451	13	22243030	1.95E-46	0.211	0.264	-0.053	NA	0.010	-0.013
cg10761558	1	54842205	1.96E-46	0.747	0.691	0.056	*SSBP3*	8.02E-08	0.031
cg08550353	6	134497627	2.00E-46	0.284	0.337	-0.054	*SGK1*	0.046	-0.010
cg00186909	12	93129288	2.00E-46	0.333	0.397	-0.065	*PLEKHG7*	3.69E-09	-0.040
**cg00988346**	1	27729801	2.11E-46	0.195	0.245	-0.050	NA	0.116	-0.009
**cg07306190**	6	34760872	2.18E-46	0.161	0.216	-0.055	*UHRF1BP1*	0.783	-0.002
cg02251243	2	74210857	2.33E-46	0.300	0.354	-0.054	NA	0.001	-0.018
cg08277306	5	598817	2.33E-46	0.671	0.609	0.061	NA	4.26E-09	0.035
cg04422903	11	64108550	2.39E-46	0.280	0.339	-0.059	*CCDC88B*	1.41E-04	-0.023
cg23709535	6	34993277	2.40E-46	0.628	0.682	-0.054	*ANKS1A*	4.89E-07	-0.031
cg11824827	16	31075547	2.41E-46	0.708	0.645	0.062	*ZNF668*	0.003	0.017
cg05251190	10	104196206	2.57E-46	0.481	0.536	-0.055	NA	4.73E-12	-0.039
cg08476055	6	45472799	2.62E-46	0.537	0.600	-0.064	*RUNX2*	2.52E-04	-0.022
**cg23221723**	8	22963367	2.65E-46	0.381	0.451	-0.070	*TNFRSF10C*	0.472	-0.005
cg19788317	4	174429300	2.67E-46	0.239	0.290	-0.051	NA	9.92E-04	-0.017
cg25024734	10	112152815	2.70E-46	0.202	0.260	-0.058	NA	4.40E-07	-0.033
cg23681017	4	681086	2.77E-46	0.507	0.559	-0.052	*MFSD7*	3.50E-05	-0.020
cg10599446	3	189073674	2.89E-46	0.757	0.694	0.063	NA	1.69E-14	0.050
cg11712187	1	10368286	2.93E-46	0.791	0.740	0.051	*KIF1B*	5.47E-05	0.021
cg04987734	14	103415873	3.05E-46	0.441	0.375	0.066	*CDC42BPB*	3.63E-08	0.032
cg16959747	12	7276714	3.11E-46	0.409	0.464	-0.055	*RBP5*	2.21E-06	-0.026
cg16173847	18	77565614	3.17E-46	0.442	0.370	0.071	NA	0.003	0.020
cg18069568	18	43683922	3.48E-46	0.362	0.427	-0.065	*HAUS1*	1.07E-04	-0.023
cg21547557	21	45565564	3.50E-46	0.747	0.690	0.057	*C21orf33*	1.78E-04	0.023
cg21230503	5	139085173	3.50E-46	0.325	0.377	-0.052	NA	1.21E-09	-0.033
**cg16548911**	16	50347766	3.64E-46	0.269	0.331	-0.062	*ADCY7*	0.229	-0.008
cg15477500	3	44957542	3.69E-46	0.471	0.522	-0.052	*ZDHHC3*	6.12E-06	-0.024
cg04972065	12	53591766	3.71E-46	0.361	0.415	-0.054	*ITGB7*	2.75E-05	-0.025
cg01900413	11	128419356	3.88E-46	0.234	0.285	-0.050	*ETS1*	0.019	-0.014
cg19506253	2	158301839	4.22E-46	0.777	0.720	0.057	*CYTIP*	4.73E-11	0.038
cg20062691	1	949392	4.25E-46	0.734	0.816	-0.082	*ISG15*	2.76E-15	-0.047
cg02534850	12	66228466	4.27E-46	0.788	0.729	0.060	*HMGA2*	1.61E-08	0.036
cg13500819	5	138725400	4.36E-46	0.742	0.685	0.057	*MZB1*	0.004	0.015
cg25897519	8	41427415	4.38E-46	0.514	0.575	-0.061	NA	4.83E-05	-0.026
cg03608974	2	173940268	4.58E-46	0.231	0.286	-0.055	*ZAK*	6.35E-06	-0.028
cg06351682	2	106974831	4.65E-46	0.802	0.744	0.058	NA	7.41E-08	0.034
**cg06600725**	12	48690915	4.71E-46	0.299	0.375	-0.075	NA	0.051	-0.014
cg10440877	2	208378475	4.74E-46	0.537	0.589	-0.051	NA	6.58E-05	-0.022
cg17146738	7	98739698	5.17E-46	0.672	0.611	0.061	*SMURF1*	5.80E-04	0.021
**cg06488150**	7	6476003	5.21E-46	0.216	0.271	-0.056	*DAGLB*	0.852	-0.002
cg05348871	11	36397127	5.57E-46	0.565	0.506	0.059	*PRR5L*	0.003	0.018
cg16904330	3	119299162	5.63E-46	0.179	0.231	-0.051	*ADPRH*	2.37E-05	-0.022
cg12706330	10	85898700	5.72E-46	0.796	0.728	0.068	*GHITM*	5.10E-09	0.041
cg20874031	2	69206176	6.06E-46	0.698	0.630	0.069	*GKN1*	1.21E-08	0.041
cg24702826	6	86171768	6.55E-46	0.305	0.364	-0.058	*NT5E*	1.27E-04	-0.025
cg10117369	1	203734256	7.11E-46	0.540	0.471	0.068	*LAX1*	1.75E-05	0.029

cg02265742	11	19127032	7.30E-46	0.587	0.647	-0.060	NA	5.92E-04	-0.022
cg03295554	11	128395450	7.48E-46	0.676	0.610	0.066	*ETS1*	6.23E-04	0.024
cg17526229	11	20177746	7.72E-46	0.255	0.306	-0.051	NA	0.001	-0.018
cg02758236	11	128562003	8.51E-46	0.207	0.260	-0.053	*FLI1*	8.31E-08	-0.031
**cg09287328**	10	134231487	8.59E-46	0.436	0.503	-0.067	NA	0.052	-0.013
cg07330114	3	11624023	8.68E-46	0.207	0.266	-0.059	*VGLL4*	4.79E-04	-0.021
cg20654462	15	93580092	8.83E-46	0.445	0.527	-0.081	NA	1.12E-05	-0.036
cg06836480	2	169928251	9.02E-46	0.504	0.568	-0.064	*DHRS9*	0.002	-0.019
cg02895724	18	48719384	9.60E-46	0.806	0.754	0.052	*MEX3C*	1.60E-07	0.029
cg08691775	8	134310658	9.80E-46	0.226	0.282	-0.055	*NDRG1*	1.28E-04	-0.022
cg18827396	4	184368864	1.10E-45	0.840	0.789	0.052	*CDKN2AIP*	1.96E-11	0.034
**cg27478863**	13	50783080	1.13E-45	0.181	0.233	-0.052	NA	0.066	-0.011
cg07142009	17	6656315	1.17E-45	0.223	0.303	-0.080	NA	2.47E-06	-0.045
cg11902728	19	35786580	1.18E-45	0.279	0.330	-0.052	*MAG*	2.92E-07	-0.025
cg21745599	19	15755338	1.20E-45	0.550	0.603	-0.052	*CYP4F3*	0.001	-0.017
cg02108620	3	42002230	1.20E-45	0.349	0.408	-0.059	*ULK4*	6.72E-08	-0.033
**cg15986587**	19	56130797	1.21E-45	0.422	0.485	-0.063	NA	0.736	-0.003
cg25373630	18	60984656	1.22E-45	0.793	0.735	0.058	*BCL2*	6.92E-13	0.042
cg09310092	19	35529201	1.28E-45	0.324	0.376	-0.052	*SCN1B*	2.48E-14	-0.040
cg17430370	2	171034661	1.42E-45	0.795	0.742	0.053	*MYO3B*	1.14E-05	0.026
cg06046490	11	320940	1.43E-45	0.172	0.242	-0.069	*IFITM3*	1.52E-04	-0.029
cg11247817	5	96294344	1.51E-45	0.287	0.345	-0.058	*LNPEP*	0.002	-0.019
cg03875330	19	11455923	1.58E-45	0.427	0.482	-0.055	*CCDC159*	7.47E-11	-0.040
cg14126601	2	37384708	1.65E-45	0.412	0.495	-0.082	*EIF2AK2*	5.59E-12	-0.060
cg11387340	6	166970727	1.68E-45	0.256	0.312	-0.056	*RPS6KA2*	1.01E-11	-0.037
cg03948781	1	205179583	1.69E-45	0.309	0.391	-0.083	*DSTYK*	0.002	-0.028
cg03724229	2	173036250	1.95E-45	0.532	0.584	-0.052	NA	0.006	-0.016
cg25754958	1	202130692	1.95E-45	0.250	0.301	-0.050	*PTPN7*	4.85E-05	-0.019
cg23491841	2	242811149	1.97E-45	0.667	0.612	0.055	*CXXC11*	2.57E-06	0.027
cg03156546	16	24759640	2.26E-45	0.355	0.423	-0.068	*TNRC6A*	0.025	-0.016
cg14544087	21	26945916	2.31E-45	0.823	0.768	0.055	*MIR155HG*	2.37E-11	0.040
cg11274172	11	75736215	2.45E-45	0.438	0.493	-0.055	*UVRAG*	2.44E-06	-0.026
cg08448711	10	92794806	2.48E-45	0.429	0.487	-0.058	NA	1.17E-08	-0.033
cg06677890	5	149887486	2.68E-45	0.216	0.268	-0.052	*NDST1*	2.00E-06	-0.027
cg04771100	8	38832335	2.90E-45	0.289	0.364	-0.075	*HTRA4*	0.018	-0.018
cg11918450	2	33359198	2.90E-45	0.470	0.526	-0.056	*LTBP1*	0.004	-0.016
cg25954134	2	178941694	2.96E-45	0.393	0.463	-0.070	*PDE11A*	7.65E-11	-0.046
cg14692284	5	118693725	2.98E-45	0.781	0.721	0.060	*TNFAIP8*	1.48E-09	0.037
cg03146219	11	71189514	3.04E-45	0.420	0.520	-0.100	*NADSYN1*	0.017	-0.021
cg03715846	9	126761082	3.09E-45	0.236	0.289	-0.053	NA	0.009	-0.013
cg13799081	10	112410313	3.11E-45	0.758	0.699	0.059	*RBM20*	0.001	0.021
cg06095752	2	102313242	3.14E-45	0.298	0.355	-0.057	*MAP4K4*	0.027	-0.014
cg27239280	16	30907679	3.19E-45	0.376	0.433	-0.056	*CTF1*	2.60E-09	-0.033
cg01574481	14	24641852	3.26E-45	0.270	0.336	-0.067	*REC8*	0.004	-0.020
cg08778216	2	101628572	3.46E-45	0.709	0.647	0.062	*RPL31*	1.02E-08	0.038
cg20661257	6	106957497	3.76E-45	0.379	0.452	-0.073	NA	0.002	-0.024
cg11752788	7	123438230	3.83E-45	0.337	0.395	-0.058	NA	0.031	-0.014
cg04174091	11	122935222	4.15E-45	0.644	0.583	0.061	NA	1.33E-10	0.039
cg25469714	10	16949821	4.16E-45	0.483	0.543	-0.060	*CUBN*	2.29E-08	-0.033
cg03128890	8	122558224	4.25E-45	0.338	0.411	-0.072	NA	1.09E-05	-0.033
cg09142144	5	35939847	4.45E-45	0.783	0.723	0.059	*CAPSL*	1.73E-10	0.040
cg18181923	14	99682269	4.60E-45	0.746	0.692	0.054	*BCL11B*	1.80E-04	0.021
cg02145701	16	88038949	4.63E-45	0.408	0.478	-0.070	*BANP*	0.007	-0.019
cg15251256	12	46101447	4.82E-45	0.575	0.632	-0.056	NA	1.95E-04	-0.021
cg05487134	17	40489569	4.89E-45	0.649	0.574	0.075	*STAT3*	7.85E-09	0.042
cg14854503	8	19540272	4.97E-45	0.304	0.368	-0.064	*CSGALNACT1*	0.007	-0.018
cg00942920	1	203734559	5.05E-45	0.590	0.522	0.068	*LAX1*	2.84E-05	0.028
cg21443659	2	217745721	5.74E-45	0.420	0.483	-0.062	NA	3.08E-16	-0.047
cg17903316	18	44181660	5.95E-45	0.244	0.295	-0.050	*LOXHD1*	5.69E-08	-0.031
cg03171795	3	70882921	6.05E-45	0.498	0.555	-0.057	NA	0.006	-0.014
cg26509012	12	131306241	6.49E-45	0.424	0.477	-0.053	*STX2*	4.60E-11	-0.035
cg25038082	17	37896074	6.58E-45	0.409	0.462	-0.053	*GRB7*	1.36E-06	-0.022
cg00615241	19	839689	6.74E-45	0.430	0.488	-0.059	*PRTN3*	0.001	-0.018
cg21800196	3	48673931	6.82E-45	0.534	0.585	-0.051	*SLC26A6*	8.96E-05	-0.021
cg14249520	11	68530177	7.11E-45	0.463	0.523	-0.061	*CPT1A*	6.13E-05	-0.022
cg20100745	8	134307728	7.30E-45	0.253	0.304	-0.051	*NDRG1*	0.031	-0.012
cg12792931	14	77499162	7.91E-45	0.542	0.489	0.052	NA	4.46E-05	0.021
cg06854264	1	200861254	8.09E-45	0.336	0.389	-0.052	*C1orf106*	1.73E-05	-0.020
**cg00929635**	20	44035918	9.41E-45	0.369	0.429	-0.059	*DBNDD2*	0.184	-0.007
cg03999941	5	957511	9.64E-45	0.761	0.703	0.058	NA	5.46E-06	0.026
cg23344523	6	160380572	9.92E-45	0.601	0.652	-0.051	NA	7.51E-07	-0.026
cg27183791	16	89381904	9.96E-45	0.662	0.583	0.078	*ANKRD11*	0.002	0.025
cg01379656	3	174970696	1.00E-44	0.759	0.709	0.051	*NAALADL2*	9.45E-12	0.039

cg00323915	7	150264987	1.02E-44	0.782	0.726	0.056	*GIMAP4*	6.33E-04	0.020
cg24714606	13	98019621	1.03E-44	0.483	0.537	-0.054	*MBNL2*	4.27E-05	-0.024
cg19371652	12	113415883	1.08E-44	0.264	0.333	-0.069	*OAS2*	2.90E-29	-0.068
**cg26960939**	17	38717206	1.10E-44	0.526	0.475	0.051	*CCR7*	0.079	0.008
cg15516558	1	161091980	1.10E-44	0.379	0.440	-0.061	*DEDD*	0.017	-0.016
cg00349404	10	106100036	1.10E-44	0.511	0.587	-0.076	NA	2.16E-12	-0.055
cg02688118	13	114918456	1.11E-44	0.513	0.573	-0.060	NA	0.003	-0.017
cg26562772	1	221890431	1.18E-44	0.438	0.493	-0.054	*DUSP10*	1.63E-05	-0.024
cg25249713	4	177045356	1.19E-44	0.807	0.754	0.053	*WDR17*	2.18E-10	0.036
cg23466339	3	138066434	1.20E-44	0.403	0.467	-0.063	*MRAS*	0.002	-0.021
**cg25541528**	7	1545819	1.22E-44	0.295	0.359	-0.064	NA	0.137	-0.009
cg26228558	8	71527496	1.26E-44	0.643	0.589	0.054	*LOC286190*	1.39E-09	0.033
**cg07971820**	17	4621234	1.33E-44	0.414	0.483	-0.070	*ARRB2*	0.240	-0.008
cg06150772	17	77300674	1.34E-44	0.673	0.606	0.067	*RBFOX3*	4.80E-05	0.030
cg01933228	1	100316637	1.35E-44	0.401	0.451	-0.051	*AGL*	3.03E-13	-0.039
**cg16696774**	19	42882838	1.38E-44	0.683	0.629	0.054	*MEGF8*	0.115	0.008
cg03284554	8	42994513	1.40E-44	0.356	0.408	-0.051	*HGSNAT*	5.47E-05	-0.022
cg19035788	2	242881634	1.42E-44	0.784	0.732	0.051	NA	8.16E-08	0.031
cg09604569	2	232161891	1.49E-44	0.379	0.433	-0.054	*ARMC9*	1.25E-04	-0.023
cg18349022	7	99222459	1.51E-44	0.603	0.542	0.061	*ZNF498*	3.02E-10	0.038
cg19149314	2	197065128	1.54E-44	0.814	0.753	0.062	*HECW2*	7.96E-10	0.040
cg14713146	2	171782647	1.62E-44	0.510	0.578	-0.068	NA	5.67E-06	-0.029
cg09644974	2	177358138	1.62E-44	0.740	0.677	0.062	NA	1.41E-12	0.048
cg15594471	11	94883474	1.79E-44	0.260	0.317	-0.057	NA	0.003	-0.017
cg15469709	1	116653300	1.94E-44	0.506	0.568	-0.062	*MAB21L3*	1.66E-04	-0.025
cg20952652	2	106757367	1.96E-44	0.452	0.508	-0.056	*UXS1*	8.05E-06	-0.027
cg23687466	11	504937	2.08E-44	0.398	0.472	-0.074	*RNH1*	5.30E-04	-0.024
cg17969271	5	171430188	2.09E-44	0.311	0.368	-0.056	*FBXW11*	1.25E-06	-0.029
cg16806041	3	59996265	2.36E-44	0.608	0.533	0.076	*FHIT*	5.44E-06	0.033
cg22443330	15	31196599	2.49E-44	0.263	0.322	-0.058	*FAN1*	1.93E-05	-0.028
cg13255398	11	16836827	2.50E-44	0.182	0.237	-0.054	*PLEKHA7*	1.88E-04	-0.022
**cg03846076**	5	172743837	2.57E-44	0.665	0.613	0.052	*STC2*	0.315	0.006
cg15785898	6	144608500	2.60E-44	0.301	0.368	-0.067	NA	1.73E-04	-0.028
cg00551647	21	27943730	2.73E-44	0.423	0.494	-0.071	*CYYR1*	0.009	-0.020
cg22916254	12	96256561	3.00E-44	0.680	0.615	0.065	*SNRPF*	2.32E-11	0.047
cg11693709	15	40542019	3.03E-44	0.424	0.499	-0.075	*PAK6*	2.66E-15	-0.064
**cg14386624**	9	91006749	3.10E-44	0.318	0.374	-0.056	*SPIN1*	0.109	-0.010
cg07790947	6	28474513	3.11E-44	0.665	0.603	0.062	*GPX6*	4.77E-14	0.052
cg07137751	14	71377000	3.33E-44	0.755	0.703	0.052	*PCNX*	9.10E-04	0.018
cg25830379	15	99264361	3.39E-44	0.782	0.720	0.062	*IGF1R*	2.65E-11	0.047
cg13770461	11	46016312	3.41E-44	0.472	0.525	-0.053	*PHF21A*	5.65E-05	-0.021
cg13162127	8	96000155	3.50E-44	0.345	0.400	-0.055	NA	1.07E-04	-0.022
cg08559712	20	16030674	3.54E-44	0.780	0.729	0.051	*MACROD2*	1.22E-04	0.020
cg11971423	17	46653711	3.60E-44	0.701	0.753	-0.052	*HOXB4*	1.08E-12	-0.036
cg16278496	14	98444476	3.74E-44	0.573	0.505	0.068	NA	6.70E-06	0.032
cg02211519	11	2407267	3.85E-44	0.677	0.612	0.065	*CD81*	7.66E-05	0.028
**cg02107842**	5	66255772	4.05E-44	0.612	0.666	-0.055	*MAST4*	0.206	-0.007
cg21024495	15	70393940	4.08E-44	0.235	0.292	-0.057	NA	2.06E-07	-0.031
**cg24679890**	19	17246356	4.12E-44	0.183	0.237	-0.054	*MYO9B*	0.343	-0.006
cg16062483	14	98444417	4.20E-44	0.685	0.625	0.060	*C14orf64*	6.86E-05	0.024
cg01271812	2	66671478	4.34E-44	0.276	0.340	-0.064	*MEIS1*	5.22E-06	-0.033
cg25783892	10	85898649	4.50E-44	0.847	0.796	0.051	*GHITM*	2.24E-10	0.035
cg27585074	19	1947960	4.58E-44	0.441	0.496	-0.055	*CSNK1G2*	0.011	-0.014
cg01247537	6	27842098	4.70E-44	0.199	0.262	-0.062	*HIST1H4L*	0.039	-0.017
cg00010672	2	55281781	4.77E-44	0.774	0.703	0.070	NA	6.63E-09	0.045
cg04131890	5	969939	4.80E-44	0.715	0.660	0.055	NA	1.32E-07	0.030
cg27374881	3	186504006	4.91E-44	0.740	0.680	0.060	*EIF4A2*	1.43E-10	0.041
cg27062514	11	10800841	4.99E-44	0.813	0.755	0.058	*CTR9*	1.27E-08	0.035
cg05370101	2	43188839	5.09E-44	0.218	0.274	-0.056	NA	0.032	-0.014
**cg21964800**	11	63272764	5.32E-44	0.458	0.523	-0.066	*LGALS12*	0.249	-0.008
cg19018267	10	13341332	5.40E-44	0.280	0.342	-0.062	*PHYH*	1.07E-06	-0.031
**cg26169783**	13	34371837	5.85E-44	0.615	0.552	0.063	NA	0.191	0.008
cg13799772	3	129800573	5.94E-44	0.348	0.402	-0.054	*ALG1L2*	5.03E-06	-0.026
cg22090404	13	51489972	6.13E-44	0.594	0.649	-0.056	*RNASEH2B*	0.006	-0.015
cg11268280	8	142316072	6.32E-44	0.344	0.399	-0.055	NA	2.23E-04	-0.017
cg16978268	18	60646671	6.57E-44	0.341	0.405	-0.064	*PHLPP1*	2.28E-05	-0.030
cg01233664	2	191612466	6.91E-44	0.793	0.742	0.051	NA	6.18E-07	0.028
cg01987702	2	74264707	7.25E-44	0.213	0.264	-0.051	NA	1.56E-09	-0.033
**cg15305633**	20	45319350	8.34E-44	0.416	0.488	-0.072	*TP53RK*	0.258	-0.008
cg22986178	11	66034784	8.47E-44	0.205	0.262	-0.057	*KLC2*	0.007	-0.017
cg20547015	12	111165713	8.60E-44	0.802	0.748	0.054	*PPP1CC*	1.48E-06	0.028
cg04202620	8	1872355	8.61E-44	0.478	0.545	-0.067	*ARHGEF10*	5.88E-04	-0.024
cg13077031	6	15266782	9.02E-44	0.428	0.479	-0.050	*JARID2*	4.71E-05	-0.023

cg03307177	3	196347230	9.68E-44	0.274	0.356	-0.082	NA	1.82E-04	-0.035
cg23532927	7	45977067	9.87E-44	0.341	0.398	-0.057	NA	9.32E-09	-0.036
cg05278074	7	92442568	9.88E-44	0.734	0.668	0.065	*CDK6*	1.67E-14	0.053
cg04347414	1	2084519	9.95E-44	0.663	0.583	0.080	*PRKCZ*	5.38E-04	0.030
cg24743301	2	112945506	1.01E-43	0.768	0.715	0.053	*FBLN7*	2.67E-06	0.026
cg23112188	14	24563095	1.11E-43	0.206	0.269	-0.063	*PCK2*	4.46E-04	-0.024
cg02127888	16	1665900	1.12E-43	0.362	0.420	-0.059	*CRAMP1L*	6.92E-05	-0.025
**cg17723958**	12	124429295	1.14E-43	0.438	0.502	-0.065	*CCDC92*	0.240	-0.008
cg03565274	3	50629708	1.22E-43	0.422	0.477	-0.055	NA	1.73E-05	-0.026
cg13591783	9	75768868	1.29E-43	0.194	0.247	-0.053	*ANXA1*	1.85E-10	-0.038
**cg11658419**	12	58290850	1.31E-43	0.275	0.332	-0.057	NA	0.224	-0.008
cg11821200	5	40685989	1.32E-43	0.605	0.555	0.051	*PTGER4*	8.16E-06	0.024
cg05246522	17	25798973	1.38E-43	0.779	0.727	0.051	*KSR1*	0.013	0.012
cg14620234	9	108003927	1.44E-43	0.707	0.655	0.052	NA	7.10E-09	0.032
cg09895920	1	153941186	1.46E-43	0.302	0.368	-0.066	*CREB3L4*	8.69E-06	-0.031
**cg26050864**	1	209849445	1.48E-43	0.314	0.371	-0.057	*G0S2*	0.075	-0.011
**cg26118045**	4	174427715	1.49E-43	0.262	0.321	-0.059	NA	0.377	-0.006
cg23218957	14	59109856	1.54E-43	0.785	0.735	0.050	*DACT1*	3.62E-07	0.029
cg03509329	14	24520788	1.55E-43	0.212	0.265	-0.053	*LRRC16B*	4.23E-04	-0.020
cg03604774	1	27849102	1.56E-43	0.709	0.647	0.062	NA	2.03E-11	0.044
cg25507226	7	157190734	1.58E-43	0.399	0.463	-0.064	*DNAJB6*	2.12E-11	-0.046
cg18817487	12	96390143	1.65E-43	0.177	0.229	-0.052	*HAL*	4.04E-08	-0.032
cg24776142	6	16513829	1.73E-43	0.385	0.442	-0.057	*ATXN1*	8.54E-05	-0.024
cg25800166	12	113375896	1.92E-43	0.541	0.605	-0.063	*OAS3*	4.79E-09	-0.035
cg12082609	2	66671727	1.94E-43	0.288	0.351	-0.063	*MEIS1*	2.90E-07	-0.034
cg09039672	12	52367989	2.07E-43	0.469	0.521	-0.052	*ACVR1B*	5.22E-05	-0.021
cg16875057	2	169006003	2.08E-43	0.649	0.710	-0.061	*STK39*	9.62E-08	-0.031
cg01223011	3	128714144	2.15E-43	0.780	0.729	0.051	*KIAA1257*	3.30E-06	0.024
cg27137887	11	46513635	2.21E-43	0.459	0.511	-0.052	*AMBRA1*	1.15E-08	-0.032
cg16312514	11	70650521	2.22E-43	0.172	0.226	-0.054	*SHANK2*	4.33E-07	-0.028
cg08358907	19	17877508	2.30E-43	0.361	0.413	-0.051	*FCHO1*	3.48E-06	-0.022
cg16553297	4	123632037	2.38E-43	0.766	0.716	0.051	NA	0.003	0.017
cg02247863	22	50983415	2.38E-43	0.712	0.763	-0.050	NA	1.37E-10	-0.028
cg25511807	11	102401438	2.44E-43	0.290	0.350	-0.061	*MMP7*	3.88E-05	-0.026
cg11204139	17	3907470	2.74E-43	0.487	0.545	-0.058	NA	2.81E-05	-0.025
**cg20728490**	10	98064175	2.80E-43	0.545	0.466	0.079	*DNTT*	0.103	0.012
cg17696006	2	26521372	2.91E-43	0.440	0.499	-0.059	NA	1.97E-06	-0.028
cg14835981	7	752715	3.04E-43	0.399	0.458	-0.059	*PRKAR1B*	1.11E-04	-0.023
cg15937641	1	117529619	3.11E-43	0.725	0.674	0.051	*PTGFRN*	2.73E-08	0.031
cg20563269	2	129104576	3.23E-43	0.554	0.627	-0.073	NA	0.003	-0.021
cg01599709	5	139725677	3.33E-43	0.276	0.333	-0.056	*HBEGF*	0.014	-0.015
cg10816760	6	142890973	3.53E-43	0.543	0.481	0.062	*LOC153910*	4.91E-06	0.029
cg01079652	1	79118191	3.55E-43	0.707	0.824	-0.117	*IFI44*	1.01E-04	-0.024
cg21581567	7	28502140	3.62E-43	0.713	0.652	0.062	*CREB5*	8.63E-12	0.045
**cg00809164**	16	56651049	3.70E-43	0.199	0.257	-0.057	NA	0.255	-0.007
**cg07332601**	8	41072867	3.70E-43	0.580	0.646	-0.066	NA	0.089	-0.011
cg13941021	12	111181439	3.70E-43	0.272	0.332	-0.060	*PPP1CC*	1.50E-05	-0.029
cg14281591	13	73636077	3.95E-43	0.465	0.535	-0.070	*KLF5*	0.013	-0.017
cg10857250	12	122986080	3.96E-43	0.212	0.265	-0.053	*ZCCHC8*	3.79E-04	-0.021
cg18677148	17	57712280	4.13E-43	0.719	0.652	0.068	*CLTC*	2.41E-12	0.050
cg19931902	20	207144	4.29E-43	0.749	0.686	0.063	*DEFB129*	1.37E-13	0.051
**cg00754604**	2	161230046	4.80E-43	0.491	0.550	-0.060	*RBMS1*	0.280	-0.006
cg05437692	4	149362435	4.82E-43	0.313	0.365	-0.053	*NR3C2*	6.40E-15	-0.042
cg00005166	16	3264442	5.25E-43	0.632	0.688	-0.056	NA	6.47E-07	-0.028
cg14965639	2	48795994	5.43E-43	0.312	0.380	-0.068	*STON1-GTF2A*	1.03E-05	-0.031
cg00329411	5	55881858	5.58E-43	0.427	0.484	-0.057	NA	1.90E-06	-0.027
cg22898924	19	4903952	5.63E-43	0.407	0.478	-0.071	*ARRDC5*	5.13E-08	-0.040
cg17100158	2	180307728	5.64E-43	0.363	0.427	-0.064	*ZNF385B*	0.032	-0.014
cg14570838	11	128562353	5.94E-43	0.172	0.223	-0.051	*FLI1*	0.024	-0.013
cg25325512	6	37142220	5.95E-43	0.337	0.409	-0.072	*PIM1*	6.21E-14	-0.058
cg13603599	11	67251939	6.01E-43	0.733	0.676	0.057	*AIP*	8.50E-04	0.020
cg17329164	6	32121259	6.87E-43	0.285	0.346	-0.061	*PPT2*	1.52E-06	-0.034
cg07163845	14	106071651	7.10E-43	0.391	0.447	-0.056	NA	1.37E-07	-0.030
**cg21233003**	9	140057464	7.13E-43	0.304	0.384	-0.080	*GRIN1*	0.378	-0.008
cg02203380	7	30173809	7.37E-43	0.209	0.263	-0.054	*C7orf41*	5.18E-12	-0.040
cg27409015	2	158114424	7.38E-43	0.276	0.329	-0.053	*GALNT5*	0.012	-0.013
cg15141868	7	37959966	7.56E-43	0.218	0.274	-0.056	*EPDR1*	2.16E-07	-0.034
cg23211791	13	41364359	7.60E-43	0.267	0.325	-0.058	*SLC25A15*	4.59E-07	-0.033
cg05575505	15	101262162	7.81E-43	0.478	0.543	-0.065	NA	6.87E-13	-0.051
cg11305121	2	121011590	8.30E-43	0.428	0.482	-0.055	*RALB*	3.48E-09	-0.034
cg00231966	17	80377299	8.41E-43	0.267	0.321	-0.053	*C17orf101*	5.83E-16	-0.046
cg14724298	4	54424764	8.59E-43	0.498	0.558	-0.060	*LNX1*	9.21E-04	-0.020
cg19678968	8	59185164	9.43E-43	0.421	0.479	-0.058	NA	0.001	-0.020

cg02346737	5	142490096	1.04E-42	0.544	0.601	-0.057	*ARHGAP26*	0.010	-0.015
cg10096929	1	156261403	1.05E-42	0.402	0.465	-0.063	*TMEM79*	5.03E-04	-0.022
cg18586277	5	76116535	1.17E-42	0.345	0.397	-0.053	*F2RL1*	9.51E-05	-0.023
cg07555731	14	21624639	1.21E-42	0.708	0.645	0.063	*OR5AU1*	1.21E-11	0.047
**cg02402436**	6	31540051	1.26E-42	0.579	0.518	0.061	*LTA*	0.823	-0.001
**cg26870438**	10	6186015	1.28E-42	0.585	0.645	-0.060	*PFKFB3*	0.585	-0.004
cg08722675	4	8412549	1.34E-42	0.397	0.452	-0.055	*ACOX3*	3.83E-05	-0.024
cg07598021	5	148728200	1.39E-42	0.681	0.630	0.051	*GRPEL2*	1.75E-07	0.030
cg24674703	11	60869960	1.43E-42	0.528	0.461	0.067	*CD5*	8.48E-07	0.031
cg11130630	12	109899089	1.50E-42	0.307	0.361	-0.054	*KCTD10*	0.001	-0.020
cg23728359	7	6290148	1.52E-42	0.824	0.765	0.059	*CYTH3*	1.25E-09	0.039
cg18808777	6	31431503	1.59E-42	0.203	0.292	-0.089	*HCP5*	7.89E-10	-0.063
cg26706520	17	15655637	1.64E-42	0.759	0.702	0.057	NA	1.68E-07	0.032
cg03974193	2	102316442	1.66E-42	0.796	0.746	0.051	*MAP4K4*	2.12E-11	0.038
cg00874877	6	32272050	1.67E-42	0.811	0.753	0.058	*C6orf10*	1.44E-13	0.048
cg19766578	2	201677983	1.68E-42	0.611	0.552	0.059	*BZW1*	1.82E-06	0.029
cg13380502	1	27718221	1.89E-42	0.254	0.304	-0.051	*GPR3*	3.32E-04	-0.019
cg26227005	1	50574517	1.96E-42	0.228	0.280	-0.053	*ELAVL4*	2.21E-04	-0.022
cg26525313	6	139466669	2.28E-42	0.819	0.766	0.053	*HECA*	2.06E-07	0.027
cg14054620	9	38622748	2.45E-42	0.304	0.372	-0.068	*FAM201A*	0.003	-0.023
cg07185587	14	32981885	2.49E-42	0.787	0.734	0.053	*AKAP6*	8.24E-08	0.030
cg09409484	1	45770648	2.56E-42	0.558	0.610	-0.053	*LOC400752*	5.61E-10	-0.034
cg13897348	1	1549699	2.58E-42	0.195	0.255	-0.060	*MIB2*	9.85E-04	-0.023
cg23047137	3	24089778	2.81E-42	0.734	0.665	0.069	NA	6.10E-10	0.048
cg08576185	16	2055684	2.91E-42	0.397	0.456	-0.058	*ZNF598*	0.001	-0.020
cg09449988	7	151328116	2.97E-42	0.545	0.608	-0.063	*PRKAG2*	2.06E-06	-0.032
cg11262262	17	35305366	2.98E-42	0.217	0.271	-0.054	*AATF*	9.79E-04	-0.020
cg10523000	1	118198739	3.03E-42	0.728	0.671	0.057	NA	2.44E-04	0.020
cg22356061	1	227954102	3.08E-42	0.413	0.358	0.055	*SNAP47*	0.006	0.014
cg10834082	15	80500594	3.12E-42	0.795	0.741	0.054	NA	5.97E-05	0.024
cg26312191	17	80279310	3.15E-42	0.478	0.421	0.057	*SECTM1*	0.001	0.018
cg04049253	11	113958706	3.26E-42	0.470	0.530	-0.060	*ZBTB16*	1.19E-04	-0.023
**cg01054652**	11	72246863	3.34E-42	0.440	0.500	-0.060	NA	0.096	-0.010
**cg06080874**	18	46501625	3.69E-42	0.287	0.341	-0.054	NA	0.067	-0.011
cg02065051	20	25674725	3.82E-42	0.390	0.448	-0.058	*ZNF337*	6.90E-07	-0.030
**cg17694130**	4	14858290	4.02E-42	0.714	0.659	0.055	NA	0.968	-0.001
cg04410989	20	35578437	4.42E-42	0.213	0.275	-0.062	*SAMHD1*	1.35E-08	-0.035
cg16727006	16	87470545	4.52E-42	0.786	0.735	0.052	*ZCCHC14*	3.65E-05	0.024
cg08129017	17	17728660	4.62E-42	0.336	0.389	-0.052	*SREBF1*	0.016	-0.013
**cg27511678**	18	48721148	4.74E-42	0.600	0.545	0.055	*MEX3C*	0.057	0.010
cg16174681	5	108220453	4.75E-42	0.309	0.363	-0.054	*FER*	1.36E-12	-0.042
cg14977069	20	62367698	4.78E-42	0.717	0.658	0.059	*LIME1*	7.45E-04	0.020
cg21901395	17	46604235	4.93E-42	0.483	0.538	-0.056	NA	2.85E-16	-0.051
cg18422371	11	36593664	5.15E-42	0.742	0.680	0.062	*RAG1*	1.01E-08	0.039
**cg15408080**	12	90057674	5.23E-42	0.417	0.360	0.057	NA	0.128	0.008
cg05350315	1	32716961	5.30E-42	0.604	0.550	0.054	*LCK*	2.90E-05	0.024
cg08183303	16	447934	5.34E-42	0.350	0.401	-0.051	*NME4*	1.56E-08	-0.029
cg12449916	2	207847811	5.44E-42	0.436	0.490	-0.054	NA	1.26E-04	-0.022
cg18978531	15	70403809	5.59E-42	0.375	0.425	-0.050	NA	8.45E-15	-0.044
cg23689722	1	3100956	5.80E-42	0.472	0.535	-0.063	*PRDM16*	1.48E-05	-0.030
cg03613942	5	86416413	6.06E-42	0.815	0.762	0.052	NA	7.75E-08	0.033
cg06889108	5	173317342	6.29E-42	0.510	0.562	-0.052	*CPEB4*	0.009	-0.014
cg09569850	2	64955034	6.46E-42	0.671	0.613	0.059	NA	2.66E-04	0.023
cg14750778	17	26899428	6.54E-42	0.625	0.571	0.054	*PIGS*	0.006	0.015
cg16936289	17	29148918	7.14E-42	0.470	0.413	0.057	*CRLF3*	1.45E-05	0.025
cg10968027	11	95405470	7.69E-42	0.365	0.420	-0.054	NA	0.031	-0.013
cg01550445	11	72929983	7.97E-42	0.527	0.580	-0.054	*P2RY2*	2.30E-07	-0.031
cg06697600	4	7070879	8.23E-42	0.261	0.314	-0.053	*GRPEL1*	0.013	-0.014
cg12311175	4	118975504	8.56E-42	0.815	0.763	0.052	*NDST3*	2.21E-06	0.027
cg02059214	3	108021106	8.72E-42	0.817	0.764	0.052	*HHLA2*	2.87E-09	0.036
cg19152255	20	52274268	9.04E-42	0.452	0.532	-0.080	NA	5.39E-04	-0.029
cg21466315	3	133294854	9.35E-42	0.713	0.661	0.052	*CDV3*	6.31E-05	0.023
**cg21672292**	8	145025128	9.43E-42	0.208	0.266	-0.058	*PLEC*	0.560	-0.004
cg10279922	2	45347089	9.85E-42	0.770	0.715	0.055	NA	1.36E-12	0.043
cg19533955	3	99790878	9.90E-42	0.592	0.653	-0.061	*FILIP1L*	2.55E-04	-0.024
cg10951380	1	158029978	9.95E-42	0.429	0.488	-0.059	*KIRREL*	2.05E-09	-0.035
cg23214895	3	150456069	1.07E-41	0.753	0.693	0.060	NA	1.17E-14	0.049
**cg08965143**	2	24308246	1.08E-41	0.347	0.419	-0.073	*TP53I3*	0.147	-0.012
**cg18459618**	17	29297478	1.09E-41	0.201	0.256	-0.054	*RNF135*	0.340	-0.006
cg10818566	2	47197798	1.13E-41	0.530	0.583	-0.053	*TTC7A*	6.60E-07	-0.029
**cg00044505**	8	48649086	1.14E-41	0.344	0.409	-0.065	NA	0.103	-0.012
cg02143404	16	85607651	1.18E-41	0.406	0.458	-0.052	NA	1.93E-08	-0.032
cg07465344	17	4849412	1.28E-41	0.717	0.663	0.055	*PFN1*	7.41E-05	0.026

cg13315706	21	46341054	1.28E-41	0.445	0.388	0.056	*ITGB2-AS1*	1.82E-05	0.025
**cg12756150**	2	241140248	1.29E-41	0.780	0.729	0.051	NA	0.252	0.006
cg11526020	17	80870163	1.29E-41	0.409	0.462	-0.053	*TBCD*	0.007	-0.017
cg18560936	7	1632620	1.34E-41	0.730	0.680	0.050	NA	0.001	0.018
**cg12492653**	11	11862867	1.40E-41	0.386	0.456	-0.069	*USP47*	0.484	-0.006
cg13408605	20	1639816	1.47E-41	0.796	0.743	0.052	*SIRPG*	6.68E-11	0.036
cg24964103	6	133035379	1.68E-41	0.450	0.518	-0.068	*VNN1*	1.76E-06	-0.034
cg00421693	5	118689293	1.69E-41	0.783	0.731	0.052	*TNFAIP8*	3.67E-08	0.032
cg21489565	5	39723183	1.80E-41	0.836	0.783	0.054	NA	9.89E-07	0.029
cg19455189	2	231742734	1.83E-41	0.175	0.226	-0.052	*ITM2C*	4.07E-04	-0.019
cg10636246	1	159046973	1.87E-41	0.303	0.368	-0.064	*AIM2*	7.58E-20	-0.066
cg15028458	3	58860290	1.88E-41	0.695	0.629	0.066	*C3orf67*	1.19E-06	0.032
cg04926881	7	29024378	1.98E-41	0.266	0.319	-0.053	*LOC10050649*	1.07E-06	-0.030
cg06784691	1	2245988	1.99E-41	0.318	0.372	-0.055	NA	0.002	-0.019
**cg09317371**	16	57180175	2.06E-41	0.518	0.571	-0.053	*CPNE2*	0.086	-0.010
cg01521220	17	46233799	2.08E-41	0.314	0.383	-0.069	*SKAP1*	9.10E-07	-0.032
cg26541218	7	47826387	2.11E-41	0.380	0.445	-0.066	*PKD1L1*	1.02E-08	-0.043
cg01538969	6	30624636	2.20E-41	0.476	0.531	-0.055	*DHX16*	0.004	-0.017
**cg16272981**	5	1489889	2.27E-41	0.803	0.740	0.063	*LPCAT1*	0.911	0.000
cg00394261	5	42483732	2.30E-41	0.781	0.727	0.054	*GHR*	8.61E-05	0.024
**cg03622263**	9	126101308	2.32E-41	0.744	0.692	0.052	NA	0.405	0.004
**cg16275295**	15	101661984	2.41E-41	0.235	0.301	-0.066	NA	0.080	-0.012
cg25792439	17	78163268	2.43E-41	0.538	0.596	-0.058	*CARD14*	0.005	-0.017
cg03466998	1	247580074	2.54E-41	0.343	0.402	-0.060	*NLRP3*	1.21E-04	-0.024
cg03764364	10	29480551	2.83E-41	0.223	0.283	-0.060	NA	2.64E-05	-0.028
cg15564619	1	2163437	3.08E-41	0.609	0.550	0.059	*SKI*	4.75E-06	0.029
cg06547017	7	105514694	3.12E-41	0.827	0.768	0.059	*ATXN7L1*	7.33E-10	0.042
cg19653417	12	132654924	3.38E-41	0.729	0.784	-0.054	NA	1.07E-07	-0.028
**cg13064679**	6	32049953	3.45E-41	0.710	0.652	0.057	*TNXB*	0.079	0.011
**cg03400131**	6	134497247	3.46E-41	0.242	0.296	-0.054	*SGK1*	0.060	-0.012
cg09365147	6	111738477	3.48E-41	0.316	0.371	-0.055	*REV3L*	8.15E-06	-0.026
cg10389644	7	1575621	3.53E-41	0.368	0.424	-0.056	*MAFK*	0.007	-0.015
cg14864167	8	66751182	3.59E-41	0.513	0.651	-0.138	*PDE7A*	1.31E-18	-0.082
cg27057509	6	30883762	3.60E-41	0.518	0.585	-0.067	*VARS2*	1.24E-10	-0.045
cg08469215	1	156261351	3.67E-41	0.521	0.572	-0.052	*TMEM79*	4.26E-06	-0.023
cg05256304	11	116969870	3.72E-41	0.509	0.570	-0.061	*SIK3*	1.80E-13	-0.050
cg16745596	19	39695801	3.73E-41	0.353	0.416	-0.063	*SYCN*	1.40E-13	-0.056
cg17516825	1	111213827	3.85E-41	0.763	0.707	0.057	NA	0.001	0.022
cg21723245	6	29973407	3.99E-41	0.623	0.680	-0.056	*ZNRD1-AS1*	1.83E-04	-0.021
cg03812676	11	67254381	4.42E-41	0.720	0.655	0.065	*AIP*	8.20E-07	0.037
**cg15609237**	2	179395885	4.60E-41	0.466	0.522	-0.056	*TTN-AS1*	0.550	-0.004
cg19696794	6	7106952	4.63E-41	0.229	0.285	-0.056	*RREB1*	0.001	-0.020
cg06135285	17	19675785	4.67E-41	0.702	0.635	0.067	*ULK2*	4.92E-12	0.049
cg13755866	9	74920620	4.86E-41	0.390	0.443	-0.053	NA	0.016	-0.014
cg18931633	8	27470575	4.87E-41	0.442	0.500	-0.058	*CLU*	1.23E-12	-0.045
cg05878073	14	74766220	5.16E-41	0.395	0.452	-0.057	*ABCD4*	4.41E-05	-0.027
cg18362003	11	122051630	5.19E-41	0.820	0.768	0.053	*MIR100HG*	1.56E-07	0.032
**cg14363249**	9	75213807	5.38E-41	0.384	0.449	-0.065	*TMC1*	0.347	-0.006
cg04018325	16	14397685	5.72E-41	0.343	0.397	-0.054	NA	2.46E-06	-0.024
cg27584135	3	15975501	6.22E-41	0.314	0.367	-0.052	NA	1.04E-10	-0.037
**cg08998950**	3	4856199	6.29E-41	0.544	0.625	-0.082	*ITPR1*	0.070	-0.015
cg11336311	1	23887324	6.29E-41	0.475	0.544	-0.069	*ID3*	0.002	-0.023
cg14339466	13	27927490	6.46E-41	0.792	0.735	0.057	NA	1.27E-08	0.036
cg15134506	10	75336540	6.78E-41	0.777	0.725	0.052	*USP54*	5.41E-06	0.027
cg12977548	4	24579968	6.86E-41	0.679	0.612	0.067	*DHX15*	1.10E-06	0.035
**cg04397884**	14	56666067	7.03E-41	0.394	0.453	-0.058	*PELI2*	0.248	-0.006
cg16523868	3	140974967	7.07E-41	0.614	0.562	0.052	*ACPL2*	9.63E-05	0.021
**cg01814191**	12	89744524	7.15E-41	0.192	0.246	-0.053	*DUSP6*	0.237	-0.008
cg24502330	1	20914028	7.28E-41	0.305	0.377	-0.072	*CDA*	3.82E-07	-0.039
cg26588061	10	12086036	7.93E-41	0.392	0.459	-0.067	*UPF2*	3.34E-05	-0.029
**cg02088785**	13	99223336	7.97E-41	0.762	0.708	0.054	*STK24*	0.344	0.004
cg20743720	6	143257108	8.15E-41	0.777	0.716	0.061	*HIVEP2*	3.94E-13	0.046
cg01535726	8	9911482	8.59E-41	0.346	0.405	-0.059	*MSRA*	0.016	-0.016
cg22292345	3	47051533	8.65E-41	0.366	0.423	-0.057	NA	0.011	-0.015
cg19998533	7	41486779	8.80E-41	0.806	0.754	0.051	NA	1.37E-08	0.033
**cg02320474**	16	1758022	8.87E-41	0.311	0.369	-0.057	*MAPK8IP3*	0.196	-0.009
cg25706447	1	27118485	9.03E-41	0.491	0.550	-0.059	*PIGV*	1.47E-04	-0.022
cg05206789	4	87894298	9.15E-41	0.311	0.365	-0.054	*AFF1*	1.25E-04	-0.023
cg07048504	1	55829278	9.56E-41	0.694	0.642	0.052	NA	7.13E-05	0.022
cg05385434	1	225998870	9.81E-41	0.393	0.457	-0.064	*EPHX1*	2.64E-04	-0.026
**cg22241838**	11	60680006	9.84E-41	0.626	0.566	0.061	*TMEM109*	0.547	0.003
cg16210979	5	112882744	9.99E-41	0.821	0.764	0.057	*YTHDC2*	7.68E-10	0.041
cg14870271	17	76976010	1.01E-40	0.334	0.405	-0.072	*LGALS3BP*	5.69E-17	-0.056

cg11975790	6	30853608	1.04E-40	0.706	0.638	0.067	*DDR1*	1.40E-10	0.049
cg15936066	8	42356637	1.08E-40	0.364	0.416	-0.052	*SLC20A2*	3.16E-06	-0.027
cg19066520	3	191046547	1.14E-40	0.713	0.652	0.060	*CCDC50*	0.002	0.020
cg07024786	7	29883787	1.17E-40	0.615	0.552	0.062	*WIPF3*	2.69E-05	0.029
cg15590153	1	64058523	1.18E-40	0.369	0.438	-0.069	*PGM1*	0.006	-0.021
cg07914200	6	22699463	1.32E-40	0.509	0.563	-0.055	NA	5.66E-06	-0.028
cg26466145	3	188665930	1.36E-40	0.382	0.443	-0.062	NA	5.80E-06	-0.031
**cg15428479**	19	18698825	1.39E-40	0.329	0.393	-0.064	*C19orf60*	0.084	-0.012
cg03602014	5	126564681	1.39E-40	0.410	0.462	-0.051	NA	0.020	-0.014
cg26242531	14	104190678	1.39E-40	0.378	0.428	-0.051	*ZFYVE21*	4.98E-12	-0.035
cg22365240	2	105374995	1.40E-40	0.551	0.488	0.063	NA	4.69E-09	0.040
**cg11800635**	2	74783088	1.58E-40	0.506	0.569	-0.063	*DOK1*	0.084	-0.012
cg04303509	19	35453690	1.61E-40	0.264	0.316	-0.053	*ZNF792*	2.21E-04	-0.022
cg26025224	15	89020059	1.73E-40	0.596	0.646	-0.050	*MRPS11*	7.08E-12	-0.036
cg21790851	13	73634643	1.74E-40	0.210	0.262	-0.052	*KLF5*	0.044	-0.013
cg20475005	7	123351286	1.74E-40	0.824	0.772	0.051	*WASL*	7.62E-08	0.031
**cg00335124**	5	58652808	1.77E-40	0.360	0.420	-0.060	*PDE4D*	0.183	0.008
cg21442881	8	142160081	1.77E-40	0.493	0.551	-0.058	*DENND3*	0.004	-0.018
cg03450844	7	150416671	1.79E-40	0.340	0.418	-0.078	*GIMAP1-GIMA*	0.047	-0.019
cg09137533	22	46469091	1.89E-40	0.370	0.427	-0.057	NA	3.03E-14	-0.049
cg16489809	10	97450247	1.91E-40	0.502	0.559	-0.057	*TCTN3*	0.010	-0.014
cg19701087	6	52859008	1.91E-40	0.568	0.637	-0.069	*GSTA4*	2.53E-11	-0.044
cg09854615	11	69259788	2.03E-40	0.536	0.594	-0.059	NA	0.009	-0.016
cg18033092	11	47024513	2.03E-40	0.579	0.629	-0.051	*C11orf49*	0.004	-0.017
cg08979352	4	99580530	2.07E-40	0.421	0.493	-0.072	*TSPAN5*	3.14E-08	-0.043
cg21793437	12	2734591	2.08E-40	0.319	0.373	-0.054	*CACNA1C*	1.62E-05	-0.026
cg04792910	15	66652179	2.11E-40	0.777	0.723	0.054	NA	7.40E-10	0.038
cg08866497	5	43781155	2.39E-40	0.730	0.671	0.059	NA	5.65E-09	0.037
cg15232539	8	19459672	2.50E-40	0.214	0.273	-0.059	*CSGALNACT1*	1.06E-05	-0.029
**cg02861504**	1	207144367	2.77E-40	0.555	0.607	-0.052	*FCAMR*	0.097	-0.009
cg02271603	7	33197291	2.78E-40	0.601	0.543	0.058	*BBS9*	2.79E-06	0.029
cg21892295	12	121157589	2.85E-40	0.284	0.346	-0.062	*UNC119B*	0.001	-0.022
**cg22446264**	6	13766814	3.30E-40	0.584	0.655	-0.071	NA	0.198	-0.009
cg02784232	3	169897975	3.38E-40	0.595	0.537	0.058	*PHC3*	5.81E-06	0.029
**cg19672546**	8	145025123	3.39E-40	0.204	0.255	-0.051	*PLEC*	0.808	-0.001
**cg21239001**	5	118638618	3.52E-40	0.556	0.609	-0.053	*TNFAIP8*	0.168	0.008
cg08726900	16	89550474	3.70E-40	0.350	0.290	0.060	*ANKRD11*	0.003	0.016
cg23166773	1	243659016	3.83E-40	0.479	0.536	-0.057	*SDCCAG8*	4.89E-08	-0.034
**cg22136363**	11	1911511	3.85E-40	0.202	0.269	-0.067	*LSP1*	0.942	0.001
cg17486314	6	90778227	4.10E-40	0.678	0.624	0.055	*BACH2*	3.26E-08	0.031
cg27380915	7	12796513	4.14E-40	0.512	0.580	-0.068	NA	0.034	-0.016
cg09395612	16	86018936	4.46E-40	0.439	0.494	-0.055	NA	5.16E-05	-0.026
cg00225902	3	66444122	4.56E-40	0.740	0.681	0.059	*LRIG1*	8.24E-07	0.032
**cg04813787**	6	167814397	4.81E-40	0.757	0.706	0.052	NA	0.612	0.003
cg00289503	20	61470028	4.90E-40	0.270	0.325	-0.055	*COL9A3*	0.003	-0.020
cg00033909	7	9674902	4.99E-40	0.693	0.641	0.052	*PER4*	1.03E-04	0.023
cg26700919	18	13375474	4.99E-40	0.778	0.727	0.052	*C18orf1*	6.84E-10	0.036
cg23723835	10	73869442	4.99E-40	0.796	0.742	0.054	*ASCC1*	1.70E-10	0.038
cg08466256	2	189957139	5.03E-40	0.766	0.706	0.059	*COL5A2*	5.76E-09	0.040
cg07243366	12	19729703	5.06E-40	0.521	0.572	-0.052	NA	0.040	-0.012
cg14167858	1	120199593	5.06E-40	0.784	0.731	0.053	NA	4.71E-05	0.025
cg18700420	5	55286352	5.47E-40	0.790	0.728	0.062	*IL6ST*	4.95E-15	0.056
**cg22094071**	8	87521456	5.57E-40	0.482	0.539	-0.057	*FAM82B*	0.085	-0.012
cg26663490	17	72443066	5.57E-40	0.234	0.285	-0.051	*GPRC5C*	0.003	-0.017
**cg15219811**	10	104259328	5.74E-40	0.503	0.562	-0.059	*ACTR1A*	0.221	-0.008
cg08462501	4	38138950	5.89E-40	0.551	0.603	-0.053	*TBC1D1*	7.11E-04	-0.021
cg15166900	3	69132709	6.02E-40	0.666	0.601	0.066	*ARL6IP5*	2.55E-07	0.036
cg13692739	9	139005232	6.24E-40	0.413	0.357	0.056	NA	2.74E-06	0.028
cg12582728	17	36162433	6.30E-40	0.780	0.725	0.056	NA	1.32E-06	0.032
cg08594554	17	72231318	6.43E-40	0.303	0.358	-0.055	*TTYH2*	0.009	-0.017
**cg13216331**	1	156025757	6.88E-40	0.329	0.387	-0.058	*LAMTOR2*	0.456	-0.006
cg15664462	3	151996146	6.92E-40	0.727	0.662	0.064	*MBNL1*	2.90E-12	0.052
**cg02516101**	22	38711891	7.04E-40	0.222	0.280	-0.058	*CSNK1E*	0.371	-0.006
cg16652347	7	132170652	7.19E-40	0.710	0.657	0.053	*PLXNA4*	3.97E-06	0.030
**cg02097429**	16	27210995	7.20E-40	0.549	0.495	0.054	NA	0.388	0.005
**cg27531490**	6	31542459	7.46E-40	0.735	0.685	0.050	*TNF*	0.171	0.007
cg14152591	10	14587920	8.41E-40	0.406	0.461	-0.055	*FAM107B*	1.16E-06	-0.028
**cg07689396**	7	633050	8.97E-40	0.194	0.247	-0.053	*PRKAR1B*	0.087	-0.010
cg10236224	16	30689953	9.23E-40	0.416	0.481	-0.065	NA	6.04E-04	-0.026
**cg26489413**	11	10476976	9.37E-40	0.283	0.334	-0.051	*AMPD3*	0.437	-0.005
cg07759162	15	75488313	9.42E-40	0.487	0.538	-0.051	NA	2.85E-05	-0.022
cg05166871	17	46233714	9.84E-40	0.244	0.301	-0.058	*SKAP1*	2.00E-04	-0.019
cg08879579	2	239478440	9.86E-40	0.361	0.412	-0.051	NA	0.039	-0.011

cg09988805	2	43278552	9.90E-40	0.701	0.641	0.060	NA	7.26E-05	0.026
cg10274453	13	43378116	1.09E-39	0.526	0.599	-0.073	NA	7.12E-07	-0.034
cg15013617	20	2795378	1.12E-39	0.373	0.428	-0.056	*C20orf141*	4.17E-08	-0.035
**cg24442740**	1	27902069	1.19E-39	0.187	0.254	-0.067	*AHDC1*	0.190	-0.011
cg12299554	15	94840953	1.25E-39	0.252	0.306	-0.054	*MCTP2*	0.016	-0.016
cg13269555	10	102822002	1.31E-39	0.455	0.515	-0.060	*KAZALD1*	3.23E-09	-0.038
**cg14306650**	9	129829146	1.39E-39	0.465	0.523	-0.058	*RALGPS1*	0.290	-0.006
cg23367341	5	171504064	1.39E-39	0.526	0.600	-0.074	*STK10*	1.05E-07	-0.041
cg13100962	11	33921158	1.42E-39	0.469	0.527	-0.058	NA	2.43E-06	-0.030
cg09375033	3	137833634	1.63E-39	0.339	0.398	-0.060	*DZIP1L*	0.001	-0.023
cg09022607	10	114712695	1.65E-39	0.333	0.383	-0.050	*TCF7L2*	0.006	-0.015
**cg11919271**	3	151102879	1.71E-39	0.493	0.560	-0.068	*P2RY12*	0.073	-0.013
cg04507925	2	210824770	1.74E-39	0.749	0.692	0.056	*UNC80*	2.41E-06	0.030
**cg19582538**	6	38127734	1.83E-39	0.497	0.566	-0.069	NA	0.388	-0.007
cg21097090	5	118693764	1.86E-39	0.725	0.668	0.057	*TNFAIP8*	3.47E-08	0.037
cg13468685	2	68592737	1.90E-39	0.317	0.374	-0.056	*PLEK*	2.23E-09	-0.040
**cg27527018**	7	149570040	1.93E-39	0.208	0.258	-0.050	*ATP6V0E2*	0.073	-0.011
cg19886655	6	2958852	1.96E-39	0.290	0.362	-0.071	*SERPINB6*	0.031	-0.018
cg11348257	1	76556226	1.96E-39	0.351	0.410	-0.059	*ST6GALNAC3*	3.47E-04	-0.024
**cg19767800**	5	92914085	2.03E-39	0.332	0.383	-0.051	*FLJ42709*	0.103	-0.009
cg00177237	21	43658316	2.03E-39	0.788	0.738	0.051	*ABCG1*	3.48E-09	0.035
cg17932911	10	101942982	2.08E-39	0.460	0.517	-0.057	*ERLIN1*	2.49E-05	-0.028
cg02033582	17	29394814	2.17E-39	0.325	0.381	-0.056	NA	6.02E-04	-0.022
cg27096572	18	73141041	2.18E-39	0.702	0.637	0.066	*C18orf62*	7.56E-11	0.048
cg08826743	8	105392295	2.36E-39	0.724	0.661	0.063	*DPYS*	3.75E-09	0.041
cg27589742	16	85296503	2.37E-39	0.497	0.580	-0.083	NA	0.004	-0.025
cg04874782	2	241900108	2.38E-39	0.199	0.255	-0.056	*LOC200772*	2.54E-04	-0.024
cg11068071	1	89592433	2.41E-39	0.638	0.573	0.065	*GBP2*	1.68E-10	0.047
cg22828110	5	75919242	2.60E-39	0.242	0.295	-0.053	*IQGAP2*	0.002	-0.019
cg13989999	20	30309717	3.11E-39	0.716	0.663	0.054	*BCL2L1*	0.005	0.013
**cg13842305**	1	151033007	3.51E-39	0.216	0.267	-0.051	*CDC42SE1*	0.052	-0.013
cg18282393	2	111788355	3.63E-39	0.829	0.772	0.056	*ACOXL*	5.81E-08	0.038
cg14293575	22	18635460	3.65E-39	0.744	0.838	-0.093	*USP18*	1.68E-15	-0.042
cg14091154	4	79358764	3.69E-39	0.723	0.665	0.058	*FRAS1*	5.96E-09	0.038
cg16144883	17	398066	3.70E-39	0.441	0.492	-0.050	NA	2.31E-04	-0.019
cg01965380	1	90321064	3.70E-39	0.743	0.693	0.051	*LRRC8D*	2.84E-08	0.032
cg04352495	14	50700827	3.77E-39	0.810	0.750	0.060	NA	3.56E-11	0.045
cg16887422	2	240035107	3.78E-39	0.471	0.535	-0.064	*HDAC4*	3.50E-05	-0.029
cg21699330	7	26193032	3.84E-39	0.160	0.215	-0.055	*NFE2L3*	2.34E-14	-0.053
cg08460732	2	10256302	3.87E-39	0.291	0.344	-0.052	NA	7.36E-04	-0.021
cg19947463	7	1113237	3.91E-39	0.396	0.464	-0.068	*C7orf50*	0.028	-0.018
cg06225476	7	116169488	3.99E-39	0.739	0.675	0.064	*CAV1*	1.44E-08	0.041
cg24850711	13	29067224	4.05E-39	0.707	0.650	0.057	*FLT1*	2.58E-05	0.025
cg03448362	1	172864347	4.07E-39	0.617	0.565	0.052	NA	0.002	0.017
cg25423174	3	12236303	4.14E-39	0.438	0.490	-0.053	NA	2.29E-07	-0.030
cg07712198	5	9015582	4.27E-39	0.424	0.479	-0.055	NA	0.007	-0.016
cg15070897	14	23284968	4.59E-39	0.483	0.538	-0.056	*SLC7A7*	0.007	-0.016
cg14380217	14	54604922	4.63E-39	0.770	0.714	0.056	NA	2.92E-12	0.044
cg06875488	4	7657070	4.65E-39	0.312	0.363	-0.051	*SORCS2*	1.61E-06	-0.026
cg21912448	1	8727007	5.04E-39	0.315	0.366	-0.051	*RERE*	2.40E-05	-0.025
cg10114555	1	113216774	5.08E-39	0.301	0.360	-0.059	*MOV10*	4.74E-04	-0.024
**cg20944315**	1	200839460	5.52E-39	0.727	0.673	0.054	NA	0.054	0.011
cg26922854	6	134839143	5.98E-39	0.534	0.596	-0.062	NA	0.002	-0.022
cg13424302	10	80516893	6.11E-39	0.328	0.382	-0.054	NA	5.68E-04	-0.021
cg23202253	12	26902211	6.20E-39	0.811	0.760	0.051	*ITPR2*	5.95E-07	0.030
cg11250194	11	61601937	6.35E-39	0.481	0.532	-0.051	*FADS2*	1.64E-09	-0.038
cg10994564	3	119306022	6.36E-39	0.705	0.650	0.055	*ADPRH*	1.03E-05	0.028
cg23840027	1	29225533	6.95E-39	0.430	0.484	-0.054	*EPB41*	0.016	-0.016
**cg26314089**	12	66567930	7.03E-39	0.545	0.614	-0.068	NA	0.098	-0.014
**cg25771013**	7	25989735	7.38E-39	0.194	0.252	-0.059	NA	0.642	0.002
cg13400077	1	162168081	7.92E-39	0.754	0.696	0.059	*NOS1AP*	7.42E-08	0.037
**cg17494897**	2	3200012	8.46E-39	0.484	0.550	-0.066	*TSSC1*	0.148	-0.011
cg19400179	3	108321607	8.92E-39	0.375	0.316	0.059	*DZIP3*	5.72E-06	0.028
cg06903031	1	110644949	9.42E-39	0.384	0.436	-0.052	NA	5.65E-06	-0.026
**cg09989037**	1	44300942	1.02E-38	0.423	0.486	-0.062	*ST3GAL3*	0.064	-0.014
cg25930962	15	44321561	1.03E-38	0.755	0.695	0.060	*FRMD5*	9.68E-11	0.044
cg11749792	8	124730091	1.04E-38	0.562	0.618	-0.056	*ANXA13*	4.00E-04	-0.021
cg06933697	16	15238104	1.04E-38	0.525	0.581	-0.056	NA	3.89E-04	-0.021
cg07023317	8	28961315	1.04E-38	0.584	0.635	-0.051	*KIF13B*	1.58E-06	-0.027
cg08142096	11	111125837	1.05E-38	0.711	0.656	0.055	*C11orf53*	3.83E-07	0.032
cg03514660	12	131418057	1.07E-38	0.233	0.289	-0.056	NA	0.007	-0.018
cg16151924	8	65577290	1.17E-38	0.735	0.682	0.053	*CYP7B1*	1.70E-04	0.022
cg00300303	1	1067223	1.21E-38	0.511	0.565	-0.054	NA	7.40E-07	-0.030

cg24576960	4	38875533	1.22E-38	0.387	0.446	-0.059	*FAM114A1*	8.33E-04	-0.022
**cg22431093**	12	32654992	1.23E-38	0.441	0.496	-0.055	*FGD4*	0.116	0.010
cg18023455	7	49894664	1.23E-38	0.736	0.674	0.062	*VWC2*	7.38E-10	0.042
**cg02586198**	16	29820119	1.35E-38	0.737	0.680	0.057	*MAZ*	0.104	0.012
cg08063160	2	238578376	1.37E-38	0.731	0.673	0.058	*LRRFIP1*	2.15E-04	0.026
cg10118456	16	16065831	1.41E-38	0.703	0.754	-0.051	*ABCC1*	2.57E-04	-0.019
**cg02185248**	17	78748494	1.45E-38	0.193	0.256	-0.062	*RPTOR*	0.540	-0.005
cg01798157	1	203276595	1.48E-38	0.466	0.384	0.082	*BTG2*	4.75E-06	0.038
cg08698997	16	88989212	1.52E-38	0.255	0.305	-0.050	*CBFA2T3*	3.38E-12	-0.040
cg14173033	17	80545310	1.53E-38	0.342	0.414	-0.072	*FOXK2*	4.51E-06	-0.038
cg17265994	11	2905024	1.54E-38	0.298	0.349	-0.051	*CDKN1C*	6.05E-04	-0.019
cg05070250	11	36620199	1.56E-38	0.802	0.747	0.055	*RAG2*	4.31E-07	0.033
**cg14111928**	10	76602391	1.60E-38	0.441	0.494	-0.053	*KAT6B*	0.076	-0.010
cg23687971	20	32262772	1.64E-38	0.366	0.419	-0.053	*NECAB3*	7.68E-06	-0.028
cg12535715	8	38831693	1.66E-38	0.427	0.493	-0.066	*HTRA4*	0.033	-0.017
cg07986199	12	2743038	1.71E-38	0.455	0.396	0.059	*CACNA1C*	8.16E-05	0.025
**cg08287903**	4	115524813	1.72E-38	0.767	0.715	0.052	*UGT8*	0.751	0.002
cg15108641	10	99263320	1.81E-38	0.377	0.429	-0.052	*UBTD1*	1.59E-06	-0.029
cg24659858	13	24270321	1.81E-38	0.585	0.516	0.068	NA	1.31E-09	0.046
cg06156844	18	61599595	1.82E-38	0.566	0.619	-0.053	*SERPINB10*	1.83E-05	-0.024
**cg03899229**	8	8821258	1.86E-38	0.201	0.259	-0.058	NA	0.071	-0.012
cg04724477	8	124780013	1.86E-38	0.453	0.506	-0.053	*FAM91A1*	9.63E-11	-0.041
**cg08422803**	21	46341067	1.88E-38	0.491	0.440	0.052	*ITGB2-AS1*	0.212	0.008
cg04573500	17	55444427	1.90E-38	0.323	0.373	-0.051	*MSI2*	0.002	-0.021
**cg16672562**	19	46801672	1.97E-38	0.361	0.481	-0.120	*HIF3A*	0.064	-0.026
cg26847642	11	103399483	2.07E-38	0.696	0.644	0.052	NA	1.19E-05	0.026
**cg00472373**	3	9833414	2.21E-38	0.748	0.695	0.053	*ARPC4*	0.727	-0.003
cg19268652	6	12119552	2.24E-38	0.693	0.626	0.067	*HIVEP1*	4.71E-07	0.034
cg10835083	2	222395101	2.28E-38	0.750	0.694	0.056	*EPHA4*	2.91E-04	0.024
cg16535080	6	30460600	2.48E-38	0.727	0.676	0.050	*HLA-E*	8.25E-04	0.019
**cg08553572**	19	947765	2.50E-38	0.361	0.412	-0.051	*ARID3A*	0.280	-0.007
cg18983132	7	78024305	2.50E-38	0.733	0.683	0.050	*MAGI2*	6.86E-08	0.032
cg07143462	2	157289513	2.66E-38	0.498	0.551	-0.053	NA	0.003	-0.018
cg01715248	9	93953608	2.67E-38	0.379	0.431	-0.052	NA	0.024	-0.015
cg13102294	6	32121393	2.77E-38	0.369	0.425	-0.056	*PPT2*	4.82E-08	-0.034
cg22375663	12	69725435	2.91E-38	0.269	0.330	-0.061	NA	1.13E-04	-0.027
cg19747465	4	75174074	3.00E-38	0.716	0.662	0.055	*EPGN*	3.76E-14	0.047
cg27227317	2	161785635	3.06E-38	0.717	0.665	0.052	NA	2.11E-07	0.031
cg15439110	3	183153901	3.12E-38	0.776	0.712	0.064	NA	1.62E-09	0.045
cg20118822	4	119831531	3.68E-38	0.784	0.723	0.061	*SYNPO2*	1.53E-11	0.047
cg17178175	2	178109973	3.71E-38	0.560	0.612	-0.052	*NFE2L2*	1.50E-04	-0.022
cg06532212	19	47260078	3.96E-38	0.441	0.495	-0.054	*FKRP*	0.008	-0.015
cg09136878	1	236135584	4.01E-38	0.407	0.480	-0.073	NA	0.014	-0.020
**cg11222173**	17	78748019	4.05E-38	0.704	0.756	-0.052	*RPTOR*	0.655	-0.003
**cg08767938**	7	25989524	4.16E-38	0.246	0.306	-0.060	NA	0.120	-0.011
cg25261377	6	30313517	4.21E-38	0.172	0.223	-0.051	*RPP21*	0.003	-0.018
cg09179723	6	30704955	4.23E-38	0.582	0.528	0.055	*FLOT1*	0.005	0.018
cg13518537	8	28618150	4.64E-38	0.364	0.415	-0.050	NA	0.002	-0.018
cg11539052	2	179347266	5.11E-38	0.690	0.640	0.050	*PLEKHA3*	6.86E-09	0.033
cg21966764	11	6192512	5.20E-38	0.784	0.732	0.051	*OR52B2*	2.82E-07	0.030
cg06705237	9	97402555	5.21E-38	0.529	0.581	-0.052	*FBP1*	8.04E-07	-0.028
cg14130039	6	32121225	5.24E-38	0.310	0.368	-0.058	*PPT2*	3.18E-05	-0.028
**cg18297196**	6	41168941	5.71E-38	0.185	0.239	-0.054	*TREML2*	0.294	0.005
**cg01373248**	3	18480297	5.71E-38	0.375	0.320	0.055	*SATB1*	0.112	0.009
cg00670915	2	101620543	6.13E-38	0.391	0.444	-0.053	*RPL31*	0.031	-0.014
**cg08638320**	1	47900265	6.35E-38	0.411	0.463	-0.053	*FOXD2-AS1*	0.157	-0.008
cg23461824	7	134605363	6.69E-38	0.770	0.716	0.054	*CALD1*	9.67E-09	0.035
cg06818377	8	56793504	6.70E-38	0.241	0.295	-0.054	*LYN*	0.008	-0.016
cg04447756	5	138644986	6.82E-38	0.777	0.723	0.054	*MATR3*	3.09E-09	0.037
cg02077256	17	15133981	7.01E-38	0.743	0.684	0.059	*PMP22*	2.51E-06	0.033
cg06127316	11	9599460	7.12E-38	0.775	0.711	0.064	*WEE1*	3.22E-09	0.045
cg24159697	1	205181237	7.20E-38	0.165	0.220	-0.055	*DSTYK*	0.002	-0.021
cg13333954	12	1098663	8.27E-38	0.374	0.426	-0.052	NA	2.61E-04	-0.021
cg04816311	7	1066650	8.29E-38	0.463	0.521	-0.059	*C7orf50*	9.80E-09	-0.036
cg04339360	13	73635568	8.60E-38	0.431	0.491	-0.060	*KLF5*	0.020	-0.016
cg23679492	17	55828084	8.78E-38	0.550	0.606	-0.057	NA	1.75E-04	-0.023
**cg27407935**	17	17723235	8.84E-38	0.192	0.242	-0.051	*SREBF1*	0.267	-0.007
cg18980148	8	134552558	9.70E-38	0.359	0.420	-0.061	*ST3GAL1*	6.82E-05	-0.028
cg19075787	2	105372087	1.06E-37	0.252	0.304	-0.052	*LOC284998*	1.64E-09	-0.036
cg13741668	9	85679694	1.07E-37	0.659	0.595	0.063	NA	8.82E-06	0.033
**cg19778003**	3	111718062	1.10E-37	0.214	0.273	-0.059	*TAGLN3*	0.181	-0.010
cg07613153	14	104436947	1.16E-37	0.713	0.652	0.061	*TDRD9*	1.84E-04	0.026
cg06202737	2	128166279	1.17E-37	0.365	0.426	-0.061	NA	6.52E-08	-0.039

cg06635946	22	46470016	1.20E-37	0.629	0.684	-0.054	NA	1.93E-16	-0.048
cg02214623	5	180671896	1.23E-37	0.454	0.512	-0.057	*GNB2L1*	1.82E-07	-0.035
**cg09550083**	2	66672337	1.26E-37	0.279	0.340	-0.061	*MEIS1*	0.074	-0.013
cg14500300	9	211689	1.30E-37	0.551	0.614	-0.063	NA	0.026	-0.016
cg20693334	17	46654330	1.31E-37	0.341	0.403	-0.062	*HOXB4*	7.76E-04	-0.021
cg10066188	2	73927710	1.31E-37	0.383	0.438	-0.056	*NAT8B*	9.68E-05	-0.025
cg10863922	6	2892150	1.39E-37	0.792	0.733	0.060	*SERPINB9*	0.037	0.015
cg11663144	21	46675770	1.41E-37	0.720	0.653	0.068	NA	1.07E-05	0.033
cg25723459	11	126245829	1.42E-37	0.451	0.502	-0.051	*ST3GAL4*	0.035	-0.012
cg22396119	8	122419020	1.42E-37	0.326	0.382	-0.056	NA	2.41E-04	-0.024
cg13959031	9	88896340	1.44E-37	0.596	0.648	-0.052	*ISCA1*	1.04E-06	-0.029
**cg08110693**	6	36407533	1.51E-37	0.474	0.543	-0.069	*PXT1*	0.324	-0.008
**cg05507566**	2	36757828	1.56E-37	0.488	0.548	-0.059	*CRIM1*	0.091	-0.011
cg13203135	21	45565328	1.56E-37	0.499	0.437	0.062	*C21orf33*	1.27E-04	0.028
cg12592359	20	30944839	1.57E-37	0.532	0.595	-0.063	*ASXL1*	4.78E-05	-0.027
cg01944137	14	64974907	1.57E-37	0.791	0.739	0.052	*ZBTB1*	7.91E-06	0.027
cg13714407	9	134604212	1.57E-37	0.802	0.747	0.055	*RAPGEF1*	1.06E-06	0.031
cg01698392	17	76871734	1.59E-37	0.404	0.458	-0.054	*TIMP2*	1.68E-09	-0.034
cg00819338	3	133646065	1.70E-37	0.316	0.368	-0.053	NA	2.53E-05	-0.027
cg11370064	3	78318277	1.72E-37	0.604	0.537	0.067	NA	8.54E-05	0.029
cg04014328	17	46653615	1.79E-37	0.603	0.659	-0.056	*HOXB4*	1.39E-06	-0.028
cg11492723	3	16577697	1.86E-37	0.430	0.495	-0.065	NA	6.05E-12	-0.052
cg05360958	12	15038440	1.87E-37	0.320	0.379	-0.059	*MGP*	5.46E-09	-0.041
**cg16729631**	8	131000261	1.92E-37	0.663	0.722	-0.060	*FAM49B*	0.901	-0.001
cg00390511	7	36701016	1.97E-37	0.503	0.563	-0.060	*AOAH*	6.25E-07	-0.033
cg17850088	1	150119278	2.02E-37	0.700	0.644	0.056	NA	1.03E-14	0.050
cg16328023	7	36382071	2.05E-37	0.209	0.261	-0.052	*KIAA0895*	4.92E-09	-0.034
cg27226424	4	183795763	2.08E-37	0.401	0.462	-0.061	NA	0.007	-0.018
**cg18643093**	5	150521257	2.11E-37	0.432	0.380	0.052	*ANXA6*	0.327	0.006
**cg24830898**	5	177964873	2.13E-37	0.362	0.414	-0.052	*COL23A1*	0.647	-0.003
cg22016995	11	614787	2.20E-37	0.895	0.954	-0.060	*IRF7*	7.24E-16	-0.022
cg15551881	9	123688715	2.25E-37	0.365	0.303	0.062	*TRAF1*	3.19E-12	0.045
cg01613294	22	36557607	2.30E-37	0.768	0.713	0.055	*APOL3*	0.032	0.015
cg18860310	4	87752504	2.32E-37	0.684	0.737	-0.053	*SLC10A6*	2.87E-05	-0.024
cg12367786	21	39290161	2.44E-37	0.741	0.689	0.052	*KCNJ6*	0.013	0.016
**cg13276306**	8	108344200	2.47E-37	0.652	0.705	-0.052	*ANGPT1*	0.149	-0.008
cg04731988	20	3051954	2.49E-37	0.386	0.443	-0.057	*OXT*	7.05E-09	-0.039
cg13554818	19	43979316	2.58E-37	0.323	0.382	-0.059	*PHLDB3*	3.28E-06	-0.033
cg21005240	12	22688743	2.59E-37	0.716	0.651	0.065	*KIAA0528*	4.26E-10	0.048
cg04410448	6	144223614	2.92E-37	0.750	0.693	0.057	*ZC2HC1B*	5.37E-09	0.037
cg07250214	17	77763166	2.99E-37	0.447	0.501	-0.055	NA	8.67E-04	-0.020
**cg11510999**	12	53591490	3.55E-37	0.579	0.638	-0.059	*ITGB7*	0.336	-0.007
cg24611970	2	159790917	3.56E-37	0.812	0.760	0.052	NA	3.37E-09	0.036
**cg01219924**	17	79004947	3.60E-37	0.476	0.526	-0.051	*BAIAP2-AS1*	0.074	-0.010
cg14264316	9	134280803	3.78E-37	0.721	0.670	0.051	NA	0.001	0.019
**cg09706122**	12	54071165	3.91E-37	0.185	0.238	-0.054	*ATP5G2*	0.725	0.002
cg15082040	4	124299999	3.91E-37	0.698	0.634	0.064	NA	5.44E-14	0.057
cg23927970	11	504933	3.92E-37	0.312	0.367	-0.055	*RNH1*	7.11E-04	-0.019
**cg10790672**	5	78384465	4.10E-37	0.508	0.456	0.052	*BHMT2*	0.220	0.008
cg07582993	20	3137766	4.19E-37	0.738	0.680	0.058	*FASTKD5*	5.44E-06	0.031
cg15046062	8	38831857	4.30E-37	0.230	0.284	-0.054	*HTRA4*	0.048	-0.012
cg25178683	17	76976267	4.45E-37	0.581	0.657	-0.076	*LGALS3BP*	1.15E-17	-0.063
cg24336674	2	204602302	4.47E-37	0.805	0.754	0.050	*CD28*	3.84E-08	0.033
**cg24628744**	5	134735654	4.50E-37	0.197	0.258	-0.061	*H2AFY*	0.158	-0.011
cg04677123	5	90466501	4.69E-37	0.420	0.473	-0.053	NA	4.44E-05	-0.024
cg27185510	7	95811907	4.73E-37	0.768	0.707	0.062	*SLC25A13*	6.56E-15	0.056
**cg18158438**	11	64322994	4.86E-37	0.627	0.572	0.055	*SLC22A11*	0.068	0.012
cg25844590	11	7621556	5.22E-37	0.510	0.581	-0.071	*PPFIBP2*	0.007	-0.020
**cg13223682**	2	174889929	5.65E-37	0.492	0.545	-0.052	NA	0.092	-0.012
cg06974428	10	22972299	5.71E-37	0.627	0.574	0.052	*PIP4K2A*	0.015	0.014
cg09502619	16	1609529	5.79E-37	0.279	0.335	-0.056	*IFT140*	0.001	-0.022
cg22249612	12	56121485	5.90E-37	0.288	0.351	-0.062	*CD63*	0.006	-0.020
cg19223064	10	30351259	6.00E-37	0.373	0.458	-0.086	NA	7.09E-04	-0.028
cg09527615	11	94883350	6.47E-37	0.485	0.548	-0.063	NA	1.60E-09	-0.041
**cg13601309**	18	77443784	6.63E-37	0.538	0.600	-0.062	*CTDP1*	0.676	0.002
cg03893901	6	106498597	6.71E-37	0.799	0.748	0.052	NA	2.04E-12	0.043
**cg03044533**	11	96065844	6.96E-37	0.515	0.578	-0.064	*MAML2*	0.328	-0.007
**cg15001032**	7	50632994	7.03E-37	0.508	0.451	0.057	*DDC*	0.615	0.004
cg10874314	10	129766623	7.13E-37	0.522	0.574	-0.052	*PTPRE*	6.15E-04	-0.020
**cg09783253**	6	157932130	7.34E-37	0.282	0.341	-0.059	*ZDHHC14*	0.091	-0.012
cg13518270	20	50415588	7.56E-37	0.727	0.665	0.062	*SALL4*	5.12E-10	0.045
cg26140802	8	60444511	7.91E-37	0.659	0.605	0.054	NA	3.58E-05	0.026
cg14213590	9	139237503	8.16E-37	0.336	0.401	-0.065	*GPSM1*	8.33E-05	-0.027

cg00790071	2	102731407	8.51E-37	0.396	0.454	-0.058	NA	1.49E-04	-0.024
cg14485185	11	118051260	8.61E-37	0.305	0.357	-0.052	NA	3.90E-05	-0.024
**cg14079545**	6	33418310	8.63E-37	0.229	0.284	-0.055	*SYNGAP1*	0.955	0.000
cg12013258	12	776351	8.65E-37	0.749	0.698	0.051	NA	3.70E-07	0.031
cg04465154	8	9045558	9.13E-37	0.399	0.460	-0.061	NA	5.49E-08	-0.040
cg11702942	8	144102584	9.29E-37	0.771	0.826	-0.055	*LY6E*	2.01E-11	-0.025
cg09236311	3	137750103	9.30E-37	0.802	0.745	0.057	*CLDN18*	1.49E-05	0.029
cg04266607	7	28221667	9.46E-37	0.796	0.743	0.053	*JAZF1*	9.11E-08	0.034
cg15925478	1	94081080	1.03E-36	0.654	0.595	0.059	*BCAR3*	0.029	0.014
cg20240347	1	204465584	1.04E-36	0.616	0.555	0.062	NA	1.06E-08	0.038
**cg20064370**	11	47733615	1.05E-36	0.445	0.495	-0.050	*AGBL2*	0.886	-0.001
cg24727114	4	124707571	1.07E-36	0.789	0.736	0.053	*LOC285419*	6.43E-06	0.028
**cg26609120**	19	50848114	1.11E-36	0.582	0.530	0.052	NA	0.787	-0.001
**cg22286764**	3	37428639	1.15E-36	0.595	0.654	-0.060	NA	0.734	-0.003
cg19118519	13	29192851	1.15E-36	0.583	0.656	-0.073	NA	3.16E-06	-0.037
cg17446739	10	71892768	1.17E-36	0.188	0.239	-0.051	*AIFM2*	2.02E-05	-0.028
cg10550074	6	36304794	1.17E-36	0.655	0.595	0.061	*C6orf222*	4.39E-13	0.051
cg08119452	13	53602385	1.18E-36	0.304	0.358	-0.053	*OLFM4*	6.31E-05	-0.025
cg04677410	11	95846117	1.19E-36	0.756	0.695	0.061	*MAML2*	2.44E-06	0.034
cg01874152	12	55375859	1.19E-36	0.811	0.757	0.054	*TESPA1*	2.17E-10	0.041
cg04656831	6	29282808	1.30E-36	0.736	0.683	0.053	NA	2.52E-08	0.034
cg02291010	5	96079433	1.32E-36	0.548	0.601	-0.053	*CAST*	0.043	-0.012
cg02818170	19	3336941	1.38E-36	0.513	0.566	-0.053	NA	4.38E-07	-0.028
cg18835865	14	52020726	1.40E-36	0.758	0.707	0.051	*FRMD6*	2.33E-05	0.025
cg15666143	3	130061073	1.56E-36	0.726	0.662	0.064	NA	5.35E-07	0.039
cg23172853	1	231842206	1.74E-36	0.460	0.511	-0.052	*DISC1*	4.77E-06	-0.026
cg13027369	3	160279320	1.74E-36	0.722	0.658	0.064	*KPNA4*	3.45E-09	0.044
cg06829157	1	93430542	1.94E-36	0.709	0.648	0.061	NA	3.65E-08	0.040
cg04789403	15	80271218	2.08E-36	0.232	0.282	-0.050	NA	5.43E-16	-0.048
cg19810954	21	46496510	2.14E-36	0.662	0.606	0.056	*ADARB1*	0.002	0.020
cg07051721	2	120097181	2.15E-36	0.295	0.345	-0.051	*C2orf76*	6.61E-05	-0.025
cg10047762	17	80875998	2.24E-36	0.628	0.679	-0.051	*TBCD*	0.028	-0.013
cg20192387	6	166856056	2.33E-36	0.589	0.650	-0.061	*RPS6KA2*	0.011	-0.017
cg01376079	11	67070233	2.38E-36	0.193	0.248	-0.055	*SSH3*	0.002	-0.020
cg17500686	15	79621783	2.43E-36	0.532	0.597	-0.065	NA	0.041	-0.016
cg04436755	15	74218767	2.46E-36	0.433	0.483	-0.051	*LOXL1*	1.69E-05	-0.023
cg21566177	5	173312866	2.58E-36	0.241	0.303	-0.062	NA	0.008	-0.022
**cg14822490**	10	124909889	2.59E-36	0.301	0.356	-0.055	*HMX2*	0.319	0.006
cg11121969	21	47268670	2.84E-36	0.828	0.772	0.056	*PCBP3*	2.81E-11	0.044
cg14934522	4	77618676	2.87E-36	0.741	0.690	0.050	*SHROOM3*	1.99E-07	0.031
cg26688582	7	91477554	2.97E-36	0.412	0.470	-0.058	NA	0.002	-0.020
cg13615971	15	92392821	2.98E-36	0.425	0.476	-0.050	NA	1.03E-06	-0.029
cg15078975	11	85958536	3.11E-36	0.777	0.710	0.067	*EED*	2.38E-09	0.046
cg18860329	13	43354421	3.13E-36	0.546	0.617	-0.071	*FAM216B*	4.99E-10	-0.043
cg27061485	16	88989387	3.18E-36	0.540	0.591	-0.052	*CBFA2T3*	3.75E-06	-0.027
cg23421166	4	76976499	3.30E-36	0.812	0.761	0.051	*ART3*	6.09E-10	0.038
cg10131879	7	8011594	3.58E-36	0.718	0.668	0.051	*GLCCI1*	5.81E-11	0.039
cg17098979	2	241562085	3.93E-36	0.330	0.404	-0.074	*GPR35*	0.001	-0.028
cg08122070	1	1689610	4.04E-36	0.676	0.727	-0.051	*NADK*	1.07E-04	-0.024
cg10420310	16	14397576	4.10E-36	0.292	0.352	-0.059	NA	3.38E-04	-0.024
cg09883188	3	160502317	4.29E-36	0.724	0.667	0.057	*PPM1L*	5.66E-11	0.043
cg14935206	11	62367764	4.36E-36	0.524	0.472	0.053	*MTA2*	0.005	0.015
cg10777461	1	10590481	4.37E-36	0.530	0.587	-0.057	*PEX14*	4.99E-04	-0.023
cg27123256	14	99664279	4.47E-36	0.405	0.466	-0.061	*BCL11B*	7.66E-06	-0.030
cg25808094	13	75892615	4.47E-36	0.786	0.735	0.051	*TBC1D4*	1.31E-09	0.037
cg14930864	2	9759203	4.49E-36	0.736	0.680	0.057	*YWHAQ*	6.24E-12	0.046
**cg11576590**	1	152011357	4.92E-36	0.379	0.441	-0.061	NA	0.358	-0.007
**cg14089503**	8	37755555	4.96E-36	0.742	0.690	0.053	*RAB11FIP1*	0.060	0.011
cg26250585	11	59950745	5.07E-36	0.265	0.319	-0.054	*MS4A6A*	2.42E-13	-0.046
**cg17210837**	5	35954233	5.37E-36	0.638	0.578	0.060	*UGT3A1*	0.050	0.014
cg19851574	6	167178233	5.42E-36	0.762	0.687	0.075	*RPS6KA2*	7.21E-06	0.041
cg01988602	3	182473048	5.45E-36	0.750	0.688	0.063	NA	5.16E-07	0.037
cg05551825	7	5735129	6.14E-36	0.228	0.170	0.058	*RNF216*	0.005	0.015
cg14971586	22	43509649	6.18E-36	0.412	0.464	-0.051	*BIK*	0.012	-0.014
**cg14045860**	11	35158283	6.56E-36	0.494	0.556	-0.062	NA	0.377	-0.005
cg15925365	6	52858998	6.63E-36	0.635	0.695	-0.059	*GSTA4*	4.83E-10	-0.039
cg19710451	17	46654202	7.18E-36	0.352	0.412	-0.060	*HOXB4*	0.024	-0.014
cg12748948	1	22106295	7.53E-36	0.586	0.517	0.069	*USP48*	6.41E-09	0.047
cg11573318	11	65196227	7.62E-36	0.687	0.630	0.057	NA	1.67E-10	0.042
cg24114587	6	150212725	7.64E-36	0.799	0.743	0.055	*RAET1E*	5.36E-06	0.031
cg05194864	12	15100977	7.67E-36	0.683	0.618	0.065	*ARHGDIB*	1.35E-12	0.051
**cg13718729**	9	140056619	7.75E-36	0.281	0.337	-0.056	*GRIN1*	0.531	-0.004
cg08853735	8	129139526	8.01E-36	0.751	0.692	0.059	NA	1.46E-08	0.042

cg12620645	12	92899917	8.05E-36	0.583	0.520	0.063	NA	0.005	0.019
cg21850879	17	29621943	8.69E-36	0.486	0.540	-0.055	*NF1*	5.08E-05	-0.025
**cg19787694**	19	846117	8.93E-36	0.372	0.435	-0.063	*PRTN3*	0.320	-0.008
cg14662886	3	39450018	9.64E-36	0.829	0.777	0.053	*SNORA6*	3.96E-08	0.035
**cg26893816**	5	33997223	1.04E-35	0.650	0.705	-0.055	*AMACR*	0.235	-0.007
cg00311984	8	145051668	1.07E-35	0.598	0.544	0.054	*PLEC*	2.24E-06	0.028
cg00059089	2	153263288	1.08E-35	0.670	0.609	0.061	*FMNL2*	1.63E-05	0.031
**cg22630754**	15	42866523	1.11E-35	0.432	0.489	-0.057	*STARD9*	0.202	-0.009
cg05849676	12	10171151	1.11E-35	0.350	0.404	-0.054	*CLEC12B*	0.003	-0.019
cg05753328	8	124171421	1.14E-35	0.492	0.558	-0.066	NA	9.90E-09	-0.042
cg14392772	9	123666481	1.16E-35	0.459	0.406	0.053	*TRAF1*	2.75E-04	0.022
cg06173216	8	120219151	1.20E-35	0.783	0.733	0.050	*MAL2*	2.13E-10	0.035
**cg16679543**	6	157913024	1.21E-35	0.566	0.629	-0.063	*ZDHHC14*	0.105	-0.013
cg18176723	15	75336436	1.21E-35	0.532	0.588	-0.055	*PPCDC*	3.55E-04	-0.024
cg26400750	14	22992073	1.25E-35	0.766	0.715	0.051	NA	8.52E-08	0.034
cg10880928	9	139715701	1.30E-35	0.370	0.434	-0.064	*RABL6*	0.003	-0.021
cg09991306	2	241975140	1.30E-35	0.775	0.704	0.071	*SNED1*	7.78E-08	0.045
cg06712886	4	153771421	1.34E-35	0.445	0.505	-0.061	*ARFIP1*	0.024	-0.017
cg10881749	1	234792390	1.42E-35	0.459	0.518	-0.059	NA	8.89E-08	-0.036
**cg00287012**	18	21593174	1.43E-35	0.584	0.639	-0.055	*TTC39C*	0.120	-0.009
cg10389812	15	99458162	1.44E-35	0.773	0.713	0.060	*IGF1R*	1.74E-12	0.051
cg17222434	1	100215737	1.45E-35	0.661	0.604	0.057	*FRRS1*	2.27E-10	0.044
cg22440848	4	184320416	1.49E-35	0.387	0.463	-0.076	NA	3.91E-05	-0.039
cg13227806	3	113811946	1.51E-35	0.420	0.496	-0.077	NA	2.20E-08	-0.048
**cg04173586**	19	2167496	1.54E-35	0.572	0.653	-0.081	*DOT1L*	0.201	-0.012
**cg13558754**	19	36247867	1.73E-35	0.229	0.282	-0.054	*HSPB6*	0.068	0.010
cg11100481	5	149867577	1.76E-35	0.522	0.577	-0.055	NA	1.85E-05	-0.027
cg12170933	14	95620298	1.80E-35	0.803	0.746	0.057	*DICER1*	1.91E-12	0.049
cg03723907	5	61262350	1.81E-35	0.628	0.576	0.052	NA	8.92E-09	0.036
cg22886575	10	124910040	1.86E-35	0.336	0.388	-0.052	*HMX2*	0.030	-0.014
cg10711778	2	202317236	2.25E-35	0.239	0.293	-0.054	*TRAK2*	0.043	-0.014
cg23489137	2	161290449	2.29E-35	0.350	0.407	-0.058	*RBMS1*	3.38E-04	-0.026
**cg00153919**	16	88859944	2.31E-35	0.255	0.317	-0.063	NA	0.945	-0.001
cg03339537	2	157184816	2.45E-35	0.421	0.480	-0.059	*NR4A2*	9.58E-11	-0.046
cg20459712	6	56815929	2.48E-35	0.526	0.473	0.054	NA	8.79E-05	0.024
**cg26786893**	11	43705792	2.49E-35	0.618	0.669	-0.051	*HSD17B12*	0.122	-0.009
cg15272641	10	121775065	2.53E-35	0.779	0.720	0.059	NA	5.23E-09	0.043
cg04757492	8	145003862	2.61E-35	0.693	0.623	0.070	*PLEC*	4.14E-06	0.041
cg06234584	3	28389645	2.69E-35	0.244	0.296	-0.052	*AZI2*	0.004	-0.019
cg15657100	2	161420987	2.76E-35	0.784	0.731	0.053	NA	7.75E-12	0.045
cg12627844	2	64245000	2.77E-35	0.471	0.522	-0.051	*VPS54*	2.11E-12	-0.042
cg16965552	1	48937016	2.83E-35	0.439	0.493	-0.054	*SPATA6*	0.009	-0.018
cg19563525	12	69909397	2.94E-35	0.752	0.701	0.051	*FRS2*	1.54E-12	0.044
**cg09696044**	20	55968294	3.05E-35	0.361	0.298	0.064	*RBM38*	0.461	0.005
cg24074477	22	17956455	3.06E-35	0.461	0.517	-0.056	*CECR2*	0.011	-0.017
cg11984636	18	74845706	3.10E-35	0.337	0.390	-0.053	*MBP*	2.17E-09	-0.037
cg07643762	4	75174080	3.14E-35	0.796	0.745	0.051	*EPGN*	1.47E-12	0.042
cg00552704	6	32121420	3.29E-35	0.279	0.336	-0.056	*PPT2*	4.22E-05	-0.027
cg11027717	11	33713904	3.43E-35	0.368	0.418	-0.050	NA	1.99E-04	-0.023
cg26759223	22	46448690	3.44E-35	0.313	0.371	-0.058	*C22orf26*	3.39E-05	-0.028
cg22437405	1	38000170	3.55E-35	0.437	0.494	-0.057	*SNIP1*	0.002	-0.019
cg09958065	10	12648526	3.72E-35	0.281	0.338	-0.057	*CAMK1D*	0.018	-0.017
cg19951006	12	56732615	3.91E-35	0.605	0.547	0.058	*IL23A*	2.44E-05	0.028
**cg14109456**	9	127040562	4.04E-35	0.292	0.343	-0.051	*NEK6*	0.121	-0.010
**cg17791799**	4	16036123	4.15E-35	0.451	0.524	-0.073	*PROM1*	0.651	-0.004
cg05568930	11	75522399	4.17E-35	0.480	0.533	-0.053	NA	1.16E-05	-0.026
cg25837979	11	79114133	4.18E-35	0.707	0.652	0.055	*ODZ4*	8.17E-08	0.036
cg15576730	19	55693151	4.19E-35	0.277	0.327	-0.050	*SYT5*	0.007	-0.016
cg18049167	6	32121261	4.30E-35	0.229	0.280	-0.051	*PPT2*	2.02E-05	-0.028
cg09912793	12	9066382	4.34E-35	0.648	0.701	-0.053	*PHC1*	1.35E-12	-0.042
cg22891070	19	46801642	4.45E-35	0.410	0.516	-0.106	*HIF3A*	0.032	-0.027
cg00282195	2	168674769	4.56E-35	0.737	0.683	0.054	*B3GALT1*	1.69E-07	0.033
cg09866565	3	137844436	4.69E-35	0.299	0.352	-0.053	*A4GNT*	2.46E-08	-0.035
**cg26872780**	2	149478494	4.84E-35	0.448	0.506	-0.058	*EPC2*	0.422	-0.006
cg04481170	3	158445995	5.08E-35	0.322	0.381	-0.059	*RARRES1*	1.92E-10	-0.046
**cg14679780**	19	4059525	5.30E-35	0.714	0.663	0.051	*ZBTB7A*	0.522	0.003
**cg02368812**	6	3019900	5.31E-35	0.425	0.489	-0.063	*NQO2*	0.961	0.000
**cg12450823**	2	85752248	5.35E-35	0.568	0.624	-0.056	NA	0.240	-0.008
cg06846495	2	232326668	5.38E-35	0.487	0.432	0.055	*NCL*	3.36E-04	0.024
cg16962115	1	236016383	5.38E-35	0.471	0.530	-0.059	*LYST*	1.50E-04	-0.025
cg14593191	11	35201133	5.41E-35	0.748	0.690	0.057	*CD44*	1.41E-12	0.048
cg11586034	1	150117110	5.51E-35	0.430	0.482	-0.052	*VPS45*	4.95E-04	-0.023
cg04258520	14	103868351	5.53E-35	0.584	0.525	0.059	*MARK3*	2.54E-07	0.035

cg06346993	6	44093974	6.08E-35	0.672	0.615	0.057	*MRPL14*	3.66E-04	0.026
cg18462898	19	19282451	6.38E-35	0.452	0.510	-0.058	*MEF2BNB-ME*	3.76E-07	-0.032
cg02003183	14	103415882	6.57E-35	0.253	0.195	0.058	*CDC42BPB*	1.62E-08	0.032
**cg27367170**	10	5488628	6.59E-35	0.223	0.276	-0.053	*NET1*	0.110	0.009
cg08351489	11	92446329	6.80E-35	0.475	0.418	0.057	*FAT3*	0.006	0.018
cg13922442	7	38312366	6.80E-35	0.811	0.760	0.051	*TARP*	1.37E-11	0.044
cg10767665	19	44279027	6.88E-35	0.428	0.492	-0.064	*KCNN4*	1.61E-06	-0.035
**cg27408285**	12	54653364	6.90E-35	0.351	0.402	-0.051	*CBX5*	0.530	-0.004
cg02486421	8	131027724	7.19E-35	0.594	0.544	0.050	*FAM49B*	0.020	0.013
cg03648724	22	50052294	7.28E-35	0.445	0.509	-0.064	NA	1.76E-05	-0.033
cg24639703	1	64986155	7.50E-35	0.729	0.669	0.060	*CACHD1*	4.95E-07	0.038
cg21386573	1	94219800	7.95E-35	0.489	0.564	-0.074	*BCAR3*	5.81E-11	-0.054
cg27031099	8	126620534	8.91E-35	0.336	0.393	-0.057	NA	1.61E-05	-0.030
cg11277662	8	143408047	9.31E-35	0.556	0.625	-0.070	*TSNARE1*	0.014	-0.019
cg19371916	1	8874984	9.45E-35	0.281	0.333	-0.053	*RERE*	0.020	-0.014
**cg20177522**	20	62410437	9.61E-35	0.517	0.568	-0.051	*ZBTB46*	0.214	-0.008
cg02423185	13	37337525	9.67E-35	0.806	0.754	0.052	NA	4.38E-08	0.035
cg12126038	14	34966313	9.90E-35	0.596	0.649	-0.053	NA	8.48E-06	-0.027
cg00013349	1	77049380	1.07E-34	0.795	0.741	0.054	*ST6GALNAC3*	7.70E-09	0.038
**cg13266327**	2	11485561	1.13E-34	0.245	0.305	-0.060	*ROCK2*	0.352	-0.008
cg25863967	7	130049683	1.14E-34	0.528	0.579	-0.050	*CEP41*	0.047	-0.012
**cg17239057**	1	224363575	1.16E-34	0.399	0.459	-0.059	NA	0.228	-0.009
cg13331196	10	88295591	1.16E-34	0.267	0.321	-0.054	NA	0.009	-0.014
cg16552271	1	15392907	1.19E-34	0.430	0.372	0.058	*KAZN*	0.005	0.020
cg17341174	7	97923834	1.23E-34	0.574	0.626	-0.052	*BAIAP2L1*	0.022	-0.015
cg01839452	14	22433489	1.23E-34	0.797	0.739	0.058	NA	1.99E-13	0.051
cg20339443	10	65934509	1.27E-34	0.791	0.735	0.056	NA	1.36E-07	0.036
cg04084786	10	27609163	1.29E-34	0.280	0.350	-0.070	NA	0.013	-0.023
cg02556393	3	168866705	1.37E-34	0.398	0.462	-0.064	*MECOM*	1.02E-07	-0.041
cg01132484	3	176916496	1.40E-34	0.698	0.647	0.051	*TBL1XR1*	1.89E-06	0.029
cg23434815	10	75839302	1.43E-34	0.703	0.644	0.059	*VCL*	3.14E-08	0.038
cg13355129	12	58637434	1.44E-34	0.691	0.628	0.063	NA	7.82E-05	0.032
cg19028706	3	42158765	1.50E-34	0.413	0.469	-0.056	*TRAK1*	1.88E-04	-0.027
cg10809094	2	238006557	1.50E-34	0.752	0.686	0.065	*COPS8*	5.03E-11	0.050
cg26567012	5	151202148	1.52E-34	0.418	0.476	-0.058	*GLRA1*	0.017	-0.015
cg10616795	5	76464212	1.56E-34	0.704	0.764	-0.060	NA	2.00E-14	-0.048
cg24750513	2	127819455	1.60E-34	0.640	0.697	-0.057	*BIN1*	2.17E-05	-0.026
cg13391244	7	120629638	1.63E-34	0.363	0.421	-0.058	*CPED1*	2.36E-06	-0.035
cg20973735	11	67251677	1.63E-34	0.619	0.567	0.052	*AIP*	2.34E-07	0.031
cg12564698	11	68458524	1.65E-34	0.687	0.622	0.065	*GAL*	1.13E-06	0.038
cg24435741	11	10471371	1.69E-34	0.581	0.637	-0.056	*AMPD3*	0.005	-0.019
cg20684197	5	142066938	1.75E-34	0.739	0.687	0.052	*FGF1*	0.001	0.022
**cg22514722**	3	127473755	1.88E-34	0.630	0.570	0.060	*MGLL*	0.236	0.009
cg22095490	18	77560089	1.91E-34	0.689	0.638	0.051	NA	5.62E-08	0.036
cg16242615	19	4059988	1.92E-34	0.337	0.389	-0.052	*ZBTB7A*	1.05E-04	-0.025
**cg08632909**	11	70048796	1.99E-34	0.423	0.488	-0.065	*FADD*	0.714	0.003
cg19746982	18	77552568	2.15E-34	0.465	0.516	-0.051	NA	0.003	-0.018
cg14477767	4	170195438	2.18E-34	0.834	0.779	0.055	NA	3.74E-13	0.046
cg08238215	2	66673985	2.21E-34	0.233	0.284	-0.051	*MEIS1*	9.11E-04	-0.022
cg00904258	7	968194	2.34E-34	0.483	0.547	-0.063	*ADAP1*	0.012	-0.018
cg16331492	2	107456855	2.44E-34	0.758	0.708	0.050	*ST6GAL2*	3.92E-08	0.032
cg26337070	2	85999873	2.49E-34	0.593	0.667	-0.074	*ATOH8*	3.25E-05	-0.034
**cg19102955**	20	5928064	2.52E-34	0.338	0.399	-0.061	*TRMT6*	0.139	-0.011
cg17808910	7	8012104	2.58E-34	0.801	0.750	0.051	*GLCCI1*	5.86E-10	0.037
cg17430979	4	6034828	2.70E-34	0.460	0.528	-0.068	*JAKMIP1*	5.07E-12	-0.055
cg18533225	22	50986813	2.80E-34	0.435	0.493	-0.058	*KLHDC7B*	8.04E-06	-0.025
cg08017911	4	84526934	2.89E-34	0.837	0.784	0.052	*AGPAT9*	1.91E-09	0.039
cg13654573	14	53576363	2.94E-34	0.684	0.631	0.053	*DDHD1*	6.45E-06	0.029
cg02736966	4	148119477	2.94E-34	0.727	0.676	0.051	NA	5.50E-06	0.029
cg07166266	17	74067713	3.05E-34	0.639	0.580	0.059	*SRP68*	0.037	0.015
cg04007350	7	158263221	3.18E-34	0.520	0.596	-0.076	*PTPRN2*	0.015	-0.022
cg10552523	11	313478	3.28E-34	0.316	0.387	-0.070	*IFITM1*	4.66E-22	-0.069
cg22992279	4	26414901	3.29E-34	0.586	0.638	-0.052	*RBPJ*	0.040	-0.013
cg16291589	20	6023178	3.56E-34	0.564	0.618	-0.054	*LRRN4*	0.002	-0.020
cg05617307	10	121413182	3.68E-34	0.833	0.780	0.052	*BAG3*	1.44E-12	0.049
cg20595271	12	111889200	3.71E-34	0.646	0.592	0.054	*SH2B3*	1.51E-08	0.037
**cg26482939**	19	3136710	4.01E-34	0.272	0.329	-0.057	*GNA15*	0.661	0.003
cg23031196	12	46664096	4.02E-34	0.541	0.475	0.066	*SLC38A1*	6.65E-05	0.030
cg18346707	2	231732249	4.11E-34	0.216	0.267	-0.051	*ITM2C*	0.003	-0.019
cg13100600	1	958351	4.38E-34	0.602	0.528	0.073	*AGRN*	2.78E-05	0.031
cg00935653	7	25897725	4.55E-34	0.384	0.435	-0.051	NA	0.017	-0.014
cg03172657	16	89163625	4.56E-34	0.534	0.455	0.080	*ACSF3*	3.79E-13	0.071
cg03921696	3	156848137	4.60E-34	0.618	0.559	0.059	NA	5.72E-05	0.027

**cg06168950**	3	154737057	4.61E-34	0.445	0.506	-0.060	NA	0.730	-0.003
**cg06100756**	17	43221575	4.70E-34	0.243	0.305	-0.061	NA	0.476	-0.006
cg10202975	5	96070916	4.71E-34	0.353	0.408	-0.055	*CAST*	0.005	-0.019
cg05136804	18	32849113	4.73E-34	0.302	0.360	-0.058	*ZSCAN30*	0.001	-0.024
cg02060682	7	36190724	5.26E-34	0.551	0.603	-0.052	NA	2.77E-05	-0.024
cg00269725	6	156983315	5.42E-34	0.590	0.656	-0.066	NA	0.006	-0.022
**cg22875823**	6	33400543	5.56E-34	0.658	0.715	-0.056	*SYNGAP1*	0.216	-0.008
**cg25357825**	3	11697138	5.78E-34	0.357	0.411	-0.054	*VGLL4*	0.454	-0.005
**cg18926797**	14	69523229	5.84E-34	0.400	0.453	-0.054	*DCAF5*	0.278	-0.007
cg20009332	8	10489118	6.19E-34	0.442	0.498	-0.056	*RP1L1*	5.72E-06	-0.030
cg22534374	1	201511610	6.32E-34	0.579	0.638	-0.059	NA	6.72E-07	-0.033
cg07958192	4	90033974	6.33E-34	0.505	0.556	-0.052	*TIGD2*	3.07E-04	-0.023
cg14263391	17	37894014	6.37E-34	0.475	0.534	-0.058	*GRB7*	0.013	-0.016
cg25456728	17	66255048	6.38E-34	0.734	0.673	0.061	*ARSG*	7.58E-10	0.044
cg20146177	8	122639115	6.47E-34	0.712	0.658	0.054	*HAS2*	9.79E-06	0.030
cg09324018	2	16839610	6.53E-34	0.329	0.381	-0.052	*FAM49A*	8.35E-06	-0.029
cg08265495	8	11212668	6.54E-34	0.641	0.589	0.052	*TDH*	7.83E-10	0.039
cg19988482	2	394572	6.93E-34	0.660	0.592	0.068	NA	3.76E-04	0.029
cg07825294	19	1415757	6.98E-34	0.416	0.480	-0.064	*DAZAP1*	3.94E-05	-0.031
cg04977856	14	89886941	7.12E-34	0.543	0.617	-0.074	*FOXN3*	0.004	-0.025
cg12546793	1	158302740	7.24E-34	0.708	0.657	0.051	*CD1B*	1.85E-04	0.024
cg06647068	12	104853274	7.46E-34	0.348	0.399	-0.051	*CHST11*	2.70E-20	-0.059
**cg07481491**	19	3607130	7.53E-34	0.264	0.320	-0.056	*TBXA2R*	0.451	-0.005
cg19825589	19	42379710	7.87E-34	0.342	0.290	0.052	*CD79A*	7.63E-05	0.024
**cg16451527**	3	56889795	7.95E-34	0.467	0.523	-0.056	*ARHGEF3*	0.099	-0.011
**cg10256249**	2	65657578	8.28E-34	0.215	0.267	-0.052	*SPRED2*	0.210	-0.009
cg20518446	11	62315034	8.32E-34	0.511	0.576	-0.065	*AHNAK*	4.35E-08	-0.041
cg06779553	8	60020513	8.46E-34	0.518	0.461	0.057	*TOX*	3.72E-12	0.045
cg11808699	15	81528661	8.59E-34	0.381	0.442	-0.060	*IL16*	4.15E-10	-0.045
**cg27632402**	6	106957404	9.11E-34	0.441	0.511	-0.070	NA	0.089	-0.014
cg09678212	3	41240163	9.23E-34	0.206	0.260	-0.053	*CTNNB1*	6.76E-06	-0.030
cg23571857	17	6658898	9.78E-34	0.568	0.620	-0.052	*XAF1*	2.93E-04	-0.022
**cg06927812**	13	41718870	1.03E-33	0.440	0.498	-0.058	NA	0.808	-0.001
**cg25077558**	5	156991510	1.09E-33	0.561	0.625	-0.064	*ADAM19*	0.089	-0.012
**cg10239163**	2	25527366	1.11E-33	0.498	0.558	-0.061	*DNMT3A*	0.827	-0.002
**cg14023999**	15	90543224	1.11E-33	0.519	0.570	-0.051	NA	0.091	-0.010
cg04067066	2	219756890	1.15E-33	0.408	0.459	-0.051	*WNT10A*	2.24E-07	-0.033
cg13488811	11	128685313	1.17E-33	0.457	0.529	-0.073	NA	0.002	-0.026
cg01612140	6	78166436	1.18E-33	0.374	0.430	-0.056	NA	0.001	-0.021
cg26821681	8	125827396	1.19E-33	0.282	0.342	-0.060	NA	2.07E-07	-0.035
cg15021238	2	173305865	1.24E-33	0.707	0.650	0.057	*ITGA6*	5.98E-11	0.048
cg21093670	11	129684147	1.35E-33	0.652	0.592	0.060	NA	3.78E-10	0.046
cg21792737	6	155776999	1.38E-33	0.569	0.513	0.057	*NOX3*	0.002	0.022
cg05337681	15	58844500	1.41E-33	0.492	0.550	-0.058	*LIPC*	0.008	-0.020
cg13311549	7	24988669	1.44E-33	0.804	0.747	0.057	*OSBPL3*	2.66E-10	0.045
cg03263337	8	62199082	1.47E-33	0.657	0.604	0.053	*CLVS1*	0.018	0.015
cg11317199	9	100850391	1.54E-33	0.678	0.591	0.087	*TRIM14*	1.13E-15	0.073
cg13515856	8	61240832	1.74E-33	0.690	0.637	0.052	NA	2.17E-07	0.035
cg13073699	17	77805596	1.76E-33	0.308	0.366	-0.058	NA	0.017	-0.017
cg19805943	2	85933069	1.86E-33	0.563	0.647	-0.084	NA	0.015	-0.023
cg14018429	9	129138146	1.90E-33	0.367	0.418	-0.051	*FAM125B*	0.008	-0.018
cg24519157	20	54987662	1.98E-33	0.641	0.591	0.050	*CASS4*	0.003	0.018
cg03503046	8	106595191	2.04E-33	0.752	0.700	0.053	*ZFPM2*	2.92E-10	0.042
cg01891736	16	11655159	2.08E-33	0.557	0.502	0.055	*LITAF*	1.42E-15	0.052
**cg07248223**	17	38717275	2.10E-33	0.407	0.351	0.056	*CCR7*	0.177	0.008
**cg09346383**	17	75641322	2.15E-33	0.678	0.621	0.057	NA	0.118	0.009
cg23422763	10	60859034	2.40E-33	0.632	0.575	0.057	NA	8.43E-05	0.028
cg09712306	20	54949299	2.40E-33	0.269	0.323	-0.054	*AURKA*	4.20E-07	-0.034
cg03048947	1	225611366	2.42E-33	0.767	0.714	0.053	*LBR*	1.63E-06	0.032
cg21602520	18	60985380	2.43E-33	0.478	0.423	0.055	*BCL2*	9.01E-04	0.022
**cg00945108**	1	12806027	2.46E-33	0.597	0.543	0.055	*C1orf158*	0.312	0.006
**cg15183258**	15	42061865	2.53E-33	0.397	0.456	-0.059	*MGA*	0.238	0.008
cg05133205	6	32121249	2.59E-33	0.443	0.513	-0.070	*PPT2*	1.75E-06	-0.041
**cg02224021**	19	836716	2.60E-33	0.255	0.309	-0.054	NA	0.069	-0.011
cg00524179	7	27166853	2.61E-33	0.795	0.741	0.054	*HOXA3*	3.37E-12	0.045
cg15436123	4	3390332	2.71E-33	0.389	0.445	-0.056	*RGS12*	5.33E-05	-0.029
cg16453474	5	40982092	2.84E-33	0.673	0.616	0.057	*C7*	3.47E-07	0.035
cg15943139	6	22061708	3.07E-33	0.776	0.724	0.052	*LINC00340*	4.87E-06	0.029
cg11721178	12	106010541	3.18E-33	0.775	0.724	0.051	NA	2.28E-13	0.047
cg11302401	6	6688847	3.52E-33	0.478	0.538	-0.060	NA	0.003	-0.022
**cg15742737**	2	64863651	3.78E-33	0.212	0.271	-0.058	*SERTAD2*	0.790	0.001
cg07147033	1	1549615	3.80E-33	0.325	0.385	-0.061	*MIB2*	9.46E-04	-0.027
cg17312546	7	24561256	3.84E-33	0.711	0.659	0.052	NA	3.51E-04	0.023

cg02068981	14	54907137	3.93E-33	0.411	0.463	-0.052	*CNIH*	2.40E-06	-0.029
cg12992827	3	101901234	3.97E-33	0.698	0.750	-0.052	NA	3.63E-04	-0.021
cg23594656	7	65796392	4.03E-33	0.792	0.741	0.051	*TPST1*	3.86E-09	0.037
cg18773597	6	119854913	4.10E-33	0.592	0.541	0.051	NA	0.002	0.018
cg17316747	2	218149237	4.13E-33	0.749	0.699	0.051	*DIRC3*	6.93E-07	0.030
**cg18012089**	21	46327720	4.21E-33	0.315	0.368	-0.053	*ITGB2*	0.067	-0.013
cg09858862	1	23887514	4.49E-33	0.439	0.498	-0.059	*ID3*	0.003	-0.019
cg17567562	3	47687980	4.51E-33	0.500	0.556	-0.057	*SMARCC1*	0.010	0.017
cg04701716	16	66596317	4.53E-33	0.755	0.698	0.057	*CKLF*	2.75E-09	0.043
cg18120259	6	43894639	4.64E-33	0.527	0.580	-0.053	*LOC10013235*	6.28E-04	-0.020
cg14573478	3	140730858	4.65E-33	0.756	0.706	0.051	NA	9.23E-11	0.040
**cg16102739**	1	9224376	4.85E-33	0.456	0.512	-0.056	NA	0.072	-0.012
cg01055591	22	17629788	4.97E-33	0.606	0.657	-0.051	*CECR5*	0.002	-0.019
cg02009395	16	20702744	5.24E-33	0.728	0.673	0.054	*ACSM1*	1.52E-09	0.041
cg23050705	1	231694386	5.36E-33	0.726	0.670	0.056	*TSNAX*	5.81E-13	0.051
cg09162993	5	127922712	5.46E-33	0.786	0.731	0.055	NA	1.05E-10	0.044
cg08966889	6	52440429	5.50E-33	0.594	0.645	-0.052	*TRAM2*	3.03E-04	-0.024
cg15414930	6	17985564	5.50E-33	0.609	0.659	-0.050	*KIF13A*	5.51E-18	-0.051
cg20326674	14	63179119	5.89E-33	0.729	0.678	0.052	*KCNH5*	1.61E-05	0.029
cg07180646	1	15481541	6.10E-33	0.528	0.587	-0.059	*TMEM51*	1.38E-05	-0.029
cg24472823	7	51296131	6.27E-33	0.769	0.713	0.056	*COBL*	2.69E-11	0.047
cg12636607	6	112154581	6.42E-33	0.796	0.742	0.053	*FYN*	1.19E-08	0.038
cg12564453	16	56995840	6.50E-33	0.428	0.491	-0.064	*CETP*	2.42E-04	-0.028
cg26406186	3	160520336	7.25E-33	0.787	0.732	0.055	*PPM1L*	4.67E-11	0.047
cg22602513	1	160429714	7.58E-33	0.750	0.689	0.061	NA	1.22E-12	0.054
cg18720622	5	1229256	7.74E-33	0.649	0.588	0.061	*SLC6A18*	4.90E-04	0.026
cg05995576	15	35049543	7.84E-33	0.739	0.686	0.053	NA	3.94E-08	0.037
cg09439920	3	99979117	8.41E-33	0.383	0.436	-0.053	*TBC1D23*	0.021	-0.015
cg07435331	17	21178476	8.47E-33	0.474	0.526	-0.051	NA	7.98E-12	-0.045
**cg27424995**	1	27902555	9.26E-33	0.175	0.237	-0.063	*AHDC1*	0.948	-0.001
cg15781504	11	4871411	9.64E-33	0.796	0.745	0.051	*OR51S1*	5.18E-07	0.032
cg13421247	12	123756945	9.66E-33	0.235	0.285	-0.050	*CDK2AP1*	0.001	-0.022
cg18468842	20	45313109	1.14E-32	0.389	0.440	-0.051	*TP53RK*	0.014	-0.016
cg15385476	1	196112818	1.16E-32	0.788	0.736	0.053	NA	8.06E-11	0.044
cg08099136	6	32811251	1.19E-32	0.337	0.398	-0.061	*PSMB8*	9.96E-21	-0.070
cg05486872	18	3066822	1.27E-32	0.349	0.404	-0.055	*MYOM1*	0.002	-0.021
**cg27141751**	10	50231850	1.44E-32	0.475	0.532	-0.057	*VSTM4*	0.488	-0.005
cg08769212	4	930351	1.46E-32	0.812	0.754	0.059	*TMEM175*	7.38E-13	0.053
cg21088514	21	35884376	1.47E-32	0.616	0.670	-0.054	*KCNE1*	4.16E-04	-0.023
cg14236955	6	19695425	1.52E-32	0.741	0.690	0.051	NA	2.55E-05	0.027
cg27049094	2	75067716	1.60E-32	0.560	0.500	0.060	*HK2*	6.68E-13	0.047
**cg11930926**	4	174429326	1.67E-32	0.199	0.251	-0.052	NA	0.483	-0.005
cg04609875	12	70763703	1.71E-32	0.752	0.701	0.051	*KCNMB4*	2.12E-08	0.035
cg17072494	6	149401166	1.72E-32	0.559	0.611	-0.052	NA	0.031	-0.013
cg22226592	1	109439213	1.74E-32	0.449	0.500	-0.051	*GPSM2*	0.049	-0.012
cg17094356	11	104789337	1.79E-32	0.701	0.643	0.057	NA	6.62E-08	0.040
cg19857379	1	25105430	1.84E-32	0.712	0.647	0.065	*CLIC4*	1.79E-10	0.055
cg26269677	5	76251688	2.02E-32	0.553	0.610	-0.056	*CRHBP*	0.003	-0.020
cg24494102	4	48015981	2.06E-32	0.719	0.668	0.051	*CNGA1*	1.13E-06	0.031
cg23738760	12	11918643	2.26E-32	0.407	0.457	-0.050	*ETV6*	4.82E-11	-0.042
cg14597388	5	54319373	2.35E-32	0.812	0.758	0.054	*GZMK*	1.92E-09	0.042
cg03249011	11	65195995	2.44E-32	0.545	0.490	0.055	NA	5.49E-07	0.033
cg19550524	11	124043067	2.44E-32	0.821	0.770	0.051	NA	7.42E-10	0.041
cg05785753	11	71189490	2.54E-32	0.477	0.530	-0.053	*NADSYN1*	0.044	-0.010
cg18818075	6	44528865	2.77E-32	0.371	0.421	-0.050	NA	3.69E-05	-0.028
cg15076824	1	159685185	2.90E-32	0.750	0.699	0.051	*CRP*	5.92E-08	0.034
cg19139092	3	69588171	2.91E-32	0.711	0.655	0.057	NA	6.31E-07	0.036
cg04863679	3	176759870	3.01E-32	0.820	0.766	0.054	*TBL1XR1*	0.003	0.021
**cg18655633**	19	48107418	3.09E-32	0.682	0.631	0.051	NA	0.776	0.000
cg08746853	12	14924953	3.12E-32	0.557	0.500	0.057	*HIST4H4*	8.86E-06	0.031
**cg22700246**	15	99978986	3.36E-32	0.714	0.663	0.051	NA	0.131	0.010
cg12492273	7	2119499	3.36E-32	0.442	0.509	-0.067	*MAD1L1*	0.028	-0.019
cg27073337	11	133998086	3.55E-32	0.767	0.711	0.055	*JAM3*	9.61E-07	0.035
cg02202664	6	155545253	3.62E-32	0.620	0.569	0.051	*TIAM2*	2.99E-08	0.035
cg20640261	6	31707019	3.96E-32	0.482	0.532	-0.051	*MSH5*	8.77E-13	-0.045
cg23484392	18	66465334	3.97E-32	0.587	0.643	-0.056	*CCDC102B*	2.92E-13	-0.049
cg09811510	3	159590447	3.99E-32	0.790	0.738	0.052	*SCHIP1*	1.90E-05	0.028
cg23260111	10	46158095	4.29E-32	0.764	0.705	0.059	*ZFAND4*	2.28E-10	0.049
cg08496387	11	11862730	4.42E-32	0.398	0.459	-0.061	*USP47*	7.55E-05	-0.032
cg19159011	7	50348870	4.55E-32	0.502	0.451	0.050	*IKZF1*	0.004	0.016
cg07891862	8	141774605	4.58E-32	0.811	0.756	0.055	*PTK2*	3.94E-14	0.053
cg17375396	11	67202808	4.73E-32	0.482	0.424	0.058	*RPS6KB2*	0.001	0.024
cg18924562	12	10242785	5.01E-32	0.776	0.723	0.052	*CLEC1A*	9.42E-06	0.027

cg23051091	8	122001888	5.19E-32	0.804	0.753	0.050	NA	4.17E-08	0.034
cg21422361	7	1884392	5.25E-32	0.567	0.618	-0.050	*MAD1L1*	0.003	-0.018
cg04570265	1	228679629	5.29E-32	0.798	0.744	0.053	*RNF187*	3.47E-14	0.050
cg01952989	7	1986334	5.40E-32	0.685	0.751	-0.066	*MAD1L1*	0.020	-0.016
cg06549901	16	55851316	5.48E-32	0.604	0.657	-0.053	*CES1*	7.49E-08	-0.036
cg08973950	7	1083309	5.50E-32	0.427	0.487	-0.061	*C7orf50*	1.65E-09	-0.045
cg14583999	3	10019040	5.60E-32	0.514	0.565	-0.051	*EMC3*	5.58E-10	-0.041
cg11111139	7	157620685	5.71E-32	0.473	0.526	-0.053	*PTPRN2*	1.23E-04	-0.024
cg17786516	3	46410422	5.81E-32	0.682	0.626	0.056	*CCR5*	1.08E-08	0.040
cg04524040	19	4153364	5.92E-32	0.529	0.472	0.057	*CREB3L3*	4.70E-04	0.022
cg07356486	3	133646095	6.21E-32	0.292	0.343	-0.051	NA	1.50E-04	-0.025
cg23907051	2	101730305	6.39E-32	0.516	0.586	-0.070	*TBC1D8*	0.023	-0.019
cg23435671	8	29211078	6.47E-32	0.290	0.346	-0.056	NA	0.001	-0.023
**cg04476865**	13	36223476	6.59E-32	0.598	0.650	-0.052	*NBEA*	0.105	-0.011
cg20022118	7	8276021	6.78E-32	0.759	0.703	0.056	*ICA1*	6.23E-13	0.051
cg19116959	4	146841472	6.86E-32	0.430	0.484	-0.053	*ZNF827*	0.007	-0.017
cg07660627	17	35481970	6.94E-32	0.743	0.681	0.062	*ACACA*	2.62E-09	0.046
cg06153925	17	78755379	7.41E-32	0.174	0.224	-0.050	*RPTOR*	0.014	-0.017
cg08328483	16	31119196	7.50E-32	0.747	0.692	0.055	*BCKDK*	7.08E-04	0.025
cg15262325	6	157296274	7.53E-32	0.820	0.769	0.052	*ARID1B*	1.49E-08	0.037
**cg20310100**	11	68081207	8.44E-32	0.361	0.412	-0.051	*LRP5*	0.105	-0.011
cg01152567	3	28294009	8.67E-32	0.769	0.717	0.052	*CMC1*	3.76E-08	0.036
cg27141474	2	38358225	8.72E-32	0.494	0.556	-0.062	NA	4.11E-04	-0.027
cg05502766	3	122604506	8.78E-32	0.745	0.689	0.056	NA	3.32E-10	0.045
**cg09729012**	3	72395774	8.92E-32	0.308	0.362	-0.054	NA	0.975	0.000
cg12550496	6	169284344	9.90E-32	0.887	0.837	0.051	NA	0.029	0.015
**cg08126542**	6	37504118	9.92E-32	0.230	0.282	-0.052	NA	0.232	-0.008
**cg09290120**	2	157291759	1.01E-31	0.469	0.525	-0.056	*GPD2*	0.223	-0.008
cg06065019	2	177356276	1.01E-31	0.335	0.275	0.061	NA	1.57E-10	0.048
**cg19535096**	4	68751711	1.02E-31	0.323	0.378	-0.055	NA	0.103	-0.011
**cg08839808**	6	156983304	1.03E-31	0.487	0.554	-0.067	NA	0.097	-0.013
cg17884674	19	1967198	1.03E-31	0.582	0.527	0.055	*CSNK1G2*	0.003	0.019
cg15090899	6	167178260	1.04E-31	0.808	0.750	0.058	*RPS6KA2*	5.33E-10	0.049
cg15602298	1	157670825	1.06E-31	0.705	0.653	0.051	*FCRL3*	0.007	0.017
cg19956889	10	11650681	1.19E-31	0.787	0.735	0.052	*USP6NL*	2.96E-13	0.049
cg19200953	13	100470738	1.21E-31	0.580	0.515	0.065	*CLYBL*	3.29E-06	0.040
cg02703190	5	93137476	1.22E-31	0.778	0.727	0.051	*FAM172A*	2.48E-10	0.042
cg22698744	21	36263808	1.24E-31	0.498	0.577	-0.079	*RUNX1*	1.14E-06	-0.044
cg26714205	6	49522045	1.24E-31	0.755	0.695	0.060	NA	4.29E-11	0.050
cg07563725	11	95408124	1.32E-31	0.791	0.735	0.056	NA	1.49E-09	0.044
**cg20464719**	19	17889512	1.49E-31	0.292	0.363	-0.071	*FCHO1*	0.981	-0.001
**cg01032675**	19	3136430	1.52E-31	0.180	0.242	-0.062	*GNA15*	0.077	0.012
cg26886572	11	3009206	1.66E-31	0.675	0.605	0.069	*NAP1L4*	1.33E-04	0.033
**cg08454507**	17	78755406	1.71E-31	0.196	0.247	-0.052	*RPTOR*	0.085	-0.013
cg24933919	13	77298665	1.78E-31	0.473	0.526	-0.053	NA	0.009	-0.017
cg14683065	10	134149184	1.92E-31	0.245	0.296	-0.052	*LRRC27*	0.025	-0.016
cg05232694	20	48809539	2.00E-31	0.389	0.443	-0.054	NA	1.64E-10	-0.045
cg17293195	11	71237522	2.01E-31	0.748	0.693	0.055	*KRTAP5-7*	1.04E-04	0.028
cg08100069	9	139014709	2.32E-31	0.402	0.453	-0.051	NA	4.21E-07	-0.030
cg03700230	7	25702848	2.34E-31	0.749	0.692	0.057	NA	5.60E-07	0.036
cg03174507	10	21789582	2.37E-31	0.384	0.435	-0.051	NA	0.027	-0.015
cg01113811	11	95927950	2.57E-31	0.675	0.620	0.055	*MAML2*	8.34E-05	0.027
cg25170091	17	202716	2.63E-31	0.294	0.350	-0.057	*RPH3AL*	4.37E-08	-0.039
cg26068677	11	92282995	2.80E-31	0.752	0.694	0.058	*FAT3*	1.66E-12	0.054
cg23533270	10	78635553	2.82E-31	0.737	0.681	0.056	*KCNMA1*	9.25E-08	0.039
cg14555759	5	39167171	2.84E-31	0.709	0.637	0.072	*FYB*	1.45E-08	0.051
cg26909797	18	9911079	2.88E-31	0.801	0.749	0.052	NA	3.64E-09	0.039
cg13302154	12	15039432	2.94E-31	0.390	0.451	-0.061	*MGP*	3.92E-11	-0.050
cg27043141	3	111851265	3.40E-31	0.747	0.695	0.052	*GCSAM*	2.74E-10	0.043
cg03411579	12	76552736	3.41E-31	0.483	0.429	0.054	NA	0.043	0.013
cg13190608	17	37322730	3.41E-31	0.506	0.556	-0.050	*ARL5C*	6.96E-07	-0.031
cg01322214	7	25219198	3.43E-31	0.418	0.484	-0.066	*C7orf31*	4.70E-06	-0.041
cg07368857	3	71276336	3.48E-31	0.704	0.650	0.054	*FOXP1*	0.008	0.014
**cg23824801**	12	54653403	3.62E-31	0.334	0.386	-0.052	*CBX5*	0.329	-0.008
cg17022548	5	139294892	3.65E-31	0.698	0.644	0.053	*NRG2*	8.09E-04	0.023
cg04247152	16	88590325	3.79E-31	0.578	0.640	-0.062	*ZFPM1*	0.006	-0.020
cg00017931	6	157932180	3.98E-31	0.221	0.275	-0.054	*ZDHHC14*	8.54E-04	-0.025
cg04137405	14	62414451	4.04E-31	0.633	0.575	0.059	NA	1.24E-05	0.032
**cg15548427**	3	158520857	4.11E-31	0.351	0.407	-0.056	*MFSD1*	0.065	-0.014
cg03201166	3	24170261	4.12E-31	0.771	0.719	0.051	*THRB*	4.83E-05	0.027
cg09863374	2	170652065	4.13E-31	0.810	0.757	0.053	NA	1.13E-05	0.030
**cg02639359**	19	17862017	4.18E-31	0.393	0.343	0.050	*FCHO1*	0.824	0.001
cg21140290	3	169975155	4.30E-31	0.800	0.742	0.058	*PRKCI*	1.01E-10	0.047

cg07350262	15	86098611	4.65E-31	0.291	0.342	-0.050	*AKAP13*	5.05E-05	-0.027
cg24789682	4	152217416	4.76E-31	0.756	0.701	0.055	NA	1.22E-06	0.035
cg12110437	8	144098888	4.86E-31	0.340	0.417	-0.077	*LOC10013366*	1.10E-08	-0.053
cg20224780	10	132942362	4.88E-31	0.733	0.674	0.059	*TCERG1L*	4.46E-14	0.058
cg26346167	3	131004472	4.95E-31	0.724	0.673	0.051	*NEK11*	4.37E-08	0.037
cg11669285	17	40558061	5.31E-31	0.621	0.673	-0.052	*PTRF*	0.004	-0.019
cg19592472	20	3052274	6.06E-31	0.364	0.437	-0.073	*OXT*	3.35E-07	-0.047
**cg13551117**	2	111979398	6.42E-31	0.380	0.435	-0.055	NA	0.060	-0.014
cg02319972	15	62798895	6.68E-31	0.701	0.646	0.055	NA	0.001	0.024
cg19743666	2	30371484	6.90E-31	0.432	0.506	-0.074	*YPEL5*	0.009	-0.024
cg21253943	4	53968201	6.96E-31	0.793	0.742	0.051	*SCFD2*	1.08E-13	0.050
**cg07176692**	1	113500329	7.08E-31	0.548	0.492	0.056	*SLC16A1*	0.728	0.002
**cg15360181**	3	143567492	7.83E-31	0.446	0.390	0.056	*SLC9A9*	0.395	0.005
cg21975834	5	133847811	7.84E-31	0.583	0.634	-0.051	NA	0.021	-0.015
cg14788046	12	31402470	8.80E-31	0.444	0.497	-0.053	NA	2.47E-07	-0.033
cg03143849	11	2904951	8.86E-31	0.511	0.562	-0.051	*CDKN1C*	8.02E-08	-0.035
**cg04450052**	6	170525426	8.93E-31	0.678	0.623	0.055	NA	0.568	0.003
**cg00549475**	17	10632715	9.34E-31	0.216	0.269	-0.053	*TMEM220*	0.479	-0.006
cg13702222	3	152017240	9.65E-31	0.394	0.340	0.053	*MBNL1*	1.93E-07	0.034
cg16756527	21	35891075	1.02E-30	0.532	0.583	-0.050	*RCAN1*	2.27E-06	-0.031
cg21879146	6	55887782	1.10E-30	0.745	0.694	0.051	NA	5.89E-08	0.036
cg12221689	2	98827983	1.31E-30	0.591	0.522	0.069	*VWA3B*	4.64E-06	0.041
cg17807458	7	150263087	1.32E-30	0.826	0.776	0.050	*GIMAP4*	4.86E-13	0.048
cg15715337	1	85600447	1.35E-30	0.378	0.437	-0.059	NA	0.003	-0.022
cg16075649	15	57595298	1.35E-30	0.422	0.492	-0.070	*LOC283663*	8.65E-06	-0.038
cg07423149	1	203156246	1.38E-30	0.406	0.458	-0.052	*CHI3L1*	8.20E-05	-0.025
cg27577554	17	30412142	1.42E-30	0.370	0.442	-0.073	NA	0.023	-0.021
cg21565575	20	35274281	1.42E-30	0.509	0.455	0.054	*SLA2*	1.14E-04	0.027
cg19409546	12	72477363	1.44E-30	0.747	0.697	0.050	NA	2.97E-07	0.034
cg19460508	22	44422195	1.75E-30	0.717	0.768	-0.051	*PARVB*	8.27E-09	-0.036
cg11798182	14	98444513	1.82E-30	0.732	0.679	0.054	NA	8.73E-05	0.027
cg27035169	14	23653098	1.88E-30	0.337	0.387	-0.050	*SLC7A8*	4.66E-07	-0.033
cg00810971	2	65085764	1.90E-30	0.457	0.507	-0.051	NA	1.85E-05	-0.027
**cg07805542**	1	9779309	1.98E-30	0.331	0.392	-0.061	*PIK3CD*	0.150	-0.012
cg11025609	12	110727072	1.98E-30	0.737	0.681	0.055	*ATP2A2*	3.62E-13	0.053
**cg07109453**	1	234669825	2.02E-30	0.562	0.615	-0.053	NA	0.051	-0.012
cg10173124	2	127963653	2.05E-30	0.730	0.672	0.058	*CYP27C1*	4.42E-14	0.058
**cg02343628**	15	93790914	2.06E-30	0.388	0.456	-0.068	NA	0.847	0.001
cg15361750	19	47839132	2.15E-30	0.261	0.311	-0.050	*GPR77*	8.86E-06	-0.029
**cg14329860**	2	51240310	2.24E-30	0.453	0.508	-0.055	*NRXN1*	0.943	-0.001
cg24306340	1	64937451	2.35E-30	0.360	0.411	-0.050	*CACHD1*	6.03E-06	-0.032
cg07277549	7	30509141	2.48E-30	0.481	0.534	-0.052	*NOD1*	0.001	-0.022
cg03811829	1	949449	2.58E-30	0.792	0.845	-0.053	*ISG15*	4.51E-05	-0.021
cg07073964	19	698371	2.59E-30	0.553	0.607	-0.054	NA	0.011	-0.018
cg01514490	10	7455338	2.62E-30	0.532	0.585	-0.052	NA	0.003	-0.021
**cg14189391**	2	25527347	2.67E-30	0.468	0.529	-0.061	*DNMT3A*	0.554	0.004
cg03257293	1	226927663	2.73E-30	0.504	0.449	0.055	*ITPKB*	0.006	0.019
cg03976856	1	2764669	2.96E-30	0.675	0.620	0.054	NA	6.36E-04	0.027
cg07510282	10	90644018	3.01E-30	0.812	0.760	0.053	*STAMBPL1*	2.17E-09	0.042
cg26337954	3	111315207	3.50E-30	0.811	0.757	0.054	*ZBED2*	8.15E-07	0.036
cg00074771	15	44120341	3.51E-30	0.729	0.673	0.056	*WDR76*	8.74E-08	0.042
cg02147208	6	21662648	3.58E-30	0.734	0.678	0.056	NA	2.29E-08	0.041
cg10499974	3	46244099	3.60E-30	0.552	0.604	-0.052	*CCR1*	1.45E-04	-0.024
cg08038054	7	93550781	3.74E-30	0.425	0.483	-0.058	*GNG11*	4.24E-07	-0.037
cg20479688	12	53046513	3.96E-30	0.787	0.735	0.052	*KRT2*	2.03E-08	0.039
**cg14931884**	10	369977	4.03E-30	0.616	0.563	0.053	*DIP2C*	0.111	0.009
**cg07805029**	1	92953256	4.16E-30	0.573	0.624	-0.051	*GFI1*	0.077	-0.010
cg22582875	17	80545272	4.56E-30	0.241	0.296	-0.055	*FOXK2*	1.40E-04	-0.030
cg12686920	17	41739806	4.69E-30	0.598	0.539	0.059	*MEOX1*	8.89E-06	0.036
cg10751070	10	96143568	4.99E-30	0.391	0.474	-0.083	NA	0.027	-0.021
cg16761097	2	68897545	5.12E-30	0.758	0.701	0.057	NA	1.63E-10	0.048
**cg25702651**	3	192675515	5.41E-30	0.248	0.310	-0.062	NA	0.053	-0.017
cg24713204	19	57019373	5.45E-30	0.274	0.325	-0.051	*ZNF471*	0.044	0.012
**cg10244976**	16	1014818	5.67E-30	0.830	0.779	0.051	*LMF1*	0.281	0.007
cg13932916	7	39606965	5.88E-30	0.747	0.696	0.051	*YAE1D1*	1.14E-05	0.029
cg19692929	14	92575120	5.91E-30	0.505	0.454	0.051	NA	7.50E-04	0.023
cg19340390	7	151087656	6.15E-30	0.209	0.259	-0.050	*WDR86*	1.40E-05	-0.030
**cg04309234**	6	106441468	6.18E-30	0.334	0.388	-0.055	NA	0.109	-0.010
cg20981219	14	77385200	6.49E-30	0.672	0.724	-0.052	NA	2.56E-08	-0.036
cg17207736	8	142237307	6.71E-30	0.495	0.557	-0.061	*SLC45A4*	0.002	-0.024
cg05492071	2	202650064	7.83E-30	0.462	0.512	-0.051	NA	0.002	-0.022
cg00106565	5	146784171	8.02E-30	0.760	0.707	0.054	*DPYSL3*	1.42E-08	0.040
cg05705813	11	86383809	8.40E-30	0.460	0.512	-0.052	*ME3*	3.62E-07	-0.038

cg07291349	4	40964962	8.52E-30	0.441	0.492	-0.051	*APBB2*	0.002	-0.019
cg06567195	8	94937996	8.75E-30	0.843	0.793	0.051	*PDP1*	1.40E-09	0.041
cg11177526	1	158900384	8.87E-30	0.618	0.552	0.066	*PYHIN1*	9.49E-06	0.039
cg09464268	17	32266	9.06E-30	0.453	0.528	-0.075	*DOC2B*	7.19E-05	-0.032
cg00133624	12	3756130	9.11E-30	0.325	0.384	-0.059	*EFCAB4B*	9.67E-04	-0.028
cg15431659	3	194901357	1.01E-29	0.322	0.372	-0.051	*XXYLT1*	2.29E-04	-0.025
**cg20907614**	8	29914963	1.06E-29	0.588	0.641	-0.053	NA	0.840	-0.002
cg22929787	5	141737266	1.10E-29	0.401	0.453	-0.052	NA	3.49E-04	-0.023
cg08166767	1	152671348	1.14E-29	0.711	0.653	0.058	*LCE2A*	3.31E-12	0.055
**cg03529261**	4	185209641	1.15E-29	0.563	0.510	0.053	NA	0.074	0.011
cg12999267	12	94376970	1.17E-29	0.414	0.346	0.068	NA	3.66E-06	0.038
cg04951822	12	113345598	1.19E-29	0.363	0.424	-0.061	*OAS1*	7.42E-25	-0.087
cg17291423	7	158263204	1.26E-29	0.368	0.431	-0.063	*PTPRN2*	0.002	-0.026
cg02132667	14	80957817	1.33E-29	0.682	0.621	0.061	NA	2.92E-04	0.025
cg19578660	10	3511600	1.34E-29	0.259	0.312	-0.052	NA	0.002	-0.023
cg02520639	8	82021210	1.45E-29	0.359	0.300	0.059	*PAG1*	0.003	0.023
**cg25019722**	6	37503610	1.58E-29	0.310	0.368	-0.058	NA	0.788	0.001
cg18642369	13	99651231	1.60E-29	0.332	0.276	0.056	*DOCK9*	0.005	0.020
**cg10575075**	10	31288634	1.62E-29	0.526	0.583	-0.057	*ZNF438*	0.291	-0.008
cg09254210	14	61873622	1.67E-29	0.713	0.645	0.068	*PRKCH*	1.96E-10	0.058
cg16533146	12	27901072	1.81E-29	0.685	0.735	-0.050	*MRPS35*	0.028	-0.013
**cg13662290**	7	150264311	1.84E-29	0.637	0.571	0.066	*GIMAP4*	0.054	0.017
cg06738031	10	126806416	1.86E-29	0.755	0.703	0.052	*CTBP2*	2.91E-12	0.048
**cg06022561**	2	242702553	1.89E-29	0.634	0.582	0.052	*D2HGDH*	0.956	0.001
**cg22613854**	3	194705954	1.90E-29	0.289	0.345	-0.056	NA	0.079	-0.013
cg04104235	2	20104329	1.93E-29	0.693	0.641	0.051	NA	2.70E-09	0.041
cg16011250	12	68024459	1.99E-29	0.615	0.564	0.051	NA	7.87E-10	0.040
cg19024980	13	100781335	2.01E-29	0.738	0.685	0.053	*PCCA*	5.30E-09	0.042
cg25772365	6	140762224	2.16E-29	0.652	0.591	0.061	NA	4.75E-08	0.044
cg17090901	8	133837475	2.29E-29	0.807	0.755	0.052	*PHF20L1*	3.07E-12	0.048
cg03899219	3	122333057	2.51E-29	0.800	0.749	0.050	*PARP15*	9.13E-12	0.047
cg19636302	19	46032854	2.58E-29	0.326	0.382	-0.056	*OPA3*	0.008	-0.019
cg11521212	4	75366983	2.58E-29	0.796	0.738	0.058	NA	6.60E-05	0.033
cg23350904	17	4648580	2.68E-29	0.218	0.269	-0.051	*ZMYND15*	0.011	-0.013
**cg19005485**	3	197272328	2.71E-29	0.498	0.559	-0.060	*BDH1*	0.181	-0.011
cg11548083	8	10208156	3.07E-29	0.415	0.468	-0.053	*MSRA*	1.10E-04	-0.027
cg16414852	4	70626128	3.08E-29	0.520	0.465	0.055	*SULT1B1*	2.73E-12	0.052
cg05385188	17	80215375	3.11E-29	0.722	0.664	0.058	*CSNK1D*	4.41E-08	0.043
cg24639969	1	247580565	3.22E-29	0.424	0.480	-0.056	*NLRP3*	2.85E-05	-0.031
**cg21069500**	5	139050159	3.24E-29	0.372	0.422	-0.050	*CXXC5*	0.470	-0.006
cg22386008	2	177737177	3.25E-29	0.754	0.699	0.055	NA	5.41E-07	0.038
cg09255851	16	24555610	3.28E-29	0.591	0.536	0.055	*RBBP6*	5.95E-04	0.025
cg09265876	12	25537944	3.29E-29	0.473	0.536	-0.063	NA	8.30E-05	-0.034
cg06038367	16	30198370	3.31E-29	0.264	0.212	0.052	*CORO1A*	0.001	0.020
cg10276433	6	47011312	3.31E-29	0.304	0.359	-0.055	*GPR110*	4.69E-04	-0.027
cg07120254	6	47199817	3.45E-29	0.712	0.655	0.056	*TNFRSF21*	1.97E-07	0.039
cg16306870	3	194868790	3.67E-29	0.260	0.314	-0.054	*XXYLT1*	0.047	-0.016
cg10170847	19	41834029	3.75E-29	0.407	0.352	0.055	NA	9.73E-05	0.028
cg00191102	8	1958171	3.75E-29	0.725	0.669	0.057	NA	2.34E-05	0.031
cg17769523	3	182003594	3.81E-29	0.774	0.721	0.053	NA	7.07E-08	0.038
cg07141002	22	38201690	4.00E-29	0.386	0.446	-0.059	*H1F0*	5.95E-04	-0.027
cg01291381	22	40811960	4.01E-29	0.283	0.337	-0.054	*MKL1*	0.007	-0.020
cg18302225	5	55776401	4.09E-29	0.737	0.678	0.060	NA	0.027	0.017
**cg02578087**	3	8671361	4.22E-29	0.795	0.737	0.057	*C3orf32*	0.701	0.003
cg20197130	12	127256717	4.43E-29	0.653	0.602	0.051	*LOC387895*	8.29E-04	0.022
cg11852794	14	92198970	4.51E-29	0.794	0.740	0.054	*CATSPERB*	1.25E-04	0.029
cg25634507	8	59413577	4.63E-29	0.623	0.567	0.057	*CYP7A1*	3.12E-12	0.053
cg00719668	20	34541013	4.66E-29	0.362	0.413	-0.050	NA	1.87E-04	-0.026
**cg03351487**	2	169003820	4.77E-29	0.535	0.594	-0.058	*STK39*	0.599	-0.003
cg19119071	6	83904067	5.02E-29	0.291	0.348	-0.057	*PGM3*	0.005	-0.024
cg07582204	3	43275279	5.12E-29	0.736	0.674	0.062	NA	5.80E-08	0.045
cg17492395	2	42180858	5.25E-29	0.790	0.739	0.051	*C2orf91*	2.94E-05	0.029
cg03571959	16	2660876	5.54E-29	0.558	0.610	-0.052	*LOC652276*	0.042	-0.014
**cg03461851**	15	62457883	5.97E-29	0.246	0.297	-0.051	*C2CD4B*	0.305	-0.007
cg11214001	5	96078430	6.42E-29	0.409	0.476	-0.067	*CAST*	0.012	-0.021
cg12656312	11	6024266	7.11E-29	0.611	0.553	0.057	*OR56A4*	0.020	0.017
cg09889350	16	56995813	7.19E-29	0.505	0.563	-0.058	*CETP*	0.002	-0.025
cg07938480	16	3265228	7.87E-29	0.646	0.589	0.057	NA	2.58E-12	0.051
cg00879541	14	52466952	9.06E-29	0.827	0.777	0.050	*C14orf166*	5.89E-08	0.039
cg22803642	10	60198125	9.44E-29	0.739	0.688	0.051	NA	2.53E-04	0.026
cg11735008	1	42380309	9.54E-29	0.702	0.644	0.058	*HIVEP3*	9.76E-06	0.032
**cg07104417**	11	69259247	9.85E-29	0.566	0.620	-0.054	NA	0.502	-0.005
cg23549571	2	71409816	1.06E-28	0.743	0.687	0.056	NA	1.54E-07	0.040

cg02648589	13	114877260	1.18E-28	0.463	0.518	-0.055	*RASA3*	0.003	-0.021
**cg11341144**	3	185656289	1.27E-28	0.458	0.520	-0.063	*TRA2B*	0.556	0.005
cg08153404	12	116946758	1.29E-28	0.372	0.439	-0.067	NA	0.005	-0.026
cg19448816	3	16974217	1.34E-28	0.499	0.558	-0.059	*PLCL2*	6.94E-09	-0.045
**cg27507473**	16	86002958	1.52E-28	0.455	0.404	0.052	NA	0.052	0.013
cg22579028	22	27056901	1.60E-28	0.552	0.615	-0.063	*MIAT*	8.68E-05	-0.028
cg23676767	1	226918736	1.69E-28	0.715	0.646	0.069	*ITPKB*	9.06E-07	0.045
**cg11609556**	17	79006044	1.75E-28	0.413	0.466	-0.053	*BAIAP2-AS1*	0.057	-0.013
cg01073479	16	3509474	1.76E-28	0.635	0.584	0.051	*NAA60*	7.78E-04	0.023
cg02958515	15	39650295	1.79E-28	0.664	0.611	0.053	NA	1.81E-04	0.027
cg07141605	12	133005406	1.88E-28	0.258	0.312	-0.054	NA	5.03E-04	-0.026
**cg09366969**	1	144932914	2.06E-28	0.262	0.316	-0.053	*PDE4DIP*	0.136	-0.011
cg13033061	3	155198149	2.30E-28	0.759	0.699	0.061	*PLCH1*	9.08E-10	0.049
cg04170440	6	154410350	2.38E-28	0.791	0.737	0.054	*OPRM1*	3.19E-07	0.038
**cg20320656**	12	51442996	2.46E-28	0.763	0.713	0.050	*LETMD1*	0.131	0.010
cg12125241	15	85575240	2.48E-28	0.767	0.712	0.055	*PDE8A*	6.35E-12	0.054
**cg18113826**	6	31583942	2.49E-28	0.194	0.266	-0.071	*AIF1*	0.495	-0.008
cg09107438	12	56819060	2.50E-28	0.675	0.625	0.050	*TIMELESS*	6.29E-06	0.030
cg06545367	7	110731527	2.52E-28	0.800	0.747	0.054	*IMMP2L*	3.86E-13	0.057
cg07099186	3	61947083	2.64E-28	0.682	0.624	0.058	*PTPRG*	5.88E-08	0.043
cg14484681	9	135158694	2.65E-28	0.792	0.741	0.051	*SETX*	6.94E-14	0.049
**cg21780711**	5	122765476	2.70E-28	0.558	0.614	-0.056	NA	0.138	-0.011
cg26053876	14	22991735	2.88E-28	0.699	0.634	0.065	NA	7.13E-10	0.056
cg07971716	1	199773294	3.03E-28	0.791	0.740	0.050	NA	3.51E-04	0.025
cg12662084	6	17809126	3.24E-28	0.651	0.718	-0.067	*KIF13A*	0.001	-0.027
cg09160955	15	89154092	3.25E-28	0.239	0.289	-0.050	NA	7.44E-04	-0.023
**cg02985240**	1	235468148	3.33E-28	0.676	0.619	0.057	*ARID4B*	0.125	0.012
**cg23950714**	5	176935364	3.45E-28	0.404	0.455	-0.052	*DOK3*	0.665	-0.003
cg13340231	8	81549775	3.51E-28	0.708	0.656	0.052	*ZNF704*	3.37E-04	0.026
cg02346997	19	7733883	4.10E-28	0.379	0.429	-0.050	*RETN*	1.50E-04	-0.024
cg22486834	6	52859107	4.31E-28	0.621	0.674	-0.053	*GSTA4*	1.64E-06	-0.034
cg07661704	4	139144433	4.40E-28	0.555	0.502	0.053	*SLC7A11*	1.62E-09	0.041
cg07579107	15	53910786	4.48E-28	0.737	0.680	0.057	*WDR72*	4.38E-10	0.048
cg02092790	17	39094800	4.94E-28	0.774	0.724	0.050	*KRT23*	3.18E-07	0.037
cg15349696	17	80545434	5.26E-28	0.486	0.539	-0.053	*FOXK2*	0.003	-0.022
cg16362173	14	50374131	5.33E-28	0.766	0.714	0.051	NA	2.50E-07	0.037
cg14544831	18	60985269	6.04E-28	0.588	0.535	0.053	*BCL2*	0.020	0.018
cg12594803	3	196908930	6.16E-28	0.338	0.414	-0.076	*DLG1*	0.036	-0.020
**cg27511181**	17	78685224	6.28E-28	0.488	0.539	-0.050	*RPTOR*	0.129	-0.010
cg20856330	6	30313546	6.46E-28	0.220	0.273	-0.053	*RPP21*	0.005	-0.023
cg26072749	17	46657274	6.49E-28	0.271	0.322	-0.051	*MIR10A*	5.83E-06	-0.031
cg17426969	13	28803434	6.59E-28	0.791	0.739	0.052	*PAN3*	1.62E-12	0.050
cg07119830	10	104412306	6.63E-28	0.458	0.401	0.057	*TRIM8*	0.002	0.021
cg03255417	13	49357650	6.76E-28	0.339	0.390	-0.051	NA	0.005	-0.021
**cg23478547**	11	69259265	6.77E-28	0.445	0.519	-0.074	NA	0.294	-0.012
**cg12149606**	14	75614186	7.08E-28	0.433	0.483	-0.050	*TMED10*	0.220	-0.009
cg10981907	11	60775545	7.11E-28	0.253	0.316	-0.063	*CD6*	0.045	-0.019
cg04319276	19	42445244	7.23E-28	0.261	0.312	-0.051	NA	0.039	-0.016
cg23901918	10	105420747	7.90E-28	0.244	0.306	-0.061	*SH3PXD2A*	0.032	-0.020
cg07816556	6	26017280	1.02E-27	0.749	0.698	0.051	*HIST1H1A*	3.79E-11	0.048
**cg04003615**	5	179486382	1.14E-27	0.562	0.615	-0.053	*RNF130*	0.595	-0.004
**cg10107890**	2	44314289	1.15E-27	0.501	0.559	-0.057	NA	0.093	-0.014
**cg10107292**	1	15504438	1.20E-27	0.263	0.320	-0.056	*TMEM51*	0.182	-0.010
cg24465935	6	153938192	1.20E-27	0.775	0.723	0.051	NA	1.31E-07	0.037
cg07585427	1	11917550	1.27E-27	0.654	0.603	0.051	*NPPB*	3.82E-06	0.030
**cg19586698**	8	92097851	1.37E-27	0.548	0.599	-0.051	*OTUD6B*	0.192	0.009
cg10186366	17	71898817	1.38E-27	0.293	0.345	-0.053	NA	0.031	-0.014
cg06963192	1	172557938	1.41E-27	0.781	0.731	0.051	*SUCO*	7.16E-12	0.047
**cg13397898**	9	133768931	1.57E-27	0.424	0.477	-0.052	*QRFP*	0.885	-0.002
cg00328227	1	109204325	1.61E-27	0.509	0.457	0.052	*HENMT1*	4.03E-07	0.033
cg16861031	12	105477526	1.71E-27	0.250	0.307	-0.056	*ALDH1L2*	0.013	-0.019
**cg04942251**	11	63687247	1.78E-27	0.539	0.599	-0.060	NA	0.223	-0.010
cg13072943	6	167011311	1.85E-27	0.483	0.535	-0.052	*RPS6KA2*	2.79E-13	-0.048
**cg17086398**	1	31896392	2.08E-27	0.384	0.329	0.054	*SERINC2*	0.247	0.009
cg16219583	5	135702333	2.26E-27	0.760	0.708	0.051	*TRPC7*	5.06E-10	0.044
cg08370787	5	148208041	2.31E-27	0.725	0.674	0.051	*ADRB2*	7.84E-06	0.031
cg07107453	1	79114976	2.37E-27	0.350	0.418	-0.068	*IFI44*	6.44E-10	-0.054
cg17633576	12	16726789	2.49E-27	0.289	0.340	-0.052	*LMO3*	0.012	-0.018
**cg20021790**	17	181288	2.55E-27	0.669	0.617	0.052	*LOC10050638*	0.546	0.004
cg20435594	11	733141	2.56E-27	0.412	0.466	-0.053	NA	0.015	-0.018
cg09635768	1	8601318	2.60E-27	0.786	0.729	0.057	*RERE*	1.10E-09	0.049
cg12437809	1	171153544	2.79E-27	0.682	0.624	0.058	*FMO2*	1.02E-12	0.056
**cg05004818**	4	8539015	3.00E-27	0.493	0.551	-0.058	NA	0.205	-0.010

cg03850986	10	116408382	3.04E-27	0.474	0.528	-0.053	*ABLIM1*	1.62E-09	-0.045
cg05702218	1	174843909	3.06E-27	0.355	0.412	-0.057	*RABGAP1L*	3.80E-06	-0.038
cg25815893	7	55199930	3.35E-27	0.744	0.687	0.056	*EGFR*	1.14E-08	0.045
**cg00531137**	16	57643932	3.37E-27	0.558	0.500	0.058	NA	0.565	0.003
cg04724319	17	1009219	3.54E-27	0.552	0.604	-0.052	*ABR*	0.001	-0.022
cg16353615	17	21367564	3.64E-27	0.387	0.440	-0.053	NA	0.016	-0.018
cg11937920	15	70995381	3.92E-27	0.698	0.646	0.052	*UACA*	1.86E-06	0.035
cg18949056	10	97317243	4.06E-27	0.753	0.702	0.051	*SORBS1*	6.04E-08	0.040
cg03466717	16	49564370	4.24E-27	0.330	0.407	-0.077	*ZNF423*	2.58E-04	-0.032
cg04962480	8	101962118	4.34E-27	0.445	0.392	0.054	*YWHAZ*	0.047	0.013
cg27646075	11	118048216	4.62E-27	0.767	0.715	0.052	*SCN2B*	7.34E-12	0.051
cg09610792	11	94798056	4.77E-27	0.750	0.695	0.054	NA	2.45E-10	0.046
cg01468579	10	122102016	5.30E-27	0.742	0.691	0.052	NA	4.37E-07	0.038
cg26166177	2	216260832	5.87E-27	0.765	0.707	0.058	*FN1*	1.37E-07	0.044
cg21830050	1	891193	6.00E-27	0.368	0.432	-0.064	*NOC2L*	0.004	-0.026
cg14602393	12	133343405	6.09E-27	0.481	0.555	-0.074	NA	2.03E-05	-0.042
**cg10548102**	12	16499963	6.12E-27	0.533	0.585	-0.052	*MGST1*	0.406	-0.006
cg05721199	3	169629136	6.40E-27	0.762	0.700	0.061	*SAMD7*	2.79E-15	0.068
cg16041798	14	64905375	6.89E-27	0.763	0.711	0.052	*MTHFD1*	5.56E-16	0.061
**cg00168785**	2	160142643	7.97E-27	0.175	0.226	-0.050	*WDSUB1*	0.060	-0.015
cg12437013	13	114161939	8.25E-27	0.499	0.571	-0.071	*TMCO3*	1.91E-04	-0.037
**cg19749916**	6	106441456	8.44E-27	0.369	0.423	-0.054	NA	0.483	-0.005
cg05757376	13	36516069	8.72E-27	0.778	0.725	0.053	*DCLK1*	6.77E-10	0.045
cg12682382	8	74787918	9.19E-27	0.477	0.581	-0.104	*UBE2W*	8.97E-04	-0.043
cg01878807	14	24422368	9.49E-27	0.375	0.428	-0.053	*DHRS4-AS1*	3.59E-05	-0.028
**cg16998831**	7	187686	1.12E-26	0.521	0.580	-0.059	NA	0.498	-0.006
cg20953364	5	31122837	1.16E-26	0.755	0.703	0.052	NA	4.70E-09	0.043
cg18100746	10	6390958	1.18E-26	0.770	0.717	0.053	NA	9.74E-09	0.043
cg05149213	10	101381631	1.19E-26	0.296	0.349	-0.053	*SLC25A28*	0.005	-0.021
cg07676709	17	46673442	1.19E-26	0.536	0.588	-0.052	*HOXB-AS3*	2.53E-07	-0.037
**cg18674234**	14	78107677	1.21E-26	0.359	0.411	-0.052	NA	0.159	-0.011
cg02265239	14	59748448	1.25E-26	0.783	0.730	0.053	*DAAM1*	2.21E-08	0.042
cg03538296	1	15392433	1.32E-26	0.821	0.767	0.055	*KAZN*	7.36E-09	0.047
cg01534390	14	23265401	1.45E-26	0.753	0.698	0.054	*SLC7A7*	1.24E-04	0.030
cg13257421	2	9987440	1.46E-26	0.595	0.648	-0.052	*TAF1B*	1.81E-06	-0.031
**cg20773479**	5	619994	1.57E-26	0.568	0.620	-0.052	*CEP72*	0.328	-0.006
cg01785719	8	143404187	1.67E-26	0.790	0.842	-0.052	*TSNARE1*	0.046	-0.011
cg27021181	10	5488346	1.74E-26	0.514	0.565	-0.052	*NET1*	3.04E-08	-0.040
cg19861978	2	139033234	1.76E-26	0.645	0.591	0.054	NA	1.44E-06	0.036
cg00830121	16	85904858	1.84E-26	0.803	0.747	0.057	NA	5.44E-11	0.052
cg16804603	6	21856613	2.00E-26	0.394	0.447	-0.053	*LINC00340*	0.037	-0.016
cg00918944	9	35908117	2.26E-26	0.317	0.381	-0.064	NA	1.36E-04	-0.034
cg17514528	1	11862907	2.30E-26	0.433	0.489	-0.056	*MTHFR*	9.78E-09	-0.045
cg15046909	6	53882926	2.35E-26	0.718	0.664	0.054	*MLIP*	0.003	0.022
cg06126421	6	30720080	2.87E-26	0.727	0.785	-0.058	NA	7.33E-07	-0.034
cg24921943	5	171847617	2.91E-26	0.313	0.381	-0.068	*SH3PXD2B*	0.044	-0.021
cg16993108	7	1986301	3.00E-26	0.724	0.781	-0.057	*MAD1L1*	0.007	-0.018
cg19430537	17	74118361	3.07E-26	0.298	0.366	-0.068	NA	0.003	-0.028
cg27490875	2	69418131	3.16E-26	0.633	0.582	0.051	*ANTXR1*	8.31E-07	0.033
cg05060607	12	8211391	3.33E-26	0.450	0.520	-0.070	*C3AR1*	0.003	-0.028
cg19213808	17	49336082	3.36E-26	0.706	0.655	0.052	*MBTD1*	4.25E-10	0.048
cg11068881	8	40925730	3.48E-26	0.809	0.757	0.051	NA	5.99E-07	0.037
cg14913512	6	4772890	3.61E-26	0.765	0.712	0.053	*CDYL*	6.06E-08	0.042
cg19466822	14	99726317	3.90E-26	0.719	0.660	0.059	*BCL11B*	0.009	0.022
**cg19563932**	6	31583915	4.03E-26	0.111	0.161	-0.050	*AIF1*	0.135	-0.012
cg21495622	22	24199125	4.42E-26	0.294	0.347	-0.053	*SLC2A11*	0.005	-0.022
cg00444581	8	141856401	4.85E-26	0.824	0.765	0.060	*PTK2*	5.56E-11	0.054
cg06033321	1	86011409	4.86E-26	0.765	0.712	0.053	*DDAH1*	0.001	0.023
cg20995304	12	48196167	5.01E-26	0.527	0.578	-0.051	*HDAC7*	0.008	-0.020
cg01309328	6	32811253	5.04E-26	0.361	0.414	-0.053	*PSMB8*	3.03E-17	-0.059
**cg14548802**	9	137675380	5.21E-26	0.328	0.277	0.051	*COL5A1*	0.072	0.011
cg13561372	7	121781778	5.67E-26	0.767	0.710	0.057	*AASS*	1.11E-09	0.050
**cg09718037**	12	117042854	6.20E-26	0.661	0.717	-0.056	NA	0.268	-0.008
cg05079547	11	131239592	6.28E-26	0.692	0.638	0.054	*NTM*	1.32E-08	0.045
cg12684958	4	74921425	7.35E-26	0.806	0.751	0.055	NA	5.24E-05	0.033
cg08832695	17	46676375	7.84E-26	0.521	0.573	-0.052	*HOXB-AS3*	1.36E-08	-0.039
**cg20469139**	17	29297458	9.72E-26	0.321	0.381	-0.060	*RNF135*	0.765	-0.002
**cg00694302**	12	47609865	1.08E-25	0.692	0.630	0.062	*PCED1B-AS1*	0.118	0.013
cg00366252	16	85436841	1.29E-25	0.277	0.329	-0.052	NA	0.013	-0.019
cg09743437	5	43000790	1.30E-25	0.349	0.410	-0.061	NA	2.12E-07	-0.043
cg17134700	6	29854631	1.33E-25	0.461	0.526	-0.065	NA	1.78E-04	-0.032
cg06426114	17	6355097	1.33E-25	0.565	0.623	-0.058	*PITPNM3*	1.50E-08	-0.045
cg09721961	4	58235517	1.38E-25	0.579	0.527	0.052	NA	1.65E-04	0.028

cg18794976	5	160802610	1.39E-25	0.676	0.625	0.051	*GABRB2*	8.90E-06	0.031
cg15173586	8	124784802	1.47E-25	0.562	0.511	0.051	*FAM91A1*	1.15E-09	0.041
**cg24175188**	3	58374923	1.55E-25	0.462	0.515	-0.053	*PXK*	0.460	-0.005
cg23201059	8	91322832	1.84E-25	0.804	0.751	0.053	*LINC00534*	2.22E-11	0.049
cg11744817	6	166876826	1.89E-25	0.321	0.396	-0.075	*RPS6KA2*	1.84E-07	-0.051
cg07757577	8	122264107	2.10E-25	0.777	0.725	0.052	NA	1.31E-05	0.033
cg11100795	5	77583915	2.13E-25	0.491	0.551	-0.059	*AP3B1*	3.27E-05	-0.034
cg17280172	11	123082796	2.31E-25	0.785	0.729	0.056	NA	5.44E-13	0.058
cg00519069	19	14491737	2.56E-25	0.400	0.348	0.052	*CD97*	0.015	0.016
cg06457011	20	39767490	2.81E-25	0.379	0.318	0.060	*PLCG1*	3.37E-04	0.028
cg12246945	7	139257086	2.84E-25	0.740	0.690	0.050	*HIPK2*	6.31E-09	0.044
cg14286514	9	32525315	2.88E-25	0.531	0.611	-0.080	*DDX58*	2.94E-04	-0.040
**cg05494467**	5	140892308	2.90E-25	0.189	0.240	-0.052	*PCDHGA1*	0.959	0.000
cg10753966	3	55556197	2.91E-25	0.624	0.558	0.066	*ERC2*	5.86E-05	0.039
cg19384448	1	10977926	2.93E-25	0.653	0.587	0.066	NA	0.018	0.021
cg09245989	1	2454811	3.03E-25	0.632	0.581	0.051	*PANK4*	2.14E-08	0.042
cg20599009	11	6945204	3.14E-25	0.799	0.747	0.052	NA	2.24E-08	0.043
cg14624145	17	37024169	3.15E-25	0.533	0.603	-0.070	NA	4.33E-06	-0.046
**cg18395636**	11	87908785	3.19E-25	0.200	0.250	-0.051	*RAB38*	0.128	0.011
cg11079711	17	38929662	3.19E-25	0.613	0.556	0.057	*KRT26*	0.015	0.020
cg14832378	14	59038939	3.28E-25	0.774	0.711	0.063	NA	1.15E-05	0.041
cg22708150	6	31649619	3.41E-25	0.510	0.562	-0.052	*LY6G5C*	5.56E-06	-0.034
cg13804575	9	109626999	4.09E-25	0.391	0.452	-0.061	*ZNF462*	0.006	-0.025
cg24115596	14	38051289	4.62E-25	0.817	0.767	0.050	NA	6.53E-09	0.043
cg17608381	6	29911550	4.77E-25	0.487	0.599	-0.112	*HLA-A*	3.36E-15	-0.117
cg14276379	9	97663142	5.38E-25	0.410	0.463	-0.053	*C9orf3*	0.001	-0.023
cg18118795	1	33117735	5.48E-25	0.581	0.530	0.052	*RBBP4*	1.73E-10	0.050
cg26300461	10	32401032	5.70E-25	0.794	0.741	0.053	NA	1.13E-07	0.041
cg09926562	15	89812172	5.75E-25	0.786	0.735	0.051	*FANCI*	3.45E-09	0.046
cg01176433	15	69754714	6.00E-25	0.351	0.405	-0.054	NA	1.74E-06	-0.035
cg24113534	13	33791982	6.34E-25	0.741	0.688	0.053	*STARD13*	1.62E-09	0.044
cg14573759	18	73966755	7.13E-25	0.766	0.715	0.051	NA	1.24E-14	0.060
cg18855195	22	45828264	7.69E-25	0.470	0.523	-0.054	*RIBC2*	6.54E-08	-0.043
cg17681079	7	27556963	7.76E-25	0.805	0.754	0.051	NA	9.54E-14	0.055
cg17100943	19	6459300	7.78E-25	0.314	0.366	-0.052	*SLC25A23*	0.016	-0.018
cg22939324	10	91128729	8.13E-25	0.756	0.703	0.053	NA	1.39E-12	0.056
**cg11853830**	5	92914036	8.51E-25	0.378	0.431	-0.054	*FLJ42709*	0.785	-0.003
cg15705930	5	123973317	9.43E-25	0.782	0.728	0.054	*ZNF608*	2.36E-07	0.040
cg14465207	2	10951250	1.07E-24	0.558	0.508	0.050	*PDIA6*	4.70E-04	0.026
cg00412851	6	15463343	1.24E-24	0.749	0.693	0.056	*JARID2*	1.53E-11	0.054
cg12905767	20	262139	1.34E-24	0.448	0.502	-0.054	*C20orf96*	0.002	-0.025
cg16077493	6	135172385	1.42E-24	0.803	0.753	0.050	NA	8.19E-13	0.054
cg01447828	19	40919465	1.58E-24	0.460	0.511	-0.051	*PRX*	7.28E-05	-0.029
cg08189186	19	38793546	1.64E-24	0.569	0.624	-0.055	*C19orf33*	2.78E-05	-0.034
cg02173540	2	240046504	1.78E-24	0.760	0.701	0.059	*HDAC4*	8.51E-12	0.061
**cg06418238**	17	78755442	1.88E-24	0.389	0.452	-0.062	*RPTOR*	0.162	-0.012
cg08605855	7	86825549	2.00E-24	0.833	0.783	0.050	*DMTF1*	6.84E-07	0.038
cg16954236	18	8638786	2.13E-24	0.785	0.731	0.054	*RAB12*	5.57E-11	0.052
cg07053841	6	29012588	2.30E-24	0.697	0.645	0.052	*OR2W1*	3.39E-04	0.029
cg17139861	4	123304626	2.33E-24	0.786	0.735	0.050	*ADAD1*	1.82E-12	0.054
**cg04193820**	17	29297414	2.48E-24	0.360	0.420	-0.059	*RNF135*	0.897	0.001
**cg27258399**	8	38832267	2.51E-24	0.321	0.374	-0.052	*HTRA4*	0.284	-0.008
**cg23061725**	2	202126379	2.51E-24	0.411	0.359	0.052	*CASP8*	0.093	0.011
**cg05618183**	16	31190476	2.61E-24	0.410	0.357	0.053	*FUS*	0.946	-0.001
cg08653292	11	58920029	2.69E-24	0.585	0.514	0.072	*FAM111A*	1.71E-05	0.047
cg07441953	11	78400028	2.85E-24	0.773	0.720	0.052	*ODZ4*	2.45E-08	0.043
cg11951169	1	155470302	2.85E-24	0.697	0.646	0.051	*ASH1L*	1.40E-08	0.042
cg06532546	1	2886654	2.91E-24	0.809	0.759	0.050	NA	0.007	0.021
cg14321777	10	133874809	2.98E-24	0.692	0.633	0.060	NA	6.34E-09	0.052
cg22202670	3	64676361	3.11E-24	0.744	0.690	0.054	*ADAMTS9-AS2*	1.25E-11	0.053
cg16015816	1	209568242	3.22E-24	0.766	0.715	0.051	NA	1.69E-08	0.043
**cg22963979**	7	1858916	3.40E-24	0.356	0.437	-0.081	*MAD1L1*	0.928	0.000
**cg01229787**	15	100048371	3.60E-24	0.687	0.631	0.056	NA	0.077	0.014
cg06023661	6	32164801	3.89E-24	0.338	0.391	-0.054	*NOTCH4*	0.012	-0.019
cg27338353	22	46471129	4.11E-24	0.421	0.472	-0.051	NA	0.002	-0.022
cg03664391	11	118048212	4.17E-24	0.753	0.702	0.051	*SCN2B*	5.90E-12	0.052
cg07172007	12	26133416	4.19E-24	0.740	0.689	0.051	*RASSF8*	2.71E-11	0.052
cg00418528	6	5463751	4.32E-24	0.793	0.738	0.055	*FARS2*	1.79E-10	0.053
cg00151370	6	16323285	4.40E-24	0.366	0.314	0.051	*ATXN1*	1.34E-04	0.028
cg26074575	3	171764199	4.42E-24	0.709	0.657	0.052	*FNDC3B*	2.93E-09	0.046
**cg19628988**	5	139040928	4.51E-24	0.304	0.356	-0.052	*CXXC5*	0.733	-0.003
**cg08634133**	7	149570071	4.59E-24	0.234	0.285	-0.051	*ATP6V0E2*	0.843	0.001
cg21884374	7	107807954	4.99E-24	0.748	0.690	0.058	*NRCAM*	1.93E-13	0.062

cg04625905	5	160280216	5.10E-24	0.753	0.703	0.050	*ATP10B*	2.28E-10	0.048
cg01533966	1	90363165	5.12E-24	0.564	0.509	0.055	*LRRC8D*	1.10E-08	0.046
cg02001279	19	940967	5.26E-24	0.142	0.203	-0.061	*ARID3A*	2.27E-04	-0.035
cg00547312	15	55473630	5.36E-24	0.739	0.681	0.058	*RSL24D1*	8.66E-12	0.058
cg15177211	7	75567469	5.38E-24	0.409	0.469	-0.060	*POR*	1.17E-05	-0.036
cg14209138	4	28227663	6.05E-24	0.797	0.744	0.053	NA	2.07E-13	0.060
cg07920414	1	41094365	6.15E-24	0.766	0.712	0.054	*RIMS3*	0.007	0.024
cg17792616	15	93580327	6.55E-24	0.621	0.681	-0.059	NA	0.002	-0.025
cg12856392	7	64126140	7.21E-24	0.696	0.638	0.058	*ZNF107*	2.15E-07	0.043
cg13212435	13	50736277	7.53E-24	0.572	0.623	-0.050	NA	4.43E-07	-0.037
cg18814252	1	147075680	7.87E-24	0.765	0.715	0.050	*BCL9*	1.77E-10	0.048
cg19925518	10	90851787	8.04E-24	0.573	0.625	-0.052	NA	0.007	-0.020
cg14588399	8	87242630	8.24E-24	0.549	0.498	0.051	*SLC7A13*	6.29E-07	0.036
cg18143869	3	151072858	8.72E-24	0.819	0.766	0.053	*P2RY12*	1.27E-09	0.049
cg13172001	2	88390175	8.80E-24	0.721	0.668	0.053	*SMYD1*	4.61E-14	0.060
cg14423702	1	116311953	1.00E-23	0.772	0.722	0.051	*CASQ2*	1.87E-09	0.044
cg04420862	3	185412244	1.03E-23	0.774	0.724	0.050	*IGF2BP2*	9.86E-04	0.025
cg16842280	17	10422189	1.05E-23	0.785	0.735	0.050	*MYH1*	2.17E-07	0.039
cg20287234	2	231789465	1.06E-23	0.397	0.454	-0.057	*GPR55*	5.41E-04	-0.029
cg21261158	13	114875412	1.13E-23	0.545	0.495	0.050	*RASA3*	3.90E-04	0.028
cg12601142	12	495079	1.13E-23	0.716	0.665	0.051	*KDM5A*	7.03E-09	0.044
cg17974460	2	202510866	1.20E-23	0.806	0.752	0.054	*MPP4*	1.85E-08	0.046
cg07568296	7	2059150	1.24E-23	0.419	0.473	-0.053	*MAD1L1*	5.09E-04	-0.027
**cg27530576**	6	33401480	1.26E-23	0.269	0.322	-0.052	*SYNGAP1*	0.445	-0.006
cg02269496	13	113105282	1.26E-23	0.691	0.634	0.057	NA	8.43E-10	0.053
cg04319895	6	72955528	1.28E-23	0.674	0.622	0.052	*RIMS1*	2.45E-06	0.037
cg06861395	4	23776092	1.52E-23	0.734	0.684	0.050	NA	3.98E-06	0.035
cg00957886	8	66700638	1.61E-23	0.799	0.745	0.054	*PDE7A*	2.93E-11	0.055
cg07008343	17	38956362	1.64E-23	0.715	0.660	0.055	*KRT28*	1.36E-09	0.049
**cg11564601**	22	30592435	1.68E-23	0.286	0.349	-0.064	NA	0.962	-0.001
**cg00113675**	6	29855347	1.73E-23	0.337	0.415	-0.078	NA	0.943	0.000
cg24466927	4	3315925	2.03E-23	0.748	0.694	0.054	*RGS12*	3.26E-09	0.049
cg17224181	3	42064950	2.10E-23	0.785	0.733	0.052	NA	1.39E-04	0.030
cg13227162	14	101351197	2.12E-23	0.736	0.681	0.055	*RTL1*	4.67E-08	0.046
cg06058209	5	89736766	2.29E-23	0.737	0.687	0.050	NA	1.15E-11	0.052
cg21969190	5	94990682	2.46E-23	0.731	0.679	0.052	*RFESD*	4.08E-12	0.054
cg20077028	12	52208899	2.63E-23	0.449	0.521	-0.072	NA	8.69E-04	-0.033
cg03667488	10	89733191	2.91E-23	0.804	0.753	0.051	NA	3.59E-13	0.055
cg02509204	20	44043235	3.06E-23	0.793	0.734	0.059	*PIGT*	1.06E-10	0.058
cg14831128	17	18780795	3.11E-23	0.757	0.704	0.053	*PRPSAP2*	1.68E-07	0.042
cg12771751	4	128647957	3.32E-23	0.779	0.727	0.052	NA	1.89E-06	0.038
cg10021941	12	8841938	3.32E-23	0.684	0.629	0.056	NA	1.10E-10	0.054
**cg18643199**	10	82363313	3.33E-23	0.614	0.671	-0.057	*SH2D4B*	0.974	0.000
cg10403394	15	63349192	3.47E-23	0.358	0.438	-0.081	*TPM1*	2.70E-05	-0.049
cg27657459	17	54853771	3.91E-23	0.711	0.655	0.056	NA	4.99E-10	0.053
**cg07570687**	10	102243282	3.93E-23	0.277	0.341	-0.064	*WNT8B*	0.052	-0.019
cg02869960	16	28206097	3.96E-23	0.798	0.746	0.051	*XPO6*	3.62E-07	0.039
cg15417641	3	53700141	3.99E-23	0.635	0.698	-0.063	*CACNA1D*	1.96E-04	-0.033
cg15226514	3	63856759	4.08E-23	0.781	0.730	0.051	*ATXN7*	4.21E-06	0.036
**cg21757281**	4	183795822	4.45E-23	0.423	0.474	-0.051	NA	0.990	-0.001
**cg02852791**	16	710674	4.51E-23	0.357	0.418	-0.061	*WDR90*	0.178	0.011
cg13411554	3	53700276	4.55E-23	0.431	0.529	-0.098	*CACNA1D*	1.00E-04	-0.055
cg01385882	11	120204978	4.55E-23	0.793	0.739	0.054	NA	3.04E-09	0.050
cg07971089	8	144629702	4.62E-23	0.136	0.192	-0.056	NA	2.55E-04	-0.033
cg24144574	7	653445	5.05E-23	0.611	0.669	-0.058	*PRKAR1B*	0.033	-0.019
cg22830091	7	75961684	5.27E-23	0.430	0.489	-0.060	*YWHAG*	0.033	-0.018
cg27154651	6	30796209	5.42E-23	0.571	0.642	-0.071	NA	1.44E-08	-0.060
cg05204104	2	235403141	5.79E-23	0.522	0.462	0.059	*ARL4C*	0.002	0.027
cg07452312	5	153775466	5.90E-23	0.336	0.392	-0.056	*GALNT10*	0.002	-0.026
**cg08136809**	19	41882642	6.71E-23	0.240	0.290	-0.050	*TMEM91*	0.501	-0.005
cg23326607	11	330945	7.09E-23	0.704	0.645	0.060	NA	0.025	0.022
cg00336149	3	53700195	7.13E-23	0.348	0.399	-0.051	*CACNA1D*	8.07E-04	-0.025
cg16660091	16	56995856	7.28E-23	0.485	0.554	-0.069	*CETP*	0.002	-0.032
cg02964555	2	157184401	7.46E-23	0.444	0.497	-0.053	*NR4A2*	3.58E-05	-0.033
cg05342835	1	33160791	7.58E-23	0.368	0.418	-0.051	*SYNC*	6.82E-05	-0.028
cg09105249	8	126366660	7.92E-23	0.817	0.765	0.052	*NSMCE2*	9.56E-09	0.047
cg21572480	1	53161198	7.99E-23	0.735	0.684	0.050	*SELRC1*	2.79E-10	0.049
cg03163459	5	76373719	8.36E-23	0.286	0.344	-0.058	*ZBED3*	0.015	-0.023
cg19696491	15	78857124	9.59E-23	0.478	0.538	-0.060	*CHRNA5*	2.52E-06	-0.042
cg19889856	7	134856654	9.81E-23	0.585	0.637	-0.053	*C7orf49*	3.26E-04	-0.028
cg10230190	1	85405081	9.97E-23	0.457	0.507	-0.050	*MCOLN2*	0.013	-0.019
cg18306106	10	13350494	1.03E-22	0.791	0.741	0.050	NA	8.05E-09	0.043
cg21470600	1	6040824	1.05E-22	0.418	0.469	-0.051	*NPHP4*	0.010	-0.018

**cg08324090**	17	7253189	1.12E-22	0.545	0.600	-0.054	*ACAP1*	0.219	-0.010
cg27514550	18	23971201	1.12E-22	0.722	0.670	0.052	*TAF4B*	2.88E-12	0.056
cg18419358	6	158384009	1.14E-22	0.449	0.512	-0.064	NA	3.00E-10	-0.061
cg08016092	12	52979607	1.15E-22	0.822	0.769	0.052	*KRT72*	5.86E-04	0.028
cg18260919	3	135869103	1.35E-22	0.769	0.711	0.058	*MSL2*	2.09E-08	0.050
cg11446398	5	77624930	1.47E-22	0.784	0.733	0.051	NA	1.35E-05	0.035
cg07481360	3	196902551	1.54E-22	0.727	0.676	0.051	*DLG1*	1.06E-08	0.044
cg13676759	5	61844855	1.61E-22	0.813	0.756	0.058	*IPO11*	1.23E-05	0.043
cg09160589	16	11654880	1.65E-22	0.527	0.475	0.052	*LITAF*	2.13E-05	0.032
**cg19856897**	7	142495846	1.66E-22	0.313	0.252	0.061	NA	0.240	0.010
cg21188533	3	53700263	1.75E-22	0.574	0.652	-0.078	*CACNA1D*	6.49E-04	-0.038
cg17056747	2	215045	1.79E-22	0.765	0.714	0.051	NA	1.63E-08	0.044
**cg10321156**	11	63687223	1.84E-22	0.341	0.394	-0.053	NA	0.289	-0.008
cg19109457	6	30460484	1.89E-22	0.566	0.514	0.052	*HLA-E*	2.59E-05	0.032
cg27009815	10	69988445	1.90E-22	0.793	0.742	0.052	NA	1.15E-07	0.041
cg15174906	6	156919703	2.10E-22	0.443	0.494	-0.051	NA	0.003	-0.023
cg03148503	18	10588980	2.14E-22	0.781	0.730	0.050	NA	1.38E-08	0.045
cg17144149	17	46656572	2.24E-22	0.373	0.425	-0.052	*HOXB4*	0.002	-0.024
**cg01347228**	17	29297391	2.27E-22	0.289	0.340	-0.051	*RNF135*	0.518	0.005
cg18477569	6	90951134	2.89E-22	0.388	0.454	-0.066	*BACH2*	0.025	-0.020
cg25885914	12	20906250	2.89E-22	0.779	0.729	0.051	*SLCO1C1*	1.01E-10	0.052
cg21758126	2	157183291	3.04E-22	0.548	0.605	-0.057	*NR4A2*	1.49E-07	-0.044
cg07201475	2	151768348	3.14E-22	0.416	0.471	-0.054	NA	0.002	-0.024
**cg27395066**	17	43221220	3.25E-22	0.293	0.350	-0.057	*ACBD4*	0.509	0.004
cg15112923	1	173155033	3.36E-22	0.756	0.704	0.052	*TNFSF4*	2.02E-12	0.057
cg26888227	10	45697308	3.37E-22	0.552	0.494	0.058	NA	4.72E-11	0.058
cg19947357	14	99864952	3.43E-22	0.658	0.603	0.054	*SETD3*	8.88E-09	0.050
cg00315837	11	59244789	3.71E-22	0.779	0.727	0.052	*OR4D10*	2.90E-09	0.047
cg11846968	20	31823545	3.84E-22	0.499	0.552	-0.053	*BPIFA1*	0.008	-0.020
cg21037057	14	57464939	3.96E-22	0.773	0.717	0.056	NA	8.77E-08	0.048
cg09627207	7	131244901	4.07E-22	0.773	0.722	0.051	NA	2.48E-08	0.043
cg22964146	10	74767376	4.55E-22	0.794	0.740	0.054	*P4HA1*	8.60E-09	0.048
cg06882842	1	227754452	4.56E-22	0.774	0.724	0.050	*ZNF678*	1.59E-11	0.053
cg01409498	19	41429786	4.61E-22	0.757	0.707	0.050	NA	1.19E-10	0.052
cg05822532	7	73442531	4.90E-22	0.218	0.270	-0.052	*ELN*	1.80E-04	-0.032
cg14501839	7	116137036	4.94E-22	0.742	0.678	0.063	NA	9.35E-04	0.033
cg15721134	10	133806677	5.55E-22	0.481	0.532	-0.051	NA	0.005	-0.022
cg13526488	17	78793807	5.86E-22	0.552	0.603	-0.051	*RPTOR*	0.045	-0.016
**cg12317815**	17	3379283	6.35E-22	0.564	0.513	0.051	*ASPA*	0.521	0.006
**cg07902156**	5	1495284	6.71E-22	0.298	0.350	-0.052	*LPCAT1*	0.365	-0.008
cg07793807	1	226823363	6.74E-22	0.517	0.464	0.053	*ITPKB*	0.039	0.016
cg05320533	16	89541815	6.96E-22	0.705	0.652	0.054	*ANKRD11*	1.91E-07	0.044
cg15845673	12	25099560	7.83E-22	0.422	0.474	-0.052	*BCAT1*	0.043	-0.018
cg24605288	7	84628407	7.85E-22	0.784	0.733	0.051	*SEMA3D*	2.36E-06	0.039
cg11537619	8	82359759	8.15E-22	0.764	0.714	0.051	*PMP2*	1.79E-07	0.042
cg10469112	3	131388542	8.78E-22	0.787	0.736	0.052	*CPNE4*	1.67E-11	0.054
cg19966146	6	49466300	9.84E-22	0.752	0.702	0.050	*GLYATL3*	6.05E-11	0.053
cg18267330	2	99095277	1.03E-21	0.747	0.693	0.054	*INPP4A*	4.55E-09	0.053
cg21399203	4	57623913	1.06E-21	0.487	0.429	0.058	NA	0.015	0.022
cg23527183	10	95253833	1.06E-21	0.758	0.708	0.050	NA	4.39E-07	0.042
**cg14055835**	19	55384515	1.16E-21	0.697	0.646	0.051	*FCAR*	0.404	0.008
cg22036538	12	6554051	1.19E-21	0.457	0.396	0.061	*CD27*	0.011	0.024
**cg13916261**	9	132800969	1.24E-21	0.691	0.624	0.067	*FNBP1*	0.451	0.009
cg03483626	1	111218276	1.26E-21	0.548	0.608	-0.060	*KCNA3*	2.77E-05	-0.038
cg05194346	15	101305443	1.28E-21	0.544	0.594	-0.051	NA	2.90E-11	-0.048
**cg04723534**	21	43373136	1.30E-21	0.386	0.439	-0.054	*C2CD2*	0.377	-0.008
**cg18367631**	7	653309	1.35E-21	0.470	0.550	-0.080	*PRKAR1B*	0.278	-0.013
cg00710736	3	125000990	1.73E-21	0.785	0.732	0.053	*ZNF148*	2.24E-07	0.044
cg19321615	11	82972903	1.73E-21	0.746	0.694	0.051	*CCDC90B*	1.11E-07	0.045
cg04733681	6	160423822	1.73E-21	0.787	0.734	0.053	*IGF2R*	2.80E-10	0.054
cg03535830	1	110934327	1.93E-21	0.774	0.723	0.051	*SLC16A4*	4.11E-12	0.057
cg13093111	17	66308577	2.10E-21	0.631	0.688	-0.057	*ARSG*	0.001	-0.028
**cg00730561**	10	102279703	2.28E-21	0.462	0.514	-0.053	*SEC31B*	0.583	-0.005
cg03448915	16	66583078	2.40E-21	0.726	0.667	0.059	*TK2*	1.24E-04	0.036
cg21921829	7	116850153	2.95E-21	0.741	0.688	0.053	*ST7*	9.57E-09	0.047
cg23001084	15	63322611	3.35E-21	0.689	0.639	0.051	NA	7.40E-06	0.034
cg01864699	6	122789987	3.38E-21	0.813	0.762	0.052	*SERINC1*	5.40E-09	0.048
cg11796910	2	11475299	4.07E-21	0.725	0.673	0.052	*ROCK2*	1.30E-10	0.055
cg06689619	4	99935464	4.22E-21	0.754	0.697	0.057	*METAP1*	6.57E-04	0.031
cg24857560	10	96521152	4.29E-21	0.746	0.693	0.053	*CYP2C19*	2.46E-13	0.064
cg11661375	5	17275997	4.44E-21	0.741	0.685	0.056	*BASP1*	3.27E-08	0.052
cg00845742	19	52453199	5.06E-21	0.801	0.750	0.051	NA	1.66E-13	0.059
**cg06963709**	7	155150793	5.07E-21	0.590	0.660	-0.071	NA	0.293	-0.011

cg02863135	2	101740862	5.17E-21	0.807	0.757	0.050	*TBC1D8*	1.14E-09	0.049
cg25304680	5	14229352	5.49E-21	0.711	0.655	0.056	*TRIO*	1.31E-11	0.061
cg00973876	17	77899208	5.62E-21	0.470	0.522	-0.051	NA	5.90E-08	-0.041
cg06640254	6	111588373	5.67E-21	0.796	0.744	0.052	*KIAA1919*	6.03E-10	0.050
cg17014647	6	16431306	5.71E-21	0.743	0.689	0.054	*ATXN1*	8.62E-07	0.044
cg18007641	4	74641828	5.84E-21	0.574	0.514	0.060	NA	1.27E-07	0.054
cg10591077	7	30030138	6.04E-21	0.689	0.634	0.056	*SCRN1*	1.00E-09	0.055
cg25413977	2	66651619	6.11E-21	0.513	0.566	-0.053	*MEIS1-AS3*	9.71E-11	-0.053
cg20861489	6	55105265	6.60E-21	0.476	0.527	-0.051	*HCRTR2*	4.82E-04	-0.030
cg04940312	11	35688285	7.33E-21	0.788	0.736	0.052	*TRIM44*	3.61E-11	0.058
cg20401567	17	46619555	7.50E-21	0.367	0.419	-0.052	NA	0.001	-0.030
**cg03818715**	6	7591348	9.09E-21	0.476	0.527	-0.051	*SNRNP48*	0.814	-0.002
cg27027668	10	127277604	9.25E-21	0.663	0.613	0.050	NA	1.32E-07	0.042
cg27080171	4	46388320	9.28E-21	0.603	0.550	0.053	*GABRA2*	1.01E-08	0.050
cg15065340	3	195632915	9.81E-21	0.551	0.606	-0.055	*TNK2*	8.16E-06	-0.033
**cg26656658**	12	121615078	1.04E-20	0.558	0.609	-0.051	*P2RX7*	0.063	-0.015
cg21243944	9	137118148	1.04E-20	0.438	0.499	-0.061	NA	1.34E-05	-0.039
cg13974313	4	48129869	1.07E-20	0.630	0.580	0.050	*TXK*	0.037	0.017
**cg27120833**	22	43193524	1.24E-20	0.492	0.544	-0.052	*ARFGAP3*	0.725	0.003
cg27130993	5	148545635	1.37E-20	0.547	0.493	0.054	*ABLIM3*	4.01E-04	0.031
**cg12542255**	19	45976195	1.69E-20	0.202	0.258	-0.056	*FOSB*	0.553	-0.005
cg18194887	7	93629937	1.81E-20	0.665	0.612	0.052	*BET1*	1.12E-11	0.057
cg25920214	6	47442665	1.87E-20	0.811	0.759	0.051	NA	9.92E-11	0.053
cg08130292	19	52870301	2.03E-20	0.798	0.748	0.050	*ZNF610*	1.67E-12	0.056
cg16271453	3	142027066	2.30E-20	0.776	0.724	0.052	*XRN1*	3.74E-09	0.048
cg19758134	1	114829082	2.52E-20	0.716	0.662	0.054	NA	1.72E-06	0.042
cg11639615	3	171543413	2.53E-20	0.426	0.479	-0.053	NA	0.012	-0.021
cg09461494	8	100262768	2.56E-20	0.776	0.725	0.051	*VPS13B*	6.85E-12	0.058
cg04327675	6	3342030	2.72E-20	0.755	0.702	0.053	*SLC22A23*	1.04E-08	0.048
cg13375690	8	66706202	3.02E-20	0.759	0.708	0.052	*PDE7A*	1.06E-08	0.051
cg23146699	3	66020673	3.14E-20	0.753	0.700	0.053	*MAGI1*	0.001	0.028
cg26924822	4	26332763	3.16E-20	0.478	0.533	-0.055	*RBPJ*	0.043	-0.017
cg21700214	18	72252164	3.17E-20	0.373	0.427	-0.054	*CNDP1*	0.001	-0.026
cg04365696	13	43834391	3.89E-20	0.762	0.710	0.052	*ENOX1*	4.76E-07	0.043
cg01966791	20	62572875	3.99E-20	0.292	0.355	-0.063	*UCKL1*	0.048	-0.021
cg09408143	16	524778	4.66E-20	0.447	0.505	-0.058	*RAB11FIP3*	0.011	-0.022
cg17502614	20	13977717	5.03E-20	0.711	0.661	0.051	*MACROD2*	1.48E-05	0.036
cg16031515	1	205743344	6.09E-20	0.690	0.637	0.052	*RAB7L1*	4.28E-07	0.042
cg04572258	2	224402829	6.67E-20	0.693	0.640	0.054	NA	0.016	0.021
**cg27366964**	10	77946906	6.79E-20	0.761	0.698	0.062	*C10orf11*	0.154	0.015
**cg09241703**	14	89831698	7.00E-20	0.490	0.439	0.051	*FOXN3*	0.128	0.011
cg23523480	5	153204284	7.16E-20	0.709	0.658	0.051	NA	2.35E-05	0.036
cg21104965	6	117869453	7.64E-20	0.517	0.450	0.067	*DCBLD1*	9.91E-07	0.052
cg14830002	1	247616686	8.02E-20	0.290	0.340	-0.050	*OR2B11*	4.30E-05	-0.034
cg17368726	7	108169094	8.04E-20	0.779	0.728	0.051	*PNPLA8*	4.33E-07	0.043
cg05584692	7	33925755	8.21E-20	0.730	0.679	0.051	NA	4.57E-04	0.030
cg26102434	3	184975890	8.56E-20	0.761	0.708	0.053	NA	4.50E-06	0.041
cg22094163	2	86703710	9.79E-20	0.598	0.545	0.053	*KDM3A*	4.65E-06	0.040
cg25814293	7	1883251	1.08E-19	0.675	0.732	-0.057	*MAD1L1*	2.89E-04	-0.031
cg05088386	4	140060774	1.12E-19	0.750	0.698	0.053	*ELF2*	2.35E-10	0.055
cg02888513	15	39205040	1.18E-19	0.792	0.742	0.051	NA	5.40E-10	0.050
cg26189283	1	155109378	1.24E-19	0.445	0.496	-0.051	*SLC50A1*	1.08E-05	-0.036
**cg19841369**	14	64663928	1.27E-19	0.243	0.296	-0.053	*SYNE2*	0.205	-0.011
**cg08123444**	2	9833101	1.37E-19	0.697	0.646	0.051	NA	0.204	0.009
cg19851526	4	130257063	1.46E-19	0.739	0.689	0.050	NA	6.59E-07	0.041
**cg25403205**	6	31584215	1.54E-19	0.230	0.300	-0.071	*AIF1*	0.674	-0.006
cg19478698	3	195530151	1.73E-19	0.574	0.624	-0.050	*MUC4*	0.004	-0.024
cg14020285	2	119401665	1.81E-19	0.614	0.562	0.052	NA	3.64E-06	0.039
cg08343347	4	36076000	1.81E-19	0.748	0.695	0.053	*ARAP2*	1.81E-08	0.050
cg22299467	19	58399865	1.85E-19	0.227	0.174	0.053	*ZNF814*	4.55E-05	0.030
**cg09926364**	14	73137242	2.26E-19	0.341	0.395	-0.054	*DPF3*	0.446	-0.008
cg01303420	3	157296174	2.68E-19	0.703	0.650	0.053	*C3orf55*	2.16E-11	0.060
cg17114584	11	613792	2.75E-19	0.535	0.603	-0.068	*IRF7*	2.97E-10	-0.050
cg20739864	15	78841455	3.09E-19	0.794	0.744	0.050	*PSMA4*	1.79E-08	0.047
cg02471078	11	688159	3.12E-19	0.674	0.620	0.054	*DEAF1*	2.03E-15	0.075
**cg26266708**	15	76630962	3.20E-19	0.313	0.365	-0.052	*ISL2*	0.199	0.011
cg11303839	7	75405967	3.28E-19	0.414	0.361	0.052	*CCL26*	0.006	0.023
**cg21667061**	15	60772094	3.38E-19	0.639	0.584	0.056	*NARG2*	0.101	0.014
cg12178432	1	26049105	4.00E-19	0.674	0.619	0.056	*MAN1C1*	7.90E-10	0.059
cg06209897	10	53635551	4.17E-19	0.387	0.445	-0.059	*PRKG1*	0.025	-0.021
cg01293346	12	11149362	4.42E-19	0.666	0.614	0.052	*PRH1-PRR4*	2.07E-10	0.057
cg14703454	3	108065259	4.50E-19	0.437	0.511	-0.074	*HHLA2*	0.015	-0.027
cg15908709	17	46676215	4.56E-19	0.379	0.431	-0.052	*HOXB-AS3*	1.11E-08	-0.045

cg05373263	2	3063115	5.13E-19	0.725	0.664	0.061	NA	2.27E-07	0.052
cg01725383	21	36259797	5.36E-19	0.299	0.354	-0.055	*RUNX1*	3.21E-05	-0.040
cg18030799	7	127429445	5.82E-19	0.752	0.700	0.051	*SND1*	5.83E-11	0.058
cg08818207	6	32820355	6.31E-19	0.454	0.506	-0.052	*TAP1*	6.34E-14	-0.060
cg17217920	17	33957395	6.86E-19	0.752	0.701	0.051	*AP2B1*	1.72E-08	0.048
cg00404280	8	144631887	7.54E-19	0.505	0.451	0.054	NA	1.01E-05	0.039
cg21790991	3	120137480	8.01E-19	0.504	0.562	-0.058	*FSTL1*	0.002	-0.029
cg10818896	6	32263335	8.69E-19	0.733	0.679	0.054	*C6orf10*	5.36E-09	0.054
cg09976051	4	178362394	9.83E-19	0.286	0.342	-0.055	*AGA*	0.003	-0.030
cg22582187	10	63394414	1.08E-18	0.592	0.539	0.053	NA	3.41E-09	0.051
cg25659893	4	24043352	1.23E-18	0.769	0.715	0.054	NA	7.81E-07	0.044
cg14159523	9	90185475	1.44E-18	0.697	0.636	0.061	*DAPK1*	0.002	0.035
**cg11742688**	1	229674241	1.45E-18	0.591	0.649	-0.059	*ABCB10*	0.172	-0.013
cg04068005	6	137162231	1.69E-18	0.748	0.691	0.057	*PEX7*	8.22E-11	0.063
cg16994041	7	151503269	1.70E-18	0.320	0.371	-0.052	*PRKAG2*	6.09E-04	-0.033
**cg27664085**	15	89157815	1.87E-18	0.392	0.449	-0.058	NA	0.378	-0.010
cg14905634	12	73057369	1.91E-18	0.540	0.490	0.051	*TRHDE*	6.95E-05	0.036
cg06082141	17	10604565	2.36E-18	0.768	0.718	0.050	*ADPRM*	4.91E-11	0.057
**cg16382047**	2	231790037	3.28E-18	0.330	0.382	-0.052	*GPR55*	0.174	-0.013
**cg16532282**	1	226271799	3.40E-18	0.360	0.412	-0.052	NA	0.926	-0.001
cg15357156	1	23884373	3.96E-18	0.741	0.684	0.057	NA	3.36E-04	0.038
cg06847429	13	112881733	4.04E-18	0.754	0.703	0.051	NA	9.19E-07	0.042
cg21855021	6	131457506	4.68E-18	0.479	0.530	-0.051	*AKAP7*	1.28E-05	-0.039
**cg11438552**	22	18919803	5.00E-18	0.523	0.467	0.056	*PRODH*	0.308	0.008
**cg03025830**	8	21905599	5.00E-18	0.562	0.630	-0.068	*FGF17*	0.161	0.017
**cg01403030**	9	19026479	5.08E-18	0.513	0.462	0.051	*FAM154A*	0.317	0.008
**cg20187011**	21	28214928	5.33E-18	0.584	0.637	-0.053	*ADAMTS1*	0.461	-0.006
cg03809021	16	89831123	5.42E-18	0.376	0.436	-0.061	*FANCA*	4.00E-07	-0.052
cg25925210	2	219576383	6.07E-18	0.277	0.334	-0.057	*TTLL4*	3.82E-08	-0.056
cg13702370	11	45844932	7.23E-18	0.606	0.549	0.057	NA	0.001	0.034
cg12870014	12	110450643	7.28E-18	0.513	0.589	-0.076	*ANKRD13A*	5.74E-04	-0.046
**cg08154101**	17	9130094	8.20E-18	0.700	0.648	0.053	*NTN1*	0.521	0.006
cg25032124	1	110946480	8.31E-18	0.722	0.671	0.051	*HBXIP*	8.86E-10	0.056
cg18598117	19	941126	8.64E-18	0.167	0.227	-0.060	*ARID3A*	0.002	-0.034
**cg04812347**	6	31584223	1.05E-17	0.194	0.262	-0.068	*AIF1*	0.624	-0.007
**cg09806625**	6	611523	1.19E-17	0.495	0.566	-0.071	*EXOC2*	0.106	-0.020
cg15536947	17	65471303	1.22E-17	0.511	0.459	0.052	*PITPNC1*	0.005	0.023
cg02284273	10	14051679	1.24E-17	0.588	0.530	0.058	*FRMD4A*	4.39E-05	0.039
cg23220897	3	16924709	1.28E-17	0.643	0.705	-0.062	NA	0.024	-0.021
**cg17448192**	3	186718821	1.34E-17	0.654	0.603	0.051	*ST6GAL1*	0.184	0.011
cg23075364	11	57267445	1.35E-17	0.481	0.535	-0.053	*SLC43A1*	0.012	-0.022
cg05929755	1	110663559	1.42E-17	0.460	0.406	0.054	NA	9.71E-04	0.030
cg14940260	6	16217882	1.53E-17	0.381	0.432	-0.051	NA	0.010	-0.023
cg00818106	10	134221503	1.95E-17	0.320	0.381	-0.061	*PWWP2B*	0.011	-0.026
cg07895381	17	42541896	2.56E-17	0.688	0.636	0.053	*GPATCH8*	7.08E-08	0.049
cg11580351	5	95019062	2.83E-17	0.641	0.586	0.056	*SPATA9*	1.19E-04	0.039
cg05323879	17	46642104	3.12E-17	0.401	0.451	-0.050	*HOXB3*	0.007	-0.022
cg11942221	22	29686496	3.15E-17	0.750	0.697	0.053	*EWSR1*	6.44E-07	0.047
**cg13479204**	17	46641708	3.24E-17	0.362	0.423	-0.061	*HOXB3*	0.065	-0.018
cg26963367	15	89157841	4.10E-17	0.572	0.624	-0.053	NA	0.046	-0.019
cg18502618	21	46902264	4.17E-17	0.546	0.597	-0.051	*COL18A1*	0.018	-0.020
cg02431972	16	89023488	4.58E-17	0.650	0.599	0.050	*CBFA2T3*	0.013	0.021
cg01074676	14	60601069	4.61E-17	0.629	0.575	0.054	*PCNXL4*	1.07E-07	0.052
cg19729744	3	194752020	4.77E-17	0.594	0.645	-0.051	NA	4.39E-09	-0.053
cg02061804	17	32453	5.99E-17	0.632	0.696	-0.064	*DOC2B*	0.001	-0.032
cg15591803	1	111733668	6.27E-17	0.439	0.497	-0.058	*DENND2D*	0.001	-0.035
**cg03445151**	2	23516881	7.26E-17	0.607	0.664	-0.057	NA	0.839	-0.003
**cg03549146**	16	69966902	7.81E-17	0.464	0.540	-0.077	*WWP2*	0.063	-0.020
**cg11787160**	12	113515332	7.86E-17	0.561	0.615	-0.054	*DTX1*	0.761	0.002
**cg25385940**	15	99789637	8.19E-17	0.323	0.387	-0.064	*TTC23*	0.180	-0.012
cg17674726	2	231743193	8.20E-17	0.288	0.345	-0.057	*ITM2C*	0.026	-0.022
cg02755555	6	30981960	8.24E-17	0.764	0.713	0.051	*MUC22*	6.00E-10	0.057
cg08977209	17	80867256	8.30E-17	0.674	0.623	0.050	*TBCD*	2.78E-12	0.065
cg04306507	14	55594613	8.55E-17	0.555	0.621	-0.066	*LGALS3*	0.019	-0.026
cg00978415	1	151345771	9.34E-17	0.691	0.635	0.055	*SELENBP1*	0.002	0.032
**cg08371391**	20	19739935	1.19E-16	0.259	0.319	-0.060	NA	0.638	-0.005
cg19268947	7	77268833	1.25E-16	0.687	0.634	0.054	*PTPN12*	3.05E-09	0.060
cg14187813	2	97651611	1.33E-16	0.667	0.613	0.054	*FAM178B*	5.77E-11	0.064
cg00901138	7	29329370	1.45E-16	0.610	0.555	0.055	*CHN2*	2.78E-04	0.034
**cg17493885**	5	176559558	1.74E-16	0.510	0.563	-0.053	*NSD1*	0.349	0.011
cg01361499	15	75467997	1.87E-16	0.283	0.334	-0.050	NA	5.72E-07	-0.045
**cg01081930**	1	92284280	2.16E-16	0.664	0.610	0.054	*TGFBR3*	0.200	0.013
cg19401033	3	107148372	2.49E-16	0.448	0.500	-0.051	NA	4.76E-04	-0.031

cg01580574	2	134949671	2.50E-16	0.657	0.606	0.050	NA	4.48E-10	0.057
cg18446336	7	2847575	2.73E-16	0.557	0.610	-0.054	*GNA12*	0.050	-0.020
cg22974630	12	54733883	3.14E-16	0.662	0.611	0.051	*COPZ1*	4.57E-09	0.054
cg14711997	7	150652864	4.20E-16	0.445	0.496	-0.051	*KCNH2*	0.022	-0.021
**cg10287786**	11	117666000	4.91E-16	0.516	0.567	-0.051	*DSCAML1*	0.483	-0.007
cg03539765	12	9217390	6.24E-16	0.217	0.269	-0.052	*LINC00612*	0.045	-0.018
cg02453013	16	1297491	7.17E-16	0.536	0.587	-0.051	NA	0.013	-0.022
cg22549408	18	57566300	7.35E-16	0.489	0.540	-0.051	*PMAIP1*	4.27E-05	-0.039
**cg26163234**	15	99789622	7.47E-16	0.196	0.246	-0.050	*TTC23*	0.463	-0.005
cg00864945	1	247291374	8.49E-16	0.198	0.251	-0.054	*ZNF124*	8.50E-04	-0.033
**cg08220243**	22	50499398	1.06E-15	0.642	0.706	-0.064	*MLC1*	0.430	-0.008
**cg15718932**	1	89592725	1.07E-15	0.650	0.593	0.057	*GBP2*	0.660	0.004
**cg19025461**	1	16163610	1.14E-15	0.320	0.373	-0.053	*FLJ37453*	0.218	-0.010
cg26280778	8	496739	1.21E-15	0.763	0.711	0.052	*C8orf42*	2.11E-04	0.038
**cg13731800**	13	101167901	1.28E-15	0.374	0.428	-0.055	*PCCA*	0.435	-0.008
cg26118221	4	8262589	1.41E-15	0.308	0.359	-0.051	NA	4.07E-04	-0.034
cg03821557	17	43023947	1.45E-15	0.777	0.725	0.052	*KIF18B*	0.016	0.025
cg01405107	17	46671635	1.68E-15	0.490	0.541	-0.051	*HOXB-AS3*	1.23E-06	-0.045
**cg15592062**	6	167189543	1.97E-15	0.303	0.356	-0.053	*RPS6KA2*	0.481	-0.007
cg18034501	3	4043162	2.03E-15	0.613	0.561	0.051	NA	5.84E-06	0.045
cg22902505	4	81119473	2.10E-15	0.450	0.500	-0.050	*PRDM8*	0.001	-0.028
cg02586212	1	192544902	2.34E-15	0.552	0.471	0.081	*RGS1*	0.040	0.031
cg05120113	3	33140160	3.76E-15	0.618	0.669	-0.050	*GLB1*	0.006	-0.024
**cg23657179**	10	77165025	6.10E-15	0.549	0.608	-0.059	*ZNF503-AS2*	0.086	-0.019
cg06268875	18	11147385	6.52E-15	0.413	0.354	0.060	*PIEZO2*	0.013	0.025
**cg00106345**	7	27138396	6.75E-15	0.399	0.452	-0.054	*HOTAIRM1*	0.067	-0.018
cg09597767	11	325915	8.36E-15	0.702	0.757	-0.055	NA	0.003	-0.028
**cg00918181**	18	11947875	8.80E-15	0.226	0.278	-0.052	NA	0.527	0.008
cg16523850	6	54156824	8.90E-15	0.553	0.500	0.053	NA	3.74E-06	0.047
**cg15910301**	14	71632612	1.18E-14	0.425	0.491	-0.066	NA	0.082	-0.022
cg03787837	6	32605385	1.22E-14	0.667	0.567	0.100	*HLA-DQA1*	1.17E-05	0.085
cg07156249	6	32822911	1.48E-14	0.269	0.332	-0.063	*PSMB9*	1.22E-13	-0.094
cg08484560	13	113413587	1.88E-14	0.420	0.484	-0.063	*ATP11A*	0.006	-0.035
cg05767404	1	247712591	2.05E-14	0.333	0.392	-0.059	*GCSAML*	7.28E-05	-0.048
cg10578782	5	38426404	2.37E-14	0.574	0.523	0.051	*EGFLAM*	0.008	0.028
cg13297560	15	99320054	2.56E-14	0.593	0.539	0.053	*IGF1R*	0.004	0.032
**cg13941682**	4	702545	4.21E-14	0.559	0.615	-0.056	*PCGF3*	0.057	-0.018
**cg09552548**	2	232538286	4.77E-14	0.432	0.493	-0.061	NA	0.144	-0.017
cg08317738	16	89034292	4.93E-14	0.294	0.239	0.056	*CBFA2T3*	0.002	0.029
cg23815491	16	72088622	5.67E-14	0.453	0.519	-0.066	*HP*	0.004	-0.037
cg08822897	11	64258103	6.35E-14	0.770	0.714	0.056	NA	0.038	0.024
cg20074159	3	110246985	7.22E-14	0.586	0.536	0.050	NA	1.06E-09	0.063
**cg18825221**	14	68749962	7.43E-14	0.563	0.497	0.065	*RAD51B*	0.734	0.005
cg02417360	13	113398673	9.13E-14	0.655	0.601	0.054	*ATP11A*	1.65E-08	0.065
**cg00160872**	2	106567892	1.06E-13	0.689	0.635	0.054	NA	0.884	0.000
cg10604476	19	10403908	1.07E-13	0.550	0.476	0.074	*ICAM5*	5.82E-11	0.094
cg02182476	19	7538084	1.11E-13	0.503	0.567	-0.064	*LOC10012857.*	0.024	-0.028
cg15021280	21	46077582	1.17E-13	0.548	0.497	0.051	*TSPEAR*	0.001	0.032
cg08993878	12	98151379	1.21E-13	0.568	0.645	-0.077	NA	7.81E-05	-0.055
**cg11497017**	12	63117540	1.40E-13	0.769	0.717	0.053	*PPM1H*	0.103	0.019
cg12239580	3	43073058	1.87E-13	0.468	0.524	-0.056	*FAM198A*	3.02E-04	-0.040
cg14521931	5	10832172	2.12E-13	0.753	0.692	0.061	NA	4.41E-04	0.045
**cg04246123**	6	29893273	2.36E-13	0.513	0.567	-0.054	*HCG4B*	0.703	0.004
cg00923880	8	19613178	2.75E-13	0.454	0.542	-0.089	NA	0.017	-0.039
**cg02175321**	11	103930844	3.42E-13	0.493	0.549	-0.055	*PDGFD*	0.421	-0.009
cg01517680	16	49499006	6.08E-13	0.354	0.411	-0.057	NA	0.037	-0.026
**cg26889659**	6	684090	7.16E-13	0.604	0.665	-0.061	*EXOC2*	0.567	-0.007
**cg26246880**	1	158900549	7.64E-13	0.595	0.543	0.052	*PYHIN1*	0.078	0.018
cg05280698	19	37825388	8.57E-13	0.493	0.414	0.079	*HKR1*	1.45E-05	0.075
**cg08479752**	19	54567279	1.02E-12	0.247	0.306	-0.058	*VSTM1*	0.249	-0.017
cg24834889	19	37825406	1.22E-12	0.461	0.399	0.063	*HKR1*	3.01E-06	0.065
cg15542713	1	42385581	1.59E-12	0.581	0.531	0.050	*HIVEP3*	0.013	0.023
**cg02584498**	2	11674057	2.40E-12	0.455	0.516	-0.061	*GREB1*	0.177	-0.015
**cg12078154**	17	78851262	3.65E-12	0.675	0.618	0.057	*RPTOR*	0.625	-0.006
**cg04712122**	14	99655747	4.36E-12	0.718	0.658	0.060	*BCL11B*	0.814	-0.002
**cg18815120**	1	231512676	5.00E-12	0.267	0.317	-0.050	*EGLN1*	0.409	-0.009
cg12024906	19	37825679	5.11E-12	0.397	0.346	0.050	*HKR1*	8.85E-06	0.052
**cg02660097**	11	68866761	6.33E-12	0.469	0.406	0.062	NA	0.059	0.023
**cg27054887**	16	67276940	6.92E-12	0.699	0.636	0.063	*FHOD1*	0.168	0.020
**cg17437088**	7	130698670	7.00E-12	0.755	0.703	0.052	*FLJ43663*	0.145	0.017
**cg13401703**	15	99789777	9.87E-12	0.298	0.352	-0.054	*TTC23*	0.511	-0.004
**cg27207756**	4	1607291	1.22E-11	0.580	0.526	0.054	NA	0.166	0.019
cg15027294	12	1609537	1.74E-11	0.417	0.470	-0.053	NA	0.016	-0.029

cg05825244	20	2730488	2.32E-11	0.539	0.468	0.071	*EBF4*	2.36E-11	0.106
**cg03861379**	5	54128677	2.40E-11	0.737	0.683	0.054	NA	0.073	0.023
cg22157099	2	15830172	2.97E-11	0.617	0.556	0.061	NA	0.024	0.030
cg08802841	12	89748726	3.13E-11	0.427	0.481	-0.054	NA	5.05E-04	-0.043
cg11351709	8	829623	3.64E-11	0.540	0.590	-0.050	NA	1.22E-05	-0.050
**cg04255230**	2	74727010	3.92E-11	0.282	0.340	-0.059	*LBX2*	0.359	0.013
cg03071500	11	325964	4.31E-11	0.522	0.590	-0.068	NA	0.003	-0.044
**cg14832490**	1	20957761	4.33E-11	0.684	0.633	0.052	NA	0.416	0.010
**cg11778734**	2	175439522	4.49E-11	0.766	0.715	0.050	*WIPF1*	0.195	0.016
cg26999154	11	43291043	6.04E-11	0.511	0.456	0.055	NA	1.33E-08	0.068
**cg15296664**	12	89748773	7.71E-11	0.510	0.565	-0.055	NA	0.244	-0.015
**cg00159523**	10	114713187	9.98E-11	0.415	0.468	-0.053	*TCF7L2*	0.745	-0.003
cg20291162	17	40259547	1.22E-10	0.645	0.594	0.051	*DHX58*	5.68E-04	0.037
**cg22307444**	8	672057	1.30E-10	0.388	0.441	-0.053	*ERICH1*	0.702	-0.005
**cg09940355**	15	62929234	1.56E-10	0.750	0.699	0.051	NA	0.701	0.005
cg14345882	6	26364793	1.65E-10	0.326	0.275	0.051	*BTN3A2*	2.10E-05	0.050
cg15234627	13	33837178	1.86E-10	0.435	0.497	-0.062	*STARD13*	0.003	-0.042
cg12948621	19	37825446	2.20E-10	0.373	0.322	0.051	*HKR1*	2.81E-09	0.075
cg07180897	6	32729130	2.66E-10	0.739	0.811	-0.072	*HLA-DQB2*	8.87E-07	-0.085
cg12414174	12	89748895	3.06E-10	0.587	0.637	-0.050	NA	4.77E-05	-0.049
**cg12894711**	7	43821949	3.57E-10	0.410	0.473	-0.064	*BLVRA*	0.059	-0.024
**cg24833027**	8	1897969	3.81E-10	0.544	0.606	-0.062	*ARHGEF10*	0.330	-0.014
cg20130571	3	35974541	4.14E-10	0.357	0.411	-0.054	NA	0.017	-0.033
**cg19264028**	1	162630153	4.86E-10	0.695	0.640	0.054	*DDR2*	0.760	0.006
cg26964592	6	32904621	5.01E-10	0.453	0.531	-0.079	*HLA-DMB*	0.001	-0.058
**cg23097878**	11	45879730	6.61E-10	0.554	0.607	-0.053	*CRY2*	0.059	-0.023
**cg23502204**	11	87905295	1.02E-09	0.573	0.623	-0.051	*RAB38*	0.123	-0.016
**cg18355337**	19	55549722	1.48E-09	0.504	0.560	-0.055	*GP6*	0.570	-0.006
cg21206147	2	23749087	2.13E-09	0.460	0.403	0.057	*KLHL29*	1.01E-04	0.053
cg27573991	1	247712512	2.37E-09	0.472	0.542	-0.070	*GCSAML*	1.07E-05	-0.078
**cg11420782**	22	36598213	2.77E-09	0.448	0.505	-0.056	*APOL4*	0.376	-0.012
**cg08960815**	7	150264767	2.85E-09	0.475	0.424	0.051	*GIMAP4*	0.632	0.006
cg04337937	13	24008382	3.29E-09	0.614	0.564	0.050	*SACS*	3.86E-05	0.055
cg02771260	11	59836817	4.59E-09	0.586	0.535	0.051	*MS4A3*	2.25E-04	0.048
**cg16994880**	20	56140952	3.10E-08	0.686	0.635	0.051	*PCK1*	0.208	0.019
**cg03680873**	3	148844300	3.48E-08	0.522	0.577	-0.055	NA	0.629	-0.009
**cg02096172**	6	6894163	4.52E-08	0.561	0.614	-0.053	NA	0.770	-0.002
**cg21700663**	15	101093900	6.28E-08	0.450	0.508	-0.059	NA	0.628	-0.004
**cg15411272**	6	29895187	6.99E-08	0.499	0.430	0.069	NA	0.134	0.030
**cg13401893**	6	30039432	1.05E-07	0.250	0.311	-0.061	*RNF39*	0.473	0.012
