# Supplementary material for: Shared and Unique Patterns of DNA Methylation in Systemic Lupus Erythematosus and Primary Sjögren's Syndrome
Source: Front Immunol. 2019 Jul 30;10:1686. doi: 10.3389/fimmu.2019.01686 (PMC6688520; doi:10.3389/fimmu.2019.01686)
Supplement: Supplementary file 2 [file Table_2.pdf]

Supplementary Table S2 *Differentially methylated CpG sites (DMCs; n=590) in the pSS case-ctrl EWAS (p<1.3E-7, | $\Delta\beta$ |>0.05) and their results in the SLE case-ctrl EWAS. DMCs with shared association in the SLE case-ctrl EWAS (p<6.6E-6 and same direction of effect; n=572) are indicated in italics. DMCs uniquely associated with pSS (p>0.05 in the SLE case-ctrl EWAS; n=5) are indicated in bold.*

CpG site	Chromosome	Position	p-value pSS-ctrl	Mean β pSS	Mean β ctrl	Methylation Δβ pSS-ctrl	Gene	p-value SLE-ctrl	Methylation Δβ SLE-ctrl
cg03607951	1	79085586	9.92E-67	0.590	0.415	-0.175	*IFI44L*	2.96E-141	-0.251
cg22862003	21	42797588	7.87E-63	0.698	0.511	-0.187	*MX1*	2.54E-126	-0.270
cg21549285	21	42799141	6.86E-59	0.827	0.568	-0.260	*MX1*	3.46E-139	-0.422
cg05552874	10	91153143	2.71E-56	0.710	0.568	-0.142	*IFIT1*	2.54E-128	-0.249
cg22930808	3	122281881	2.40E-55	0.706	0.516	-0.190	*PARP9*	1.39E-105	-0.271
cg06981309	3	146260954	4.10E-51	0.540	0.392	-0.149	*PLSCR1*	4.93E-157	-0.237
cg05696877	1	79088769	2.85E-50	0.671	0.475	-0.196	*IFI44L*	1.93E-120	-0.263
cg00959259	3	122281975	2.60E-48	0.578	0.409	-0.170	*PARP9*	9.28E-105	-0.229
cg26312951	21	42797847	1.32E-41	0.435	0.294	-0.141	*MX1*	1.34E-82	-0.176
cg01190666	20	62204908	8.81E-41	0.545	0.478	-0.068	*PRIC285*	1.20E-111	-0.102
cg23570810	11	315102	6.14E-38	0.686	0.545	-0.141	*IFITM1*	1.57E-75	-0.196
cg09358725	11	33914088	8.46E-35	0.445	0.380	-0.065	*LMO2*	1.67E-143	-0.101
cg10959651	2	7018020	1.34E-34	0.268	0.193	-0.075	*RSAD2*	2.92E-110	-0.110
cg05455036	1	202828149	1.48E-34	0.402	0.351	-0.051	NA	1.60E-178	-0.094
cg10718056	6	28884599	3.17E-34	0.434	0.380	-0.054	*TRIM27*	1.34E-203	-0.113
cg08926253	11	614761	3.84E-34	0.597	0.519	-0.077	*IRF7*	6.80E-81	-0.128
cg20098015	22	50971140	2.15E-33	0.490	0.390	-0.100	*ODF3B*	7.09E-96	-0.155
cg03038262	11	315262	2.69E-32	0.573	0.480	-0.093	*IFITM1*	1.55E-50	-0.123
cg01028142	2	7004578	2.83E-32	0.879	0.805	-0.073	*CMPK2*	1.24E-64	-0.152
cg26801613	1	87793510	8.59E-32	0.675	0.613	-0.062	*LMO4*	9.44E-84	-0.076
cg13304609	1	79085162	2.74E-31	0.850	0.785	-0.065	*IFI44L*	4.95E-63	-0.128
cg21331324	20	57583000	8.94E-31	0.372	0.318	-0.055	*CTSZ*	1.34E-104	-0.074
cg23772226	22	29225779	1.06E-30	0.435	0.377	-0.058	NA	3.84E-180	-0.114
cg19739596	11	59824161	1.24E-30	0.469	0.416	-0.053	*MS4A3*	1.53E-184	-0.110
cg24777399	2	109855574	1.99E-30	0.456	0.403	-0.053	*SH3RF3*	2.47E-138	-0.088
cg01971407	11	313624	3.26E-30	0.482	0.411	-0.071	*IFITM1*	3.81E-53	-0.082
cg04858148	4	81117016	7.90E-30	0.333	0.280	-0.053	*PRDM8*	2.08E-140	-0.090
cg18150584	1	23887816	2.70E-29	0.349	0.298	-0.051	NA	9.90E-162	-0.095
cg26164488	2	64440295	2.80E-29	0.382	0.324	-0.058	NA	7.48E-153	-0.105
cg19371652	12	113415883	2.90E-29	0.333	0.266	-0.068	*OAS2*	1.08E-44	-0.069
cg00602811	2	145278564	4.13E-29	0.505	0.432	-0.073	*ZEB2*	1.30E-47	-0.067
cg15994604	11	34676683	5.15E-29	0.391	0.336	-0.055	*EHF*	1.10E-169	-0.104
cg03739609	6	31555016	5.88E-29	0.461	0.406	-0.055	*LST1*	8.24E-171	-0.108
cg14600987	11	1952678	7.31E-29	0.384	0.334	-0.051	*TNNT3*	1.80E-127	-0.085
cg06708720	12	1099075	8.07E-29	0.521	0.466	-0.055	*ERC1*	8.84E-88	-0.065
cg15545247	12	123201372	9.26E-29	0.496	0.440	-0.056	*HCAR3*	3.75E-173	-0.119
cg06298740	1	227125826	1.44E-28	0.421	0.366	-0.055	NA	1.85E-174	-0.109
cg08628635	6	20483859	1.77E-28	0.482	0.428	-0.055	*E2F3*	8.69E-208	-0.129
cg05439368	15	45028098	2.42E-28	0.608	0.538	-0.070	*TRIM69*	1.10E-18	-0.041
cg24448340	1	179921042	2.43E-28	0.414	0.354	-0.060	NA	4.13E-163	-0.113
cg27230882	1	110976749	3.61E-28	0.447	0.394	-0.053	NA	9.12E-184	-0.112
cg16640599	4	119732131	3.84E-28	0.437	0.387	-0.050	*SEC24D*	7.57E-195	-0.112
cg02012974	3	66492992	8.38E-28	0.443	0.387	-0.056	*LRIG1*	7.87E-163	-0.112
cg02656594	16	27412496	9.04E-28	0.465	0.401	-0.064	*IL21R*	2.12E-126	-0.100
cg10152449	7	2444534	9.45E-28	0.388	0.307	-0.081	*CHST12*	3.58E-102	-0.113
cg23387863	15	77472416	1.08E-27	0.800	0.747	-0.052	*PEAK1*	5.39E-28	-0.038
cg24405567	15	70787565	1.87E-27	0.487	0.421	-0.066	NA	1.17E-135	-0.114
cg17980508	1	79085713	2.50E-27	0.356	0.294	-0.061	*IFI44L*	3.77E-179	-0.140
cg21991396	1	247581417	4.22E-27	0.521	0.464	-0.057	*NLRP3*	5.82E-184	-0.125
cg27209729	11	64428925	4.57E-27	0.614	0.535	-0.079	*NRXN2*	9.25E-50	-0.079
cg21249659	12	10324843	5.04E-27	0.446	0.396	-0.050	*OLR1*	2.46E-169	-0.111
cg19351604	8	1870722	1.51E-26	0.534	0.477	-0.057	*ARHGEF10*	1.74E-168	-0.122
cg15373592	6	56405167	1.53E-26	0.381	0.324	-0.057	*RNU6-71*	5.83E-108	-0.085
cg20222562	6	113993995	2.08E-26	0.520	0.455	-0.065	NA	2.80E-154	-0.129
cg05523603	22	50973101	3.78E-26	0.718	0.642	-0.076	NA	4.72E-71	-0.127
cg25823926	12	94153360	4.03E-26	0.321	0.270	-0.051	*CRADD*	4.87E-125	-0.082
cg05642546	7	37298927	4.99E-26	0.448	0.394	-0.054	*ELMO1*	9.78E-182	-0.124
cg20045320	11	319555	5.32E-26	0.553	0.458	-0.095	NA	1.01E-63	-0.128
cg25278941	6	139795527	5.33E-26	0.320	0.270	-0.050	*LOC645434*	1.19E-98	-0.072
cg16452651	21	35016873	5.66E-26	0.480	0.429	-0.052	*ITSN1*	1.78E-191	-0.116
cg06257058	7	99683264	6.89E-26	0.471	0.415	-0.056	NA	1.88E-172	-0.121
cg00450651	5	139485902	1.12E-25	0.296	0.245	-0.051	NA	1.31E-91	-0.068
cg06422467	6	30720484	1.50E-25	0.412	0.360	-0.052	NA	2.19E-137	-0.091
cg16163847	13	52165356	1.58E-25	0.474	0.421	-0.053	*WDFY2*	3.60E-166	-0.112
cg24422316	11	60930346	2.02E-25	0.461	0.410	-0.052	*VPS37C*	2.94E-123	-0.092
cg03574571	19	35820181	2.32E-25	0.461	0.408	-0.052	*CD22*	2.03E-143	-0.096
cg08557970	6	166856094	2.40E-25	0.494	0.441	-0.053	*RPS6KA2*	6.39E-158	-0.110

cg11313468	19	41782183	2.65E-25	0.492	0.438	-0.054	*HNRNPUL1*	6.68E-194	-0.118
cg06311422	6	56406336	2.90E-25	0.384	0.324	-0.060	*RNU6-71*	7.24E-122	-0.096
cg24967811	12	123503709	3.29E-25	0.420	0.369	-0.051	*PITPNM2*	1.24E-185	-0.119
cg08764162	10	31147088	4.62E-25	0.489	0.431	-0.058	*ZNF438*	6.46E-177	-0.124
cg04730794	5	169144438	7.06E-25	0.550	0.498	-0.052	*DOCK2*	2.27E-200	-0.136
cg04951822	12	113345598	7.42E-25	0.424	0.337	-0.087	*OAS1*	1.19E-29	-0.061
cg13413719	6	3592887	8.32E-25	0.342	0.282	-0.060	NA	1.17E-104	-0.088
cg08356637	11	8385767	1.01E-24	0.395	0.339	-0.056	NA	4.71E-88	-0.079
cg09376835	8	131347294	2.18E-24	0.393	0.339	-0.054	*ASAP1*	4.61E-139	-0.096
cg16967583	2	241807859	2.32E-24	0.353	0.302	-0.051	*AGXT*	1.35E-114	-0.086
cg21171320	9	73178360	2.85E-24	0.365	0.311	-0.054	*TRPM3*	2.03E-94	-0.078
cg15058645	2	175528343	2.94E-24	0.408	0.354	-0.054	*WIPF1*	2.58E-153	-0.106
cg22107533	15	45028083	4.10E-24	0.496	0.436	-0.060	*TRIM69*	2.84E-17	-0.037
cg08106973	1	40399833	4.33E-24	0.390	0.336	-0.054	NA	1.94E-139	-0.103
cg21964466	5	39526937	5.26E-24	0.383	0.330	-0.054	NA	2.36E-112	-0.085
cg00855901	1	79085765	7.45E-24	0.275	0.217	-0.057	*IFI44L*	1.00E-135	-0.108
cg24760467	10	102760784	9.33E-24	0.580	0.522	-0.058	*LZTS2*	8.00E-123	-0.105
cg07033722	1	40539032	1.94E-23	0.499	0.447	-0.052	*PPT1*	1.00E-174	-0.125
cg12510708	7	26193805	2.00E-23	0.337	0.274	-0.063	*NFE2L3*	1.77E-85	-0.085
cg00201133	10	35740216	2.13E-23	0.380	0.328	-0.052	*CCNY*	1.55E-118	-0.094
cg04112866	2	105925965	2.15E-23	0.429	0.371	-0.058	*TGFBRAP1*	4.64E-130	-0.104
cg10549986	2	7018153	2.40E-23	0.165	0.105	-0.060	*RSAD2*	1.41E-82	-0.081
cg01119452	7	37287850	2.95E-23	0.418	0.359	-0.059	*ELMO1*	8.64E-137	-0.110
cg02856190	11	94282491	3.38E-23	0.424	0.374	-0.051	*FUT4*	2.91E-161	-0.107
cg03568673	13	20796216	3.55E-23	0.503	0.451	-0.052	*GJB6*	1.78E-176	-0.125
cg02297838	13	92002454	4.86E-23	0.297	0.245	-0.052	*MIR17HG*	2.66E-76	-0.067
cg04084354	1	56721795	5.36E-23	0.343	0.288	-0.055	NA	3.09E-109	-0.086
cg02052762	17	4090525	5.78E-23	0.534	0.479	-0.055	*ANKFY1*	2.75E-172	-0.136
cg17990365	11	319718	5.92E-23	0.614	0.531	-0.083	*IFITM3*	9.48E-56	-0.110
cg00913954	1	36852956	6.43E-23	0.535	0.478	-0.056	*STK40*	9.63E-121	-0.100
cg09704136	7	143082002	7.43E-23	0.384	0.332	-0.052	*ZYX*	2.10E-109	-0.091
cg14180511	1	206946187	8.40E-23	0.419	0.363	-0.056	*IL10*	2.72E-103	-0.092
cg21519701	17	62252524	9.75E-23	0.435	0.376	-0.059	*TEX2*	1.42E-155	-0.123
cg06857116	17	15885326	9.88E-23	0.518	0.464	-0.054	*ZSWIM7*	1.03E-125	-0.099
cg26404422	11	128367010	1.49E-22	0.627	0.679	0.052	*ETS1*	8.49E-87	0.073
cg12306086	4	106117747	1.78E-22	0.409	0.353	-0.056	*TET2*	1.49E-145	-0.108
cg10552523	11	313478	4.66E-22	0.387	0.317	-0.069	*IFITM1*	3.28E-34	-0.070
cg07488141	7	47560215	4.76E-22	0.442	0.387	-0.055	*TNS3*	3.91E-136	-0.104
cg25140783	1	24861872	5.55E-22	0.560	0.618	0.058	*RCAN3*	8.02E-148	0.114
cg14102437	2	33359688	5.71E-22	0.402	0.346	-0.057	*LTBP1*	9.20E-123	-0.102
cg10501210	1	207997020	8.16E-22	0.638	0.553	-0.085	NA	9.37E-12	-0.040
cg26106166	10	16821670	1.32E-21	0.606	0.666	0.060	*RSU1*	9.06E-96	0.097
cg11638399	8	29441416	1.74E-21	0.443	0.392	-0.051	NA	6.60E-152	-0.114
cg03626208	12	2443169	2.07E-21	0.425	0.371	-0.054	*CACNA1C*	9.85E-139	-0.104
cg25363080	5	81452987	3.02E-21	0.344	0.293	-0.052	*ATG10*	1.74E-93	-0.076
cg13870520	4	77131705	3.81E-21	0.479	0.427	-0.052	*SCARB2*	6.39E-157	-0.109
cg01087254	15	57052190	8.56E-21	0.412	0.357	-0.055	NA	6.82E-150	-0.118
cg08099136	6	32811251	9.96E-21	0.398	0.328	-0.070	*PSMB8*	1.19E-32	-0.061
cg12110801	19	5992284	9.99E-21	0.497	0.446	-0.051	*LOC1001285*	2.21E-143	-0.106
cg00573770	2	145278485	1.13E-20	0.372	0.317	-0.056	*ZEB2*	1.20E-23	-0.043
cg26861460	22	44575455	1.23E-20	0.449	0.397	-0.052	*PARVG*	2.68E-147	-0.111
cg09853238	6	149532290	1.45E-20	0.254	0.202	-0.052	NA	6.86E-82	-0.070
cg09122035	11	319667	1.46E-20	0.492	0.398	-0.094	NA	3.11E-72	-0.129
cg24420366	10	3938541	1.89E-20	0.530	0.479	-0.050	NA	6.17E-177	-0.121
cg05057534	2	28497669	2.31E-20	0.450	0.391	-0.058	*BRE*	6.31E-148	-0.114
cg04846203	1	167690438	2.37E-20	0.332	0.280	-0.052	*MPZL1*	5.93E-124	-0.098
cg15612947	5	14464064	2.47E-20	0.477	0.425	-0.051	*TRIO*	3.95E-83	-0.086
cg11098259	15	58430391	2.52E-20	0.395	0.345	-0.050	*AQP9*	1.14E-151	-0.109
cg06647068	12	104853274	2.70E-20	0.399	0.341	-0.059	*CHST11*	7.46E-34	-0.051
cg18907610	12	11992925	3.63E-20	0.417	0.364	-0.053	*RNU6-19*	7.21E-127	-0.095
cg16411857	16	57023191	4.01E-20	0.265	0.202	-0.063	NA	3.71E-58	-0.075
cg02215171	4	89379156	4.17E-20	0.414	0.361	-0.053	*HERC5*	1.12E-98	-0.098
cg25932290	15	89939252	5.31E-20	0.420	0.369	-0.051	*LOC254559*	5.50E-116	-0.096
cg10636246	1	159046973	7.58E-20	0.368	0.302	-0.066	*AIM2*	1.87E-41	-0.064
cg05894970	3	119041204	7.70E-20	0.318	0.266	-0.053	*ARHGAP31*	3.28E-77	-0.073
cg01787084	16	87371097	1.05E-19	0.420	0.354	-0.066	*FBXO31*	1.43E-126	-0.126
cg26478599	7	41747322	1.06E-19	0.506	0.453	-0.054	*INHBA-AS1*	1.19E-141	-0.113
cg12785694	3	160122168	1.23E-19	0.338	0.282	-0.057	*SMC4*	4.08E-68	-0.076
cg02952913	1	247336686	1.64E-19	0.430	0.371	-0.059	*ZNF124*	5.28E-126	-0.113
cg11377047	1	26881009	1.81E-19	0.384	0.329	-0.055	*RPS6KA1*	1.59E-111	-0.099
cg07493197	17	53573969	1.89E-19	0.352	0.299	-0.052	NA	5.38E-92	-0.088
cg08669096	11	121229765	2.12E-19	0.331	0.280	-0.052	NA	1.08E-101	-0.086
cg22737154	2	64631614	2.13E-19	0.506	0.452	-0.053	NA	2.72E-65	-0.071

cg21190228	2	240132342	2.16E-19	0.415	0.365	-0.050	*HDAC4*	8.99E-147	-0.108
cg08876103	1	39572323	2.26E-19	0.438	0.383	-0.056	*MACF1*	2.76E-47	-0.062
cg07375836	17	35717813	2.53E-19	0.358	0.306	-0.052	*ACACA*	1.92E-132	-0.104
cg16646054	5	150157726	2.76E-19	0.535	0.478	-0.058	*C5orf62*	1.26E-76	-0.086
cg23815853	12	48147034	4.28E-19	0.540	0.488	-0.052	*RAPGEF3*	1.53E-135	-0.117
cg05580141	12	49071788	4.35E-19	0.506	0.454	-0.052	*KANSL2*	1.19E-132	-0.112
cg01234420	22	46453808	4.49E-19	0.529	0.471	-0.058	*LOC150381*	7.68E-58	-0.073
cg26750893	2	38043481	4.54E-19	0.616	0.564	-0.052	NA	4.97E-88	-0.088
cg24217948	18	42261980	4.59E-19	0.720	0.666	-0.054	*SETBP1*	8.48E-20	-0.039
cg11187245	6	31323397	9.69E-19	0.724	0.664	-0.060	*HLA-B*	5.42E-09	-0.030
cg24079727	15	57317980	1.03E-18	0.461	0.410	-0.051	*TCF12*	1.01E-146	-0.113
cg08450017	3	45984838	1.30E-18	0.473	0.534	0.062	*CXCR6*	1.44E-130	0.130
cg14864167	8	66751182	1.31E-18	0.651	0.569	-0.082	*PDE7A*	3.59E-41	-0.138
cg23975251	1	10604446	1.39E-18	0.561	0.615	0.054	*PEX14*	6.82E-65	0.078
cg14499058	8	141129321	1.47E-18	0.417	0.366	-0.051	*TRAPPC9*	1.16E-70	-0.073
cg15407162	6	28192457	2.57E-18	0.382	0.330	-0.052	*ZNF193*	3.17E-90	-0.086
cg14753356	6	30720108	4.69E-18	0.484	0.423	-0.060	NA	1.29E-72	-0.087
cg15414930	6	17985564	5.51E-18	0.659	0.608	-0.051	*KIF13A*	5.50E-33	-0.050
cg16463452	13	29195249	6.11E-18	0.485	0.434	-0.052	NA	5.66E-114	-0.101
cg09101941	5	133775258	6.22E-18	0.381	0.329	-0.052	NA	2.30E-113	-0.096
cg08128734	1	206685423	6.92E-18	0.641	0.573	-0.068	*RASSF5*	3.52E-12	-0.039
cg08243626	10	6442501	7.61E-18	0.416	0.363	-0.053	NA	3.19E-127	-0.107
cg13618516	17	79129078	8.22E-18	0.528	0.476	-0.052	*AATK*	1.86E-144	-0.118
cg15382933	11	33745399	8.95E-18	0.464	0.410	-0.054	*CD59*	5.17E-129	-0.110
cg22848646	1	203171499	1.10E-17	0.467	0.415	-0.052	NA	1.60E-121	-0.105
cg25178683	17	76976267	1.15E-17	0.657	0.594	-0.063	*LGALS3BP*	4.45E-37	-0.076
cg07356722	12	131353328	1.28E-17	0.691	0.749	0.057	NA	1.72E-52	0.069
cg05949397	11	44976423	1.46E-17	0.540	0.490	-0.050	NA	1.29E-130	-0.115
cg15418499	11	112028288	1.87E-17	0.453	0.402	-0.051	*IL18*	2.05E-106	-0.093
cg03240301	7	23387383	1.89E-17	0.360	0.309	-0.051	*IGF2BP3*	2.50E-93	-0.085
cg23318020	13	74805375	2.46E-17	0.662	0.718	0.056	NA	6.92E-53	0.070
cg01309328	6	32811253	3.03E-17	0.414	0.354	-0.059	*PSMB8*	5.04E-26	-0.053
cg23213327	2	7016509	4.04E-17	0.497	0.445	-0.052	*RSAD2*	4.69E-92	-0.102
cg24681307	1	110526191	4.17E-17	0.419	0.367	-0.051	*AHCYL1*	2.02E-115	-0.096
cg16526047	1	949893	5.32E-17	0.674	0.619	-0.054	*ISG15*	3.82E-55	-0.090
cg08998192	6	32805570	5.34E-17	0.477	0.421	-0.056	*TAP2*	4.06E-23	-0.047
cg14870271	17	76976010	5.69E-17	0.405	0.349	-0.056	*LGALS3BP*	1.01E-40	-0.072
cg09014329	17	42431748	5.92E-17	0.421	0.490	0.069	*FAM171A2*	4.59E-14	0.043
cg21380380	7	8011838	7.28E-17	0.630	0.688	0.058	*GLCCI1*	1.84E-56	0.077
cg12836863	13	32889023	9.13E-17	0.529	0.468	-0.061	*BRCA2*	1.58E-93	-0.111
cg09628499	17	56299729	1.06E-16	0.698	0.761	0.063	NA	2.38E-21	0.048
cg04903759	3	167238645	1.37E-16	0.465	0.413	-0.053	*WDR49*	5.60E-139	-0.121
cg07919744	15	52873609	1.65E-16	0.734	0.799	0.066	*FAM214A*	9.11E-19	0.047
cg21821982	3	31547255	2.02E-16	0.457	0.406	-0.050	NA	3.99E-134	-0.116
cg21901395	17	46604235	2.85E-16	0.538	0.488	-0.051	NA	4.93E-42	-0.056
cg02039987	2	157291262	2.87E-16	0.485	0.539	0.054	*GPD2*	5.53E-49	0.068
cg01769341	1	3526110	2.93E-16	0.486	0.435	-0.051	*MEGF6*	3.60E-56	-0.061
cg04940329	17	39093054	3.26E-16	0.406	0.351	-0.054	*KRT23*	2.14E-92	-0.096
cg25050392	6	24933103	3.29E-16	0.690	0.741	0.051	NA	8.30E-75	0.079
cg23352030	20	62198469	3.35E-16	0.702	0.766	0.064	*PRIC285*	1.59E-48	0.086
cg01572694	17	46657555	3.81E-16	0.561	0.505	-0.056	NA	7.40E-79	-0.096
cg13303534	3	171261809	4.41E-16	0.494	0.440	-0.053	NA	8.58E-53	-0.072
cg13304638	17	80834089	5.37E-16	0.565	0.511	-0.054	*TBCD*	4.76E-133	-0.133
cg01827633	2	219610103	5.41E-16	0.403	0.353	-0.051	*TTLL4*	2.58E-127	-0.109
cg16041798	14	64905375	5.56E-16	0.711	0.771	0.061	*MTHFD1*	6.89E-27	0.052
cg23592421	14	105147461	1.10E-15	0.437	0.384	-0.053	NA	1.20E-64	-0.078
cg11317199	9	100850391	1.13E-15	0.591	0.664	0.073	*TRIM14*	1.54E-33	0.087
cg01891736	16	11655159	1.42E-15	0.502	0.554	0.052	*LITAF*	2.08E-33	0.055
cg09642739	8	42771492	1.46E-15	0.744	0.815	0.071	*HOOK3*	3.75E-17	0.049
cg02471078	11	688159	2.03E-15	0.620	0.696	0.075	*DEAF1*	3.12E-19	0.054
cg11693709	15	40542019	2.66E-15	0.499	0.435	-0.064	*PAK6*	3.03E-44	-0.075
cg05721199	3	169629136	2.79E-15	0.700	0.768	0.068	*SAMD7*	6.40E-27	0.061
cg26478401	10	90749920	2.97E-15	0.319	0.266	-0.053	*FAS*	3.60E-51	-0.069
cg01765174	9	100880960	3.01E-15	0.497	0.446	-0.051	*TRIM14*	1.75E-26	-0.048
cg21498062	6	21157865	3.21E-15	0.751	0.802	0.051	*CDKAL1*	8.80E-48	0.063
cg17608381	6	29911550	3.36E-15	0.599	0.482	-0.117	*HLA-A*	4.77E-25	-0.112
cg23615741	10	101297642	3.49E-15	0.436	0.368	-0.068	NA	1.61E-62	-0.107
cg05171937	12	27396765	3.55E-15	0.538	0.487	-0.051	*STK38L*	1.56E-134	-0.120
cg07283015	18	22039857	3.98E-15	0.534	0.481	-0.053	*HRH4*	7.08E-137	-0.120
cg00069309	6	17931997	4.65E-15	0.789	0.841	0.052	*KIF13A*	6.10E-16	0.036
cg03763873	13	43565901	4.93E-15	0.201	0.143	-0.057	*EPSTI1*	3.24E-65	-0.079
cg18700420	5	55286352	4.95E-15	0.728	0.783	0.056	*IL6ST*	5.47E-40	0.062
cg04542977	1	51982669	5.39E-15	0.334	0.284	-0.050	*EPS15*	6.85E-16	-0.033

cg27185510	7	95811907	6.56E-15	0.707	0.763	0.056	*SLC25A13*	4.73E-37	0.062
cg13679804	3	59734950	7.57E-15	0.427	0.376	-0.051	NA	3.11E-109	-0.103
cg12033075	1	9788767	8.28E-15	0.328	0.383	0.055	*PIK3CD*	5.62E-49	0.075
cg17850088	1	150119278	1.03E-14	0.644	0.694	0.050	NA	2.02E-37	0.056
cg03713585	2	175693153	1.07E-14	0.783	0.846	0.063	*CHN1*	4.25E-14	0.042
cg14573759	18	73966755	1.24E-14	0.715	0.775	0.060	NA	7.13E-25	0.051
cg23973972	18	72152075	1.43E-14	0.557	0.615	0.058	NA	3.39E-23	0.049
cg21878650	5	64558623	1.46E-14	0.289	0.237	-0.052	*ADAMTS6*	8.83E-53	-0.070
cg15132282	2	64488961	1.55E-14	0.385	0.334	-0.051	NA	2.72E-105	-0.101
cg25576997	14	56257750	1.57E-14	0.456	0.405	-0.051	*LINC00520*	4.11E-116	-0.114
cg05278074	7	92442568	1.67E-14	0.668	0.721	0.053	*CDK6*	9.88E-44	0.065
cg10599446	3	189073674	1.69E-14	0.694	0.744	0.050	NA	2.89E-46	0.063
cg02796420	2	100016281	1.74E-14	0.762	0.821	0.059	*EIF5B*	7.65E-16	0.044
cg14468508	9	134304802	1.89E-14	0.725	0.784	0.059	*PRRC2B*	9.72E-16	0.041
cg20533754	8	1245698	1.99E-14	0.690	0.743	0.053	*LOC286083*	6.97E-22	0.042
cg01401219	2	136541665	2.08E-14	0.726	0.782	0.056	*UBXN4*	1.58E-23	0.048
cg18154117	1	39649937	2.27E-14	0.663	0.716	0.053	*MACF1*	1.45E-46	0.068
cg21699330	7	26193032	2.34E-14	0.215	0.162	-0.053	*NFE2L3*	3.84E-39	-0.055
cg22454769	2	106015767	2.49E-14	0.469	0.528	0.059	*FHL2*	8.14E-07	0.026
cg24654185	2	99812274	2.56E-14	0.698	0.758	0.061	*MRPL30*	9.06E-21	0.047
cg24362960	3	144064632	2.59E-14	0.746	0.800	0.054	NA	3.77E-23	0.045
cg21654379	3	32864307	3.22E-14	0.712	0.775	0.063	*TRIM71*	6.32E-19	0.049
cg23219064	2	84171179	3.35E-14	0.771	0.822	0.051	NA	1.44E-16	0.036
cg04570265	1	228679629	3.47E-14	0.744	0.795	0.050	*RNF187*	5.29E-32	0.053
cg07891862	8	141774605	3.94E-14	0.756	0.809	0.053	*PTK2*	4.58E-32	0.055
cg20408505	6	29911494	4.29E-14	0.465	0.412	-0.053	*HLA-A*	4.50E-09	-0.029
cg10173124	2	127963653	4.42E-14	0.672	0.730	0.058	*CYP27C1*	2.05E-30	0.058
cg20224780	10	132942362	4.46E-14	0.674	0.732	0.058	*TCERG1L*	4.88E-31	0.059
cg13172001	2	88390175	4.61E-14	0.668	0.728	0.060	*SMYD1*	8.80E-24	0.053
cg16349093	5	90485840	4.65E-14	0.422	0.363	-0.059	NA	1.47E-79	-0.099
cg11742479	1	40026520	4.74E-14	0.667	0.724	0.057	*PABPC4*	3.11E-18	0.041
cg07790947	6	28474513	4.77E-14	0.603	0.655	0.052	*GPX6*	3.11E-44	0.062
cg18655898	7	19745167	4.95E-14	0.746	0.803	0.056	*TWISTNB*	1.31E-16	0.041
cg15082040	4	124299999	5.44E-14	0.634	0.691	0.057	NA	3.91E-37	0.064
cg26032021	14	54804413	6.18E-14	0.723	0.782	0.059	NA	5.40E-21	0.049
cg25325512	6	37142220	6.21E-14	0.409	0.351	-0.058	*PIM1*	5.95E-43	-0.072
cg08818207	6	32820355	6.34E-14	0.506	0.446	-0.060	*TAP1*	6.31E-19	-0.052
cg18568335	14	105792505	6.49E-14	0.484	0.431	-0.052	*PACS2*	5.38E-109	-0.108
cg18040409	22	50865814	8.41E-14	0.774	0.829	0.055	*PPP6R2*	3.66E-21	0.046
cg11594821	6	30458601	8.44E-14	0.377	0.326	-0.051	*HLA-E*	4.53E-06	-0.020
cg17681079	7	27556963	9.54E-14	0.754	0.809	0.055	NA	7.76E-25	0.051
cg22078805	17	42432046	9.87E-14	0.366	0.444	0.077	*FAM171A2*	6.26E-10	0.045
cg00403478	6	33040769	1.09E-13	0.261	0.210	-0.051	*HLA-DPA1*	2.46E-04	-0.016
cg07283217	5	3283037	1.10E-13	0.743	0.800	0.057	NA	2.17E-20	0.047
cg19342782	1	70821806	1.13E-13	0.502	0.448	-0.054	*HHLA3*	3.37E-111	-0.124
cg07156249	6	32822911	1.22E-13	0.332	0.238	-0.094	*PSMB9*	1.48E-14	-0.063
cg06434738	16	54115019	1.26E-13	0.730	0.785	0.054	*FTO*	3.41E-20	0.045
cg19931902	20	207144	1.37E-13	0.686	0.737	0.051	*DEFB129*	4.29E-43	0.063
cg16745596	19	39695801	1.40E-13	0.416	0.360	-0.056	*SYCN*	3.73E-41	-0.063
cg15412313	11	74801987	1.62E-13	0.776	0.827	0.051	*OR2AT4*	1.40E-19	0.042
cg00845742	19	52453199	1.66E-13	0.750	0.810	0.059	NA	5.06E-21	0.051
cg19892525	11	108157498	1.73E-13	0.621	0.686	0.065	*ATM*	5.09E-13	0.042
cg01614723	5	40908778	1.83E-13	0.783	0.834	0.051	*C7*	6.18E-16	0.036
cg08160706	16	11876111	1.85E-13	0.801	0.851	0.050	*ZC3H7A*	5.88E-17	0.039
cg18699337	17	44159144	1.87E-13	0.723	0.781	0.058	*KANSL1*	9.36E-21	0.048
cg21884374	7	107807954	1.93E-13	0.690	0.752	0.062	*NRCAM*	4.99E-24	0.058
cg17752799	7	136700699	1.93E-13	0.748	0.801	0.053	*CHRM2*	5.41E-14	0.036
cg01839452	14	22433489	1.99E-13	0.739	0.790	0.051	NA	1.23E-34	0.058
cg14209138	4	28227663	2.07E-13	0.744	0.804	0.060	NA	6.05E-24	0.053
cg02903983	14	22968031	2.14E-13	0.651	0.706	0.055	NA	1.86E-50	0.074
cg25367249	12	96127072	2.33E-13	0.679	0.735	0.056	*NTN4*	8.21E-23	0.049
cg24857560	10	96521152	2.46E-13	0.693	0.758	0.064	*CYP2C19*	4.29E-21	0.053
cg26246536	8	975053	2.68E-13	0.685	0.747	0.062	NA	1.13E-17	0.048
cg22229470	6	168107104	2.92E-13	0.472	0.399	-0.073	NA	7.16E-05	-0.025
cg21507487	14	71941057	2.93E-13	0.747	0.801	0.054	NA	8.47E-21	0.045
cg21192452	12	49593546	3.31E-13	0.736	0.787	0.051	NA	1.81E-12	0.031
cg24070990	1	228634724	3.33E-13	0.574	0.625	0.051	NA	4.24E-07	0.023
cg15331332	6	29692111	3.36E-13	0.580	0.530	-0.050	*HLA-F*	4.29E-06	-0.023
cg03667488	10	89733191	3.59E-13	0.753	0.808	0.055	NA	2.91E-23	0.051
cg11025609	12	110727072	3.62E-13	0.681	0.734	0.053	*ATP2A2*	1.98E-30	0.055
cg16868784	12	112447736	3.78E-13	0.753	0.806	0.053	*TMEM116*	4.75E-21	0.045
cg03172657	16	89163625	3.79E-13	0.455	0.525	0.071	*ACSF3*	4.56E-34	0.080
cg06545367	7	110731527	3.86E-13	0.747	0.803	0.057	*IMMP2L*	2.52E-28	0.054

cg26192520	8	101960390	3.89E-13	0.626	0.681	0.055	YWHAZ	6.52E-48	0.075
cg03906115	2	33359529	4.33E-13	0.369	0.318	-0.051	LTBP1	1.41E-95	-0.102
cg10550074	6	36304794	4.39E-13	0.595	0.645	0.051	C6orf222	1.17E-36	0.061
cg15799109	15	67418428	5.03E-13	0.786	0.841	0.055	SMAD3	1.29E-18	0.043
cg02372037	12	42865986	5.28E-13	0.774	0.825	0.051	PRICKLE1	4.92E-22	0.045
cg17280172	11	123082796	5.44E-13	0.729	0.787	0.058	NA	2.31E-25	0.056
cg23050705	1	231694386	5.81E-13	0.670	0.721	0.051	TSNAX	5.36E-33	0.056
cg03167648	11	13094027	6.13E-13	0.571	0.629	0.057	NA	1.78E-14	0.040
cg27455918	7	92370191	6.21E-13	0.695	0.752	0.056	CDK6	1.06E-22	0.050
cg20022118	7	8276021	6.23E-13	0.703	0.754	0.051	ICA1	6.78E-32	0.056
cg02508251	2	153508150	6.48E-13	0.647	0.699	0.051	PRPF40A	3.58E-14	0.035
cg26383537	19	41817029	6.61E-13	0.721	0.772	0.051	CCDC97	2.23E-11	0.031
cg05575505	15	101262162	6.87E-13	0.543	0.492	-0.051	NA	7.81E-43	-0.065
cg17162808	20	33266296	6.96E-13	0.707	0.757	0.050	PIGU	3.63E-14	0.036
cg08769212	4	930351	7.38E-13	0.754	0.806	0.053	TMEM175	1.46E-32	0.059
cg16077493	6	135172385	8.19E-13	0.753	0.807	0.054	NA	1.42E-24	0.050
cg06985880	7	108525723	8.42E-13	0.752	0.809	0.057	C7orf66	6.74E-16	0.042
cg02910194	3	173438075	8.73E-13	0.758	0.812	0.054	NLGN1	1.58E-18	0.046
cg23381224	10	89311690	8.82E-13	0.728	0.782	0.054	MINPP1	7.59E-20	0.047
cg19962304	2	170135359	9.01E-13	0.802	0.853	0.050	LRP2	1.02E-17	0.040
cg12437809	1	171153544	1.02E-12	0.624	0.680	0.056	FMO2	2.79E-27	0.058
cg21999071	5	14313270	1.14E-12	0.761	0.811	0.050	TRIO	5.69E-22	0.042
cg12494208	17	57743594	1.19E-12	0.773	0.826	0.053	CLTC	1.87E-22	0.048
cg22602513	1	160429714	1.22E-12	0.689	0.743	0.054	NA	7.58E-33	0.061
cg22880770	2	97166341	1.24E-12	0.603	0.664	0.061	NEURL3	3.26E-13	0.042
cg15664152	7	129008407	1.30E-12	0.265	0.213	-0.052	AHCYL2	2.84E-23	-0.044
cg05194864	12	15100977	1.35E-12	0.618	0.669	0.051	ARHGDIB	7.67E-36	0.065
cg00211174	3	127332098	1.36E-12	0.513	0.462	-0.051	MCM2	2.61E-132	-0.126
cg22939324	10	91128729	1.39E-12	0.703	0.759	0.056	NA	8.13E-25	0.053
cg22790005	6	27657814	1.49E-12	0.779	0.834	0.055	NA	1.50E-21	0.050
cg02083528	1	78280850	1.66E-12	0.777	0.830	0.052	FAM73A	1.64E-17	0.042
cg26068677	11	92282995	1.66E-12	0.694	0.748	0.054	FAT3	2.80E-31	0.058
cg08130292	19	52870301	1.67E-12	0.748	0.804	0.056	ZNF610	2.03E-20	0.050
cg16570846	3	197522465	1.69E-12	0.743	0.795	0.052	LRCH3	1.91E-19	0.048
cg00426518	5	115693708	1.71E-12	0.762	0.815	0.053	NA	1.33E-20	0.046
cg10389812	15	99458162	1.74E-12	0.713	0.764	0.051	IGF1R	1.44E-35	0.060
cg17139861	4	123304626	1.82E-12	0.735	0.790	0.054	ADAD1	2.33E-24	0.050
cg12874219	3	149218938	1.83E-12	0.739	0.791	0.052	TM4SF4	2.37E-23	0.048
cg27177006	17	26635378	1.90E-12	0.693	0.744	0.050	NA	3.41E-10	0.030
cg15112923	1	173155033	2.02E-12	0.704	0.760	0.057	TNFSF4	3.36E-22	0.052
cg26155520	1	943647	2.02E-12	0.612	0.667	0.055	NA	6.25E-13	0.036
cg11333454	17	26609296	2.05E-12	0.733	0.783	0.050	KRT18P55	5.96E-15	0.036
cg00349404	10	106100036	2.16E-12	0.587	0.532	-0.055	NA	1.10E-44	-0.076
cg10208303	1	182959540	2.29E-12	0.761	0.819	0.058	NA	1.83E-16	0.045
cg01399379	12	108298374	2.32E-12	0.749	0.800	0.050	NA	2.38E-16	0.039
cg18677148	17	57712280	2.41E-12	0.652	0.702	0.050	CLTC	4.13E-43	0.068
cg07938480	16	3265228	2.58E-12	0.589	0.639	0.051	NA	7.87E-29	0.057
cg22895198	5	74978020	2.59E-12	0.769	0.821	0.052	POC5	1.81E-19	0.044
**cg21400344**	1	25870172	2.68E-12	0.521	0.465	-0.056	LDLRAP1	0.133	-0.008
cg16414852	4	70626128	2.73E-12	0.465	0.517	0.052	SULT1B1	3.08E-29	0.055
cg12861602	8	99045811	2.74E-12	0.648	0.709	0.060	MATN2	1.73E-13	0.042
cg08977209	17	80867256	2.78E-12	0.623	0.688	0.065	TBCD	8.30E-17	0.050
cg27514550	18	23971201	2.88E-12	0.670	0.726	0.056	TAF4B	1.12E-22	0.052
cg15664462	3	151996146	2.90E-12	0.662	0.714	0.052	MBNL1	6.92E-40	0.064
cg03523434	14	63568606	2.95E-12	0.696	0.746	0.051	KCNH5	9.11E-18	0.040
cg03847230	1	100675407	2.97E-12	0.708	0.759	0.051	DBT	1.80E-16	0.040
cg25634507	8	59413577	3.12E-12	0.567	0.620	0.053	CYP7A1	4.63E-29	0.057
cg21664909	2	1646050	3.15E-12	0.740	0.793	0.053	PXDN	9.51E-23	0.048
cg08166767	1	152671348	3.31E-12	0.653	0.708	0.055	LCE2A	1.14E-29	0.058
cg23371655	17	4225165	3.42E-12	0.707	0.764	0.058	UBE2G1	8.96E-15	0.043
cg01082299	6	31431969	3.66E-12	0.611	0.558	-0.053	HCP5	4.84E-12	-0.038
cg23794135	12	81190974	3.81E-12	0.783	0.836	0.052	NA	2.40E-21	0.046
cg01603234	1	218990709	3.84E-12	0.765	0.816	0.051	NA	3.32E-17	0.040
cg14064774	4	48540281	3.92E-12	0.769	0.823	0.054	FRYL	1.74E-18	0.045
cg16246489	5	134735675	4.02E-12	0.412	0.360	-0.052	H2AFY	2.88E-77	-0.100
cg05657416	6	27105792	4.03E-12	0.739	0.792	0.054	HIST1H4I	1.82E-16	0.043
cg21969190	5	94990682	4.08E-12	0.679	0.733	0.054	RFESD	2.46E-23	0.052
cg03535830	1	110934327	4.11E-12	0.723	0.780	0.057	SLC16A4	1.93E-21	0.051
cg02872426	6	110736772	4.23E-12	0.558	0.464	-0.094	DDO	0.007	-0.023
cg24916055	6	78109388	4.24E-12	0.754	0.807	0.053	NA	1.24E-18	0.045
cg04875128	15	31775895	4.48E-12	0.168	0.225	0.057	OTUD7A	0.003	0.014
cg02357416	22	29668384	4.56E-12	0.728	0.778	0.051	EWSR1	3.32E-22	0.046
cg20838429	2	163100512	4.63E-12	0.691	0.745	0.053	FAP	8.29E-22	0.049

cg24606240	1	205720650	4.80E-12	0.653	0.705	0.052	NUCKS1	9.52E-23	0.048
cg13722419	2	160088036	4.85E-12	0.757	0.808	0.052	TANC1	9.47E-15	0.040
cg00692752	8	123086057	4.96E-12	0.699	0.751	0.053	NA	7.89E-24	0.050
cg17430979	4	6034828	5.07E-12	0.528	0.473	-0.055	JAKMIP1	2.70E-34	-0.068
cg05701478	8	19029020	5.13E-12	0.795	0.846	0.051	NA	1.91E-14	0.037
cg12651154	12	26820570	5.19E-12	0.795	0.846	0.051	ITPR2	7.01E-18	0.042
cg14126601	2	37384708	5.59E-12	0.495	0.435	-0.060	EIF2AK2	1.65E-45	-0.082
cg25865570	10	11046383	5.60E-12	0.745	0.806	0.061	CELF2	9.03E-18	0.047
cg03664391	11	118048212	5.90E-12	0.702	0.754	0.052	SCN2B	4.17E-24	0.051
cg13535736	9	111863775	6.04E-12	0.582	0.633	0.050	TMEM245	6.55E-59	0.080
cg11492723	3	16577697	6.05E-12	0.495	0.443	-0.052	NA	1.86E-37	-0.065
cg12125241	15	85575240	6.35E-12	0.712	0.766	0.054	PDE8A	2.48E-28	0.055
cg14904733	4	151501623	6.69E-12	0.717	0.767	0.051	LRBA	7.38E-19	0.043
cg09461494	8	100262768	6.85E-12	0.725	0.783	0.058	VPS13B	2.56E-20	0.051
cg19772620	8	130861109	6.92E-12	0.642	0.694	0.051	FAM49B	7.64E-12	0.032
cg18030105	11	3396237	7.33E-12	0.684	0.739	0.055	ZNF195	5.37E-06	0.023
cg27646075	11	118048216	7.34E-12	0.715	0.766	0.051	SCN2B	4.62E-27	0.052
cg13906813	6	33040914	7.80E-12	0.289	0.238	-0.051	HLA-DPA1	1.82E-09	-0.029
cg19385799	3	78164711	7.80E-12	0.767	0.818	0.051	NA	1.80E-14	0.038
cg11689744	1	170525537	7.97E-12	0.643	0.696	0.053	NA	2.20E-06	0.024
cg02173540	2	240046504	8.51E-12	0.701	0.762	0.061	HDAC4	1.78E-24	0.059
cg22231620	20	57605092	8.59E-12	0.743	0.796	0.053	SLMO2-ATP5	3.07E-19	0.046
cg07015465	5	167912659	8.60E-12	0.703	0.760	0.057	RARS	1.81E-18	0.048
cg00547312	15	55473630	8.66E-12	0.681	0.739	0.058	RSL24D1	5.36E-24	0.058
cg22145091	11	73133280	8.83E-12	0.744	0.797	0.054	FAM168A	4.18E-22	0.050
cg13031097	6	31322577	8.87E-12	0.389	0.327	-0.062	HLA-B	4.92E-15	-0.049
cg17340170	19	21686146	8.98E-12	0.749	0.800	0.052	NA	2.75E-17	0.041
cg23087877	10	22022646	9.01E-12	0.749	0.806	0.057	MLLT10	4.20E-17	0.047
cg14665062	5	88462828	9.34E-12	0.749	0.800	0.051	NA	1.02E-15	0.039
cg03310038	2	98852704	9.41E-12	0.783	0.834	0.052	VWA3B	2.79E-15	0.039
cg16480008	10	31288991	9.47E-12	0.775	0.827	0.052	ZNF438	4.21E-14	0.039
cg10617091	12	46273255	9.55E-12	0.699	0.753	0.054	ARID2	1.48E-19	0.048
cg22928999	2	48046329	9.99E-12	0.789	0.843	0.054	FBXO11	2.98E-17	0.046
cg02540338	2	217243695	1.01E-11	0.776	0.829	0.052	NA	4.13E-16	0.042
cg04721485	2	128494954	1.01E-11	0.794	0.845	0.051	WDR33	4.80E-17	0.042
**cg25824217**	6	33040535	1.05E-11	0.444	0.391	-0.053	HLA-DPA1	0.479	-0.004
cg06371658	3	10292843	1.08E-11	0.741	0.792	0.051	TATDN2	2.09E-20	0.045
cg12588917	6	29692082	1.09E-11	0.440	0.390	-0.051	HLA-F	8.13E-13	-0.037
cg18194887	7	93629937	1.12E-11	0.612	0.670	0.057	BET1	1.81E-20	0.052
cg06058209	5	89736766	1.15E-11	0.687	0.739	0.052	NA	2.29E-23	0.050
cg21013983	18	56987316	1.20E-11	0.774	0.825	0.050	CPLX4	7.41E-20	0.044
cg22202670	3	64676361	1.25E-11	0.690	0.743	0.053	ADAMTS9-A	3.11E-24	0.054
cg25304680	5	14229352	1.31E-11	0.655	0.716	0.061	TRIO	5.49E-21	0.056
cg16669619	18	52630472	1.36E-11	0.783	0.836	0.053	NA	5.39E-15	0.042
cg15519465	1	8738385	1.36E-11	0.731	0.785	0.055	RERE	1.64E-18	0.047
cg19422253	3	149212391	1.41E-11	0.585	0.643	0.058	TM4SF4	6.21E-11	0.036
cg00412851	6	15463343	1.53E-11	0.693	0.747	0.054	JARID2	1.24E-24	0.056
cg06882842	1	227754452	1.59E-11	0.724	0.777	0.053	ZNF678	4.56E-22	0.050
**cg12899747**	3	25391527	1.63E-11	0.349	0.288	-0.061	NA	0.207	-0.008
cg19935040	17	42432165	1.64E-11	0.546	0.599	0.054	FAM171A2	7.56E-05	0.021
cg10469112	3	131388542	1.67E-11	0.736	0.790	0.054	CPNE4	8.78E-22	0.052
cg17374433	10	119876327	1.67E-11	0.742	0.795	0.053	CASC2	3.94E-18	0.047
cg09363424	13	27829014	1.75E-11	0.773	0.824	0.051	RPL21	1.80E-20	0.047
cg16617349	10	74337803	1.77E-11	0.724	0.775	0.052	MICU1	1.06E-22	0.048
cg02376843	5	172083710	1.98E-11	0.575	0.628	0.053	NEURL1B	2.06E-13	0.038
cg10439456	15	45028270	1.99E-11	0.435	0.384	-0.050	TRIM69	5.18E-08	-0.029
cg20230856	3	176769657	2.12E-11	0.772	0.825	0.053	TBL1XR1	9.15E-16	0.044
cg01303420	3	157296174	2.16E-11	0.650	0.710	0.060	C3orf55	2.68E-19	0.053
cg26884161	1	151303917	2.21E-11	0.761	0.811	0.050	NA	8.73E-22	0.046
cg10288467	3	20371290	2.30E-11	0.766	0.818	0.052	NA	2.57E-17	0.043
cg05825244	20	2730488	2.36E-11	0.468	0.574	0.106	EBF4	2.32E-11	0.071
cg05902527	13	56236197	2.63E-11	0.677	0.733	0.056	NA	6.85E-18	0.048
cg07172007	12	26133416	2.71E-11	0.689	0.741	0.052	RASSF8	4.19E-24	0.051
cg10665848	6	29631447	2.72E-11	0.761	0.815	0.054	MOG	5.05E-17	0.044
cg12867312	7	140454740	2.83E-11	0.750	0.803	0.053	BRAF	4.95E-17	0.044
cg00957886	8	66700638	2.93E-11	0.745	0.800	0.055	PDE7A	1.61E-23	0.054
cg26166145	6	32340208	3.04E-11	0.728	0.788	0.060	C6orf10	1.22E-13	0.045
cg04874074	1	6855140	3.14E-11	0.654	0.709	0.055	CAMTA1	9.10E-18	0.046
cg20481837	4	151501627	3.14E-11	0.769	0.820	0.051	LRBA	5.17E-15	0.040
cg26995035	13	113785830	3.17E-11	0.736	0.787	0.052	F10	2.33E-18	0.044
cg23923934	6	31322914	3.34E-11	0.730	0.680	-0.050	HLA-B	1.92E-11	-0.038
cg09386303	12	48551862	3.35E-11	0.711	0.762	0.051	ASB8	2.38E-19	0.046
cg10508111	12	48515382	3.36E-11	0.711	0.764	0.054	PFKM	9.34E-21	0.049

cg23036613	13	50068486	3.43E-11	0.733	0.784	0.051	*SETDB2*	6.69E-13	0.036
cg13634966	8	27661589	3.44E-11	0.743	0.799	0.055	*ESCO2*	2.12E-18	0.048
cg01873305	1	36931324	3.48E-11	0.669	0.723	0.054	*MRPS15*	1.12E-13	0.042
cg02951695	10	134400351	3.50E-11	0.704	0.755	0.052	*INPP5A*	1.72E-11	0.034
cg04940312	11	35688285	3.61E-11	0.736	0.794	0.058	*TRIM44*	7.33E-21	0.052
cg20438043	7	107601757	3.62E-11	0.674	0.727	0.053	*LAMB1*	2.23E-19	0.048
cg01082910	10	36053547	3.87E-11	0.466	0.519	0.053	NA	4.59E-15	0.042
cg13302154	12	15039432	3.92E-11	0.451	0.401	-0.050	*MGP*	2.94E-31	-0.061
cg03926598	6	26617378	4.17E-11	0.752	0.802	0.050	NA	7.38E-18	0.045
cg26714205	6	49522045	4.29E-11	0.695	0.745	0.050	NA	1.24E-31	0.060
cg26888227	10	45697308	4.72E-11	0.494	0.553	0.058	NA	3.37E-22	0.058
cg24663638	5	118817903	4.83E-11	0.789	0.839	0.050	*HSD17B4*	1.73E-14	0.039
cg13127694	14	104656055	4.85E-11	0.720	0.773	0.053	NA	8.40E-19	0.046
cg06082141	17	10604565	4.91E-11	0.718	0.775	0.057	*ADPRM*	2.36E-18	0.050
cg23378033	12	65672031	5.03E-11	0.459	0.408	-0.051	*MSRB3*	5.36E-71	-0.100
cg17052170	8	144099482	5.36E-11	0.637	0.570	-0.067	*LOC1001336*	1.50E-58	-0.134
cg16833110	11	6942045	5.38E-11	0.722	0.775	0.054	*OR2D3*	4.23E-16	0.045
cg00830121	16	85904858	5.44E-11	0.747	0.799	0.052	NA	1.84E-26	0.057
cg00444581	8	141856401	5.56E-11	0.765	0.819	0.054	*PTK2*	4.85E-26	0.060
cg16954236	18	8638786	5.57E-11	0.731	0.784	0.052	*RAB12*	2.13E-24	0.054
cg23881653	2	84199313	5.71E-11	0.785	0.836	0.050	NA	1.65E-22	0.049
cg03922048	8	64085098	5.74E-11	0.703	0.761	0.058	*YTHDF3*	3.21E-15	0.046
cg14187813	2	97651611	5.77E-11	0.613	0.677	0.064	*FAM178B*	1.33E-16	0.054
cg21386573	1	94219800	5.81E-11	0.564	0.509	-0.054	*BCAR3*	7.95E-35	-0.074
cg10604476	19	10403908	5.82E-11	0.476	0.570	0.094	*ICAM5*	1.07E-13	0.074
cg18030799	7	127429445	5.83E-11	0.700	0.759	0.058	*SND1*	5.82E-19	0.051
cg19966146	6	49466300	6.05E-11	0.702	0.755	0.053	*GLYATL3*	9.84E-22	0.050
cg13661243	2	177372587	6.14E-11	0.726	0.777	0.051	NA	4.94E-23	0.049
cg01369829	16	22029878	6.47E-11	0.724	0.775	0.051	*C16orf52*	7.44E-19	0.047
cg25412594	2	56152003	6.59E-11	0.739	0.790	0.051	*EFEMP1*	1.40E-20	0.046
cg00804078	6	110736941	6.63E-11	0.350	0.285	-0.065	*DDO*	3.97E-12	-0.045
cg06298244	17	39086292	7.19E-11	0.727	0.783	0.056	*KRT23*	2.75E-18	0.050
cg05673752	5	126562449	7.46E-11	0.746	0.804	0.058	NA	2.46E-16	0.047
cg07164639	6	110736958	7.57E-11	0.403	0.343	-0.060	*DDO*	3.25E-07	-0.031
cg26826329	11	8612947	7.88E-11	0.740	0.793	0.053	*STK33*	7.17E-17	0.046
cg04068005	6	137162231	8.22E-11	0.691	0.754	0.063	*PEX7*	1.69E-18	0.057
cg23223755	3	9596849	8.65E-11	0.689	0.740	0.051	*LHFPL4*	1.58E-19	0.048
cg15015070	1	78567723	9.52E-11	0.757	0.808	0.050	*GIPC2*	5.14E-20	0.047
cg17591195	2	205130274	9.65E-11	0.776	0.827	0.050	NA	8.64E-14	0.038
cg12176856	15	85586841	9.70E-11	0.755	0.806	0.051	*PDE8A*	1.03E-13	0.040
cg25413977	2	66651619	9.71E-11	0.566	0.513	-0.053	*MEIS1-AS3*	6.11E-21	-0.053
cg01431340	6	110680085	9.80E-11	0.337	0.278	-0.059	*METTL24*	4.08E-09	-0.035
cg25920214	6	47442665	9.92E-11	0.759	0.813	0.053	NA	1.87E-20	0.051
cg18104920	10	80798089	1.00E-10	0.736	0.788	0.052	*LOC283050*	6.39E-19	0.046
cg25885914	12	20906250	1.01E-10	0.729	0.781	0.052	*SLCO1C1*	2.89E-22	0.051
cg26009944	1	52210504	1.04E-10	0.751	0.804	0.053	*OSBPL9*	5.65E-07	0.027
cg26627687	17	58823592	1.05E-10	0.736	0.787	0.050	*BCAS3*	2.17E-16	0.044
cg02509204	20	44043235	1.06E-10	0.734	0.792	0.058	*PIGT*	3.06E-23	0.059
cg13789938	10	122614164	1.06E-10	0.667	0.719	0.052	*MIR5694*	1.89E-19	0.046
cg10021941	12	8841938	1.10E-10	0.629	0.683	0.054	NA	3.32E-23	0.056
cg01566282	19	53794975	1.10E-10	0.739	0.795	0.055	*BIRC8*	8.27E-15	0.043
cg24024511	7	26676346	1.18E-10	0.775	0.828	0.054	*C7orf71*	2.87E-17	0.046
cg01409498	19	41429786	1.19E-10	0.707	0.759	0.052	NA	4.61E-22	0.050
cg13078567	8	95657036	1.22E-10	0.758	0.810	0.051	*ESRP1*	1.65E-19	0.048
cg11796910	2	11475299	1.30E-10	0.673	0.728	0.055	*ROCK2*	4.07E-21	0.052
cg25187338	11	133229387	1.51E-10	0.741	0.793	0.051	*OPCML*	2.21E-19	0.046
cg01021224	5	35049428	1.53E-10	0.729	0.779	0.050	*PRLR*	2.18E-21	0.047
cg23961757	5	95122534	1.55E-10	0.741	0.795	0.055	*RHOBTB3*	1.35E-18	0.049
cg04221681	10	13337496	1.59E-10	0.756	0.806	0.050	*PHYH*	2.11E-19	0.047
cg19857379	1	25105430	1.79E-10	0.647	0.702	0.055	*CLIC4*	1.84E-32	0.065
cg00418528	6	5463751	1.79E-10	0.738	0.791	0.053	*FARS2*	4.32E-24	0.055
cg17536287	6	80224471	1.86E-10	0.668	0.721	0.052	*LCA5*	3.23E-13	0.039
cg06121514	15	25362188	1.91E-10	0.648	0.700	0.052	*IPW*	1.13E-16	0.045
cg22910295	19	10403862	1.93E-10	0.425	0.475	0.050	*ICAM5*	1.12E-12	0.040
cg09254210	14	61873622	1.96E-10	0.645	0.703	0.058	*PRKCH*	1.67E-29	0.068
cg01293346	12	11149362	2.07E-10	0.614	0.671	0.057	*PRH1-PRR4*	4.42E-19	0.052
cg07943849	7	151201868	2.29E-10	0.688	0.739	0.051	*RHEB*	2.62E-15	0.041
cg05088386	4	140060774	2.35E-10	0.698	0.753	0.055	*ELF2*	1.12E-19	0.053
cg03298704	4	99427937	2.38E-10	0.671	0.723	0.051	*TSPAN5*	6.62E-20	0.049
cg19990483	14	51030485	2.44E-10	0.758	0.810	0.052	*ATL1*	4.01E-17	0.047
cg15729062	6	30579288	2.47E-10	0.764	0.817	0.053	*PPP1R10*	6.33E-18	0.045
cg00083959	15	51243589	2.77E-10	0.779	0.829	0.050	*AP4E1*	1.27E-17	0.043
cg04733681	6	160423822	2.80E-10	0.734	0.788	0.054	*IGF2R*	1.73E-21	0.053

cg18419358	6	158384009	3.00E-10	0.512	0.451	-0.061	NA	1.14E-22	-0.064
cg09321086	11	120941709	3.14E-10	0.667	0.718	0.051	*TBCEL*	6.58E-17	0.045
cg23766254	17	42431859	3.65E-10	0.328	0.383	0.055	*FAM171A2*	9.68E-10	0.036
cg12990174	12	25359932	4.39E-10	0.747	0.798	0.050	*KRAS*	2.49E-15	0.042
cg07368265	1	219375142	4.46E-10	0.630	0.681	0.050	*LYPLAL1*	7.54E-13	0.036
cg01580574	2	134949671	4.48E-10	0.606	0.664	0.057	NA	2.50E-16	0.050
cg27657459	17	54853771	4.99E-10	0.655	0.707	0.053	NA	3.91E-23	0.056
cg21798848	5	32706733	5.13E-10	0.743	0.793	0.050	NA	5.38E-20	0.048
cg22819952	14	71472713	5.24E-10	0.527	0.581	0.054	*PCNX*	1.18E-15	0.045
cg06026331	20	60912101	5.35E-10	0.732	0.787	0.054	*LAMA5*	1.55E-12	0.039
cg02888513	15	39205040	5.40E-10	0.742	0.792	0.050	NA	1.18E-19	0.051
cg17824939	12	88421705	5.84E-10	0.696	0.751	0.055	*C12orf50*	2.60E-16	0.048
cg02755555	6	30981960	6.00E-10	0.713	0.770	0.057	*MUC22*	8.24E-17	0.051
cg06640254	6	111588373	6.03E-10	0.744	0.794	0.050	*KIAA1919*	5.67E-21	0.052
cg20361427	7	7610535	6.44E-10	0.665	0.718	0.053	*MIOS*	1.09E-04	0.022
cg07107453	1	79114976	6.44E-10	0.418	0.363	-0.054	*IFI44*	2.37E-27	-0.068
cg26053876	14	22991735	7.13E-10	0.634	0.690	0.056	NA	2.88E-28	0.065
cg18808777	6	31431503	7.89E-10	0.292	0.229	-0.063	*HCP5*	1.59E-42	-0.089
cg12178432	1	26049105	7.90E-10	0.619	0.678	0.059	*MAN1C1*	4.00E-19	0.056
cg02269496	13	113105282	8.43E-10	0.634	0.687	0.053	NA	1.26E-23	0.057
cg25032124	1	110946480	8.86E-10	0.671	0.727	0.056	*HBXIP*	8.31E-18	0.051
cg06270074	4	153353276	9.62E-10	0.776	0.829	0.053	*FBXW7*	4.15E-12	0.038
cg10591077	7	30030138	1.00E-09	0.634	0.689	0.055	*SCRN1*	6.04E-21	0.056
cg26026821	10	70712079	1.04E-09	0.699	0.750	0.051	NA	1.14E-14	0.044
cg20074159	3	110246985	1.06E-09	0.536	0.599	0.063	NA	7.22E-14	0.050
cg13561372	7	121781778	1.11E-09	0.710	0.760	0.050	*AASS*	5.67E-26	0.057
cg14569423	9	126523663	1.16E-09	0.710	0.760	0.050	*DENND1A*	1.47E-13	0.040
**cg08468401**	3	14303131	1.18E-09	0.342	0.287	-0.054	NA	0.847	-0.001
cg04770282	16	46826414	1.24E-09	0.683	0.733	0.050	NA	5.37E-13	0.038
cg23925154	12	127414940	1.49E-09	0.706	0.761	0.055	NA	8.51E-17	0.050
cg20315690	11	124707157	2.33E-09	0.425	0.374	-0.051	NA	1.79E-07	-0.028
cg12948621	19	37825446	2.81E-09	0.322	0.397	0.075	*HKR1*	2.20E-10	0.051
cg06480496	6	31430675	3.03E-09	0.312	0.258	-0.054	NA	8.66E-09	-0.034
cg19268947	7	77268833	3.05E-09	0.634	0.693	0.060	*PTPN12*	1.25E-16	0.054
cg03184410	4	77613822	3.08E-09	0.719	0.772	0.054	*SHROOM3*	1.13E-14	0.045
cg22582187	10	63394414	3.41E-09	0.539	0.590	0.051	NA	1.08E-18	0.053
cg11661235	1	151827551	3.52E-09	0.673	0.726	0.052	*THEM5*	2.08E-17	0.049
cg15447971	8	121270185	3.78E-09	0.685	0.737	0.052	*COL14A1*	1.13E-13	0.043
cg16900255	15	74037743	4.03E-09	0.389	0.441	0.052	*C15orf59*	5.04E-10	0.035
cg19729744	3	194752020	4.39E-09	0.645	0.592	-0.053	NA	4.77E-17	-0.051
cg02951062	4	74269908	4.53E-09	0.670	0.723	0.053	*ALB*	5.94E-16	0.048
cg18267330	2	99095277	4.55E-09	0.693	0.746	0.053	*INPP4A*	1.03E-21	0.054
cg22974630	12	54733883	4.57E-09	0.611	0.665	0.054	*COPZ1*	3.14E-16	0.051
cg11792281	17	26443366	4.84E-09	0.638	0.714	0.076	*NLK*	3.49E-04	0.031
cg18019037	1	200409325	5.18E-09	0.753	0.803	0.050	NA	1.98E-13	0.041
cg13558371	9	126135610	5.25E-09	0.157	0.213	0.056	*CRB2*	1.62E-04	0.023
cg10818896	6	32263335	5.36E-09	0.679	0.733	0.054	*C6orf10*	8.69E-19	0.054
cg05344046	11	3731649	5.95E-09	0.711	0.764	0.053	*NUP98*	1.14E-15	0.047
cg12634306	1	40098811	6.10E-09	0.738	0.789	0.051	*HEYL*	0.002	0.017
cg03127310	1	119553960	6.21E-09	0.729	0.780	0.051	NA	3.52E-17	0.048
cg14321777	10	133874809	6.34E-09	0.633	0.685	0.052	NA	2.98E-24	0.060
cg10323490	2	88469007	7.33E-09	0.561	0.616	0.054	*THNSL2*	5.82E-14	0.047
cg06413398	6	110736865	7.43E-09	0.321	0.269	-0.051	*DDO*	4.60E-08	-0.031
cg24892069	10	33562205	8.14E-09	0.616	0.561	-0.055	*NRP1*	1.61E-11	-0.044
cg25609749	6	3743352	8.76E-09	0.323	0.273	-0.050	*PXDC1*	1.85E-16	-0.047
cg19947357	14	99864952	8.88E-09	0.603	0.653	0.050	*SETD3*	3.43E-22	0.054
cg22706992	5	115322511	9.11E-09	0.670	0.721	0.052	*AQPEP*	4.01E-17	0.050
cg21705506	17	33842181	9.75E-09	0.515	0.567	0.052	NA	8.42E-08	0.032
cg11531783	7	104696669	9.88E-09	0.668	0.720	0.052	*MLL5*	1.48E-11	0.040
cg13375690	8	66706202	1.06E-08	0.708	0.758	0.051	*PDE7A*	3.02E-20	0.052
cg12110437	8	144098888	1.10E-08	0.417	0.364	-0.053	*LOC1001336*	4.86E-31	-0.077
cg14502625	5	33162283	1.28E-08	0.434	0.485	0.050	NA	2.42E-07	0.030
cg26999154	11	43291043	1.33E-08	0.456	0.524	0.068	NA	6.04E-11	0.055
cg27154651	6	30796209	1.44E-08	0.642	0.582	-0.060	NA	5.42E-23	-0.071
cg14555759	5	39167171	1.45E-08	0.637	0.688	0.051	*FYB*	2.84E-31	0.072
cg24449629	19	52646265	1.49E-08	0.354	0.417	0.063	NA	1.30E-05	0.031
cg02417360	13	113398673	1.65E-08	0.601	0.665	0.065	*ATP11A*	9.13E-14	0.054
cg07159131	4	122867651	1.74E-08	0.755	0.806	0.051	*TRPC3*	1.17E-15	0.049
cg08343347	4	36076000	1.81E-08	0.695	0.746	0.050	*ARAP2*	1.81E-19	0.053
cg19421125	12	6882856	2.24E-08	0.278	0.226	-0.052	*LAG3*	5.34E-11	-0.040
cg22516137	17	80657589	2.39E-08	0.681	0.731	0.050	*RAB40B*	1.48E-12	0.041
cg11661375	5	17275997	3.27E-08	0.685	0.737	0.052	*BASP1*	4.44E-21	0.056
cg17461271	1	172717683	3.55E-08	0.576	0.655	0.079	NA	0.019	0.021

cg21321768	18	19176371	3.80E-08	0.558	0.611	0.053	*ESCO1*	7.21E-11	0.041
cg25925210	2	219576383	3.82E-08	0.334	0.278	-0.056	*TTLL4*	6.07E-18	-0.057
cg10342490	11	106698562	4.19E-08	0.581	0.639	0.058	*GUCY1A2*	0.001	0.022
cg09973676	8	82006417	5.16E-08	0.338	0.394	0.056	*PAG1*	8.00E-56	0.121
cg26563141	2	88124876	5.74E-08	0.507	0.565	0.058	*RGPD1*	1.84E-11	0.048
cg07730007	12	107377277	5.93E-08	0.687	0.739	0.051	*MTERFD3*	1.68E-05	0.026
cg27192248	15	65285669	7.02E-08	0.417	0.352	-0.065	NA	5.00E-08	-0.046
cg16533245	17	10612342	7.02E-08	0.758	0.811	0.053	*ADPRM*	4.57E-09	0.036
cg15123819	5	99388688	7.26E-08	0.422	0.472	0.051	NA	5.57E-09	0.036
**cg22805491**	14	51172404	1.02E-07	0.622	0.700	0.077	NA	0.096	0.016
cg01074676	14	60601069	1.07E-07	0.575	0.627	0.052	*PCNXL4*	4.61E-17	0.054
cg18007641	4	74641828	1.27E-07	0.514	0.567	0.054	NA	5.84E-21	0.060
