# Supplementary material for: Shared and Unique Patterns of DNA Methylation in Systemic Lupus Erythematosus and Primary Sjögren's Syndrome
Source: Front Immunol. 2019 Jul 30;10:1686. doi: 10.3389/fimmu.2019.01686 (PMC6688520; doi:10.3389/fimmu.2019.01686)
Supplement: Supplementary file 3 [file Table_3.pdf]

Supplementary Table S3 *Combined set of shared differentially methylated CpG sites (DMCs; n=4,945) between the SLE case-control EWAS and the pSS case-ctrl EWAS (p<1.3E-7, | Δβ |>0.05 in one of the analyses and p<6.6E-6 and same direction of effect in the other analysis) ordered by their chromosomal position*

CpG site	Chromosome	Position	Gene	Mean β ctrl	Mean β SLE	Mean β pSS	Methylation Δβ SLE-ctrl	Methylation Δβ pSS-ctrl	p-value SLE-ctrl	p-value pSS-ctrl
cg26155520	1	943647	NA	0.612	0.648	0.667	0.036	0.055	6.25E-13	2.02E-12
cg20062691	1	949392	*ISG15*	0.816	0.734	0.769	-0.082	-0.047	4.25E-46	2.76E-15
cg16526047	1	949893	*ISG15*	0.674	0.584	0.619	-0.090	-0.054	3.82E-55	5.32E-17
cg07123796	1	950971	NA	0.455	0.395	0.426	-0.060	-0.028	1.19E-82	3.80E-14
cg07787977	1	962651	*AGRN*	0.632	0.702	0.671	0.070	0.039	5.04E-66	4.30E-13
cg00300303	1	1067223	NA	0.565	0.511	0.535	-0.054	-0.030	1.21E-38	7.40E-07
cg07213830	1	1093940	NA	0.403	0.328	0.378	-0.075	-0.025	1.74E-133	8.24E-17
cg07718444	1	1094080	NA	0.341	0.289	0.322	-0.052	-0.019	5.27E-84	8.91E-08
cg14144728	1	1101462	NA	0.684	0.739	0.698	0.055	0.015	2.15E-106	4.29E-06
cg00992048	1	1695585	*NADK*	0.406	0.336	0.388	-0.070	-0.017	2.71E-117	1.86E-06
cg17023856	1	2036508	*PRKCZ*	0.480	0.388	0.436	-0.092	-0.045	3.93E-128	7.76E-20
cg18188739	1	2058941	*PRKCZ*	0.265	0.201	0.240	-0.064	-0.024	3.02E-82	4.00E-08
cg23682913	1	2080710	*PRKCZ*	0.291	0.226	0.268	-0.064	-0.023	7.02E-107	4.82E-09
cg26930596	1	2082315	*PRKCZ*	0.550	0.424	0.511	-0.126	-0.038	4.66E-136	1.42E-09
cg02481000	1	2082349	*PRKCZ*	0.385	0.321	0.364	-0.065	-0.021	2.03E-122	4.03E-08
cg15564619	1	2163437	*SKI*	0.550	0.609	0.579	0.059	0.029	3.08E-41	4.75E-06
cg25139649	1	2165579	*SKI*	0.405	0.308	0.370	-0.097	-0.036	1.04E-88	6.27E-08
cg13420413	1	2347015	NA	0.658	0.711	0.681	0.053	0.023	3.89E-50	8.50E-07
cg09245989	1	2454811	*PANK4*	0.581	0.632	0.623	0.051	0.042	3.03E-25	2.14E-08
cg24150662	1	2949673	NA	0.799	0.855	0.821	0.056	0.022	5.70E-63	3.57E-06
cg02655351	1	3498101	*MEGF6*	0.354	0.273	0.312	-0.081	-0.042	6.40E-112	7.30E-17
cg27306802	1	3510561	*MEGF6*	0.340	0.265	0.306	-0.075	-0.034	2.59E-64	1.57E-07
cg01769341	1	3526110	*MEGF6*	0.486	0.425	0.435	-0.061	-0.051	3.60E-56	2.93E-16
cg10378348	1	6244619	NA	0.246	0.193	0.216	-0.053	-0.030	2.18E-81	9.92E-13
cg04500986	1	6526531	*PLEKHG5*	0.623	0.682	0.660	0.059	0.037	6.92E-94	2.49E-22
cg18263572	1	6649337	*ZBTB48*	0.411	0.327	0.362	-0.083	-0.049	1.67E-141	1.57E-31
cg04874074	1	6855140	*CAMTA1*	0.654	0.700	0.709	0.046	0.055	9.10E-18	3.14E-11
cg22229039	1	7842159	NA	0.327	0.247	0.288	-0.081	-0.039	2.86E-73	5.21E-10
cg08840010	1	8000314	*TNFRSF9*	0.461	0.350	0.431	-0.111	-0.030	7.14E-120	1.40E-06
cg24663338	1	8005108	NA	0.501	0.379	0.456	-0.122	-0.046	5.22E-144	1.23E-13
cg20014974	1	8271918	NA	0.312	0.256	0.290	-0.057	-0.022	4.04E-80	1.15E-07
cg02847588	1	8271997	NA	0.410	0.331	0.385	-0.079	-0.025	2.53E-129	6.16E-11
cg11283860	1	8273352	NA	0.409	0.326	0.388	-0.083	-0.020	3.80E-131	2.66E-06
cg17597631	1	8443425	*RERE*	0.276	0.215	0.247	-0.061	-0.029	3.09E-54	1.28E-06
cg15702277	1	8477935	*RERE*	0.316	0.253	0.297	-0.063	-0.019	5.75E-99	3.24E-06
cg09635768	1	8601318	*RERE*	0.729	0.786	0.778	0.057	0.049	2.60E-27	1.10E-09
cg15519465	1	8738385	*RERE*	0.731	0.777	0.785	0.047	0.055	1.64E-18	1.36E-11
cg06635351	1	8882925	NA	0.396	0.304	0.353	-0.092	-0.043	5.93E-102	8.20E-13
cg04993279	1	8940460	*ENO1*	0.438	0.333	0.392	-0.104	-0.046	2.96E-160	1.04E-20
cg12763828	1	9129646	*SLC2A5*	0.397	0.307	0.355	-0.090	-0.042	4.52E-134	4.28E-17
cg00310940	1	9129648	*SLC2A5*	0.262	0.207	0.237	-0.055	-0.025	2.63E-84	4.71E-09
cg13869484	1	9140563	NA	0.592	0.658	0.626	0.065	0.034	2.55E-63	6.08E-09
cg16104584	1	9149734	NA	0.349	0.276	0.321	-0.073	-0.028	8.87E-69	3.89E-06
cg17952939	1	9154250	NA	0.409	0.329	0.384	-0.080	-0.025	6.95E-101	4.77E-07
cg25103337	1	9293583	*H6PD*	0.459	0.347	0.411	-0.112	-0.048	9.64E-145	9.78E-18
cg20305595	1	9293833	*H6PD*	0.468	0.410	0.438	-0.058	-0.030	3.95E-93	4.34E-15
cg25017777	1	9298817	*H6PD*	0.422	0.354	0.394	-0.068	-0.028	4.87E-125	5.87E-15
cg02276314	1	9301104	*H6PD*	0.658	0.592	0.633	-0.066	-0.025	3.16E-91	5.93E-08
cg06779253	1	9375627	*SPSB1*	0.546	0.597	0.564	0.050	0.018	1.32E-70	8.40E-07
cg04972459	1	9460204	NA	0.391	0.311	0.357	-0.081	-0.035	4.43E-146	5.22E-16
cg23098018	1	9775755	*PIK3CD*	0.684	0.757	0.712	0.073	0.028	7.76E-79	8.08E-09
cg09706586	1	9775838	*PIK3CD*	0.675	0.741	0.713	0.065	0.037	3.26E-108	2.47E-20
cg11947782	1	9775985	*PIK3CD*	0.749	0.820	0.778	0.071	0.030	3.05E-83	8.48E-09
cg01943221	1	9776133	*PIK3CD*	0.687	0.753	0.711	0.066	0.024	2.27E-81	1.23E-07
cg07499142	1	9788715	*PIK3CD*	0.508	0.591	0.543	0.082	0.035	2.46E-99	8.91E-11
cg12033075	1	9788767	*PIK3CD*	0.328	0.403	0.383	0.075	0.055	5.62E-49	8.28E-15
cg03971555	1	9788769	*PIK3CD*	0.292	0.372	0.336	0.080	0.044	3.57E-51	1.86E-09
cg07970040	1	9789165	*CLSTN1*	0.733	0.797	0.763	0.064	0.030	1.64E-127	2.00E-16
cg04340595	1	9789174	*CLSTN1*	0.529	0.624	0.574	0.095	0.045	5.32E-125	7.68E-18
cg06829969	1	10460205	*PGD*	0.582	0.488	0.534	-0.095	-0.048	1.93E-103	3.99E-16
cg22515654	1	10590672	*PEX14*	0.572	0.497	0.534	-0.075	-0.037	2.74E-50	7.58E-08
cg23975251	1	10604446	*PEX14*	0.561	0.640	0.615	0.078	0.054	6.82E-65	1.39E-18
cg15078958	1	10997656	NA	0.656	0.722	0.682	0.066	0.026	7.88E-112	6.45E-11
cg08945450	1	11795905	*AGTRAP*	0.329	0.261	0.300	-0.068	-0.029	4.10E-103	1.04E-11
cg21826784	1	11795937	*AGTRAP*	0.313	0.239	0.284	-0.074	-0.029	3.48E-109	3.31E-11
cg15527643	1	11795946	*AGTRAP*	0.366	0.300	0.334	-0.067	-0.032	6.59E-109	1.18E-17
cg25467652	1	11795976	*AGTRAP*	0.259	0.203	0.226	-0.056	-0.033	1.49E-76	3.06E-15
cg22501942	1	11798278	*AGTRAP*	0.332	0.253	0.291	-0.080	-0.041	7.35E-173	8.35E-30
cg17514528	1	11862907	*MTHFR*	0.489	0.433	0.444	-0.056	-0.045	2.30E-26	9.78E-09
cg07585427	1	11917550	*NPPB*	0.603	0.654	0.632	0.051	0.030	1.27E-27	3.82E-06
cg10058766	1	12186079	*TNFRSF8*	0.366	0.294	0.343	-0.072	-0.024	4.59E-92	1.21E-06
cg09247175	1	12203571	*TNFRSF8*	0.302	0.251	0.280	-0.051	-0.022	5.90E-85	4.20E-09

cg24492058	1	12203751	*TNFRSF8*	0.289	0.239	0.270	-0.050	-0.019	4.27E-68	3.18E-06
cg05599723	1	12241073	*TNFRSF1B*	0.492	0.376	0.454	-0.116	-0.038	5.76E-143	3.37E-12
cg06620723	1	12404945	*VPS13D*	0.261	0.197	0.236	-0.064	-0.025	6.56E-73	2.04E-06
cg23983887	1	12509707	*VPS13D*	0.526	0.592	0.564	0.066	0.038	2.80E-48	1.47E-09
cg17494034	1	12513497	*VPS13D*	0.666	0.723	0.699	0.058	0.034	9.98E-85	7.04E-16
cg04505750	1	12774815	*AADACL3*	0.341	0.266	0.315	-0.075	-0.026	7.39E-89	3.74E-06
cg19620994	1	12774904	*AADACL3*	0.468	0.357	0.431	-0.111	-0.037	1.66E-149	2.40E-12
cg07708453	1	14032034	*PRDM2*	0.454	0.342	0.426	-0.112	-0.028	2.86E-154	3.40E-07
cg03538296	1	15392433	*KAZN*	0.767	0.821	0.814	0.055	0.047	1.32E-26	7.36E-09
cg15146752	1	16482767	*EPHA2*	0.513	0.456	0.487	-0.057	-0.026	6.96E-96	5.09E-13
cg18787437	1	16499673	NA	0.304	0.250	0.272	-0.054	-0.033	7.17E-67	2.79E-11
cg15553418	1	16696547	*SZRD1*	0.428	0.324	0.382	-0.104	-0.046	2.63E-147	5.77E-20
cg19414383	1	17528238	NA	0.505	0.563	0.530	0.058	0.025	1.09E-58	2.93E-07
cg08038629	1	17751673	*RCC2*	0.290	0.222	0.255	-0.068	-0.035	8.21E-122	9.25E-18
cg05523906	1	17751761	*RCC2*	0.476	0.411	0.447	-0.065	-0.028	1.35E-69	6.59E-08
cg16265859	1	19401069	*UBR4*	0.661	0.740	0.698	0.078	0.037	2.25E-117	8.93E-16
cg02862467	1	19407897	*UBR4*	0.466	0.348	0.419	-0.117	-0.046	2.03E-149	3.02E-15
cg00968167	1	19810215	*CAPZB*	0.555	0.621	0.585	0.066	0.030	3.96E-47	2.92E-06
cg24502330	1	20914028	*CDA*	0.377	0.305	0.338	-0.072	-0.039	7.28E-41	3.82E-07
cg06984156	1	20929640	*CDA*	0.365	0.285	0.334	-0.080	-0.031	1.67E-141	1.01E-15
cg08539965	1	21396338	*EIF4G3*	0.492	0.368	0.452	-0.125	-0.041	2.19E-179	3.06E-14
cg25007705	1	21588799	*ECE1*	0.342	0.264	0.315	-0.078	-0.027	2.17E-106	1.88E-08
cg16603916	1	21615863	*ECE1*	0.422	0.348	0.400	-0.074	-0.022	2.11E-107	3.49E-07
cg26348226	1	21617442	*ECE1*	0.260	0.205	0.234	-0.055	-0.026	7.59E-62	5.74E-07
cg13086983	1	21664810	*ECE1*	0.412	0.310	0.364	-0.102	-0.048	5.90E-172	9.62E-27
cg12748948	1	22106295	*USP48*	0.517	0.586	0.564	0.069	0.047	7.53E-36	6.41E-09
cg06830981	1	22142491	*LDLRAD2*	0.337	0.283	0.305	-0.054	-0.032	3.46E-68	3.37E-16
cg08608952	1	22383198	*CDC42*	0.721	0.774	0.749	0.053	0.028	1.76E-53	7.67E-08
cg12483005	1	23474871	*LUZP1*	0.719	0.778	0.760	0.059	0.041	1.62E-50	1.28E-11
cg15598244	1	23696413	*ZNF436*	0.203	0.150	0.180	-0.053	-0.023	4.75E-63	1.69E-06
cg16555909	1	23721285	*TCEA3*	0.573	0.493	0.540	-0.080	-0.034	5.63E-95	1.75E-10
cg01015663	1	23729692	*TCEA3*	0.487	0.406	0.443	-0.080	-0.043	1.37E-78	1.98E-13
cg18150584	1	23887816	NA	0.349	0.254	0.298	-0.095	-0.051	9.90E-162	2.70E-29
cg10511985	1	24053853	NA	0.416	0.326	0.388	-0.089	-0.028	2.13E-187	1.92E-16
cg21696012	1	24131000	*HMGCL*	0.498	0.392	0.461	-0.106	-0.037	1.42E-125	3.97E-11
cg22675447	1	24745395	*NIPAL3*	0.446	0.349	0.406	-0.097	-0.040	2.85E-96	2.61E-10
cg15519096	1	24833311	*RCAN3*	0.743	0.810	0.769	0.068	0.026	1.22E-118	1.24E-10
cg20146241	1	24861604	*RCAN3*	0.412	0.500	0.444	0.088	0.032	1.25E-80	1.71E-07
cg01519464	1	24861818	*RCAN3*	0.760	0.824	0.793	0.064	0.033	3.94E-101	1.69E-14
cg25140783	1	24861872	*RCAN3*	0.560	0.674	0.618	0.114	0.058	8.02E-148	5.55E-22
cg26692749	1	24861919	*RCAN3*	0.686	0.758	0.722	0.072	0.035	1.49E-76	2.86E-10
cg03655701	1	24885664	*NCMAP*	0.389	0.294	0.341	-0.096	-0.049	5.16E-126	1.40E-21
cg07201717	1	25031947	NA	0.302	0.243	0.276	-0.059	-0.026	1.29E-102	2.04E-12
cg19857379	1	25105430	*CLIC4*	0.647	0.712	0.702	0.065	0.055	1.84E-32	1.79E-10
cg03961551	1	25251730	*RUNX3*	0.547	0.645	0.570	0.098	0.023	8.68E-151	3.61E-08
cg07236781	1	25291041	*RUNX3*	0.791	0.846	0.813	0.054	0.022	1.29E-83	2.02E-08
cg13461622	1	25291385	*RUNX3*	0.735	0.795	0.757	0.061	0.023	3.93E-88	1.45E-07
cg06640822	1	25291472	*RUNX3*	0.653	0.723	0.690	0.070	0.037	3.00E-113	3.43E-19
cg27058497	1	25291546	*RUNX3*	0.530	0.623	0.568	0.092	0.038	2.26E-97	1.35E-10
cg09993145	1	25291905	*RUNX3*	0.445	0.552	0.481	0.107	0.036	3.05E-110	6.28E-10
cg17973115	1	25333445	NA	0.387	0.292	0.357	-0.095	-0.031	9.36E-101	4.54E-07
cg22125902	1	26002535	*MAN1C1*	0.604	0.682	0.633	0.078	0.029	3.73E-115	4.21E-10
cg12178432	1	26049105	*MAN1C1*	0.619	0.674	0.678	0.056	0.059	4.00E-19	7.90E-10
cg27430637	1	26646801	*CD52*	0.514	0.570	0.533	0.056	0.019	2.31E-103	9.49E-10
cg24585377	1	26857774	*RPS6KA1*	0.394	0.320	0.351	-0.074	-0.043	4.18E-81	1.01E-13
cg07785552	1	26869620	*RPS6KA1*	0.368	0.305	0.342	-0.062	-0.026	1.23E-115	3.50E-15
cg23955417	1	26880928	*RPS6KA1*	0.280	0.214	0.241	-0.067	-0.039	4.98E-75	1.88E-14
cg11377047	1	26881009	*RPS6KA1*	0.384	0.286	0.329	-0.099	-0.055	1.59E-111	1.81E-19
cg10104487	1	27329335	NA	0.570	0.511	0.523	-0.059	-0.047	1.50E-49	9.57E-18
cg27531366	1	27627058	*WDTC1*	0.362	0.282	0.318	-0.080	-0.044	3.79E-112	2.45E-21
cg07551364	1	27693782	*MAP3K6*	0.430	0.376	0.393	-0.054	-0.036	7.83E-64	3.04E-14
cg11529819	1	27695677	*FCN3*	0.324	0.263	0.296	-0.060	-0.028	1.26E-102	1.23E-13
cg27513684	1	27729053	NA	0.283	0.225	0.255	-0.058	-0.028	3.75E-102	3.60E-15
cg03604774	1	27849102	NA	0.647	0.709	0.692	0.062	0.044	1.56E-43	2.03E-11
cg12759387	1	27849177	NA	0.498	0.555	0.519	0.058	0.022	2.62E-84	3.94E-09
cg24534743	1	27884345	*AHDC1*	0.398	0.338	0.377	-0.060	-0.020	6.50E-152	1.90E-13
cg27543538	1	27902687	*AHDC1*	0.377	0.310	0.352	-0.067	-0.024	3.62E-82	2.11E-07
cg14510299	1	27928494	*AHDC1*	0.363	0.284	0.335	-0.079	-0.028	2.97E-102	5.66E-09
cg12465678	1	27953336	*FGR*	0.348	0.275	0.315	-0.072	-0.033	2.71E-94	3.14E-12
cg14181576	1	27961563	*FGR*	0.272	0.203	0.244	-0.069	-0.028	4.09E-82	1.73E-07
cg16922167	1	27961746	*FGR*	0.501	0.414	0.468	-0.087	-0.033	6.91E-98	5.03E-10
cg21115433	1	27961868	*FGR*	0.251	0.193	0.222	-0.058	-0.029	6.47E-62	1.15E-08
cg06875162	1	28184973	NA	0.486	0.421	0.447	-0.065	-0.040	3.30E-66	1.49E-12
cg20945221	1	28423646	NA	0.493	0.431	0.461	-0.062	-0.032	2.01E-77	5.84E-12
cg14288403	1	28503368	*PTAFR*	0.276	0.225	0.258	-0.050	-0.018	9.81E-84	6.06E-07
cg08123074	1	28764523	*PHACTR4*	0.212	0.148	0.188	-0.063	-0.024	1.89E-82	2.06E-06
cg23847017	1	28764854	*PHACTR4*	0.309	0.231	0.277	-0.078	-0.032	2.68E-91	7.46E-09
cg00956142	1	28765031	*PHACTR4*	0.343	0.259	0.299	-0.084	-0.044	2.28E-91	4.61E-15

cg00261690	1	28856281	*RCC1*	0.499	0.394	0.460	-0.105	-0.039	1.83E-131	6.53E-14
cg25187161	1	29510025	*SRSF4*	0.316	0.257	0.274	-0.059	-0.042	3.67E-60	6.08E-18
cg24155427	1	31242051	NA	0.310	0.248	0.281	-0.062	-0.029	1.99E-117	1.20E-15
cg12297231	1	31531229	*PUM1*	0.716	0.767	0.760	0.051	0.044	7.79E-47	8.17E-17
cg09017001	1	31870508	NA	0.440	0.353	0.407	-0.087	-0.033	9.38E-120	1.21E-11
cg24401262	1	31956405	NA	0.378	0.276	0.329	-0.102	-0.049	3.10E-137	3.21E-17
cg04552418	1	31958245	NA	0.474	0.353	0.430	-0.121	-0.043	6.08E-132	2.03E-12
cg01804679	1	32707303	*MTMR9LP*	0.280	0.224	0.257	-0.056	-0.023	2.06E-84	3.70E-08
cg09942999	1	32707389	NA	0.280	0.217	0.252	-0.063	-0.028	6.21E-77	3.08E-09
cg10204320	1	32708276	NA	0.251	0.199	0.225	-0.052	-0.026	3.24E-66	1.06E-08
cg07571745	1	32715428	*LCK*	0.492	0.553	0.513	0.061	0.020	2.09E-82	3.01E-07
cg11683242	1	32716557	*LCK*	0.779	0.838	0.808	0.059	0.029	7.85E-113	1.54E-15
cg16348358	1	32731477	*LCK*	0.472	0.398	0.435	-0.073	-0.037	3.98E-99	1.76E-15
cg14781242	1	32738251	*LCK*	0.684	0.735	0.708	0.052	0.025	8.92E-64	5.07E-09
cg10325497	1	32739049	*LCK*	0.706	0.776	0.745	0.070	0.039	1.57E-150	2.94E-32
cg17223520	1	32739114	*LCK*	0.766	0.816	0.796	0.051	0.030	1.14E-81	6.57E-17
cg26856257	1	32805350	NA	0.211	0.157	0.171	-0.055	-0.040	3.25E-50	4.10E-15
cg18118795	1	33117735	*RBBP4*	0.530	0.581	0.579	0.052	0.050	5.48E-25	1.73E-10
cg02073304	1	33457500	NA	0.305	0.235	0.269	-0.071	-0.037	1.74E-129	2.01E-21
cg19383974	1	33463643	NA	0.350	0.286	0.329	-0.063	-0.020	1.20E-112	1.78E-08
cg11254053	1	33497125	*AK2*	0.475	0.360	0.427	-0.115	-0.047	1.68E-184	3.05E-23
cg07979236	1	33516461	NA	0.456	0.355	0.412	-0.100	-0.043	5.96E-66	2.90E-07
cg23202722	1	33793808	*PHC2*	0.416	0.312	0.372	-0.104	-0.044	1.73E-129	1.42E-14
cg12633102	1	35676489	NA	0.687	0.751	0.716	0.064	0.029	5.23E-84	2.20E-11
cg24525364	1	35730504	NA	0.237	0.182	0.209	-0.055	-0.028	1.15E-90	1.02E-11
cg22526531	1	35913782	*KIAA0319L*	0.575	0.469	0.545	-0.106	-0.030	8.51E-126	1.79E-07
cg10959672	1	35972127	*KIAA0319L*	0.716	0.786	0.748	0.071	0.032	2.44E-122	3.00E-14
cg13550410	1	36004549	*KIAA0319L*	0.725	0.782	0.742	0.058	0.017	2.82E-97	3.54E-06
cg16356622	1	36351841	*EIF2C1*	0.480	0.401	0.442	-0.079	-0.038	4.94E-102	5.27E-16
cg15142192	1	36521401	*EIF2C3*	0.448	0.345	0.415	-0.103	-0.033	4.13E-101	2.49E-07
cg16394290	1	36788416	*FAM176B*	0.345	0.267	0.311	-0.077	-0.034	6.43E-61	1.66E-06
cg05798664	1	36825645	*STK40*	0.375	0.299	0.352	-0.076	-0.023	4.88E-105	1.19E-06
cg00913954	1	36852956	*STK40*	0.535	0.435	0.478	-0.100	-0.056	9.63E-121	6.43E-23
cg04430911	1	36914349	*OSCP1*	0.421	0.330	0.387	-0.092	-0.034	4.57E-201	4.03E-28
cg01873305	1	36931324	*MRPS15*	0.669	0.711	0.723	0.042	0.054	1.12E-13	3.48E-11
cg11674933	1	36948313	*CSF3R*	0.276	0.220	0.248	-0.057	-0.028	1.87E-80	4.25E-11
cg13084345	1	36948733	*CSF3R*	0.218	0.164	0.190	-0.054	-0.027	7.41E-118	3.55E-17
cg07285167	1	36948981	*CSF3R*	0.388	0.282	0.341	-0.107	-0.048	2.08E-149	7.25E-18
cg13423759	1	37937403	*LOC728431*	0.252	0.179	0.221	-0.073	-0.030	2.03E-94	1.15E-10
cg13100137	1	37937945	*LOC728431*	0.574	0.508	0.526	-0.066	-0.048	1.37E-60	1.16E-16
cg24428600	1	37952273	NA	0.242	0.188	0.218	-0.054	-0.024	1.43E-118	1.83E-14
cg04496824	1	38276835	*MTF1*	0.298	0.235	0.274	-0.063	-0.024	6.57E-83	3.01E-07
cg26528311	1	38462546	*FHL3*	0.354	0.280	0.327	-0.074	-0.028	6.71E-100	4.02E-09
cg17434008	1	38974111	NA	0.381	0.295	0.343	-0.087	-0.038	8.66E-106	4.96E-12
cg08876103	1	39572323	*MACF1*	0.438	0.377	0.383	-0.062	-0.056	2.76E-47	2.26E-19
cg18154117	1	39649937	*MACF1*	0.663	0.731	0.716	0.068	0.053	1.45E-46	2.27E-14
cg11742479	1	40026520	*PABPC4*	0.667	0.708	0.724	0.041	0.057	3.11E-18	4.74E-14
cg00255699	1	40307257	*TRIT1*	0.708	0.774	0.730	0.065	0.022	7.25E-89	2.07E-06
cg08106973	1	40399833	NA	0.390	0.287	0.336	-0.103	-0.054	1.94E-139	4.33E-24
cg01869896	1	40420299	*MFSD2A*	0.596	0.523	0.564	-0.073	-0.031	1.35E-86	1.21E-10
cg07033722	1	40539032	*PPT1*	0.499	0.375	0.447	-0.125	-0.052	1.00E-174	1.94E-23
cg11177980	1	41982115	*HIVEP3*	0.344	0.293	0.326	-0.051	-0.018	2.15E-64	4.75E-06
cg12666727	1	42128487	*HIVEP3*	0.189	0.137	0.163	-0.053	-0.026	4.79E-53	8.31E-07
cg13423554	1	42317463	*HIVEP3*	0.267	0.200	0.244	-0.067	-0.023	2.70E-93	3.00E-07
cg21830221	1	42334284	*HIVEP3*	0.672	0.736	0.704	0.064	0.032	7.24E-61	5.19E-09
cg24501381	1	43001072	*CCDC30*	0.537	0.421	0.501	-0.116	-0.035	5.03E-136	9.47E-10
cg00917471	1	43228316	*LEPRE1*	0.375	0.302	0.343	-0.074	-0.033	4.60E-82	5.53E-10
cg19636224	1	44176844	*ST3GAL3*	0.520	0.428	0.491	-0.092	-0.029	9.06E-110	3.39E-08
cg20634514	1	44703750	*ERI3*	0.476	0.416	0.451	-0.060	-0.026	2.74E-53	5.71E-06
cg21578207	1	44870088	*RNF220*	0.264	0.214	0.232	-0.050	-0.033	5.97E-59	6.95E-12
cg13573582	1	44887933	*RNF220*	0.495	0.401	0.460	-0.094	-0.035	9.76E-95	9.62E-09
cg18022893	1	44951225	*RNF220*	0.351	0.280	0.322	-0.072	-0.029	2.64E-62	4.82E-07
cg13051977	1	45148001	*C1orf228*	0.365	0.288	0.333	-0.077	-0.032	3.20E-88	1.35E-08
cg26297462	1	45292204	*PTCH2*	0.311	0.257	0.287	-0.054	-0.024	4.36E-90	2.29E-11
cg15684702	1	45297445	*PTCH2*	0.428	0.348	0.396	-0.080	-0.032	4.11E-71	7.73E-08
cg09409484	1	45770648	*LOC400752*	0.610	0.558	0.576	-0.053	-0.034	2.56E-42	5.61E-10
cg17412005	1	45806886	*TOE1*	0.285	0.225	0.249	-0.059	-0.036	3.50E-68	1.89E-12
cg17156633	1	46215776	*IPP*	0.386	0.324	0.357	-0.062	-0.029	3.96E-68	4.43E-10
cg21407196	1	46751975	*LRRC41*	0.722	0.778	0.756	0.056	0.034	1.54E-81	8.05E-16
cg22228439	1	46766431	*LRRC41*	0.411	0.329	0.379	-0.082	-0.032	2.91E-150	3.17E-15
cg01440934	1	47051752	*MKNK1*	0.483	0.376	0.445	-0.107	-0.038	3.38E-120	1.21E-09
cg07150145	1	47656137	*PDZK1IP1*	0.350	0.299	0.328	-0.051	-0.022	9.42E-102	8.03E-13
cg02291556	1	47656140	*PDZK1IP1*	0.262	0.208	0.245	-0.054	-0.018	1.23E-94	3.14E-06
cg04542977	1	51982669	*EPS15*	0.334	0.301	0.284	-0.033	-0.050	6.85E-16	5.39E-15
cg26009944	1	52210504	*OSBPL9*	0.751	0.778	0.804	0.027	0.053	5.65E-07	1.04E-10
cg00754357	1	53019727	*ZCCHC11*	0.465	0.377	0.419	-0.088	-0.046	4.22E-99	6.81E-14
cg21572480	1	53161198	*SELRC1*	0.684	0.735	0.733	0.050	0.049	7.99E-23	2.79E-10
cg14039246	1	53365446	*ECHDC2*	0.430	0.341	0.398	-0.089	-0.032	1.69E-93	1.52E-07

cg24107728	1	53760337	*LRP8*	0.500	0.581	0.530	0.081	0.030	4.87E-72	2.27E-06
cg12299361	1	53796769	NA	0.420	0.328	0.395	-0.092	-0.026	4.46E-145	2.10E-11
cg17734802	1	54358862	*DIO1*	0.449	0.342	0.403	-0.107	-0.046	2.16E-146	1.37E-17
cg01182697	1	54520432	*TCEANC2*	0.384	0.296	0.344	-0.088	-0.040	1.39E-125	3.06E-16
cg20793071	1	54520450	*TCEANC2*	0.247	0.192	0.225	-0.055	-0.022	4.59E-67	4.39E-06
cg06659338	1	54562040	*TCEANC2*	0.441	0.367	0.409	-0.074	-0.032	4.59E-112	3.11E-14
cg24593372	1	54562121	*TCEANC2*	0.386	0.323	0.355	-0.063	-0.031	1.01E-106	1.55E-16
cg00112238	1	54719938	*SSBP3*	0.331	0.261	0.301	-0.070	-0.030	1.15E-133	3.59E-17
cg10761558	1	54842205	*SSBP3*	0.691	0.747	0.722	0.056	0.031	1.96E-46	8.02E-08
cg10710218	1	55012989	*ACOT11*	0.411	0.324	0.374	-0.087	-0.037	1.47E-85	2.16E-09
cg02905900	1	55013517	*ACOT11*	0.328	0.266	0.299	-0.062	-0.029	1.00E-139	2.68E-19
cg10266490	1	55013709	*ACOT11*	0.314	0.241	0.271	-0.073	-0.043	7.81E-77	2.50E-13
cg26406563	1	55117536	*HEATR8-TTC*	0.367	0.296	0.334	-0.072	-0.033	3.97E-115	2.62E-14
cg17901584	1	55353706	*DHCR24*	0.557	0.479	0.522	-0.078	-0.035	2.28E-54	4.52E-08
cg04084354	1	56721795	NA	0.343	0.258	0.288	-0.086	-0.055	3.09E-109	5.36E-23
cg07477602	1	56961319	*PPAP2B*	0.458	0.362	0.409	-0.096	-0.049	4.08E-90	2.54E-13
cg22396959	1	57013771	*PPAP2B*	0.534	0.457	0.503	-0.077	-0.032	7.80E-75	1.02E-08
cg06208288	1	58858074	NA	0.644	0.739	0.681	0.095	0.036	3.61E-109	1.21E-11
cg01923724	1	58864209	NA	0.479	0.398	0.453	-0.081	-0.026	5.56E-122	2.63E-09
cg21536783	1	59041407	*TACSTD2*	0.444	0.368	0.419	-0.076	-0.025	1.17E-112	1.48E-07
cg08941853	1	59449033	NA	0.329	0.275	0.306	-0.054	-0.024	6.96E-57	7.37E-07
cg23670353	1	59833489	*FGGY*	0.421	0.306	0.376	-0.115	-0.046	5.18E-162	4.40E-16
cg10220544	1	60171660	*FGGY*	0.415	0.323	0.379	-0.091	-0.036	2.56E-97	1.26E-08
cg24370698	1	64058614	*PGM1*	0.408	0.330	0.372	-0.078	-0.036	1.54E-47	5.43E-06
cg24306340	1	64937451	*CACHD1*	0.411	0.360	0.378	-0.050	-0.032	2.35E-30	6.03E-06
cg26224018	1	64942153	*CACHD1*	0.387	0.326	0.359	-0.062	-0.029	9.57E-59	2.17E-06
cg24639703	1	64986155	*CACHD1*	0.669	0.729	0.707	0.060	0.038	7.50E-35	4.95E-07
cg13390284	1	65531864	NA	0.485	0.418	0.452	-0.067	-0.032	9.55E-75	4.99E-09
cg07842386	1	67632028	*IL23R*	0.739	0.797	0.771	0.058	0.032	4.34E-66	1.20E-10
cg09018107	1	67798929	*IL12RB2*	0.730	0.806	0.774	0.076	0.044	1.21E-71	7.44E-13
cg14849855	1	67807960	*IL12RB2*	0.707	0.768	0.729	0.062	0.022	1.92E-95	2.79E-08
cg16110541	1	68679600	*WLS*	0.395	0.327	0.371	-0.069	-0.024	7.19E-101	1.11E-09
cg19342782	1	70821806	*HHLA3*	0.502	0.378	0.448	-0.124	-0.054	3.37E-111	1.13E-13
cg18854765	1	74665362	*FPGT*	0.389	0.301	0.364	-0.088	-0.025	6.72E-107	3.74E-06
cg00013349	1	77049380	*ST6GALNAC3*	0.741	0.795	0.780	0.054	0.038	1.07E-34	7.70E-09
cg02083528	1	78280850	*FAM73A*	0.777	0.819	0.830	0.042	0.052	1.64E-17	1.66E-12
cg15015070	1	78567723	*GIPC2*	0.757	0.804	0.808	0.047	0.050	5.14E-20	9.52E-11
cg13304609	1	79085162	*IFI44L*	0.850	0.722	0.785	-0.128	-0.065	4.95E-63	2.74E-31
cg03607951	1	79085586	*IFI44L*	0.590	0.339	0.415	-0.251	-0.175	2.96E-141	9.92E-67
cg17980508	1	79085713	*IFI44L*	0.356	0.216	0.294	-0.140	-0.061	3.77E-179	2.50E-27
cg00855901	1	79085765	*IFI44L*	0.275	0.167	0.217	-0.108	-0.057	1.00E-135	7.45E-24
cg05696877	1	79088769	*IFI44L*	0.671	0.408	0.475	-0.263	-0.196	1.93E-120	2.85E-50
cg07107453	1	79114976	*IFI44*	0.418	0.350	0.363	-0.068	-0.054	2.37E-27	6.44E-10
cg19869443	1	84766821	*SAMD13*	0.417	0.331	0.378	-0.087	-0.040	5.01E-93	1.14E-10
cg03204322	1	84767878	*SAMD13*	0.260	0.199	0.231	-0.062	-0.029	1.53E-58	4.34E-07
cg26801613	1	87793510	*LMO4*	0.675	0.599	0.613	-0.076	-0.062	9.44E-84	8.59E-32
cg20633321	1	87793607	*LMO4*	0.331	0.260	0.285	-0.071	-0.046	6.32E-73	2.33E-15
cg11068071	1	89592433	*GBP2*	0.573	0.638	0.620	0.065	0.047	2.41E-39	1.68E-10
cg11839020	1	90309998	*LRRC8D*	0.563	0.636	0.588	0.073	0.025	9.01E-84	1.99E-07
cg01965380	1	90321064	*LRRC8D*	0.693	0.743	0.725	0.051	0.032	3.70E-39	2.84E-08
cg01533966	1	90363165	*LRRC8D*	0.509	0.564	0.555	0.055	0.046	5.12E-24	1.10E-08
cg25320328	1	92953037	*GFI1*	0.385	0.318	0.355	-0.067	-0.030	4.55E-58	5.72E-07
cg06829157	1	93430542	NA	0.648	0.709	0.687	0.061	0.040	1.94E-36	3.65E-08
cg21386573	1	94219800	*BCAR3*	0.564	0.489	0.509	-0.074	-0.054	7.95E-35	5.81E-11
cg22401679	1	95080047	NA	0.447	0.381	0.413	-0.066	-0.034	1.46E-46	3.13E-07
cg04079215	1	95095221	NA	0.449	0.366	0.418	-0.083	-0.031	2.21E-120	8.10E-11
cg06788267	1	95258162	NA	0.298	0.236	0.264	-0.062	-0.034	1.56E-77	3.49E-13
cg21263566	1	95561502	*TMEM56*	0.455	0.355	0.417	-0.099	-0.038	1.08E-128	2.29E-13
cg14378231	1	98404032	NA	0.577	0.486	0.543	-0.091	-0.034	8.26E-114	1.12E-10
cg17222434	1	100215737	*FRRS1*	0.604	0.661	0.648	0.057	0.044	1.45E-35	2.27E-10
cg01933228	1	100316637	*AGL*	0.451	0.401	0.412	-0.051	-0.039	1.35E-44	3.03E-13
cg03847230	1	100675407	*DBT*	0.708	0.747	0.759	0.040	0.051	1.80E-16	2.97E-12
cg04295372	1	101184332	*VCAM1*	0.461	0.399	0.424	-0.062	-0.037	1.33E-48	2.86E-09
cg04205769	1	101488098	*DPH5*	0.503	0.399	0.471	-0.104	-0.032	2.53E-105	7.70E-08
cg15447231	1	101537255	NA	0.390	0.327	0.359	-0.063	-0.031	5.42E-69	1.76E-08
cg17972213	1	101704898	*S1PR1*	0.710	0.788	0.740	0.078	0.030	3.06E-96	4.69E-10
cg03050965	1	101705237	*S1PR1*	0.718	0.782	0.746	0.063	0.028	6.90E-100	5.29E-12
cg13854219	1	101757037	NA	0.369	0.287	0.319	-0.082	-0.050	5.93E-99	4.37E-19
cg06207961	1	108661230	NA	0.356	0.287	0.314	-0.069	-0.042	3.10E-61	1.97E-12
cg24323958	1	108741884	*SLC25A24*	0.364	0.280	0.322	-0.084	-0.042	2.24E-92	7.86E-14
cg00328227	1	109204325	*HENMT1*	0.457	0.509	0.491	0.052	0.033	1.61E-27	4.03E-07
cg09131339	1	109914235	*SORT1*	0.306	0.241	0.277	-0.065	-0.029	1.46E-94	1.17E-11
cg23616212	1	109941201	*SORT1*	0.372	0.313	0.339	-0.059	-0.033	3.28E-48	1.31E-07
cg19244300	1	110113304	*GNAI3*	0.420	0.326	0.385	-0.094	-0.035	7.46E-140	1.15E-12
cg16140253	1	110166563	*AMPD2*	0.351	0.290	0.326	-0.061	-0.024	1.02E-99	7.09E-11
cg20253872	1	110166571	*AMPD2*	0.385	0.326	0.360	-0.059	-0.025	3.32E-108	5.35E-13
cg13229972	1	110335018	NA	0.373	0.303	0.339	-0.070	-0.034	1.33E-140	2.31E-20
cg19595244	1	110526158	*AHCYL1*	0.479	0.386	0.440	-0.092	-0.039	3.51E-124	3.74E-13

cg24681307	1	110526191	*AHCYL1*	0.419	0.323	0.367	-0.096	-0.051	2.02E-115	4.17E-17
cg06903031	1	110644949	NA	0.436	0.384	0.410	-0.052	-0.026	9.42E-39	5.65E-06
cg24058365	1	110923328	*SLC16A4*	0.427	0.340	0.401	-0.087	-0.026	3.59E-92	6.16E-06
cg03535830	1	110934327	*SLC16A4*	0.723	0.774	0.780	0.051	0.057	1.93E-21	4.11E-12
cg25032124	1	110946480	*HBXIP*	0.671	0.722	0.727	0.051	0.056	8.31E-18	8.86E-10
cg27230882	1	110976749	NA	0.447	0.335	0.394	-0.112	-0.053	9.12E-184	3.61E-28
cg13925011	1	111216387	*KCNA3*	0.667	0.731	0.689	0.063	0.021	2.03E-78	3.49E-07
cg26518580	1	111742515	*DENND2D*	0.700	0.770	0.727	0.070	0.027	7.64E-120	3.21E-10
cg09826895	1	111744309	*DENND2D*	0.645	0.696	0.672	0.050	0.027	1.61E-57	1.63E-09
cg10541466	1	113425263	NA	0.280	0.223	0.246	-0.056	-0.034	1.32E-69	1.88E-14
cg20784259	1	114518035	*HIPK1*	0.210	0.160	0.189	-0.050	-0.021	1.31E-61	2.67E-06
cg19758134	1	114829082	NA	0.662	0.716	0.705	0.054	0.042	2.52E-20	1.72E-06
cg14423702	1	116311953	*CASQ2*	0.722	0.772	0.766	0.051	0.044	1.00E-23	1.87E-09
cg22353097	1	116521539	*SLC22A15*	0.487	0.405	0.453	-0.081	-0.034	9.41E-75	1.14E-08
cg14027204	1	117529478	*PTGFRN*	0.709	0.765	0.742	0.056	0.033	6.72E-83	4.47E-16
cg15937641	1	117529619	*PTGFRN*	0.674	0.725	0.705	0.051	0.031	3.11E-43	2.73E-08
cg03127310	1	119553960	NA	0.729	0.777	0.780	0.048	0.051	3.52E-17	6.21E-09
cg07814932	1	120437090	*ADAM30*	0.609	0.499	0.566	-0.110	-0.044	2.69E-122	5.48E-14
cg22712955	1	144935409	*PDE4DIP*	0.714	0.778	0.744	0.064	0.030	5.16E-55	3.06E-07
cg21854332	1	144989624	*PDE4DIP*	0.365	0.270	0.322	-0.096	-0.043	4.07E-183	2.22E-27
cg07906625	1	145021703	*PDE4DIP*	0.279	0.223	0.256	-0.056	-0.023	1.12E-115	1.40E-12
cg16511445	1	145116797	*SEC22B*	0.287	0.219	0.259	-0.068	-0.028	4.15E-83	1.98E-08
cg24694018	1	145457621	*POLR3GL*	0.661	0.607	0.637	-0.054	-0.024	1.70E-59	3.42E-08
cg22713444	1	145512660	*RBM8A*	0.451	0.332	0.410	-0.118	-0.041	2.61E-179	2.00E-16
cg18814252	1	147075680	*BCL9*	0.715	0.765	0.762	0.050	0.048	7.87E-24	1.77E-10
cg07790752	1	147101904	NA	0.495	0.394	0.465	-0.101	-0.031	2.32E-91	2.19E-07
cg07600998	1	147146108	NA	0.400	0.344	0.359	-0.056	-0.041	8.75E-67	6.96E-19
cg17850088	1	150119278	NA	0.644	0.700	0.694	0.056	0.050	2.02E-37	1.03E-14
cg19736900	1	150132950	NA	0.280	0.223	0.246	-0.057	-0.034	4.18E-71	5.70E-13
cg26118326	1	150547677	*MCL1*	0.664	0.727	0.702	0.063	0.038	4.48E-54	2.21E-10
cg14827807	1	150943828	*CERS2*	0.381	0.319	0.357	-0.062	-0.023	2.11E-130	8.67E-15
cg13477111	1	150951737	NA	0.295	0.227	0.263	-0.068	-0.032	3.23E-121	3.83E-16
cg26884161	1	151303917	NA	0.761	0.806	0.811	0.046	0.050	8.73E-22	2.21E-11
cg03005293	1	151805241	*RORC*	0.346	0.272	0.317	-0.074	-0.029	7.70E-121	3.36E-14
cg11661235	1	151827551	*THEM5*	0.673	0.722	0.726	0.049	0.052	2.08E-17	3.52E-09
cg10069121	1	152009711	*S100A11*	0.339	0.281	0.303	-0.059	-0.036	4.24E-49	6.01E-09
cg08166767	1	152671348	*LCE2A*	0.653	0.711	0.708	0.058	0.055	1.14E-29	3.31E-12
cg02956542	1	153321421	*PGLYRP4*	0.340	0.259	0.301	-0.080	-0.038	2.29E-83	3.50E-11
cg03514239	1	153329781	*S100A9*	0.277	0.219	0.248	-0.059	-0.030	7.27E-130	5.77E-20
cg16139316	1	153330758	*S100A9*	0.394	0.302	0.358	-0.092	-0.036	4.56E-112	3.39E-10
cg01431057	1	153362927	*S100A8*	0.461	0.373	0.426	-0.088	-0.035	2.24E-134	1.15E-16
cg20335425	1	153363264	*S100A8*	0.384	0.323	0.365	-0.061	-0.020	1.67E-85	3.71E-07
cg20070090	1	153363489	*S100A8*	0.444	0.345	0.405	-0.098	-0.038	1.29E-121	4.04E-13
cg24898863	1	153363580	*S100A8*	0.322	0.247	0.285	-0.075	-0.037	3.44E-128	7.52E-21
cg17496887	1	153387707	*S100A7A*	0.525	0.436	0.490	-0.090	-0.035	9.51E-152	1.77E-16
cg21196487	1	153538964	*S100A2*	0.282	0.231	0.255	-0.051	-0.027	8.63E-47	5.22E-07
cg08129092	1	153746211	*INTS3*	0.253	0.190	0.205	-0.064	-0.048	7.07E-69	9.61E-20
cg00421624	1	153746588	*SLC27A3*	0.328	0.262	0.294	-0.066	-0.035	2.41E-59	1.00E-08
cg01402255	1	153800699	*GATAD2B*	0.431	0.332	0.393	-0.099	-0.037	4.63E-138	4.04E-12
cg26336059	1	153958977	*RAB13*	0.318	0.232	0.290	-0.086	-0.029	2.65E-136	2.78E-11
cg16586406	1	154164994	*TPM3*	0.341	0.259	0.297	-0.082	-0.045	1.52E-130	9.77E-25
cg00791854	1	154392070	*IL6R*	0.291	0.233	0.262	-0.058	-0.029	1.16E-74	1.85E-10
cg21262032	1	154437693	*IL6R*	0.696	0.746	0.730	0.051	0.034	1.89E-47	6.35E-13
cg12473916	1	154943651	*SHC1*	0.346	0.296	0.314	-0.050	-0.031	3.30E-72	2.76E-17
cg10446869	1	155147431	*TRIM46*	0.368	0.303	0.340	-0.065	-0.028	5.21E-54	3.88E-06
cg16416987	1	155177561	*MTX1*	0.361	0.284	0.324	-0.078	-0.037	5.12E-153	3.32E-22
cg11951169	1	155470302	*ASH1L*	0.646	0.697	0.689	0.051	0.042	2.85E-24	1.40E-08
cg21284779	1	155910526	*RXFP4*	0.392	0.337	0.369	-0.056	-0.024	3.95E-90	1.18E-13
cg16310095	1	155952959	NA	0.406	0.354	0.385	-0.052	-0.021	7.09E-87	1.40E-08
cg05954120	1	156254757	*TMEM79*	0.426	0.334	0.389	-0.092	-0.038	2.20E-163	1.46E-20
cg08469215	1	156261351	*TMEM79*	0.572	0.521	0.549	-0.052	-0.023	3.67E-41	4.26E-06
cg12617080	1	156509844	*IQGAP3*	0.362	0.306	0.326	-0.055	-0.036	2.95E-108	1.40E-28
cg12126706	1	156889128	*LRRC71*	0.424	0.341	0.383	-0.083	-0.041	3.36E-56	9.33E-10
cg16374333	1	157103641	*ETV3*	0.754	0.816	0.780	0.063	0.026	2.40E-91	8.52E-10
cg00699569	1	157536296	NA	0.377	0.297	0.342	-0.080	-0.035	1.20E-97	4.02E-11
cg00995854	1	157802305	*CD5L*	0.434	0.343	0.391	-0.091	-0.043	1.28E-84	2.77E-11
cg10951380	1	158029978	*KIRREL*	0.488	0.429	0.454	-0.059	-0.035	9.95E-42	2.05E-09
cg13765621	1	158149228	*CD1D*	0.326	0.249	0.286	-0.078	-0.041	8.65E-89	1.56E-11
cg18234111	1	158155757	*CD1D*	0.269	0.217	0.222	-0.052	-0.047	1.01E-55	4.72E-19
cg15593510	1	158369112	*OR10T2*	0.773	0.826	0.795	0.053	0.022	7.90E-88	4.39E-09
cg10636246	1	159046973	*AIM2*	0.368	0.303	0.302	-0.064	-0.066	1.87E-41	7.58E-20
cg24642483	1	159261560	*FCER1A*	0.523	0.465	0.490	-0.059	-0.033	1.76E-63	3.90E-14
cg15076824	1	159685185	*CRP*	0.699	0.750	0.733	0.051	0.034	2.90E-32	5.92E-08
cg22602513	1	160429714	NA	0.689	0.750	0.743	0.061	0.054	7.58E-33	1.22E-12
cg18920397	1	160765805	*LY9*	0.587	0.678	0.621	0.090	0.034	5.37E-176	3.70E-22
cg01367992	1	160766535	*LY9*	0.783	0.838	0.808	0.056	0.025	1.01E-89	9.54E-10
cg11939496	1	160833560	*CD244*	0.572	0.484	0.539	-0.088	-0.033	1.56E-94	2.90E-10
cg04891053	1	161053558	*PVRL4*	0.439	0.365	0.404	-0.074	-0.035	2.23E-103	5.16E-17

cg17833106	1	161060000	*PVRL4*	0.465	0.369	0.420	-0.097	-0.045	1.21E-200	8.86E-31
cg17013990	1	161091682	*DEDD*	0.404	0.306	0.363	-0.099	-0.041	2.40E-135	2.41E-15
cg17904988	1	161168451	*NDUFS2*	0.326	0.252	0.288	-0.073	-0.037	4.81E-114	5.41E-16
cg24049880	1	161171211	*NDUFS2*	0.277	0.224	0.248	-0.053	-0.029	4.65E-64	4.06E-10
cg07356342	1	161183820	*NDUFS2*	0.428	0.332	0.390	-0.096	-0.037	3.42E-180	8.71E-20
cg20609803	1	161184305	*FCER1G*	0.427	0.320	0.378	-0.106	-0.049	2.54E-177	4.77E-25
cg20806175	1	161186839	*FCER1G*	0.348	0.274	0.319	-0.074	-0.029	1.13E-135	3.87E-15
cg00974864	1	161601053	*FCGR3B*	0.390	0.309	0.356	-0.081	-0.034	2.57E-140	4.52E-18
cg13400077	1	162168081	*NOS1AP*	0.696	0.754	0.733	0.059	0.037	7.92E-39	7.42E-08
cg13178361	1	162532502	*UAP1*	0.513	0.402	0.467	-0.112	-0.047	7.32E-180	2.56E-21
cg10972897	1	165859714	*UCK2*	0.404	0.322	0.371	-0.082	-0.033	2.12E-129	2.96E-12
cg26792900	1	167074894	*DUSP27*	0.335	0.257	0.291	-0.078	-0.044	9.15E-113	4.06E-21
cg23114435	1	167442612	*CD247*	0.597	0.692	0.637	0.095	0.040	5.43E-167	1.11E-22
cg03727968	1	167451830	*CD247*	0.761	0.819	0.793	0.058	0.032	3.37E-78	8.97E-13
cg09473725	1	167485873	*CD247*	0.722	0.779	0.747	0.057	0.025	1.80E-61	2.08E-06
cg09032544	1	167487295	*CD247*	0.681	0.763	0.711	0.082	0.030	1.86E-118	5.18E-11
cg09554443	1	167487762	*CD247*	0.386	0.455	0.417	0.069	0.031	1.61E-51	2.42E-08
cg04846203	1	167690438	*MPZL1*	0.332	0.234	0.280	-0.098	-0.052	5.93E-124	2.37E-20
cg01565130	1	167690726	*MPZL1*	0.357	0.268	0.317	-0.089	-0.040	2.73E-95	2.03E-10
cg15210526	1	167733310	*MPZL1*	0.519	0.405	0.484	-0.114	-0.035	3.25E-116	8.64E-09
cg11689744	1	170525537	NA	0.643	0.667	0.696	0.024	0.053	2.20E-06	7.97E-12
cg12437809	1	171153544	*FMO2*	0.624	0.682	0.680	0.058	0.056	2.79E-27	1.02E-12
cg14028598	1	171714300	NA	0.590	0.465	0.548	-0.124	-0.042	8.83E-152	1.18E-14
cg16696002	1	172189728	*DNM3*	0.413	0.355	0.387	-0.058	-0.027	4.27E-51	4.35E-06
cg17591816	1	172239092	*DNM3*	0.526	0.428	0.490	-0.098	-0.036	6.40E-107	1.26E-10
cg00501765	1	172410217	*C1orf105*	0.506	0.410	0.471	-0.096	-0.035	1.12E-181	1.71E-18
cg06963192	1	172557938	*SUCO*	0.731	0.781	0.778	0.051	0.047	1.41E-27	7.16E-12
cg17756730	1	172608644	NA	0.644	0.733	0.679	0.088	0.035	1.08E-177	5.26E-19
cg10161121	1	172628020	*FASLG*	0.757	0.818	0.785	0.061	0.028	2.30E-99	1.12E-11
cg06983746	1	172628065	*FASLG*	0.685	0.741	0.713	0.057	0.029	8.06E-69	1.91E-09
cg00071250	1	172628263	*FASLG*	0.693	0.770	0.718	0.078	0.026	1.34E-141	7.27E-11
cg09183124	1	173092323	NA	0.460	0.363	0.426	-0.097	-0.034	7.14E-89	1.45E-07
cg15112923	1	173155033	*TNFSF4*	0.704	0.756	0.760	0.052	0.057	3.36E-22	2.02E-12
cg21876925	1	173175328	*TNFSF4*	0.269	0.210	0.239	-0.059	-0.031	8.70E-53	1.37E-07
cg03945538	1	173447427	*PRDX6*	0.415	0.320	0.365	-0.095	-0.050	4.21E-170	9.31E-34
cg05702218	1	174843909	*RABGAP1L*	0.412	0.355	0.374	-0.057	-0.038	3.06E-27	3.80E-06
cg16651347	1	175042297	*TNN*	0.404	0.314	0.372	-0.090	-0.032	1.06E-166	1.49E-18
cg10620680	1	178025100	NA	0.309	0.238	0.276	-0.071	-0.032	2.97E-80	2.42E-09
cg19399532	1	178512495	*C1orf220*	0.305	0.248	0.283	-0.057	-0.022	4.20E-103	8.51E-10
cg12486710	1	178512616	*C1orf220*	0.414	0.354	0.384	-0.059	-0.030	1.17E-62	1.17E-08
cg24448340	1	179921042	NA	0.414	0.301	0.354	-0.113	-0.060	4.13E-163	2.43E-28
cg18278694	1	179945973	*CEP350*	0.428	0.346	0.393	-0.082	-0.035	1.90E-109	1.71E-11
cg01022200	1	180881086	*KIAA1614*	0.277	0.218	0.252	-0.058	-0.025	5.33E-64	2.91E-06
cg12903530	1	181097897	NA	0.456	0.398	0.426	-0.059	-0.030	9.82E-120	1.26E-20
cg25727853	1	181098609	NA	0.454	0.402	0.424	-0.052	-0.030	7.20E-60	5.24E-11
cg22622057	1	182053203	NA	0.248	0.194	0.226	-0.054	-0.022	2.54E-86	6.37E-09
cg26546113	1	182810711	*DHX9*	0.667	0.735	0.705	0.068	0.038	7.25E-62	5.00E-10
cg10208303	1	182959540	NA	0.761	0.805	0.819	0.045	0.058	1.83E-16	2.29E-12
cg09472600	1	183537770	*NCF2*	0.333	0.258	0.304	-0.075	-0.029	8.09E-80	2.36E-07
cg13222915	1	184598594	NA	0.516	0.466	0.489	-0.050	-0.027	1.25E-56	7.95E-10
cg07234097	1	184761873	*FAM129A*	0.316	0.252	0.290	-0.063	-0.026	1.88E-100	8.64E-12
cg04231677	1	184808004	*FAM129A*	0.394	0.326	0.365	-0.068	-0.029	9.22E-79	2.58E-09
cg12141056	1	184944785	*FAM129A*	0.736	0.790	0.769	0.053	0.033	4.76E-54	2.12E-10
cg15385476	1	196112818	NA	0.736	0.788	0.780	0.053	0.044	1.16E-32	8.06E-11
cg12799049	1	197811487	NA	0.345	0.285	0.311	-0.061	-0.034	4.55E-59	1.35E-09
cg19312427	1	198174503	*NEK7*	0.407	0.331	0.380	-0.076	-0.027	2.12E-86	2.12E-06
cg25408950	1	198290080	*NEK7*	0.390	0.307	0.345	-0.083	-0.045	1.62E-97	5.12E-17
cg27058077	1	200337470	*C1orf98*	0.307	0.249	0.289	-0.059	-0.019	9.52E-82	3.53E-06
cg07586235	1	200385219	NA	0.423	0.337	0.397	-0.086	-0.026	8.45E-90	3.51E-06
cg18019037	1	200409325	NA	0.753	0.794	0.803	0.041	0.050	1.98E-13	5.18E-09
cg11721610	1	200580225	*KIF14*	0.347	0.273	0.315	-0.074	-0.032	7.06E-84	1.53E-08
cg03040292	1	200847096	NA	0.692	0.768	0.724	0.076	0.032	3.61E-123	6.84E-18
cg06834507	1	200876957	*C1orf106*	0.308	0.246	0.272	-0.062	-0.036	4.71E-113	1.82E-23
cg01523881	1	200985689	*KIF21B*	0.297	0.239	0.267	-0.058	-0.030	1.12E-64	6.21E-09
cg22534374	1	201511610	NA	0.638	0.579	0.605	-0.059	-0.033	6.32E-34	6.72E-07
cg03471150	1	201797198	*IPO9*	0.501	0.430	0.457	-0.071	-0.044	8.90E-100	2.87E-20
cg17932662	1	202123442	*PTPN7*	0.501	0.393	0.467	-0.107	-0.033	9.02E-160	2.56E-12
cg24880665	1	202772164	*KDM5B*	0.531	0.426	0.490	-0.105	-0.042	9.03E-105	1.59E-09
cg05455036	1	202828149	NA	0.402	0.308	0.351	-0.094	-0.051	1.60E-178	1.48E-34
cg02769705	1	202975059	*LOC401980*	0.487	0.385	0.448	-0.102	-0.038	5.07E-107	1.16E-10
cg09122593	1	203020141	*PPFIA4*	0.319	0.262	0.282	-0.057	-0.037	1.08E-105	3.63E-26
cg19081101	1	203156625	*CHI3L1*	0.466	0.354	0.436	-0.112	-0.030	5.67E-128	6.22E-07
cg22848646	1	203171499	NA	0.467	0.362	0.415	-0.105	-0.052	1.60E-121	1.10E-17
cg25705508	1	203199023	*CHIT1*	0.441	0.360	0.418	-0.081	-0.023	9.00E-127	4.83E-07
cg01798157	1	203276595	*BTG2*	0.384	0.466	0.422	0.082	0.038	1.48E-38	4.75E-06
cg23893629	1	203733971	*LAX1*	0.710	0.780	0.749	0.070	0.040	1.17E-72	7.13E-13
cg06958535	1	203734478	*LAX1*	0.638	0.733	0.677	0.095	0.039	1.06E-139	2.45E-14
cg02573357	1	203738799	*LAX1*	0.370	0.280	0.325	-0.089	-0.044	5.01E-125	4.19E-20

cg26895569	1	204329222	*PLEKHA6*	0.324	0.259	0.296	-0.065	-0.029	9.61E-71	9.50E-08
cg20240347	1	204465584	NA	0.555	0.616	0.593	0.062	0.038	1.04E-36	1.06E-08
cg24450653	1	205060293	*RBBP5*	0.594	0.535	0.570	-0.059	-0.024	9.01E-82	8.69E-08
cg17889831	1	205181581	*DSTYK*	0.384	0.297	0.341	-0.087	-0.043	2.97E-98	9.95E-15
cg24606240	1	205720650	*NUCKS1*	0.653	0.701	0.705	0.048	0.052	9.52E-23	4.80E-12
cg16031515	1	205743344	*RAB7L1*	0.637	0.690	0.679	0.052	0.042	6.09E-20	4.28E-07
cg08128734	1	206685423	*RASSF5*	0.641	0.602	0.573	-0.039	-0.068	3.52E-12	6.92E-18
cg06949439	1	206729034	*RASSF5*	0.587	0.654	0.611	0.067	0.024	2.20E-58	3.33E-06
cg01442843	1	206729040	*RASSF5*	0.718	0.782	0.743	0.064	0.025	4.09E-100	4.41E-09
cg02035751	1	206729099	*RASSF5*	0.679	0.752	0.713	0.072	0.034	1.71E-114	3.99E-19
cg17220933	1	206729613	*RASSF5*	0.687	0.746	0.706	0.059	0.019	3.12E-93	1.35E-06
cg05756492	1	206729685	*RASSF5*	0.689	0.752	0.710	0.063	0.021	2.20E-86	4.37E-06
cg06650246	1	206897270	*MAPKAPK2*	0.354	0.271	0.331	-0.083	-0.023	2.24E-146	5.37E-08
cg10978799	1	206945924	*IL10*	0.327	0.274	0.287	-0.054	-0.040	7.27E-56	8.78E-16
cg17744604	1	206946166	*IL10*	0.399	0.308	0.359	-0.092	-0.041	1.68E-92	3.19E-11
cg14180511	1	206946187	*IL10*	0.419	0.327	0.363	-0.092	-0.056	2.72E-103	8.40E-23
cg12073436	1	206958014	NA	0.619	0.707	0.654	0.088	0.035	9.58E-98	7.88E-09
cg12380764	1	206971195	*IL19*	0.408	0.311	0.368	-0.097	-0.041	2.94E-110	1.55E-12
cg17100176	1	207096358	*FAIM3*	0.597	0.676	0.626	0.079	0.028	1.84E-106	6.23E-10
cg02233614	1	207227965	*PFKFB2*	0.255	0.196	0.228	-0.059	-0.027	4.37E-80	1.08E-09
cg01710147	1	207233419	*PFKFB2*	0.380	0.299	0.344	-0.081	-0.036	2.93E-119	8.94E-15
cg22491058	1	207277466	*C4BPA*	0.416	0.306	0.368	-0.110	-0.048	6.29E-152	2.43E-19
cg14856606	1	207277547	*C4BPA*	0.341	0.284	0.313	-0.058	-0.028	5.60E-59	4.57E-08
cg22687766	1	207490952	NA	0.430	0.369	0.395	-0.061	-0.035	5.17E-63	1.19E-12
cg10501210	1	207997020	NA	0.638	0.598	0.553	-0.040	-0.085	9.37E-12	8.16E-22
cg24315421	1	208040253	NA	0.250	0.183	0.223	-0.067	-0.027	8.66E-65	2.36E-06
cg09788693	1	208063733	*CD34*	0.238	0.187	0.205	-0.051	-0.032	2.41E-60	2.02E-11
cg21697512	1	208081541	*CD34*	0.530	0.435	0.481	-0.096	-0.050	2.69E-121	3.03E-19
cg16015816	1	209568242	NA	0.715	0.766	0.758	0.051	0.043	3.22E-24	1.69E-08
cg07168232	1	209822928	*LAMB3*	0.439	0.376	0.403	-0.063	-0.036	2.73E-93	1.68E-16
cg07434244	1	209847729	*G0S2*	0.337	0.278	0.315	-0.060	-0.022	9.52E-123	2.10E-11
cg16395183	1	210771699	*HHAT*	0.516	0.457	0.486	-0.059	-0.030	1.58E-48	1.38E-06
cg09825327	1	211503219	*TRAF5*	0.696	0.770	0.727	0.074	0.031	2.71E-124	1.09E-14
cg02922776	1	212420632	NA	0.384	0.315	0.352	-0.069	-0.032	1.86E-62	1.91E-07
cg02343275	1	212589072	*TMEM206*	0.294	0.237	0.264	-0.056	-0.030	4.06E-78	2.14E-11
cg06873024	1	215130954	NA	0.417	0.358	0.370	-0.060	-0.048	2.47E-49	5.63E-14
cg19631762	1	218698561	NA	0.260	0.202	0.227	-0.059	-0.034	6.48E-64	2.46E-10
cg01603234	1	218990709	NA	0.765	0.805	0.816	0.040	0.051	3.32E-17	3.84E-12
cg07368265	1	219375142	*LYPLAL1*	0.630	0.667	0.681	0.036	0.050	7.54E-13	4.46E-10
cg09255910	1	221055790	*HLX*	0.477	0.394	0.451	-0.082	-0.025	2.72E-136	5.97E-10
cg17852326	1	221055964	*HLX*	0.414	0.333	0.379	-0.081	-0.035	5.68E-114	8.03E-14
cg12129117	1	223940656	*CAPN2*	0.393	0.322	0.371	-0.071	-0.022	1.21E-124	1.19E-11
cg15134583	1	224015075	*TP53BP2*	0.382	0.292	0.342	-0.089	-0.040	1.72E-128	2.54E-15
cg03048947	1	225611366	*LBR*	0.714	0.767	0.746	0.053	0.032	2.42E-33	1.63E-06
cg10826999	1	225862288	NA	0.458	0.349	0.416	-0.109	-0.042	4.21E-140	2.08E-14
cg14520947	1	225942842	NA	0.513	0.389	0.473	-0.123	-0.039	4.72E-142	4.86E-10
cg20334115	1	226107899	*PYCR2*	0.393	0.322	0.370	-0.070	-0.023	4.73E-98	1.17E-07
cg21422623	1	226912213	*ITPKB*	0.668	0.744	0.699	0.076	0.031	9.99E-99	2.75E-10
cg23676767	1	226918736	*ITPKB*	0.646	0.715	0.691	0.069	0.045	1.69E-28	9.06E-07
cg05306109	1	226924257	*ITPKB*	0.718	0.773	0.743	0.055	0.025	1.16E-76	3.11E-09
cg06298740	1	227125826	NA	0.421	0.312	0.366	-0.109	-0.055	1.85E-174	1.44E-28
cg06882842	1	227754452	*ZNF678*	0.724	0.774	0.777	0.050	0.053	4.56E-22	1.59E-11
cg01428095	1	228134229	*WNT9A*	0.261	0.209	0.234	-0.052	-0.027	5.93E-70	3.49E-10
cg24070990	1	228634724	NA	0.574	0.597	0.625	0.023	0.051	4.24E-07	3.33E-13
cg04570265	1	228679629	*RNF187*	0.744	0.798	0.795	0.053	0.050	5.29E-32	3.47E-14
cg25349990	1	229294380	NA	0.412	0.320	0.373	-0.092	-0.039	8.82E-100	5.16E-10
cg03123320	1	229394665	NA	0.502	0.446	0.468	-0.056	-0.034	2.44E-66	8.87E-14
cg17165759	1	229577027	NA	0.681	0.741	0.707	0.060	0.026	6.75E-60	1.65E-06
cg06706156	1	230183251	NA	0.474	0.367	0.427	-0.107	-0.047	4.83E-132	2.53E-15
cg13359998	1	230241764	*GALNT2*	0.258	0.194	0.216	-0.064	-0.041	1.00E-70	4.95E-15
cg22153407	1	230290089	*GALNT2*	0.527	0.439	0.494	-0.088	-0.033	5.60E-99	5.71E-10
cg11527913	1	230410329	*GALNT2*	0.333	0.273	0.304	-0.061	-0.029	3.07E-55	1.23E-06
cg23050705	1	231694386	*TSNAX*	0.670	0.726	0.721	0.056	0.051	5.36E-33	5.81E-13
cg23172853	1	231842206	*DISC1*	0.511	0.460	0.485	-0.052	-0.026	1.74E-36	4.77E-06
cg09548403	1	232840764	NA	0.251	0.194	0.219	-0.058	-0.032	3.69E-74	3.09E-11
cg16647844	1	232903627	NA	0.420	0.327	0.373	-0.093	-0.046	7.51E-105	3.11E-15
cg01723892	1	233004866	NA	0.370	0.278	0.328	-0.092	-0.042	1.28E-109	3.39E-13
cg07223106	1	233155919	*PCNXL2*	0.483	0.422	0.456	-0.061	-0.027	2.44E-69	2.28E-08
cg00980058	1	233248709	*PCNXL2*	0.233	0.182	0.209	-0.052	-0.025	4.34E-78	1.03E-09
cg04273604	1	234609003	*TARBP1*	0.212	0.155	0.184	-0.057	-0.028	4.38E-62	9.90E-08
cg10881749	1	234792390	NA	0.518	0.459	0.481	-0.059	-0.036	1.42E-35	8.89E-08
cg13420364	1	234857659	NA	0.451	0.396	0.409	-0.054	-0.042	2.80E-51	1.83E-15
cg10718614	1	234914480	NA	0.328	0.255	0.303	-0.074	-0.026	1.29E-156	2.21E-15
cg02889973	1	234977572	NA	0.331	0.244	0.291	-0.087	-0.041	1.77E-98	3.64E-12
cg08231648	1	235011814	NA	0.343	0.275	0.310	-0.068	-0.034	1.57E-85	7.91E-13
cg17759224	1	235098018	NA	0.280	0.225	0.245	-0.055	-0.035	1.36E-49	1.79E-09
cg26837399	1	236209798	*NID1*	0.458	0.345	0.412	-0.113	-0.046	7.29E-147	1.23E-17
cg17451941	1	236254344	NA	0.397	0.302	0.348	-0.096	-0.050	6.17E-125	4.13E-18

cg11384661	1	238025414	NA	0.323	0.254	0.292	-0.070	-0.032	7.88E-79	1.16E-08
cg08622198	1	239979505	*CHRM3*	0.444	0.349	0.410	-0.095	-0.034	1.58E-181	1.31E-18
cg23166773	1	243659016	*SDCCAG8*	0.536	0.479	0.501	-0.057	-0.034	3.83E-40	4.89E-08
cg23012600	1	244088110	*LOC339529*	0.427	0.333	0.392	-0.094	-0.035	1.21E-97	1.38E-08
cg00967073	1	244443687	NA	0.311	0.230	0.263	-0.080	-0.048	4.66E-110	4.30E-19
cg01012879	1	244613363	*ADSS*	0.616	0.547	0.584	-0.069	-0.032	1.70E-69	4.02E-09
cg09855706	1	244965648	NA	0.346	0.271	0.315	-0.075	-0.031	1.18E-79	1.08E-07
cg25025181	1	246378510	*SMYD3*	0.402	0.303	0.362	-0.099	-0.040	1.92E-119	8.03E-12
cg02952913	1	247336686	*ZNF124*	0.430	0.317	0.371	-0.113	-0.059	5.28E-126	1.64E-19
cg24404823	1	247496053	*ZNF496*	0.398	0.333	0.360	-0.065	-0.038	3.84E-77	5.24E-14
cg04020309	1	247496094	*ZNF496*	0.429	0.348	0.383	-0.080	-0.045	4.39E-89	2.10E-16
cg21824010	1	247578859	*NLRP3*	0.534	0.484	0.499	-0.050	-0.035	3.38E-56	1.90E-14
cg26112639	1	247580106	*NLRP3*	0.303	0.245	0.273	-0.058	-0.030	1.98E-103	6.84E-16
cg21991396	1	247581417	*NLRP3*	0.521	0.396	0.464	-0.125	-0.057	5.82E-184	4.22E-27
cg23230830	1	248903100	*LYPD8*	0.349	0.264	0.313	-0.085	-0.036	2.16E-81	9.55E-09
cg17056747	2	215045	NA	0.714	0.765	0.758	0.051	0.044	1.79E-22	1.63E-08
cg05464506	2	420052	NA	0.689	0.750	0.716	0.061	0.027	6.75E-107	3.55E-13
cg21664909	2	1646050	*PXDN*	0.740	0.788	0.793	0.048	0.053	9.51E-23	3.15E-12
cg05373263	2	3063115	NA	0.664	0.725	0.716	0.061	0.052	5.13E-19	2.27E-07
cg20808080	2	6962835	NA	0.727	0.793	0.773	0.066	0.046	6.34E-54	4.42E-13
cg01028142	2	7004578	*CMPK2*	0.879	0.727	0.805	-0.152	-0.073	1.24E-64	2.83E-32
cg23213327	2	7016509	*RSAD2*	0.497	0.395	0.445	-0.102	-0.052	4.69E-92	4.04E-17
cg10959651	2	7018020	*RSAD2*	0.268	0.157	0.193	-0.110	-0.075	2.92E-110	1.34E-34
cg10549986	2	7018153	*RSAD2*	0.165	0.083	0.105	-0.081	-0.060	1.41E-82	2.40E-23
cg19240637	2	7172297	*RNF144A*	0.560	0.493	0.527	-0.067	-0.033	5.27E-65	4.17E-10
cg23073974	2	8039990	NA	0.430	0.353	0.394	-0.077	-0.036	7.36E-82	1.84E-10
cg22489510	2	8675599	NA	0.447	0.361	0.408	-0.086	-0.039	6.22E-130	7.31E-17
cg00534022	2	8686087	NA	0.474	0.376	0.428	-0.098	-0.046	1.14E-135	4.05E-17
cg13231954	2	8724414	NA	0.296	0.231	0.267	-0.065	-0.029	2.14E-70	7.76E-08
cg04341343	2	8784813	NA	0.282	0.216	0.256	-0.066	-0.025	9.82E-117	2.72E-11
cg21992696	2	9125026	*MBOAT2*	0.354	0.293	0.318	-0.061	-0.036	6.74E-67	1.19E-12
cg21547649	2	9142741	*MBOAT2*	0.408	0.325	0.374	-0.083	-0.034	1.22E-120	1.27E-11
cg16903174	2	9468351	*ASAP2*	0.510	0.442	0.476	-0.067	-0.034	8.62E-71	1.20E-09
cg26306976	2	9564901	*ITGB1BP1*	0.643	0.556	0.608	-0.087	-0.036	1.39E-48	1.75E-06
cg25739938	2	9610621	*CPSF3*	0.572	0.451	0.530	-0.121	-0.042	9.59E-181	1.21E-18
cg14930864	2	9759203	*YWHAQ*	0.680	0.736	0.726	0.057	0.046	4.49E-36	6.24E-12
cg13257421	2	9987440	*TAF1B*	0.648	0.595	0.617	-0.052	-0.031	1.46E-26	1.81E-06
cg25588844	2	10037561	*TAF1B*	0.372	0.293	0.330	-0.079	-0.042	1.47E-87	1.03E-14
cg14118850	2	10447890	*HPCAL1*	0.485	0.384	0.441	-0.101	-0.044	1.75E-123	1.96E-15
cg11796910	2	11475299	*ROCK2*	0.673	0.725	0.728	0.052	0.055	4.07E-21	1.30E-10
cg12647574	2	12824729	NA	0.389	0.315	0.359	-0.074	-0.030	2.55E-103	3.58E-11
cg04104235	2	20104329	NA	0.641	0.693	0.682	0.051	0.041	1.93E-29	2.70E-09
cg13471188	2	20638399	NA	0.423	0.323	0.381	-0.100	-0.042	1.12E-129	4.84E-14
cg11227278	2	23749277	*KLHL29*	0.667	0.727	0.694	0.060	0.027	1.09E-74	2.04E-09
cg08840913	2	24139933	*ATAD2B*	0.516	0.463	0.489	-0.053	-0.027	6.32E-49	2.59E-07
cg26752663	2	25142016	*ADCY3*	0.713	0.769	0.735	0.056	0.022	1.07E-70	3.35E-07
cg00483217	2	25321917	*EFR3B*	0.448	0.365	0.419	-0.083	-0.029	1.99E-118	8.68E-09
cg02607972	2	25964061	*ASXL2*	0.511	0.396	0.464	-0.115	-0.046	7.33E-161	6.09E-19
cg02641801	2	26213508	NA	0.359	0.297	0.331	-0.063	-0.028	1.62E-68	1.28E-07
cg17959183	2	26297671	*RAB10*	0.527	0.409	0.486	-0.118	-0.041	9.39E-137	8.49E-13
cg17696006	2	26521372	NA	0.499	0.440	0.470	-0.059	-0.028	2.91E-43	1.97E-06
cg07495389	2	27233872	*MAPRE3*	0.401	0.309	0.361	-0.092	-0.040	5.77E-145	7.38E-18
cg05979400	2	27295591	*OST4*	0.317	0.253	0.288	-0.064	-0.029	2.33E-143	7.29E-22
cg15078838	2	28114349	*RBKS*	0.277	0.198	0.242	-0.079	-0.035	1.99E-89	4.74E-09
cg05057534	2	28497669	*BRE*	0.450	0.336	0.391	-0.114	-0.058	6.31E-148	2.31E-20
cg24738611	2	29149895	*WDR43*	0.386	0.310	0.358	-0.076	-0.028	5.59E-102	1.41E-09
cg21005412	2	29149902	*WDR43*	0.546	0.474	0.518	-0.072	-0.028	1.31E-97	8.97E-10
cg17250262	2	30457110	*LBH*	0.525	0.605	0.554	0.080	0.029	6.44E-152	3.86E-18
cg07214920	2	31378999	NA	0.747	0.804	0.777	0.057	0.030	5.90E-60	5.14E-08
cg01627252	2	31397283	*CAPN14*	0.474	0.373	0.432	-0.101	-0.043	5.26E-134	3.12E-16
cg19410609	2	31541350	NA	0.455	0.345	0.421	-0.110	-0.034	7.05E-116	5.24E-08
cg25124300	2	31607761	*XDH*	0.534	0.429	0.484	-0.105	-0.050	1.30E-126	2.94E-18
cg22805603	2	32490766	*NLRC4*	0.481	0.389	0.452	-0.092	-0.029	7.55E-176	5.64E-14
cg07055315	2	32490835	*NLRC4*	0.446	0.340	0.405	-0.106	-0.041	3.15E-121	4.23E-12
cg15135286	2	33359281	*LTBP1*	0.480	0.376	0.436	-0.105	-0.044	3.56E-154	9.57E-18
cg13652008	2	33359356	*LTBP1*	0.554	0.444	0.512	-0.110	-0.041	1.17E-144	3.56E-14
cg03906115	2	33359529	*LTBP1*	0.369	0.267	0.318	-0.102	-0.051	1.41E-95	4.33E-13
cg08357990	2	33359550	*LTBP1*	0.205	0.151	0.166	-0.054	-0.039	1.06E-52	1.96E-11
cg14102437	2	33359688	*LTBP1*	0.402	0.300	0.346	-0.102	-0.057	9.20E-123	5.71E-22
cg18200741	2	33780366	*RASGRP3*	0.298	0.246	0.273	-0.051	-0.025	7.43E-62	1.00E-08
cg21932231	2	36668864	*CRIM1*	0.508	0.427	0.467	-0.081	-0.041	9.37E-64	3.93E-11
cg22957691	2	36790185	*FEZ2*	0.495	0.376	0.455	-0.119	-0.041	2.58E-136	1.63E-11
cg17326313	2	37383568	*EIF2AK2*	0.156	0.087	0.112	-0.068	-0.044	5.37E-132	2.51E-28
cg14126601	2	37384708	*EIF2AK2*	0.495	0.412	0.435	-0.082	-0.060	1.65E-45	5.59E-12
cg00841141	2	37416819	*SULT6B1*	0.522	0.426	0.491	-0.096	-0.032	4.38E-121	1.87E-09
cg05627557	2	37418009	NA	0.377	0.295	0.346	-0.082	-0.031	2.62E-170	3.68E-16
cg07671644	2	37545668	*PRKD3*	0.312	0.246	0.283	-0.066	-0.030	3.26E-74	6.05E-08
cg19190900	2	37553621	NA	0.339	0.262	0.297	-0.077	-0.042	3.43E-110	1.64E-17

cg13286582	2	37883934	*CDC42EP3*	0.568	0.628	0.605	0.060	0.038	2.72E-62	1.34E-13
cg26750893	2	38043481	NA	0.616	0.528	0.564	-0.088	-0.052	4.97E-88	4.54E-19
cg18107006	2	38831166	*HNRPLL*	0.334	0.256	0.287	-0.078	-0.047	2.66E-93	6.57E-18
cg10898024	2	40266366	*SLC8A1-AS1*	0.379	0.303	0.346	-0.076	-0.033	1.04E-64	2.93E-08
cg22409100	2	40658918	*SLC8A1*	0.347	0.273	0.305	-0.073	-0.042	4.82E-102	1.76E-19
cg08178956	2	42345201	NA	0.368	0.293	0.338	-0.076	-0.031	1.81E-79	2.31E-07
cg09535475	2	42444827	*EML4*	0.510	0.405	0.480	-0.105	-0.031	1.97E-107	8.12E-07
cg10588617	2	43027940	NA	0.564	0.614	0.594	0.050	0.030	1.17E-50	4.84E-09
cg18739675	2	43041125	NA	0.303	0.231	0.260	-0.071	-0.043	3.80E-78	1.52E-15
cg17517296	2	43107458	NA	0.434	0.350	0.403	-0.085	-0.032	2.77E-137	1.70E-13
cg01479187	2	43158610	NA	0.367	0.271	0.323	-0.095	-0.044	5.33E-138	9.72E-15
cg23008718	2	43188851	NA	0.320	0.252	0.297	-0.068	-0.023	1.83E-101	5.57E-08
cg03717364	2	43202481	NA	0.286	0.224	0.255	-0.062	-0.031	3.47E-55	3.53E-08
cg12542656	2	43269469	NA	0.376	0.324	0.348	-0.052	-0.028	1.17E-49	6.88E-08
cg19220272	2	43312367	NA	0.284	0.234	0.261	-0.050	-0.023	1.57E-70	2.87E-08
cg09489567	2	43364053	NA	0.597	0.658	0.624	0.062	0.027	5.81E-117	3.77E-14
cg09308580	2	43405947	NA	0.378	0.323	0.351	-0.056	-0.028	5.07E-73	1.50E-10
cg21473786	2	44311918	NA	0.518	0.435	0.482	-0.083	-0.036	4.16E-107	7.26E-12
cg02059176	2	44327637	NA	0.376	0.304	0.341	-0.072	-0.034	4.37E-69	7.37E-09
cg10279922	2	45347089	NA	0.715	0.770	0.757	0.055	0.043	9.85E-42	1.36E-12
cg22284398	2	46119607	*PRKCE*	0.288	0.219	0.256	-0.070	-0.033	2.84E-117	2.15E-16
cg25941751	2	46613544	*EPAS1*	0.399	0.305	0.362	-0.093	-0.036	2.89E-124	1.13E-11
cg27485921	2	46747379	*ATP6V1E2*	0.377	0.295	0.347	-0.082	-0.030	1.08E-118	1.08E-09
cg16097858	2	46765700	NA	0.279	0.227	0.245	-0.053	-0.034	3.65E-56	5.01E-11
cg10818566	2	47197798	*TTC7A*	0.583	0.530	0.554	-0.053	-0.029	1.13E-41	6.60E-07
cg20090162	2	47261900	*TTC7A*	0.442	0.356	0.421	-0.086	-0.022	4.86E-113	2.75E-06
cg04781916	2	48013473	*MSH6*	0.693	0.598	0.656	-0.095	-0.036	6.97E-73	8.12E-09
cg22928999	2	48046329	*FBXO11*	0.789	0.835	0.843	0.046	0.054	2.98E-17	9.99E-12
cg24608504	2	54760330	*SPTBN1*	0.503	0.395	0.466	-0.108	-0.037	6.09E-136	8.26E-12
cg03664994	2	55246602	*RTN4*	0.425	0.331	0.391	-0.093	-0.034	2.87E-105	4.92E-10
cg00010672	2	55281781	NA	0.703	0.774	0.749	0.070	0.045	4.77E-44	6.63E-09
cg15742777	2	55339218	NA	0.238	0.182	0.206	-0.057	-0.033	9.25E-92	6.13E-19
cg05403316	2	55339939	NA	0.312	0.238	0.280	-0.074	-0.032	2.71E-119	1.12E-15
cg13359689	2	55636555	*CCDC88A*	0.380	0.324	0.351	-0.056	-0.029	1.92E-96	2.62E-16
cg25412594	2	56152003	*EFEMP1*	0.739	0.785	0.790	0.046	0.051	1.40E-20	6.59E-11
cg07227049	2	58335008	*VRK2*	0.576	0.451	0.535	-0.124	-0.041	1.45E-152	1.07E-14
cg21406144	2	59919051	NA	0.568	0.488	0.543	-0.080	-0.026	2.08E-101	7.07E-07
cg11690884	2	60533586	NA	0.308	0.238	0.269	-0.070	-0.039	1.73E-99	1.40E-16
cg02334333	2	60687392	*BCL11A*	0.389	0.311	0.354	-0.078	-0.034	1.56E-144	8.79E-17
cg17935536	2	60755743	*BCL11A*	0.371	0.298	0.345	-0.073	-0.025	1.20E-135	1.06E-11
cg02054108	2	61607478	*USP34*	0.637	0.551	0.605	-0.085	-0.032	2.75E-96	6.03E-10
cg27360727	2	62408302	NA	0.309	0.253	0.283	-0.056	-0.026	9.74E-93	4.51E-12
cg17253709	2	62442007	*B3GNT2*	0.403	0.341	0.370	-0.062	-0.032	8.40E-63	5.59E-09
cg12627844	2	64245000	*VPS54*	0.522	0.471	0.480	-0.051	-0.042	2.77E-35	2.11E-12
cg08471738	2	64373042	*PELI1*	0.589	0.656	0.628	0.067	0.039	3.09E-53	1.43E-10
cg26164488	2	64440295	NA	0.382	0.277	0.324	-0.105	-0.058	7.48E-153	2.80E-29
cg15132282	2	64488961	NA	0.385	0.284	0.334	-0.101	-0.051	2.72E-105	1.55E-14
cg06233202	2	64501134	NA	0.302	0.235	0.273	-0.067	-0.029	6.14E-116	2.20E-13
cg22737154	2	64631614	NA	0.506	0.434	0.452	-0.071	-0.053	2.72E-65	2.13E-19
cg14209730	2	64632636	NA	0.637	0.532	0.589	-0.105	-0.048	3.27E-106	2.67E-17
cg08241318	2	64885581	NA	0.413	0.329	0.378	-0.084	-0.035	4.96E-103	5.32E-11
cg22900266	2	65089000	NA	0.471	0.403	0.447	-0.068	-0.024	3.07E-88	1.27E-08
cg25413977	2	66651619	*MEIS1-AS3*	0.566	0.513	0.513	-0.053	-0.053	6.11E-21	9.71E-11
cg01271812	2	66671478	*MEIS1*	0.340	0.276	0.307	-0.064	-0.033	4.34E-44	5.22E-06
cg09535924	2	66671659	*MEIS1*	0.308	0.255	0.278	-0.053	-0.030	4.82E-54	4.85E-10
cg12082609	2	66671727	*MEIS1*	0.351	0.288	0.317	-0.063	-0.034	1.94E-43	2.90E-07
cg13468685	2	68592737	*PLEK*	0.374	0.317	0.333	-0.056	-0.040	1.90E-39	2.23E-09
cg16761097	2	68897545	NA	0.701	0.758	0.749	0.057	0.048	5.12E-30	1.63E-10
cg18670846	2	68917482	NA	0.259	0.201	0.231	-0.058	-0.028	1.57E-64	1.58E-08
cg15700582	2	68960656	*ARHGAP25*	0.362	0.280	0.320	-0.082	-0.042	2.34E-114	4.69E-16
cg20874031	2	69206176	*GKN1*	0.630	0.698	0.670	0.069	0.041	6.06E-46	1.21E-08
cg13358873	2	69271042	*ANTXR1*	0.475	0.410	0.444	-0.065	-0.031	2.67E-58	2.37E-07
cg27490875	2	69418131	*ANTXR1*	0.582	0.633	0.615	0.051	0.033	3.16E-26	8.31E-07
cg04834204	2	69423553	*ANTXR1*	0.475	0.392	0.447	-0.083	-0.028	1.33E-81	2.76E-06
cg04075726	2	69499425	NA	0.483	0.390	0.454	-0.093	-0.029	1.44E-94	1.08E-06
cg24171555	2	69852693	*AAK1*	0.444	0.383	0.420	-0.061	-0.024	4.09E-69	4.19E-06
cg25929198	2	69867834	*AAK1*	0.725	0.777	0.748	0.052	0.023	1.15E-72	3.92E-08
cg22792910	2	70009351	*ANXA4*	0.313	0.260	0.283	-0.053	-0.030	2.29E-51	1.09E-08
cg00901982	2	70257298	*PCBP1-AS1*	0.404	0.303	0.357	-0.101	-0.047	2.16E-107	2.29E-16
cg25420477	2	70319121	NA	0.690	0.765	0.721	0.074	0.030	1.95E-108	2.72E-11
cg07586956	2	70336043	NA	0.746	0.810	0.783	0.064	0.037	2.05E-80	4.54E-15
cg12244275	2	70355397	NA	0.226	0.167	0.190	-0.059	-0.036	5.04E-100	1.41E-19
cg23549571	2	71409816	NA	0.687	0.743	0.726	0.056	0.040	1.06E-28	1.54E-07
cg21852792	2	71678463	NA	0.445	0.354	0.417	-0.091	-0.028	9.66E-89	2.06E-06
cg17639959	2	73297338	*SFXN5*	0.387	0.316	0.352	-0.071	-0.035	2.99E-104	1.34E-14
cg17329648	2	73297389	*SFXN5*	0.359	0.299	0.328	-0.060	-0.031	3.86E-48	7.11E-07
cg19445690	2	74210890	NA	0.366	0.311	0.345	-0.055	-0.021	1.37E-69	9.17E-07
cg11236515	2	74213762	NA	0.753	0.815	0.774	0.062	0.021	4.39E-109	7.57E-09

cg11525409	2	74236068	NA	0.519	0.427	0.486	-0.092	-0.033	5.17E-123	2.00E-12
cg01987702	2	74264707	NA	0.264	0.213	0.231	-0.051	-0.033	7.25E-44	1.56E-09
cg22989958	2	74783039	*DOK1*	0.433	0.361	0.402	-0.072	-0.032	3.68E-79	3.14E-09
cg05644602	2	74795186	*C2orf65*	0.578	0.496	0.548	-0.082	-0.030	1.27E-75	9.60E-07
cg18638581	2	75059602	*HK2*	0.350	0.279	0.319	-0.071	-0.031	8.01E-119	3.09E-13
cg27049094	2	75067716	*HK2*	0.500	0.560	0.547	0.060	0.047	1.60E-32	6.68E-13
cg23219064	2	84171179	NA	0.771	0.807	0.822	0.036	0.051	1.44E-16	3.35E-14
cg23881653	2	84199313	NA	0.785	0.834	0.836	0.049	0.050	1.65E-22	5.71E-11
cg16256243	2	85648512	NA	0.483	0.381	0.441	-0.102	-0.041	2.18E-155	1.19E-16
cg04213390	2	85743934	NA	0.499	0.388	0.463	-0.112	-0.037	2.64E-137	1.86E-11
cg27432242	2	85777747	*GGCX*	0.374	0.299	0.347	-0.075	-0.027	2.66E-85	2.08E-07
cg03682581	2	85832389	*C2orf68*	0.430	0.332	0.386	-0.098	-0.045	7.01E-99	8.36E-12
cg08079596	2	85999628	*ATOH8*	0.232	0.178	0.202	-0.054	-0.030	3.41E-53	1.51E-09
cg09113070	2	86114036	*ST3GAL5*	0.443	0.377	0.412	-0.066	-0.031	1.09E-71	1.13E-08
cg22094163	2	86703710	*KDM3A*	0.545	0.598	0.586	0.053	0.040	9.79E-20	4.65E-06
cg23230929	2	87883547	NA	0.509	0.458	0.478	-0.051	-0.031	3.87E-55	8.71E-12
cg26563141	2	88124876	*RGPD1*	0.507	0.555	0.565	0.048	0.058	1.84E-11	5.74E-08
cg13172001	2	88390175	*SMYD1*	0.668	0.721	0.728	0.053	0.060	8.80E-24	4.61E-14
cg10323490	2	88469007	*THNSL2*	0.561	0.608	0.616	0.047	0.054	5.82E-14	7.33E-09
cg08468732	2	95722029	NA	0.658	0.716	0.685	0.058	0.026	5.97E-78	7.13E-10
cg00094734	2	96830479	NA	0.698	0.751	0.723	0.053	0.025	2.98E-62	6.98E-08
cg22880770	2	97166341	*NEURL3*	0.603	0.645	0.664	0.042	0.061	3.26E-13	1.24E-12
cg13355542	2	97199248	NA	0.323	0.252	0.291	-0.071	-0.032	4.83E-107	3.04E-13
cg04722215	2	97205147	*ARID5A*	0.589	0.664	0.627	0.075	0.038	5.59E-100	9.29E-16
cg20312012	2	97331035	*FER1L5*	0.481	0.366	0.439	-0.115	-0.042	2.07E-142	1.48E-14
cg00423969	2	97359879	*FER1L5*	0.305	0.253	0.281	-0.052	-0.024	3.56E-76	5.49E-10
cg15009198	2	97429502	*CNNM4*	0.373	0.295	0.343	-0.077	-0.030	4.75E-74	8.41E-07
cg14187813	2	97651611	*FAM178B*	0.613	0.667	0.677	0.054	0.064	1.33E-16	5.77E-11
cg25564535	2	98329463	*ZAP70*	0.709	0.764	0.727	0.055	0.018	5.50E-74	3.21E-06
cg08859278	2	98329691	*ZAP70*	0.661	0.736	0.694	0.075	0.032	3.72E-119	5.88E-15
cg12221689	2	98827983	*VWA3B*	0.522	0.591	0.562	0.069	0.041	1.31E-30	4.64E-06
cg03310038	2	98852704	*VWA3B*	0.783	0.822	0.834	0.039	0.052	2.79E-15	9.41E-12
cg16469046	2	99062999	*INPP4A*	0.703	0.771	0.745	0.068	0.042	2.38E-76	5.10E-15
cg18267330	2	99095277	*INPP4A*	0.693	0.747	0.746	0.054	0.053	1.03E-21	4.55E-09
cg20923498	2	99096920	*INPP4A*	0.714	0.770	0.744	0.057	0.030	1.95E-74	2.19E-11
cg13584531	2	99280070	*MGAT4A*	0.740	0.799	0.763	0.059	0.023	1.13E-102	2.21E-10
cg02592133	2	99526035	*KIAA1211L*	0.268	0.210	0.248	-0.058	-0.020	8.39E-80	4.53E-06
cg24654185	2	99812274	*MRPL30*	0.698	0.745	0.758	0.047	0.061	9.06E-21	2.56E-14
cg02796420	2	100016281	*EIF5B*	0.762	0.806	0.821	0.044	0.059	7.65E-16	1.74E-14
cg14057383	2	100636602	*AFF3*	0.640	0.570	0.618	-0.071	-0.022	3.03E-91	7.01E-07
cg27315109	2	100677411	*AFF3*	0.763	0.814	0.786	0.051	0.023	1.34E-70	8.49E-10
cg14918548	2	101350221	NA	0.394	0.308	0.351	-0.086	-0.044	2.05E-80	6.82E-11
cg08778216	2	101628572	*RPL31*	0.647	0.709	0.684	0.062	0.038	3.46E-45	1.02E-08
cg02863135	2	101740862	*TBC1D8*	0.757	0.807	0.805	0.050	0.049	5.17E-21	1.14E-09
cg05991009	2	102080674	*RFX8*	0.459	0.347	0.412	-0.112	-0.048	2.78E-165	2.13E-22
cg03974193	2	102316442	*MAP4K4*	0.746	0.796	0.784	0.051	0.038	1.66E-42	2.12E-11
cg13522882	2	102316496	*MAP4K4*	0.783	0.835	0.804	0.052	0.021	4.17E-95	4.34E-09
cg10667895	2	102577876	NA	0.408	0.320	0.373	-0.088	-0.036	5.02E-193	2.23E-25
cg14325025	2	102608155	*IL1R2*	0.289	0.219	0.254	-0.070	-0.035	7.35E-91	1.06E-11
cg17142183	2	102608192	*IL1R2*	0.424	0.338	0.389	-0.086	-0.034	6.61E-76	2.27E-07
cg21603144	2	102608233	*IL1R2*	0.385	0.319	0.349	-0.066	-0.036	4.37E-64	7.93E-10
cg13399261	2	102681071	NA	0.326	0.249	0.287	-0.077	-0.038	5.19E-118	1.77E-16
cg05886087	2	102758186	NA	0.433	0.327	0.393	-0.106	-0.040	1.92E-128	3.46E-12
cg19075787	2	105372087	*LOC284998*	0.304	0.252	0.268	-0.052	-0.036	1.06E-37	1.64E-09
cg22365240	2	105374995	NA	0.488	0.551	0.528	0.063	0.040	1.40E-40	4.69E-09
cg04112866	2	105925965	*TGFBRAP1*	0.429	0.324	0.371	-0.104	-0.058	4.64E-130	2.15E-23
cg22454769	2	106015767	*FHL2*	0.469	0.495	0.528	0.026	0.059	8.14E-07	2.49E-14
cg19828970	2	106371647	*NCK2*	0.415	0.320	0.367	-0.094	-0.048	8.78E-102	4.47E-15
cg21661768	2	106512578	NA	0.676	0.737	0.700	0.061	0.024	2.62E-88	4.62E-08
cg20995188	2	106713059	*UXS1*	0.741	0.798	0.770	0.057	0.029	1.49E-75	1.87E-12
cg25305879	2	106814630	NA	0.505	0.389	0.462	-0.116	-0.043	3.84E-190	1.80E-20
cg06351682	2	106974831	NA	0.744	0.802	0.778	0.058	0.034	4.65E-46	7.41E-08
cg16331492	2	107456855	*ST6GAL2*	0.708	0.758	0.740	0.050	0.032	2.44E-34	3.92E-08
cg03681335	2	108903965	*SULT1C2*	0.475	0.409	0.436	-0.066	-0.039	9.94E-65	1.26E-11
cg10236239	2	108994514	*SULT1C4*	0.324	0.252	0.288	-0.073	-0.036	1.60E-103	4.90E-17
cg05036173	2	108994528	*SULT1C4*	0.269	0.212	0.248	-0.057	-0.021	5.71E-76	5.13E-07
cg10075819	2	109229337	*LIMS1*	0.697	0.774	0.723	0.077	0.025	1.01E-126	6.82E-10
cg24777399	2	109855574	*SH3RF3*	0.456	0.368	0.403	-0.088	-0.053	2.47E-138	1.99E-30
cg00010078	2	109967172	*SH3RF3*	0.534	0.457	0.490	-0.077	-0.044	2.97E-85	6.23E-15
cg18282393	2	111788355	*ACOXL*	0.772	0.829	0.811	0.056	0.038	3.63E-39	5.81E-08
cg12966876	2	111873850	*ACOXL*	0.703	0.763	0.728	0.060	0.025	2.71E-60	1.72E-06
cg09570614	2	112124380	NA	0.521	0.467	0.487	-0.054	-0.034	3.67E-55	9.34E-13
cg23262488	2	112468472	NA	0.255	0.205	0.228	-0.051	-0.028	1.34E-59	1.90E-10
cg01154505	2	112940409	*FBLN7*	0.767	0.837	0.799	0.069	0.032	3.09E-119	4.84E-15
cg24743301	2	112945506	*FBLN7*	0.715	0.768	0.741	0.053	0.026	1.01E-43	2.67E-06
cg09841889	2	113552033	NA	0.311	0.251	0.274	-0.060	-0.037	3.22E-50	1.10E-09
cg15836722	2	113593785	*IL1B*	0.403	0.350	0.374	-0.053	-0.029	1.80E-46	4.14E-07
cg01290568	2	113593859	*IL1B*	0.419	0.333	0.375	-0.086	-0.044	2.48E-114	1.10E-16

cg03989987	2	113885277	*IL1RN*	0.288	0.222	0.255	-0.066	-0.033	1.45E-74	1.13E-10
cg13288906	2	114656403	*ACTR3*	0.505	0.582	0.547	0.077	0.042	5.62E-61	4.65E-10
cg24475517	2	118737667	*CCDC93*	0.491	0.379	0.442	-0.112	-0.050	7.00E-163	1.89E-22
cg14020285	2	119401665	NA	0.562	0.614	0.601	0.052	0.039	1.81E-19	3.64E-06
cg11009736	2	119699682	*MARCO*	0.341	0.261	0.291	-0.079	-0.049	6.70E-81	7.66E-16
cg19284751	2	119699789	*MARCO*	0.390	0.305	0.355	-0.085	-0.035	8.89E-118	1.05E-11
cg25079915	2	120192027	*TMEM37*	0.392	0.313	0.366	-0.079	-0.026	4.74E-105	2.10E-07
cg24441810	2	120436039	*TMEM177*	0.590	0.671	0.640	0.081	0.050	1.36E-70	9.76E-14
cg03154580	2	121009176	*RALB*	0.437	0.369	0.409	-0.068	-0.028	1.10E-76	2.44E-07
cg11305121	2	121011590	*RALB*	0.482	0.428	0.448	-0.055	-0.034	8.30E-43	3.48E-09
cg10927461	2	122530159	NA	0.330	0.254	0.290	-0.077	-0.040	2.00E-88	1.91E-12
cg17105014	2	127413363	*GYPC*	0.368	0.284	0.336	-0.084	-0.032	2.49E-85	9.52E-07
cg10173124	2	127963653	*CYP27C1*	0.672	0.730	0.730	0.058	0.058	2.05E-30	4.42E-14
cg06373940	2	128052778	*ERCC3*	0.566	0.438	0.516	-0.128	-0.050	1.85E-138	4.90E-18
cg06202737	2	128166279	NA	0.426	0.365	0.387	-0.061	-0.039	1.17E-37	6.52E-08
cg25949447	2	128169860	NA	0.526	0.412	0.489	-0.114	-0.037	1.19E-117	1.37E-10
cg04721485	2	128494954	*WDR33*	0.794	0.836	0.845	0.042	0.051	4.80E-17	1.01E-11
cg01765406	2	129231478	NA	0.511	0.456	0.470	-0.055	-0.041	1.87E-47	1.75E-13
cg23229770	2	129491004	NA	0.431	0.350	0.391	-0.081	-0.040	5.20E-69	1.68E-09
cg04065472	2	131148175	NA	0.246	0.194	0.207	-0.052	-0.039	1.84E-56	1.44E-13
cg11464160	2	131870398	*PLEKHB2*	0.398	0.307	0.372	-0.091	-0.026	9.71E-120	4.71E-07
cg21221263	2	134933179	NA	0.425	0.324	0.388	-0.101	-0.037	1.30E-157	9.05E-16
cg01580574	2	134949671	NA	0.606	0.657	0.664	0.050	0.057	2.50E-16	4.48E-10
cg20063095	2	134977141	NA	0.467	0.374	0.430	-0.093	-0.037	2.07E-96	7.95E-09
cg01401219	2	136541665	*UBXN4*	0.726	0.774	0.782	0.048	0.056	1.58E-23	2.08E-14
cg12313149	2	136763629	NA	0.499	0.389	0.459	-0.110	-0.040	9.79E-122	1.77E-11
cg23374992	2	136872067	*CXCR4*	0.306	0.227	0.277	-0.078	-0.029	8.27E-98	7.89E-09
cg12595667	2	136872094	*CXCR4*	0.291	0.228	0.264	-0.063	-0.027	5.38E-68	3.36E-07
cg19861978	2	139033234	NA	0.591	0.645	0.627	0.054	0.036	1.76E-26	1.44E-06
cg19867914	2	144234430	*ARHGAP15*	0.415	0.331	0.382	-0.084	-0.033	5.79E-112	1.52E-10
cg14768164	2	145025049	*GTDC1*	0.739	0.794	0.761	0.054	0.022	1.05E-99	7.29E-10
cg07780074	2	145090814	*GTDC1*	0.555	0.502	0.525	-0.053	-0.030	1.20E-47	2.77E-08
cg00573770	2	145278485	*ZEB2*	0.372	0.329	0.317	-0.043	-0.056	1.20E-23	1.13E-20
cg00602811	2	145278564	*ZEB2*	0.505	0.437	0.432	-0.067	-0.073	1.30E-47	4.13E-29
cg12940181	2	145353012	NA	0.380	0.281	0.345	-0.099	-0.034	2.25E-124	2.90E-09
cg09727050	2	152214177	*TNFAIP6*	0.450	0.349	0.417	-0.101	-0.033	4.82E-131	8.79E-11
cg26639076	2	152331806	*RIF1*	0.369	0.293	0.339	-0.076	-0.030	2.62E-83	1.05E-07
cg02508251	2	153508150	*PRPF40A*	0.647	0.682	0.699	0.035	0.051	3.58E-14	6.48E-13
cg02916962	2	157180144	NA	0.287	0.223	0.253	-0.064	-0.034	6.30E-49	2.07E-08
cg21758126	2	157183291	*NR4A2*	0.605	0.548	0.561	-0.057	-0.044	3.04E-22	1.49E-07
cg03339537	2	157184816	*NR4A2*	0.480	0.421	0.433	-0.059	-0.046	2.45E-35	9.58E-11
cg06101180	2	157185558	*NR4A2*	0.417	0.357	0.391	-0.060	-0.026	2.41E-90	1.37E-10
cg02039987	2	157291262	*GPD2*	0.485	0.553	0.539	0.068	0.054	5.53E-49	2.87E-16
cg02276944	2	157291826	*GPD2*	0.497	0.437	0.469	-0.060	-0.028	2.27E-86	1.06E-11
cg15736127	2	157292127	*GPD2*	0.469	0.363	0.438	-0.105	-0.031	3.50E-103	1.59E-06
cg19506253	2	158301839	*CYTIP*	0.720	0.777	0.758	0.057	0.038	4.22E-46	4.73E-11
cg09499849	2	158695158	*ACVR1*	0.308	0.239	0.276	-0.069	-0.033	1.47E-93	6.48E-11
cg24611970	2	159790917	NA	0.760	0.812	0.796	0.052	0.036	3.56E-37	3.37E-09
cg13722419	2	160088036	*TANC1*	0.757	0.796	0.808	0.040	0.052	9.47E-15	4.85E-12
cg08287940	2	160491979	NA	0.558	0.442	0.514	-0.116	-0.044	3.13E-198	2.64E-25
cg09643312	2	160655081	*CD302*	0.475	0.389	0.429	-0.086	-0.046	1.94E-78	9.36E-13
cg00760729	2	161236088	*RBMS1*	0.407	0.312	0.371	-0.095	-0.036	1.91E-122	6.55E-12
cg15657100	2	161420987	NA	0.731	0.784	0.776	0.053	0.045	2.76E-35	7.75E-12
cg27227317	2	161785635	NA	0.665	0.717	0.696	0.052	0.031	3.06E-38	2.11E-07
cg20838429	2	163100512	*FAP*	0.691	0.741	0.745	0.049	0.053	8.29E-22	4.63E-12
cg08066875	2	163197084	NA	0.559	0.504	0.530	-0.055	-0.029	1.97E-55	3.47E-09
cg03452895	2	163199311	*GCA*	0.587	0.519	0.558	-0.068	-0.029	1.92E-64	1.15E-06
cg15739581	2	166626783	*GALNT3*	0.479	0.394	0.451	-0.085	-0.028	2.99E-110	5.12E-08
cg00282195	2	168674769	*B3GALT1*	0.683	0.737	0.716	0.054	0.033	4.56E-35	1.69E-07
cg16875057	2	169006003	*STK39*	0.710	0.649	0.680	-0.061	-0.031	2.08E-43	9.62E-08
cg11775828	2	169100915	*STK39*	0.427	0.480	0.466	0.054	0.040	7.88E-48	2.18E-13
cg02743878	2	169937094	*DHRS9*	0.339	0.278	0.309	-0.061	-0.030	1.12E-69	1.95E-09
cg01462353	2	169939873	*DHRS9*	0.467	0.365	0.437	-0.102	-0.030	5.53E-118	2.91E-08
cg09128944	2	169967580	NA	0.402	0.309	0.368	-0.092	-0.034	7.94E-102	7.33E-09
cg19962304	2	170135359	*LRP2*	0.802	0.842	0.853	0.040	0.050	1.02E-17	9.01E-13
cg14713146	2	171782647	NA	0.578	0.510	0.549	-0.068	-0.029	1.62E-44	5.67E-06
cg16350446	2	172338747	*DCAF17*	0.403	0.314	0.377	-0.090	-0.026	2.53E-108	2.85E-06
cg12345526	2	173159062	NA	0.549	0.472	0.520	-0.077	-0.029	1.08E-72	3.56E-07
cg25743819	2	173245704	NA	0.315	0.252	0.276	-0.063	-0.039	5.09E-48	7.55E-09
cg15021238	2	173305865	*ITGA6*	0.650	0.707	0.698	0.057	0.048	1.24E-33	5.98E-11
cg00883212	2	173795201	*RAPGEF4*	0.546	0.471	0.516	-0.075	-0.029	1.08E-58	2.94E-06
cg03608974	2	173940268	*ZAK*	0.286	0.231	0.258	-0.055	-0.028	4.58E-46	6.35E-06
cg11119767	2	174024669	*ZAK*	0.441	0.334	0.398	-0.106	-0.043	1.34E-131	2.14E-13
cg17258387	2	174924430	NA	0.367	0.305	0.343	-0.062	-0.024	3.18E-139	8.30E-18
cg15058645	2	175528343	*WIPF1*	0.408	0.302	0.354	-0.106	-0.054	2.58E-153	2.94E-24
cg19275653	2	175532338	*WIPF1*	0.284	0.212	0.259	-0.072	-0.025	1.44E-86	7.31E-07
cg00405190	2	175545838	*WIPF1*	0.443	0.332	0.398	-0.111	-0.045	5.35E-151	4.44E-18
cg03713585	2	175693153	*CHN1*	0.783	0.825	0.846	0.042	0.063	4.25E-14	1.07E-14

cg07484739	2	177356020	NA	0.661	0.729	0.691	0.067	0.029	1.33E-71	7.23E-09
cg06065019	2	177356276	NA	0.275	0.335	0.322	0.061	0.048	1.01E-31	1.57E-10
cg09644974	2	177358138	NA	0.677	0.740	0.725	0.062	0.048	1.62E-44	1.41E-12
cg13661243	2	177372587	NA	0.726	0.775	0.777	0.049	0.051	4.94E-23	6.14E-11
cg22386008	2	177737177	NA	0.699	0.754	0.737	0.055	0.038	3.25E-29	5.41E-07
cg25954134	2	178941694	*PDE11A*	0.463	0.393	0.417	-0.070	-0.046	2.96E-45	7.65E-11
cg12011522	2	179192421	*OSBPL6*	0.643	0.697	0.665	0.053	0.022	2.10E-54	5.80E-06
cg11539052	2	179347266	*PLEKHA3*	0.640	0.690	0.673	0.050	0.033	5.11E-38	6.86E-09
cg01231381	2	180130081	*SESTD1*	0.517	0.428	0.485	-0.090	-0.032	4.92E-105	2.09E-08
cg08466256	2	189957139	*COL5A2*	0.706	0.766	0.747	0.059	0.040	5.03E-40	5.76E-09
cg01080713	2	190126396	NA	0.700	0.760	0.734	0.060	0.035	1.59E-62	3.34E-11
cg04361126	2	190446792	*SLC40A1*	0.274	0.217	0.245	-0.057	-0.029	2.98E-65	2.37E-09
cg18011672	2	190522601	NA	0.644	0.727	0.681	0.082	0.037	3.01E-109	6.69E-12
cg05969591	2	191044915	*C2orf88*	0.551	0.497	0.526	-0.054	-0.025	1.01E-63	7.05E-08
cg04144521	2	191276183	*MFSD6*	0.442	0.348	0.404	-0.094	-0.039	8.99E-126	1.84E-13
cg01233664	2	191612466	NA	0.742	0.793	0.770	0.051	0.028	6.91E-44	6.18E-07
cg03022510	2	192476634	NA	0.401	0.312	0.370	-0.090	-0.031	4.47E-173	1.14E-15
cg10800620	2	196398826	NA	0.673	0.741	0.701	0.068	0.028	7.16E-89	3.50E-09
cg19149314	2	197065128	*HECW2*	0.753	0.814	0.793	0.062	0.040	1.54E-44	7.96E-10
cg18854872	2	198292151	*SF3B1*	0.513	0.446	0.489	-0.068	-0.025	7.30E-85	8.50E-08
cg19766578	2	201677983	*BZW1*	0.552	0.611	0.581	0.059	0.029	1.68E-42	1.82E-06
cg18119407	2	201980504	*CFLAR*	0.643	0.693	0.669	0.050	0.026	8.92E-50	1.06E-09
cg17974460	2	202510866	*MPP4*	0.752	0.806	0.799	0.054	0.046	1.20E-23	1.85E-08
cg24336674	2	204602302	*CD28*	0.754	0.805	0.787	0.050	0.033	4.47E-37	3.84E-08
cg17591195	2	205130274	NA	0.776	0.814	0.827	0.038	0.050	8.64E-14	9.65E-11
cg11608958	2	207836769	NA	0.489	0.400	0.449	-0.089	-0.039	9.04E-156	3.69E-20
cg02796279	2	208101334	NA	0.466	0.351	0.423	-0.115	-0.043	2.56E-142	1.58E-14
cg04507925	2	210824770	*UNC80*	0.692	0.749	0.722	0.056	0.030	1.74E-39	2.41E-06
cg15724534	2	211018363	*KANSL1L*	0.595	0.513	0.569	-0.082	-0.026	6.81E-83	8.78E-07
cg26166177	2	216260832	*FN1*	0.707	0.765	0.751	0.058	0.044	5.87E-27	1.37E-07
cg02540338	2	217243695	NA	0.776	0.819	0.829	0.042	0.052	4.13E-16	1.01E-11
cg21443659	2	217745721	NA	0.483	0.420	0.435	-0.062	-0.047	5.74E-45	3.08E-16
cg26839652	2	217881615	NA	0.682	0.751	0.722	0.069	0.040	7.02E-65	2.18E-10
cg17316747	2	218149237	*DIRC3*	0.699	0.749	0.729	0.051	0.030	4.13E-33	6.93E-07
cg25941354	2	218989983	*CXCR2*	0.355	0.301	0.318	-0.054	-0.037	3.97E-78	4.33E-23
cg15768138	2	219030752	*CXCR1*	0.499	0.434	0.462	-0.065	-0.037	7.19E-48	9.36E-08
cg04952446	2	219197867	*PNKD*	0.301	0.241	0.280	-0.060	-0.021	7.31E-74	4.03E-06
cg27380499	2	219233446	NA	0.314	0.252	0.293	-0.062	-0.021	1.43E-76	3.12E-06
cg05007997	2	219246985	*SLC11A1*	0.364	0.291	0.336	-0.073	-0.028	1.65E-109	1.45E-10
cg25925210	2	219576383	*TTLL4*	0.334	0.277	0.278	-0.057	-0.056	6.07E-18	3.82E-08
cg11123644	2	219597614	*TTLL4*	0.346	0.260	0.311	-0.086	-0.035	1.19E-112	8.21E-12
cg01827633	2	219610103	*TTLL4*	0.403	0.294	0.353	-0.109	-0.051	2.58E-127	5.41E-16
cg04067066	2	219756890	*WNT10A*	0.459	0.408	0.426	-0.051	-0.033	1.15E-33	2.24E-07
cg06755438	2	219885614	*CCDC108*	0.451	0.361	0.413	-0.090	-0.038	1.05E-86	2.73E-09
cg03878190	2	220047358	*FAM134A*	0.271	0.212	0.242	-0.060	-0.030	4.05E-55	2.16E-07
cg02776283	2	220074531	*ABCB6*	0.246	0.181	0.221	-0.065	-0.024	1.42E-93	1.04E-08
cg22175006	2	223757281	*ACSL3*	0.528	0.434	0.491	-0.094	-0.037	1.55E-92	7.59E-10
cg04936009	2	223846845	NA	0.549	0.466	0.521	-0.082	-0.028	3.07E-77	4.48E-06
cg19674851	2	224720888	NA	0.530	0.429	0.495	-0.101	-0.035	9.46E-97	6.58E-09
cg23363263	2	224751989	*WDFY1*	0.589	0.461	0.547	-0.128	-0.042	3.53E-150	9.13E-13
cg15457217	2	224780409	*WDFY1*	0.602	0.505	0.575	-0.097	-0.026	7.36E-120	1.78E-07
cg25757820	2	224819307	NA	0.587	0.460	0.550	-0.127	-0.037	6.52E-183	6.87E-13
cg06867755	2	225397387	*CUL3*	0.502	0.425	0.470	-0.077	-0.032	7.16E-103	6.04E-13
cg04168675	2	225809655	*DOCK10*	0.672	0.745	0.720	0.073	0.048	4.71E-66	1.26E-13
cg02806733	2	231731972	*ITM2C*	0.249	0.197	0.216	-0.052	-0.033	1.24E-57	4.45E-13
cg14649507	2	231735037	*ITM2C*	0.361	0.278	0.324	-0.083	-0.037	8.47E-174	1.08E-25
cg18665334	2	231735434	*ITM2C*	0.362	0.290	0.320	-0.072	-0.041	6.75E-113	6.85E-23
cg04519775	2	231849693	*LOC348761*	0.688	0.762	0.713	0.074	0.025	6.28E-139	3.50E-11
cg27051231	2	232213012	NA	0.444	0.338	0.399	-0.105	-0.044	5.96E-145	4.86E-16
cg01616956	2	232393196	*NMUR1*	0.735	0.801	0.763	0.066	0.027	5.08E-72	4.98E-07
cg17490196	2	232507842	NA	0.414	0.343	0.371	-0.071	-0.044	3.80E-86	5.61E-18
cg06109482	2	232531087	NA	0.391	0.328	0.368	-0.063	-0.022	6.74E-100	1.29E-11
cg27227507	2	232546142	NA	0.413	0.344	0.381	-0.069	-0.032	2.85E-100	1.30E-12
cg20627754	2	232582394	NA	0.525	0.454	0.489	-0.071	-0.036	2.45E-56	5.08E-09
cg14342532	2	233916534	NA	0.361	0.281	0.330	-0.080	-0.031	1.12E-74	2.48E-06
cg22666015	2	233981885	*INPP5D*	0.450	0.331	0.411	-0.119	-0.039	2.33E-126	8.42E-10
cg04280772	2	235372807	NA	0.770	0.823	0.794	0.053	0.024	2.12E-82	4.11E-11
cg01297806	2	235949554	*SH3BP4*	0.463	0.407	0.437	-0.056	-0.026	1.16E-70	7.66E-09
cg10809094	2	238006557	*COPS8*	0.686	0.752	0.736	0.065	0.050	1.50E-34	5.03E-11
cg21346966	2	238341793	NA	0.595	0.648	0.618	0.053	0.022	2.91E-117	1.71E-11
cg20386303	2	238350541	NA	0.718	0.772	0.737	0.054	0.019	3.07E-85	1.44E-06
cg18141622	2	238525524	NA	0.442	0.361	0.399	-0.081	-0.043	2.79E-79	2.37E-13
cg20513777	2	238533299	NA	0.538	0.461	0.501	-0.077	-0.037	5.99E-67	1.12E-08
cg07063912	2	238598832	*LRRFIP1*	0.378	0.294	0.339	-0.085	-0.040	7.62E-88	3.37E-11
cg21302563	2	238936238	*UBE2F*	0.442	0.376	0.406	-0.066	-0.036	3.63E-61	2.07E-09
cg01950511	2	239289051	*TRAF3IP1*	0.445	0.345	0.415	-0.100	-0.030	1.04E-104	5.22E-07
cg20382154	2	239463716	*LOC151171*	0.286	0.221	0.258	-0.065	-0.028	2.51E-68	9.74E-07
cg05758467	2	240035894	*HDAC4*	0.489	0.402	0.444	-0.086	-0.045	2.96E-92	1.67E-13

cg02173540	2	240046504	*HDAC4*	0.701	0.760	0.762	0.059	0.061	1.78E-24	8.51E-12
cg21190228	2	240132342	*HDAC4*	0.415	0.307	0.365	-0.108	-0.050	8.99E-147	2.16E-19
cg17420028	2	240142589	*HDAC4*	0.641	0.572	0.616	-0.070	-0.025	7.85E-74	4.61E-07
cg03169059	2	240145022	*HDAC4*	0.411	0.319	0.375	-0.091	-0.035	9.35E-111	3.31E-10
cg10094994	2	240162323	*HDAC4*	0.370	0.280	0.336	-0.090	-0.034	2.83E-107	2.36E-08
cg26495711	2	240169280	*HDAC4*	0.712	0.766	0.733	0.054	0.021	2.75E-86	3.37E-07
cg11564239	2	241644188	NA	0.579	0.637	0.601	0.058	0.022	1.77E-99	8.49E-11
cg12565788	2	241644240	NA	0.643	0.717	0.675	0.074	0.032	1.85E-70	5.38E-08
cg16967583	2	241807859	*AGXT*	0.353	0.267	0.302	-0.086	-0.051	1.35E-114	2.32E-24
cg24371033	2	241807924	*AGXT*	0.365	0.293	0.330	-0.073	-0.036	7.16E-127	7.30E-20
cg09991306	2	241975140	*SNED1*	0.704	0.775	0.748	0.071	0.045	1.30E-35	7.78E-08
cg19397991	2	242048218	*PASK*	0.625	0.688	0.655	0.064	0.030	1.78E-82	3.06E-11
cg07030287	2	242174797	*HDLBP*	0.372	0.306	0.349	-0.066	-0.023	2.38E-124	1.01E-10
cg03610527	2	242174889	*HDLBP*	0.553	0.454	0.528	-0.099	-0.025	4.47E-124	9.62E-08
cg20489909	2	242711046	NA	0.377	0.310	0.356	-0.067	-0.020	3.76E-137	7.83E-10
cg14453145	2	242801896	*PDCD1*	0.737	0.799	0.760	0.061	0.022	2.20E-83	1.06E-06
cg00795812	2	242802009	*PDCD1*	0.584	0.658	0.608	0.074	0.023	5.09E-117	3.47E-08
cg27051683	2	242802069	*PDCD1*	0.526	0.594	0.541	0.067	0.015	1.24E-124	3.59E-07
cg03889044	2	242802099	*PDCD1*	0.594	0.685	0.626	0.091	0.032	2.94E-190	5.34E-18
cg17322655	2	242802127	*PDCD1*	0.604	0.691	0.634	0.086	0.030	5.79E-160	2.04E-14
cg20805133	2	242802192	*PDCD1*	0.610	0.706	0.639	0.096	0.029	6.06E-119	9.10E-08
cg11654325	2	242810927	*CXXC11*	0.623	0.681	0.652	0.058	0.029	3.71E-48	4.12E-07
cg23491841	2	242811149	*CXXC11*	0.612	0.667	0.639	0.055	0.027	1.97E-45	2.57E-06
cg19035788	2	242881634	NA	0.732	0.784	0.763	0.051	0.031	1.42E-44	8.16E-08
cg11485152	3	3171664	*TRNT1*	0.421	0.342	0.379	-0.078	-0.042	5.74E-91	1.36E-13
cg18034501	3	4043162	NA	0.561	0.613	0.606	0.051	0.045	2.03E-15	5.84E-06
cg24159247	3	4575483	*ITPR1*	0.277	0.210	0.252	-0.068	-0.026	1.52E-114	2.36E-09
cg02105211	3	4625188	*ITPR1*	0.595	0.513	0.555	-0.082	-0.040	3.36E-66	2.69E-09
cg18471664	3	5028143	NA	0.286	0.228	0.252	-0.058	-0.034	1.55E-95	6.08E-20
cg13475822	3	9210116	*SRGAP3*	0.628	0.687	0.655	0.059	0.027	1.16E-73	6.29E-10
cg01564818	3	9464436	*SETD5*	0.283	0.220	0.252	-0.062	-0.030	9.00E-101	9.64E-15
cg23223755	3	9596849	*LHFPL4*	0.689	0.738	0.740	0.048	0.051	1.58E-19	8.65E-11
cg14583999	3	10019040	*EMC3*	0.565	0.514	0.524	-0.051	-0.041	5.60E-32	5.58E-10
cg10317164	3	10274142	*IRAK2*	0.362	0.287	0.336	-0.076	-0.026	1.93E-129	1.43E-10
cg06371658	3	10292843	*TATDN2*	0.741	0.786	0.792	0.045	0.051	2.09E-20	1.08E-11
cg03920999	3	10334760	*GHRLOS*	0.342	0.270	0.308	-0.072	-0.033	2.03E-123	4.91E-17
cg05844788	3	10336053	*GHRL*	0.292	0.227	0.258	-0.065	-0.034	1.88E-120	1.35E-22
cg17571559	3	11267525	*HRH1*	0.436	0.332	0.392	-0.104	-0.044	2.24E-117	9.22E-12
cg01796438	3	11312864	*ATG7*	0.451	0.363	0.415	-0.088	-0.035	1.40E-144	1.61E-15
cg12120750	3	11592700	*ATG7*	0.314	0.254	0.293	-0.060	-0.021	9.56E-74	2.00E-06
cg21442998	3	11597936	*VGLL4*	0.441	0.363	0.414	-0.078	-0.027	4.63E-113	1.62E-09
cg00668519	3	11597941	*VGLL4*	0.336	0.273	0.313	-0.063	-0.022	1.69E-96	1.29E-07
cg00691123	3	11632974	*VGLL4*	0.498	0.408	0.457	-0.090	-0.041	9.40E-120	2.24E-15
cg02309841	3	11758425	*VGLL4*	0.473	0.375	0.429	-0.098	-0.045	9.57E-143	2.98E-22
cg25423174	3	12236303	NA	0.490	0.438	0.461	-0.053	-0.030	4.14E-39	2.29E-07
cg18537222	3	12435731	*PPARG*	0.513	0.415	0.482	-0.098	-0.031	8.35E-101	1.49E-07
cg01526217	3	12524194	NA	0.430	0.327	0.393	-0.103	-0.037	1.95E-103	4.59E-09
cg13071069	3	13005914	*IQSEC1*	0.393	0.317	0.365	-0.076	-0.028	1.62E-129	1.64E-14
cg06143615	3	13462903	*NUP210*	0.587	0.647	0.618	0.060	0.031	3.41E-110	7.78E-21
cg02555923	3	14319984	NA	0.297	0.240	0.272	-0.057	-0.026	5.81E-107	3.05E-13
cg12209946	3	14987434	*FGD5-AS1*	0.523	0.422	0.488	-0.101	-0.035	2.06E-106	2.18E-08
cg07480608	3	15352493	*SH3BP5*	0.765	0.823	0.790	0.058	0.024	7.57E-69	2.41E-07
cg02930866	3	15482580	*EAF1*	0.255	0.201	0.230	-0.054	-0.025	4.35E-71	2.31E-09
cg27584135	3	15975501	NA	0.367	0.314	0.329	-0.052	-0.037	6.22E-41	1.04E-10
cg22230912	3	16331335	*OXNAD1*	0.732	0.784	0.755	0.052	0.023	1.54E-51	6.31E-06
cg12172478	3	16357591	*RFTN1*	0.717	0.767	0.742	0.050	0.026	3.94E-51	2.30E-08
cg00203037	3	16360321	*RFTN1*	0.523	0.416	0.484	-0.107	-0.039	9.67E-93	1.26E-07
cg11492723	3	16577697	NA	0.495	0.430	0.443	-0.065	-0.052	1.86E-37	6.05E-12
cg06213060	3	16577726	NA	0.379	0.317	0.336	-0.062	-0.043	9.42E-51	2.45E-12
cg19448816	3	16974217	*PLCL2*	0.558	0.499	0.513	-0.059	-0.045	1.34E-28	6.94E-09
cg16503724	3	17130667	*PLCL2*	0.504	0.560	0.528	0.056	0.023	2.32E-51	3.06E-06
cg10288467	3	20371290	NA	0.766	0.809	0.818	0.043	0.052	2.57E-17	2.30E-11
cg26266876	3	23957486	*RPL15*	0.294	0.217	0.258	-0.077	-0.036	8.66E-127	4.93E-17
cg23047137	3	24089778	NA	0.665	0.734	0.713	0.069	0.048	2.81E-42	6.10E-10
cg01152567	3	28294009	*CMC1*	0.717	0.769	0.752	0.052	0.036	8.67E-32	3.76E-08
cg21821982	3	31547255	NA	0.457	0.340	0.406	-0.116	-0.050	3.99E-134	2.02E-16
cg10789050	3	31713847	*OSBPL10*	0.371	0.307	0.334	-0.064	-0.037	6.01E-47	2.91E-08
cg02570354	3	31728749	*OSBPL10*	0.385	0.286	0.336	-0.099	-0.049	4.18E-123	5.57E-20
cg21654379	3	32864307	*TRIM71*	0.712	0.761	0.775	0.049	0.063	6.32E-19	3.22E-14
cg08229360	3	37904475	*CTDSPL*	0.262	0.210	0.234	-0.052	-0.027	8.95E-135	7.09E-24
cg16665765	3	37934293	*CTDSPL*	0.400	0.348	0.360	-0.051	-0.040	1.53E-46	3.24E-14
cg01534527	3	38003247	*CTDSPL*	0.545	0.494	0.512	-0.050	-0.032	2.84E-50	9.47E-11
cg24310395	3	39309435	*CX3CR1*	0.728	0.792	0.751	0.064	0.023	3.88E-98	1.14E-07
cg14662886	3	39450018	*SNORA6*	0.777	0.829	0.811	0.053	0.035	9.64E-36	3.96E-08
cg02108620	3	42002230	*ULK4*	0.408	0.349	0.375	-0.059	-0.033	1.20E-45	6.72E-08
cg15033269	3	42631489	*SEC22C*	0.474	0.359	0.428	-0.115	-0.047	2.12E-156	2.49E-18
cg10960375	3	42694144	*ZBTB47*	0.462	0.408	0.424	-0.054	-0.038	4.47E-56	7.88E-15
cg01802713	3	42695307	*ZBTB47*	0.313	0.262	0.282	-0.051	-0.031	5.40E-53	1.13E-10

cg07582204	3	43275279	NA	0.674	0.736	0.719	0.062	0.045	5.12E-29	5.80E-08
cg09417547	3	43289436	NA	0.507	0.402	0.468	-0.104	-0.039	1.36E-114	7.27E-11
cg01801643	3	43387641	*SNRK*	0.709	0.772	0.750	0.063	0.041	3.72E-60	1.34E-12
cg18771737	3	43448041	*ANO10*	0.501	0.434	0.464	-0.067	-0.037	3.10E-73	2.01E-12
cg24595152	3	43733831	*ABHD5*	0.378	0.295	0.347	-0.082	-0.031	2.29E-155	1.35E-18
cg15501219	3	44375962	NA	0.462	0.394	0.421	-0.068	-0.042	9.13E-89	8.33E-19
cg15477500	3	44957542	*ZDHHC3*	0.522	0.471	0.498	-0.052	-0.024	3.69E-46	6.12E-06
cg05380759	3	45579354	*LARS2*	0.409	0.316	0.375	-0.094	-0.035	5.44E-161	4.34E-15
cg25437886	3	45677346	*LIMD1*	0.524	0.401	0.476	-0.123	-0.048	8.85E-149	2.86E-15
cg16283183	3	45718805	*LIMD1*	0.427	0.331	0.397	-0.096	-0.030	5.01E-114	4.56E-08
cg26763394	3	45957664	NA	0.681	0.738	0.711	0.057	0.030	3.96E-86	2.76E-16
cg08450017	3	45984838	*CXCR6*	0.473	0.602	0.534	0.130	0.062	1.44E-130	1.30E-18
cg01178899	3	45985168	*CXCR6*	0.776	0.826	0.800	0.051	0.024	5.97E-60	1.88E-07
cg17786516	3	46410422	*CCR5*	0.626	0.682	0.666	0.056	0.040	5.81E-32	1.08E-08
cg16021018	3	46449313	*CCRL2*	0.266	0.210	0.240	-0.055	-0.026	8.83E-57	6.44E-07
cg20496896	3	46579532	*LRRC2*	0.747	0.798	0.768	0.050	0.021	3.43E-60	4.81E-07
cg18461635	3	46990143	*CCDC12*	0.805	0.858	0.822	0.053	0.017	1.53E-96	5.50E-06
cg15157945	3	47023603	*NBEAL2*	0.317	0.251	0.281	-0.066	-0.037	8.76E-132	1.27E-23
cg19440734	3	48264753	*CAMP*	0.265	0.211	0.241	-0.054	-0.024	2.29E-62	2.38E-07
cg12030667	3	48471659	*PLXNB1*	0.277	0.211	0.257	-0.066	-0.020	5.88E-126	9.64E-09
cg01313363	3	48471695	*PLXNB1*	0.342	0.277	0.321	-0.066	-0.021	1.69E-139	7.60E-13
cg01870865	3	48507087	*TREX1*	0.365	0.286	0.327	-0.079	-0.038	2.95E-127	2.49E-20
cg05557266	3	49057259	*DALRD3*	0.273	0.215	0.238	-0.059	-0.035	3.05E-54	2.07E-10
cg14184400	3	49460057	*AMT*	0.444	0.381	0.420	-0.064	-0.025	3.06E-79	1.94E-08
cg08510456	3	49591008	*BSN*	0.355	0.275	0.320	-0.080	-0.035	4.26E-80	5.71E-08
cg24296397	3	49692537	*BSN*	0.552	0.627	0.590	0.075	0.038	3.43E-83	1.65E-14
cg26226650	3	50276265	*GNAI2*	0.366	0.280	0.329	-0.086	-0.037	1.95E-153	4.23E-19
cg15275312	3	50363059	*TUSC2*	0.347	0.255	0.303	-0.091	-0.043	3.25E-153	5.12E-23
cg02310103	3	50391305	*CYB561D2*	0.373	0.321	0.344	-0.053	-0.030	4.82E-55	5.10E-10
cg20133901	3	51602121	*RAD54L2*	0.305	0.239	0.276	-0.066	-0.029	1.73E-108	1.26E-12
cg22084410	3	51987688	NA	0.403	0.317	0.367	-0.087	-0.037	1.82E-112	3.31E-12
cg15698299	3	52233019	*ALAS1*	0.305	0.249	0.282	-0.056	-0.023	6.31E-91	3.78E-10
cg13241645	3	52235959	*ALAS1*	0.566	0.491	0.527	-0.075	-0.039	2.38E-74	9.41E-12
cg24575128	3	52502445	*NISCH*	0.476	0.406	0.439	-0.070	-0.038	1.43E-78	5.79E-12
cg01381934	3	52529064	*STAB1*	0.361	0.272	0.329	-0.089	-0.032	3.82E-117	3.60E-10
cg02792780	3	52529341	*STAB1*	0.299	0.248	0.281	-0.051	-0.018	8.84E-72	4.78E-06
cg11485012	3	52909345	*TMEM110-N*	0.678	0.731	0.707	0.054	0.029	8.57E-55	1.03E-08
cg11184109	3	53164962	*RFT1*	0.291	0.211	0.255	-0.080	-0.035	2.63E-105	2.95E-11
cg01465769	3	53226412	*PRKCD*	0.385	0.321	0.354	-0.064	-0.031	1.37E-66	6.13E-09
cg11014468	3	53362965	*DCP1A*	0.528	0.431	0.492	-0.097	-0.036	4.96E-168	1.57E-17
cg11229273	3	57015101	*ARHGEF3*	0.308	0.242	0.275	-0.067	-0.033	8.86E-83	8.44E-12
cg15572396	3	58321349	*PXK*	0.399	0.315	0.363	-0.084	-0.036	1.92E-134	3.11E-16
cg23549972	3	58389654	*PXK*	0.672	0.742	0.715	0.070	0.043	9.41E-58	1.48E-10
cg15028458	3	58860290	*C3orf67*	0.629	0.695	0.661	0.066	0.032	1.88E-41	1.19E-06
cg13679804	3	59734950	NA	0.427	0.325	0.376	-0.103	-0.051	3.11E-109	7.57E-15
cg16806041	3	59996265	*FHIT*	0.533	0.608	0.566	0.076	0.033	2.36E-44	5.44E-06
cg07099186	3	61947083	*PTPRG*	0.624	0.682	0.668	0.058	0.043	2.64E-28	5.88E-08
cg15226514	3	63856759	*ATXN7*	0.730	0.781	0.767	0.051	0.036	4.08E-23	4.21E-06
cg13878360	3	64069147	*LOC1002878*	0.354	0.290	0.327	-0.064	-0.028	5.61E-78	6.60E-08
cg22202670	3	64676361	*ADAMTS9-A*	0.690	0.744	0.743	0.054	0.053	3.11E-24	1.25E-11
cg00225902	3	66444122	*LRIG1*	0.681	0.740	0.714	0.059	0.032	4.56E-40	8.24E-07
cg02012974	3	66492992	*LRIG1*	0.443	0.331	0.387	-0.112	-0.056	7.87E-163	8.38E-28
cg15166900	3	69132709	*ARL6IP5*	0.601	0.666	0.637	0.066	0.036	6.02E-40	2.55E-07
cg11377213	3	69370218	*FRMD4B*	0.350	0.264	0.302	-0.086	-0.048	3.49E-144	2.13E-29
cg19139092	3	69588171	NA	0.655	0.711	0.691	0.057	0.036	2.91E-32	6.31E-07
cg25811633	3	71626654	*FOXP1*	0.376	0.288	0.334	-0.088	-0.041	1.32E-108	3.62E-15
cg02520804	3	71629054	*FOXP1*	0.523	0.421	0.491	-0.102	-0.032	5.00E-99	1.17E-07
cg15037823	3	71730474	*EIF4E3*	0.443	0.368	0.410	-0.075	-0.033	6.46E-64	1.98E-07
cg03652525	3	72149324	NA	0.460	0.408	0.430	-0.052	-0.029	1.08E-52	2.22E-08
cg26108120	3	72440793	*RYBP*	0.382	0.295	0.334	-0.087	-0.048	7.60E-108	7.66E-20
cg02855045	3	72777245	NA	0.410	0.325	0.372	-0.085	-0.038	8.53E-128	1.93E-17
cg12115800	3	73144435	NA	0.516	0.446	0.494	-0.070	-0.022	7.27E-95	2.65E-06
cg19385799	3	78164711	NA	0.767	0.805	0.818	0.038	0.051	1.80E-14	7.80E-12
cg10752508	3	98313516	*CPOX*	0.446	0.353	0.412	-0.093	-0.034	8.91E-95	3.47E-08
cg04411201	3	99614305	*FILIP1L*	0.407	0.312	0.364	-0.094	-0.043	1.74E-142	5.10E-20
cg14454680	3	99794010	*FILIP1L*	0.429	0.342	0.398	-0.087	-0.032	1.27E-78	3.38E-07
cg23528247	3	99832772	*C3orf26*	0.398	0.298	0.356	-0.100	-0.042	6.96E-119	4.41E-14
cg04730047	3	99979355	*TBC1D23*	0.307	0.252	0.280	-0.055	-0.027	9.77E-51	2.13E-06
cg26645827	3	100322629	NA	0.363	0.293	0.334	-0.070	-0.029	2.11E-63	8.81E-07
cg20567280	3	101289945	NA	0.721	0.776	0.758	0.055	0.037	2.34E-48	3.26E-10
cg02059214	3	108021106	*HHLA2*	0.764	0.817	0.800	0.052	0.036	8.72E-42	2.87E-09
cg14479344	3	108126795	*MYH15*	0.412	0.337	0.384	-0.076	-0.028	1.72E-80	9.30E-07
cg19400179	3	108321607	*DZIP3*	0.316	0.375	0.344	0.059	0.028	8.92E-39	5.72E-06
cg13916298	3	108896099	NA	0.295	0.233	0.261	-0.061	-0.033	7.32E-102	1.08E-16
cg18560366	3	109057564	*DPPA4*	0.273	0.220	0.247	-0.053	-0.026	6.87E-68	1.24E-09
cg20074159	3	110246985	NA	0.536	0.586	0.599	0.050	0.063	7.22E-14	1.06E-09
cg20672711	3	110965888	NA	0.514	0.423	0.477	-0.091	-0.037	1.98E-88	6.58E-09
cg26337954	3	111315207	*ZBED2*	0.757	0.811	0.792	0.054	0.036	3.50E-30	8.15E-07

cg27043141	3	111851265	*GCSAM*	0.695	0.747	0.739	0.052	0.043	3.40E-31	2.74E-10
cg24157392	3	112217973	*BTLA*	0.626	0.698	0.661	0.071	0.035	3.35E-89	5.64E-13
cg10708271	3	112692897	*CD200R1*	0.712	0.780	0.754	0.067	0.042	1.52E-51	1.44E-10
cg18548864	3	112995278	*BOC*	0.353	0.279	0.323	-0.074	-0.029	2.16E-106	4.37E-12
cg08267038	3	113325595	*SIDT1*	0.291	0.231	0.262	-0.061	-0.029	4.11E-55	3.98E-07
cg13227806	3	113811946	NA	0.496	0.420	0.448	-0.077	-0.048	1.51E-35	2.20E-08
cg21137173	3	113953856	*ZNF80*	0.767	0.825	0.810	0.058	0.043	4.01E-47	3.78E-13
cg05894970	3	119041204	*ARHGAP31*	0.318	0.246	0.266	-0.073	-0.053	3.28E-77	7.70E-20
cg05961212	3	119298195	*ADPRH*	0.343	0.288	0.312	-0.054	-0.031	3.45E-50	8.20E-08
cg02863947	3	119499190	*NR1I2*	0.434	0.336	0.393	-0.098	-0.041	1.50E-156	5.03E-19
cg02241759	3	119528300	*NR1I2*	0.399	0.301	0.366	-0.098	-0.032	3.17E-123	1.17E-09
cg14527029	3	120401095	*HGD*	0.772	0.704	0.749	-0.068	-0.023	2.61E-71	1.45E-06
cg09447811	3	121972621	*CASR*	0.439	0.352	0.400	-0.087	-0.039	4.71E-89	9.03E-11
cg21932814	3	122044172	*CSTA*	0.432	0.334	0.386	-0.098	-0.046	1.89E-137	9.82E-19
cg22930808	3	122281881	*PARP9*	0.706	0.435	0.516	-0.271	-0.190	1.39E-105	2.40E-55
cg00959259	3	122281975	*PARP9*	0.578	0.349	0.409	-0.229	-0.170	9.28E-105	2.60E-48
cg03899219	3	122333057	*PARP15*	0.749	0.800	0.797	0.050	0.047	2.51E-29	9.13E-12
cg01948202	3	122400474	*PARP14*	0.156	0.097	0.116	-0.059	-0.041	4.92E-52	4.93E-10
cg09359351	3	122509111	*HSPBAP1*	0.480	0.415	0.436	-0.064	-0.043	2.27E-50	1.20E-10
cg10627428	3	122514814	*DIRC2*	0.292	0.223	0.264	-0.069	-0.028	1.80E-90	1.29E-09
cg05502766	3	122604506	NA	0.689	0.745	0.734	0.056	0.045	8.78E-32	3.32E-10
cg14516183	3	122928186	*SEC22A*	0.530	0.407	0.496	-0.123	-0.034	6.09E-155	1.85E-09
cg07638500	3	123371420	*MYLK*	0.307	0.253	0.286	-0.055	-0.021	1.90E-94	3.49E-09
cg01894985	3	123589179	*MYLK*	0.667	0.728	0.697	0.060	0.030	4.30E-52	3.53E-07
cg00710736	3	125000990	*ZNF148*	0.732	0.785	0.777	0.053	0.044	1.73E-21	2.24E-07
cg26313511	3	125053815	*ZNF148*	0.416	0.330	0.383	-0.086	-0.033	1.75E-155	3.01E-17
cg16945415	3	125980095	NA	0.328	0.258	0.301	-0.070	-0.027	2.02E-132	1.81E-16
cg01357892	3	126191181	*ZXDC*	0.232	0.182	0.205	-0.050	-0.027	1.73E-87	7.13E-15
cg00391741	3	127307227	*TPRA1*	0.678	0.613	0.651	-0.065	-0.026	4.30E-59	3.00E-06
cg12678686	3	127327369	*MCM2*	0.431	0.338	0.401	-0.092	-0.030	2.01E-110	1.98E-07
cg00211174	3	127332098	*MCM2*	0.513	0.387	0.462	-0.126	-0.051	2.61E-132	1.36E-12
cg14788242	3	127773954	*SEC61A1*	0.431	0.351	0.406	-0.080	-0.025	7.66E-76	5.57E-06
cg16899991	3	127868625	NA	0.441	0.341	0.401	-0.100	-0.040	1.28E-106	5.34E-13
cg06806891	3	128323079	NA	0.473	0.411	0.458	-0.063	-0.016	7.57E-130	7.13E-08
cg08359464	3	128370361	*RPN1*	0.518	0.412	0.482	-0.106	-0.036	2.12E-103	4.27E-09
cg19982684	3	128403035	NA	0.408	0.318	0.371	-0.090	-0.037	4.16E-127	6.38E-17
cg01223011	3	128714144	*KIAA1257*	0.729	0.780	0.754	0.051	0.024	2.15E-43	3.30E-06
cg03389538	3	128779498	*GP9*	0.377	0.306	0.347	-0.071	-0.030	2.27E-140	1.26E-16
cg16824282	3	128779590	*GP9*	0.479	0.377	0.443	-0.102	-0.035	1.81E-120	7.48E-10
cg02726291	3	128779596	*GP9*	0.383	0.302	0.353	-0.082	-0.030	3.37E-107	3.34E-09
cg07280097	3	128779601	*GP9*	0.383	0.296	0.349	-0.087	-0.034	2.01E-124	2.54E-11
cg00221718	3	128914668	NA	0.334	0.250	0.299	-0.084	-0.035	5.67E-129	1.30E-13
cg16850690	3	129318459	*PLXND1*	0.338	0.275	0.313	-0.063	-0.025	8.21E-132	6.43E-17
cg12196294	3	129575053	*TMCC1*	0.469	0.363	0.443	-0.105	-0.026	3.66E-129	2.29E-06
cg13799772	3	129800573	*ALG1L2*	0.402	0.348	0.376	-0.054	-0.026	5.94E-44	5.03E-06
cg15666143	3	130061073	NA	0.662	0.726	0.701	0.064	0.039	1.56E-36	5.35E-07
cg07892374	3	130568122	*ATP2C1*	0.299	0.238	0.275	-0.061	-0.024	1.35E-117	1.35E-11
cg05936004	3	130693828	*ATP2C1*	0.530	0.415	0.486	-0.115	-0.044	8.01E-155	1.96E-16
cg26346167	3	131004472	*NEK11*	0.673	0.724	0.710	0.051	0.037	4.95E-31	4.37E-08
cg10469112	3	131388542	*CPNE4*	0.736	0.787	0.790	0.052	0.054	8.78E-22	1.67E-11
cg15958424	3	132036067	*ACPP*	0.406	0.320	0.371	-0.085	-0.035	3.84E-118	1.75E-12
cg19770281	3	132261993	NA	0.306	0.237	0.276	-0.070	-0.030	3.12E-82	2.04E-08
cg11642298	3	133193741	*BFSP2*	0.460	0.372	0.410	-0.089	-0.050	6.90E-110	1.18E-17
cg24686551	3	134047274	NA	0.436	0.326	0.401	-0.109	-0.035	7.10E-122	8.55E-09
cg22074114	3	134326341	*KY*	0.457	0.358	0.414	-0.099	-0.044	5.26E-134	2.18E-18
cg18260919	3	135869103	*MSL2*	0.711	0.769	0.761	0.058	0.050	1.35E-22	2.09E-08
cg00382999	3	136649333	*NCK1*	0.446	0.343	0.407	-0.102	-0.039	3.70E-132	2.12E-13
cg09866565	3	137844436	*A4GNT*	0.352	0.299	0.316	-0.053	-0.035	4.69E-35	2.46E-08
cg04554122	3	137938348	*ARMC8*	0.418	0.333	0.382	-0.086	-0.037	4.05E-111	4.39E-12
cg27200630	3	138494226	NA	0.333	0.245	0.292	-0.088	-0.041	4.60E-129	1.02E-17
cg14573478	3	140730858	NA	0.706	0.756	0.746	0.051	0.040	4.65E-33	9.23E-11
cg09599062	3	141995701	NA	0.348	0.266	0.304	-0.082	-0.044	2.58E-82	7.37E-14
cg16271453	3	142027066	*XRN1*	0.724	0.776	0.772	0.052	0.048	2.30E-20	3.74E-09
cg13408795	3	143050350	*SLC9A9*	0.468	0.402	0.431	-0.065	-0.036	7.67E-65	8.65E-12
cg25945642	3	143321098	*SLC9A9*	0.486	0.396	0.444	-0.090	-0.043	1.36E-69	5.21E-09
cg24362960	3	144064632	NA	0.746	0.791	0.800	0.045	0.054	3.77E-23	2.59E-14
cg06981309	3	146260954	*PLSCR1*	0.540	0.304	0.392	-0.237	-0.149	4.93E-157	4.10E-51
cg04121771	3	149191624	*TM4SF4*	0.548	0.483	0.513	-0.065	-0.035	1.42E-54	2.88E-10
cg19422253	3	149212391	*TM4SF4*	0.585	0.620	0.643	0.036	0.058	6.21E-11	1.41E-11
cg12874219	3	149218938	*TM4SF4*	0.739	0.787	0.791	0.048	0.052	2.37E-23	1.83E-12
cg05902522	3	149348590	*WWTR1*	0.608	0.542	0.586	-0.066	-0.022	6.70E-76	5.69E-06
cg07578772	3	150420821	*FAM194A*	0.715	0.788	0.754	0.073	0.038	9.68E-97	1.40E-15
cg23214895	3	150456069	NA	0.693	0.753	0.741	0.060	0.049	1.07E-41	1.17E-14
cg24721964	3	150662697	*CLRN1*	0.293	0.231	0.245	-0.062	-0.047	3.12E-63	2.55E-17
cg15814508	3	150996078	*P2RY14*	0.252	0.191	0.219	-0.061	-0.033	1.52E-92	1.61E-14
cg03379681	3	151058527	*P2RY12*	0.263	0.207	0.225	-0.056	-0.038	2.16E-78	1.24E-19
cg18143869	3	151072858	*P2RY12*	0.766	0.819	0.815	0.053	0.049	8.72E-24	1.27E-09
cg15664462	3	151996146	*MBNL1*	0.662	0.727	0.714	0.064	0.052	6.92E-40	2.90E-12

cg13702222	3	152017240	*MBNL1*	0.340	0.394	0.374	0.053	0.034	9.65E-31	1.93E-07
cg03857198	3	152927597	NA	0.470	0.358	0.428	-0.112	-0.042	4.65E-187	7.78E-22
cg13033061	3	155198149	*PLCH1*	0.699	0.759	0.747	0.061	0.049	2.30E-28	9.08E-10
cg26690511	3	155422103	*PLCH1*	0.429	0.367	0.401	-0.062	-0.028	4.10E-50	1.19E-06
cg25234117	3	155423168	*PLCH1*	0.342	0.275	0.314	-0.067	-0.028	5.59E-87	6.91E-09
cg25375916	3	155570275	*SLC33A1*	0.310	0.245	0.282	-0.065	-0.028	6.75E-76	5.66E-08
cg02551745	3	156809115	*LOC1004988*	0.424	0.322	0.382	-0.103	-0.043	3.67E-126	1.16E-14
cg01303420	3	157296174	*C3orf55*	0.650	0.703	0.710	0.053	0.060	2.68E-19	2.16E-11
cg04481170	3	158445995	*RARRES1*	0.381	0.322	0.335	-0.059	-0.046	5.08E-35	1.92E-10
cg25050026	3	158519410	*MFSD1*	0.237	0.178	0.209	-0.059	-0.028	7.98E-79	1.12E-09
cg13388769	3	159665668	NA	0.771	0.823	0.801	0.052	0.030	3.09E-52	1.09E-08
cg05581878	3	159886981	NA	0.482	0.426	0.457	-0.056	-0.026	7.87E-53	1.64E-06
cg12785694	3	160122168	*SMC4*	0.338	0.262	0.282	-0.076	-0.057	4.08E-68	1.23E-19
cg13027369	3	160279320	*KPNA4*	0.658	0.722	0.702	0.064	0.044	1.74E-36	3.45E-09
cg02470874	3	160475336	*PPM1L*	0.364	0.313	0.341	-0.052	-0.023	7.00E-59	1.89E-07
cg09883188	3	160502317	*PPM1L*	0.667	0.724	0.709	0.057	0.043	4.29E-36	5.66E-11
cg26406186	3	160520336	*PPM1L*	0.732	0.787	0.779	0.055	0.047	7.25E-33	4.67E-11
cg04903759	3	167238645	*WDR49*	0.465	0.345	0.413	-0.121	-0.053	5.60E-139	1.37E-16
cg02556393	3	168866705	*MECOM*	0.462	0.398	0.421	-0.064	-0.041	1.37E-34	1.02E-07
cg05721199	3	169629136	*SAMD7*	0.700	0.762	0.768	0.061	0.068	6.40E-27	2.79E-15
cg02784232	3	169897975	*PHC3*	0.537	0.595	0.566	0.058	0.029	3.38E-40	5.81E-06
cg21140290	3	169975155	*PRKCI*	0.742	0.800	0.789	0.058	0.047	4.30E-31	1.01E-10
cg02499139	3	171146337	*TNIK*	0.287	0.234	0.266	-0.054	-0.021	1.07E-66	1.29E-06
cg13303534	3	171261809	NA	0.494	0.422	0.440	-0.072	-0.053	8.58E-53	4.41E-16
cg09467433	3	171283606	NA	0.490	0.375	0.451	-0.115	-0.039	1.09E-121	1.10E-09
cg26074575	3	171764199	*FNDC3B*	0.657	0.709	0.704	0.052	0.046	4.42E-24	2.93E-09
cg04319611	3	171784422	*FNDC3B*	0.403	0.334	0.373	-0.069	-0.031	1.28E-66	5.91E-08
cg14016236	3	171792545	*FNDC3B*	0.430	0.347	0.392	-0.083	-0.038	2.91E-92	1.62E-11
cg06740950	3	171878318	*FNDC3B*	0.435	0.335	0.388	-0.100	-0.047	3.00E-110	2.34E-13
cg18802998	3	172444423	NA	0.436	0.360	0.401	-0.076	-0.035	3.19E-73	8.95E-10
cg02910194	3	173438075	*NLGN1*	0.758	0.804	0.812	0.046	0.054	1.58E-18	8.73E-13
cg06160853	3	174155855	NA	0.380	0.303	0.349	-0.077	-0.031	4.14E-69	2.85E-06
cg01379656	3	174970696	*NAALADL2*	0.709	0.759	0.747	0.051	0.039	1.00E-44	9.45E-12
cg20230856	3	176769657	*TBL1XR1*	0.772	0.815	0.825	0.044	0.053	9.15E-16	2.12E-11
cg01132484	3	176916496	*TBL1XR1*	0.647	0.698	0.676	0.051	0.029	1.40E-34	1.89E-06
cg01178099	3	177391387	*LINC00578*	0.491	0.385	0.454	-0.106	-0.037	1.03E-121	1.60E-10
cg17769523	3	182003594	NA	0.721	0.774	0.759	0.053	0.038	3.81E-29	7.07E-08
cg01988602	3	182473048	NA	0.688	0.750	0.725	0.063	0.037	5.45E-36	5.16E-07
cg27368333	3	182677779	*DCUN1D1*	0.320	0.249	0.295	-0.070	-0.024	5.97E-85	4.71E-06
cg15439110	3	183153901	NA	0.712	0.776	0.758	0.064	0.045	3.12E-38	1.62E-09
cg23189692	3	184050393	*EIF4G1*	0.381	0.297	0.352	-0.084	-0.029	1.16E-108	5.29E-08
cg20438472	3	184293365	*EPHB3*	0.543	0.473	0.510	-0.070	-0.033	1.35E-77	2.52E-10
cg15334250	3	184297495	*EPHB3*	0.387	0.287	0.348	-0.099	-0.039	9.92E-86	1.02E-07
cg27214730	3	184297522	*EPHB3*	0.253	0.190	0.228	-0.063	-0.025	1.49E-74	3.35E-06
cg04132186	3	184322709	NA	0.292	0.228	0.267	-0.064	-0.025	1.94E-84	6.07E-08
cg26102434	3	184975890	NA	0.708	0.761	0.749	0.053	0.041	8.56E-20	4.50E-06
cg10498502	3	185928164	*DGKG*	0.427	0.356	0.389	-0.070	-0.038	4.57E-87	1.50E-14
cg27374881	3	186504006	*EIF4A2*	0.680	0.740	0.721	0.060	0.041	4.91E-44	1.43E-10
cg10168494	3	187635183	NA	0.303	0.228	0.254	-0.075	-0.049	3.43E-95	1.57E-21
cg06739107	3	188399479	*LPP*	0.495	0.382	0.463	-0.114	-0.032	1.42E-128	3.22E-08
cg25853622	3	188425256	*LPP*	0.416	0.312	0.377	-0.103	-0.039	2.99E-128	8.90E-11
cg26466145	3	188665930	NA	0.443	0.382	0.412	-0.062	-0.031	1.36E-40	5.80E-06
cg20168849	3	188995030	*TPRG1*	0.476	0.391	0.436	-0.085	-0.040	8.59E-95	6.88E-13
cg10599446	3	189073674	NA	0.694	0.757	0.744	0.063	0.050	2.89E-46	1.69E-14
cg10475153	3	190024765	*CLDN1*	0.576	0.634	0.601	0.057	0.025	1.04E-73	3.43E-09
cg16588163	3	190251464	*IL1RAP*	0.358	0.287	0.325	-0.070	-0.033	4.17E-89	5.06E-12
cg04051365	3	193586394	NA	0.444	0.340	0.401	-0.104	-0.044	2.53E-148	3.88E-17
cg14475875	3	193856076	*HES1*	0.257	0.189	0.220	-0.068	-0.037	6.72E-68	5.43E-11
cg07141504	3	194030706	NA	0.247	0.192	0.220	-0.054	-0.027	7.50E-87	2.93E-12
cg05323324	3	194120023	*GP5*	0.332	0.257	0.291	-0.075	-0.041	8.47E-109	2.47E-17
cg00181432	3	194393818	*LSG1*	0.431	0.353	0.387	-0.077	-0.044	8.91E-95	2.00E-15
cg04252203	3	194696866	NA	0.425	0.337	0.393	-0.088	-0.032	2.06E-135	5.12E-13
cg22831526	3	194706168	NA	0.313	0.244	0.284	-0.069	-0.030	3.59E-62	4.80E-07
cg19729744	3	194752020	NA	0.645	0.594	0.592	-0.051	-0.053	4.77E-17	4.39E-09
cg21501175	3	194893148	*XXYLT1*	0.459	0.360	0.422	-0.099	-0.037	3.06E-99	3.22E-08
cg09010699	3	195171693	NA	0.558	0.431	0.527	-0.126	-0.030	8.57E-194	5.13E-10
cg03296935	3	195946851	*SLC51A*	0.293	0.237	0.265	-0.055	-0.027	5.76E-78	4.41E-11
cg04855678	3	195946921	*SLC51A*	0.368	0.272	0.331	-0.095	-0.036	1.52E-158	1.08E-16
cg21470711	3	195995846	*PCYT1A*	0.303	0.233	0.272	-0.070	-0.031	8.00E-76	8.32E-08
cg00828556	3	196351986	NA	0.400	0.295	0.368	-0.105	-0.031	2.16E-128	1.06E-07
cg25771026	3	196352027	NA	0.442	0.361	0.423	-0.082	-0.019	6.71E-127	2.71E-06
cg06690535	3	196352142	NA	0.416	0.321	0.372	-0.095	-0.044	7.93E-147	8.82E-20
cg04840813	3	196363641	NA	0.314	0.260	0.289	-0.054	-0.025	1.65E-62	2.52E-07
cg07481360	3	196902551	*DLG1*	0.676	0.727	0.720	0.051	0.044	1.54E-22	1.06E-08
cg18095675	3	197272311	*BDH1*	0.637	0.529	0.605	-0.108	-0.032	6.48E-148	4.35E-11
cg16570846	3	197522465	*LRCH3*	0.743	0.790	0.795	0.048	0.052	1.91E-19	1.69E-12
cg17952262	4	683240	*MFSD7*	0.324	0.271	0.301	-0.053	-0.024	2.65E-77	2.19E-10
cg08769212	4	930351	*TMEM175*	0.754	0.812	0.806	0.059	0.053	1.46E-32	7.38E-13

cg11977605	4	1076247	*RNF212*	0.308	0.247	0.275	-0.061	-0.033	8.07E-96	1.80E-16
cg26923863	4	1221838	*CTBP1*	0.281	0.204	0.253	-0.077	-0.028	5.45E-96	2.16E-08
cg10085474	4	1221935	*CTBP1*	0.395	0.322	0.371	-0.073	-0.024	2.03E-117	2.29E-08
cg07241925	4	1294566	*MAEA*	0.416	0.330	0.386	-0.086	-0.030	3.88E-119	7.36E-11
cg21326139	4	1294783	*MAEA*	0.390	0.297	0.353	-0.092	-0.036	6.05E-130	8.18E-14
cg24597363	4	1294996	*MAEA*	0.506	0.393	0.469	-0.113	-0.037	9.95E-124	1.35E-09
cg23447233	4	1295047	*MAEA*	0.327	0.247	0.305	-0.080	-0.022	2.33E-115	1.85E-06
cg24973755	4	1304972	*MAEA*	0.724	0.807	0.760	0.084	0.036	2.07E-111	1.07E-13
cg10133462	4	1305113	*MAEA*	0.741	0.803	0.775	0.062	0.034	4.66E-96	1.57E-17
cg15457390	4	2844730	*ADD1*	0.228	0.174	0.203	-0.055	-0.025	1.30E-67	1.27E-07
cg24466927	4	3315925	*RGS12*	0.694	0.748	0.743	0.054	0.049	2.03E-23	3.26E-09
cg08472008	4	3531603	*LRPAP1*	0.384	0.287	0.351	-0.097	-0.033	2.92E-135	3.20E-11
cg17430979	4	6034828	*JAKMIP1*	0.528	0.460	0.473	-0.068	-0.055	2.70E-34	5.07E-12
cg26233331	4	6695614	*S100P*	0.366	0.276	0.329	-0.090	-0.037	9.13E-173	4.92E-22
cg15686615	4	6781565	NA	0.504	0.453	0.479	-0.051	-0.025	9.90E-74	6.34E-11
cg16506970	4	6809109	*KIAA0232*	0.448	0.350	0.414	-0.098	-0.033	2.06E-94	1.75E-07
cg26414257	4	6888038	NA	0.307	0.245	0.278	-0.062	-0.029	4.54E-110	1.01E-15
cg14594044	4	6914621	*TBC1D14*	0.540	0.479	0.516	-0.061	-0.025	4.02E-86	7.37E-09
cg14647287	4	6927183	*TBC1D14*	0.407	0.334	0.375	-0.073	-0.032	5.30E-102	1.04E-15
cg06496803	4	6940915	*TBC1D14*	0.443	0.370	0.415	-0.073	-0.028	1.98E-95	1.05E-08
cg06875488	4	7657070	*SORCS2*	0.363	0.312	0.337	-0.051	-0.026	4.65E-39	1.61E-06
cg04177251	4	8193123	NA	0.328	0.264	0.301	-0.065	-0.028	4.98E-67	9.81E-08
cg25982657	4	8193353	NA	0.296	0.244	0.271	-0.051	-0.025	8.04E-60	2.58E-08
cg10049789	4	8202531	*SH3TC1*	0.345	0.264	0.298	-0.080	-0.046	7.87E-51	1.52E-09
cg04370174	4	8207250	*SH3TC1*	0.376	0.303	0.339	-0.073	-0.037	3.91E-114	7.22E-18
cg02933639	4	8207325	*SH3TC1*	0.434	0.374	0.413	-0.060	-0.021	8.59E-81	2.26E-07
cg04605681	4	8229159	*SH3TC1*	0.337	0.266	0.313	-0.071	-0.023	8.40E-108	3.71E-08
cg07248017	4	8230689	*SH3TC1*	0.670	0.727	0.700	0.057	0.030	9.70E-91	1.67E-15
cg19339902	4	8292261	*HTRA3*	0.383	0.313	0.351	-0.070	-0.033	1.51E-74	1.55E-09
cg16785938	4	10022984	*SLC2A9*	0.423	0.347	0.391	-0.076	-0.031	6.24E-127	1.82E-15
cg25117600	4	10023201	*SLC2A9*	0.338	0.254	0.291	-0.085	-0.048	4.75E-77	2.27E-12
cg22358291	4	10101553	*WDR1*	0.735	0.799	0.762	0.064	0.027	1.66E-124	1.15E-12
cg20391895	4	10686394	*CLNK*	0.301	0.245	0.268	-0.056	-0.033	3.20E-75	2.09E-13
cg10438589	4	14531493	NA	0.468	0.392	0.435	-0.076	-0.033	7.54E-58	1.14E-06
cg00708789	4	16083440	*PROM1*	0.342	0.267	0.304	-0.075	-0.038	1.51E-92	1.99E-14
cg06861395	4	23776092	NA	0.684	0.734	0.719	0.050	0.035	1.52E-23	3.98E-06
cg25659893	4	24043352	NA	0.715	0.769	0.760	0.054	0.044	1.23E-18	7.81E-07
cg12977548	4	24579968	*DHX15*	0.612	0.679	0.647	0.067	0.035	6.86E-41	1.10E-06
cg04349839	4	25789390	*SEL1L3*	0.559	0.480	0.520	-0.080	-0.040	8.61E-82	2.31E-11
cg05065948	4	26275089	NA	0.512	0.422	0.476	-0.089	-0.036	9.47E-99	2.42E-11
cg04366628	4	26290463	NA	0.374	0.301	0.341	-0.073	-0.033	8.83E-60	5.51E-07
cg14209138	4	28227663	NA	0.744	0.797	0.804	0.053	0.060	6.05E-24	2.07E-13
cg08343347	4	36076000	*ARAP2*	0.695	0.748	0.746	0.053	0.050	1.81E-19	1.81E-08
cg00812557	4	38073835	*TBC1D1*	0.330	0.255	0.289	-0.075	-0.040	1.88E-153	1.96E-27
cg24279243	4	38676559	*KLF3*	0.250	0.186	0.213	-0.063	-0.036	4.45E-47	2.81E-07
cg14665413	4	38859728	*TLR6*	0.266	0.203	0.230	-0.063	-0.036	2.62E-76	1.08E-12
cg14578677	4	38859770	*TLR6*	0.516	0.398	0.474	-0.119	-0.043	1.78E-121	6.38E-10
cg04747180	4	39116218	*KLHL5*	0.470	0.351	0.421	-0.119	-0.050	3.77E-161	2.91E-20
cg24403649	4	39172243	NA	0.454	0.391	0.428	-0.063	-0.026	1.24E-133	1.01E-14
cg13084677	4	39482740	*LOC401127*	0.444	0.336	0.403	-0.108	-0.041	1.65E-114	4.56E-10
cg20847110	4	39482781	*LOC401127*	0.423	0.322	0.379	-0.101	-0.044	4.58E-138	2.33E-16
cg10925991	4	40324096	NA	0.366	0.296	0.333	-0.070	-0.032	1.31E-82	2.77E-10
cg16000989	4	41983716	*DCAF4L1*	0.532	0.449	0.502	-0.083	-0.031	6.47E-75	2.35E-06
cg27080171	4	46388320	*GABRA2*	0.550	0.603	0.600	0.053	0.050	9.28E-21	1.01E-08
cg00876141	4	47837775	*CORIN*	0.381	0.311	0.343	-0.070	-0.038	7.84E-105	9.11E-20
cg24494102	4	48015981	*CNGA1*	0.668	0.719	0.699	0.051	0.031	2.06E-32	1.13E-06
cg22767754	4	48038871	*NIPAL1*	0.297	0.239	0.275	-0.058	-0.023	1.84E-94	4.44E-08
cg02600394	4	48136234	*TXK*	0.647	0.718	0.683	0.071	0.036	2.61E-129	4.07E-21
cg20981615	4	48136280	*TXK*	0.682	0.756	0.721	0.074	0.040	3.19E-123	5.05E-21
cg17984638	4	48136452	*TXK*	0.778	0.838	0.812	0.061	0.034	1.88E-96	4.96E-15
cg08700083	4	48382815	*SLAIN2*	0.408	0.319	0.377	-0.089	-0.030	5.51E-106	6.05E-08
cg14064774	4	48540281	*FRYL*	0.769	0.813	0.823	0.045	0.054	1.74E-18	3.92E-12
cg22152446	4	48754953	*FRYL*	0.477	0.375	0.444	-0.101	-0.033	1.29E-103	1.95E-08
cg21253943	4	53968201	*SCFD2*	0.742	0.793	0.791	0.051	0.050	6.96E-31	1.08E-13
cg06495586	4	54424787	*LNX1*	0.283	0.226	0.244	-0.057	-0.039	3.47E-59	2.86E-13
cg10129391	4	54582825	NA	0.407	0.342	0.367	-0.064	-0.040	4.44E-53	3.96E-11
cg18242288	4	54793620	NA	0.615	0.503	0.572	-0.111	-0.043	5.40E-133	5.38E-14
cg18463607	4	56718320	*EXOC1*	0.770	0.827	0.800	0.057	0.030	3.84E-84	4.57E-11
cg20618651	4	56718365	*EXOC1*	0.692	0.766	0.738	0.074	0.046	1.19E-112	2.04E-22
cg16414852	4	70626128	*SULT1B1*	0.465	0.520	0.517	0.055	0.052	3.08E-29	2.73E-12
cg11363527	4	72125918	*SLC4A4*	0.277	0.220	0.246	-0.057	-0.031	2.67E-77	1.20E-11
cg02951062	4	74269908	*ALB*	0.670	0.718	0.723	0.048	0.053	5.94E-16	4.53E-09
cg18007641	4	74641828	NA	0.514	0.574	0.567	0.060	0.054	5.84E-21	1.27E-07
cg12586707	4	74738902	NA	0.281	0.222	0.243	-0.059	-0.038	3.95E-71	1.62E-15
cg18356190	4	74964260	*CXCL2*	0.287	0.235	0.262	-0.052	-0.025	1.01E-53	7.84E-07
cg19747465	4	75174074	*EPGN*	0.662	0.716	0.708	0.055	0.047	3.00E-38	3.76E-14
cg07643762	4	75174080	*EPGN*	0.745	0.796	0.787	0.051	0.042	3.14E-35	1.47E-12
cg23421166	4	76976499	*ART3*	0.761	0.812	0.799	0.051	0.038	3.30E-36	6.09E-10

cg22301128	4	77011716	*ART3*	0.326	0.261	0.298	-0.065	-0.028	3.92E-57	1.68E-06
cg13870520	4	77131705	*SCARB2*	0.479	0.370	0.427	-0.109	-0.052	6.39E-157	3.81E-21
cg05146536	4	77140678	*FAM47E*	0.304	0.234	0.263	-0.070	-0.041	2.34E-86	4.73E-18
cg03184410	4	77613822	*SHROOM3*	0.719	0.764	0.772	0.045	0.054	1.13E-14	3.08E-09
cg14934522	4	77618676	*SHROOM3*	0.690	0.741	0.721	0.050	0.031	2.87E-36	1.99E-07
cg14091154	4	79358764	*FRAS1*	0.665	0.723	0.703	0.058	0.038	3.69E-39	5.96E-09
cg10713839	4	81047158	NA	0.644	0.700	0.680	0.057	0.036	1.70E-49	2.80E-11
cg04858148	4	81117016	*PRDM8*	0.333	0.243	0.280	-0.090	-0.053	2.08E-140	7.90E-30
cg11193767	4	83930905	*LIN54*	0.417	0.315	0.377	-0.102	-0.040	3.67E-203	3.98E-23
cg03321813	4	84257153	*HPSE*	0.335	0.262	0.308	-0.074	-0.028	2.39E-118	2.64E-11
cg08017911	4	84526934	*AGPAT9*	0.784	0.837	0.824	0.052	0.039	2.89E-34	1.91E-09
cg02394698	4	86594376	*ARHGAP24*	0.329	0.260	0.297	-0.068	-0.032	1.25E-87	3.34E-10
cg02262553	4	87849250	*LOC1005067*	0.426	0.315	0.380	-0.111	-0.046	7.19E-140	9.52E-16
cg10338518	4	87895137	*AFF1*	0.461	0.345	0.416	-0.117	-0.046	5.06E-146	1.29E-17
cg19320820	4	87966326	*AFF1*	0.310	0.229	0.281	-0.081	-0.029	1.65E-98	1.27E-07
cg02979487	4	88014081	*AFF1*	0.300	0.241	0.282	-0.059	-0.019	1.68E-91	5.16E-06
cg03926751	4	88140261	*KLHL8*	0.424	0.315	0.382	-0.109	-0.041	1.48E-123	3.19E-10
cg02549628	4	88896208	*SPP1*	0.243	0.189	0.219	-0.054	-0.024	1.50E-67	1.13E-07
cg02215171	4	89379156	*HERC5*	0.414	0.316	0.361	-0.098	-0.053	1.12E-98	4.17E-20
cg06884401	4	89978251	*FAM13A*	0.309	0.239	0.283	-0.070	-0.026	5.02E-97	2.17E-09
cg04536922	4	89978566	*FAM13A*	0.303	0.224	0.268	-0.079	-0.035	8.54E-78	5.18E-08
cg14665366	4	90227740	*GPRIN3*	0.757	0.817	0.787	0.060	0.029	1.18E-77	7.64E-10
cg13681468	4	90227879	*GPRIN3*	0.737	0.796	0.772	0.060	0.035	1.04E-49	1.61E-08
cg03658557	4	96012317	*BMPR1B*	0.290	0.228	0.255	-0.062	-0.035	1.44E-75	1.51E-11
cg03234557	4	99404186	*TSPAN5*	0.745	0.796	0.762	0.051	0.017	7.45E-87	4.19E-07
cg03298704	4	99427937	*TSPAN5*	0.671	0.721	0.723	0.049	0.051	6.62E-20	2.38E-10
cg08979352	4	99580530	*TSPAN5*	0.493	0.421	0.449	-0.072	-0.043	2.07E-40	3.14E-08
cg20107506	4	100874826	NA	0.508	0.396	0.475	-0.111	-0.032	5.44E-108	1.15E-07
cg02193956	4	103680201	*MANBA*	0.413	0.319	0.378	-0.093	-0.035	6.12E-91	5.64E-08
cg12306086	4	106117747	*TET2*	0.409	0.301	0.353	-0.108	-0.056	1.49E-145	1.78E-22
cg17862558	4	106137048	*TET2*	0.738	0.799	0.777	0.061	0.039	3.28E-57	9.91E-13
cg22365313	4	108636320	*PAPSS1*	0.391	0.308	0.351	-0.082	-0.040	4.28E-99	7.19E-13
cg26701826	4	108814604	*SGMS2*	0.413	0.314	0.373	-0.099	-0.039	3.94E-160	6.02E-16
cg01734045	4	109967535	*COL25A1*	0.288	0.233	0.254	-0.055	-0.034	9.58E-62	3.74E-11
cg09451574	4	113069076	*C4orf32*	0.628	0.551	0.578	-0.076	-0.049	8.56E-49	6.85E-13
cg25198847	4	113192515	NA	0.547	0.440	0.510	-0.107	-0.037	9.16E-103	2.20E-08
cg12311175	4	118975504	*NDST3*	0.763	0.815	0.790	0.052	0.027	8.56E-42	2.21E-06
cg16640599	4	119732131	*SEC24D*	0.437	0.325	0.387	-0.112	-0.050	7.57E-195	3.84E-28
cg20118822	4	119831531	*SYNPO2*	0.723	0.784	0.770	0.061	0.047	3.68E-38	1.53E-11
cg12973294	4	119869807	*SYNPO2*	0.546	0.468	0.511	-0.078	-0.035	5.11E-57	7.74E-07
cg13541713	4	119947251	*SYNPO2*	0.449	0.355	0.418	-0.094	-0.031	5.91E-100	5.58E-08
cg24504349	4	120974344	NA	0.448	0.335	0.403	-0.113	-0.044	1.89E-146	5.87E-15
cg07159131	4	122867651	*TRPC3*	0.755	0.803	0.806	0.049	0.051	1.17E-15	1.74E-08
cg17139861	4	123304626	*ADAD1*	0.735	0.786	0.790	0.050	0.054	2.33E-24	1.82E-12
cg19782686	4	124180616	*SPATA5*	0.440	0.363	0.399	-0.077	-0.041	2.60E-79	9.53E-13
cg15082040	4	124299999	NA	0.634	0.698	0.691	0.064	0.057	3.91E-37	5.44E-14
cg26306329	4	124400196	NA	0.327	0.251	0.303	-0.075	-0.024	1.10E-99	1.11E-06
cg23829102	4	124476694	NA	0.251	0.188	0.209	-0.063	-0.042	1.32E-75	3.48E-16
cg24727114	4	124707571	*LOC285419*	0.736	0.789	0.764	0.053	0.028	1.07E-36	6.43E-06
cg04525852	4	125632538	*ANKRD50*	0.322	0.234	0.278	-0.088	-0.044	5.29E-110	6.72E-15
cg12771751	4	128647957	NA	0.727	0.779	0.765	0.052	0.038	3.32E-23	1.89E-06
cg04998379	4	129491708	NA	0.227	0.167	0.199	-0.060	-0.029	1.33E-69	1.85E-09
cg19851526	4	130257063	NA	0.689	0.739	0.730	0.050	0.041	1.46E-19	6.59E-07
cg07661704	4	139144433	*SLC7A11*	0.502	0.555	0.543	0.053	0.041	4.40E-28	1.62E-09
cg05088386	4	140060774	*ELF2*	0.698	0.750	0.753	0.053	0.055	1.12E-19	2.35E-10
cg03418002	4	140655478	*MGST2*	0.479	0.373	0.446	-0.105	-0.032	5.68E-110	4.70E-08
cg16596440	4	140684596	*MAML3*	0.263	0.206	0.231	-0.057	-0.031	2.00E-71	4.41E-11
cg03322353	4	141069770	*MAML3*	0.498	0.394	0.456	-0.104	-0.043	1.26E-114	6.16E-12
cg00563107	4	141173528	NA	0.574	0.491	0.527	-0.084	-0.048	2.18E-83	1.58E-16
cg11088672	4	142678613	NA	0.370	0.307	0.337	-0.063	-0.032	6.28E-60	2.01E-09
cg01719995	4	144104893	*USP38*	0.334	0.258	0.303	-0.076	-0.030	1.79E-96	7.53E-09
cg03967798	4	145268453	NA	0.437	0.368	0.402	-0.068	-0.035	3.37E-65	6.50E-11
cg16102040	4	146685656	*ZNF827*	0.614	0.518	0.585	-0.096	-0.030	1.29E-121	1.42E-10
cg07091220	4	146752115	*ZNF827*	0.460	0.392	0.428	-0.067	-0.031	7.68E-52	7.25E-07
cg02736966	4	148119477	NA	0.676	0.727	0.705	0.051	0.029	2.94E-34	5.50E-06
cg26338428	4	148767763	*ARHGAP10*	0.692	0.763	0.737	0.071	0.045	2.07E-57	2.45E-11
cg05437692	4	149362435	*NR3C2*	0.365	0.313	0.324	-0.053	-0.042	4.82E-43	6.40E-15
cg14904733	4	151501623	*LRBA*	0.717	0.759	0.767	0.043	0.051	7.38E-19	6.69E-12
cg20481837	4	151501627	*LRBA*	0.769	0.810	0.820	0.040	0.051	5.17E-15	3.14E-11
cg24789682	4	152217416	NA	0.701	0.756	0.736	0.055	0.035	4.76E-31	1.22E-06
cg06270074	4	153353276	*FBXW7*	0.776	0.814	0.829	0.038	0.053	4.15E-12	9.62E-10
cg17385088	4	153611560	NA	0.489	0.398	0.457	-0.091	-0.032	1.41E-152	1.55E-12
cg12081645	4	157920476	NA	0.340	0.263	0.303	-0.077	-0.037	3.68E-78	1.11E-09
cg00861646	4	160026608	NA	0.369	0.290	0.338	-0.079	-0.030	6.57E-88	6.08E-08
cg21058391	4	160027487	NA	0.558	0.487	0.509	-0.072	-0.050	5.45E-61	1.41E-14
cg04399899	4	160189254	*RAPGEF2*	0.407	0.321	0.374	-0.086	-0.033	3.06E-92	5.33E-08
cg24221738	4	166125552	NA	0.757	0.811	0.778	0.054	0.022	1.65E-65	6.41E-06
cg04256466	4	169752398	*PALLD*	0.267	0.213	0.231	-0.054	-0.036	6.43E-56	9.63E-12

cg14477767	4	170195438	NA	0.779	0.834	0.825	0.055	0.046	2.18E-34	3.74E-13
cg03580292	4	174411723	NA	0.349	0.263	0.300	-0.087	-0.049	2.40E-96	9.04E-18
cg25249713	4	177045356	*WDR17*	0.754	0.807	0.790	0.053	0.036	1.19E-44	2.18E-10
cg14022913	4	178229651	*NEIL3*	0.416	0.333	0.383	-0.084	-0.033	5.51E-77	6.16E-08
cg04556418	4	184367379	*CDKN2AIP*	0.621	0.550	0.590	-0.071	-0.031	7.93E-72	8.62E-09
cg18827396	4	184368864	*CDKN2AIP*	0.789	0.840	0.822	0.052	0.034	1.10E-45	1.96E-11
cg03427849	4	184578976	*TRAPPC11*	0.525	0.585	0.558	0.060	0.034	2.22E-51	8.45E-09
cg09544144	4	185777688	NA	0.368	0.306	0.331	-0.061	-0.037	4.27E-53	1.19E-10
cg12599598	4	185923887	NA	0.492	0.407	0.460	-0.084	-0.032	4.17E-75	4.51E-07
cg08277306	5	598817	NA	0.609	0.671	0.645	0.061	0.035	2.33E-46	4.26E-09
cg03999941	5	957511	NA	0.703	0.761	0.730	0.058	0.026	9.64E-45	5.46E-06
cg13674369	5	969918	NA	0.719	0.770	0.747	0.050	0.028	9.34E-66	9.42E-11
cg04131890	5	969939	NA	0.660	0.715	0.691	0.055	0.030	4.80E-44	1.32E-07
cg21946374	5	1108401	*SLC12A7*	0.416	0.342	0.391	-0.074	-0.025	2.71E-139	3.38E-12
cg18997983	5	1109031	*SLC12A7*	0.463	0.366	0.420	-0.097	-0.042	3.28E-121	1.56E-12
cg02739870	5	1113320	*SLC12A7*	0.349	0.278	0.306	-0.071	-0.043	4.90E-69	7.54E-14
cg24000908	5	1113358	*SLC12A7*	0.335	0.284	0.292	-0.051	-0.043	1.52E-56	8.68E-22
cg00675600	5	1255458	*TERT*	0.746	0.803	0.779	0.057	0.033	4.55E-111	2.37E-23
cg16429735	5	1268949	*TERT*	0.670	0.732	0.710	0.061	0.039	1.54E-49	3.55E-10
cg26002008	5	1477719	*LPCAT1*	0.378	0.304	0.355	-0.074	-0.023	4.95E-120	2.16E-09
cg14049990	5	1514140	*LPCAT1*	0.389	0.317	0.361	-0.072	-0.028	9.95E-113	4.41E-11
cg23939096	5	1555791	NA	0.429	0.324	0.393	-0.105	-0.037	1.16E-106	2.81E-08
cg07283217	5	3283037	NA	0.743	0.790	0.800	0.047	0.057	2.17E-20	1.10E-13
cg23275644	5	10457079	*ROPN1L*	0.546	0.460	0.512	-0.085	-0.034	7.69E-82	3.78E-08
cg17390918	5	10502512	NA	0.432	0.359	0.402	-0.073	-0.030	2.53E-78	1.23E-07
cg08944026	5	10626811	*ANKRD33B*	0.389	0.295	0.352	-0.094	-0.037	2.75E-133	5.55E-15
cg25304680	5	14229352	*TRIO*	0.655	0.711	0.716	0.056	0.061	5.49E-21	1.31E-11
cg21999071	5	14313270	*TRIO*	0.761	0.803	0.811	0.042	0.050	5.69E-22	1.14E-12
cg15612947	5	14464064	*TRIO*	0.477	0.391	0.425	-0.086	-0.051	3.95E-83	2.47E-20
cg07573020	5	14707001	*ANKH*	0.728	0.795	0.756	0.067	0.028	9.64E-135	1.49E-15
cg11661375	5	17275997	*BASP1*	0.685	0.741	0.737	0.056	0.052	4.44E-21	3.27E-08
cg20953364	5	31122837	NA	0.703	0.755	0.746	0.052	0.043	1.16E-26	4.70E-09
cg11814087	5	32391135	*ZFR*	0.534	0.420	0.490	-0.115	-0.044	4.71E-128	4.03E-12
cg26873329	5	32522933	NA	0.520	0.411	0.490	-0.109	-0.030	6.63E-121	1.84E-08
cg21798848	5	32706733	NA	0.743	0.791	0.793	0.048	0.050	5.38E-20	5.13E-10
cg14502625	5	33162283	NA	0.434	0.464	0.485	0.030	0.050	2.42E-07	1.28E-08
cg01021224	5	35049428	*PRLR*	0.729	0.776	0.779	0.047	0.050	2.18E-21	1.53E-10
cg08534016	5	35771584	*SPEF2*	0.591	0.494	0.556	-0.097	-0.035	2.00E-117	4.78E-11
cg09142144	5	35939847	*CAPSL*	0.723	0.783	0.764	0.059	0.040	4.45E-45	1.73E-10
cg14555759	5	39167171	*FYB*	0.637	0.709	0.688	0.072	0.051	2.84E-31	1.45E-08
cg07669182	5	39203755	*FYB*	0.479	0.536	0.511	0.057	0.031	5.32E-58	2.56E-10
cg00907842	5	39219245	*FYB*	0.353	0.256	0.320	-0.097	-0.033	7.87E-127	5.14E-10
cg13703437	5	39219698	*FYB*	0.435	0.331	0.394	-0.104	-0.041	2.52E-122	4.42E-12
cg21964466	5	39526937	NA	0.383	0.298	0.330	-0.085	-0.054	2.36E-112	5.26E-24
cg21489565	5	39723183	NA	0.783	0.836	0.812	0.054	0.029	1.80E-41	9.89E-07
cg13345558	5	40796738	*PRKAA1*	0.517	0.427	0.474	-0.090	-0.043	1.29E-147	1.65E-23
cg01614723	5	40908778	*C7*	0.783	0.819	0.834	0.036	0.051	6.18E-16	1.83E-13
cg16453474	5	40982092	*C7*	0.616	0.673	0.651	0.057	0.035	2.84E-33	3.47E-07
cg07749597	5	42840698	NA	0.433	0.348	0.386	-0.085	-0.047	1.24E-63	5.63E-13
cg26842080	5	42922968	NA	0.498	0.402	0.464	-0.096	-0.034	3.02E-108	2.07E-09
cg09743437	5	43000790	NA	0.410	0.349	0.368	-0.061	-0.043	1.30E-25	2.12E-07
cg14707231	5	43001138	NA	0.380	0.311	0.345	-0.069	-0.035	1.10E-46	4.98E-07
cg08866497	5	43781155	NA	0.671	0.730	0.708	0.059	0.037	2.39E-40	5.65E-09
cg10471743	5	49739158	NA	0.435	0.322	0.405	-0.113	-0.030	7.22E-139	3.20E-07
cg17934470	5	49959703	NA	0.434	0.324	0.387	-0.110	-0.048	2.07E-144	9.51E-18
cg03978682	5	53411773	*ARL15*	0.529	0.478	0.497	-0.051	-0.032	5.99E-47	1.94E-10
cg26118943	5	53816563	*SNX18*	0.339	0.265	0.309	-0.074	-0.029	6.79E-79	4.71E-08
cg11948905	5	53920730	NA	0.393	0.284	0.347	-0.109	-0.046	4.44E-140	1.41E-17
cg14597388	5	54319373	*GZMK*	0.758	0.812	0.800	0.054	0.042	2.35E-32	1.92E-09
cg07363330	5	55148493	*IL31RA*	0.451	0.359	0.411	-0.093	-0.040	2.00E-93	1.36E-11
cg05650238	5	55215955	*IL31RA*	0.358	0.291	0.323	-0.068	-0.035	1.03E-73	1.14E-10
cg18700420	5	55286352	*IL6ST*	0.728	0.790	0.783	0.062	0.056	5.47E-40	4.95E-15
cg23506143	5	55761274	NA	0.459	0.366	0.419	-0.094	-0.040	1.32E-96	8.54E-12
cg20536364	5	55790102	NA	0.727	0.672	0.693	-0.056	-0.035	2.97E-50	2.28E-11
cg00329411	5	55881858	NA	0.484	0.427	0.457	-0.057	-0.027	5.58E-43	1.90E-06
cg01233897	5	56060091	NA	0.323	0.258	0.281	-0.064	-0.041	2.48E-75	1.01E-15
cg12491659	5	57786356	*GAPT*	0.303	0.244	0.275	-0.059	-0.028	1.16E-73	8.76E-09
cg15834202	5	58865918	*PDE4D*	0.586	0.661	0.624	0.075	0.039	6.17E-59	2.85E-08
cg10487428	5	59559218	*PDE4D*	0.507	0.402	0.477	-0.104	-0.030	1.97E-156	7.79E-09
cg03723907	5	61262350	NA	0.576	0.628	0.612	0.052	0.036	1.81E-35	8.92E-09
cg26184474	5	61623163	*KIF2A*	0.480	0.381	0.451	-0.099	-0.029	1.60E-116	1.86E-08
cg21878650	5	64558623	*ADAMTS6*	0.289	0.219	0.237	-0.070	-0.052	8.83E-53	1.46E-14
cg05088356	5	66124167	*MAST4*	0.761	0.817	0.788	0.056	0.027	1.77E-79	4.41E-10
cg26096752	5	66530611	NA	0.321	0.256	0.290	-0.065	-0.031	4.26E-90	7.20E-12
cg00067720	5	67521141	*PIK3R1*	0.346	0.262	0.307	-0.084	-0.039	9.52E-92	1.00E-11
cg26646867	5	68785451	NA	0.419	0.352	0.386	-0.067	-0.033	2.19E-55	3.99E-08
cg04710768	5	71803282	*ZNF366*	0.260	0.205	0.227	-0.055	-0.033	2.30E-60	4.83E-10
cg19725489	5	72252795	*FCHO2*	0.226	0.165	0.196	-0.061	-0.030	1.34E-83	1.82E-10

cg20969424	5	73690902	NA	0.302	0.224	0.254	-0.078	-0.048	8.43E-104	5.70E-19
cg23361127	5	73939283	NA	0.477	0.382	0.437	-0.095	-0.040	1.55E-122	2.10E-16
cg15835713	5	74147080	*FAM169A*	0.275	0.225	0.245	-0.051	-0.031	8.35E-51	2.13E-09
cg03880355	5	74162880	*FAM169A*	0.365	0.295	0.331	-0.070	-0.034	1.04E-50	2.17E-06
cg04654716	5	74162924	*FAM169A*	0.521	0.466	0.485	-0.056	-0.037	1.16E-75	2.35E-17
cg22895198	5	74978020	*POC5*	0.769	0.813	0.821	0.044	0.052	1.81E-19	2.59E-12
cg11922563	5	75016955	NA	0.475	0.367	0.440	-0.108	-0.035	1.59E-122	6.77E-09
cg10586870	5	75722317	*IQGAP2*	0.408	0.345	0.376	-0.063	-0.032	7.73E-68	3.18E-08
cg08241785	5	75919281	*IQGAP2*	0.328	0.259	0.300	-0.068	-0.027	6.47E-82	1.75E-07
cg00499700	5	76116088	*F2RL1*	0.443	0.367	0.411	-0.076	-0.032	7.34E-123	9.81E-15
cg14619949	5	76116169	*F2RL1*	0.476	0.393	0.438	-0.082	-0.037	5.29E-78	7.10E-09
cg21842274	5	76248637	*CRHBP*	0.500	0.443	0.470	-0.057	-0.030	3.47E-49	4.54E-08
cg16545105	5	76248749	*CRHBP*	0.570	0.513	0.535	-0.057	-0.035	5.82E-52	1.44E-10
cg10616795	5	76464212	NA	0.764	0.704	0.716	-0.060	-0.048	1.56E-34	2.00E-14
cg12559197	5	76654783	*PDE8B*	0.403	0.323	0.372	-0.080	-0.031	3.67E-94	2.55E-09
cg03606269	5	77779546	NA	0.227	0.166	0.199	-0.061	-0.028	6.83E-63	1.46E-07
cg02375208	5	77804381	*LHFPL2*	0.329	0.257	0.289	-0.072	-0.040	3.76E-82	3.09E-14
cg08672140	5	77845045	*LHFPL2*	0.257	0.202	0.222	-0.055	-0.035	5.76E-62	1.16E-12
cg01267797	5	79542470	*SERINC5*	0.312	0.258	0.288	-0.054	-0.024	3.23E-70	7.03E-09
cg25363080	5	81452987	*ATG10*	0.344	0.269	0.293	-0.076	-0.052	1.74E-93	3.02E-21
cg06885823	5	82675005	NA	0.486	0.376	0.443	-0.110	-0.043	7.30E-153	1.11E-15
cg03613942	5	86416413	NA	0.762	0.815	0.796	0.052	0.033	6.06E-42	7.75E-08
cg14665062	5	88462828	NA	0.749	0.788	0.800	0.039	0.051	1.02E-15	9.34E-12
cg06058209	5	89736766	NA	0.687	0.737	0.739	0.050	0.052	2.29E-23	1.15E-11
cg16349093	5	90485840	NA	0.422	0.323	0.363	-0.099	-0.059	1.47E-79	4.65E-14
cg02703190	5	93137476	*FAM172A*	0.727	0.778	0.769	0.051	0.042	1.22E-31	2.48E-10
cg24786875	5	94027853	*ANKRD32*	0.504	0.412	0.473	-0.093	-0.031	3.82E-131	3.92E-11
cg21969190	5	94990682	*RFESD*	0.679	0.731	0.733	0.052	0.054	2.46E-23	4.08E-12
cg23961757	5	95122534	*RHOBTB3*	0.741	0.790	0.795	0.049	0.055	1.35E-18	1.55E-10
cg16998950	5	95252658	*ELL2*	0.447	0.332	0.410	-0.115	-0.037	1.42E-120	1.17E-09
cg25539505	5	95634103	NA	0.543	0.446	0.516	-0.097	-0.027	2.08E-107	4.15E-06
cg06465076	5	96027378	*CAST*	0.583	0.466	0.554	-0.117	-0.029	3.51E-135	8.78E-07
cg08175413	5	96294187	*LNPEP*	0.316	0.226	0.266	-0.090	-0.050	3.69E-102	4.86E-17
cg07799277	5	98368443	NA	0.402	0.315	0.364	-0.087	-0.038	3.15E-117	7.12E-14
cg15123819	5	99388688	NA	0.422	0.458	0.472	0.036	0.051	5.57E-09	7.26E-08
cg16174681	5	108220453	*FER*	0.363	0.309	0.321	-0.054	-0.042	4.75E-42	1.36E-12
cg18897025	5	110413671	*TSLP*	0.516	0.414	0.482	-0.102	-0.034	1.41E-106	4.92E-09
cg16210979	5	112882744	*YTHDC2*	0.764	0.821	0.805	0.057	0.041	9.99E-41	7.68E-10
cg21611170	5	114938389	*TMED7-TICA*	0.340	0.277	0.308	-0.062	-0.031	9.86E-108	1.34E-15
cg08675743	5	114938439	*TMED7-TICA*	0.270	0.220	0.244	-0.050	-0.026	6.15E-68	3.33E-09
cg22706992	5	115322511	*AQPEP*	0.670	0.720	0.721	0.050	0.052	4.01E-17	9.11E-09
cg00426518	5	115693708	NA	0.762	0.808	0.815	0.046	0.053	1.33E-20	1.71E-12
cg17727597	5	118159086	NA	0.692	0.757	0.727	0.065	0.035	3.16E-51	1.69E-08
cg00524900	5	118609965	*TNFAIP8*	0.347	0.275	0.318	-0.072	-0.029	3.63E-84	7.53E-08
cg01057573	5	118683890	*TNFAIP8*	0.477	0.369	0.440	-0.108	-0.038	2.74E-126	5.69E-11
cg00421693	5	118689293	*TNFAIP8*	0.731	0.783	0.763	0.052	0.032	1.69E-41	3.67E-08
cg14692284	5	118693725	*TNFAIP8*	0.721	0.781	0.758	0.060	0.037	2.98E-45	1.48E-09
cg21097090	5	118693764	*TNFAIP8*	0.668	0.725	0.704	0.057	0.037	1.86E-39	3.47E-08
cg24663638	5	118817903	*HSD17B4*	0.789	0.828	0.839	0.039	0.050	1.73E-14	4.83E-11
cg03553758	5	121456948	NA	0.355	0.287	0.314	-0.067	-0.041	2.67E-84	4.22E-18
cg27054084	5	122851781	*CSNK1G3*	0.526	0.582	0.551	0.056	0.025	2.90E-60	2.74E-08
cg15705930	5	123973317	*ZNF608*	0.728	0.782	0.769	0.054	0.040	9.43E-25	2.36E-07
cg24650940	5	123988226	*ZNF608*	0.454	0.403	0.411	-0.050	-0.042	7.05E-51	4.38E-20
cg26413942	5	124081751	*ZNF608*	0.421	0.352	0.380	-0.070	-0.042	3.56E-85	9.45E-18
cg05673752	5	126562449	NA	0.746	0.793	0.804	0.047	0.058	2.46E-16	7.46E-11
cg07854457	5	126898200	NA	0.317	0.254	0.287	-0.063	-0.030	6.39E-63	1.85E-07
cg09162993	5	127922712	NA	0.731	0.786	0.775	0.055	0.044	5.46E-33	1.05E-10
cg11010552	5	131338478	*ACSL6*	0.587	0.533	0.554	-0.054	-0.033	3.32E-52	5.35E-12
cg07870237	5	131348431	*ACSL6*	0.316	0.257	0.292	-0.059	-0.024	4.72E-60	2.26E-06
cg11293275	5	131543977	*P4HA2*	0.348	0.273	0.311	-0.075	-0.037	3.88E-122	9.75E-16
cg15375424	5	131823451	*IRF1*	0.687	0.758	0.710	0.072	0.023	1.47E-97	2.59E-08
cg23208285	5	131879579	*IL5*	0.776	0.828	0.807	0.052	0.031	1.21E-48	1.23E-08
cg19696103	5	132354130	*ZCCHC10*	0.502	0.404	0.478	-0.098	-0.024	3.60E-139	6.55E-06
cg06851151	5	133436368	NA	0.545	0.623	0.574	0.077	0.029	9.94E-183	1.70E-23
cg20682563	5	133460874	*TCF7*	0.624	0.694	0.654	0.070	0.030	6.11E-124	1.21E-13
cg09101941	5	133775258	NA	0.381	0.285	0.329	-0.096	-0.052	2.30E-113	6.22E-18
cg16141752	5	133802474	NA	0.315	0.246	0.290	-0.069	-0.025	3.51E-88	4.52E-07
cg10785340	5	134715577	*H2AFY*	0.329	0.246	0.299	-0.082	-0.030	1.24E-109	8.45E-09
cg01550148	5	134735544	*H2AFY*	0.314	0.253	0.291	-0.060	-0.023	1.07E-72	1.13E-06
cg16246489	5	134735675	*H2AFY*	0.412	0.312	0.360	-0.100	-0.052	2.88E-77	4.02E-12
cg01610123	5	134770100	NA	0.371	0.300	0.339	-0.072	-0.033	5.63E-81	1.07E-09
cg16219583	5	135702333	*TRPC7*	0.708	0.760	0.753	0.051	0.044	2.26E-27	5.06E-10
cg09530650	5	137024163	*KLHL3*	0.627	0.559	0.602	-0.068	-0.025	3.36E-83	5.87E-08
cg13023677	5	137675418	*FAM53C*	0.270	0.200	0.243	-0.070	-0.027	4.29E-97	1.49E-07
cg24854175	5	138126560	*CTNNA1*	0.764	0.816	0.789	0.053	0.025	4.92E-49	1.33E-06
cg04447756	5	138644986	*MATR3*	0.723	0.777	0.760	0.054	0.037	6.82E-38	3.09E-09
cg18446110	5	138714654	*SLC23A1*	0.446	0.340	0.406	-0.106	-0.040	2.33E-144	1.34E-13
cg20566766	5	138723816	*MZB1*	0.391	0.338	0.368	-0.053	-0.023	2.21E-102	1.92E-12

cg01938023	5	138855699	*TMEM173*	0.465	0.395	0.427	-0.070	-0.038	7.87E-100	2.50E-16
cg01408486	5	139027024	*CXXC5*	0.288	0.232	0.261	-0.056	-0.027	1.73E-89	6.83E-15
cg04416898	5	139040055	*CXXC5*	0.336	0.271	0.290	-0.066	-0.047	2.94E-87	5.97E-23
cg18814344	5	139040546	*CXXC5*	0.396	0.330	0.367	-0.066	-0.029	6.05E-97	9.87E-13
cg20455854	5	139040849	*CXXC5*	0.437	0.383	0.410	-0.054	-0.027	2.27E-49	4.29E-09
cg21230503	5	139085173	NA	0.377	0.325	0.345	-0.052	-0.033	3.50E-46	1.21E-09
cg00450651	5	139485902	NA	0.296	0.228	0.245	-0.068	-0.051	1.31E-91	1.12E-25
cg23098195	5	141770115	NA	0.289	0.234	0.256	-0.055	-0.033	2.40E-97	8.65E-20
cg27579771	5	142431272	*ARHGAP26*	0.433	0.348	0.387	-0.085	-0.046	1.53E-84	1.70E-14
cg01288724	5	142575122	*ARHGAP26*	0.432	0.335	0.395	-0.097	-0.037	4.68E-104	7.48E-09
cg18805457	5	145829759	*TCERG1*	0.459	0.398	0.423	-0.062	-0.036	1.14E-48	9.11E-11
cg00106565	5	146784171	*DPYSL3*	0.707	0.760	0.747	0.054	0.040	8.02E-30	1.42E-08
cg06676778	5	148209341	NA	0.597	0.663	0.628	0.066	0.031	1.44E-56	6.38E-07
cg07598021	5	148728200	*GRPEL2*	0.630	0.681	0.660	0.051	0.030	1.39E-42	1.75E-07
cg16684117	5	148808456	*MIR143HG*	0.326	0.248	0.300	-0.078	-0.026	1.11E-150	2.64E-12
cg21242144	5	148808487	*MIR143HG*	0.318	0.252	0.290	-0.065	-0.028	1.77E-124	3.54E-15
cg09109411	5	149319112	*PDE6A*	0.473	0.372	0.427	-0.101	-0.046	1.23E-155	1.33E-22
cg17781669	5	149868359	NA	0.351	0.287	0.318	-0.064	-0.034	2.26E-54	5.15E-09
cg07038243	5	149868855	NA	0.343	0.285	0.324	-0.058	-0.019	4.65E-80	5.83E-06
cg27044455	5	149878183	NA	0.462	0.377	0.429	-0.086	-0.033	1.12E-115	1.90E-11
cg06677890	5	149887486	*NDST1*	0.268	0.216	0.241	-0.052	-0.027	2.68E-45	2.00E-06
cg16674484	5	149887497	*NDST1*	0.372	0.297	0.338	-0.075	-0.034	1.83E-65	9.46E-08
cg15167202	5	149997153	*SYNPO*	0.523	0.434	0.488	-0.089	-0.035	1.48E-112	4.42E-12
cg06654628	5	150018914	*SYNPO*	0.323	0.264	0.296	-0.059	-0.027	3.19E-76	4.01E-11
cg05876069	5	150020288	*SYNPO*	0.426	0.375	0.407	-0.050	-0.019	1.20E-86	1.64E-09
cg16646054	5	150157726	*C5orf62*	0.535	0.449	0.478	-0.086	-0.058	1.26E-76	2.76E-19
cg04159302	5	150162068	*C5orf62*	0.494	0.425	0.448	-0.069	-0.046	1.03E-57	1.29E-12
cg12684668	5	150403466	*GPX3*	0.530	0.444	0.496	-0.086	-0.034	7.36E-110	9.26E-11
cg11125805	5	150678162	*SLC36A3*	0.416	0.309	0.374	-0.107	-0.041	2.88E-121	1.92E-12
cg26389330	5	151057860	*SPARC*	0.351	0.278	0.327	-0.073	-0.023	1.67E-83	4.67E-06
cg13579752	5	153577937	*GALNT10*	0.663	0.719	0.689	0.056	0.025	2.88E-82	1.84E-10
cg26655340	5	154230141	*C5orf4*	0.421	0.341	0.384	-0.080	-0.037	1.97E-124	2.45E-18
cg10257049	5	154230308	*C5orf4*	0.390	0.310	0.358	-0.081	-0.032	3.23E-120	1.52E-12
cg08445740	5	156594400	*FAM71B*	0.699	0.760	0.747	0.061	0.049	3.96E-56	2.85E-18
cg00510689	5	156594470	*FAM71B*	0.722	0.783	0.771	0.061	0.049	6.76E-47	7.25E-15
cg17547295	5	157282503	*CLINT1*	0.520	0.429	0.478	-0.091	-0.042	1.31E-95	8.79E-12
cg01829672	5	157282617	*CLINT1*	0.307	0.244	0.275	-0.063	-0.032	8.19E-72	1.33E-09
cg08305575	5	158018481	NA	0.438	0.348	0.393	-0.090	-0.046	7.34E-110	1.08E-15
cg21211213	5	158127763	*EBF1*	0.289	0.223	0.248	-0.066	-0.040	7.89E-118	3.26E-24
cg06030535	5	158637684	*RNF145*	0.401	0.301	0.365	-0.100	-0.036	6.25E-150	2.71E-13
cg08688907	5	159890925	NA	0.641	0.581	0.601	-0.059	-0.039	7.83E-65	1.03E-15
cg04625905	5	160280216	*ATP10B*	0.703	0.753	0.752	0.050	0.048	5.10E-24	2.28E-10
cg07015465	5	167912659	*RARS*	0.703	0.751	0.760	0.048	0.057	1.81E-18	8.60E-12
cg04730794	5	169144438	*DOCK2*	0.550	0.414	0.498	-0.136	-0.052	2.27E-200	7.06E-25
cg22794304	5	169734762	NA	0.344	0.276	0.315	-0.068	-0.030	1.63E-99	8.51E-13
cg17969271	5	171430188	*FBXW11*	0.368	0.311	0.339	-0.056	-0.029	2.09E-44	1.25E-06
cg23367341	5	171504064	*STK10*	0.600	0.526	0.558	-0.074	-0.041	1.39E-39	1.05E-07
cg02376843	5	172083710	*NEURL1B*	0.575	0.614	0.628	0.038	0.053	2.06E-13	1.98E-11
cg07496207	5	172280684	*ERGIC1*	0.484	0.381	0.446	-0.102	-0.038	1.57E-136	1.67E-11
cg20977312	5	172748917	*STC2*	0.569	0.459	0.539	-0.109	-0.030	4.11E-140	3.66E-08
cg14746387	5	172879299	NA	0.418	0.323	0.383	-0.095	-0.035	4.78E-115	1.30E-10
cg20954870	5	173070254	NA	0.529	0.426	0.485	-0.102	-0.044	3.14E-102	5.94E-12
cg02640809	5	173213710	NA	0.229	0.179	0.188	-0.050	-0.041	1.45E-63	4.72E-20
cg25213720	5	176734343	*MXD3*	0.351	0.266	0.307	-0.085	-0.043	7.78E-99	2.04E-16
cg15225325	5	176921820	*PDLIM7*	0.391	0.324	0.357	-0.067	-0.034	1.49E-66	5.72E-10
cg07608052	5	176937527	*DOK3*	0.437	0.372	0.414	-0.064	-0.023	1.29E-93	1.55E-08
cg19619956	5	176967557	*FAM193B*	0.539	0.442	0.508	-0.096	-0.031	1.47E-146	7.04E-12
cg22051146	5	177895047	*COL23A1*	0.359	0.285	0.329	-0.074	-0.030	1.49E-75	5.54E-07
cg05206633	5	177913434	*COL23A1*	0.605	0.491	0.571	-0.114	-0.034	6.69E-81	4.36E-07
cg01561304	5	177913485	*COL23A1*	0.229	0.175	0.204	-0.054	-0.026	6.43E-62	1.35E-07
cg04569651	5	177956199	*COL23A1*	0.416	0.337	0.382	-0.079	-0.034	3.10E-156	1.14E-22
cg17288892	5	178956874	NA	0.303	0.245	0.272	-0.058	-0.031	2.53E-60	9.49E-10
cg13995774	5	179189810	*MAML1*	0.438	0.327	0.394	-0.111	-0.044	1.54E-146	3.78E-19
cg02596779	5	179496893	*RNF130*	0.211	0.159	0.192	-0.052	-0.019	9.66E-85	4.46E-08
cg25154482	5	179497098	*RNF130*	0.306	0.241	0.276	-0.065	-0.030	1.31E-70	1.33E-07
cg17554875	5	179520998	NA	0.300	0.243	0.279	-0.057	-0.021	1.48E-75	3.37E-06
cg02214623	5	180671896	*GNB2L1*	0.512	0.454	0.477	-0.057	-0.035	1.23E-37	1.82E-07
cg15253304	6	209809	NA	0.693	0.765	0.717	0.073	0.024	3.89E-102	4.50E-08
cg19295314	6	1635640	*GMDS*	0.469	0.409	0.446	-0.060	-0.023	7.71E-78	2.53E-07
cg09571972	6	2104322	*GMDS*	0.372	0.280	0.333	-0.092	-0.039	1.00E-129	2.73E-14
cg27215185	6	2197772	*GMDS*	0.665	0.612	0.641	-0.053	-0.024	1.67E-60	1.32E-07
cg12586150	6	2840792	*SERPINB1*	0.330	0.248	0.297	-0.082	-0.033	5.19E-108	8.91E-10
cg20726195	6	2892148	*SERPINB9*	0.588	0.662	0.623	0.074	0.034	1.46E-60	2.88E-07
cg15068318	6	2958750	*SERPINB6*	0.359	0.297	0.341	-0.062	-0.019	9.14E-93	5.85E-06
cg01137047	6	2970877	*SERPINB6*	0.416	0.359	0.369	-0.057	-0.047	3.49E-68	3.91E-23
cg09740468	6	3025325	NA	0.460	0.370	0.427	-0.090	-0.033	9.32E-121	3.13E-11
cg16153042	6	3282307	*SLC22A23*	0.571	0.518	0.544	-0.053	-0.027	1.68E-55	1.45E-08
cg04327675	6	3342030	*SLC22A23*	0.702	0.755	0.751	0.053	0.048	2.72E-20	1.04E-08

cg13413719	6	3592887	NA	0.342	0.254	0.282	-0.088	-0.060	1.17E-104	8.32E-25
cg25609749	6	3743352	*PXDC1*	0.323	0.277	0.273	-0.047	-0.050	1.85E-16	8.76E-09
cg19772161	6	3746570	*PXDC1*	0.318	0.266	0.298	-0.052	-0.020	4.49E-98	4.31E-09
cg14913512	6	4772890	*CDYL*	0.712	0.765	0.754	0.053	0.042	3.61E-26	6.06E-08
cg23610213	6	4802019	*CDYL*	0.732	0.786	0.762	0.054	0.030	1.96E-61	6.62E-10
cg03187073	6	4890079	*CDYL*	0.404	0.304	0.358	-0.100	-0.045	2.28E-119	8.72E-15
cg27307183	6	4890095	*CDYL*	0.418	0.314	0.371	-0.104	-0.047	1.31E-151	1.80E-19
cg07599389	6	4890278	*CDYL*	0.377	0.289	0.339	-0.088	-0.039	5.82E-155	2.22E-23
cg16393899	6	5136301	*LYRM4*	0.495	0.393	0.458	-0.102	-0.037	1.79E-106	1.16E-08
cg26704043	6	5282702	*FARS2*	0.407	0.343	0.386	-0.064	-0.021	3.94E-79	1.25E-06
cg00418528	6	5463751	*FARS2*	0.738	0.793	0.791	0.055	0.053	4.32E-24	1.79E-10
cg17611046	6	5672954	*FARS2*	0.510	0.443	0.473	-0.067	-0.037	1.25E-55	5.68E-10
cg02212836	6	6589075	*LY86-AS1*	0.575	0.647	0.601	0.073	0.026	8.14E-118	4.27E-12
cg07970799	6	6614719	*LY86-AS1*	0.740	0.802	0.765	0.062	0.025	3.03E-104	3.20E-10
cg17646250	6	6759768	NA	0.338	0.283	0.313	-0.056	-0.026	4.05E-80	1.68E-10
cg22626169	6	6890951	NA	0.507	0.407	0.457	-0.101	-0.050	1.04E-122	1.17E-19
cg16312968	6	6900945	NA	0.327	0.252	0.294	-0.075	-0.033	3.28E-92	1.85E-10
cg19228647	6	6901002	NA	0.276	0.220	0.249	-0.056	-0.027	4.15E-85	8.28E-13
cg10287970	6	7115345	*RREB1*	0.428	0.329	0.380	-0.099	-0.048	4.40E-141	2.43E-21
cg20893838	6	7145478	*RREB1*	0.384	0.306	0.357	-0.078	-0.027	1.40E-121	1.21E-09
cg14919455	6	7167468	*RREB1*	0.458	0.357	0.427	-0.101	-0.031	1.84E-177	2.31E-14
cg07714276	6	7169632	*RREB1*	0.370	0.279	0.329	-0.091	-0.041	1.52E-199	1.29E-32
cg09326832	6	7243643	*RREB1*	0.307	0.251	0.281	-0.056	-0.026	5.44E-54	2.43E-06
cg10471548	6	7403054	*RIOK1*	0.456	0.358	0.426	-0.098	-0.030	1.10E-117	5.83E-08
cg17951878	6	7698374	NA	0.298	0.232	0.258	-0.065	-0.040	3.39E-72	1.00E-12
cg12656896	6	8438818	*LOC1005062*	0.374	0.285	0.341	-0.089	-0.033	7.74E-112	2.58E-09
cg14112601	6	10520874	*GCNT2*	0.280	0.217	0.246	-0.063	-0.034	1.11E-105	1.91E-15
cg24690479	6	10521142	*GCNT2*	0.348	0.284	0.313	-0.064	-0.035	1.92E-71	1.98E-12
cg22378252	6	10521452	*GCNT2*	0.266	0.203	0.233	-0.063	-0.032	4.13E-86	5.80E-13
cg07186962	6	11393478	NA	0.367	0.281	0.333	-0.086	-0.034	1.28E-103	1.89E-10
cg10787197	6	11779941	*ADTRP*	0.547	0.486	0.503	-0.061	-0.044	3.64E-50	9.79E-14
cg19268652	6	12119552	*HIVEP1*	0.626	0.693	0.660	0.067	0.034	2.24E-38	4.71E-07
cg14622879	6	12234001	NA	0.213	0.160	0.190	-0.053	-0.023	4.91E-61	5.06E-06
cg26514623	6	13295561	*LOC1001303*	0.565	0.448	0.529	-0.117	-0.036	2.06E-152	5.06E-12
cg01702055	6	13303065	NA	0.285	0.230	0.247	-0.055	-0.038	3.40E-53	2.70E-12
cg26942829	6	13408158	*GFOD1*	0.503	0.387	0.464	-0.116	-0.039	1.62E-133	9.15E-12
cg08622666	6	13695743	*RANBP9*	0.712	0.785	0.749	0.073	0.037	3.96E-84	1.36E-11
cg25025545	6	14136628	*CD83*	0.424	0.321	0.384	-0.103	-0.040	7.02E-153	5.44E-15
cg03446062	6	14477353	NA	0.479	0.389	0.436	-0.091	-0.043	2.00E-106	1.01E-14
cg22164009	6	14729030	NA	0.397	0.303	0.351	-0.094	-0.045	1.28E-145	4.56E-24
cg24219974	6	14729722	NA	0.746	0.800	0.773	0.054	0.026	7.09E-76	3.83E-13
cg26076724	6	15090163	NA	0.423	0.334	0.392	-0.089	-0.031	2.63E-122	2.04E-10
cg14662750	6	15280024	*JARID2*	0.435	0.361	0.389	-0.073	-0.046	1.15E-86	6.94E-20
cg20487608	6	15288126	*JARID2*	0.349	0.262	0.311	-0.087	-0.038	7.35E-99	1.22E-10
cg16362595	6	15305678	*JARID2*	0.641	0.698	0.672	0.057	0.031	1.95E-51	1.24E-08
cg00412851	6	15463343	*JARID2*	0.693	0.749	0.747	0.056	0.054	1.24E-24	1.53E-11
cg11938455	6	15622032	*DTNBP1*	0.231	0.177	0.205	-0.054	-0.027	5.18E-83	2.16E-11
cg03717755	6	16136539	*MYLIP*	0.621	0.688	0.659	0.067	0.039	4.46E-69	1.97E-11
cg17014647	6	16431306	*ATXN1*	0.689	0.743	0.733	0.054	0.044	5.71E-21	8.62E-07
cg05968188	6	16483997	*ATXN1*	0.748	0.807	0.768	0.059	0.020	1.27E-102	5.27E-08
cg24067911	6	16729610	*ATXN1*	0.363	0.306	0.328	-0.057	-0.035	2.01E-49	1.03E-09
cg00278392	6	16802505	NA	0.439	0.324	0.400	-0.115	-0.039	7.99E-161	3.51E-13
cg18493214	6	17764205	*KIF13A*	0.385	0.298	0.341	-0.087	-0.044	3.73E-95	1.57E-12
cg00069309	6	17931997	*KIF13A*	0.789	0.825	0.841	0.036	0.052	6.10E-16	4.65E-15
cg15414930	6	17985564	*KIF13A*	0.659	0.609	0.608	-0.050	-0.051	5.50E-33	5.51E-18
cg21119074	6	20211654	*MBOAT1*	0.265	0.212	0.221	-0.053	-0.044	1.09E-52	1.19E-15
cg08628635	6	20483859	*E2F3*	0.482	0.354	0.428	-0.129	-0.055	8.69E-208	1.77E-28
cg20488123	6	20689061	*CDKAL1*	0.643	0.713	0.671	0.070	0.028	2.50E-88	4.94E-09
cg06512263	6	20709867	*CDKAL1*	0.283	0.218	0.257	-0.065	-0.026	2.01E-86	3.27E-08
cg21498062	6	21157865	*CDKAL1*	0.751	0.814	0.802	0.063	0.051	8.80E-48	3.21E-15
cg06972043	6	21348636	NA	0.706	0.770	0.744	0.064	0.038	4.46E-49	8.47E-09
cg02147208	6	21662648	NA	0.678	0.734	0.719	0.056	0.041	3.58E-30	2.29E-08
cg15943139	6	22061708	*LINC00340*	0.724	0.776	0.753	0.052	0.029	3.07E-33	4.87E-06
cg02832512	6	22113516	*LINC00340*	0.521	0.446	0.485	-0.074	-0.036	1.26E-86	3.52E-13
cg00895196	6	22147182	*LINC00340*	0.789	0.845	0.822	0.056	0.032	3.07E-57	1.61E-13
cg07914200	6	22699463	NA	0.563	0.509	0.536	-0.055	-0.028	1.32E-40	5.66E-06
cg14628914	6	24659344	*TDP2*	0.539	0.448	0.508	-0.090	-0.031	2.32E-119	1.30E-10
cg16544737	6	24925385	NA	0.650	0.710	0.698	0.060	0.049	6.99E-51	4.13E-16
cg25050392	6	24933103	NA	0.690	0.769	0.741	0.079	0.051	8.30E-75	3.29E-16
cg10699171	6	24936965	NA	0.620	0.703	0.665	0.082	0.045	9.20E-102	7.16E-16
cg12449049	6	25088999	*CMAHP*	0.663	0.603	0.616	-0.061	-0.047	2.37E-49	2.41E-15
cg17313269	6	25965330	*TRIM38*	0.488	0.374	0.455	-0.114	-0.033	7.88E-141	4.21E-09
cg07816556	6	26017280	*HIST1H1A*	0.698	0.749	0.746	0.051	0.048	1.02E-27	3.79E-11
cg13015616	6	26086585	*HFE*	0.747	0.800	0.772	0.053	0.025	1.16E-99	6.58E-13
cg03926598	6	26617378	NA	0.752	0.796	0.802	0.045	0.050	7.38E-18	4.17E-11
cg05657416	6	27105792	*HIST1H4I*	0.739	0.781	0.792	0.043	0.054	1.82E-16	4.03E-12
cg22790005	6	27657814	NA	0.779	0.828	0.834	0.050	0.055	1.50E-21	1.49E-12
cg15407162	6	28192457	*ZNF193*	0.382	0.296	0.330	-0.086	-0.052	3.17E-90	2.57E-18

cg07790947	6	28474513	*GPX6*	0.603	0.665	0.655	0.062	0.052	3.11E-44	4.77E-14
cg14268557	6	28874547	*TRIM27*	0.485	0.372	0.452	-0.112	-0.033	2.83E-116	1.47E-06
cg05314350	6	28874702	*TRIM27*	0.485	0.381	0.444	-0.104	-0.041	2.15E-136	1.37E-14
cg11389756	6	28875354	*TRIM27*	0.423	0.317	0.387	-0.107	-0.037	3.15E-138	5.14E-13
cg08279189	6	28875370	*TRIM27*	0.495	0.389	0.463	-0.107	-0.033	1.89E-89	1.47E-06
cg10718056	6	28884599	*TRIM27*	0.434	0.321	0.380	-0.113	-0.054	1.34E-203	3.17E-34
cg17344091	6	28885444	*TRIM27*	0.366	0.261	0.324	-0.105	-0.042	5.31E-134	7.73E-14
cg00259404	6	28885568	*TRIM27*	0.374	0.274	0.341	-0.100	-0.033	3.16E-129	5.31E-09
cg20986887	6	28887284	*TRIM27*	0.353	0.273	0.323	-0.080	-0.030	2.85E-80	4.16E-07
cg04656831	6	29282808	NA	0.683	0.736	0.717	0.053	0.034	1.30E-36	2.52E-08
cg12646029	6	29427451	*OR2H1*	0.698	0.752	0.728	0.054	0.030	1.64E-48	5.18E-08
cg13638427	6	29617320	NA	0.274	0.210	0.244	-0.064	-0.030	6.37E-73	3.78E-09
cg10665848	6	29631447	*MOG*	0.761	0.805	0.815	0.044	0.054	5.05E-17	2.72E-11
cg12588917	6	29692082	*HLA-F*	0.440	0.403	0.390	-0.037	-0.051	8.13E-13	1.09E-11
cg15331332	6	29692111	*HLA-F*	0.580	0.557	0.530	-0.023	-0.050	4.29E-06	3.36E-13
cg20408505	6	29911494	*HLA-A*	0.465	0.436	0.412	-0.029	-0.053	4.50E-09	4.29E-14
cg17608381	6	29911550	*HLA-A*	0.599	0.487	0.482	-0.112	-0.117	4.77E-25	3.36E-15
cg01044849	6	30002723	*ZNRD1-AS1*	0.572	0.473	0.547	-0.098	-0.025	4.21E-148	3.26E-08
cg13831136	6	30070342	NA	0.428	0.336	0.395	-0.093	-0.033	2.93E-123	7.16E-10
cg00578614	6	30070403	NA	0.434	0.333	0.394	-0.101	-0.040	9.03E-127	1.74E-13
cg10009801	6	30168645	*TRIM26*	0.382	0.300	0.340	-0.081	-0.042	9.04E-94	4.34E-14
cg08850243	6	30169768	*TRIM26*	0.365	0.262	0.325	-0.102	-0.040	6.34E-166	8.52E-20
cg14799927	6	30175074	*TRIM26*	0.525	0.450	0.495	-0.075	-0.030	8.83E-61	1.01E-06
cg26193268	6	30290443	*HCG18*	0.677	0.746	0.704	0.069	0.027	1.48E-68	2.26E-06
cg13079571	6	30297257	*TRIM39*	0.548	0.447	0.522	-0.101	-0.026	1.16E-111	5.63E-06
cg09020199	6	30297320	*TRIM39*	0.516	0.422	0.483	-0.093	-0.032	1.09E-124	1.53E-11
cg11327408	6	30297329	*TRIM39*	0.535	0.433	0.506	-0.102	-0.029	5.00E-135	2.47E-08
cg04425551	6	30297338	*TRIM39*	0.532	0.435	0.504	-0.097	-0.028	6.95E-138	3.44E-09
cg07905808	6	30297389	*TRIM39*	0.585	0.505	0.563	-0.080	-0.022	1.60E-110	1.68E-06
cg21426759	6	30303126	*TRIM39*	0.581	0.469	0.538	-0.112	-0.043	1.32E-128	4.71E-13
cg11594821	6	30458601	*HLA-E*	0.377	0.357	0.326	-0.020	-0.051	4.53E-06	8.44E-14
cg17615629	6	30459867	*HLA-E*	0.657	0.714	0.681	0.058	0.025	8.80E-52	5.25E-06
cg02678305	6	30460322	*HLA-E*	0.699	0.753	0.719	0.055	0.020	1.18E-102	3.62E-10
cg09569347	6	30460548	*HLA-E*	0.583	0.649	0.609	0.066	0.026	3.69E-119	1.16E-14
cg15729062	6	30579288	*PPP1R10*	0.764	0.810	0.817	0.045	0.053	6.33E-18	2.47E-10
cg11748260	6	30624395	*DHX16*	0.392	0.320	0.362	-0.072	-0.030	3.28E-83	8.57E-08
cg01999566	6	30624449	*DHX16*	0.322	0.256	0.291	-0.065	-0.031	1.22E-120	1.18E-16
cg09379497	6	30624467	*DHX16*	0.524	0.435	0.489	-0.089	-0.035	8.98E-119	6.24E-11
cg01211396	6	30624478	*DHX16*	0.548	0.475	0.523	-0.073	-0.025	1.11E-100	9.29E-08
cg21513254	6	30624502	*DHX16*	0.418	0.343	0.396	-0.076	-0.022	1.72E-96	5.49E-06
cg23672659	6	30648020	*PPP1R18*	0.629	0.703	0.654	0.074	0.026	4.01E-147	6.21E-14
cg11554650	6	30653191	*PPP1R18*	0.619	0.678	0.637	0.059	0.018	2.68E-106	8.62E-08
cg06126421	6	30720080	NA	0.785	0.727	0.751	-0.058	-0.034	2.87E-26	7.33E-07
cg14753356	6	30720108	NA	0.484	0.397	0.423	-0.087	-0.060	1.29E-72	4.69E-18
cg19913563	6	30720261	NA	0.347	0.252	0.301	-0.094	-0.045	6.36E-132	9.18E-18
cg03720100	6	30720263	NA	0.349	0.263	0.306	-0.085	-0.043	1.20E-110	4.44E-16
cg06422467	6	30720484	NA	0.412	0.321	0.360	-0.091	-0.052	2.19E-137	1.50E-25
cg16137928	6	30720491	NA	0.449	0.357	0.404	-0.092	-0.045	9.71E-146	5.66E-22
cg27154651	6	30796209	NA	0.642	0.571	0.582	-0.071	-0.060	5.42E-23	1.44E-08
cg22028727	6	30796243	NA	0.400	0.332	0.356	-0.068	-0.045	1.94E-72	5.32E-17
cg08469255	6	30851069	*DDR1*	0.698	0.748	0.726	0.051	0.029	1.50E-70	4.89E-13
cg11975790	6	30853608	*DDR1*	0.638	0.706	0.688	0.067	0.049	1.04E-40	1.40E-10
cg27057509	6	30883762	*VARS2*	0.585	0.518	0.540	-0.067	-0.045	3.60E-41	1.24E-10
cg06879746	6	30883768	*VARS2*	0.664	0.598	0.622	-0.066	-0.042	7.67E-48	3.35E-11
cg08827454	6	30922981	NA	0.342	0.269	0.318	-0.073	-0.024	5.59E-97	1.69E-06
cg02755555	6	30981960	*MUC22*	0.713	0.764	0.770	0.051	0.057	8.24E-17	6.00E-10
cg13031097	6	31322577	*HLA-B*	0.389	0.340	0.327	-0.049	-0.062	4.92E-15	8.87E-12
cg23923934	6	31322914	*HLA-B*	0.730	0.692	0.680	-0.038	-0.050	1.92E-11	3.34E-11
cg11187245	6	31323397	*HLA-B*	0.724	0.694	0.664	-0.030	-0.060	5.42E-09	9.69E-19
cg06480496	6	31430675	NA	0.312	0.278	0.258	-0.034	-0.054	8.66E-09	3.03E-09
cg18808777	6	31431503	*HCP5*	0.292	0.203	0.229	-0.089	-0.063	1.59E-42	7.89E-10
cg01082299	6	31431969	*HCP5*	0.611	0.573	0.558	-0.038	-0.053	4.84E-12	3.66E-12
cg22318806	6	31540411	*LTA*	0.549	0.632	0.579	0.083	0.030	3.81E-139	1.58E-12
cg13815684	6	31540440	*LTA*	0.719	0.771	0.737	0.052	0.018	7.79E-94	5.32E-08
cg17709873	6	31540456	*LTA*	0.668	0.750	0.708	0.082	0.040	8.97E-84	2.44E-11
cg16280132	6	31540459	*LTA*	0.525	0.613	0.558	0.089	0.034	4.79E-106	2.65E-11
cg26348243	6	31540461	*LTA*	0.525	0.642	0.573	0.116	0.047	2.77E-117	6.27E-14
cg00501919	6	31540750	*LTA*	0.578	0.649	0.599	0.071	0.021	3.78E-111	2.67E-07
cg01360627	6	31544931	*TNF*	0.675	0.762	0.699	0.087	0.024	9.85E-112	1.91E-06
cg09637172	6	31545252	*TNF*	0.776	0.840	0.799	0.064	0.023	3.89E-85	3.85E-07
cg02249390	6	31550612	*LTB*	0.687	0.757	0.713	0.070	0.026	1.56E-124	4.83E-13
cg27616007	6	31554829	*LST1*	0.252	0.187	0.215	-0.065	-0.037	1.19E-96	5.64E-17
cg14324675	6	31554848	*LST1*	0.343	0.267	0.307	-0.076	-0.036	3.77E-100	9.29E-14
cg03739609	6	31555016	*LST1*	0.461	0.352	0.406	-0.108	-0.055	8.24E-171	5.88E-29
cg24211388	6	31582837	*AIF1*	0.435	0.331	0.393	-0.104	-0.042	5.34E-136	6.55E-16
cg00774102	6	31590870	*PRRC2A*	0.419	0.333	0.382	-0.086	-0.038	3.00E-156	1.30E-21
cg15809217	6	31607648	*BAG6*	0.386	0.314	0.344	-0.072	-0.042	7.18E-49	2.45E-08
cg22708150	6	31649619	*LY6G5C*	0.562	0.510	0.528	-0.052	-0.034	3.41E-25	5.56E-06

cg10914115	6	31704828	*CLIC1*	0.300	0.247	0.273	-0.053	-0.027	1.51E-87	4.43E-13
cg21769117	6	31705273	*CLIC1*	0.208	0.155	0.186	-0.053	-0.022	1.28E-84	2.21E-08
cg20640261	6	31707019	*MSH5*	0.532	0.482	0.487	-0.051	-0.045	3.96E-32	8.77E-13
cg16656520	6	31800885	NA	0.371	0.293	0.342	-0.077	-0.029	3.24E-81	6.13E-07
cg05569328	6	32044384	*TNXB*	0.247	0.193	0.219	-0.054	-0.028	4.84E-69	4.46E-09
cg05133205	6	32121249	*PPT2*	0.513	0.443	0.472	-0.070	-0.041	2.59E-33	1.75E-06
cg17329164	6	32121259	*PPT2*	0.346	0.285	0.312	-0.061	-0.034	6.87E-43	1.52E-06
cg13102294	6	32121393	*PPT2*	0.425	0.369	0.391	-0.056	-0.034	2.77E-38	4.82E-08
cg06264679	6	32121433	*PPT2*	0.439	0.374	0.398	-0.066	-0.042	1.82E-46	1.39E-09
cg12401918	6	32164723	*NOTCH4*	0.295	0.230	0.261	-0.065	-0.035	1.10E-69	6.19E-10
cg13185046	6	32165321	*NOTCH4*	0.371	0.308	0.334	-0.063	-0.037	6.69E-89	1.28E-15
cg20241876	6	32180045	*NOTCH4*	0.393	0.316	0.368	-0.077	-0.025	2.49E-179	7.93E-16
cg16602097	6	32182013	*NOTCH4*	0.378	0.312	0.338	-0.066	-0.039	5.90E-83	3.69E-17
cg10818896	6	32263335	*C6orf10*	0.679	0.733	0.733	0.054	0.054	8.69E-19	5.36E-09
cg00874877	6	32272050	*C6orf10*	0.753	0.811	0.801	0.058	0.048	1.67E-42	1.44E-13
cg26166145	6	32340208	*C6orf10*	0.728	0.773	0.788	0.045	0.060	1.22E-13	3.04E-11
cg07180897	6	32729130	*HLA-DQB2*	0.811	0.739	0.727	-0.072	-0.085	2.66E-10	8.87E-07
cg23090653	6	32765352	NA	0.247	0.189	0.221	-0.058	-0.026	8.99E-84	1.03E-08
cg03639929	6	32765402	NA	0.281	0.200	0.250	-0.081	-0.031	5.42E-95	7.70E-08
cg08998192	6	32805570	*TAP2*	0.477	0.431	0.421	-0.047	-0.056	4.06E-23	5.34E-17
cg08099136	6	32811251	*PSMB8*	0.398	0.337	0.328	-0.061	-0.070	1.19E-32	9.96E-21
cg01309328	6	32811253	*PSMB8*	0.414	0.361	0.354	-0.053	-0.059	5.04E-26	3.03E-17
cg08818207	6	32820355	*TAP1*	0.506	0.454	0.446	-0.052	-0.060	6.31E-19	6.34E-14
cg07156249	6	32822911	*PSMB9*	0.332	0.269	0.238	-0.063	-0.094	1.48E-14	1.22E-13
cg23581252	6	32897912	NA	0.475	0.384	0.443	-0.091	-0.032	4.68E-129	8.92E-12
cg10411221	6	32904317	*HLA-DMB*	0.429	0.350	0.404	-0.078	-0.024	4.28E-150	7.34E-11
cg19268453	6	32905114	*HLA-DMB*	0.495	0.409	0.462	-0.086	-0.032	6.52E-111	2.04E-11
cg01374870	6	32905127	*HLA-DMB*	0.437	0.328	0.390	-0.110	-0.048	6.58E-162	3.15E-20
cg04903089	6	32905190	*HLA-DMB*	0.351	0.268	0.313	-0.083	-0.038	1.10E-133	1.11E-17
cg10453850	6	32905320	*HLA-DMB*	0.376	0.283	0.333	-0.093	-0.042	1.34E-120	2.07E-15
cg21578596	6	32906460	*HLA-DMB*	0.364	0.271	0.321	-0.093	-0.043	2.42E-156	1.02E-21
cg09146459	6	32921200	*HLA-DMA*	0.335	0.250	0.301	-0.085	-0.034	2.56E-109	3.73E-12
cg18888137	6	32945759	*BRD2*	0.434	0.329	0.391	-0.105	-0.043	7.95E-132	6.64E-15
cg13906813	6	33040914	*HLA-DPA1*	0.289	0.261	0.238	-0.029	-0.051	1.82E-09	7.80E-12
cg15818109	6	33142006	*COL11A2*	0.493	0.440	0.469	-0.053	-0.024	7.10E-49	1.04E-06
cg05454562	6	33254447	*WDR46*	0.511	0.409	0.478	-0.103	-0.033	5.16E-154	2.42E-12
cg27055685	6	33258248	*WDR46*	0.282	0.221	0.259	-0.061	-0.023	4.82E-81	6.96E-07
cg26819753	6	33265313	*RGL2*	0.337	0.286	0.312	-0.050	-0.024	2.32E-76	9.99E-11
cg01471293	6	33265322	*RGL2*	0.267	0.205	0.240	-0.062	-0.028	3.23E-63	1.68E-06
cg19182289	6	33265386	*RGL2*	0.227	0.177	0.200	-0.051	-0.027	7.60E-61	1.10E-08
cg12118504	6	33265393	*RGL2*	0.395	0.314	0.359	-0.081	-0.035	1.90E-123	1.33E-14
cg01300096	6	33384490	*CUTA*	0.650	0.704	0.667	0.054	0.017	1.07E-88	9.14E-07
cg20118717	6	33400505	*SYNGAP1*	0.457	0.366	0.424	-0.091	-0.033	5.74E-109	2.94E-10
cg14725580	6	34663155	*C6orf106*	0.283	0.219	0.259	-0.064	-0.024	3.06E-82	5.86E-07
cg23709535	6	34993277	*ANKS1A*	0.682	0.628	0.651	-0.054	-0.031	2.40E-46	4.89E-07
cg06902099	6	35002597	*ANKS1A*	0.304	0.234	0.270	-0.070	-0.034	3.31E-86	2.32E-12
cg04739880	6	35017865	*ANKS1A*	0.426	0.352	0.396	-0.075	-0.030	2.18E-68	1.54E-06
cg26668919	6	35311534	*PPARD*	0.256	0.197	0.223	-0.059	-0.033	5.48E-71	9.26E-12
cg23254569	6	35451487	*TEAD3*	0.465	0.360	0.430	-0.105	-0.035	5.95E-139	5.07E-12
cg17568962	6	35456312	*TEAD3*	0.269	0.215	0.243	-0.054	-0.026	1.28E-112	4.10E-15
cg03591753	6	35659141	*FKBP5*	0.515	0.458	0.496	-0.058	-0.019	2.93E-100	1.27E-07
cg25114611	6	35696870	*LOC285847*	0.361	0.290	0.319	-0.071	-0.042	1.69E-79	3.59E-16
cg13719443	6	35700382	*LOC285847*	0.217	0.162	0.197	-0.055	-0.019	3.22E-88	2.66E-06
cg01618151	6	35707957	*ARMC12*	0.445	0.355	0.404	-0.090	-0.040	2.42E-147	2.41E-21
cg22027204	6	36216248	*PNPLA1*	0.292	0.225	0.252	-0.067	-0.040	4.49E-76	5.51E-14
cg06639585	6	36236936	*PNPLA1*	0.475	0.420	0.453	-0.055	-0.022	1.14E-74	4.62E-08
cg10550074	6	36304794	*C6orf222*	0.595	0.655	0.645	0.061	0.051	1.17E-36	4.39E-13
cg05389236	6	36635087	NA	0.474	0.389	0.439	-0.085	-0.035	2.19E-98	5.07E-13
cg23575275	6	36653973	*CDKN1A*	0.356	0.271	0.322	-0.085	-0.034	3.22E-103	1.10E-09
cg18964319	6	36665554	*RAB44*	0.337	0.256	0.308	-0.082	-0.030	1.11E-87	1.31E-08
cg12926693	6	36665611	*RAB44*	0.401	0.321	0.368	-0.080	-0.033	1.89E-144	1.20E-20
cg05569131	6	36665620	*RAB44*	0.384	0.304	0.350	-0.080	-0.034	3.40E-153	1.52E-20
cg26027052	6	36821364	NA	0.364	0.271	0.319	-0.093	-0.045	1.78E-135	5.89E-20
cg15512851	6	36973518	*FGD2*	0.322	0.253	0.286	-0.070	-0.036	1.50E-83	2.09E-11
cg17291166	6	37051189	NA	0.540	0.451	0.503	-0.089	-0.037	1.86E-150	1.17E-17
cg25325512	6	37142220	*PIM1*	0.409	0.337	0.351	-0.072	-0.058	5.95E-43	6.21E-14
cg08784966	6	37248768	*TBC1D22B*	0.535	0.474	0.512	-0.060	-0.023	1.32E-106	3.08E-11
cg09130674	6	39195019	*KCNK5*	0.292	0.217	0.253	-0.075	-0.039	5.57E-140	5.15E-24
cg08415973	6	40346114	NA	0.391	0.332	0.363	-0.058	-0.027	1.58E-57	8.80E-08
cg19136371	6	40357140	NA	0.663	0.725	0.700	0.062	0.037	4.77E-56	1.53E-11
cg01980222	6	41130917	*TREM2*	0.380	0.299	0.345	-0.081	-0.034	1.09E-97	4.77E-12
cg10725937	6	41130978	*TREM2*	0.468	0.365	0.421	-0.102	-0.046	9.19E-138	3.62E-19
cg10981439	6	41254433	*TREM1*	0.474	0.363	0.435	-0.111	-0.039	8.07E-135	2.42E-12
cg21328082	6	41254471	*TREM1*	0.435	0.324	0.393	-0.110	-0.042	1.82E-141	7.11E-14
cg09310966	6	41254825	*TREM1*	0.659	0.605	0.630	-0.054	-0.029	4.29E-47	2.68E-07
cg06196379	6	41254885	*TREM1*	0.334	0.269	0.297	-0.065	-0.037	1.75E-79	1.50E-16
cg08053935	6	41691270	*TFEB*	0.351	0.301	0.330	-0.050	-0.021	1.55E-102	8.38E-13
cg02255484	6	41735215	NA	0.316	0.262	0.288	-0.053	-0.028	1.11E-47	1.35E-06

cg25367558	6	41840102	*USP49*	0.626	0.533	0.596	-0.093	-0.030	4.06E-92	2.98E-07
cg11854981	6	42219847	*TRERF1*	0.415	0.340	0.386	-0.075	-0.029	1.17E-78	9.60E-08
cg06407843	6	43279607	NA	0.455	0.352	0.421	-0.103	-0.034	1.60E-109	2.70E-08
cg03143046	6	43758007	NA	0.454	0.340	0.404	-0.114	-0.050	3.21E-116	1.83E-13
cg12682870	6	43766804	NA	0.290	0.226	0.254	-0.063	-0.036	6.70E-89	5.94E-16
cg12481212	6	44020245	NA	0.390	0.311	0.356	-0.078	-0.034	5.61E-107	9.57E-14
cg04609694	6	44204009	NA	0.530	0.409	0.494	-0.120	-0.036	4.27E-156	1.92E-11
cg07188523	6	44528793	NA	0.511	0.422	0.469	-0.089	-0.042	3.62E-112	6.96E-14
cg00879824	6	44747344	NA	0.243	0.189	0.198	-0.054	-0.045	1.78E-52	2.76E-15
cg23261343	6	45413792	*RUNX2*	0.373	0.279	0.327	-0.094	-0.046	9.02E-137	1.47E-19
cg18434367	6	45891455	*CLIC5*	0.626	0.681	0.653	0.056	0.027	1.27E-49	8.66E-07
cg07120254	6	47199817	*TNFRSF21*	0.655	0.712	0.694	0.056	0.039	3.45E-29	1.97E-07
cg25920214	6	47442665	NA	0.759	0.811	0.813	0.051	0.053	1.87E-20	9.92E-11
cg19966146	6	49466300	*GLYATL3*	0.702	0.752	0.755	0.050	0.053	9.84E-22	6.05E-11
cg26714205	6	49522045	NA	0.695	0.755	0.745	0.060	0.050	1.24E-31	4.29E-11
cg07418892	6	52150456	*MCM3*	0.781	0.835	0.816	0.054	0.035	2.66E-50	3.54E-10
cg15448894	6	52840469	NA	0.432	0.351	0.401	-0.081	-0.030	1.20E-70	4.48E-07
cg15925365	6	52858998	*GSTA4*	0.695	0.635	0.655	-0.059	-0.039	6.63E-36	4.83E-10
cg19701087	6	52859008	*GSTA4*	0.637	0.568	0.593	-0.069	-0.044	1.91E-40	2.53E-11
cg22486834	6	52859107	*GSTA4*	0.674	0.621	0.640	-0.053	-0.034	4.31E-28	1.64E-06
cg16523850	6	54156824	NA	0.500	0.553	0.547	0.053	0.047	8.90E-15	3.74E-06
cg21879146	6	55887782	NA	0.694	0.745	0.729	0.051	0.036	1.10E-30	5.89E-08
cg15373592	6	56405167	*RNU6-71*	0.381	0.296	0.324	-0.085	-0.057	5.83E-108	1.53E-26
cg06311422	6	56406336	*RNU6-71*	0.384	0.288	0.324	-0.096	-0.060	7.24E-122	2.90E-25
cg15599182	6	56623606	NA	0.732	0.786	0.759	0.054	0.027	8.63E-63	2.40E-08
cg04319895	6	72955528	*RIMS1*	0.622	0.674	0.658	0.052	0.037	1.28E-23	2.45E-06
cg04911669	6	73697178	*KCNQ5*	0.558	0.464	0.516	-0.094	-0.042	2.05E-76	1.73E-09
cg04958236	6	74276973	NA	0.632	0.565	0.604	-0.067	-0.028	1.11E-77	3.51E-09
cg03760308	6	76310551	*SENP6*	0.570	0.499	0.534	-0.071	-0.036	2.56E-64	2.22E-08
cg24916055	6	78109388	NA	0.754	0.799	0.807	0.045	0.053	1.24E-18	4.24E-12
cg17536287	6	80224471	*LCA5*	0.668	0.707	0.721	0.039	0.052	3.23E-13	1.86E-10
cg21727359	6	82459006	*FAM46A*	0.424	0.328	0.382	-0.096	-0.042	3.55E-124	6.69E-16
cg04346283	6	82459454	*FAM46A*	0.365	0.274	0.316	-0.091	-0.050	4.29E-126	1.93E-20
cg09501509	6	89745356	NA	0.498	0.390	0.453	-0.108	-0.045	3.98E-140	8.44E-15
cg02159996	6	89927233	*GABRR1*	0.516	0.457	0.480	-0.059	-0.036	3.59E-70	3.33E-15
cg00333528	6	89927485	*GABRR1*	0.340	0.269	0.300	-0.072	-0.040	4.02E-110	6.55E-21
cg17486314	6	90778227	*BACH2*	0.624	0.678	0.655	0.055	0.031	4.10E-40	3.26E-08
cg27644327	6	90845852	*BACH2*	0.329	0.243	0.307	-0.086	-0.022	8.01E-139	2.97E-07
cg04932082	6	91113506	NA	0.370	0.282	0.334	-0.088	-0.036	1.13E-140	2.70E-16
cg03893901	6	106498597	NA	0.748	0.799	0.791	0.052	0.043	6.71E-37	2.04E-12
cg02331198	6	106988121	*AIM1*	0.380	0.300	0.345	-0.080	-0.035	9.88E-132	2.35E-16
cg02836135	6	108052093	*SCML4*	0.759	0.816	0.783	0.057	0.023	3.80E-94	1.76E-08
cg25015371	6	108874950	NA	0.492	0.435	0.461	-0.057	-0.031	7.24E-55	2.39E-10
cg09377088	6	108910284	*FOXO3*	0.572	0.512	0.538	-0.060	-0.035	2.71E-62	2.11E-12
cg04642300	6	109288592	*ARMC2*	0.292	0.232	0.256	-0.060	-0.036	1.99E-60	6.13E-11
cg16226866	6	110418140	NA	0.477	0.371	0.444	-0.105	-0.032	3.50E-100	6.42E-07
cg01431340	6	110680085	*METTL24*	0.337	0.302	0.278	-0.035	-0.059	4.08E-09	9.80E-11
cg06413398	6	110736865	*DDO*	0.321	0.290	0.269	-0.031	-0.051	4.60E-08	7.43E-09
cg00804078	6	110736941	*DDO*	0.350	0.305	0.285	-0.045	-0.065	3.97E-12	6.63E-11
cg07164639	6	110736958	*DDO*	0.403	0.372	0.343	-0.031	-0.060	3.25E-07	7.57E-11
cg06640254	6	111588373	*KIAA1919*	0.744	0.796	0.794	0.052	0.050	5.67E-21	6.03E-10
cg04350675	6	111873161	*TRAF3IP2-AS*	0.394	0.309	0.362	-0.085	-0.032	1.45E-83	1.08E-06
cg12636607	6	112154581	*FYN*	0.742	0.796	0.780	0.053	0.038	6.42E-33	1.19E-08
cg02789394	6	112191479	*FYN*	0.775	0.830	0.814	0.055	0.038	1.04E-51	1.10E-12
cg20222562	6	113993995	NA	0.520	0.391	0.455	-0.129	-0.065	2.80E-154	2.08E-26
cg16074643	6	116424276	*NT5DC1*	0.296	0.243	0.262	-0.053	-0.034	4.47E-47	2.63E-09
cg02575483	6	117826706	*DCBLD1*	0.378	0.288	0.339	-0.090	-0.039	2.22E-102	3.49E-11
cg21104965	6	117869453	*DCBLD1*	0.450	0.517	0.502	0.067	0.052	7.64E-20	9.91E-07
cg04344000	6	117869857	*DCBLD1*	0.730	0.793	0.754	0.063	0.024	3.05E-100	5.62E-09
cg01864699	6	122789987	*SERINC1*	0.762	0.813	0.810	0.052	0.048	3.38E-21	5.40E-09
cg16203203	6	125284659	NA	0.291	0.227	0.265	-0.064	-0.026	1.04E-65	1.98E-06
cg11610626	6	128222053	*THEMIS*	0.604	0.667	0.632	0.063	0.028	8.46E-69	8.14E-08
cg20267828	6	128222390	*THEMIS*	0.721	0.778	0.746	0.058	0.026	8.70E-75	3.25E-09
cg01699630	6	131894023	*ARG1*	0.558	0.439	0.526	-0.120	-0.032	3.54E-172	1.74E-10
cg24964103	6	133035379	*VNN1*	0.518	0.450	0.485	-0.068	-0.034	1.68E-41	1.76E-06
cg22825487	6	133055183	*VNN3*	0.483	0.385	0.447	-0.098	-0.035	2.81E-85	8.06E-07
cg21834463	6	134538515	*SGK1*	0.490	0.402	0.452	-0.088	-0.039	7.94E-76	2.80E-09
cg16001689	6	135042205	NA	0.359	0.279	0.310	-0.080	-0.049	3.39E-91	2.90E-19
cg08773226	6	135061398	NA	0.507	0.404	0.476	-0.103	-0.031	5.72E-123	2.04E-08
cg16077493	6	135172385	NA	0.753	0.803	0.807	0.050	0.054	1.42E-24	8.19E-13
cg26288715	6	135506834	*MYB*	0.370	0.280	0.335	-0.090	-0.035	2.27E-103	4.57E-10
cg14564351	6	135644405	*AHI1*	0.329	0.247	0.297	-0.082	-0.031	1.41E-91	4.93E-08
cg24481882	6	136450701	*PDE7B*	0.472	0.386	0.434	-0.086	-0.037	9.08E-113	1.03E-13
cg21889703	6	136607649	*BCLAF1*	0.641	0.703	0.675	0.062	0.034	1.52E-46	4.08E-08
cg01544877	6	136609152	*BCLAF1*	0.734	0.790	0.763	0.055	0.029	1.06E-46	4.18E-06
cg00235484	6	136825415	*MAP7*	0.511	0.404	0.474	-0.107	-0.037	2.52E-105	5.85E-08
cg02673417	6	137105249	*MAP3K5*	0.330	0.250	0.290	-0.081	-0.040	1.64E-121	2.35E-18
cg04068005	6	137162231	*PEX7*	0.691	0.748	0.754	0.057	0.063	1.69E-18	8.22E-11

cg01357222	6	137619143	NA	0.467	0.369	0.422	-0.098	-0.045	3.36E-136	9.51E-19
cg06132620	6	138820503	*NHSL1*	0.244	0.187	0.214	-0.057	-0.030	2.99E-72	1.68E-10
cg26525313	6	139466669	*HECA*	0.766	0.819	0.794	0.053	0.027	2.28E-42	2.06E-07
cg23280720	6	139483193	*HECA*	0.614	0.708	0.640	0.094	0.026	7.44E-145	6.52E-09
cg25278941	6	139795527	*LOC645434*	0.320	0.248	0.270	-0.072	-0.050	1.19E-98	5.33E-26
cg22406102	6	139947852	NA	0.190	0.137	0.162	-0.052	-0.028	4.44E-90	5.20E-13
cg25772365	6	140762224	NA	0.591	0.652	0.635	0.061	0.044	2.16E-29	4.75E-08
cg10816760	6	142890973	*LOC153910*	0.481	0.543	0.511	0.062	0.029	3.53E-43	4.91E-06
cg22751954	6	143023932	NA	0.280	0.222	0.252	-0.057	-0.028	2.48E-104	4.19E-13
cg20743720	6	143257108	*HIVEP2*	0.716	0.777	0.763	0.061	0.046	8.15E-41	3.94E-13
cg17586302	6	144013969	*PHACTR2*	0.523	0.420	0.488	-0.103	-0.036	2.06E-125	2.95E-10
cg04410448	6	144223614	*ZC2HC1B*	0.693	0.750	0.730	0.057	0.037	2.92E-37	5.37E-09
cg02566775	6	144382964	*PLAGL1*	0.454	0.386	0.423	-0.067	-0.030	1.52E-69	2.06E-08
cg00488091	6	144536326	NA	0.429	0.359	0.401	-0.070	-0.028	1.77E-81	3.41E-09
cg16199747	6	147235033	*STXBP5-AS1*	0.307	0.232	0.258	-0.075	-0.048	3.83E-83	3.43E-17
cg03052078	6	147527113	*STXBP5*	0.416	0.340	0.382	-0.076	-0.034	7.34E-64	2.50E-06
cg09853238	6	149532290	NA	0.254	0.184	0.202	-0.070	-0.052	6.86E-82	1.45E-20
cg20331980	6	149554503	NA	0.694	0.745	0.718	0.051	0.023	3.90E-67	4.76E-08
cg07346187	6	149775621	*ZC3H12D*	0.296	0.227	0.271	-0.068	-0.025	2.45E-96	3.06E-09
cg04494800	6	149775853	*ZC3H12D*	0.407	0.354	0.385	-0.053	-0.022	5.39E-51	5.92E-06
cg14417099	6	150073879	*PCMT1*	0.439	0.331	0.401	-0.108	-0.038	9.87E-148	1.74E-13
cg24114587	6	150212725	*RAET1E*	0.743	0.799	0.774	0.055	0.031	7.64E-36	5.36E-06
cg24863152	6	151134876	*PLEKHG1*	0.599	0.654	0.613	0.056	0.014	2.48E-115	5.50E-06
cg16336494	6	151188259	*MTHFD1L*	0.357	0.278	0.312	-0.079	-0.045	4.04E-83	2.49E-14
cg21691367	6	151325642	*MTHFD1L*	0.402	0.322	0.356	-0.080	-0.046	3.92E-78	1.25E-14
cg07805500	6	151380818	*MTHFD1L*	0.386	0.303	0.358	-0.083	-0.028	2.69E-85	3.26E-06
cg26696162	6	151709522	*ZBTB2*	0.343	0.261	0.310	-0.082	-0.033	6.17E-107	8.55E-11
cg18132851	6	152085641	*ESR1*	0.305	0.243	0.274	-0.063	-0.031	7.59E-53	2.24E-07
cg14685356	6	152639548	*SYNE1*	0.438	0.345	0.400	-0.092	-0.038	1.85E-130	2.67E-15
cg19384905	6	153299788	*FBXO5*	0.349	0.279	0.323	-0.070	-0.026	2.16E-84	1.01E-06
cg24465935	6	153938192	NA	0.723	0.775	0.761	0.051	0.037	1.20E-27	1.31E-07
cg04170440	6	154410350	*OPRM1*	0.737	0.791	0.775	0.054	0.038	2.38E-28	3.19E-07
cg19963522	6	154677796	*IPCEF1*	0.359	0.282	0.317	-0.077	-0.042	1.70E-96	3.91E-19
cg08486269	6	154677972	*IPCEF1*	0.246	0.187	0.222	-0.059	-0.023	4.40E-64	4.59E-06
cg19690214	6	154678326	*IPCEF1*	0.373	0.311	0.337	-0.062	-0.036	1.06E-46	4.33E-09
cg21551549	6	155053894	*SCAF8*	0.345	0.286	0.322	-0.059	-0.023	9.34E-68	6.15E-06
cg02202664	6	155545253	*TIAM2*	0.569	0.620	0.604	0.051	0.035	3.62E-32	2.99E-08
cg05081167	6	156967138	NA	0.266	0.202	0.223	-0.064	-0.044	1.50E-75	1.59E-17
cg15262325	6	157296274	*ARID1B*	0.769	0.820	0.805	0.052	0.037	7.53E-32	1.49E-08
cg06012428	6	157477204	*ARID1B*	0.698	0.640	0.671	-0.058	-0.027	8.14E-76	7.98E-10
cg03209720	6	157727126	*TMEM242*	0.341	0.284	0.322	-0.057	-0.019	7.42E-97	2.38E-07
cg17310882	6	158066609	*ZDHHC14*	0.432	0.351	0.400	-0.081	-0.032	3.25E-98	4.74E-10
cg17852032	6	158066811	*ZDHHC14*	0.306	0.250	0.279	-0.056	-0.027	8.41E-71	7.76E-10
cg23206115	6	158066900	*ZDHHC14*	0.533	0.428	0.494	-0.105	-0.039	2.12E-114	1.10E-10
cg18747197	6	158110124	NA	0.528	0.468	0.498	-0.061	-0.031	8.54E-69	1.83E-11
cg00581764	6	158183853	NA	0.294	0.234	0.270	-0.061	-0.024	3.53E-85	4.60E-08
cg12063847	6	158184542	NA	0.343	0.292	0.320	-0.051	-0.022	1.16E-75	7.96E-09
cg18419358	6	158384009	NA	0.512	0.449	0.451	-0.064	-0.061	1.14E-22	3.00E-10
cg13568659	6	159438801	NA	0.639	0.700	0.669	0.061	0.031	2.45E-51	1.02E-07
cg23344523	6	160380572	NA	0.652	0.601	0.626	-0.051	-0.026	9.92E-45	7.51E-07
cg04733681	6	160423822	*IGF2R*	0.734	0.787	0.788	0.053	0.054	1.73E-21	2.80E-10
cg07567256	6	163757048	NA	0.277	0.222	0.258	-0.056	-0.020	5.32E-69	4.60E-06
cg07646791	6	166667648	NA	0.267	0.194	0.242	-0.073	-0.025	3.37E-88	2.25E-06
cg17530030	6	166699419	NA	0.309	0.250	0.274	-0.059	-0.035	1.41E-58	9.82E-11
cg06491415	6	166825479	*RPS6KA2*	0.703	0.767	0.728	0.065	0.025	7.43E-126	1.05E-13
cg08557970	6	166856094	*RPS6KA2*	0.494	0.383	0.441	-0.110	-0.053	6.39E-158	2.40E-25
cg11744817	6	166876826	*RPS6KA2*	0.396	0.321	0.345	-0.075	-0.051	1.89E-25	1.84E-07
cg06702850	6	166876890	*RPS6KA2*	0.421	0.325	0.374	-0.096	-0.047	2.27E-68	6.12E-11
cg07253384	6	166877002	*RPS6KA2*	0.345	0.255	0.300	-0.090	-0.044	5.41E-105	1.56E-14
cg02287007	6	166877038	*RPS6KA2*	0.372	0.284	0.336	-0.088	-0.036	8.82E-81	4.07E-08
cg11387340	6	166970727	*RPS6KA2*	0.312	0.256	0.275	-0.056	-0.037	1.68E-45	1.01E-11
cg13072943	6	167011311	*RPS6KA2*	0.535	0.483	0.487	-0.052	-0.048	1.85E-27	2.79E-13
cg15090899	6	167178260	*RPS6KA2*	0.750	0.808	0.798	0.058	0.049	1.04E-31	5.33E-10
cg26954951	6	167507568	NA	0.553	0.607	0.580	0.054	0.026	2.40E-74	5.35E-12
cg06682039	6	170536047	NA	0.487	0.405	0.448	-0.082	-0.038	1.83E-52	1.79E-07
cg06684088	7	119131	NA	0.422	0.312	0.388	-0.110	-0.034	1.11E-111	2.85E-07
cg04650322	7	119499	NA	0.317	0.264	0.297	-0.053	-0.020	5.36E-73	7.93E-07
cg10251229	7	630581	*PRKAR1B*	0.584	0.509	0.553	-0.075	-0.031	2.25E-50	3.70E-06
cg02217713	7	643155	*PRKAR1B*	0.524	0.459	0.475	-0.065	-0.050	2.64E-66	2.01E-21
cg21511321	7	1004997	*COX19*	0.371	0.300	0.341	-0.071	-0.030	4.73E-112	1.10E-12
cg16245431	7	1005089	*COX19*	0.361	0.284	0.330	-0.077	-0.031	7.69E-94	5.86E-09
cg06242730	7	1064100	*C7orf50*	0.328	0.253	0.291	-0.075	-0.037	9.29E-125	2.30E-17
cg04816311	7	1066650	*C7orf50*	0.521	0.463	0.486	-0.059	-0.036	8.29E-38	9.80E-09
cg17158941	7	1073255	*C7orf50*	0.765	0.816	0.783	0.052	0.019	2.14E-89	2.49E-07
cg08973950	7	1083309	*C7orf50*	0.487	0.427	0.443	-0.061	-0.045	5.50E-32	1.65E-09
cg19340455	7	1095720	*C7orf50*	0.290	0.230	0.269	-0.059	-0.020	1.07E-97	2.75E-07
cg23657099	7	1102138	*C7orf50*	0.332	0.263	0.296	-0.069	-0.036	7.29E-69	1.76E-09
cg24025721	7	1220962	NA	0.569	0.482	0.534	-0.087	-0.035	2.12E-98	3.37E-12

cg25843426	7	1497307	*MICALL2*	0.367	0.304	0.341	-0.063	-0.026	1.17E-76	2.87E-09
cg08525429	7	1553444	NA	0.387	0.312	0.357	-0.075	-0.030	7.81E-85	4.19E-08
cg26697605	7	1850801	NA	0.385	0.293	0.348	-0.093	-0.037	4.00E-86	4.41E-08
cg15997393	7	1961869	*MAD1L1*	0.666	0.751	0.710	0.085	0.044	3.37E-93	3.91E-16
cg18542546	7	1982333	*MAD1L1*	0.539	0.420	0.509	-0.119	-0.030	5.47E-139	6.31E-07
cg02827175	7	1986245	*MAD1L1*	0.666	0.577	0.637	-0.089	-0.029	3.75E-78	2.15E-06
cg01128574	7	2058865	*MAD1L1*	0.410	0.344	0.382	-0.066	-0.028	8.71E-96	1.25E-11
cg13844341	7	2116368	*MAD1L1*	0.568	0.479	0.540	-0.089	-0.028	1.82E-144	2.52E-12
cg03116466	7	2116512	*MAD1L1*	0.432	0.363	0.408	-0.068	-0.023	3.74E-142	2.42E-13
cg02185182	7	2185550	*MAD1L1*	0.509	0.420	0.485	-0.089	-0.024	8.25E-90	1.44E-06
cg10152449	7	2444534	*CHST12*	0.388	0.275	0.307	-0.113	-0.081	3.58E-102	9.45E-28
cg00892324	7	2565575	*LFNG*	0.376	0.299	0.347	-0.076	-0.029	1.03E-135	4.43E-13
cg23510807	7	2567893	*LFNG*	0.649	0.702	0.668	0.052	0.018	5.51E-108	2.59E-08
cg04919592	7	2607232	*IQCE*	0.467	0.382	0.444	-0.084	-0.023	7.19E-130	1.65E-07
cg19869035	7	2653955	*IQCE*	0.447	0.350	0.414	-0.096	-0.033	1.18E-143	1.10E-12
cg16481332	7	2654053	*IQCE*	0.451	0.346	0.408	-0.104	-0.043	7.33E-138	8.83E-15
cg09595020	7	2654120	*IQCE*	0.409	0.348	0.380	-0.061	-0.029	1.07E-64	4.44E-08
cg14933494	7	2661902	NA	0.354	0.296	0.322	-0.058	-0.032	4.34E-92	9.86E-20
cg11967765	7	2774195	*GNA12*	0.352	0.280	0.329	-0.072	-0.023	1.48E-110	1.18E-07
cg23587176	7	4746837	*FOXK1*	0.658	0.559	0.616	-0.099	-0.043	1.17E-114	1.73E-16
cg04261496	7	4753002	*FOXK1*	0.390	0.299	0.364	-0.091	-0.025	3.32E-118	1.39E-07
cg20367388	7	5258485	*WIPI2*	0.419	0.316	0.374	-0.103	-0.045	2.38E-91	1.51E-09
cg15613991	7	5277080	NA	0.221	0.170	0.197	-0.051	-0.023	1.11E-59	2.06E-07
cg06898168	7	5447353	*TNRC18*	0.429	0.369	0.409	-0.060	-0.020	7.13E-117	1.46E-09
cg20427144	7	5518918	*FBXL18*	0.451	0.381	0.424	-0.071	-0.028	2.73E-70	2.71E-06
cg04682905	7	5523636	*FBXL18*	0.474	0.405	0.452	-0.069	-0.022	3.06E-103	9.85E-08
cg11554295	7	5523691	*FBXL18*	0.476	0.412	0.457	-0.063	-0.019	1.70E-102	1.33E-06
cg23728359	7	6290148	*CYTH3*	0.765	0.824	0.804	0.059	0.039	1.52E-42	1.25E-09
cg06987246	7	6659785	*ZNF853*	0.543	0.645	0.580	0.102	0.037	1.23E-148	4.53E-15
cg04089901	7	7557590	*COL28A1*	0.424	0.323	0.376	-0.101	-0.048	1.16E-133	3.72E-18
cg10131879	7	8011594	*GLCCI1*	0.668	0.718	0.706	0.051	0.039	3.58E-36	5.81E-11
cg21380380	7	8011838	*GLCCI1*	0.630	0.707	0.688	0.077	0.058	1.84E-56	7.28E-17
cg17808910	7	8012104	*GLCCI1*	0.750	0.801	0.787	0.051	0.037	2.58E-34	5.86E-10
cg20022118	7	8276021	*ICA1*	0.703	0.759	0.754	0.056	0.051	6.78E-32	6.23E-13
cg26251585	7	11456440	*THSD7A*	0.682	0.740	0.711	0.057	0.028	1.67E-46	7.57E-07
cg11384744	7	16794623	*TSPAN13*	0.370	0.292	0.332	-0.078	-0.038	1.23E-88	2.83E-11
cg12192749	7	17029795	NA	0.449	0.354	0.401	-0.095	-0.048	8.42E-123	5.18E-19
cg12512771	7	17730027	NA	0.269	0.209	0.244	-0.060	-0.025	4.68E-68	2.12E-06
cg18655898	7	19745167	*TWISTNB*	0.746	0.787	0.803	0.041	0.056	1.31E-16	4.95E-14
cg17344321	7	22617382	NA	0.342	0.247	0.304	-0.094	-0.038	8.03E-117	5.47E-12
cg16954385	7	23246896	NA	0.507	0.397	0.471	-0.110	-0.036	4.70E-130	4.74E-11
cg03240301	7	23387383	*IGF2BP3*	0.360	0.276	0.309	-0.085	-0.051	2.50E-93	1.89E-17
cg17209188	7	23387396	*IGF2BP3*	0.467	0.382	0.430	-0.085	-0.037	2.01E-98	1.19E-11
cg21621538	7	24614206	*MPP6*	0.368	0.314	0.333	-0.054	-0.035	5.37E-56	7.40E-13
cg13311549	7	24988669	*OSBPL3*	0.747	0.804	0.792	0.057	0.045	1.44E-33	2.66E-10
cg23302989	7	25174492	*C7orf31*	0.542	0.422	0.498	-0.120	-0.043	6.17E-180	6.45E-17
cg01322214	7	25219198	*C7orf31*	0.484	0.418	0.442	-0.066	-0.041	3.43E-31	4.70E-06
cg03700230	7	25702848	NA	0.692	0.749	0.728	0.057	0.036	2.34E-31	5.60E-07
cg21699330	7	26193032	*NFE2L3*	0.215	0.160	0.162	-0.055	-0.053	3.84E-39	2.34E-14
cg12510708	7	26193805	*NFE2L3*	0.337	0.252	0.274	-0.085	-0.063	1.77E-85	2.00E-23
cg24024511	7	26676346	*C7orf71*	0.775	0.820	0.828	0.046	0.054	2.87E-17	1.18E-10
cg00524179	7	27166853	*HOXA3*	0.741	0.795	0.786	0.054	0.045	2.61E-33	3.37E-12
cg25975690	7	27199726	NA	0.397	0.334	0.363	-0.064	-0.034	1.33E-46	8.27E-07
cg03217995	7	27203430	*HOXA10-HOX*	0.305	0.238	0.276	-0.068	-0.029	1.19E-60	2.95E-06
cg21007852	7	27203546	*HOXA10-HOX*	0.258	0.202	0.226	-0.056	-0.033	1.84E-71	1.60E-12
cg17681079	7	27556963	NA	0.754	0.805	0.809	0.051	0.055	7.76E-25	9.54E-14
cg02842382	7	27615496	*HIBADH*	0.354	0.301	0.324	-0.053	-0.030	2.88E-51	5.77E-09
cg17342588	7	27737875	NA	0.294	0.235	0.264	-0.059	-0.030	4.98E-67	3.20E-10
cg23597162	7	28102341	*JAZF1*	0.514	0.425	0.482	-0.090	-0.033	8.78E-110	3.30E-10
cg20704555	7	28127748	*JAZF1*	0.506	0.407	0.472	-0.099	-0.034	7.55E-106	1.13E-08
cg02010481	7	28218524	*JAZF1*	0.225	0.167	0.192	-0.058	-0.032	1.09E-64	4.52E-11
cg04266607	7	28221667	*JAZF1*	0.743	0.796	0.777	0.053	0.034	9.46E-37	9.11E-08
cg21581567	7	28502140	*CREB5*	0.652	0.713	0.697	0.062	0.045	3.62E-43	8.63E-12
cg04926881	7	29024378	*LOC1005064*	0.319	0.266	0.289	-0.053	-0.030	1.98E-41	1.07E-06
cg10591077	7	30030138	*SCRN1*	0.634	0.689	0.689	0.056	0.055	6.04E-21	1.00E-09
cg02203380	7	30173809	*C7orf41*	0.263	0.209	0.222	-0.054	-0.040	7.37E-43	5.18E-12
cg07136111	7	30829005	*FAM188B*	0.423	0.325	0.379	-0.098	-0.044	3.49E-134	4.69E-16
cg06312985	7	32961866	*RP9P*	0.364	0.278	0.326	-0.086	-0.038	2.25E-118	4.32E-13
cg02271603	7	33197291	*BBS9*	0.543	0.601	0.572	0.058	0.029	2.78E-40	2.79E-06
cg06732873	7	35731578	*HERPUD2*	0.634	0.706	0.678	0.071	0.043	5.53E-56	4.94E-12
cg21762695	7	36022695	NA	0.471	0.382	0.431	-0.089	-0.040	7.79E-76	2.37E-09
cg16328023	7	36382071	*KIAA0895*	0.261	0.209	0.227	-0.052	-0.034	2.05E-37	4.92E-09
cg25028542	7	36429120	*ANLN*	0.260	0.202	0.230	-0.058	-0.030	3.52E-60	5.49E-09
cg15388107	7	36700916	*AOAH*	0.433	0.365	0.402	-0.068	-0.031	2.54E-82	2.32E-10
cg00390511	7	36701016	*AOAH*	0.563	0.503	0.529	-0.060	-0.033	1.97E-37	6.25E-07
cg08044454	7	37024552	*ELMO1*	0.414	0.312	0.370	-0.102	-0.044	2.28E-140	4.52E-16
cg01119452	7	37287850	*ELMO1*	0.418	0.307	0.359	-0.110	-0.059	8.64E-137	2.95E-23
cg05642546	7	37298927	*ELMO1*	0.448	0.323	0.394	-0.124	-0.054	9.78E-182	4.99E-26

cg05383910	7	37431792	*ELMO1*	0.285	0.219	0.253	-0.066	-0.032	2.71E-74	1.86E-08
cg26065247	7	37734052	NA	0.363	0.288	0.325	-0.075	-0.038	4.02E-160	6.23E-28
cg15141868	7	37959966	*EPDR1*	0.274	0.218	0.240	-0.056	-0.034	7.56E-43	2.16E-07
cg13922442	7	38312366	*TARP*	0.760	0.811	0.804	0.051	0.044	6.80E-35	1.37E-11
cg05988548	7	38695932	NA	0.379	0.329	0.356	-0.050	-0.023	6.93E-55	2.36E-07
cg19998533	7	41486779	NA	0.754	0.806	0.787	0.051	0.033	8.80E-41	1.37E-08
cg01412469	7	41732237	*INHBA*	0.396	0.345	0.358	-0.050	-0.037	1.81E-58	2.49E-16
cg26478599	7	41747322	*INHBA-AS1*	0.506	0.393	0.453	-0.113	-0.054	1.19E-141	1.06E-19
cg02088996	7	41817771	*INHBA-AS1*	0.285	0.224	0.243	-0.061	-0.042	1.49E-79	4.70E-20
cg23007665	7	43690247	*COA1*	0.591	0.673	0.636	0.082	0.045	2.16E-76	1.22E-12
cg12375586	7	43698429	*COA1*	0.759	0.820	0.779	0.061	0.019	8.76E-100	7.88E-07
cg01399219	7	44145110	*AEBP1*	0.226	0.172	0.202	-0.054	-0.024	6.57E-74	4.18E-08
cg12573289	7	45075791	*CCM2*	0.469	0.528	0.487	0.058	0.018	2.02E-106	1.52E-08
cg23532927	7	45977067	NA	0.398	0.341	0.362	-0.057	-0.036	9.87E-44	9.32E-09
cg16329896	7	47515060	*TNS3*	0.370	0.296	0.342	-0.074	-0.028	8.18E-89	6.49E-09
cg27574991	7	47537004	*TNS3*	0.361	0.288	0.324	-0.073	-0.037	5.77E-85	6.25E-12
cg07488141	7	47560215	*TNS3*	0.442	0.338	0.387	-0.104	-0.055	3.91E-136	4.76E-22
cg17996401	7	47611235	*TNS3*	0.323	0.268	0.301	-0.055	-0.022	1.04E-85	5.49E-08
cg26541218	7	47826387	*PKD1L1*	0.445	0.380	0.402	-0.066	-0.043	2.11E-41	1.02E-08
cg07880727	7	47846424	*C7orf69*	0.345	0.266	0.307	-0.080	-0.039	1.24E-104	7.25E-15
cg02703728	7	48127997	*UPP1*	0.291	0.233	0.252	-0.058	-0.039	1.47E-61	6.23E-13
cg18023455	7	49894664	*VWC2*	0.674	0.736	0.715	0.062	0.042	1.23E-38	7.38E-10
cg18743793	7	50464661	*IKZF1*	0.674	0.737	0.706	0.063	0.032	1.20E-84	1.05E-11
cg20732703	7	50744032	*GRB10*	0.421	0.329	0.382	-0.091	-0.038	3.75E-98	4.61E-11
cg24472823	7	51296131	*COBL*	0.713	0.769	0.760	0.056	0.047	6.27E-33	2.69E-11
cg25815893	7	55199930	*EGFR*	0.687	0.744	0.732	0.056	0.045	3.35E-27	1.14E-08
cg21959598	7	55637719	*VOPP1*	0.712	0.782	0.743	0.070	0.031	1.07E-95	8.52E-11
cg12856392	7	64126140	*ZNF107*	0.638	0.696	0.681	0.058	0.043	7.21E-24	2.15E-07
cg01323964	7	65219171	*CCT6P1*	0.516	0.397	0.480	-0.120	-0.036	9.85E-148	1.40E-10
cg23594656	7	65796392	*TPST1*	0.741	0.792	0.778	0.051	0.037	4.03E-33	3.86E-09
cg04484126	7	65839403	NA	0.453	0.344	0.408	-0.109	-0.045	8.69E-180	7.16E-26
cg18550262	7	72812030	NA	0.318	0.261	0.292	-0.057	-0.026	1.54E-55	8.19E-08
cg17314888	7	73442282	*ELN*	0.305	0.248	0.280	-0.057	-0.025	9.84E-57	3.01E-07
cg18325315	7	73897230	*GTF2IRD1*	0.547	0.427	0.507	-0.121	-0.040	2.13E-151	4.64E-14
cg25444339	7	75194698	*HIP1*	0.394	0.294	0.349	-0.101	-0.045	2.30E-201	8.76E-32
cg06825878	7	75472540	NA	0.297	0.235	0.267	-0.062	-0.030	3.87E-54	8.52E-08
cg20720686	7	75582881	*POR*	0.560	0.456	0.521	-0.104	-0.039	1.05E-162	2.75E-16
cg20748065	7	75583421	*POR*	0.315	0.246	0.288	-0.069	-0.027	2.37E-106	7.75E-10
cg27342333	7	75592418	*POR*	0.350	0.288	0.312	-0.062	-0.037	3.62E-68	4.79E-14
cg07733719	7	75683547	*MDH2*	0.438	0.347	0.400	-0.091	-0.038	5.86E-179	1.44E-23
cg03707599	7	75956931	*YWHAG*	0.350	0.275	0.318	-0.075	-0.032	1.30E-64	1.45E-06
cg10738119	7	75957040	*YWHAG*	0.434	0.342	0.399	-0.093	-0.036	1.35E-133	1.50E-12
cg26162326	7	75957061	*YWHAG*	0.459	0.364	0.433	-0.095	-0.026	1.90E-123	5.51E-07
cg19268947	7	77268833	*PTPN12*	0.634	0.687	0.693	0.054	0.060	1.25E-16	3.05E-09
cg18983132	7	78024305	*MAGI2*	0.683	0.733	0.714	0.050	0.032	2.50E-38	6.86E-08
cg06745740	7	81399587	*HGF*	0.244	0.188	0.212	-0.056	-0.031	2.79E-48	2.07E-07
cg24605288	7	84628407	*SEMA3D*	0.733	0.784	0.772	0.051	0.039	7.85E-22	2.36E-06
cg08605855	7	86825549	*DMTF1*	0.783	0.833	0.821	0.050	0.038	2.00E-24	6.84E-07
cg03834767	7	90794392	*CDK14*	0.273	0.213	0.243	-0.060	-0.030	4.13E-67	2.03E-09
cg07302959	7	92198639	*FAM133DP*	0.387	0.286	0.349	-0.101	-0.038	2.19E-129	6.23E-12
cg08300570	7	92353454	*CDK6*	0.480	0.370	0.436	-0.110	-0.044	3.29E-149	8.22E-17
cg27455918	7	92370191	*CDK6*	0.695	0.745	0.752	0.050	0.056	1.06E-22	6.21E-13
cg05278074	7	92442568	*CDK6*	0.668	0.734	0.721	0.065	0.053	9.88E-44	1.67E-14
cg07266431	7	92460113	*CDK6*	0.211	0.156	0.175	-0.055	-0.036	2.21E-67	4.61E-13
cg19103429	7	92460784	*CDK6*	0.284	0.227	0.244	-0.057	-0.040	1.55E-47	2.58E-12
cg08038054	7	93550781	*GNG11*	0.483	0.425	0.446	-0.058	-0.037	3.74E-30	4.24E-07
cg18194887	7	93629937	*BET1*	0.612	0.665	0.670	0.052	0.057	1.81E-20	1.12E-11
cg27185510	7	95811907	*SLC25A13*	0.707	0.768	0.763	0.062	0.056	4.73E-37	6.56E-15
cg26460483	7	97914016	*BRI3*	0.328	0.263	0.302	-0.065	-0.027	7.14E-142	2.22E-17
cg01877450	7	97915802	*BRI3*	0.428	0.362	0.409	-0.065	-0.019	1.26E-112	1.27E-08
cg24407596	7	98718683	*SMURF1*	0.441	0.379	0.417	-0.062	-0.024	9.42E-59	5.36E-06
cg18349022	7	99222459	*ZNF498*	0.542	0.603	0.580	0.061	0.038	1.51E-44	3.02E-10
cg14714629	7	99495452	*TRIM4*	0.351	0.285	0.327	-0.066	-0.024	5.48E-89	8.00E-07
cg06257058	7	99683264	NA	0.471	0.350	0.415	-0.121	-0.056	1.88E-172	6.89E-26
cg07212384	7	99761053	*GAL3ST4*	0.703	0.769	0.728	0.066	0.025	1.53E-83	2.95E-07
cg17442155	7	99955675	*PILRB*	0.522	0.407	0.474	-0.115	-0.049	3.50E-143	4.52E-14
cg03121834	7	99970448	*PILRA*	0.333	0.273	0.303	-0.061	-0.030	6.59E-95	9.34E-15
cg02192746	7	99971016	*PILRA*	0.356	0.283	0.327	-0.073	-0.029	1.12E-129	3.94E-15
cg00030432	7	100028974	*MEPCE*	0.464	0.382	0.444	-0.082	-0.020	1.11E-132	9.58E-07
cg22728904	7	100433983	NA	0.265	0.209	0.241	-0.055	-0.024	2.20E-62	8.01E-07
cg00374672	7	100463416	*SLC12A9*	0.326	0.249	0.290	-0.077	-0.036	1.35E-94	8.96E-13
cg19279257	7	100463508	*TRIP6*	0.448	0.379	0.418	-0.069	-0.030	1.60E-60	1.70E-07
cg26305174	7	100463583	*TRIP6*	0.423	0.341	0.393	-0.083	-0.030	1.13E-86	4.76E-09
cg04742719	7	100463759	*TRIP6*	0.413	0.335	0.384	-0.078	-0.029	1.45E-96	4.55E-10
cg01008256	7	100463985	*TRIP6*	0.354	0.280	0.322	-0.074	-0.032	8.48E-77	3.22E-09
cg12967723	7	100464145	*TRIP6*	0.426	0.356	0.405	-0.069	-0.021	4.28E-84	2.55E-07
cg08132858	7	101361395	NA	0.398	0.319	0.367	-0.079	-0.031	8.02E-125	5.43E-16
cg23723486	7	101361745	NA	0.414	0.352	0.386	-0.061	-0.027	1.30E-83	2.82E-11

cg25711558	7	101499638	*CUX1*	0.398	0.298	0.358	-0.100	-0.040	3.94E-113	2.75E-12
cg23445003	7	101535821	*CUX1*	0.460	0.344	0.414	-0.116	-0.047	2.36E-167	6.52E-18
cg02508830	7	102576358	*FBXL13*	0.309	0.235	0.276	-0.074	-0.033	4.92E-97	2.56E-11
cg05671644	7	102632303	*FBXL13*	0.483	0.406	0.447	-0.077	-0.036	2.00E-79	1.73E-11
cg25968394	7	104604511	NA	0.525	0.424	0.496	-0.101	-0.028	5.63E-120	6.78E-08
cg11531783	7	104696669	*MLL5*	0.668	0.708	0.720	0.040	0.052	1.48E-11	9.88E-09
cg14427668	7	105319437	*ATXN7L1*	0.282	0.227	0.263	-0.056	-0.019	1.81E-114	1.96E-08
cg00423035	7	105319486	*ATXN7L1*	0.405	0.319	0.372	-0.086	-0.033	7.12E-108	7.61E-11
cg08645907	7	105319679	*ATXN7L1*	0.278	0.220	0.257	-0.058	-0.021	2.08E-82	4.10E-08
cg14989988	7	105319819	*ATXN7L1*	0.483	0.423	0.446	-0.060	-0.037	6.52E-55	1.95E-11
cg06547017	7	105514694	*ATXN7L1*	0.768	0.827	0.809	0.059	0.042	3.12E-41	7.33E-10
cg00139681	7	106125168	NA	0.463	0.406	0.434	-0.057	-0.029	4.93E-48	3.20E-07
cg09803764	7	107572753	*LAMB1*	0.450	0.363	0.415	-0.087	-0.035	3.78E-94	2.02E-09
cg20438043	7	107601757	*LAMB1*	0.674	0.723	0.727	0.048	0.053	2.23E-19	3.62E-11
cg21884374	7	107807954	*NRCAM*	0.690	0.748	0.752	0.058	0.062	4.99E-24	1.93E-13
cg17368726	7	108169094	*PNPLA8*	0.728	0.779	0.771	0.051	0.043	8.04E-20	4.33E-07
cg06985880	7	108525723	*C7orf66*	0.752	0.794	0.809	0.042	0.057	6.74E-16	8.42E-13
cg06545367	7	110731527	*IMMP2L*	0.747	0.800	0.803	0.054	0.057	2.52E-28	3.86E-13
cg19724567	7	112063079	*IFRD1*	0.276	0.217	0.241	-0.059	-0.035	1.43E-67	1.00E-11
cg06225476	7	116169488	*CAV1*	0.675	0.739	0.716	0.064	0.041	3.99E-39	1.44E-08
cg05667818	7	116786870	*ST7*	0.384	0.302	0.338	-0.082	-0.046	2.58E-94	1.35E-16
cg21921829	7	116850153	*ST7*	0.688	0.741	0.735	0.053	0.047	2.95E-21	9.57E-09
cg13391244	7	120629638	*CPED1*	0.421	0.363	0.387	-0.058	-0.035	1.63E-34	2.36E-06
cg13561372	7	121781778	*AASS*	0.710	0.767	0.760	0.057	0.050	5.67E-26	1.11E-09
cg20475005	7	123351286	*WASL*	0.772	0.824	0.803	0.051	0.031	1.74E-40	7.62E-08
cg22539431	7	127423253	*SND1*	0.422	0.340	0.381	-0.082	-0.041	4.34E-86	1.26E-12
cg18030799	7	127429445	*SND1*	0.700	0.752	0.759	0.051	0.058	5.82E-19	5.83E-11
cg06173720	7	127670993	*LRRC4*	0.317	0.248	0.291	-0.069	-0.027	6.70E-106	3.36E-09
cg02538772	7	127699264	*SND1*	0.679	0.749	0.713	0.070	0.034	6.57E-99	2.84E-13
cg14523734	7	128020864	NA	0.715	0.779	0.746	0.064	0.031	9.12E-144	6.01E-21
cg12517576	7	128048308	*IMPDH1*	0.367	0.276	0.330	-0.090	-0.037	1.67E-152	5.21E-18
cg13914531	7	128579876	*IRF5*	0.260	0.201	0.229	-0.059	-0.031	2.29E-102	4.97E-16
cg05904013	7	128579933	*IRF5*	0.418	0.331	0.387	-0.087	-0.031	3.59E-176	1.06E-16
cg04864179	7	128579964	*IRF5*	0.559	0.482	0.520	-0.077	-0.039	9.67E-86	4.76E-13
cg24126180	7	128580582	*IRF5*	0.277	0.208	0.248	-0.069	-0.028	4.08E-77	9.40E-07
cg15664152	7	129008407	*AHCYL2*	0.265	0.221	0.213	-0.044	-0.052	2.84E-23	1.30E-12
cg02041484	7	129266958	*NRF1*	0.409	0.308	0.377	-0.101	-0.032	1.03E-140	2.83E-10
cg19513582	7	129481640	*UBE2H*	0.561	0.465	0.536	-0.096	-0.025	1.09E-119	2.73E-06
cg13710553	7	129598625	NA	0.343	0.267	0.300	-0.076	-0.043	4.04E-114	1.29E-19
cg06917763	7	130033247	NA	0.445	0.374	0.416	-0.071	-0.029	1.65E-67	3.02E-08
cg02490034	7	130125836	*MEST*	0.429	0.370	0.402	-0.059	-0.027	7.80E-54	6.07E-07
cg22544881	7	130712346	*FLJ43663*	0.302	0.238	0.269	-0.065	-0.033	1.39E-89	8.27E-14
cg09627207	7	131244901	NA	0.722	0.773	0.765	0.051	0.043	4.07E-22	2.48E-08
cg16652347	7	132170652	*PLXNA4*	0.657	0.710	0.686	0.053	0.030	7.19E-40	3.97E-06
cg10679182	7	133225096	*EXOC4*	0.482	0.376	0.455	-0.106	-0.027	2.53E-134	2.10E-07
cg23461824	7	134605363	*CALD1*	0.716	0.770	0.752	0.054	0.035	6.69E-38	9.67E-09
cg17752799	7	136700699	*CHRM2*	0.748	0.784	0.801	0.036	0.053	5.41E-14	1.93E-13
cg22523050	7	137564212	*CREB3L2*	0.459	0.397	0.432	-0.062	-0.027	6.49E-63	7.03E-07
cg09271097	7	137620868	*CREB3L2*	0.371	0.292	0.326	-0.079	-0.044	1.43E-47	1.25E-07
cg09205945	7	137662133	*CREB3L2*	0.460	0.356	0.421	-0.104	-0.039	4.86E-109	5.62E-11
cg12924654	7	138348384	*SVOPL*	0.632	0.574	0.592	-0.058	-0.039	5.16E-57	1.48E-14
cg19972822	7	138778744	*ZC3HAV1*	0.614	0.667	0.640	0.053	0.026	3.47E-54	3.61E-07
cg01686975	7	138816336	NA	0.320	0.248	0.284	-0.072	-0.036	9.37E-95	3.62E-13
cg09848074	7	139172610	NA	0.298	0.237	0.276	-0.061	-0.022	4.17E-77	4.49E-06
cg12246945	7	139257086	*HIPK2*	0.690	0.740	0.734	0.050	0.044	2.84E-25	6.31E-09
cg10271819	7	139426030	*HIPK2*	0.387	0.302	0.349	-0.086	-0.039	1.85E-156	2.63E-22
cg18554789	7	139432178	*HIPK2*	0.361	0.264	0.322	-0.097	-0.039	5.14E-132	9.15E-14
cg12013713	7	139760671	*PARP12*	0.741	0.660	0.695	-0.081	-0.046	4.06E-57	1.25E-15
cg01003666	7	139929429	NA	0.574	0.634	0.601	0.060	0.027	4.42E-55	4.64E-07
cg01012242	7	140043078	*SLC37A3*	0.411	0.325	0.374	-0.086	-0.037	3.51E-127	1.61E-14
cg12867312	7	140454740	*BRAF*	0.750	0.794	0.803	0.044	0.053	4.95E-17	2.83E-11
cg18463686	7	141646690	*CLEC5A*	0.454	0.343	0.418	-0.111	-0.036	1.19E-137	1.85E-10
cg19735804	7	142421812	NA	0.306	0.249	0.263	-0.057	-0.042	2.38E-50	7.72E-14
cg18642720	7	142493865	NA	0.730	0.789	0.764	0.059	0.034	1.64E-50	2.19E-08
cg09704136	7	143082002	*ZYX*	0.384	0.293	0.332	-0.091	-0.052	2.10E-109	7.43E-23
cg05908241	7	143109367	*EPHA1-AS1*	0.303	0.237	0.265	-0.066	-0.038	4.63E-96	1.53E-17
cg16844053	7	148404978	*CUL1*	0.479	0.387	0.452	-0.093	-0.028	1.78E-137	4.01E-09
cg00601368	7	149318081	*ZNF767*	0.669	0.737	0.689	0.068	0.020	2.50E-105	9.72E-07
cg23903252	7	149569998	*ATP6V0E2*	0.304	0.253	0.279	-0.051	-0.025	1.43E-46	2.24E-06
cg21108554	7	150061328	NA	0.311	0.238	0.281	-0.072	-0.030	1.18E-82	2.05E-08
cg04642165	7	150063431	NA	0.367	0.290	0.333	-0.077	-0.033	1.17E-96	9.30E-11
cg18734433	7	150086169	*ZNF775*	0.448	0.394	0.426	-0.054	-0.022	1.11E-81	5.99E-10
cg03800150	7	150216489	*GIMAP7*	0.733	0.798	0.773	0.065	0.040	3.59E-72	3.07E-14
cg17807458	7	150263087	*GIMAP4*	0.776	0.826	0.824	0.050	0.048	1.32E-30	4.86E-13
cg21887193	7	150786082	*AGAP3*	0.291	0.205	0.262	-0.086	-0.029	9.95E-88	1.49E-06
cg03305017	7	151036715	NA	0.417	0.329	0.386	-0.088	-0.030	1.25E-103	3.67E-08
cg01545109	7	151087534	*WDR86*	0.324	0.264	0.299	-0.060	-0.025	3.70E-59	4.56E-07
cg07943849	7	151201868	*RHEB*	0.688	0.728	0.739	0.041	0.051	2.62E-15	2.29E-10

cg09449988	7	151328116	*PRKAG2*	0.608	0.545	0.576	-0.063	-0.032	2.97E-42	2.06E-06
cg22615330	7	151502322	*PRKAG2*	0.466	0.401	0.441	-0.065	-0.025	6.33E-67	2.20E-06
cg04058799	7	151547969	*PRKAG2*	0.605	0.545	0.571	-0.061	-0.034	9.53E-68	1.11E-13
cg03381237	7	155446645	*RBM33*	0.475	0.398	0.450	-0.077	-0.026	1.30E-91	1.33E-06
cg10702418	7	157090031	NA	0.469	0.371	0.432	-0.098	-0.036	1.31E-104	3.47E-09
cg04088945	7	157090146	NA	0.548	0.462	0.513	-0.086	-0.035	2.28E-72	4.25E-07
cg25507226	7	157190734	*DNAJB6*	0.463	0.399	0.417	-0.064	-0.046	1.58E-43	2.12E-11
cg13736939	7	157647241	*PTPRN2*	0.484	0.397	0.448	-0.086	-0.035	4.15E-99	1.23E-11
cg04598292	7	158495856	*NCAPG2*	0.305	0.246	0.275	-0.060	-0.030	7.99E-70	3.12E-10
cg10522770	7	158646253	NA	0.318	0.258	0.292	-0.061	-0.026	8.03E-79	4.39E-09
cg26246536	8	975053	NA	0.685	0.734	0.747	0.048	0.062	1.13E-17	2.68E-13
cg20533754	8	1245698	*LOC286083*	0.690	0.733	0.743	0.042	0.053	6.97E-22	1.99E-14
cg20610654	8	1708447	NA	0.693	0.755	0.722	0.062	0.029	7.73E-59	1.68E-07
cg19351604	8	1870722	*ARHGEF10*	0.534	0.411	0.477	-0.122	-0.057	1.74E-168	1.51E-26
cg14023009	8	1870798	*ARHGEF10*	0.245	0.190	0.221	-0.055	-0.024	2.12E-64	7.95E-07
cg22062741	8	1897075	*ARHGEF10*	0.512	0.412	0.475	-0.100	-0.037	1.30E-124	2.84E-12
cg20305578	8	1908301	NA	0.331	0.265	0.306	-0.066	-0.025	1.35E-69	4.90E-06
cg05888181	8	1952319	*KBTBD11*	0.462	0.363	0.427	-0.099	-0.035	2.23E-94	4.43E-08
cg20849109	8	1954777	*KBTBD11*	0.365	0.284	0.330	-0.081	-0.035	1.04E-87	5.48E-11
cg24906819	8	8231531	*SGK223*	0.475	0.372	0.437	-0.102	-0.037	1.13E-119	2.21E-11
cg04465154	8	9045558	NA	0.460	0.399	0.420	-0.061	-0.040	9.13E-37	5.49E-08
cg13601595	8	10468038	*RP1L1*	0.488	0.413	0.458	-0.074	-0.029	7.63E-80	1.83E-09
cg20009332	8	10489118	*RP1L1*	0.498	0.442	0.468	-0.056	-0.030	6.19E-34	5.72E-06
cg08265495	8	11212668	*TDH*	0.589	0.641	0.628	0.052	0.039	6.54E-34	7.83E-10
cg13008174	8	11640960	*NEIL2*	0.339	0.267	0.306	-0.072	-0.033	7.55E-148	3.28E-23
cg13007701	8	13078829	*DLC1*	0.617	0.554	0.590	-0.063	-0.027	2.60E-65	2.43E-07
cg05701478	8	19029020	NA	0.795	0.833	0.846	0.037	0.051	1.91E-14	5.13E-12
cg00158770	8	19271585	*CSGALNACT1*	0.630	0.540	0.585	-0.091	-0.045	2.78E-55	4.73E-09
cg18325192	8	19540396	*CSGALNACT1*	0.353	0.292	0.332	-0.061	-0.020	3.45E-112	2.43E-09
cg10145533	8	20350779	NA	0.750	0.817	0.778	0.067	0.028	1.01E-96	4.94E-09
cg02889774	8	21824555	*XPO7*	0.527	0.443	0.489	-0.084	-0.038	1.72E-109	1.02E-14
cg03594078	8	22131675	*PIWIL2*	0.460	0.361	0.422	-0.099	-0.038	5.61E-116	7.72E-12
cg12858593	8	22131899	*PIWIL2*	0.358	0.284	0.318	-0.073	-0.040	5.47E-73	1.90E-12
cg15942979	8	22497061	*BIN3*	0.636	0.705	0.660	0.069	0.025	5.22E-112	4.91E-09
cg09080173	8	22605334	*PEBP4*	0.275	0.216	0.243	-0.059	-0.032	2.40E-90	2.10E-14
cg18444544	8	22852741	*RHOBTB2*	0.396	0.309	0.360	-0.087	-0.036	1.68E-102	2.06E-10
cg14918744	8	23018464	*TNFRSF10D*	0.733	0.790	0.756	0.057	0.023	4.92E-91	6.59E-08
cg07892167	8	23144983	*R3HCC1*	0.230	0.177	0.202	-0.053	-0.028	3.46E-65	1.99E-09
cg04789529	8	25072319	*DOCK5*	0.468	0.374	0.423	-0.094	-0.045	2.75E-116	1.07E-19
cg22022716	8	26276541	NA	0.599	0.510	0.563	-0.089	-0.036	3.36E-114	9.68E-13
cg00122406	8	26431736	*DPYSL2*	0.250	0.193	0.225	-0.057	-0.025	4.97E-112	3.18E-14
cg16292768	8	27467783	*CLU*	0.619	0.528	0.578	-0.091	-0.040	2.89E-82	1.32E-10
cg12729838	8	27469186	*CLU*	0.334	0.265	0.307	-0.068	-0.027	2.91E-141	1.02E-16
cg13488078	8	27469338	*CLU*	0.325	0.263	0.297	-0.062	-0.028	3.00E-79	1.75E-09
cg18931633	8	27470575	*CLU*	0.500	0.442	0.456	-0.058	-0.045	4.87E-41	1.23E-12
cg13634966	8	27661589	*ESCO2*	0.743	0.792	0.799	0.048	0.055	2.12E-18	3.44E-11
cg03746834	8	28196926	*PNOC*	0.357	0.287	0.332	-0.070	-0.025	1.00E-81	1.11E-06
cg08606497	8	28244769	*ZNF395*	0.529	0.459	0.482	-0.069	-0.047	5.87E-76	1.98E-16
cg11893955	8	28918821	NA	0.685	0.759	0.715	0.074	0.029	3.04E-121	6.36E-13
cg07023317	8	28961315	*KIF13B*	0.635	0.584	0.608	-0.051	-0.027	1.04E-38	1.58E-06
cg00936790	8	29027844	*KIF13B*	0.693	0.751	0.722	0.058	0.029	1.15E-49	1.30E-06
cg06238667	8	29086832	*KIF13B*	0.693	0.763	0.723	0.070	0.030	9.63E-78	4.66E-08
cg11638399	8	29441416	NA	0.443	0.330	0.392	-0.114	-0.051	6.60E-152	1.74E-21
cg18813599	8	29937472	*MIR548O2*	0.679	0.747	0.715	0.068	0.036	2.04E-121	4.08E-23
cg10705306	8	37337009	NA	0.247	0.187	0.215	-0.060	-0.031	3.54E-81	1.83E-10
cg10557578	8	37758453	*RAB11FIP1*	0.433	0.361	0.411	-0.072	-0.023	4.87E-117	4.56E-08
cg20870000	8	38584896	*TACC1*	0.741	0.793	0.768	0.051	0.027	2.32E-71	2.17E-11
cg11068881	8	40925730	NA	0.757	0.809	0.795	0.051	0.037	3.48E-26	5.99E-07
cg13171679	8	41258634	NA	0.390	0.297	0.349	-0.093	-0.041	2.28E-134	2.41E-15
cg02707799	8	41814854	*KAT6A*	0.491	0.559	0.536	0.068	0.045	9.92E-48	3.11E-11
cg01632240	8	42009223	*AP3M2*	0.482	0.406	0.456	-0.076	-0.025	5.17E-86	1.97E-06
cg18404652	8	42009622	*AP3M2*	0.622	0.687	0.647	0.065	0.025	6.79E-90	5.32E-09
cg26243740	8	42012513	*AP3M2*	0.525	0.597	0.565	0.072	0.039	4.72E-55	3.19E-09
cg15936066	8	42356637	*SLC20A2*	0.416	0.364	0.389	-0.052	-0.027	1.08E-40	3.16E-06
cg22855020	8	42356731	*SLC20A2*	0.486	0.412	0.455	-0.075	-0.032	1.57E-56	9.65E-08
cg09642739	8	42771492	*HOOK3*	0.744	0.793	0.815	0.049	0.071	3.75E-17	1.46E-15
cg03714676	8	48174649	*KIAA0146*	0.527	0.441	0.497	-0.086	-0.030	2.65E-85	4.07E-07
cg14547461	8	48176671	*KIAA0146*	0.565	0.484	0.540	-0.082	-0.026	9.53E-79	5.06E-06
cg24163242	8	48648813	NA	0.396	0.326	0.370	-0.070	-0.026	8.05E-70	3.11E-06
cg02098075	8	49643972	*EFCAB1*	0.463	0.368	0.437	-0.095	-0.026	2.26E-111	1.45E-06
cg21285555	8	52771466	*PCMTD1*	0.483	0.380	0.443	-0.102	-0.040	3.10E-142	2.31E-14
cg12277366	8	54931229	*TCEA1*	0.705	0.764	0.731	0.060	0.026	1.62E-50	2.66E-06
cg25634507	8	59413577	*CYP7A1*	0.567	0.623	0.620	0.057	0.053	4.63E-29	3.12E-12
cg06779553	8	60020513	*TOX*	0.461	0.518	0.506	0.057	0.045	8.46E-34	3.72E-12
cg13515856	8	61240832	NA	0.637	0.690	0.672	0.052	0.035	1.74E-33	2.17E-07
cg17922998	8	61863697	NA	0.290	0.222	0.262	-0.068	-0.028	9.66E-81	4.08E-08
cg04882213	8	61880392	NA	0.322	0.236	0.276	-0.085	-0.045	3.45E-71	4.52E-10
cg07538204	8	62612575	*ASPH*	0.295	0.216	0.264	-0.079	-0.031	6.21E-96	8.81E-09

cg07118376	8	62624872	*ASPH*	0.466	0.368	0.417	-0.098	-0.049	1.13E-121	2.45E-17
cg09092280	8	62646214	NA	0.359	0.276	0.311	-0.083	-0.048	8.14E-102	2.33E-19
cg03922048	8	64085098	*YTHDF3*	0.703	0.749	0.761	0.046	0.058	3.21E-15	5.74E-11
cg00957886	8	66700638	*PDE7A*	0.745	0.799	0.800	0.054	0.055	1.61E-23	2.93E-11
cg13375690	8	66706202	*PDE7A*	0.708	0.759	0.758	0.052	0.051	3.02E-20	1.06E-08
cg14864167	8	66751182	*PDE7A*	0.651	0.513	0.569	-0.138	-0.082	3.59E-41	1.31E-18
cg20794855	8	67365340	*ADHFE1*	0.482	0.391	0.453	-0.092	-0.029	4.86E-128	2.47E-08
cg00345314	8	67624394	*SGK3*	0.463	0.392	0.422	-0.072	-0.041	5.95E-50	6.38E-09
cg07896832	8	67975874	*CSPP1*	0.484	0.378	0.449	-0.107	-0.035	4.46E-96	1.99E-07
cg21320567	8	67975880	*CSPP1*	0.503	0.397	0.463	-0.107	-0.040	8.60E-111	9.75E-11
cg13652336	8	68864012	*PREX2*	0.263	0.211	0.240	-0.052	-0.023	6.09E-61	4.91E-06
cg26228558	8	71527496	*LOC286190*	0.589	0.643	0.623	0.054	0.033	1.26E-44	1.39E-09
cg09973676	8	82006417	*PAG1*	0.338	0.459	0.394	0.121	0.056	8.00E-56	5.16E-08
cg25230327	8	82042993	NA	0.332	0.273	0.290	-0.059	-0.042	8.46E-47	2.06E-11
cg11537619	8	82359759	*PMP2*	0.714	0.764	0.755	0.051	0.042	8.15E-22	1.79E-07
cg02573176	8	82607505	*SLC10A5*	0.402	0.342	0.377	-0.060	-0.025	9.44E-63	1.18E-06
cg05071334	8	86195487	*CA13*	0.468	0.391	0.439	-0.077	-0.029	5.41E-92	4.33E-08
cg14588399	8	87242630	*SLC7A13*	0.498	0.549	0.534	0.051	0.036	8.24E-24	6.29E-07
cg23201059	8	91322832	*LINC00534*	0.751	0.804	0.800	0.053	0.049	1.84E-25	2.22E-11
cg18507018	8	92035517	*TMEM55A*	0.498	0.394	0.468	-0.104	-0.029	7.52E-102	2.07E-06
cg06567195	8	94937996	*PDP1*	0.793	0.843	0.834	0.051	0.041	8.75E-30	1.40E-09
cg13078567	8	95657036	*ESRP1*	0.758	0.806	0.810	0.048	0.051	1.65E-19	1.22E-10
cg20274462	8	95980625	NA	0.282	0.225	0.258	-0.057	-0.024	6.78E-64	2.22E-06
cg00219816	8	96280555	*C8orf37*	0.504	0.428	0.467	-0.077	-0.037	5.61E-80	1.36E-11
cg15980656	8	97583236	*SDC2*	0.439	0.355	0.407	-0.084	-0.033	2.72E-79	4.17E-08
cg07673230	8	98789233	*LAPTM4B*	0.530	0.459	0.488	-0.071	-0.043	7.53E-50	4.23E-09
cg12861602	8	99045811	*MATN2*	0.648	0.690	0.709	0.042	0.060	1.73E-13	2.74E-12
cg09461494	8	100262768	*VPS13B*	0.725	0.776	0.783	0.051	0.058	2.56E-20	6.85E-12
cg26192520	8	101960390	*YWHAZ*	0.626	0.701	0.681	0.075	0.055	6.52E-48	3.89E-13
cg20162696	8	102167694	NA	0.322	0.245	0.290	-0.077	-0.032	1.05E-90	1.62E-09
cg11849798	8	102209920	*ZNF706*	0.395	0.314	0.351	-0.081	-0.044	3.43E-98	1.37E-16
cg20611272	8	103548145	NA	0.651	0.742	0.687	0.091	0.036	1.67E-171	1.86E-17
cg03752203	8	103874399	*AZIN1*	0.660	0.717	0.698	0.058	0.038	6.60E-51	1.17E-10
cg10011091	8	104424477	*SLC25A32*	0.406	0.301	0.357	-0.105	-0.050	2.04E-132	3.26E-20
cg08826743	8	105392295	*DPYS*	0.661	0.724	0.703	0.063	0.041	2.36E-39	3.75E-09
cg11091914	8	105598224	*LRP12*	0.308	0.247	0.283	-0.060	-0.024	1.92E-60	6.06E-06
cg20788020	8	105677501	NA	0.676	0.731	0.714	0.055	0.038	5.41E-51	3.81E-13
cg17679987	8	106535882	*ZFPM2*	0.707	0.764	0.728	0.057	0.021	7.45E-77	2.03E-06
cg03503046	8	106595191	*ZFPM2*	0.700	0.752	0.741	0.053	0.042	2.04E-33	2.92E-10
cg05783185	8	119121281	*EXT1*	0.377	0.303	0.345	-0.074	-0.032	2.13E-75	6.98E-08
cg06173216	8	120219151	*MAL2*	0.733	0.783	0.768	0.050	0.035	1.20E-35	2.13E-10
cg15447971	8	121270185	*COL14A1*	0.685	0.728	0.737	0.043	0.052	1.13E-13	3.78E-09
cg05127574	8	121714454	*SNTB1*	0.782	0.838	0.811	0.056	0.029	3.57E-61	1.05E-08
cg13074203	8	121798364	*SNTB1*	0.377	0.308	0.342	-0.069	-0.035	1.88E-69	3.93E-09
cg08598221	8	121824929	*SNTB1*	0.555	0.483	0.513	-0.072	-0.042	1.14E-71	9.06E-15
cg23051091	8	122001888	NA	0.753	0.804	0.787	0.050	0.034	5.19E-32	4.17E-08
cg00692752	8	123086057	NA	0.699	0.748	0.751	0.050	0.053	7.89E-24	4.96E-12
cg15531512	8	123706630	NA	0.472	0.399	0.440	-0.073	-0.032	2.34E-57	7.88E-07
cg05753328	8	124171421	NA	0.558	0.492	0.516	-0.066	-0.042	1.14E-35	9.90E-09
cg12002745	8	124179875	NA	0.307	0.240	0.270	-0.067	-0.037	3.12E-110	3.21E-22
cg04724477	8	124780013	*FAM91A1*	0.506	0.453	0.465	-0.053	-0.041	1.86E-38	9.63E-11
cg15173586	8	124784802	*FAM91A1*	0.511	0.562	0.552	0.051	0.041	1.47E-25	1.15E-09
cg20364222	8	125259763	NA	0.309	0.257	0.280	-0.051	-0.029	4.89E-47	7.84E-08
cg26780125	8	125388806	NA	0.377	0.285	0.338	-0.092	-0.039	4.93E-114	1.91E-11
cg16657152	8	125784769	NA	0.406	0.309	0.359	-0.097	-0.047	6.92E-122	1.57E-16
cg26821681	8	125827396	NA	0.342	0.282	0.306	-0.060	-0.035	1.19E-33	2.07E-07
cg09105249	8	126366660	*NSMCE2*	0.765	0.817	0.812	0.052	0.047	7.92E-23	9.56E-09
cg19089701	8	126398731	NA	0.304	0.239	0.279	-0.065	-0.025	3.55E-66	3.95E-06
cg00207280	8	126441393	*TRIB1*	0.283	0.225	0.256	-0.058	-0.027	5.22E-75	4.02E-09
cg25684151	8	126588554	NA	0.441	0.346	0.404	-0.095	-0.037	9.39E-138	2.33E-13
cg01495299	8	128003923	NA	0.295	0.243	0.265	-0.052	-0.029	8.17E-53	2.70E-09
cg12616923	8	128228107	NA	0.339	0.277	0.312	-0.062	-0.026	1.74E-60	5.73E-08
cg14752089	8	128773042	NA	0.311	0.237	0.276	-0.074	-0.035	4.81E-110	3.83E-16
cg20516032	8	128994030	*PVT1*	0.747	0.802	0.772	0.055	0.025	1.66E-79	2.96E-10
cg07305933	8	129079496	*PVT1*	0.423	0.328	0.386	-0.095	-0.037	5.55E-132	1.20E-12
cg08853735	8	129139526	NA	0.692	0.751	0.733	0.059	0.042	8.01E-36	1.46E-08
cg00444740	8	129162178	NA	0.411	0.319	0.375	-0.092	-0.035	2.71E-110	2.30E-10
cg17477578	8	129552914	NA	0.731	0.796	0.762	0.065	0.031	7.74E-77	6.43E-09
cg25760227	8	130691556	NA	0.414	0.335	0.366	-0.079	-0.047	8.18E-91	1.91E-17
cg17951713	8	130698161	NA	0.408	0.316	0.369	-0.091	-0.038	2.13E-97	1.79E-09
cg19772620	8	130861109	*FAM49B*	0.642	0.675	0.694	0.032	0.051	7.64E-12	6.92E-12
cg19123356	8	130898833	*FAM49B*	0.615	0.706	0.647	0.091	0.032	1.92E-162	5.00E-15
cg20612299	8	131308633	*ASAP1*	0.447	0.352	0.403	-0.095	-0.045	2.56E-141	6.16E-20
cg27627524	8	131325625	*ASAP1*	0.418	0.314	0.372	-0.104	-0.046	2.13E-141	1.86E-16
cg09376835	8	131347294	*ASAP1*	0.393	0.297	0.339	-0.096	-0.054	4.61E-139	2.18E-24
cg17090901	8	133837475	*PHF20L1*	0.755	0.807	0.803	0.052	0.048	2.29E-29	3.07E-12
cg00799984	8	134307105	*NDRG1*	0.479	0.409	0.459	-0.070	-0.020	9.52E-128	1.42E-08
cg14499058	8	141129321	*TRAPPC9*	0.417	0.344	0.366	-0.073	-0.051	1.16E-70	1.47E-18

cg11006453	8	141599185	*EIF2C2*	0.724	0.783	0.749	0.059	0.026	8.05E-86	9.51E-10
cg23731089	8	141599208	*EIF2C2*	0.764	0.822	0.784	0.058	0.021	4.50E-83	4.64E-06
cg19352830	8	141599356	*EIF2C2*	0.765	0.815	0.786	0.050	0.021	3.85E-67	2.42E-07
cg07891862	8	141774605	*PTK2*	0.756	0.811	0.809	0.055	0.053	4.58E-32	3.94E-14
cg00444581	8	141856401	*PTK2*	0.765	0.824	0.819	0.060	0.054	4.85E-26	5.56E-11
cg12809098	8	142165310	*DENND3*	0.612	0.500	0.584	-0.111	-0.028	4.94E-122	3.24E-06
cg15891546	8	142179908	*DENND3*	0.612	0.501	0.570	-0.111	-0.042	6.36E-90	2.24E-08
cg09859659	8	142180109	*DENND3*	0.626	0.520	0.590	-0.106	-0.036	2.97E-119	3.59E-10
cg07231045	8	142219444	NA	0.523	0.414	0.478	-0.109	-0.045	2.81E-110	1.32E-11
cg15586392	8	142238691	*SLC45A4*	0.539	0.445	0.500	-0.094	-0.039	2.97E-99	2.74E-10
cg01081737	8	142238752	*SLC45A4*	0.546	0.448	0.503	-0.098	-0.042	6.85E-117	1.50E-14
cg07769015	8	142238770	*SLC45A4*	0.565	0.482	0.534	-0.083	-0.031	2.59E-87	1.65E-08
cg03316587	8	142245164	NA	0.360	0.291	0.332	-0.069	-0.028	6.73E-73	5.50E-07
cg12110437	8	144098888	*LOC1001336*	0.417	0.340	0.364	-0.077	-0.053	4.86E-31	1.10E-08
cg17052170	8	144099482	*LOC1001336*	0.637	0.503	0.570	-0.134	-0.067	1.50E-58	5.36E-11
cg11702942	8	144102584	*LY6E*	0.826	0.771	0.800	-0.055	-0.025	9.29E-37	2.01E-11
cg08170427	8	144410815	*TOP1MT*	0.461	0.392	0.424	-0.068	-0.037	9.10E-57	1.53E-08
cg17117981	8	144410972	*TOP1MT*	0.561	0.501	0.539	-0.060	-0.022	2.73E-60	2.80E-06
cg17159187	8	144420118	*TOP1MT*	0.466	0.371	0.430	-0.095	-0.035	8.56E-135	1.98E-13
cg11284147	8	144437914	*TOP1MT*	0.416	0.342	0.383	-0.073	-0.033	1.70E-56	1.03E-06
cg22312904	8	144639260	*GSDMD*	0.490	0.427	0.466	-0.063	-0.024	5.01E-60	3.82E-06
cg24351694	8	144655447	*C8orf73*	0.292	0.237	0.267	-0.055	-0.025	1.33E-75	7.19E-09
cg01048372	8	144655484	*C8orf73*	0.438	0.360	0.411	-0.078	-0.026	1.35E-142	1.24E-13
cg20127859	8	144656271	*C8orf73*	0.280	0.216	0.258	-0.064	-0.022	8.76E-120	3.78E-09
cg13509702	8	144896307	*SCRIB*	0.495	0.423	0.463	-0.071	-0.032	1.25E-78	1.19E-10
cg04757492	8	145003862	*PLEC*	0.623	0.693	0.664	0.070	0.041	2.61E-35	4.14E-06
cg03280622	8	145023013	*PLEC*	0.346	0.271	0.304	-0.075	-0.042	1.33E-137	6.81E-25
cg00311984	8	145051668	*PLEC*	0.544	0.598	0.573	0.054	0.028	1.07E-35	2.24E-06
cg05451722	8	145065385	*GRINA*	0.306	0.250	0.282	-0.056	-0.024	1.28E-58	2.27E-06
cg27263049	8	145086300	*SPATC1*	0.245	0.194	0.213	-0.051	-0.033	2.02E-51	1.10E-10
cg24710655	8	145086714	*SPATC1*	0.281	0.219	0.257	-0.062	-0.024	3.82E-84	2.15E-08
cg05494008	8	145579285	*TMEM249*	0.280	0.229	0.255	-0.051	-0.025	7.31E-77	1.60E-11
cg04943741	8	145651369	*VPS28*	0.552	0.481	0.530	-0.072	-0.022	1.17E-98	2.45E-07
cg10639435	8	146104221	*ZNF250*	0.437	0.494	0.458	0.058	0.021	9.61E-55	1.95E-06
cg14080050	9	33159025	*B4GALT1*	0.249	0.192	0.221	-0.058	-0.028	2.23E-83	1.67E-12
cg01004762	9	34956991	*KIAA1045*	0.482	0.427	0.451	-0.056	-0.031	1.24E-48	1.77E-08
cg13534791	9	35042344	*C9orf131*	0.409	0.341	0.384	-0.068	-0.026	3.05E-138	1.29E-15
cg05000446	9	35042395	*C9orf131*	0.553	0.423	0.510	-0.129	-0.043	3.19E-156	9.61E-13
cg12971694	9	35618412	*CD72*	0.343	0.266	0.307	-0.077	-0.036	8.98E-124	4.00E-19
cg13876315	9	35650526	*SIT1*	0.691	0.749	0.715	0.058	0.024	2.76E-97	6.02E-11
cg15518883	9	35650561	*SIT1*	0.712	0.776	0.741	0.064	0.029	1.46E-98	5.30E-13
cg11825899	9	35844849	*TMEM8B*	0.508	0.435	0.462	-0.073	-0.046	1.12E-65	1.86E-13
cg15248035	9	36169949	*CCIN*	0.460	0.351	0.415	-0.109	-0.045	1.70E-126	1.35E-15
cg13630493	9	36190154	*CLTA*	0.297	0.238	0.268	-0.059	-0.030	1.08E-61	1.51E-08
cg13422921	9	37788648	NA	0.482	0.395	0.437	-0.086	-0.045	1.07E-77	1.80E-11
cg14397918	9	72078829	*APBA1*	0.411	0.312	0.369	-0.099	-0.042	5.33E-145	6.54E-16
cg21171320	9	73178360	*TRPM3*	0.365	0.287	0.311	-0.078	-0.054	2.03E-94	2.85E-24
cg14565465	9	74298933	*TMEM2*	0.368	0.295	0.341	-0.073	-0.028	3.58E-89	1.33E-07
cg07194321	9	74524266	*FAM108B1*	0.234	0.175	0.195	-0.059	-0.039	4.21E-67	1.90E-14
cg13591783	9	75768868	*ANXA1*	0.247	0.194	0.209	-0.053	-0.038	1.29E-43	1.85E-10
cg16509045	9	77502963	*TRPM6*	0.489	0.389	0.452	-0.100	-0.037	2.54E-123	6.95E-11
cg13765061	9	78830510	*PCSK5*	0.370	0.296	0.340	-0.075	-0.031	1.81E-73	3.90E-07
cg14459032	9	78905335	*PCSK5*	0.491	0.432	0.468	-0.059	-0.023	1.37E-62	1.51E-06
cg13740985	9	80930413	*PSAT1*	0.372	0.290	0.339	-0.082	-0.033	2.31E-89	3.78E-08
cg13895650	9	84228185	*TLE1*	0.629	0.559	0.600	-0.070	-0.030	2.35E-58	2.83E-07
cg13959031	9	88896340	*ISCA1*	0.648	0.596	0.618	-0.052	-0.029	1.44E-37	1.04E-06
cg14419046	9	90043268	NA	0.438	0.358	0.413	-0.080	-0.025	1.67E-89	1.81E-06
cg14531665	9	91058614	*SPIN1*	0.430	0.363	0.413	-0.066	-0.017	6.03E-117	1.29E-07
cg13554018	9	91091556	*SPIN1*	0.647	0.720	0.677	0.073	0.030	5.76E-102	4.95E-12
cg14054883	9	93619467	*SYK*	0.749	0.805	0.777	0.056	0.028	7.16E-60	4.90E-08
cg13932501	9	94060853	*AUH*	0.537	0.459	0.505	-0.078	-0.032	1.05E-80	2.51E-09
cg14290576	9	94181612	*NFIL3*	0.395	0.305	0.348	-0.090	-0.047	1.70E-134	2.19E-21
cg13918640	9	94351568	NA	0.450	0.355	0.406	-0.095	-0.044	1.02E-86	2.71E-11
cg13819552	9	95799870	NA	0.398	0.332	0.369	-0.065	-0.028	3.28E-110	5.59E-15
cg06705237	9	97402555	*FBP1*	0.581	0.529	0.553	-0.052	-0.028	5.21E-38	8.04E-07
cg13573745	9	97405553	NA	0.360	0.275	0.320	-0.085	-0.040	1.25E-98	2.16E-13
cg21234955	9	97713896	*C9orf3*	0.690	0.758	0.718	0.068	0.028	1.32E-62	3.60E-06
cg14172094	9	100394654	*NCBP1*	0.267	0.213	0.234	-0.054	-0.033	3.43E-48	2.28E-08
cg11317199	9	100850391	*TRIM14*	0.591	0.678	0.664	0.087	0.073	1.54E-33	1.13E-15
cg01765174	9	100880960	*TRIM14*	0.497	0.449	0.446	-0.048	-0.051	1.75E-26	3.01E-15
cg13709496	9	101011718	*TBC1D2*	0.407	0.317	0.373	-0.090	-0.034	4.77E-151	7.68E-17
cg14426785	9	101876671	*TGFBR1*	0.409	0.353	0.376	-0.057	-0.033	3.77E-48	1.69E-08
cg14085060	9	107827215	NA	0.303	0.238	0.274	-0.066	-0.030	6.08E-116	5.32E-17
cg14620234	9	108003927	NA	0.655	0.707	0.687	0.052	0.032	1.44E-43	7.10E-09
cg13603332	9	108053809	*SLC44A1*	0.423	0.321	0.391	-0.102	-0.032	3.98E-127	4.46E-09
cg14057303	9	109683834	*ZNF462*	0.642	0.717	0.667	0.075	0.025	2.37E-130	2.58E-09
cg13535736	9	111863775	*TMEM245*	0.582	0.663	0.633	0.080	0.050	6.55E-59	6.04E-12
cg13661827	9	111885602	NA	0.510	0.431	0.471	-0.079	-0.038	1.18E-63	1.17E-08

cg13633625	9	114706971	NA	0.530	0.401	0.485	-0.129	-0.046	4.22E-180	2.17E-16
cg13999433	9	117156883	*AKNA*	0.488	0.380	0.451	-0.107	-0.036	2.80E-136	2.04E-10
cg14230719	9	123492289	NA	0.279	0.219	0.250	-0.061	-0.029	1.18E-83	2.39E-12
cg15551881	9	123688715	*TRAF1*	0.303	0.365	0.347	0.062	0.045	2.25E-37	3.19E-12
cg21492378	9	123850279	*CNTRL*	0.500	0.388	0.465	-0.113	-0.036	6.82E-126	9.19E-09
cg13584244	9	124030048	*GSN*	0.406	0.339	0.380	-0.067	-0.026	4.51E-86	6.85E-08
cg14186336	9	124045139	*GSN*	0.438	0.351	0.393	-0.087	-0.045	1.70E-130	3.97E-19
cg14399183	9	124048308	*GSN*	0.291	0.218	0.264	-0.073	-0.027	1.72E-113	6.63E-10
cg13569051	9	124051703	*GSN*	0.526	0.443	0.498	-0.083	-0.028	2.39E-97	1.75E-07
cg14115756	9	125795935	*GPR21*	0.371	0.283	0.336	-0.088	-0.035	2.95E-108	7.78E-10
cg14584535	9	125797187	*GPR21*	0.449	0.359	0.407	-0.090	-0.042	2.28E-94	4.64E-12
cg14569423	9	126523663	*DENND1A*	0.710	0.750	0.760	0.040	0.050	1.47E-13	1.16E-09
cg13487666	9	127021157	*NEK6*	0.256	0.204	0.233	-0.052	-0.023	5.18E-58	9.12E-07
cg13753351	9	127134207	*PSMB7*	0.464	0.347	0.419	-0.116	-0.045	1.42E-154	1.19E-15
cg11557901	9	128022358	NA	0.501	0.377	0.462	-0.123	-0.039	1.27E-133	5.29E-10
cg02677635	9	129099507	*FAM125B*	0.448	0.343	0.410	-0.105	-0.038	8.58E-118	3.24E-10
cg13618969	9	129184186	*FAM125B*	0.611	0.494	0.584	-0.117	-0.027	6.21E-131	1.58E-06
cg14369981	9	129294452	NA	0.438	0.356	0.417	-0.083	-0.021	5.24E-125	2.07E-07
cg13916080	9	129624994	*ZBTB34*	0.336	0.247	0.304	-0.089	-0.032	8.17E-131	1.08E-11
cg13595556	9	129648322	NA	0.546	0.424	0.510	-0.122	-0.037	2.18E-165	6.61E-12
cg14413165	9	130524573	*SH2D3C*	0.348	0.297	0.331	-0.051	-0.018	6.04E-108	1.62E-09
cg13599613	9	130524902	*SH2D3C*	0.557	0.439	0.527	-0.118	-0.030	1.29E-131	1.38E-07
cg00188748	9	130564509	*FPGS*	0.405	0.327	0.363	-0.077	-0.042	2.26E-68	6.54E-10
cg01937819	9	130616110	*ENG*	0.250	0.194	0.217	-0.056	-0.033	8.02E-77	4.38E-13
cg13914004	9	130659670	*ST6GALNAC6*	0.295	0.239	0.273	-0.056	-0.022	1.97E-65	3.24E-06
cg14586500	9	130697656	*DPM2*	0.342	0.262	0.298	-0.081	-0.045	1.61E-91	1.22E-14
cg14522718	9	130868874	*SLC25A25*	0.744	0.799	0.780	0.056	0.036	3.26E-49	1.29E-09
cg13583162	9	131545865	NA	0.399	0.346	0.377	-0.053	-0.023	9.85E-63	9.40E-07
cg00118808	9	132097931	NA	0.341	0.260	0.306	-0.081	-0.035	2.52E-153	2.87E-21
cg13537102	9	132360781	NA	0.520	0.461	0.496	-0.059	-0.024	1.10E-73	7.30E-08
cg14288424	9	132429922	*PRRX2*	0.391	0.333	0.364	-0.058	-0.027	4.97E-102	1.66E-14
cg21228270	9	133871033	NA	0.325	0.261	0.295	-0.064	-0.030	5.40E-92	5.63E-13
cg14611767	9	134127575	NA	0.539	0.433	0.499	-0.106	-0.040	3.52E-120	1.88E-12
cg14468508	9	134304802	*PRRC2B*	0.725	0.765	0.784	0.041	0.059	9.72E-16	1.89E-14
cg14560703	9	134585592	*RAPGEF1*	0.552	0.627	0.599	0.075	0.047	5.27E-52	3.89E-10
cg13714407	9	134604212	*RAPGEF1*	0.747	0.802	0.778	0.055	0.031	1.57E-37	1.06E-06
cg14484681	9	135158694	*SETX*	0.741	0.792	0.791	0.051	0.049	2.65E-28	6.94E-14
cg14550760	9	135936732	*CEL*	0.426	0.372	0.398	-0.054	-0.028	2.87E-59	7.23E-08
cg14018735	9	136270734	*C9orf96*	0.459	0.388	0.423	-0.071	-0.036	2.74E-94	4.56E-14
cg00411595	9	136339349	*SLC2A6*	0.296	0.240	0.272	-0.055	-0.024	2.76E-92	1.36E-09
cg13410614	9	136341915	*SLC2A6*	0.411	0.308	0.363	-0.102	-0.048	6.80E-133	9.08E-18
cg02127980	9	137252116	*RXRA*	0.432	0.367	0.406	-0.065	-0.025	1.89E-75	4.58E-08
cg14236758	9	137252129	*RXRA*	0.494	0.430	0.467	-0.064	-0.027	8.03E-77	5.28E-10
cg13413384	9	137302231	*RXRA*	0.445	0.374	0.423	-0.071	-0.022	2.16E-136	1.01E-10
cg14479377	9	138742730	*CAMSAP1*	0.640	0.712	0.688	0.072	0.049	4.81E-47	2.84E-10
cg13594542	9	138850547	*UBAC1*	0.331	0.264	0.309	-0.067	-0.021	5.11E-95	1.14E-06
cg13692739	9	139005232	NA	0.357	0.413	0.385	0.056	0.028	6.24E-40	2.74E-06
cg08100069	9	139014709	NA	0.453	0.402	0.423	-0.051	-0.030	2.32E-31	4.21E-07
cg13807386	9	139098502	*QSOX2*	0.403	0.325	0.374	-0.079	-0.029	8.88E-119	1.33E-12
cg13683534	9	139221076	*DKFZP434A0*	0.339	0.268	0.302	-0.071	-0.037	1.58E-129	4.79E-21
cg14225168	9	139403827	*NOTCH1*	0.455	0.390	0.427	-0.065	-0.028	1.03E-54	4.19E-06
cg00407944	9	139424152	*NOTCH1*	0.322	0.256	0.305	-0.066	-0.017	1.34E-131	6.72E-07
cg14464245	9	139580482	*AGPAT2*	0.530	0.463	0.490	-0.068	-0.041	2.42E-85	6.71E-17
cg13565499	9	139592589	NA	0.322	0.243	0.276	-0.079	-0.046	2.11E-88	2.02E-14
cg14333394	9	139812357	*TRAF2*	0.450	0.381	0.431	-0.068	-0.019	1.88E-130	1.49E-09
cg02679745	9	139927646	*FUT7*	0.309	0.245	0.276	-0.064	-0.033	2.12E-131	3.64E-22
cg01544270	9	140300227	*EXD3*	0.351	0.282	0.324	-0.069	-0.027	1.00E-108	2.86E-11
cg14135809	9	140502926	*ARRDC1*	0.374	0.300	0.346	-0.073	-0.028	3.30E-118	6.90E-12
cg01595717	9	140586201	*EHMT1*	0.224	0.170	0.203	-0.055	-0.021	3.31E-84	7.64E-08
cg23128584	10	323649	*DIP2C*	0.465	0.375	0.428	-0.090	-0.036	8.86E-87	2.10E-09
cg19334176	10	443029	*DIP2C*	0.464	0.393	0.427	-0.071	-0.036	8.80E-57	5.03E-09
cg15986644	10	516683	*DIP2C*	0.819	0.871	0.838	0.052	0.019	2.85E-90	5.28E-07
cg17433678	10	3135764	*PFKP*	0.390	0.317	0.350	-0.073	-0.040	1.26E-67	2.72E-11
cg26644674	10	3138505	*PFKP*	0.470	0.379	0.430	-0.092	-0.040	2.54E-64	2.87E-07
cg09455513	10	3792283	NA	0.363	0.279	0.327	-0.085	-0.036	3.88E-114	1.47E-13
cg24420366	10	3938541	NA	0.530	0.408	0.479	-0.121	-0.050	6.17E-177	1.89E-20
cg25597390	10	5333615	NA	0.260	0.205	0.230	-0.055	-0.030	1.99E-49	1.69E-07
cg20636714	10	5476521	*NET1*	0.636	0.704	0.683	0.068	0.047	8.03E-65	3.64E-16
cg27021181	10	5488346	*NET1*	0.565	0.514	0.525	-0.052	-0.040	1.74E-26	3.04E-08
cg26105232	10	6105656	*IL2RA*	0.453	0.376	0.423	-0.077	-0.030	4.04E-62	5.89E-06
cg18100746	10	6390958	NA	0.717	0.770	0.760	0.053	0.043	1.18E-26	9.74E-09
cg08243626	10	6442501	NA	0.416	0.309	0.363	-0.107	-0.053	3.19E-127	7.61E-18
cg05103064	10	7311500	*SFMBT2*	0.782	0.842	0.809	0.059	0.027	4.26E-103	9.35E-14
cg05008854	10	7825899	*KIN*	0.468	0.380	0.433	-0.089	-0.035	5.49E-134	6.97E-13
cg25865570	10	11046383	*CELF2*	0.745	0.793	0.806	0.047	0.061	9.03E-18	5.60E-12
cg19956889	10	11650681	*USP6NL*	0.735	0.787	0.785	0.052	0.049	1.19E-31	2.96E-13
cg03412153	10	13139217	NA	0.682	0.745	0.705	0.063	0.023	1.75E-100	1.98E-08
cg04221681	10	13337496	*PHYH*	0.756	0.803	0.806	0.047	0.050	2.11E-19	1.59E-10

cg19018267	10	13341332	*PHYH*	0.342	0.280	0.311	-0.062	-0.031	5.40E-44	1.07E-06
cg18306106	10	13350494	NA	0.741	0.791	0.784	0.050	0.043	1.03E-22	8.05E-09
cg14152591	10	14587920	*FAM107B*	0.461	0.406	0.432	-0.055	-0.028	8.41E-40	1.16E-06
cg06752040	10	14647373	*FAM107B*	0.617	0.676	0.646	0.059	0.029	1.58E-56	5.51E-08
cg23379806	10	14650660	*FAM107B*	0.321	0.250	0.285	-0.071	-0.036	1.42E-117	1.46E-19
cg26106166	10	16821670	*RSU1*	0.606	0.703	0.666	0.097	0.060	9.06E-96	1.32E-21
cg25469714	10	16949821	*CUBN*	0.543	0.483	0.510	-0.060	-0.033	4.16E-45	2.29E-08
cg07732336	10	17038941	*CUBN*	0.399	0.334	0.358	-0.064	-0.041	1.69E-51	3.53E-11
cg26063719	10	17273187	*VIM*	0.333	0.273	0.293	-0.059	-0.039	7.62E-54	1.52E-12
cg01816726	10	17496989	*ST8SIA6*	0.261	0.203	0.231	-0.058	-0.030	1.06E-60	1.76E-07
cg16929850	10	18989097	NA	0.686	0.741	0.718	0.054	0.031	5.23E-53	4.90E-10
cg20569108	10	21822303	*MLLT10*	0.322	0.243	0.293	-0.079	-0.029	6.48E-86	5.46E-07
cg23087877	10	22022646	*MLLT10*	0.749	0.796	0.806	0.047	0.057	4.20E-17	9.01E-12
cg14728071	10	22030034	*MLLT10*	0.771	0.822	0.797	0.051	0.027	1.59E-58	8.74E-08
cg19378631	10	22606072	*COMMD3*	0.253	0.188	0.228	-0.065	-0.024	1.55E-74	7.61E-07
cg10763374	10	22608858	*COMMD3*	0.361	0.298	0.322	-0.063	-0.039	2.68E-70	8.44E-15
cg26511108	10	22608951	*COMMD3*	0.282	0.219	0.255	-0.063	-0.028	9.47E-68	2.17E-07
cg16152753	10	23732088	NA	0.362	0.306	0.337	-0.056	-0.026	1.72E-48	6.23E-06
cg16766382	10	28588024	NA	0.633	0.700	0.658	0.068	0.025	1.05E-64	5.16E-06
cg11316887	10	29924694	*SVIL*	0.746	0.813	0.772	0.067	0.026	3.38E-113	5.04E-11
cg11915901	10	31074432	NA	0.276	0.220	0.257	-0.057	-0.019	6.66E-83	4.47E-06
cg08764162	10	31147088	*ZNF438*	0.489	0.365	0.431	-0.124	-0.058	6.46E-177	4.62E-25
cg25888881	10	31288287	*ZNF438*	0.420	0.334	0.376	-0.086	-0.044	3.23E-123	1.49E-20
cg16480008	10	31288991	*ZNF438*	0.775	0.814	0.827	0.039	0.052	4.21E-14	9.47E-12
cg26443127	10	31987230	NA	0.453	0.362	0.418	-0.091	-0.035	8.06E-166	3.53E-19
cg26300461	10	32401032	NA	0.741	0.794	0.782	0.053	0.041	5.70E-25	1.13E-07
cg25179876	10	33483109	*NRP1*	0.375	0.296	0.335	-0.079	-0.040	1.10E-89	3.57E-13
cg24892069	10	33562205	*NRP1*	0.616	0.572	0.561	-0.044	-0.055	1.61E-11	8.14E-09
cg11147278	10	34062234	NA	0.303	0.226	0.272	-0.077	-0.030	5.93E-92	4.29E-08
cg17679427	10	35480749	*CREM*	0.408	0.329	0.370	-0.079	-0.039	2.70E-89	4.01E-13
cg11411220	10	35630062	*CCNY*	0.386	0.313	0.356	-0.073	-0.029	3.05E-88	7.65E-10
cg23856138	10	35658321	*CCNY*	0.256	0.192	0.231	-0.064	-0.025	2.23E-79	1.04E-06
cg17262810	10	35676874	*CCNY*	0.468	0.374	0.436	-0.094	-0.032	1.62E-104	4.86E-08
cg00201133	10	35740216	*CCNY*	0.380	0.286	0.328	-0.094	-0.052	1.55E-118	2.13E-23
cg01082910	10	36053547	NA	0.466	0.508	0.519	0.042	0.053	4.59E-15	3.87E-11
cg25596082	10	43626561	NA	0.268	0.216	0.246	-0.052	-0.022	5.26E-54	4.61E-06
cg23568192	10	44289380	NA	0.490	0.387	0.461	-0.103	-0.029	1.10E-101	5.56E-06
cg26888227	10	45697308	NA	0.494	0.552	0.553	0.058	0.058	3.37E-22	4.72E-11
cg23054840	10	45920485	*ALOX5*	0.384	0.303	0.347	-0.081	-0.037	6.56E-97	1.10E-12
cg23260111	10	46158095	*ZFAND4*	0.705	0.764	0.753	0.059	0.049	4.29E-32	2.28E-10
cg05880248	10	48432664	*GDF10*	0.315	0.257	0.289	-0.058	-0.027	8.78E-108	1.38E-13
cg25467634	10	49684658	*ARHGAP22*	0.286	0.231	0.260	-0.055	-0.026	2.64E-89	9.88E-13
cg11095743	10	49815243	*ARHGAP22*	0.293	0.230	0.247	-0.063	-0.046	8.92E-81	2.63E-23
cg07345108	10	49893463	*WDFY4*	0.245	0.194	0.207	-0.051	-0.038	6.95E-71	4.88E-21
cg07472373	10	50595181	*DRGX*	0.507	0.376	0.457	-0.130	-0.050	1.00E-167	5.58E-17
cg06801028	10	51575592	*NCOA4*	0.398	0.307	0.363	-0.091	-0.035	1.21E-122	1.61E-11
cg16814786	10	51575763	*NCOA4*	0.401	0.309	0.374	-0.092	-0.027	1.68E-101	1.97E-06
cg22582187	10	63394414	NA	0.539	0.592	0.590	0.053	0.051	1.08E-18	3.41E-09
cg18522931	10	63776828	*ARID5B*	0.362	0.281	0.317	-0.081	-0.045	8.97E-87	6.09E-14
cg00928816	10	63809098	*ARID5B*	0.739	0.796	0.763	0.057	0.024	4.50E-72	4.58E-08
cg16389209	10	63809121	*ARID5B*	0.674	0.745	0.701	0.070	0.027	1.40E-91	4.67E-10
cg20339443	10	65934509	NA	0.735	0.791	0.772	0.056	0.036	1.27E-34	1.36E-07
cg04100124	10	69611755	NA	0.460	0.365	0.429	-0.095	-0.032	1.37E-107	2.59E-07
cg27009815	10	69988445	NA	0.742	0.793	0.783	0.052	0.041	1.90E-22	1.15E-07
cg26026821	10	70712079	NA	0.699	0.742	0.750	0.044	0.051	1.14E-14	1.04E-09
cg06748146	10	71104724	*HK1*	0.703	0.758	0.729	0.055	0.026	1.67E-59	4.35E-07
cg04859918	10	71893017	*AIFM2*	0.497	0.436	0.469	-0.060	-0.028	9.29E-76	1.03E-09
cg14260169	10	72344881	NA	0.352	0.271	0.308	-0.081	-0.044	2.24E-65	1.06E-09
cg09914304	10	72362292	*PRF1*	0.552	0.606	0.574	0.054	0.022	2.18E-88	5.52E-10
cg12433559	10	72362730	*PRF1*	0.634	0.712	0.667	0.078	0.033	1.87E-176	8.44E-23
cg02374486	10	72362809	*PRF1*	0.639	0.725	0.674	0.086	0.035	1.21E-125	3.72E-13
cg15293582	10	72362866	*PRF1*	0.690	0.756	0.711	0.066	0.021	2.07E-107	1.52E-06
cg08874645	10	73093425	*SLC29A3*	0.287	0.234	0.272	-0.053	-0.015	2.11E-90	5.96E-06
cg04005943	10	73350048	*CDH23*	0.308	0.246	0.264	-0.062	-0.044	2.25E-61	1.11E-15
cg23056923	10	73497629	*C10orf105*	0.442	0.373	0.410	-0.069	-0.032	5.57E-144	1.38E-20
cg04304802	10	73499965	*CDH23*	0.275	0.207	0.241	-0.068	-0.035	1.54E-91	2.94E-12
cg14614581	10	73844130	*SPOCK2*	0.733	0.804	0.765	0.071	0.032	1.25E-127	4.20E-16
cg23723835	10	73869442	*ASCC1*	0.742	0.796	0.780	0.054	0.038	4.99E-40	1.70E-10
cg07904073	10	74017122	NA	0.280	0.208	0.247	-0.072	-0.033	3.73E-74	1.53E-09
cg04858631	10	74035570	*DDIT4*	0.575	0.636	0.618	0.061	0.043	2.33E-48	1.62E-12
cg20080702	10	74046947	NA	0.593	0.539	0.566	-0.054	-0.027	3.87E-49	8.03E-08
cg05988358	10	74082489	NA	0.652	0.709	0.685	0.057	0.032	3.72E-117	5.37E-23
cg16617349	10	74337803	*MICU1*	0.724	0.772	0.775	0.048	0.052	1.06E-22	1.77E-11
cg09553839	10	74358778	*MICU1*	0.517	0.415	0.470	-0.103	-0.048	5.52E-136	3.95E-21
cg27592794	10	74454766	*MCU*	0.284	0.216	0.258	-0.068	-0.026	1.14E-91	3.82E-09
cg04389994	10	74590936	*MCU*	0.629	0.551	0.592	-0.077	-0.036	6.94E-78	3.95E-09
cg22964146	10	74767376	*P4HA1*	0.740	0.794	0.788	0.054	0.048	4.55E-22	8.60E-09
cg13584882	10	74836656	*P4HA1*	0.459	0.402	0.431	-0.056	-0.028	5.72E-53	1.12E-07

cg15134506	10	75336540	*USP54*	0.725	0.777	0.751	0.052	0.027	6.78E-41	5.41E-06
cg19157819	10	75405260	*SYNPO2L*	0.367	0.278	0.333	-0.089	-0.034	1.52E-108	2.37E-09
cg02227879	10	75576192	*CAMK2G*	0.486	0.375	0.449	-0.111	-0.037	2.46E-113	4.80E-09
cg25252561	10	75610927	*CAMK2G*	0.288	0.237	0.259	-0.051	-0.029	2.82E-80	1.61E-16
cg07512258	10	75619420	*CAMK2G*	0.702	0.776	0.730	0.074	0.028	2.51E-148	1.92E-13
cg04084348	10	75677011	*PLAU*	0.283	0.218	0.251	-0.065	-0.032	3.67E-99	2.66E-13
cg23434815	10	75839302	*VCL*	0.644	0.703	0.682	0.059	0.038	1.43E-34	3.14E-08
cg04764812	10	76582315	NA	0.356	0.274	0.318	-0.082	-0.038	4.41E-102	9.14E-13
cg15075988	10	76974078	*VDAC2*	0.453	0.340	0.413	-0.113	-0.040	8.17E-143	4.15E-13
cg13852284	10	77548353	*C10orf11*	0.522	0.431	0.489	-0.092	-0.034	5.35E-109	1.00E-09
cg23533270	10	78635553	*KCNMA1*	0.681	0.737	0.720	0.056	0.039	2.82E-31	9.25E-08
cg26465532	10	79446426	NA	0.740	0.793	0.770	0.053	0.030	8.95E-47	2.09E-07
cg10692363	10	80327890	NA	0.466	0.403	0.432	-0.064	-0.034	1.03E-49	1.28E-08
cg13650740	10	80516517	NA	0.352	0.294	0.331	-0.058	-0.020	6.59E-87	5.02E-08
cg05162858	10	80697460	NA	0.366	0.267	0.319	-0.100	-0.048	1.27E-113	3.82E-14
cg18104920	10	80798089	*LOC283050*	0.736	0.782	0.788	0.046	0.052	6.39E-19	1.00E-10
cg13186559	10	80854035	*ZMIZ1*	0.378	0.313	0.352	-0.066	-0.027	3.90E-106	1.77E-11
cg23865980	10	80904376	*ZMIZ1*	0.335	0.257	0.302	-0.078	-0.033	1.78E-102	1.47E-10
cg08458487	10	81709191	*SFTPD*	0.389	0.290	0.342	-0.099	-0.047	9.11E-146	3.67E-22
cg04329351	10	81955971	*ANXA11*	0.750	0.808	0.777	0.058	0.026	9.46E-66	1.11E-07
cg23258615	10	81961468	*ANXA11*	0.308	0.251	0.291	-0.057	-0.017	7.50E-100	6.44E-06
cg25783892	10	85898649	*GHITM*	0.796	0.847	0.830	0.051	0.035	4.50E-44	2.24E-10
cg12706330	10	85898700	*GHITM*	0.728	0.796	0.768	0.068	0.041	5.72E-46	5.10E-09
cg17940587	10	89292231	*MINPP1*	0.304	0.233	0.275	-0.071	-0.028	9.12E-101	2.32E-10
cg23381224	10	89311690	*MINPP1*	0.728	0.775	0.782	0.047	0.054	7.59E-20	8.82E-13
cg03667488	10	89733191	NA	0.753	0.804	0.808	0.051	0.055	2.91E-23	3.59E-13
cg17766305	10	90147030	*RNLS*	0.383	0.292	0.352	-0.091	-0.032	2.47E-81	1.51E-06
cg25907743	10	90519680	*LIPN*	0.298	0.221	0.266	-0.077	-0.032	1.02E-102	1.94E-10
cg03249630	10	90611782	*ANKRD22*	0.452	0.345	0.412	-0.108	-0.040	1.88E-103	2.62E-09
cg01561719	10	90611855	*ANKRD22*	0.454	0.353	0.405	-0.101	-0.049	5.97E-135	1.62E-19
cg24065504	10	90613015	*ANKRD22*	0.376	0.289	0.344	-0.087	-0.032	1.73E-101	1.37E-08
cg07510282	10	90644018	*STAMBPL1*	0.760	0.812	0.801	0.053	0.042	3.01E-30	2.17E-09
cg26478401	10	90749920	*FAS*	0.319	0.250	0.266	-0.069	-0.053	3.60E-51	2.97E-15
cg02314339	10	91020653	NA	0.753	0.682	0.717	-0.071	-0.036	1.02E-68	4.70E-13
cg22939324	10	91128729	NA	0.703	0.756	0.759	0.053	0.056	8.13E-25	1.39E-12
cg18044543	10	91144795	*IFIT1B*	0.373	0.298	0.344	-0.076	-0.029	1.92E-90	3.28E-08
cg05552874	10	91153143	*IFIT1*	0.710	0.461	0.568	-0.249	-0.142	2.54E-128	2.71E-56
cg06376949	10	91173811	*IFIT5*	0.372	0.298	0.336	-0.074	-0.036	1.55E-59	2.05E-09
cg22534097	10	92681082	*ANKRD1*	0.477	0.382	0.438	-0.095	-0.039	1.17E-100	3.83E-10
cg08448711	10	92794806	NA	0.487	0.429	0.454	-0.058	-0.033	2.48E-45	1.17E-08
cg20180364	10	94448532	*HHEX*	0.389	0.299	0.347	-0.090	-0.043	2.05E-115	1.05E-16
cg11276093	10	95123774	*MYOF*	0.527	0.454	0.494	-0.073	-0.033	1.75E-75	5.69E-09
cg23527183	10	95253833	NA	0.708	0.758	0.749	0.050	0.042	1.06E-21	4.39E-07
cg24857560	10	96521152	*CYP2C19*	0.693	0.746	0.758	0.053	0.064	4.29E-21	2.46E-13
cg18949056	10	97317243	*SORBS1*	0.702	0.753	0.742	0.051	0.040	4.06E-27	6.04E-08
cg13471990	10	97515222	*ENTPD1*	0.309	0.236	0.283	-0.073	-0.026	9.06E-92	1.18E-06
cg04451770	10	97515372	*ENTPD1*	0.404	0.311	0.364	-0.093	-0.041	8.66E-132	1.85E-14
cg12497543	10	97515377	*ENTPD1*	0.396	0.307	0.357	-0.089	-0.040	1.01E-120	3.52E-13
cg27479162	10	98450737	*PIK3AP1*	0.403	0.322	0.360	-0.082	-0.043	1.78E-111	7.29E-22
cg13383814	10	98745012	*C10orf12*	0.231	0.174	0.210	-0.058	-0.021	9.34E-111	1.15E-08
cg12182708	10	99223744	*MMS19*	0.447	0.333	0.398	-0.114	-0.048	3.38E-186	5.15E-25
cg17296078	10	99259721	*MMS19*	0.330	0.256	0.297	-0.075	-0.033	3.43E-113	2.72E-13
cg15108641	10	99263320	*UBTD1*	0.429	0.377	0.400	-0.052	-0.029	1.81E-38	1.59E-06
cg08181251	10	99443455	*AVPI1*	0.383	0.297	0.347	-0.086	-0.036	1.10E-124	9.10E-15
cg04902811	10	99478719	NA	0.301	0.242	0.268	-0.059	-0.033	4.77E-130	1.33E-24
cg13424029	10	101297508	NA	0.356	0.265	0.307	-0.091	-0.050	1.98E-100	5.45E-19
cg15903032	10	101297605	NA	0.486	0.433	0.453	-0.052	-0.033	8.41E-60	3.46E-14
cg23615741	10	101297642	NA	0.436	0.330	0.368	-0.107	-0.068	1.61E-62	3.49E-15
cg11180667	10	101415865	NA	0.406	0.308	0.367	-0.098	-0.039	1.67E-112	1.63E-11
cg26746309	10	101943528	*ERLIN1*	0.356	0.259	0.306	-0.097	-0.049	6.29E-151	2.80E-22
cg19499884	10	102760724	*LZTS2*	0.431	0.335	0.389	-0.096	-0.042	1.91E-136	3.88E-17
cg24760467	10	102760784	*LZTS2*	0.580	0.474	0.522	-0.105	-0.058	8.00E-123	9.33E-24
cg23534216	10	102821552	*KAZALD1*	0.396	0.332	0.373	-0.064	-0.022	1.18E-82	2.69E-07
cg26605164	10	102821565	*KAZALD1*	0.501	0.404	0.468	-0.098	-0.033	1.08E-106	2.65E-09
cg13269555	10	102822002	*KAZALD1*	0.515	0.455	0.477	-0.060	-0.038	1.31E-39	3.23E-09
cg02891579	10	102823812	*KAZALD1*	0.316	0.255	0.290	-0.061	-0.025	9.53E-142	1.46E-17
cg02505177	10	103574626	*MGEA5*	0.600	0.685	0.623	0.085	0.023	2.07E-149	1.39E-11
cg05251190	10	104196206	NA	0.536	0.481	0.498	-0.055	-0.039	2.57E-46	4.73E-12
cg04774043	10	104498430	*SFXN2*	0.336	0.266	0.308	-0.069	-0.027	3.92E-81	1.02E-07
cg14939082	10	104535990	*WBP1L*	0.306	0.246	0.287	-0.060	-0.020	1.90E-77	5.39E-06
cg16396933	10	104954103	*NT5C2*	0.449	0.380	0.412	-0.070	-0.037	2.03E-55	3.79E-09
cg02921623	10	105250537	NA	0.336	0.406	0.370	0.070	0.034	3.81E-50	5.33E-08
cg21359303	10	106068573	NA	0.770	0.830	0.791	0.060	0.020	3.71E-111	5.43E-08
cg00349404	10	106100036	NA	0.587	0.511	0.532	-0.076	-0.055	1.10E-44	2.16E-12
cg24394891	10	111646370	*XPNPEP1*	0.456	0.395	0.424	-0.060	-0.032	2.84E-52	3.05E-08
cg04350202	10	111653363	*XPNPEP1*	0.494	0.417	0.461	-0.077	-0.033	5.83E-105	1.90E-12
cg18780288	10	111659903	*XPNPEP1*	0.305	0.230	0.276	-0.075	-0.030	3.87E-100	1.76E-09
cg17841267	10	112117449	NA	0.727	0.800	0.750	0.074	0.024	6.01E-122	1.69E-08

cg25024734	10	112152815	NA	0.260	0.202	0.227	-0.058	-0.033	2.70E-46	4.40E-07
cg00066854	10	113987376	NA	0.402	0.306	0.364	-0.096	-0.038	1.51E-161	3.58E-17
cg24103651	10	113987401	NA	0.302	0.232	0.261	-0.070	-0.041	4.04E-95	1.06E-18
cg17056676	10	116301354	*ABLIM1*	0.581	0.660	0.615	0.079	0.034	3.89E-152	7.84E-21
cg03850986	10	116408382	*ABLIM1*	0.528	0.474	0.483	-0.053	-0.045	3.04E-27	1.62E-09
cg01185921	10	116444463	*ABLIM1*	0.592	0.537	0.552	-0.054	-0.039	9.32E-51	5.35E-13
cg10858945	10	116528318	NA	0.315	0.258	0.288	-0.057	-0.026	1.61E-63	1.16E-08
cg04886849	10	118997146	NA	0.385	0.308	0.359	-0.077	-0.026	2.36E-128	1.00E-09
cg17374433	10	119876327	*CASC2*	0.742	0.789	0.795	0.047	0.053	3.94E-18	1.67E-11
cg08078028	10	120802096	*EIF3A*	0.677	0.576	0.635	-0.101	-0.042	1.98E-138	5.16E-19
cg15361215	10	120873630	*FAM45B*	0.345	0.263	0.309	-0.082	-0.036	9.24E-96	1.79E-10
cg24902461	10	121031392	*GRK5*	0.406	0.327	0.371	-0.078	-0.034	1.01E-74	3.00E-08
cg15580458	10	121155124	*GRK5*	0.416	0.324	0.378	-0.092	-0.037	2.03E-138	1.52E-14
cg05617307	10	121413182	*BAG3*	0.780	0.833	0.829	0.052	0.049	3.68E-34	1.44E-12
cg20636248	10	121439845	NA	0.443	0.344	0.404	-0.099	-0.038	1.47E-164	2.16E-19
cg15272641	10	121775065	NA	0.720	0.779	0.763	0.059	0.043	2.53E-35	5.23E-09
cg01468579	10	122102016	NA	0.691	0.742	0.729	0.052	0.038	5.30E-27	4.37E-07
cg13789938	10	122614164	*MIR5694*	0.667	0.713	0.719	0.046	0.052	1.89E-19	1.06E-10
cg02556345	10	124181965	*PLEKHA1*	0.451	0.506	0.480	0.055	0.029	1.77E-48	1.89E-07
cg06474225	10	124228770	*HTRA1*	0.353	0.284	0.318	-0.069	-0.035	9.84E-74	3.25E-10
cg19975346	10	126366131	*FAM53B*	0.508	0.426	0.480	-0.082	-0.028	2.96E-132	2.88E-10
cg09519218	10	126390317	*FAM53B*	0.377	0.317	0.350	-0.061	-0.027	1.93E-122	4.41E-17
cg04516672	10	126411663	*FAM53B*	0.601	0.661	0.639	0.060	0.037	7.43E-54	3.38E-10
cg06738031	10	126806416	*CTBP2*	0.703	0.755	0.752	0.052	0.048	1.86E-29	2.91E-12
cg27027668	10	127277604	NA	0.613	0.663	0.655	0.050	0.042	9.25E-21	1.32E-07
cg07053114	10	129794994	*PTPRE*	0.360	0.272	0.330	-0.088	-0.029	4.82E-130	6.83E-11
cg07986469	10	129795003	*PTPRE*	0.441	0.349	0.408	-0.093	-0.033	1.40E-178	9.97E-19
cg20224780	10	132942362	*TCERG1L*	0.674	0.733	0.732	0.059	0.058	4.88E-31	4.46E-14
cg14321777	10	133874809	NA	0.633	0.692	0.685	0.060	0.052	2.98E-24	6.34E-09
cg11579421	10	134211857	*PWWP2B*	0.352	0.283	0.330	-0.069	-0.021	1.29E-86	5.53E-06
cg22045288	10	134258421	*C10orf91*	0.347	0.284	0.317	-0.063	-0.030	1.85E-104	2.69E-16
cg16943126	10	134311114	NA	0.349	0.281	0.324	-0.068	-0.025	4.21E-86	5.91E-09
cg06760238	10	134400036	*INPP5A*	0.431	0.341	0.394	-0.091	-0.038	1.13E-83	1.09E-08
cg02951695	10	134400351	*INPP5A*	0.704	0.737	0.755	0.034	0.052	1.72E-11	3.50E-11
cg09163720	10	134400506	*INPP5A*	0.600	0.490	0.568	-0.110	-0.032	6.29E-104	1.23E-07
cg14953730	10	134498830	*INPP5A*	0.315	0.261	0.290	-0.055	-0.026	1.66E-61	5.27E-08
cg10552523	11	313478	*IFITM1*	0.387	0.316	0.317	-0.070	-0.069	3.28E-34	4.66E-22
cg01971407	11	313624	*IFITM1*	0.482	0.400	0.411	-0.082	-0.071	3.81E-53	3.26E-30
cg23570810	11	315102	*IFITM1*	0.686	0.490	0.545	-0.196	-0.141	1.57E-75	6.14E-38
cg03038262	11	315262	*IFITM1*	0.573	0.450	0.480	-0.123	-0.093	1.55E-50	2.69E-32
cg20045320	11	319555	NA	0.553	0.425	0.458	-0.128	-0.095	1.01E-63	5.32E-26
cg09122035	11	319667	NA	0.492	0.363	0.398	-0.129	-0.094	3.11E-72	1.46E-20
cg17990365	11	319718	*IFITM3*	0.614	0.504	0.531	-0.110	-0.083	9.48E-56	5.92E-23
cg12912872	11	330536	NA	0.673	0.727	0.705	0.054	0.032	3.62E-59	4.61E-11
cg01704474	11	504918	*RNH1*	0.383	0.321	0.350	-0.062	-0.033	2.06E-48	2.65E-08
cg20813589	11	504930	*RNH1*	0.446	0.388	0.415	-0.058	-0.030	8.34E-51	2.33E-09
cg17114584	11	613792	*IRF7*	0.603	0.535	0.553	-0.068	-0.050	2.75E-19	2.97E-10
cg08926253	11	614761	*IRF7*	0.597	0.468	0.519	-0.128	-0.077	6.80E-81	3.84E-34
cg22016995	11	614787	*IRF7*	0.954	0.895	0.932	-0.060	-0.022	2.20E-37	7.24E-16
cg02471078	11	688159	*DEAF1*	0.620	0.674	0.696	0.054	0.075	3.12E-19	2.03E-15
cg14522803	11	818834	*PNPLA2*	0.428	0.355	0.408	-0.073	-0.020	9.45E-137	1.69E-08
cg24427660	11	818892	*PNPLA2*	0.430	0.329	0.393	-0.101	-0.037	2.60E-189	6.95E-20
cg18313182	11	818903	*PNPLA2*	0.443	0.364	0.419	-0.079	-0.024	5.71E-176	1.64E-14
cg06449094	11	822397	*PNPLA2*	0.550	0.470	0.525	-0.081	-0.026	1.37E-79	1.31E-06
cg06072036	11	822402	*PNPLA2*	0.426	0.334	0.393	-0.092	-0.033	1.13E-107	2.06E-10
cg09524686	11	829182	*EFCAB4A*	0.350	0.276	0.322	-0.074	-0.028	1.42E-146	1.13E-16
cg19303748	11	844085	*TSPAN4*	0.235	0.181	0.214	-0.054	-0.020	1.21E-92	8.22E-08
cg04552852	11	844390	*TSPAN4*	0.456	0.396	0.423	-0.060	-0.033	7.04E-85	9.22E-15
cg12464638	11	844400	*TSPAN4*	0.562	0.502	0.525	-0.061	-0.038	5.41E-64	9.34E-13
cg01636582	11	844593	*TSPAN4*	0.284	0.228	0.261	-0.056	-0.023	1.39E-119	2.12E-13
cg06964027	11	1073496	*MUC2*	0.269	0.211	0.241	-0.058	-0.029	5.23E-99	2.83E-14
cg23240927	11	1320497	*TOLLIP*	0.514	0.399	0.473	-0.115	-0.041	2.12E-111	1.17E-09
cg07546654	11	1320546	*TOLLIP*	0.464	0.354	0.418	-0.109	-0.046	4.45E-121	1.94E-13
cg09566995	11	1320634	*TOLLIP*	0.341	0.250	0.298	-0.090	-0.043	3.09E-100	1.52E-12
cg14004685	11	1750302	*MOB2*	0.655	0.716	0.700	0.061	0.044	6.96E-58	2.72E-16
cg26868156	11	1892180	*LSP1*	0.661	0.730	0.692	0.069	0.031	1.91E-73	3.14E-10
cg24024833	11	1892608	*LSP1*	0.523	0.588	0.552	0.066	0.029	1.68E-98	2.98E-13
cg14600987	11	1952678	*TNNT3*	0.384	0.299	0.334	-0.085	-0.051	1.80E-127	7.31E-29
cg26983198	11	1952756	*TNNT3*	0.315	0.242	0.281	-0.073	-0.033	2.77E-85	3.21E-10
cg12949760	11	2542862	*KCNQ1*	0.430	0.368	0.395	-0.062	-0.035	1.80E-61	2.33E-10
cg06294475	11	2698623	*KCNQ1OT1*	0.370	0.280	0.335	-0.090	-0.035	1.05E-133	3.13E-13
cg05438727	11	2800047	*KCNQ1*	0.348	0.293	0.327	-0.055	-0.022	4.81E-79	1.18E-07
cg03155200	11	2833259	*KCNQ1*	0.442	0.349	0.396	-0.093	-0.046	3.11E-83	5.18E-11
cg12141659	11	2847462	*KCNQ1*	0.264	0.199	0.235	-0.065	-0.029	2.59E-136	9.65E-18
cg21130221	11	2848310	*KCNQ1*	0.501	0.443	0.459	-0.059	-0.042	1.16E-68	2.42E-17
cg03143849	11	2904951	*CDKN1C*	0.562	0.511	0.527	-0.051	-0.035	8.86E-31	8.02E-08
cg25427871	11	2920735	*SLC22A18AS*	0.337	0.281	0.312	-0.056	-0.025	3.20E-76	8.12E-09
cg01418188	11	3145609	*OSBPL5*	0.332	0.273	0.299	-0.058	-0.033	2.53E-99	3.15E-18

cg14183540	11	3175007	*OSBPL5*	0.416	0.333	0.375	-0.083	-0.042	5.87E-135	1.78E-23
cg18591228	11	3175552	*OSBPL5*	0.501	0.413	0.473	-0.088	-0.028	7.83E-100	1.59E-07
cg15964132	11	3175636	*OSBPL5*	0.495	0.403	0.468	-0.092	-0.027	6.50E-108	3.31E-07
cg06005892	11	3177622	*OSBPL5*	0.420	0.339	0.390	-0.080	-0.029	1.62E-112	2.00E-10
cg14488317	11	3181446	*OSBPL5*	0.286	0.224	0.260	-0.061	-0.025	1.64E-73	7.68E-07
cg26391219	11	3189207	NA	0.419	0.337	0.388	-0.082	-0.032	9.56E-128	1.91E-14
cg18030105	11	3396237	*ZNF195*	0.684	0.707	0.739	0.023	0.055	5.37E-06	7.33E-12
cg05344046	11	3731649	*NUP98*	0.711	0.758	0.764	0.047	0.053	1.14E-15	5.95E-09
cg15069471	11	3880668	*STIM1*	0.743	0.793	0.773	0.050	0.030	6.36E-53	4.12E-09
cg02770745	11	3926837	*STIM1*	0.481	0.368	0.446	-0.112	-0.034	4.19E-123	2.19E-08
cg15781504	11	4871411	*OR51S1*	0.745	0.796	0.777	0.051	0.032	9.64E-33	5.18E-07
cg21966764	11	6192512	*OR52B2*	0.732	0.784	0.762	0.051	0.030	5.20E-38	2.82E-07
cg10611016	11	6225759	*C11orf42*	0.522	0.410	0.487	-0.112	-0.035	4.61E-130	2.64E-09
cg15511120	11	6598119	NA	0.388	0.293	0.345	-0.095	-0.043	3.60E-135	1.93E-17
cg16833110	11	6942045	*OR2D3*	0.722	0.767	0.775	0.045	0.054	4.23E-16	5.38E-11
cg20599009	11	6945204	NA	0.747	0.799	0.790	0.052	0.043	3.14E-25	2.24E-08
cg17084751	11	6953558	*ZNF215*	0.638	0.698	0.671	0.060	0.033	1.65E-52	1.38E-08
cg14838970	11	7533817	*PPFIBP2*	0.369	0.301	0.345	-0.068	-0.024	1.00E-77	4.38E-07
cg01607369	11	7598673	*PPFIBP2*	0.367	0.313	0.340	-0.053	-0.026	1.39E-68	1.57E-09
cg15228694	11	7692131	*CYB5R2*	0.514	0.420	0.468	-0.094	-0.046	7.20E-70	7.79E-11
cg12165551	11	8385712	NA	0.340	0.259	0.303	-0.080	-0.036	1.09E-124	3.25E-16
cg08356637	11	8385767	NA	0.395	0.315	0.339	-0.079	-0.056	4.71E-88	1.01E-24
cg26826329	11	8612947	*STK33*	0.740	0.786	0.793	0.046	0.053	7.17E-17	7.88E-11
cg06127316	11	9599460	*WEE1*	0.711	0.775	0.756	0.064	0.045	7.12E-38	3.22E-09
cg06876053	11	9810051	*SBF2-AS1*	0.283	0.228	0.243	-0.055	-0.041	1.43E-64	5.56E-16
cg01116568	11	10476529	*AMPD3*	0.414	0.342	0.386	-0.072	-0.028	4.27E-68	5.87E-06
cg19132462	11	10476608	*AMPD3*	0.410	0.327	0.379	-0.083	-0.031	4.02E-98	1.01E-08
cg07329251	11	10476662	*AMPD3*	0.286	0.232	0.259	-0.054	-0.027	2.30E-63	2.12E-09
cg17471425	11	10614014	*MRVI1-AS1*	0.241	0.185	0.206	-0.056	-0.035	2.33E-81	1.55E-16
cg05382956	11	10627849	*MRVI1*	0.297	0.238	0.267	-0.059	-0.030	2.92E-67	2.78E-09
cg26262049	11	10628012	*MRVI1*	0.404	0.326	0.356	-0.077	-0.047	1.61E-79	4.01E-16
cg24505713	11	10770035	NA	0.301	0.229	0.270	-0.072	-0.032	1.18E-118	9.74E-14
cg27062514	11	10800841	*CTR9*	0.755	0.813	0.790	0.058	0.035	4.99E-44	1.27E-08
cg02501827	11	11862879	*USP47*	0.263	0.198	0.237	-0.064	-0.026	7.51E-76	1.88E-07
cg03821121	11	12148288	*MICAL2*	0.354	0.285	0.325	-0.069	-0.029	2.10E-134	4.22E-17
cg07702424	11	12306515	NA	0.591	0.484	0.553	-0.107	-0.038	3.24E-153	1.35E-13
cg03167648	11	13094027	NA	0.571	0.611	0.629	0.040	0.057	1.78E-14	6.13E-13
cg12907644	11	18270341	*SAA2-SAA4*	0.565	0.512	0.542	-0.053	-0.023	2.85E-53	2.48E-06
cg15484375	11	18287647	*SAA1*	0.500	0.441	0.463	-0.059	-0.037	2.04E-56	1.77E-12
cg16964946	11	27442973	*LGR4*	0.514	0.429	0.483	-0.085	-0.031	2.93E-80	1.80E-07
cg19238415	11	32916120	*QSER1*	0.374	0.279	0.327	-0.096	-0.047	9.92E-119	7.18E-17
cg06710328	11	33217195	NA	0.323	0.249	0.288	-0.074	-0.034	1.69E-84	2.38E-10
cg04225088	11	33278186	*HIPK3*	0.526	0.410	0.487	-0.115	-0.039	4.91E-130	2.76E-11
cg05501357	11	33308269	*HIPK3*	0.472	0.365	0.443	-0.107	-0.029	2.21E-151	3.24E-09
cg25600606	11	33308345	*HIPK3*	0.611	0.476	0.566	-0.136	-0.045	1.68E-183	3.70E-17
cg15382933	11	33745399	*CD59*	0.464	0.353	0.410	-0.110	-0.054	5.17E-129	8.95E-18
cg24453664	11	33758413	*CD59*	0.350	0.269	0.321	-0.081	-0.028	1.57E-98	1.20E-07
cg16568681	11	33758505	*CD59*	0.353	0.276	0.318	-0.077	-0.035	4.92E-76	4.32E-09
cg02224369	11	33758529	*CD59*	0.316	0.254	0.289	-0.062	-0.027	2.07E-60	5.52E-07
cg04590170	11	33893895	*LMO2*	0.461	0.391	0.428	-0.071	-0.033	8.48E-70	3.74E-08
cg15356966	11	33913187	*LMO2*	0.374	0.295	0.346	-0.080	-0.028	9.89E-85	1.90E-06
cg20147326	11	33913538	*LMO2*	0.275	0.204	0.247	-0.071	-0.028	2.51E-128	1.79E-11
cg09358725	11	33914088	*LMO2*	0.445	0.344	0.380	-0.101	-0.065	1.67E-143	8.46E-35
cg13100962	11	33921158	NA	0.527	0.469	0.497	-0.058	-0.030	1.42E-39	2.43E-06
cg13289202	11	34322226	*ABTB2*	0.336	0.269	0.311	-0.067	-0.025	1.14E-77	1.56E-06
cg04645070	11	34393106	NA	0.266	0.212	0.235	-0.054	-0.032	6.96E-50	2.28E-09
cg15994604	11	34676683	*EHF*	0.391	0.287	0.336	-0.104	-0.055	1.10E-169	5.15E-29
cg13523125	11	35072710	NA	0.351	0.277	0.325	-0.074	-0.026	3.27E-71	6.38E-06
cg14082886	11	35164485	*CD44*	0.487	0.571	0.529	0.085	0.042	1.93E-87	8.46E-13
cg14593191	11	35201133	*CD44*	0.690	0.748	0.738	0.057	0.048	5.41E-35	1.41E-12
cg08688659	11	35249965	*CD44*	0.470	0.351	0.422	-0.119	-0.047	1.90E-180	7.39E-19
cg04940312	11	35688285	*TRIM44*	0.736	0.788	0.794	0.052	0.058	7.33E-21	3.61E-11
cg18422371	11	36593664	*RAG1*	0.680	0.742	0.719	0.062	0.039	5.15E-42	1.01E-08
cg21671607	11	36616015	*RAG2*	0.230	0.177	0.203	-0.053	-0.026	4.36E-55	6.65E-08
cg05070250	11	36620199	*RAG2*	0.747	0.802	0.779	0.055	0.033	1.56E-38	4.31E-07
cg26999154	11	43291043	NA	0.456	0.511	0.524	0.055	0.068	6.04E-11	1.33E-08
cg14780449	11	44578801	NA	0.613	0.557	0.590	-0.056	-0.023	4.98E-76	8.62E-08
cg22854223	11	44586505	*CD82*	0.233	0.180	0.208	-0.053	-0.025	2.99E-77	6.99E-10
cg21108085	11	44591098	*CD82*	0.217	0.158	0.177	-0.059	-0.039	5.75E-61	1.95E-13
cg03211593	11	44731693	NA	0.313	0.250	0.285	-0.063	-0.028	1.11E-123	4.90E-17
cg24012880	11	44880910	*TSPAN18*	0.465	0.364	0.419	-0.101	-0.045	7.68E-90	7.63E-11
cg23500396	11	44896984	*TSPAN18*	0.726	0.665	0.705	-0.062	-0.021	2.41E-73	1.85E-06
cg05949397	11	44976423	NA	0.540	0.426	0.490	-0.115	-0.050	1.29E-130	1.46E-17
cg08794544	11	45825518	*SLC35C1*	0.353	0.288	0.322	-0.065	-0.031	6.00E-97	3.07E-13
cg24305156	11	45871936	*CRY2*	0.394	0.310	0.360	-0.084	-0.034	1.27E-147	1.86E-16
cg21002528	11	45903754	*CRY2*	0.474	0.380	0.434	-0.094	-0.039	8.04E-126	8.64E-15
cg27609217	11	46258039	NA	0.403	0.317	0.369	-0.087	-0.034	7.04E-99	3.89E-10
cg22948808	11	46360763	*DGKZ*	0.405	0.337	0.370	-0.068	-0.035	7.10E-97	2.31E-14

cg02211741	11	46383924	*DGKZ*	0.293	0.229	0.270	-0.064	-0.024	8.71E-100	1.12E-09
cg27137887	11	46513635	*AMBRA1*	0.511	0.459	0.479	-0.052	-0.032	2.21E-43	1.15E-08
cg20090290	11	46543695	*AMBRA1*	0.466	0.352	0.425	-0.114	-0.041	3.41E-152	1.36E-14
cg16389345	11	46697382	*ATG13*	0.558	0.501	0.529	-0.058	-0.029	4.95E-68	2.51E-09
cg23595304	11	46879516	*LRP4*	0.396	0.297	0.347	-0.099	-0.049	1.59E-148	2.08E-24
cg24909660	11	47276469	*NR1H3*	0.377	0.289	0.337	-0.088	-0.040	7.11E-167	1.28E-25
cg21203569	11	47279365	*NR1H3*	0.337	0.277	0.307	-0.061	-0.031	3.28E-56	3.44E-07
cg15945333	11	47399213	*SPI1*	0.380	0.294	0.344	-0.086	-0.035	1.02E-100	6.25E-10
cg15982099	11	47399813	*SPI1*	0.390	0.293	0.349	-0.097	-0.041	7.03E-119	2.37E-14
cg07675031	11	47399893	*SPI1*	0.347	0.269	0.323	-0.078	-0.024	8.86E-92	1.41E-06
cg10435245	11	47399998	*SPI1*	0.442	0.346	0.408	-0.096	-0.035	1.82E-104	7.60E-10
cg08594651	11	47415397	NA	0.387	0.307	0.356	-0.080	-0.031	1.28E-96	1.84E-08
cg23582982	11	47416109	NA	0.333	0.280	0.316	-0.053	-0.017	4.77E-73	1.92E-06
cg13047308	11	47471339	*RAPSN*	0.611	0.516	0.580	-0.095	-0.031	1.90E-119	1.27E-08
cg00005619	11	47608722	*FAM180B*	0.570	0.499	0.540	-0.071	-0.031	2.14E-92	3.35E-10
cg18512352	11	47633146	NA	0.336	0.262	0.301	-0.074	-0.035	3.82E-118	7.71E-15
cg18065599	11	47968995	NA	0.364	0.278	0.324	-0.086	-0.040	3.72E-155	4.30E-22
cg23646614	11	48083100	*PTPRJ*	0.243	0.191	0.215	-0.053	-0.028	1.34E-92	1.68E-14
cg26197915	11	48085137	*PTPRJ*	0.347	0.289	0.320	-0.058	-0.027	1.99E-55	3.93E-07
cg10426076	11	57158282	*PRG2*	0.474	0.370	0.435	-0.103	-0.039	7.05E-128	1.62E-12
cg23547429	11	57195025	*SLC43A3*	0.330	0.272	0.297	-0.058	-0.033	7.02E-129	1.17E-26
cg11554335	11	57336941	*UBE2L6*	0.536	0.460	0.506	-0.077	-0.030	7.79E-89	4.14E-08
cg20907456	11	57405372	NA	0.281	0.228	0.258	-0.052	-0.023	4.95E-75	4.74E-09
cg00135888	11	57407543	NA	0.290	0.237	0.264	-0.052	-0.026	1.37E-92	4.83E-12
cg21272996	11	57529255	*TMX2-CTNNI*	0.394	0.304	0.346	-0.090	-0.048	1.83E-100	9.62E-16
cg18213545	11	57529400	*TMX2-CTNNI*	0.336	0.265	0.310	-0.071	-0.026	9.85E-114	4.52E-11
cg19210276	11	57529465	*TMX2-CTNNI*	0.371	0.285	0.342	-0.086	-0.029	5.25E-102	7.63E-08
cg09878888	11	57529614	*TMX2-CTNNI*	0.313	0.238	0.291	-0.075	-0.022	1.72E-99	3.71E-06
cg00315837	11	59244789	*OR4D10*	0.727	0.779	0.774	0.052	0.047	3.71E-22	2.90E-09
cg14328641	11	59822727	*MS4A3*	0.372	0.290	0.340	-0.081	-0.032	3.80E-76	1.14E-07
cg17173423	11	59823993	*MS4A3*	0.362	0.287	0.324	-0.074	-0.038	1.82E-80	1.47E-12
cg13321166	11	59824038	*MS4A3*	0.387	0.296	0.352	-0.091	-0.035	3.48E-129	2.00E-15
cg03348161	11	59824089	*MS4A3*	0.450	0.339	0.406	-0.111	-0.044	5.59E-127	3.25E-16
cg19739596	11	59824161	*MS4A3*	0.469	0.359	0.416	-0.110	-0.053	1.53E-184	1.24E-30
cg26250585	11	59950745	*MS4A6A*	0.319	0.265	0.272	-0.054	-0.046	5.07E-36	2.42E-13
cg04353769	11	59951557	*MS4A6A*	0.387	0.303	0.348	-0.084	-0.039	5.49E-164	1.19E-26
cg08716584	11	60157161	*MS4A7*	0.415	0.322	0.371	-0.093	-0.044	1.10E-68	1.27E-07
cg14132103	11	60680165	*TMEM109*	0.687	0.742	0.708	0.055	0.020	1.17E-77	4.59E-07
cg08792703	11	60779666	*CD6*	0.270	0.208	0.250	-0.062	-0.020	2.68E-97	2.82E-06
cg24068972	11	60833459	NA	0.640	0.703	0.662	0.063	0.022	9.79E-97	6.59E-09
cg24674703	11	60869960	*CD5*	0.461	0.528	0.492	0.067	0.031	1.43E-42	8.48E-07
cg00009088	11	60930188	*VPS37C*	0.520	0.407	0.485	-0.113	-0.035	2.38E-109	1.24E-07
cg24422316	11	60930346	*VPS37C*	0.461	0.369	0.410	-0.092	-0.052	2.94E-123	2.02E-25
cg21971799	11	60932010	NA	0.289	0.226	0.253	-0.064	-0.036	1.74E-64	4.63E-11
cg20242427	11	61115736	*DAK*	0.480	0.366	0.448	-0.114	-0.033	2.35E-105	1.46E-06
cg25324164	11	61598330	*FADS2*	0.284	0.219	0.246	-0.065	-0.037	2.27E-70	1.22E-11
cg11250194	11	61601937	*FADS2*	0.532	0.481	0.494	-0.051	-0.038	6.35E-39	1.64E-09
cg21988813	11	61730880	*BEST1*	0.309	0.231	0.271	-0.078	-0.038	4.66E-110	2.11E-15
cg19446990	11	61739400	NA	0.418	0.341	0.373	-0.077	-0.045	1.09E-113	2.01E-24
cg16177481	11	61740158	NA	0.217	0.160	0.190	-0.056	-0.027	8.33E-93	1.29E-10
cg14789828	11	62201168	*AHNAK*	0.232	0.180	0.207	-0.051	-0.025	2.99E-74	1.56E-09
cg20518446	11	62315034	*AHNAK*	0.576	0.511	0.534	-0.065	-0.041	8.32E-34	4.35E-08
cg25505880	11	62574338	*STX5*	0.410	0.306	0.361	-0.103	-0.049	1.87E-210	1.33E-37
cg21702188	11	62574403	*STX5*	0.344	0.257	0.299	-0.087	-0.045	4.15E-166	5.05E-28
cg19957411	11	63273333	*LGALS12*	0.410	0.350	0.387	-0.060	-0.022	3.60E-75	5.47E-07
cg12792363	11	63274030	*LGALS12*	0.320	0.245	0.294	-0.075	-0.027	8.78E-136	4.08E-11
cg19240569	11	63331821	*HRASLS2*	0.674	0.748	0.695	0.074	0.021	1.65E-126	7.46E-07
cg18550212	11	63435428	*ATL3*	0.302	0.243	0.274	-0.059	-0.028	6.02E-52	2.29E-06
cg10675058	11	63858880	*MACROD1*	0.470	0.398	0.445	-0.073	-0.025	1.99E-96	1.15E-08
cg14654385	11	63973006	*FERMT3*	0.437	0.350	0.404	-0.087	-0.033	1.16E-158	8.15E-19
cg10718809	11	64087106	*PRDX5*	0.401	0.319	0.378	-0.081	-0.023	9.31E-137	5.83E-09
cg15009090	11	64163340	NA	0.304	0.230	0.262	-0.074	-0.042	7.02E-60	2.45E-10
cg26732720	11	64392533	*NRXN2*	0.278	0.218	0.242	-0.061	-0.037	6.73E-120	1.45E-25
cg27209729	11	64428925	*NRXN2*	0.614	0.535	0.535	-0.079	-0.079	9.25E-50	4.57E-27
cg14378925	11	64635728	*EHD1*	0.741	0.809	0.769	0.068	0.029	1.81E-99	7.15E-12
cg07019638	11	64635774	*EHD1*	0.798	0.855	0.829	0.057	0.031	7.69E-91	1.15E-13
cg11321083	11	64638190	*EHD1*	0.345	0.290	0.314	-0.055	-0.031	1.46E-56	3.63E-11
cg17087974	11	65171397	*FRMD8*	0.380	0.292	0.347	-0.088	-0.033	2.35E-214	1.67E-29
cg03249011	11	65195995	NA	0.490	0.545	0.523	0.055	0.033	2.44E-32	5.49E-07
cg11573318	11	65196227	NA	0.630	0.687	0.672	0.057	0.042	7.62E-36	1.67E-10
cg05436845	11	65378622	*MAP3K11*	0.416	0.330	0.386	-0.086	-0.030	2.44E-149	8.07E-17
cg08908247	11	66055170	*YIF1A*	0.391	0.324	0.365	-0.067	-0.026	2.99E-121	1.20E-12
cg22406869	11	66276941	*DPP3*	0.435	0.380	0.400	-0.055	-0.035	5.07E-63	1.07E-13
cg13980609	11	66883117	NA	0.358	0.293	0.330	-0.065	-0.028	1.78E-67	4.58E-08
cg04407063	11	67051977	*ADRBK1*	0.453	0.350	0.406	-0.103	-0.047	5.05E-149	6.31E-24
cg16449084	11	67069814	*SSH3*	0.407	0.314	0.371	-0.093	-0.036	3.32E-167	5.40E-19
cg20705781	11	67070238	*SSH3*	0.374	0.304	0.346	-0.071	-0.028	5.31E-62	6.01E-06
cg15453278	11	67134607	*CLCF1*	0.385	0.320	0.362	-0.065	-0.023	6.11E-124	6.74E-12

cg18101140	11	67142001	*CLCF1*	0.278	0.206	0.239	-0.073	-0.039	1.07E-106	1.96E-17
cg10346364	11	67142268	*CLCF1*	0.354	0.285	0.329	-0.069	-0.024	7.03E-111	9.91E-10
cg10422334	11	67183616	*CARNS1*	0.640	0.701	0.666	0.060	0.025	7.15E-116	4.13E-14
cg03559915	11	67201998	*RPS6KB2*	0.661	0.732	0.695	0.071	0.034	8.81E-103	1.08E-15
cg02685484	11	67203360	*PTPRCAP*	0.655	0.711	0.680	0.056	0.025	4.89E-96	7.03E-12
cg25256924	11	67205739	*PTPRCAP*	0.598	0.682	0.626	0.084	0.028	3.55E-74	1.58E-06
cg16408081	11	67205869	*PTPRCAP*	0.547	0.625	0.574	0.078	0.027	5.80E-123	1.10E-13
cg23468927	11	67206263	*PTPRCAP*	0.641	0.713	0.671	0.072	0.029	1.66E-132	4.53E-15
cg12044599	11	67206308	*PTPRCAP*	0.659	0.746	0.692	0.087	0.032	1.54E-138	3.44E-14
cg19528654	11	67251154	*AIP*	0.772	0.825	0.793	0.053	0.021	2.79E-91	9.80E-09
cg20973735	11	67251677	*AIP*	0.567	0.619	0.598	0.052	0.031	1.63E-34	2.34E-07
cg03812676	11	67254381	*AIP*	0.655	0.720	0.692	0.065	0.037	4.42E-41	8.20E-07
cg22224704	11	67352041	*GSTP1*	0.345	0.290	0.315	-0.056	-0.031	2.74E-81	9.21E-14
cg18229767	11	67776576	*ALDH3B1*	0.255	0.203	0.232	-0.052	-0.022	3.00E-56	2.38E-06
cg02331303	11	67777823	*ALDH3B1*	0.401	0.311	0.359	-0.090	-0.043	2.43E-172	6.44E-30
cg07730301	11	67777952	*ALDH3B1*	0.415	0.345	0.385	-0.070	-0.030	6.14E-138	3.92E-21
cg05463966	11	67803951	*NDUFS8*	0.335	0.259	0.304	-0.076	-0.032	2.44E-113	1.48E-12
cg12070987	11	67804055	*NDUFS8*	0.490	0.437	0.473	-0.054	-0.017	1.71E-96	4.08E-10
cg18160691	11	68081686	*LRP5*	0.383	0.315	0.359	-0.068	-0.023	1.28E-122	1.56E-10
cg21746120	11	68142234	*LRP5*	0.441	0.390	0.418	-0.051	-0.024	7.14E-53	2.72E-07
cg10133100	11	68276883	*PPP6R3*	0.457	0.385	0.422	-0.073	-0.036	2.79E-86	1.32E-12
cg12564698	11	68458524	*GAL*	0.622	0.687	0.660	0.065	0.038	1.65E-34	1.13E-06
cg23756264	11	68605511	*CPT1A*	0.319	0.248	0.290	-0.071	-0.029	1.24E-124	6.11E-15
cg20363347	11	69061473	*MYEOV*	0.297	0.239	0.268	-0.058	-0.029	1.12E-52	6.16E-07
cg15297628	11	69240844	NA	0.627	0.705	0.667	0.078	0.040	2.26E-109	1.67E-18
cg22111527	11	69260136	NA	0.392	0.297	0.364	-0.094	-0.028	7.86E-126	5.93E-08
cg17007896	11	69261044	NA	0.385	0.309	0.354	-0.076	-0.031	6.83E-125	1.96E-14
cg17222829	11	70433293	*SHANK2*	0.429	0.336	0.387	-0.094	-0.042	1.36E-88	2.49E-10
cg16312514	11	70650521	*SHANK2*	0.226	0.172	0.198	-0.054	-0.028	2.22E-43	4.33E-07
cg02845204	11	71259439	*KRTAP5-9*	0.350	0.293	0.326	-0.057	-0.025	9.08E-52	3.00E-06
cg13048008	11	71725340	*NUMA1*	0.444	0.391	0.426	-0.053	-0.018	1.52E-81	1.28E-06
cg10533990	11	71846733	*FOLR3*	0.388	0.301	0.348	-0.087	-0.040	3.54E-158	1.94E-23
cg25634666	11	71846788	*FOLR3*	0.384	0.293	0.351	-0.091	-0.033	6.12E-163	7.21E-16
cg25306006	11	71932630	*FOLR2*	0.371	0.305	0.333	-0.066	-0.038	3.82E-87	5.11E-16
cg10687644	11	72527565	*ATG16L2*	0.394	0.314	0.365	-0.080	-0.030	1.09E-138	7.71E-16
cg08900384	11	72546168	NA	0.364	0.290	0.335	-0.074	-0.029	1.41E-151	2.35E-17
cg24496021	11	72662461	*FCHSD2*	0.504	0.398	0.475	-0.107	-0.030	7.65E-113	2.36E-07
cg08069287	11	72868833	NA	0.406	0.349	0.381	-0.057	-0.026	3.40E-60	4.99E-08
cg19502359	11	72912441	NA	0.398	0.306	0.372	-0.092	-0.026	1.62E-130	5.13E-07
cg01550445	11	72929983	*P2RY2*	0.580	0.527	0.549	-0.054	-0.031	7.97E-42	2.30E-07
cg04099543	11	72983405	*P2RY6*	0.368	0.299	0.341	-0.069	-0.027	1.57E-132	1.56E-15
cg12959048	11	73096162	*RELT*	0.416	0.346	0.376	-0.070	-0.040	1.79E-87	1.59E-16
cg22145091	11	73133280	*FAM168A*	0.744	0.794	0.797	0.050	0.054	4.18E-22	8.83E-12
cg13953458	11	73681281	*DNAJB13*	0.632	0.694	0.663	0.062	0.032	3.27E-66	8.69E-11
cg25429672	11	73692155	*UCP2*	0.714	0.786	0.743	0.071	0.029	6.04E-108	3.42E-10
cg11775521	11	74178795	*KCNE3*	0.312	0.235	0.280	-0.077	-0.032	8.54E-111	1.65E-12
cg18838431	11	74178800	*KCNE3*	0.353	0.271	0.322	-0.081	-0.031	4.91E-124	2.82E-12
cg15412313	11	74801987	*OR2AT4*	0.776	0.818	0.827	0.042	0.051	1.40E-19	1.62E-13
cg01522592	11	75235311	*GDPD5*	0.733	0.788	0.753	0.055	0.020	3.55E-100	8.34E-09
cg17472111	11	75287721	NA	0.299	0.244	0.268	-0.055	-0.031	4.28E-62	1.21E-10
cg15587792	11	75584802	*UVRAG*	0.302	0.234	0.269	-0.069	-0.034	4.97E-108	2.22E-13
cg11274172	11	75736215	*UVRAG*	0.493	0.438	0.467	-0.055	-0.026	2.45E-45	2.44E-06
cg20430631	11	76340765	NA	0.405	0.305	0.359	-0.099	-0.046	6.31E-106	6.28E-14
cg13505393	11	76377572	*LRRC32*	0.345	0.284	0.318	-0.061	-0.027	3.14E-115	5.72E-13
cg23595413	11	76574308	*ACER3*	0.376	0.279	0.339	-0.097	-0.038	1.13E-134	2.40E-12
cg25948980	11	76777325	*CAPN5*	0.241	0.184	0.218	-0.058	-0.023	1.12E-76	1.71E-07
cg17355865	11	76887139	*MYO7A*	0.390	0.308	0.352	-0.082	-0.038	1.06E-135	7.65E-20
cg14630206	11	77121864	*PAK1*	0.474	0.406	0.438	-0.068	-0.036	3.87E-69	2.25E-11
cg07780377	11	77939490	*GAB2*	0.439	0.382	0.399	-0.057	-0.040	3.71E-58	4.69E-14
cg07441953	11	78400028	*ODZ4*	0.720	0.773	0.763	0.052	0.043	2.85E-24	2.45E-08
cg25837979	11	79114133	*ODZ4*	0.652	0.707	0.687	0.055	0.036	4.18E-35	8.17E-08
cg19321615	11	82972903	*CCDC90B*	0.694	0.746	0.739	0.051	0.045	1.73E-21	1.11E-07
cg15078975	11	85958536	*EED*	0.710	0.777	0.755	0.067	0.046	3.11E-36	2.38E-09
cg05705813	11	86383809	*ME3*	0.512	0.460	0.475	-0.052	-0.038	8.40E-30	3.62E-07
cg09416908	11	86384670	*ME3*	0.430	0.323	0.382	-0.107	-0.048	1.19E-116	2.15E-14
cg27095222	11	88090861	NA	0.271	0.211	0.236	-0.060	-0.035	3.38E-68	3.61E-12
cg24406240	11	88153520	NA	0.476	0.373	0.445	-0.103	-0.031	3.37E-131	1.27E-09
cg26068677	11	92282995	*FAT3*	0.694	0.752	0.748	0.058	0.054	2.80E-31	1.66E-12
cg07744502	11	93271083	*C11orf75*	0.297	0.243	0.267	-0.054	-0.030	3.00E-89	9.03E-17
cg15412815	11	93271088	*C11orf75*	0.336	0.257	0.299	-0.079	-0.037	3.32E-64	3.44E-07
cg01580228	11	93467095	NA	0.420	0.316	0.380	-0.104	-0.040	9.82E-137	5.33E-13
cg21208539	11	93864142	*PANX1*	0.358	0.278	0.318	-0.081	-0.040	1.65E-100	2.80E-15
cg06526620	11	94278324	*FUT4*	0.522	0.446	0.496	-0.076	-0.025	3.70E-75	1.82E-06
cg10283505	11	94278912	*FUT4*	0.523	0.428	0.482	-0.095	-0.041	8.06E-123	2.83E-15
cg13300301	11	94279068	*FUT4*	0.469	0.385	0.430	-0.083	-0.038	1.43E-80	1.74E-09
cg02856190	11	94282491	*FUT4*	0.424	0.317	0.374	-0.107	-0.051	2.91E-161	3.38E-23
cg16436762	11	94307971	*PIWIL4*	0.430	0.339	0.395	-0.091	-0.035	1.27E-86	2.69E-08
cg25371036	11	94500749	*AMOTL1*	0.743	0.685	0.698	-0.058	-0.046	5.29E-47	1.32E-18

cg09610792	11	94798056	NA	0.695	0.750	0.741	0.054	0.046	4.77E-27	2.45E-10
cg09527615	11	94883350	NA	0.548	0.485	0.507	-0.063	-0.041	6.47E-37	1.60E-09
cg20062681	11	94988642	NA	0.693	0.624	0.658	-0.070	-0.035	8.13E-92	6.07E-16
cg24941342	11	95093809	NA	0.419	0.305	0.370	-0.114	-0.049	9.22E-159	2.40E-19
cg07563725	11	95408124	NA	0.735	0.791	0.779	0.056	0.044	1.32E-31	1.49E-09
cg07530172	11	95431373	NA	0.341	0.258	0.298	-0.083	-0.043	8.53E-113	1.46E-16
cg04677410	11	95846117	*MAML2*	0.695	0.756	0.729	0.061	0.034	1.19E-36	2.44E-06
cg14296561	11	102203542	*BIRC3*	0.493	0.417	0.458	-0.076	-0.035	3.14E-85	3.16E-10
cg26207239	11	102231936	*BIRC2*	0.661	0.720	0.693	0.058	0.032	4.40E-110	1.51E-19
cg17094356	11	104789337	NA	0.643	0.701	0.683	0.057	0.040	1.79E-32	6.62E-08
cg19892525	11	108157498	*ATM*	0.621	0.663	0.686	0.042	0.065	5.09E-13	1.73E-13
cg07188863	11	110055425	*RDX*	0.398	0.323	0.372	-0.075	-0.026	1.51E-140	4.10E-13
cg10264003	11	110198965	NA	0.494	0.556	0.520	0.061	0.026	2.08E-61	6.49E-08
cg24504014	11	110297121	NA	0.555	0.461	0.526	-0.093	-0.028	8.67E-133	5.19E-09
cg24500630	11	110436181	NA	0.364	0.296	0.335	-0.068	-0.030	2.60E-73	3.36E-07
cg08142096	11	111125837	*C11orf53*	0.656	0.711	0.688	0.055	0.032	1.05E-38	3.83E-07
cg15418499	11	112028288	*IL18*	0.453	0.360	0.402	-0.093	-0.051	2.05E-106	1.87E-17
cg05687149	11	112035945	*IL18*	0.320	0.265	0.295	-0.055	-0.025	2.34E-56	3.60E-07
cg15818008	11	112126170	*C11orf34*	0.488	0.404	0.456	-0.084	-0.032	2.90E-67	3.17E-06
cg12445422	11	113920436	NA	0.534	0.464	0.493	-0.070	-0.041	7.67E-84	8.55E-16
cg04226002	11	113953462	*ZBTB16*	0.433	0.331	0.389	-0.102	-0.045	1.02E-134	1.52E-19
cg14596967	11	113956944	*ZBTB16*	0.496	0.421	0.460	-0.076	-0.037	2.10E-87	3.44E-12
cg19299755	11	116706051	NA	0.465	0.398	0.439	-0.067	-0.026	6.83E-82	1.11E-09
cg23193059	11	116706090	NA	0.456	0.354	0.425	-0.102	-0.032	5.16E-104	3.50E-06
cg03044513	11	116706153	NA	0.364	0.301	0.339	-0.063	-0.025	1.09E-85	3.05E-08
cg19160629	11	116742145	*SIK3*	0.347	0.281	0.313	-0.066	-0.034	8.56E-70	1.68E-09
cg08195176	11	116781303	*SIK3*	0.511	0.436	0.483	-0.075	-0.028	1.69E-119	1.51E-13
cg20608306	11	116969690	*SIK3*	0.264	0.210	0.243	-0.054	-0.021	1.63E-75	1.25E-06
cg05256304	11	116969870	*SIK3*	0.570	0.509	0.520	-0.061	-0.050	3.72E-41	1.80E-13
cg11054816	11	117183853	*BACE1*	0.455	0.361	0.420	-0.094	-0.034	1.23E-98	2.40E-08
cg01142676	11	117695591	*FXYD2*	0.311	0.230	0.274	-0.082	-0.037	9.96E-105	2.04E-12
cg15929395	11	117695828	*FXYD2*	0.424	0.343	0.394	-0.082	-0.030	4.89E-144	1.93E-15
cg02049955	11	118045910	*SCN2B*	0.457	0.390	0.425	-0.068	-0.032	1.75E-91	2.01E-11
cg03664391	11	118048212	*SCN2B*	0.702	0.753	0.754	0.051	0.052	4.17E-24	5.90E-12
cg27646075	11	118048216	*SCN2B*	0.715	0.767	0.766	0.052	0.051	4.62E-27	7.34E-12
cg07376232	11	118084920	*AMICA1*	0.218	0.166	0.191	-0.051	-0.027	7.40E-57	1.98E-08
cg13751113	11	118085214	*AMICA1*	0.266	0.205	0.241	-0.061	-0.025	3.23E-80	3.89E-08
cg25155064	11	118100782	*MPZL3*	0.653	0.724	0.692	0.071	0.039	2.42E-74	1.28E-11
cg21127597	11	118109650	*MPZL3*	0.398	0.306	0.369	-0.092	-0.029	9.58E-131	1.50E-09
cg25643644	11	118210017	*CD3D*	0.741	0.800	0.784	0.059	0.043	8.33E-58	3.23E-14
cg07728874	11	118213272	*CD3D*	0.744	0.797	0.771	0.053	0.027	4.12E-73	1.19E-11
cg05160234	11	118214762	*CD3G*	0.668	0.734	0.699	0.066	0.032	7.09E-91	4.62E-13
cg03254928	11	118214810	*CD3G*	0.642	0.708	0.669	0.066	0.026	3.99E-101	8.47E-12
cg15880738	11	118215112	*CD3G*	0.690	0.760	0.717	0.070	0.026	3.94E-90	1.27E-08
cg14081270	11	118286105	*LOC1001316*	0.300	0.226	0.267	-0.074	-0.033	2.46E-112	9.75E-13
cg03962678	11	118398094	*TTC36*	0.344	0.286	0.323	-0.059	-0.021	5.66E-88	5.03E-07
cg08473858	11	118476352	*PHLDB1*	0.391	0.326	0.360	-0.065	-0.031	2.74E-121	9.21E-20
cg05587627	11	118657363	*DDX6*	0.710	0.772	0.739	0.061	0.028	2.20E-73	2.59E-09
cg23575688	11	119486443	NA	0.336	0.258	0.309	-0.078	-0.027	1.14E-108	8.76E-09
cg01385882	11	120204978	NA	0.739	0.793	0.789	0.054	0.050	4.55E-23	3.04E-09
cg09321086	11	120941709	*TBCEL*	0.667	0.713	0.718	0.045	0.051	6.58E-17	3.14E-10
cg08669096	11	121229765	NA	0.331	0.245	0.280	-0.086	-0.052	1.08E-101	2.12E-19
cg19462635	11	121237843	NA	0.442	0.365	0.399	-0.077	-0.043	1.90E-77	6.25E-13
cg03055520	11	121460793	*SORL1*	0.288	0.230	0.255	-0.058	-0.033	5.77E-107	1.88E-18
cg20069407	11	121460973	*SORL1*	0.297	0.226	0.250	-0.071	-0.047	2.95E-74	9.36E-16
cg17510957	11	121466629	*SORL1*	0.645	0.565	0.610	-0.080	-0.035	2.22E-69	4.38E-08
cg18362003	11	122051630	*MIR100HG*	0.768	0.820	0.800	0.053	0.032	5.19E-41	1.56E-07
cg21170682	11	122205382	NA	0.462	0.356	0.412	-0.106	-0.049	8.17E-158	3.66E-26
cg03463948	11	122612858	*UBASH3B*	0.523	0.399	0.484	-0.124	-0.039	2.83E-155	5.74E-12
cg04174091	11	122935222	NA	0.583	0.644	0.622	0.061	0.039	4.15E-45	1.33E-10
cg05928849	11	122993208	*CLMP*	0.483	0.379	0.446	-0.104	-0.037	1.57E-167	7.86E-15
cg17280172	11	123082796	NA	0.729	0.785	0.787	0.056	0.058	2.31E-25	5.44E-13
cg11596902	11	123324834	NA	0.453	0.357	0.411	-0.097	-0.042	1.51E-102	6.97E-12
cg05868564	11	123351497	NA	0.232	0.175	0.206	-0.057	-0.026	2.83E-73	6.29E-08
cg19550524	11	124043067	NA	0.770	0.821	0.811	0.051	0.041	2.44E-32	7.42E-10
cg03056494	11	124595710	NA	0.347	0.274	0.308	-0.073	-0.040	1.17E-97	1.46E-16
cg20315690	11	124707157	NA	0.425	0.397	0.374	-0.028	-0.051	1.79E-07	2.33E-09
cg09684429	11	124768015	*ROBO4*	0.330	0.275	0.299	-0.055	-0.031	1.92E-80	1.86E-13
cg19008371	11	126226766	*ST3GAL4*	0.309	0.250	0.278	-0.059	-0.030	2.18E-75	2.29E-10
cg15339435	11	128169380	NA	0.731	0.781	0.753	0.050	0.022	1.28E-77	1.09E-08
cg25068347	11	128343784	*ETS1*	0.770	0.828	0.787	0.058	0.017	8.78E-107	4.44E-06
cg26404422	11	128367010	*ETS1*	0.627	0.699	0.679	0.073	0.052	8.49E-87	1.49E-22
cg23514374	11	128372328	*ETS1*	0.795	0.850	0.822	0.056	0.028	5.68E-95	1.92E-12
cg11760500	11	128385880	*ETS1*	0.756	0.825	0.793	0.069	0.036	6.90E-84	2.93E-11
cg04165073	11	128388475	*ETS1*	0.645	0.720	0.678	0.074	0.032	9.51E-121	1.10E-14
cg25578781	11	128446070	*ETS1*	0.345	0.270	0.306	-0.075	-0.039	1.42E-93	3.49E-13
cg13746740	11	128554939	*FLI1*	0.283	0.225	0.260	-0.057	-0.023	5.82E-79	1.77E-07
cg19896824	11	128555529	*FLI1*	0.360	0.303	0.338	-0.057	-0.022	6.35E-74	4.88E-07

cg02758236	11	128562003	*FLI1*	0.260	0.207	0.229	-0.053	-0.031	8.51E-46	8.31E-08
cg16024530	11	128673534	*FLI1*	0.309	0.257	0.284	-0.051	-0.024	2.57E-124	1.39E-17
cg21093670	11	129684147	NA	0.592	0.652	0.638	0.060	0.046	1.35E-33	3.78E-10
cg27255275	11	129766154	*NFRKB*	0.468	0.380	0.431	-0.089	-0.037	2.41E-87	3.89E-11
cg09586924	11	130032815	*ST14*	0.248	0.193	0.221	-0.055	-0.027	1.65E-82	5.46E-10
cg05079547	11	131239592	*NTM*	0.638	0.692	0.683	0.054	0.045	6.28E-26	1.32E-08
cg25187338	11	133229387	*OPCML*	0.741	0.787	0.793	0.046	0.051	2.21E-19	1.51E-10
cg27073337	11	133998086	*JAM3*	0.711	0.767	0.747	0.055	0.035	3.55E-32	9.61E-07
cg13255542	11	134120785	*THYN1*	0.458	0.359	0.425	-0.099	-0.034	3.26E-135	6.48E-11
cg19037107	11	134126323	*ACAD8*	0.603	0.496	0.578	-0.107	-0.024	7.63E-149	2.26E-06
cg09447621	11	134254646	*B3GAT1*	0.234	0.184	0.209	-0.051	-0.025	3.25E-63	6.49E-09
cg12601142	12	495079	*KDM5A*	0.665	0.716	0.709	0.051	0.044	1.13E-23	7.03E-09
cg11900393	12	721502	*NINJ2*	0.638	0.574	0.610	-0.064	-0.028	1.08E-50	2.24E-06
cg24804768	12	754911	*NINJ2*	0.434	0.355	0.391	-0.079	-0.043	1.20E-98	1.70E-18
cg12013258	12	776351	NA	0.698	0.749	0.729	0.051	0.031	8.65E-37	3.70E-07
cg06708720	12	1099075	*ERC1*	0.521	0.457	0.466	-0.065	-0.055	8.84E-88	8.07E-29
cg21648069	12	1157718	*ERC1*	0.437	0.352	0.408	-0.085	-0.029	3.99E-77	4.09E-06
cg26363039	12	1609549	NA	0.257	0.195	0.231	-0.063	-0.026	5.02E-71	8.45E-07
cg17150898	12	1702116	*FBXL14*	0.592	0.666	0.617	0.074	0.024	5.26E-79	1.27E-06
cg01268901	12	1744957	*WNT5B*	0.423	0.321	0.379	-0.102	-0.045	1.73E-177	5.39E-25
cg01924292	12	1815334	*ADIPOR2*	0.499	0.386	0.462	-0.112	-0.037	2.02E-153	1.62E-11
cg21602614	12	1949586	*CACNA2D4*	0.242	0.191	0.217	-0.051	-0.025	2.71E-52	8.86E-07
cg17737835	12	2027805	*CACNA2D4*	0.435	0.369	0.397	-0.066	-0.038	2.05E-85	1.56E-15
cg19369955	12	2030178	NA	0.253	0.193	0.224	-0.060	-0.030	3.55E-93	3.92E-13
cg22250531	12	2030200	NA	0.248	0.190	0.223	-0.058	-0.026	1.08E-91	9.23E-11
cg16303353	12	2393684	*CACNA1C*	0.408	0.310	0.363	-0.099	-0.045	1.78E-126	1.72E-15
cg03626208	12	2443169	*CACNA1C*	0.425	0.321	0.371	-0.104	-0.054	9.85E-139	2.07E-21
cg16728539	12	2451169	*CACNA1C*	0.426	0.329	0.384	-0.097	-0.042	3.91E-119	1.65E-14
cg13371705	12	2452955	*CACNA1C*	0.440	0.354	0.397	-0.086	-0.043	1.11E-88	9.78E-13
cg05824594	12	2734503	*CACNA1C*	0.352	0.296	0.324	-0.056	-0.028	6.84E-61	2.95E-09
cg11958128	12	3226445	*TSPAN9*	0.274	0.216	0.248	-0.058	-0.026	1.71E-72	8.42E-09
cg23702046	12	3371110	*TSPAN9*	0.608	0.544	0.577	-0.064	-0.031	2.28E-49	1.57E-07
cg14834893	12	4398032	*CCND2*	0.355	0.276	0.322	-0.079	-0.033	1.54E-99	2.17E-10
cg27317813	12	4398508	*CCND2*	0.324	0.246	0.298	-0.078	-0.026	3.02E-100	3.18E-07
cg09799980	12	4398618	*CCND2*	0.334	0.251	0.298	-0.083	-0.036	2.30E-135	2.60E-16
cg06270401	12	4699085	*DYRK4*	0.484	0.372	0.440	-0.113	-0.044	6.61E-177	2.46E-19
cg09418321	12	4699618	*DYRK4*	0.413	0.333	0.376	-0.080	-0.037	2.91E-110	4.80E-13
cg22222281	12	6308758	*CD9*	0.449	0.378	0.415	-0.071	-0.034	8.68E-61	1.19E-08
cg08519905	12	6308774	*CD9*	0.397	0.340	0.361	-0.057	-0.037	5.78E-57	2.57E-12
cg08418872	12	6442954	*TNFRSF1A*	0.688	0.748	0.716	0.060	0.028	2.74E-62	1.67E-08
cg16846069	12	6462681	*SCNN1A*	0.432	0.351	0.401	-0.081	-0.031	2.20E-107	3.01E-11
cg26215727	12	6485537	*SCNN1A*	0.470	0.388	0.445	-0.082	-0.025	9.33E-122	1.15E-07
cg24621362	12	6492890	*LTBR*	0.312	0.245	0.275	-0.067	-0.037	3.50E-94	1.12E-14
cg19476647	12	6492948	*LTBR*	0.336	0.259	0.305	-0.077	-0.030	1.22E-103	2.18E-10
cg23079808	12	6493003	*LTBR*	0.345	0.250	0.310	-0.095	-0.034	4.52E-122	1.22E-09
cg26306994	12	6493521	*LTBR*	0.415	0.349	0.381	-0.066	-0.033	9.48E-93	8.54E-14
cg13298466	12	6658164	*IFFO1*	0.579	0.463	0.535	-0.116	-0.044	1.72E-123	1.34E-11
cg21638533	12	6658625	*IFFO1*	0.364	0.269	0.336	-0.095	-0.028	9.10E-119	4.94E-07
cg13549277	12	6659520	*IFFO1*	0.397	0.325	0.370	-0.072	-0.027	3.30E-142	8.14E-14
cg10838410	12	6659524	*IFFO1*	0.262	0.199	0.233	-0.062	-0.029	1.25E-83	8.80E-11
cg12024104	12	6662581	*IFFO1*	0.508	0.430	0.477	-0.078	-0.030	3.95E-86	2.48E-09
cg24392132	12	6753626	*ACRBP*	0.234	0.182	0.211	-0.052	-0.023	4.64E-78	1.01E-08
cg19421125	12	6882856	*LAG3*	0.278	0.238	0.226	-0.040	-0.052	5.34E-11	2.24E-08
cg26538140	12	6996791	NA	0.391	0.300	0.362	-0.091	-0.029	6.77E-120	8.07E-08
cg24437859	12	7066614	*PTPN6*	0.727	0.799	0.751	0.072	0.025	4.32E-87	3.62E-06
cg16642299	12	7071383	NA	0.325	0.258	0.293	-0.066	-0.031	1.45E-56	1.35E-06
cg16959747	12	7276714	*RBP5*	0.464	0.409	0.438	-0.055	-0.026	3.11E-46	2.21E-06
cg23590660	12	8205602	*FOXJ2*	0.323	0.241	0.284	-0.082	-0.038	1.35E-109	7.26E-13
cg12690978	12	8801526	*MFAP5*	0.761	0.820	0.781	0.059	0.020	1.68E-91	3.61E-07
cg10021941	12	8841938	NA	0.629	0.684	0.683	0.056	0.054	3.32E-23	1.10E-10
cg09912793	12	9066382	*PHC1*	0.701	0.648	0.659	-0.053	-0.042	4.34E-35	1.35E-12
cg13955347	12	9106586	NA	0.538	0.614	0.579	0.076	0.040	6.76E-51	3.80E-08
cg26806779	12	9141775	*KLRG1*	0.436	0.372	0.400	-0.064	-0.036	2.89E-63	1.59E-10
cg13553498	12	9821986	*CLEC2D*	0.718	0.790	0.767	0.072	0.049	2.86E-54	1.35E-12
cg11905488	12	10123604	*CLEC12A*	0.547	0.471	0.517	-0.076	-0.030	6.09E-76	2.45E-07
cg21250433	12	10283763	*CLEC7A*	0.628	0.572	0.598	-0.056	-0.031	7.47E-55	5.04E-10
cg21249659	12	10324843	*OLR1*	0.446	0.335	0.396	-0.111	-0.050	2.46E-169	5.04E-27
cg21685770	12	10324918	*OLR1*	0.517	0.403	0.481	-0.114	-0.036	7.48E-144	9.81E-12
cg05377120	12	10456885	*KLRD1*	0.767	0.826	0.790	0.059	0.023	5.93E-100	2.79E-09
cg01293346	12	11149362	*PRH1-PRR4*	0.614	0.666	0.671	0.052	0.057	4.42E-19	2.07E-10
cg14830166	12	11821908	*ETV6*	0.330	0.258	0.294	-0.071	-0.036	5.10E-93	1.40E-13
cg14693090	12	11899281	*ETV6*	0.363	0.276	0.336	-0.087	-0.027	4.90E-109	7.12E-07
cg23738760	12	11918643	*ETV6*	0.457	0.407	0.415	-0.050	-0.042	2.26E-32	4.82E-11
cg18907610	12	11992925	*RNU6-19*	0.417	0.322	0.364	-0.095	-0.053	7.21E-127	3.63E-20
cg20618695	12	13251495	*GSG1*	0.290	0.222	0.265	-0.068	-0.024	3.02E-85	4.59E-07
cg12371569	12	13278271	NA	0.253	0.193	0.219	-0.060	-0.034	1.07E-65	2.13E-10
cg20184271	12	14413090	NA	0.484	0.387	0.447	-0.097	-0.038	2.52E-118	3.33E-13
cg06872313	12	14413185	NA	0.426	0.348	0.398	-0.077	-0.027	2.59E-93	1.26E-08

cg05360958	12	15038440	*MGP*	0.379	0.320	0.338	-0.059	-0.041	1.87E-37	5.46E-09
cg13302154	12	15039432	*MGP*	0.451	0.390	0.401	-0.061	-0.050	2.94E-31	3.92E-11
cg05194864	12	15100977	*ARHGDIB*	0.618	0.683	0.669	0.065	0.051	7.67E-36	1.35E-12
cg01591037	12	15134481	*PDE6H*	0.485	0.371	0.450	-0.114	-0.034	1.89E-135	2.42E-09
cg24522654	12	19535154	NA	0.700	0.762	0.727	0.062	0.027	8.18E-83	8.37E-10
cg21913301	12	20677257	*PDE3A*	0.687	0.743	0.721	0.056	0.034	1.25E-47	3.28E-09
cg25885914	12	20906250	*SLCO1C1*	0.729	0.779	0.781	0.051	0.052	2.89E-22	1.01E-10
cg20759281	12	21796914	*LDHB*	0.284	0.222	0.255	-0.061	-0.029	3.89E-67	5.02E-08
cg21005240	12	22688743	*KIAA0528*	0.651	0.716	0.699	0.065	0.048	2.59E-37	4.26E-10
cg10456459	12	22843015	*ETNK1*	0.507	0.384	0.471	-0.123	-0.036	2.82E-171	9.63E-13
cg24011341	12	24577904	*SOX5*	0.741	0.799	0.778	0.058	0.037	1.60E-61	2.65E-13
cg27093403	12	25083077	*BCAT1*	0.514	0.463	0.491	-0.051	-0.023	4.28E-56	3.93E-09
cg12990174	12	25359932	*KRAS*	0.747	0.789	0.798	0.042	0.050	2.49E-15	4.39E-10
cg13134916	12	25707564	*IFLTD1*	0.399	0.312	0.369	-0.088	-0.030	3.06E-85	7.75E-07
cg24850296	12	25707569	*IFLTD1*	0.341	0.275	0.315	-0.067	-0.026	1.33E-66	6.02E-07
cg07643696	12	25844221	NA	0.419	0.346	0.384	-0.073	-0.035	7.43E-75	1.36E-09
cg07172007	12	26133416	*RASSF8*	0.689	0.740	0.741	0.051	0.052	4.19E-24	2.71E-11
cg09010707	12	26392567	NA	0.387	0.322	0.354	-0.065	-0.033	1.61E-113	3.56E-18
cg12651154	12	26820570	*ITPR2*	0.795	0.836	0.846	0.042	0.051	7.01E-18	5.19E-12
cg25338454	12	26900022	*ITPR2*	0.614	0.538	0.582	-0.077	-0.032	7.54E-90	4.90E-10
cg23202253	12	26902211	*ITPR2*	0.760	0.811	0.790	0.051	0.030	6.20E-39	5.95E-07
cg24957609	12	27125095	*TM7SF3*	0.481	0.552	0.525	0.071	0.044	8.63E-65	1.60E-14
cg04904784	12	27170664	NA	0.540	0.452	0.512	-0.088	-0.028	1.85E-103	2.54E-07
cg01982833	12	27174596	*MED21*	0.422	0.345	0.381	-0.077	-0.041	1.85E-52	1.52E-08
cg17419815	12	27234924	*C12orf71*	0.593	0.480	0.555	-0.113	-0.038	3.26E-110	1.17E-09
cg05171937	12	27396765	*STK38L*	0.538	0.418	0.487	-0.120	-0.051	1.56E-134	3.55E-15
cg23787937	12	29303160	NA	0.268	0.200	0.239	-0.067	-0.029	1.02E-59	3.15E-06
cg07684519	12	29303184	NA	0.281	0.199	0.246	-0.083	-0.036	1.88E-87	8.11E-09
cg05697976	12	29376483	*FAR2*	0.404	0.305	0.367	-0.099	-0.037	7.17E-107	8.35E-10
cg14788046	12	31402470	NA	0.497	0.444	0.464	-0.053	-0.033	8.80E-31	2.47E-07
cg06640997	12	32081573	NA	0.736	0.797	0.765	0.060	0.029	1.13E-73	3.69E-11
cg01871722	12	32120103	*KIAA1551*	0.776	0.832	0.806	0.056	0.030	5.64E-65	3.56E-10
cg18464274	12	42537617	*GXYLT1*	0.388	0.330	0.348	-0.058	-0.040	1.35E-53	6.93E-12
cg01821022	12	42775908	*PPHLN1*	0.395	0.306	0.365	-0.089	-0.030	2.17E-121	4.39E-09
cg02372037	12	42865986	*PRICKLE1*	0.774	0.819	0.825	0.045	0.051	4.92E-22	5.28E-13
cg21185662	12	45662797	*ANO6*	0.450	0.388	0.425	-0.062	-0.025	1.92E-62	2.84E-06
cg07062336	12	45970082	NA	0.305	0.247	0.276	-0.058	-0.029	3.82E-67	1.53E-08
cg10617091	12	46273255	*ARID2*	0.699	0.747	0.753	0.048	0.054	1.48E-19	9.55E-12
cg09050775	12	46762708	*SLC38A2*	0.639	0.707	0.677	0.068	0.038	1.25E-64	5.32E-10
cg16788865	12	46778801	NA	0.432	0.332	0.387	-0.100	-0.045	4.17E-103	1.16E-13
cg01023672	12	47477223	NA	0.454	0.349	0.421	-0.105	-0.033	4.18E-111	3.06E-07
cg09169413	12	48103670	*ENDOU*	0.424	0.359	0.389	-0.065	-0.035	1.71E-67	2.37E-11
cg23815853	12	48147034	*RAPGEF3*	0.540	0.424	0.488	-0.117	-0.052	1.53E-135	4.28E-19
cg04381888	12	48195549	*HDAC7*	0.314	0.253	0.284	-0.060	-0.029	1.47E-93	4.56E-13
cg01088404	12	48214523	*HDAC7*	0.387	0.292	0.338	-0.095	-0.049	4.71E-89	1.01E-13
cg05190176	12	48290002	*VDR*	0.527	0.466	0.502	-0.061	-0.024	4.03E-61	3.66E-06
cg10508111	12	48515382	*PFKM*	0.711	0.759	0.764	0.049	0.054	9.34E-21	3.36E-11
cg09386303	12	48551862	*ASB8*	0.711	0.757	0.762	0.046	0.051	2.38E-19	3.35E-11
cg05580141	12	49071788	*KANSL2*	0.506	0.394	0.454	-0.112	-0.052	1.19E-132	4.35E-19
cg05581469	12	49413435	*PRKAG1*	0.422	0.321	0.382	-0.101	-0.040	5.08E-140	1.42E-14
cg21192452	12	49593546	NA	0.736	0.767	0.787	0.031	0.051	1.81E-12	3.31E-13
cg15661753	12	49627010	NA	0.307	0.231	0.266	-0.076	-0.041	8.29E-86	6.82E-13
cg15244101	12	49627624	NA	0.350	0.269	0.319	-0.081	-0.031	5.14E-114	7.18E-11
cg01923089	12	49627746	NA	0.343	0.271	0.314	-0.072	-0.029	1.97E-101	5.81E-11
cg04070601	12	50067887	*FMNL3*	0.694	0.757	0.730	0.063	0.035	6.40E-115	8.73E-20
cg22032626	12	50203811	*NCKAP5L*	0.381	0.311	0.345	-0.070	-0.037	3.66E-142	1.09E-30
cg14405137	12	50219283	*NCKAP5L*	0.329	0.252	0.293	-0.077	-0.037	8.37E-108	3.45E-14
cg19055828	12	51139321	*DIP2B*	0.516	0.396	0.490	-0.120	-0.027	3.64E-157	6.10E-07
cg22163059	12	51610927	*POU6F1*	0.363	0.309	0.336	-0.054	-0.027	4.06E-57	4.31E-08
cg24637417	12	51636921	*DAZAP2*	0.387	0.286	0.347	-0.101	-0.040	2.43E-157	3.92E-17
cg19265103	12	51784644	*GALNT6*	0.252	0.201	0.227	-0.052	-0.025	7.61E-75	7.83E-10
cg15029631	12	52078762	*SCN8A*	0.484	0.383	0.450	-0.101	-0.033	1.09E-121	5.78E-09
cg22566906	12	52399713	*GRASP*	0.268	0.215	0.235	-0.052	-0.033	1.96E-63	6.36E-13
cg14242936	12	52404134	*GRASP*	0.441	0.373	0.400	-0.069	-0.041	2.30E-86	1.26E-17
cg19374752	12	52404151	*GRASP*	0.513	0.453	0.474	-0.060	-0.039	6.53E-87	6.43E-22
cg06611426	12	52404161	*GRASP*	0.472	0.397	0.427	-0.075	-0.045	3.96E-82	1.89E-18
cg20479688	12	53046513	*KRT2*	0.735	0.787	0.774	0.052	0.039	3.96E-30	2.03E-08
cg13937905	12	53612551	*RARG*	0.854	0.906	0.897	0.052	0.043	3.14E-64	4.55E-20
cg11606261	12	53775336	*SP1*	0.323	0.237	0.298	-0.086	-0.025	1.33E-134	7.75E-08
cg08730245	12	53902893	*NPFF*	0.592	0.509	0.561	-0.083	-0.031	2.66E-121	1.27E-10
cg03998264	12	53932355	*ATF7*	0.401	0.290	0.355	-0.111	-0.046	5.74E-168	3.09E-18
cg08445469	12	53970794	*ATF7*	0.502	0.451	0.473	-0.051	-0.029	2.98E-54	7.82E-11
cg08269188	12	54655894	*CBX5*	0.248	0.187	0.210	-0.061	-0.038	6.70E-88	5.17E-18
cg09303642	12	54690818	*NFE2*	0.442	0.339	0.400	-0.103	-0.042	3.47E-149	4.27E-16
cg22974630	12	54733883	*COPZ1*	0.611	0.662	0.665	0.051	0.054	3.14E-16	4.57E-09
cg21163444	12	54765670	*ZNF385A*	0.453	0.381	0.428	-0.073	-0.026	1.68E-119	6.89E-12
cg22824738	12	54765988	*ZNF385A*	0.303	0.241	0.278	-0.062	-0.025	2.15E-121	2.12E-12
cg17465423	12	54784180	*ZNF385A*	0.338	0.282	0.307	-0.056	-0.031	2.51E-51	4.37E-09

cg20909017	12	54805345	*ITGA5*	0.298	0.228	0.270	-0.069	-0.028	5.03E-118	7.87E-15
cg10821261	12	54822725	NA	0.352	0.289	0.322	-0.062	-0.030	3.36E-82	3.04E-10
cg10659811	12	54833402	NA	0.494	0.418	0.445	-0.076	-0.048	1.07E-105	4.53E-22
cg21072795	12	54920931	*NCKAP1L*	0.357	0.293	0.322	-0.063	-0.035	5.77E-59	3.20E-10
cg01874152	12	55375859	*TESPA1*	0.757	0.811	0.799	0.054	0.041	1.19E-36	2.17E-10
cg04606020	12	56139467	*GDF11*	0.392	0.326	0.357	-0.066	-0.035	4.93E-56	8.45E-09
cg17865265	12	56236845	*MMP19*	0.610	0.553	0.580	-0.057	-0.030	5.87E-61	3.13E-09
cg03509949	12	56236869	*MMP19*	0.541	0.469	0.499	-0.073	-0.042	3.21E-92	3.26E-17
cg08338281	12	56322667	*WIBG*	0.653	0.724	0.688	0.070	0.035	1.77E-86	5.82E-12
cg07679948	12	56329641	*DGKA*	0.795	0.854	0.831	0.059	0.035	1.30E-85	3.77E-15
cg24414325	12	56414442	*IKZF4*	0.465	0.365	0.432	-0.100	-0.032	5.42E-132	1.04E-10
cg00026033	12	56414490	*IKZF4*	0.380	0.298	0.353	-0.083	-0.028	3.82E-169	3.03E-17
cg01565774	12	56414533	*IKZF4*	0.275	0.216	0.250	-0.059	-0.025	2.20E-134	2.41E-14
cg16268734	12	56690194	*CS*	0.497	0.395	0.462	-0.102	-0.036	3.19E-144	3.67E-12
cg09107438	12	56819060	*TIMELESS*	0.625	0.675	0.655	0.050	0.030	2.50E-28	6.29E-06
cg12614630	12	57387318	*GPR182*	0.497	0.392	0.455	-0.105	-0.042	1.56E-127	5.48E-12
cg10233454	12	57529389	*LRP1*	0.437	0.358	0.392	-0.080	-0.046	8.13E-86	8.09E-16
cg17651972	12	57620054	*NXPH4*	0.458	0.392	0.427	-0.066	-0.031	5.33E-63	5.86E-08
cg05758804	12	57917389	*MBD6*	0.295	0.243	0.271	-0.052	-0.024	2.42E-99	7.82E-12
cg16834726	12	57937408	*DCTN2*	0.504	0.447	0.477	-0.057	-0.027	1.10E-48	2.20E-06
cg17332198	12	58209913	*AVIL*	0.497	0.445	0.471	-0.051	-0.025	1.67E-60	1.56E-08
cg04046364	12	58210661	*AVIL*	0.392	0.296	0.358	-0.095	-0.033	1.85E-133	6.71E-12
cg26620147	12	58210716	*AVIL*	0.348	0.274	0.316	-0.074	-0.032	2.05E-74	1.85E-08
cg07000713	12	62463441	*FAM19A2*	0.735	0.792	0.764	0.057	0.030	1.63E-55	1.67E-07
cg03726817	12	62650767	NA	0.269	0.204	0.247	-0.065	-0.022	1.89E-76	5.62E-06
cg03919488	12	63179691	*PPM1H*	0.444	0.370	0.419	-0.074	-0.026	1.57E-88	3.67E-07
cg10524576	12	63322644	*PPM1H*	0.318	0.267	0.284	-0.051	-0.035	1.10E-57	3.60E-14
cg12707346	12	64960957	NA	0.407	0.316	0.364	-0.092	-0.044	2.63E-124	1.44E-16
cg23378033	12	65672031	*MSRB3*	0.459	0.359	0.408	-0.100	-0.051	5.36E-71	5.03E-11
cg15175129	12	65672052	*MSRB3*	0.333	0.270	0.291	-0.064	-0.042	1.86E-57	5.16E-12
cg02534850	12	66228466	*HMGA2*	0.729	0.788	0.765	0.060	0.036	4.27E-46	1.61E-08
cg12866960	12	66635398	*IRAK3*	0.501	0.389	0.466	-0.112	-0.035	7.20E-127	6.22E-10
cg16011250	12	68024459	NA	0.564	0.615	0.604	0.051	0.040	1.99E-29	7.87E-10
cg05224770	12	68553819	*IFNG*	0.669	0.742	0.712	0.074	0.044	4.49E-59	8.25E-11
cg24596116	12	68665317	NA	0.400	0.328	0.373	-0.072	-0.027	4.23E-103	2.55E-09
cg04697624	12	69002178	NA	0.333	0.259	0.299	-0.074	-0.034	2.47E-99	1.51E-11
cg25194194	12	69143431	*SLC35E3*	0.495	0.432	0.471	-0.064	-0.024	8.61E-90	1.18E-08
cg02266731	12	69357333	*CPM*	0.338	0.262	0.313	-0.077	-0.026	1.86E-98	1.26E-07
cg19563525	12	69909397	*FRS2*	0.701	0.752	0.745	0.051	0.044	2.94E-35	1.54E-12
cg04609875	12	70763703	*KCNMB4*	0.701	0.752	0.736	0.051	0.035	1.71E-32	2.12E-08
cg19409546	12	72477363	NA	0.697	0.747	0.731	0.050	0.034	1.44E-30	2.97E-07
cg26244575	12	76354015	NA	0.592	0.682	0.627	0.090	0.035	1.20E-139	1.09E-14
cg10502324	12	76372038	NA	0.562	0.628	0.586	0.065	0.023	7.40E-69	1.77E-06
cg18038207	12	80581062	NA	0.468	0.405	0.423	-0.063	-0.045	2.67E-55	2.32E-13
cg23794135	12	81190974	NA	0.783	0.830	0.836	0.046	0.052	2.40E-21	3.81E-12
cg17824939	12	88421705	*C12orf50*	0.696	0.744	0.751	0.048	0.055	2.60E-16	5.84E-10
cg03614132	12	89453916	NA	0.377	0.317	0.348	-0.060	-0.029	3.99E-49	5.35E-06
cg06655216	12	89619329	NA	0.703	0.760	0.733	0.057	0.029	6.93E-66	3.32E-09
cg02806156	12	89728752	NA	0.480	0.358	0.438	-0.122	-0.042	2.48E-163	9.68E-14
cg12045443	12	89777963	NA	0.357	0.273	0.326	-0.084	-0.031	2.69E-131	1.80E-12
cg16962463	12	89968675	NA	0.303	0.243	0.275	-0.060	-0.029	1.84E-53	1.39E-06
cg00186909	12	93129288	*PLEKHG7*	0.397	0.333	0.357	-0.065	-0.040	2.00E-46	3.69E-09
cg20121012	12	93516072	*LOC643339*	0.570	0.490	0.545	-0.080	-0.024	4.58E-108	9.56E-08
cg00593607	12	93530780	*LOC643339*	0.464	0.354	0.418	-0.110	-0.045	4.37E-141	2.13E-15
cg07051833	12	93808395	*UBE2N*	0.388	0.314	0.360	-0.074	-0.028	2.16E-125	3.40E-12
cg06630241	12	93967711	*SOCS2*	0.399	0.317	0.358	-0.083	-0.041	4.99E-67	1.15E-08
cg25823926	12	94153360	*CRADD*	0.321	0.239	0.270	-0.082	-0.051	4.87E-125	4.03E-26
cg25006194	12	94288553	NA	0.323	0.251	0.300	-0.073	-0.023	2.34E-96	5.38E-07
cg12999267	12	94376970	NA	0.346	0.414	0.384	0.068	0.038	1.17E-29	3.66E-06
cg12877335	12	94539319	NA	0.450	0.393	0.414	-0.057	-0.036	9.83E-86	8.22E-20
cg26514961	12	94566784	*PLXNC1*	0.444	0.347	0.410	-0.097	-0.034	1.44E-103	5.61E-08
cg25367249	12	96127072	*NTN4*	0.679	0.728	0.735	0.049	0.056	8.21E-23	2.33E-13
cg22916254	12	96256561	*SNRPF*	0.615	0.680	0.662	0.065	0.047	3.00E-44	2.32E-11
cg25137372	12	96390059	*HAL*	0.365	0.287	0.331	-0.077	-0.034	4.62E-93	1.32E-11
cg18817487	12	96390143	*HAL*	0.229	0.177	0.197	-0.052	-0.032	1.65E-43	4.04E-08
cg02927448	12	100532162	*UHRF1BP1L*	0.352	0.281	0.318	-0.071	-0.034	6.55E-90	4.00E-11
cg13135241	12	104765292	NA	0.404	0.321	0.367	-0.083	-0.037	1.12E-118	1.68E-15
cg06647068	12	104853274	*CHST11*	0.399	0.348	0.341	-0.051	-0.059	7.46E-34	2.70E-20
cg11721178	12	106010541	NA	0.724	0.775	0.771	0.051	0.047	3.18E-33	2.28E-13
cg01797169	12	107711489	*BTBD11*	0.355	0.275	0.312	-0.080	-0.042	9.76E-89	8.17E-14
cg17208953	12	108054842	NA	0.582	0.502	0.550	-0.081	-0.032	2.44E-90	1.09E-08
cg01399379	12	108298374	NA	0.749	0.788	0.800	0.039	0.050	2.38E-16	2.32E-12
cg00159243	12	109023799	*SELPLG*	0.325	0.273	0.299	-0.052	-0.026	2.64E-58	1.63E-09
cg05233670	12	109058033	*CORO1C*	0.426	0.359	0.390	-0.067	-0.036	5.23E-70	3.27E-11
cg17446956	12	109235032	*SSH1*	0.543	0.455	0.519	-0.088	-0.024	6.96E-111	5.91E-06
cg26508200	12	109235071	*SSH1*	0.572	0.458	0.531	-0.115	-0.041	1.28E-132	3.77E-13
cg08866695	12	109596187	*ACACB*	0.328	0.263	0.303	-0.065	-0.024	5.01E-74	2.14E-06
cg22274234	12	109888858	*KCTD10*	0.343	0.282	0.315	-0.060	-0.028	9.86E-88	1.17E-12

cg22742001	12	110435418	*GIT2*	0.410	0.305	0.365	-0.105	-0.045	1.10E-126	4.11E-15
cg11025609	12	110727072	*ATP2A2*	0.681	0.737	0.734	0.055	0.053	1.98E-30	3.62E-13
cg23807570	12	110787107	*ATP2A2*	0.476	0.371	0.437	-0.105	-0.039	2.76E-137	1.50E-12
cg20547015	12	111165713	*PPP1CC*	0.748	0.802	0.776	0.054	0.028	8.60E-44	1.48E-06
cg20595271	12	111889200	*SH2B3*	0.592	0.646	0.629	0.054	0.037	3.71E-34	1.51E-08
cg20884605	12	112205368	*ALDH2*	0.292	0.233	0.272	-0.059	-0.020	4.98E-85	1.22E-06
cg16868784	12	112447736	*TMEM116*	0.753	0.798	0.806	0.045	0.053	4.75E-21	3.78E-13
cg04951822	12	113345598	*OAS1*	0.424	0.363	0.337	-0.061	-0.087	1.19E-29	7.42E-25
cg25800166	12	113375896	*OAS3*	0.605	0.541	0.569	-0.063	-0.035	1.92E-43	4.79E-09
cg19371652	12	113415883	*OAS2*	0.333	0.264	0.266	-0.069	-0.068	1.08E-44	2.90E-29
cg10877430	12	113527336	*DTX1*	0.489	0.558	0.507	0.070	0.018	2.11E-116	1.50E-08
cg01620165	12	113530915	*DTX1*	0.364	0.301	0.328	-0.063	-0.035	6.10E-67	4.85E-12
cg21593001	12	113531060	*DTX1*	0.405	0.345	0.376	-0.060	-0.028	4.40E-76	1.18E-12
cg01616876	12	113544928	*RASAL1*	0.309	0.237	0.268	-0.072	-0.041	1.01E-73	5.33E-13
cg00859441	12	113799660	*PLBD2*	0.552	0.446	0.514	-0.106	-0.039	1.06E-116	2.55E-10
cg04831327	12	114299076	*RBM19*	0.477	0.373	0.433	-0.103	-0.044	1.06E-183	3.03E-23
cg07043361	12	114404971	*RBM19*	0.393	0.300	0.351	-0.093	-0.042	3.46E-97	1.23E-11
cg12136387	12	116820108	NA	0.374	0.293	0.339	-0.081	-0.035	6.16E-124	2.14E-14
cg23975840	12	117042895	NA	0.625	0.527	0.594	-0.098	-0.030	2.45E-79	3.73E-06
cg00355804	12	117103567	NA	0.541	0.478	0.505	-0.063	-0.036	9.24E-47	1.55E-08
cg14224600	12	117477926	*TESC*	0.498	0.432	0.472	-0.066	-0.026	7.39E-74	1.76E-08
cg17627898	12	118782453	*TAOK3*	0.409	0.323	0.384	-0.086	-0.025	4.02E-100	6.05E-06
cg01015899	12	120663812	*PXN*	0.327	0.268	0.305	-0.059	-0.021	1.75E-82	4.44E-07
cg04256697	12	120688557	*PXN*	0.568	0.646	0.612	0.078	0.044	9.60E-64	1.39E-11
cg16942632	12	121726240	*CAMKK2*	0.449	0.354	0.409	-0.095	-0.039	5.92E-83	8.36E-09
cg21340621	12	122020166	*KDM2B*	0.346	0.286	0.325	-0.060	-0.021	2.28E-74	5.35E-06
cg08059719	12	122444450	NA	0.701	0.775	0.733	0.074	0.033	3.45E-134	3.13E-16
cg14480046	12	122444580	NA	0.707	0.781	0.734	0.074	0.027	1.58E-132	7.30E-12
cg23474890	12	122467179	*BCL7A*	0.700	0.776	0.734	0.076	0.034	3.75E-69	8.46E-08
cg22729539	12	122519119	*MLXIP*	0.221	0.161	0.198	-0.061	-0.023	2.99E-83	6.35E-07
cg08120831	12	122669186	*LRRC43*	0.295	0.236	0.275	-0.059	-0.021	1.49E-81	6.28E-07
cg10802680	12	122712075	*DIABLO*	0.633	0.691	0.657	0.059	0.025	1.10E-80	1.93E-09
cg14094409	12	122712093	*DIABLO*	0.691	0.765	0.719	0.074	0.028	6.16E-125	5.18E-12
cg19529732	12	122712101	*DIABLO*	0.539	0.626	0.569	0.086	0.030	1.07E-95	1.54E-07
cg21805118	12	122712153	*DIABLO*	0.698	0.765	0.725	0.067	0.027	1.43E-108	9.38E-11
cg25190513	12	123201362	*HCAR3*	0.563	0.451	0.526	-0.112	-0.037	5.42E-96	4.72E-08
cg15545247	12	123201372	*HCAR3*	0.496	0.377	0.440	-0.119	-0.056	3.75E-173	9.26E-29
cg17346246	12	123214864	*HCAR1*	0.789	0.851	0.828	0.061	0.039	1.48E-95	2.08E-20
cg00357958	12	123215010	*HCAR1*	0.583	0.662	0.612	0.079	0.029	3.59E-94	1.64E-08
cg05290737	12	123215151	*HCAR1*	0.703	0.761	0.732	0.058	0.029	9.35E-66	1.13E-09
cg18637761	12	123468780	*PITPNM2*	0.240	0.185	0.211	-0.055	-0.029	3.64E-109	1.03E-18
cg24967811	12	123503709	*PITPNM2*	0.420	0.301	0.369	-0.119	-0.051	1.24E-185	3.29E-25
cg05384139	12	123548027	*PITPNM2*	0.341	0.283	0.310	-0.058	-0.031	3.67E-93	1.72E-19
cg05132828	12	123560479	*PITPNM2*	0.576	0.653	0.601	0.078	0.025	3.09E-145	1.76E-13
cg23825057	12	124014409	*RILPL1*	0.491	0.405	0.465	-0.086	-0.026	1.55E-106	1.32E-06
cg22135566	12	124416315	*DNAH10*	0.694	0.754	0.720	0.061	0.026	4.42E-88	3.54E-10
cg04930596	12	124864528	*NCOR2*	0.447	0.370	0.419	-0.077	-0.028	1.39E-103	2.21E-09
cg07241090	12	124864594	*NCOR2*	0.380	0.298	0.348	-0.082	-0.032	2.66E-133	2.51E-14
cg17387577	12	124864657	*NCOR2*	0.371	0.297	0.345	-0.075	-0.027	3.03E-87	1.11E-07
cg17825194	12	124864681	*NCOR2*	0.375	0.305	0.350	-0.069	-0.024	1.41E-112	6.73E-11
cg26406891	12	125002940	*NCOR2*	0.327	0.262	0.294	-0.065	-0.033	4.01E-77	1.34E-11
cg17187521	12	125003379	*NCOR2*	0.464	0.373	0.426	-0.091	-0.038	4.74E-103	1.48E-10
cg01612883	12	125023115	*NCOR2*	0.369	0.314	0.350	-0.055	-0.019	9.55E-114	8.48E-10
cg00530564	12	125036429	*NCOR2*	0.323	0.254	0.302	-0.069	-0.021	1.09E-126	8.78E-08
cg09789874	12	125262045	NA	0.467	0.349	0.423	-0.118	-0.044	3.11E-123	4.62E-11
cg23925154	12	127414940	NA	0.706	0.755	0.761	0.050	0.055	8.51E-17	1.49E-09
cg19717347	12	129304956	*SLC15A4*	0.489	0.379	0.445	-0.110	-0.043	1.11E-138	3.36E-13
cg26509012	12	131306241	*STX2*	0.477	0.424	0.442	-0.053	-0.035	6.49E-45	4.60E-11
cg07356722	12	131353328	NA	0.691	0.760	0.749	0.069	0.057	1.72E-52	1.28E-17
cg00404861	12	131590393	*GPR133*	0.580	0.651	0.606	0.071	0.026	4.74E-95	1.13E-09
cg21726551	12	131590460	*GPR133*	0.630	0.700	0.661	0.070	0.030	5.21E-73	2.59E-09
cg07920381	12	132303685	NA	0.553	0.468	0.515	-0.085	-0.038	1.87E-88	3.40E-11
cg04468671	12	132337407	NA	0.481	0.416	0.453	-0.065	-0.027	1.39E-111	1.35E-12
cg07744430	12	132345502	NA	0.473	0.416	0.438	-0.057	-0.035	7.21E-90	1.50E-18
cg19257402	12	132356988	NA	0.362	0.297	0.339	-0.065	-0.024	1.35E-104	7.02E-09
cg21649013	12	132381683	*ULK1*	0.333	0.277	0.312	-0.056	-0.021	6.14E-102	9.66E-10
cg16355945	12	132654742	NA	0.331	0.259	0.293	-0.072	-0.038	4.23E-58	8.41E-08
cg19653417	12	132654924	NA	0.784	0.729	0.756	-0.054	-0.028	3.38E-41	1.07E-07
cg25939853	12	133343428	NA	0.452	0.359	0.407	-0.093	-0.045	4.65E-68	8.28E-09
cg03568673	13	20796216	*GJB6*	0.503	0.379	0.451	-0.125	-0.052	1.78E-176	3.55E-23
cg07879785	13	21095712	*CRYL1*	0.585	0.511	0.558	-0.074	-0.027	6.48E-92	1.47E-08
cg01124810	13	21144699	*IFT88*	0.571	0.516	0.552	-0.055	-0.019	1.71E-66	4.10E-06
cg05830425	13	21654356	NA	0.515	0.411	0.487	-0.104	-0.028	3.20E-133	3.01E-07
cg00792968	13	22635873	NA	0.756	0.812	0.778	0.056	0.023	1.38E-69	3.81E-06
cg24659858	13	24270321	NA	0.516	0.585	0.562	0.068	0.046	1.81E-38	1.31E-09
cg02659854	13	24824078	*SPATA13*	0.403	0.337	0.373	-0.066	-0.030	4.02E-79	4.15E-09
cg09088496	13	24825973	*SPATA13*	0.745	0.810	0.774	0.065	0.029	4.74E-100	3.51E-11
cg13237068	13	25485381	*CENPJ*	0.384	0.315	0.343	-0.069	-0.041	3.01E-68	1.10E-12

cg05868813	13	26594483	*ATP8A2*	0.383	0.325	0.356	-0.058	-0.027	8.50E-50	1.47E-06
cg09363424	13	27829014	*RPL21*	0.773	0.820	0.824	0.047	0.051	1.80E-20	1.75E-11
cg26885400	13	27842278	NA	0.377	0.315	0.343	-0.061	-0.034	5.56E-74	1.78E-12
cg14339466	13	27927490	NA	0.735	0.792	0.771	0.057	0.036	6.46E-41	1.27E-08
cg11588787	13	28181449	*LNX2*	0.312	0.257	0.285	-0.056	-0.027	1.42E-54	6.79E-07
cg20227511	13	28670602	*FLT3*	0.526	0.426	0.498	-0.100	-0.028	8.66E-109	1.18E-06
cg26472910	13	28673762	*FLT3*	0.271	0.217	0.245	-0.054	-0.027	4.27E-53	7.81E-07
cg17426969	13	28803434	*PAN3*	0.739	0.791	0.789	0.052	0.050	6.59E-28	1.62E-12
cg19118519	13	29192851	NA	0.656	0.583	0.619	-0.073	-0.037	1.15E-36	3.16E-06
cg16463452	13	29195249	NA	0.485	0.385	0.434	-0.101	-0.052	5.66E-114	6.11E-18
cg20956594	13	29202889	NA	0.502	0.409	0.474	-0.093	-0.029	2.72E-86	2.52E-06
cg02097152	13	29291701	*SLC46A3*	0.482	0.390	0.435	-0.092	-0.046	8.56E-120	5.68E-19
cg02788021	13	30882905	*KATNAL1*	0.405	0.310	0.360	-0.095	-0.045	5.77E-127	1.44E-16
cg25936902	13	30982971	NA	0.407	0.328	0.378	-0.079	-0.029	1.86E-119	3.31E-11
cg26814703	13	31019673	NA	0.316	0.248	0.283	-0.067	-0.033	7.93E-94	1.55E-15
cg01504447	13	31041221	*HMGB1*	0.249	0.187	0.207	-0.062	-0.042	1.52E-81	1.12E-17
cg00648883	13	32524761	*EEF1DP3*	0.326	0.248	0.287	-0.078	-0.039	5.43E-100	1.31E-13
cg21253130	13	32609104	*FRY*	0.493	0.427	0.469	-0.065	-0.023	3.06E-77	8.62E-07
cg12836863	13	32889023	*BRCA2*	0.529	0.418	0.468	-0.111	-0.061	1.58E-93	9.13E-17
cg24113534	13	33791982	*STARD13*	0.688	0.741	0.732	0.053	0.044	6.34E-25	1.62E-09
cg05757376	13	36516069	*DCLK1*	0.725	0.778	0.770	0.053	0.045	8.72E-27	6.77E-10
cg25179758	13	36918614	*SPG20*	0.359	0.298	0.331	-0.061	-0.027	5.18E-66	1.68E-08
cg02423185	13	37337525	NA	0.754	0.806	0.789	0.052	0.035	9.67E-35	4.38E-08
cg11162888	13	40762188	*LINC00332*	0.731	0.787	0.762	0.056	0.031	1.27E-76	2.50E-13
cg23211791	13	41364359	*SLC25A15*	0.325	0.267	0.292	-0.058	-0.033	7.60E-43	4.59E-07
cg02420027	13	41632640	NA	0.432	0.361	0.405	-0.071	-0.026	4.80E-118	8.63E-10
cg09909069	13	41860793	NA	0.752	0.809	0.786	0.057	0.033	1.11E-56	3.30E-10
cg25365958	13	42615991	*DGKH*	0.266	0.206	0.240	-0.060	-0.026	6.03E-93	3.03E-10
cg18860329	13	43354421	*FAM216B*	0.617	0.546	0.574	-0.071	-0.043	3.13E-36	4.99E-10
cg07409200	13	43354674	*FAM216B*	0.390	0.295	0.343	-0.095	-0.047	1.54E-82	1.56E-12
cg26529044	13	43354767	*FAM216B*	0.260	0.208	0.223	-0.052	-0.037	3.59E-49	3.47E-13
cg10274453	13	43378116	NA	0.599	0.526	0.566	-0.073	-0.034	1.09E-39	7.12E-07
cg03763873	13	43565901	*EPSTI1*	0.201	0.122	0.143	-0.079	-0.057	3.24E-65	4.93E-15
cg04365696	13	43834391	*ENOX1*	0.710	0.762	0.753	0.052	0.043	3.89E-20	4.76E-07
cg02969426	13	45775005	*GTF2F2*	0.653	0.565	0.628	-0.088	-0.025	1.95E-102	3.44E-06
cg11218434	13	45911764	*TPT1*	0.775	0.825	0.792	0.051	0.017	1.80E-105	8.36E-08
cg06172950	13	46075084	*COG3*	0.305	0.239	0.270	-0.067	-0.035	8.50E-83	9.93E-13
cg07880943	13	46744500	*LCP1*	0.612	0.670	0.635	0.058	0.022	5.59E-94	1.11E-08
cg04118119	13	47371987	*ESD*	0.498	0.388	0.455	-0.110	-0.042	9.95E-135	2.13E-12
cg05699739	13	47373579	NA	0.309	0.245	0.285	-0.065	-0.025	2.64E-81	6.20E-08
cg03126694	13	49001104	*RB1*	0.405	0.323	0.378	-0.082	-0.026	4.79E-104	8.60E-07
cg20405809	13	49117085	NA	0.350	0.289	0.324	-0.061	-0.026	3.09E-98	1.14E-10
cg25989526	13	49147573	NA	0.385	0.302	0.346	-0.082	-0.039	4.82E-84	2.15E-11
cg03278564	13	49200201	NA	0.485	0.372	0.445	-0.113	-0.041	1.83E-153	5.76E-16
cg02043329	13	49740771	*FNDC3A*	0.676	0.621	0.653	-0.055	-0.024	4.04E-54	9.09E-07
cg15207619	13	49975643	*CAB39L*	0.240	0.180	0.205	-0.060	-0.034	2.69E-47	4.07E-08
cg02210115	13	49990767	*CAB39L*	0.643	0.580	0.614	-0.064	-0.029	1.86E-47	5.98E-06
cg23036613	13	50068486	*SETDB2*	0.733	0.769	0.784	0.036	0.051	6.69E-13	3.43E-11
cg17056618	13	50216117	NA	0.676	0.616	0.649	-0.060	-0.027	1.24E-61	2.03E-08
cg24553417	13	50244794	*EBPL*	0.491	0.378	0.449	-0.113	-0.042	2.27E-146	3.03E-16
cg18546006	13	50569769	*DLEU2*	0.272	0.202	0.235	-0.070	-0.037	2.05E-89	2.23E-14
cg09145126	13	50570018	*DLEU2*	0.497	0.375	0.447	-0.121	-0.050	9.69E-158	7.58E-21
cg13212435	13	50736277	NA	0.623	0.572	0.586	-0.050	-0.037	7.53E-24	4.43E-07
cg01449715	13	51491406	*RNASEH2B*	0.289	0.217	0.259	-0.072	-0.031	3.62E-92	2.47E-09
cg24013213	13	51640142	*GUCY1B2*	0.298	0.242	0.268	-0.056	-0.030	3.26E-67	8.16E-10
cg16692277	13	51640948	NA	0.259	0.208	0.233	-0.051	-0.026	3.89E-62	9.79E-09
cg11366363	13	51641069	NA	0.499	0.439	0.465	-0.059	-0.034	4.51E-67	9.82E-12
cg26776551	13	51944507	*INTS6*	0.386	0.293	0.348	-0.093	-0.039	2.66E-148	1.23E-17
cg16163847	13	52165356	*WDFY2*	0.474	0.362	0.421	-0.112	-0.053	3.60E-166	1.58E-25
cg05902527	13	56236197	NA	0.677	0.725	0.733	0.048	0.056	6.85E-18	2.63E-11
cg03274669	13	74805233	NA	0.752	0.803	0.776	0.051	0.023	3.03E-63	1.94E-07
cg23318020	13	74805375	NA	0.662	0.732	0.718	0.070	0.056	6.92E-53	2.46E-17
cg18577167	13	74805590	NA	0.698	0.751	0.725	0.053	0.027	1.01E-73	2.40E-10
cg25808094	13	75892615	*TBC1D4*	0.735	0.786	0.772	0.051	0.037	4.47E-36	1.31E-09
cg09009788	13	77894098	*MYCBP2*	0.700	0.647	0.677	-0.053	-0.023	2.11E-64	1.25E-08
cg05969360	13	80443435	NA	0.755	0.807	0.789	0.053	0.034	3.65E-59	2.67E-12
cg23665802	13	92002338	*MIR17HG*	0.368	0.306	0.320	-0.061	-0.048	1.12E-55	6.88E-18
cg02297838	13	92002454	*MIR17HG*	0.297	0.230	0.245	-0.067	-0.052	2.66E-76	4.86E-23
cg06340704	13	97877417	*MBNL2*	0.705	0.767	0.736	0.061	0.031	2.44E-49	4.56E-08
cg07804470	13	99201496	*STK24*	0.414	0.309	0.370	-0.105	-0.044	6.70E-124	4.99E-13
cg25032321	13	99227533	*STK24*	0.674	0.736	0.704	0.062	0.031	1.37E-83	7.71E-12
cg10057295	13	99230004	*STK24*	0.313	0.250	0.272	-0.063	-0.040	3.98E-80	1.77E-16
cg05257202	13	99934873	*UBAC2*	0.321	0.390	0.344	0.069	0.023	3.07E-71	1.02E-06
cg09855140	13	100004097	*UBAC2*	0.753	0.809	0.778	0.056	0.025	1.92E-86	3.81E-10
cg19200953	13	100470738	*CLYBL*	0.515	0.580	0.556	0.065	0.040	1.21E-31	3.29E-06
cg19024980	13	100781335	*PCCA*	0.685	0.738	0.727	0.053	0.042	2.01E-29	5.30E-09
cg25084760	13	100886412	*PCCA*	0.486	0.383	0.455	-0.103	-0.030	6.66E-115	5.69E-08
cg25814649	13	107143980	*EFNB2*	0.368	0.297	0.331	-0.071	-0.037	1.38E-70	1.43E-10

cg18158859	13	107144026	*EFNB2*	0.298	0.230	0.268	-0.068	-0.030	1.10E-56	3.81E-06
cg09646392	13	108921052	*TNFSF13B*	0.448	0.364	0.419	-0.084	-0.029	9.21E-79	2.08E-06
cg14677983	13	111318022	*CARS2*	0.447	0.375	0.411	-0.073	-0.037	9.68E-127	5.83E-20
cg08400494	13	111318490	*CARS2*	0.377	0.296	0.347	-0.082	-0.030	2.93E-86	1.19E-08
cg05294112	13	112617069	NA	0.358	0.279	0.318	-0.079	-0.039	3.47E-64	6.99E-08
cg06847429	13	112881733	NA	0.703	0.754	0.746	0.051	0.042	4.04E-18	9.19E-07
cg02269496	13	113105282	NA	0.634	0.691	0.687	0.057	0.053	1.26E-23	8.43E-10
cg18571045	13	113238006	*TUBGCP3*	0.383	0.282	0.341	-0.100	-0.041	1.61E-136	3.47E-15
cg02417360	13	113398673	*ATP11A*	0.601	0.655	0.665	0.054	0.065	9.13E-14	1.65E-08
cg26995035	13	113785830	*F10*	0.736	0.780	0.787	0.044	0.052	2.33E-18	3.17E-11
cg18535410	13	113793268	*F10*	0.417	0.323	0.380	-0.094	-0.036	3.62E-124	1.27E-11
cg14507533	13	114066074	NA	0.625	0.548	0.594	-0.077	-0.031	1.71E-75	4.19E-09
cg20628942	13	114171501	*TMCO3*	0.488	0.411	0.460	-0.077	-0.028	1.06E-88	2.91E-07
cg19023589	13	114182159	*TMCO3*	0.593	0.470	0.551	-0.123	-0.042	2.75E-123	6.47E-11
cg22535089	13	114184462	*TMCO3*	0.444	0.351	0.419	-0.094	-0.026	2.57E-122	5.18E-07
cg08183317	13	114261934	*TFDP1*	0.331	0.254	0.307	-0.077	-0.024	4.44E-114	6.01E-08
cg11703212	13	114271629	*TFDP1*	0.553	0.430	0.510	-0.123	-0.043	5.34E-135	1.28E-12
cg06380691	13	114828264	*RASA3*	0.331	0.257	0.304	-0.074	-0.027	9.94E-122	1.11E-10
cg16962442	13	114828455	*RASA3*	0.445	0.370	0.411	-0.075	-0.034	3.03E-89	6.80E-12
cg10800346	13	114829496	*RASA3*	0.434	0.327	0.395	-0.107	-0.040	2.24E-107	2.19E-09
cg05296619	13	114834683	*RASA3*	0.471	0.377	0.438	-0.094	-0.033	7.91E-156	5.92E-15
cg04388548	13	114905640	NA	0.590	0.655	0.614	0.065	0.024	3.80E-85	7.51E-09
cg12701674	13	114908876	NA	0.533	0.592	0.565	0.060	0.032	3.76E-68	5.06E-12
cg01925950	13	115022228	*CDC16*	0.390	0.300	0.349	-0.090	-0.041	1.62E-134	5.66E-17
cg16701266	14	21024898	*RNASE9*	0.395	0.308	0.358	-0.087	-0.038	6.81E-84	1.13E-09
cg01620379	14	21052361	*RNASE11*	0.467	0.364	0.423	-0.104	-0.044	7.03E-136	1.55E-14
cg07300408	14	21058360	*RNASE12*	0.436	0.341	0.397	-0.095	-0.039	1.60E-63	8.32E-07
cg21882356	14	21153451	*ANG*	0.497	0.400	0.453	-0.097	-0.044	1.59E-182	6.27E-28
cg09842118	14	21359737	*RNASE3*	0.304	0.247	0.278	-0.057	-0.027	2.37E-53	1.83E-06
cg07525077	14	21359943	*RNASE3*	0.387	0.297	0.349	-0.089	-0.037	2.87E-92	3.35E-10
cg23327851	14	21423684	*RNASE2*	0.385	0.310	0.357	-0.075	-0.028	3.29E-160	5.70E-15
cg18915856	14	21483641	NA	0.435	0.333	0.387	-0.102	-0.048	1.66E-170	5.86E-24
cg00502926	14	21510056	*RNASE7*	0.482	0.412	0.445	-0.069	-0.036	8.23E-58	8.67E-09
cg18575809	14	21540329	*ARHGEF40*	0.318	0.255	0.279	-0.063	-0.039	8.83E-57	1.75E-10
cg07555731	14	21624639	*OR5AU1*	0.645	0.708	0.692	0.063	0.047	1.21E-42	1.21E-11
cg12774429	14	21853470	*SUPT16H*	0.387	0.298	0.342	-0.088	-0.045	1.67E-136	9.82E-22
cg01839452	14	22433489	NA	0.739	0.797	0.790	0.058	0.051	1.23E-34	1.99E-13
cg02903983	14	22968031	NA	0.651	0.725	0.706	0.074	0.055	1.86E-50	2.14E-13
cg01797899	14	22978195	NA	0.754	0.811	0.783	0.056	0.029	1.19E-85	3.35E-12
cg01867764	14	22986466	NA	0.674	0.729	0.694	0.056	0.020	3.66E-74	1.74E-06
cg01666652	14	22987689	NA	0.727	0.781	0.755	0.054	0.027	5.57E-64	1.51E-10
cg26053876	14	22991735	NA	0.634	0.699	0.690	0.065	0.056	2.88E-28	7.13E-10
cg26400750	14	22992073	NA	0.715	0.766	0.749	0.051	0.034	1.25E-35	8.52E-08
cg03035162	14	22993190	NA	0.743	0.797	0.763	0.055	0.021	2.27E-83	8.95E-08
cg20042612	14	23027728	NA	0.776	0.838	0.806	0.062	0.030	1.87E-71	1.49E-09
cg02999224	14	23284559	*SLC7A7*	0.439	0.344	0.405	-0.096	-0.034	1.43E-128	5.52E-11
cg01508380	14	23305585	*MMP14*	0.427	0.365	0.399	-0.062	-0.028	1.73E-46	5.32E-06
cg26118358	14	23321005	NA	0.417	0.335	0.388	-0.081	-0.028	2.39E-117	1.01E-09
cg04756491	14	23385384	*RBM23*	0.463	0.350	0.433	-0.112	-0.030	1.26E-113	1.88E-06
cg16502747	14	23531625	*ACIN1*	0.432	0.356	0.405	-0.077	-0.027	2.62E-123	1.57E-10
cg25099490	14	23583133	NA	0.379	0.292	0.345	-0.087	-0.034	5.41E-154	7.43E-16
cg15377871	14	23588325	*CEBPE*	0.594	0.502	0.558	-0.092	-0.036	7.07E-121	8.66E-13
cg00554993	14	23588616	*CEBPE*	0.338	0.251	0.303	-0.087	-0.035	3.23E-122	1.26E-12
cg15527515	14	23630709	*SLC7A8*	0.406	0.303	0.367	-0.103	-0.039	2.98E-131	9.42E-12
cg27035169	14	23653098	*SLC7A8*	0.387	0.337	0.354	-0.050	-0.033	1.88E-30	4.66E-07
cg18449389	14	23776482	*BCL2L2*	0.276	0.217	0.250	-0.060	-0.026	4.89E-85	5.44E-10
cg11792874	14	24014501	*THTPA*	0.386	0.326	0.368	-0.061	-0.018	8.12E-113	3.85E-08
cg10264529	14	24562064	*PCK2*	0.527	0.435	0.487	-0.092	-0.040	1.13E-169	1.89E-21
cg09378756	14	24569543	*PCK2*	0.340	0.258	0.304	-0.082	-0.036	9.19E-123	1.32E-13
cg16473141	14	24641501	*REC8*	0.290	0.231	0.264	-0.059	-0.026	2.13E-58	7.92E-07
cg16045731	14	24777708	*CIDEB*	0.566	0.507	0.546	-0.059	-0.020	1.43E-76	1.63E-06
cg00951869	14	24805349	*RIPK3*	0.336	0.263	0.296	-0.074	-0.041	2.88E-156	2.69E-30
cg11152302	14	24867472	*NYNRIN*	0.573	0.507	0.534	-0.065	-0.039	4.27E-70	2.87E-14
cg12890903	14	24867954	*NYNRIN*	0.321	0.256	0.290	-0.065	-0.031	2.43E-107	5.41E-14
cg01697902	14	25046117	*CTSG*	0.450	0.341	0.408	-0.109	-0.041	2.39E-152	1.04E-14
cg24777950	14	25046121	*CTSG*	0.412	0.313	0.382	-0.099	-0.030	2.06E-119	3.51E-08
cg10348234	14	31698967	NA	0.273	0.221	0.248	-0.053	-0.026	2.07E-56	7.46E-07
cg18437792	14	31699236	NA	0.401	0.310	0.369	-0.091	-0.032	5.77E-112	1.90E-08
cg07185587	14	32981885	*AKAP6*	0.734	0.787	0.764	0.053	0.030	2.49E-42	8.24E-08
cg18410685	14	34838703	NA	0.326	0.262	0.289	-0.064	-0.038	4.86E-71	8.05E-12
cg02219601	14	35835511	NA	0.298	0.244	0.263	-0.054	-0.035	2.94E-75	8.51E-16
cg14179944	14	35867515	NA	0.443	0.381	0.412	-0.062	-0.031	1.33E-63	8.61E-09
cg07917901	14	35870184	NA	0.446	0.330	0.400	-0.117	-0.046	5.86E-168	1.36E-18
cg24115596	14	38051289	NA	0.767	0.817	0.810	0.050	0.043	4.62E-25	6.53E-09
cg24743156	14	39734964	*CTAGE5*	0.433	0.347	0.404	-0.086	-0.029	1.49E-92	4.46E-07
cg13277939	14	39735211	*CTAGE5*	0.441	0.334	0.397	-0.107	-0.044	5.82E-138	7.83E-17
cg18976418	14	39735496	*CTAGE5*	0.310	0.256	0.285	-0.054	-0.025	1.19E-51	2.50E-06
cg16362173	14	50374131	NA	0.714	0.766	0.751	0.051	0.037	5.33E-28	2.50E-07

cg22991506	14	50468241	*C14orf182*	0.589	0.665	0.618	0.075	0.028	7.01E-131	3.04E-14
cg04352495	14	50700827	NA	0.750	0.810	0.794	0.060	0.045	3.77E-39	3.56E-11
cg19990483	14	51030485	*ATL1*	0.758	0.805	0.810	0.047	0.052	4.01E-17	2.44E-10
cg08572565	14	51298737	*NIN*	0.294	0.235	0.262	-0.059	-0.032	5.78E-65	5.92E-10
cg00879541	14	52466952	*C14orf166*	0.777	0.827	0.815	0.050	0.039	9.06E-29	5.89E-08
cg23648239	14	52701606	NA	0.467	0.361	0.430	-0.107	-0.037	7.78E-137	1.32E-10
cg13654573	14	53576363	*DDHD1*	0.631	0.684	0.660	0.053	0.029	2.94E-34	6.45E-06
cg14380217	14	54604922	NA	0.714	0.770	0.758	0.056	0.044	4.63E-39	2.92E-12
cg26032021	14	54804413	NA	0.723	0.772	0.782	0.049	0.059	5.40E-21	6.18E-14
cg02068981	14	54907137	*CNIH*	0.463	0.411	0.434	-0.052	-0.029	3.93E-33	2.40E-06
cg26437697	14	55143755	*SAMD4A*	0.566	0.479	0.539	-0.088	-0.028	2.77E-101	1.06E-06
cg14187687	14	55249770	*SAMD4A*	0.517	0.403	0.471	-0.114	-0.045	1.76E-114	2.59E-12
cg14053764	14	55761432	*FBXO34*	0.740	0.796	0.770	0.056	0.030	3.21E-93	1.35E-14
cg25576997	14	56257750	*LINC00520*	0.456	0.341	0.405	-0.114	-0.051	4.11E-116	1.57E-14
cg14138235	14	56281968	NA	0.449	0.356	0.400	-0.093	-0.049	2.05E-129	3.55E-19
cg16770832	14	56604516	*PELI2*	0.662	0.608	0.639	-0.054	-0.023	1.31E-67	1.32E-09
cg21037057	14	57464939	NA	0.717	0.773	0.765	0.056	0.048	3.96E-22	8.77E-08
cg24026619	14	59027109	NA	0.283	0.223	0.252	-0.059	-0.031	1.32E-54	1.46E-08
cg02078710	14	59066094	NA	0.661	0.730	0.690	0.068	0.028	6.37E-119	2.47E-15
cg23218957	14	59109856	*DACT1*	0.735	0.785	0.764	0.050	0.029	1.54E-43	3.62E-07
cg02265239	14	59748448	*DAAM1*	0.730	0.783	0.772	0.053	0.042	1.25E-26	2.21E-08
cg01074676	14	60601069	*PCNXL4*	0.575	0.629	0.627	0.054	0.052	4.61E-17	1.07E-07
cg17306848	14	61793064	*PRKCH*	0.745	0.809	0.783	0.064	0.038	2.33E-63	6.19E-12
cg09254210	14	61873622	*PRKCH*	0.645	0.713	0.703	0.068	0.058	1.67E-29	1.96E-10
cg03523434	14	63568606	*KCNH5*	0.696	0.735	0.746	0.040	0.051	9.11E-18	2.95E-12
cg11833768	14	63940209	*PPP2R5E*	0.455	0.366	0.420	-0.089	-0.036	5.78E-85	2.12E-08
cg16041798	14	64905375	*MTHFD1*	0.711	0.763	0.771	0.052	0.061	6.89E-27	5.56E-16
cg14412134	14	64912417	*MTHFD1*	0.722	0.789	0.762	0.068	0.040	3.40E-64	1.67E-11
cg01860774	14	64969374	*ZBTB25*	0.753	0.810	0.778	0.056	0.025	6.44E-89	2.17E-10
cg15889847	14	65183532	*PLEKHG3*	0.439	0.356	0.410	-0.083	-0.029	3.97E-155	6.02E-15
cg25603108	14	68714746	*RAD51B*	0.301	0.247	0.268	-0.054	-0.033	3.42E-52	7.85E-10
cg26298914	14	68798365	*RAD51B*	0.502	0.375	0.466	-0.127	-0.036	5.57E-164	6.69E-10
cg23526087	14	68973466	*RAD51B*	0.622	0.695	0.649	0.073	0.027	1.57E-142	8.83E-14
cg19662895	14	69074455	NA	0.419	0.362	0.391	-0.057	-0.028	2.77E-50	2.20E-07
cg13067714	14	69091453	NA	0.558	0.490	0.524	-0.068	-0.034	1.36E-96	6.14E-13
cg04346672	14	69229910	NA	0.436	0.374	0.408	-0.062	-0.028	4.57E-87	2.50E-10
cg06617636	14	69256690	*ZFP36L1*	0.770	0.830	0.795	0.061	0.026	8.98E-111	2.76E-11
cg08169020	14	69256888	*ZFP36L1*	0.572	0.681	0.621	0.109	0.049	3.00E-137	5.39E-18
cg10099732	14	69256977	*ZFP36L1*	0.594	0.680	0.625	0.085	0.030	2.70E-103	5.21E-09
cg10318744	14	69282256	NA	0.624	0.705	0.663	0.081	0.039	8.39E-72	1.42E-08
cg04958055	14	69404437	*ACTN1*	0.325	0.251	0.299	-0.073	-0.026	2.95E-154	9.51E-14
cg02057747	14	69408849	*ACTN1*	0.357	0.290	0.332	-0.067	-0.025	7.97E-168	8.19E-18
cg23251057	14	69480702	NA	0.326	0.260	0.286	-0.066	-0.040	8.32E-99	1.32E-20
cg19725377	14	69535979	*DCAF5*	0.377	0.289	0.340	-0.088	-0.036	1.23E-114	3.59E-12
cg02175033	14	70098523	*KIAA0247*	0.418	0.317	0.391	-0.101	-0.027	4.49E-136	3.72E-07
cg16565031	14	70186289	NA	0.419	0.320	0.378	-0.099	-0.041	1.35E-151	1.56E-17
cg01448863	14	70264043	*SLC10A1*	0.405	0.302	0.363	-0.102	-0.042	1.94E-112	4.47E-11
cg21088438	14	70264869	*SLC10A1*	0.427	0.327	0.386	-0.100	-0.040	4.38E-105	1.85E-10
cg22819952	14	71472713	*PCNX*	0.527	0.572	0.581	0.045	0.054	1.18E-15	5.24E-10
cg05859308	14	71712645	NA	0.693	0.763	0.718	0.070	0.025	1.85E-94	7.23E-08
cg21507487	14	71941057	NA	0.747	0.792	0.801	0.045	0.054	8.47E-21	2.93E-13
cg05111779	14	72990359	*RGS6*	0.421	0.329	0.386	-0.092	-0.035	1.02E-90	8.96E-09
cg22924250	14	73358241	*DPF3*	0.314	0.246	0.275	-0.068	-0.039	1.80E-60	1.25E-09
cg26217402	14	74238381	*C14orf43*	0.550	0.639	0.583	0.088	0.033	5.92E-136	1.01E-13
cg16713889	14	74409552	*FAM161B*	0.511	0.401	0.469	-0.109	-0.042	1.17E-104	3.14E-11
cg16786178	14	75764329	NA	0.386	0.291	0.340	-0.095	-0.046	2.39E-158	1.14E-22
cg00980622	14	75884845	NA	0.538	0.413	0.504	-0.126	-0.034	1.57E-150	1.87E-09
cg11083848	14	76371681	*TTLL5*	0.457	0.352	0.415	-0.105	-0.042	1.59E-122	8.21E-14
cg20981219	14	77385200	NA	0.724	0.672	0.688	-0.052	-0.036	6.49E-30	2.56E-08
cg24171453	14	78079686	*SPTLC2*	0.277	0.211	0.240	-0.066	-0.037	2.14E-96	7.99E-18
cg14373410	14	81426234	*TSHR*	0.750	0.809	0.781	0.059	0.030	3.15E-56	2.05E-08
cg11071202	14	88462554	NA	0.340	0.268	0.316	-0.073	-0.025	6.36E-92	7.99E-07
cg25015038	14	89604062	NA	0.385	0.330	0.359	-0.055	-0.025	2.41E-51	1.31E-06
cg16384774	14	89626932	*FOXN3*	0.668	0.605	0.642	-0.063	-0.026	1.12E-62	5.78E-07
cg14023350	14	89631066	*FOXN3*	0.396	0.326	0.363	-0.071	-0.034	3.42E-111	5.03E-16
cg18569623	14	89631502	*FOXN3*	0.356	0.277	0.318	-0.079	-0.038	2.30E-119	4.77E-18
cg07102397	14	89864727	*FOXN3*	0.594	0.478	0.564	-0.115	-0.030	2.37E-158	1.85E-10
cg06719445	14	89925468	*FOXN3*	0.432	0.328	0.386	-0.104	-0.046	9.57E-125	1.50E-15
cg13152690	14	91695017	NA	0.642	0.732	0.675	0.090	0.033	4.38E-176	1.59E-17
cg14214797	14	91860613	*CCDC88C*	0.696	0.763	0.723	0.067	0.027	4.92E-155	8.10E-18
cg26290716	14	91862813	*CCDC88C*	0.411	0.337	0.386	-0.074	-0.026	1.67E-106	2.00E-09
cg04794141	14	92325159	*TC2N*	0.322	0.265	0.293	-0.057	-0.029	1.26E-53	7.80E-08
cg08796342	14	92334029	*TC2N*	0.628	0.708	0.653	0.080	0.026	7.47E-136	4.11E-09
cg19453686	14	92334271	*TC2N*	0.758	0.820	0.782	0.063	0.024	1.68E-82	2.23E-08
cg01288337	14	92991035	*RIN3*	0.344	0.277	0.322	-0.067	-0.022	2.18E-89	1.02E-06
cg25066772	14	93136682	*RIN3*	0.286	0.228	0.248	-0.058	-0.038	4.85E-64	1.34E-13
cg17319795	14	93170808	*LGMN*	0.310	0.243	0.268	-0.067	-0.042	1.43E-68	2.34E-13
cg19011001	14	93539613	*ITPK1*	0.381	0.305	0.356	-0.075	-0.024	6.89E-132	3.03E-11

cg01655008	14	93652954	*TMEM251*	0.597	0.502	0.559	-0.096	-0.038	5.63E-105	2.15E-11
cg07481273	14	93653027	*TMEM251*	0.515	0.399	0.466	-0.116	-0.050	1.59E-141	7.60E-17
cg23893332	14	94576048	*IFI27*	0.451	0.373	0.420	-0.078	-0.030	1.27E-143	1.13E-14
cg10070185	14	94857151	*SERPINA1*	0.449	0.397	0.427	-0.053	-0.023	2.29E-93	2.25E-11
cg12170933	14	95620298	*DICER1*	0.746	0.803	0.795	0.057	0.049	1.80E-35	1.91E-12
cg06634140	14	95956325	NA	0.524	0.428	0.485	-0.096	-0.038	1.74E-115	1.28E-11
cg16405432	14	95973710	NA	0.405	0.327	0.371	-0.077	-0.034	4.78E-138	4.78E-18
cg23661721	14	95991371	NA	0.445	0.354	0.413	-0.090	-0.032	7.14E-94	3.29E-07
cg18091385	14	99502466	NA	0.454	0.389	0.429	-0.065	-0.025	3.62E-66	3.86E-07
cg16452866	14	99655676	*BCL11B*	0.753	0.815	0.781	0.062	0.028	4.30E-71	1.46E-07
cg02963266	14	99681710	*BCL11B*	0.679	0.738	0.705	0.059	0.026	3.97E-61	4.67E-07
cg19947357	14	99864952	*SETD3*	0.603	0.658	0.653	0.054	0.050	3.43E-22	8.88E-09
cg17813891	14	100532036	*EVL*	0.703	0.765	0.729	0.062	0.026	1.63E-73	4.35E-08
cg14245199	14	100540291	*EVL*	0.725	0.786	0.752	0.062	0.028	2.01E-107	4.03E-12
cg04166500	14	100571607	*EVL*	0.670	0.735	0.698	0.065	0.028	1.23E-115	2.04E-14
cg13227162	14	101351197	*RTL1*	0.681	0.736	0.726	0.055	0.046	2.12E-23	4.67E-08
cg19009471	14	101908998	NA	0.411	0.468	0.433	0.057	0.022	6.88E-51	5.58E-06
cg17904575	14	102290370	*PPP2R5C*	0.655	0.723	0.694	0.068	0.039	1.78E-63	1.43E-10
cg08253808	14	102676957	*WDR20*	0.509	0.400	0.478	-0.109	-0.031	3.15E-101	5.22E-06
cg05398700	14	102677141	*WDR20*	0.545	0.439	0.504	-0.106	-0.041	6.27E-91	2.85E-10
cg14315912	14	102677294	*WDR20*	0.557	0.484	0.539	-0.073	-0.019	5.66E-129	1.18E-07
cg16364675	14	103096221	*RCOR1*	0.350	0.282	0.323	-0.067	-0.027	1.19E-66	9.28E-07
cg14543285	14	103129037	*RCOR1*	0.519	0.427	0.490	-0.092	-0.029	1.74E-144	1.05E-11
cg21057323	14	103412980	*CDC42BPB*	0.566	0.653	0.594	0.086	0.028	2.10E-83	6.48E-06
cg04987734	14	103415873	*CDC42BPB*	0.375	0.441	0.407	0.066	0.032	3.05E-46	3.63E-08
cg02003183	14	103415882	*CDC42BPB*	0.195	0.253	0.227	0.058	0.032	6.57E-35	1.62E-08
cg04189326	14	103481271	*CDC42BPB*	0.403	0.347	0.380	-0.056	-0.023	2.15E-78	4.85E-08
cg04258520	14	103868351	*MARK3*	0.525	0.584	0.560	0.059	0.035	5.53E-35	2.54E-07
cg23090046	14	104094619	*KLC1*	0.525	0.471	0.508	-0.054	-0.017	7.08E-89	8.18E-07
cg23051349	14	104094779	*KLC1*	0.265	0.192	0.241	-0.072	-0.024	1.30E-89	2.78E-06
cg27170268	14	104171695	*XRCC3*	0.416	0.335	0.379	-0.081	-0.037	3.40E-69	1.79E-08
cg26242531	14	104190678	*ZFYVE21*	0.428	0.378	0.393	-0.051	-0.035	1.39E-40	4.98E-12
cg13127694	14	104656055	NA	0.720	0.766	0.773	0.046	0.053	8.40E-19	4.85E-11
cg23592421	14	105147461	NA	0.437	0.360	0.384	-0.078	-0.053	1.20E-64	1.10E-15
cg26333564	14	105147781	NA	0.374	0.314	0.342	-0.059	-0.032	7.36E-101	1.06E-18
cg05873568	14	105155145	*INF2*	0.271	0.214	0.249	-0.056	-0.022	1.02E-86	2.38E-08
cg18875357	14	105391263	*PLD4*	0.271	0.220	0.256	-0.051	-0.015	6.93E-89	6.07E-06
cg19676553	14	105751629	*BRF1*	0.472	0.386	0.440	-0.085	-0.032	1.56E-144	5.72E-15
cg16162970	14	105779952	*PACS2*	0.351	0.273	0.326	-0.078	-0.026	8.83E-99	9.00E-07
cg18568335	14	105792505	*PACS2*	0.484	0.376	0.431	-0.108	-0.052	5.38E-109	6.49E-14
cg07163845	14	106071651	NA	0.447	0.391	0.417	-0.056	-0.030	7.10E-43	1.37E-07
cg06121514	15	25362188	*IPW*	0.648	0.693	0.700	0.045	0.052	1.13E-16	1.91E-10
cg11826104	15	25650611	*UBE3A*	0.613	0.526	0.586	-0.087	-0.027	2.35E-84	4.39E-06
cg20049422	15	26044289	*ATP10A*	0.410	0.327	0.380	-0.083	-0.030	7.49E-87	5.41E-07
cg18083248	15	26108946	*ATP10A*	0.359	0.301	0.333	-0.057	-0.026	1.99E-79	1.75E-09
cg18385440	15	29212889	*APBA2*	0.353	0.302	0.329	-0.051	-0.024	7.60E-83	1.20E-12
cg20900050	15	31689509	NA	0.461	0.383	0.431	-0.077	-0.030	5.19E-94	3.61E-08
cg04870120	15	32999748	NA	0.477	0.393	0.442	-0.084	-0.036	8.15E-51	1.94E-06
cg05327068	15	34634045	*NOP10*	0.512	0.425	0.474	-0.087	-0.037	1.33E-81	1.54E-09
cg05995576	15	35049543	NA	0.686	0.739	0.723	0.053	0.037	7.84E-33	3.94E-08
cg02888513	15	39205040	NA	0.742	0.792	0.792	0.051	0.050	1.18E-19	5.40E-10
cg06232205	15	40120808	*GPR176*	0.753	0.805	0.782	0.052	0.030	5.49E-51	1.48E-08
cg12929983	15	40399004	*BMF*	0.391	0.294	0.357	-0.096	-0.034	1.07E-129	7.73E-11
cg11693709	15	40542019	*PAK6*	0.499	0.424	0.435	-0.075	-0.064	3.03E-44	2.66E-15
cg02240622	15	40601467	*PLCB2*	0.427	0.337	0.391	-0.089	-0.036	8.13E-146	1.67E-16
cg20317748	15	40633124	*C15orf52*	0.439	0.348	0.401	-0.092	-0.038	1.86E-88	1.36E-09
cg01987202	15	40633194	*C15orf52*	0.316	0.258	0.289	-0.058	-0.028	2.48E-103	9.70E-15
cg05059480	15	40633202	*C15orf52*	0.438	0.369	0.415	-0.069	-0.023	8.33E-147	1.21E-14
cg07047589	15	40642998	*PHGR1*	0.287	0.234	0.261	-0.052	-0.025	4.49E-47	4.06E-07
cg27057329	15	40803244	NA	0.353	0.269	0.316	-0.085	-0.037	3.27E-125	5.22E-13
cg22079077	15	41061384	*DNAJC17*	0.376	0.316	0.355	-0.060	-0.021	6.89E-101	9.31E-09
cg16764778	15	41061410	*DNAJC17*	0.339	0.278	0.318	-0.061	-0.021	5.99E-92	2.41E-07
cg23125506	15	41198534	NA	0.364	0.301	0.338	-0.063	-0.027	2.64E-68	3.61E-07
cg21953058	15	41309253	*INO80*	0.419	0.343	0.387	-0.076	-0.032	5.28E-65	2.77E-06
cg08180028	15	41796044	*LTK*	0.456	0.382	0.432	-0.074	-0.024	3.19E-160	5.26E-13
cg22894805	15	41983773	*MGA*	0.437	0.349	0.404	-0.087	-0.033	3.01E-114	1.90E-10
cg10142436	15	42209178	*EHD4*	0.297	0.233	0.273	-0.064	-0.024	4.11E-82	1.21E-06
cg21574244	15	42568411	*GANC*	0.448	0.347	0.413	-0.101	-0.035	1.55E-118	3.84E-10
cg14833933	15	42652294	*CAPN3*	0.405	0.342	0.378	-0.063	-0.027	1.36E-57	1.63E-06
cg15902038	15	42693448	*CAPN3*	0.306	0.245	0.279	-0.061	-0.027	4.66E-79	9.50E-09
cg05573378	15	42876795	*STARD9*	0.516	0.457	0.487	-0.058	-0.028	2.15E-88	4.46E-12
cg25875049	15	43531947	*TGM5*	0.385	0.307	0.338	-0.078	-0.047	1.23E-101	1.36E-21
cg15159104	15	43809865	*RNU6-28*	0.326	0.267	0.299	-0.058	-0.027	4.66E-96	1.43E-12
cg14184693	15	44085947	*SERF2*	0.432	0.373	0.402	-0.060	-0.030	3.15E-108	2.09E-15
cg00074771	15	44120341	*WDR76*	0.673	0.729	0.715	0.056	0.042	3.51E-30	8.74E-08
cg25930962	15	44321561	*FRMD5*	0.695	0.755	0.739	0.060	0.044	1.03E-38	9.68E-11
cg22107533	15	45028083	*TRIM69*	0.496	0.459	0.436	-0.037	-0.060	2.84E-17	4.10E-24
cg05439368	15	45028098	*TRIM69*	0.608	0.568	0.538	-0.041	-0.070	1.10E-18	2.42E-28

cg15022400	15	45028161	*TRIM69*	0.214	0.163	0.176	-0.051	-0.037	4.31E-51	1.40E-15
cg10439456	15	45028270	*TRIM69*	0.435	0.405	0.384	-0.029	-0.050	5.18E-08	1.99E-11
cg21244086	15	45479462	*SHF*	0.405	0.338	0.380	-0.067	-0.026	3.78E-111	9.68E-13
cg01626885	15	45937757	*SQRDL*	0.332	0.269	0.301	-0.063	-0.031	3.00E-100	6.45E-15
cg19405329	15	49440632	*COPS2*	0.497	0.391	0.457	-0.106	-0.040	5.00E-126	8.93E-12
cg16762030	15	50148234	NA	0.438	0.330	0.393	-0.108	-0.045	2.24E-135	9.99E-16
cg23671196	15	50405520	*ATP8B4*	0.339	0.259	0.301	-0.079	-0.038	1.37E-138	2.88E-21
cg27123392	15	50570473	*GABPB1*	0.531	0.419	0.486	-0.112	-0.045	3.21E-150	3.54E-19
cg00083959	15	51243589	*AP4E1*	0.779	0.821	0.829	0.043	0.050	1.27E-17	2.77E-10
cg05492904	15	51604503	*CYP19A1*	0.526	0.440	0.497	-0.086	-0.029	2.08E-91	8.47E-07
cg06192883	15	52554171	*MYO5C*	0.298	0.358	0.338	0.061	0.040	6.37E-55	1.62E-15
cg24590708	15	52554357	*MYO5C*	0.518	0.599	0.557	0.080	0.038	1.28E-67	2.56E-09
cg27633342	15	52588279	*MYO5C*	0.449	0.384	0.421	-0.065	-0.028	7.18E-60	1.85E-06
cg07919744	15	52873609	*FAM214A*	0.734	0.780	0.799	0.047	0.066	9.11E-19	1.65E-16
cg07579107	15	53910786	*WDR72*	0.680	0.737	0.729	0.057	0.048	4.48E-28	4.38E-10
cg00547312	15	55473630	*RSL24D1*	0.681	0.739	0.739	0.058	0.058	5.36E-24	8.66E-12
cg07037635	15	55615438	*PIGB*	0.358	0.284	0.319	-0.073	-0.039	2.04E-86	4.64E-12
cg13160251	15	56286575	*NEDD4*	0.363	0.290	0.332	-0.073	-0.032	7.95E-79	1.51E-08
cg16402415	15	57021851	*ZNF280D*	0.463	0.374	0.429	-0.089	-0.034	8.88E-90	3.46E-09
cg01087254	15	57052190	NA	0.412	0.295	0.357	-0.118	-0.055	6.82E-150	8.56E-21
cg24079727	15	57317980	*TCF12*	0.461	0.348	0.410	-0.113	-0.051	1.01E-146	1.03E-18
cg11098259	15	58430391	*AQP9*	0.395	0.286	0.345	-0.109	-0.050	1.14E-151	2.52E-20
cg03311274	15	58624344	NA	0.287	0.228	0.251	-0.059	-0.036	6.99E-94	9.68E-18
cg20626983	15	58650472	NA	0.735	0.786	0.766	0.051	0.030	4.14E-67	3.15E-13
cg24848787	15	58844359	*LIPC*	0.274	0.213	0.244	-0.061	-0.031	1.71E-62	9.16E-08
cg21149266	15	59146882	*FAM63B*	0.283	0.212	0.253	-0.071	-0.030	9.84E-96	4.47E-11
cg05061769	15	59700360	NA	0.470	0.380	0.441	-0.091	-0.029	4.64E-99	3.99E-07
cg06738887	15	60688010	*ANXA2*	0.362	0.273	0.326	-0.090	-0.036	7.27E-128	1.91E-15
cg19667460	15	60863379	*RORA*	0.750	0.803	0.772	0.052	0.021	2.78E-67	6.96E-07
cg09251291	15	63483946	*RAB8B*	0.685	0.741	0.707	0.057	0.023	1.54E-60	3.01E-06
cg08783514	15	63772233	NA	0.688	0.752	0.723	0.063	0.035	7.67E-61	2.89E-10
cg05265081	15	64723065	*TRIP4*	0.344	0.276	0.317	-0.069	-0.028	2.61E-90	2.56E-09
cg18711535	15	64736348	*TRIP4*	0.712	0.775	0.755	0.063	0.043	7.32E-53	1.19E-11
cg13149245	15	65105500	NA	0.333	0.280	0.303	-0.053	-0.030	3.25E-67	4.06E-10
cg10207553	15	65277977	*SPG21*	0.264	0.205	0.241	-0.059	-0.023	5.99E-85	3.50E-07
cg27192248	15	65285669	NA	0.417	0.371	0.352	-0.046	-0.065	5.00E-08	7.02E-08
cg27665985	15	66116727	NA	0.325	0.265	0.298	-0.060	-0.027	1.52E-59	3.23E-07
cg09492074	15	66263624	*MEGF11*	0.341	0.285	0.315	-0.056	-0.026	1.95E-59	2.01E-07
cg04792910	15	66652179	NA	0.723	0.777	0.761	0.054	0.038	2.11E-40	7.40E-10
cg27058988	15	67200879	NA	0.245	0.179	0.203	-0.066	-0.041	2.22E-75	1.04E-13
cg24231804	15	67316861	NA	0.269	0.207	0.221	-0.062	-0.047	6.74E-75	3.04E-20
cg17184477	15	67360705	*SMAD3*	0.668	0.739	0.698	0.070	0.030	6.10E-139	8.42E-16
cg15799109	15	67418428	*SMAD3*	0.786	0.829	0.841	0.043	0.055	1.29E-18	5.03E-13
cg22706883	15	67814880	*C15orf61*	0.454	0.391	0.411	-0.064	-0.043	4.82E-47	1.97E-11
cg00154119	15	68174054	NA	0.451	0.390	0.427	-0.061	-0.025	7.34E-89	1.87E-08
cg11530995	15	68497992	*CALML4*	0.374	0.324	0.355	-0.050	-0.020	6.82E-65	2.04E-07
cg08642285	15	68498589	*CALML4*	0.236	0.180	0.218	-0.056	-0.018	5.48E-108	8.21E-08
cg25874953	15	69082449	*ANP32A*	0.327	0.271	0.301	-0.056	-0.026	7.19E-84	1.61E-11
cg01176433	15	69754714	NA	0.405	0.351	0.370	-0.054	-0.035	6.00E-25	1.74E-06
cg16125725	15	70101302	NA	0.544	0.411	0.501	-0.133	-0.043	4.57E-156	1.45E-13
cg21024495	15	70393940	NA	0.292	0.235	0.260	-0.057	-0.031	4.08E-44	2.06E-07
cg18978531	15	70403809	NA	0.425	0.375	0.381	-0.050	-0.044	5.59E-42	8.45E-15
cg26962618	15	70767183	NA	0.341	0.256	0.308	-0.084	-0.033	1.42E-89	1.71E-08
cg24405567	15	70787565	NA	0.487	0.373	0.421	-0.114	-0.066	1.17E-135	1.87E-27
cg01229658	15	70924395	NA	0.424	0.333	0.394	-0.092	-0.031	8.40E-101	1.22E-07
cg11937920	15	70995381	*UACA*	0.646	0.698	0.681	0.052	0.035	3.92E-27	1.86E-06
cg12157761	15	71005778	*UACA*	0.502	0.388	0.458	-0.115	-0.044	2.91E-139	2.47E-17
cg11357013	15	73588054	*NEO1*	0.449	0.335	0.400	-0.115	-0.050	1.34E-168	4.67E-21
cg16900255	15	74037743	*C15orf59*	0.389	0.424	0.441	0.035	0.052	5.04E-10	4.03E-09
cg22242148	15	74215283	*LOXL1-AS1*	0.391	0.306	0.356	-0.086	-0.035	2.45E-121	9.09E-13
cg00028013	15	74218697	*LOXL1*	0.567	0.501	0.528	-0.066	-0.039	1.67E-49	3.28E-10
cg09642075	15	74303043	*PML*	0.660	0.601	0.629	-0.059	-0.031	1.82E-65	1.01E-10
cg02430430	15	74532450	*CCDC33*	0.326	0.272	0.297	-0.054	-0.029	6.65E-84	5.12E-15
cg20040747	15	74715105	*SEMA7A*	0.355	0.291	0.326	-0.064	-0.029	7.39E-88	1.60E-12
cg07630255	15	75185989	*MPI*	0.767	0.820	0.792	0.053	0.025	2.49E-88	5.23E-11
cg26488183	15	75195105	*FAM219B*	0.366	0.269	0.317	-0.097	-0.050	9.18E-130	1.13E-19
cg06070324	15	75322058	*PPCDC*	0.364	0.271	0.324	-0.093	-0.040	8.12E-183	2.47E-25
cg01361499	15	75467997	NA	0.334	0.283	0.289	-0.050	-0.045	1.87E-16	5.72E-07
cg12063992	15	75865604	*PTPN9*	0.384	0.313	0.352	-0.072	-0.032	9.73E-73	1.21E-08
cg19013753	15	75915192	*SNUPN*	0.507	0.561	0.534	0.055	0.027	2.50E-48	1.44E-07
cg23032427	15	76635953	NA	0.450	0.392	0.418	-0.058	-0.032	3.85E-52	1.75E-08
cg10577241	15	77456283	*PEAK1*	0.724	0.783	0.762	0.059	0.037	3.41E-68	1.52E-14
cg23387863	15	77472416	*PEAK1*	0.800	0.761	0.747	-0.038	-0.052	5.39E-28	1.08E-27
cg08130265	15	77519170	*PEAK1*	0.486	0.408	0.453	-0.079	-0.034	8.04E-77	1.19E-08
cg20739864	15	78841455	*PSMA4*	0.744	0.794	0.791	0.050	0.047	3.09E-19	1.79E-08
cg19696491	15	78857124	*CHRNA5*	0.538	0.478	0.496	-0.060	-0.042	9.59E-23	2.52E-06
cg19993258	15	78914264	*CHRNA3*	0.658	0.719	0.685	0.061	0.026	1.79E-96	6.58E-13
cg04789403	15	80271218	NA	0.282	0.232	0.233	-0.050	-0.048	2.08E-36	5.43E-16

cg06856840	15	80446451	*FAH*	0.344	0.268	0.307	-0.075	-0.037	9.74E-116	3.02E-16
cg11808699	15	81528661	*IL16*	0.442	0.381	0.397	-0.060	-0.045	8.59E-34	4.15E-10
cg04093633	15	82469848	*EFTUD1*	0.454	0.372	0.412	-0.082	-0.042	1.80E-110	2.89E-16
cg21253742	15	85305079	*ZNF592*	0.667	0.727	0.703	0.060	0.035	1.02E-50	6.56E-09
cg12125241	15	85575240	*PDE8A*	0.712	0.767	0.766	0.055	0.054	2.48E-28	6.35E-12
cg12176856	15	85586841	*PDE8A*	0.755	0.795	0.806	0.040	0.051	1.03E-13	9.70E-11
cg09874482	15	86098694	*AKAP13*	0.365	0.280	0.326	-0.085	-0.038	3.21E-128	7.88E-16
cg13374432	15	86192745	*AKAP13*	0.350	0.273	0.317	-0.078	-0.033	1.49E-78	5.51E-09
cg12058064	15	86315201	*KLHL25*	0.454	0.369	0.426	-0.085	-0.028	8.68E-165	6.13E-15
cg26025224	15	89020059	*MRPS11*	0.646	0.596	0.610	-0.050	-0.036	1.73E-40	7.08E-12
cg07069934	15	89192956	*ISG20*	0.663	0.739	0.688	0.075	0.025	5.56E-70	4.47E-06
cg13460556	15	89709638	*ABHD2*	0.550	0.490	0.520	-0.059	-0.030	7.87E-57	2.73E-08
cg09926562	15	89812172	*FANCI*	0.735	0.786	0.780	0.051	0.046	5.75E-25	3.45E-09
cg25932290	15	89939252	*LOC254559*	0.420	0.325	0.369	-0.096	-0.051	5.50E-116	5.31E-20
cg18443571	15	90547692	*ZNF710*	0.511	0.453	0.482	-0.058	-0.029	6.22E-70	5.38E-10
cg23035449	15	90578555	*ZNF710*	0.361	0.297	0.335	-0.065	-0.027	1.32E-102	2.71E-11
cg11358405	15	91382972	NA	0.250	0.176	0.225	-0.074	-0.025	2.19E-116	6.16E-09
cg27535502	15	91395896	NA	0.745	0.800	0.767	0.055	0.022	4.39E-76	8.75E-08
cg19348484	15	91413236	*FURIN*	0.306	0.233	0.279	-0.073	-0.027	2.08E-122	9.39E-12
cg25647583	15	91427184	*FES*	0.436	0.353	0.404	-0.084	-0.033	5.84E-149	1.03E-17
cg05180856	15	91428056	*FES*	0.428	0.364	0.405	-0.064	-0.023	2.24E-113	3.47E-11
cg06330618	15	91428456	*FES*	0.375	0.314	0.347	-0.061	-0.028	3.17E-104	3.61E-14
cg26899598	15	91429088	*FES*	0.247	0.193	0.228	-0.054	-0.019	1.36E-80	2.54E-06
cg09782560	15	92058845	NA	0.381	0.290	0.335	-0.091	-0.046	4.66E-119	1.77E-17
cg13615971	15	92392821	NA	0.476	0.425	0.447	-0.050	-0.029	2.98E-36	1.03E-06
cg27614723	15	92399897	*SLCO3A1*	0.580	0.524	0.543	-0.056	-0.037	2.26E-57	4.70E-14
cg21594043	15	92491578	*SLCO3A1*	0.395	0.331	0.357	-0.064	-0.038	2.12E-59	8.26E-11
cg18646851	15	92934809	NA	0.411	0.319	0.379	-0.093	-0.033	1.76E-116	1.90E-10
cg14372792	15	93188381	*FAM174B*	0.321	0.264	0.293	-0.057	-0.029	3.32E-55	1.04E-07
cg17934775	15	93450129	*CHD2*	0.553	0.630	0.592	0.077	0.038	9.29E-101	7.08E-15
cg14138540	15	93571988	NA	0.288	0.221	0.265	-0.067	-0.023	7.08E-112	7.00E-09
cg23054181	15	99048945	*FAM169B*	0.523	0.405	0.484	-0.117	-0.039	1.33E-162	5.96E-16
cg25830379	15	99264361	*IGF1R*	0.720	0.782	0.766	0.062	0.047	3.39E-44	2.65E-11
cg27534520	15	99343918	*IGF1R*	0.340	0.266	0.309	-0.074	-0.031	9.45E-94	1.83E-09
cg10389812	15	99458162	*IGF1R*	0.713	0.773	0.764	0.060	0.051	1.44E-35	1.74E-12
cg16261114	15	99551997	*PGPEP1L*	0.425	0.333	0.375	-0.092	-0.049	3.25E-109	1.47E-16
cg01840419	15	100890979	NA	0.264	0.213	0.241	-0.051	-0.023	1.37E-51	1.20E-06
cg24595580	15	100890996	NA	0.440	0.346	0.399	-0.094	-0.041	6.16E-105	5.21E-14
cg05575505	15	101262162	NA	0.543	0.478	0.492	-0.065	-0.051	7.81E-43	6.87E-13
cg05194346	15	101305443	NA	0.594	0.544	0.546	-0.051	-0.048	1.28E-21	2.90E-11
cg13085627	15	101597169	*LRRK1*	0.625	0.547	0.601	-0.078	-0.024	4.48E-98	1.44E-07
cg20357538	15	101777761	*CHSY1*	0.287	0.219	0.264	-0.068	-0.023	3.11E-106	4.31E-08
cg08165960	15	101777800	*CHSY1*	0.320	0.246	0.291	-0.074	-0.029	4.42E-114	9.34E-12
cg19997662	15	101784653	*CHSY1*	0.353	0.272	0.318	-0.081	-0.035	3.15E-71	9.93E-08
cg03462322	16	126397	*MPG*	0.279	0.211	0.257	-0.069	-0.022	5.03E-98	1.66E-06
cg08183303	16	447934	*NME4*	0.401	0.350	0.372	-0.051	-0.029	5.34E-42	1.56E-08
cg03363565	16	474528	*RAB11FIP3*	0.436	0.330	0.397	-0.106	-0.039	7.10E-140	5.23E-14
cg09830866	16	771714	*FAM173A*	0.505	0.585	0.528	0.080	0.023	1.63E-67	2.66E-06
cg02889001	16	1519785	*CLCN7*	0.435	0.339	0.401	-0.097	-0.034	3.12E-104	3.01E-08
cg01042641	16	1575979	*IFT140*	0.559	0.620	0.588	0.061	0.029	1.34E-74	1.15E-09
cg00905101	16	1988798	*MSRB1*	0.350	0.274	0.314	-0.076	-0.036	8.63E-131	5.12E-19
cg05211189	16	2055403	*ZNF598*	0.411	0.338	0.377	-0.073	-0.035	7.47E-83	1.10E-09
cg05251269	16	2083128	*SLC9A3R2*	0.342	0.285	0.319	-0.057	-0.023	1.43E-76	2.64E-09
cg01500402	16	2256716	*MLST8*	0.247	0.197	0.221	-0.050	-0.026	9.50E-90	1.97E-13
cg08276042	16	2740348	*KCTD5*	0.399	0.311	0.364	-0.088	-0.035	1.23E-148	9.74E-20
cg00268476	16	2740589	*KCTD5*	0.440	0.384	0.411	-0.057	-0.029	2.64E-64	7.36E-12
cg02593884	16	2984574	*FLYWCH1*	0.527	0.448	0.506	-0.079	-0.021	8.63E-105	6.68E-07
cg10228555	16	3088480	*LOC1001287*	0.399	0.330	0.379	-0.070	-0.020	5.41E-91	2.57E-06
cg08978665	16	3115707	*IL32*	0.764	0.819	0.790	0.055	0.026	5.11E-59	4.06E-08
cg08659421	16	3117862	*IL32*	0.682	0.742	0.712	0.060	0.030	2.39E-91	2.87E-14
cg08937107	16	3136857	NA	0.335	0.271	0.312	-0.065	-0.024	9.64E-89	2.23E-08
cg00005166	16	3264442	NA	0.688	0.632	0.660	-0.056	-0.028	5.25E-43	6.47E-07
cg07938480	16	3265228	NA	0.589	0.646	0.639	0.057	0.051	7.87E-29	2.58E-12
cg03689552	16	3292971	*MEFV*	0.443	0.333	0.398	-0.110	-0.046	1.77E-153	1.16E-19
cg09934892	16	3559551	*CLUAP1*	0.676	0.734	0.696	0.058	0.020	3.17E-76	1.66E-06
cg08367804	16	3639160	*SLX4*	0.492	0.420	0.470	-0.072	-0.022	3.39E-90	1.14E-06
cg01352090	16	4103533	*ADCY9*	0.251	0.189	0.227	-0.062	-0.024	9.07E-99	2.65E-09
cg07906046	16	4131584	*ADCY9*	0.561	0.631	0.586	0.070	0.025	2.57E-70	4.05E-06
cg03905718	16	4289417	*SRL*	0.499	0.434	0.464	-0.065	-0.035	5.63E-48	8.67E-07
cg08494738	16	4369512	NA	0.705	0.777	0.729	0.072	0.024	4.65E-102	9.79E-08
cg01406317	16	4397291	*PAM16*	0.441	0.330	0.395	-0.110	-0.046	3.69E-184	1.31E-23
cg03427191	16	4697112	*MGRN1*	0.725	0.785	0.752	0.060	0.027	1.43E-98	1.21E-10
cg00736299	16	4730465	*MGRN1*	0.513	0.431	0.493	-0.082	-0.020	5.89E-105	2.41E-06
cg27050111	16	8738240	*METTL22*	0.360	0.288	0.335	-0.072	-0.025	6.20E-92	1.20E-06
cg08703231	16	8738304	*METTL22*	0.433	0.343	0.397	-0.090	-0.036	5.15E-151	1.01E-17
cg04234631	16	8738441	*METTL22*	0.493	0.421	0.468	-0.072	-0.026	2.31E-104	1.85E-08
cg02435083	16	8943436	NA	0.385	0.293	0.347	-0.092	-0.038	5.90E-153	7.92E-19
cg26434370	16	10909361	*FAM18A*	0.466	0.362	0.420	-0.104	-0.046	4.74E-175	1.29E-25

cg03014241	16	11348611	*SOCS1*	0.720	0.777	0.759	0.057	0.039	1.78E-54	8.83E-15
cg02301079	16	11591651	NA	0.275	0.222	0.254	-0.052	-0.020	1.87E-86	2.74E-09
cg01891736	16	11655159	*LITAF*	0.502	0.557	0.554	0.055	0.052	2.08E-33	1.42E-15
cg27097542	16	11706435	NA	0.361	0.290	0.339	-0.071	-0.022	4.49E-100	1.93E-07
cg09662852	16	11707685	NA	0.300	0.238	0.265	-0.062	-0.035	8.52E-64	3.06E-12
cg09481483	16	11734383	NA	0.291	0.222	0.264	-0.069	-0.027	1.50E-94	1.54E-08
cg03382501	16	11794641	*TXNDC11*	0.306	0.225	0.273	-0.081	-0.032	4.38E-113	5.56E-11
cg08633074	16	11837719	*TXNDC11*	0.404	0.300	0.359	-0.104	-0.045	4.40E-121	6.27E-12
cg08160706	16	11876111	*ZC3H7A*	0.801	0.840	0.851	0.039	0.050	5.88E-17	1.85E-13
cg02386311	16	12073297	*SNX29*	0.357	0.296	0.314	-0.062	-0.043	1.14E-50	6.37E-16
cg01858712	16	12156525	*SNX29*	0.335	0.257	0.300	-0.078	-0.035	3.63E-113	1.43E-13
cg04018325	16	14397685	NA	0.397	0.343	0.373	-0.054	-0.024	5.72E-41	2.46E-06
cg26985681	16	15135397	*NTAN1*	0.427	0.348	0.395	-0.079	-0.032	1.61E-80	6.19E-08
cg08220149	16	15766327	*NDE1*	0.339	0.252	0.309	-0.087	-0.030	2.05E-82	4.44E-06
cg03308839	16	15797297	*NDE1*	0.503	0.384	0.459	-0.119	-0.045	4.97E-138	2.09E-12
cg01281601	16	15931190	*MYH11*	0.683	0.633	0.660	-0.051	-0.023	5.24E-49	5.51E-07
cg10111352	16	16082095	*ABCC1*	0.386	0.304	0.355	-0.082	-0.031	5.75E-101	1.47E-09
cg04730825	16	16116191	*ABCC1*	0.357	0.299	0.331	-0.058	-0.027	2.03E-98	1.98E-12
cg08363067	16	16170085	*ABCC1*	0.401	0.315	0.367	-0.086	-0.034	4.92E-165	2.34E-20
cg01332683	16	16190393	*ABCC1*	0.443	0.368	0.404	-0.075	-0.040	7.88E-104	3.13E-16
cg09770579	16	17407429	*XYLT1*	0.490	0.429	0.451	-0.061	-0.040	1.59E-60	4.37E-14
cg02009395	16	20702744	*ACSM1*	0.673	0.728	0.714	0.054	0.041	5.24E-33	1.52E-09
cg06654079	16	21169179	*TMEM159*	0.463	0.362	0.427	-0.101	-0.036	1.33E-112	9.50E-09
cg25957124	16	21171084	*TMEM159*	0.356	0.278	0.331	-0.079	-0.025	2.64E-125	1.61E-09
cg04528038	16	21171097	*TMEM159*	0.279	0.213	0.261	-0.067	-0.018	1.79E-98	6.27E-06
cg05240166	16	21171239	*TMEM159*	0.443	0.356	0.406	-0.087	-0.037	1.74E-77	1.08E-07
cg01574390	16	21623651	*METTL9*	0.382	0.300	0.348	-0.082	-0.033	6.18E-133	2.03E-15
cg01369829	16	22029878	*C16orf52*	0.724	0.772	0.775	0.047	0.051	7.44E-19	6.47E-11
cg26828017	16	22409023	NA	0.459	0.369	0.428	-0.090	-0.031	1.52E-89	2.06E-07
cg09529437	16	24136792	*PRKCB*	0.347	0.289	0.319	-0.058	-0.027	7.17E-74	6.08E-09
cg02115302	16	27237719	*NSMCE1*	0.732	0.798	0.750	0.066	0.018	2.54E-153	1.31E-08
cg02654449	16	27254785	*NSMCE1*	0.252	0.198	0.225	-0.054	-0.027	4.90E-125	8.23E-18
cg08008352	16	27322356	NA	0.356	0.280	0.328	-0.076	-0.028	6.89E-104	9.39E-10
cg02656594	16	27412496	*IL21R*	0.465	0.365	0.401	-0.100	-0.064	2.12E-126	9.04E-28
cg02787852	16	27414536	*IL21R*	0.669	0.737	0.696	0.067	0.027	2.15E-120	2.19E-13
cg10454258	16	27443933	*IL21R*	0.436	0.354	0.394	-0.082	-0.042	6.92E-94	2.13E-14
cg02869960	16	28206097	*XPO6*	0.746	0.798	0.785	0.051	0.039	3.96E-23	3.62E-07
cg17122311	16	28517278	*IL27*	0.610	0.552	0.586	-0.059	-0.024	1.41E-60	1.18E-06
cg00201760	16	28518385	*IL27*	0.400	0.324	0.367	-0.076	-0.032	3.39E-99	1.75E-10
cg07866632	16	28584802	*CCDC101*	0.372	0.301	0.349	-0.070	-0.022	1.76E-122	5.68E-09
cg08961793	16	28628118	*SULT1A1*	0.423	0.328	0.375	-0.095	-0.048	5.92E-131	2.06E-20
cg06208158	16	29168730	NA	0.372	0.305	0.345	-0.067	-0.027	1.66E-107	1.97E-11
cg02640604	16	29690271	*QPRT*	0.445	0.376	0.408	-0.068	-0.037	6.24E-71	5.76E-12
cg06453916	16	29690524	*QPRT*	0.341	0.276	0.298	-0.065	-0.042	1.00E-82	1.72E-17
cg02747254	16	29938183	*KCTD13*	0.423	0.365	0.388	-0.059	-0.035	2.74E-66	1.60E-12
cg05329317	16	30126595	*MAPK3*	0.742	0.796	0.775	0.053	0.033	1.34E-72	2.94E-13
cg27583010	16	30198505	*CORO1A*	0.508	0.622	0.538	0.114	0.030	3.65E-131	3.30E-08
cg06749872	16	30198509	*CORO1A*	0.570	0.649	0.593	0.079	0.023	6.39E-133	4.17E-09
cg26843872	16	30368792	*TBC1D10B*	0.358	0.271	0.320	-0.088	-0.038	1.90E-120	2.53E-14
cg16391678	16	30485597	*ITGAL*	0.700	0.787	0.722	0.087	0.022	3.61E-125	1.59E-06
cg07000116	16	30616649	*ZNF689*	0.552	0.434	0.502	-0.118	-0.050	9.62E-132	1.10E-15
cg00084577	16	30721139	*SRCAP*	0.599	0.517	0.574	-0.082	-0.026	4.54E-78	4.70E-06
cg09674546	16	30721220	*SRCAP*	0.336	0.273	0.311	-0.063	-0.025	4.33E-99	1.12E-10
cg03836521	16	30721270	*SRCAP*	0.437	0.385	0.418	-0.052	-0.019	5.10E-90	2.46E-09
cg25773585	16	30721715	*SRCAP*	0.447	0.368	0.416	-0.078	-0.031	1.05E-69	8.30E-07
cg01688936	16	30770794	*C16orf93*	0.457	0.390	0.435	-0.067	-0.023	1.52E-113	4.21E-10
cg05322500	16	30824294	NA	0.264	0.198	0.233	-0.066	-0.031	9.23E-154	1.21E-21
cg27239280	16	30907679	*CTF1*	0.433	0.376	0.399	-0.056	-0.033	3.19E-45	2.60E-09
cg10421029	16	30936028	*FBXL19*	0.301	0.246	0.264	-0.055	-0.038	1.50E-82	5.86E-20
cg03984055	16	31117318	NA	0.337	0.286	0.321	-0.051	-0.016	8.47E-81	5.69E-07
cg05299836	16	31119067	*BCKDK*	0.681	0.753	0.700	0.071	0.018	5.83E-136	3.49E-07
cg04407248	16	31148196	*PRSS8*	0.582	0.491	0.550	-0.092	-0.032	3.46E-96	6.90E-09
cg01059100	16	31214607	*PYCARD*	0.328	0.268	0.295	-0.060	-0.033	9.10E-79	2.92E-13
cg08729927	16	31467076	NA	0.370	0.313	0.339	-0.057	-0.031	2.55E-61	1.57E-09
cg04770282	16	46826414	NA	0.683	0.721	0.733	0.038	0.050	5.37E-13	1.24E-09
cg04106092	16	47760096	NA	0.633	0.580	0.599	-0.054	-0.034	1.96E-55	2.34E-12
cg02938045	16	48222834	*ABCC11*	0.707	0.635	0.684	-0.072	-0.023	2.59E-98	4.27E-08
cg03977385	16	48468609	NA	0.268	0.206	0.237	-0.062	-0.031	4.25E-98	1.36E-13
cg02961101	16	48533155	NA	0.309	0.245	0.272	-0.064	-0.037	1.29E-68	2.27E-12
cg01014262	16	48533930	NA	0.311	0.235	0.272	-0.076	-0.039	4.89E-123	1.48E-18
cg16771652	16	50730385	*NOD2*	0.268	0.214	0.245	-0.054	-0.023	1.84E-53	2.12E-06
cg04172533	16	50743027	*NOD2*	0.315	0.253	0.278	-0.062	-0.037	9.67E-75	6.49E-14
cg05261686	16	50743296	*NOD2*	0.348	0.289	0.323	-0.059	-0.026	5.73E-55	2.43E-06
cg05094137	16	53125208	*CHD9*	0.262	0.212	0.234	-0.050	-0.028	1.51E-61	3.49E-09
cg05768044	16	53243559	*CHD9*	0.438	0.345	0.390	-0.093	-0.048	6.57E-113	9.71E-17
cg00625110	16	53741731	*FTO*	0.417	0.335	0.388	-0.082	-0.029	3.19E-87	2.63E-07
cg26580413	16	54025348	*FTO*	0.363	0.308	0.325	-0.055	-0.038	1.12E-48	2.37E-13
cg06434738	16	54115019	*FTO*	0.730	0.775	0.785	0.045	0.054	3.41E-20	1.26E-13

cg09812990	16	55367838	NA	0.273	0.222	0.248	-0.051	-0.025	2.01E-61	1.14E-07
cg06549901	16	55851316	*CES1*	0.657	0.604	0.621	-0.053	-0.036	5.48E-32	7.49E-08
cg27073113	16	56228744	*GNAO1*	0.312	0.248	0.287	-0.064	-0.025	9.66E-62	5.12E-06
cg27369423	16	56228901	*GNAO1*	0.306	0.223	0.279	-0.082	-0.027	6.65E-114	1.17E-07
cg16411857	16	57023191	NA	0.265	0.190	0.202	-0.075	-0.063	3.71E-58	4.01E-20
cg02670686	16	57125674	*CPNE2*	0.258	0.201	0.228	-0.057	-0.030	1.55E-76	3.42E-13
cg05481929	16	57505058	*POLR2C*	0.577	0.518	0.548	-0.058	-0.029	1.73E-59	3.54E-08
cg16547186	16	57512988	*DOK4*	0.297	0.238	0.267	-0.059	-0.030	1.97E-64	6.15E-09
cg26955845	16	57680131	*GPR56*	0.564	0.508	0.534	-0.056	-0.030	1.48E-48	1.60E-08
cg08368934	16	57701455	*GPR97*	0.436	0.359	0.410	-0.077	-0.026	6.41E-133	3.64E-11
cg12125117	16	57701461	*GPR97*	0.428	0.364	0.406	-0.064	-0.022	2.12E-103	1.20E-08
cg02042712	16	57702239	*GPR97*	0.314	0.258	0.280	-0.056	-0.035	9.10E-86	3.12E-19
cg08894588	16	58027941	NA	0.607	0.538	0.583	-0.069	-0.024	2.24E-80	6.61E-07
cg02990302	16	58155189	*C16orf80*	0.428	0.347	0.392	-0.082	-0.037	5.44E-97	7.31E-11
cg08987251	16	58769104	*GOT2*	0.457	0.393	0.426	-0.064	-0.031	1.36E-51	6.31E-07
cg02078525	16	66400395	*CDH5*	0.368	0.304	0.336	-0.064	-0.032	2.68E-85	4.70E-14
cg00401972	16	66400404	*CDH5*	0.470	0.406	0.439	-0.064	-0.031	5.38E-88	3.23E-13
cg00044665	16	66400411	*CDH5*	0.373	0.304	0.342	-0.069	-0.031	2.65E-118	7.76E-17
cg04466886	16	66521191	*BEAN1*	0.392	0.336	0.363	-0.057	-0.030	8.24E-110	1.69E-18
cg04701716	16	66596317	*CKLF*	0.698	0.755	0.741	0.057	0.043	4.53E-33	2.75E-09
cg09504873	16	66774626	*DYNC1LI2*	0.438	0.352	0.410	-0.085	-0.028	2.96E-96	6.26E-07
cg06352352	16	67181466	*C16orf70*	0.477	0.392	0.443	-0.086	-0.034	3.13E-123	8.86E-13
cg26719831	16	67279967	*FHOD1*	0.292	0.237	0.264	-0.054	-0.028	1.23E-95	1.69E-14
cg05382012	16	67517841	*AGRP*	0.485	0.406	0.450	-0.079	-0.035	1.68E-85	3.61E-10
cg09086087	16	68000763	*SLC12A4*	0.404	0.347	0.385	-0.057	-0.019	1.49E-116	1.40E-09
cg10512278	16	68027839	*DPEP2*	0.365	0.310	0.344	-0.055	-0.021	2.38E-88	7.73E-10
cg04304036	16	68334619	*SLC7A6OS*	0.430	0.332	0.398	-0.097	-0.032	1.88E-131	7.47E-11
cg10061770	16	68366844	*PRMT7*	0.377	0.280	0.338	-0.098	-0.039	3.51E-135	5.62E-13
cg10313337	16	68823690	*CDH1*	0.483	0.369	0.442	-0.114	-0.041	3.29E-209	5.09E-23
cg08327038	16	69200251	*CIRH1A*	0.759	0.813	0.785	0.054	0.026	2.92E-61	2.27E-07
cg04703221	16	69967063	*WWP2*	0.442	0.351	0.405	-0.091	-0.037	2.05E-67	1.84E-07
cg05261349	16	70473841	*ST3GAL2*	0.552	0.493	0.527	-0.059	-0.025	1.82E-67	3.07E-07
cg00864954	16	70587308	*SF3B3*	0.745	0.796	0.765	0.051	0.020	8.67E-94	4.03E-08
cg22381196	16	72041376	*DHODH*	0.502	0.384	0.460	-0.118	-0.042	3.09E-191	1.27E-19
cg06172871	16	72088244	*HP*	0.398	0.304	0.353	-0.094	-0.046	8.53E-144	3.42E-22
cg09699830	16	72516874	NA	0.693	0.769	0.740	0.076	0.046	8.52E-73	1.13E-14
cg00528616	16	75036318	*ZNRF1*	0.311	0.241	0.280	-0.070	-0.032	7.65E-118	2.59E-15
cg27641961	16	75097037	*ZNRF1*	0.361	0.282	0.326	-0.079	-0.035	6.08E-106	8.07E-12
cg05552543	16	75322715	NA	0.594	0.651	0.620	0.057	0.025	2.09E-142	2.15E-19
cg07861456	16	75654252	*ADAT1*	0.319	0.261	0.295	-0.059	-0.024	1.68E-51	6.44E-06
cg27027055	16	79306292	NA	0.738	0.798	0.767	0.059	0.029	3.91E-94	6.20E-13
cg04851702	16	81528974	*CMIP*	0.304	0.235	0.282	-0.069	-0.022	4.24E-98	3.28E-06
cg06823060	16	81616874	*CMIP*	0.597	0.503	0.569	-0.095	-0.028	2.91E-112	1.32E-07
cg05315365	16	82096597	*HSD17B2*	0.680	0.610	0.654	-0.070	-0.025	1.49E-76	1.46E-07
cg06528601	16	84652562	*COTL1*	0.415	0.361	0.389	-0.054	-0.025	4.91E-62	1.95E-08
cg05128520	16	84676746	NA	0.431	0.353	0.393	-0.078	-0.038	7.12E-82	4.91E-10
cg26562921	16	84760429	*USP10*	0.350	0.273	0.326	-0.077	-0.024	1.80E-110	3.92E-08
cg06465011	16	84860871	*CRISPLD2*	0.469	0.366	0.430	-0.103	-0.039	4.03E-109	5.96E-12
cg02579377	16	85047901	NA	0.394	0.300	0.363	-0.094	-0.031	2.74E-114	2.91E-09
cg07165260	16	85062881	*KIAA0513*	0.649	0.734	0.678	0.085	0.029	1.09E-135	1.67E-11
cg27260684	16	85063742	*KIAA0513*	0.468	0.359	0.427	-0.109	-0.041	4.20E-145	4.31E-13
cg03543954	16	85116335	*KIAA0513*	0.290	0.217	0.265	-0.072	-0.025	2.92E-95	1.44E-06
cg02853355	16	85132373	*FAM92B*	0.537	0.453	0.507	-0.084	-0.031	3.86E-78	3.49E-07
cg00036976	16	85343281	NA	0.274	0.219	0.246	-0.055	-0.028	1.30E-91	4.10E-14
cg00834923	16	85393998	NA	0.706	0.758	0.729	0.052	0.023	1.19E-68	6.13E-09
cg08550517	16	85414644	NA	0.477	0.398	0.440	-0.079	-0.037	9.43E-82	8.06E-13
cg05474761	16	85414689	NA	0.380	0.324	0.356	-0.056	-0.025	1.35E-54	4.78E-08
cg27614309	16	85478580	NA	0.733	0.785	0.761	0.052	0.028	2.48E-89	2.23E-14
cg09363128	16	85478932	NA	0.653	0.704	0.674	0.051	0.021	2.14E-61	2.04E-06
cg09832245	16	85494611	NA	0.380	0.291	0.339	-0.090	-0.041	8.71E-127	5.70E-16
cg26963632	16	85558148	NA	0.287	0.233	0.246	-0.054	-0.042	1.38E-64	1.89E-21
cg09734761	16	85569506	NA	0.515	0.436	0.484	-0.079	-0.031	2.17E-92	3.75E-09
cg07298177	16	85577847	NA	0.471	0.373	0.432	-0.098	-0.039	3.23E-148	2.36E-18
cg02143404	16	85607651	NA	0.458	0.406	0.426	-0.052	-0.032	1.18E-41	1.93E-08
cg00830121	16	85904858	NA	0.747	0.803	0.799	0.057	0.052	1.84E-26	5.44E-11
cg02549595	16	85947219	*IRF8*	0.566	0.505	0.543	-0.062	-0.024	1.60E-58	4.76E-06
cg02178957	16	85964200	NA	0.534	0.484	0.512	-0.050	-0.022	4.33E-60	9.76E-07
cg08428292	16	85981373	NA	0.502	0.597	0.524	0.095	0.023	1.62E-127	6.98E-07
cg09714919	16	85981947	NA	0.394	0.325	0.364	-0.069	-0.030	4.45E-96	7.27E-13
cg01787084	16	87371097	*FBXO31*	0.420	0.294	0.354	-0.126	-0.066	1.43E-126	1.05E-19
cg08830502	16	87588559	NA	0.576	0.494	0.544	-0.082	-0.033	2.41E-118	1.52E-11
cg00858400	16	87904580	*SLC7A5*	0.525	0.452	0.486	-0.073	-0.039	1.95E-97	7.64E-18
cg01445100	16	88103339	*BANP*	0.700	0.757	0.726	0.057	0.026	2.92E-91	2.85E-10
cg03776194	16	88770966	*RNF166*	0.457	0.527	0.487	0.070	0.030	5.42E-90	2.67E-11
cg10491452	16	88866627	NA	0.288	0.214	0.258	-0.074	-0.030	1.74E-74	3.90E-07
cg08472633	16	88907370	*GALNS*	0.396	0.324	0.375	-0.072	-0.021	6.41E-103	4.77E-06
cg05579598	16	88989069	*CBFA2T3*	0.422	0.365	0.397	-0.057	-0.025	8.09E-49	3.19E-06
cg08698997	16	88989212	*CBFA2T3*	0.305	0.255	0.265	-0.050	-0.040	1.52E-38	3.38E-12

cg27061485	16	88989387	*CBFA2T3*	0.591	0.540	0.564	-0.052	-0.027	3.18E-36	3.75E-06
cg05557991	16	89003641	*CBFA2T3*	0.281	0.207	0.257	-0.074	-0.024	6.42E-98	5.68E-06
cg01366670	16	89004378	*CBFA2T3*	0.379	0.319	0.355	-0.060	-0.024	4.74E-130	2.27E-14
cg04540712	16	89009691	*CBFA2T3*	0.721	0.773	0.742	0.052	0.021	1.45E-71	7.43E-08
cg23606023	16	89042948	*CBFA2T3*	0.239	0.189	0.217	-0.051	-0.023	5.19E-87	8.46E-11
cg06436854	16	89043527	*CBFA2T3*	0.190	0.136	0.164	-0.054	-0.026	4.87E-57	3.36E-07
cg01404893	16	89043547	*CBFA2T3*	0.361	0.285	0.322	-0.076	-0.039	1.42E-114	1.31E-20
cg03172657	16	89163625	*ACSF3*	0.455	0.534	0.525	0.080	0.071	4.56E-34	3.79E-13
cg05453411	16	89300123	NA	0.324	0.254	0.304	-0.070	-0.019	1.74E-178	4.40E-11
cg07929642	16	89390685	*LOC1002870*	0.768	0.826	0.786	0.058	0.018	2.94E-87	4.94E-06
cg00759807	16	89390789	*LOC1002870*	0.635	0.729	0.667	0.094	0.032	1.36E-119	1.52E-09
cg03517226	16	89408322	*ANKRD11*	0.752	0.805	0.769	0.053	0.017	3.57E-103	2.81E-07
cg20391948	16	89408367	*ANKRD11*	0.654	0.712	0.674	0.058	0.020	2.55E-75	3.73E-06
cg06159562	16	89424793	*ANKRD11*	0.543	0.627	0.592	0.085	0.050	2.81E-54	3.92E-12
cg02226192	16	89461734	*ANKRD11*	0.484	0.393	0.466	-0.091	-0.018	8.49E-165	1.06E-06
cg05320533	16	89541815	*ANKRD11*	0.652	0.705	0.696	0.054	0.044	6.96E-22	1.91E-07
cg03809021	16	89831123	*FANCA*	0.436	0.376	0.385	-0.061	-0.052	5.42E-18	4.00E-07
cg05399244	17	179611	*LOC1005063*	0.412	0.336	0.370	-0.075	-0.042	3.77E-98	1.87E-18
cg25170091	17	202716	*RPH3AL*	0.350	0.294	0.311	-0.057	-0.039	2.63E-31	4.37E-08
cg07881210	17	398090	NA	0.319	0.256	0.291	-0.063	-0.028	1.22E-74	1.34E-08
cg08106108	17	695095	*RNMTL1*	0.701	0.626	0.675	-0.075	-0.026	1.76E-82	1.99E-07
cg01841306	17	908923	*ABR*	0.379	0.323	0.355	-0.056	-0.024	9.42E-72	3.07E-08
cg14236443	17	943270	*ABR*	0.469	0.416	0.444	-0.053	-0.025	9.72E-58	1.26E-08
cg16374343	17	1014352	*ABR*	0.391	0.329	0.364	-0.062	-0.027	5.15E-128	1.46E-16
cg04042333	17	1104665	NA	0.675	0.768	0.714	0.094	0.040	5.84E-113	5.47E-11
cg19504184	17	1170806	NA	0.324	0.267	0.290	-0.057	-0.034	7.30E-89	2.91E-20
cg04204452	17	1479213	*SLC43A2*	0.449	0.380	0.428	-0.069	-0.021	6.25E-91	1.57E-06
cg13937155	17	1548881	*SCARF1*	0.377	0.296	0.336	-0.081	-0.041	2.17E-91	9.68E-13
cg00869668	17	1549012	*SCARF1*	0.390	0.308	0.354	-0.082	-0.036	4.09E-113	1.72E-15
cg12485428	17	1549098	*SCARF1*	0.410	0.326	0.372	-0.084	-0.038	2.84E-156	5.48E-24
cg08677655	17	1549108	*SCARF1*	0.268	0.205	0.237	-0.063	-0.031	2.68E-68	3.01E-10
cg25166437	17	1549284	*SCARF1*	0.281	0.219	0.250	-0.063	-0.031	2.76E-110	1.74E-16
cg06053959	17	1617291	*MIR22HG*	0.407	0.330	0.360	-0.077	-0.046	4.27E-110	2.33E-28
cg22516975	17	1617465	*MIR22HG*	0.376	0.307	0.351	-0.068	-0.025	5.54E-122	9.88E-11
cg22242539	17	1665220	*SERPINF1*	0.365	0.302	0.335	-0.063	-0.030	3.91E-103	2.36E-14
cg15816464	17	2026533	*SMG6*	0.328	0.265	0.305	-0.063	-0.023	1.41E-89	6.00E-08
cg23403980	17	2060623	*SMG6*	0.346	0.274	0.316	-0.072	-0.030	1.79E-150	1.79E-22
cg12509499	17	2095179	*SMG6*	0.546	0.461	0.519	-0.085	-0.027	1.84E-81	3.98E-06
cg19869422	17	2186014	*SMG6*	0.592	0.537	0.564	-0.055	-0.028	3.71E-60	4.93E-09
cg09635667	17	2300514	*MNT*	0.409	0.327	0.382	-0.082	-0.027	1.12E-152	1.70E-14
cg04960169	17	2839005	*RAP1GAP2*	0.403	0.320	0.371	-0.083	-0.032	5.34E-107	2.45E-09
cg09915396	17	2907895	*RAP1GAP2*	0.333	0.273	0.314	-0.060	-0.019	5.87E-73	6.25E-06
cg00463732	17	3704621	*ITGAE*	0.448	0.369	0.424	-0.079	-0.024	3.32E-97	4.23E-07
cg11239575	17	3705765	*ITGAE*	0.577	0.478	0.549	-0.100	-0.029	5.35E-106	2.56E-07
cg06468347	17	3705875	*ITGAE*	0.371	0.281	0.338	-0.090	-0.033	1.17E-112	9.41E-09
cg14587065	17	3807247	*P2RX1*	0.466	0.411	0.443	-0.056	-0.024	5.89E-70	3.11E-07
cg22044342	17	3819339	*P2RX1*	0.453	0.367	0.428	-0.086	-0.026	1.06E-89	5.63E-06
cg25031824	17	3819363	*P2RX1*	0.511	0.413	0.467	-0.098	-0.044	1.68E-106	4.31E-17
cg24886563	17	3820150	*P2RX1*	0.282	0.231	0.261	-0.052	-0.022	5.96E-93	1.83E-09
cg07005444	17	3820796	*P2RX1*	0.386	0.317	0.364	-0.069	-0.022	1.37E-118	7.48E-10
cg22437987	17	3820910	*P2RX1*	0.251	0.195	0.226	-0.056	-0.024	9.21E-97	1.55E-11
cg11477010	17	4079262	*ANKFY1*	0.772	0.825	0.799	0.053	0.028	1.89E-68	7.03E-11
cg00277591	17	4079652	*ANKFY1*	0.707	0.784	0.744	0.077	0.036	2.47E-99	2.93E-17
cg13468144	17	4081428	*ANKFY1*	0.609	0.490	0.566	-0.119	-0.043	2.12E-132	1.38E-12
cg02052762	17	4090525	*ANKFY1*	0.534	0.399	0.479	-0.136	-0.055	2.75E-172	5.78E-23
cg23371655	17	4225165	*UBE2G1*	0.707	0.749	0.764	0.043	0.058	8.96E-15	3.42E-12
cg23123250	17	4440116	*SPNS2*	0.450	0.371	0.414	-0.079	-0.036	4.40E-73	1.76E-09
cg20438687	17	4761644	*MINK1*	0.741	0.684	0.711	-0.057	-0.030	9.43E-60	7.13E-11
cg19060895	17	5419436	*NLRP1*	0.402	0.343	0.381	-0.060	-0.021	8.11E-99	5.44E-08
cg06426114	17	6355097	*PITPNM3*	0.623	0.565	0.578	-0.058	-0.045	1.33E-25	1.50E-08
cg13221924	17	6495080	*KIAA0753*	0.479	0.378	0.435	-0.101	-0.045	1.51E-140	1.55E-18
cg03161309	17	6563585	*ALOX15P1*	0.332	0.256	0.303	-0.076	-0.029	1.41E-87	1.95E-07
cg07142009	17	6656315	NA	0.303	0.223	0.259	-0.080	-0.045	1.17E-45	2.47E-06
cg26661623	17	7019262	*ASGR2*	0.564	0.498	0.529	-0.066	-0.035	8.04E-51	9.11E-08
cg05148465	17	7034129	NA	0.346	0.261	0.316	-0.085	-0.030	6.77E-116	4.63E-09
cg11316905	17	7035367	NA	0.328	0.273	0.307	-0.054	-0.021	4.89E-94	6.00E-10
cg14018648	17	7083015	*ASGR1*	0.427	0.344	0.395	-0.082	-0.032	5.23E-105	1.07E-10
cg05331340	17	7083064	*ASGR1*	0.481	0.415	0.455	-0.067	-0.026	3.91E-102	6.05E-10
cg25627098	17	7328734	*C17orf74*	0.242	0.190	0.221	-0.052	-0.021	4.81E-76	1.82E-07
cg12045829	17	7452926	*TNFSF12*	0.531	0.617	0.566	0.086	0.035	1.47E-60	4.04E-06
cg05872923	17	7461260	*TNFSF13*	0.322	0.244	0.293	-0.078	-0.029	9.73E-93	1.61E-07
cg13358186	17	7461775	*TNFSF13*	0.288	0.224	0.261	-0.065	-0.027	7.46E-139	2.36E-16
cg01479396	17	7529693	*SAT2*	0.348	0.253	0.313	-0.096	-0.035	8.40E-161	1.08E-17
cg02308232	17	7742762	*KDM6B*	0.466	0.361	0.428	-0.105	-0.038	2.46E-136	2.66E-13
cg12353788	17	7792061	*CHD3*	0.659	0.736	0.686	0.077	0.027	4.74E-114	3.33E-09
cg04674060	17	7792063	*CHD3*	0.535	0.618	0.568	0.082	0.033	1.19E-146	2.98E-17
cg00582663	17	7815834	*CHD3*	0.393	0.309	0.355	-0.084	-0.038	5.83E-102	1.29E-11
cg02067584	17	8094552	*C17orf59*	0.461	0.345	0.420	-0.116	-0.041	1.77E-126	1.11E-10

cg04460364	17	8370017	*NDEL1*	0.514	0.417	0.486	-0.097	-0.029	4.89E-129	1.07E-08
cg00409104	17	8762014	*PIK3R6*	0.358	0.275	0.321	-0.083	-0.036	1.64E-159	2.90E-22
cg01565508	17	8869961	*PIK3R5*	0.472	0.367	0.435	-0.105	-0.037	1.57E-131	3.34E-11
cg06001524	17	9264709	*STX8*	0.492	0.383	0.447	-0.109	-0.046	4.38E-141	1.76E-16
cg20207911	17	9802796	*RCVRN*	0.515	0.445	0.487	-0.070	-0.028	1.81E-62	5.54E-06
cg03762242	17	9940004	*GAS7*	0.332	0.260	0.301	-0.073	-0.031	1.02E-126	1.32E-15
cg18369972	17	9940121	*GAS7*	0.357	0.279	0.315	-0.078	-0.043	2.72E-67	1.12E-11
cg16768966	17	9940227	*GAS7*	0.463	0.370	0.424	-0.093	-0.040	4.82E-108	1.24E-12
cg16842280	17	10422189	*MYH1*	0.735	0.785	0.773	0.050	0.039	1.05E-23	2.17E-07
cg06082141	17	10604565	*ADPRM*	0.718	0.768	0.775	0.050	0.057	2.36E-18	4.91E-11
cg16533245	17	10612342	*ADPRM*	0.758	0.795	0.811	0.036	0.053	4.57E-09	7.02E-08
cg02077256	17	15133981	*PMP22*	0.684	0.743	0.718	0.059	0.033	7.01E-38	2.51E-06
cg26706520	17	15655637	NA	0.702	0.759	0.734	0.057	0.032	1.64E-42	1.68E-07
cg07563400	17	15849556	*ADORA2B*	0.345	0.295	0.314	-0.050	-0.031	4.86E-49	2.89E-11
cg08826152	17	15869607	*ADORA2B*	0.365	0.293	0.336	-0.072	-0.029	1.16E-63	3.69E-06
cg06857116	17	15885326	*ZSWIM7*	0.518	0.419	0.464	-0.099	-0.054	1.03E-125	9.88E-23
cg27615938	17	16344574	*C17orf76-AS.*	0.348	0.288	0.316	-0.059	-0.032	7.84E-58	5.67E-10
cg11733958	17	17023057	*MPRIP*	0.724	0.776	0.748	0.052	0.025	8.07E-78	2.19E-09
cg08024038	17	17616235	*RAI1*	0.489	0.424	0.463	-0.065	-0.026	4.97E-79	1.17E-07
cg26588076	17	17741631	*SREBF1*	0.322	0.242	0.292	-0.080	-0.030	1.06E-118	3.75E-10
cg14831128	17	18780795	*PRPSAP2*	0.704	0.757	0.747	0.053	0.042	3.11E-23	1.68E-07
cg25708364	17	18905779	*SLC5A10*	0.451	0.384	0.421	-0.067	-0.029	5.41E-84	1.70E-10
cg09606564	17	19290353	*MFAP4*	0.227	0.168	0.204	-0.059	-0.023	1.52E-65	9.62E-07
cg04384031	17	19631485	NA	0.296	0.225	0.267	-0.071	-0.029	5.21E-137	2.75E-14
cg08817867	17	19656554	NA	0.304	0.235	0.273	-0.070	-0.031	5.64E-122	4.69E-20
cg06135285	17	19675785	*ULK2*	0.635	0.702	0.684	0.067	0.049	4.67E-41	4.92E-12
cg12156887	17	19883716	NA	0.355	0.294	0.331	-0.061	-0.024	3.33E-67	7.61E-07
cg02656560	17	19967600	*SPECC1*	0.300	0.249	0.272	-0.051	-0.028	1.13E-63	1.53E-09
cg07435331	17	21178476	NA	0.526	0.474	0.481	-0.051	-0.045	8.47E-33	7.98E-12
cg02980566	17	21411890	NA	0.320	0.263	0.291	-0.058	-0.030	1.04E-65	3.12E-10
cg15922057	17	25784714	NA	0.316	0.259	0.286	-0.057	-0.029	6.38E-54	1.79E-07
cg10334489	17	25798878	*KSR1*	0.647	0.716	0.666	0.069	0.020	2.43E-146	8.28E-09
cg24476449	17	25799212	*KSR1*	0.680	0.740	0.701	0.060	0.022	8.91E-88	2.80E-07
cg20673721	17	25859382	*KSR1*	0.524	0.418	0.476	-0.107	-0.048	3.18E-105	7.48E-14
cg12249234	17	25867613	*KSR1*	0.428	0.320	0.386	-0.108	-0.043	1.16E-119	1.45E-11
cg11333454	17	26609296	*KRT18P55*	0.733	0.769	0.783	0.036	0.050	5.96E-15	2.05E-12
cg27177006	17	26635378	NA	0.693	0.723	0.744	0.030	0.050	3.41E-10	1.90E-12
cg25356504	17	26707476	*SARM1*	0.348	0.268	0.318	-0.079	-0.029	2.99E-123	2.72E-11
cg08032476	17	27045176	*RAB34*	0.354	0.288	0.331	-0.066	-0.023	7.78E-108	9.84E-09
cg17742559	17	27068495	*NEK8*	0.365	0.298	0.339	-0.067	-0.026	7.62E-112	2.53E-11
cg22006208	17	27309169	*SEZ6*	0.460	0.390	0.429	-0.070	-0.031	1.13E-85	1.26E-09
cg21210041	17	27443831	*MYO18A*	0.506	0.433	0.483	-0.073	-0.023	4.07E-103	2.19E-07
cg14554468	17	28696496	NA	0.380	0.279	0.339	-0.101	-0.041	1.71E-124	1.80E-14
cg03419051	17	29814382	*RAB11FIP4*	0.557	0.505	0.525	-0.052	-0.032	2.34E-53	1.53E-10
cg22798362	17	30850485	*MYO1D*	0.782	0.843	0.816	0.061	0.034	1.67E-74	7.92E-13
cg01636591	17	32646156	*CCL8*	0.633	0.580	0.595	-0.053	-0.038	2.72E-62	8.94E-19
cg08154963	17	33426885	*RAD51L3-RFI*	0.478	0.370	0.442	-0.107	-0.035	2.06E-179	9.00E-14
cg13451886	17	33568791	*SLFN5*	0.751	0.810	0.781	0.059	0.031	1.61E-76	1.15E-10
cg04862556	17	33775933	*SLFN13*	0.365	0.298	0.340	-0.067	-0.025	1.90E-96	3.37E-09
cg16563370	17	33775952	*SLFN13*	0.408	0.327	0.372	-0.080	-0.036	8.45E-129	9.51E-18
cg13443575	17	33775961	*SLFN13*	0.345	0.276	0.321	-0.070	-0.025	4.07E-96	2.57E-09
cg02380585	17	33776683	*SLFN13*	0.345	0.262	0.306	-0.082	-0.039	1.80E-92	2.30E-11
cg03677056	17	33817217	NA	0.410	0.336	0.380	-0.073	-0.030	8.89E-91	3.22E-10
cg03182688	17	33825300	NA	0.358	0.284	0.327	-0.075	-0.032	1.93E-91	3.85E-11
cg24895173	17	33825374	NA	0.402	0.324	0.371	-0.078	-0.031	1.58E-75	3.87E-07
cg21705506	17	33842181	NA	0.515	0.547	0.567	0.032	0.052	8.42E-08	9.75E-09
cg17217920	17	33957395	*AP2B1*	0.701	0.752	0.750	0.051	0.048	6.86E-19	1.72E-08
cg15939920	17	35457385	*ACACA*	0.416	0.352	0.388	-0.064	-0.027	1.80E-86	1.41E-10
cg07660627	17	35481970	*ACACA*	0.681	0.743	0.727	0.062	0.046	6.94E-32	2.62E-09
cg07375836	17	35717813	*ACACA*	0.358	0.254	0.306	-0.104	-0.052	1.92E-132	2.53E-19
cg12582728	17	36162433	NA	0.725	0.780	0.756	0.056	0.032	6.30E-40	1.32E-06
cg13536447	17	36188913	NA	0.442	0.322	0.401	-0.121	-0.042	1.08E-140	1.21E-11
cg12361262	17	36571637	NA	0.339	0.259	0.300	-0.080	-0.039	3.47E-71	1.09E-09
cg14624145	17	37024169	NA	0.603	0.533	0.557	-0.070	-0.046	3.15E-25	4.33E-06
cg00760203	17	37254921	*PLXDC1*	0.296	0.223	0.274	-0.072	-0.022	3.45E-87	3.27E-06
cg13190608	17	37322730	*ARL5C*	0.556	0.506	0.526	-0.050	-0.031	3.41E-31	6.96E-07
cg14377681	17	37875709	*ERBB2*	0.420	0.338	0.380	-0.082	-0.041	5.90E-122	2.35E-18
cg13761998	17	37894403	*GRB7*	0.362	0.290	0.339	-0.072	-0.022	3.13E-96	1.90E-07
cg17740645	17	37894413	*GRB7*	0.349	0.275	0.323	-0.073	-0.025	3.71E-120	2.70E-12
cg07327299	17	37896063	*GRB7*	0.434	0.366	0.410	-0.068	-0.024	5.88E-70	1.15E-06
cg25038082	17	37896074	*GRB7*	0.462	0.409	0.441	-0.053	-0.022	6.58E-45	1.36E-06
cg08441850	17	37956902	*IKZF3*	0.588	0.655	0.626	0.066	0.038	1.68E-84	2.67E-15
cg12655416	17	38077870	*ORMDL3*	0.225	0.175	0.207	-0.051	-0.018	6.54E-89	4.55E-07
cg15412772	17	38175389	*MED24*	0.555	0.468	0.528	-0.087	-0.027	1.02E-163	2.59E-13
cg11804928	17	38220298	*THRA*	0.324	0.256	0.296	-0.069	-0.029	1.25E-77	2.41E-08
cg11826961	17	38221639	*THRA*	0.578	0.525	0.543	-0.053	-0.035	6.02E-60	1.64E-15
cg26854588	17	38440015	NA	0.759	0.811	0.782	0.052	0.023	1.36E-68	7.90E-08
cg05824218	17	38499096	*RARA*	0.594	0.526	0.565	-0.068	-0.029	9.30E-89	3.95E-11

cg07944862	17	38501174	*RARA*	0.608	0.543	0.573	-0.065	-0.035	6.92E-62	1.05E-09
cg17980404	17	38601676	*IGFBP4*	0.363	0.282	0.314	-0.081	-0.050	2.55E-115	1.22E-24
cg15379400	17	38655492	*TNS4*	0.404	0.352	0.380	-0.052	-0.024	5.06E-64	4.12E-08
cg25248278	17	38696507	NA	0.658	0.729	0.687	0.071	0.029	1.47E-115	7.93E-14
cg03917473	17	38764244	NA	0.680	0.747	0.707	0.068	0.027	1.72E-86	5.36E-09
cg07008343	17	38956362	*KRT28*	0.660	0.715	0.709	0.055	0.049	1.64E-23	1.36E-09
cg06298244	17	39086292	*KRT23*	0.727	0.777	0.783	0.050	0.056	2.75E-18	7.19E-11
cg04940329	17	39093054	*KRT23*	0.406	0.309	0.351	-0.096	-0.054	2.14E-92	3.26E-16
cg02092790	17	39094800	*KRT23*	0.724	0.774	0.761	0.050	0.037	4.94E-28	3.18E-07
cg08058191	17	39781130	*KRT17*	0.302	0.240	0.275	-0.062	-0.027	2.26E-64	8.96E-07
cg26535158	17	40175841	*NKIRAS2*	0.389	0.315	0.371	-0.074	-0.018	6.42E-119	1.01E-06
cg00435173	17	40284046	*RAB5C*	0.494	0.405	0.449	-0.090	-0.045	1.49E-103	1.07E-17
cg13053608	17	40345673	*GHDC*	0.379	0.291	0.338	-0.088	-0.041	2.73E-151	3.21E-23
cg02832357	17	40429406	*STAT5B*	0.256	0.192	0.209	-0.065	-0.047	8.97E-86	3.57E-21
cg05117208	17	40438312	*STAT5A*	0.417	0.351	0.386	-0.066	-0.031	4.06E-63	2.74E-08
cg05744184	17	40463806	*STAT5A*	0.345	0.274	0.323	-0.071	-0.022	2.86E-129	5.89E-11
cg25330422	17	40467382	*STAT3*	0.665	0.723	0.704	0.058	0.039	1.47E-56	1.47E-13
cg05487134	17	40489569	*STAT3*	0.574	0.649	0.616	0.075	0.042	4.89E-45	7.85E-09
cg24312520	17	40489584	*STAT3*	0.518	0.593	0.556	0.075	0.038	7.61E-53	5.85E-08
cg27196880	17	40570233	*PTRF*	0.401	0.305	0.354	-0.096	-0.047	1.59E-142	7.16E-21
cg06968912	17	40573643	*PTRF*	0.220	0.163	0.188	-0.057	-0.032	8.66E-60	2.93E-09
cg08857797	17	40927699	*VPS25*	0.599	0.534	0.573	-0.064	-0.026	7.61E-62	2.12E-06
cg19317715	17	40996578	*AOC2*	0.398	0.337	0.363	-0.060	-0.035	5.85E-50	7.06E-08
cg22396850	17	41121153	*PTGES3L-AAI*	0.349	0.270	0.317	-0.079	-0.032	7.17E-138	1.03E-14
cg25070639	17	41170693	*VAT1*	0.346	0.263	0.318	-0.082	-0.028	2.06E-120	7.48E-10
cg10499832	17	41450195	*LOC1001305*	0.432	0.357	0.407	-0.074	-0.024	1.21E-87	4.23E-06
cg06484100	17	41799087	NA	0.504	0.442	0.480	-0.062	-0.025	2.26E-65	2.06E-06
cg14039779	17	41857714	*DUSP3*	0.507	0.421	0.473	-0.087	-0.034	5.10E-98	1.79E-10
cg26534477	17	42147600	*G6PC3*	0.313	0.238	0.285	-0.075	-0.028	1.07E-121	2.46E-13
cg09014329	17	42431748	*FAM171A2*	0.421	0.464	0.490	0.043	0.069	4.59E-14	5.92E-17
cg23766254	17	42431859	*FAM171A2*	0.328	0.365	0.383	0.036	0.055	9.68E-10	3.65E-10
cg22078805	17	42432046	*FAM171A2*	0.366	0.411	0.444	0.045	0.077	6.26E-10	9.87E-14
cg07895381	17	42541896	*GPATCH8*	0.636	0.688	0.685	0.053	0.049	2.56E-17	7.08E-08
cg08351131	17	43242591	*HEXIM2*	0.401	0.337	0.365	-0.064	-0.036	1.26E-92	2.76E-21
cg19601328	17	43368729	*MAP3K14*	0.334	0.259	0.303	-0.075	-0.031	3.33E-93	2.28E-09
cg16826777	17	43376882	*MAP3K14*	0.451	0.395	0.419	-0.056	-0.033	1.33E-89	3.83E-16
cg03244997	17	43503144	*ARHGAP27*	0.415	0.358	0.389	-0.057	-0.026	3.65E-94	1.55E-11
cg16281322	17	43510478	*ARHGAP27*	0.458	0.384	0.433	-0.074	-0.026	2.25E-79	1.59E-06
cg04487659	17	43510575	*ARHGAP27*	0.352	0.278	0.310	-0.074	-0.041	1.15E-150	8.00E-36
cg05533539	17	44104521	*MAPT*	0.406	0.317	0.378	-0.089	-0.027	7.41E-128	9.97E-08
cg18699337	17	44159144	*KANSL1*	0.723	0.771	0.781	0.048	0.058	9.36E-21	1.87E-13
cg27050612	17	46133198	*NFE2L1*	0.312	0.255	0.285	-0.058	-0.027	7.49E-106	3.27E-16
cg01521220	17	46233799	*SKAP1*	0.383	0.314	0.351	-0.069	-0.032	2.08E-41	9.10E-07
cg11210138	17	46233945	*SKAP1*	0.511	0.447	0.476	-0.064	-0.035	2.93E-48	9.73E-10
cg21901395	17	46604235	NA	0.538	0.483	0.488	-0.056	-0.051	4.93E-42	2.85E-16
cg26841048	17	46622454	*HOXB2*	0.600	0.527	0.553	-0.072	-0.047	4.13E-65	5.06E-14
cg22777724	17	46622516	*HOXB2*	0.449	0.378	0.421	-0.071	-0.028	4.73E-70	5.12E-07
cg23530553	17	46622536	*HOXB2*	0.648	0.575	0.602	-0.074	-0.047	3.63E-89	3.47E-19
cg06942183	17	46622607	*HOXB2*	0.362	0.310	0.325	-0.052	-0.037	4.66E-47	1.09E-10
cg19104015	17	46646043	*HOXB3*	0.293	0.228	0.265	-0.065	-0.028	2.09E-65	8.60E-07
cg04014328	17	46653615	*HOXB4*	0.659	0.603	0.631	-0.056	-0.028	1.79E-37	1.39E-06
cg11971423	17	46653711	*HOXB4*	0.753	0.701	0.716	-0.052	-0.036	3.60E-44	1.08E-12
cg26072749	17	46657274	*MIR10A*	0.322	0.271	0.290	-0.051	-0.031	6.49E-28	5.83E-06
cg15649236	17	46657504	NA	0.388	0.302	0.356	-0.086	-0.032	6.25E-69	8.07E-07
cg01572694	17	46657555	NA	0.561	0.465	0.505	-0.096	-0.056	7.40E-79	3.81E-16
cg04514255	17	46658170	NA	0.515	0.449	0.467	-0.066	-0.048	2.89E-65	5.91E-20
cg14285150	17	46659019	NA	0.397	0.341	0.372	-0.056	-0.025	8.60E-48	5.24E-07
cg02311193	17	46660002	NA	0.470	0.381	0.432	-0.089	-0.038	1.29E-102	7.11E-11
cg21242356	17	46667683	NA	0.390	0.310	0.356	-0.080	-0.034	4.53E-100	2.23E-13
cg13994241	17	46667725	NA	0.501	0.407	0.455	-0.094	-0.046	1.64E-103	2.05E-18
cg01351315	17	46667737	NA	0.485	0.377	0.445	-0.108	-0.040	5.71E-111	9.12E-12
cg25960038	17	46667789	NA	0.386	0.293	0.342	-0.093	-0.043	1.92E-115	3.64E-17
cg09595185	17	46667812	NA	0.456	0.357	0.412	-0.098	-0.044	5.29E-98	1.37E-12
cg00711072	17	46669489	*HOXB-AS3*	0.345	0.254	0.315	-0.091	-0.030	1.49E-87	5.59E-06
cg01405107	17	46671635	*HOXB-AS3*	0.541	0.490	0.496	-0.051	-0.045	1.68E-15	1.23E-06
cg07676709	17	46673442	*HOXB-AS3*	0.588	0.536	0.551	-0.052	-0.037	1.19E-26	2.53E-07
cg15908709	17	46676215	*HOXB-AS3*	0.431	0.379	0.385	-0.052	-0.045	4.56E-19	1.11E-08
cg08832695	17	46676375	*HOXB-AS3*	0.573	0.521	0.534	-0.052	-0.039	7.84E-26	1.36E-08
cg01833923	17	47286719	*GNGT2*	0.542	0.603	0.572	0.061	0.030	2.15E-126	3.15E-20
cg17839611	17	47286802	*GNGT2*	0.611	0.686	0.647	0.075	0.036	1.86E-122	4.25E-19
cg25451120	17	47287444	*GNGT2*	0.650	0.704	0.667	0.054	0.017	1.36E-74	3.16E-06
cg16378063	17	47329552	*FLJ40194*	0.482	0.379	0.446	-0.103	-0.036	3.96E-114	7.86E-10
cg27059140	17	47355215	NA	0.645	0.580	0.613	-0.066	-0.032	9.13E-60	1.92E-09
cg26664457	17	47394423	*ZNF652*	0.418	0.329	0.376	-0.089	-0.043	1.64E-113	3.60E-15
cg25649895	17	48356195	*TMEM92*	0.564	0.511	0.532	-0.053	-0.032	7.09E-49	6.60E-11
cg03919657	17	48502312	*ACSF2*	0.425	0.361	0.399	-0.064	-0.026	2.05E-112	8.03E-16
cg18038894	17	48506385	*ACSF2*	0.536	0.420	0.490	-0.116	-0.046	2.42E-191	3.70E-23
cg15046489	17	48857353	NA	0.769	0.827	0.794	0.058	0.025	7.35E-114	4.09E-13

cg19213808	17	49336082	*MBTD1*	0.655	0.706	0.703	0.052	0.048	3.36E-26	4.25E-10
cg04071967	17	53511321	NA	0.411	0.314	0.371	-0.097	-0.040	1.77E-180	3.83E-21
cg07493197	17	53573969	NA	0.352	0.263	0.299	-0.088	-0.052	5.38E-92	1.89E-19
cg27657459	17	54853771	NA	0.655	0.711	0.707	0.056	0.053	3.91E-23	4.99E-10
cg14272075	17	55122538	NA	0.501	0.390	0.462	-0.111	-0.040	3.86E-122	1.05E-11
cg06078469	17	55335955	*MSI2*	0.295	0.241	0.268	-0.054	-0.027	3.79E-90	4.28E-14
cg08562099	17	55389809	*MSI2*	0.442	0.365	0.400	-0.077	-0.042	1.53E-96	3.58E-16
cg04176246	17	55434640	*MSI2*	0.360	0.290	0.333	-0.070	-0.027	8.07E-93	1.80E-08
cg12441066	17	55456535	*MSI2*	0.382	0.328	0.357	-0.054	-0.026	1.55E-68	1.29E-09
cg06966839	17	55685941	*MSI2*	0.438	0.371	0.418	-0.067	-0.020	1.33E-101	2.15E-06
cg23924737	17	55928753	*MRPS23*	0.527	0.431	0.489	-0.096	-0.039	6.86E-120	1.89E-13
cg09628499	17	56299729	NA	0.698	0.747	0.761	0.048	0.063	2.38E-21	1.06E-16
cg24489015	17	56316162	*LPO*	0.362	0.280	0.329	-0.083	-0.033	9.19E-85	1.20E-07
cg04266202	17	56352895	*MPO*	0.524	0.418	0.485	-0.106	-0.039	2.35E-133	2.20E-12
cg11151395	17	56355299	*MPO*	0.552	0.442	0.518	-0.110	-0.034	8.78E-136	5.01E-10
cg22331200	17	56355362	*MPO*	0.569	0.474	0.533	-0.095	-0.036	5.73E-110	4.88E-13
cg02138358	17	56358318	*MPO*	0.400	0.324	0.371	-0.075	-0.029	2.76E-160	4.57E-17
cg04988978	17	56359578	*MPO*	0.236	0.182	0.210	-0.054	-0.026	4.03E-67	6.21E-08
cg23632656	17	56362296	NA	0.229	0.176	0.206	-0.053	-0.022	7.17E-73	6.32E-07
cg10575089	17	56606542	*SEPT4*	0.702	0.764	0.721	0.062	0.018	3.41E-103	6.39E-07
cg18677148	17	57712280	*CLTC*	0.652	0.719	0.702	0.068	0.050	4.13E-43	2.41E-12
cg12494208	17	57743594	*CLTC*	0.773	0.822	0.826	0.048	0.053	1.87E-22	1.19E-12
cg14032089	17	57917589	*VMP1*	0.768	0.827	0.798	0.059	0.030	9.69E-61	3.34E-08
cg06471491	17	57917974	NA	0.546	0.612	0.585	0.067	0.039	1.38E-46	9.00E-10
cg01248878	17	58155376	*HEATR6*	0.238	0.180	0.211	-0.058	-0.027	5.66E-85	1.05E-09
cg00691729	17	58468300	*USP32*	0.467	0.387	0.429	-0.080	-0.038	2.13E-63	2.41E-08
cg13984330	17	58637589	NA	0.583	0.505	0.553	-0.077	-0.029	4.57E-80	1.38E-07
cg08477158	17	58807667	*BCAS3*	0.360	0.302	0.333	-0.058	-0.026	8.42E-54	4.59E-07
cg26627687	17	58823592	*BCAS3*	0.736	0.780	0.787	0.044	0.050	2.17E-16	1.05E-10
cg19457909	17	59206070	*BCAS3*	0.649	0.588	0.627	-0.061	-0.022	3.80E-69	1.46E-06
cg15723222	17	59222476	*BCAS3*	0.360	0.292	0.338	-0.067	-0.022	5.51E-93	4.92E-08
cg15285436	17	59226714	*BCAS3*	0.471	0.370	0.435	-0.102	-0.037	5.39E-88	5.24E-09
cg02275530	17	59328313	*BCAS3*	0.469	0.380	0.428	-0.089	-0.041	5.25E-104	1.96E-12
cg10588135	17	59329903	*BCAS3*	0.311	0.244	0.277	-0.068	-0.034	1.98E-101	2.57E-14
cg11628739	17	60731254	*MRC2*	0.284	0.231	0.256	-0.052	-0.028	1.72E-120	1.57E-19
cg21380024	17	61042803	*MIR548W*	0.483	0.551	0.526	0.068	0.044	1.91E-65	2.53E-14
cg23527902	17	61515708	*CYB561*	0.356	0.287	0.328	-0.068	-0.027	6.06E-100	1.23E-10
cg07695566	17	61525112	*CYB561*	0.484	0.419	0.457	-0.065	-0.027	5.87E-105	1.90E-13
cg19982668	17	61753497	*MAP3K3*	0.378	0.304	0.340	-0.074	-0.038	6.83E-111	1.26E-16
cg09121543	17	61774794	*LIMD2*	0.405	0.516	0.440	0.111	0.035	4.58E-125	7.44E-10
cg20157577	17	61780203	*STRADA*	0.679	0.744	0.711	0.065	0.032	1.36E-123	7.37E-18
cg10275770	17	62084205	*ICAM2*	0.338	0.281	0.312	-0.057	-0.026	2.82E-66	1.59E-08
cg12793803	17	62084217	*ICAM2*	0.327	0.269	0.296	-0.058	-0.031	3.80E-99	1.10E-16
cg21519701	17	62252524	*TEX2*	0.435	0.312	0.376	-0.123	-0.059	1.42E-155	9.75E-23
cg17837191	17	62318673	*TEX2*	0.497	0.379	0.448	-0.118	-0.049	7.47E-170	3.20E-20
cg20830994	17	62401400	*PECAM1*	0.633	0.698	0.654	0.065	0.021	6.59E-116	3.19E-09
cg24361586	17	62744534	NA	0.556	0.465	0.523	-0.091	-0.033	5.46E-132	9.56E-13
cg15798221	17	62773704	*LOC146880*	0.263	0.208	0.244	-0.055	-0.019	1.40E-97	1.01E-07
cg05467716	17	62774079	*LOC146880*	0.402	0.312	0.356	-0.090	-0.046	9.76E-165	2.34E-29
cg25554496	17	63036095	*GNA13*	0.776	0.830	0.799	0.054	0.023	1.16E-85	4.55E-09
cg00954105	17	63739259	*CEP112*	0.757	0.811	0.792	0.054	0.035	1.31E-51	1.24E-10
cg14648237	17	64422393	*PRKCA*	0.361	0.285	0.328	-0.077	-0.033	3.45E-119	3.86E-14
cg15015109	17	65136480	*HELZ*	0.467	0.363	0.427	-0.103	-0.039	1.14E-174	1.92E-18
cg15797314	17	65437724	*PITPNC1*	0.751	0.806	0.774	0.055	0.023	9.11E-75	4.35E-07
cg26049998	17	65602730	*PITPNC1*	0.377	0.304	0.344	-0.073	-0.034	7.06E-101	3.47E-13
cg25456728	17	66255048	*ARSG*	0.673	0.734	0.717	0.061	0.044	6.38E-34	7.58E-10
cg04938381	17	66374702	*ARSG*	0.431	0.381	0.408	-0.050	-0.023	4.27E-51	1.09E-06
cg27366162	17	66375195	*ARSG*	0.470	0.413	0.447	-0.056	-0.022	1.81E-64	2.93E-07
cg25161129	17	66452343	*WIPI1*	0.415	0.338	0.381	-0.077	-0.034	3.76E-61	1.07E-06
cg20155875	17	66452567	*WIPI1*	0.578	0.513	0.546	-0.065	-0.032	1.98E-62	2.07E-08
cg21727223	17	66755770	NA	0.419	0.347	0.381	-0.072	-0.038	6.86E-75	1.38E-11
cg20561863	17	66905630	*ABCA8*	0.231	0.179	0.198	-0.052	-0.033	6.82E-69	3.18E-14
cg12323063	17	67497879	*MAP2K6*	0.548	0.442	0.513	-0.106	-0.035	1.53E-137	1.75E-10
cg23398508	17	70536199	*LINC00673*	0.276	0.221	0.250	-0.055	-0.026	3.04E-50	4.00E-06
cg07834069	17	72524918	*CD300LB*	0.418	0.316	0.372	-0.102	-0.045	4.25E-112	5.50E-15
cg14075731	17	72527607	*CD300LB*	0.253	0.201	0.222	-0.053	-0.031	1.23E-66	2.39E-13
cg03469804	17	72732432	*RAB37*	0.517	0.443	0.469	-0.074	-0.048	1.02E-71	1.89E-16
cg14196186	17	72732698	*RAB37*	0.268	0.207	0.241	-0.062	-0.028	9.28E-79	2.19E-09
cg10755085	17	72732722	*RAB37*	0.274	0.220	0.250	-0.054	-0.024	1.16E-68	6.50E-08
cg06289802	17	72748189	*SLC9A3R1*	0.381	0.309	0.352	-0.072	-0.029	1.29E-93	4.27E-10
cg22247041	17	73004677	NA	0.614	0.665	0.641	0.051	0.026	1.59E-49	1.91E-07
cg04686354	17	73261880	*MRPS7*	0.507	0.413	0.471	-0.093	-0.035	1.12E-174	1.31E-22
cg23599820	17	73456199	*KIAA0195*	0.387	0.333	0.360	-0.054	-0.026	2.47E-89	2.39E-12
cg14691671	17	73642503	*RECQL5*	0.404	0.337	0.370	-0.067	-0.034	2.48E-68	1.55E-09
cg14715778	17	73840989	*UNC13D*	0.233	0.180	0.204	-0.053	-0.029	3.02E-88	2.83E-16
cg11061343	17	73993843	*TEN1-CDK3*	0.303	0.234	0.275	-0.069	-0.028	6.58E-144	2.70E-15
cg10138630	17	74024966	*EVPL*	0.357	0.304	0.332	-0.053	-0.025	1.22E-84	1.08E-11
cg16672637	17	74138356	*FOXJ1*	0.566	0.506	0.530	-0.061	-0.036	8.73E-72	2.63E-14

cg24809269	17	74260692	*FAM100B*	0.711	0.778	0.735	0.068	0.024	3.60E-98	5.23E-08
cg06245711	17	74260776	*FAM100B*	0.521	0.599	0.571	0.078	0.050	1.58E-49	1.92E-11
cg08837215	17	74443080	*UBE2O*	0.556	0.462	0.521	-0.094	-0.035	2.65E-112	5.19E-09
cg13533061	17	74712429	*JMJD6*	0.329	0.254	0.288	-0.075	-0.042	1.74E-104	2.47E-18
cg25699034	17	74721824	*METTL23*	0.339	0.268	0.301	-0.071	-0.038	5.08E-62	8.33E-10
cg05729480	17	75276428	*SEPT9*	0.456	0.382	0.429	-0.074	-0.027	9.46E-96	3.88E-08
cg03330678	17	75316233	*SEPT9*	0.407	0.322	0.381	-0.086	-0.027	8.26E-136	6.03E-11
cg08772789	17	75318655	*SEPT9*	0.448	0.350	0.402	-0.098	-0.045	1.37E-135	3.28E-17
cg00030296	17	75318732	*SEPT9*	0.416	0.335	0.377	-0.081	-0.038	2.02E-143	1.22E-18
cg07324245	17	75445905	*SEPT9*	0.726	0.788	0.762	0.062	0.036	3.24E-128	3.73E-23
cg20772590	17	75446431	*SEPT9*	0.699	0.770	0.731	0.070	0.031	8.56E-117	1.60E-14
cg14885762	17	75446450	*SEPT9*	0.692	0.768	0.717	0.076	0.025	3.76E-120	1.48E-08
cg21204860	17	75446565	*SEPT9*	0.560	0.627	0.580	0.067	0.020	1.48E-115	1.03E-07
cg19654743	17	75446592	*SEPT9*	0.689	0.762	0.719	0.073	0.030	1.07E-126	2.75E-16
cg06513247	17	75446661	*SEPT9*	0.544	0.612	0.565	0.069	0.021	3.38E-102	2.88E-08
cg17922695	17	75451809	*SEPT9*	0.627	0.683	0.651	0.056	0.024	2.57E-69	3.65E-08
cg27627381	17	75452100	*SEPT9*	0.715	0.771	0.736	0.056	0.021	1.17E-97	7.06E-09
cg04837616	17	75880542	NA	0.555	0.498	0.523	-0.057	-0.031	8.20E-65	5.65E-11
cg05637296	17	76129475	*TMC8*	0.710	0.765	0.737	0.056	0.027	1.48E-84	6.72E-11
cg26003388	17	76129533	*TMC8*	0.816	0.866	0.838	0.050	0.022	1.03E-76	2.81E-08
cg14210726	17	76136952	*TMC8*	0.429	0.356	0.409	-0.073	-0.020	7.16E-121	1.10E-07
cg20740903	17	76170799	*TK1*	0.493	0.398	0.461	-0.094	-0.032	9.52E-110	1.93E-10
cg00945209	17	76801579	*USP36*	0.609	0.682	0.627	0.073	0.018	1.58E-106	6.27E-06
cg01698392	17	76871734	*TIMP2*	0.458	0.404	0.424	-0.054	-0.034	1.59E-37	1.68E-09
cg14870271	17	76976010	*LGALS3BP*	0.405	0.334	0.349	-0.072	-0.056	1.01E-40	5.69E-17
cg25178683	17	76976267	*LGALS3BP*	0.657	0.581	0.594	-0.076	-0.063	4.45E-37	1.15E-17
cg14421700	17	77755965	*CBX2*	0.353	0.284	0.327	-0.069	-0.026	7.59E-118	1.10E-13
cg01870907	17	77775687	NA	0.242	0.182	0.218	-0.060	-0.023	1.14E-120	1.02E-11
cg09351315	17	77775823	NA	0.337	0.276	0.308	-0.062	-0.029	1.79E-89	1.13E-12
cg00483030	17	77810509	*CBX4*	0.401	0.305	0.358	-0.096	-0.043	1.22E-176	1.45E-26
cg00973876	17	77899208	NA	0.522	0.470	0.480	-0.051	-0.041	5.62E-21	5.90E-08
cg04157865	17	77914162	*TBC1D16*	0.536	0.465	0.512	-0.071	-0.025	6.37E-65	4.38E-06
cg20443278	17	77962098	*TBC1D16*	0.623	0.673	0.644	0.050	0.021	6.09E-92	1.35E-10
cg03837680	17	77967529	*TBC1D16*	0.335	0.251	0.306	-0.084	-0.029	7.82E-138	3.93E-12
cg18758433	17	78623601	*RPTOR*	0.682	0.753	0.724	0.071	0.042	8.51E-88	5.95E-18
cg22386583	17	78753756	*RPTOR*	0.581	0.506	0.548	-0.075	-0.033	5.05E-65	1.13E-07
cg16115689	17	78764256	*RPTOR*	0.466	0.531	0.486	0.065	0.020	9.09E-99	9.82E-07
cg26360197	17	78821604	*RPTOR*	0.681	0.742	0.709	0.061	0.028	3.93E-68	5.69E-09
cg16541275	17	78821754	*RPTOR*	0.568	0.642	0.604	0.074	0.036	1.98E-109	1.79E-17
cg02251850	17	78851503	*RPTOR*	0.719	0.793	0.742	0.074	0.024	6.33E-102	1.55E-06
cg26783127	17	79128918	*AATK*	0.480	0.353	0.437	-0.128	-0.043	1.01E-132	3.41E-12
cg16732654	17	79129022	*AATK*	0.454	0.351	0.405	-0.104	-0.049	1.34E-105	5.52E-14
cg23334433	17	79129051	*AATK*	0.501	0.377	0.463	-0.124	-0.038	6.40E-112	5.33E-08
cg13618516	17	79129078	*AATK*	0.528	0.411	0.476	-0.118	-0.052	1.86E-144	8.22E-18
cg26729913	17	79135071	*AATK*	0.423	0.355	0.396	-0.068	-0.026	4.89E-79	2.74E-08
cg03823431	17	79229385	*SLC38A10*	0.387	0.328	0.371	-0.059	-0.016	9.23E-103	1.31E-06
cg05919238	17	79244158	*SLC38A10*	0.669	0.546	0.623	-0.123	-0.046	1.83E-157	6.39E-18
cg01905773	17	79297618	*TMEM105*	0.489	0.412	0.459	-0.077	-0.030	1.12E-88	1.86E-09
cg25616968	17	79416203	*BAHCC1*	0.461	0.391	0.428	-0.071	-0.033	2.74E-51	3.64E-08
cg17102495	17	79421644	*BAHCC1*	0.424	0.353	0.403	-0.071	-0.021	1.03E-119	1.13E-07
cg02500691	17	79476186	NA	0.646	0.700	0.671	0.054	0.025	6.63E-53	9.03E-08
cg05271255	17	79821342	NA	0.249	0.188	0.221	-0.061	-0.028	2.10E-75	2.96E-09
cg19573490	17	79870317	*PCYT2*	0.443	0.333	0.398	-0.111	-0.046	4.48E-159	2.68E-17
cg08574915	17	79924772	NA	0.331	0.255	0.303	-0.076	-0.027	1.11E-94	1.85E-08
cg10505658	17	80084571	*CCDC57*	0.631	0.707	0.663	0.076	0.031	7.18E-100	2.99E-11
cg21577598	17	80084751	*CCDC57*	0.683	0.754	0.712	0.071	0.029	4.95E-71	8.22E-08
cg21773646	17	80085082	*CCDC57*	0.592	0.665	0.622	0.072	0.030	1.12E-115	2.86E-13
cg17846016	17	80181015	NA	0.495	0.375	0.453	-0.120	-0.042	2.89E-138	5.24E-12
cg27355141	17	80190136	*SLC16A3*	0.316	0.261	0.289	-0.055	-0.027	6.13E-87	4.38E-14
cg03956820	17	80190154	*SLC16A3*	0.434	0.362	0.401	-0.072	-0.033	2.82E-89	1.69E-16
cg15296856	17	80199608	NA	0.318	0.263	0.291	-0.056	-0.027	5.73E-118	1.39E-17
cg05604874	17	80200785	*CSNK1D*	0.448	0.353	0.414	-0.094	-0.033	3.18E-77	3.91E-06
cg00660167	17	80200974	*CSNK1D*	0.502	0.387	0.468	-0.115	-0.034	9.80E-110	1.20E-06
cg24328095	17	80205093	*CSNK1D*	0.281	0.229	0.256	-0.052	-0.025	1.65E-62	1.41E-07
cg05385188	17	80215375	*CSNK1D*	0.664	0.722	0.707	0.058	0.043	3.11E-29	4.41E-08
cg19914554	17	80275401	*CD7*	0.611	0.663	0.632	0.052	0.021	1.17E-64	1.00E-07
cg20186396	17	80275498	*CD7*	0.629	0.688	0.654	0.059	0.025	5.79E-110	1.06E-13
cg25662857	17	80275542	*CD7*	0.619	0.686	0.645	0.066	0.025	4.36E-95	2.68E-09
cg09455342	17	80346849	NA	0.549	0.465	0.518	-0.084	-0.031	2.00E-75	4.32E-07
cg00231966	17	80377299	*C17orf101*	0.321	0.267	0.275	-0.053	-0.046	8.41E-43	5.83E-16
cg13984746	17	80398412	*HEXDC*	0.412	0.324	0.372	-0.088	-0.040	1.40E-99	7.90E-13
cg11690666	17	80415469	*NARF*	0.582	0.475	0.540	-0.107	-0.041	7.51E-167	1.74E-20
cg14173033	17	80545310	*FOXK2*	0.414	0.342	0.376	-0.072	-0.038	1.53E-38	4.51E-06
cg13424393	17	80569923	NA	0.506	0.403	0.473	-0.103	-0.033	5.03E-109	1.77E-08
cg25197500	17	80581805	*WDR45L*	0.624	0.495	0.584	-0.129	-0.040	4.32E-115	9.42E-10
cg22516137	17	80657589	*RAB40B*	0.681	0.722	0.731	0.041	0.050	1.48E-12	2.39E-08
cg17852482	17	80833054	*TBCD*	0.475	0.373	0.442	-0.102	-0.033	1.19E-173	1.29E-12
cg03778909	17	80833393	*TBCD*	0.447	0.348	0.406	-0.099	-0.040	1.53E-153	6.92E-18

cg07409471	17	80833638	*TBCD*	0.589	0.483	0.549	-0.106	-0.039	1.56E-111	2.06E-11
cg13304638	17	80834089	*TBCD*	0.565	0.432	0.511	-0.133	-0.054	4.76E-133	5.37E-16
cg06218079	17	80834228	*TBCD*	0.543	0.433	0.507	-0.110	-0.036	1.02E-75	1.52E-06
cg08977209	17	80867256	*TBCD*	0.623	0.674	0.688	0.050	0.065	8.30E-17	2.78E-12
cg24914483	18	2653997	*CBX3P2*	0.443	0.338	0.412	-0.105	-0.030	5.96E-120	5.36E-07
cg26708427	18	3250274	*MYL12A*	0.769	0.820	0.796	0.051	0.027	8.50E-60	2.28E-09
cg00533990	18	3255878	*MYL12A*	0.432	0.357	0.405	-0.075	-0.027	6.89E-88	5.05E-07
cg16954236	18	8638786	*RAB12*	0.731	0.785	0.784	0.054	0.052	2.13E-24	5.57E-11
cg21923525	18	9474143	*RALBP1*	0.353	0.279	0.324	-0.074	-0.029	6.41E-151	1.02E-15
cg13049483	18	9712461	*RAB31*	0.461	0.398	0.426	-0.063	-0.035	1.15E-55	1.78E-09
cg26909797	18	9911079	NA	0.749	0.801	0.789	0.052	0.039	2.88E-31	3.64E-09
cg20670923	18	9915771	*VAPA*	0.247	0.190	0.226	-0.057	-0.020	4.50E-84	3.29E-07
cg03148503	18	10588980	NA	0.730	0.781	0.776	0.050	0.045	2.14E-22	1.38E-08
cg16498391	18	12421072	*SLMO1*	0.368	0.295	0.343	-0.072	-0.025	4.89E-110	1.17E-09
cg26700919	18	13375474	*C18orf1*	0.727	0.778	0.763	0.052	0.036	4.99E-40	6.84E-10
cg21243597	18	13375540	*C18orf1*	0.577	0.650	0.615	0.073	0.039	2.51E-48	3.21E-08
cg25140190	18	13641529	*C18orf1*	0.465	0.385	0.431	-0.081	-0.035	1.08E-58	2.31E-06
cg19389293	18	13641872	*C18orf1*	0.408	0.324	0.372	-0.085	-0.036	1.03E-97	8.77E-11
cg04967578	18	13641894	*C18orf1*	0.575	0.485	0.541	-0.090	-0.034	1.31E-65	2.34E-07
cg23679819	18	13730665	*RNMT*	0.584	0.665	0.623	0.081	0.039	2.33E-89	8.57E-13
cg21321768	18	19176371	*ESCO1*	0.558	0.599	0.611	0.041	0.053	7.21E-11	3.80E-08
cg26052586	18	20717411	*CABLES1*	0.320	0.256	0.299	-0.064	-0.022	2.71E-93	3.56E-07
cg26551026	18	20717693	*CABLES1*	0.577	0.508	0.540	-0.069	-0.037	9.17E-64	5.92E-11
cg21243631	18	21154260	*NPC1*	0.500	0.570	0.525	0.070	0.025	1.71E-96	1.39E-08
cg12709880	18	21163172	*NPC1*	0.554	0.448	0.518	-0.106	-0.036	2.80E-120	9.24E-10
cg20937934	18	21452788	*LAMA3*	0.794	0.856	0.825	0.062	0.031	2.56E-105	9.98E-15
cg13270625	18	21452819	*LAMA3*	0.759	0.817	0.792	0.057	0.033	5.83E-115	3.54E-21
cg03634729	18	21452829	*LAMA3*	0.676	0.752	0.717	0.075	0.040	1.48E-135	2.77E-22
cg17205313	18	21572748	*TTC39C*	0.715	0.767	0.740	0.052	0.026	1.43E-61	1.46E-08
cg25952192	18	21656374	*TTC39C*	0.604	0.669	0.633	0.065	0.029	8.29E-79	2.71E-09
cg07283015	18	22039857	*HRH4*	0.534	0.414	0.481	-0.120	-0.053	7.08E-137	3.98E-15
cg27514550	18	23971201	*TAF4B*	0.670	0.722	0.726	0.052	0.056	1.12E-22	2.88E-12
cg25784280	18	32289350	*DTNA*	0.404	0.318	0.360	-0.086	-0.044	1.02E-88	2.35E-13
cg05714729	18	33234096	*GALNT1*	0.423	0.334	0.377	-0.089	-0.046	2.08E-108	1.61E-16
cg24217948	18	42261980	*SETBP1*	0.720	0.681	0.666	-0.039	-0.054	8.48E-20	4.59E-19
cg21579539	18	43751229	NA	0.606	0.544	0.584	-0.062	-0.023	4.25E-73	1.48E-06
cg06418113	18	43915901	*RNF165*	0.315	0.251	0.286	-0.065	-0.029	9.78E-65	2.65E-07
cg17903316	18	44181660	*LOXHD1*	0.295	0.244	0.264	-0.050	-0.031	5.95E-45	5.69E-08
cg20211629	18	48352490	*MRO*	0.529	0.453	0.493	-0.076	-0.036	3.47E-73	8.10E-10
cg21417204	18	48609268	*SMAD4*	0.625	0.693	0.670	0.068	0.045	2.33E-54	9.07E-14
cg02895724	18	48719384	*MEX3C*	0.754	0.806	0.783	0.052	0.029	9.60E-46	1.60E-07
cg16669619	18	52630472	NA	0.783	0.825	0.836	0.042	0.053	5.39E-15	1.36E-11
cg21013983	18	56987316	*CPLX4*	0.774	0.818	0.825	0.044	0.050	7.41E-20	1.20E-11
cg05875463	18	60385273	*PHLPP1*	0.442	0.351	0.403	-0.091	-0.039	5.83E-105	1.59E-11
cg08223235	18	60903834	*BCL2*	0.374	0.272	0.336	-0.101	-0.038	1.12E-99	3.44E-08
cg27569829	18	60984485	*BCL2*	0.562	0.627	0.589	0.066	0.028	1.10E-54	5.74E-06
cg25373630	18	60984656	*BCL2*	0.735	0.793	0.777	0.058	0.042	1.22E-45	6.92E-13
cg01056135	18	61253151	*SERPINB13*	0.373	0.311	0.345	-0.061	-0.028	1.42E-53	3.69E-06
cg23582644	18	61557687	*SERPINB2*	0.552	0.478	0.521	-0.074	-0.030	1.00E-68	2.33E-08
cg20875821	18	61557735	*SERPINB2*	0.453	0.359	0.406	-0.094	-0.047	1.10E-114	1.90E-19
cg23696618	18	61583699	*SERPINB10*	0.447	0.362	0.408	-0.085	-0.039	6.36E-104	1.36E-13
cg23484392	18	66465334	*CCDC102B*	0.643	0.587	0.595	-0.056	-0.049	3.97E-32	2.92E-13
cg23973972	18	72152075	NA	0.557	0.607	0.615	0.049	0.058	3.39E-23	1.43E-14
cg16387467	18	72166016	*CNDP2*	0.223	0.169	0.196	-0.054	-0.027	7.06E-70	1.30E-09
cg27096572	18	73141041	*C18orf62*	0.637	0.702	0.685	0.066	0.048	2.18E-39	7.56E-11
cg14573759	18	73966755	NA	0.715	0.766	0.775	0.051	0.060	7.13E-25	1.24E-14
cg12645852	18	74198774	*ZNF516*	0.441	0.336	0.393	-0.105	-0.048	2.95E-151	9.51E-22
cg11984636	18	74845706	*MBP*	0.390	0.337	0.352	-0.053	-0.037	3.10E-35	2.17E-09
cg16604566	18	74845829	*MBP*	0.390	0.296	0.344	-0.094	-0.046	9.18E-111	5.39E-15
cg22324981	18	77283493	*NFATC1*	0.618	0.702	0.664	0.084	0.046	5.90E-100	6.05E-16
cg06784563	18	77284509	*NFATC1*	0.502	0.576	0.529	0.074	0.027	2.65E-98	7.58E-10
cg06705017	18	77552402	NA	0.550	0.468	0.512	-0.083	-0.038	1.97E-59	4.88E-08
cg22095490	18	77560089	NA	0.638	0.689	0.674	0.051	0.036	1.91E-34	5.62E-08
cg18338021	19	544349	*GZMM*	0.719	0.773	0.742	0.054	0.023	1.81E-83	3.05E-10
cg01799015	19	707791	*PALM*	0.613	0.541	0.585	-0.072	-0.028	3.11E-69	2.87E-08
cg02147126	19	827715	*AZU1*	0.452	0.363	0.422	-0.089	-0.030	1.39E-142	5.62E-15
cg14663914	19	827739	*AZU1*	0.397	0.319	0.362	-0.078	-0.035	3.44E-110	6.05E-15
cg10752406	19	827776	*AZU1*	0.322	0.258	0.302	-0.064	-0.020	7.17E-97	2.57E-08
cg15610437	19	827821	*AZU1*	0.487	0.416	0.464	-0.070	-0.023	3.53E-110	1.08E-10
cg24347562	19	839678	*PRTN3*	0.407	0.342	0.377	-0.065	-0.030	1.17E-75	1.85E-10
cg12230709	19	840873	*PRTN3*	0.246	0.186	0.224	-0.060	-0.022	1.67E-86	9.37E-07
cg24304425	19	840985	*PRTN3*	0.367	0.303	0.344	-0.064	-0.023	4.83E-78	1.95E-06
cg02082462	19	848001	*PRTN3*	0.305	0.242	0.278	-0.063	-0.027	1.30E-57	1.28E-06
cg23357789	19	848026	*PRTN3*	0.314	0.250	0.286	-0.064	-0.028	6.89E-80	8.64E-09
cg22761077	19	850975	*ELANE*	0.253	0.193	0.233	-0.060	-0.021	5.32E-96	1.50E-07
cg15530560	19	851290	*ELANE*	0.487	0.409	0.465	-0.078	-0.022	1.01E-139	5.78E-09
cg06100973	19	852114	*ELANE*	0.419	0.320	0.375	-0.099	-0.044	3.45E-96	3.11E-11
cg08091707	19	859554	*CFD*	0.250	0.190	0.224	-0.060	-0.025	3.85E-77	1.32E-07

cg18084554	19	929046	*ARID3A*	0.394	0.313	0.362	-0.081	-0.031	4.45E-165	3.55E-19
cg25298189	19	935259	*ARID3A*	0.493	0.419	0.471	-0.074	-0.022	8.05E-96	2.99E-08
cg01774027	19	947712	*ARID3A*	0.389	0.321	0.360	-0.067	-0.029	9.56E-79	9.18E-09
cg16239536	19	1079617	*HMHA1*	0.678	0.747	0.706	0.069	0.028	6.06E-88	3.27E-09
cg19649900	19	1155030	*SBNO2*	0.432	0.366	0.412	-0.067	-0.021	1.98E-80	2.26E-06
cg10819238	19	1155184	*SBNO2*	0.299	0.237	0.275	-0.062	-0.024	8.75E-83	2.67E-08
cg06578434	19	1155225	*SBNO2*	0.504	0.423	0.483	-0.081	-0.021	1.36E-117	5.52E-07
cg23866916	19	1155738	*SBNO2*	0.553	0.486	0.530	-0.067	-0.023	8.71E-94	1.14E-07
cg08408668	19	1173241	*SBNO2*	0.407	0.336	0.373	-0.071	-0.034	4.15E-122	3.65E-22
cg18655369	19	1177575	NA	0.382	0.299	0.346	-0.083	-0.036	4.21E-145	1.34E-18
cg05989054	19	1402626	*GAMT*	0.243	0.189	0.222	-0.054	-0.020	9.45E-94	1.61E-08
cg22684041	19	1904554	*SCAMP4*	0.285	0.222	0.259	-0.063	-0.026	2.57E-112	8.36E-13
cg05345285	19	2215718	*DOT1L*	0.815	0.866	0.840	0.051	0.025	2.22E-74	5.99E-10
cg02856688	19	2266542	NA	0.465	0.403	0.430	-0.061	-0.034	6.63E-52	8.06E-09
cg24339704	19	2529022	*GNG7*	0.534	0.432	0.503	-0.102	-0.031	1.41E-118	4.92E-08
cg04603130	19	2550027	*GNG7*	0.469	0.359	0.426	-0.110	-0.043	1.82E-95	2.80E-09
cg17617491	19	2607726	*GNG7*	0.719	0.781	0.752	0.061	0.033	1.03E-109	1.90E-17
cg22459924	19	2607850	*GNG7*	0.640	0.717	0.670	0.077	0.030	7.56E-102	2.19E-09
cg19477361	19	2607903	*GNG7*	0.628	0.680	0.654	0.052	0.026	2.96E-103	1.90E-15
cg24723883	19	2608495	*GNG7*	0.753	0.816	0.776	0.063	0.023	1.02E-87	8.33E-07
cg18229071	19	2695245	*GNG7*	0.314	0.254	0.285	-0.060	-0.029	4.01E-133	8.83E-20
cg04330326	19	2770448	*SGTA*	0.355	0.294	0.333	-0.061	-0.022	3.17E-106	2.11E-10
cg26794830	19	3193804	*NCLN*	0.323	0.256	0.292	-0.067	-0.032	2.76E-107	2.87E-14
cg02818170	19	3336941	NA	0.566	0.513	0.538	-0.053	-0.028	1.38E-36	4.38E-07
cg14389547	19	3398778	*NFIC*	0.474	0.411	0.452	-0.062	-0.021	2.13E-100	1.39E-08
cg15658793	19	3398810	*NFIC*	0.462	0.400	0.443	-0.062	-0.019	2.71E-94	8.05E-08
cg07564563	19	3548977	*MFSD12*	0.497	0.403	0.468	-0.094	-0.030	8.57E-134	1.40E-10
cg19584038	19	3553032	*MFSD12*	0.344	0.269	0.305	-0.075	-0.039	7.13E-174	1.05E-32
cg22757101	19	3607136	*TBXA2R*	0.301	0.239	0.275	-0.062	-0.026	8.56E-66	8.35E-07
cg19225308	19	3607139	*TBXA2R*	0.256	0.198	0.232	-0.057	-0.023	2.78E-68	1.20E-06
cg23846712	19	3607608	*TBXA2R*	0.363	0.299	0.323	-0.064	-0.040	7.68E-81	1.08E-16
cg05343404	19	3608327	*TBXA2R*	0.278	0.228	0.259	-0.050	-0.019	4.15E-141	1.13E-15
cg04603976	19	4052706	*ZBTB7A*	0.372	0.319	0.346	-0.053	-0.027	9.21E-71	2.83E-11
cg07328519	19	4064300	*ZBTB7A*	0.337	0.283	0.304	-0.054	-0.033	6.46E-72	1.21E-16
cg26925688	19	4303957	*FSD1*	0.527	0.595	0.553	0.068	0.026	4.11E-126	6.14E-16
cg24926276	19	4539943	*LRG1*	0.525	0.443	0.497	-0.082	-0.028	5.75E-107	6.83E-10
cg08837884	19	4540782	*LRG1*	0.682	0.629	0.660	-0.054	-0.022	2.29E-55	1.84E-06
cg25806655	19	4543498	*SEMA6B*	0.477	0.406	0.452	-0.072	-0.026	2.14E-71	1.08E-07
cg02849956	19	4634827	NA	0.279	0.219	0.237	-0.061	-0.042	5.63E-77	4.52E-19
cg22898924	19	4903952	*ARRDC5*	0.478	0.407	0.437	-0.071	-0.040	5.63E-43	5.13E-08
cg18351781	19	4950888	*UHRF1*	0.468	0.355	0.435	-0.113	-0.033	4.04E-122	3.62E-08
cg24236839	19	5038822	*KDM4B*	0.410	0.337	0.378	-0.073	-0.032	7.04E-135	8.90E-20
cg15621731	19	5074616	*KDM4B*	0.415	0.333	0.383	-0.082	-0.031	1.19E-90	1.05E-08
cg07584494	19	5074758	*KDM4B*	0.494	0.428	0.475	-0.065	-0.019	8.00E-90	3.63E-06
cg09001777	19	5851504	*FUT3*	0.286	0.230	0.259	-0.056	-0.027	2.31E-105	1.10E-13
cg12110801	19	5992284	*LOC1001285*	0.497	0.391	0.446	-0.106	-0.051	2.21E-143	9.99E-21
cg10736454	19	7739455	NA	0.330	0.273	0.308	-0.057	-0.022	2.17E-64	2.28E-06
cg05641903	19	7767584	*FCER2*	0.443	0.370	0.409	-0.073	-0.034	1.97E-90	4.65E-13
cg01482790	19	8513923	*HNRNPM*	0.611	0.675	0.648	0.064	0.037	6.90E-59	4.04E-11
cg02319986	19	8568712	*PRAM1*	0.445	0.348	0.418	-0.097	-0.027	2.71E-166	1.09E-09
cg06655349	19	10332165	*S1PR2*	0.490	0.377	0.450	-0.114	-0.041	2.95E-124	7.62E-11
cg00500176	19	10396158	*ICAM1*	0.550	0.494	0.517	-0.056	-0.033	1.34E-47	3.48E-09
cg22910295	19	10403862	*ICAM5*	0.425	0.465	0.475	0.040	0.050	1.12E-12	1.93E-10
cg10604476	19	10403908	*ICAM5*	0.476	0.550	0.570	0.074	0.094	1.07E-13	5.82E-11
cg15538607	19	10416150	*ZGLP1*	0.372	0.303	0.343	-0.069	-0.030	2.67E-101	1.43E-12
cg03722295	19	10519375	NA	0.653	0.726	0.690	0.073	0.036	3.47E-62	6.86E-08
cg01279933	19	10663970	*ATG4D*	0.555	0.613	0.578	0.058	0.023	8.47E-94	3.95E-11
cg14160422	19	10735548	*SLC44A2*	0.732	0.795	0.757	0.063	0.025	3.48E-101	2.69E-10
cg26847100	19	10748778	*SLC44A2*	0.380	0.290	0.350	-0.090	-0.030	8.61E-116	4.32E-10
cg08315613	19	11074303	*SMARCA4*	0.554	0.625	0.584	0.071	0.029	1.16E-102	7.28E-13
cg22898082	19	11074428	*SMARCA4*	0.542	0.602	0.570	0.060	0.029	4.44E-81	5.09E-13
cg03875330	19	11455923	*CCDC159*	0.482	0.427	0.442	-0.055	-0.040	1.58E-45	7.47E-11
cg04857672	19	11495077	*EPOR*	0.228	0.177	0.207	-0.051	-0.021	6.40E-83	7.65E-09
cg20086579	19	11665102	*ELOF1*	0.648	0.715	0.677	0.066	0.029	3.33E-77	4.06E-08
cg17658002	19	11689997	*ACP5*	0.350	0.296	0.325	-0.054	-0.025	3.38E-56	8.71E-07
cg01558916	19	11804727	NA	0.434	0.331	0.396	-0.104	-0.038	3.30E-150	1.73E-13
cg19821297	19	12890029	NA	0.545	0.479	0.519	-0.066	-0.026	1.67E-58	3.05E-06
cg19867991	19	13051573	*CALR*	0.403	0.331	0.368	-0.072	-0.035	1.39E-92	7.35E-13
cg04334723	19	13054427	*CALR*	0.441	0.342	0.397	-0.099	-0.044	3.31E-122	2.40E-15
cg25605731	19	13054434	*CALR*	0.432	0.314	0.394	-0.118	-0.038	1.69E-143	1.00E-09
cg08806632	19	13214412	*LYL1*	0.264	0.194	0.229	-0.070	-0.035	1.23E-88	5.39E-13
cg17753124	19	13259872	*STX10*	0.547	0.491	0.520	-0.056	-0.028	1.96E-62	6.77E-08
cg20281375	19	13946770	*LOC284454*	0.334	0.267	0.296	-0.067	-0.038	9.81E-116	5.15E-22
cg16572540	19	13947229	NA	0.394	0.342	0.360	-0.052	-0.033	4.11E-73	4.24E-18
cg24704287	19	13951481	NA	0.334	0.282	0.304	-0.051	-0.030	4.00E-54	2.96E-12
cg06905453	19	14089284	*RFX1*	0.285	0.221	0.257	-0.064	-0.028	4.93E-91	6.37E-11
cg03624195	19	14090310	*RFX1*	0.386	0.311	0.367	-0.075	-0.020	9.43E-100	4.15E-06
cg18109874	19	14115611	*RFX1*	0.496	0.439	0.468	-0.057	-0.028	1.03E-72	4.38E-11

cg02327522	19	14633030	NA	0.791	0.848	0.822	0.057	0.031	5.91E-83	3.51E-14
cg23690893	19	14671371	*TECR*	0.442	0.374	0.412	-0.068	-0.030	1.80E-76	2.39E-09
cg13973985	19	14785604	*EMR3*	0.251	0.198	0.227	-0.054	-0.025	3.14E-58	1.98E-06
cg15552238	19	14785749	*EMR3*	0.233	0.176	0.203	-0.057	-0.030	7.75E-72	7.93E-10
cg22027399	19	15217635	*SYDE1*	0.336	0.274	0.310	-0.061	-0.026	1.49E-75	2.76E-07
cg20078972	19	15391832	*BRD4*	0.412	0.306	0.366	-0.106	-0.046	1.16E-117	4.88E-14
cg08044694	19	15391927	*BRD4*	0.408	0.316	0.370	-0.093	-0.039	1.77E-121	2.01E-14
cg10377921	19	15391946	*BRD4*	0.511	0.447	0.479	-0.064	-0.032	2.42E-98	1.10E-15
cg27067618	19	15751949	*CYP4F3*	0.272	0.217	0.245	-0.055	-0.027	9.18E-54	3.64E-07
cg02381279	19	16394366	NA	0.414	0.308	0.375	-0.106	-0.039	2.46E-116	4.67E-10
cg18473733	19	16437362	*KLF2*	0.588	0.654	0.614	0.065	0.026	3.96E-125	1.67E-15
cg02668248	19	16437789	*KLF2*	0.545	0.623	0.584	0.078	0.039	1.31E-84	5.15E-14
cg19280540	19	16553711	*EPS15L1*	0.400	0.332	0.381	-0.068	-0.018	1.87E-113	8.60E-07
cg19418318	19	17219073	*MYO9B*	0.511	0.416	0.477	-0.094	-0.034	3.25E-69	1.92E-06
cg07839313	19	17514600	*BST2*	0.507	0.444	0.464	-0.063	-0.043	8.70E-51	1.72E-17
cg08358907	19	17877508	*FCHO1*	0.413	0.361	0.391	-0.051	-0.022	2.30E-43	3.48E-06
cg10614223	19	18111389	*ARRDC2*	0.594	0.653	0.614	0.059	0.020	1.35E-93	1.27E-07
cg24137511	19	18260330	*MAST3*	0.858	0.913	0.882	0.055	0.024	3.61E-73	1.00E-06
cg06706159	19	18260350	*MAST3*	0.823	0.899	0.854	0.077	0.032	1.61E-82	5.67E-07
cg27287167	19	18267904	*PIK3R2*	0.416	0.365	0.392	-0.050	-0.024	1.57E-86	2.71E-13
cg19640821	19	18503517	*LRRC25*	0.248	0.195	0.222	-0.054	-0.026	9.94E-72	3.14E-10
cg19839325	19	18761132	*KLHL26*	0.452	0.395	0.424	-0.057	-0.028	7.49E-48	9.77E-07
cg05336188	19	18959506	*UPF1*	0.406	0.353	0.376	-0.054	-0.030	3.72E-59	4.91E-14
cg23956760	19	19178708	*SLC25A42*	0.492	0.410	0.453	-0.082	-0.038	1.35E-103	3.59E-14
cg18462898	19	19282451	*MEF2BNB-M*	0.510	0.452	0.478	-0.058	-0.032	6.38E-35	3.76E-07
cg17340170	19	21686146	NA	0.749	0.789	0.800	0.041	0.052	2.75E-17	8.98E-12
cg13977235	19	33172072	NA	0.467	0.351	0.428	-0.115	-0.039	1.75E-152	8.71E-13
cg08700306	19	33686390	*LRP3*	0.399	0.332	0.368	-0.068	-0.032	5.63E-125	5.80E-19
cg09310092	19	35529201	*SCN1B*	0.376	0.324	0.336	-0.052	-0.040	1.28E-45	2.48E-14
cg27461196	19	35630106	*FXYD1*	0.376	0.302	0.349	-0.074	-0.027	1.01E-145	3.10E-17
cg18503912	19	35630279	*FXYD1*	0.477	0.361	0.430	-0.116	-0.048	1.26E-136	1.89E-15
cg17540545	19	35630355	*FXYD1*	0.315	0.241	0.283	-0.074	-0.032	1.28E-128	6.23E-14
cg10830496	19	35634556	*FXYD7*	0.290	0.233	0.257	-0.057	-0.033	2.76E-59	7.93E-10
cg22628286	19	35643121	*FXYD7*	0.260	0.200	0.230	-0.060	-0.031	1.86E-77	2.64E-11
cg11902728	19	35786580	*MAG*	0.330	0.279	0.305	-0.052	-0.025	1.18E-45	2.92E-07
cg15001615	19	35819775	*CD22*	0.248	0.190	0.217	-0.058	-0.030	4.28E-57	7.98E-08
cg15743985	19	35819809	*CD22*	0.364	0.283	0.332	-0.080	-0.031	9.82E-76	3.94E-06
cg19977428	19	35819985	*CD22*	0.255	0.201	0.231	-0.055	-0.024	4.70E-74	4.23E-08
cg03574571	19	35820181	*CD22*	0.461	0.365	0.408	-0.096	-0.052	2.03E-143	2.32E-25
cg21640432	19	35939561	*FFAR2*	0.344	0.256	0.305	-0.088	-0.039	5.55E-181	1.63E-23
cg03068068	19	36203512	*ZBTB32*	0.592	0.660	0.629	0.068	0.037	8.70E-55	3.40E-09
cg08539991	19	36203832	*ZBTB32*	0.715	0.788	0.739	0.072	0.023	6.77E-134	9.50E-10
cg24091474	19	36399185	*TYROBP*	0.387	0.316	0.364	-0.071	-0.023	4.15E-105	4.96E-08
cg17925829	19	36399328	*TYROBP*	0.333	0.275	0.304	-0.058	-0.029	8.54E-73	8.40E-10
cg21495704	19	36399346	*TYROBP*	0.361	0.286	0.334	-0.075	-0.027	9.53E-83	4.23E-07
cg24834889	19	37825406	*HKR1*	0.399	0.461	0.464	0.063	0.065	1.22E-12	3.01E-06
cg12948621	19	37825446	*HKR1*	0.322	0.373	0.397	0.051	0.075	2.20E-10	2.81E-09
cg21281463	19	38423760	*SIPA1L3*	0.324	0.248	0.288	-0.076	-0.037	3.58E-97	6.04E-14
cg26831416	19	39142011	*ACTN4*	0.393	0.298	0.353	-0.094	-0.040	4.97E-167	1.34E-25
cg15581429	19	39369353	*SIRT2*	0.648	0.716	0.680	0.068	0.031	3.66E-83	5.28E-11
cg16745596	19	39695801	*SYCN*	0.416	0.353	0.360	-0.063	-0.056	3.73E-41	1.40E-13
cg03301498	19	39889222	*MED29*	0.373	0.289	0.332	-0.084	-0.041	2.01E-150	1.67E-23
cg05209306	19	39899376	*ZFP36*	0.681	0.762	0.714	0.082	0.033	3.60E-114	1.18E-13
cg14085840	19	40939429	NA	0.308	0.253	0.280	-0.055	-0.028	8.63E-83	5.87E-13
cg23990942	19	40946878	*SERTAD3*	0.444	0.365	0.411	-0.079	-0.033	3.51E-175	2.13E-23
cg25734726	19	40948296	*SERTAD3*	0.520	0.436	0.482	-0.084	-0.037	5.67E-104	5.72E-13
cg01409498	19	41429786	NA	0.707	0.757	0.759	0.050	0.052	4.61E-22	1.19E-10
cg22840076	19	41782156	*HNRNPUL1*	0.523	0.435	0.486	-0.089	-0.037	1.65E-138	3.86E-16
cg11313468	19	41782183	*HNRNPUL1*	0.492	0.374	0.438	-0.118	-0.054	6.68E-194	2.65E-25
cg26383537	19	41817029	*CCDC97*	0.721	0.752	0.772	0.031	0.051	2.23E-11	6.61E-13
cg21500909	19	41870640	*TMEM91*	0.449	0.358	0.405	-0.091	-0.044	3.80E-96	1.64E-14
cg13736811	19	41873930	*TMEM91*	0.394	0.295	0.348	-0.100	-0.046	3.41E-121	8.51E-15
cg21658670	19	41930894	*BCKDHA*	0.429	0.352	0.408	-0.076	-0.021	1.51E-123	7.67E-07
cg21126943	19	42259395	*CEACAM6*	0.342	0.264	0.301	-0.078	-0.041	6.70E-130	1.86E-22
cg04790874	19	42381950	*CD79A*	0.399	0.326	0.372	-0.073	-0.026	5.21E-88	3.61E-07
cg03731797	19	42900540	NA	0.299	0.223	0.263	-0.076	-0.037	2.02E-155	2.08E-26
cg14779825	19	43099845	*CEACAM8*	0.335	0.267	0.301	-0.068	-0.034	2.05E-85	1.02E-11
cg12032655	19	43856746	*CD177*	0.546	0.470	0.510	-0.076	-0.036	1.14E-46	1.30E-06
cg13554818	19	43979316	*PHLDB3*	0.382	0.323	0.349	-0.059	-0.033	2.58E-37	3.28E-06
cg25750408	19	44159698	*PLAUR*	0.661	0.598	0.634	-0.063	-0.027	1.12E-91	4.76E-11
cg10767665	19	44279027	*KCNN4*	0.492	0.428	0.456	-0.064	-0.035	6.88E-35	1.61E-06
cg26476925	19	45245446	NA	0.365	0.290	0.338	-0.075	-0.026	2.80E-150	9.02E-13
cg02768785	19	45912979	*ERCC1*	0.300	0.238	0.273	-0.062	-0.027	2.85E-70	2.46E-08
cg09997082	19	46170946	*GIPR*	0.494	0.432	0.453	-0.062	-0.041	5.67E-49	2.87E-11
cg18467978	19	47134906	NA	0.625	0.558	0.594	-0.067	-0.031	5.41E-64	1.69E-09
cg04556210	19	47840110	*GPR77*	0.302	0.238	0.279	-0.064	-0.023	8.33E-101	1.87E-08
cg17705615	19	47991237	*NAPA*	0.406	0.356	0.384	-0.051	-0.022	7.48E-78	2.35E-09
cg23314866	19	48016020	*NAPA*	0.285	0.225	0.261	-0.060	-0.024	7.96E-93	5.09E-10

cg23511285	19	48205761	*GLTSCR1*	0.542	0.459	0.518	-0.083	-0.024	2.88E-104	3.13E-07
cg27064845	19	48252623	*GLTSCR2*	0.643	0.708	0.661	0.065	0.019	6.64E-130	7.03E-08
cg03100209	19	49921142	*CCDC155*	0.294	0.228	0.258	-0.065	-0.036	9.79E-58	6.14E-09
cg07569288	19	50002551	*RPS11*	0.624	0.706	0.659	0.081	0.035	1.44E-115	1.41E-12
cg07071036	19	50014987	*FCGRT*	0.295	0.235	0.267	-0.060	-0.028	6.53E-81	1.64E-10
cg18081863	19	50015527	*FCGRT*	0.392	0.317	0.364	-0.075	-0.028	4.61E-90	2.18E-09
cg09293816	19	50015532	*FCGRT*	0.416	0.347	0.389	-0.069	-0.027	2.38E-89	1.88E-10
cg11263420	19	50019310	*FCGRT*	0.284	0.213	0.245	-0.070	-0.039	3.71E-136	1.95E-24
cg07377519	19	50269083	*AP2A1*	0.366	0.269	0.323	-0.097	-0.043	2.00E-131	1.44E-16
cg25261331	19	51628121	*SIGLEC9*	0.345	0.292	0.323	-0.053	-0.021	2.88E-66	1.64E-07
cg22469897	19	51858276	*ETFB*	0.752	0.804	0.781	0.052	0.029	3.01E-61	5.76E-10
cg10126923	19	51875451	*NKG7*	0.365	0.290	0.336	-0.076	-0.030	4.11E-109	6.55E-11
cg04264075	19	51876470	*NKG7*	0.266	0.213	0.232	-0.053	-0.033	1.49E-115	2.21E-24
cg25417766	19	52191468	NA	0.388	0.326	0.355	-0.061	-0.033	6.64E-54	9.07E-08
cg00845742	19	52453199	NA	0.750	0.801	0.810	0.051	0.059	5.06E-21	1.66E-13
cg08130292	19	52870301	*ZNF610*	0.748	0.798	0.804	0.050	0.056	2.03E-20	1.67E-12
cg01566282	19	53794975	*BIRC8*	0.739	0.782	0.795	0.043	0.055	8.27E-15	1.10E-10
cg26633373	19	54377836	*MYADM*	0.425	0.344	0.403	-0.081	-0.022	2.75E-107	1.69E-06
cg01718139	19	54566838	*VSTM1*	0.555	0.446	0.522	-0.109	-0.033	1.12E-102	1.02E-06
cg25930786	19	54584636	*TARM1*	0.427	0.325	0.386	-0.102	-0.041	5.10E-151	1.81E-14
cg21073459	19	54604098	*OSCAR*	0.400	0.329	0.379	-0.072	-0.021	1.25E-123	2.05E-08
cg14511156	19	54604124	*OSCAR*	0.296	0.239	0.277	-0.056	-0.019	2.56E-84	3.27E-06
cg18447740	19	54604187	*OSCAR*	0.342	0.262	0.309	-0.079	-0.033	1.97E-135	9.05E-17
cg22820233	19	55385581	*FCAR*	0.460	0.356	0.420	-0.104	-0.041	1.15E-102	2.35E-09
cg05252264	19	55385587	*FCAR*	0.376	0.280	0.336	-0.096	-0.040	8.77E-88	2.14E-08
cg09470754	19	55838021	*TMEM150B*	0.391	0.301	0.357	-0.090	-0.033	8.46E-181	4.35E-19
cg11311352	19	56058710	NA	0.286	0.222	0.255	-0.064	-0.031	7.90E-122	5.01E-18
cg24805411	19	56158851	*CCDC106*	0.340	0.288	0.316	-0.052	-0.024	6.79E-71	1.11E-08
cg02348449	19	58630429	*ZSCAN18*	0.426	0.360	0.401	-0.066	-0.025	1.39E-111	1.89E-12
cg19931902	20	207144	*DEFB129*	0.686	0.749	0.737	0.063	0.051	4.29E-43	1.37E-13
cg23206873	20	1303672	*SDCBP2*	0.390	0.316	0.359	-0.074	-0.031	2.81E-97	2.72E-11
cg13226290	20	1448595	*NSFL1C*	0.472	0.373	0.440	-0.099	-0.032	7.89E-118	7.02E-09
cg07571344	20	1472071	*SIRPB2*	0.254	0.194	0.220	-0.060	-0.034	8.34E-52	2.50E-08
cg10131232	20	1610358	*SIRPG*	0.442	0.364	0.399	-0.078	-0.043	6.11E-51	1.77E-09
cg13408605	20	1639816	*SIRPG*	0.743	0.796	0.779	0.052	0.036	1.47E-41	6.68E-11
cg00637104	20	1785303	NA	0.483	0.399	0.458	-0.084	-0.025	1.82E-87	2.36E-06
cg14613594	20	1878646	*SIRPA*	0.384	0.323	0.357	-0.061	-0.027	1.97E-92	5.60E-12
cg10206397	20	2085344	*STK35*	0.556	0.454	0.522	-0.102	-0.034	4.94E-115	9.97E-09
cg01978937	20	2671574	NA	0.277	0.203	0.250	-0.074	-0.027	4.19E-96	5.93E-08
cg05825244	20	2730488	*EBF4*	0.468	0.539	0.574	0.071	0.106	2.32E-11	2.36E-11
cg15013617	20	2795378	*C20orf141*	0.428	0.373	0.394	-0.056	-0.035	1.12E-39	4.17E-08
cg21278103	20	2928233	*PTPRA*	0.402	0.325	0.364	-0.077	-0.038	2.63E-88	5.06E-13
cg04731988	20	3051954	*OXT*	0.443	0.386	0.404	-0.057	-0.039	2.49E-37	7.05E-09
cg19592472	20	3052274	*OXT*	0.437	0.364	0.390	-0.073	-0.047	6.06E-31	3.35E-07
cg07582993	20	3137766	*FASTKD5*	0.680	0.738	0.711	0.058	0.031	4.19E-37	5.44E-06
cg26130864	20	3208100	*SLC4A11*	0.502	0.427	0.467	-0.075	-0.036	6.31E-97	1.94E-12
cg17661220	20	3693179	NA	0.359	0.301	0.338	-0.058	-0.021	3.71E-74	5.85E-07
cg05753799	20	3746315	*C20orf27*	0.369	0.287	0.333	-0.082	-0.035	3.70E-163	6.71E-20
cg14165142	20	3778655	*CDC25B*	0.653	0.738	0.678	0.085	0.025	2.81E-125	2.60E-07
cg02737268	20	3780182	*CDC25B*	0.418	0.517	0.451	0.098	0.033	2.48E-69	4.16E-06
cg22147446	20	4879261	*SLC23A2*	0.432	0.372	0.407	-0.059	-0.025	2.07E-73	3.60E-09
cg05822633	20	11898531	*BTBD3*	0.320	0.269	0.289	-0.051	-0.031	4.46E-49	1.46E-09
cg14801864	20	17540975	*BFSP1*	0.485	0.417	0.458	-0.068	-0.027	1.35E-82	1.05E-09
cg05955436	20	17660838	*RRBP1*	0.337	0.256	0.295	-0.081	-0.042	2.04E-68	2.40E-09
cg12339328	20	18491484	*SEC23B*	0.509	0.414	0.467	-0.096	-0.043	4.28E-119	5.74E-15
cg14083146	20	20694136	*RALGAPA2*	0.495	0.427	0.465	-0.068	-0.029	3.62E-60	6.46E-07
cg09971811	20	24930099	*CST7*	0.370	0.293	0.323	-0.077	-0.047	9.72E-76	3.14E-15
cg02065051	20	25674725	*ZNF337*	0.448	0.390	0.418	-0.058	-0.030	3.82E-42	6.90E-07
cg24727216	20	30947724	*ASXL1*	0.545	0.601	0.565	0.055	0.020	5.81E-70	2.17E-06
cg24484138	20	31070190	*C20orf112*	0.427	0.328	0.398	-0.100	-0.029	5.63E-109	5.33E-06
cg22052056	20	31351813	*DNMT3B*	0.690	0.624	0.641	-0.066	-0.049	1.03E-75	9.00E-23
cg13788819	20	31352228	*DNMT3B*	0.304	0.239	0.267	-0.064	-0.036	1.36E-83	5.10E-13
cg09835408	20	31352399	*DNMT3B*	0.386	0.304	0.347	-0.082	-0.039	5.23E-142	1.91E-21
cg26553763	20	31352874	*DNMT3B*	0.561	0.496	0.514	-0.065	-0.047	6.46E-60	4.05E-17
cg04087207	20	31669392	*BPIFB4*	0.415	0.321	0.374	-0.094	-0.041	1.11E-106	9.46E-13
cg11231701	20	31946710	*CDK5RAP1*	0.470	0.396	0.439	-0.074	-0.030	1.67E-70	2.97E-07
cg16678522	20	32149963	*CBFA2T2*	0.592	0.516	0.555	-0.076	-0.037	7.38E-79	2.57E-10
cg02138331	20	32893975	*AHCY*	0.679	0.599	0.652	-0.080	-0.027	2.75E-80	1.27E-07
cg17162808	20	33266296	*PIGU*	0.707	0.743	0.757	0.036	0.050	3.63E-14	6.96E-13
cg16151538	20	33676388	*TRPC4AP*	0.615	0.549	0.582	-0.066	-0.033	3.35E-91	8.42E-15
cg17206393	20	33681223	*TRPC4AP*	0.375	0.275	0.332	-0.100	-0.043	1.04E-108	1.08E-11
cg21211882	20	34770405	*EPB41L1*	0.332	0.267	0.310	-0.065	-0.021	5.26E-98	5.93E-08
cg04410989	20	35578437	*SAMHD1*	0.275	0.213	0.240	-0.062	-0.035	4.42E-42	1.35E-08
cg01141721	20	36010619	*SRC*	0.508	0.421	0.475	-0.087	-0.033	1.85E-74	9.39E-07
cg20709530	20	36040249	NA	0.339	0.280	0.299	-0.058	-0.040	5.67E-69	4.05E-15
cg16959758	20	36796513	NA	0.346	0.283	0.319	-0.063	-0.026	3.39E-113	3.54E-14
cg00514723	20	40244770	*CHD6*	0.395	0.311	0.361	-0.085	-0.034	1.55E-99	7.18E-10
cg14609407	20	43883172	*SLPI*	0.410	0.323	0.383	-0.087	-0.026	1.54E-91	6.08E-06

cg08548498	20	43883546	*SLPI*	0.284	0.224	0.247	-0.060	-0.036	7.99E-63	9.32E-13
cg02509204	20	44043235	*PIGT*	0.734	0.793	0.792	0.059	0.058	3.06E-23	1.06E-10
cg13334949	20	44471068	*SNX21*	0.306	0.247	0.270	-0.059	-0.036	3.38E-94	3.54E-18
cg21310090	20	44538669	*PLTP*	0.446	0.350	0.408	-0.097	-0.038	6.13E-147	1.31E-16
cg21602257	20	44538675	*PLTP*	0.451	0.351	0.409	-0.100	-0.042	2.36E-175	4.94E-26
cg18059369	20	44543173	NA	0.279	0.202	0.246	-0.077	-0.033	3.02E-152	3.64E-17
cg13733708	20	44597685	*ZNF335*	0.485	0.402	0.446	-0.082	-0.038	2.27E-134	6.49E-18
cg25948982	20	45179157	*OCSTAMP*	0.232	0.176	0.211	-0.056	-0.021	5.34E-109	2.80E-09
cg11173131	20	45179226	*OCSTAMP*	0.340	0.254	0.305	-0.086	-0.035	3.04E-95	5.52E-09
cg01700462	20	45179230	*OCSTAMP*	0.388	0.291	0.347	-0.096	-0.041	1.30E-112	3.12E-12
cg17801864	20	45179345	*OCSTAMP*	0.323	0.250	0.291	-0.074	-0.033	2.23E-97	4.64E-11
cg01880463	20	45179413	*OCSTAMP*	0.268	0.199	0.230	-0.069	-0.038	1.05E-100	4.06E-16
cg03082779	20	45947025	*ZMYND8*	0.341	0.263	0.311	-0.078	-0.031	5.14E-195	5.80E-24
cg02341503	20	45947123	*ZMYND8*	0.386	0.313	0.361	-0.073	-0.025	2.57E-176	1.37E-15
cg09046309	20	45947226	*ZMYND8*	0.457	0.349	0.418	-0.108	-0.039	9.41E-124	4.75E-11
cg25541209	20	45947892	*ZMYND8*	0.427	0.350	0.400	-0.077	-0.027	2.48E-79	1.23E-06
cg26206598	20	47445432	*PREX1*	0.556	0.493	0.528	-0.063	-0.028	4.70E-57	1.18E-07
cg18112953	20	47448545	NA	0.490	0.395	0.454	-0.095	-0.036	1.33E-116	2.00E-10
cg06122230	20	47887219	*ZNFX1*	0.596	0.489	0.553	-0.107	-0.043	4.32E-103	5.56E-11
cg00340958	20	48328658	*B4GALT5*	0.485	0.373	0.439	-0.112	-0.046	1.54E-175	6.41E-20
cg22941573	20	48761279	*TMEM189*	0.559	0.460	0.513	-0.099	-0.045	1.81E-119	2.57E-18
cg05232694	20	48809539	NA	0.443	0.389	0.398	-0.054	-0.045	2.00E-31	1.64E-10
cg27585822	20	49253552	*FAM65C*	0.405	0.332	0.370	-0.073	-0.035	7.98E-127	5.67E-16
cg22214889	20	49410828	*BCAS4*	0.241	0.188	0.218	-0.053	-0.023	3.93E-61	4.05E-08
cg20039443	20	50108912	*NFATC2*	0.456	0.389	0.434	-0.067	-0.021	6.41E-97	1.95E-08
cg13518270	20	50415588	*SALL4*	0.665	0.727	0.711	0.062	0.045	7.56E-37	5.12E-10
cg15878616	20	52492209	*SUMO1P1*	0.517	0.403	0.489	-0.114	-0.028	8.92E-139	2.75E-06
cg09712306	20	54949299	*AURKA*	0.323	0.269	0.289	-0.054	-0.034	2.40E-33	4.20E-07
cg03030267	20	57394000	*GNAS-AS1*	0.769	0.822	0.794	0.053	0.026	1.47E-79	1.61E-11
cg17398227	20	57582787	*CTSZ*	0.332	0.276	0.308	-0.056	-0.023	2.63E-55	4.46E-06
cg08545593	20	57582856	*CTSZ*	0.429	0.327	0.387	-0.102	-0.041	8.09E-116	4.00E-12
cg01623438	20	57582894	*CTSZ*	0.345	0.262	0.306	-0.083	-0.040	2.13E-124	4.95E-17
cg21331324	20	57583000	*CTSZ*	0.372	0.299	0.318	-0.074	-0.055	1.34E-104	8.94E-31
cg22145559	20	57583009	*CTSZ*	0.348	0.270	0.307	-0.078	-0.041	3.89E-132	1.04E-22
cg18245281	20	57583076	*CTSZ*	0.329	0.256	0.299	-0.073	-0.029	1.09E-118	5.95E-15
cg17928895	20	57583091	*CTSZ*	0.342	0.261	0.303	-0.080	-0.039	4.20E-106	1.13E-13
cg12787553	20	57583188	*CTSZ*	0.330	0.258	0.295	-0.072	-0.035	1.28E-102	2.37E-14
cg09315134	20	57583195	*CTSZ*	0.326	0.252	0.292	-0.074	-0.033	7.99E-104	9.49E-12
cg22231620	20	57605092	*SLMO2-ATP5*	0.743	0.789	0.796	0.046	0.053	3.07E-19	8.59E-12
cg24334029	20	58296208	*PHACTR3*	0.659	0.583	0.624	-0.076	-0.035	3.32E-56	7.01E-08
cg25724895	20	58630390	*C20orf197*	0.355	0.289	0.326	-0.066	-0.028	6.20E-114	2.88E-14
cg01521397	20	60590872	*TAF4*	0.490	0.390	0.459	-0.100	-0.031	4.72E-89	4.87E-06
cg21341487	20	60760919	*GTPBP5*	0.427	0.331	0.390	-0.097	-0.037	2.11E-168	5.87E-19
cg06026331	20	60912101	*LAMA5*	0.732	0.772	0.787	0.039	0.054	1.55E-12	5.35E-10
cg00727912	20	61583736	*SLC17A9*	0.281	0.228	0.251	-0.053	-0.030	3.01E-76	1.64E-13
cg23352030	20	62198469	*PRIC285*	0.702	0.788	0.766	0.086	0.064	1.59E-48	3.35E-16
cg04300115	20	62200199	*PRIC285*	0.572	0.645	0.611	0.073	0.040	3.22E-63	7.83E-14
cg03590031	20	62200285	*PRIC285*	0.772	0.822	0.806	0.051	0.035	4.67E-49	1.20E-12
cg01190666	20	62204908	*PRIC285*	0.545	0.443	0.478	-0.102	-0.068	1.20E-111	8.81E-41
cg00446123	20	62367888	*LIME1*	0.533	0.656	0.563	0.123	0.030	1.88E-122	2.90E-06
cg20513976	20	62367893	*LIME1*	0.454	0.565	0.483	0.111	0.029	3.88E-96	5.25E-06
cg12413156	20	62368256	*LIME1*	0.567	0.659	0.598	0.092	0.032	9.42E-107	6.61E-10
cg09868035	20	62492074	*ABHD16B*	0.335	0.277	0.311	-0.058	-0.024	1.52E-113	2.75E-14
cg19222525	20	62492220	*ABHD16B*	0.325	0.273	0.306	-0.052	-0.019	2.66E-91	5.26E-09
cg23085846	20	62522518	*TPD52L2*	0.493	0.415	0.470	-0.078	-0.023	2.82E-116	5.84E-08
cg07543138	21	16434067	*NRIP1*	0.378	0.297	0.332	-0.081	-0.046	1.15E-99	8.14E-18
cg14544087	21	26945916	*MIR155HG*	0.768	0.823	0.809	0.055	0.040	2.31E-45	2.37E-11
cg14414154	21	27538021	*APP*	0.475	0.356	0.426	-0.119	-0.049	5.53E-156	1.08E-16
cg22076474	21	30677172	*BACH1*	0.437	0.356	0.404	-0.081	-0.033	1.33E-78	4.80E-08
cg06287548	21	34773372	NA	0.441	0.351	0.402	-0.090	-0.039	2.11E-129	1.92E-16
cg08173915	21	34774164	*IFNGR2*	0.490	0.377	0.447	-0.113	-0.043	8.32E-144	1.39E-15
cg17356733	21	34774627	*IFNGR2*	0.557	0.449	0.522	-0.107	-0.035	3.77E-105	2.67E-08
cg02019444	21	35016787	*ITSN1*	0.315	0.240	0.284	-0.075	-0.031	9.84E-136	1.71E-14
cg16452651	21	35016873	*ITSN1*	0.480	0.365	0.429	-0.116	-0.052	1.78E-191	5.66E-26
cg15125868	21	35746798	*FAM165B*	0.394	0.299	0.355	-0.096	-0.040	1.45E-144	1.58E-15
cg15683970	21	35747081	*FAM165B*	0.310	0.254	0.277	-0.056	-0.033	4.77E-48	3.15E-09
cg03801286	21	35884508	*KCNE1*	0.402	0.307	0.362	-0.095	-0.040	3.17E-166	6.89E-22
cg13707794	21	35889668	*RCAN1*	0.325	0.259	0.299	-0.066	-0.026	1.12E-62	3.00E-06
cg16756527	21	35891075	*RCAN1*	0.583	0.532	0.552	-0.050	-0.031	1.02E-30	2.27E-06
cg22698744	21	36263808	*RUNX1*	0.577	0.498	0.533	-0.079	-0.044	1.24E-31	1.14E-06
cg00291213	21	36398056	*RUNX1*	0.363	0.278	0.317	-0.084	-0.046	7.76E-142	2.75E-25
cg14641757	21	36410464	*RUNX1-IT1*	0.747	0.805	0.773	0.059	0.027	3.17E-86	3.63E-11
cg00673191	21	37536923	*DOPEY2*	0.525	0.472	0.502	-0.053	-0.023	8.00E-52	2.37E-06
cg01261013	21	37691747	*MORC3*	0.551	0.454	0.518	-0.097	-0.033	5.70E-105	1.38E-09
cg00990740	21	39871287	*ERG*	0.301	0.240	0.279	-0.062	-0.022	3.92E-77	3.83E-06
cg17228105	21	39871301	*ERG*	0.511	0.432	0.477	-0.078	-0.034	1.03E-74	6.55E-09
cg17432627	21	40124277	*LINC00114*	0.488	0.410	0.451	-0.079	-0.037	1.25E-52	7.56E-07
cg00168694	21	40193056	*ETS2*	0.452	0.338	0.411	-0.115	-0.042	2.09E-142	8.47E-14

cg24341911	21	40689521	*BRWD1-AS1*	0.312	0.242	0.276	-0.070	-0.035	6.64E-65	3.56E-09
cg22862003	21	42797588	*MX1*	0.698	0.427	0.511	-0.270	-0.187	2.54E-126	7.87E-63
cg26312951	21	42797847	*MX1*	0.435	0.259	0.294	-0.176	-0.141	1.34E-82	1.32E-41
cg21549285	21	42799141	*MX1*	0.827	0.405	0.568	-0.422	-0.260	3.46E-139	6.86E-59
cg01176028	21	43653234	*ABCG1*	0.267	0.216	0.240	-0.051	-0.027	1.20E-61	9.04E-10
cg00177237	21	43658316	*ABCG1*	0.738	0.788	0.773	0.051	0.035	2.03E-39	3.48E-09
cg27280688	21	43823809	*UBASH3A*	0.736	0.793	0.768	0.058	0.032	3.23E-49	2.55E-08
cg09354050	21	43824262	*UBASH3A*	0.721	0.793	0.752	0.072	0.030	9.24E-122	2.66E-13
cg07391141	21	44035034	NA	0.610	0.660	0.636	0.050	0.026	3.23E-63	2.53E-10
cg08136416	21	44060833	NA	0.328	0.262	0.283	-0.066	-0.045	1.00E-82	1.82E-21
cg25491402	21	44101491	*PDE9A*	0.665	0.734	0.692	0.069	0.027	3.51E-139	5.60E-15
cg14171527	21	44473840	*CBS*	0.445	0.354	0.406	-0.091	-0.039	1.90E-89	8.26E-11
cg23036852	21	44809983	NA	0.281	0.217	0.249	-0.064	-0.032	1.40E-87	1.25E-11
cg22726155	21	45575559	NA	0.701	0.770	0.734	0.069	0.033	7.05E-86	9.36E-13
cg19344545	21	45575573	NA	0.685	0.754	0.719	0.069	0.034	1.08E-78	1.00E-11
cg08039560	21	45575832	NA	0.724	0.779	0.757	0.055	0.033	6.30E-59	1.82E-11
cg01772743	21	45576085	NA	0.709	0.766	0.741	0.057	0.032	5.19E-56	2.49E-09
cg23366234	21	45713704	*AIRE*	0.779	0.838	0.809	0.059	0.030	1.13E-73	8.34E-11
cg26472802	21	45713719	*AIRE*	0.702	0.782	0.739	0.080	0.038	2.07E-77	2.33E-10
cg06812844	21	45773782	*TRPM2*	0.406	0.312	0.365	-0.094	-0.041	1.42E-121	4.30E-14
cg23312431	21	45773997	*TRPM2*	0.435	0.342	0.404	-0.093	-0.031	3.60E-137	7.14E-13
cg24548817	21	45774294	*TRPM2*	0.475	0.368	0.441	-0.106	-0.034	3.30E-139	7.51E-10
cg07806328	21	45774664	*TRPM2*	0.646	0.589	0.619	-0.057	-0.027	4.59E-59	7.88E-08
cg07781197	21	46046362	*KRTAP10-9*	0.766	0.818	0.790	0.052	0.024	1.23E-86	1.44E-10
cg16501560	21	46078007	*TSPEAR*	0.584	0.668	0.609	0.084	0.025	5.72E-124	1.39E-10
cg10871721	21	46235052	*SUMO3*	0.443	0.387	0.415	-0.057	-0.029	8.16E-71	4.38E-09
cg04217515	21	46325853	*ITGB2*	0.477	0.416	0.448	-0.061	-0.029	2.34E-55	3.64E-08
cg14112356	21	46348443	*ITGB2-AS1*	0.567	0.654	0.594	0.087	0.027	3.99E-163	2.68E-12
cg11327657	21	46388162	*FAM207A*	0.334	0.250	0.302	-0.085	-0.032	9.44E-94	1.02E-07
cg22635096	21	46550644	*ADARB1*	0.399	0.347	0.364	-0.052	-0.035	3.72E-55	1.11E-14
cg07658590	21	46963587	*SLC19A1*	0.453	0.392	0.416	-0.061	-0.037	1.89E-58	2.46E-11
cg11121969	21	47268670	*PCBP3*	0.772	0.828	0.816	0.056	0.044	2.84E-36	2.81E-11
cg22217449	21	47845788	*PCNT*	0.765	0.818	0.792	0.053	0.027	1.17E-56	1.08E-07
cg19716090	22	17560707	NA	0.617	0.538	0.578	-0.079	-0.040	1.21E-77	4.09E-11
cg13762691	22	17956560	*CECR2*	0.312	0.250	0.285	-0.062	-0.027	2.66E-68	5.64E-07
cg07197230	22	17956641	*CECR2*	0.316	0.232	0.284	-0.085	-0.033	2.34E-103	1.20E-08
cg14293575	22	18635460	*USP18*	0.838	0.744	0.795	-0.093	-0.042	3.65E-39	1.68E-15
cg18731680	22	19953712	*COMT*	0.423	0.338	0.384	-0.085	-0.039	1.50E-115	2.52E-15
cg01138652	22	19973978	*ARVCF*	0.358	0.286	0.327	-0.072	-0.031	3.28E-68	3.52E-07
cg20208009	22	19974048	*ARVCF*	0.371	0.290	0.338	-0.081	-0.033	8.42E-105	3.09E-10
cg06850285	22	21921269	*UBE2L3*	0.234	0.174	0.203	-0.060	-0.031	2.76E-105	4.34E-18
cg01800253	22	22290866	*PPM1F*	0.359	0.267	0.323	-0.092	-0.036	5.55E-118	3.02E-11
cg07187268	22	24105186	*C22orf15*	0.467	0.412	0.445	-0.055	-0.022	2.89E-62	7.60E-07
cg05580073	22	24803248	*SPECC1L*	0.379	0.306	0.334	-0.073	-0.044	2.89E-75	4.21E-14
cg26001125	22	24823050	*ADORA2A*	0.678	0.745	0.698	0.066	0.020	7.35E-146	1.73E-09
cg01373166	22	24823389	*ADORA2A*	0.628	0.683	0.649	0.056	0.021	3.10E-72	1.49E-07
cg03091668	22	24903402	*UPB1*	0.382	0.308	0.350	-0.073	-0.032	1.41E-80	8.21E-08
cg26253663	22	25799504	NA	0.486	0.374	0.441	-0.112	-0.045	1.55E-118	6.20E-15
cg15382568	22	25800078	NA	0.337	0.254	0.303	-0.082	-0.033	5.85E-94	8.85E-09
cg13345299	22	26876075	*HPS4*	0.301	0.226	0.261	-0.075	-0.040	3.87E-112	4.80E-19
cg07924575	22	26881231	*HPS4*	0.568	0.517	0.543	-0.052	-0.025	8.32E-63	1.35E-07
cg02455383	22	27014116	*CRYBB1*	0.402	0.339	0.358	-0.062	-0.043	6.60E-76	1.83E-19
cg23772226	22	29225779	NA	0.435	0.321	0.377	-0.114	-0.058	3.84E-180	1.06E-30
cg18036081	22	29605147	*EMID1*	0.399	0.330	0.366	-0.069	-0.033	1.06E-131	3.67E-20
cg02357416	22	29668384	*EWSR1*	0.728	0.774	0.778	0.046	0.051	3.32E-22	4.56E-12
cg11942221	22	29686496	*EWSR1*	0.697	0.750	0.745	0.053	0.047	3.15E-17	6.44E-07
cg23414595	22	30129607	*ZMAT5*	0.361	0.310	0.337	-0.051	-0.024	4.54E-95	5.89E-13
cg14742445	22	30195299	*ASCC2*	0.403	0.312	0.366	-0.091	-0.037	8.95E-185	2.21E-25
cg22115126	22	30283578	*MTMR3*	0.323	0.250	0.299	-0.073	-0.025	6.55E-92	1.54E-06
cg23825480	22	31336785	*MORC2*	0.533	0.622	0.576	0.089	0.043	1.08E-85	1.17E-12
cg01606027	22	31607212	*LIMK2*	0.345	0.270	0.317	-0.075	-0.028	1.94E-75	2.50E-06
cg10827767	22	31794522	*DRG1*	0.655	0.586	0.627	-0.069	-0.029	9.15E-73	5.15E-09
cg11860005	22	32149125	*DEPDC5*	0.336	0.265	0.301	-0.071	-0.035	1.11E-152	2.26E-26
cg00965578	22	36560801	*APOL3*	0.324	0.248	0.287	-0.077	-0.038	3.77E-135	6.54E-23
cg22088368	22	37404888	*TEX33*	0.278	0.222	0.254	-0.055	-0.024	2.44E-70	8.20E-08
cg04995291	22	37546325	*IL2RB*	0.670	0.724	0.699	0.054	0.029	9.54E-49	1.72E-07
cg26999345	22	37584441	*C1QTNF6*	0.377	0.310	0.352	-0.068	-0.026	2.74E-113	9.27E-12
cg02527527	22	38303310	*MICALL1*	0.372	0.295	0.340	-0.077	-0.033	4.35E-106	2.66E-13
cg20686403	22	38438206	NA	0.420	0.329	0.392	-0.090	-0.028	2.40E-200	6.92E-16
cg11324504	22	38474147	*SLC16A8*	0.402	0.316	0.377	-0.086	-0.025	4.17E-121	4.88E-09
cg00159439	22	38597193	*MAFF*	0.692	0.743	0.707	0.051	0.015	2.16E-94	5.47E-06
cg01403010	22	38609534	*MAFF*	0.393	0.340	0.373	-0.053	-0.020	3.28E-72	4.49E-07
cg26449680	22	38714272	*CSNK1E*	0.426	0.375	0.402	-0.051	-0.024	9.83E-55	1.34E-09
cg10142237	22	38714395	*CSNK1E*	0.460	0.399	0.428	-0.061	-0.032	2.13E-72	1.01E-13
cg01441777	22	38714416	*CSNK1E*	0.463	0.407	0.433	-0.056	-0.030	5.46E-78	2.47E-16
cg07313319	22	38714426	*CSNK1E*	0.529	0.452	0.483	-0.076	-0.046	8.85E-69	7.57E-14
cg10546888	22	38890734	*DDX17*	0.483	0.544	0.517	0.060	0.034	3.45E-57	2.48E-10
cg20623350	22	40289840	*ENTHD1*	0.500	0.439	0.472	-0.061	-0.028	2.27E-54	8.02E-07

cg25276892	22	40573076	*TNRC6B*	0.547	0.607	0.581	0.060	0.034	1.18E-51	1.06E-09
cg16093065	22	40720633	*TNRC6B*	0.795	0.846	0.825	0.052	0.031	1.88E-57	9.18E-10
cg08362785	22	40814878	*MKL1*	0.606	0.658	0.636	0.052	0.030	1.03E-53	2.61E-10
cg05162166	22	41684460	NA	0.485	0.387	0.437	-0.098	-0.048	4.06E-128	2.66E-17
cg24414363	22	42336273	*CENPM*	0.584	0.668	0.604	0.083	0.020	2.84E-172	9.54E-10
cg07044422	22	42828516	*NFAM1*	0.254	0.195	0.225	-0.059	-0.029	6.97E-81	3.55E-10
cg25044876	22	43041146	*CYB5R3*	0.434	0.348	0.395	-0.086	-0.040	2.91E-90	3.31E-11
cg19460508	22	44422195	*PARVB*	0.768	0.717	0.732	-0.051	-0.036	1.75E-30	8.27E-09
cg12555844	22	44568337	*PARVG*	0.331	0.262	0.299	-0.069	-0.032	8.24E-104	9.80E-14
cg09080522	22	44568387	*PARVG*	0.268	0.200	0.243	-0.068	-0.025	8.75E-70	3.45E-06
cg16873414	22	44568699	*PARVG*	0.295	0.237	0.272	-0.059	-0.023	1.87E-63	4.62E-06
cg26861460	22	44575455	*PARVG*	0.449	0.338	0.397	-0.111	-0.052	2.68E-147	1.23E-20
cg14942952	22	44576268	*PARVG*	0.541	0.475	0.501	-0.065	-0.039	2.14E-54	2.80E-10
cg18659081	22	44588350	*PARVG*	0.466	0.350	0.416	-0.116	-0.050	2.49E-154	3.92E-19
cg16166559	22	45072588	*PRR5*	0.202	0.151	0.180	-0.051	-0.022	8.03E-70	1.92E-07
cg22018086	22	45124555	*PRR5-ARHGA*	0.327	0.258	0.293	-0.069	-0.034	3.32E-100	9.34E-14
cg05249836	22	45609402	*KIAA0930*	0.545	0.430	0.504	-0.116	-0.041	1.54E-122	1.66E-11
cg18855195	22	45828264	*RIBC2*	0.523	0.470	0.481	-0.054	-0.043	7.69E-25	6.54E-08
cg01234420	22	46453808	*LOC150381*	0.529	0.456	0.471	-0.073	-0.058	7.68E-58	4.49E-19
cg09978533	22	46465160	NA	0.353	0.253	0.311	-0.100	-0.043	2.34E-98	5.72E-10
cg03834031	22	46465717	NA	0.240	0.179	0.206	-0.061	-0.034	2.13E-84	1.89E-13
cg09137533	22	46469091	NA	0.427	0.370	0.379	-0.057	-0.049	1.89E-40	3.03E-14
cg06635946	22	46470016	NA	0.684	0.629	0.635	-0.054	-0.048	1.20E-37	1.93E-16
cg27172287	22	46974976	NA	0.483	0.416	0.458	-0.067	-0.025	1.04E-129	5.83E-12
cg24157349	22	47081751	*CERK*	0.321	0.252	0.288	-0.069	-0.033	1.16E-84	4.62E-11
cg23659250	22	50174065	*BRD1*	0.525	0.449	0.498	-0.076	-0.027	3.95E-65	3.89E-06
cg23762465	22	50360628	NA	0.396	0.319	0.366	-0.077	-0.030	8.23E-144	1.28E-14
cg20194973	22	50524676	*MLC1*	0.378	0.312	0.344	-0.066	-0.035	1.41E-64	6.54E-10
cg18040409	22	50865814	*PPP6R2*	0.774	0.819	0.829	0.046	0.055	3.66E-21	8.41E-14
cg10416593	22	50966123	*TYMP*	0.646	0.584	0.603	-0.062	-0.043	1.82E-47	1.73E-12
cg11224765	22	50971109	*ODF3B*	0.324	0.236	0.287	-0.088	-0.037	2.41E-75	7.44E-08
cg20098015	22	50971140	*ODF3B*	0.490	0.335	0.390	-0.155	-0.100	7.09E-96	2.15E-33
cg05523603	22	50973101	NA	0.718	0.591	0.642	-0.127	-0.076	4.72E-71	3.78E-26
cg08425796	22	50981121	NA	0.384	0.289	0.352	-0.095	-0.031	1.54E-93	5.87E-07
cg02247863	22	50983415	NA	0.763	0.712	0.734	-0.050	-0.028	2.38E-43	1.37E-10
cg07596065	22	50984393	NA	0.600	0.547	0.564	-0.054	-0.036	3.69E-51	1.13E-15
cg10531637	22	51000048	*SYCE3*	0.611	0.670	0.626	0.060	0.016	1.35E-130	8.51E-08
