# Supplementary material for: Shared and Unique Patterns of DNA Methylation in Systemic Lupus Erythematosus and Primary Sjögren's Syndrome
Source: Front Immunol. 2019 Jul 30;10:1686. doi: 10.3389/fimmu.2019.01686 (PMC6688520; doi:10.3389/fimmu.2019.01686)
Supplement: Supplementary file 4 [file Table_4.pdf]

Supplementary Table S4 *Functional pathway analysis of genes with differentially methylated CpG sites (DMCs) shared in SLE and pSS*

Pathway	Molecules	p-value
Neutrophil degranulation	*ACPP,ALDH3B1,ANXA2,ARG1,ARMC8,ATG7,ATP8B4,AZU1,BRI3,CD44, CD59,CDA,CEACAM6,CHI3L1,CHIT1,CLEC5A,CRISPLD2,CTSG,CTSZ,DOCK2, ELANE,FCAR,FCER1G,FCGR3B,FOLR3,GALNS,GHDC,GSN,HP,HPSE, IMPDH1,ITGAL,LPCAT1,LRG1,MPO,MS4A3,NBEAL2,OLR1,OSCAR,P2RX1, PECAM1,PRDX6,PRG2,PSMB7,RAB10,RAB44,RAB5C,RNASE2,S100A8, S100A9,S100P,SERPINB1,SERPINB10,SLC11A1,SLC15A4,SLC2A5,SLC44A2, TARM1,TMEM173,TNFAIP6,TNFRSF1B,TOLLIP,TRPM2,TYROBP,UBR4, VAT1*	1.12E-11
Innate immune System	*ACPP,AHCYL1,ALDH3B1,ANXA2,APP,ARG1,ARMC8,ATG7,ATP6V1E2, ATP8B4,AZU1,BCL2,BIRC2,BPIFB4,BRI3,C4BPA,CAMK2G,CD247,CD300LB, CD3G,CD44,CD59,CDA,CDKN1A,CEACAM6,CHI3L1,CHIT1,CLEC5A,CLU, CRISPLD2,CTSG,CTSZ,CUL1,CUL3,DNM3,DOCK2,ELANE,ELMO1,ERBB2, FCAR,FCER1G,FCGR3B,FCN3,FOLR3,GALNS,GHDC,GSN,HLA-E,HP,HPSE, IL1B,IMPDH1,IRAK2,IRAK3,ITGAL,ITPR1,KLRD1,KSR1,LCK,LPCAT1,LRG1, MAP2K6,MAP3K11,MAPKAPK2,MEFV,MPO,MS4A3,NBEAL2,NCK1, NFATC1,NLRC4,NLRP3,OLR1,OSCAR,P2RX1,PANX1,PECAM1,PHLPP1, PIK3AP1,PIK3CD,PRDX6,PRG2,PRKCA,PRKCE,PSMB7,RAB10,RAB44, RAB5C,RASA3,RIPK3,RNASE2,RPS6KA1,RPS6KA2,RPS6KB2,S100A7A, S100A8,S100A9,S100P,SARM1,SERPINB1,SERPINB10,SLC11A1,SLC15A4, SLC2A5,SLC44A2,SPTBN1,SYNGAP1,TARM1,TLR6,TMEM173,TNFAIP6, TNFRSF1B,TOLLIP,TRAF2,TREM1,TREM2,TREX1,TRPM2,TXK,TYROBP, UBE2N,UBR4,VAT1,WIPF1*	6.52E-10
Keratinocyte differentiation	*BCL2,ETS1,ETS2,FASLG,MAP2K6,MAP3K5,PRKCA,PRKCE,SP1,TNF, TNFRSF1B,TRAF2*	4.90E-06
p38 MAPK signaling	*CCM2,MAP2K6,MAP3K3,MAP3K5,MAPKAPK2,MKNK1,TNF,TRAF2*	1.31E-05
IL2 signaling events mediated by STAT5	*BCL2,CCND2,CDK6,FASLG,LCK,LTA,PRF1,SP1,STAT5A*	2.23E-05
Regulation of retinoblastoma protein	*ATF7,BRD2,CBX4,CDKN1A,CTBP1,PPARG,RB1,RUNX2,SFTPD,SMARCA4, SPI1,TFDP1*	4.23E-05
Sphingolipid signaling pathway	*ABCC1,BCL2,CERS2,FCER1G,GNA12,GNAI2,GNAI3,MAP3K5,PIK3CD, PLCB2,PRKCA,PRKCE,PRKCZ,S1PR1,S1PR2,SGMS2,TNF,TRAF2*	7.68E-05
C-MYB transcription factor network	*BCL2,CBX4,CD34,CDK6,CDKN1A,ELANE,ETS1,ETS2,HIPK2,MAD1L1, MPO,MYB,SP1,SPI1*	7.99E-05
Sphingosine 1-phosphate (S1P) pathway	*ABCC1,GNA12,GNAI2,GNAI3,GNAO1,S1PR1,S1PR2*	8.81E-05
AMPK signaling pathway	*ACACA,ADIPOR2,CPT1A,CREB3L2,G6PC3,PCK2,PFKFB2,PIK3CD,PPARG, PRKAA1,PRKAG1,RAB10,RPS6KB2,RPTOR,SREBF1,STRADA,TBC1D1,ULK1*	1.07E-04
Cholinergic synapse	*BCL2,CACNA1C,CAMK2G,CHRM3,CREB3L2,GNAI2,GNAI3,GNAO1,GNG7, GNGT2,ITPR1,KCNQ1,PIK3CD,PIK3R5,PIK3R6,PLCB2,PRKCA*	1.29E-04
Apoptosis signaling pathway	*ATF7,BCL2,BIRC2,CRADD,DIABLO,EIF2AK2,FASLG,LTA,LTB,MAP3K5, PIK3CD,PRKCA,PRKCE,TNF,TNFRSF1B,TRAF2*	1.36E-04
Pre-NOTCH expression and processing	*ATP2A2,E2F3,FURIN,LFNG,MAML1,MAML3,NOTCH1,NOTCH4,ST3GAL3, TFDP1*	1.95E-04

Apelin signaling pathway	*CALML4,CDH1,GNAI2,GNAI3,GNG7,GNGT2,HDAC4,ITPR1,KLF2,NRF1, PIK3R5,PIK3R6,PLCB2,PRKAA1,PRKAG1,PRKCE,RPS6KB2,SLC8A1,SMAD3*	1.99E-04
Inflammasomes	*APP,BCL2,MEFV,NLRC4,NLRP3,PANX1*	2.01E-04
PDGF signaling pathway	*ARHGAP15,ARHGAP26,EHF,ETS1,FLI1,ITPR1,MAPKAPK2,MKNK1,NCK1, NCK2,PIK3CD,PIK3R5,PRKCA,PRR5-ARHGAP8,RPS6KA1,RPS6KA2, RPS6KB2,STAT5A*	2.01E-04
Nucleotide-binding domain, leucine rich repeat containing receptor (NLR) signaling pathways	*APP,BCL2,BIRC2,IRAK2,MAP2K6,MEFV,NLRC4,NLRP3,PANX1,UBE2N*	2.35E-04
Insulin resistance	*CPT1A,CREB3L2,G6PC3,NR1H3,OGA,PCK2,PIK3CD,PRKAA1,PRKAG1, PRKCE,PRKCZ,RPS6KA1,RPS6KA2,RPS6KB2,SREBF1,TNF*	2.40E-04
Hematopoietic cell lineage	*CD22,CD34,CD3G,CD44,CD59,CD7,CSF3R,FLT3,GP5,GP9,HLA-DMA,HLA-DMB,IL1B,ITGA5,TNF*	2.56E-04
Pre-NOTCH processing in Golgi	*ATP2A2,FURIN,LFNG,NOTCH1,NOTCH4,ST3GAL3*	2.87E-04
Downstream signaling in naive CD8+ T cells	*CD247,CD3G,FASLG,NFATC1,PRF1,PRKCA,PRKCE,PTPN7,TNF,TNFRSF9*	3.34E-04
Caspase cascade in apoptosis	*APP,BCL2,BIRC2,CRADD,DIABLO,GSN,PRF1,SREBF1,TNF,TRAF2*	3.34E-04
HTLV-I infection	*CALR,CCND2,CD3G,CDC16,CDKN1A,E2F3,ETS1,ETS2,HLA-DMA,HLA-DMB, HLA-E,ITGAL,LCK,LTA,LTBR,MAP3K3,MYB,NFATC1,PIK3CD,RB1,SMAD3, SPI1,STAT5A,TERT,TNF,VDAC2,WNT5B,ZFP36*	4.10E-04
Chagas disease (American trypanosomiasis)	*CALR,CD247,CD3G,FASLG,GNAI2,GNAI3,GNAO1,IFNGR2,IL10,IL1B, PIK3CD,PLCB2,SMAD3,TLR6,TNF*	4.47E-04
IL1-mediated signaling events	*IL1B,IRAK3,MAP2K6,MAP3K3,PRKCZ,TMED7-TICAM2,TOLLIP,UBE2N*	5.09E-04
African trypanosomiasis	*F2RL1,FASLG,IL10,IL18,IL1B,PLCB2,PRKCA,TNF*	5.09E-04
S1P5 pathway	*GNA12,GNAI2,GNAI3,GNAO1*	5.35E-04
Nef and signal transduction	*CD247,DOCK2,ELMO1,LCK*	5.35E-04
S1P1 pathway	*ABCC1,GNAI2,GNAI3,GNAO1,PLCB2,S1PR1*	5.45E-04
Pathways in cancer	*BCL2,BIRC2,CDH1,CDK6,CDKN1A,CSF3R,CTBP1,E2F3,EPAS1,ERBB2,ETS1, FASLG,FLT3,GNA12,GNAI2,GNAI3,GNG7,GNGT2,LAMA3,NCOA4,PIK3CD, PLCB2,PPARG,PRKCA,RALBP1,RASSF5,RB1,RUNX1,RXRA,SMAD3,SPI1, STAT5A,TCF7,TPM3,TRAF2,TRAF5,WNT5B,ZBTB16*	5.71E-04
Hemostasis	*ACTN1,ACTN4,ANXA2,APP,ATP2A2,CD44,CEACAM6,CLU,DGKZ,DOCK2, DOCK5,F10,FAM49B,FCER1G,FERMT3,GNA12,GNAI2,GNAI3,GNG7, GNGT2,GP5,GP9,GRB7,INPP5D,ITGA5,ITGAL,ITPK1,ITPR1,KIF2A,LCK,MYB, NFE2,OLR1,P2RX1,PDE9A,PECAM1,PHACTR2,PIK3CD,PIK3R5,PIK3R6, PRKCA,PRKCE,PRKCZ,RAD51B,RAPGEF3,RCOR1,SERPINB2,SLC16A8, SLC7A7,SLC7A8,SLC8A1,STIM1,TBXA2R,TREM1,WDR1*	6.50E-04
