# Supplementary material for: Shared and Unique Patterns of DNA Methylation in Systemic Lupus Erythematosus and Primary Sjögren's Syndrome
Source: Front Immunol. 2019 Jul 30;10:1686. doi: 10.3389/fimmu.2019.01686 (PMC6688520; doi:10.3389/fimmu.2019.01686)
Supplement: Supplementary file 5 [file Table_5.pdf]

Supplementary Table S5 *Functional pathway analysis of genes with differentially methylated CpG sites (DMCs) uniquely associated with SLE*

Pathway	Molecules	p-value
Hemostasis	*ANGPT1, ANGPT4, ARRB2, CBX5, CLU, CREBBP, DAGLB, F2R, FAM49B, FCAMR, GNA12, GNA15, GP6, GRB7, ITGB2, ITPR1, KIF21B, KLC1, MAFG, MGLL, MYB, P2RX7, P2RY12, PHACTR2, PIK3CD, PRKAR1B, PRTN3, RAD51B, SLC16A1, TBXA2R*	3.01×10^{-5}
Innate immune system	*ADCY7, AMPD3, ANGPT1, ARPC4, ARRB2, ATP11A, ATP6V0E2, CASP10, CASP8, CD247, CD59, CLEC4C, CLU, COTL1, CREBBP, DOK3, DUSP6, FADD, FCAR, FGF17, GRIN1, GSTP1, HLA-E, ITGB2, ITPR1, LAMTOR2, LPCAT1, LRRFIP1, MGST1, MYO10, MYO9B, OLFM4, P2RX7, PDGFRB, PELI2, PIK3CD, PIP5K1A, PRKAR1B, PRTN3, PSMB8, PTPRJ, RNF135, RPS6KA2, SPRED2, SYNGAP1, TNFAIP6, UBA3, UBR4, WIPF1*	4.05×10^{-5}
FasL/ CD95L signaling	*CASP10, CASP8, FADD*	8.83×10^{-5}
NF-κB activation through FADD/RIP-1 pathway mediated by caspase-8 and -10	*CASP10, CASP8, FADD, RNF135*	1.16×10^{-4}
Signaling by NOTCH	*ARRB2, CREBBP, DTX1, HEYL, LFNG, MAML2, NCOR2, ST3GAL3, ST3GAL6, TFDP1*	1.29×10^{-4}
Regulation of actin cytoskeleton	*ARPC4, F2R, FGF11, FGF17, GNA12, ITGAE, ITGB2, ITGB7, MYH10, PDGFD, PDGFRB, PIK3CD, PIP5K1A, ROCK2*	1.52×10^{-4}
