# Supplementary material for: Shared and Unique Patterns of DNA Methylation in Systemic Lupus Erythematosus and Primary Sjögren's Syndrome
Source: Front Immunol. 2019 Jul 30;10:1686. doi: 10.3389/fimmu.2019.01686 (PMC6688520; doi:10.3389/fimmu.2019.01686)
Supplement: Supplementary file 6 [file Table_6.pdf]

Supplemenatry Table S6 *Differentially methylated CpG sites (DMCs; n=2,244) in the association analysis comparing DNA methylation in patients with* SLE and patients with pSS (Bonferroni adjusted p<1.3E-07 and average methylation difference |Δβ|>0.05)

CpG site	Chromosome	Position	p-value[‡]	Mean β SLE	Mean β pSS	Methylation Δβ SLE-pSS	Gene	CpG Island Context	Gene Property	Exon/Intron Region
cg09010699	3	195171693	1.31E-68	0.431	0.527	-0.096	NA	NA	NA	NA
cg26341831	1	226036279	4.62E-65	0.679	0.597	0.082	*TMEM63A*	NA	GeneBody	Intron
cg20686403	22	38438206	3.45E-63	0.329	0.392	-0.063	NA	Shelf	NA	NA
cg01823925	5	156967901	1.98E-61	0.753	0.680	0.073	*ADAM19*	NA	GeneBody	Intron
cg10313337	16	68823690	3.40E-60	0.369	0.442	-0.073	*CDH1*	NA	GeneBody	Intron
cg07708453	1	14032034	1.50E-59	0.342	0.426	-0.084	*PRDM2*	NA	5UTR	Exon
cg05453411	16	89300123	1.85E-59	0.254	0.304	-0.050	NA	Shore	NA	NA
cg02319986	19	8568712	5.36E-59	0.348	0.418	-0.070	*PRAM1*	Shore	TSS1500	NA
cg17852482	17	80833054	1.11E-58	0.373	0.442	-0.069	*TBCD*	Shelf	GeneBody	Intron
cg01699630	6	131894023	2.20E-57	0.439	0.526	-0.087	*ARG1*	NA	TSS1500	NA
cg06470558	5	176816828	2.91E-57	0.646	0.593	0.054	*SLC34A1*	NA	3UTR	Exon
cg22713444	1	145512660	4.66E-57	0.332	0.410	-0.077	*RBM8A*	Shelf	3UTR	Exon
cg01582066	22	32057065	1.50E-56	0.593	0.512	0.081	NA	Shore	NA	NA
cg14919455	6	7167468	1.87E-56	0.357	0.427	-0.069	*RREB1*	NA	5UTR	Intron
cg13595556	9	129648322	2.05E-56	0.424	0.510	-0.086	NA	NA	NA	NA
cg24414363	22	42336273	2.46E-56	0.668	0.604	0.063	*CENPM*	NA	GeneBody	Intron
cg24427660	11	818892	6.13E-56	0.329	0.393	-0.063	*PNPLA2*	Shore	TSS200	NA
cg10487428	5	59559218	7.46E-56	0.402	0.477	-0.075	*PDE4D*	NA	5UTR	Intron
cg22381196	16	72041376	1.10E-55	0.384	0.460	-0.076	*DHODH*	Shore	TSS1500	NA
cg19055828	12	51139321	1.33E-55	0.396	0.490	-0.094	*DIP2B*	NA	3UTR	Exon
cg02226192	16	89461734	3.87E-55	0.393	0.466	-0.073	*ANKRD11*	NA	5UTR	Intron
cg19123356	8	130898833	1.29E-54	0.706	0.647	0.060	*FAM49B*	NA	ncRNA	Intron
cg08154963	17	33426885	1.32E-54	0.370	0.442	-0.072	*RAD51L3-RFFL*	NA	ncRNA	Intron
cg08539965	1	21396338	1.63E-54	0.368	0.452	-0.084	*EIF4G3*	NA	5UTR	Intron
cg10511985	1	24053853	3.34E-54	0.326	0.388	-0.061	NA	NA	NA	NA
cg10456459	12	22843015	4.83E-54	0.384	0.471	-0.087	*ETNK1*	NA	3UTR	Exon
cg25757820	2	224819307	4.70E-53	0.460	0.550	-0.091	NA	Shelf	NA	NA
cg17087974	11	65171397	5.74E-53	0.292	0.347	-0.055	*FRMD8*	Shore	GeneBody	Intron
cg06270401	12	4699085	2.00E-52	0.372	0.440	-0.069	*DYRK4*	NA	TSS200	NA
cg20611272	8	103548145	4.04E-52	0.742	0.687	0.055	NA	NA	NA	NA
cg03857198	3	152927597	4.06E-52	0.358	0.428	-0.070	NA	NA	NA	NA
cg13152690	14	91695017	4.39E-52	0.732	0.675	0.056	NA	NA	NA	NA
cg13633625	9	114706971	4.90E-52	0.401	0.485	-0.084	NA	NA	NA	NA
cg03022510	2	192476634	4.91E-52	0.312	0.370	-0.059	NA	NA	NA	NA
cg22805603	2	32490766	4.91E-52	0.389	0.452	-0.063	*NLRC4*	NA	TSS1500	Exon
cg19037107	11	134126323	5.93E-52	0.496	0.578	-0.083	*ACAD8*	Shelf	GeneBody	Intron
cg14112356	21	46348443	6.04E-52	0.654	0.594	0.060	*ITGB2-AS1*	Shelf	ncRNA	Exon
cg25739938	2	9610621	6.60E-52	0.451	0.530	-0.080	*CPSF3*	Shelf	GeneBody	Intron
cg25600606	11	33308345	1.02E-51	0.476	0.566	-0.091	*HIPK3*	NA	FirstExon	Exon
cg17756730	1	172608644	1.67E-51	0.733	0.679	0.054	NA	NA	NA	NA
cg18313182	11	818903	2.52E-51	0.364	0.419	-0.055	*PNPLA2*	Shore	5UTR	Exon
cg01692842	11	134126364	8.45E-51	0.414	0.503	-0.089	*ACAD8*	Shelf	GeneBody	Intron
cg09470754	19	55838021	8.70E-51	0.301	0.357	-0.056	*TMEM150B*	NA	TSS1500	NA
cg04730794	5	169144438	1.13E-50	0.414	0.498	-0.084	*DOCK2*	NA	GeneBody	Exon
cg13429095	1	206913187	2.23E-50	0.639	0.563	0.076	NA	NA	NA	NA
cg11193767	4	83930905	5.35E-50	0.315	0.377	-0.062	*LIN54*	Shelf	5UTR	Intron
cg23280720	6	139483193	9.74E-50	0.708	0.640	0.068	*HECA*	NA	GeneBody	Intron
cg17385088	4	153611560	2.32E-49	0.398	0.457	-0.059	NA	NA	NA	NA
cg03889044	2	242802099	3.23E-49	0.685	0.626	0.059	*PDCD1*	Shelf	TSS1500	NA
cg00278392	6	16802505	3.26E-49	0.324	0.400	-0.075	NA	NA	NA	NA
cg12711832	8	68258899	4.32E-49	0.323	0.406	-0.083	NA	Shelf	NA	NA
cg00501765	1	172410217	5.72E-49	0.410	0.471	-0.061	*C1orf105*	Shelf	GeneBody	Intron
cg26298914	14	68798365	5.87E-49	0.375	0.466	-0.091	*RAD51B*	NA	GeneBody	Intron
cg08628635	6	20483859	6.83E-49	0.354	0.428	-0.074	*E2F3*	NA	GeneBody	Intron
cg07472373	10	50595181	3.00E-48	0.376	0.457	-0.080	*DRGX*	NA	GeneBody	Intron
cg25305879	2	106814630	3.21E-48	0.389	0.462	-0.073	NA	Shelf	NA	NA
cg15015109	17	65136480	3.97E-48	0.363	0.427	-0.064	*HELZ*	NA	GeneBody	Intron
cg11014468	3	53362965	4.51E-48	0.431	0.492	-0.061	*DCP1A*	NA	GeneBody	Intron
cg23302989	7	25174492	6.34E-48	0.422	0.498	-0.077	*C7orf31*	NA	3UTR	Exon
cg04430911	1	36914349	8.01E-48	0.330	0.387	-0.058	*OSCP1*	Shore	GeneBody	Intron
cg01406317	16	4397291	8.36E-48	0.330	0.395	-0.065	*PAM16*	Shelf	GeneBody	Intron
cg18920397	1	160765805	8.67E-48	0.678	0.621	0.056	*LY9*	NA	TSS200	NA
cg11606261	12	53775336	1.42E-47	0.237	0.298	-0.061	*SP1*	Shore	5UTR	Intron
cg25444339	7	75194698	1.70E-47	0.294	0.349	-0.056	*HIP1*	NA	GeneBody	Intron
cg14192130	6	167535764	1.80E-47	0.770	0.710	0.061	*CCR6*	NA	5UTR	Intron
cg05627557	2	37418009	2.02E-47	0.295	0.346	-0.051	NA	NA	NA	NA
cg14516183	3	122928186	2.30E-47	0.407	0.496	-0.089	*SEC22A*	NA	FirstExon	Exon
cg01323964	7	65219171	3.59E-47	0.397	0.480	-0.083	*CCT6P1*	Shelf	ncRNA	Intron
cg13977235	19	33172072	5.74E-47	0.351	0.428	-0.076	NA	Shelf	NA	NA
cg19502359	11	72912441	5.85E-47	0.306	0.372	-0.066	NA	NA	NA	NA
cg18038894	17	48506385	6.37E-47	0.420	0.490	-0.070	*ACSF2*	Shelf	GeneBody	Intron
cg11358199	2	8453529	7.63E-47	0.740	0.683	0.057	*LINC00299*	NA	ncRNA	Intron
cg06465076	5	96027378	7.87E-47	0.466	0.554	-0.088	*CAST*	NA	GeneBody	Intron

cg05299836	16	31119067	8.07E-47	0.753	0.700	0.053	*BCKDK*	Shore	TSS1500	NA
cg14742445	22	30195299	1.16E-46	0.312	0.366	-0.054	*ASCC2*	NA	GeneBody	Intron
cg15889847	14	65183532	2.25E-46	0.356	0.410	-0.055	*PLEKHG3*	NA	5UTR	Intron
cg04831327	12	114299076	3.37E-46	0.373	0.433	-0.060	*RBM19*	NA	GeneBody	Intron
cg08688659	11	35249965	4.11E-46	0.351	0.422	-0.072	*CD44*	NA	GeneBody	Intron
cg11357013	15	73588054	4.51E-46	0.335	0.400	-0.065	*NEO1*	NA	GeneBody	Intron
cg10667895	2	102577876	4.55E-46	0.320	0.373	-0.052	NA	NA	NA	NA
cg05501357	11	33308269	6.45E-46	0.365	0.443	-0.077	*HIPK3*	NA	FirstExon	Exon
cg21127597	11	118109650	8.27E-46	0.306	0.369	-0.063	*MPZL3*	NA	GeneBody	Intron
cg07356342	1	161183820	9.30E-46	0.332	0.390	-0.058	*NDUFS2*	NA	3UTR	Exon
cg05904013	7	128579933	1.10E-45	0.331	0.387	-0.056	*IRF5*	Shore	5UTR	Intron
cg10471743	5	49739158	1.45E-45	0.322	0.405	-0.083	NA	Shore	NA	NA
cg06758539	5	75975285	2.01E-45	0.459	0.535	-0.076	*IQGAP2*	NA	GeneBody	Intron
cg24737761	6	106245659	2.23E-45	0.469	0.552	-0.083	NA	NA	NA	NA
cg25634666	11	71846788	2.62E-45	0.293	0.351	-0.058	*FOLR3*	NA	5UTR	Exon
cg02175033	14	70098523	2.73E-45	0.317	0.391	-0.073	*KIAA0247*	NA	5UTR	Intron
cg20241876	6	32180045	2.81E-45	0.316	0.368	-0.052	*NOTCH4*	NA	GeneBody	Intron
cg05380759	3	45579354	3.07E-45	0.316	0.375	-0.059	*LARS2*	NA	GeneBody	Intron
cg05454562	6	33254447	3.15E-45	0.409	0.478	-0.070	*WDR46*	Shelf	GeneBody	Intron
cg08622198	1	239979505	3.77E-45	0.349	0.410	-0.061	*CHRM3*	NA	5UTR	Intron
cg01044849	6	30002723	4.79E-45	0.473	0.547	-0.073	*ZNRD1-AS1*	NA	ncRNA	Exon
cg17932662	1	202123442	5.52E-45	0.393	0.467	-0.074	*PTPN7*	NA	GeneBody	Exon
cg03801286	21	35884508	5.74E-45	0.307	0.362	-0.055	*KCNE1*	NA	5UTR	Exon
cg24420366	10	3938541	7.02E-45	0.408	0.479	-0.071	NA	NA	NA	NA
cg24967811	12	123503709	7.35E-45	0.301	0.369	-0.068	*PITPNM2*	NA	GeneBody	Intron
cg06987246	7	6659785	7.68E-45	0.645	0.580	0.065	*ZNF853*	Shelf	GeneBody	Intron
cg04484126	7	65839403	8.04E-45	0.344	0.408	-0.064	NA	Shore	NA	NA
cg07102397	14	89864727	8.81E-45	0.478	0.564	-0.086	*FOXN3*	NA	GeneBody	Intron
cg05533539	17	44104521	9.40E-45	0.317	0.378	-0.061	*MAPT*	NA	3UTR	Exon
cg23445003	7	101535821	9.78E-45	0.344	0.414	-0.069	*CUX1*	NA	GeneBody	Intron
cg02423817	11	67203661	1.18E-44	0.681	0.612	0.069	*PTPRCAP*	CpGIsland	GeneBody	Exon
cg04609694	6	44204009	1.34E-44	0.409	0.494	-0.084	NA	Shore	NA	NA
cg08287940	2	160491979	1.65E-44	0.442	0.514	-0.071	NA	NA	NA	NA
cg10679182	7	133225096	1.97E-44	0.376	0.455	-0.078	*EXOC4*	NA	GeneBody	Intron
cg00026033	12	56414490	2.22E-44	0.298	0.353	-0.055	*IKZF4*	NA	TSS200	NA
cg25170017	11	64644487	2.41E-44	0.635	0.554	0.082	*EHD1*	Shore	GeneBody	Intron
cg20720686	7	75582881	3.13E-44	0.456	0.521	-0.065	*POR*	NA	5UTR	Intron
cg00340958	20	48328658	3.15E-44	0.373	0.439	-0.066	*B4GALT5*	Shore	GeneBody	Intron
cg19696103	5	132354130	3.48E-44	0.404	0.478	-0.074	*ZCCHC10*	NA	GeneBody	Intron
cg02505177	10	103574626	4.38E-44	0.685	0.623	0.062	*MGEA5*	Shelf	GeneBody	Intron
cg23054181	15	99048945	4.67E-44	0.405	0.484	-0.078	*FAM169B*	NA	5UTR	Intron
cg07986469	10	129795003	4.83E-44	0.349	0.408	-0.060	*PTPRE*	NA	5UTR	Intron
cg07285167	1	36948981	5.63E-44	0.282	0.341	-0.059	*CSF3R*	NA	TSS200	NA
cg26233331	4	6695614	7.60E-44	0.276	0.329	-0.052	*S100P*	NA	5UTR	Exon
cg25099490	14	23583133	7.70E-44	0.292	0.345	-0.053	NA	NA	NA	NA
cg21658670	19	41930894	7.77E-44	0.352	0.408	-0.056	*BCKDHA*	NA	3UTR	Exon
cg00066854	10	113987376	8.38E-44	0.306	0.364	-0.058	NA	NA	NA	NA
cg00071250	1	172628263	9.10E-44	0.770	0.718	0.052	*FASLG*	NA	5UTR	Exon
cg25705508	1	203199023	9.19E-44	0.360	0.418	-0.058	*CHIT1*	NA	TSS200	NA
cg16565031	14	70186289	1.18E-43	0.320	0.378	-0.057	NA	NA	NA	NA
cg10718056	6	28884599	1.18E-43	0.321	0.380	-0.058	*TRIM27*	NA	GeneBody	Intron
cg07863022	17	75315108	1.20E-43	0.229	0.295	-0.066	*SEPT9*	NA	5UTR	Intron
cg07564563	19	3548977	1.33E-43	0.403	0.468	-0.064	*MFSD12*	Shore	GeneBody	Intron
cg15878616	20	52492209	1.33E-43	0.403	0.489	-0.086	*SUMO1P1*	NA	ncRNA	Exon
cg07733719	7	75683547	1.41E-43	0.347	0.400	-0.053	*MDH2*	NA	GeneBody	Intron
cg03568673	13	20796216	1.42E-43	0.379	0.451	-0.072	*GJB6*	NA	3UTR	Exon
cg02806156	12	89728752	1.56E-43	0.358	0.438	-0.080	NA	NA	NA	NA
cg20977312	5	172748917	1.70E-43	0.459	0.539	-0.079	*STC2*	NA	GeneBody	Intron
cg15530560	19	851290	1.91E-43	0.409	0.465	-0.056	*ELANE*	Shelf	TSS1500	NA
cg10210690	1	55138059	2.06E-43	0.723	0.668	0.056	*HEATR8-TTC4*	NA	ncRNA	Intron
cg16651347	1	175042297	2.10E-43	0.314	0.372	-0.057	*TNN*	NA	5UTR	Intron
cg13178361	1	162532502	2.14E-43	0.402	0.467	-0.065	*UAP1*	Shore	5UTR	Intron
cg00828556	3	196351986	2.19E-43	0.295	0.368	-0.074	NA	NA	NA	NA
cg18542546	7	1982333	2.39E-43	0.420	0.509	-0.089	*MAD1L1*	Shelf	GeneBody	Intron
cg00980622	14	75884845	2.88E-43	0.413	0.504	-0.091	NA	NA	NA	NA
cg05642546	7	37298927	3.17E-43	0.323	0.394	-0.070	*ELMO1*	NA	GeneBody	Exon
cg18505752	6	32808752	3.58E-43	0.715	0.658	0.057	*PSMB8*	Shelf	GeneBody	Exon
cg06650246	1	206897270	3.91E-43	0.271	0.331	-0.060	*MAPKAPK2*	NA	GeneBody	Intron
cg13255542	11	134120785	4.32E-43	0.359	0.425	-0.066	*THYN1*	Shelf	GeneBody	Intron
cg14414154	21	27538021	5.28E-43	0.356	0.426	-0.070	*APP*	Shelf	GeneBody	Intron
cg05928849	11	122993208	6.03E-43	0.379	0.446	-0.067	*CLMP*	NA	GeneBody	Intron
cg16844053	7	148404978	6.09E-43	0.387	0.452	-0.065	*CUL1*	NA	5UTR	Intron
cg15033269	3	42631489	6.56E-43	0.359	0.428	-0.068	*SEC22C*	Shore	5UTR	Intron
cg14165142	20	3778655	6.58E-43	0.738	0.678	0.060	*CDC25B*	Shelf	GeneBody	Intron
cg03961551	1	25251730	7.60E-43	0.645	0.570	0.075	*RUNX3*	Shelf	GeneBody	Intron
cg14522803	11	818834	8.04E-43	0.355	0.408	-0.052	*PNPLA2*	Shore	TSS200	NA
cg21341487	20	60760919	8.67E-43	0.331	0.390	-0.060	*GTPBP5*	Shelf	5UTR	Intron
cg22528270	7	151505116	9.01E-43	0.279	0.356	-0.077	*PRKAG2*	NA	5UTR	Intron

cg00483217	2	25321917	1.20E-42	0.365	0.419	-0.054	*EFR3B*	NA	GeneBody	Intron
cg20609803	1	161184305	1.46E-42	0.320	0.378	-0.057	*FCER1G*	NA	TSS1500	NA
cg17164954	6	157345266	1.55E-42	0.281	0.350	-0.069	*ARID1B*	Shelf	GeneBody	Intron
cg20700740	1	9339683	1.56E-42	0.359	0.452	-0.093	NA	NA	NA	NA
cg11313468	19	41782183	1.58E-42	0.374	0.438	-0.064	*HNRNPUL1*	NA	GeneBody	Exon
cg10411221	6	32904317	1.67E-42	0.350	0.404	-0.054	*HLA-DMB*	NA	GeneBody	Intron
cg21221263	2	134933179	1.68E-42	0.324	0.388	-0.063	NA	NA	NA	NA
cg16449084	11	67069814	1.81E-42	0.314	0.371	-0.057	*SSH3*	Shore	TSS1500	Exon
cg27644327	6	90845852	1.96E-42	0.243	0.307	-0.064	*BACH2*	NA	5UTR	Intron
cg19510565	1	31217240	1.99E-42	0.713	0.659	0.054	*LAPTM5*	NA	GeneBody	Intron
cg05936004	3	130693828	2.08E-42	0.415	0.486	-0.071	*ATP2C1*	NA	GeneBody	Intron
cg18603538	7	127894591	2.08E-42	0.734	0.670	0.064	*LEP*	NA	GeneBody	Exon
cg25332499	11	64756382	2.55E-42	0.799	0.747	0.052	*BATF2*	NA	3UTR	Exon
cg16640599	4	119732131	2.94E-42	0.325	0.387	-0.061	*SEC24D*	NA	GeneBody	Intron
cg27260684	16	85063742	3.24E-42	0.359	0.427	-0.069	*KIAA0513*	Shore	5UTR	Intron
cg07917901	14	35870184	3.42E-42	0.330	0.400	-0.071	NA	Shelf	NA	NA
cg06030535	5	158637684	3.48E-42	0.301	0.365	-0.064	*RNF145*	Shore	TSS1500	NA
cg11254053	1	33497125	3.61E-42	0.360	0.427	-0.067	*AK2*	NA	GeneBody	Intron
cg05296619	13	114834683	4.03E-42	0.377	0.438	-0.061	*RASA3*	Shore	GeneBody	Intron
cg04855678	3	195946921	4.14E-42	0.272	0.331	-0.059	*SLC51A*	NA	GeneBody	Intron
cg03998264	12	53932355	4.53E-42	0.290	0.355	-0.065	*ATF7*	NA	GeneBody	Intron
cg08180028	15	41796044	5.68E-42	0.382	0.432	-0.050	*LTK*	Shore	3UTR	Exon
cg10718809	11	64087106	7.19E-42	0.319	0.378	-0.058	*PRDX5*	Shore	GeneBody	Intron
cg11767757	21	40145404	7.34E-42	0.722	0.658	0.064	NA	NA	NA	NA
cg04686354	17	73261880	8.99E-42	0.413	0.471	-0.058	*MRPS7*	Shelf	GeneBody	Exon
cg23670353	1	59833489	9.53E-42	0.306	0.376	-0.070	*FGGY*	NA	GeneBody	Intron
cg25505880	11	62574338	1.11E-41	0.306	0.361	-0.054	*STX5*	Shore	3UTR	Exon
cg23954655	13	99223562	1.29E-41	0.541	0.480	0.061	*STK24*	NA	GeneBody	Intron
cg05025071	19	6887530	1.52E-41	0.349	0.432	-0.083	*EMR1*	NA	TSS200	NA
cg04071967	17	53511321	1.58E-41	0.314	0.371	-0.057	NA	NA	NA	NA
cg17369406	21	34918578	1.62E-41	0.299	0.363	-0.064	*SON*	Shelf	GeneBody	Exon
cg13753351	9	127134207	1.64E-41	0.347	0.419	-0.072	*PSMB7*	NA	GeneBody	Intron
cg24941342	11	95093809	2.63E-41	0.305	0.370	-0.065	NA	NA	NA	NA
cg23648239	14	52701606	2.85E-41	0.361	0.430	-0.069	NA	NA	NA	NA
cg03431524	7	100142441	3.03E-41	0.716	0.658	0.058	*AGFG2*	NA	GeneBody	Intron
cg01268901	12	1744957	3.27E-41	0.321	0.379	-0.058	*WNT5B*	Shelf	GeneBody	Intron
cg04919592	7	2607232	3.34E-41	0.382	0.444	-0.061	*IQCE*	NA	GeneBody	Intron
cg02021919	5	86707074	3.65E-41	0.439	0.518	-0.079	*CCNH*	Shore	GeneBody	Exon
cg22526531	1	35913782	4.17E-41	0.469	0.545	-0.076	*KIAA0319L*	NA	GeneBody	Exon
cg20477259	6	31544960	4.42E-41	0.752	0.683	0.068	*TNF*	Shelf	GeneBody	Exon
cg25025545	6	14136628	4.50E-41	0.321	0.384	-0.063	*CD83*	NA	3UTR	Exon
cg07227049	2	58335008	4.68E-41	0.451	0.535	-0.083	*VRK2*	NA	ncRNA	Intron
cg00030432	7	100028974	4.89E-41	0.382	0.444	-0.062	*MEPCE*	Shore	5UTR	Exon
cg19620994	1	12774904	5.31E-41	0.357	0.431	-0.074	*AADACL3*	NA	TSS1500	NA
cg08843850	1	199092823	5.69E-41	0.322	0.393	-0.071	NA	NA	NA	NA
cg20636248	10	121439845	5.81E-41	0.344	0.404	-0.060	NA	NA	NA	NA
cg03463948	11	122612858	6.80E-41	0.399	0.484	-0.085	*UBASH3B*	NA	GeneBody	Intron
cg26162326	7	75957061	7.35E-41	0.364	0.433	-0.069	*YWHAG*	Shore	3UTR	Exon
cg27485921	2	46747379	7.93E-41	0.295	0.347	-0.053	*ATP6V1E2*	NA	TSS1500	NA
cg06070324	15	75322058	1.04E-40	0.271	0.324	-0.053	*PPCDC*	NA	GeneBody	Intron
cg00259404	6	28885568	1.06E-40	0.274	0.341	-0.067	*TRIM27*	NA	GeneBody	Intron
cg11151395	17	56355299	1.20E-40	0.442	0.518	-0.076	*MPO*	CpGIsland	GeneBody	Exon
cg01591037	12	15134481	1.25E-40	0.371	0.450	-0.080	*PDE6H*	NA	3UTR	Exon
cg12182708	10	99223744	1.29E-40	0.333	0.398	-0.066	*MMS19*	NA	GeneBody	Exon
cg09166556	1	156724277	1.33E-40	0.552	0.652	-0.100	NA	Shelf	NA	NA
cg21893651	6	30001368	1.36E-40	0.363	0.437	-0.074	*ZNRD1-AS1*	NA	ncRNA	Intron
cg04046364	12	58210661	1.41E-40	0.296	0.358	-0.062	*AVIL*	NA	TSS1500	NA
cg16452651	21	35016873	1.46E-40	0.365	0.429	-0.064	*ITSN1*	Shore	5UTR	Intron
cg19244300	1	110113304	1.52E-40	0.326	0.385	-0.059	*GNAI3*	NA	GeneBody	Intron
cg16625218	8	30272502	1.65E-40	0.737	0.676	0.062	*RBPMS*	NA	GeneBody	Intron
cg05830425	13	21654356	2.07E-40	0.411	0.487	-0.075	NA	NA	NA	NA
cg18095675	3	197272311	2.11E-40	0.529	0.605	-0.076	*BDH1*	NA	GeneBody	Intron
cg15125868	21	35746798	2.32E-40	0.299	0.355	-0.056	*FAM165B*	Shore	TSS1500	NA
cg16684117	5	148808456	2.34E-40	0.248	0.300	-0.052	*MIR143HG*	NA	ncRNA	Exon
cg24637417	12	51636921	2.37E-40	0.286	0.347	-0.060	*DAZAP2*	Shelf	3UTR	Exon
cg19460836	17	79047872	2.39E-40	0.437	0.530	-0.093	*BAIAP2*	Shelf	GeneBody	Intron
cg08850243	6	30169768	2.70E-40	0.262	0.325	-0.062	*TRIM26*	NA	5UTR	Intron
cg18325315	7	73897230	2.89E-40	0.427	0.507	-0.081	*GTF2IRD1*	Shelf	5UTR	Intron
cg03278564	13	49200201	3.17E-40	0.372	0.445	-0.073	NA	NA	NA	NA
cg16256243	2	85648512	3.23E-40	0.381	0.441	-0.061	NA	Shelf	NA	NA
cg13916080	9	129624994	3.23E-40	0.247	0.304	-0.056	*ZBTB34*	Shore	5UTR	Intron
cg26701826	4	108814604	3.26E-40	0.314	0.373	-0.059	*SGMS2*	NA	5UTR	Intron
cg25771026	3	196352027	3.76E-40	0.361	0.423	-0.062	NA	NA	NA	NA
cg09572125	6	33400477	3.90E-40	0.211	0.291	-0.079	*SYNGAP1*	NA	GeneBody	Exon
cg02041484	7	129266958	3.90E-40	0.308	0.377	-0.069	*NRF1*	NA	5UTR	Intron
cg13599613	9	130524902	4.03E-40	0.439	0.527	-0.088	*SH2D3C*	NA	GeneBody	Intron
cg13536447	17	36188913	4.07E-40	0.322	0.401	-0.079	NA	NA	NA	NA
cg07110356	17	56355431	4.14E-40	0.423	0.515	-0.092	*MPO*	CpGIsland	GeneBody	Exon

cg18915856	14	21483641	4.41E-40	0.333	0.387	-0.053	NA	NA	NA	NA
cg15412772	17	38175389	4.70E-40	0.468	0.528	-0.060	*MED24*	NA	ncRNA	Exon
cg10981439	6	41254433	4.78E-40	0.363	0.435	-0.072	*TREM1*	NA	5UTR	Exon
cg14417099	6	150073879	5.01E-40	0.331	0.401	-0.070	*PCMT1*	Shelf	GeneBody	Intron
cg13603332	9	108053809	5.19E-40	0.321	0.391	-0.070	*SLC44A1*	NA	GeneBody	Intron
cg09255910	1	221055790	5.24E-40	0.394	0.451	-0.057	*HLX*	Shore	GeneBody	Intron
cg23797615	11	67132472	5.37E-40	0.702	0.651	0.050	*CLCF1*	NA	3UTR	Exon
cg24406240	11	88153520	5.55E-40	0.373	0.445	-0.072	NA	NA	NA	NA
cg19240569	11	63331821	5.77E-40	0.748	0.695	0.053	*HRASLS2*	NA	TSS1500	NA
cg00705730	2	106438120	6.03E-40	0.477	0.564	-0.086	*NCK2*	NA	5UTR	Intron
cg22535089	13	114184462	6.09E-40	0.351	0.419	-0.068	*TMCO3*	Shore	GeneBody	Intron
cg14654385	11	63973006	6.67E-40	0.350	0.404	-0.054	*FERMT3*	Shore	TSS1500	NA
cg00610577	12	12008666	7.10E-40	0.178	0.231	-0.053	*RNU6-19*	NA	ncRNA	Intron
cg24608504	2	54760330	8.83E-40	0.395	0.466	-0.071	*SPTBN1*	NA	GeneBody	Intron
cg08363067	16	16170085	9.55E-40	0.315	0.367	-0.052	*ABCC1*	NA	GeneBody	Intron
cg12196294	3	129575053	9.93E-40	0.363	0.443	-0.079	*TMCC1*	NA	5UTR	Intron
cg14268557	6	28874547	1.08E-39	0.372	0.452	-0.080	*TRIM27*	NA	GeneBody	Intron
cg15690542	1	151172905	1.09E-39	0.739	0.686	0.053	*PIP5K1A*	Shore	GeneBody	Intron
cg07033722	1	40539032	1.10E-39	0.375	0.447	-0.073	*PPT1*	NA	3UTR	Exon
cg17313269	6	25965330	1.22E-39	0.374	0.455	-0.080	*TRIM38*	NA	5UTR	Intron
cg24504349	4	120974344	1.35E-39	0.335	0.403	-0.068	NA	NA	NA	NA
cg24548817	21	45774294	1.39E-39	0.368	0.441	-0.073	*TRPM2*	NA	GeneBody	Intron
cg23312431	21	45773997	1.41E-39	0.342	0.404	-0.061	*TRPM2*	NA	GeneBody	Intron
cg18463686	7	141646690	1.41E-39	0.343	0.418	-0.075	*CLEC5A*	NA	5UTR	Exon
cg08796342	14	92334029	1.45E-39	0.708	0.653	0.055	*TC2N*	NA	TSS200	NA
cg02052762	17	4090525	1.64E-39	0.399	0.479	-0.081	*ANKFY1*	NA	GeneBody	Intron
cg00168694	21	40193056	2.21E-39	0.338	0.411	-0.073	*ETS2*	Shore	GeneBody	Intron
cg02927682	1	54844424	2.28E-39	0.319	0.400	-0.082	*SSBP3*	NA	GeneBody	Intron
cg06684088	7	119131	2.29E-39	0.312	0.388	-0.076	NA	Shore	NA	NA
cg23254569	6	35451487	2.30E-39	0.360	0.430	-0.070	*TEAD3*	Shelf	GeneBody	Intron
cg13709496	9	101011718	2.44E-39	0.317	0.373	-0.056	*TBC1D2*	NA	GeneBody	Intron
cg17479280	1	156466088	2.46E-39	0.745	0.689	0.056	*MEF2D*	Shelf	5UTR	Intron
cg16391678	16	30485597	2.47E-39	0.787	0.722	0.065	*ITGAL*	CpGIsland	GeneBody	Exon
cg05000446	9	35042395	2.55E-39	0.423	0.510	-0.086	*C9orf131*	NA	GeneBody	Intron
cg18554789	7	139432178	2.90E-39	0.264	0.322	-0.058	*HIPK2*	NA	GeneBody	Intron
cg05919238	17	79244158	3.01E-39	0.546	0.623	-0.077	*SLC38A10*	NA	GeneBody	Intron
cg08947915	4	1742463	3.65E-39	0.310	0.382	-0.072	*TACC3*	NA	GeneBody	Intron
cg19513582	7	129481640	3.80E-39	0.465	0.536	-0.071	*UBE2H*	NA	GeneBody	Intron
cg24501381	1	43001072	3.86E-39	0.421	0.501	-0.081	*CCDC30*	NA	5UTR	Intron
cg13086983	1	21664810	3.88E-39	0.310	0.364	-0.054	*ECE1*	NA	GeneBody	Intron
cg03837680	17	77967529	4.00E-39	0.251	0.306	-0.054	*TBC1D16*	Shore	GeneBody	Intron
cg00907842	5	39219245	6.14E-39	0.256	0.320	-0.064	*FYB*	NA	5UTR	Intron
cg25853622	3	188425256	6.44E-39	0.312	0.377	-0.065	*LPP*	NA	GeneBody	Intron
cg11464160	2	131870398	6.64E-39	0.307	0.372	-0.065	*PLEKHB2*	NA	5UTR	Intron
cg06885823	5	82675005	6.75E-39	0.376	0.443	-0.067	NA	NA	NA	NA
cg13618969	9	129184186	8.13E-39	0.494	0.584	-0.090	*FAM125B*	NA	GeneBody	Exon
cg05008854	10	7825899	8.44E-39	0.380	0.433	-0.054	*KIN*	Shelf	ncRNA	Intron
cg04993279	1	8940460	8.95E-39	0.333	0.392	-0.058	*ENO1*	Shore	TSS1500	NA
cg19081101	1	203156625	9.72E-39	0.354	0.436	-0.082	*CHI3L1*	NA	TSS1500	NA
cg16125725	15	70101302	9.72E-39	0.411	0.501	-0.091	NA	NA	NA	NA
cg26443127	10	31987230	1.00E-38	0.362	0.418	-0.056	NA	NA	NA	NA
cg26514623	6	13295561	1.11E-38	0.448	0.529	-0.081	*LOC100130357*	NA	5UTR	Exon
cg04425551	6	30297338	1.13E-38	0.435	0.504	-0.069	*TRIM39*	Shelf	FirstExon	Exon
cg14520947	1	225942842	1.22E-38	0.389	0.473	-0.084	NA	NA	NA	NA
cg24504014	11	110297121	1.29E-38	0.461	0.526	-0.065	NA	Shelf	NA	NA
cg01924292	12	1815334	1.42E-38	0.386	0.462	-0.075	*ADIPOR2*	NA	5UTR	Intron
cg26535158	17	40175841	1.59E-38	0.315	0.371	-0.056	*NKIRAS2*	Shelf	GeneBody	Exon
cg17344091	6	28885444	1.71E-38	0.261	0.324	-0.063	*TRIM27*	NA	GeneBody	Intron
cg14028598	1	171714300	1.87E-38	0.465	0.548	-0.082	NA	Shelf	NA	NA
cg07052231	12	7363540	1.96E-38	0.428	0.510	-0.082	*PEX5*	NA	3UTR	Exon
cg26538140	12	6996791	2.03E-38	0.300	0.362	-0.062	NA	Shelf	NA	NA
cg12940181	2	145353012	2.04E-38	0.281	0.345	-0.065	NA	NA	NA	NA
cg12058064	15	86315201	2.12E-38	0.369	0.426	-0.057	*KLHL25*	Shelf	5UTR	Intron
cg21232161	2	135011626	2.25E-38	0.295	0.349	-0.054	*MGAT5*	NA	TSS1500	NA
cg21190228	2	240132342	2.33E-38	0.307	0.365	-0.057	*HDAC4*	NA	GeneBody	Intron
cg26313511	3	125053815	2.40E-38	0.330	0.383	-0.053	*ZNF148*	NA	5UTR	Intron
cg08764162	10	31147088	2.58E-38	0.365	0.431	-0.066	*ZNF438*	NA	GeneBody	Intron
cg27583010	16	30198505	2.67E-38	0.622	0.538	0.084	*CORO1A*	Shelf	GeneBody	Exon
cg21328082	6	41254471	2.76E-38	0.324	0.393	-0.069	*TREM1*	NA	TSS200	NA
cg26381210	10	88632654	3.04E-38	0.290	0.361	-0.071	*BMPR1A*	NA	5UTR	Intron
cg20090290	11	46543695	3.07E-38	0.352	0.425	-0.073	*AMBRA1*	NA	GeneBody	Intron
cg02240622	15	40601467	3.11E-38	0.337	0.391	-0.053	*PLCB2*	NA	TSS1500	NA
cg01580228	11	93467095	3.28E-38	0.316	0.380	-0.064	NA	NA	NA	NA
cg07165260	16	85062881	3.33E-38	0.734	0.678	0.056	*KIAA0513*	Shore	5UTR	Intron
cg23828876	3	196001706	3.35E-38	0.801	0.747	0.054	*PCYT1A*	NA	5UTR	Intron
cg23745290	4	106392021	3.62E-38	0.398	0.480	-0.082	*PPA2*	Shelf	GeneBody	Intron
cg26776551	13	51944507	3.69E-38	0.293	0.348	-0.054	*INTS6*	NA	GeneBody	Intron
cg20055861	15	68055293	3.71E-38	0.772	0.722	0.050	*MAP2K5*	NA	GeneBody	Intron

cg12832726	11	68096165	3.88E-38	0.190	0.252	-0.062	*LRP5*	NA	GeneBody	Intron
cg21854332	1	144989624	3.91E-38	0.270	0.322	-0.052	*PDE4DIP*	NA	GeneBody	Intron
cg13999433	9	117156883	3.95E-38	0.380	0.451	-0.071	*AKNA*	Shelf	TSS200	NA
cg17833106	1	161060000	4.06E-38	0.369	0.420	-0.051	*PVRL4*	NA	TSS1500	NA
cg21519701	17	62252524	4.45E-38	0.312	0.376	-0.064	*TEX2*	NA	GeneBody	Intron
cg21882356	14	21153451	4.70E-38	0.400	0.453	-0.053	*ANG*	Shelf	5UTR	Intron
cg20805133	2	242802192	4.78E-38	0.706	0.639	0.067	*PDCD1*	Shelf	TSS1500	NA
cg24484138	20	31070190	4.82E-38	0.328	0.398	-0.070	*C20orf112*	CpGIsland	5UTR	Intron
cg02796279	2	208101334	5.13E-38	0.351	0.423	-0.072	NA	NA	NA	NA
cg12809098	8	142165310	5.50E-38	0.500	0.584	-0.084	*DENND3*	NA	GeneBody	Intron
cg01944288	9	135036217	5.60E-38	0.227	0.284	-0.057	*NTNG2*	Shelf	TSS1500	NA
cg09342997	7	129339065	5.72E-38	0.419	0.507	-0.088	*NRF1*	NA	GeneBody	Intron
cg21991396	1	247581417	6.02E-38	0.396	0.464	-0.068	*NLRP3*	NA	5UTR	Exon
cg08428292	16	85981373	6.08E-38	0.597	0.524	0.072	NA	Shore	NA	NA
cg17322655	2	242802127	6.50E-38	0.691	0.634	0.056	*PDCD1*	Shelf	TSS1500	NA
cg21685770	12	10324918	6.90E-38	0.403	0.481	-0.078	*OLR1*	NA	TSS200	NA
cg21638533	12	6658625	6.97E-38	0.269	0.336	-0.067	*IFFO1*	Shore	ncRNA	Intron
cg14134128	17	76774832	8.03E-38	0.733	0.679	0.054	*CYTH1*	Shelf	GeneBody	Intron
cg00446123	20	62367888	8.09E-38	0.656	0.563	0.092	*LIME1*	Shore	TSS200	NA
cg11327408	6	30297329	8.14E-38	0.433	0.506	-0.073	*TRIM39*	Shelf	FirstExon	Exon
cg07702424	11	12306515	1.04E-37	0.484	0.553	-0.069	NA	NA	NA	NA
cg12299361	1	53796769	1.31E-37	0.328	0.395	-0.067	NA	Shelf	NA	NA
cg23338668	8	74240259	1.31E-37	0.466	0.556	-0.090	NA	NA	NA	NA
cg04552418	1	31958245	1.41E-37	0.353	0.430	-0.077	NA	NA	NA	NA
cg09303642	12	54690818	1.43E-37	0.339	0.400	-0.061	*NFE2*	NA	5UTR	Intron
cg03340036	4	89446409	1.54E-37	0.448	0.538	-0.090	*PIGY*	Shore	TSS1500	NA
cg17496887	1	153387707	1.57E-37	0.436	0.490	-0.054	*S100A7A*	NA	TSS1500	NA
cg18446110	5	138714654	1.66E-37	0.340	0.406	-0.066	*SLC23A1*	Shore	GeneBody	Intron
cg04460364	17	8370017	1.69E-37	0.417	0.486	-0.068	*NDEL1*	NA	GeneBody	Intron
cg06958535	1	203734478	1.81E-37	0.733	0.677	0.056	*LAX1*	NA	5UTR	Exon
cg14397918	9	72078829	1.82E-37	0.312	0.369	-0.057	*APBA1*	NA	GeneBody	Intron
cg16558770	15	90548037	2.06E-37	0.322	0.390	-0.068	*ZNF710*	Shelf	5UTR	Intron
cg19635401	6	118873071	2.07E-37	0.405	0.490	-0.085	*CEP85L*	NA	GeneBody	Intron
cg14021478	6	33384537	2.10E-37	0.652	0.596	0.056	*CUTA*	Shore	GeneBody	Exon
cg09635667	17	2300514	2.11E-37	0.327	0.382	-0.055	*MNT*	Shelf	GeneBody	Intron
cg17837191	17	62318673	2.13E-37	0.379	0.448	-0.069	*TEX2*	NA	5UTR	Intron
cg01479396	17	7529693	2.36E-37	0.253	0.313	-0.060	*SAT2*	Shore	3UTR	Exon
cg02147126	19	827715	2.75E-37	0.363	0.422	-0.059	*AZU1*	NA	TSS200	NA
cg12929983	15	40399004	2.87E-37	0.294	0.357	-0.063	*BMF*	Shore	TSS1500	Intron
cg14057303	9	109683834	3.17E-37	0.717	0.667	0.050	*ZNF462*	NA	5UTR	Intron
cg10168457	17	79882876	3.61E-37	0.246	0.296	-0.051	*MAFG*	Shore	5UTR	Intron
cg01697902	14	25046117	3.64E-37	0.341	0.408	-0.067	*CTSG*	NA	TSS1500	NA
cg02856190	11	94282491	3.71E-37	0.317	0.374	-0.056	*FUT4*	Shelf	3UTR	Exon
cg09145126	13	50570018	3.81E-37	0.375	0.447	-0.072	*DLEU2*	Shore	ncRNA	Intron
cg03637218	5	115209107	3.86E-37	0.442	0.533	-0.091	*AP3S1*	NA	GeneBody	Intron
cg01558916	19	11804727	4.18E-37	0.331	0.396	-0.066	NA	Shore	NA	NA
cg21163717	8	21769903	4.19E-37	0.694	0.619	0.075	*DOK2*	CpGIsland	GeneBody	Exon
cg05436845	11	65378622	4.74E-37	0.330	0.386	-0.055	*MAP3K11*	Shelf	GeneBody	Intron
cg13770399	6	64281639	5.32E-37	0.236	0.313	-0.076	*PTP4A1*	Shore	TSS1500	NA
cg03363565	16	474528	5.34E-37	0.330	0.397	-0.067	*RAB11FIP3*	Shore	TSS1500	NA
cg06257058	7	99683264	5.93E-37	0.350	0.415	-0.065	NA	Shelf	NA	NA
cg08130912	14	64687388	6.57E-37	0.235	0.293	-0.058	*SYNE2*	NA	GeneBody	Intron
cg26942829	6	13408158	7.30E-37	0.387	0.464	-0.077	*GFOD1*	NA	5UTR	Intron
cg12866960	12	66635398	7.35E-37	0.389	0.466	-0.077	*IRAK3*	NA	GeneBody	Intron
cg11283860	1	8273352	8.21E-37	0.326	0.388	-0.063	NA	Shelf	NA	NA
cg10075819	2	109229337	8.82E-37	0.774	0.723	0.052	*LIMS1*	NA	5UTR	Intron
cg11703212	13	114271629	9.24E-37	0.430	0.510	-0.080	*TFDP1*	NA	GeneBody	Intron
cg15580458	10	121155124	9.37E-37	0.324	0.378	-0.054	*GRK5*	NA	GeneBody	Intron
cg19573490	17	79870317	9.89E-37	0.333	0.398	-0.065	*PCYT2*	Shore	TSS1500	Exon
cg26382697	11	2406712	1.01E-36	0.816	0.761	0.055	*CD81*	CpGIsland	GeneBody	Intron
cg22491058	1	207277466	1.16E-36	0.306	0.368	-0.062	*C4BPA*	NA	TSS200	NA
cg17090611	8	17017866	1.27E-36	0.432	0.521	-0.089	*ZDHHC2*	Shelf	GeneBody	Intron
cg11119767	2	174024669	1.28E-36	0.334	0.398	-0.064	*ZAK*	NA	GeneBody	Intron
cg04756491	14	23385384	1.40E-36	0.350	0.433	-0.083	*RBM23*	Shelf	5UTR	Intron
cg04304036	16	68334619	1.44E-36	0.332	0.398	-0.065	*SLC7A6OS*	NA	3UTR	Exon
cg18382353	10	45495981	1.50E-36	0.204	0.261	-0.057	*ZNF22*	Shore	TSS1500	Exon
cg22666015	2	233981885	1.58E-36	0.331	0.411	-0.080	*INPP5D*	NA	GeneBody	Intron
cg24079727	15	57317980	1.58E-36	0.348	0.410	-0.062	*TCF12*	NA	GeneBody	Intron
cg25103337	1	9293583	1.60E-36	0.347	0.411	-0.065	*H6PD*	Shore	TSS1500	NA
cg01565508	17	8869961	1.66E-36	0.367	0.435	-0.068	*PIK3R5*	Shore	TSS1500	NA
cg23363263	2	224751989	1.70E-36	0.461	0.547	-0.086	*WDFY1*	NA	GeneBody	Intron
cg21602257	20	44538675	1.74E-36	0.351	0.409	-0.058	*PLTP*	Shore	GeneBody	Intron
cg02308232	17	7742762	1.94E-36	0.361	0.428	-0.067	*KDM6B*	Shelf	TSS1500	NA
cg06749872	16	30198509	2.01E-36	0.649	0.593	0.056	*CORO1A*	Shelf	GeneBody	Exon
cg19023589	13	114182159	2.03E-36	0.470	0.551	-0.081	*TMCO3*	Shelf	GeneBody	Intron
cg19619956	5	176967557	2.05E-36	0.442	0.508	-0.066	*FAM193B*	NA	ncRNA	Intron
cg02607972	2	25964061	2.13E-36	0.396	0.464	-0.069	*ASXL2*	NA	3UTR	Exon
cg08472008	4	3531603	2.15E-36	0.287	0.351	-0.064	*LRPAP1*	Shore	GeneBody	Intron

cg15075988	10	76974078	2.19E-36	0.340	0.413	-0.073	*VDAC2*	Shelf	ncRNA	Intron
cg18659081	22	44588350	2.29E-36	0.350	0.416	-0.066	*PARVG*	NA	GeneBody	Intron
cg01796438	3	11312864	2.64E-36	0.363	0.415	-0.053	*ATG7*	Shore	TSS1500	NA
cg01402255	1	153800699	3.01E-36	0.332	0.393	-0.061	*GATAD2B*	NA	FirstExon	Exon
cg21326139	4	1294783	3.19E-36	0.297	0.353	-0.056	*MAEA*	NA	GeneBody	Intron
cg25684151	8	126588554	3.21E-36	0.346	0.404	-0.058	NA	NA	NA	NA
cg03330678	17	75316233	3.56E-36	0.322	0.381	-0.059	*SEPT9*	NA	5UTR	Intron
cg12709880	18	21163172	3.74E-36	0.448	0.518	-0.071	*NPC1*	Shelf	GeneBody	Intron
cg17250262	2	30457110	4.43E-36	0.605	0.554	0.050	*LBH*	Shore	GeneBody	Intron
cg01763719	2	239463774	4.60E-36	0.366	0.452	-0.085	*LOC151171*	NA	ncRNA	Exon
cg05132828	12	123560479	4.62E-36	0.653	0.601	0.052	*PITPNM2*	NA	5UTR	Intron
cg09467433	3	171283606	4.77E-36	0.375	0.451	-0.076	NA	NA	NA	NA
cg26434370	16	10909361	5.12E-36	0.362	0.420	-0.058	*FAM18A*	Shelf	GeneBody	Intron
cg07302959	7	92198639	5.14E-36	0.286	0.349	-0.063	*FAM133DP*	NA	ncRNA	Intron
cg10408731	7	65214843	5.29E-36	0.419	0.511	-0.092	*LOC441242*	Shore	ncRNA	Intron
cg02863947	3	119499190	5.30E-36	0.336	0.393	-0.056	*NR1I2*	NA	TSS200	NA
cg13047308	11	47471339	5.58E-36	0.516	0.580	-0.065	*RAPSN*	NA	TSS1500	NA
cg18146927	1	200835888	5.60E-36	0.743	0.693	0.050	NA	NA	NA	NA
cg04213390	2	85743934	5.86E-36	0.388	0.463	-0.075	NA	NA	NA	NA
cg15545247	12	123201372	6.57E-36	0.377	0.440	-0.064	*HCAR3*	NA	5UTR	Exon
cg23819411	3	182929273	7.10E-36	0.312	0.392	-0.081	*MCF2L2*	NA	GeneBody	Intron
cg24777950	14	25046121	7.37E-36	0.313	0.382	-0.069	*CTSG*	NA	TSS1500	NA
cg21536783	1	59041407	7.52E-36	0.368	0.419	-0.052	*TACSTD2*	Shore	3UTR	Exon
cg23114435	1	167442612	7.63E-36	0.692	0.637	0.055	*CD247*	NA	GeneBody	Intron
cg24686551	3	134047274	7.68E-36	0.326	0.401	-0.074	NA	NA	NA	NA
cg03621504	12	116571240	7.79E-36	0.257	0.318	-0.061	*MED13L*	NA	GeneBody	Intron
cg14543285	14	103129037	8.38E-36	0.427	0.490	-0.063	*RCOR1*	NA	GeneBody	Intron
cg05991009	2	102080674	8.40E-36	0.347	0.412	-0.064	*RFX8*	NA	GeneBody	Intron
cg15135286	2	33359281	8.98E-36	0.376	0.436	-0.061	*LTBP1*	NA	TSS1500	Intron
cg27307183	6	4890095	1.06E-35	0.314	0.371	-0.057	*CDYL*	NA	GeneBody	Intron
cg12897164	8	124529273	1.09E-35	0.743	0.682	0.061	*FBXO32*	NA	GeneBody	Intron
cg23807570	12	110787107	1.21E-35	0.371	0.437	-0.066	*ATP2A2*	NA	3UTR	Exon
cg19717347	12	129304956	1.31E-35	0.379	0.445	-0.067	*SLC15A4*	Shelf	GeneBody	Intron
cg11884933	7	2774414	1.31E-35	0.297	0.369	-0.072	*GNA12*	Shore	GeneBody	Intron
cg12415479	3	129311698	1.38E-35	0.192	0.253	-0.061	*PLXND1*	NA	GeneBody	Intron
cg23447233	4	1295047	1.38E-35	0.247	0.305	-0.058	*MAEA*	NA	GeneBody	Intron
cg26692003	3	13063165	1.43E-35	0.852	0.795	0.057	*IQSEC1*	NA	GeneBody	Intron
cg15089806	1	43418199	1.46E-35	0.730	0.678	0.052	*SLC2A1*	NA	GeneBody	Intron
cg08703231	16	8738304	1.52E-35	0.343	0.397	-0.054	*METTL22*	NA	GeneBody	Intron
cg03547581	1	205684841	1.56E-35	0.247	0.312	-0.066	*NUCKS1*	NA	3UTR	Exon
cg25605731	19	13054434	1.59E-35	0.314	0.394	-0.080	*CALR*	Shelf	GeneBody	Exon
cg25084760	13	100886412	1.71E-35	0.383	0.455	-0.072	*PCCA*	NA	GeneBody	Intron
cg25006077	3	152176018	1.74E-35	0.488	0.560	-0.072	*MBNL1*	NA	GeneBody	Intron
cg16954385	7	23246896	1.76E-35	0.397	0.471	-0.074	NA	Shore	NA	NA
cg23314364	8	29230998	1.83E-35	0.308	0.377	-0.069	NA	NA	NA	NA
cg21492378	9	123850279	1.87E-35	0.388	0.465	-0.077	*CNTRL*	NA	TSS1500	NA
cg14369981	9	129294452	2.07E-35	0.356	0.417	-0.061	NA	NA	NA	NA
cg05954120	1	156254757	2.09E-35	0.334	0.389	-0.054	*TMEM79*	NA	5UTR	Intron
cg03778909	17	80833393	2.15E-35	0.348	0.406	-0.058	*TBCD*	Shelf	GeneBody	Intron
cg27230882	1	110976749	2.47E-35	0.335	0.394	-0.059	NA	NA	NA	NA
cg03370752	11	61136373	2.48E-35	0.503	0.572	-0.069	*TMEM138*	NA	3UTR	Exon
cg06526020	6	34308880	2.58E-35	0.447	0.533	-0.086	*RPS10-NUDT3*	NA	GeneBody	Intron
cg12517576	7	128048308	2.99E-35	0.276	0.330	-0.053	*IMPDH1*	Shelf	GeneBody	Intron
cg26831416	19	39142011	3.11E-35	0.298	0.353	-0.054	*ACTN4*	Shelf	GeneBody	Intron
cg13652008	2	33359356	3.29E-35	0.444	0.512	-0.068	*LTBP1*	NA	TSS1500	Intron
cg25647583	15	91427184	3.42E-35	0.353	0.404	-0.051	*FES*	Shelf	TSS1500	NA
cg03910874	6	209712	3.43E-35	0.677	0.619	0.058	NA	NA	NA	NA
cg23595413	11	76574308	3.44E-35	0.279	0.339	-0.059	*ACER3*	Shelf	GeneBody	Intron
cg01821022	12	42775908	3.62E-35	0.306	0.365	-0.059	*PPHLN1*	NA	GeneBody	Intron
cg14480116	2	65594890	3.88E-35	0.386	0.469	-0.083	*SPRED2*	NA	GeneBody	Intron
cg24663338	1	8005108	3.99E-35	0.379	0.456	-0.076	NA	Shelf	NA	NA
cg20312012	2	97331035	4.18E-35	0.366	0.439	-0.072	*FER1L5*	NA	GeneBody	Intron
cg25930786	19	54584636	4.31E-35	0.325	0.386	-0.061	*TARM1*	NA	TSS200	NA
cg17429236	7	150694028	4.35E-35	0.190	0.248	-0.059	*NOS3*	NA	GeneBody	Intron
cg11518509	1	114525428	4.80E-35	0.171	0.229	-0.058	NA	NA	NA	NA
cg13831136	6	30070342	5.64E-35	0.336	0.395	-0.060	NA	Shore	NA	NA
cg02399831	3	112113161	5.97E-35	0.372	0.447	-0.076	NA	NA	NA	NA
cg07481273	14	93653027	6.19E-35	0.399	0.466	-0.066	*TMEM251*	Shore	3UTR	Exon
cg14693090	12	11899281	6.29E-35	0.276	0.336	-0.060	*ETV6*	NA	GeneBody	Intron
cg08773226	6	135061398	6.69E-35	0.404	0.476	-0.072	NA	NA	NA	NA
cg16041412	19	2460037	6.71E-35	0.396	0.484	-0.087	NA	Shore	NA	NA
cg01440934	1	47051752	6.90E-35	0.376	0.445	-0.070	*MKNK1*	NA	ncRNA	Intron
cg17291166	6	37051189	7.74E-35	0.451	0.503	-0.052	NA	NA	NA	NA
cg16149628	11	1792574	8.03E-35	0.510	0.594	-0.084	NA	NA	NA	NA
cg19410609	2	31541350	8.18E-35	0.345	0.421	-0.076	NA	NA	NA	NA
cg24595152	3	43733831	8.25E-35	0.295	0.347	-0.052	*ABHD5*	Shore	GeneBody	Intron
cg09455881	3	47388736	8.34E-35	0.284	0.347	-0.063	NA	NA	NA	NA
cg07136111	7	30829005	8.38E-35	0.325	0.379	-0.053	*FAM188B*	NA	GeneBody	Intron

cg22957691	2	36790185	8.54E-35	0.376	0.455	-0.079	*FEZ2*	NA	GeneBody	Intron
cg04451770	10	97515372	9.15E-35	0.311	0.364	-0.052	*ENTPD1*	NA	TSS1500	Intron
cg19739596	11	59824161	9.20E-35	0.359	0.416	-0.057	*MS4A3*	NA	5UTR	Exon
cg23013137	14	24676542	9.42E-35	0.721	0.670	0.051	*TSSK4*	NA	GeneBody	Exon
cg24252148	11	68081257	9.88E-35	0.266	0.322	-0.056	*LRP5*	Shore	GeneBody	Intron
cg01850135	16	4516078	1.02E-34	0.758	0.696	0.062	*NMRAL1*	NA	GeneBody	Intron
cg21249659	12	10324843	1.03E-34	0.335	0.396	-0.061	*OLR1*	NA	TSS200	NA
cg02862467	1	19407897	1.04E-34	0.348	0.419	-0.071	*UBR4*	NA	GeneBody	Exon
cg15929395	11	117695828	1.16E-34	0.343	0.394	-0.051	*FXYD2*	NA	GeneBody	Intron
cg22318806	6	31540411	1.17E-34	0.632	0.579	0.053	*LTA*	NA	5UTR	Intron
cg08244301	19	17610751	1.19E-34	0.242	0.306	-0.063	*SLC27A1*	Shore	GeneBody	Intron
cg19320820	4	87966326	1.21E-34	0.229	0.281	-0.052	*AFF1*	NA	GeneBody	Intron
cg02435083	16	8943436	1.28E-34	0.293	0.347	-0.054	NA	NA	NA	NA
cg10264529	14	24562064	1.42E-34	0.435	0.487	-0.052	*PCK2*	Shore	TSS1500	NA
cg06068369	6	105837504	1.46E-34	0.416	0.473	-0.057	*PREP*	NA	GeneBody	Intron
cg17159187	8	144420118	1.58E-34	0.371	0.430	-0.059	*TOP1MT*	Shelf	5UTR	Intron
cg21285555	8	52771466	1.76E-34	0.380	0.443	-0.063	*PCMTD1*	NA	GeneBody	Intron
cg11389756	6	28875354	1.84E-34	0.317	0.387	-0.070	*TRIM27*	NA	GeneBody	Intron
cg15086439	1	236563070	1.88E-34	0.377	0.470	-0.094	*EDARADD*	Shelf	GeneBody	Intron
cg01923724	1	58864209	1.90E-34	0.398	0.453	-0.055	NA	NA	NA	NA
cg10826999	1	225862288	1.90E-34	0.349	0.416	-0.067	NA	Shelf	NA	NA
cg26336059	1	153958977	1.93E-34	0.232	0.290	-0.057	*RAB13*	NA	TSS200	NA
cg08458487	10	81709191	2.06E-34	0.290	0.342	-0.052	*SFTPD*	NA	TSS1500	NA
cg19676553	14	105751629	2.08E-34	0.386	0.440	-0.054	*BRF1*	NA	GeneBody	Intron
cg27051231	2	232213012	2.10E-34	0.338	0.399	-0.061	NA	NA	NA	NA
cg24211388	6	31582837	2.20E-34	0.331	0.393	-0.062	*AIF1*	NA	TSS200	NA
cg25437886	3	45677346	2.21E-34	0.401	0.476	-0.075	*LIMD1*	NA	GeneBody	Intron
cg05599723	1	12241073	2.31E-34	0.376	0.454	-0.078	*TNFRSF1B*	NA	GeneBody	Intron
cg05581469	12	49413435	2.36E-34	0.321	0.382	-0.061	*PRKAG1*	Shore	TSS1500	Exon
cg23384708	6	31544934	2.51E-34	0.718	0.665	0.053	*TNF*	Shelf	GeneBody	Exon
cg04118119	13	47371987	2.51E-34	0.388	0.455	-0.067	*ESD*	Shore	TSS1500	NA
cg03689552	16	3292971	2.53E-34	0.333	0.398	-0.065	*MEFV*	NA	3UTR	Exon
cg26062204	3	150661892	2.57E-34	0.843	0.793	0.050	*CLRN1*	NA	GeneBody	Intron
cg00992048	1	1695585	2.74E-34	0.336	0.388	-0.052	*NADK*	Shelf	GeneBody	Intron
cg06739107	3	188399479	2.84E-34	0.382	0.463	-0.081	*LPP*	NA	GeneBody	Intron
cg22111527	11	69260136	2.93E-34	0.297	0.364	-0.067	NA	Shore	NA	NA
cg06298740	1	227125826	3.08E-34	0.312	0.366	-0.054	NA	Shore	NA	NA
cg27193519	16	4714443	3.24E-34	0.275	0.343	-0.067	*MGRN1*	NA	GeneBody	Intron
cg06294475	11	2698623	3.29E-34	0.280	0.335	-0.055	*KCNQ1OT1*	NA	ncRNA	Exon
cg16268734	12	56690194	3.38E-34	0.395	0.462	-0.066	*CS*	Shelf	GeneBody	Intron
cg19869035	7	2653955	3.66E-34	0.350	0.414	-0.064	*IQCE*	NA	3UTR	Exon
cg26105956	1	154471433	3.68E-34	0.423	0.513	-0.090	*SHE*	Shelf	GeneBody	Intron
cg20794855	8	67365340	3.76E-34	0.391	0.453	-0.063	*ADHFE1*	NA	GeneBody	Intron
cg08300570	7	92353454	4.02E-34	0.370	0.436	-0.066	*CDK6*	NA	GeneBody	Intron
cg00483030	17	77810509	4.09E-34	0.305	0.358	-0.053	*CBX4*	Shore	GeneBody	Intron
cg20934416	5	17444401	4.26E-34	0.432	0.525	-0.093	NA	NA	NA	NA
cg26588076	17	17741631	4.34E-34	0.242	0.292	-0.050	*SREBF1*	Shore	TSS1500	NA
cg14885762	17	75446450	4.70E-34	0.768	0.717	0.050	*SEPT9*	Shore	GeneBody	Intron
cg16692439	22	26831446	5.03E-34	0.287	0.346	-0.059	*ASPHD2*	Shore	GeneBody	Intron
cg01861537	11	121507724	5.22E-34	0.380	0.457	-0.077	NA	NA	NA	NA
cg09727050	2	152214177	5.29E-34	0.349	0.417	-0.068	*TNFAIP6*	NA	5UTR	Exon
cg02111865	2	30371990	5.35E-34	0.384	0.465	-0.081	*YPEL5*	Shore	5UTR	Intron
cg06001524	17	9264709	5.97E-34	0.383	0.447	-0.064	*STX8*	NA	GeneBody	Intron
cg19157819	10	75405260	6.58E-34	0.278	0.333	-0.055	*SYNPO2L*	Shelf	3UTR	Exon
cg13474450	8	142222685	6.70E-34	0.453	0.529	-0.076	*SLC45A4*	Shore	GeneBody	Intron
cg03892838	2	238599734	6.83E-34	0.186	0.249	-0.064	*LRRFIP1*	Shore	TSS1500	Intron
cg25344401	7	4755415	7.03E-34	0.436	0.526	-0.090	*FOXK1*	Shelf	GeneBody	Intron
cg08173915	21	34774164	7.26E-34	0.377	0.447	-0.070	*IFNGR2*	Shore	TSS1500	NA
cg20429104	18	74114570	7.27E-34	0.275	0.346	-0.071	*ZNF516*	CpGIsland	GeneBody	Intron
cg07006935	7	55620641	7.31E-34	0.288	0.357	-0.068	*VOPP1*	NA	GeneBody	Intron
cg17554464	12	56864388	7.53E-34	0.382	0.455	-0.072	*SPRYD4*	NA	3UTR	Exon
cg12323063	17	67497879	7.81E-34	0.442	0.513	-0.070	*MAP2K6*	NA	GeneBody	Intron
cg02847588	1	8271997	7.86E-34	0.331	0.385	-0.054	NA	CpGIsland	NA	NA
cg08161480	1	226114565	8.11E-34	0.314	0.371	-0.057	NA	Shore	NA	NA
cg17846016	17	80181015	8.13E-34	0.375	0.453	-0.078	NA	Shore	NA	NA
cg00795812	2	242802009	8.22E-34	0.658	0.608	0.051	*PDCD1*	Shelf	TSS1500	NA
cg20847110	4	39482781	8.24E-34	0.322	0.379	-0.057	*LOC401127*	Shore	ncRNA	Exon
cg15527515	14	23630709	8.49E-34	0.303	0.367	-0.064	*SLC7A8*	NA	GeneBody	Intron
cg03308839	16	15797297	8.64E-34	0.384	0.459	-0.074	*NDE1*	NA	GeneBody	Intron
cg10611016	11	6225759	8.81E-34	0.410	0.487	-0.077	*C11orf42*	NA	TSS1500	NA
cg18571045	13	113238006	9.11E-34	0.282	0.341	-0.059	*TUBGCP3*	Shelf	GeneBody	Intron
cg10061770	16	68366844	9.51E-34	0.280	0.338	-0.058	*PRMT7*	NA	GeneBody	Intron
cg00405190	2	175545838	9.66E-34	0.332	0.398	-0.065	*WIPF1*	Shore	5UTR	Intron
cg22193912	17	79881523	9.87E-34	0.316	0.400	-0.084	*MAFG*	Shelf	5UTR	Intron
cg01360627	6	31544931	1.02E-33	0.762	0.699	0.062	*TNF*	Shelf	GeneBody	Exon
cg11153071	17	78748077	1.09E-33	0.453	0.541	-0.088	*RPTOR*	NA	GeneBody	Intron
cg06578434	19	1155225	1.12E-33	0.423	0.483	-0.060	*SBNO2*	Shelf	5UTR	Intron
cg16283183	3	45718805	1.13E-33	0.331	0.397	-0.066	*LIMD1*	NA	3UTR	Exon

cg24553417	13	50244794	1.14E-33	0.378	0.449	-0.071	*EBPL*	NA	GeneBody	Intron
cg13079571	6	30297257	1.27E-33	0.447	0.522	-0.075	*TRIM39*	Shelf	FirstExon	Exon
cg04747180	4	39116218	1.27E-33	0.351	0.421	-0.070	*KLHL5*	NA	GeneBody	Intron
cg27510066	8	19536244	1.31E-33	0.446	0.531	-0.085	*CSGALNACT1*	NA	ncRNA	Intron
cg11948905	5	53920730	1.32E-33	0.284	0.347	-0.063	NA	NA	NA	NA
cg10357682	20	3745817	1.32E-33	0.429	0.514	-0.085	*C20orf27*	Shelf	5UTR	Intron
cg24414325	12	56414442	1.39E-33	0.365	0.432	-0.067	*IKZF4*	NA	TSS1500	NA
cg18478531	7	74204775	1.43E-33	0.354	0.423	-0.069	NA	Shore	NA	NA
cg07496207	5	172280684	1.44E-33	0.381	0.446	-0.064	*ERGIC1*	NA	GeneBody	Intron
cg11690666	17	80415469	1.46E-33	0.475	0.540	-0.065	*NARF*	Shore	TSS1500	NA
cg09121543	17	61774794	1.51E-33	0.516	0.440	0.076	*LIMD2*	Shelf	3UTR	Exon
cg02770745	11	3926837	1.65E-33	0.368	0.446	-0.078	*STIM1*	NA	GeneBody	Intron
cg22652378	17	78533842	1.65E-33	0.416	0.471	-0.055	*RPTOR*	NA	GeneBody	Intron
cg04266202	17	56352895	1.69E-33	0.418	0.485	-0.067	*MPO*	Shelf	GeneBody	Intron
cg11638399	8	29441416	1.74E-33	0.330	0.392	-0.062	NA	NA	NA	NA
cg19030682	17	79218991	1.76E-33	0.819	0.761	0.057	*SLC38A10*	NA	3UTR	Exon
cg06519434	3	38664257	1.76E-33	0.748	0.697	0.051	*SCN5A*	NA	GeneBody	Intron
cg00382999	3	136649333	1.79E-33	0.343	0.407	-0.063	*NCK1*	NA	GeneBody	Intron
cg08368934	16	57701455	1.85E-33	0.359	0.410	-0.051	*GPR97*	NA	TSS1500	NA
cg02740606	11	67206418	1.86E-33	0.829	0.777	0.052	*PTPRCAP*	Shelf	TSS1500	Exon
cg04261496	7	4753002	1.90E-33	0.299	0.364	-0.065	*FOXK1*	NA	GeneBody	Intron
cg13984928	17	3704574	1.99E-33	0.383	0.478	-0.095	*ITGAE*	NA	TSS200	NA
cg13844341	7	2116368	2.08E-33	0.479	0.540	-0.061	*MAD1L1*	CpGIsland	GeneBody	Intron
cg27051683	2	242802069	2.27E-33	0.594	0.541	0.052	*PDCD1*	Shelf	TSS1500	NA
cg14210726	17	76136952	2.39E-33	0.356	0.409	-0.053	*TMC8*	CpGIsland	GeneBody	Exon
cg15457217	2	224780409	2.48E-33	0.505	0.575	-0.070	*WDFY1*	NA	GeneBody	Intron
cg17586302	6	144013969	2.54E-33	0.420	0.488	-0.068	*PHACTR2*	NA	GeneBody	Intron
cg15210526	1	167733310	2.59E-33	0.405	0.484	-0.079	*MPZL1*	NA	GeneBody	Intron
cg16350446	2	172338747	2.96E-33	0.314	0.377	-0.063	*DCAF17*	NA	ncRNA	Exon
cg14619064	17	56355331	3.28E-33	0.555	0.612	-0.057	*MPO*	CpGIsland	GeneBody	Exon
cg01543184	17	79881543	3.33E-33	0.356	0.435	-0.079	*MAFG*	Shelf	5UTR	Intron
cg23581252	6	32897912	3.43E-33	0.384	0.443	-0.059	NA	NA	NA	NA
cg15296767	11	64122743	3.60E-33	0.311	0.377	-0.065	*CCDC88B*	Shelf	GeneBody	Exon
cg02368508	16	12060182	3.74E-33	0.778	0.725	0.053	*TNFRSF17*	NA	GeneBody	Exon
cg04051365	3	193586394	3.83E-33	0.340	0.401	-0.060	NA	Shore	NA	NA
cg05971678	10	125770089	3.96E-33	0.398	0.478	-0.081	*CHST15*	NA	GeneBody	Intron
cg14315912	14	102677294	4.25E-33	0.484	0.539	-0.054	*WDR20*	Shelf	GeneBody	Intron
cg04225088	11	33278186	4.31E-33	0.410	0.487	-0.076	*HIPK3*	Shore	TSS1500	NA
cg08044454	7	37024552	4.38E-33	0.312	0.370	-0.058	*ELMO1*	NA	ncRNA	Intron
cg17446956	12	109235032	4.50E-33	0.455	0.519	-0.064	*SSH1*	NA	GeneBody	Intron
cg06655349	19	10332165	4.60E-33	0.377	0.450	-0.073	*S1PR2*	Shelf	3UTR	Exon
cg09046309	20	45947226	4.62E-33	0.349	0.418	-0.069	*ZMYND8*	NA	GeneBody	Intron
cg03316570	6	43877657	4.74E-33	0.737	0.682	0.055	*LOC100132354*	NA	ncRNA	Intron
cg13912964	7	100867281	4.82E-33	0.535	0.595	-0.060	*ZNHIT1*	NA	3UTR	Exon
cg09789874	12	125262045	4.97E-33	0.349	0.423	-0.074	NA	NA	NA	NA
cg11557901	9	128022358	5.32E-33	0.377	0.462	-0.085	NA	Shore	NA	NA
cg00759807	16	89390789	5.46E-33	0.729	0.667	0.062	*LOC100287036*	NA	GeneBody	Exon
cg13084677	4	39482740	5.85E-33	0.336	0.403	-0.067	*LOC401127*	Shore	ncRNA	Exon
cg01400750	4	145956168	6.11E-33	0.393	0.469	-0.076	*ANAPC10*	NA	GeneBody	Intron
cg23240927	11	1320497	7.09E-33	0.399	0.473	-0.074	*TOLLIP*	Shelf	GeneBody	Intron
cg17980508	1	79085713	7.15E-33	0.216	0.294	-0.078	*IFI44L*	NA	TSS1500	NA
cg13057663	8	37805275	7.54E-33	0.434	0.519	-0.085	NA	NA	NA	NA
cg21574244	15	42568411	7.69E-33	0.347	0.413	-0.067	*GANC*	Shelf	GeneBody	Intron
cg03926751	4	88140261	8.22E-33	0.315	0.382	-0.067	*KLHL8*	Shore	5UTR	Intron
cg12554857	4	95264019	8.62E-33	0.256	0.311	-0.055	*HPGDS*	NA	5UTR	Exon
cg19405329	15	49440632	9.31E-33	0.391	0.457	-0.066	*COPS2*	NA	GeneBody	Intron
cg26928972	3	122043799	9.43E-33	0.385	0.464	-0.079	*CSTA*	NA	TSS1500	NA
cg17959183	2	26297671	9.49E-33	0.409	0.486	-0.076	*RAB10*	NA	GeneBody	Intron
cg11361201	5	156690796	1.00E-32	0.252	0.303	-0.051	NA	Shelf	NA	NA
cg19975346	10	126366131	1.01E-32	0.426	0.480	-0.053	*FAM53B*	NA	GeneBody	Intron
cg03126694	13	49001104	1.02E-32	0.323	0.378	-0.056	*RB1*	NA	GeneBody	Intron
cg10471548	6	7403054	1.12E-32	0.358	0.426	-0.068	*RIOK1*	NA	5UTR	Exon
cg11098259	15	58430391	1.12E-32	0.286	0.345	-0.059	*AQP9*	NA	TSS200	NA
cg02262553	4	87849250	1.13E-32	0.315	0.380	-0.065	*LOC100506746*	NA	ncRNA	Intron
cg26508200	12	109235071	1.14E-32	0.458	0.531	-0.073	*SSH1*	NA	GeneBody	Intron
cg01374870	6	32905127	1.23E-32	0.328	0.390	-0.062	*HLA-DMB*	NA	GeneBody	Exon
cg27176729	1	181056650	1.25E-32	0.330	0.409	-0.079	*IER5*	Shore	TSS1500	NA
cg00765705	12	124865130	1.44E-32	0.292	0.366	-0.073	*NCOR2*	NA	GeneBody	Intron
cg23353000	17	42120896	1.47E-32	0.319	0.382	-0.063	*LSM12*	NA	GeneBody	Intron
cg09800500	12	24992256	1.59E-32	0.828	0.776	0.052	*BCAT1*	NA	GeneBody	Intron
cg26930596	1	2082315	1.60E-32	0.424	0.511	-0.088	*PRKCZ*	CpGIsland	GeneBody	Exon
cg04042333	17	1104665	1.65E-32	0.768	0.714	0.054	NA	Shelf	NA	NA
cg04739200	6	135517046	1.66E-32	0.404	0.481	-0.077	*MYB*	NA	GeneBody	Exon
cg26217402	14	74238381	1.78E-32	0.639	0.583	0.055	*C14orf43*	NA	5UTR	Intron
cg05656688	1	25254088	1.81E-32	0.723	0.662	0.061	*RUNX3*	Shore	GeneBody	Exon
cg24597363	4	1294996	1.97E-32	0.393	0.469	-0.077	*MAEA*	NA	GeneBody	Intron
cg17734802	1	54358862	2.01E-32	0.342	0.403	-0.061	*DIO1*	Shelf	TSS1500	NA
cg15739581	2	166626783	2.08E-32	0.394	0.451	-0.057	*GALNT3*	NA	FirstExon	Exon

cg26215727	12	6485537	2.21E-32	0.388	0.445	-0.057	*SCNN1A*	NA	TSS1500	Intron
cg21073459	19	54604098	2.30E-32	0.329	0.379	-0.050	*OSCAR*	Shore	5UTR	Exon
cg13277939	14	39735211	2.37E-32	0.334	0.397	-0.063	*CTAGE5*	Shore	TSS1500	Intron
cg06801028	10	51575592	2.39E-32	0.307	0.363	-0.057	*NCOA4*	Shelf	GeneBody	Intron
cg07495389	2	27233872	2.48E-32	0.309	0.361	-0.052	*MAPRE3*	NA	5UTR	Intron
cg23202722	1	33793808	2.53E-32	0.312	0.372	-0.060	*PHC2*	NA	GeneBody	Intron
cg13298466	12	6658164	2.53E-32	0.463	0.535	-0.072	*IFFO1*	Shore	ncRNA	Intron
cg01178099	3	177391387	2.60E-32	0.385	0.454	-0.069	*LINC00578*	NA	ncRNA	Intron
cg09859659	8	142180109	2.60E-32	0.520	0.590	-0.071	*DENND3*	Shelf	GeneBody	Intron
cg24339704	19	2529022	2.60E-32	0.432	0.503	-0.071	*GNG7*	Shelf	5UTR	Intron
cg07283015	18	22039857	2.63E-32	0.414	0.481	-0.067	*HRH4*	NA	TSS1500	NA
cg01087254	15	57052190	2.75E-32	0.295	0.357	-0.062	NA	NA	NA	NA
cg10665891	12	117042917	2.77E-32	0.385	0.475	-0.090	NA	NA	NA	NA
cg18460107	12	7902153	2.78E-32	0.741	0.686	0.055	*CLEC4C*	NA	TSS200	NA
cg16509045	9	77502963	3.02E-32	0.389	0.452	-0.064	*TRPM6*	Shore	TSS1500	Exon
cg01735277	15	75077691	3.12E-32	0.686	0.634	0.052	*CSK*	Shelf	5UTR	Intron
cg04813880	16	21171067	3.13E-32	0.159	0.222	-0.063	*TMEM159*	Shore	5UTR	Intron
cg17517296	2	43107458	3.13E-32	0.350	0.403	-0.053	NA	NA	NA	NA
cg00660167	17	80200974	3.18E-32	0.387	0.468	-0.081	*CSNK1D*	Shore	3UTR	Exon
cg00446046	3	187686918	3.24E-32	0.387	0.465	-0.078	NA	NA	NA	NA
cg26934362	5	1519265	3.25E-32	0.821	0.766	0.055	*LPCAT1*	Shelf	GeneBody	Intron
cg25968394	7	104604511	3.29E-32	0.424	0.496	-0.072	NA	NA	NA	NA
cg00593607	12	93530780	3.32E-32	0.354	0.418	-0.064	*LOC643339*	NA	ncRNA	Intron
cg17547928	4	737930	3.45E-32	0.360	0.412	-0.052	*PCGF3*	Shore	GeneBody	Intron
cg05249836	22	45609402	3.49E-32	0.430	0.504	-0.074	*KIAA0930*	NA	TSS1500	Intron
cg18503912	19	35630279	3.49E-32	0.361	0.430	-0.069	*FXYD1*	Shelf	TSS200	Intron
cg11235297	5	1108315	3.57E-32	0.287	0.349	-0.062	*SLC12A7*	Shore	GeneBody	Intron
cg02067584	17	8094552	3.83E-32	0.345	0.420	-0.075	*C17orf59*	Shore	TSS1500	NA
cg27627524	8	131325625	4.29E-32	0.314	0.372	-0.058	*ASAP1*	NA	GeneBody	Intron
cg25801976	8	48648112	4.30E-32	0.260	0.316	-0.057	*KIAA0146*	Shore	3UTR	Exon
cg11258381	1	117350694	4.37E-32	0.767	0.706	0.062	NA	NA	NA	NA
cg13171679	8	41258634	4.97E-32	0.297	0.349	-0.053	NA	NA	NA	NA
cg03739609	6	31555016	4.97E-32	0.352	0.406	-0.054	*LST1*	NA	5UTR	Exon
cg02231590	2	231737958	4.98E-32	0.772	0.708	0.065	*ITM2C*	NA	GeneBody	Intron
cg08840010	1	8000314	5.30E-32	0.350	0.431	-0.081	*TNFRSF9*	Shelf	5UTR	Intron
cg25957124	16	21171084	5.51E-32	0.278	0.331	-0.054	*TMEM159*	Shore	5UTR	Intron
cg10785340	5	134715577	5.81E-32	0.246	0.299	-0.052	*H2AFY*	NA	GeneBody	Intron
cg23944572	5	154101659	5.89E-32	0.656	0.598	0.058	*LARP1*	NA	GeneBody	Intron
cg16163847	13	52165356	6.23E-32	0.362	0.421	-0.059	*WDFY2*	NA	GeneBody	Intron
cg24475517	2	118737667	6.46E-32	0.379	0.442	-0.062	*CCDC93*	NA	GeneBody	Intron
cg06632549	6	31529993	7.56E-32	0.716	0.655	0.061	NA	NA	NA	NA
cg05344747	11	33754357	7.85E-32	0.465	0.547	-0.082	*CD59*	Shelf	5UTR	Intron
cg10738119	7	75957040	8.49E-32	0.342	0.399	-0.057	*YWHAG*	Shore	3UTR	Exon
cg17934470	5	49959703	9.07E-32	0.324	0.387	-0.063	NA	Shelf	NA	NA
cg07561747	1	200983313	9.17E-32	0.386	0.450	-0.064	*KIF21B*	NA	GeneBody	Intron
cg21937128	1	150971889	9.24E-32	0.251	0.310	-0.059	*FAM63A*	NA	GeneBody	Exon
cg21095280	6	36724036	9.31E-32	0.741	0.689	0.052	*CPNE5*	NA	GeneBody	Exon
cg23772226	22	29225779	9.34E-32	0.321	0.377	-0.056	NA	NA	NA	NA
cg08418670	19	4374567	1.00E-31	0.460	0.534	-0.074	*SH3GL1*	Shelf	GeneBody	Intron
cg24786875	5	94027853	1.04E-31	0.412	0.473	-0.062	*ANKRD32*	NA	GeneBody	Exon
cg09535475	2	42444827	1.06E-31	0.405	0.480	-0.075	*EML4*	NA	GeneBody	Intron
cg08776660	16	1670473	1.06E-31	0.789	0.738	0.052	*CRAMP1L*	NA	GeneBody	Intron
cg25070639	17	41170693	1.16E-31	0.263	0.318	-0.054	*VAT1*	Shelf	GeneBody	Exon
cg26873329	5	32522933	1.23E-31	0.411	0.490	-0.079	NA	NA	NA	NA
cg06706156	1	230183251	1.23E-31	0.367	0.427	-0.061	NA	NA	NA	NA
cg21440776	12	6658378	1.25E-31	0.340	0.414	-0.074	*IFFO1*	Shore	ncRNA	Intron
cg07241090	12	124864594	1.35E-31	0.298	0.348	-0.050	*NCOR2*	NA	GeneBody	Intron
cg04886849	10	118997146	1.36E-31	0.308	0.359	-0.051	NA	Shelf	NA	NA
cg11922563	5	75016955	1.36E-31	0.367	0.440	-0.073	NA	Shelf	NA	NA
cg12645852	18	74198774	1.38E-31	0.336	0.393	-0.057	*ZNF516*	Shelf	5UTR	Intron
cg26620021	19	40788926	1.46E-31	0.789	0.738	0.051	*AKT2*	Shore	5UTR	Intron
cg02969426	13	45775005	1.53E-31	0.565	0.628	-0.062	*GTF2F2*	NA	GeneBody	Intron
cg09571972	6	2104322	1.69E-31	0.280	0.333	-0.053	*GMDS*	NA	GeneBody	Intron
cg27087781	16	30108404	1.79E-31	0.292	0.349	-0.057	*YPEL3*	Shore	TSS1500	NA
cg15029631	12	52078762	1.84E-31	0.383	0.450	-0.067	*SCN8A*	NA	GeneBody	Intron
cg26002008	5	1477719	1.93E-31	0.304	0.355	-0.051	*LPCAT1*	NA	GeneBody	Intron
cg13410614	9	136341915	1.95E-31	0.308	0.363	-0.054	*SLC2A6*	Shore	GeneBody	Intron
cg19636224	1	44176844	2.16E-31	0.428	0.491	-0.063	*ST3GAL3*	Shelf	5UTR	Intron
cg07298177	16	85577847	2.16E-31	0.373	0.432	-0.059	NA	NA	NA	NA
cg25913761	15	90727560	2.33E-31	0.699	0.646	0.054	*SEMA4B*	Shore	TSS1500	NA
cg01048372	8	144655484	2.36E-31	0.360	0.411	-0.051	*C8orf73*	Shelf	TSS1500	NA
cg24361586	17	62744534	2.36E-31	0.465	0.523	-0.057	NA	NA	NA	NA
cg23193059	11	116706090	2.43E-31	0.354	0.425	-0.071	NA	Shore	NA	NA
cg27369423	16	56228901	2.52E-31	0.223	0.279	-0.056	*GNAO1*	Shelf	GeneBody	Intron
cg14642045	12	109538736	2.55E-31	0.260	0.322	-0.062	*UNG*	Shelf	GeneBody	Intron
cg00009088	11	60930188	2.62E-31	0.407	0.485	-0.078	*VPS37C*	Shore	TSS1500	NA
cg00994616	12	6855223	2.62E-31	0.214	0.267	-0.053	NA	Shelf	NA	NA
cg17346145	17	77755547	2.65E-31	0.297	0.352	-0.055	*CBX2*	Shelf	GeneBody	Exon

cg26184474	5	61623163	2.67E-31	0.381	0.451	-0.070	*KIF2A*	NA	GeneBody	Intron
cg17080697	6	30297382	2.82E-31	0.404	0.473	-0.068	*TRIM39*	Shelf	FirstExon	Exon
cg06690535	3	196352142	2.85E-31	0.321	0.372	-0.051	NA	NA	NA	NA
cg04858164	15	57324333	2.89E-31	0.463	0.564	-0.101	*TCF12*	NA	GeneBody	Intron
cg19351604	8	1870722	3.02E-31	0.411	0.477	-0.065	*ARHGEF10*	NA	GeneBody	Intron
cg01462353	2	169939873	3.08E-31	0.365	0.437	-0.072	*DHRS9*	NA	GeneBody	Exon
cg26008365	1	153958797	3.42E-31	0.184	0.245	-0.062	*RAB13*	NA	5UTR	Exon
cg13703437	5	39219698	3.44E-31	0.331	0.394	-0.063	*FYB*	NA	TSS200	Intron
cg21263566	1	95561502	3.52E-31	0.355	0.417	-0.061	*TMEM56*	NA	5UTR	Intron
cg13189271	6	15365693	4.01E-31	0.216	0.275	-0.059	*JARID2*	NA	GeneBody	Intron
cg09740468	6	3025325	4.13E-31	0.370	0.427	-0.057	NA	NA	NA	NA
cg10687644	11	72527565	4.17E-31	0.314	0.365	-0.051	*ATG16L2*	Shore	GeneBody	Intron
cg16998950	5	95252658	4.41E-31	0.332	0.410	-0.078	*ELL2*	NA	GeneBody	Intron
cg14578677	4	38859770	4.43E-31	0.398	0.474	-0.076	*TLR6*	NA	TSS1500	NA
cg16762030	15	50148234	4.89E-31	0.330	0.393	-0.064	NA	NA	NA	NA
cg03610527	2	242174889	5.09E-31	0.454	0.528	-0.073	*HDLBP*	NA	GeneBody	Intron
cg13469851	9	26936447	5.25E-31	0.474	0.549	-0.074	*PLAA*	NA	GeneBody	Intron
cg21999229	6	31540014	5.74E-31	0.776	0.725	0.051	*LTA*	NA	5UTR	Exon
cg09017001	1	31870508	6.51E-31	0.353	0.407	-0.054	NA	NA	NA	NA
cg07241925	4	1294566	6.58E-31	0.330	0.386	-0.056	*MAEA*	NA	GeneBody	Intron
cg02677635	9	129099507	6.72E-31	0.343	0.410	-0.067	*FAM125B*	Shore	GeneBody	Intron
cg01800253	22	22290866	7.05E-31	0.267	0.323	-0.056	*PPM1F*	Shore	GeneBody	Intron
cg16815882	1	35908609	7.37E-31	0.305	0.355	-0.050	*KIAA0319L*	NA	GeneBody	Exon
cg00439981	6	170684299	7.59E-31	0.449	0.536	-0.087	*FAM120B*	Shore	GeneBody	Intron
cg17442155	7	99955675	7.72E-31	0.407	0.474	-0.067	*PILRB*	NA	5UTR	Exon
cg07905808	6	30297389	8.14E-31	0.505	0.563	-0.057	*TRIM39*	Shelf	FirstExon	Exon
cg09878888	11	57529614	8.23E-31	0.238	0.291	-0.053	*TMX2-CTNND1*	NA	ncRNA	Intron
cg13226290	20	1448595	8.23E-31	0.373	0.440	-0.067	*NSFL1C*	Shore	TSS1500	NA
cg01492538	7	2774543	8.31E-31	0.311	0.376	-0.064	*GNA12*	CpGIsland	GeneBody	Intron
cg00578614	6	30070403	8.38E-31	0.333	0.394	-0.062	NA	Shore	NA	NA
cg18390025	10	103986736	8.73E-31	0.317	0.395	-0.078	*ELOVL3*	Shore	GeneBody	Intron
cg12614630	12	57387318	9.52E-31	0.392	0.455	-0.063	*GPR182*	NA	TSS1500	NA
cg24496021	11	72662461	9.79E-31	0.398	0.475	-0.077	*FCHSD2*	NA	GeneBody	Intron
cg18838431	11	74178800	1.07E-30	0.271	0.322	-0.050	*KCNE3*	CpGIsland	TSS200	NA
cg02999224	14	23284559	1.12E-30	0.344	0.405	-0.061	*SLC7A7*	NA	5UTR	Exon
cg13468144	17	4081428	1.28E-30	0.490	0.566	-0.076	*ANKFY1*	NA	GeneBody	Intron
cg23079808	12	6493003	1.31E-30	0.250	0.310	-0.061	*LTBR*	CpGIsland	TSS200	Intron
cg09115713	16	88832476	1.38E-30	0.836	0.781	0.054	*PIEZO1*	NA	GeneBody	Intron
cg00554993	14	23588616	1.39E-30	0.251	0.303	-0.052	*CEBPE*	NA	5UTR	Exon
cg24914483	18	2653997	1.41E-30	0.338	0.412	-0.075	*CBX3P2*	Shore	ncRNA	Intron
cg03221483	17	75315081	1.53E-30	0.342	0.420	-0.078	*SEPT9*	NA	5UTR	Intron
cg27664407	16	5116674	1.60E-30	0.309	0.373	-0.064	*C16orf89*	NA	TSS1500	NA
cg16162970	14	105779952	1.61E-30	0.273	0.326	-0.052	*PACS2*	Shore	TSS1500	Intron
cg25941751	2	46613544	1.68E-30	0.305	0.362	-0.057	*EPAS1*	NA	3UTR	Exon
cg26562921	16	84760429	1.75E-30	0.273	0.326	-0.053	*USP10*	NA	GeneBody	Intron
cg06706813	3	194826411	1.82E-30	0.545	0.617	-0.071	*XXYLT1*	NA	GeneBody	Intron
cg01023672	12	47477223	1.84E-30	0.349	0.421	-0.072	NA	Shelf	NA	NA
cg25539505	5	95634103	1.88E-30	0.446	0.516	-0.070	NA	NA	NA	NA
cg20227511	13	28670602	1.89E-30	0.426	0.498	-0.071	*FLT3*	Shelf	GeneBody	Intron
cg26244575	12	76354015	1.89E-30	0.682	0.627	0.055	NA	NA	NA	NA
cg15553418	1	16696547	2.03E-30	0.324	0.382	-0.058	*SZRD1*	Shelf	GeneBody	Intron
cg02227879	10	75576192	2.08E-30	0.375	0.449	-0.073	*CAMK2G*	NA	GeneBody	Intron
cg16102040	4	146685656	2.12E-30	0.518	0.585	-0.066	*ZNF827*	Shelf	3UTR	Intron
cg06654079	16	21169179	2.18E-30	0.362	0.427	-0.065	*TMEM159*	Shore	TSS1500	Intron
cg02241759	3	119528300	2.28E-30	0.301	0.366	-0.065	*NR1I2*	Shore	GeneBody	Intron
cg25079915	2	120192027	2.33E-30	0.313	0.366	-0.053	*TMEM37*	Shore	GeneBody	Intron
cg12649238	6	36669758	2.50E-30	0.520	0.596	-0.076	*RAB44*	NA	5UTR	Intron
cg14985891	1	116270626	2.55E-30	0.355	0.428	-0.073	*CASQ2*	NA	GeneBody	Intron
cg09032544	1	167487295	2.70E-30	0.763	0.711	0.052	*CD247*	NA	GeneBody	Intron
cg00945209	17	76801579	2.84E-30	0.682	0.627	0.055	*USP36*	Shelf	GeneBody	Intron
cg25779483	4	89978300	3.04E-30	0.231	0.290	-0.059	*FAM13A*	NA	5UTR	Exon
cg18905668	11	18388147	3.05E-30	0.212	0.271	-0.058	*GTF2H1*	NA	3UTR	Exon
cg20094462	19	848071	3.13E-30	0.334	0.392	-0.058	*PRTN3*	Shelf	3UTR	Exon
cg01057573	5	118683890	3.15E-30	0.369	0.440	-0.070	*TNFAIP8*	NA	GeneBody	Intron
cg16814786	10	51575763	3.46E-30	0.309	0.374	-0.065	*NCOA4*	Shelf	GeneBody	Intron
cg13995774	5	179189810	3.63E-30	0.327	0.394	-0.067	*MAML1*	NA	GeneBody	Intron
cg08557970	6	166856094	3.63E-30	0.383	0.441	-0.057	*RPS6KA2*	NA	GeneBody	Intron
cg21696012	1	24131000	3.68E-30	0.392	0.461	-0.069	*HMGCL*	Shelf	GeneBody	Exon
cg17525495	17	56401734	3.84E-30	0.400	0.471	-0.071	*BZRAP1*	CpGIsland	GeneBody	Intron
cg10877430	12	113527336	3.87E-30	0.558	0.507	0.052	*DTX1*	Shelf	GeneBody	Intron
cg24234270	7	2152821	4.10E-30	0.208	0.260	-0.052	*MAD1L1*	Shore	GeneBody	Intron
cg19673549	5	54916621	4.34E-30	0.423	0.506	-0.083	NA	NA	NA	NA
cg19563510	17	79881483	4.44E-30	0.225	0.292	-0.067	*MAFG*	Shelf	5UTR	Intron
cg07814567	4	100736658	4.68E-30	0.403	0.469	-0.066	*DAPP1*	NA	TSS1500	NA
cg12045443	12	89777963	4.92E-30	0.273	0.326	-0.052	NA	NA	NA	NA
cg23085846	20	62522518	4.95E-30	0.415	0.470	-0.055	*TPD52L2*	Shelf	3UTR	Exon
cg14628914	6	24659344	5.07E-30	0.448	0.508	-0.060	*TDP2*	NA	GeneBody	Intron
cg24056365	1	110834615	5.09E-30	0.394	0.463	-0.069	*LOC440600*	NA	ncRNA	Intron

cg26076724	6	15090163	5.13E-30	0.334	0.392	-0.058	NA	NA	NA	NA
cg17262810	10	35676874	5.18E-30	0.374	0.436	-0.062	*CCNY*	NA	5UTR	Intron
cg25356504	17	26707476	5.22E-30	0.268	0.318	-0.050	*SARM1*	Shelf	GeneBody	Intron
cg22595920	1	9716050	5.31E-30	0.257	0.316	-0.059	*PIK3CD*	Shelf	5UTR	Intron
cg13321166	11	59824038	5.37E-30	0.296	0.352	-0.056	*MS4A3*	NA	TSS200	NA
cg18276112	7	4755032	6.38E-30	0.388	0.464	-0.076	*FOXK1*	Shelf	GeneBody	Intron
cg02374486	10	72362809	6.53E-30	0.725	0.674	0.051	*PRF1*	Shelf	TSS1500	NA
cg17627898	12	118782453	6.93E-30	0.323	0.384	-0.061	*TAOK3*	NA	5UTR	Intron
cg20893838	6	7145478	6.96E-30	0.306	0.357	-0.051	*RREB1*	Shelf	5UTR	Intron
cg02243157	2	64047802	7.04E-30	0.477	0.553	-0.076	NA	NA	NA	NA
cg09452568	5	54275198	7.14E-30	0.282	0.354	-0.072	*ESM1*	NA	GeneBody	Exon
cg06738887	15	60688010	7.39E-30	0.273	0.326	-0.054	*ANXA2*	Shore	5UTR	Intron
cg26736341	6	31545342	7.72E-30	0.803	0.753	0.050	*TNF*	Shelf	3UTR	Exon
cg05886087	2	102758186	7.80E-30	0.327	0.393	-0.066	NA	Shore	NA	NA
cg04252203	3	194696866	7.94E-30	0.337	0.393	-0.056	NA	NA	NA	NA
cg03418002	4	140655478	8.11E-30	0.373	0.446	-0.073	*MGST2*	Shore	3UTR	Intron
cg26837399	1	236209798	8.20E-30	0.345	0.412	-0.067	*NID1*	NA	GeneBody	Intron
cg14438453	6	39786566	8.26E-30	0.403	0.462	-0.059	*DAAM2*	NA	5UTR	Intron
cg01502428	17	76850266	8.29E-30	0.464	0.550	-0.085	*TIMP2*	Shore	3UTR	Exon
cg07076915	16	2174754	8.42E-30	0.331	0.391	-0.061	*PKD1*	Shelf	GeneBody	Intron
cg14554468	17	28696496	8.46E-30	0.279	0.339	-0.060	NA	NA	NA	NA
cg20613972	4	84037158	8.72E-30	0.466	0.543	-0.076	*PLAC8*	NA	TSS1500	NA
cg04411201	3	99614305	8.72E-30	0.312	0.364	-0.051	*FILIP1L*	NA	GeneBody	Intron
cg05148465	17	7034129	9.10E-30	0.261	0.316	-0.055	NA	NA	NA	NA
cg13618516	17	79129078	9.43E-30	0.411	0.476	-0.065	*AATK*	Shelf	GeneBody	Intron
cg04932082	6	91113506	9.65E-30	0.282	0.334	-0.053	NA	NA	NA	NA
cg16501560	21	46078007	1.02E-29	0.668	0.609	0.058	*TSPEAR*	NA	GeneBody	Intron
cg21426759	6	30303126	1.02E-29	0.469	0.538	-0.069	*TRIM39*	NA	GeneBody	Intron
cg20090162	2	47261900	1.08E-29	0.356	0.421	-0.065	*TTC7A*	NA	GeneBody	Intron
cg14437551	6	31539986	1.08E-29	0.780	0.720	0.060	*LTA*	NA	5UTR	Exon
cg15244101	12	49627624	1.15E-29	0.269	0.319	-0.050	NA	Shore	NA	NA
cg19607845	6	52934457	1.20E-29	0.327	0.393	-0.066	*FBXO9*	Shelf	GeneBody	Intron
cg03048029	21	34101413	1.21E-29	0.370	0.446	-0.077	*GCFC1-AS1*	Shore	ncRNA	Exon
cg08944026	5	10626811	1.22E-29	0.295	0.352	-0.057	*ANKRD33B*	NA	GeneBody	Intron
cg16226866	6	110418140	1.27E-29	0.371	0.444	-0.073	NA	NA	NA	NA
cg11814087	5	32391135	1.33E-29	0.420	0.490	-0.070	*ZFR*	NA	GeneBody	Intron
cg03624195	19	14090310	1.39E-29	0.311	0.367	-0.056	*RFX1*	Shore	GeneBody	Exon
cg11908131	2	106685045	1.40E-29	0.373	0.444	-0.071	*C2orf40*	Shelf	GeneBody	Intron
cg16303353	12	2393684	1.45E-29	0.310	0.363	-0.053	*CACNA1C*	NA	GeneBody	Intron
cg08276042	16	2740348	1.48E-29	0.311	0.364	-0.053	*KCTD5*	NA	GeneBody	Intron
cg00760729	2	161236088	1.50E-29	0.312	0.371	-0.060	*RBMS1*	NA	GeneBody	Intron
cg08044694	19	15391927	1.55E-29	0.316	0.370	-0.054	*BRD4*	NA	TSS1500	NA
cg09109411	5	149319112	1.57E-29	0.372	0.427	-0.055	*PDE6A*	NA	GeneBody	Intron
cg19323289	11	19463903	1.62E-29	0.744	0.692	0.051	*NAV2*	NA	GeneBody	Intron
cg00324097	17	75446549	1.63E-29	0.556	0.505	0.051	*SEPT9*	Shore	GeneBody	Intron
cg15958424	3	132036067	1.63E-29	0.320	0.371	-0.050	*ACPP*	NA	TSS200	NA
cg19011001	14	93539613	1.76E-29	0.305	0.356	-0.051	*ITPK1*	NA	GeneBody	Intron
cg01172150	16	30817443	1.97E-29	0.210	0.262	-0.052	NA	Shore	NA	NA
cg09020199	6	30297320	2.04E-29	0.422	0.483	-0.061	*TRIM39*	Shelf	FirstExon	Exon
cg18507018	8	92035517	2.07E-29	0.394	0.468	-0.074	*TMEM55A*	NA	GeneBody	Intron
cg02579377	16	85047901	2.15E-29	0.300	0.363	-0.063	NA	Shelf	NA	NA
cg14118850	2	10447890	2.21E-29	0.384	0.441	-0.057	*HPCAL1*	Shelf	5UTR	Intron
cg01377358	20	30948211	2.22E-29	0.683	0.630	0.053	*ASXL1*	Shore	GeneBody	Intron
cg18854765	1	74665362	2.24E-29	0.301	0.364	-0.062	*FPGT*	Shore	GeneBody	Exon
cg04144521	2	191276183	2.27E-29	0.348	0.404	-0.055	*MFSD6*	Shelf	5UTR	Intron
cg24906819	8	8231531	2.30E-29	0.372	0.437	-0.065	*SGK223*	NA	GeneBody	Intron
cg26783127	17	79128918	2.43E-29	0.353	0.437	-0.085	*AATK*	Shelf	GeneBody	Intron
cg18517055	17	80581701	2.48E-29	0.499	0.584	-0.085	*WDR45L*	NA	GeneBody	Intron
cg05314350	6	28874702	2.61E-29	0.381	0.444	-0.063	*TRIM27*	NA	GeneBody	Intron
cg09501509	6	89745356	2.66E-29	0.390	0.453	-0.063	NA	NA	NA	NA
cg23825057	12	124014409	2.84E-29	0.405	0.465	-0.060	*RILPL1*	Shelf	GeneBody	Intron
cg10338518	4	87895137	3.27E-29	0.345	0.416	-0.071	*AFF1*	NA	GeneBody	Intron
cg10958452	1	44114346	3.39E-29	0.345	0.418	-0.072	*KDM4A*	Shore	TSS1500	NA
cg18351781	19	4950888	3.62E-29	0.355	0.435	-0.080	*UHRF1*	Shelf	GeneBody	Exon
cg00261690	1	28856281	3.69E-29	0.394	0.460	-0.066	*RCC1*	Shore	5UTR	Intron
cg21310090	20	44538669	3.77E-29	0.350	0.408	-0.058	*PLTP*	Shore	GeneBody	Intron
cg05798664	1	36825645	3.86E-29	0.299	0.352	-0.053	*STK40*	NA	GeneBody	Intron
cg11239575	17	3705765	3.86E-29	0.478	0.549	-0.071	*ITGAE*	NA	TSS1500	NA
cg21170682	11	122205382	4.00E-29	0.356	0.412	-0.057	NA	NA	NA	NA
cg16378063	17	47329552	4.05E-29	0.379	0.446	-0.067	*FLJ40194*	NA	ncRNA	Intron
cg08700083	4	48382815	4.08E-29	0.319	0.377	-0.059	*SLAIN2*	NA	GeneBody	Intron
cg15142192	1	36521401	4.12E-29	0.345	0.415	-0.070	*EIF2C3*	NA	3UTR	Exon
cg12157761	15	71005778	4.43E-29	0.388	0.458	-0.071	*UACA*	NA	GeneBody	Intron
cg14059339	6	28875356	4.51E-29	0.376	0.456	-0.080	*TRIM27*	NA	GeneBody	Intron
cg21057323	14	103412980	4.66E-29	0.653	0.594	0.058	*CDC42BPB*	NA	GeneBody	Exon
cg11168614	14	87179368	4.69E-29	0.418	0.485	-0.067	NA	NA	NA	NA
cg04903759	3	167238645	4.70E-29	0.345	0.413	-0.068	*WDR49*	NA	GeneBody	Intron
cg06468347	17	3705875	4.82E-29	0.281	0.338	-0.057	*ITGAE*	NA	TSS1500	NA

cg13471188	2	20638399	4.89E-29	0.323	0.381	-0.058	NA	NA	NA	NA
cg19047868	17	46669485	4.92E-29	0.198	0.258	-0.060	*HOXB-AS3*	CpGIsland	ncRNA	Intron
cg25790531	6	113755716	5.83E-29	0.509	0.587	-0.078	NA	NA	NA	NA
cg05931439	14	23305957	5.89E-29	0.230	0.299	-0.069	*MMP14*	CpGIsland	5UTR	Exon
cg11123644	2	219597614	5.89E-29	0.260	0.311	-0.051	*TTLL4*	NA	5UTR	Intron
cg18537222	3	12435731	6.20E-29	0.415	0.482	-0.067	*PPARG*	NA	GeneBody	Intron
cg22242148	15	74215283	6.21E-29	0.306	0.356	-0.050	*LOXL1-AS1*	Shelf	ncRNA	Intron
cg08165796	8	117484298	6.30E-29	0.370	0.448	-0.078	NA	NA	NA	NA
cg04347477	12	125002007	6.63E-29	0.280	0.351	-0.072	*NCOR2*	CpGIsland	5UTR	Intron
cg01381934	3	52529064	6.64E-29	0.272	0.329	-0.057	*STAB1*	NA	TSS1500	NA
cg01627252	2	31397283	6.87E-29	0.373	0.432	-0.058	*CAPN14*	NA	3UTR	Exon
cg16024891	17	2839082	7.18E-29	0.242	0.297	-0.055	*RAP1GAP2*	NA	GeneBody	Intron
cg10426076	11	57158282	7.42E-29	0.370	0.435	-0.065	*PRG2*	NA	TSS200	NA
cg20181887	12	123753272	7.43E-29	0.325	0.413	-0.088	*CDK2AP1*	Shore	ncRNA	Intron
cg14575356	6	130013903	7.56E-29	0.455	0.533	-0.078	*ARHGAP18*	NA	GeneBody	Intron
cg04382396	19	852311	7.61E-29	0.415	0.481	-0.066	*ELANE*	Shelf	5UTR	Exon
cg04100124	10	69611755	8.39E-29	0.365	0.429	-0.064	NA	Shelf	NA	NA
cg02251850	17	78851503	8.79E-29	0.793	0.742	0.050	*RPTOR*	Shore	GeneBody	Intron
cg14533068	6	158507953	8.80E-29	0.460	0.541	-0.081	*SYNJ2*	CpGIsland	GeneBody	Exon
cg23210971	4	88049275	9.04E-29	0.490	0.560	-0.070	*AFF1*	NA	GeneBody	Intron
cg06352352	16	67181466	9.08E-29	0.392	0.443	-0.052	*C16orf70*	Shore	3UTR	Exon
cg11180667	10	101415865	9.28E-29	0.308	0.367	-0.059	NA	Shelf	NA	NA
cg00211174	3	127332098	9.37E-29	0.387	0.462	-0.075	*MCM2*	NA	GeneBody	Intron
cg20107506	4	100874826	9.67E-29	0.396	0.475	-0.079	NA	Shelf	NA	NA
cg22894805	15	41983773	1.00E-28	0.349	0.404	-0.054	*MGA*	NA	GeneBody	Intron
cg05486924	3	194979565	1.01E-28	0.258	0.317	-0.059	*XXYLT1*	Shore	GeneBody	Intron
cg06373940	2	128052778	1.01E-28	0.438	0.516	-0.078	*ERCC3*	Shore	TSS1500	NA
cg17013990	1	161091682	1.02E-28	0.306	0.363	-0.057	*DEDD*	Shelf	3UTR	Exon
cg27317813	12	4398508	1.08E-28	0.246	0.298	-0.052	*CCND2*	NA	GeneBody	Intron
cg08169020	14	69256888	1.17E-28	0.681	0.621	0.060	*ZFP36L1*	CpGIsland	GeneBody	Exon
cg15361231	1	193075191	1.27E-28	0.417	0.486	-0.069	*GLRX2*	Shore	TSS1500	Exon
cg06317209	12	58210878	1.35E-28	0.334	0.409	-0.075	*AVIL*	NA	TSS1500	NA
cg18888137	6	32945759	1.37E-28	0.329	0.391	-0.063	*BRD2*	NA	GeneBody	Exon
cg12313149	2	136763629	1.38E-28	0.389	0.459	-0.070	NA	NA	NA	NA
cg26633373	19	54377836	1.42E-28	0.344	0.403	-0.059	*MYADM*	Shore	3UTR	Exon
cg04452195	13	20762982	1.45E-28	0.377	0.451	-0.075	*GJB2*	Shelf	3UTR	Exon
cg21932814	3	122044172	1.47E-28	0.334	0.386	-0.052	*CSTA*	NA	FirstExon	Exon
cg14272075	17	55122538	1.48E-28	0.390	0.462	-0.072	NA	Shore	NA	NA
cg12044599	11	67206308	1.49E-28	0.746	0.692	0.055	*PTPRCAP*	Shelf	TSS1500	Exon
cg20704555	7	28127748	1.50E-28	0.407	0.472	-0.065	*JAZF1*	NA	GeneBody	Intron
cg07948875	1	169187004	1.54E-28	0.281	0.338	-0.057	*NME7*	NA	GeneBody	Intron
cg24401262	1	31956405	1.58E-28	0.276	0.329	-0.053	NA	NA	NA	NA
cg08223235	18	60903834	1.62E-28	0.272	0.336	-0.063	*BCL2*	NA	GeneBody	Intron
cg16343924	6	29430158	1.68E-28	0.735	0.682	0.053	*OR2H1*	NA	FirstExon	Exon
cg23206115	6	158066900	1.72E-28	0.428	0.494	-0.066	*ZDHHC14*	NA	GeneBody	Intron
cg27295342	3	195849368	1.73E-28	0.202	0.257	-0.054	NA	NA	NA	NA
cg01526217	3	12524194	1.79E-28	0.327	0.393	-0.065	NA	Shore	NA	NA
cg06823060	16	81616874	1.86E-28	0.503	0.569	-0.066	*CMIP*	NA	GeneBody	Intron
cg12963656	3	101659687	1.91E-28	0.242	0.305	-0.064	NA	NA	NA	NA
cg04658021	17	8056967	1.92E-28	0.319	0.385	-0.066	*PER1*	Shore	TSS1500	NA
cg26847100	19	10748778	1.99E-28	0.290	0.350	-0.060	*SLC44A2*	Shore	GeneBody	Intron
cg08633074	16	11837719	2.04E-28	0.300	0.359	-0.059	*TXNDC11*	Shore	TSS1500	NA
cg00841141	2	37416819	2.08E-28	0.426	0.491	-0.065	*SULT6B1*	NA	TSS1500	NA
cg11324504	22	38474147	2.11E-28	0.316	0.377	-0.061	*SLC16A8*	Shelf	3UTR	Exon
cg23575688	11	119486443	2.23E-28	0.258	0.309	-0.051	NA	NA	NA	NA
cg11525409	2	74236068	2.28E-28	0.427	0.486	-0.059	NA	NA	NA	NA
cg15511120	11	6598119	2.37E-28	0.293	0.345	-0.052	NA	NA	NA	NA
cg08939373	11	33563246	2.52E-28	0.377	0.447	-0.071	*KIAA1549L*	NA	TSS1500	NA
cg02575483	6	117826706	2.53E-28	0.288	0.339	-0.051	*DCBLD1*	NA	GeneBody	Intron
cg21870229	21	45145841	2.64E-28	0.240	0.296	-0.056	*PDXK*	Shelf	GeneBody	Intron
cg07055315	2	32490835	2.85E-28	0.340	0.405	-0.065	*NLRC4*	NA	TSS1500	NA
cg07895132	17	33825172	2.99E-28	0.346	0.412	-0.066	NA	NA	NA	NA
cg01553231	5	141072866	3.06E-28	0.721	0.654	0.067	NA	Shore	NA	NA
cg15058645	2	175528343	3.08E-28	0.302	0.354	-0.052	*WIPF1*	NA	5UTR	Intron
cg10725937	6	41130978	3.10E-28	0.365	0.421	-0.056	*TREM2*	NA	TSS200	NA
cg16824282	3	128779590	3.12E-28	0.377	0.443	-0.067	*GP9*	NA	TSS200	NA
cg05422796	18	5297381	3.49E-28	0.366	0.442	-0.076	*ZFP161*	Shelf	TSS1500	NA
cg22084410	3	51987688	3.49E-28	0.317	0.367	-0.050	NA	Shelf	NA	NA
cg14414903	2	240171712	3.54E-28	0.292	0.361	-0.069	*HDAC4*	NA	GeneBody	Intron
cg18279004	3	111802305	3.56E-28	0.453	0.529	-0.076	NA	Shelf	NA	NA
cg25199552	1	23350054	4.06E-28	0.392	0.465	-0.073	*KDM1A*	Shelf	GeneBody	Intron
cg13424393	17	80569923	4.09E-28	0.403	0.473	-0.070	NA	Shore	NA	NA
cg24165638	19	831456	4.11E-28	0.467	0.527	-0.060	*AZU1*	Shelf	GeneBody	Intron
cg18646851	15	92934809	4.13E-28	0.319	0.379	-0.060	NA	Shore	NA	NA
cg21368063	2	27338538	4.27E-28	0.765	0.713	0.052	*CGREF1*	Shelf	5UTR	Intron
cg06407843	6	43279607	4.56E-28	0.352	0.421	-0.069	NA	Shelf	NA	NA
cg09993145	1	25291905	4.59E-28	0.552	0.481	0.071	*RUNX3*	NA	TSS1500	NA
cg26842080	5	42922968	4.62E-28	0.402	0.464	-0.062	NA	Shore	NA	NA

cg23575275	6	36653973	4.63E-28	0.271	0.322	-0.051	*CDKN1A*	Shore	3UTR	Exon
cg08183317	13	114261934	4.69E-28	0.254	0.307	-0.053	*TFDP1*	Shore	GeneBody	Intron
cg07305933	8	129079496	4.80E-28	0.328	0.386	-0.058	*PVT1*	NA	ncRNA	Intron
cg16139316	1	153330758	4.87E-28	0.302	0.358	-0.056	*S100A9*	NA	5UTR	Exon
cg17745697	2	38138126	5.00E-28	0.282	0.342	-0.060	NA	NA	NA	NA
cg07213830	1	1093940	5.04E-28	0.328	0.378	-0.050	NA	Shore	NA	NA
cg07053114	10	129794994	5.42E-28	0.272	0.330	-0.058	*PTPRE*	NA	5UTR	Intron
cg04089901	7	7557590	5.93E-28	0.323	0.376	-0.053	*COL28A1*	NA	GeneBody	Intron
cg09684160	1	17054957	6.51E-28	0.724	0.672	0.052	NA	Shelf	NA	NA
cg05651511	17	78754090	6.53E-28	0.304	0.377	-0.072	*RPTOR*	NA	GeneBody	Intron
cg17741993	6	31544694	6.79E-28	0.632	0.567	0.065	*TNF*	Shelf	GeneBody	Intron
cg07000116	16	30616649	7.13E-28	0.434	0.502	-0.068	*ZNF689*	Shore	GeneBody	Exon
cg03406844	2	152220529	7.14E-28	0.450	0.523	-0.073	*TNFAIP6*	NA	GeneBody	Exon
cg23511285	19	48205761	7.23E-28	0.459	0.518	-0.059	*GLTSCR1*	CpGIsland	3UTR	Exon
cg23597162	7	28102341	7.72E-28	0.425	0.482	-0.057	*JAZF1*	NA	GeneBody	Intron
cg10593922	10	30711382	8.24E-28	0.339	0.407	-0.068	NA	NA	NA	NA
cg23933241	17	33823690	8.69E-28	0.386	0.452	-0.066	NA	NA	NA	NA
cg01620379	14	21052361	9.02E-28	0.364	0.423	-0.060	*RNASE11*	NA	FirstExon	Exon
cg06502279	16	474467	9.44E-28	0.456	0.534	-0.078	*RAB11FIP3*	Shore	TSS1500	NA
cg03797115	4	110625010	9.64E-28	0.301	0.373	-0.072	*CASP6*	Shore	TSS1500	NA
cg08078028	10	120802096	9.96E-28	0.576	0.635	-0.059	*EIF3A*	CpGIsland	GeneBody	Exon
cg26898932	17	28442480	1.03E-27	0.404	0.487	-0.083	*NSRP1*	Shore	TSS1500	NA
cg26514961	12	94566784	1.05E-27	0.347	0.410	-0.063	*PLXNC1*	NA	GeneBody	Intron
cg13448978	1	27961796	1.06E-27	0.291	0.360	-0.069	*FGR*	CpGIsland	TSS200	NA
cg15736127	2	157292127	1.10E-27	0.363	0.438	-0.074	*GPD2*	CpGIsland	5UTR	Exon
cg17566541	11	1912287	1.10E-27	0.285	0.345	-0.061	*LSP1*	NA	3UTR	Intron
cg01229658	15	70924395	1.10E-27	0.333	0.394	-0.061	NA	NA	NA	NA
cg27586417	16	85577800	1.16E-27	0.461	0.536	-0.075	NA	NA	NA	NA
cg10283505	11	94278912	1.24E-27	0.428	0.482	-0.054	*FUT4*	CpGIsland	3UTR	Exon
cg23599224	4	68334446	1.24E-27	0.446	0.524	-0.078	NA	NA	NA	NA
cg17775490	20	45179354	1.32E-27	0.229	0.288	-0.059	*OCSTAMP*	NA	TSS200	NA
cg07546654	11	1320546	1.34E-27	0.354	0.418	-0.064	*TOLLIP*	Shelf	GeneBody	Intron
cg01822050	20	304156	1.36E-27	0.421	0.496	-0.074	NA	Shore	NA	NA
cg04407063	11	67051977	1.37E-27	0.350	0.406	-0.056	*ADRBK1*	Shore	GeneBody	Intron
cg19447962	17	17628656	1.41E-27	0.315	0.392	-0.077	*RAI1*	Shore	5UTR	Intron
cg13304638	17	80834089	1.44E-27	0.432	0.511	-0.079	*TBCD*	NA	GeneBody	Intron
cg20078972	19	15391832	1.44E-27	0.306	0.366	-0.060	*BRD4*	NA	TSS1500	NA
cg08402433	7	3020155	1.45E-27	0.278	0.331	-0.054	*CARD11*	NA	5UTR	Intron
cg08253808	14	102676957	1.46E-27	0.400	0.478	-0.078	*WDR20*	Shelf	GeneBody	Intron
cg16684817	1	28202173	1.46E-27	0.376	0.457	-0.081	*THEMIS2*	Shelf	GeneBody	Intron
cg04205769	1	101488098	1.48E-27	0.399	0.471	-0.072	*DPH5*	Shelf	GeneBody	Intron
cg22742001	12	110435418	1.49E-27	0.305	0.365	-0.060	*GIT2*	Shore	TSS1500	NA
cg04904784	12	27170664	1.53E-27	0.452	0.512	-0.060	NA	Shelf	NA	NA
cg17463083	17	62153284	1.56E-27	0.288	0.352	-0.064	*ERN1*	NA	GeneBody	Intron
cg06098215	10	51575702	1.61E-27	0.270	0.324	-0.054	*NCOA4*	Shelf	GeneBody	Intron
cg19092837	1	10271724	1.81E-27	0.447	0.517	-0.070	*KIF1B*	Shore	5UTR	Intron
cg09393619	3	195897833	1.85E-27	0.367	0.437	-0.071	NA	NA	NA	NA
cg08091050	4	26208051	1.86E-27	0.481	0.556	-0.074	NA	NA	NA	NA
cg05616969	2	118982661	2.15E-27	0.230	0.283	-0.053	NA	Shore	NA	NA
cg11327657	21	46388162	2.40E-27	0.250	0.302	-0.052	*FAM207A*	Shelf	GeneBody	Intron
cg20070090	1	153363489	2.46E-27	0.345	0.405	-0.060	*S100A8*	NA	5UTR	Intron
cg01662869	16	4730410	2.49E-27	0.347	0.412	-0.065	*MGRN1*	Shelf	GeneBody	Intron
cg02593884	16	2984574	2.50E-27	0.448	0.506	-0.057	*FLYWCH1*	NA	GeneBody	Intron
cg09390241	4	8174148	2.51E-27	0.749	0.670	0.079	NA	NA	NA	NA
cg23274824	1	45079966	2.66E-27	0.756	0.703	0.053	*RNF220*	Shelf	GeneBody	Exon
cg24448340	1	179921042	2.67E-27	0.301	0.354	-0.053	NA	Shelf	NA	NA
cg22570970	6	15401067	2.72E-27	0.247	0.310	-0.063	*JARID2*	NA	GeneBody	Intron
cg18315380	7	1095666	2.79E-27	0.290	0.340	-0.050	*C7orf50*	Shore	GeneBody	Intron
cg11083848	14	76371681	2.85E-27	0.352	0.415	-0.063	*TTLL5*	NA	GeneBody	Intron
cg08359464	3	128370361	2.91E-27	0.412	0.482	-0.070	*RPN1*	Shore	TSS1500	NA
cg21179618	9	139424642	2.92E-27	0.346	0.406	-0.060	*NOTCH1*	Shelf	GeneBody	Intron
cg08472633	16	88907370	2.98E-27	0.324	0.375	-0.051	*GALNS*	Shore	GeneBody	Intron
cg21727359	6	82459006	2.99E-27	0.328	0.382	-0.054	*FAM46A*	Shelf	3UTR	Exon
cg10287970	6	7115345	3.00E-27	0.329	0.380	-0.051	*RREB1*	NA	5UTR	Intron
cg19867914	2	144234430	3.09E-27	0.331	0.382	-0.051	*ARHGAP15*	NA	GeneBody	Intron
cg17161520	11	67174843	3.17E-27	0.758	0.703	0.055	*TBC1D10C*	Shelf	ncRNA	Intron
cg05949667	11	134126504	3.26E-27	0.487	0.543	-0.056	*ACAD8*	Shelf	GeneBody	Exon
cg02520804	3	71629054	3.26E-27	0.421	0.491	-0.070	*FOXP1*	Shore	5UTR	Intron
cg11809958	12	32654929	3.54E-27	0.435	0.507	-0.072	*FGD4*	NA	TSS200	NA
cg26306329	4	124400196	3.60E-27	0.251	0.303	-0.051	NA	NA	NA	NA
cg03348161	11	59824089	3.68E-27	0.339	0.406	-0.067	*MS4A3*	NA	TSS200	NA
cg18660064	1	23504632	3.69E-27	0.235	0.302	-0.066	NA	CpGIsland	NA	NA
cg18242288	4	54793620	3.71E-27	0.503	0.572	-0.068	NA	NA	NA	NA
cg19645639	1	113500384	3.89E-27	0.693	0.632	0.061	*SLC16A1*	Shelf	TSS1500	NA
cg16506970	4	6809109	3.90E-27	0.350	0.414	-0.065	*KIAA0232*	NA	5UTR	Intron
cg18437792	14	31699236	4.58E-27	0.310	0.369	-0.059	NA	NA	NA	NA
cg22243039	17	42147465	4.61E-27	0.229	0.282	-0.053	*G6PC3*	Shelf	TSS1500	NA
cg14685356	6	152639548	4.71E-27	0.345	0.400	-0.054	*SYNE1*	NA	GeneBody	Intron

cg09504873	16	66774626	4.74E-27	0.352	0.410	-0.057	*DYNC1LI2*	NA	GeneBody	Intron
cg23428738	12	4312337	4.96E-27	0.457	0.529	-0.072	NA	NA	NA	NA
cg17571559	3	11267525	4.97E-27	0.332	0.392	-0.060	*HRH1*	NA	TSS200	Intron
cg11125805	5	150678162	5.01E-27	0.309	0.374	-0.066	*SLC36A3*	NA	GeneBody	Exon
cg10599444	14	23305941	5.10E-27	0.279	0.338	-0.060	*MMP14*	CpGIsland	5UTR	Exon
cg13194425	17	7341936	5.18E-27	0.264	0.329	-0.065	*FGF11*	Shore	TSS1500	NA
cg24474182	3	151047307	5.19E-27	0.362	0.431	-0.069	*P2RY13*	NA	FirstExon	Exon
cg12405599	3	128370463	5.42E-27	0.416	0.494	-0.078	*RPN1*	Shore	TSS1500	NA
cg23463608	19	2607757	5.50E-27	0.559	0.493	0.066	*GNG7*	CpGIsland	5UTR	Intron
cg01766396	2	198436483	5.52E-27	0.364	0.431	-0.066	*RFTN2*	NA	3UTR	Exon
cg23304647	7	2778058	5.59E-27	0.270	0.335	-0.065	*GNA12*	Shelf	GeneBody	Intron
cg06059360	3	42657618	5.91E-27	0.442	0.525	-0.083	*NKTR*	NA	GeneBody	Intron
cg03143046	6	43758007	6.00E-27	0.340	0.404	-0.064	NA	NA	NA	NA
cg23189692	3	184050393	6.30E-27	0.297	0.352	-0.055	*EIF4G1*	Shelf	GeneBody	Intron
cg20242427	11	61115736	6.69E-27	0.366	0.448	-0.081	*DAK*	NA	3UTR	Exon
cg12678686	3	127327369	6.70E-27	0.338	0.401	-0.062	*MCM2*	Shelf	GeneBody	Intron
cg00444740	8	129162178	6.82E-27	0.319	0.375	-0.057	NA	NA	NA	NA
cg21082028	17	46659993	6.89E-27	0.204	0.264	-0.060	NA	CpGIsland	NA	NA
cg04226002	11	113953462	6.97E-27	0.331	0.389	-0.058	*ZBTB16*	Shore	GeneBody	Intron
cg23415995	4	128704651	7.03E-27	0.386	0.448	-0.062	*HSPA4L*	Shore	GeneBody	Intron
cg03215181	4	122873487	7.03E-27	0.221	0.277	-0.056	*TRPC3*	Shore	TSS1500	NA
cg16603916	1	21615863	7.04E-27	0.348	0.400	-0.052	*ECE1*	Shore	GeneBody	Intron
cg00796963	6	84936558	7.16E-27	0.369	0.427	-0.058	*KIAA1009*	Shore	5UTR	Intron
cg08534016	5	35771584	7.20E-27	0.494	0.556	-0.062	*SPEF2*	NA	GeneBody	Intron
cg11050793	12	124876650	7.22E-27	0.225	0.281	-0.056	*NCOR2*	Shelf	GeneBody	Intron
cg15377871	14	23588325	7.28E-27	0.502	0.558	-0.056	*CEBPE*	NA	5UTR	Exon
cg23939096	5	1555791	7.31E-27	0.324	0.393	-0.069	NA	Shelf	NA	NA
cg13852284	10	77548353	7.47E-27	0.431	0.489	-0.058	*C10orf11*	NA	GeneBody	Intron
cg16481332	7	2654053	7.51E-27	0.346	0.408	-0.061	*IQCE*	NA	3UTR	Exon
cg04079215	1	95095221	7.78E-27	0.366	0.418	-0.053	NA	NA	NA	NA
cg03249630	10	90611782	7.87E-27	0.345	0.412	-0.067	*ANKRD22*	NA	TSS200	NA
cg10807894	12	125169804	8.77E-27	0.729	0.675	0.053	NA	NA	NA	NA
cg19725377	14	69535979	8.99E-27	0.289	0.340	-0.052	*DCAF5*	NA	GeneBody	Intron
cg02061820	3	152046751	9.15E-27	0.244	0.299	-0.055	*MBNL1*	NA	GeneBody	Intron
cg01309569	1	5950209	9.19E-27	0.611	0.665	-0.054	*NPHP4*	Shore	GeneBody	Intron
cg20612299	8	131308633	9.33E-27	0.352	0.403	-0.050	*ASAP1*	NA	GeneBody	Intron
cg19595244	1	110526158	9.54E-27	0.386	0.440	-0.054	*AHCYL1*	Shore	TSS1500	NA
cg20121012	12	93516072	9.83E-27	0.490	0.545	-0.056	*LOC643339*	NA	ncRNA	Intron
cg20118717	6	33400505	9.86E-27	0.366	0.424	-0.059	*SYNGAP1*	NA	GeneBody	Exon
cg01135648	4	41983611	9.97E-27	0.456	0.514	-0.058	*DCAF4L1*	Shore	TSS200	NA
cg07474797	16	89185916	1.01E-26	0.314	0.369	-0.055	*ACSF3*	Shore	ncRNA	Intron
cg01448863	14	70264043	1.04E-26	0.302	0.363	-0.060	*SLC10A1*	NA	TSS200	NA
cg05193538	1	26097991	1.08E-26	0.238	0.289	-0.051	*MAN1C1*	CpGIsland	GeneBody	Intron
cg06436185	7	151442351	1.08E-26	0.297	0.370	-0.073	*PRKAG2*	NA	GeneBody	Intron
cg26861460	22	44575455	1.08E-26	0.338	0.397	-0.059	*PARVG*	NA	TSS1500	Intron
cg10852154	6	42421757	1.09E-26	0.274	0.330	-0.056	NA	Shore	NA	NA
cg04340435	13	114145973	1.11E-26	0.277	0.328	-0.051	*DCUN1D2*	CpGIsland	TSS1500	Intron
cg23334433	17	79129051	1.12E-26	0.377	0.463	-0.086	*AATK*	Shelf	GeneBody	Intron
cg06287548	21	34773372	1.13E-26	0.351	0.402	-0.050	NA	Shelf	NA	NA
cg22074114	3	134326341	1.16E-26	0.358	0.414	-0.056	*KY*	NA	GeneBody	Intron
cg20222562	6	113993995	1.20E-26	0.391	0.455	-0.065	NA	NA	NA	NA
cg23568192	10	44289380	1.21E-26	0.387	0.461	-0.073	NA	NA	NA	NA
cg25949447	2	128169860	1.27E-26	0.412	0.489	-0.077	NA	Shelf	NA	NA
cg06401414	8	141599436	1.39E-26	0.717	0.654	0.063	*EIF2C2*	NA	GeneBody	Intron
cg24800754	19	6234327	1.41E-26	0.273	0.325	-0.052	*MLLT1*	Shelf	GeneBody	Intron
cg07804470	13	99201496	1.42E-26	0.309	0.370	-0.061	*STK24*	NA	GeneBody	Intron
cg02307823	10	89675901	1.42E-26	0.384	0.453	-0.069	*PTEN*	NA	GeneBody	Intron
cg24591861	15	66697173	1.43E-26	0.246	0.302	-0.056	*MAP2K1*	NA	GeneBody	Intron
cg19499884	10	102760724	1.46E-26	0.335	0.389	-0.054	*LZTS2*	Shore	5UTR	Intron
cg05613718	7	38355100	1.51E-26	0.271	0.326	-0.055	NA	Shelf	NA	NA
cg07268332	11	10476494	1.57E-26	0.405	0.461	-0.056	*AMPD3*	Shelf	TSS200	Intron
cg01366985	6	25167695	1.62E-26	0.225	0.278	-0.053	NA	NA	NA	NA
cg14746387	5	172879299	1.67E-26	0.323	0.383	-0.060	NA	NA	NA	NA
cg08297985	16	85343488	1.73E-26	0.219	0.269	-0.050	NA	NA	NA	NA
cg21442998	3	11597936	1.74E-26	0.363	0.414	-0.052	*VGLL4*	NA	3UTR	Exon
cg13541713	4	119947251	1.80E-26	0.355	0.418	-0.063	*SYNPO2*	NA	GeneBody	Intron
cg25025181	1	246378510	1.85E-26	0.303	0.362	-0.059	*SMYD3*	NA	GeneBody	Intron
cg06494464	22	24992604	1.85E-26	0.560	0.620	-0.060	*GGT1*	Shelf	5UTR	Intron
cg21161403	8	1894604	1.86E-26	0.389	0.439	-0.051	*ARHGEF10*	Shelf	GeneBody	Intron
cg06379435	19	3344273	1.93E-26	0.351	0.431	-0.080	NA	NA	NA	NA
cg07212327	8	130947389	2.03E-26	0.457	0.537	-0.080	*FAM49B*	Shelf	ncRNA	Intron
cg16728539	12	2451169	2.05E-26	0.329	0.384	-0.055	*CACNA1C*	NA	GeneBody	Intron
cg25197500	17	80581805	2.11E-26	0.495	0.584	-0.089	*WDR45L*	NA	GeneBody	Intron
cg26780125	8	125388806	2.11E-26	0.285	0.338	-0.054	NA	Shelf	NA	NA
cg25389087	18	74824413	2.12E-26	0.432	0.511	-0.079	*MBP*	CpGIsland	5UTR	Intron
cg26118358	14	23321005	2.20E-26	0.335	0.388	-0.053	NA	NA	NA	NA
cg26877678	17	25856540	2.20E-26	0.375	0.435	-0.060	*KSR1*	NA	5UTR	Intron
cg00859441	12	113799660	2.27E-26	0.446	0.514	-0.068	*PLBD2*	Shelf	GeneBody	Intron

cg05057534	2	28497669	2.27E-26	0.336	0.391	-0.056	*BRE*	NA	GeneBody	Intron
cg19356022	1	154943932	2.29E-26	0.371	0.443	-0.073	*SHC1*	Shelf	5UTR	Intron
cg17344321	7	22617382	2.30E-26	0.247	0.304	-0.056	NA	NA	NA	NA
cg25953692	6	31695415	2.38E-26	0.240	0.299	-0.059	*DDAH2*	Shelf	GeneBody	Exon
cg08243626	10	6442501	2.39E-26	0.309	0.363	-0.054	NA	NA	NA	NA
cg13870520	4	77131705	2.57E-26	0.370	0.427	-0.057	*SCARB2*	Shelf	GeneBody	Intron
cg05978306	17	1373774	2.63E-26	0.284	0.348	-0.064	*MYO1C*	Shore	GeneBody	Exon
cg10800346	13	114829496	2.65E-26	0.327	0.395	-0.068	*RASA3*	NA	GeneBody	Intron
cg18444544	8	22852741	2.67E-26	0.309	0.360	-0.051	*RHOBTB2*	NA	GeneBody	Intron
cg19529732	12	122712101	2.95E-26	0.626	0.569	0.057	*DIABLO*	Shore	TSS200	NA
cg12413156	20	62368256	2.99E-26	0.659	0.598	0.061	*LIME1*	Shore	5UTR	Intron
cg10206397	20	2085344	3.04E-26	0.454	0.522	-0.068	*STK35*	Shore	GeneBody	Intron
cg07186962	6	11393478	3.12E-26	0.281	0.333	-0.052	NA	NA	NA	NA
cg14187687	14	55249770	3.24E-26	0.403	0.471	-0.069	*SAMD4A*	NA	GeneBody	Intron
cg25303150	10	134211874	3.38E-26	0.235	0.285	-0.050	*PWWP2B*	Shore	GeneBody	Intron
cg07814932	1	120437090	3.54E-26	0.499	0.566	-0.066	*ADAM30*	Shore	FirstExon	Exon
cg06033531	5	176980153	3.63E-26	0.382	0.451	-0.068	*FAM193B*	Shore	ncRNA	Intron
cg15248035	9	36169949	3.85E-26	0.351	0.415	-0.064	*CCIN*	Shelf	FirstExon	Exon
cg13736811	19	41873930	3.90E-26	0.295	0.348	-0.054	*TMEM91*	Shelf	5UTR	Intron
cg26605164	10	102821565	3.93E-26	0.404	0.468	-0.065	*KAZALD1*	CpGIsland	5UTR	Exon
cg14510299	1	27928494	4.04E-26	0.284	0.335	-0.051	*AHDC1*	Shore	5UTR	Intron
cg06374610	10	50328992	4.14E-26	0.687	0.636	0.051	NA	NA	NA	NA
cg22840076	19	41782156	4.16E-26	0.435	0.486	-0.052	*HNRNPUL1*	NA	GeneBody	Exon
cg25007705	1	21588799	4.18E-26	0.264	0.315	-0.051	*ECE1*	NA	GeneBody	Intron
cg16408081	11	67205869	4.62E-26	0.625	0.574	0.051	*PTPRCAP*	Shelf	TSS1500	Exon
cg19268453	6	32905114	4.70E-26	0.409	0.462	-0.053	*HLA-DMB*	NA	GeneBody	Exon
cg27123392	15	50570473	4.77E-26	0.419	0.486	-0.067	*GABPB1*	NA	3UTR	Exon
cg11597277	20	52492248	4.97E-26	0.584	0.669	-0.084	*SUMO1P1*	NA	ncRNA	Exon
cg06881398	1	55661307	5.04E-26	0.447	0.514	-0.067	*USP24*	NA	GeneBody	Intron
cg18997983	5	1109031	5.07E-26	0.366	0.420	-0.055	*SLC12A7*	CpGIsland	GeneBody	Intron
cg09694051	12	27181973	5.17E-26	0.444	0.532	-0.088	*MED21*	NA	3UTR	Exon
cg02889001	16	1519785	5.29E-26	0.339	0.401	-0.062	*CLCN7*	NA	GeneBody	Intron
cg27044455	5	149878183	5.35E-26	0.377	0.429	-0.053	NA	NA	NA	NA
cg09163720	10	134400506	5.49E-26	0.490	0.568	-0.078	*INPP5A*	NA	GeneBody	Intron
cg10094994	2	240162323	5.52E-26	0.280	0.336	-0.056	*HDAC4*	NA	GeneBody	Intron
cg02347002	5	138719090	5.70E-26	0.350	0.410	-0.060	*SLC23A1*	NA	TSS200	NA
cg23911433	5	134611106	5.74E-26	0.429	0.502	-0.073	NA	NA	NA	NA
cg25393284	2	942653	5.82E-26	0.709	0.658	0.050	NA	Shelf	NA	NA
cg07197230	22	17956641	5.93E-26	0.232	0.284	-0.052	*CECR2*	NA	FirstExon	Exon
cg01718139	19	54566838	6.34E-26	0.446	0.522	-0.076	*VSTM1*	NA	GeneBody	Intron
cg14609407	20	43883172	6.47E-26	0.323	0.383	-0.060	*SLPI*	NA	FirstExon	Exon
cg20740903	17	76170799	6.53E-26	0.398	0.461	-0.062	*TK1*	NA	3UTR	Exon
cg26396370	2	10194752	6.62E-26	0.505	0.590	-0.085	*KLF11*	NA	3UTR	Exon
cg02098075	8	49643972	6.68E-26	0.368	0.437	-0.069	*EFCAB1*	Shelf	GeneBody	Exon
cg22820233	19	55385581	6.81E-26	0.356	0.420	-0.063	*FCAR*	NA	5UTR	Exon
cg09417547	3	43289436	7.01E-26	0.402	0.468	-0.066	NA	NA	NA	NA
cg21814550	14	53170147	7.06E-26	0.425	0.492	-0.067	NA	Shelf	NA	NA
cg18812353	15	56385430	7.08E-26	0.503	0.556	-0.053	*RFX7*	NA	3UTR	Exon
cg09370867	1	27961680	7.25E-26	0.315	0.368	-0.054	*FGR*	CpGIsland	5UTR	Exon
cg03594078	8	22131675	7.35E-26	0.361	0.422	-0.061	*PIWIL2*	Shore	TSS1500	NA
cg08463297	17	19976435	7.56E-26	0.219	0.274	-0.055	*SPECC1*	NA	5UTR	Intron
cg04488111	1	154897518	7.61E-26	0.430	0.506	-0.076	*PMVK*	NA	3UTR	Exon
cg07409471	17	80833638	7.80E-26	0.483	0.549	-0.066	*TBCD*	Shelf	GeneBody	Intron
cg18897025	5	110413671	7.83E-26	0.414	0.482	-0.067	*TSLP*	NA	ncRNA	Exon
cg22152446	4	48754953	7.93E-26	0.375	0.444	-0.068	*FRYL*	NA	5UTR	Intron
cg21320567	8	67975880	7.95E-26	0.397	0.463	-0.066	*CSPP1*	Shore	TSS1500	NA
cg02381279	19	16394366	8.46E-26	0.308	0.375	-0.067	NA	Shore	NA	NA
cg17356733	21	34774627	8.47E-26	0.449	0.522	-0.072	*IFNGR2*	Shore	TSS1500	NA
cg01288724	5	142575122	8.53E-26	0.335	0.395	-0.060	*ARHGAP26*	NA	GeneBody	Intron
cg26841425	10	45958771	8.58E-26	0.381	0.444	-0.063	*MARCH8*	NA	GeneBody	Intron
cg15982099	11	47399813	8.66E-26	0.293	0.349	-0.056	*SPI1*	CpGIsland	GeneBody	Intron
cg06751366	16	50280723	9.42E-26	0.209	0.260	-0.051	NA	Shore	NA	NA
cg11183072	17	37894397	1.02E-25	0.184	0.240	-0.057	*GRB7*	NA	TSS1500	Intron
cg02012974	3	66492992	1.02E-25	0.331	0.387	-0.056	*LRIG1*	NA	GeneBody	Intron
cg00465970	3	129024601	1.03E-25	0.346	0.403	-0.057	NA	NA	NA	NA
cg17952939	1	9154250	1.07E-25	0.329	0.384	-0.055	NA	NA	NA	NA
cg22062741	8	1897075	1.08E-25	0.412	0.475	-0.063	*ARHGEF10*	Shelf	GeneBody	Intron
cg19266387	3	183596123	1.08E-25	0.234	0.293	-0.059	*PARL*	NA	GeneBody	Intron
cg04605532	1	234843592	1.12E-25	0.432	0.494	-0.063	NA	NA	NA	NA
cg17100176	1	207096358	1.12E-25	0.676	0.626	0.051	*FAIM3*	NA	TSS1500	NA
cg07375836	17	35717813	1.14E-25	0.254	0.306	-0.052	*ACACA*	Shore	5UTR	Intron
cg01231381	2	180130081	1.17E-25	0.428	0.485	-0.057	*SESTD1*	Shore	TSS1500	NA
cg21002528	11	45903754	1.17E-25	0.380	0.434	-0.054	*CRY2*	Shelf	3UTR	Exon
cg12212198	13	114302021	1.23E-25	0.234	0.290	-0.056	NA	Shore	NA	NA
cg01827633	2	219610103	1.28E-25	0.294	0.353	-0.058	*TTLL4*	NA	GeneBody	Intron
cg23137881	4	159857583	1.29E-25	0.431	0.502	-0.072	*C4orf45*	NA	GeneBody	Intron
cg26391219	11	3189207	1.36E-25	0.337	0.388	-0.050	NA	Shelf	NA	NA
cg05624376	2	169939876	1.36E-25	0.341	0.410	-0.069	*DHRS9*	NA	GeneBody	Exon

cg05255811	6	39192009	1.41E-25	0.356	0.419	-0.064	*KCNK5*	NA	GeneBody	Intron
cg03000848	16	396164	1.52E-25	0.805	0.744	0.061	*AXIN1*	NA	FirstExon	Exon
cg01393945	5	10457034	1.54E-25	0.352	0.415	-0.063	*ROPN1L*	NA	GeneBody	Intron
cg08126118	13	43930918	1.57E-25	0.351	0.418	-0.067	*ENOX1*	NA	GeneBody	Intron
cg23528247	3	99832772	1.58E-25	0.298	0.356	-0.058	*C3orf26*	NA	TSS1500	Intron
cg20184271	12	14413090	1.59E-25	0.387	0.447	-0.059	NA	NA	NA	NA
cg14115756	9	125795935	1.63E-25	0.283	0.336	-0.053	*GPR21*	NA	TSS1500	Intron
cg10099732	14	69256977	1.68E-25	0.680	0.625	0.055	*ZFP36L1*	CpGIsland	GeneBody	Exon
cg19284751	2	119699789	1.72E-25	0.305	0.355	-0.050	*MARCO*	NA	5UTR	Exon
cg24235882	4	54928822	1.78E-25	0.423	0.484	-0.062	*CHIC2*	Shore	GeneBody	Intron
cg25140783	1	24861872	1.85E-25	0.674	0.618	0.056	*RCAN3*	CpGIsland	3UTR	Exon
cg04947907	12	133345285	1.93E-25	0.196	0.253	-0.058	NA	NA	NA	NA
cg21821982	3	31547255	1.93E-25	0.340	0.406	-0.066	NA	NA	NA	NA
cg17928286	1	205414220	1.93E-25	0.277	0.331	-0.055	NA	NA	NA	NA
cg23924737	17	55928753	2.12E-25	0.431	0.489	-0.057	*MRPS23*	Shore	TSS1500	NA
cg16422316	16	71844250	2.28E-25	0.412	0.484	-0.072	*AP1G1*	Shore	TSS1500	NA
cg00335124	5	58652808	2.29E-25	0.360	0.428	-0.068	*PDE4D*	NA	GeneBody	Intron
cg05697976	12	29376483	2.30E-25	0.305	0.367	-0.062	*FAR2*	NA	TSS200	NA
cg12744859	17	46669492	2.32E-25	0.192	0.246	-0.054	*HOXB-AS3*	CpGIsland	ncRNA	Intron
cg02551745	3	156809115	2.49E-25	0.322	0.382	-0.060	*LOC100498859*	Shelf	ncRNA	Intron
cg01431057	1	153362927	2.53E-25	0.373	0.426	-0.053	*S100A8*	NA	FirstExon	Exon
cg26645401	11	9686514	2.56E-25	0.185	0.242	-0.057	*SWAP70*	Shore	GeneBody	Intron
cg17973115	1	25333445	2.61E-25	0.292	0.357	-0.065	NA	NA	NA	NA
cg04075726	2	69499425	2.62E-25	0.390	0.454	-0.064	NA	NA	NA	NA
cg13569051	9	124051703	2.63E-25	0.443	0.498	-0.055	*GSN*	NA	5UTR	Intron
cg09553839	10	74358778	2.70E-25	0.415	0.470	-0.055	*MICU1*	NA	5UTR	Intron
cg12209946	3	14987434	2.70E-25	0.422	0.488	-0.066	*FGD5-AS1*	Shore	ncRNA	Exon
cg10296205	6	159084032	2.74E-25	0.272	0.334	-0.062	*SYTL3*	NA	5UTR	Intron
cg06208288	1	58858074	2.78E-25	0.739	0.681	0.059	NA	NA	NA	NA
cg23815853	12	48147034	2.87E-25	0.424	0.488	-0.064	*RAPGEF3*	NA	GeneBody	Intron
cg12073436	1	206958014	2.93E-25	0.707	0.654	0.053	NA	NA	NA	NA
cg14611767	9	134127575	2.97E-25	0.433	0.499	-0.066	NA	NA	NA	NA
cg24058365	1	110923328	2.97E-25	0.340	0.401	-0.061	*SLC16A4*	NA	GeneBody	Intron
cg07231045	8	142219444	3.17E-25	0.414	0.478	-0.064	NA	Shore	NA	NA
cg02769705	1	202975059	3.22E-25	0.385	0.448	-0.064	*LOC401980*	Shore	ncRNA	Intron
cg09146459	6	32921200	3.24E-25	0.250	0.301	-0.051	*HLA-DMA*	NA	TSS1500	NA
cg04238983	11	65210600	3.27E-25	0.367	0.425	-0.059	NA	NA	NA	NA
cg13438337	9	78774369	3.35E-25	0.443	0.517	-0.074	*PCSK5*	NA	GeneBody	Intron
cg21088438	14	70264869	3.38E-25	0.327	0.386	-0.060	*SLC10A1*	NA	TSS1500	NA
cg00534022	2	8686087	3.44E-25	0.376	0.428	-0.052	NA	NA	NA	NA
cg14378231	1	98404032	3.89E-25	0.486	0.543	-0.057	NA	NA	NA	NA
cg00736299	16	4730465	3.99E-25	0.431	0.493	-0.062	*MGRN1*	Shelf	GeneBody	Intron
cg17900199	10	1156548	4.02E-25	0.685	0.634	0.051	*WDR37*	NA	GeneBody	Intron
cg10111352	16	16082095	4.05E-25	0.304	0.355	-0.052	*ABCC1*	NA	GeneBody	Intron
cg02266731	12	69357333	4.10E-25	0.262	0.313	-0.051	*CPM*	NA	TSS1500	NA
cg20203469	11	134126284	4.15E-25	0.532	0.605	-0.073	*ACAD8*	Shelf	GeneBody	Intron
cg02309841	3	11758425	4.28E-25	0.375	0.429	-0.054	*VGLL4*	Shelf	5UTR	Intron
cg20513976	20	62367893	4.42E-25	0.565	0.483	0.082	*LIME1*	Shore	TSS200	NA
cg12249234	17	25867613	4.65E-25	0.320	0.386	-0.065	*KSR1*	NA	5UTR	Intron
cg13221924	17	6495080	4.74E-25	0.378	0.435	-0.057	*KIAA0753*	NA	GeneBody	Intron
cg00479463	19	33726786	4.78E-25	0.673	0.617	0.056	NA	CpGIsland	NA	NA
cg05061769	15	59700360	4.83E-25	0.380	0.441	-0.061	NA	NA	NA	NA
cg25711558	7	101499638	5.14E-25	0.298	0.358	-0.060	*CUX1*	NA	GeneBody	Intron
cg03169059	2	240145022	5.17E-25	0.319	0.375	-0.056	*HDAC4*	NA	GeneBody	Intron
cg22022716	8	26276541	5.17E-25	0.510	0.563	-0.053	NA	NA	NA	NA
cg06810647	16	1665094	5.23E-25	0.186	0.238	-0.052	*CRAMP1L*	CpGIsland	GeneBody	Intron
cg12306086	4	106117747	5.36E-25	0.301	0.353	-0.053	*TET2*	NA	5UTR	Intron
cg23018755	17	79881529	5.43E-25	0.356	0.418	-0.062	*MAFG*	Shelf	5UTR	Intron
cg00423035	7	105319486	5.64E-25	0.319	0.372	-0.053	*ATXN7L1*	NA	GeneBody	Intron
cg16624482	21	43548126	5.70E-25	0.348	0.415	-0.068	*UMODL1*	CpGIsland	GeneBody	Intron
cg02788021	13	30882905	6.01E-25	0.310	0.360	-0.051	*KATNAL1*	Shore	TSS1500	NA
cg22313574	8	27468981	6.19E-25	0.224	0.287	-0.062	*CLU*	Shelf	ncRNA	Intron
cg18112953	20	47448545	6.25E-25	0.395	0.454	-0.059	NA	Shelf	NA	NA
cg14523804	17	75995968	6.30E-25	0.539	0.602	-0.063	NA	NA	NA	NA
cg17591816	1	172239092	6.80E-25	0.428	0.490	-0.062	*DNM3*	NA	GeneBody	Intron
cg08670658	11	67052992	7.02E-25	0.380	0.449	-0.068	*ADRBK1*	CpGIsland	3UTR	Exon
cg05580141	12	49071788	7.04E-25	0.394	0.454	-0.060	*KANSL2*	Shelf	GeneBody	Intron
cg06072036	11	822402	7.05E-25	0.334	0.393	-0.059	*PNPLA2*	Shelf	GeneBody	Exon
cg26478599	7	41747322	7.06E-25	0.393	0.453	-0.060	*INHBA-AS1*	Shore	ncRNA	Intron
cg00454592	2	12013993	7.51E-25	0.497	0.563	-0.067	NA	NA	NA	NA
cg08279189	6	28875370	7.53E-25	0.389	0.463	-0.074	*TRIM27*	NA	GeneBody	Intron
cg09379497	6	30624467	7.62E-25	0.435	0.489	-0.054	*DHX16*	NA	GeneBody	Exon
cg08944236	16	53242355	8.15E-25	0.262	0.314	-0.052	*CHD9*	NA	GeneBody	Intron
cg23731826	15	90371692	8.29E-25	0.435	0.492	-0.057	NA	NA	NA	NA
cg18934187	18	51882189	8.35E-25	0.509	0.578	-0.069	*STARD6*	Shore	TSS1500	NA
cg03626208	12	2443169	8.54E-25	0.321	0.371	-0.050	*CACNA1C*	NA	GeneBody	Intron
cg01212284	3	182814959	8.62E-25	0.530	0.585	-0.055	*MCCC1*	Shore	GeneBody	Intron
cg20001791	6	16239799	8.66E-25	0.305	0.374	-0.069	*GMPR*	Shore	GeneBody	Intron

cg00711072	17	46669489	8.94E-25	0.254	0.315	-0.061	*HOXB-AS3*	CpGIsland	ncRNA	Intron
cg01627405	19	2235127	8.98E-25	0.269	0.325	-0.056	*PLEKHJ1*	Shore	GeneBody	Intron
cg06465011	16	84860871	9.47E-25	0.366	0.430	-0.065	*CRISPLD2*	NA	5UTR	Intron
cg21224380	12	109240533	9.92E-25	0.436	0.496	-0.060	*SSH1*	NA	GeneBody	Intron
cg14039246	1	53365446	1.00E-24	0.341	0.398	-0.057	*ECHDC2*	NA	GeneBody	Intron
cg08220149	16	15766327	1.04E-24	0.252	0.309	-0.057	*NDE1*	NA	GeneBody	Intron
cg05557991	16	89003641	1.04E-24	0.207	0.257	-0.050	*CBFA2T3*	Shelf	GeneBody	Intron
cg24453664	11	33758413	1.08E-24	0.269	0.321	-0.053	*CD59*	Shore	TSS1500	NA
cg26437697	14	55143755	1.11E-24	0.479	0.539	-0.060	*SAMD4A*	NA	GeneBody	Intron
cg01521397	20	60590872	1.12E-24	0.390	0.459	-0.069	*TAF4*	Shelf	GeneBody	Intron
cg21210041	17	27443831	1.13E-24	0.433	0.483	-0.050	*MYO18A*	NA	GeneBody	Intron
cg27449572	10	100024278	1.20E-24	0.254	0.313	-0.059	*LOXL4*	Shelf	5UTR	Intron
cg07586235	1	200385219	1.23E-24	0.337	0.397	-0.060	NA	NA	NA	NA
cg02579959	15	100890963	1.28E-24	0.448	0.515	-0.067	NA	Shore	NA	NA
cg05413628	16	1521656	1.33E-24	0.344	0.431	-0.087	*CLCN7*	Shelf	GeneBody	Intron
cg15401418	17	75316383	1.38E-24	0.399	0.471	-0.072	*SEPT9*	NA	5UTR	Intron
cg03035167	2	201336269	1.38E-24	0.757	0.701	0.056	*SPATS2L*	NA	GeneBody	Intron
cg15835339	10	10828105	1.39E-24	0.462	0.533	-0.071	*SFTA1P*	NA	ncRNA	Intron
cg23128949	12	124986124	1.43E-24	0.294	0.346	-0.052	*NCOR2*	NA	5UTR	Intron
cg19118951	21	35575070	1.60E-24	0.271	0.325	-0.053	NA	Shore	NA	NA
cg05492904	15	51604503	1.68E-24	0.440	0.497	-0.057	*CYP19A1*	NA	5UTR	Intron
cg03658236	12	104063640	1.69E-24	0.435	0.506	-0.072	*STAB2*	NA	GeneBody	Intron
cg13573582	1	44887933	1.72E-24	0.401	0.460	-0.059	*RNF220*	Shelf	GeneBody	Intron
cg03322353	4	141069770	1.76E-24	0.394	0.456	-0.062	*MAML3*	Shelf	GeneBody	Intron
cg04695882	16	20756332	1.82E-24	0.294	0.349	-0.056	NA	Shelf	NA	NA
cg16393899	6	5136301	1.83E-24	0.393	0.458	-0.065	*LYRM4*	Shore	GeneBody	Intron
cg06392753	2	102783282	1.98E-24	0.320	0.376	-0.056	*IL1R1*	NA	GeneBody	Intron
cg19005210	6	41168960	1.98E-24	0.239	0.297	-0.058	*TREML2*	NA	TSS200	NA
cg01723892	1	233004866	2.03E-24	0.278	0.328	-0.050	NA	NA	NA	NA
cg06719445	14	89925468	2.05E-24	0.328	0.386	-0.058	*FOXN3*	NA	5UTR	Intron
cg05171937	12	27396765	2.15E-24	0.418	0.487	-0.069	*STK38L*	Shore	TSS1500	NA
cg08696931	12	123754071	2.20E-24	0.174	0.224	-0.050	*CDK2AP1*	Shore	ncRNA	Intron
cg00808648	14	105779910	2.22E-24	0.268	0.321	-0.053	*PACS2*	Shore	TSS1500	Intron
cg12213811	12	122251152	2.27E-24	0.558	0.610	-0.052	*SETD1B*	Shore	GeneBody	Intron
cg15167202	5	149997153	2.32E-24	0.434	0.488	-0.054	*SYNPO*	NA	TSS200	Intron
cg22033586	2	157292113	2.32E-24	0.326	0.403	-0.077	*GPD2*	CpGIsland	5UTR	Exon
cg17879101	10	126329354	2.37E-24	0.264	0.317	-0.053	*FAM53B*	NA	GeneBody	Intron
cg05888181	8	1952319	2.41E-24	0.363	0.427	-0.064	*KBTBD11*	Shore	3UTR	Exon
cg01261013	21	37691747	2.42E-24	0.454	0.518	-0.064	*MORC3*	Shore	TSS1500	NA
cg26405020	15	91427363	2.51E-24	0.265	0.316	-0.051	*FES*	Shelf	TSS1500	NA
cg18535410	13	113793268	2.66E-24	0.323	0.380	-0.057	*F10*	NA	GeneBody	Intron
cg19982684	3	128403035	2.66E-24	0.318	0.371	-0.053	NA	Shelf	NA	NA
cg05429448	3	101659630	2.84E-24	0.212	0.267	-0.055	NA	NA	NA	NA
cg26047334	2	218785909	2.91E-24	0.683	0.630	0.054	*TNS1*	NA	5UTR	Intron
cg19210276	11	57529465	3.12E-24	0.285	0.342	-0.057	*TMX2-CTNND1*	NA	ncRNA	Intron
cg15334250	3	184297495	3.38E-24	0.287	0.348	-0.061	*EPHB3*	Shelf	GeneBody	Exon
cg07377519	19	50269083	3.38E-24	0.269	0.323	-0.054	*AP2A1*	Shore	TSS1500	NA
cg12446199	1	167408841	3.44E-24	0.168	0.219	-0.051	*CD247*	CpGIsland	GeneBody	Intron
cg08905487	19	55013821	3.44E-24	0.384	0.464	-0.080	*LAIR2*	NA	TSS200	NA
cg01432609	17	78638554	3.85E-24	0.224	0.275	-0.051	*RPTOR*	NA	GeneBody	Intron
cg27076223	4	40632858	4.46E-24	0.310	0.361	-0.050	*RBM47*	Shore	TSS1500	NA
cg15964132	11	3175636	4.48E-24	0.403	0.468	-0.065	*OSBPL5*	NA	5UTR	Intron
cg08399444	12	13248548	4.50E-24	0.440	0.513	-0.073	*GSG1*	NA	5UTR	Exon
cg23426156	8	126557901	4.54E-24	0.769	0.718	0.051	NA	NA	NA	NA
cg01357222	6	137619143	4.59E-24	0.369	0.422	-0.053	NA	NA	NA	NA
cg22331200	17	56355362	4.60E-24	0.474	0.533	-0.059	*MPO*	CpGIsland	GeneBody	Exon
cg26706803	16	50293530	4.80E-24	0.254	0.315	-0.061	NA	NA	NA	NA
cg26348243	6	31540461	4.84E-24	0.642	0.573	0.069	*LTA*	NA	5UTR	Intron
cg11726288	1	11902283	4.86E-24	0.217	0.275	-0.057	*CLCN6*	Shelf	3UTR	Exon
cg03736807	12	54654045	4.96E-24	0.456	0.518	-0.063	*CBX5*	NA	TSS1500	Intron
cg15382933	11	33745399	5.01E-24	0.353	0.410	-0.057	*CD59*	NA	5UTR	Intron
cg05216056	6	28887836	5.01E-24	0.546	0.609	-0.062	*TRIM27*	Shelf	GeneBody	Exon
cg11842502	6	111862202	5.26E-24	0.415	0.483	-0.069	*TRAF3IP2-AS1*	NA	ncRNA	Intron
cg10588962	17	46667587	5.41E-24	0.286	0.352	-0.066	NA	Shore	NA	NA
cg21513254	6	30624502	5.50E-24	0.343	0.396	-0.054	*DHX16*	NA	GeneBody	Exon
cg18723409	11	1911547	5.53E-24	0.203	0.261	-0.058	*LSP1*	NA	3UTR	Intron
cg06303635	10	45958759	5.62E-24	0.394	0.454	-0.060	*MARCH8*	NA	GeneBody	Intron
cg01632240	8	42009223	5.86E-24	0.406	0.456	-0.050	*AP3M2*	Shore	TSS1500	NA
cg08132815	19	4950822	5.89E-24	0.530	0.611	-0.081	*UHRF1*	Shelf	GeneBody	Intron
cg15744128	2	27850964	5.97E-24	0.314	0.369	-0.055	*GPN1*	NA	TSS1500	Intron
cg25573640	14	64228599	6.28E-24	0.525	0.589	-0.064	NA	NA	NA	NA
cg25273707	11	76037066	6.46E-24	0.624	0.566	0.058	NA	Shelf	NA	NA
cg14066298	6	30297565	6.47E-24	0.405	0.459	-0.054	*TRIM39*	Shelf	GeneBody	Intron
cg27609217	11	46258039	6.53E-24	0.317	0.369	-0.052	NA	Shelf	NA	NA
cg13253847	17	56402238	6.90E-24	0.312	0.363	-0.051	*BZRAP1*	CpGIsland	GeneBody	Exon
cg12380764	1	206971195	7.03E-24	0.311	0.368	-0.057	*IL19*	NA	TSS1500	NA
cg24880665	1	202772164	7.43E-24	0.426	0.490	-0.063	*KDM5B*	Shelf	GeneBody	Intron
cg13085627	15	101597169	7.47E-24	0.547	0.601	-0.054	*LRRK1*	Shelf	GeneBody	Exon

cg02268192	14	92981666	7.55E-24	0.293	0.355	-0.061	*RIN3*	Shore	GeneBody	Intron
cg02243630	4	154386540	7.55E-24	0.244	0.296	-0.052	*KIAA0922*	Shore	TSS1500	NA
cg17567562	3	47687980	7.73E-24	0.500	0.573	-0.074	*SMARCC1*	NA	GeneBody	Intron
cg22044342	17	3819339	8.53E-24	0.367	0.428	-0.061	*P2RX1*	NA	GeneBody	Intron
cg08632810	8	141609470	8.59E-24	0.242	0.305	-0.064	*EIF2C2*	NA	GeneBody	Intron
cg00514723	20	40244770	8.68E-24	0.311	0.361	-0.051	*CHD6*	Shore	5UTR	Intron
cg10576245	1	233249267	8.69E-24	0.237	0.289	-0.052	*PCNXL2*	NA	GeneBody	Intron
cg12110801	19	5992284	8.72E-24	0.391	0.446	-0.055	*LOC100128568*	Shelf	ncRNA	Intron
cg16276850	17	38498914	8.92E-24	0.355	0.414	-0.059	*RARA*	Shelf	GeneBody	Intron
cg01561719	10	90611855	9.19E-24	0.353	0.405	-0.052	*ANKRD22*	NA	TSS200	NA
cg01554529	1	11722935	9.41E-24	0.304	0.373	-0.069	*FBXO6*	Shore	TSS1500	Exon
cg17206393	20	33681223	9.62E-24	0.275	0.332	-0.057	*TRPC4AP*	Shore	TSS1500	NA
cg08772789	17	75318655	9.63E-24	0.350	0.402	-0.053	*SEPT9*	NA	5UTR	Intron
cg02726291	3	128779596	9.70E-24	0.302	0.353	-0.052	*GP9*	NA	TSS200	NA
cg11916609	2	102927488	9.85E-24	0.388	0.456	-0.068	*IL1RL1*	NA	TSS1500	NA
cg10453850	6	32905320	9.89E-24	0.283	0.333	-0.051	*HLA-DMB*	NA	GeneBody	Intron
cg21471199	1	19218212	1.02E-23	0.466	0.529	-0.063	*ALDH4A1*	NA	GeneBody	Intron
cg10020520	16	30976186	1.04E-23	0.424	0.480	-0.056	*SETD1A*	NA	GeneBody	Exon
cg01700462	20	45179230	1.07E-23	0.291	0.347	-0.056	*OCSTAMP*	NA	TSS200	NA
cg03094134	6	30297174	1.10E-23	0.387	0.444	-0.057	*TRIM39*	Shelf	FirstExon	Exon
cg07280097	3	128779601	1.10E-23	0.296	0.349	-0.053	*GP9*	NA	TSS200	NA
cg00235484	6	136825415	1.17E-23	0.404	0.474	-0.071	*MAP7*	NA	GeneBody	Intron
cg00874051	21	35570816	1.19E-23	0.289	0.352	-0.063	NA	Shelf	NA	NA
cg17419815	12	27234924	1.28E-23	0.480	0.555	-0.075	*C12orf71*	NA	FirstExon	Exon
cg25218152	11	128554586	1.31E-23	0.402	0.465	-0.063	NA	NA	NA	NA
cg03305017	7	151036715	1.31E-23	0.329	0.386	-0.058	NA	Shelf	NA	NA
cg00916899	20	35944932	1.33E-23	0.170	0.222	-0.053	*MANBAL*	NA	3UTR	Exon
cg03171478	22	37572916	1.36E-23	0.301	0.364	-0.063	NA	NA	NA	NA
cg07658508	4	973177	1.39E-23	0.378	0.453	-0.075	*SLC26A1*	NA	3UTR	Exon
cg08730245	12	53902893	1.40E-23	0.509	0.561	-0.053	*NPFF*	NA	TSS1500	NA
cg18810664	10	31074367	1.47E-23	0.217	0.286	-0.069	NA	CpGIsland	NA	NA
cg25190513	12	123201362	1.50E-23	0.451	0.526	-0.074	*HCAR3*	NA	5UTR	Exon
cg20922701	18	11978319	1.51E-23	0.508	0.568	-0.060	NA	Shelf	NA	NA
cg07896832	8	67975874	1.54E-23	0.378	0.449	-0.071	*CSPP1*	Shore	TSS1500	NA
cg24690314	6	144018564	1.57E-23	0.491	0.556	-0.065	*PHACTR2*	NA	GeneBody	Intron
cg10752508	3	98313516	1.61E-23	0.353	0.412	-0.059	*CPOX*	Shore	TSS1500	NA
cg19989043	6	47468193	1.66E-23	0.291	0.344	-0.053	*CD2AP*	NA	GeneBody	Intron
cg11671363	5	148810177	1.68E-23	0.301	0.364	-0.063	*MIR143HG*	NA	ncRNA	Exon
cg03835987	4	111120249	1.81E-23	0.337	0.402	-0.065	*ELOVL6*	Shore	TSS1500	NA
cg20449670	1	153498959	1.83E-23	0.411	0.486	-0.075	NA	NA	NA	NA
cg00699986	20	25057444	2.07E-23	0.343	0.410	-0.067	*VSX1*	Shore	ncRNA	Intron
cg18349298	3	158450550	2.11E-23	0.325	0.388	-0.064	*RARRES1*	CpGIsland	TSS1500	NA
cg21406144	2	59919051	2.12E-23	0.488	0.543	-0.054	NA	NA	NA	NA
cg07133930	10	70821371	2.20E-23	0.271	0.325	-0.054	NA	NA	NA	NA
cg13366774	12	10336861	2.28E-23	0.517	0.590	-0.073	*TMEM52B*	NA	GeneBody	Intron
cg15042302	7	47981215	2.32E-23	0.340	0.405	-0.065	*PKD1L1*	NA	GeneBody	Intron
cg21852792	2	71678463	2.35E-23	0.354	0.417	-0.063	NA	Shelf	NA	NA
cg25104397	10	104535920	2.36E-23	0.251	0.309	-0.058	*WBP1L*	NA	5UTR	Exon
cg25198847	4	113192515	2.38E-23	0.440	0.510	-0.070	NA	NA	NA	NA
cg22848646	1	203171499	2.42E-23	0.362	0.415	-0.053	NA	NA	NA	NA
cg03664994	2	55246602	2.51E-23	0.331	0.391	-0.059	*RTN4*	NA	GeneBody	Intron
cg17974145	7	101671556	2.63E-23	0.474	0.527	-0.054	*CUX1*	NA	GeneBody	Intron
cg10011091	8	104424477	2.63E-23	0.301	0.357	-0.055	*SLC25A32*	Shelf	GeneBody	Intron
cg01283625	10	13568099	2.68E-23	0.416	0.483	-0.067	NA	Shore	NA	NA
cg02327530	7	100091786	2.86E-23	0.286	0.347	-0.062	*NYAP1*	Shore	3UTR	Exon
cg08545593	20	57582856	2.88E-23	0.327	0.387	-0.060	*CTSZ*	Shore	TSS1500	NA
cg18317439	17	643637	2.92E-23	0.343	0.415	-0.072	*FAM57A*	NA	GeneBody	Intron
cg10341242	16	50347849	3.04E-23	0.326	0.377	-0.050	*ADCY7*	NA	GeneBody	Intron
cg00637104	20	1785303	3.20E-23	0.399	0.458	-0.059	NA	Shore	NA	NA
cg04334723	19	13054427	3.41E-23	0.342	0.397	-0.055	*CALR*	Shelf	GeneBody	Exon
cg00686823	3	127311038	3.42E-23	0.293	0.358	-0.065	*TPRA1*	Shore	TSS1500	NA
cg19259111	11	46478214	3.52E-23	0.596	0.653	-0.057	*AMBRA1*	NA	GeneBody	Intron
cg16713889	14	74409552	3.57E-23	0.401	0.469	-0.067	*FAM161B*	NA	GeneBody	Intron
cg09128944	2	169967580	3.61E-23	0.309	0.368	-0.058	NA	NA	NA	NA
cg02802072	17	79229124	3.80E-23	0.297	0.357	-0.060	*SLC38A10*	NA	GeneBody	Intron
cg25446061	2	169967583	3.81E-23	0.282	0.344	-0.062	NA	NA	NA	NA
cg08837215	17	74443080	3.82E-23	0.462	0.521	-0.059	*UBE2O*	NA	GeneBody	Intron
cg19069360	12	1922058	3.82E-23	0.387	0.466	-0.078	*CACNA2D4*	NA	GeneBody	Intron
cg15188623	15	90608429	3.85E-23	0.311	0.376	-0.065	*ZNF710*	Shore	5UTR	Intron
cg26445561	11	92925402	3.87E-23	0.523	0.589	-0.067	*SLC36A4*	NA	GeneBody	Intron
cg25752703	3	128710390	3.91E-23	0.283	0.349	-0.066	*KIAA1257*	Shore	GeneBody	Intron
cg24743156	14	39734964	3.94E-23	0.347	0.404	-0.056	*CTAGE5*	Shore	TSS1500	Intron
cg21501175	3	194893148	3.95E-23	0.360	0.422	-0.062	*XXYLT1*	NA	GeneBody	Intron
cg24065504	10	90613015	4.00E-23	0.289	0.344	-0.055	*ANKRD22*	NA	TSS1500	NA
cg13801402	1	114429965	4.36E-23	0.475	0.550	-0.075	*AP4B1-AS1*	NA	ncRNA	Intron
cg14822490	10	124909889	4.36E-23	0.301	0.362	-0.061	*HMX2*	CpGIsland	3UTR	Exon
cg01950511	2	239289051	4.39E-23	0.345	0.415	-0.070	*TRAF3IP1*	NA	GeneBody	Intron
cg02717454	16	3928799	4.42E-23	0.222	0.274	-0.052	*CREBBP*	Shore	GeneBody	Intron

cg12106976	6	152002859	4.43E-23	0.227	0.283	-0.056	NA	NA	NA	NA
cg12656896	6	8438818	4.45E-23	0.285	0.341	-0.056	*LOC100506207*	Shelf	ncRNA	Intron
cg07092111	10	32621403	4.60E-23	0.655	0.605	0.050	*EPC1*	NA	GeneBody	Intron
cg12339328	20	18491484	4.64E-23	0.414	0.467	-0.053	*SEC23B*	Shelf	FirstExon	Exon
cg09098522	10	31435673	4.73E-23	0.302	0.355	-0.052	NA	NA	NA	NA
cg10435245	11	47399998	4.88E-23	0.346	0.408	-0.062	*SPI1*	CpGIsland	5UTR	Exon
cg00591868	19	19729048	4.97E-23	0.700	0.643	0.057	*PBX4*	Shore	GeneBody	Intron
cg20091384	19	2700927	5.14E-23	0.747	0.693	0.054	*GNG7*	Shore	5UTR	Intron
cg19584136	10	111975951	5.23E-23	0.396	0.459	-0.064	*MXI1*	NA	GeneBody	Intron
cg02952913	1	247336686	5.29E-23	0.317	0.371	-0.054	*ZNF124*	Shore	TSS1500	NA
cg02193956	4	103680201	5.64E-23	0.319	0.378	-0.059	*MANBA*	Shore	GeneBody	Intron
cg10909080	17	79881468	5.79E-23	0.334	0.389	-0.055	*MAFG*	Shelf	5UTR	Intron
cg01556552	1	10695686	6.00E-23	0.464	0.530	-0.066	NA	Shelf	NA	NA
cg18899117	2	206411031	6.22E-23	0.314	0.366	-0.052	*PARD3B*	NA	GeneBody	Intron
cg22438810	9	130911792	6.34E-23	0.464	0.515	-0.051	*LCN2*	NA	5UTR	Exon
cg01119452	7	37287850	6.45E-23	0.307	0.359	-0.051	*ELMO1*	NA	GeneBody	Intron
cg21201401	20	62367884	6.47E-23	0.563	0.468	0.095	*LIME1*	Shore	TSS200	NA
cg17500228	5	448790	6.68E-23	0.457	0.537	-0.081	*EXOC3*	NA	GeneBody	Intron
cg13758331	2	111893676	6.78E-23	0.310	0.374	-0.065	*BCL2L11*	NA	GeneBody	Intron
cg22136363	11	1911511	6.80E-23	0.202	0.270	-0.068	*LSP1*	NA	3UTR	Intron
cg23661721	14	95991371	7.46E-23	0.354	0.413	-0.059	NA	NA	NA	NA
cg25576997	14	56257750	7.53E-23	0.341	0.405	-0.063	*LINC00520*	NA	ncRNA	Intron
cg12927730	2	97260774	7.59E-23	0.635	0.579	0.056	*KANSL3*	NA	ncRNA	Exon
cg08594681	8	27468684	7.67E-23	0.244	0.308	-0.064	*CLU*	Shelf	ncRNA	Exon
cg17105014	2	127413363	7.69E-23	0.284	0.336	-0.052	*GYPC*	Shore	TSS200	NA
cg07145284	11	66084631	7.92E-23	0.259	0.318	-0.059	*CD248*	Shelf	TSS200	NA
cg25298189	19	935259	8.06E-23	0.419	0.471	-0.052	*ARID3A*	Shelf	GeneBody	Intron
cg09791504	1	151701849	8.57E-23	0.580	0.530	0.050	*RIIAD1*	NA	3UTR	Intron
cg12708994	6	116691460	8.58E-23	0.332	0.400	-0.068	*DSE*	Shore	5UTR	Intron
cg03565868	11	47400146	8.59E-23	0.345	0.404	-0.059	*SPI1*	Shore	TSS200	NA
cg10702418	7	157090031	9.50E-23	0.371	0.432	-0.061	NA	Shelf	NA	NA
cg16061354	17	1528465	9.58E-23	0.392	0.470	-0.078	*SLC43A2*	Shelf	GeneBody	Intron
cg12930392	3	196481615	1.01E-22	0.326	0.377	-0.051	*PAK2*	NA	5UTR	Intron
cg19254152	7	1083473	1.03E-22	0.202	0.252	-0.050	*C7orf50*	Shelf	GeneBody	Intron
cg07052737	2	26224428	1.06E-22	0.438	0.501	-0.064	NA	NA	NA	NA
cg00760203	17	37254921	1.06E-22	0.223	0.274	-0.051	*PLXDC1*	NA	GeneBody	Intron
cg17187521	12	125003379	1.07E-22	0.373	0.426	-0.053	*NCOR2*	Shore	5UTR	Intron
cg05363534	11	65210645	1.09E-22	0.367	0.421	-0.054	NA	NA	NA	NA
cg22675447	1	24745395	1.09E-22	0.349	0.406	-0.057	*NIPAL3*	Shelf	5UTR	Intron
cg25367558	6	41840102	1.10E-22	0.533	0.596	-0.063	*USP49*	NA	5UTR	Intron
cg19674851	2	224720888	1.11E-22	0.429	0.495	-0.066	NA	NA	NA	NA
cg16802439	16	88907184	1.13E-22	0.402	0.459	-0.057	*GALNS*	Shore	GeneBody	Intron
cg27416489	2	149823115	1.14E-22	0.459	0.524	-0.065	*KIF5C*	NA	GeneBody	Intron
cg10552964	6	35991802	1.30E-22	0.410	0.465	-0.055	*SLC26A8*	Shelf	5UTR	Intron
cg21887193	7	150786082	1.32E-22	0.205	0.262	-0.057	*AGAP3*	Shelf	GeneBody	Intron
cg16280132	6	31540459	1.32E-22	0.613	0.558	0.055	*LTA*	NA	5UTR	Intron
cg25124300	2	31607761	1.36E-22	0.429	0.484	-0.055	*XDH*	NA	GeneBody	Intron
cg07675031	11	47399893	1.36E-22	0.269	0.323	-0.054	*SPI1*	CpGIsland	FirstExon	Exon
cg00203037	3	16360321	1.38E-22	0.416	0.484	-0.068	*RFTN1*	NA	GeneBody	Intron
cg01441127	1	181012492	1.39E-22	0.478	0.546	-0.068	*MR1*	NA	GeneBody	Intron
cg26288715	6	135506834	1.40E-22	0.280	0.335	-0.055	*MYB*	Shelf	GeneBody	Intron
cg07737292	16	56892460	1.46E-22	0.352	0.433	-0.081	*MIR138-2*	NA	ncRNA	Exon
cg16145324	6	36020012	1.52E-22	0.463	0.515	-0.052	*MAPK14*	NA	GeneBody	Intron
cg05656486	1	161171383	1.54E-22	0.252	0.306	-0.054	*NDUFS2*	Shore	TSS1500	Intron
cg09697978	2	37501815	1.59E-22	0.358	0.418	-0.060	*PRKD3*	NA	GeneBody	Exon
cg14564351	6	135644405	1.59E-22	0.247	0.297	-0.051	*AHI1*	NA	GeneBody	Exon
cg23012600	1	244088110	1.66E-22	0.333	0.392	-0.059	*LOC339529*	NA	ncRNA	Intron
cg03187073	6	4890079	1.67E-22	0.304	0.358	-0.055	*CDYL*	NA	GeneBody	Intron
cg03106245	11	47399980	1.71E-22	0.388	0.459	-0.072	*SPI1*	CpGIsland	5UTR	Exon
cg06740950	3	171878318	1.77E-22	0.335	0.388	-0.052	*FNDC3B*	NA	GeneBody	Intron
cg22431093	12	32654992	1.80E-22	0.441	0.505	-0.064	*FGD4*	NA	TSS200	NA
cg05604874	17	80200785	1.82E-22	0.353	0.414	-0.061	*CSNK1D*	CpGIsland	3UTR	Exon
cg26697605	7	1850801	1.83E-22	0.293	0.348	-0.056	NA	NA	NA	NA
cg17694130	4	14858290	1.87E-22	0.714	0.658	0.056	NA	NA	NA	NA
cg04340595	1	9789174	1.91E-22	0.624	0.574	0.050	*CLSTN1*	Shore	3UTR	Exon
cg09978533	22	46465160	1.91E-22	0.253	0.311	-0.058	NA	Shore	NA	NA
cg16899991	3	127868625	2.09E-22	0.341	0.401	-0.060	NA	Shelf	NA	NA
cg07834069	17	72524918	2.09E-22	0.316	0.372	-0.056	*CD300LB*	NA	GeneBody	Intron
cg05799811	1	167487396	2.13E-22	0.737	0.683	0.055	*CD247*	NA	GeneBody	Intron
cg06634140	14	95956325	2.14E-22	0.428	0.485	-0.058	NA	NA	NA	NA
cg01479187	2	43158610	2.20E-22	0.271	0.323	-0.052	NA	NA	NA	NA
cg01249544	10	69372655	2.23E-22	0.692	0.641	0.051	*CTNNA3*	NA	GeneBody	Intron
cg15309361	7	2152625	2.26E-22	0.272	0.334	-0.062	*MAD1L1*	Shelf	GeneBody	Intron
cg01232748	4	54928819	2.37E-22	0.382	0.437	-0.055	*CHIC2*	Shore	GeneBody	Intron
cg17903590	1	28623081	2.38E-22	0.741	0.687	0.054	NA	NA	NA	NA
cg26561681	1	21376633	2.45E-22	0.330	0.396	-0.066	*EIF4G3*	NA	5UTR	Intron
cg25181170	10	25162298	2.51E-22	0.347	0.410	-0.063	*PRTFDC1*	NA	GeneBody	Intron
cg02997962	20	20036779	2.55E-22	0.337	0.405	-0.068	*CRNKL1*	Shelf	TSS200	Intron

cg21875980	1	231553510	2.58E-22	0.385	0.472	-0.087	*EGLN1*	Shelf	GeneBody	Intron
cg01081737	8	142238752	2.71E-22	0.448	0.503	-0.056	*SLC45A4*	Shelf	TSS200	NA
cg09042678	20	18117663	2.73E-22	0.329	0.387	-0.058	*PET117*	Shore	TSS1500	NA
cg20544852	6	28874479	2.75E-22	0.448	0.524	-0.076	*TRIM27*	NA	GeneBody	Intron
cg13088432	8	9522664	2.84E-22	0.367	0.434	-0.067	*TNKS*	NA	GeneBody	Intron
cg05949397	11	44976423	2.86E-22	0.426	0.490	-0.065	NA	Shelf	NA	NA
cg16922167	1	27961746	2.97E-22	0.414	0.468	-0.054	*FGR*	CpGIsland	TSS200	NA
cg23361127	5	73939283	3.01E-22	0.382	0.437	-0.055	NA	Shore	NA	NA
cg01058360	7	151442371	3.02E-22	0.430	0.511	-0.080	*PRKAG2*	NA	GeneBody	Intron
cg25936358	13	114301996	3.13E-22	0.255	0.310	-0.055	NA	Shore	NA	NA
cg00298324	20	822788	3.47E-22	0.202	0.253	-0.051	*FAM110A*	Shore	5UTR	Intron
cg16657152	8	125784769	3.50E-22	0.309	0.359	-0.050	NA	NA	NA	NA
cg00463732	17	3704621	3.66E-22	0.369	0.424	-0.055	*ITGAE*	NA	TSS200	NA
cg04776231	7	77168113	3.66E-22	0.377	0.450	-0.073	*PTPN12*	Shore	GeneBody	Intron
cg22153407	1	230290089	3.72E-22	0.439	0.494	-0.055	*GALNT2*	NA	GeneBody	Intron
cg13299325	6	447777	3.86E-22	0.705	0.652	0.052	NA	NA	NA	NA
cg26253663	22	25799504	4.00E-22	0.374	0.441	-0.067	NA	Shore	NA	NA
cg24926276	19	4539943	4.58E-22	0.443	0.497	-0.054	*LRG1*	Shelf	GeneBody	Intron
cg07790752	1	147101904	4.74E-22	0.394	0.465	-0.071	NA	NA	NA	NA
cg09803764	7	107572753	4.77E-22	0.363	0.415	-0.052	*LAMB1*	NA	GeneBody	Exon
cg16768966	17	9940227	4.84E-22	0.370	0.424	-0.054	*GAS7*	NA	TSS200	Intron
cg19132462	11	10476608	4.96E-22	0.327	0.379	-0.052	*AMPD3*	Shelf	TSS200	Intron
cg15945333	11	47399213	4.97E-22	0.294	0.344	-0.050	*SPI1*	Shore	GeneBody	Intron
cg01915791	19	36247526	4.99E-22	0.188	0.246	-0.058	*HSPB6*	Shore	GeneBody	Intron
cg15010903	17	76850256	5.00E-22	0.458	0.522	-0.064	*TIMP2*	Shore	3UTR	Exon
cg00782708	2	44933278	5.02E-22	0.513	0.564	-0.051	*CAMKMT*	NA	GeneBody	Intron
cg05372753	6	11804635	5.11E-22	0.442	0.503	-0.061	NA	NA	NA	NA
cg22707529	6	143999715	5.55E-22	0.199	0.264	-0.065	*PHACTR2*	Shore	GeneBody	Intron
cg15011899	13	111854118	5.76E-22	0.287	0.342	-0.055	*ARHGEF7*	NA	GeneBody	Intron
cg07239716	12	89744488	5.82E-22	0.226	0.285	-0.059	*DUSP6*	Shore	GeneBody	Exon
cg07306190	6	34760872	6.05E-22	0.161	0.214	-0.052	*UHRF1BP1*	Shore	GeneBody	Intron
cg16788865	12	46778801	6.08E-22	0.332	0.387	-0.056	NA	Shore	NA	NA
cg24493367	4	106635507	6.11E-22	0.310	0.362	-0.052	*GSTCD*	NA	5UTR	Intron
cg07079231	16	21169331	6.14E-22	0.544	0.612	-0.068	*TMEM159*	Shore	TSS1500	Intron
cg07417146	6	33400528	6.56E-22	0.541	0.612	-0.072	*SYNGAP1*	NA	GeneBody	Exon
cg08450017	3	45984838	6.62E-22	0.602	0.534	0.068	*CXCR6*	NA	TSS200	Intron
cg08129583	2	127413367	6.70E-22	0.303	0.356	-0.052	*GYPC*	Shore	TSS200	NA
cg11173131	20	45179226	6.72E-22	0.254	0.305	-0.051	*OCSTAMP*	NA	TSS200	NA
cg06812844	21	45773782	6.76E-22	0.312	0.365	-0.053	*TRPM2*	NA	GeneBody	Intron
cg08537367	16	87469199	6.80E-22	0.246	0.298	-0.053	*ZCCHC14*	NA	GeneBody	Intron
cg14603031	7	2563184	6.84E-22	0.221	0.271	-0.050	*LFNG*	Shelf	GeneBody	Intron
cg04364261	2	219233650	6.87E-22	0.252	0.306	-0.054	NA	Shore	NA	NA
cg06122230	20	47887219	7.15E-22	0.489	0.553	-0.064	*ZNFX1*	NA	GeneBody	Exon
cg02054108	2	61607478	7.25E-22	0.551	0.605	-0.053	*USP34*	NA	GeneBody	Exon
cg09205945	7	137662133	7.27E-22	0.356	0.421	-0.065	*CREB3L2*	NA	GeneBody	Intron
cg06043201	8	28974428	7.30E-22	0.366	0.426	-0.060	*KIF13B*	NA	GeneBody	Exon
cg15514380	21	38737243	7.78E-22	0.231	0.285	-0.054	NA	NA	NA	NA
cg17707487	13	114261869	7.78E-22	0.377	0.428	-0.051	*TFDP1*	Shore	GeneBody	Intron
cg01351315	17	46667737	7.93E-22	0.377	0.445	-0.067	NA	Shore	NA	NA
cg09183124	1	173092323	7.99E-22	0.363	0.426	-0.063	NA	NA	NA	NA
cg08438529	16	1052939	8.03E-22	0.653	0.599	0.054	NA	Shelf	NA	NA
cg25349990	1	229294380	8.14E-22	0.320	0.373	-0.053	NA	NA	NA	NA
cg18591228	11	3175552	8.20E-22	0.413	0.473	-0.060	*OSBPL5*	NA	5UTR	Intron
cg18297196	6	41168941	8.31E-22	0.185	0.245	-0.060	*TREML2*	NA	TSS200	NA
cg05162858	10	80697460	8.37E-22	0.267	0.319	-0.052	NA	NA	NA	NA
cg22585927	3	9958613	8.64E-22	0.236	0.286	-0.050	*IL17RC*	Shore	TSS200	NA
cg17785202	1	14870226	8.86E-22	0.410	0.485	-0.075	NA	NA	NA	NA
cg16547579	20	4954333	8.92E-22	0.210	0.264	-0.055	*SLC23A2*	NA	5UTR	Intron
cg03381237	7	155446645	9.06E-22	0.398	0.450	-0.051	*RBM33*	NA	GeneBody	Intron
cg27412857	10	11387394	9.98E-22	0.347	0.410	-0.063	NA	Shore	NA	NA
cg00789960	14	61227118	1.01E-21	0.494	0.563	-0.069	*MNAT1*	NA	GeneBody	Intron
cg01040749	10	134400440	1.03E-21	0.545	0.632	-0.087	*INPP5A*	NA	GeneBody	Intron
cg19293468	17	1973400	1.05E-21	0.707	0.652	0.055	*SMG6*	NA	ncRNA	Intron
cg14018648	17	7083015	1.05E-21	0.344	0.395	-0.050	*ASGR1*	NA	TSS200	NA
cg15520845	1	205688760	1.12E-21	0.501	0.574	-0.073	*NUCKS1*	NA	GeneBody	Exon
cg15132282	2	64488961	1.12E-21	0.284	0.334	-0.050	NA	NA	NA	NA
cg09357350	3	171894094	1.14E-21	0.434	0.491	-0.057	*FNDC3B*	NA	GeneBody	Intron
cg19638572	1	206733139	1.20E-21	0.538	0.483	0.055	*RASSF5*	Shelf	GeneBody	Intron
cg23735745	1	230248121	1.22E-21	0.274	0.326	-0.052	*GALNT2*	NA	GeneBody	Intron
cg11169848	11	67142030	1.24E-21	0.212	0.272	-0.060	*CLCF1*	Shore	TSS1500	Intron
cg17036458	17	28037399	1.38E-21	0.390	0.458	-0.067	*SSH2*	NA	GeneBody	Intron
cg09069499	8	26216910	1.38E-21	0.532	0.597	-0.065	*PPP2R2A*	NA	GeneBody	Intron
cg11054816	11	117183853	1.39E-21	0.361	0.420	-0.060	*BACE1*	Shelf	GeneBody	Intron
cg06992688	14	94491958	1.41E-21	0.415	0.487	-0.072	*OTUB2*	Shore	TSS1500	NA
cg13558754	19	36247867	1.48E-21	0.229	0.292	-0.064	*HSPB6*	Shore	FirstExon	Exon
cg04960169	17	2839005	1.53E-21	0.320	0.371	-0.051	*RAP1GAP2*	NA	GeneBody	Intron
cg05304531	1	26797576	1.54E-21	0.240	0.295	-0.055	*DHDDS*	Shore	3UTR	Exon
cg04603130	19	2550027	1.56E-21	0.359	0.426	-0.067	*GNG7*	Shelf	5UTR	Intron

cg11354682	19	10978833	1.65E-21	0.762	0.708	0.054	*C19orf38*	Shelf	GeneBody	Intron
cg27367170	10	5488628	1.79E-21	0.223	0.285	-0.062	*NET1*	CpGIsland	ncRNA	Exon
cg19851816	22	50657907	1.86E-21	0.221	0.279	-0.058	*TUBGCP6*	Shore	GeneBody	Intron
cg21088119	8	11251471	1.86E-21	0.702	0.651	0.051	*C8orf12*	NA	ncRNA	Intron
cg23587176	7	4746837	1.95E-21	0.559	0.616	-0.057	*FOXK1*	NA	GeneBody	Intron
cg15410418	5	59559180	1.99E-21	0.426	0.482	-0.056	*PDE4D*	NA	5UTR	Intron
cg26412374	11	63437874	2.01E-21	0.282	0.332	-0.051	*ATL3*	Shore	GeneBody	Intron
cg11826104	15	25650611	2.02E-21	0.526	0.586	-0.060	*UBE3A*	NA	GeneBody	Exon
cg20464143	5	16728219	2.05E-21	0.225	0.279	-0.054	*MYO10*	NA	GeneBody	Intron
cg19097880	11	128160637	2.07E-21	0.707	0.655	0.052	NA	NA	NA	NA
cg00777445	17	48970357	2.14E-21	0.450	0.517	-0.067	NA	NA	NA	NA
cg02156723	1	12100631	2.21E-21	0.816	0.762	0.054	NA	NA	NA	NA
cg01787084	16	87371097	2.29E-21	0.294	0.354	-0.060	*FBXO31*	Shelf	GeneBody	Intron
cg01328500	3	197639792	2.29E-21	0.820	0.762	0.058	*IQCG*	NA	GeneBody	Intron
cg16402415	15	57021851	2.50E-21	0.374	0.429	-0.055	*ZNF280D*	Shelf	5UTR	Intron
cg18715793	19	4518890	2.66E-21	0.454	0.522	-0.069	*PLIN4*	NA	TSS1500	NA
cg19342782	1	70821806	2.68E-21	0.378	0.448	-0.070	*HHLA3*	Shore	GeneBody	Intron
cg09763373	16	596899	2.75E-21	0.463	0.521	-0.058	*SOLH*	Shelf	FirstExon	Exon
cg02976588	1	150135546	2.88E-21	0.261	0.315	-0.054	NA	NA	NA	NA
cg09416908	11	86384670	3.08E-21	0.323	0.382	-0.059	*ME3*	Shore	TSS1500	NA
cg04904468	10	81202867	3.33E-21	0.465	0.524	-0.059	*ZCCHC24*	Shelf	GeneBody	Intron
cg07125981	10	35330496	3.42E-21	0.429	0.502	-0.074	*CUL2*	NA	GeneBody	Intron
cg14419424	10	65388604	3.79E-21	0.235	0.295	-0.060	NA	Shore	NA	NA
cg09706122	12	54071165	3.91E-21	0.185	0.240	-0.056	*ATP5G2*	Shore	TSS1500	NA
cg14088811	11	47399994	4.06E-21	0.312	0.378	-0.066	*SPI1*	CpGIsland	5UTR	Exon
cg07805500	6	151380818	4.11E-21	0.303	0.358	-0.055	*MTHFD1L*	NA	GeneBody	Intron
cg12684668	5	150403466	4.28E-21	0.444	0.496	-0.052	*GPX3*	Shelf	GeneBody	Intron
cg07428959	4	129783057	4.40E-21	0.388	0.450	-0.063	*PHF17*	NA	GeneBody	Exon
cg23351010	3	73109785	4.42E-21	0.412	0.467	-0.055	*PPP4R2*	NA	GeneBody	Intron
cg02185182	7	2185550	4.43E-21	0.420	0.485	-0.064	*MAD1L1*	Shore	GeneBody	Intron
cg02226672	16	68398533	4.49E-21	0.456	0.510	-0.054	*SMPD3*	NA	GeneBody	Intron
cg03853208	7	25989763	4.55E-21	0.240	0.307	-0.066	NA	Shore	NA	NA
cg27365701	12	89744150	4.58E-21	0.282	0.336	-0.054	*DUSP6*	Shore	GeneBody	Intron
cg27386431	1	200990482	4.81E-21	0.588	0.536	0.051	*KIF21B*	Shore	GeneBody	Intron
cg27058497	1	25291546	5.19E-21	0.623	0.568	0.055	*RUNX3*	NA	TSS200	NA
cg22534097	10	92681082	5.37E-21	0.382	0.438	-0.056	*ANKRD1*	NA	TSS200	NA
cg12488187	12	65671664	5.52E-21	0.437	0.498	-0.061	*MSRB3*	Shore	TSS1500	NA
cg26433561	1	110426090	5.65E-21	0.484	0.535	-0.052	NA	NA	NA	NA
cg26828017	16	22409023	6.21E-21	0.369	0.428	-0.059	NA	NA	NA	NA
cg14844236	12	123753212	6.30E-21	0.312	0.363	-0.051	*CDK2AP1*	Shelf	ncRNA	Intron
cg02287007	6	166877038	6.31E-21	0.284	0.336	-0.052	*RPS6KA2*	NA	GeneBody	Intron
cg21239001	5	118638618	6.43E-21	0.556	0.617	-0.061	*TNFAIP8*	NA	GeneBody	Intron
cg18004847	19	1155056	6.50E-21	0.422	0.475	-0.053	*SBNO2*	Shelf	5UTR	Intron
cg10028625	3	172494364	6.89E-21	0.420	0.487	-0.067	*ECT2*	NA	GeneBody	Intron
cg01586609	11	113846937	6.94E-21	0.407	0.496	-0.089	*HTR3A*	NA	GeneBody	Intron
cg07525077	14	21359943	7.13E-21	0.297	0.349	-0.052	*RNASE3*	NA	FirstExon	Exon
cg19047804	6	30297500	7.52E-21	0.553	0.616	-0.063	*TRIM39*	Shelf	FirstExon	Exon
cg06005892	11	3177622	7.88E-21	0.339	0.390	-0.051	*OSBPL5*	Shelf	5UTR	Intron
cg14919082	2	54901055	8.32E-21	0.314	0.370	-0.056	NA	NA	NA	NA
cg10499832	17	41450195	8.36E-21	0.357	0.407	-0.050	*LOC100130581*	Shelf	ncRNA	Intron
cg13622546	9	132402607	8.45E-21	0.531	0.582	-0.051	*ASB6*	Shore	GeneBody	Intron
cg14419046	9	90043268	8.82E-21	0.358	0.413	-0.055	NA	NA	NA	NA
cg04860291	3	53286651	9.14E-21	0.283	0.342	-0.059	*TKT*	Shelf	GeneBody	Intron
cg02311193	17	46660002	9.22E-21	0.381	0.432	-0.051	NA	CpGIsland	NA	NA
cg13525276	14	81426012	9.26E-21	0.407	0.331	0.076	*TSHR*	NA	GeneBody	Intron
cg18634760	13	46679242	9.29E-21	0.358	0.429	-0.071	*CPB2*	NA	TSS200	NA
cg20317748	15	40633124	9.37E-21	0.348	0.401	-0.053	*C15orf52*	NA	FirstExon	Exon
cg02931058	2	239047245	9.59E-21	0.693	0.642	0.051	*KLHL30*	Shelf	TSS200	NA
cg11789534	22	37655614	1.02E-20	0.316	0.379	-0.063	NA	CpGIsland	NA	NA
cg17766305	10	90147030	1.02E-20	0.292	0.352	-0.059	*RNLS*	NA	GeneBody	Intron
cg18122767	14	101191872	1.03E-20	0.695	0.645	0.050	*DLK1*	Shore	TSS1500	NA
cg05875463	18	60385273	1.16E-20	0.351	0.403	-0.052	*PHLPP1*	Shore	GeneBody	Intron
cg13134916	12	25707564	1.25E-20	0.312	0.369	-0.057	*IFLTD1*	NA	TSS1500	Intron
cg20063095	2	134977141	1.30E-20	0.374	0.430	-0.056	NA	NA	NA	NA
cg20430631	11	76340765	1.30E-20	0.305	0.359	-0.054	NA	NA	NA	NA
cg27099293	10	71895572	1.45E-20	0.551	0.602	-0.051	NA	Shelf	NA	NA
cg09791366	1	228661675	1.45E-20	0.329	0.381	-0.052	NA	Shelf	NA	NA
cg02830749	4	154451068	1.45E-20	0.293	0.346	-0.053	*KIAA0922*	NA	GeneBody	Intron
cg16553238	1	9154254	1.47E-20	0.254	0.312	-0.058	NA	NA	NA	NA
cg26962618	15	70767183	1.50E-20	0.256	0.308	-0.051	NA	NA	NA	NA
cg14669379	1	112058559	1.54E-20	0.167	0.217	-0.051	*ADORA3*	CpGIsland	GeneBody	Intron
cg17951713	8	130698161	1.58E-20	0.316	0.369	-0.053	NA	NA	NA	NA
cg04350675	6	111873161	1.58E-20	0.309	0.362	-0.053	*TRAF3IP2-AS1*	NA	ncRNA	Intron
cg16846069	12	6462681	1.64E-20	0.351	0.401	-0.050	*SCNN1A*	NA	GeneBody	Intron
cg03371275	11	95974543	1.66E-20	0.450	0.513	-0.063	*MAML2*	NA	GeneBody	Intron
cg02102075	16	474430	1.67E-20	0.465	0.529	-0.064	*RAB11FIP3*	Shore	TSS1500	NA
cg13581015	12	1769824	1.68E-20	0.353	0.420	-0.067	NA	Shore	NA	NA
cg22626169	6	6890951	1.74E-20	0.407	0.457	-0.051	NA	NA	NA	NA

cg04572930	7	4754834	1.76E-20	0.375	0.443	-0.068	*FOXK1*	Shelf	GeneBody	Intron
cg15621731	19	5074616	1.88E-20	0.333	0.383	-0.050	*KDM4B*	Shore	GeneBody	Intron
cg02188225	6	30459255	1.88E-20	0.696	0.646	0.050	*HLA-E*	Shore	GeneBody	Intron
cg05065948	4	26275089	1.90E-20	0.422	0.476	-0.053	NA	NA	NA	NA
cg12067024	1	153387689	1.92E-20	0.605	0.658	-0.052	*S100A7A*	NA	TSS1500	NA
cg05418105	22	50981406	2.12E-20	0.507	0.593	-0.086	NA	Shelf	NA	NA
cg08783253	17	40996565	2.13E-20	0.500	0.555	-0.055	*AOC2*	NA	TSS200	NA
cg02402436	6	31540051	2.23E-20	0.579	0.517	0.062	*LTA*	NA	5UTR	Exon
cg09129050	11	64478374	2.35E-20	0.439	0.502	-0.063	*NRXN2*	Shore	GeneBody	Intron
cg20765716	17	3704602	2.63E-20	0.405	0.474	-0.069	*ITGAE*	NA	TSS200	NA
cg20569108	10	21822303	2.73E-20	0.243	0.293	-0.050	*MLLT10*	Shore	TSS1500	NA
cg15699693	5	150054944	2.75E-20	0.513	0.581	-0.068	*MYOZ3*	Shelf	GeneBody	Intron
cg13381110	18	60646614	2.76E-20	0.529	0.620	-0.091	*PHLPP1*	NA	GeneBody	Exon
cg13524037	6	32904074	2.79E-20	0.292	0.356	-0.064	*HLA-DMB*	NA	GeneBody	Intron
cg06872313	12	14413185	2.90E-20	0.348	0.398	-0.050	NA	NA	NA	NA
cg03310874	7	4850260	3.50E-20	0.380	0.443	-0.062	*RADIL*	Shore	GeneBody	Intron
cg11781421	19	19367084	3.57E-20	0.392	0.448	-0.057	*HAPLN4*	Shore	3UTR	Exon
cg16478536	6	408730	3.68E-20	0.277	0.328	-0.051	*IRF4*	NA	3UTR	Exon
cg15724534	2	211018363	3.91E-20	0.513	0.569	-0.056	*KANSL1L*	NA	FirstExon	Exon
cg24746726	4	70726093	3.91E-20	0.403	0.465	-0.061	*SULT1E1*	NA	TSS1500	NA
cg21088259	17	81039990	3.95E-20	0.444	0.505	-0.061	*METRNL*	Shore	GeneBody	Intron
cg24131359	12	69346994	3.95E-20	0.502	0.585	-0.083	*CPM*	NA	5UTR	Intron
cg07043361	12	114404971	4.02E-20	0.300	0.351	-0.051	*RBM19*	Shore	TSS1500	NA
cg20367388	7	5258485	4.02E-20	0.316	0.374	-0.058	*WIPI2*	NA	GeneBody	Intron
cg04407248	16	31148196	4.21E-20	0.491	0.550	-0.059	*PRSS8*	NA	TSS1500	NA
cg20673721	17	25859382	4.41E-20	0.418	0.476	-0.059	*KSR1*	NA	5UTR	Intron
cg05111779	14	72990359	4.49E-20	0.329	0.386	-0.056	*RGS6*	NA	GeneBody	Intron
cg13589463	14	100920326	4.84E-20	0.498	0.559	-0.061	*WDR25*	NA	GeneBody	Intron
cg16272981	5	1489889	5.13E-20	0.803	0.741	0.062	*LPCAT1*	CpGIsland	GeneBody	Exon
cg10225149	19	15491808	5.28E-20	0.405	0.467	-0.063	*AKAP8*	Shore	TSS1500	Intron
cg01032675	19	3136430	5.36E-20	0.180	0.254	-0.075	*GNA15*	NA	5UTR	Exon
cg15704521	7	2773877	5.85E-20	0.513	0.589	-0.076	*GNA12*	Shore	GeneBody	Intron
cg20008101	14	55595227	6.34E-20	0.368	0.436	-0.068	*LGALS3*	Shore	TSS1500	NA
cg11707219	8	21905756	6.39E-20	0.366	0.425	-0.059	*FGF17*	CpGIsland	GeneBody	Exon
cg14001992	4	154073813	6.49E-20	0.262	0.322	-0.061	*TRIM2*	Shore	TSS1500	NA
cg10710218	1	55012989	6.86E-20	0.324	0.374	-0.050	*ACOT11*	NA	TSS1500	NA
cg15628518	8	145025059	7.15E-20	0.315	0.385	-0.070	*PLEC*	Shelf	TSS200	Intron
cg15356966	11	33913187	7.32E-20	0.295	0.346	-0.051	*LMO2*	NA	5UTR	Intron
cg07385778	3	72320634	7.84E-20	0.241	0.291	-0.050	NA	NA	NA	NA
cg06102602	12	96895446	7.93E-20	0.358	0.418	-0.060	NA	NA	NA	NA
cg07401324	11	48036852	8.36E-20	0.374	0.434	-0.060	*PTPRJ*	NA	GeneBody	Intron
cg07006075	6	16251782	8.74E-20	0.610	0.662	-0.052	*GMPR*	NA	GeneBody	Intron
cg17825714	6	31695903	8.75E-20	0.386	0.436	-0.051	*DDAH2*	CpGIsland	GeneBody	Intron
cg07363330	5	55148493	8.94E-20	0.359	0.411	-0.053	*IL31RA*	NA	GeneBody	Intron
cg24595580	15	100890996	9.26E-20	0.346	0.399	-0.053	NA	Shore	NA	NA
cg12401842	12	8276175	9.31E-20	0.386	0.445	-0.059	*CLEC4A*	NA	TSS200	NA
cg07377422	19	46318633	9.53E-20	0.160	0.211	-0.052	*RSPH6A*	CpGIsland	TSS200	NA
cg25664938	3	119030023	9.75E-20	0.427	0.489	-0.061	*ARHGAP31*	NA	GeneBody	Intron
cg03072035	10	73076664	9.94E-20	0.447	0.500	-0.052	NA	Shelf	NA	NA
cg21781157	20	47874111	1.00E-19	0.433	0.483	-0.050	*ZNFX1*	NA	GeneBody	Exon
cg23889010	20	43882990	1.02E-19	0.366	0.423	-0.057	*SLPI*	NA	GeneBody	Intron
cg00625110	16	53741731	1.07E-19	0.335	0.388	-0.053	*FTO*	Shelf	GeneBody	Intron
cg22438763	1	47900256	1.09E-19	0.234	0.298	-0.064	*FOXD2-AS1*	Shore	ncRNA	Exon
cg07453440	18	11948154	1.19E-19	0.288	0.355	-0.067	NA	CpGIsland	NA	NA
cg19676182	4	24981695	1.21E-19	0.334	0.391	-0.057	*CCDC149*	NA	5UTR	Exon
cg06100973	19	852114	1.21E-19	0.320	0.375	-0.055	*ELANE*	Shelf	TSS200	NA
cg02749463	17	46646359	1.22E-19	0.345	0.401	-0.057	*HOXB3*	NA	5UTR	Intron
cg14788242	3	127773954	1.24E-19	0.351	0.406	-0.054	*SEC61A1*	Shelf	GeneBody	Intron
cg04231085	16	85561302	1.25E-19	0.383	0.439	-0.056	NA	NA	NA	NA
cg04354689	16	2660830	1.25E-19	0.609	0.680	-0.071	*LOC652276*	CpGIsland	ncRNA	Exon
cg24762231	12	54690793	1.33E-19	0.355	0.428	-0.074	*NFE2*	NA	5UTR	Intron
cg08215318	16	86016387	1.33E-19	0.422	0.479	-0.056	NA	NA	NA	NA
cg02929855	12	1922067	1.33E-19	0.406	0.481	-0.075	*CACNA2D4*	NA	GeneBody	Intron
cg00242341	11	72447419	1.35E-19	0.398	0.458	-0.060	*ARAP1*	NA	5UTR	Intron
cg05479618	11	351125	1.39E-19	0.287	0.341	-0.054	NA	NA	NA	NA
cg12077963	17	4079306	1.50E-19	0.766	0.707	0.059	*ANKFY1*	NA	GeneBody	Intron
cg02538681	10	90611911	1.51E-19	0.458	0.515	-0.056	*ANKRD22*	NA	TSS200	NA
cg09273112	2	86732413	1.52E-19	0.511	0.586	-0.076	*RNF103-CHMP.*	NA	3UTR	Exon
cg04611801	17	56402029	1.52E-19	0.304	0.358	-0.054	*BZRAP1*	CpGIsland	GeneBody	Intron
cg15183258	15	42061865	1.54E-19	0.397	0.464	-0.067	*MGA*	NA	3UTR	Exon
cg17744604	1	206946166	1.54E-19	0.308	0.359	-0.051	*IL10*	NA	TSS1500	NA
cg14854517	12	48152428	1.56E-19	0.287	0.339	-0.052	*RAPGEF3*	Shore	TSS200	Exon
cg06755438	2	219885614	1.64E-19	0.361	0.413	-0.051	*CCDC108*	NA	GeneBody	Intron
cg25139649	1	2165579	1.67E-19	0.308	0.370	-0.061	*SKI*	NA	GeneBody	Intron
cg17108629	11	87908805	1.72E-19	0.217	0.269	-0.053	*RAB38*	Shore	TSS200	NA
cg20956594	13	29202889	1.74E-19	0.409	0.474	-0.065	NA	NA	NA	NA
cg18964319	6	36665554	1.76E-19	0.256	0.308	-0.052	*RAB44*	NA	TSS200	NA
cg17929770	19	46318514	1.78E-19	0.169	0.220	-0.051	*RSPH6A*	CpGIsland	5UTR	Exon

cg21167046	4	1295078	1.83E-19	0.329	0.384	-0.055	*MAEA*	NA	GeneBody	Intron
cg15100426	2	219187432	1.84E-19	0.520	0.576	-0.055	*PNKD*	Shore	TSS1500	Intron
cg16407947	10	5930543	1.94E-19	0.362	0.437	-0.075	*FBXO18*	Shore	TSS1500	Intron
cg16396191	13	42184765	2.03E-19	0.436	0.508	-0.071	*VWA8*	NA	GeneBody	Intron
cg11848173	12	56121015	2.04E-19	0.289	0.341	-0.052	*CD63*	Shore	GeneBody	Exon
cg23221723	8	22963367	2.17E-19	0.381	0.446	-0.065	*TNFRSF10C*	Shelf	GeneBody	Intron
cg12509499	17	2095179	2.39E-19	0.461	0.519	-0.059	*SMG6*	NA	GeneBody	Intron
cg07016549	16	1630488	2.48E-19	0.475	0.526	-0.051	*IFT140*	NA	GeneBody	Intron
cg11723077	6	158508188	2.73E-19	0.327	0.382	-0.055	*SYNJ2*	Shore	GeneBody	Intron
cg17222829	11	70433293	2.75E-19	0.336	0.387	-0.051	*SHANK2*	NA	GeneBody	Intron
cg19903805	14	92333771	2.83E-19	0.570	0.513	0.057	*TC2N*	NA	5UTR	Exon
cg12475879	17	14251824	2.84E-19	0.444	0.522	-0.079	NA	Shelf	NA	NA
cg16437908	2	85640810	2.85E-19	0.253	0.311	-0.058	*CAPG*	Shore	5UTR	Intron
cg20049422	15	26044289	2.87E-19	0.327	0.380	-0.053	*ATP10A*	NA	GeneBody	Intron
cg14191024	11	110070509	2.88E-19	0.496	0.549	-0.053	*RDX*	NA	GeneBody	Intron
cg00901982	2	70257298	2.95E-19	0.303	0.357	-0.053	*PCBP1-AS1*	NA	ncRNA	Intron
cg06766034	22	47082260	2.96E-19	0.369	0.443	-0.074	*CERK*	Shore	3UTR	Exon
cg21185109	17	7774912	3.02E-19	0.343	0.393	-0.050	NA	NA	NA	NA
cg27434890	6	135517041	3.54E-19	0.496	0.555	-0.058	*MYB*	NA	GeneBody	Exon
cg05252264	19	55385587	3.56E-19	0.280	0.336	-0.056	*FCAR*	NA	5UTR	Exon
cg07139162	16	70465351	3.64E-19	0.428	0.483	-0.055	*ST3GAL2*	NA	5UTR	Intron
cg07568841	7	30362781	3.74E-19	0.353	0.296	0.057	*ZNRF2*	NA	GeneBody	Intron
cg23975840	12	117042895	3.95E-19	0.527	0.594	-0.067	NA	NA	NA	NA
cg10220544	1	60171660	3.95E-19	0.323	0.379	-0.055	*FGGY*	NA	GeneBody	Intron
cg24807547	6	37504484	3.96E-19	0.329	0.381	-0.052	NA	Shore	NA	NA
cg01655008	14	93652954	4.17E-19	0.502	0.559	-0.058	*TMEM251*	Shore	FirstExon	Exon
cg13728797	8	126465410	4.26E-19	0.537	0.592	-0.056	NA	NA	NA	NA
cg14079545	6	33418310	4.33E-19	0.229	0.284	-0.055	*SYNGAP1*	Shelf	GeneBody	Intron
cg13466002	17	4621252	4.41E-19	0.430	0.494	-0.063	*ARRB2*	NA	ncRNA	Exon
cg04087207	20	31669392	4.65E-19	0.321	0.374	-0.053	*BPIFB4*	NA	FirstExon	Exon
cg11596902	11	123324834	4.69E-19	0.357	0.411	-0.055	NA	NA	NA	NA
cg27186013	4	95264127	4.76E-19	0.271	0.322	-0.052	*HPGDS*	NA	TSS200	NA
cg02901136	1	153348305	4.80E-19	0.461	0.512	-0.050	*S100A12*	NA	TSS1500	NA
cg21648069	12	1157718	5.10E-19	0.352	0.408	-0.056	*ERC1*	NA	GeneBody	Intron
cg22175006	2	223757281	5.37E-19	0.434	0.491	-0.057	*ACSL3*	NA	5UTR	Intron
cg03714676	8	48174649	5.52E-19	0.441	0.497	-0.056	*KIAA0146*	Shore	GeneBody	Intron
cg16520357	13	53625240	5.56E-19	0.494	0.551	-0.057	*OLFM4*	NA	3UTR	Exon
cg17840719	5	178368253	5.65E-19	0.187	0.245	-0.058	*ZNF454*	CpGIsland	5UTR	Exon
cg01477971	2	103405951	5.68E-19	0.358	0.415	-0.058	*TMEM182*	NA	GeneBody	Intron
cg13679804	3	59734950	6.32E-19	0.325	0.376	-0.052	NA	NA	NA	NA
cg00472373	3	9833414	6.66E-19	0.748	0.693	0.055	*ARPC4*	Shore	TSS1500	Intron
cg10804656	10	22623460	6.73E-19	0.461	0.530	-0.069	NA	CpGIsland	NA	NA
cg11952340	15	63814440	6.93E-19	0.448	0.503	-0.054	*USP3*	NA	GeneBody	Intron
cg19713460	22	39745530	7.22E-19	0.163	0.213	-0.050	*SYNGR1*	Shore	TSS1500	NA
cg00199007	20	61583910	8.01E-19	0.158	0.209	-0.051	*SLC17A9*	CpGIsland	TSS200	NA
cg06488150	7	6476003	8.18E-19	0.216	0.269	-0.054	*DAGLB*	NA	GeneBody	Exon
cg03408904	22	17956462	8.21E-19	0.354	0.413	-0.059	*CECR2*	NA	TSS200	NA
cg04936009	2	223846845	8.41E-19	0.466	0.521	-0.055	NA	NA	NA	NA
cg12016746	11	68084453	8.50E-19	0.396	0.461	-0.065	*LRP5*	Shelf	GeneBody	Intron
cg25256924	11	67205739	8.93E-19	0.682	0.626	0.056	*PTPRCAP*	Shelf	TSS1500	Exon
cg01498832	17	78682934	9.05E-19	0.412	0.471	-0.059	*RPTOR*	NA	GeneBody	Intron
cg15586392	8	142238691	9.34E-19	0.445	0.500	-0.055	*SLC45A4*	Shelf	TSS200	NA
cg24713204	19	57019373	9.49E-19	0.274	0.337	-0.063	*ZNF471*	CpGIsland	5UTR	Intron
cg06815950	12	111619455	9.49E-19	0.325	0.380	-0.055	*CUX2*	NA	GeneBody	Intron
cg00809164	16	56651049	9.52E-19	0.199	0.249	-0.050	NA	CpGIsland	NA	NA
cg20732703	7	50744032	9.86E-19	0.329	0.382	-0.053	*GRB10*	NA	5UTR	Intron
cg14039779	17	41857714	1.02E-18	0.421	0.473	-0.052	*DUSP3*	Shore	TSS1500	NA
cg15980656	8	97583236	1.03E-18	0.355	0.407	-0.051	*SDC2*	NA	GeneBody	Intron
cg18625627	14	81426015	1.05E-18	0.329	0.265	0.064	*TSHR*	NA	GeneBody	Intron
cg03376308	10	3497811	1.06E-18	0.369	0.419	-0.051	NA	Shelf	NA	NA
cg24742520	1	19506481	1.09E-18	0.383	0.444	-0.061	*UBR4*	NA	GeneBody	Intron
cg15285436	17	59226714	1.10E-18	0.370	0.435	-0.065	*BCAS3*	NA	GeneBody	Intron
cg00661777	7	106511741	1.19E-18	0.633	0.577	0.055	*PIK3CG*	Shelf	GeneBody	Intron
cg07786220	17	78683082	1.22E-18	0.287	0.339	-0.052	*RPTOR*	NA	GeneBody	Intron
cg21762728	6	170165905	1.30E-18	0.465	0.530	-0.065	*C6orf70*	NA	GeneBody	Intron
cg04399899	4	160189254	1.32E-18	0.321	0.374	-0.054	*RAPGEF2*	NA	5UTR	Exon
cg08425796	22	50981121	1.36E-18	0.289	0.352	-0.064	NA	Shelf	NA	NA
cg26305174	7	100463583	1.37E-18	0.341	0.393	-0.053	*TRIP6*	Shore	TSS1500	Exon
cg10827754	2	101038262	1.44E-18	0.377	0.434	-0.057	NA	Shelf	NA	NA
cg05162166	22	41684460	1.45E-18	0.387	0.437	-0.050	NA	Shore	NA	NA
cg16732654	17	79129022	1.48E-18	0.351	0.405	-0.055	*AATK*	Shelf	GeneBody	Intron
cg15891546	8	142179908	1.50E-18	0.501	0.570	-0.069	*DENND3*	Shelf	GeneBody	Intron
cg26118045	4	174427715	1.60E-18	0.262	0.315	-0.053	NA	Shore	NA	NA
cg07769015	8	142238770	1.76E-18	0.482	0.534	-0.052	*SLC45A4*	Shelf	TSS200	NA
cg12122241	20	1920407	1.76E-18	0.418	0.477	-0.059	*SIRPA*	Shelf	3UTR	Exon
cg02570354	3	31728749	1.78E-18	0.286	0.336	-0.050	*OSBPL10*	NA	GeneBody	Intron
cg26870438	10	6186015	1.78E-18	0.585	0.641	-0.057	*PFKFB3*	Shore	TSS1500	NA
cg11876048	17	55337654	1.87E-18	0.397	0.456	-0.058	*MSI2*	Shelf	GeneBody	Intron

cg14547461	8	48176671	1.90E-18	0.484	0.540	-0.056	*KIAA0146*	Shelf	GeneBody	Intron
cg13736939	7	157647241	2.04E-18	0.397	0.448	-0.051	*PTPRN2*	Shore	GeneBody	Intron
cg20954870	5	173070254	2.15E-18	0.426	0.485	-0.059	NA	NA	NA	NA
cg21949194	2	39355435	2.16E-18	0.500	0.444	0.057	NA	Shelf	NA	NA
cg03120555	7	630473	2.42E-18	0.285	0.336	-0.050	*PRKAR1B*	Shore	GeneBody	Intron
cg24312537	8	38831332	2.46E-18	0.434	0.492	-0.057	*HTRA4*	Shore	TSS1500	Exon
cg15986587	19	56130797	2.78E-18	0.422	0.483	-0.061	NA	Shelf	NA	NA
cg12754495	3	151102703	3.03E-18	0.349	0.405	-0.056	*P2RY12*	NA	TSS200	Intron
cg13441156	3	10335288	3.20E-18	0.439	0.503	-0.064	*GHRL*	NA	TSS1500	NA
cg14398691	17	37895771	3.22E-18	0.413	0.464	-0.051	*GRB7*	NA	GeneBody	Intron
cg18568335	14	105792505	3.23E-18	0.376	0.431	-0.055	*PACS2*	Shelf	GeneBody	Intron
cg11939496	1	160833560	3.42E-18	0.484	0.539	-0.055	*CD244*	NA	TSS1500	NA
cg05398700	14	102677141	3.82E-18	0.439	0.504	-0.065	*WDR20*	Shelf	GeneBody	Intron
cg27598107	2	102759782	3.90E-18	0.341	0.403	-0.062	NA	Shore	NA	NA
cg24257776	3	47051546	3.93E-18	0.313	0.369	-0.055	NA	CpGIsland	NA	NA
cg03432176	2	160655066	4.04E-18	0.327	0.385	-0.058	*CD302*	Shore	TSS1500	Intron
cg19672546	8	145025123	4.18E-18	0.204	0.254	-0.050	*PLEC*	Shelf	TSS200	Intron
cg09646392	13	108921052	4.26E-18	0.364	0.419	-0.055	*TNFSF13B*	NA	TSS1500	NA
cg11839815	3	127539037	4.40E-18	0.365	0.429	-0.064	*MGLL*	Shelf	GeneBody	Intron
cg16567676	4	95264074	4.47E-18	0.318	0.370	-0.052	*HPGDS*	NA	TSS200	NA
cg00855901	1	79085765	4.55E-18	0.167	0.217	-0.051	*IFI44L*	NA	TSS1500	NA
cg24025721	7	1220962	4.56E-18	0.482	0.534	-0.052	NA	NA	NA	NA
cg14567424	11	67349598	4.58E-18	0.520	0.573	-0.053	*GSTP1*	Shore	TSS1500	NA
cg17714799	4	110625080	4.60E-18	0.312	0.364	-0.052	*CASP6*	Shore	TSS1500	NA
cg04834204	2	69423553	4.61E-18	0.392	0.447	-0.055	*ANTXR1*	NA	GeneBody	Intron
cg06358171	1	54822008	4.97E-18	0.305	0.366	-0.061	*SSBP3*	CpGIsland	GeneBody	Intron
cg16847428	4	26095438	5.12E-18	0.513	0.586	-0.072	NA	NA	NA	NA
cg12691572	10	114574959	5.12E-18	0.247	0.304	-0.057	*VTI1A*	NA	GeneBody	Intron
cg17359629	13	114259310	5.14E-18	0.449	0.520	-0.071	*TFDP1*	Shore	GeneBody	Intron
cg02185248	17	78748494	5.33E-18	0.193	0.251	-0.057	*RPTOR*	NA	GeneBody	Intron
cg09069886	8	131000415	5.42E-18	0.431	0.482	-0.052	*FAM49B*	NA	ncRNA	Intron
cg20672711	3	110965888	5.62E-18	0.423	0.477	-0.054	NA	NA	NA	NA
cg07047068	11	844686	5.81E-18	0.272	0.327	-0.054	*TSPAN4*	Shore	5UTR	Intron
cg00153919	16	88859944	6.15E-18	0.255	0.316	-0.061	NA	NA	NA	NA
cg13300273	1	200842890	6.16E-18	0.235	0.316	-0.081	*GPR25*	CpGIsland	FirstExon	Exon
cg06902025	7	1545815	6.51E-18	0.230	0.283	-0.053	NA	Shore	NA	NA
cg19927816	19	46319153	6.53E-18	0.268	0.333	-0.065	*RSPH6A*	CpGIsland	TSS1500	Exon
cg14101485	19	3369759	6.77E-18	0.342	0.411	-0.068	*NFIC*	Shelf	GeneBody	Intron
cg18395636	11	87908785	6.87E-18	0.200	0.262	-0.062	*RAB38*	Shore	TSS200	NA
cg22109530	1	178310534	7.23E-18	0.643	0.590	0.053	*RASAL2*	NA	TSS200	Intron
cg10133935	19	1423753	7.74E-18	0.553	0.606	-0.054	*DAZAP1*	Shelf	GeneBody	Intron
cg26056277	2	166982925	8.16E-18	0.333	0.385	-0.052	*SCN1A*	NA	5UTR	Intron
cg13857354	2	241519705	8.27E-18	0.408	0.468	-0.060	NA	NA	NA	NA
cg03682581	2	85832389	8.41E-18	0.332	0.386	-0.053	*C2orf68*	Shelf	3UTR	Exon
cg12156512	19	47258676	1.02E-17	0.429	0.493	-0.064	*FKRP*	Shore	5UTR	Exon
cg27424995	1	27902555	1.02E-17	0.175	0.236	-0.061	*AHDC1*	CpGIsland	5UTR	Intron
cg14022913	4	178229651	1.03E-17	0.333	0.383	-0.051	*NEIL3*	Shore	TSS1500	NA
cg25771013	7	25989735	1.08E-17	0.194	0.255	-0.061	NA	Shore	NA	NA
cg22825487	6	133055183	1.21E-17	0.385	0.447	-0.062	*VNN3*	NA	ncRNA	Intron
cg14817490	5	392920	1.30E-17	0.256	0.309	-0.053	*AHRR*	NA	GeneBody	Intron
cg17614506	7	47803072	1.33E-17	0.740	0.689	0.051	*LINC00525*	NA	ncRNA	Exon
cg26609120	19	50848114	1.42E-17	0.582	0.529	0.053	NA	NA	NA	NA
cg19893585	8	145025064	1.42E-17	0.380	0.439	-0.058	*PLEC*	Shelf	TSS200	Intron
cg23506143	5	55761274	1.44E-17	0.366	0.419	-0.053	NA	NA	NA	NA
cg16548911	16	50347766	1.47E-17	0.269	0.323	-0.054	*ADCY7*	NA	GeneBody	Intron
cg13918640	9	94351568	1.49E-17	0.355	0.406	-0.051	NA	NA	NA	NA
cg10274029	16	85981336	1.53E-17	0.636	0.586	0.051	NA	Shore	NA	NA
cg17536595	6	43612908	1.56E-17	0.171	0.230	-0.060	*RSPH9*	CpGIsland	FirstExon	Exon
cg19837174	10	6389707	1.66E-17	0.691	0.637	0.054	NA	NA	NA	NA
cg13601309	18	77443784	1.71E-17	0.538	0.603	-0.064	*CTDP1*	Shelf	5UTR	Intron
cg06107293	15	22915986	1.89E-17	0.356	0.412	-0.056	*CYFIP1*	NA	5UTR	Intron
cg25031824	17	3819363	1.94E-17	0.413	0.467	-0.054	*P2RX1*	NA	GeneBody	Intron
cg06449094	11	822397	2.01E-17	0.470	0.525	-0.055	*PNPLA2*	Shelf	GeneBody	Exon
cg11833768	14	63940209	2.02E-17	0.366	0.420	-0.053	*PPP2R5E*	NA	GeneBody	Intron
cg13057898	1	3703894	2.07E-17	0.831	0.781	0.050	*LRRC47*	CpGIsland	GeneBody	Intron
cg02516101	22	38711891	2.15E-17	0.222	0.274	-0.052	*CSNK1E*	Shore	5UTR	Intron
cg16015295	17	78793479	2.20E-17	0.419	0.477	-0.058	*RPTOR*	Shelf	GeneBody	Intron
cg11006267	5	139017424	2.24E-17	0.111	0.179	-0.068	NA	CpGIsland	NA	NA
cg06526620	11	94278324	2.49E-17	0.446	0.496	-0.051	*FUT4*	CpGIsland	FirstExon	Exon
cg06784232	8	19463150	2.63E-17	0.502	0.564	-0.062	*CSGALNACT1*	Shelf	ncRNA	Intron
cg14209730	2	64632636	2.82E-17	0.532	0.589	-0.057	NA	NA	NA	NA
cg22941573	20	48761279	3.00E-17	0.460	0.513	-0.053	*TMEM189*	NA	GeneBody	Intron
cg21672292	8	145025128	3.07E-17	0.208	0.262	-0.054	*PLEC*	Shelf	TSS200	Intron
cg00591421	10	106468736	3.24E-17	0.595	0.654	-0.059	*SORCS3*	NA	GeneBody	Intron
cg23837109	10	75670435	3.32E-17	0.306	0.362	-0.056	*PLAU*	Shore	TSS1500	Exon
cg16436762	11	94307971	3.38E-17	0.339	0.395	-0.055	*PIWIL4*	NA	GeneBody	Intron
cg16942632	12	121726240	3.56E-17	0.354	0.409	-0.056	*CAMKK2*	NA	5UTR	Intron
cg26482939	19	3136710	3.57E-17	0.272	0.332	-0.061	*GNA15*	NA	GeneBody	Intron

cg06653796	20	62367805	3.60E-17	0.393	0.326	0.067	*LIME1*	Shore	TSS200	NA
cg05206633	5	177913434	3.62E-17	0.491	0.571	-0.080	*COL23A1*	NA	GeneBody	Intron
cg08930843	10	31182298	3.68E-17	0.354	0.404	-0.050	*ZNF438*	NA	5UTR	Intron
cg21932542	7	4850345	3.83E-17	0.434	0.494	-0.059	*RADIL*	Shore	GeneBody	Intron
cg27408171	6	2958808	3.84E-17	0.258	0.314	-0.056	*SERPINB6*	NA	GeneBody	Intron
cg10371414	16	79451857	4.30E-17	0.379	0.437	-0.058	NA	NA	NA	NA
cg20819482	8	9472057	4.40E-17	0.451	0.514	-0.063	*TNKS*	NA	GeneBody	Intron
cg13637654	10	103538719	4.66E-17	0.219	0.270	-0.051	*FGF8*	Shelf	5UTR	Intron
cg00084577	16	30721139	4.79E-17	0.517	0.574	-0.056	*SRCAP*	NA	GeneBody	Intron
cg20760057	6	130017522	4.93E-17	0.387	0.439	-0.052	*ARHGAP18*	NA	GeneBody	Intron
cg24592751	4	140088996	5.10E-17	0.411	0.464	-0.053	NA	NA	NA	NA
cg14171527	21	44473840	5.34E-17	0.354	0.406	-0.052	*CBS*	CpGIsland	3UTR	Exon
cg09950920	2	208734940	5.41E-17	0.534	0.594	-0.059	*PLEKHM3*	NA	GeneBody	Intron
cg17285931	2	240205408	5.49E-17	0.517	0.576	-0.059	*HDAC4*	NA	GeneBody	Intron
cg02138331	20	32893975	5.63E-17	0.599	0.652	-0.053	*AHCY*	Shelf	5UTR	Intron
cg22943115	5	149522468	5.68E-17	0.384	0.443	-0.059	*PDGFRB*	NA	5UTR	Intron
cg25769469	5	71643841	5.74E-17	0.299	0.350	-0.051	*PTCD2*	NA	GeneBody	Intron
cg10142520	11	65344604	5.80E-17	0.280	0.227	0.054	*EHBP1L1*	Shore	GeneBody	Intron
cg07319315	2	135149599	6.10E-17	0.543	0.596	-0.052	*MGAT5*	NA	GeneBody	Intron
cg19903071	2	160473251	6.47E-17	0.266	0.316	-0.050	*BAZ2B*	Shore	TSS200	NA
cg18397975	5	33997484	6.50E-17	0.545	0.603	-0.058	*AMACR*	NA	GeneBody	Intron
cg23128584	10	323649	6.68E-17	0.375	0.428	-0.054	*DIP2C*	NA	GeneBody	Intron
cg01752594	13	50696070	6.81E-17	0.458	0.519	-0.062	*DLEU2*	Shore	ncRNA	Intron
cg21233003	9	140057464	7.11E-17	0.304	0.376	-0.072	*GRIN1*	CpGIsland	GeneBody	Intron
cg23275644	5	10457079	7.60E-17	0.460	0.512	-0.052	*ROPN1L*	NA	GeneBody	Intron
cg27428551	12	122155069	8.08E-17	0.561	0.616	-0.055	*TMEM120B*	Shelf	GeneBody	Intron
cg01426713	6	106517533	8.41E-17	0.389	0.442	-0.054	NA	NA	NA	NA
cg06305891	3	195897904	8.57E-17	0.299	0.351	-0.052	NA	NA	NA	NA
cg24492202	4	2802612	9.19E-17	0.374	0.428	-0.054	*SH3BP2*	NA	5UTR	Intron
cg13216331	1	156025757	9.21E-17	0.329	0.381	-0.052	*LAMTOR2*	Shore	GeneBody	Intron
cg22712983	2	219187374	9.51E-17	0.586	0.663	-0.077	*PNKD*	Shore	TSS1500	Intron
cg15605096	1	19178957	1.04E-16	0.748	0.696	0.052	*TAS1R2*	Shore	GeneBody	Intron
cg12492653	11	11862867	1.05E-16	0.386	0.450	-0.064	*USP47*	Shore	TSS200	NA
cg09729012	3	72395774	1.11E-16	0.308	0.362	-0.055	NA	NA	NA	NA
cg10089801	2	203036235	1.16E-16	0.217	0.268	-0.051	NA	CpGIsland	NA	NA
cg16964946	11	27442973	1.21E-16	0.429	0.483	-0.053	*LGR4*	NA	GeneBody	Intron
cg02853355	16	85132373	1.23E-16	0.453	0.507	-0.053	*FAM92B*	NA	3UTR	Exon
cg15742737	2	64863651	1.24E-16	0.212	0.272	-0.059	*SERTAD2*	NA	FirstExon	Exon
cg25761791	19	3165800	1.27E-16	0.302	0.365	-0.063	NA	Shelf	NA	NA
cg26767974	2	240143979	1.30E-16	0.252	0.303	-0.051	*HDAC4*	NA	GeneBody	Intron
cg16660547	10	114574152	1.38E-16	0.519	0.577	-0.059	*VTI1A*	NA	GeneBody	Intron
cg10933959	15	100890907	1.43E-16	0.474	0.532	-0.058	*FLJ42289*	Shore	ncRNA	Exon
cg21964800	11	63272764	1.59E-16	0.458	0.515	-0.058	*LGALS12*	NA	TSS1500	Intron
cg11343072	2	160473528	1.63E-16	0.408	0.461	-0.052	*BAZ2B*	Shore	TSS1500	NA
cg03546163	6	35654363	1.78E-16	0.474	0.576	-0.102	*FKBP5*	Shore	5UTR	Intron
cg08632909	11	70048796	1.89E-16	0.423	0.491	-0.068	*FADD*	Shore	TSS1500	NA
cg23029655	12	53591756	2.03E-16	0.311	0.366	-0.055	*ITGB7*	CpGIsland	GeneBody	Intron
cg20351875	12	65015547	2.12E-16	0.373	0.423	-0.051	*RASSF3*	NA	GeneBody	Intron
cg08939850	17	78800806	2.16E-16	0.338	0.398	-0.060	*RPTOR*	Shore	GeneBody	Intron
cg25467336	7	2757616	2.46E-16	0.364	0.420	-0.055	NA	Shore	NA	NA
cg19935471	8	99048265	3.03E-16	0.480	0.537	-0.057	*MATN2*	NA	3UTR	Exon
cg20146241	1	24861604	3.16E-16	0.500	0.444	0.056	*RCAN3*	Shore	GeneBody	Exon
cg05681757	12	32655034	3.32E-16	0.423	0.476	-0.053	*FGD4*	NA	TSS200	NA
cg00121045	16	1416053	3.34E-16	0.386	0.443	-0.056	*UNKL*	Shore	3UTR	Exon
cg02827175	7	1986245	3.35E-16	0.577	0.637	-0.060	*MAD1L1*	NA	GeneBody	Intron
cg02668773	17	56356963	3.46E-16	0.447	0.503	-0.056	*MPO*	Shore	GeneBody	Exon
cg15341512	10	126396176	3.50E-16	0.464	0.518	-0.054	*FAM53B*	NA	5UTR	Intron
cg11239720	4	152967415	4.16E-16	0.404	0.461	-0.056	NA	NA	NA	NA
cg17467898	7	2289054	4.33E-16	0.273	0.331	-0.058	*NUDT1*	NA	GeneBody	Intron
cg02259081	12	53268433	4.41E-16	0.202	0.253	-0.051	NA	Shore	NA	NA
cg08886823	16	68270882	4.43E-16	0.178	0.230	-0.052	*ESRP2*	CpGIsland	TSS1500	NA
cg00754604	2	161230046	4.44E-16	0.491	0.544	-0.053	*RBMS1*	NA	GeneBody	Intron
cg07151747	2	158977473	4.99E-16	0.531	0.595	-0.065	*UPP2*	NA	GeneBody	Intron
cg18998911	3	111718245	5.59E-16	0.316	0.373	-0.057	*TAGLN3*	NA	5UTR	Intron
cg02737268	20	3780182	5.64E-16	0.517	0.451	0.066	*CDC25B*	Shelf	GeneBody	Intron
cg20941855	3	111718457	5.74E-16	0.466	0.523	-0.057	*TAGLN3*	NA	GeneBody	Intron
cg09595185	17	46667812	6.30E-16	0.357	0.412	-0.054	NA	Shore	NA	NA
cg07069934	15	89192956	6.38E-16	0.739	0.688	0.050	*ISG20*	NA	GeneBody	Intron
cg08398132	2	8557381	6.42E-16	0.479	0.529	-0.050	NA	NA	NA	NA
cg05089897	21	38886664	6.60E-16	0.564	0.617	-0.053	*DYRK1A*	NA	3UTR	Exon
cg14363249	9	75213807	6.88E-16	0.384	0.442	-0.058	*TMC1*	NA	5UTR	Intron
cg01022219	18	13641735	6.89E-16	0.465	0.516	-0.051	*C18orf1*	CpGIsland	GeneBody	Intron
cg15305633	20	45319350	7.05E-16	0.416	0.480	-0.064	*TP53RK*	Shore	TSS1500	NA
cg05644602	2	74795186	7.24E-16	0.496	0.548	-0.052	*C2orf65*	NA	GeneBody	Intron
cg19677302	12	33048259	7.53E-16	0.511	0.563	-0.052	*PKP2*	Shore	GeneBody	Intron
cg17142183	2	102608192	8.03E-16	0.338	0.389	-0.051	*IL1R2*	NA	TSS200	NA
cg06760238	10	134400036	8.04E-16	0.341	0.394	-0.053	*INPP5A*	NA	GeneBody	Intron
cg17478979	6	149772150	8.23E-16	0.412	0.475	-0.063	*ZC3H12D*	CpGIsland	GeneBody	Exon

cg26607031	1	9602701	8.46E-16	0.406	0.460	-0.053	*SLC25A33*	Shelf	GeneBody	Intron
cg06218079	17	80834228	8.84E-16	0.433	0.507	-0.074	*TBCD*	NA	GeneBody	Intron
cg20336172	7	2773782	9.82E-16	0.288	0.338	-0.051	*GNA12*	Shore	GeneBody	Intron
cg23548201	3	195623792	9.89E-16	0.472	0.530	-0.057	*TNK2*	Shore	TSS1500	Intron
cg05389236	6	36635087	1.03E-15	0.389	0.439	-0.050	NA	NA	NA	NA
cg02287710	14	102027660	1.06E-15	0.349	0.403	-0.054	*DIO3*	CpGIsland	TSS200	NA
cg04586126	5	176734633	1.11E-15	0.385	0.445	-0.060	*MXD3*	Shelf	GeneBody	Exon
cg22375763	19	4540003	1.17E-15	0.400	0.453	-0.054	*LRG1*	Shelf	FirstExon	Exon
cg25541528	7	1545819	1.27E-15	0.295	0.350	-0.054	NA	Shore	NA	NA
cg13467628	2	365559	1.29E-15	0.446	0.509	-0.063	NA	NA	NA	NA
cg02879423	2	224806760	1.35E-15	0.298	0.350	-0.051	*WDFY1*	Shelf	GeneBody	Intron
cg16000989	4	41983716	1.41E-15	0.449	0.502	-0.053	*DCAF4L1*	Shore	5UTR	Exon
cg25416319	17	37080373	1.45E-15	0.408	0.461	-0.052	NA	NA	NA	NA
cg05648510	17	943342	1.45E-15	0.422	0.476	-0.054	*ABR*	NA	GeneBody	Intron
cg12058385	2	144448686	1.46E-15	0.433	0.495	-0.062	*ARHGAP15*	NA	GeneBody	Intron
cg23181573	17	3863976	1.48E-15	0.411	0.463	-0.052	*ATP2A3*	Shelf	GeneBody	Intron
cg04422741	3	151102836	1.48E-15	0.352	0.405	-0.053	*P2RY12*	NA	TSS1500	Exon
cg19586698	8	92097851	1.54E-15	0.548	0.608	-0.060	*OTUD6B*	NA	3UTR	Exon
cg03479209	3	58020999	1.54E-15	0.509	0.459	0.050	*FLNB*	NA	GeneBody	Intron
cg03906115	2	33359529	1.55E-15	0.267	0.318	-0.051	*LTBP1*	NA	TSS200	Intron
cg24012880	11	44880910	1.55E-15	0.364	0.419	-0.055	*TSPAN18*	NA	5UTR	Intron
cg24107728	1	53760337	1.58E-15	0.581	0.530	0.051	*LRP8*	NA	GeneBody	Intron
cg15448894	6	52840469	1.59E-15	0.351	0.401	-0.050	NA	NA	NA	NA
cg24442740	1	27902069	1.63E-15	0.187	0.243	-0.057	*AHDC1*	CpGIsland	5UTR	Intron
cg22241838	11	60680006	1.72E-15	0.626	0.569	0.057	*TMEM109*	Shore	TSS1500	NA
cg00929635	20	44035918	1.76E-15	0.369	0.422	-0.052	*DBNDD2*	CpGIsland	GeneBody	Intron
cg16729631	8	131000261	1.82E-15	0.663	0.722	-0.059	*FAM49B*	NA	ncRNA	Intron
cg21815704	1	193075249	1.84E-15	0.388	0.441	-0.053	*GLRX2*	Shore	TSS1500	NA
cg13682345	1	90172255	2.00E-15	0.337	0.388	-0.051	*LRRC8C*	NA	GeneBody	Intron
cg14306650	9	129829146	2.06E-15	0.465	0.517	-0.051	*RALGPS1*	NA	GeneBody	Intron
cg15227911	17	7792059	2.07E-15	0.334	0.282	0.052	*CHD3*	Shelf	TSS200	Intron
cg21861151	2	239478365	2.14E-15	0.331	0.397	-0.066	NA	NA	NA	NA
cg08591265	17	56082867	2.46E-15	0.687	0.633	0.054	*SRSF1*	Shore	ncRNA	Exon
cg08722383	7	5594654	2.67E-15	0.312	0.363	-0.051	NA	CpGIsland	NA	NA
cg11341144	3	185656289	2.92E-15	0.458	0.525	-0.067	*TRA2B*	Shore	TSS1500	NA
cg11147155	1	150595206	3.04E-15	0.357	0.408	-0.051	*ENSA*	NA	3UTR	Exon
cg14454680	3	99794010	3.11E-15	0.342	0.398	-0.055	*FILIP1L*	NA	5UTR	Intron
cg09287629	2	203036208	3.23E-15	0.300	0.353	-0.053	NA	CpGIsland	NA	NA
cg10583119	19	54237755	3.58E-15	0.567	0.516	0.051	NA	NA	NA	NA
cg24733624	12	9517562	4.05E-15	0.666	0.616	0.050	NA	NA	NA	NA
cg02343628	15	93790914	4.15E-15	0.388	0.457	-0.068	NA	NA	NA	NA
cg12094903	6	32808689	4.15E-15	0.562	0.510	0.052	*PSMB8*	Shore	3UTR	Exon
cg17125472	11	69259831	4.17E-15	0.459	0.513	-0.054	NA	Shore	NA	NA
cg09315878	1	1152580	4.26E-15	0.796	0.743	0.052	*SDF4*	CpGIsland	3UTR	Exon
cg13429260	11	73356744	4.27E-15	0.409	0.459	-0.051	*PLEKHB1*	NA	TSS1500	NA
cg17723958	12	124429295	4.29E-15	0.438	0.494	-0.057	*CCDC92*	NA	5UTR	Intron
cg10502231	1	224363449	4.39E-15	0.248	0.299	-0.051	NA	NA	NA	NA
cg24383418	3	27770852	5.26E-15	0.306	0.362	-0.056	NA	Shore	NA	NA
cg08400494	13	111318490	5.46E-15	0.296	0.347	-0.051	*CARS2*	NA	GeneBody	Intron
cg08900409	19	18475669	5.54E-15	0.415	0.474	-0.060	*PGPEP1*	NA	3UTR	Exon
cg06939451	5	81043172	5.58E-15	0.461	0.511	-0.051	*SSBP2*	Shelf	GeneBody	Intron
cg26177213	7	4754566	6.33E-15	0.360	0.412	-0.053	*FOXK1*	Shelf	GeneBody	Intron
cg00357551	5	169407472	6.34E-15	0.416	0.470	-0.054	*DOCK2*	NA	GeneBody	Intron
cg06927812	13	41718870	7.25E-15	0.440	0.497	-0.057	NA	NA	NA	NA
cg14181409	17	9802860	7.91E-15	0.358	0.410	-0.052	*RCVRN*	NA	GeneBody	Intron
cg09455342	17	80346849	8.32E-15	0.465	0.518	-0.053	NA	Shelf	NA	NA
cg07971820	17	4621234	8.58E-15	0.414	0.476	-0.062	*ARRB2*	NA	ncRNA	Exon
cg02392124	10	28876249	9.44E-15	0.384	0.435	-0.051	*WAC*	NA	GeneBody	Intron
cg11224765	22	50971109	9.80E-15	0.236	0.287	-0.051	*ODF3B*	Shore	TSS200	NA
cg22833612	2	9487886	9.99E-15	0.393	0.443	-0.051	*ASAP2*	NA	GeneBody	Intron
cg14045860	11	35158283	1.07E-14	0.494	0.550	-0.057	NA	Shelf	NA	NA
cg10702770	1	39283780	1.08E-14	0.286	0.337	-0.051	NA	CpGIsland	NA	NA
cg25019722	6	37503610	1.22E-14	0.310	0.368	-0.058	NA	CpGIsland	NA	NA
cg12602909	3	4512309	1.26E-14	0.318	0.376	-0.059	NA	Shelf	NA	NA
cg16275295	15	101661984	1.42E-14	0.235	0.289	-0.054	NA	NA	NA	NA
cg19418318	19	17219073	1.44E-14	0.416	0.477	-0.060	*MYO9B*	NA	GeneBody	Intron
cg26169783	13	34371837	1.62E-14	0.615	0.560	0.055	NA	NA	NA	NA
cg15649236	17	46657504	1.66E-14	0.302	0.356	-0.054	NA	Shelf	NA	NA
cg07569918	1	212002970	1.73E-14	0.440	0.497	-0.057	*LPGAT1*	Shore	5UTR	Intron
cg22286764	3	37428639	1.74E-14	0.595	0.652	-0.057	NA	NA	NA	NA
cg19429405	10	73497609	1.84E-14	0.422	0.472	-0.051	*C10orf105*	NA	TSS200	Intron
cg21816330	17	27044629	1.85E-14	0.168	0.221	-0.053	*RAB34*	CpGIsland	5UTR	Exon
cg25472296	10	129076569	2.14E-14	0.493	0.545	-0.052	*DOCK1*	NA	GeneBody	Intron
cg27342781	16	84566279	2.25E-14	0.439	0.498	-0.058	NA	NA	NA	NA
cg22033476	2	43532275	2.27E-14	0.382	0.437	-0.055	*THADA*	NA	GeneBody	Intron
cg27576485	17	40558063	2.28E-14	0.395	0.450	-0.055	*PTRF*	Shore	GeneBody	Intron
cg24507266	8	145027948	2.42E-14	0.273	0.324	-0.051	*PLEC*	CpGIsland	GeneBody	Intron
cg25902939	19	18544350	2.52E-14	0.256	0.313	-0.057	*SSBP4*	Shelf	GeneBody	Intron

cg09830866	16	771714	2.73E-14	0.585	0.528	0.056	*FAM173A*	CpGIsland	GeneBody	Intron
cg22446264	6	13766814	2.75E-14	0.584	0.645	-0.062	NA	Shelf	NA	NA
cg01294808	5	3599686	3.00E-14	0.209	0.261	-0.052	*IRX1*	CpGIsland	GeneBody	Exon
cg27255275	11	129766154	3.22E-14	0.380	0.431	-0.052	*NFRKB*	Shore	TSS1500	NA
cg15609237	2	179395885	3.32E-14	0.466	0.519	-0.053	*TTN-AS1*	NA	ncRNA	Intron
cg08965143	2	24308246	3.37E-14	0.347	0.408	-0.061	*TP53I3*	Shore	TSS1500	NA
cg10239163	2	25527366	3.58E-14	0.498	0.557	-0.059	*DNMT3A*	NA	GeneBody	Intron
cg16313807	18	77723226	3.68E-14	0.739	0.680	0.058	*HSBP1L1*	Shore	TSS1500	NA
cg05954830	1	159838031	3.77E-14	0.388	0.447	-0.059	NA	NA	NA	NA
cg01141721	20	36010619	3.80E-14	0.421	0.475	-0.053	*SRC*	Shore	5UTR	Intron
cg12599598	4	185923887	4.16E-14	0.407	0.460	-0.052	NA	Shelf	NA	NA
cg06126721	17	1478065	4.35E-14	0.367	0.429	-0.062	*SLC43A2*	Shore	3UTR	Exon
cg06168950	3	154737057	4.96E-14	0.445	0.502	-0.057	NA	NA	NA	NA
cg26996569	12	121829743	5.30E-14	0.447	0.380	0.067	NA	NA	NA	NA
cg02088785	13	99223336	5.42E-14	0.762	0.712	0.050	*STK24*	NA	GeneBody	Intron
cg03450844	7	150416671	5.51E-14	0.340	0.400	-0.060	*GIMAP1-GIMA*	Shore	GeneBody	Intron
cg02368812	6	3019900	5.79E-14	0.425	0.489	-0.064	*NQO2*	NA	3UTR	Exon
cg22195627	19	12305869	6.56E-14	0.160	0.218	-0.058	NA	CpGIsland	NA	NA
cg11478024	6	35004780	6.79E-14	0.465	0.521	-0.055	*ANKS1A*	NA	GeneBody	Intron
cg04088945	7	157090146	6.83E-14	0.462	0.513	-0.051	NA	Shelf	NA	NA
cg04397884	14	56666067	7.21E-14	0.394	0.446	-0.052	*PELI2*	NA	GeneBody	Intron
cg14189391	2	25527347	7.23E-14	0.468	0.533	-0.065	*DNMT3A*	NA	GeneBody	Intron
cg16292768	8	27467783	7.42E-14	0.528	0.578	-0.050	*CLU*	NA	ncRNA	Intron
cg19942731	22	45609421	7.58E-14	0.331	0.383	-0.053	*KIAA0930*	NA	TSS1500	Intron
cg18067134	3	71084634	8.52E-14	0.498	0.554	-0.055	*FOXP1*	NA	GeneBody	Intron
cg23281529	13	114823829	8.97E-14	0.349	0.409	-0.060	*RASA3*	NA	GeneBody	Intron
cg15001032	7	50632994	9.21E-14	0.508	0.455	0.053	*DDC*	NA	5UTR	Exon
cg00044505	8	48649086	9.33E-14	0.344	0.397	-0.053	NA	Shore	NA	NA
cg08110693	6	36407533	9.39E-14	0.474	0.535	-0.061	*PXT1*	Shelf	5UTR	Intron
cg07176692	1	113500329	9.68E-14	0.548	0.494	0.054	*SLC16A1*	Shelf	TSS1500	NA
cg02578087	3	8671361	1.03E-13	0.795	0.740	0.054	*C3orf32*	NA	GeneBody	Exon
cg04703221	16	69967063	1.07E-13	0.351	0.405	-0.053	*WWP2*	NA	GeneBody	Intron
cg17789193	19	14533491	1.11E-13	0.323	0.379	-0.056	NA	Shelf	NA	NA
cg20728490	10	98064175	1.12E-13	0.545	0.478	0.067	*DNTT*	NA	5UTR	Exon
cg07685563	19	4954869	1.12E-13	0.623	0.675	-0.052	*UHRF1*	CpGIsland	GeneBody	Intron
cg23479922	5	16179633	1.28E-13	0.229	0.299	-0.070	*MARCH11*	CpGIsland	FirstExon	Exon
cg15818008	11	112126170	1.28E-13	0.404	0.456	-0.052	*C11orf34*	NA	GeneBody	Exon
cg12045829	17	7452926	1.35E-13	0.617	0.566	0.051	*TNFSF12*	Shore	GeneBody	Intron
cg25826463	19	3369820	1.36E-13	0.412	0.463	-0.051	*NFIC*	Shelf	GeneBody	Intron
cg18655633	19	48107418	1.48E-13	0.682	0.631	0.050	NA	Shelf	NA	NA
cg09696044	20	55968294	1.61E-13	0.361	0.303	0.058	*RBM38*	Shelf	GeneBody	Intron
cg15219811	10	104259328	1.79E-13	0.503	0.554	-0.051	*ACTR1A*	Shelf	GeneBody	Intron
cg06600725	12	48690915	1.82E-13	0.299	0.360	-0.061	NA	NA	NA	NA
cg25397054	7	2874568	1.98E-13	0.358	0.424	-0.066	*GNA12*	NA	GeneBody	Intron
cg08832851	12	92535822	2.06E-13	0.482	0.430	0.052	*LOC256021*	Shelf	5UTR	Intron
cg19582538	6	38127734	2.18E-13	0.497	0.559	-0.062	NA	NA	NA	NA
cg13521018	10	134429846	2.26E-13	0.634	0.581	0.052	*INPP5A*	NA	GeneBody	Intron
cg09351156	6	18436845	2.27E-13	0.363	0.418	-0.055	*RNF144B*	NA	GeneBody	Intron
cg15084543	1	79472408	2.40E-13	0.307	0.372	-0.066	*ELTD1*	CpGIsland	5UTR	Exon
cg13718729	9	140056619	2.51E-13	0.281	0.333	-0.052	*GRIN1*	CpGIsland	GeneBody	Intron
cg04771100	8	38832335	2.61E-13	0.289	0.346	-0.057	*HTRA4*	CpGIsland	GeneBody	Intron
cg20464719	19	17889512	2.71E-13	0.292	0.362	-0.071	*FCHO1*	NA	GeneBody	Exon
cg00982136	2	201726139	2.86E-13	0.506	0.451	0.055	*CLK1*	Shelf	ncRNA	Exon
cg17394978	5	131824502	2.93E-13	0.503	0.452	0.050	*IRF1*	Shore	GeneBody	Intron
cg23621097	17	1962236	2.94E-13	0.155	0.208	-0.053	*HIC1*	CpGIsland	3UTR	Exon
cg03044533	11	96065844	3.01E-13	0.515	0.572	-0.057	*MAML2*	NA	GeneBody	Intron
cg03156546	16	24759640	3.21E-13	0.355	0.407	-0.052	*TNRC6A*	NA	GeneBody	Intron
cg25351606	6	100917427	3.27E-13	0.230	0.290	-0.060	NA	CpGIsland	NA	NA
cg10603275	3	239665	3.42E-13	0.148	0.199	-0.051	*CHL1*	CpGIsland	5UTR	Intron
cg04967578	18	13641894	3.57E-13	0.485	0.541	-0.056	*C18orf1*	CpGIsland	GeneBody	Intron
cg26314089	12	66567930	3.58E-13	0.545	0.600	-0.054	NA	Shelf	NA	NA
cg02145701	16	88038949	3.67E-13	0.408	0.459	-0.051	*BANP*	Shore	GeneBody	Intron
cg13623290	6	4779680	3.93E-13	0.466	0.519	-0.053	*CDYL*	Shelf	GeneBody	Intron
cg17239057	1	224363575	4.12E-13	0.399	0.450	-0.051	NA	NA	NA	NA
cg05494467	5	140892308	4.26E-13	0.189	0.241	-0.052	*PCDHGA1*	Shore	3UTR	Exon
cg11937033	7	155150681	4.34E-13	0.464	0.523	-0.059	NA	NA	NA	NA
cg11576590	1	152011357	4.41E-13	0.379	0.434	-0.055	NA	Shelf	NA	NA
cg09287328	10	134231487	4.41E-13	0.436	0.490	-0.054	NA	NA	NA	NA
cg20813518	1	81929622	4.65E-13	0.385	0.440	-0.055	NA	NA	NA	NA
cg25282976	17	79005047	4.70E-13	0.352	0.403	-0.051	*BAIAP2-AS1*	Shore	ncRNA	Exon
cg25341726	16	28518331	5.27E-13	0.392	0.442	-0.050	*IL27*	NA	TSS200	NA
cg17074014	17	3704494	5.28E-13	0.391	0.447	-0.057	*ITGAE*	NA	5UTR	Exon
cg04450052	6	170525426	5.39E-13	0.678	0.626	0.052	NA	NA	NA	NA
cg20907614	8	29914963	5.52E-13	0.588	0.639	-0.051	NA	NA	NA	NA
cg02852791	16	710674	6.71E-13	0.357	0.429	-0.073	*WDR90*	Shore	GeneBody	Exon
cg07481491	19	3607130	7.66E-13	0.264	0.315	-0.051	*TBXA2R*	CpGIsland	TSS1500	NA
cg20469139	17	29297458	8.11E-13	0.321	0.378	-0.057	*RNF135*	Shore	TSS1500	NA
cg04781916	2	48013473	8.95E-13	0.598	0.656	-0.059	*MSH6*	Shore	GeneBody	Intron

cg26872780	2	149478494	9.22E-13	0.448	0.500	-0.052	*EPC2*	NA	GeneBody	Intron
cg01347228	17	29297391	1.01E-12	0.289	0.345	-0.057	*RNF135*	Shore	TSS1500	NA
cg07890553	3	119182858	1.03E-12	0.458	0.406	0.052	*TMEM39A*	NA	TSS1500	NA
cg04193820	17	29297414	1.06E-12	0.360	0.420	-0.060	*RNF135*	Shore	TSS1500	NA
cg19886655	6	2958852	1.08E-12	0.290	0.344	-0.053	*SERPINB6*	NA	GeneBody	Intron
cg27380915	7	12796513	1.11E-12	0.512	0.563	-0.051	NA	NA	NA	NA
cg06100756	17	43221575	1.20E-12	0.243	0.299	-0.055	NA	Shelf	NA	NA
cg18329187	14	103989711	1.22E-12	0.339	0.414	-0.075	*CKB*	CpGIsland	TSS1500	NA
cg08248297	19	16311377	1.28E-12	0.561	0.616	-0.055	*AP1M1*	Shelf	GeneBody	Intron
cg07332601	8	41072867	1.35E-12	0.580	0.635	-0.055	NA	NA	NA	NA
cg17494897	2	3200012	1.47E-12	0.484	0.540	-0.055	*TSSC1*	NA	GeneBody	Intron
cg23213327	2	7016509	1.56E-12	0.395	0.445	-0.050	*RSAD2*	NA	TSS1500	NA
cg14329860	2	51240310	1.87E-12	0.453	0.507	-0.054	*NRXN1*	NA	GeneBody	Intron
cg19900896	5	49737492	1.88E-12	0.321	0.263	0.058	*EMB*	Shore	TSS1500	NA
cg13266327	2	11485561	2.02E-12	0.245	0.296	-0.051	*ROCK2*	Shore	TSS1500	NA
cg22514722	3	127473755	2.02E-12	0.630	0.579	0.052	*MGLL*	NA	GeneBody	Intron
cg26266708	15	76630962	2.06E-12	0.313	0.376	-0.063	*ISL2*	Shore	GeneBody	Intron
cg16889557	12	50426531	2.19E-12	0.475	0.527	-0.053	NA	Shore	NA	NA
cg03146219	11	71189514	2.41E-12	0.420	0.499	-0.079	*NADSYN1*	NA	GeneBody	Exon
cg17352215	15	90698455	2.48E-12	0.548	0.599	-0.052	NA	NA	NA	NA
cg01721313	3	169751609	2.53E-12	0.437	0.487	-0.050	NA	Shelf	NA	NA
cg07300408	14	21058360	2.66E-12	0.341	0.397	-0.056	*RNASE12*	NA	3UTR	Exon
cg08998950	3	4856199	2.88E-12	0.544	0.610	-0.066	*ITPR1*	NA	GeneBody	Exon
cg04911669	6	73697178	2.94E-12	0.464	0.516	-0.052	*KCNQ5*	NA	GeneBody	Intron
cg09017434	5	16179660	2.94E-12	0.227	0.286	-0.059	*MARCH11*	CpGIsland	FirstExon	Exon
cg18113826	6	31583942	3.34E-12	0.194	0.258	-0.064	*AIF1*	Shelf	GeneBody	Intron
cg14509403	22	30476281	3.41E-12	0.336	0.387	-0.051	*HORMAD2*	CpGIsland	TSS200	NA
cg16158027	2	196520930	3.59E-12	0.538	0.589	-0.051	*SLC39A10*	Shore	TSS1500	NA
cg15360181	3	143567492	3.69E-12	0.446	0.395	0.051	*SLC9A9*	NA	TSS200	NA
cg11510999	12	53591490	3.84E-12	0.579	0.632	-0.053	*ITGB7*	CpGIsland	GeneBody	Intron
cg13935558	7	138144067	3.96E-12	0.540	0.601	-0.060	*TRIM24*	Shore	TSS1500	NA
cg19787694	19	846117	3.97E-12	0.372	0.427	-0.055	*PRTN3*	Shore	GeneBody	Intron
cg17791799	4	16036123	4.34E-12	0.451	0.520	-0.069	*PROM1*	NA	GeneBody	Intron
cg22963979	7	1858916	4.35E-12	0.356	0.437	-0.081	*MAD1L1*	NA	GeneBody	Intron
cg06981309	3	146260954	4.43E-12	0.304	0.392	-0.088	*PLSCR1*	Shore	5UTR	Intron
cg05336395	13	53421688	4.44E-12	0.273	0.324	-0.050	*PCDH8*	Shelf	FirstExon	Exon
cg18067859	17	33776345	4.73E-12	0.545	0.598	-0.052	*SLFN13*	Shore	TSS1500	NA
cg27183791	16	89381904	4.78E-12	0.662	0.609	0.053	*ANKRD11*	NA	GeneBody	Intron
cg03192598	19	12305854	4.84E-12	0.115	0.166	-0.051	NA	CpGIsland	NA	NA
cg08634133	7	149570071	5.69E-12	0.234	0.286	-0.052	*ATP6V0E2*	Shore	5UTR	Exon
cg27141751	10	50231850	6.65E-12	0.475	0.527	-0.052	*VSTM4*	NA	GeneBody	Intron
cg27120833	22	43193524	8.99E-12	0.492	0.546	-0.054	*ARFGAP3*	NA	3UTR	Exon
cg12836863	13	32889023	8.99E-12	0.418	0.468	-0.050	*BRCA2*	Shelf	TSS1500	NA
cg22537604	19	43857074	9.36E-12	0.544	0.601	-0.057	*CD177*	NA	TSS1500	NA
cg15379633	22	23487586	1.01E-11	0.280	0.335	-0.055	*RAB36*	Shelf	FirstExon	Exon
cg25537993	19	58545182	1.25E-11	0.171	0.224	-0.053	*ZSCAN1*	CpGIsland	TSS1500	NA
cg15428479	19	18698825	1.28E-11	0.329	0.381	-0.051	*C19orf60*	Shore	TSS1500	NA
cg22544863	19	15580400	1.39E-11	0.185	0.235	-0.050	*PGLYRP2*	Shelf	GeneBody	Intron
cg07979236	1	33516461	1.43E-11	0.355	0.412	-0.057	NA	Shore	NA	NA
cg06022561	2	242702553	1.55E-11	0.634	0.583	0.050	*D2HGDH*	NA	GeneBody	Intron
cg16173847	18	77565614	1.91E-11	0.442	0.391	0.051	NA	Shore	NA	NA
cg11919271	3	151102879	2.07E-11	0.493	0.548	-0.055	*P2RY12*	NA	TSS1500	Exon
cg03025830	8	21905599	2.19E-11	0.562	0.647	-0.085	*FGF17*	CpGIsland	GeneBody	Exon
cg03956042	3	194206497	2.19E-11	0.516	0.463	0.053	NA	Shore	NA	NA
cg16672562	19	46801672	2.26E-11	0.361	0.455	-0.094	*HIF3A*	Shore	5UTR	Exon
cg07468327	16	54170217	2.26E-11	0.471	0.521	-0.050	NA	NA	NA	NA
cg18523477	20	55967503	2.40E-11	0.531	0.475	0.056	*RBM38*	Shelf	GeneBody	Intron
cg02265721	12	64916007	2.45E-11	0.520	0.570	-0.050	NA	NA	NA	NA
cg11564601	22	30592435	2.53E-11	0.286	0.348	-0.063	NA	NA	NA	NA
cg00551647	21	27943730	2.75E-11	0.423	0.474	-0.052	*CYYR1*	Shore	GeneBody	Intron
cg19078576	5	17217877	2.89E-11	0.111	0.164	-0.054	*BASP1*	Shore	5UTR	Exon
cg06783429	7	127673038	3.19E-11	0.731	0.787	-0.056	*SND1*	Shore	GeneBody	Intron
cg22034855	2	20792009	3.59E-11	0.519	0.570	-0.051	NA	NA	NA	NA
cg05575639	8	61593696	3.83E-11	0.512	0.565	-0.053	*CHD7*	Shore	5UTR	Intron
cg00531137	16	57643932	3.96E-11	0.558	0.503	0.055	NA	NA	NA	NA
cg26644674	10	3138505	4.22E-11	0.379	0.430	-0.051	*PFKP*	NA	GeneBody	Intron
cg17444090	1	232735450	5.49E-11	0.353	0.406	-0.053	NA	NA	NA	NA
cg19102955	20	5928064	5.78E-11	0.338	0.388	-0.051	*TRMT6*	Shelf	GeneBody	Intron
cg04173586	19	2167496	5.83E-11	0.572	0.640	-0.069	*DOT1L*	Shore	GeneBody	Intron
cg11800635	2	74783088	7.20E-11	0.506	0.557	-0.052	*DOK1*	Shore	GeneBody	Exon
cg00113675	6	29855347	7.70E-11	0.337	0.415	-0.078	NA	CpGIsland	NA	NA
cg18424841	20	61315444	7.96E-11	0.627	0.689	-0.061	NA	CpGIsland	NA	NA
cg05552874	10	91153143	8.06E-11	0.461	0.568	-0.107	*IFIT1*	NA	GeneBody	Intron
cg14281591	13	73636077	8.32E-11	0.465	0.518	-0.053	*KLF5*	Shore	GeneBody	Exon
cg04347414	1	2084519	8.75E-11	0.663	0.613	0.050	*PRKCZ*	Shore	GeneBody	Intron
cg05618183	16	31190476	9.40E-11	0.410	0.356	0.054	*FUS*	Shore	TSS1500	NA
cg19947463	7	1113237	9.73E-11	0.396	0.446	-0.050	*C7orf50*	NA	GeneBody	Intron
cg03832522	2	218898668	1.10E-10	0.245	0.302	-0.057	NA	CpGIsland	NA	NA

cg08797194	13	96705123	1.17E-10	0.336	0.402	-0.066	*UGGT2*	Shore	GeneBody	Intron
cg27395066	17	43221220	1.22E-10	0.293	0.354	-0.061	*ACBD4*	Shelf	3UTR	Exon
cg08468401	3	14303131	1.24E-10	0.341	0.287	0.053	NA	NA	NA	NA
cg03948781	1	205179583	1.52E-10	0.309	0.363	-0.054	*DSTYK*	Shore	GeneBody	Intron
cg18121224	5	176559563	1.55E-10	0.589	0.643	-0.054	*NSD1*	CpGIsland	TSS1500	NA
cg13397898	9	133768931	2.27E-10	0.424	0.475	-0.051	*QRFP*	NA	FirstExon	Exon
cg03351487	2	169003820	2.41E-10	0.535	0.590	-0.055	*STK39*	NA	GeneBody	Intron
cg25077558	5	156991510	3.37E-10	0.561	0.613	-0.052	*ADAM19*	NA	GeneBody	Intron
cg06633438	19	6272158	3.45E-10	0.400	0.452	-0.052	*MLLT1*	CpGIsland	GeneBody	Intron
cg05680237	6	27103185	3.61E-10	0.608	0.659	-0.052	NA	Shelf	NA	NA
cg00817464	10	111662876	3.63E-10	0.888	0.837	0.051	*XPNPEP1*	NA	GeneBody	Intron
cg23730027	3	57995180	3.80E-10	0.338	0.389	-0.050	*FLNB*	CpGIsland	GeneBody	Intron
cg27632402	6	106957404	3.86E-10	0.441	0.497	-0.056	NA	Shelf	NA	NA
cg19152255	20	52274268	4.72E-10	0.452	0.503	-0.051	NA	Shelf	NA	NA
cg17493885	5	176559558	5.29E-10	0.510	0.575	-0.064	*NSD1*	CpGIsland	TSS1500	NA
cg20563269	2	129104576	7.02E-10	0.554	0.606	-0.052	NA	Shore	NA	NA
cg13912224	6	52172083	7.04E-10	0.391	0.456	-0.065	NA	CpGIsland	NA	NA
cg05563515	6	30039027	7.97E-10	0.614	0.692	-0.078	*RNF39*	Shelf	GeneBody	Intron
cg20958434	14	106174538	8.38E-10	0.567	0.516	0.051	NA	NA	NA	NA
cg16998831	7	187686	9.32E-10	0.521	0.575	-0.054	NA	Shore	NA	NA
cg11853830	5	92914036	9.32E-10	0.378	0.428	-0.051	*FLJ42709*	Shore	ncRNA	Intron
cg18643199	10	82363313	1.03E-09	0.614	0.671	-0.057	*SH2D4B*	NA	GeneBody	Intron
cg20034226	17	18529285	1.10E-09	0.567	0.511	0.056	NA	Shore	NA	NA
cg23478547	11	69259265	1.38E-09	0.445	0.508	-0.063	NA	Shore	NA	NA
cg12048225	6	32808669	1.58E-09	0.446	0.390	0.056	*PSMB8*	Shore	3UTR	Exon
cg24585035	5	32522852	1.60E-09	0.555	0.609	-0.054	NA	NA	NA	NA
cg05156137	21	35898975	1.63E-09	0.335	0.284	0.051	*RCAN1*	NA	GeneBody	Intron
cg20945085	2	74875227	1.80E-09	0.357	0.410	-0.053	*C2orf65*	CpGIsland	TSS200	NA
cg00664406	3	51740875	1.81E-09	0.386	0.437	-0.051	*GRM2*	CpGIsland	TSS1500	NA
cg01079652	1	79118191	2.03E-09	0.707	0.800	-0.093	*IFI44*	NA	GeneBody	Intron
cg09136878	1	236135584	2.36E-09	0.407	0.461	-0.053	NA	NA	NA	NA
cg22891070	19	46801642	2.72E-09	0.410	0.488	-0.078	*HIF3A*	Shore	5UTR	Exon
cg23687466	11	504937	2.74E-09	0.398	0.449	-0.050	*RNH1*	Shore	5UTR	Exon
cg01017257	1	15059738	3.02E-09	0.574	0.625	-0.051	*KAZN*	NA	GeneBody	Intron
cg08839808	6	156983304	3.12E-09	0.487	0.540	-0.054	NA	NA	NA	NA
cg27589742	16	85296503	3.56E-09	0.497	0.555	-0.058	NA	NA	NA	NA
cg22894896	17	29886890	4.38E-09	0.460	0.511	-0.051	NA	CpGIsland	NA	NA
cg16055526	6	33083287	4.79E-09	0.616	0.561	0.055	*HLA-DPB2*	Shore	ncRNA	Intron
cg23348081	12	14413690	5.46E-09	0.551	0.601	-0.050	NA	NA	NA	NA
cg25844590	11	7621556	6.32E-09	0.510	0.561	-0.051	*PPFIBP2*	NA	GeneBody	Intron
cg06106599	11	70048801	6.66E-09	0.520	0.578	-0.058	*FADD*	Shore	TSS1500	NA
cg19318393	1	223936508	6.75E-09	0.416	0.472	-0.056	*CAPN2*	CpGIsland	GeneBody	Intron
cg09907509	13	37248244	7.33E-09	0.185	0.243	-0.057	*SERTM1*	CpGIsland	5UTR	Exon
cg27577554	17	30412142	7.40E-09	0.370	0.422	-0.052	NA	NA	NA	NA
cg23907051	2	101730305	8.73E-09	0.516	0.568	-0.051	*TBC1D8*	NA	GeneBody	Intron
cg25403205	6	31584215	8.96E-09	0.230	0.294	-0.064	*AIF1*	Shelf	GeneBody	Exon
cg21549285	21	42799141	1.08E-08	0.405	0.568	-0.162	*MX1*	Shore	5UTR	Exon
cg11277662	8	143408047	1.21E-08	0.556	0.606	-0.050	*TSNARE1*	CpGIsland	GeneBody	Intron
cg14375499	17	6899207	1.43E-08	0.547	0.599	-0.051	*ALOX12*	CpGIsland	TSS200	Intron
cg12542255	19	45976195	2.49E-08	0.202	0.253	-0.051	*FOSB*	Shelf	GeneBody	Exon
cg04942251	11	63687247	2.88E-08	0.539	0.589	-0.050	NA	Shelf	NA	NA
cg26306976	2	9564901	3.83E-08	0.556	0.608	-0.052	*ITGB1BP1*	Shore	TSS1500	Intron
cg00918181	18	11947875	4.27E-08	0.226	0.286	-0.059	NA	CpGIsland	NA	NA
cg19805943	2	85933069	4.41E-08	0.563	0.624	-0.061	NA	NA	NA	NA
cg05404236	13	110437093	4.98E-08	0.290	0.346	-0.056	*IRS2*	CpGIsland	FirstExon	Exon
cg11379081	1	209405050	5.64E-08	0.264	0.320	-0.056	NA	CpGIsland	NA	NA
cg11787160	12	113515332	6.43E-08	0.561	0.617	-0.057	*DTX1*	CpGIsland	GeneBody	Exon
cg04007350	7	158263221	6.58E-08	0.520	0.574	-0.054	*PTPRN2*	NA	GeneBody	Intron
cg06804705	21	40125580	7.15E-08	0.708	0.762	-0.055	*LINC00114*	NA	ncRNA	Intron
cg12899747	3	25391527	9.40E-08	0.341	0.288	0.053	NA	NA	NA	NA
cg03395546	19	41222599	1.05E-07	0.384	0.329	0.056	*ADCK4*	CpGIsland	5UTR	Exon
cg08371391	20	19739935	1.09E-07	0.259	0.314	-0.055	NA	Shore	NA	NA
cg16532282	1	226271799	1.18E-07	0.360	0.411	-0.051	NA	CpGIsland	NA	NA
cg10635122	19	52391090	1.26E-07	0.311	0.362	-0.051	*ZNF577*	CpGIsland	ncRNA	Exon
cg09973676	8	82006417	1.28E-07	0.459	0.394	0.065	*PAG1*	NA	5UTR	Intron

‡ P-value of the SLE-pSS case-case association analysis of differential DNA methylation
