# Supplementary material for: Shared and Unique Patterns of DNA Methylation in Systemic Lupus Erythematosus and Primary Sjögren's Syndrome
Source: Front Immunol. 2019 Jul 30;10:1686. doi: 10.3389/fimmu.2019.01686 (PMC6688520; doi:10.3389/fimmu.2019.01686)
Supplement: Supplementary file 7 [file Table_7.pdf]

Supplementary Table S7 *Functional pathway analysis of genes with differentially methylated CpG sites (DMCs) in the case-case association analysis comparing DNA methylation in patients with SLE and patients with pSS*

Pathway	Molecules	p-value
Neutrophil degranulation	*ACPP, AMPD3, ANXA2, ARG1, ATG7, AZU1, CD44, CD59, CD63, CHI3L1, CHIT1, CLEC4C, CLEC5A, CRISPLD2, CTSG, CTSZ, DOCK2, ELANE, FCAR, FCER1G, FGR, FOLR3, GALNS, GSN, IMPDH1, IQGAP2, ITGAL, LCN2, LGALS3, LPCAT1, LRG1, MANBA, MAPK14, MPO, MS4A3, OLR1, OSCAR, P2RX1, PDXK, PLAC8, PRG2, PRTN3, PSMB7, PTPRJ, RAB10, RAB44, RNASE3, S100A8, S100A9, S100P, SLC15A4, SLC44A2, SLPI, TARM1, TBC1D10C, TIMP2, TMEM63A, TNFAIP6, TNFRSF1B, TOLLIP, TRPM2, UBR4, VAT1, YPEL5*	4.19×10^{-11}
Innate immune system	*ACPP, ADCY7, AHCYL1, AKT2, AMPD3, ANXA2, APP, ARG1, ATG7, ATP6V1E2, AZU1, BAIAP2, BCL2, C4BPA, CAMK2G, CARD11, CCR6, CD247, CD300LB, CD44, CD59, CD63, CDKN1A, CHI3L1, CHIT1, CLEC4A, CLEC4C, CLEC5A, CLU, CREBBP, CRISPLD2, CSK, CTSG, CTSZ, CUL1, DNM3, DOCK2, DUSP6, ELANE, ELMO1, FCAR, FCER1G, FGF17, FGR, FOLR3, GALNS, GSN, HLA-E, IMPDH1, IQGAP2, IRAK3, ITGAL, KSR1, LCN2, LGALS3, LPCAT1, LRG1, LRRFIP1, MANBA, MAP2K1, MAP2K6, MAP3K11, MAPK14, MAPKAPK2, MEFV, MPO, MS4A3, MYO10, MYO1C, NCK1, NLRC4, NLRP3, NOS3, OLR1, OSCAR, P2RX1, PAK2, PDXK, PHLPP1, PIK3CD, PIP5K1A, PLAC8, PRG2, PRTN3, PSMB7, PSMB8, PTEN, PTPRJ, RAB10, RAB44, RASA3, RNASE3, RPS6KA2, S100A7A, S100A8, S100A9, S100P, SARM1, SHC1, SLC15A4, SLC44A2, SLPI, SPRED2, SPTBN1, SYNGAP1, TARM1, TBC1D10C, TIMP2, TLR6, TMEM63A, TNFAIP6, TNFRSF1B, TOLLIP, TREM1, TREM2, TRPM2, UBR4, VAT1, WIPF1, YPEL5*	3.22×10^{-9}
C-MYB transcription factor network	*BCL2, CASP6, CBX4, CDK6, CDKN1A, CREBBP, ELANE, ETS2, HIPK2, MAD1L1, MPO, MYB, PRTN3, SKI, SP1, SPI1*	3.37×10^{-6}
Regulation of transcriptional activity by PML	*CREBBP, FASLG, PRAM1, RARA, RB1, TNF, TNFRSF1B*	1.59×10^{-5}
Notch-HLH transcription pathway	*CREBBP, MAML1, MAML2, MAML3, NOTCH1, NOTCH4*	3.04×10^{-5}
Cyclin D associated events in G1	*CCND2, CCNH, CDK6, CDKN1A, CUL1, E2F3, MNAT1, PPP2R2A, RB1, TFDP1*	3.07×10^{-5}
Pre-NOTCH expression and processing	*ATP2A2, CREBBP, E2F3, LFNG, MAML1, MAML2, MAML3, NOTCH1, NOTCH4, ST3GAL3, TFDP1*	3.14×10^{-5}
Sphingolipid signaling pathway	*ABCC1, ADORA3, AKT2, BCL2, FCER1G, GNA12, GNAI3, MAP2K1, MAPK14, NOS3, PIK3CD, PLCB2, PPP2R2A, PRKCZ, PTEN, S1PR2, SGMS2, TNF*	6.12×10^{-5}
Metal sequestration by antimicrobial proteins	*LCN2, S100A7A, S100A8, S100A9*	1.17×10^{-4}
Hemostasis	*ACTN4, AKT2, ANXA2, APP, ATP2A2, CBX5, CD44, CD63, CLU, CREBBP, CSK, DOCK2, DOK2, EHD1, F10, FAM49B, FCER1G, FERMT3, FGR, GNA12, GNA15, GNAI3, GNG7, GP9, GRB7, GYPC, INPP5D, ITGAL, ITPK1, KDM1A, KIF13B, KIF1B, KIF21B, KIF2A, MAFG, MAPK14, MYB, NFE2, NOS3, OLR1, P2RX1,*	1.32×10^{-4}

	PHACTR2, PIK3CD, PIK3R5, PRKCZ, PRTN3, RAD51B, RAPGEF3, RCOR1, SHC1, SLC16A1, SLC16A8, SLC7A7, SLC7A8, STIM1, TREM1, TRPC3	
IL2 signaling events mediated by STAT5	*BCL2, CCND2, CDK6, FASLG, LTA, PRF1, SHC1, SP1*	$1.43x10^{-4}$
Pre-NOTCH transcription and translation	*CREBBP, E2F3, MAML1, MAML2, MAML3, NOTCH1, NOTCH4, TFDP1*	$1.43x10^{-4}$
Map kinase inactivation of SMRT corepressor	*MAP2K1, MAPK14, NCOR2, RARA, ZBTB16*	$1.61x10^{-4}$
Regulation of retinoblastoma protein	*ATF7, BRD2, CBX4, CDKN1A, CREBBP, MAPK14, PPARG, RB1, SFTPD, SPI1, TFDP1*	$1.72x10^{-4}$
Insulin resistance	*AKT2, CREB3L2, G6PC3, NOS3, OGA, PCK2, PIK3CD, PRKAG1, PRKAG2, PRKCZ, PTEN, RPS6KA2, SLC27A1, SLC2A1, SREBF1, TNF*	$1.96x10^{-4}$
Notch signaling pathway	*CDKN1A, CUL1, DLK1, DTX1, ENO1, KDM1A, MAML1, MAML2, NCOR2, NOTCH1, NOTCH4*	$2.38x10^{-4}$
HIF-1 signaling pathway	*AKT2, BCL2, CAMK2G, CDKN1A, CREBBP, CUL2, EGLN1, ENO1, IFNGR2, LTBR, MAP2K1, MKNK1, NOS3, PIK3CD, SLC2A1*	$3.33x10^{-4}$
Chagas disease (American trypanosomiasis)	*AKT2, CALR, CD247, FASLG, GNA15, GNAI3, GNAO1, IFNGR2, IL10, MAPK14, PIK3CD, PLCB2, PPP2R2A, TLR6, TNF*	$3.71x10^{-4}$
Signaling by NOTCH	*ATP2A2, CREBBP, CUL1, DLK1, DTX1, E2F3, HDAC4, LFNG, MAML1, MAML2, MAML3, NCOR2, NOTCH1, NOTCH4, ST3GAL3, TFDP1*	$3.71x10^{-4}$
