# Supplementary material for: Shared and Unique Patterns of DNA Methylation in Systemic Lupus Erythematosus and Primary Sjögren's Syndrome
Source: Front Immunol. 2019 Jul 30;10:1686. doi: 10.3389/fimmu.2019.01686 (PMC6688520; doi:10.3389/fimmu.2019.01686)
Supplement: Supplementary file 8 [file Image_1.pdf]

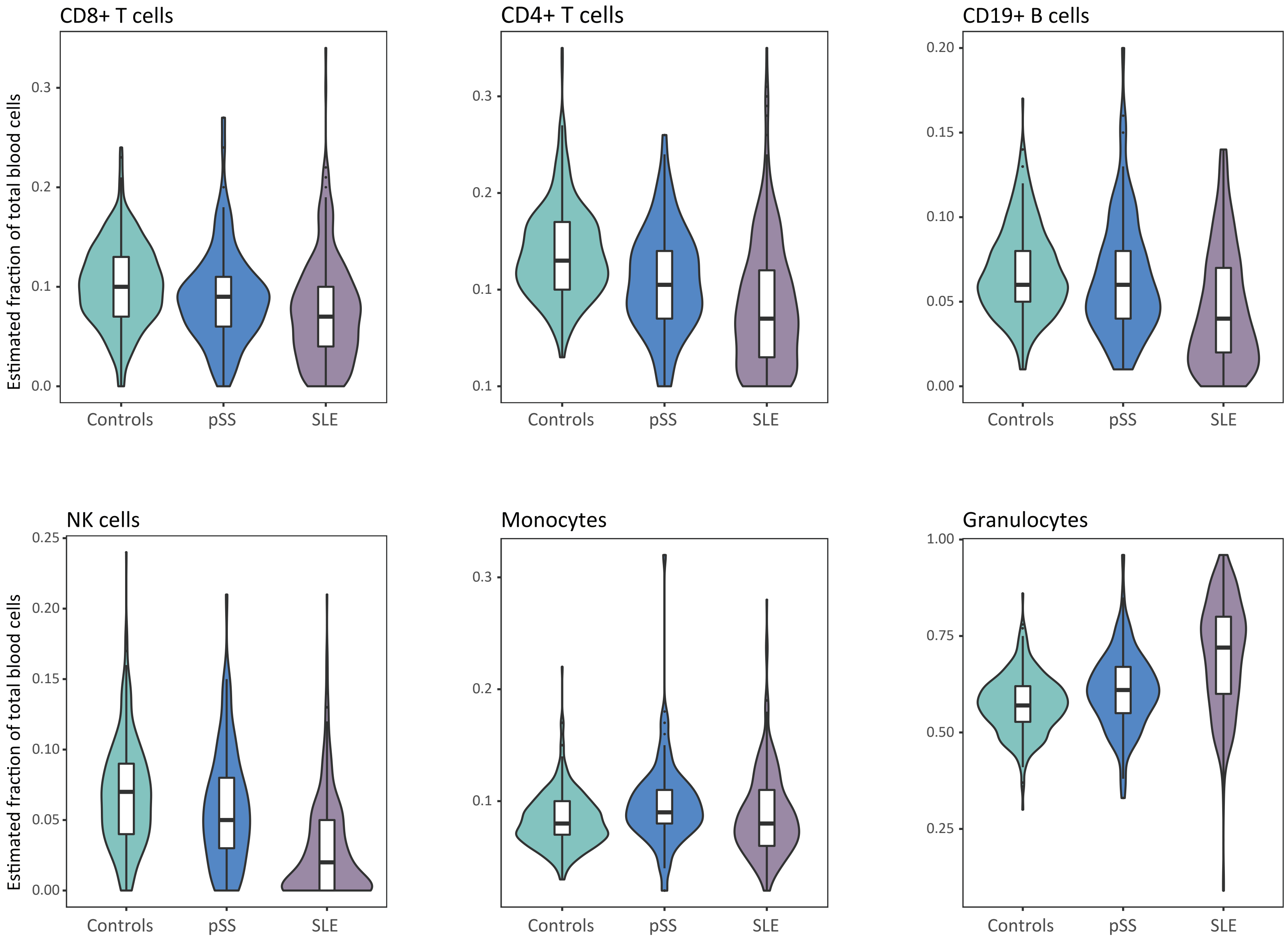


**Supplementary Figure S1**

**Blood cell type distribution estimates.** Violin plots with box plots depicting the estimated cell type fractions in whole blood samples from healthy controls (n=400), patients with pSS (n=100) and patients with SLE (n=347) for CD8+ T cells, CD4+ T cells, CD19+ B cells, natural killer (NK) cells, monocytes and granulocytes.
