# Supplementary material for: Shared and Unique Patterns of DNA Methylation in Systemic Lupus Erythematosus and Primary Sjögren's Syndrome
Source: Front Immunol. 2019 Jul 30;10:1686. doi: 10.3389/fimmu.2019.01686 (PMC6688520; doi:10.3389/fimmu.2019.01686)
Supplement: Supplementary file 9 [file Image_2.pdf]

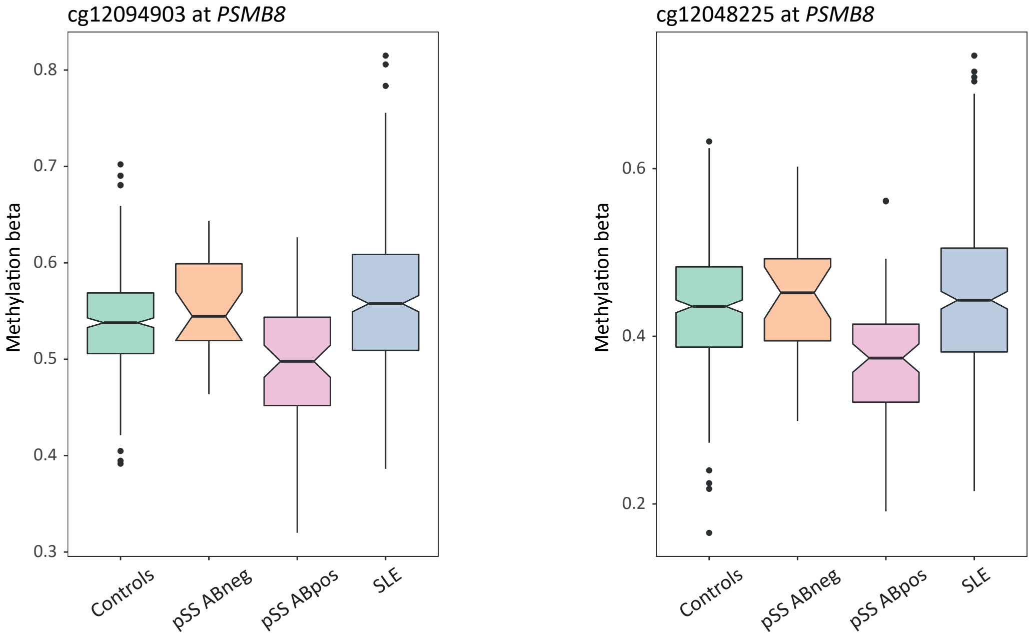

Supplementary Figure S2

Differential DNA methylation at the *PSMB8* locus. Box plots of DNA methylation levels for controls (n=400), patients with pSS stratified for anti-SSA/SSB negative pSS (n=25) and anti-SSA/SSB positive pSS (n=75) and patients with SLE (n=347) with a notch indicating the group median methylation β at cg12094903 and cg12048225 located within the PSMB8-TAP2 locus on chromosome 6.
