# Supplementary material for: Shared and Unique Patterns of DNA Methylation in Systemic Lupus Erythematosus and Primary Sjögren's Syndrome
Source: Front Immunol. 2019 Jul 30;10:1686. doi: 10.3389/fimmu.2019.01686 (PMC6688520; doi:10.3389/fimmu.2019.01686)
Supplement: Supplementary file 10 [file Image_3.pdf]

A

	all DMCs	hyper SLE	hypo SLE
CpG Island	**0.23**	**0.20**	**0.23**
Shore	0.86	0.83	0.86
Shelf	**1.32**	**1.62**	**1.28**
Open Sea	**1.60**	**1.52**	**1.61**

B

	all DMCs	hyper SLE	hypo SLE
Exon	0.86	1.13	**0.82**
Intron	**1.32**	1.22	**1.33**
NA	**0.79**	0.74	**0.79**

C

	all DMCs	hyper SLE	hypo SLE
TSS1500	**0.78**	0.74	**0.79**
TSS200	**0.48**	0.57	**0.47**
5UTR	0.95	0.99	0.95
1stExon	0.67	**0.28**	0.72
GeneBody	**1.31**	1.36	**1.30**
3UTR	**1.34**	1.33	1.34
ncRNA	1.10	1.37	1.08
NA	0.93	0.84	0.94

D

		all DMCs	hyper SLE	hypo SLE
H3K4me1 Enhancer	T	1.07	**2.01**	0.96
	B	**1.19**	**2.13**	1.08
H3K27ac Enhancer	T	**0.79**	**2.40**	**0.60**
	B	**0.85**	**2.18**	**0.69**
H3K4me3 Promoter	T	**0.49**	**1.47**	**0.37**
	B	**0.53**	**1.11**	**0.46**
H3K36me3 Transcribed gene body	T	**1.85**	**2.05**	**1.82**
	B	**1.97**	**2.65**	**1.89**
H3K27me3 Repressive	T	**0.23**	**0.08**	**0.25**
	B	**0.39**	**0.11**	**0.43**
H3K9me3 Heterochromatin	T	0.66	na	0.74
	B	0.62	0.38	0.65
DHS Euchromatin	T	**0.68**	**1.80**	**0.54**
	B	**0.73**	**1.52**	**0.63**

Supplementary Figure S3

Functional regional distribution. Heat maps of the functional regional distribution of differentially methylated CpG sites (DMCs) in the association analysis of patients with SLE compared to patients with pSS. The plots show the functional genomic distribution of all associated DMCs (n=2,244; left panel), of the DMCs with increased methylation in SLE compared to pSS (n=242; middle panel) and of the DMCs with decreased methylation in SLE compared to pSS (n=2,002; right panel) annotated **(A)** in relation to the CpG island context, **(B)** in relation to exonic and intronic genomic regions, **(C)** in relation to functional gene regions and **(D)** in relation to genomic overlap with chromatin states in reference $CD3^{+}$ T cells and $CD19^{+}$ B cells. The color scale indicates fold-enrichment (red) or fold-depletion (blue) of DMCs in each functionally annotated region. Bold numbers represent annotations to which the DMCs significantly differ compared with the distribution of all probes on the HM450k array (post-QC probe set) (X_2-test $p<0.0035$).

Abbreviations: TSS1500, 1500 bp upstream of transcription start site (TSS); TSS200, 200 bp upstream of TSS; ncRNA, non-coding RNA; NA, probe not annotated to a defined gene property or region; n.a., not applicable
